# Cooperative Bifurcated Chalcogen Bonding and Hydrogen Bonding as Stereocontrolling Elements for Selective Strain-Release Septanosylation

**DOI:** 10.1021/jacs.3c06984

**Published:** 2023-11-30

**Authors:** Wenpeng Ma, Jan-Lukas Kirchhoff, Carsten Strohmann, Bastian Grabe, Charles C. J. Loh

**Affiliations:** † Abteilung Chemische Biologie, Max-Planck-Institut für Molekulare Physiologie, Otto-Hahn-Straße 11, Dortmund 44227, Germany; ‡ Fakultät für Chemie und Chemische Biologie, Technische Universität Dortmund, Otto-Hahn-Straße 4a, Dortmund 44227, Germany; § Fakultät für Chemie und Chemische Biologie, Anorganische Chemie, Technische Universität Dortmund, Otto-Hahn-Straße 6, Dortmund 44227, Germany

## Abstract

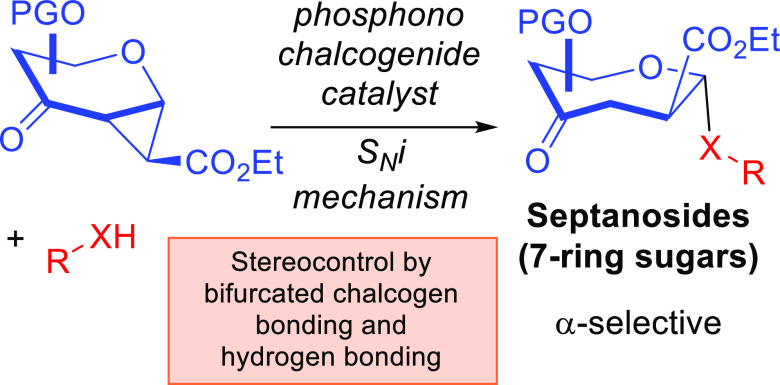

The exploitation of noncovalent interactions (NCIs) is emerging as a vital handle in tackling broad stereoselectivity challenges in synthesis. In particular, there has been significant recent interest in the harnessing of unconventional NCIs to surmount difficult selectivity challenges in glycosylations. Herein, we disclose the exploitation of an unconventional bifurcated chalcogen bonding and hydrogen bonding (HB) network, which paves the way for a robust catalytic strategy into biologically useful seven-membered ring sugars. Through ^13^C nuclear magnetic resonance (NMR) in situ monitoring, NMR titration experiments, and density functional theory (DFT) modeling, we propose a remarkable contemporaneous activation of multiple functional groups consisting of a bifurcated chalcogen bonding mechanism working hand-in-hand with HB activation. Significantly, the ester moiety installed on the glycosyl donor is critical in the establishment of the postulated ternary complex for stereocontrol. Through the ^13^C kinetic isotopic effect and kinetic studies, our data corroborated that a dissociative S_N_i-type mechanism forms the stereocontrolling basis for the excellent α-selectivity.

## 1 Introduction

**Figure 1:**
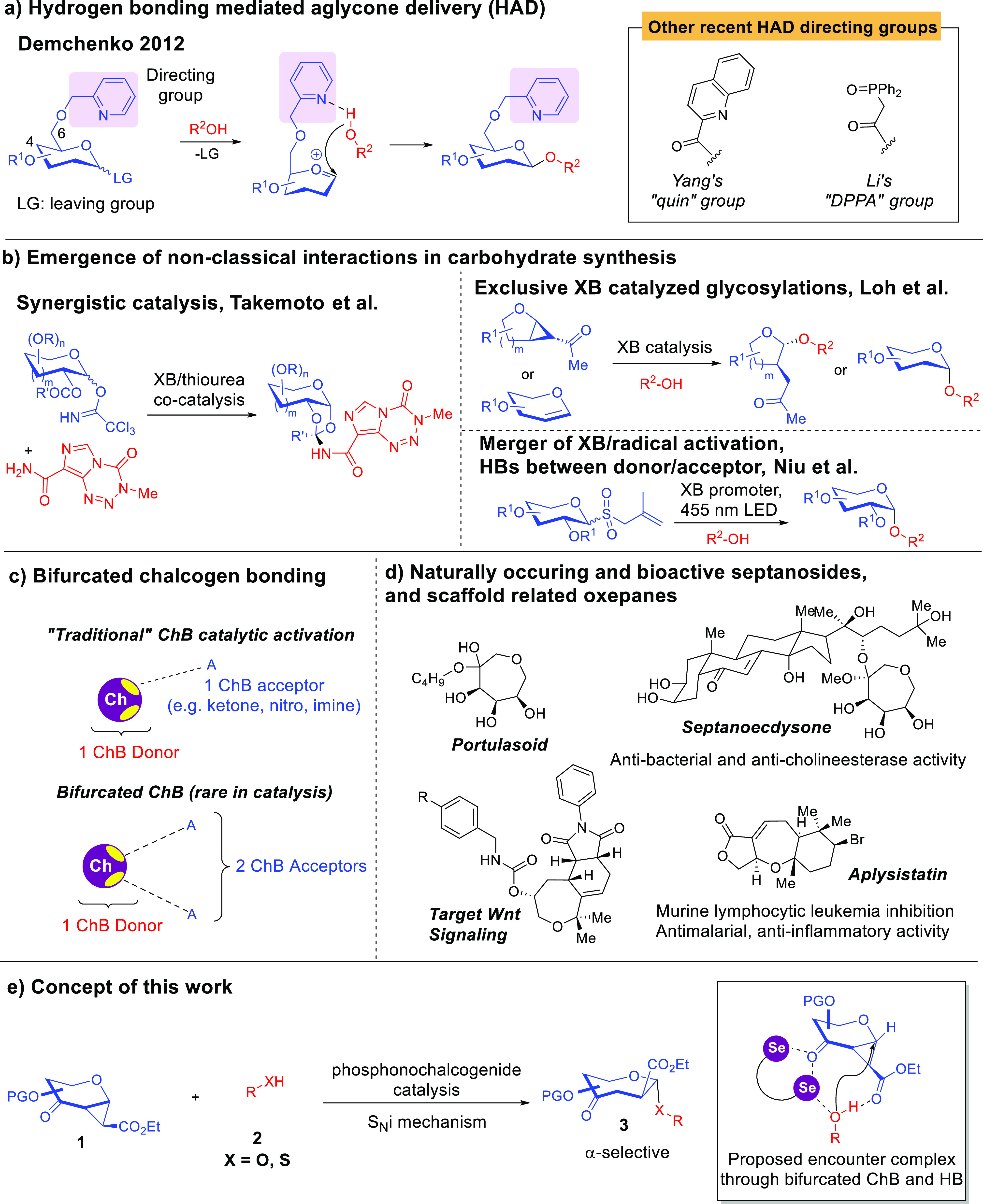
Literature-known HB-mediated aglycone delivery, unconventional NCIs in carbohydrate synthesis, bifurcated chalcogen bonding, and current work. (a) Classical HB-based anomeric control by harnessing HAD. (b) Emergence of nonclassical halogen bonding catalyzed carbohydrate synthesis. (c) Traditional ChB activation vs bifurcated ChB. (d) Prevalence of the septanoside scaffold in bioactive molecules and oxepane chemotype in natural products. (e) Concept of this work.

The realization of noncovalent interactions (NCIs) as a powerful stereocontrolling element is gaining broad recognition in synthesis.^1^ However, in the challenging domain of stereoselective carbohydrate synthesis^2^—one of the most difficult challenges in modern organic synthesis due to its vast stereochemical complexity—harnessing the immense potential of NCIs to tackle the broad range of stereoselectivity changes is still in its infancy.^3^ Carbohydrates are also extremely sensitive to an array of substrate effects unseen in other substrate classes,^2b,4^ often hindering method generality. The recognition of NCIs, particularly classical hydrogen bonding (HB) as a powerful stereocontrolling force in controlling anomeric selectivity is elegantly demonstrated through the field defining HB-mediated aglycone delivery (HAD) strategy pioneered by Demchenko and Yasomanee by installing the highly tractable picoloyl (pico) directing group (Figure 1a).^5^ This strategy has later found important applications in natural product synthesis such as the access of tiacumicin B.^6^ Subsequent developments of other variants of directing groups for HAD such as Yang’s quinolinecarbonyl (quin) based groups^7^ and more recently, Li’s 2-(diphenylphosphinoyl)acetyl (DPPA) groups^8^ also shown substantial robustness in accessing challenging polysaccharides. Another lately significant contribution was reported by Niu et al.,^9^ where the inherent ether oxygen on the protected C2-hydroxyl group could be harnessed as a HB acceptor, furnishing a HAD-type aglycone delivery without an explicit directing group strategy.

However, the exploration of the arsenal of NCIs beyond classical HBs to steer anomeric selectivity is substantially understudied, and very little is understood about how tinkering with the palette of unconventional NCIs cooperatively could bring about stereoselectivity benefits.

The family of more directional NCIs that is commonly recognized to operate through sigma hole activation,^10^ is an underexploited synthetic tool that often brings forth counterintuitive catalytic mechanisms. Exemplified clearly in recent advances, sigma hole catalysis has blossomed into a formidable chemical glycosylation tool, particularly in difficult glycosidic bond-forming events. The exploitation of halogen bonding (XB) catalysis in particular,^10a,10e^ has lately contributed to significant progress in glycosylations (Figure 1b). After the 2014 seminal proof-of-concept work by Huber and Codée in employing XB as a stoichiometric activator of a glycosyl halide,^11^ Takemoto and co-workers reported in 2018 a thiourea and a benzoimidazolium XB catalyzed *N*-glycofunctionalization.^12^ Exclusive XB catalytic activation was later reported by our group in strain-release glycosylations,^13^ as well as in 2-deoxyglycosylations.^14^ In these cases, distinctive advantages such as in the elevation of anomeric selectivity,^13^ and in profoundly expanded glycosyl donor and acceptor scopes were observed compared with HB catalysis.^15^ Niu et al. lately capitalized on a merger of concepts of XB-assisted radical activation and an ether-mediated HB-based aglycone delivery to achieve 1,2-*cis*-glycosides in a highly stereoconvergent fashion with broad substrate utility.^9^

In light of the above, we were interested in unraveling new glycosylation capabilities by tapping upon chalcogen bonding (ChB),^10b,10c^ and unconventional bifurcated ChB variants (Figure 1c). Differing from XB, since chalcogens are known to contain two sigma holes per chalcogenic atom, they are able to engage in bifurcation.^16^ However, to the best of our understanding, previously reported ChB catalytic methods are limited to single sigma hole manifolds, and the simultaneous tapping upon their bifurcation potential is still unexplored (Figure 1c). Such bifurcated manifolds could offer expanded dimensions of stereoselectivity control for complex glycosylation hurdles that are still not adequately addressed via classical HB-based methods.

The glycosylation of seven-membered ring sugars known as septanosides is therefore of synthetic interest.^17^ This carbohydrate motif is a homologated seven-ring analogue of more commonly encountered six-ring pyranosides, and some septanosides possess biological relevance (Figure 1d).^17b^ Septanosides extracted from *Atriplex portulacoides* roots such as portulasoid and septanoecdysone are known to possess antibacterial properties as well as anticholinesterase activity.^18^ Recently, septanosides were demonstrated as useful biological probes that can penetrate the bacterial membrane of *Escherichia coli*.^19^ The central oxepane core of septanosides bears scaffold resemblance to the 7-ring oxacycle architecture in marine natural products, such as hemibrevetoxin B and aplysistatin,^20^ although these molecules are often fused and are not defined as septanosides. Oxepane is further a known chemotype of interest, as it targets the cancer-relevant Wnt-signaling pathway.^21^ Further, septanosides are recognized as useful carbohydrate mimetic building blocks for oligosaccharide synthesis.^22^

On the other hand, understanding stereoselective septanosylations lags behind pyrano- and furanosylations.^17^ Particularly, the control of anomeric selectivity is unsatisfactorily addressed.^23^ In the septanosylation examples known in the literature, acidic and basic conditions were often unfavorable for anomeric selectivity. For instance, the presence of Brønsted acid facilitates hydrolytic scission of the newly formed septanosyl glycosidic linkage.^24^ In other examples, employing stoichiometric strong bases increased the susceptibility of epimerization.^23a,25^ When common Lewis acids such as TMSOTf were employed,^23b^ diminished anomeric selectivity was noted previously upon glycosyl acceptor modification and the usage of galactosyl donors. Further, in a single reported example, thiol-containing nucleophiles cannot be used to access *S*-septanosides.^26^ However, we do recognize the caveat that these synthetic downsides could also be attributed to specific conditions and substrates used in the above references; thus, caution should be exercised to avoid overgeneralizing these characteristics across the entire septanoside substrate class.

We herein present a demonstration of a stereoselective septanosylation method (Figure 1e) that harnesses unconventional bifurcated ChB and HB catalytically for both anomeric stereocontrol and substrate activation. This paves the usage of nonclassical NCIs in aglycone delivery beyond currently reported HAD manifolds. Further, the exploitation of this blend of nonclassical NCIs opened up reliable access toward a very broad range of *O*- and *S*-septanosides that tolerate a wide range of glycosyl acceptors on both gluco- and galactocyclopropanated glycosyl donors. Notably, conventional activation modes by means of thiourea, XB, and standard Lewis acid catalysis were unsuitable. Through NMR titrations and ^13^C NMR in situ monitoring experiments, we determine that contemporaneous activation on multiple functional groups through a blend of nonconventional NCIs is operative. ^13^C kinetic isotopic effect measurements and kinetics collectively support a dissociative S_N_i-type mechanism, by which the catalytic nonclassical NCI network fixates the glycosyl acceptor on the α-face and guides the facial delivery of the aglycone. Control experiments verified that the presence of an ester moiety on the cyclopropanated glycosyl donor was also critical in the establishment of a ternary assembly without which stereocontrol could not be sustained.

## 2 Results and Discussion

### 2.1 Establishment of the Septanosylation Strategy

We commenced our study by employing the cyclopropanated glycosyl donor **1a**(27) and the diacetone galactose acceptor **2a** as substrates in our septanosylation strategy. We first tested a series of Wang’s bidentate phosphonochalcogenides.^28^ Initial screening with catalysts **A–B** gave low but promising yields of our desired septanoside with excellent anomeric selectivity (Table 1, entries 1 and 2). The reaction conditions were further fine-tuned by modifying the temperature and catalyst loadings (Table 1, entries 3–7). Delightfully, we eventually arrived at the optimized conditions using 2 mol % of diphosphonoselenide catalyst **A** at 35 °C (Table 1, entry 6). A further solvent screening comparing the performance of common solvents also revealed that DCE is the optimal solvent for this protocol (Supporting Information Supplementary Table 1). By further investigating the 3-carbon linker catalyst **C**, and comparing the results with **A** and **B**, we noted that the linker length is critical for the septanosylation to proceed (Table 1, entries 6–8).

**Table 1:**
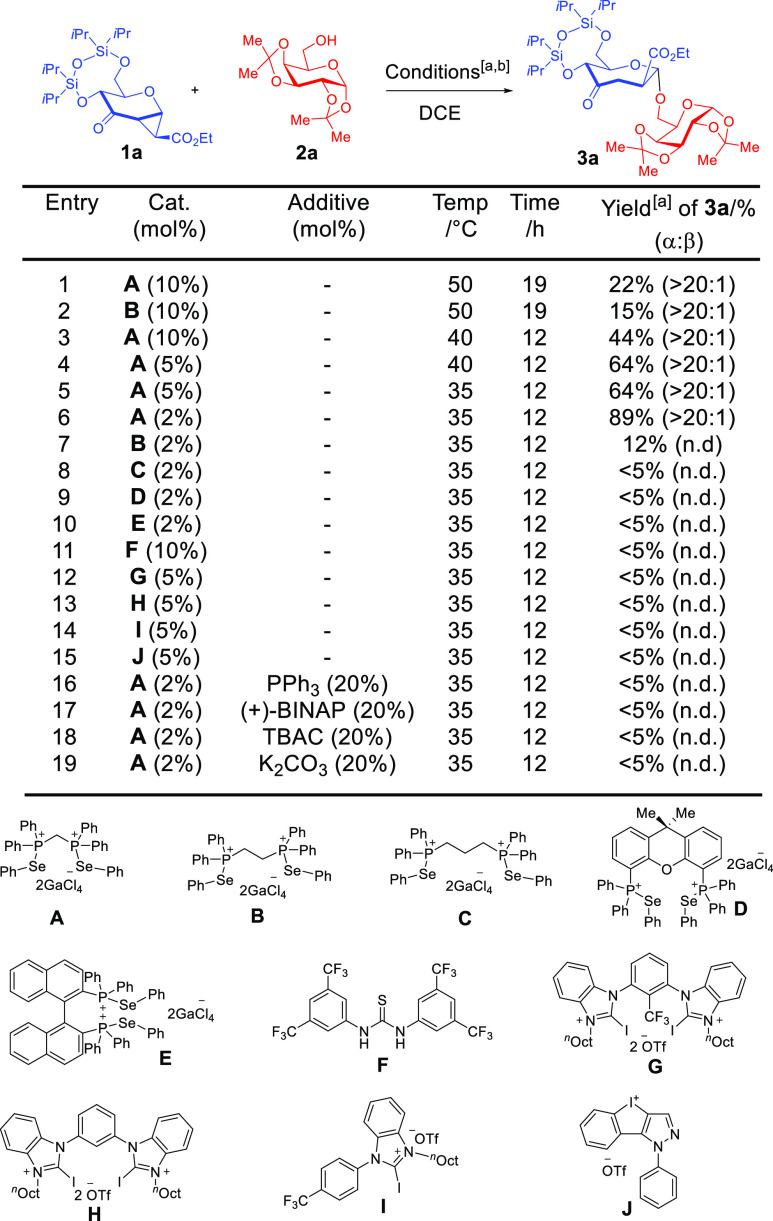
Selected Optimization of ChB Catalyzed Septanosylation and the Influence of Poisoning Additives^a^

a Condition: **1a** (0.1 mmol), **2a** (0.2 mmol), catalyst, DCE (0.2 M), argon. [a] Yields were determined by crude ^1^H NMR spectra analysis using 1,3,5-trimethoxybenzene as an internal standard.

Comparatively, using more commonly known noncovalent catalysts such as the HB Schreiner’s thiourea **F** (Table 1, entry 11),^29^ a variety of robust XB catalysts such as Huber’s *bis*-benzoimidazolium salts **G** and **H** (Table 1, entry 12 and 13),^30^ monodentate imidazolium salt **I** (Table 1, entry 14)^31^ which had previously been proven useful in furano- and pyranosylations^13,14^ did not yield any desired product in the septanosylation. The more Lewis acidic hypervalent iodine(III) catalyst **J** (Table 1, entry 15)^32^ also did not yield the desired product.

To better understand the nature of the noncovalent activation, a series of control experiments using poisoning additives specific to sigma hole inhibition were essential.^33^ Employment of 20 mol % of phosphines such as PPh_3_ and BINAP shut down the septanosylation completely (Table 1, entries 16 and 17).^33b^ Further, the addition of tetrabutyl ammonium chloride (TBAC) had an analogous inhibitory effect on the catalyst (Table 1, entry 18) due to the documented substantially higher affinity of halides to chalcogen bonding donors.^34^ These poisoning controls support the postulate that ChB activation is operative in our reaction. Addition of 20 mol % of the inorganic base K_2_CO_3_ also terminated the reaction (Table 1, entry 19) which suggests that the catalytic mode involves protic elementary steps.

We attribute this observation to the “proton mopping” effect of the base, rather than trace acid influences, which constrict proton transfer elementary steps. A control experiment using 2 mol % TsOH·H_2_O as the catalyst did not result in any observable reaction, trace acid catalysis is hence unlikely to be operative (see the Supporting Information Supplementary Table 1). The use of more forceful conditions such as 20 mol % of TsOH·H_2_O gave only a 28% yield of **3a** with substantial decomposition, as conversion was 89%. This ascertains the unsuitability of harsher employment of Brønsted acid catalysis in this protocol. Employing exact conditions from previously reported TMSOTf-mediated septanosylation to access **3d** resulted only in decomposition, and there was no observable product (see the Supporting Information Supplementary Figure 4-2).^23b^

### 2.2 Evaluation of Substrate Scope

With an optimized protocol in hand, we proceeded to expand the substrate scope (Table 2). Delightfully, we observed a robust performance that is amenable to a vast substrate scope. Of greater significance is that this method yields septanosides with excellent α-anomeric selectivity from glucosyl as well as galactosyl donors and tolerates a sizable range of acceptors.

**Table 2:**
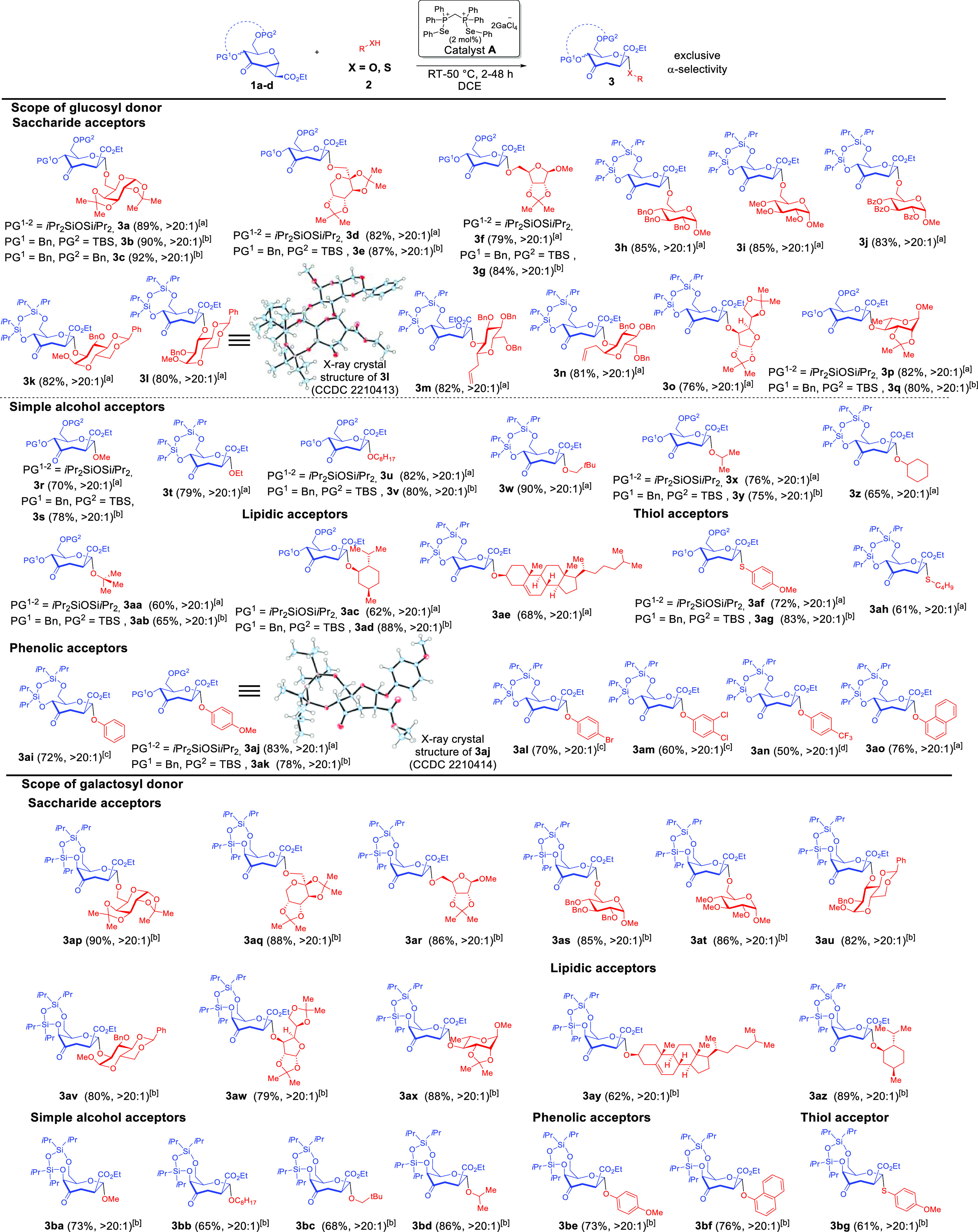
Substrate Scope^a^

a Conditions: glycosyl donor **1** (0.2 mmol), glycosyl acceptor **2** (0.4 mmol), and catalyst **A** (2 mol %) in DCE (1 mL), argon. Isolated yields after silica gel chromatography, α/β ratios are shown in parentheses, respectively. α/β ratios were determined by crude ^1^H NMR spectra analysis. [a] 35 °C [b] RT. [c] 50 °C [d] 45 °C, catalyst **A** (5 mol %). Me = methyl. Et = ethyl. *i*Pr = isopropyl. TBS = *tert*-butyldimethylsilyl. RT = room temperature. For X-ray structures, thermal ellipsoids shown at 50% probability and the monomeric component of the dimeric unit cell for **3l** is displayed.

A wide variety of naturally occurring scaffolds representative of rich biologically relevant glycosidic chemical space were tolerated in the acceptor fragment. This include d-galactopyranoses (**3a–3c** and **3ap**), d-fructopyranoses (**3d–3e** and **3aq**), d-ribofuranoses (**3f–3g** and **3ar**), d-glucopyranoses (**3h–3n** and **3as–3av**), d-glucofuranoses (**3o** and **3aw**), and l-rhamnopyranose (**3p–3q** and **3ax**). These examples also involve primary and secondary alcohols in various chemical environments as well as configurational modifications at the anomeric positions such as in analogues **3m–3n**.

Furthermore, biologically important chiral lipidic alcohols such as monoterpene menthol (**3ac–3ad** and **3az**) and cholesterol (**3ae** and **3ay**) can also be incorporated. We additionally investigated a series of commonly available primary (**3r–3w** and **3ba–3bc**), secondary (**3x–3z** and **3bd**), and tertiary alcohols (**3aa–3ab**) of varying steric hindrances as potential nucleophiles and delightfully noticed that the broad palette of simple alcohols were all amenable using our strategy.

Phenolic acceptors were also well tolerated, encompassing unsubstituted (**3ai**), substituted phenols bearing electron-donating (**3aj**, **3ak** and **3be**), electron-withdrawing substituents (**3al**, **3am** and **3an**) and naphthols (**3ao** and **3bf**). The milder ChB catalyzed conditions, in contrast to harsher TMSOTf catalyzed methods,^26^ meant that thiol-based acceptors could also be easily assimilated opening access to *S*-septanosides **3af–3ah** and **3bg** with generally very good yields and exclusive α-selectivity. To ensure a vigorous stereochemical assignment, X-ray crystallography on derivatives **3l** and **3aj** unambiguously established the α-anomeric configuration.

### 2.3 Mechanistic Studies

**Figure 2:**
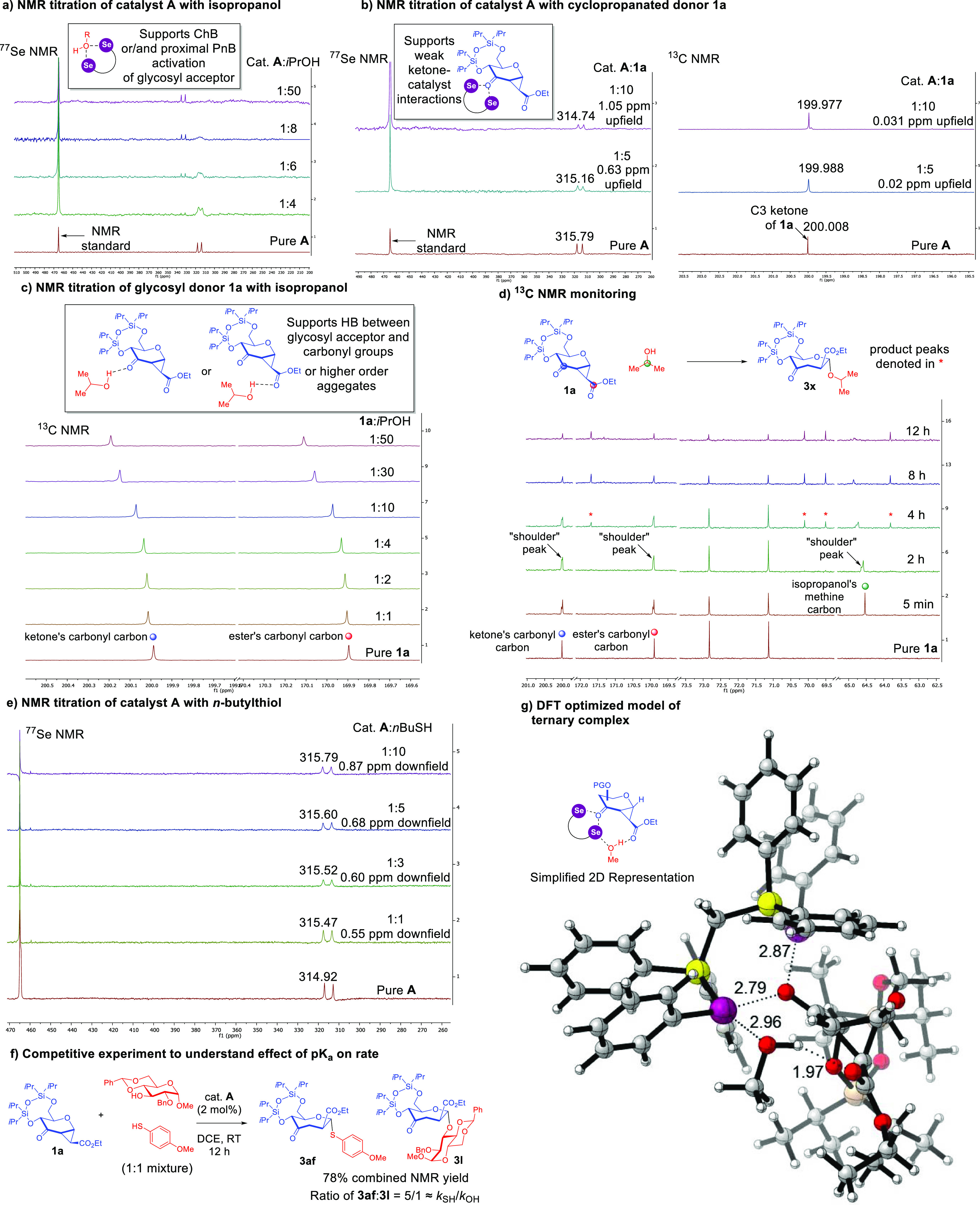
Control and NMR experiments to illuminate the role of unconventional NCIs in the mechanism. (a) NMR titration of catalyst **A** with isopropanol as a model glycosyl acceptor. (b) NMR titration of catalyst **A** with glycosyl donor **1a**. (c) NMR titration of glycosyl donor **1a** with isopropanol as a model glycosyl acceptor. (d) ^13^C in situ NMR monitoring. (e) NMR titration of catalyst **A** with *n*-butylthiol. (f) Competitive experiment to understand the effect of p*K*_a_ on rate. (g) DFT optimized model of the catalyst-donor–acceptor complex (NCIs denoted using dotted lines and distances in Å were labeled. Atom colors: purple = selenium, yellow = phosphorus, brown = silicon, gray = carbon, white = hydrogen). All NMR experiments in the figure are conducted in CD_2_Cl_2_.

To illuminate the noncovalent influences operative in our method, we first conducted a series of NMR titrations using donor **1a** and 2-propanol as a model acceptor. Using ^77^Se NMR spectroscopy, a titration of catalyst **A** against increasing amounts of isopropanol (Figure 2a, see the Supporting Information Supplementary Figure 5-1) yielded a titration profile reminiscent of slow exchange on the ^77^Se NMR time scale,^35^ within which the doublet corresponding to the catalyst’s seleniums at 315 ppm broadens and diminished. A new downfield shifted doublet peak plausibly representing the catalyst-isopropyl alcohol complex at 333 ppm was observed. Using the ^77^Se titration data, we determined the binding constant to be 1.3 M^–1^. This observation supports the postulate that a glycosyl acceptor activation mode through ChB by catalyst **A** could be operative. Interestingly, a ^31^P NMR titration also revealed a broadening and diminishment of the phosphonium peak at 32.2 ppm and the emergence of a new major downfield peak at 36.5 ppm, suggesting a slow exchange on the ^31^P NMR time scale (see the Supporting Information Supplementary Discussion 1). As such, transient pnictogen bonding (PnB) interactions between the phosphorus atom of the phosphonium ion and the glycosyl acceptor could also play an ancillary role in the activation manifold.^36^ Due to the one-bond proximity of phosphorus to the selenium atom, we also do not exclude the possibility that the observed ^77^Se chemical shift perturbation consists of contributions from oxygen–phosphorus interactions.

Next, we conducted parallel ^77^Se and ^13^C NMR titrations between catalyst **A** and the glycosyl donor **1a** (Figure 2b, see the Supporting Information Supplementary Figures 7 and 8). Generally, the chemical shift perturbations were lower, but not negligible. The ^77^Se chemical shift perturbation observed was significantly lower (∼1 ppm) than the former titration against the glycosyl acceptor. Parallel data from ^13^C NMR titrations also corroborated that there was a small chemical shift (∼0.031 ppm) in the C3 ketone moiety. In light of previous knowledge of ketone activation by ChB catalysis,^33a^ a ketone binding manifold from catalyst **A** is likely operative although the apparent weaker interaction could be attributed to steric clashes with the disiloxane group.

A third set of ^13^C NMR titration between the glycosyl donor and isopropanol was conducted to better understand the occurrence of donor–acceptor NCI interactions (Figure 2c, see Supporting Information Supplementary Figure 9-1). Accordingly, we observed concurrently a downfield shift on both the ester carbonyl and the ketone ^13^C resonances. When attempting to evaluate the binding constant, we have managed to fit the titration data optimally to a 1:2 binding isotherm. This titration indicated that the alcohol could establish HB with the carbonyl groups on **1a**, possibly through higher-order aggregation.

To capture the actual catalytic manifold in real-time by which all three reagents, i.e., the glycosyl donor **1a**, isopropanol, and catalyst **A** are concomitantly reacting, we conducted an in situ ^13^C NMR monitoring experiment (Figure 2d, see the Supporting Information Supplementary Figure 10-1). Intriguing, we observed the appearance and subsequent disappearance of a downfield slow exchange “shoulder” peak (∼0.1 ppm) contemporaneously at three distinctive chemical resonances in the initial period: (a) ∼200 ppm corresponding to the C3 ketone, (b) ∼170 ppm corresponding to the apical ester on the cyclopropane, and (c) ∼64.5 ppm corresponding to the methine carbon on isopropanol, likely due to the formation of the noncovalent trimeric complex. The disappearance of this new set of peaks subsequently led to the appearance of product **3x**. The NMR titration series and the ^13^C monitoring collectively support the postulate of the establishment of a ternary assembly, within which a noncovalent catalytic activation network that involves both carbonyl groups, the isopropyl alcohol’s hydroxyl group, and a ChB activation of the isopropyl alcohol’s oxygen is operative. The magnitude of the discernible temporal downfield shifting of both carbonyl moieties is at a low ∼0.1 ppm range, values which are more in line with magnitudes of weaker NCIs based on our previous studies on keto-cyclopropanes.^13^ Further, magnitudes of downfield ^13^C shifting on the ketone carbon through distinctive protic mechanisms previously reported by us^13^ and others^37^ are significantly larger (2–8 ppm range) than our current observations. A control experiment by mixing trifluoroacetic acid and substrate **1a** in a 1:1 ratio also afforded a 1.4 ppm downfield shift (see the Supporting Information Supplementary Figure 11) on the ^13^C ketone resonance which could be attributed to the fundamental covalent (via Brønsted acidity) vs noncovalent differences in the activation mode. Further, even a control experiment using catalytic trifluoroacetic acid at conditions analogous to our catalytic protocol did not yield any product (see Supporting Information Supplementary Figure 11). As a consequence of the large difference in chemical shift perturbations and the negative control experiment, an alternative Brønsted acid hypothesis involving covalent protonation of the C3 ketone as a consequence of ChB activation of the hydroxyl moiety is highly unlikely in our protocol.

To further obtain evidence to support this postulated trimeric noncovalent assembly, we additionally conducted a two-parallel ^1^H NMR titration to study the hydroxyl proton on isopropanol (see Supporting Information Supplementary Figure 12-1). We first mixed donor **1a** with pure isopropanol in a 0.5:1 ratio; a 0.07 ppm shift on the hydroxyl proton resonance was observed. By increasing the amount of donor **1a**, the titration continued to yield a downfield shift of the OH proton resonance, which is in line with a HB-type titration profile.^38^ In the second parallel three-component NMR titration, after adding 2 mol % **A** to pure isopropanol, we noticed a 0.07 ppm shift on the hydroxyl proton resonance. In the subsequent titration, we added increasing amounts of glycosyl donor **1a** against this fixed ratio (1:0.02) of isopropanol/**A** (see Supporting Information Supplementary Figure 12-2). The titration continued to yield a downfield shift of the OH proton resonance, which is in line with an HB-type titration profile. We directly compared this three-component result with the former two-component titration between the glycosyl donor and isopropanol (see Supporting Information Supplementary Table 17) to yield a better understanding of the catalyst effect on the donor–acceptor complex. Particularly, there is a clear downfield chemical shift in the range of 0.3–0.5 ppm when the catalyst is present versus the absence of catalyst for all the ratios compared, which points toward a stronger HB in the presence of the catalyst. This set of NMR titration experiments collectively supports the following: (1) donor **1a** interacts with the glycosyl acceptor’s hydroxyl proton via HB; (2) catalyst **A** interacts with the HB donor–acceptor complex (consideration of both Supporting Information Supplementary Table 17 and Figure 2a) to strengthen the point (1) stated HB through establishing a trimeric assembly.

In a bid to reveal the noncovalent nature of the trimeric assembly detected in situ ^13^C NMR, we posited that a suitable catalytic poison^33a,33c^ with much higher affinity to the chalcogenide introduced at the time-point where these slow exchange peaks first appear (∼1 h) should disturb the noncovalent network and revert the assembly back into the initial substrates without product formation. As such, we spiked the reaction in an NMR tube using 10 mol % of PPh_3_ and TBAC respectively in two separate tubes independently (see the Supporting Information Supplementary Figures 10-2 to 10-5). We noted as hypothesized that both poisons performed similarly and diminished the new set of downfield peaks, and no traces of product were detected after 18 h. The detection of only pure donor **1a** peaks eventually in these controls further evidence the reversible noncovalent nature of the trimeric complex formed.

Since our method also accommodates thiol nucleophiles, where the larger sulfur atom is known to be more polarizable and possesses a more diffused electron cloud,^39^ we conducted an NMR titration between catalyst **A** and *n*-butylthiol to investigate possible noncovalent divergences when thiols were employed (Figure 2e). We noted a concentration-dependent fast exchange downfield shift on the ^77^Se NMR resonance, albeit with a smaller magnitude of downfield shift, ascertaining catalyst-thiol ChB interactions. We attribute the lower magnitude to possible contributions of polarization, orbital mixing, and dispersion components to the ChB interaction,^10b,40^ which may result in a larger shielding effect.

Since the spectrum of nucleophiles employed in our scope spans a considerable range from thiophenols (p*K*_a_ ∼ 6.6) to secondary alcohols (p*K*_a_ ∼ 16), we were interested in understanding the effect of acceptor p*K*_a_ on the reaction. As such, we designed a competitive experiment whereby **1a** is reacted with a 1:1 mixture of a representative secondary alcohol derived from d-glucose and *p*-methoxylthiophenol (Figure 2f). Since the rate constants of the parallel occurring reactions between both acceptors could be estimated by the ratios of the glycosylation products obtained, a measured 5:1 ratio of **3af/3l** in the crude ^1^H NMR suggested that an approximate 2-fold increase in p*K*_a_ resulted in a 5-fold rate acceleration. Taking into account the literature-known relation between lower p*K*_a_ and better HB donating ability,^41^ this competitive experiment supports the postulate that HB is directly involved in the rate-limiting step (rls). Further, this experiment also indicated that HB is a critical interaction within the ternary assembly that we propose.

To gain a visual insight into the geometric disposition of the ternary complex, we then modeled the catalyst-donor–acceptor ternary complex using ORCA^42^ at the M06-2X-D3(0)/def2-SVP/CPCM(1,2-dichloroethane) level of theory (Figure 2g).^43^ Our density functional theory (DFT) optimized geometry revealed an intriguing noncovalent network consisting of 3 chalcogen bonds, particularly with one selenium atom engaging in a rare bifurcated ChB between the ketone oxygen and the hydroxyl oxygen of the acceptor. Further, the hydroxyl group engages in an additional HB with the ester carbonyl oxygen. This suite of interactions was also confirmed by the IGMH analysis^44^ (see the Supporting Information, Computational Details Section), which revealed the participating intermolecular NCIs through colored isosurfaces. Some salient spatial features of this complex deserve further mention: (1) the glycosyl acceptor is positioned below the pyranoside plane to enable an α-facial attack; (2) an aromatic ring directly attached to a phosphorus is shielding the β-face, which could prevent β-facial nucleophilic approach and hinder a putative upfolding of the ester moiety during the ring opening. Such a ternary conformation is also in line with the transient peaks detected in our ^13^C NMR monitoring, as both carbonyl carbons and the α-carbon to the hydroxyl group will likely experience concurrent ^13^C chemical shift perturbations.

**Figure 3:**
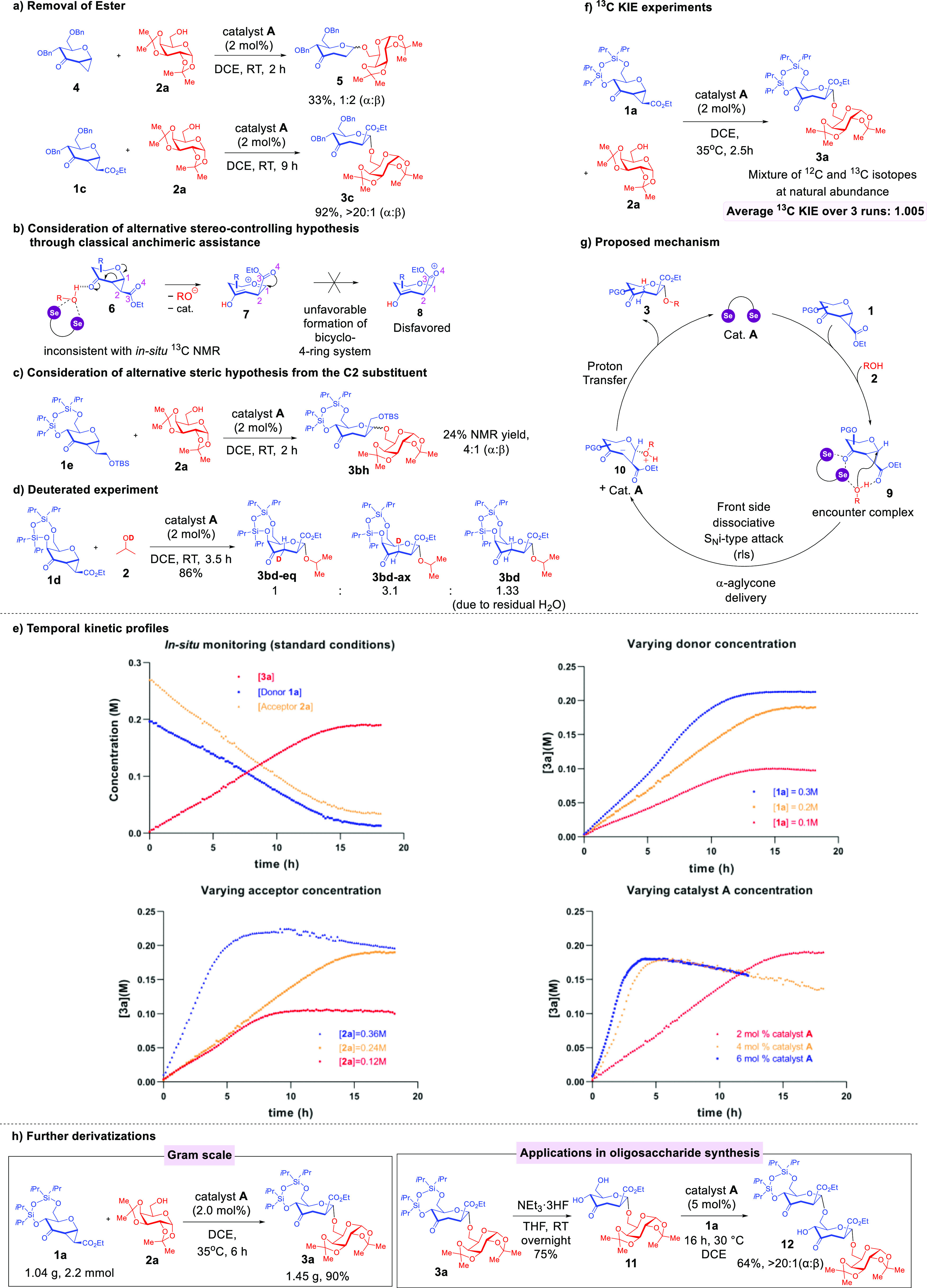
Control and deuterated experiments, ^13^C KIE experiment, temporal kinetic profiles, proposed mechanism, upscaling, and further derivatizations. (a) Control the experiment by removing the cyclopropyl ester moiety. (b) Considering alternative hypothesis through anchimeric assistance. (c) Considering alternative steric hypothesis from the C2 substituent. (d) Scrambling of deuterated labels on C3 suggested a stepwise mechanism. (e) Kinetic studies revealed positive orders with respect to the glycosyl donor, glycosyl acceptor, and catalyst. (f) ^13^C KIE studies support the postulate of a dissociative S_N_i-type mechanism. (g) Proposed mechanism. (h) Further derivatizations.

Next, we are interested in understanding the consistent excellent α-stereoselectivity observed in the substrate scope and its connection to the NCI manifold we unraveled. To this end, a series of control experiments were designed. First, by subjecting a cyclopropanated donor **4** with the ester moiety truncated to the exact reaction conditions of the ChB catalyzed protocol, we observed a huge diminishment of the anomeric selectivity to 1:2 (Figure 3a). Comparing the same reaction using donor **1c**, it is evident that the presence of an ester led to a marked increase in anomeric selectivity.

While considering an apparent anchimeric assistance-based pathway grounded in the liberation of Brønsted acid through catalyst activation of the glycosyl acceptor to rationalize the stereoselectivity might deceptively appear to be a plausible alternative hypothesis (Figure 3b), this hypothetical activation manifold would result in the cleavage of the cyclopropyl C1–C2 bond leading to intermediate **7**, and culminates in the forming of a thermodynamically implausible 4-ring intermediate **8**.^45^ Mechanistic entry into such oxepane-type systems would hence demand a highly unlikely scenario of overcoming a strain energy of 20 kcal mol^–1^ higher than the commonly encountered 5-ring dioxacarbenium ion congener in carbohydrate chemistry.^46^ Furthermore, our aforementioned in situ ^13^C NMR chemical shift magnitudes and contemporaneous appearance of downfield peaks on three resonances (Figure 2d) were not consistent with this hypothetical scenario.

Another stereocontrolling scenario whereby the β-steric hindrance imparted by the ester substituent through intermediate **7** was also considered (Figure 3c). To probe this possibility, we synthesized a control substrate **1e** which bears a sterically bulky OTBS substituent instead of an ester functionality to eliminate the hydroxyl–carbonyl HB that could guide the aglycone delivery based on our postulated ternary encounter complex. We hypothesized that **1e** should reproduce the exclusive α-selectivity should this alternative postulate be viable. While we did observe a modest stereoselectivity (4:1 α/β) in favor of the α-anomer, the α-selectivity is still a stark contrast compared to our entire substrate scope with a considerably less sterically hindered ester substituent. Meticulous scrutiny of all crude ^1^H NMR of our reactions using the ester substrates **1a–d** also ascertained that no β-anomer could be observed within the NMR detection limits. This suggests that steric contributions are likely marginal and is highly unlikely a compelling rationale to convincingly explain the consistent exclusive α-selectivity observed whenever ester-containing substrates were employed.

To gain deeper mechanistic insights, when we reacted **1d** with deuterated isopropanol under standard conditions using catalyst **A** (Figure 3d), the deuterium labels on C3 of the resulting septanoside **3** were scrambled in a ratio of ∼1:3, which is supportive of a stepwise addition rather than a concerted process. Further, we carried out a series of NMR monitoring experiments to untangle the kinetic profile of the septanosylation (Figure 3e), by performing the model reaction of **1a** and **2a** in CD_2_Cl_2_ (see the Supporting Information Supplementary Figure 15) and plotted the reaction profile at standard conditions. Upon permutating the concentration of donor **1a**, we noticed a positive correlation between the **1a** concentration and reaction rate. Similarly, by increasing the concentration of acceptor **2a**, we also noted a leftward shift of the temporal kinetic profile, consistent with a positive reaction order with respect to the glycosyl acceptor. Lastly, the increase in the reaction rate as a consequence of increasing catalyst loading is also in line with a positive reaction order with respect to catalyst **A**. By further computing the reaction orders (see the Supporting Information Supplementary Figures 26–30), we determined that the order is 0.6 with respect to donor **1a**, 1 with respect to the acceptor **2a** and 1.4 with respect to cat. **A**. These orders were reproducible in an independent replication of the exact kinetic study. We did however notice that while rates increased at higher catalyst loadings, there was also a diminishment of septanoside product **3a** when the reaction was allowed to run for longer durations, which suggests that excessive catalyst could result in product decomposition. In all, these NMR monitoring data holistically support the postulate that the glycosyl donor, glycosyl acceptor, and catalyst **A** are pivotally involved in the rate-limiting step (rls).

Finally, a major implication of all our above-mentioned mechanistic data would involve an asynchronous S_N_i-type mechanism to explain the excellent anomeric selectivity. To this end, we conducted competitive ^13^C KIE studies at natural abundance using quantitative ^13^C NMR technique (Figure 3f)—a technique used in the literature as a diagnostic indicator for S_N_i-type mechanisms^47^—on the model septanosylation between **1a** and **2a** through three reproducible replicates on a 600 MHz NMR and obtained an average of KIE of 1.005 which points toward a highly dissociative mechanism of either S_N_1 or S_N_i nature. By considering this KIE value concurrently with positive reaction orders determined for all substrates, as well as the implausibility of C2-steric hindrance serving as a productive stereocontrolling entity that a putative S_N_1 mechanism requires, we exclude the pure S_N_1 manifold and postulate that our mechanism is congruent with a dissociative concerted S_N_i mechanistic proposal whereby nonclassical NCIs between glycosyl donor, acceptor and catalyst **A** are involved in the α-aglycone delivery in the rls.

By virtue of our mechanistic data, we propose the following mechanism (Figure 3g). The mechanism commences by formation of ternary encounter complex **9** from the reacting substrates and catalyst **A**. This complex involves a noncovalent network comprising a bifurcated ChB activation between the alcohol oxygen of the glycosyl acceptor and the ketone; and a HB between the hydroxyl proton of the glycosyl acceptor and the ester. This is postulated by considering ^13^C NMR monitoring, NMR titration data, and DFT modeling collectively. The complex sets the stage for a subsequent rate-limiting, α-stereocontrolled, and retentive front-face dissociative S_N_i-type nucleophilic attack. Due to the disposition of the cyclopropyl group on the α-face, this noncovalent network would hereby position the glycosyl acceptor for an α-facial attack.

By virtue of this NCI-guided pathway, the aglycone would be selectively delivered to the anomeric carbon to form zwitterion **10**. This postulate is buttressed by a combination of both ^13^C KIE experiments and kinetic experiments. Subsequent to the glycosidic linkage formation, an unselective proton transfer to C3 of the septanoside as revealed by deuterated experiments would eventually yield **3**.

To demonstrate the upscaling utility of our protocol, we successfully reproduced the model septanosylation between **1a** and **2a** at a 1.04 g scale with comparable yields (90%) and excellent α-stereoselectivity (Figure 3h). Last but not least, our new strategy performed robustly in iterative oligosaccharide synthesis (Figure 3h). We first employed NEt_3_·3HF to cleave the disiloxane protecting group of **3a** to unmask the O5 and O7 hydroxyl groups to form **11**. Further, by resubjecting the glycosyl donor **1a** to the ChB catalysis condition with diol **11**, a regio- and stereoselective construction of the α(1 → 7)-glycosidic linkage could be achieved to generate trisaccharide **12**.

## 3 Conclusions

In conclusion, we demonstrate a remarkable noncovalent catalytic activation manifold whereby bifurcated ChB and HB can be harnessed synergistically to gain broad and stereoselective access into biologically important but hitherto synthetically challenging seven-ring containing septanosides. NMR titrations and in situ ^13^C NMR monitoring offered an intriguing view into the noncovalent network which is responsible for the superior α-aglycone delivery. Through ^13^C KIE studies, control substrates, and evaluating the kinetic orders with respect to the reactants, our data supports the postulate that a dissociative S_N_i-type mechanism is operative within our strategy. Our method thus paves a new direction whereby nonclassical NCIs could be effectively exploited in the realm of selective aglycone delivery, thus opening up synthetic routes toward valuable 7-ring glycomimetics.

## Supporting Information Available

Detailed experimental procedures, spectra data, crystallographic data of **3l**, **3aj**, and analytical data (PDF)

## Accession Codes

CCDC 2210413 and 2210414 contain the supplementary crystallographic data for this paper. These data can be obtained free of charge via www.ccdc.cam.ac.uk/data_request/cif, or by emailing data_request@ccdc.cam.ac.uk, or by contacting The Cambridge Crystallographic Data Centre, 12 Union Road, Cambridge CB2 1EZ, UK; fax: +44 1223 336033.
