# Supplementary material for: Cooperative Bifurcated Chalcogen Bonding and Hydrogen Bonding as Stereocontrolling Elements for Selective Strain-Release Septanosylation
Source: J Am Chem Soc. 2023 Nov 30;145(49):26611–22. doi: 10.1021/jacs.3c06984 (PMC10722516; doi:10.1021/jacs.3c06984)
Supplement: Supplementary file 1 — ja3c06984_si_001.pdf [file ja3c06984_si_001.pdf]

Supplementary Information

Cooperative bifurcated chalcogen bonding and hydrogen bonding as stereocontrolling elements for selective strain-release septanosylation

Wenpeng Ma,[†,‡] Jan-Lukas Kirchhoff,[#] Carsten Strohmann[#] Bastian Grabe[‡] and Charles C. J. Loh [†,‡*]

[†]Abteilung Chemische Biologie, Max-Planck-Institut für Molekulare Physiologie, Otto-Hahn-Straße 11, 44227, Dortmund, Germany.

[‡]Fakultät für Chemie und Chemische Biologie, Technische Universität Dortmund, Otto-Hahn-Straße 4a, 44227, Dortmund, Germany.

[#]Fakultät für Chemie und Chemische Biologie, Anorganische Chemie, Technische Universität Dortmund, Otto-Hahn-Straße 6, 44227 Dortmund, Germany

Contents

Supplementary Methods 3
General Information 3
General procedure for optimization of septanosylation 3
General procedure of additives effects 3
General procedure for chalcogen bonding catalyzed glycosylation 3
General procedures for control experiments without ester directing group 4
Competitive experiment between thiophenol and representative secondary alcohol saccharide 4
Method for titration experiments 5
Method for deuterated experiments 5
General method for *in-situ* NMR monitoring 6
NMR monitoring under standard condition 6
^{13}C NMR monitoring of standard reaction 6
^{13}C NMR in-situ poisoning of complex peaks with PPh_3 and TBAC 6
^{77}Se NMR monitoring of standard reaction 6
General method for concentration dependence studies: 6
General experimental procedure for the deprotection of disiloxane group 7
Method for synthesis of trisaccharide **12** 7
Optimization for ChB catalyzed septanosylation 8
X-Ray Crystallography Data 9
Synthesis of catalysts 46
Synthesis of cyclopropanated glycosyl donors 51
NMR data 62
NMR data for control experiment 93
Control experiment on ester-truncated donor **4** 93
Control experiment on OTBS donor **1e** 94
Using catalyst **A** on ester containing donor **1c** 99
Competitive experiment between thiophenol and representative secondary alcohol saccharide 100
Benchmarking our method with previously known TMSOTf promoted method 101
NMR data for titration experiments 103
^{77}Se NMR shift on catalyst **A** with isopropanol 103
^{77}Se NMR shift on catalyst **A** with butanethiol 105
^{31}P NMR shift of catalyst **A** 106
^{77}Se NMR shift on catalyst **A** with **1a** 107
^{13}C NMR shift of carbonyl group and ester group on donor **1a** with catalyst 108
^{13}C NMR shift of carbonyl carbon of donor **1a** with isopropanol 112
^{13}C NMR shift of carbonyl carbon of donor **1a** with butanethiol 114
^{13}C NMR monitoring of standard reaction 115
^{13}C NMR in-situ poisoning of complex peaks with PPh_3 and TBAC 116
^{77}Se-NMR monitoring of standard reaction 118
^{13}C NMR shift of carbonyl carbon of donor **1a** with trifluoroacetate 119
^{1}H NMR shift of hydroxyl signal on isopropanol 120
Deuterated experiments 123
NMR monitoring data for kinetics studies 124
Concentration dependence studies 128
Kinetic Analysis 145
Repetition of the kinetic study to ensure reproducibility 151
Kinetic Analysis (Repetition) 171
^{13}C KIE measurements 178
Computational Details 182

Supplementary Discussion 186
References 188
NMR Spectra 191

Supplementary Methods

General Information

Unless otherwise stated, all reactions were set up under inert atmosphere (argon) utilizing glassware that were oven dried and cooled under argon purging. Silica Gel Flash Column Chromatography was performed on *Silica gel Merck 60* (*particle size 40-63 μm.* Starting materials were purchased directly from commercial suppliers (Sigma Aldrich, Acros, Alfa Aesar, VWR, TCI) and used without further purifications unless otherwise stated. All solvents were dried according to standard procedures or brought from commercial suppliers. Reactions were monitored using thin-layer chromatography (TLC) on *Merck silica gel aluminium plates with F254 indicator*. Visualization of the developed plates was performed under UV light (254 nm) or $KMnO_4$ stain.

NMR characterization data (^{1}H NMR, ^{13}C NMR and 2D spectra) were collected at 300 K on a *Bruker DRX400 (400 MHz)*, *Bruker DRX500 (500 MHz)*, *INOVA500 (500 MHz), DRX600 (600 MHz)* and *Bruker DRX700 (700 MHz)* using $CDCl_3$ and CD_2Cl_2 as solvent. Data for ^{1}H NMR are reported as follows: chemical shift (δ ppm), multiplicity (s = singlet, d = doublet, t = triplet, q = quartet, m = multiplet, br = broad), coupling constant (Hz), referenced to the solvent resonance as internal standard ($CDCl_3$: δ = 7.26 ppm for ^{1}H, δ = 77.16 ppm for ^{13}C; CD_2Cl_2: δ = 5.32 ppm for ^{1}H, δ = 53.84 ppm for ^{13}C).

High resolution mass spectra were recorded on an *LTQ Orbitrap* mass spectrometer coupled to an *Accela HPLC*-System (HPLC column: *Hypersyl GOLD*, 50 mm x 1 mm, particle size 1.9 μm, ionization method: electron spray ionization) and Bruker ultrafleXtreme MALDI-TOF-TOF (3 decimal accuracy). Optical rotations were measured in a *Schmidt + Haensch Polartronic HH8* polarimeter equipped with a sodium lamp source (589 nm), and are reported as follows: $[\alpha]_D^{T\,°C}$ (*c* = g/100 mL, solvent).

The anomeric selectivity was determined by ^{1}H-NMR of the crude reaction mixture *via* integration of characteristic signals in the ^{1}H NMR spectra, Chemical yields refer to isolated substances after flash column chromatography. NMR yields were determined using 1,3,5-trimethoxybenzene or mesitylene as internal standard.

General procedure for optimization of septanosylation

(See Table 1, Entry 1-11, 17-31; Supplementary Table 1): A mixture of catalyst (2-20 mol%), cyclopropanated sugar **1a** (0.1 mmol, 1 equiv.) and glycosyl acceptor **2a** (0.2 mmol, 2 equiv.) was sealed in a dry tube under argon, then dry solvent (0.5 mL) was added. The mixture was stirred for indicated time. Upon completion of the reaction, the reaction mixture was filtered over a short silica plug and flushed with 10 mL of DCM. The filtrate was then evaporated and the determination of the anomeric selectivity (α/β) is by ^{1}H-NMR analysis of this concentrated crude mixture with 1,3,5-trimethoxybenzene as the internal standard.

General procedure of additives effects

(See Table 1, Entry 12-16; Supplementary Table 1): A mixture of catalyst **A** (2 mol%), cyclopropanated sugar **1a** (0.1 mmol, 1 equiv.), glycosyl acceptor **2a** (0.2 mmol, 2 equiv.), additive (PPh_3, BINAP, TBAC and K_2CO_3, 0.02 mmol, 0.2 equiv) were sealed in a dry tube under argon, then dry DCE (0.5 mL) was added. The mixture was stirred at 35 °C for 12 h. Upon completion of the reaction, the reaction mixture was filtered over a short silica plug and flushed with 10 mL of DCM. The filtrate was then evaporated and the determination of the anomeric selectivity (α/β) is by ^{1}H-NMR analysis of this concentrated crude mixture with 1,3,5-trimethoxybenzene as the internal standard.

General procedure for chalcogen bonding catalyzed glycosylation

Procedure A: To an oven dried dram vial purged with an argon balloon was charged catalyst **A** (4.48 mg, 0.004 mmol, 2 mol%), cyclopropanated sugar donor (0.2 mmol, 1 equiv.), glycosyl acceptor (0.4 mmol, 2 equiv.) and then dry DCE (1 mL) was added. The dram vial was sealed and the mixture was immersed in a **35 °C** heating block stirred for indicated time. Upon completion of the reaction, the reaction mixture was filtered over a short silica plug and flushed with 5-10 mL of DCM. The filtrate was

then evaporated and the determination of the anomeric selectivity (α/β) is by ^{1}H-NMR analysis of this concentrated crude mixture with 1,3,5-trimethoxybenzene as the internal standard. The crude mixture is subsequently loaded onto silica gel and subjected to flash column chromatography for purification.

Procedure B: To an oven dried dram vial purged with an argon balloon was charged catalyst **A** (4.48 mg, 0.004 mmol, 2 mol%), cyclopropanated sugar donor (0.2 mmol, 1 equiv.), glycosyl acceptor (0.4 mmol, 2 equiv.) and then dry DCE (1 mL) was added. The dram vial was sealed and stirred **at room temperature** for indicated time. Upon completion of the reaction, the reaction mixture was filtered over a short silica plug and flushed with 5-10 mL of DCM. The filtrate was then evaporated and the determination of the anomeric selectivity (α/β) is by ^{1}H-NMR analysis of this concentrated crude mixture with 1,3,5-trimethoxybenzene as the internal standard. The crude mixture is subsequently loaded onto silica gel and subjected to flash column chromatography for purification.

Procedure C: To an oven dried dram vial purged with an argon balloon was charged catalyst **A** (4.48 mg, 0.004 mmol, 2 mol%), cyclopropanated sugar donor (0.2 mmol, 1 equiv.), glycosyl acceptor (0.4 mmol, 2 equiv.) and then dry DCE (1 mL) was added. The dram vial was sealed and the mixture was immersed in a **50 °C** heating block stirred for indicated time. Upon completion of the reaction, the reaction mixture was filtered over a short silica plug and flushed with 5-10 mL of DCM. The filtrate was then evaporated and the determination of the anomeric selectivity (α/β) is by ^{1}H-NMR analysis of this concentrated crude mixture with 1,3,5-trimethoxybenzene as the internal standard. The crude mixture is subsequently loaded onto silica gel and subjected to flash column chromatography for purification.

General procedures for control experiments without ester directing group

(See manuscript **Figure 3a**, **Supplementary Figure 2**): To an oven dried dram vial purged with an argon balloon was charged catalyst **A** (4.48 mg, 0.004 mmol, 2 mol%), cyclopropanated sugar donor **4** (0.2 mmol, 1 equiv.), glycosyl acceptor **2a** (0.4 mmol, 2 equiv.) and then dry DCE (1 mL) was added. The dram vial was sealed and the mixture was stirred for 2h at room temperature. Upon completion of the reaction, the reaction mixture was filtered over a short silica plug and flushed with 10 mL of DCM. The filtrate was then evaporated and the determination of the anomeric selectivity (α/β) is by ^{1}H-NMR analysis of this concentrated crude mixture with 1,3,5-trimethoxybenzene as the internal standard. The crude mixture is subsequently loaded onto silica gel and subjected to flash column chromatography for purification.

(See manuscript **Figure 3a**, **Supplementary Figure 3-5**): To an oven dried dram vial purged with an argon balloon was charged catalyst **A** (4.48 mg, 0.004 mmol, 2 mol%), cyclopropanated sugar donor **1c** (0.2 mmol, 1 equiv.), glycosyl acceptor **2a** (0.4 mmol, 2 equiv.) and then dry DCE (1 mL) was added. The dram vial was sealed and the mixture was stirred for 9h at room temperature. Upon completion of the reaction, the reaction mixture was filtered over a short silica plug and flushed with 10 mL of DCM. The filtrate was then evaporated and the determination of the anomeric selectivity (α/β) is by ^{1}H-NMR analysis of this concentrated crude mixture with 1,3,5-trimethoxybenzene as the internal standard. The crude mixture is subsequently loaded onto silica gel and subjected to flash column chromatography for purification.

(See manuscript **Figure 3c**, **Supplementary Figure 3-1, Figure 3-2, Figure 3-3, Figure 3-4**): To an oven dried dram vial purged with an argon balloon was charged catalyst **A** (4.48 mg, 0.004 mmol, 2 mol%), cyclopropanated sugar donor **1e** (0.2 mmol, 1 equiv.), glycosyl acceptor **2a** (0.4 mmol, 2 equiv.) and then dry DCE (1 mL) was added. The dram vial was sealed and the mixture was stirred for 2h at room temperature. Upon completion of the reaction, the reaction mixture was filtered over a short silica plug and flushed with 10 mL of DCM. The filtrate was then evaporated and the determination of the anomeric selectivity (α/β) is by ^{1}H-NMR analysis of this concentrated crude mixture with 1,3,5-trimethoxybenzene as the internal standard. The crude mixture is subsequently loaded onto silica gel and subjected to flash column chromatography for purification.

Competitive experiment between thiophenol and representative secondary alcohol saccharide

(See manuscript **Figure 2h, Supplementary Figure 3-6**): To an oven dried dram vial purged with an argon balloon was charged catalyst **A** (4.48 mg, 0.004 mmol, 2 mol%), cyclopropanated sugar donor **1a** (0.2 mmol, 1 equiv.), glycosyl acceptor **2af** (0.2 mmol, 1.0 equiv.), glycosyl acceptor **2l** (0.2 mmol, 1.0 equiv.) and then dry DCE (1 mL) was added. The dram vial was sealed and the mixture was stirred

for 12h at room temperature. Upon completion of the reaction, the reaction mixture was filtered over a short silica plug and flushed with 10 mL of DCM. The filtrate was then evaporated and the determination of the anomeric selectivity (α/β) is by ^{1}H-NMR analysis of this concentrated crude mixture with 1,3,5-trimethoxybenzene as the internal standard.

Method for titration experiments

(see manuscript **Figure 2a**, **Supplementary Figure 5-1**, ^{77}Se NMR): To an NMR tube, filled with catalyst **A** (22.3 mg, 0.02 mmol) and varying amounts of isopropanol were added (Note: isopropanol to catalyst **A** molar ratios are 1:1, 2:1, 3:1, 4:1, 5:1, 6:1, 7:1, 8:1, 9:1, 10:1, 20:1, 50:1). Varying amounts of CD_2Cl_2 were added in order to reach a total volume of 500 µL. The detected ^{77}Se shift of catalyst **A** are shown respectively.

(see manuscript **Figure 2g**, **Supplementary Figure 5-3**, ^{77}Se NMR): To an NMR tube, filled with catalyst **A** (25.0 mg, 0.022 mmol) and varying amounts of n-butylthiol were added (Note: *n*-butylthiol to catalyst **A** molar ratios are 1:1, 3:1, 5:1,10:1). Varying amounts of CD_2Cl_2 were added in order to reach a total volume of 600 µL. The detected ^{77}Se shift of catalyst **A** are shown respectively.

(see **Supplementary Figure 6**, ^{31}P NMR): To an NMR tube, filled with catalyst **A** (22.3 mg, 0.02 mmol) and varying amounts of isopropanol were added (Note: isopropanol to catalyst **A** molar ratios are 1:1, 2:1, 3:1, 4:1, 5:1, 6:1, 7:1, 8:1, 9:1, 10:1, 20:1, 50:1). Varying amounts of CD_2Cl_2 were added in order to reach a total volume of 500 µL. The detected ^{31}P shifts of catalyst **A** are shown respectively.

(see manuscript **Figure 2b**, **Supplementary Figure 7**, ^{77}Se NMR): To an NMR tube, filled with catalyst **A** (22.3 mg, 0.02 mmol) and varying amounts of donor **1a** were added (Note: donor to catalyst **A** molar ratios are 1:1, 2:1, 3:1, 4:1, 5:1, 6:1, 10:1). Varying amounts of CD_2Cl_2 were added in order to reach a total volume of 500 µL. The detected ^{77}Se shift of catalyst **A** are shown respectively.

(see manuscript **Figure 2b**, **Supplementary Figure 8-1**, ^{13}C NMR): To an NMR tube, filled with catalyst **A** (22.3 mg, 0.02 mmol) and varying amounts of donor **1a** were added (Note: donor to catalyst **A** molar ratios are 1:1, 2:1, 3:1, 4:1, 5:1, 6:1, 10:1). Varying amounts of CD_2Cl_2 were added in order to reach a total volume of 500 µL. The detected ^{13}C shifts of donor **1a** are shown respectively.

(see manuscript **Supplementary Figure 8-2**, ^{13}C NMR): To an NMR tube, filled with donor **1a** (10 mg, 0.021 mmol) and varying amounts of catalyst **A** were added (Note: catalyst **A** to donor **1a** molar ratio are 1:20, 1:10, 1:5, 1:2.5, 1:1.67, 1:1, 1:0.67). Varying amounts of CD_2Cl_2 were added in order to reach a total volume of 600 µL. The detected ^{13}C shifts of donor **1a** are shown respectively.

(see manuscript **Supplementary Figure 8-3**, ^{13}C NMR): To an NMR tube, filled with donor **1a** (30 mg, 0.063 mmol) and varying amounts of catalyst **A** were added (Note: catalyst **A** to donor **1a** molar ratio are 1:20, 1:10, 1:5, 1:2). Varying amounts of CD_2Cl_2 were added in order to reach a total volume of 600 µL. The detected ^{13}C shifts of donor **1a** are shown respectively.

(see manuscript **Supplementary Figure 8-4**, ^{13}C NMR): To an NMR tube, filled with donor **1a** (60 mg, 0.127 mmol) and varying amounts of catalyst **A** were added (Note: catalyst **A** to donor **1a** molar ratio are 1:20,1:5). Varying amounts of CD_2Cl_2 were added in order to reach a total volume of 600 µL. The detected ^{13}C shifts of donor **1a** are shown respectively.

(see manuscript **Figure 2c**, **Supplementary Figure 9-1**, ^{13}C NMR): To an NMR tube, filled with donor **1a** (24 mg, 0.05 mmol) and varying amounts of isopropanol were added (Note: isopropanol to donor **1a** molar ratio are 1:1, 2:1, 3:1, 4:1, 6:1, 10:1, 20:1, 30:1, 50:1). Varying amounts of CD_2Cl_2 were added in order to reach a total volume of 500 µL. The detected ^{13}C shifts of donor **1a** are shown respectively.

(see manuscript **Figure 2c**, **Supplementary Figure 9-4**, ^{13}C NMR): To an NMR tube, filled with donor **1a** (24 mg, 0.05 mmol) and varying amounts of *n*-butanethiol were added (Note: *n*-butanethiol to donor **1a** molar ratio are 1:1, 2:1, 4:1, 8:1, 10:1, 20:1, 50:1). Varying amounts of CD_2Cl_2 were added in order to reach a total volume of 600 µL. The detected ^{13}C shifts of donor **1a** are shown respectively.

Method for deuterated experiments

(see manuscript **Figure 3d**, **Supplementary Figure 14**): To an oven dried dram vial purged with an argon balloon was charged catalyst **A** (4.48 mg, 0.004 mmol, 2 mol%), cyclopropanated sugar donor **1d** (95 mg, 0.2 mmol, 1 equiv.), glycosyl acceptor propan-2-ol-d (0.3 mmol, 1.5 equiv.) and then dry DCE (1 mL) was added. The dram vial was sealed and stirred **at room temperature** for 3.5 h. The reaction mixture was filtered over a short silica plug and flushed with 10 mL of DCM. The filtrate was then evaporated and the determination of the anomeric selectivity (α/β) is by ^{1}H-NMR analysis of this concentrated crude mixture with 1,3,5-trimethoxybenzene as the internal standard. The crude mixture is subsequently loaded onto silica gel and subjected to flash column chromatography for purification.

General method for *in-situ* NMR monitoring

NMR monitoring under standard condition

(see manuscript **Figure 3e**, **Supplementary Figure 15**) To a dry NMR tube, acceptor **2a** (31.2 mg, 0.012 mmol), catalyst **A** (2.2 mg, 0.002 mmol), cyclopropanated donor **1a** (47.3 mg, 0.1 mmol) and 1,1,2,2-tetrachloroethane (5.0 μL) as the internal standard, CD_2Cl_2 was added in order to reach a total volume of 500 μL. Afterwards, the tube was sealed with cap and measured on the NMR spectrometer at 35 ^{o}C by recording ^{1}H spectra at different time.

^{13}C NMR monitoring of standard reaction

(see manuscript **Figure 2d**, **Supplementary Figure 10-1**) To a dry NMR tube, isopropanol (6.0 mg, 0.01 mmol), catalyst **A** (2.2 mg, 0.002 mmol), cyclopropanated donor **1a** (47.3 mg, 0.1 mmol), CD_2Cl_2 was added in order to reach a total volume of 500 μL. Afterwards, the tube was sealed with cap and measured on the NMR spectrometer room temperature by recording ^{13}C spectra at different time.

^{13}C NMR in-situ poisoning of complex peaks with PPh_3 and TBAC

(see manuscript **Figure 2d**, **Supplementary Figure 10-2~ Supplementary Figure 10-5**) To a dry NMR tube, isopropanol (6.0 mg, 0.01 mmol), catalyst **A** (2.2 mg, 0.002 mmol), cyclopropanated donor **1a** (47.3 mg, 0.1 mmol), CD_2Cl_2 was added in order to reach a total volume of 500 μL. Afterwards, the tube was sealed with cap and measured on the NMR spectrometer room temperature by recording ^{13}C spectra after 1 hour, then add PPh_3 or TBAC (0.1eq, 0.01 mmol) in the reaction and measured ^{13}C-NMR at once and after 18h.

^{77}Se NMR monitoring of standard reaction

(see **Supplementary Figure 10-6**) To a dry NMR tube was charged cyclopropanated donor **1a** (47.3 mg, 0.1 mmol), catalyst **A** (2.2 mg, 0.002 mmol) and isopropanol (6 mg, 0.01 mmol, 1eq). CD_2Cl_2 was then added in order to reach a total volume of 0.5 mL. Afterwards, the tube was sealed with cap and measured on the NMR spectrometer at room temperature by recording ^{77}Se-NMR spectra every 1 hour. Each ^{77}Se-NMR spectrum acquisition involves 1000 scans and takes approximately 40 minutes

General method for concentration dependence studies:

Donor 1a concentration dependence: (see manuscript **Figure 3e**, **Supplementary Figure 17**) **experiment 1 (0.1M)**: To a dry NMR tube, acceptor **2a** (31.2 mg, 0.012 mmol), catalyst **A** (2.2 mg, 0.002 mmol), cyclopropanated donor **1a** (23.6 mg, 0.05 mmol) and 1,1,2,2-tetrachloroethane (5 μL) as the internal standard, CD_2Cl_2 was added in order to reach a total volume of 500 μL. Afterwards, the tube was sealed with cap and measured on the NMR spectrometer at 35 ^{o}C by recording ^{1}H spectra at different time.

Donor 1a concentration dependence: (see manuscript **Figure 3e**, **Supplementary Figure 18**) **experiment 2 (0.3M)**: To a dry NMR tube, acceptor **2a** (31.2 mg, 0.012 mmol), catalyst **A** (2.2 mg, 0.002 mmol), cyclopropanated donor **1a** (70.9 mg, 0.15 mmol) and 1,1,2,2-tetrachloroethane (5 μL) as the internal standard, CD_2Cl_2 was added in order to reach a total volume of 500 μL. Afterwards, the tube was sealed with cap and measured on the NMR spectrometer at 35 ^{o}C by recording ^{1}H spectra at different time.

Acceptor 2a concentration dependence: (see manuscript **Figure 3e**, **Supplementary Figure 20**) **experiment 3 (0.12M)**: To a dry NMR tube, acceptor **2a** (16 mg, 0.06 mmol), catalyst **A** (2.2 mg, 0.002 mmol), cyclopropanated donor **1a** (47.3 mg, 0.1 mmol) and 1,1,2,2-tetrachloroethane (5 μL) as the

internal standard, CD_2Cl_2 was added in order to reach a total volume of 500 μL. Afterwards, the tube was sealed with cap and measured on the NMR spectrometer at 35 ^{o}C by recording ^{1}H spectra at different time.

Acceptor 2a concentration dependence: (see manuscript **Figure 3e**, **Supplementary Figure 21**) **experiment 4 (0.36M)**: To a dry NMR tube, acceptor **2a** (46.9 mg, 0.18 mmol), catalyst **A** (2.2 mg, 0.002 mmol), cyclopropanated donor **1a** (47.3 mg, 0.1 mmol) and 1,1,2,2-tetrachloroethane (5 μL) as the internal standard, CD_2Cl_2 was added in order to reach a total volume of 500 μL. Afterwards, the tube was sealed with cap and measured on the NMR spectrometer at 35 ^{o}C by recording ^{1}H spectra at different time.

Catalyst A concentration dependence: (see manuscript **Figure 3e**, **Supplementary Figure 23**) **experiment 5 (4.0 mol%)**: To a dry NMR tube, acceptor **2a** (31.2 mg, 0.012 mmol), catalyst **A** (4.4 mg, 0.004 mmol), cyclopropanated donor **1a** (47.3 mg, 0.1 mmol) and 1,1,2,2-tetrachloroethane (5 μL) as the internal standard, CD_2Cl_2 was added in order to reach a total volume of 500 μL. Afterwards, the tube was sealed with cap and measured on the NMR spectrometer at 35 ^{o}C by recording ^{1}H spectra at different time.

Catalyst A concentration dependence: (see manuscript **Figure 3e**, **Supplementary Figure 24**) **experiment 6 (6.0 mol%)**: To a dry NMR tube, acceptor **2a** (31.2 mg, 0.012 mmol), catalyst **A** (6.6 mg, 0.006 mmol), cyclopropanated donor **1a** (47.3 mg, 0.1 mmol) and 1,1,2,2-tetrachloroethane (5 μL) as the internal standard, CD_2Cl_2 was added in order to reach a total volume of 500 μL. Afterwards, the tube was sealed with cap and measured on the NMR spectrometer at 35 ^{o}C by recording ^{1}H spectra at different time.

General experimental procedure for the deprotection of disiloxane group

A mixture of **3a** (147 mg, 0.2 mmol) and triethylamine trihydrofluoride (0.1 mL, 0.6 mmol) in THF (5 mL) was stirred at room temperature for overnight. After completion of the reaction, as indicated by TLC, was concentrated under reduced pressure and the crude compound was purified by column chromatography over silica gel to afford the pure alcohol **11**.

Method for synthesis of trisaccharide 12

To an oven dried dram vial purged with an argon balloon was charged catalyst **A** (5.6 mg, 0.005 mmol, 5 mol%), cyclopropanated sugar donor **1a** (47 mg, 0.1 mmol, 1 equiv.), glycosyl acceptor **11** (73.5 mg, 0.15 mmol, 1.5 equiv.) and then dry DCE (0.5 mL) was added. The dram vial was sealed and the mixture was immersed in a **30 °C** heating block stirred for 16 h. Upon completion of the reaction, the reaction mixture was filtered over a short silica plug and flushed with 15 mL of DCM. The filtrate was then evaporated and the determination of the anomeric selectivity (α/β) is by ^{1}H-NMR analysis of this concentrated crude mixture with 1,3,5-trimethoxybenzene as the internal standard. The crude mixture is subsequently loaded onto silica gel and subjected to flash column chromatography for purification.

Optimization for ChB catalyzed septanosylation

Supplementary Table 1:

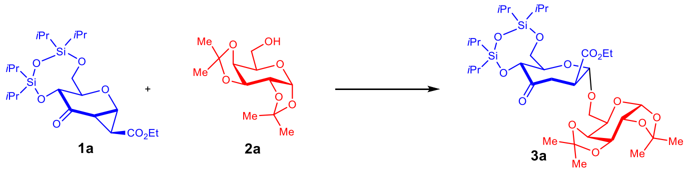

Entry	Cat (mol%)	Solvent	Additive (mol%)	Temp (°C)	Time (h)	Yield (**3a**)	α : β
1	**A** (10 %)	DCE	/	50	19	22%	>20:1
2	**B** (10 %)	DCE	/	50	19	15%	>20:1
3	**A** (10 %)	DCE	/	40	12	44%	>20:1
4	**A** (5 %)	DCE	/	40	12	64%	>20:1
5	**A** (5 %)	DCE	/	35	12	64%	>20:1
6	**A** (2 %)	DCE	/	35	12	89%	>20:1
7	**A** (2 %)	DCE	/	RT	12	54%	>20:1
8	**A** (2 %)	CH_2Cl_2	/	35	12	58%	>20:1
9	**A** (2 %)	CH_2Cl_2	/	RT	24	83%	>20:1
10	**A** (2 %)	Toluene	/	35	12	<5%	n.d.
11	**A** (2 %)	THF	/	35	12	65%	>20:1
12	**A** (2 %)	DCE	PPh_3 (20%)	35	12	<5%	n.d.
13	**A** (2 %)	DCE	(+)-BINAP (20%)	35	12	<5%	n.d.
14	**A** (2 %)	DCE	TBAC (20%)	35	12	<5%	n.d.
15	**A** (2 %)	DCE	K_2CO_3 (20%)	35	12	<5%	n.d.
16	**A** (2 %)	DCE	3 Å molecular sieve (50 mg)	35	12	<5%	n.d.
17	**B** (2 %)	DCE	/	35	12	12%	n.d.
18	**C** (2 %)	DCE	/	35	12	<5%	n.d.
19	**D** (2 %)	DCE	/	35	12	<5%	n.d.
20	**E** (2 %)	DCE	/	35	12	<5%	n.d.
21	**F** (10 %)	DCE	/	35	12	<5%	n.d.
22	**G** (5 %)	DCE	/	35	12	<5%	n.d.
23	**H** (5 %)	DCE	/	35	12	<5%	n.d.
24	**I** (5 %)	DCE	/	35	12	<5%	n.d.
25	**J** (5 %)	DCE	/	35	12	<5%	n.d.
26	**K** (5 %)	DCE	/	35	18	<5%	n.d.
27	TMSOTf (2 %)	DCE	/	35	12	22% (90% conv.)	n.d.
28	TMSOTf (20 %)	DCE	/	35	12	<5% (100% conv.)	n.d.
29	TsOH·H_2O (2 %)	DCE	/	35	12	<5% (<5% conv.)	n.d.
30	TsOH·H_2O (20 %)	DCE	/	35	12	28% (89% conv.)	n.d.
31	HOTf (20 %)	DCE	/	35	12	<5% (100% conv.)	n.d.

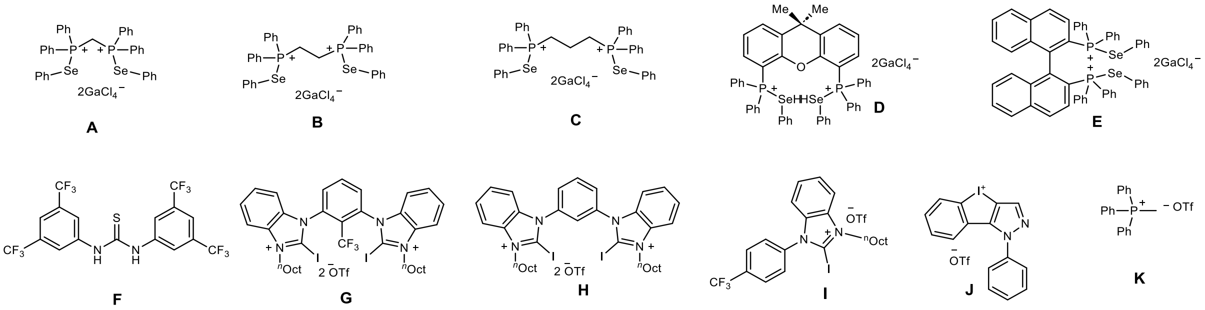

Conditions: **1a** (0.1 mmol), **2a** (0.2 mmol), catalyst, temp.; solvent (0.2 M), time, argon; yield and α/β ratio were determined by crude ^{1}H NMR spectra analysis using 1,3,5-trimethoxybenzene as an internal standard. TBAC= tetrabutylammonium chloride.

X-Ray Crystallography Data

(1) Crystal data for 3l (Crystal identifier cu_B2479_0m): CCDC Deposition Number is 2210413. Unit Cell Parameters: a 9.4016(10) b 38.732(4) c 12.2304(10) $P2_1$.

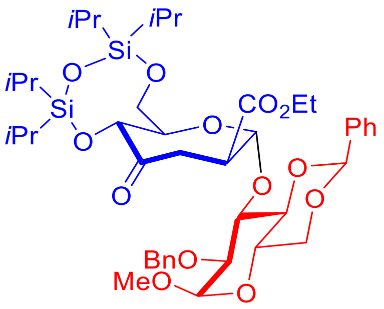

3l

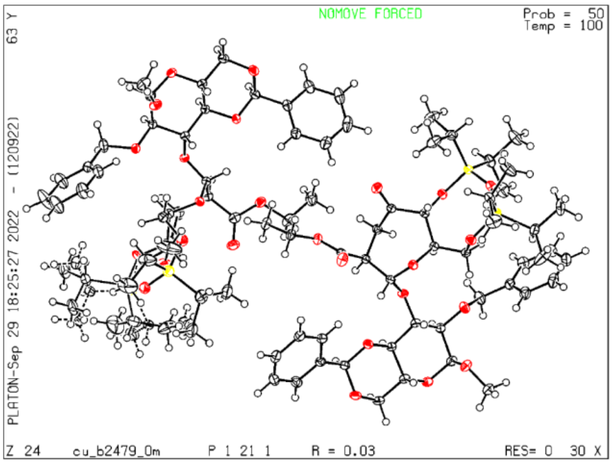

There is a crystallographic disorder on a disiloxane's isopropyl group which is resolved and leads to the structures **cu_B2479_0m-1** and crystal **cu_B2479_0m-2** in the ratio of 0.494 : 0.506 respectively (OLEX2 rendered structures of the two resolved parts are shown below).

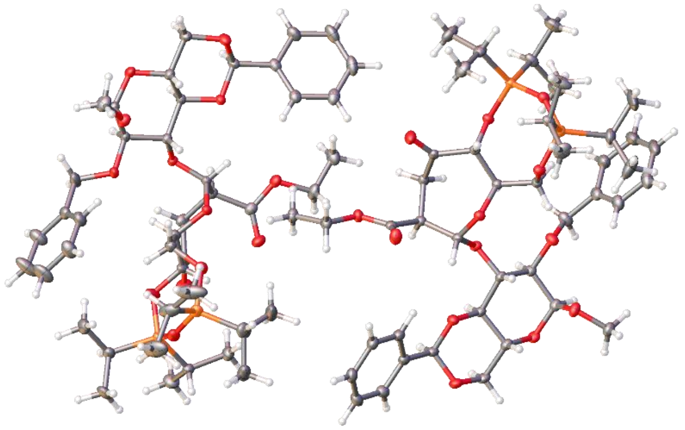

Supplementary Figure crystal cu_B2479_0m-1

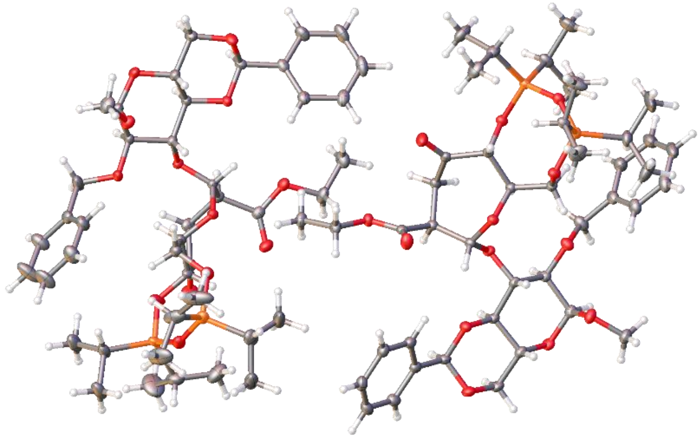

Supplementary Figure crystal cu_B2479_0m-2

Table 2 Crystal data and structure refinement for cu_B2479_0m.

Identification code	cu_B2479_0m
Empirical formula	$C_{43}H_{64}O_{13}Si_2$
Formula weight/gmol^{-1}	845.12
Temperature/K	100.0
Crystal system	monoclinic
Space group	$P2_1$
a/Å	9.4016(10)
b/Å	38.732(4)
c/Å	12.2304(10)
α/°	90
β/°	93.664(5)
γ/°	90
Volume/Å^3	4444.5(7)
Z	4
ρ_{calc}g/cm^3	1.263
μ/mm^{-1}	1.242
F(000)	1816.0
Crystal size/mm^3	0.416 × 0.116 × 0.111
Radiation	CuKα (λ = 1.54178)
2Θ range for data collection/°	4.562 to 149.832
Index ranges	-11 ≤ h ≤ 11, -48 ≤ k ≤ 48, -15 ≤ l ≤ 15
Reflections collected	148824
Independent reflections	18204 [R_{int} = 0.0354, R_{sigma} = 0.0159]
Data/restraints/parameters	18204/1/1131
Goodness-of-fit on F^2	1.040
Final R indexes [I>=2σ (I)]	R_1 = 0.0269, wR_2 = 0.0684
Final R indexes [all data]	R_1 = 0.0274, wR_2 = 0.0688
Largest diff. peak/hole / e Å^{-3}	0.24/-0.25
Flack parameter	-0.004(5)

Table 3 Fractional Atomic Coordinates (×10^4) and Equivalent Isotropic Displacement Parameters (Å^2×10^3) for cu_B2479_0m. U_{eq} is defined as 1/3 of the trace of the orthogonalised U_{IJ} tensor.

Atom	*x*	*y*	*z*	U(eq)
Si1	-1450.5(6)	-3315.3(2)	-9666.5(4)	17.38(11)
Si2	-4329.5(5)	-3132.3(2)	-8626.4(4)	16.68(10)
Si3	-7080.3(7)	-6550.7(2)	-5378.6(5)	25.78(13)
Si4B	-9984(3)	-6880.4(7)	-5738(3)	22.1(5)
O4	-3869.8(14)	-4322.9(4)	-10006.9(11)	16.8(3)
O17	-9746.8(14)	-5632.1(4)	-4919.2(11)	17.1(3)
O21	-11433.1(14)	-5394.2(4)	-3794.2(11)	16.5(3)
O7	-6557.4(15)	-4921.5(4)	-7822.5(12)	20.0(3)
O24	-10629.8(16)	-5856.0(4)	-2084.2(12)	20.7(3)
O26	-9540.9(17)	-4263.4(4)	-2220.0(12)	22.9(3)
O23	-8915.7(16)	-5478.4(4)	-696.2(12)	23.8(3)
O22	-10529.2(16)	-5018.2(4)	-649.0(11)	21.3(3)
O10	-3719.6(15)	-4477.4(4)	-14239.0(12)	22.8(3)
O20	-11287.3(16)	-4955.3(4)	-6892.7(12)	23.0(3)
O2	-1941.3(15)	-3711.8(4)	-10007.4(12)	20.0(3)
O11	-5106.4(15)	-4028.2(4)	-12914.9(12)	21.7(3)
O9	-5712.1(15)	-4839.1(4)	-14447.5(11)	20.2(3)
O13	-5118.5(16)	-5204.4(4)	-11702.8(11)	19.1(3)
O8	-5750.0(14)	-4495.6(4)	-11231.5(11)	17.3(3)
O1	-2777.7(15)	-3113.6(4)	-9148.0(11)	19.6(3)
O12	-5225.6(17)	-5656.5(4)	-12974.1(12)	22.9(3)
O3	-5123.6(14)	-3500.3(4)	-8994.8(11)	18.0(3)
O5	-5410.6(18)	-4079.2(4)	-7338.4(12)	28.5(3)
O15	-7723.0(17)	-6176.8(4)	-4992.4(13)	25.6(3)
O18	-12756.5(19)	-6205.7(5)	-6922.1(14)	34.2(4)
O25	-10127.2(15)	-4714.2(4)	-3438.8(11)	17.8(3)
O14	-8395.0(18)	-6812.1(4)	-5742.2(14)	28.7(3)
O6	-4290.1(16)	-4950.0(4)	-8287.0(13)	26.1(3)

O19	-11213.9(17)	-5503.6(4)	-7496.3(12)	26.7(3)
O16	-10904.5(17)	-6521.6(4)	-5452.3(15)	30.6(4)
C16	-5513(2)	-4065.7(5)	-8326.6(16)	18.0(4)
C20	-5483(2)	-4844.0(5)	-8443.9(16)	17.5(4)
C60	-12850(2)	-5726.2(6)	-5734.5(18)	23.3(4)
C18	-6032(2)	-4616.1(5)	-9391.4(16)	17.2(4)
C57	-10116(2)	-5982.9(5)	-4672.2(17)	19.1(4)
C80	-10305(2)	-4358.9(5)	-3213.7(16)	19.0(4)
C43	-4266(2)	-5667.7(6)	-10154.4(17)	22.8(4)
C34	-5642(2)	-4963.2(5)	-12512.5(16)	16.9(4)
C77	-10696(2)	-4918.0(5)	-2594.5(16)	16.8(4)
C14	-4315(2)	-3968.0(5)	-10097.9(16)	16.3(4)
C42	-3724(2)	-5900.1(6)	-9368.3(18)	26.1(5)
C86	-8910(2)	-4302.4(6)	-4910.4(18)	23.1(4)
C38	-5070(2)	-5785.2(6)	-11076.0(17)	20.7(4)
C37	-5657(2)	-5538.5(5)	-11947.7(17)	20.0(4)
C5	1288(3)	-3562.4(7)	-8954(2)	35.7(6)
C4	-3(2)	-3370.5(5)	-8568.6(18)	20.7(4)
C56	-8760(2)	-6150.7(6)	-4190.2(18)	23.7(4)
C41	-3988(2)	-6252.0(6)	-9494.5(19)	27.1(5)
C25	-5205(2)	-4495.7(6)	-14244.0(16)	19.7(4)
C24	-5655(2)	-4364.0(5)	-13132.5(16)	18.0(4)
C26	-3216(2)	-4444.3(6)	-15317.4(18)	25.4(4)
C64	-10887(3)	-4827.3(6)	-7953.9(18)	28.1(5)
C23	-5127(2)	-4603.5(5)	-12210.6(15)	17.0(4)
C66	-10533(2)	-5297.8(5)	-2854.4(15)	16.2(4)
C15	-4505(2)	-3836.0(5)	-8944.5(16)	15.9(4)
C81	-9726(2)	-4152.7(5)	-4133.4(17)	20.3(4)
C71	-10628(3)	-6442.4(6)	-1487.7(18)	24.4(4)
C58	-10698(2)	-6167.2(5)	-5713.3(18)	22.4(4)
C17	-6641(2)	-4272.0(5)	-8983.1(17)	18.5(4)

C27	-6049(2)	-3760.1(6)	-13301(2)	27.6(5)
C62	-10787(2)	-5373.5(5)	-4804.8(16)	16.6(4)
C68	-10351(2)	-5381.5(5)	-773.6(16)	20.4(4)
C61	-12011(2)	-5385.1(5)	-5690.9(16)	19.5(4)
C19	-4941(2)	-4574.1(5)	-10253.4(16)	16.7(4)
C63	-11461(2)	-5295.2(6)	-6804.5(17)	21.2(4)
C35	-5164(2)	-5074.7(5)	-13624.4(16)	19.5(4)
C59	-12140(2)	-6038.0(6)	-6209.7(18)	24.7(4)
C1	-1036(3)	-3081.6(6)	-10945.9(19)	30.5(5)
C85	-8388(2)	-4102.8(7)	-5741.7(18)	28.7(5)
C7	-5531(2)	-2795.6(5)	-9273(2)	25.1(4)
C67	-11016(2)	-5509.6(5)	-1893.2(16)	18.1(4)
C84	-8687(3)	-3753.1(7)	-5793(2)	35.0(6)
C33	-5948(3)	-3114.1(7)	-13256(2)	33.4(5)
C39	-5349(2)	-6135.8(6)	-11193.6(19)	24.6(4)
C40	-4809(2)	-6367.9(6)	-10407(2)	27.8(5)
C28	-5237(2)	-3425.4(6)	-13344.4(17)	24.4(4)
C13	-3216(2)	-3765.5(5)	-10688.0(16)	18.4(4)
C22	-7639(2)	-5256.5(6)	-6482.8(18)	24.4(4)
C78	-9912(2)	-4829.5(5)	-1503.8(16)	18.9(4)
C73	-8464(3)	-6775.5(7)	-1373(2)	37.2(6)
C70	-11386(3)	-6102.6(6)	-1476(2)	34.8(6)
C11	-5495(3)	-3076.6(8)	-6559(2)	37.4(6)
C10	-4069(2)	-3085.2(6)	-7102.7(17)	25.7(4)
C12	-3094(3)	-3359.4(7)	-6560.3(19)	35.0(5)
C76	-11406(3)	-6745.3(6)	-1529(2)	36.6(6)
C36	-5749(2)	-5434.8(6)	-13852.3(17)	22.4(4)
C69	-8325(3)	-5492.8(7)	405.9(19)	32.8(5)
C29	-3792(3)	-3423.5(6)	-13503.0(19)	28.9(5)
C30	-3061(3)	-3111.0(7)	-13566.9(19)	35.3(6)
C72	-9152(3)	-6459.3(6)	-1392(2)	32.3(5)

C21	-6248(2)	-5172.2(6)	-6952.7(17)	21.4(4)
C82	-10022(3)	-3800.5(6)	-4190(2)	28.3(5)
C9	-5718(3)	-2848.6(7)	-10518(2)	35.5(6)
C65	-11147(3)	-4444.1(7)	-7961(2)	33.1(5)
C79	-10062(2)	-4445.0(6)	-1302.9(17)	23.4(4)
C83	-9500(3)	-3601.3(7)	-5021(2)	36.8(6)
C47	-6032(3)	-6462.9(7)	-6588(2)	34.7(5)
C31	-3769(3)	-2802.7(7)	-13473(2)	40.0(6)
C8	-5006(3)	-2428.2(6)	-9012(3)	40.9(6)
C32	-5219(4)	-2802.6(7)	-13325(2)	41.4(6)
C74	-9239(3)	-7075.3(7)	-1423(3)	46.5(7)
C6	431(3)	-3028.2(7)	-8007(3)	40.3(7)
C49	-5961(4)	-6781.4(7)	-7341(3)	47.4(8)
C3	153(3)	-3247.9(11)	-11555(2)	52.2(9)
C75	-10718(3)	-7060.2(7)	-1483(3)	54.4(9)
C46	-5640(5)	-7128.2(9)	-4369(3)	64.4(11)
C48	-6554(4)	-6150.9(8)	-7229(3)	58.6(10)
C55A	-10153(6)	-6562.6(17)	-8279(5)	28.0(12)
C53A	-10487(6)	-6892.3(19)	-7619(7)	21.4(11)
C44	-5997(4)	-6745.9(8)	-4195(2)	47.7(8)
C2	-831(7)	-2693.3(10)	-10809(3)	88.4(17)
C45	-4652(4)	-6533.1(10)	-3913(3)	68.8(12)
C54A	-11966(6)	-7026.0(14)	-7991(5)	32.9(12)
C50A	-10973(5)	-7216.7(11)	-5373(4)	24.4(11)
C52A	-10338(7)	-7563.2(12)	-5714(5)	36.3(14)
C51A	-10792(8)	-7172.1(14)	-4137(5)	36.8(15)
C55B	-10189(7)	-6750(3)	-8038(6)	48(2)
C53B	-10501(8)	-7014(2)	-7163(7)	40.3(15)
C54B	-12054(9)	-7124(2)	-7336(8)	69(2)
Si4A	-10210(3)	-6844.1(7)	-6105(3)	17.1(5)
C51B	-11860(6)	-7269.6(14)	-4423(6)	43.8(16)

C52B	-9623(7)	-7566.0(13)	-4990(6)	43.5(17)
C50B	-10300(6)	-7220.0(12)	-4696(6)	29.6(13)

Table 4 Anisotropic Displacement Parameters (Å^2×10^3) for cu_B2479_0m. The Anisotropic displacement factor exponent takes the form: -2π^2[h^{2}a*^{2}U$_{11}$+2hka*b*U$_{12}$+…].

Atom	U_{11}	U_{22}	U_{33}	U_{23}	U_{13}	U_{12}
Si1	15.9(2)	17.7(2)	18.7(2)	0.4(2)	2.16(19)	-2.39(19)
Si2	16.9(2)	14.3(2)	18.8(2)	-0.89(19)	0.69(19)	0.03(19)
Si3	28.4(3)	21.3(3)	28.1(3)	2.1(2)	5.2(2)	8.6(2)
Si4B	23.6(10)	13.8(8)	29.4(14)	-4.0(9)	5.2(9)	-1.0(6)
O4	16.2(6)	15.7(7)	18.3(6)	-1.0(5)	0.1(5)	0.7(5)
O17	17.9(7)	14.0(6)	19.8(7)	-1.3(5)	3.5(5)	-0.2(5)
O21	17.5(6)	17.4(6)	14.9(6)	-1.9(5)	2.7(5)	-1.3(5)
O7	20.2(7)	20.3(7)	19.5(7)	4.0(6)	1.7(5)	-0.5(6)
O24	27.2(7)	14.8(7)	21.1(7)	2.5(5)	8.5(6)	1.2(6)
O26	30.7(8)	18.8(7)	18.8(7)	-1.6(6)	-3.0(6)	-5.4(6)
O23	24.2(7)	29.0(8)	17.9(7)	0.6(6)	-0.5(6)	5.3(6)
O22	28.5(8)	20.3(7)	15.6(6)	0.6(5)	5.7(6)	2.4(6)
O10	18.2(7)	33.8(8)	16.4(7)	2.2(6)	1.1(5)	-1.8(6)
O20	27.7(8)	25.0(8)	16.4(7)	2.2(6)	2.3(6)	3.4(6)
O2	17.9(7)	19.6(7)	22.4(7)	-3.0(6)	1.6(6)	-1.4(5)
O11	22.6(7)	18.5(7)	23.3(7)	1.1(6)	-5.1(6)	-2.1(6)
O9	21.6(7)	23.7(7)	15.0(6)	-1.9(6)	-2.3(5)	-0.4(6)
O13	23.5(7)	16.8(7)	16.8(6)	-1.6(5)	-0.4(5)	-0.7(5)
O8	19.2(6)	18.7(7)	13.9(6)	-1.8(5)	-1.2(5)	3.0(5)
O1	18.1(6)	17.3(6)	23.2(7)	0.3(6)	0.4(5)	-0.3(5)
O12	29.1(8)	21.0(7)	18.6(7)	-5.7(6)	1.8(6)	1.0(6)
O3	17.6(6)	15.5(7)	20.8(7)	0.0(5)	0.8(5)	0.9(5)
O5	39.6(9)	28.8(8)	17.4(7)	-0.5(6)	4.3(6)	-8.8(7)
O15	24.6(8)	20.6(7)	32.3(8)	2.5(6)	7.6(6)	6.5(6)
O18	30.7(9)	38.7(9)	33.4(9)	-15.8(7)	2.5(7)	-13.1(7)
O25	22.9(7)	14.4(6)	16.2(6)	0.5(5)	2.0(5)	-1.4(5)

O14	33.7(9)	20.0(8)	34.1(9)	1.5(6)	14.5(7)	5.8(6)
O6	18.8(7)	32.4(8)	27.1(8)	8.5(6)	1.2(6)	3.6(6)
O19	29.6(8)	30.5(8)	20.5(7)	-6.1(6)	4.7(6)	-0.8(6)
O16	27.2(8)	15.4(7)	51.0(10)	-11.2(7)	16.8(7)	-6.1(6)
C16	21.4(9)	14.1(9)	18.9(9)	-0.9(7)	4.1(7)	2.9(7)
C20	20.7(10)	15.2(9)	16.3(9)	-3.6(7)	-0.5(7)	-2.0(7)
C60	16.0(9)	29.0(11)	24.9(10)	-3.6(9)	1.1(8)	-2.5(8)
C18	19.0(9)	15.8(9)	16.6(9)	-1.3(7)	-0.2(7)	-0.5(7)
C57	22.3(10)	14.5(9)	21.2(10)	-1.5(7)	5.9(8)	-2.1(7)
C80	21.2(9)	16.1(9)	19.3(10)	-1.4(7)	-1.6(8)	-0.4(7)
C43	25.6(10)	22.4(10)	20.9(10)	-3.3(8)	4.2(8)	-2.3(8)
C34	17.3(9)	18.8(9)	14.6(9)	-1.9(7)	0.7(7)	0.3(7)
C77	18.9(9)	15.7(9)	16.1(9)	0.6(7)	2.2(7)	-0.1(7)
C14	17.0(9)	15.4(9)	16.4(9)	-0.8(7)	0.3(7)	0.1(7)
C42	29.1(11)	29.4(11)	20.2(10)	0.2(8)	4.1(9)	-2.4(9)
C86	23.4(10)	23.8(10)	21.6(10)	0.5(8)	-2.8(8)	-5.2(8)
C38	19.6(9)	21.6(10)	21.3(10)	-2.8(8)	4.8(8)	-0.9(8)
C37	21.5(10)	18.4(10)	20.0(9)	-5.4(8)	2.3(8)	-1.4(8)
C5	24.1(11)	45.9(15)	36.7(13)	-1.2(11)	-0.4(10)	15.5(11)
C4	17.4(9)	16.6(9)	27.9(10)	-1.4(8)	-0.2(8)	0.1(7)
C56	29.8(11)	18.6(10)	23.2(10)	0.4(8)	4.9(9)	3.4(8)
C41	28.1(11)	28.4(12)	25.3(11)	6.3(9)	5.6(9)	0.4(9)
C25	18.2(9)	24.5(10)	16.0(9)	0.6(8)	-1.7(7)	-1.1(8)
C24	18.0(9)	19.3(10)	16.3(9)	-0.6(7)	-1.3(7)	-0.9(7)
C26	24.8(10)	31.5(12)	20.2(10)	0.6(8)	4.4(8)	-1.4(9)
C64	28.9(11)	36.3(13)	19.5(10)	8.4(9)	4.7(8)	5.6(10)
C23	17.2(9)	20.3(10)	13.5(9)	-2.0(7)	1.0(7)	0.9(7)
C66	18.4(9)	16.7(9)	13.7(9)	-0.7(7)	1.5(7)	-0.4(7)
C15	17.3(9)	13.5(9)	16.7(9)	-1.3(7)	0.4(7)	0.4(7)
C81	18.5(9)	19.6(10)	22.0(10)	2.4(8)	-5.3(8)	-3.6(8)
C71	34.4(12)	20.0(10)	19.2(10)	4.1(8)	3.8(9)	0.6(9)

C58	23.0(10)	17.0(10)	28.0(11)	-7.3(8)	7.9(8)	-6.2(8)
C17	17.4(9)	17.2(9)	21.1(9)	0.9(7)	3.4(7)	0.2(7)
C27	25.7(11)	21.2(11)	35.0(12)	3.3(9)	-4.4(9)	0.0(9)
C62	19.5(9)	14.3(9)	16.3(9)	-1.4(7)	3.6(7)	0.1(7)
C68	23.7(10)	20.3(10)	17.4(9)	0.8(8)	3.7(8)	3.0(8)
C61	19.0(9)	20.8(10)	18.6(9)	-1.7(8)	2.4(8)	1.2(8)
C19	20.3(9)	14.5(9)	15.1(9)	-1.3(7)	-0.9(7)	1.4(7)
C63	17.4(9)	27.5(11)	18.2(9)	-0.8(8)	-1.4(7)	1.9(8)
C35	19.2(9)	24.2(10)	15.0(9)	-2.3(8)	0.6(7)	1.4(8)
C59	23.0(10)	26.4(11)	25.2(10)	-5.1(9)	6.3(8)	-9.5(9)
C1	34.2(12)	33.3(13)	24.6(11)	4.9(9)	6.4(9)	-10.7(10)
C85	25.3(11)	39.4(13)	20.8(10)	3.7(9)	-2.9(8)	-9.3(10)
C7	19.4(10)	17.3(10)	38.2(12)	2.5(9)	-0.1(9)	2.6(8)
C67	21.2(9)	15.6(9)	17.8(9)	1.7(7)	4.7(7)	0.7(7)
C84	31.9(12)	40.6(14)	31.5(12)	18.0(11)	-5.9(10)	-10.1(11)
C33	45.9(14)	24.8(11)	30.3(12)	1.4(10)	8.9(10)	-1.0(11)
C39	23.0(10)	22.9(11)	28.0(11)	-3.3(9)	1.9(8)	-3.0(8)
C40	29.4(11)	19.2(10)	35.2(12)	1.7(9)	6.2(9)	-3.4(9)
C28	33.7(12)	23.2(11)	15.9(9)	2.3(8)	-2.1(8)	-4.1(9)
C13	20.0(9)	19.5(9)	15.7(9)	-1.0(7)	1.2(7)	-2.7(8)
C22	21.9(10)	30.3(11)	21.1(10)	4.8(8)	2.3(8)	-3.7(9)
C78	22.8(10)	18.8(10)	15.1(9)	-0.3(7)	2.3(7)	0.2(8)
C73	31.4(13)	30.2(13)	49.2(15)	8.9(11)	-3.8(11)	1.9(10)
C70	38.3(13)	19.5(11)	49.1(15)	5.9(10)	21.8(12)	-1.2(9)
C11	30.5(12)	56.5(17)	26.3(12)	-9.8(11)	9.6(9)	-2.8(12)
C10	25.6(10)	30.7(12)	20.8(10)	-6.8(9)	2.1(8)	-6.3(9)
C12	36.2(13)	48.8(15)	19.8(10)	1.2(10)	-0.6(9)	3.8(11)
C76	34.5(13)	23.3(12)	50.1(15)	2.7(11)	-10.7(11)	-0.2(10)
C36	25.6(10)	24.8(11)	16.6(9)	-5.9(8)	1.0(8)	0.1(8)
C69	32.7(12)	44.4(15)	20.6(11)	2.4(10)	-3.8(9)	2.1(11)
C29	33.0(12)	28.8(12)	24.4(11)	6.0(9)	-1.9(9)	-3.6(10)

C30	38.5(13)	41.3(14)	25.3(11)	9.0(10)	-3.8(10)	-15.5(11)
C72	35.5(13)	22.9(11)	39.2(13)	2.9(10)	7.0(10)	-5.0(10)
C21	21.2(10)	25.7(11)	17.0(9)	4.1(8)	-1.7(8)	-1.9(8)
C82	27.1(11)	20.5(11)	36.7(12)	4.6(9)	-1.9(9)	1.9(9)
C9	36.4(13)	30.7(12)	37.7(13)	8.7(10)	-10.3(11)	4.8(10)
C65	37.4(13)	33.5(13)	27.6(12)	8.8(10)	-5.6(10)	-4.7(10)
C79	33.0(11)	19.2(10)	17.9(9)	-2.7(8)	0.4(8)	-1.2(8)
C83	36.0(13)	24.6(12)	48.4(15)	16.0(11)	-7.1(11)	-3.1(10)
C47	24.7(11)	34.2(13)	46.5(15)	0.8(11)	13.0(10)	-0.1(10)
C31	67.0(19)	28.9(13)	24.1(12)	2.4(10)	3.8(12)	-20.0(13)
C8	48.0(16)	17.4(11)	56.5(17)	0.2(11)	-1.8(13)	3.1(10)
C32	66.4(19)	21.1(12)	37.9(14)	-1.2(10)	11.9(13)	-5.9(12)
C74	45.0(16)	20.5(12)	72(2)	9.5(12)	-11.0(14)	7.5(11)
C6	32.9(13)	24.4(12)	60.5(17)	-10.0(11)	-22.4(12)	2.3(10)
C49	63.3(19)	31.0(14)	51.9(17)	9.2(12)	35.1(15)	14.3(13)
C3	36.9(14)	95(3)	26.4(13)	8.3(14)	11.6(11)	3.1(15)
C75	43.9(16)	20.0(13)	96(3)	4.0(14)	-20.0(17)	-1.2(11)
C46	105(3)	40.9(17)	44.8(17)	2.8(14)	-13.6(18)	37.5(19)
C48	94(3)	27.5(14)	60(2)	13.1(13)	51.3(19)	11.3(15)
C55A	34(3)	29(3)	21(3)	3(2)	2.0(19)	-2(2)
C53A	24(3)	20(3)	21(3)	-4(2)	3(2)	-3(2)
C44	71(2)	38.9(15)	31.7(13)	-4.7(11)	-7.6(13)	29.7(15)
C2	176(5)	44(2)	50(2)	7.9(16)	40(3)	-47(3)
C45	85(3)	55(2)	61(2)	-26.7(17)	-38.3(19)	42.6(19)
C54A	35(3)	31(3)	32(3)	-5(2)	-7(2)	-6(2)
C50A	29(2)	17(2)	27(3)	0.3(17)	7(2)	3.8(17)
C52A	49(3)	13(2)	47(3)	2(2)	8(3)	0(2)
C51A	56(4)	28(3)	28(3)	6(2)	7(3)	-1(3)
C55B	40(4)	76(7)	27(4)	-10(4)	2(3)	2(5)
C53B	54(4)	31(4)	35(4)	-9(3)	1(3)	3(3)
C54B	76(5)	67(5)	60(5)	-8(4)	-21(4)	-36(4)

Si4A	18.9(9)	12.2(8)	20.4(12)	-0.3(8)	2.5(8)	-0.9(6)
C51B	33(3)	28(3)	74(4)	6(3)	24(3)	-2(2)
C52B	44(3)	19(2)	70(5)	8(2)	21(3)	4(2)
C50B	28(3)	18(2)	43(4)	3(2)	13(3)	-0.7(18)

Table 5 Bond Lengths for cu_B2479_0m.

Atom	Atom	Length/Å	Atom	Atom	Length/Å
Si1	O2	1.6496(15)	C80	C81	1.509(3)
Si1	O1	1.6343(15)	C43	C42	1.390(3)
Si1	C4	1.862(2)	C43	C38	1.393(3)
Si1	C1	1.870(2)	C34	C23	1.513(3)
Si2	O1	1.6308(15)	C34	C35	1.522(3)
Si2	O3	1.6579(15)	C77	C66	1.515(3)
Si2	C7	1.868(2)	C77	C78	1.521(3)
Si2	C10	1.873(2)	C14	C15	1.522(3)
Si3	O15	1.6500(16)	C14	C13	1.516(3)
Si3	O14	1.6371(19)	C42	C41	1.392(3)
Si3	C47	1.860(3)	C86	C81	1.386(3)
Si3	C44	1.875(3)	C86	C85	1.391(3)
Si4B	O14	1.517(3)	C38	C37	1.510(3)
Si4B	O16	1.686(3)	C38	C39	1.389(3)
Si4B	C53B	1.853(8)	C5	C4	1.524(3)
Si4B	C50B	1.868(7)	C4	C6	1.536(3)
O4	C14	1.439(2)	C41	C40	1.390(3)
O4	C19	1.419(2)	C25	C24	1.536(3)
O17	C57	1.439(2)	C24	C23	1.519(3)
O17	C62	1.413(2)	C64	C65	1.504(4)
O21	C66	1.432(2)	C66	C67	1.526(3)
O21	C62	1.414(2)	C81	C82	1.393(3)
O7	C20	1.336(2)	C71	C70	1.497(3)
O7	C21	1.456(2)	C71	C76	1.381(3)

O24	C67	1.413(2)	C71	C72	1.387(3)
O24	C70	1.428(3)	C58	C59	1.533(3)
O26	C80	1.421(2)	C27	C28	1.507(3)
O26	C79	1.436(3)	C62	C61	1.531(3)
O23	C68	1.398(3)	C68	C67	1.550(3)
O23	C69	1.426(3)	C61	C63	1.528(3)
O22	C68	1.426(3)	C35	C36	1.519(3)
O22	C78	1.429(2)	C1	C3	1.525(4)
O10	C25	1.398(2)	C1	C2	1.524(4)
O10	C26	1.435(2)	C85	C84	1.384(4)
O20	C64	1.461(2)	C7	C9	1.534(4)
O20	C63	1.332(3)	C7	C8	1.533(3)
O2	C13	1.430(2)	C84	C83	1.384(4)
O11	C24	1.418(2)	C33	C28	1.386(3)
O11	C27	1.426(3)	C33	C32	1.393(4)
O9	C25	1.429(3)	C39	C40	1.389(3)
O9	C35	1.430(2)	C28	C29	1.385(3)
O13	C34	1.426(2)	C22	C21	1.498(3)
O13	C37	1.414(2)	C78	C79	1.517(3)
O8	C23	1.429(2)	C73	C72	1.385(4)
O8	C19	1.409(2)	C73	C74	1.370(4)
O12	C37	1.419(2)	C11	C10	1.535(3)
O12	C36	1.437(3)	C10	C12	1.527(3)
O3	C15	1.424(2)	C76	C75	1.380(4)
O5	C16	1.207(3)	C29	C30	1.396(3)
O15	C56	1.430(3)	C30	C31	1.375(4)
O18	C59	1.205(3)	C82	C83	1.391(4)
O25	C80	1.415(2)	C47	C49	1.543(4)
O25	C77	1.430(2)	C47	C48	1.505(4)
O14	Si4A	1.740(3)	C31	C32	1.386(5)
O6	C20	1.198(3)	C74	C75	1.389(4)

O19	C63	1.203(3)	C46	C44	1.536(4)
O16	C58	1.425(3)	C55A	C53A	1.554(9)
O16	Si4A	1.640(3)	C53A	C54A	1.525(7)
C16	C15	1.534(3)	C53A	Si4A	1.862(8)
C16	C17	1.516(3)	C44	C45	1.530(6)
C20	C18	1.520(3)	C50A	C52A	1.537(6)
C60	C61	1.538(3)	C50A	C51A	1.520(7)
C60	C59	1.514(3)	C50A	Si4A	1.866(5)
C18	C17	1.546(3)	C55B	C53B	1.521(12)
C18	C19	1.525(3)	C53B	C54B	1.522(11)
C57	C56	1.516(3)	C51B	C50B	1.537(7)
C57	C58	1.530(3)	C52B	C50B	1.536(7)

Table 6 Bond Angles for cu_B2479_0m.

Atom	Atom	Atom	Angle/˚	Atom	Atom	Atom	Angle/˚
O2	Si1	C4	104.78(8)	C77	C66	C67	108.75(16)
O2	Si1	C1	108.00(10)	O3	C15	C16	106.70(15)
O1	Si1	O2	109.58(8)	O3	C15	C14	109.77(16)
O1	Si1	C4	108.42(9)	C14	C15	C16	112.01(16)
O1	Si1	C1	107.37(10)	C86	C81	C80	122.09(19)
C4	Si1	C1	118.49(10)	C86	C81	C82	119.6(2)
O1	Si2	O3	109.30(8)	C82	C81	C80	118.4(2)
O1	Si2	C7	109.58(9)	C76	C71	C70	119.7(2)
O1	Si2	C10	108.76(9)	C76	C71	C72	119.2(2)
O3	Si2	C7	103.62(9)	C72	C71	C70	121.0(2)
O3	Si2	C10	112.46(9)	O16	C58	C57	108.03(18)
C7	Si2	C10	112.99(11)	O16	C58	C59	105.87(17)
O15	Si3	C47	106.83(10)	C57	C58	C59	115.95(17)
O15	Si3	C44	108.88(11)	C16	C17	C18	111.35(16)
O14	Si3	O15	109.67(9)	O11	C27	C28	109.44(18)
O14	Si3	C47	109.05(11)	O17	C62	O21	112.66(15)

O14	Si3	C44	108.89(13)	O17	C62	C61	113.78(16)
C47	Si3	C44	113.47(14)	O21	C62	C61	105.74(15)
O14	Si4B	O16	112.06(18)	O23	C68	O22	112.18(17)
O14	Si4B	C53B	104.2(3)	O23	C68	C67	107.87(16)
O14	Si4B	C50B	108.9(3)	O22	C68	C67	111.50(16)
O16	Si4B	C53B	108.5(3)	C62	C61	C60	114.23(17)
O16	Si4B	C50B	109.4(3)	C63	C61	C60	111.53(17)
C53B	Si4B	C50B	113.7(4)	C63	C61	C62	109.99(16)
C19	O4	C14	116.06(14)	O4	C19	C18	115.19(16)
C62	O17	C57	118.09(15)	O8	C19	O4	111.70(15)
C62	O21	C66	114.96(14)	O8	C19	C18	105.06(16)
C20	O7	C21	116.24(16)	O20	C63	C61	110.35(17)
C67	O24	C70	114.03(16)	O19	C63	O20	125.2(2)
C80	O26	C79	111.21(16)	O19	C63	C61	124.5(2)
C68	O23	C69	112.97(17)	O9	C35	C34	109.50(16)
C68	O22	C78	111.74(15)	O9	C35	C36	110.29(16)
C25	O10	C26	112.88(15)	C36	C35	C34	107.48(17)
C63	O20	C64	116.58(17)	O18	C59	C60	120.3(2)
C13	O2	Si1	119.54(13)	O18	C59	C58	118.9(2)
C24	O11	C27	113.29(16)	C60	C59	C58	120.57(18)
C25	O9	C35	111.72(15)	C3	C1	Si1	113.7(2)
C37	O13	C34	110.45(15)	C2	C1	Si1	114.7(2)
C19	O8	C23	114.86(15)	C2	C1	C3	112.3(3)
Si2	O1	Si1	148.73(10)	C84	C85	C86	119.8(2)
C37	O12	C36	111.41(15)	C9	C7	Si2	110.76(16)
C15	O3	Si2	126.70(12)	C8	C7	Si2	112.43(17)
C56	O15	Si3	122.44(14)	C8	C7	C9	110.2(2)
C80	O25	C77	110.01(15)	O24	C67	C66	106.91(15)
Si3	O14	Si4A	145.53(13)	O24	C67	C68	110.90(16)
Si4B	O14	Si3	146.44(15)	C66	C67	C68	112.69(17)
C58	O16	Si4B	131.95(17)	C85	C84	C83	120.3(2)

C58	O16	Si4A	124.07(17)	C28	C33	C32	120.4(3)
O5	C16	C15	120.40(18)	C38	C39	C40	120.2(2)
O5	C16	C17	121.04(19)	C39	C40	C41	120.4(2)
C17	C16	C15	118.56(17)	C33	C28	C27	119.8(2)
O7	C20	C18	109.39(16)	C29	C28	C27	120.9(2)
O6	C20	O7	124.46(19)	C29	C28	C33	119.3(2)
O6	C20	C18	126.11(19)	O2	C13	C14	111.69(16)
C59	C60	C61	117.47(17)	O22	C78	C77	109.24(16)
C20	C18	C17	111.66(16)	O22	C78	C79	109.72(16)
C20	C18	C19	112.24(16)	C79	C78	C77	108.57(17)
C19	C18	C17	114.24(16)	C74	C73	C72	120.2(3)
O17	C57	C56	106.17(16)	O24	C70	C71	109.26(19)
O17	C57	C58	110.12(16)	C11	C10	Si2	111.80(16)
C56	C57	C58	111.76(17)	C12	C10	Si2	113.66(16)
O26	C80	C81	108.26(16)	C12	C10	C11	110.1(2)
O25	C80	O26	111.10(16)	C75	C76	C71	120.2(3)
O25	C80	C81	108.49(16)	O12	C36	C35	107.99(16)
C42	C43	C38	120.3(2)	C28	C29	C30	120.2(2)
O13	C34	C23	110.06(15)	C31	C30	C29	120.4(2)
O13	C34	C35	109.03(16)	C73	C72	C71	120.5(2)
C23	C34	C35	111.80(17)	O7	C21	C22	106.67(17)
O25	C77	C66	109.71(15)	C83	C82	C81	120.0(2)
O25	C77	C78	109.03(16)	O26	C79	C78	108.33(17)
C66	C77	C78	110.69(16)	C84	C83	C82	120.0(2)
O4	C14	C15	107.45(15)	C49	C47	Si3	111.93(18)
O4	C14	C13	109.07(16)	C48	C47	Si3	112.88(18)
C13	C14	C15	113.05(16)	C48	C47	C49	110.9(3)
C43	C42	C41	120.2(2)	C30	C31	C32	119.7(2)
C81	C86	C85	120.4(2)	C31	C32	C33	120.0(3)
C43	C38	C37	121.32(19)	C73	C74	C75	119.6(3)
C39	C38	C43	119.5(2)	C76	C75	C74	120.3(3)

C39	C38	C37	119.19(19)	C55A	C53A	Si4A	114.4(4)
O13	C37	O12	111.30(16)	C54A	C53A	C55A	109.6(6)
O13	C37	C38	108.64(16)	C54A	C53A	Si4A	113.4(5)
O12	C37	C38	107.97(16)	C46	C44	Si3	113.2(2)
C5	C4	Si1	113.14(16)	C45	C44	Si3	110.9(2)
C5	C4	C6	111.4(2)	C45	C44	C46	111.4(3)
C6	C4	Si1	112.61(15)	C52A	C50A	Si4A	112.2(3)
O15	C56	C57	110.89(17)	C51A	C50A	C52A	110.4(5)
C40	C41	C42	119.4(2)	C51A	C50A	Si4A	111.6(4)
O10	C25	O9	111.70(17)	C55B	C53B	Si4B	115.0(5)
O10	C25	C24	108.08(16)	C55B	C53B	C54B	108.4(7)
O9	C25	C24	110.83(16)	C54B	C53B	Si4B	113.6(6)
O11	C24	C25	110.70(16)	O14	Si4A	C53A	109.5(2)
O11	C24	C23	108.86(15)	O14	Si4A	C50A	109.5(2)
C23	C24	C25	111.03(16)	O16	Si4A	O14	103.64(17)
O20	C64	C65	106.79(19)	O16	Si4A	C53A	121.7(2)
O8	C23	C34	109.38(16)	O16	Si4A	C50A	100.3(2)
O8	C23	C24	108.15(16)	C53A	Si4A	C50A	111.5(3)
C34	C23	C24	107.27(15)	C51B	C50B	Si4B	115.6(4)
O21	C66	C77	111.05(16)	C52B	C50B	Si4B	111.6(5)
O21	C66	C67	106.80(15)	C52B	C50B	C51B	110.9(4)

Table 7 Torsion Angles for cu_B2479_0m.

A	B	C	D	Angle/˚	A	B	C	D	Angle/˚
Si1	O2	C13	C14	-117.00(16)	C56	C57	C58	O16	56.2(2)
Si2	O3	C15	C16	128.18(15)	C56	C57	C58	C59	174.73(18)
Si2	O3	C15	C14	-110.25(16)	C25	O9	C35	C34	60.7(2)
Si3	O15	C56	C57	-119.48(17)	C25	O9	C35	C36	178.75(16)
Si3	O14	Si4A	O16	-29.1(3)	C25	C24	C23	O8	-171.67(15)
Si3	O14	Si4A	C53A	102.2(4)	C25	C24	C23	C34	-53.8(2)
Si3	O14	Si4A	C50A	-135.4(3)	C24	O11	C27	C28	-163.29(18)

Si4B	O16	C58	C57	-105.5(3)	C26	O10	C25	O9	83.7(2)
Si4B	O16	C58	C59	129.7(2)	C26	O10	C25	C24	-154.17(17)
O4	C14	C15	O3	-172.45(14)	C64	O20	C63	O19	5.7(3)
O4	C14	C15	C16	-54.1(2)	C64	O20	C63	C61	-174.65(17)
O4	C14	C13	O2	-73.07(19)	C23	O8	C19	O4	83.15(19)
O17	C57	C56	O15	-66.3(2)	C23	O8	C19	C18	-151.30(16)
O17	C57	C58	O16	173.91(16)	C23	C34	C35	O9	-59.5(2)
O17	C57	C58	C59	-67.5(2)	C23	C34	C35	C36	-179.31(16)
O17	C62	C61	C60	59.6(2)	C66	O21	C62	O17	70.8(2)
O17	C62	C61	C63	-66.7(2)	C66	O21	C62	C61	-164.34(15)
O21	C66	C67	O24	69.59(19)	C66	C77	C78	O22	-62.4(2)
O21	C66	C67	C68	-168.33(15)	C66	C77	C78	C79	177.93(17)
O21	C62	C61	C60	-64.5(2)	C15	C16	C17	C18	83.9(2)
O21	C62	C61	C63	169.18(16)	C15	C14	C13	O2	46.4(2)
O7	C20	C18	C17	61.2(2)	C81	C86	C85	C84	-0.1(3)
O7	C20	C18	C19	-168.99(16)	C81	C82	C83	C84	0.0(4)
O26	C80	C81	C86	108.7(2)	C71	C76	C75	C74	-1.7(5)
O26	C80	C81	C82	-70.9(2)	C58	O16	Si4A	O14	69.0(3)
O23	C68	C67	O24	46.7(2)	C58	O16	Si4A	C53A	-54.6(4)
O23	C68	C67	C66	-73.1(2)	C58	O16	Si4A	C50A	-177.9(2)
O22	C68	C67	O24	170.27(15)	C58	C57	C56	O15	53.8(2)
O22	C68	C67	C66	50.5(2)	C17	C16	C15	O3	93.48(19)
O22	C78	C79	O26	-175.23(16)	C17	C16	C15	C14	-26.7(2)
O10	C25	C24	O11	55.2(2)	C17	C18	C19	O4	47.1(2)
O10	C25	C24	C23	-65.9(2)	C17	C18	C19	O8	-76.24(19)
O2	Si1	O1	Si2	15.2(2)	C27	O11	C24	C25	90.6(2)
O2	Si1	C4	C5	62.01(18)	C27	O11	C24	C23	-147.05(18)
O2	Si1	C4	C6	-170.53(18)	C27	C28	C29	C30	178.5(2)
O2	Si1	C1	C3	-59.7(2)	C62	O17	C57	C56	-146.22(16)
O2	Si1	C1	C2	169.2(3)	C62	O17	C57	C58	92.64(19)
O11	C24	C23	O8	66.2(2)	C62	O21	C66	C77	92.14(19)

O11	C24	C23	C34	-175.90(16)	C62	O21	C66	C67	-149.44(15)
O11	C27	C28	C33	-154.0(2)	C62	C61	C63	O20	-78.2(2)
O11	C27	C28	C29	27.9(3)	C62	C61	C63	O19	101.5(2)
O9	C25	C24	O11	177.87(15)	C68	O22	C78	C77	64.0(2)
O9	C25	C24	C23	56.8(2)	C68	O22	C78	C79	-177.09(17)
O9	C35	C36	O12	-177.04(15)	C61	C60	C59	O18	-128.5(2)
O13	C34	C23	O8	-65.86(19)	C61	C60	C59	C58	57.9(3)
O13	C34	C23	C24	177.07(15)	C19	O4	C14	C15	101.57(18)
O13	C34	C35	O9	178.59(15)	C19	O4	C14	C13	-135.55(16)
O13	C34	C35	C36	58.8(2)	C19	O8	C23	C34	91.08(18)
O1	Si1	O2	C13	52.23(15)	C19	O8	C23	C24	-152.41(16)
O1	Si1	C4	C5	178.96(16)	C19	C18	C17	C16	-68.0(2)
O1	Si1	C4	C6	-53.6(2)	C63	O20	C64	C65	164.71(19)
O1	Si1	C1	C3	-177.79(19)	C35	O9	C25	O10	60.7(2)
O1	Si1	C1	C2	51.1(3)	C35	O9	C25	C24	-59.9(2)
O1	Si2	O3	C15	47.32(17)	C35	C34	C23	O8	172.82(15)
O1	Si2	C7	C9	59.11(18)	C35	C34	C23	C24	55.7(2)
O1	Si2	C7	C8	-64.7(2)	C59	C60	C61	C62	-73.9(2)
O1	Si2	C10	C11	176.27(17)	C59	C60	C61	C63	51.6(3)
O1	Si2	C10	C12	-58.31(19)	C1	Si1	O2	C13	-64.43(16)
O3	Si2	O1	Si1	-21.0(2)	C1	Si1	O1	Si2	132.28(19)
O3	Si2	C7	C9	-57.45(17)	C1	Si1	C4	C5	-58.4(2)
O3	Si2	C7	C8	178.71(18)	C1	Si1	C4	C6	69.0(2)
O3	Si2	C10	C11	-62.5(2)	C85	C86	C81	C80	-179.54(19)
O3	Si2	C10	C12	62.90(19)	C85	C86	C81	C82	0.1(3)
O5	C16	C15	O3	-86.1(2)	C85	C84	C83	C82	-0.1(4)
O5	C16	C15	C14	153.75(19)	C7	Si2	O1	Si1	-133.90(18)
O5	C16	C17	C18	-96.5(2)	C7	Si2	O3	C15	164.08(16)
O15	Si3	O14	Si4B	-13.1(4)	C7	Si2	C10	C11	54.4(2)
O15	Si3	O14	Si4A	15.7(3)	C7	Si2	C10	C12	179.79(17)
O15	Si3	C47	C49	-153.5(2)	C67	O24	C70	C71	-164.12(19)

O15	Si3	C47	C48	-27.6(3)	C33	C28	C29	C30	0.4(3)
O15	Si3	C44	C46	166.5(3)	C39	C38	C37	O13	-174.30(18)
O15	Si3	C44	C45	-67.4(2)	C39	C38	C37	O12	-53.5(2)
O25	C80	C81	C86	-12.0(3)	C28	C33	C32	C31	-0.6(4)
O25	C80	C81	C82	168.39(18)	C28	C29	C30	C31	0.0(3)
O25	C77	C66	O21	-68.1(2)	C13	C14	C15	O3	67.1(2)
O25	C77	C66	C67	174.68(15)	C13	C14	C15	C16	-174.51(16)
O25	C77	C78	O22	176.78(15)	C78	O22	C68	O23	63.2(2)
O25	C77	C78	C79	57.2(2)	C78	O22	C68	C67	-57.9(2)
O14	Si3	O15	C56	52.24(19)	C78	C77	C66	O21	171.55(15)
O14	Si3	C47	C49	-35.1(2)	C78	C77	C66	C67	54.3(2)
O14	Si3	C47	C48	90.8(2)	C73	C74	C75	C76	2.2(6)
O14	Si3	C44	C46	47.0(3)	C70	O24	C67	C66	-160.74(19)
O14	Si3	C44	C45	173.1(2)	C70	O24	C67	C68	76.1(2)
O14	Si4B	O16	C58	34.5(4)	C70	C71	C76	C75	-176.6(3)
O14	Si4B	C53B	C55B	-58.6(6)	C70	C71	C72	C73	178.1(2)
O14	Si4B	C53B	C54B	175.5(6)	C10	Si2	O1	Si1	102.1(2)
O14	Si4B	C50B	C51B	168.4(4)	C10	Si2	O3	C15	-73.58(17)
O14	Si4B	C50B	C52B	-63.6(5)	C10	Si2	C7	C9	-179.45(16)
O6	C20	C18	C17	-121.2(2)	C10	Si2	C7	C8	56.7(2)
O6	C20	C18	C19	8.6(3)	C76	C71	C70	O24	-144.2(2)
O16	Si4B	O14	Si3	16.9(5)	C76	C71	C72	C73	1.9(4)
O16	Si4B	C53B	C55B	60.9(6)	C36	O12	C37	O13	-61.0(2)
O16	Si4B	C53B	C54B	-64.9(7)	C36	O12	C37	C38	179.81(16)
O16	Si4B	C50B	C51B	45.6(5)	C69	O23	C68	O22	78.6(2)
O16	Si4B	C50B	C52B	173.6(4)	C69	O23	C68	C67	-158.21(18)
O16	C58	C59	O18	-48.8(3)	C29	C30	C31	C32	-0.7(4)
O16	C58	C59	C60	124.9(2)	C30	C31	C32	C33	0.9(4)
C20	O7	C21	C22	-171.82(17)	C72	C71	C70	O24	39.6(3)
C20	C18	C17	C16	60.7(2)	C72	C71	C76	C75	-0.4(4)
C20	C18	C19	O4	-81.3(2)	C72	C73	C74	C75	-0.6(5)

C20	C18	C19	O8	155.34(15)	C21	O7	C20	O6	-4.7(3)
C60	C61	C63	O20	154.03(17)	C21	O7	C20	C18	172.91(16)
C60	C61	C63	O19	-26.3(3)	C79	O26	C80	O25	-62.3(2)
C57	O17	C62	O21	48.3(2)	C79	O26	C80	C81	178.66(17)
C57	O17	C62	C61	-72.0(2)	C47	Si3	O15	C56	170.29(17)
C57	C58	C59	O18	-168.5(2)	C47	Si3	O14	Si4B	-129.8(3)
C57	C58	C59	C60	5.1(3)	C47	Si3	O14	Si4A	-101.0(3)
C80	O26	C79	C78	58.6(2)	C47	Si3	C44	C46	-74.7(3)
C80	O25	C77	C66	178.67(15)	C47	Si3	C44	C45	51.5(2)
C80	O25	C77	C78	-60.0(2)	C32	C33	C28	C27	-178.2(2)
C80	C81	C82	C83	179.6(2)	C32	C33	C28	C29	-0.1(3)
C43	C42	C41	C40	-0.7(3)	C74	C73	C72	C71	-1.4(4)
C43	C38	C37	O13	6.2(3)	C55A	C53A	Si4A	O14	-70.1(4)
C43	C38	C37	O12	127.0(2)	C55A	C53A	Si4A	O16	50.7(5)
C43	C38	C39	C40	-1.1(3)	C55A	C53A	Si4A	C50A	168.6(4)
C34	O13	C37	O12	60.9(2)	C44	Si3	O15	C56	-66.8(2)
C34	O13	C37	C38	179.64(16)	C44	Si3	O14	Si4B	105.9(3)
C34	C35	C36	O12	-57.7(2)	C44	Si3	O14	Si4A	134.7(3)
C77	O25	C80	O26	62.5(2)	C44	Si3	C47	C49	86.5(2)
C77	O25	C80	C81	-178.64(15)	C44	Si3	C47	C48	-147.6(2)
C77	C66	C67	O24	-170.50(16)	C54A	C53A	Si4A	O14	163.2(4)
C77	C66	C67	C68	-48.4(2)	C54A	C53A	Si4A	O16	-76.0(5)
C77	C78	C79	O26	-55.9(2)	C54A	C53A	Si4A	C50A	41.9(5)
C14	O4	C19	O8	46.7(2)	C52A	C50A	Si4A	O14	-63.8(5)
C14	O4	C19	C18	-73.1(2)	C52A	C50A	Si4A	O16	-172.3(4)
C42	C43	C38	C37	-179.3(2)	C52A	C50A	Si4A	C53A	57.5(5)
C42	C43	C38	C39	1.2(3)	C51A	C50A	Si4A	O14	60.6(4)
C42	C41	C40	C39	0.8(3)	C51A	C50A	Si4A	O16	-47.9(4)
C86	C81	C82	C83	0.0(3)	C51A	C50A	Si4A	C53A	-178.1(4)
C86	C85	C84	C83	0.1(4)	C53B	Si4B	O14	Si3	134.0(4)
C38	C43	C42	C41	-0.3(3)	C53B	Si4B	O16	C58	-80.0(4)

C38	C39	C40	C41	0.1(3)	C53B	Si4B	C50B	C51B	-75.9(5)
C37	O13	C34	C23	176.87(16)	C53B	Si4B	C50B	C52B	52.1(5)
C37	O13	C34	C35	-60.2(2)	Si4A	O16	C58	C57	-125.3(2)
C37	O12	C36	C35	59.4(2)	Si4A	O16	C58	C59	109.9(2)
C37	C38	C39	C40	179.4(2)	C50B	Si4B	O14	Si3	-104.4(3)
C4	Si1	O2	C13	168.38(14)	C50B	Si4B	O16	C58	155.5(3)
C4	Si1	O1	Si2	-98.60(19)	C50B	Si4B	C53B	C55B	-177.1(5)
C4	Si1	C1	C3	59.1(2)	C50B	Si4B	C53B	C54B	57.1(7)
C4	Si1	C1	C2	-72.0(3)					

Table 8 Hydrogen Atom Coordinates (Å×10^4) and Isotropic Displacement Parameters (Å^2×10^3) for cu_B2479_0m.

**Atom**	***x***	***y***	***z***	**U(eq)**
H60A	-13086.03	-5784.28	-4978.95	28
H60B	-13761.19	-5686.11	-6166.8	28
H18	-6855.97	-4742.51	-9760.94	21
H57	-10852.61	-5984.46	-4117.53	23
H80	-11340.25	-4306.53	-3164.22	23
H43	-4086.44	-5427.73	-10062.92	27
H34	-6707.03	-4965.08	-12540.78	20
H77	-11730.52	-4863.25	-2554.55	20
H14	-5251.57	-3956.17	-10532.92	20
H42	-3172.65	-5818.62	-8743.13	31
H86	-8706.75	-4542.49	-4875.3	28
H37	-6721.3	-5535.41	-11957.13	24
H5A	975.99	-3777.24	-9318.27	54
H5B	1944.26	-3616.2	-8321.88	54
H5C	1775.32	-3417.24	-9469.63	54
H4	-404.45	-3518.52	-7993.47	25
H56A	-8365.92	-6012.19	-3561.49	28
H56B	-8980.95	-6384.14	-3917.89	28
H41	-3609.92	-6411.48	-8961.86	33

H25	-5625.26	-4340.78	-14833.86	24
H24	-6718.83	-4354.24	-13153.46	22
H26A	-3667.23	-4620.64	-15798.11	38
H26B	-2179.23	-4475.17	-15281.29	38
H26C	-3456.86	-4214.52	-15608.99	38
H64A	-11470.83	-4939.81	-8554.95	34
H64B	-9869.83	-4876.65	-8052.29	34
H23	-4062.79	-4597.61	-12111.71	20
H66	-9516.37	-5351.08	-2981.48	19
H15	-3555.61	-3825.27	-8527.88	19
H58	-9979.36	-6150.62	-6279.35	27
H17A	-7013.01	-4133.85	-9619.77	22
H17B	-7444.17	-4320.93	-8520.78	22
H27A	-6833.58	-3735.58	-12803.57	33
H27B	-6469.4	-3818.73	-14039.97	33
H62	-10309.5	-5143.49	-4844	20
H68	-10842.45	-5500.89	-178.75	24
H61	-12697.01	-5199.07	-5512.25	23
H19	-4464.73	-4801.95	-10349	20
H35	-4098.86	-5078.84	-13608.38	23
H1	-1911.26	-3107.29	-11448.19	37
H85	-7827.78	-4206.32	-6272.14	34
H7	-6488.17	-2822.43	-8970.8	30
H67	-12076.95	-5495.04	-1886.92	22
H84	-8332.02	-3616.73	-6361.25	42
H33	-6939.95	-3113.28	-13147.93	40
H39	-5910.48	-6217.15	-11813.97	30
H40	-5002.17	-6607.47	-10492.97	33
H13A	-2982.07	-3892.43	-11355.88	22
H13B	-3622.77	-3539.14	-10917.38	22
H22A	-8301.19	-5349.43	-7060.74	37

H22B	-8043.4	-5046.59	-6180.16	37
H22C	-7483.02	-5428.61	-5900.13	37
H78	-8881.03	-4890.96	-1526.4	23
H73	-7452.33	-6784.63	-1324.06	45
H70A	-12369.53	-6128.6	-1804.86	42
H70B	-11439.76	-6022.14	-710.98	42
H11A	-6083.66	-2887.87	-6872.42	56
H11B	-5319.19	-3039.83	-5768.74	56
H11C	-5993.64	-3296.5	-6687.16	56
H10	-3599.14	-2856.78	-6956.99	31
H12A	-3541.38	-3586.92	-6653.15	53
H12B	-2934.43	-3308.07	-5777.44	53
H12C	-2178.86	-3359.26	-6902.18	53
H76	-12417.73	-6736.77	-1590.38	44
H36A	-6803.8	-5430.35	-13895	27
H36B	-5432.02	-5520.86	-14559.19	27
H69A	-7329.48	-5567.8	412.56	49
H69B	-8869.45	-5657.04	823.38	49
H69C	-8371.76	-5263.32	739.52	49
H29	-3295.56	-3635.46	-13568.61	35
H30	-2070.03	-3111.31	-13675.94	42
H72	-8609.02	-6252.42	-1340.47	39
H21A	-5573.23	-5073.68	-6380.79	26
H21B	-5817.57	-5382.82	-7250.12	26
H82	-10581.28	-3696.26	-3659.9	34
H9A	-4800.89	-2813.75	-10840.43	53
H9B	-6413.65	-2682.06	-10832.95	53
H9C	-6057.66	-3083.68	-10674.32	53
H65A	-10922.01	-4348.53	-8671.26	50
H65B	-10539.21	-4335.25	-7378.04	50
H65C	-12149.97	-4398.87	-7837.52	50

H79A	-11073.98	-4385.95	-1222.32	28
H79B	-9505.25	-4378.43	-620.85	28
H83	-9701.97	-3361.13	-5059.03	44
H47	-5033.53	-6411.97	-6301.69	42
H31	-3266.72	-2590.68	-13509.17	48
H8A	-4903.61	-2396.5	-8215.59	61
H8B	-5696.71	-2261.07	-9330.66	61
H8C	-4081.61	-2392.06	-9321.64	61
H32	-5715.07	-2590.23	-13270.86	50
H74	-8766.91	-7292.22	-1416.52	56
H6A	862.02	-2876.16	-8534.5	61
H6B	1122.71	-3073.7	-7389.98	61
H6C	-413.89	-2916.37	-7738.18	61
H49A	-5531.79	-6975.16	-6923.05	71
H49B	-5379.68	-6726.98	-7955.19	71
H49C	-6925.63	-6844.21	-7621.47	71
H3A	1068.97	-3209.9	-11143.26	78
H3B	179.62	-3144.31	-12284	78
H3C	-24.28	-3496.43	-11627.55	78
H75	-11258.05	-7267.6	-1492.36	65
H46A	-6524.11	-7259.81	-4501.97	97
H46B	-5108.49	-7218.03	-3714.59	97
H46C	-5058.08	-7150.66	-5002.51	97
H48A	-7500.29	-6198.41	-7578.47	88
H48B	-5890.39	-6097.83	-7791.66	88
H48C	-6610.17	-5953.34	-6732.55	88
H55A	-10189.37	-6617.67	-9062.33	42
H55B	-9198.82	-6478.36	-8044.09	42
H55C	-10858.99	-6383.79	-8146.72	42
H53A	-9796.34	-7072.41	-7834.5	26
H44	-6590.64	-6734.25	-3545.82	57

H2A	-1623.95	-2597.13	-10424.29	133
H2B	-806.8	-2585.05	-11532.06	133
H2C	67.85	-2648	-10383.57	133
H45A	-4025.98	-6544.17	-4522.66	103
H45B	-4152.31	-6626.54	-3250.31	103
H45C	-4917.3	-6292.42	-3786.05	103
H54A	-12175.59	-7233.93	-7573.98	49
H54B	-12678.54	-6848.13	-7864.22	49
H54C	-11994.36	-7081.76	-8773.62	49
H50A	-12018.66	-7223.56	-5580.07	29
H52A	-9305.13	-7561.51	-5543.37	54
H52B	-10536.07	-7597.7	-6502.34	54
H52C	-10767.92	-7751.23	-5312.01	54
H51A	-11175.62	-7374.98	-3778.12	55
H51B	-11306.3	-6965.33	-3922.51	55
H51C	-9777.39	-7147.3	-3913.66	55
H55D	-10784.65	-6545.58	-7954.87	72
H55E	-10400.63	-6851.41	-8765.05	72
H55F	-9181.77	-6684.47	-7957.93	72
H53B	-9917.04	-7222.56	-7311.64	48
H54D	-12272.6	-7294.1	-6778.3	103
H54E	-12671.24	-6921.87	-7276.47	103
H54F	-12217.25	-7226.72	-8065.85	103
H51D	-12252.13	-7048.3	-4196.41	66
H51E	-11907.02	-7437.27	-3826.37	66
H51F	-12416.68	-7354.89	-5072.56	66
H52D	-8632.75	-7527.83	-5166.07	65
H52E	-10158.91	-7666.91	-5625.02	65
H52F	-9644.66	-7724.1	-4365.37	65
H50B	-9779.2	-7141.76	-4001.63	36

Table 9 Atomic Occupancy for cu_B2479_0m.

**Atom**	***Occupancy***	**Atom**	***Occupancy***	**Atom**	***Occupancy***
Si4B	0.506(6)	C55A	0.494(6)	H55A	0.494(6)
H55B	0.494(6)	H55C	0.494(6)	C53A	0.494(6)
H53A	0.494(6)	C54A	0.494(6)	H54A	0.494(6)
H54B	0.494(6)	H54C	0.494(6)	C50A	0.494(6)
H50A	0.494(6)	C52A	0.494(6)	H52A	0.494(6)
H52B	0.494(6)	H52C	0.494(6)	C51A	0.494(6)
H51A	0.494(6)	H51B	0.494(6)	H51C	0.494(6)
C55B	0.506(6)	H55D	0.506(6)	H55E	0.506(6)
H55F	0.506(6)	C53B	0.506(6)	H53B	0.506(6)
C54B	0.506(6)	H54D	0.506(6)	H54E	0.506(6)
H54F	0.506(6)	Si4A	0.494(6)	C51B	0.506(6)
H51D	0.506(6)	H51E	0.506(6)	H51F	0.506(6)
C52B	0.506(6)	H52D	0.506(6)	H52E	0.506(6)
H52F	0.506(6)	C50B	0.506(6)	H50B	0.506(6)

Experimental

Single crystals of $C_{43}H_{64}O_{13}Si_2$ **[cu_B2479_0m]**. A suitable crystal was selected and measured on a **Bruker D8 VENTURE area detector** diffractometer. The crystal was kept at 100.0 K during data collection. Using Olex2,[1] the structure was solved with the SHELXT[2] structure solution program using Intrinsic Phasing and refined with the SHELXL[3] refinement package using Least Squares minimization.

Crystal structure determination of [cu_B2479_0m]

Crystal Data for $C_{43}H_{64}O_{13}Si_2$ (M =845.12 g/mol): monoclinic, space group $P2_1$ (no. 4), a = 9.4016(10) Å, b = 38.732(4) Å, c = 12.2304(10) Å, β = 93.664(5)°, V = 4444.5(7) Å^3, Z = 4, T = 100.0 K, μ(CuKα) = 1.242 mm^{-1}, $Dcalc$ = 1.263 g/cm^3, 148824 reflections measured (4.562° ≤ 2Θ ≤ 149.832°), 18204 unique (R_{int} = 0.0354, R_{sigma} = 0.0159) which were used in all calculations. The final R_1 was 0.0269 (I > 2σ(I)) and wR_2 was 0.0688 (all data).

Refinement model description

Number of restraints - 1, number of constraints - unknown.

Details:

1. Others
 Sof(Si4B)=Sof(C55B)=Sof(H55D)=Sof(H55E)=Sof(H55F)=Sof(C53B)=Sof(H53B)=
 Sof(C54B)=Sof(H54D)=Sof(H54E)=Sof(H54F)=Sof(C51B)=Sof(H51D)=Sof(H51E)=
 Sof(H51F)=Sof(C52B)=Sof(H52D)=Sof(H52E)=Sof(H52F)=Sof(C50B)=Sof(H50B)=1-FVAR(1)
 Sof(C55A)=Sof(H55A)=Sof(H55B)=Sof(H55C)=Sof(C53A)=Sof(H53A)=Sof(C54A)=
 Sof(H54A)=Sof(H54B)=Sof(H54C)=Sof(C50A)=Sof(H50A)=Sof(C52A)=Sof(H52A)=
 Sof(H52B)=Sof(H52C)=Sof(C51A)=Sof(H51A)=Sof(H51B)=Sof(H51C)=Sof(Si4A)=FVAR(1)

2.a Ternary CH refined with riding coordinates:
C18(H18), C57(H57), C80(H80), C34(H34), C77(H77), C14(H14), C37(H37), C4(H4), C25(H25), C24(H24), C23(H23), C66(H66), C15(H15), C58(H58), C62(H62), C68(H68), C61(H61), C19(H19), C35(H35), C1(H1), C7(H7), C67(H67), C78(H78), C10(H10), C47(H47), C53A(H53A), C44(H44), C50A(H50A), C53B(H53B), C50B(H50B)
2.b Secondary CH2 refined with riding coordinates:
C60(H60A,H60B), C56(H56A,H56B), C64(H64A,H64B), C17(H17A,H17B), C27(H27A, H27B), C13(H13A,H13B), C70(H70A,H70B), C36(H36A,H36B), C21(H21A,H21B), C79(H79A,H79B)
2.c Me refined with riding coordinates:
C54A(H54A,H54B,H54C), C54B(H54D,H54E,H54F)
2.d Aromatic/amide H refined with riding coordinates:
C43(H43), C42(H42), C86(H86), C41(H41), C85(H85), C84(H84), C33(H33), C39(H39), C40(H40), C73(H73), C76(H76), C29(H29), C30(H30), C72(H72), C82(H82), C83(H83), C31(H31), C32(H32), C74(H74), C75(H75)
2.e Idealised Me refined as rotating group:
C5(H5A,H5B,H5C), C26(H26A,H26B,H26C), C22(H22A,H22B,H22C), C11(H11A,H11B, H11C), C12(H12A,H12B,H12C), C69(H69A,H69B,H69C), C9(H9A,H9B,H9C), C65(H65A, H65B,H65C), C8(H8A,H8B,H8C), C6(H6A,H6B,H6C), C49(H49A,H49B,H49C), C3(H3A,H3B, H3C), C46(H46A,H46B,H46C), C48(H48A,H48B,H48C), C55A(H55A,H55B,H55C), C2(H2A, H2B,H2C), C45(H45A,H45B,H45C), C52A(H52A,H52B,H52C), C51A(H51A,H51B,H51C), C55B(H55D,H55E,H55F), C51B(H51D,H51E,H51F), C52B(H52D,H52E,H52F)

(2) Crystal data for 3aj (Crystal identifier mo_B2485_0m): CCDC Deposition Number is 2210414, Unit Cell Parameters: a 8.5595(18) b 10.6209(16) c 35.017(4) $P2_12_12_1$.

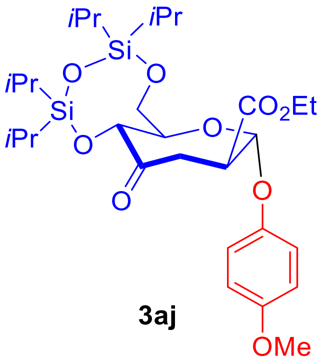

3aj

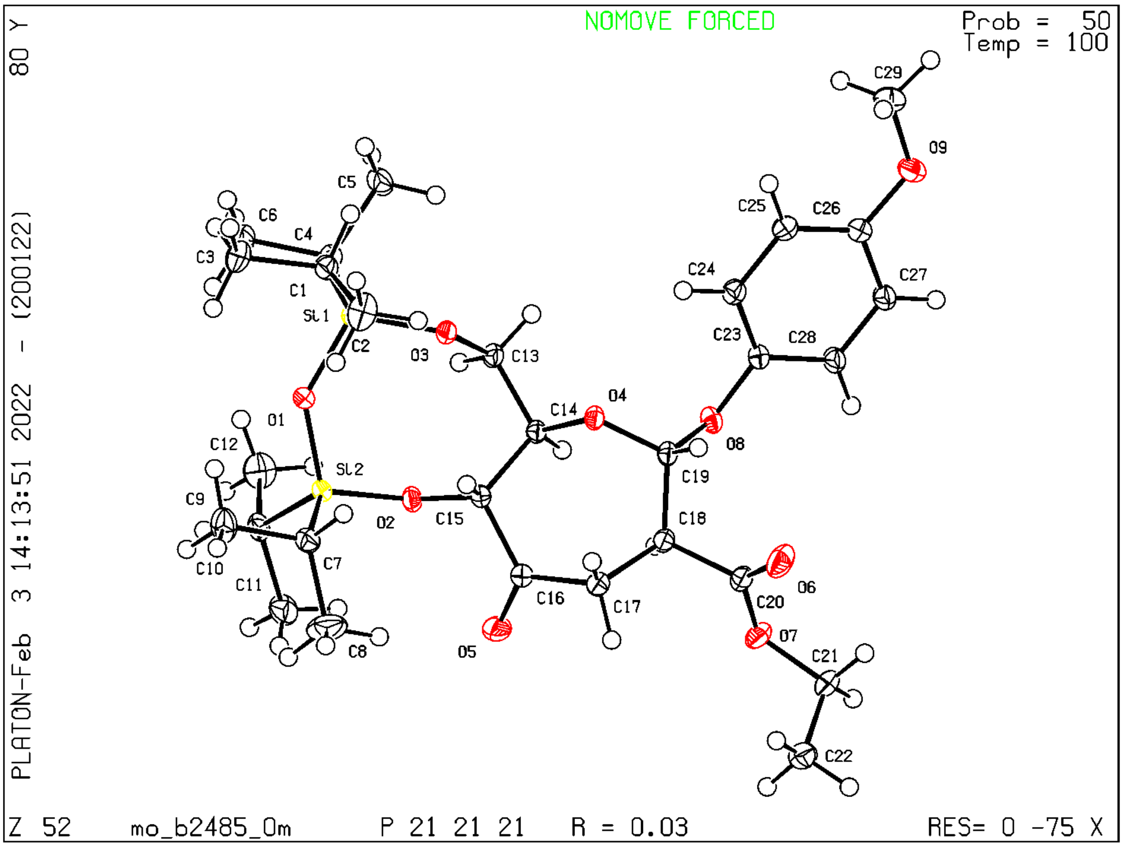

Supplementary Table 10 Crystal data and structure refinement for mo_B2485_0m.

Identification code	mo_B2485_0m
Empirical formula	$C_{29}H_{48}O_9Si_2$
Formula weight/gmol^{-1}	596.85
Temperature/K	100.00
Crystal system	orthorhombic
Space group	$P2_12_12_1$

a/Å	8.5595(18)
b/Å	10.6209(16)
c/Å	35.017(4)
α/°	90
β/°	90
γ/°	90
Volume/Å^3	3183.3(9)
Z	4
ρ_{calc}g/cm^3	1.245
μ/mm^{-1}	0.160
F(000)	1288.0
Crystal size/mm^3	0.422 × 0.342 × 0.318
Radiation	MoKα (λ = 0.71073)
2Θ range for data collection/°	4.486 to 80.844
Index ranges	-15 ≤ h ≤ 15, -19 ≤ k ≤ 19, -63 ≤ l ≤ 62
Reflections collected	271100
Independent reflections	20123 [R_{int} = 0.0542, R_{sigma} = 0.0219]
Data/restraints/parameters	20123/0/371
Goodness-of-fit on F^2	1.044
Final R indexes [I>=2σ (I)]	R_1 = 0.0309, wR_2 = 0.0842
Final R indexes [all data]	R_1 = 0.0355, wR_2 = 0.0873
Largest diff. peak/hole / e Å^{-3}	0.69/-0.23
Flack parameter	0.015(15)

Supplementary Table 11 Fractional Atomic Coordinates (×10^4) and Equivalent Isotropic Displacement Parameters (Å^2×10^3) for mo_B2485_0m. U_{eq} is defined as 1/3 of the trace of the orthogonalised U_{IJ} tensor.

Atom	*x*	*y*	*z*	U(eq)
Si2	6345.7(3)	8045.4(2)	6881.3(2)	14.66(4)
Si1	6395.7(3)	10467.8(2)	6403.9(2)	13.89(4)
O1	6369.5(9)	9550.6(6)	6779.4(2)	17.11(10)
O2	5898.7(8)	7253.6(6)	6489.4(2)	17.30(11)

O4	7511.4(8)	7466.4(6)	5530.9(2)	15.32(10)
O8	6335.8(8)	6490.8(6)	4999.2(2)	17.39(10)
O3	6761.9(8)	9628.4(6)	6017.0(2)	15.85(10)
O7	7254.7(9)	3092.6(7)	5223.1(2)	21.97(12)
O6	9201.7(12)	4319.0(8)	4998.2(3)	30.27(17)
O9	6768.0(11)	8935.4(7)	3613.9(2)	25.01(14)
O5	6469.9(12)	4835.4(7)	6371.5(2)	28.84(16)
C28	6172.1(12)	6538.5(9)	4329.3(2)	19.29(14)
C6	3965.0(13)	11998.3(10)	6709.2(3)	23.24(16)
C18	7384.0(11)	5157.0(8)	5460.1(2)	16.18(12)
C1	8081.2(12)	11582.4(9)	6440.0(2)	18.94(14)
C24	7195.9(12)	8365.4(9)	4654.2(3)	19.36(14)
C14	6263.7(10)	7480.8(7)	5804.8(2)	14.56(11)
C20	8060.7(12)	4162.2(8)	5195.0(3)	18.80(14)
C4	4416.6(11)	11206.5(9)	6358.8(2)	18.49(14)
C23	6607.8(10)	7152.0(8)	4665.2(2)	16.00(12)
C21	7868.3(14)	2001.5(9)	5017.4(3)	24.27(17)
C25	7317.1(12)	8990.4(9)	4301.7(3)	20.15(14)
C15	6920.7(10)	6978.6(8)	6187.3(2)	15.29(12)
C19	7559.5(10)	6451.6(8)	5274.5(2)	15.18(12)
C17	8222.5(11)	5066.9(9)	5849.3(2)	18.52(13)
C10	4712.6(12)	7702.5(10)	7217.9(3)	20.80(15)
C9	8700.6(14)	8510.9(12)	7413.0(3)	28.03(19)
C13	5647.3(10)	8816.6(8)	5841.5(2)	16.49(12)
C11	4472.1(17)	6273.6(12)	7267.3(3)	30.5(2)
C7	8356.5(12)	7677.4(9)	7064.3(3)	20.27(15)
C3	8136.7(15)	12338.0(11)	6814.0(3)	26.22(18)
C26	6828.2(11)	8392.8(9)	3967.9(2)	18.51(13)
C22	8877.2(14)	1225.1(10)	5278.7(3)	25.75(18)
C16	7156.9(11)	5555.0(9)	6162.5(2)	18.19(13)
C2	9634.9(13)	10892.2(13)	6383.0(4)	29.9(2)

C27	6284.0(13)	7153.5(9)	3982.5(2)	20.07(14)
C29	7282.6(16)	10201.9(10)	3580.1(3)	28.1(2)
C12	3190.8(14)	8326.8(14)	7086.2(4)	31.9(2)
C5	4245.5(16)	11971.2(12)	5989.1(3)	28.4(2)
C8	8703(2)	6293.0(12)	7154.3(5)	39.4(3)

Supplementary Table 12 Anisotropic Displacement Parameters (Å^2×10^3) for mo_B2485_0m. The Anisotropic displacement factor exponent takes the form: -2π^2[h^{2}a*^{2}U$_{11}$+2hka*b*U$_{12}$+…].

Atom	U_{11}	U_{22}	U_{33}	U_{23}	U_{13}	U_{12}
Si2	18.48(9)	14.67(9)	10.82(8)	0.71(6)	1.08(7)	0.00(8)
Si1	17.22(9)	12.63(8)	11.84(8)	-0.51(6)	-0.41(7)	0.58(7)
O1	23.8(3)	14.5(2)	13.0(2)	0.79(18)	-0.4(2)	0.8(2)
O2	21.2(3)	18.3(3)	12.5(2)	-1.93(18)	3.18(19)	-2.1(2)
O4	17.7(2)	15.3(2)	13.0(2)	-2.59(17)	1.72(18)	-2.77(19)
O8	17.7(2)	21.2(3)	13.2(2)	-0.83(19)	-0.8(2)	-3.8(2)
O3	19.4(3)	14.5(2)	13.6(2)	-1.91(18)	0.70(18)	-1.32(19)
O7	25.7(3)	14.0(2)	26.2(3)	-4.5(2)	1.9(2)	-0.8(2)
O6	33.5(4)	23.4(3)	33.9(4)	-8.3(3)	15.6(3)	-2.8(3)
O9	34.9(4)	20.4(3)	19.7(3)	4.3(2)	-2.2(3)	-2.9(3)
O5	42.7(5)	17.6(3)	26.2(3)	3.6(2)	10.8(3)	-1.4(3)
C28	24.4(4)	18.7(3)	14.8(3)	-1.4(2)	-0.9(3)	-4.1(3)
C6	27.3(4)	24.6(4)	17.8(3)	-3.2(3)	0.2(3)	8.1(3)
C18	17.8(3)	15.0(3)	15.7(3)	-2.4(2)	1.5(2)	-0.2(2)
C1	23.3(4)	17.0(3)	16.5(3)	-0.8(2)	-0.6(3)	-3.2(3)
C24	22.7(4)	17.4(3)	18.0(3)	-2.0(3)	-0.1(3)	-2.2(3)
C14	16.2(3)	14.9(3)	12.6(2)	-1.8(2)	0.4(2)	-1.3(2)
C20	22.2(4)	16.0(3)	18.2(3)	-3.6(3)	1.5(3)	0.0(3)
C4	21.1(3)	19.0(3)	15.3(3)	-1.2(2)	-1.6(3)	4.3(3)
C23	16.4(3)	17.8(3)	13.8(3)	-1.4(2)	0.0(2)	-0.7(2)
C21	34.2(5)	15.5(3)	23.2(4)	-5.3(3)	-0.6(3)	1.6(3)
C25	23.0(4)	16.6(3)	20.9(3)	-0.8(3)	-0.4(3)	-1.9(3)
C15	18.9(3)	14.7(3)	12.2(3)	-0.9(2)	1.0(2)	-0.7(2)

C19	16.5(3)	16.0(3)	13.0(3)	-2.4(2)	0.4(2)	-1.3(2)
C17	21.8(3)	16.7(3)	17.1(3)	-1.3(2)	-0.7(3)	2.2(3)
C10	24.8(4)	23.0(4)	14.6(3)	-1.2(3)	4.9(3)	-4.5(3)
C9	25.0(4)	37.4(5)	21.8(4)	-6.0(4)	-6.3(3)	4.6(4)
C13	18.9(3)	16.6(3)	14.1(3)	-2.5(2)	-2.3(2)	1.1(2)
C11	41.8(6)	26.7(5)	22.9(4)	1.5(3)	8.1(4)	-11.6(4)
C7	22.7(4)	21.2(3)	16.9(3)	1.3(3)	-1.0(3)	3.6(3)
C3	30.3(5)	23.4(4)	25.0(4)	-8.7(3)	-2.5(3)	-3.2(4)
C26	20.3(3)	17.8(3)	17.4(3)	1.2(3)	-0.3(3)	0.1(3)
C22	29.9(5)	21.2(4)	26.1(4)	0.9(3)	3.8(3)	3.5(3)
C16	23.9(4)	15.6(3)	15.1(3)	-0.3(2)	0.6(3)	0.3(3)
C2	20.3(4)	36.3(6)	33.3(5)	-10.3(4)	1.7(4)	-2.4(4)
C27	26.1(4)	18.8(3)	15.3(3)	-0.7(2)	-1.4(3)	-3.4(3)
C29	35.3(5)	21.2(4)	27.8(4)	6.6(3)	1.5(4)	-3.1(4)
C12	22.2(4)	39.6(6)	34.0(5)	-1.2(5)	6.5(4)	0.5(4)
C5	37.0(5)	30.9(5)	17.2(3)	2.3(3)	-2.4(3)	14.6(4)
C8	45.0(7)	24.7(5)	48.4(7)	4.8(5)	-16.8(6)	9.7(5)

Supplementary Table 13 Bond Lengths for mo_B2485_0m.

Atom	Atom	Length/Å	Atom	Atom	Length/Å
Si2	O1	1.6382(7)	C28	C27	1.3821(12)
Si2	O2	1.6543(7)	C6	C4	1.5370(13)
Si2	C10	1.8643(10)	C18	C20	1.5210(12)
Si2	C7	1.8777(11)	C18	C19	1.5283(12)
Si1	O1	1.6367(7)	C18	C17	1.5434(13)
Si1	O3	1.6519(7)	C1	C3	1.5368(13)
Si1	C1	1.8705(10)	C1	C2	1.5316(16)
Si1	C4	1.8736(10)	C24	C23	1.3841(13)
O2	C15	1.4035(10)	C24	C25	1.4053(13)
O4	C14	1.4356(10)	C14	C15	1.5475(11)
O4	C19	1.4034(10)	C14	C13	1.5191(12)

O8	C23	1.3839(10)	C4	C5	1.5350(13)
O8	C19	1.4241(11)	C21	C22	1.5042(16)
O3	C13	1.4251(11)	C25	C26	1.3945(13)
O7	C20	1.3327(12)	C15	C16	1.5280(13)
O7	C21	1.4619(12)	C17	C16	1.5177(13)
O6	C20	1.2068(13)	C10	C11	1.5412(16)
O9	C26	1.3677(11)	C10	C12	1.5327(17)
O9	C29	1.4204(14)	C9	C7	1.5366(14)
O5	C16	1.2104(12)	C7	C8	1.5328(16)
C28	C23	1.3953(12)	C26	C27	1.3972(13)

Supplementary Table 14 Bond Angles for mo_B2485_0m.

Atom	Atom	Atom	Angle/˚	Atom	Atom	Atom	Angle/˚
O1	Si2	O2	108.56(3)	O6	C20	O7	125.36(9)
O1	Si2	C10	109.74(4)	O6	C20	C18	124.11(9)
O1	Si2	C7	105.43(4)	C6	C4	Si1	112.90(6)
O2	Si2	C10	104.58(4)	C5	C4	Si1	112.26(7)
O2	Si2	C7	112.91(4)	C5	C4	C6	111.09(8)
C10	Si2	C7	115.52(5)	O8	C23	C28	115.49(8)
O1	Si1	O3	109.90(4)	O8	C23	C24	123.86(7)
O1	Si1	C1	109.45(4)	C24	C23	C28	120.57(8)
O1	Si1	C4	107.73(4)	O7	C21	C22	109.95(8)
O3	Si1	C1	104.52(4)	C26	C25	C24	119.94(8)
O3	Si1	C4	109.17(4)	O2	C15	C14	110.75(7)
C1	Si1	C4	115.98(5)	O2	C15	C16	109.34(7)
Si1	O1	Si2	139.12(4)	C16	C15	C14	109.87(7)
C15	O2	Si2	125.94(6)	O4	C19	O8	112.89(7)
C19	O4	C14	117.22(6)	O4	C19	C18	114.59(7)
C23	O8	C19	117.59(7)	O8	C19	C18	104.00(7)
C13	O3	Si1	123.58(6)	C16	C17	C18	109.72(8)
C20	O7	C21	116.95(8)	C11	C10	Si2	111.31(7)

C26	O9	C29	117.58(9)	C12	C10	Si2	111.25(7)
C27	C28	C23	120.09(8)	C12	C10	C11	110.26(10)
C20	C18	C19	109.15(7)	O3	C13	C14	111.65(7)
C20	C18	C17	108.59(7)	C9	C7	Si2	109.08(7)
C19	C18	C17	112.68(7)	C8	C7	Si2	116.58(9)
C3	C1	Si1	114.33(7)	C8	C7	C9	110.62(9)
C2	C1	Si1	110.98(7)	O9	C26	C25	125.39(9)
C2	C1	C3	109.51(9)	O9	C26	C27	114.69(8)
C23	C24	C25	119.41(8)	C25	C26	C27	119.87(8)
O4	C14	C15	107.72(7)	O5	C16	C15	121.75(8)
O4	C14	C13	108.96(7)	O5	C16	C17	120.87(9)
C13	C14	C15	112.02(6)	C17	C16	C15	117.30(7)
O7	C20	C18	110.48(8)	C28	C27	C26	120.04(8)

Supplementary Table 15 Torsion Angles for mo_B2485_0m.

A	B	C	D	Angle/˚	A	B	C	D	Angle/˚
Si2	O2	C15	C14	-123.59(7)	C20	C18	C19	O4	-158.39(8)
Si2	O2	C15	C16	115.20(7)	C20	C18	C19	O8	77.91(9)
Si1	O3	C13	C14	-124.20(7)	C20	C18	C17	C16	-153.65(8)
O1	Si2	O2	C15	78.29(8)	C4	Si1	O1	Si2	107.31(8)
O1	Si2	C10	C11	170.28(8)	C4	Si1	O3	C13	-43.59(8)
O1	Si2	C10	C12	46.88(9)	C4	Si1	C1	C3	68.57(8)
O1	Si2	C7	C9	58.32(8)	C4	Si1	C1	C2	-166.97(7)
O1	Si2	C7	C8	-175.54(9)	C23	O8	C19	O4	88.29(9)
O1	Si1	O3	C13	74.37(7)	C23	O8	C19	C18	-146.91(7)
O1	Si1	C1	C3	-53.53(8)	C23	C28	C27	C26	-0.25(16)
O1	Si1	C1	C2	70.93(8)	C23	C24	C25	C26	0.81(15)
O1	Si1	C4	C6	60.06(8)	C21	O7	C20	O6	4.22(16)
O1	Si1	C4	C5	-173.42(7)	C21	O7	C20	C18	-173.37(8)
O2	Si2	O1	Si1	-14.25(9)	C25	C24	C23	O8	-175.13(9)
O2	Si2	C10	C11	54.01(9)	C25	C24	C23	C28	1.52(14)

O2	Si2	C10	C12	-69.39(9)	C25	C26	C27	C28	2.57(15)
O2	Si2	C7	C9	176.68(7)	C15	C14	C13	O3	50.65(9)
O2	Si2	C7	C8	-57.18(10)	C19	O4	C14	C15	99.46(8)
O2	C15	C16	O5	2.93(13)	C19	O4	C14	C13	-138.82(7)
O2	C15	C16	C17	179.55(7)	C19	O8	C23	C28	133.85(8)
O4	C14	C15	O2	165.33(6)	C19	O8	C23	C24	-49.34(12)
O4	C14	C15	C16	-73.77(8)	C19	C18	C20	O7	-146.08(8)
O4	C14	C13	O3	-68.42(8)	C19	C18	C20	O6	36.29(14)
O3	Si1	O1	Si2	-11.54(9)	C19	C18	C17	C16	85.30(9)
O3	Si1	C1	C3	-171.19(7)	C17	C18	C20	O7	90.72(10)
O3	Si1	C1	C2	-46.73(8)	C17	C18	C20	O6	-86.91(12)
O3	Si1	C4	C6	179.37(7)	C17	C18	C19	O4	-37.66(10)
O3	Si1	C4	C5	-54.12(8)	C17	C18	C19	O8	-161.37(7)
O9	C26	C27	C28	-174.94(10)	C10	Si2	O1	Si1	-127.98(8)
C18	C17	C16	O5	102.43(11)	C10	Si2	O2	C15	-164.62(7)
C18	C17	C16	C15	-74.22(10)	C10	Si2	C7	C9	-63.02(9)
C1	Si1	O1	Si2	-125.78(8)	C10	Si2	C7	C8	63.12(10)
C1	Si1	O3	C13	-168.27(7)	C13	C14	C15	O2	45.53(9)
C1	Si1	C4	C6	-62.94(8)	C13	C14	C15	C16	166.43(7)
C1	Si1	C4	C5	63.57(8)	C7	Si2	O1	Si1	106.99(8)
C24	C25	C26	O9	174.38(10)	C7	Si2	O2	C15	-38.23(8)
C24	C25	C26	C27	-2.85(15)	C7	Si2	C10	C11	-70.74(9)
C14	O4	C19	O8	70.52(9)	C7	Si2	C10	C12	165.87(8)
C14	O4	C19	C18	-48.29(10)	C27	C28	C23	O8	175.11(9)
C14	C15	C16	O5	-118.81(10)	C27	C28	C23	C24	-1.81(15)
C14	C15	C16	C17	57.81(10)	C29	O9	C26	C25	1.73(16)
C20	O7	C21	C22	94.13(11)	C29	O9	C26	C27	179.09(10)

Supplementary Table 16 Hydrogen Atom Coordinates (Å×10^4) and Isotropic Displacement Parameters (Å^2×10^3) for mo_B2485_0m.

**Atom**	***x***	***y***	***z***	**U(eq)**
H28	5797.9	5697.04	4338.99	23

H6A	4164.78	11513.27	6942.26	35
H6B	2853.34	12214.49	6695.55	35
H6C	4588.87	12771.82	6713.34	35
H18	6247.39	4979.92	5498.48	19
H1	7975.49	12202.04	6226.52	23
H24	7514.69	8772.18	4882.92	23
H14	5404.06	6915.52	5715.33	17
H4	3645.52	10501.01	6343.2	22
H21A	8490.75	2287.74	4795.49	29
H21B	6991.78	1482.5	4921.25	29
H25	7731.85	9819.29	4290.87	24
H15	7951.37	7388.99	6238.23	18
H19	8581.51	6476.08	5136.3	18
H17A	8505.74	4179.97	5902.01	22
H17B	9195.4	5570.5	5842.86	22
H10	4994.6	8062.18	7472.68	25
H9A	8574.08	9398.26	7342.73	42
H9B	9774.61	8364.78	7499.22	42
H9C	7972.5	8301.39	7619.43	42
H13A	5385.46	9144.55	5584.7	20
H13B	4677.9	8809.77	5995.68	20
H11A	4223.75	5895.3	7019.35	46
H11B	3609.19	6122.5	7445.44	46
H11C	5430.29	5894.49	7368.47	46
H7	9106.44	7936.73	6859.96	24
H3A	7150.66	12793.76	6847.55	39
H3B	9002.82	12940.52	6803.85	39
H3C	8292.01	11761.54	7029.24	39
H22A	9306.1	508.18	5137.06	39
H22B	8247.96	916.57	5492.9	39
H22C	9735.03	1744.85	5376.19	39

H2A	9835.11	10345.07	6603.04	45
H2B	10481.43	11508.47	6359.34	45
H2C	9582.27	10381.29	6150.35	45
H27	5990.8	6733.25	3754.01	24
H29A	7109.14	10496.41	3318.25	42
H29B	8398.95	10249.67	3640.53	42
H29C	6694.87	10733.56	3758.07	42
H12A	3341.97	9239.91	7070.02	48
H12B	2358.24	8140.86	7269.82	48
H12C	2899.3	7998.91	6834.48	48
H5A	4957.94	12693.17	5996.22	43
H5B	3167.34	12271.64	5965.49	43
H5C	4502.18	11437.01	5769.74	43
H8A	8099.29	6032.45	7378.5	59
H8B	9820.92	6191.52	7206.93	59
H8C	8412.15	5770.04	6935.01	59

Experimental

Single crystals of $C_{43}H_{64}O_{13}Si_2$ **[mo_B2485_0m]**. A suitable crystal was selected and measured on a **Bruker D8 VENTURE area detector** diffractometer. The crystal was kept at 100.0 K during data collection. Using Olex2,[1] the structure was solved with the SHELXT[2] structure solution program using Intrinsic Phasing and refined with the SHELXL[3] refinement package using Least Squares minimization.

Crystal structure determination of [mo_B2485_0m]

Crystal Data for $C_{29}H_{48}O_9Si_2$ (M =596.85 g/mol): orthorhombic, space group $P2_12_12_1$ (no. 19), a = 8.5595(18) Å, b = 10.6209(16) Å, c = 35.017(4) Å, V = 3183.3(9) Å3, Z = 4, T = 100.00 K, μ(MoKα) = 0.160 mm-1, $Dcalc$ = 1.245 g/cm^3, 271100 reflections measured (4.486° ≤ 2Θ ≤ 80.844°), 20123 unique (R_{int} = 0.0542, R_{sigma} = 0.0219) which were used in all calculations. The final R_1 was 0.0309 (I > 2σ(I)) and wR_2 was 0.0873 (all data).

Refinement model description

Number of restraints - 0, number of constraints - unknown.

Details:

1.a Ternary CH refined with riding coordinates:

C18(H18), C1(H1), C14(H14), C4(H4), C15(H15), C19(H19), C10(H10), C7(H7)

1.b Secondary CH2 refined with riding coordinates:

C21(H21A,H21B), C17(H17A,H17B), C13(H13A,H13B)

1.c Aromatic/amide H refined with riding coordinates:

C28(H28), C24(H24), C25(H25), C27(H27)

1.d Idealised Me refined as rotating group:

C6(H6A,H6B,H6C), C9(H9A,H9B,H9C), C11(H11A,H11B,H11C), C3(H3A,H3B,H3C),

C22(H22A,H22B,H22C), C2(H2A,H2B,H2C), C29(H29A,H29B,H29C), C12(H12A,H12B,H12C),

C5(H5A,H5B,H5C), C8(H8A,H8B,H8C)

Synthesis of catalysts

Catalysts **A-E** were prepared according to the literature procedure:[4] ArSeCl (2.0 mmol) was added to a solution of $GaCl_3$ (2.0 mmol) in dry CH_2Cl_2 (10.0 mL) and the reaction mixture was stirred for 5 minutes to give a dark orange solution. Then phosphine (1.0 mmol) was added to the above reaction mixture

and the reaction was run for 1 h to generate a colourless solution. Subsequently, the reaction mixture was concentrated to give a saturated solution (~3 mL CH_2Cl_2) under reduced pressure and then 10.0 mL *n*-hexane was slowly added. The two-phase solution was then placed for 2 h at room temperature under argon to give white solid or colourless crystals. The precipitate was filtered off and washed by anhydrous diethyl ether to afford pure catalyst.

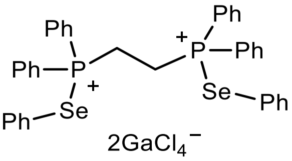

B

White solid (92% yield). **^{1}H NMR** (500 MHz, CD_2Cl_2): 7.89 (t, *J*= 7.5 Hz, 4H), 7.74–7.70(m, 8H),7.62–7.58(m, 8H), 7.40 (t, *J*= 7.5 Hz, 2H),7.10 (t, *J*= 7.8 Hz, 4H), 7.02–7.00(m, 4H),3.09 (d, *J*= 3.3 Hz, 4H); **^{13}C NMR** (126 MHz, CD_2Cl_2): δ137.70, 137.05, 133.92, 133.87, 133.83, 132.38, 131.57, 131.51, 131.46, 131.35, 117.60, 116.27 (d, *J* = 75.6 Hz), 20.42 (d, *J* = 41.6 Hz).

The analytical data are in accordance with the reported literature.[4]

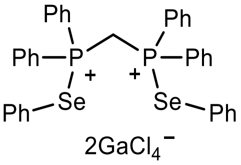

A

White solid (91% yield). **^{1}H NMR** (600 MHz, CD_2Cl_2): δ 7.84-7.82 (m, 4H), 7.67-7.62 (m, 16H), 7.35-7.32 (m, 2H), 7.10-7.09 (m, 8H), 5.20 (bs, 2H); **^{13}C NMR** (151 MHz, CD_2Cl_2): δ 138.01, 137.60-137.49 (m), 134.35 (d, *J* = 11.5 Hz), 132.48, 131.60 (d, *J* = 13.9 Hz), 131.31, 118.79, 28.26 (t, *J* = 36.5 Hz).

The analytical data are in accordance with the reported literature.[5]

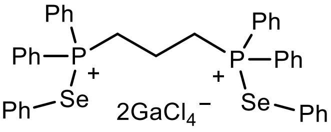

C

White solid (90% yield). **^{1}H NMR** (600 MHz, CD_2Cl_2): 7.81 (t, *J* = 7.4 Hz, 4H), 7.65 (td, *J* = 7.8, 3.6 Hz, 8H), 7.60-7.56 (m, 8H), 7.45 (t, *J* = 7.5 Hz, 2H), 7.22 (t, *J* = 7.7 Hz, 4H), 7.15 (d, *J* = 7.7 Hz, 4H), 3.18 (td, *J* = 11.9, 7.7 Hz, 4H), 1.77 (dt, *J* = 12.4, 8.8 Hz, 2H); **^{13}C NMR** (151 MHz, CD_2Cl_2): δ 137.79, 136.51, 136.49, 133.66, 133.62, 133.59, 132.33, 131.40, 131.17, 131.08, 117.87, 117.31 (d, *J* = 75.5 Hz), 26.30 (d, *J* = 63.4 Hz), 17.26. **^{31}P NMR** (243 MHz, CD_2Cl_2): δ 39.69; ^{77}Se NMR (114 MHz, CD_2Cl_2): δ 281.92 (d, $J_{Se\text{-}P}$= 448.84Hz); HRMS (ESI+) exact mass calculated for $[M]^{2+}$ ($C_{39}H_{36}P_2Se_2$) requires m/z 363.03059, found m/z 363.03075.

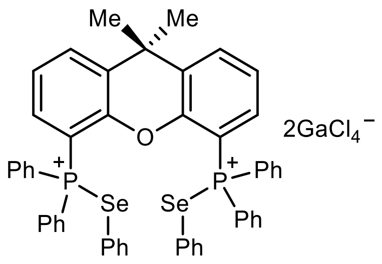

D

White solid (90% yield).^{1}**H NMR** (500 MHz, CD_2Cl_2): δ 8.16 (d, *J* = 7.9 Hz, 2H), 7.66–7.60 (m, 4H), 7.56–7.52 (m, 8H), 7.49–7.42 (m, 13H), 7.20 (t, *J* = 7.7 Hz, 4H), 7.13–7.11 (m, 5H), 1.97 (s, 6H); ^{13}**C NMR** (126 MHz, CD_2Cl_2): δ 152.84 (d, *J* = 2.5 Hz), 137.96 (d, *J* = 3.6 Hz), 137.02 (d, *J* =7.4 Hz), 136.84 (d, *J* = 2.6 Hz), 136.32 (d, *J* = 3.4 Hz), 134.59 (d, *J* = 10.7 Hz), 133.58 (d, *J* = 6.7 Hz), 132.30 (d, *J* = 3.6 Hz), 131.12 (d, *J* = 2.2 Hz), 131.00 (d, *J* = 13.5 Hz), 126.46 (d, *J* = 13.3 Hz), 121.01 (d, *J* = 8.4 Hz), 119.75 (d, *J* = 78.2Hz), 105.26 (d, *J* = 77.8 Hz), 34.20.

The analytical data are in accordance with the reported literature.[4]

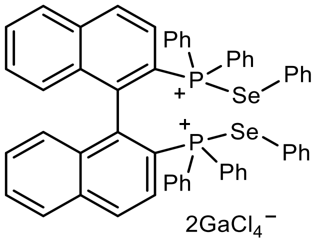

E

White solid (92% yield). ^{1}**H NMR** (600 MHz, CD_2Cl_2): δ 8.60 (dd, *J* = 5.9 Hz, 2H), 8.24 (d, *J* = 8.1 Hz, 2H), 8.16–8.13 (m, 2H), 7.76–7.68 (m, 6H), 7.50–7.45 (m, 8H), 7.32–7.23 (m, 10H), 7.10–7.04 (m, 8H), 6.72 (t, *J* = 7.5 Hz, 2H), 6.30 (d, *J* = 8.4 Hz, 2H); ^{13}**C NMR** (151 MHz, CD_2Cl_2): δ 139.81 (dd, *J* = 9.0, 5.0 Hz), 137.87 (d, *J* = 3.6 Hz), 136.62 (d, *J* = 2.6 Hz), 136.35 (d, *J* = 3.4 Hz), 135.85 (d, *J* = 3.3 Hz), 134.86 (d, *J* = 10.5 Hz), 134.19 (d, *J* = 10.6 Hz), 134.02 (d, *J* = 12.5 Hz), 133.11 (d, *J* = 11.7 Hz), 132.13 (d, *J* = 3.6 Hz), 131.70, 131.07, 131.05, 131.01, 130.94, 130.92, 130.85, 129.85, 129.60, 128.62 (d, *J* = 10.3 Hz), 126.15, 121.31, 121.21 (d, *J* = 7.5 Hz), 120.81, 118.98, 118.23 (d, *J* = 18.4 Hz), 117.53.

The analytical data are in accordance with the reported literature.[4]

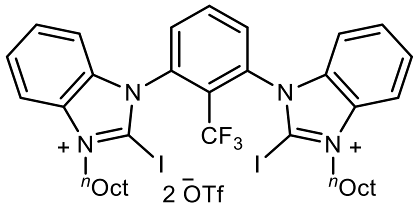

G

Catalyst **G** was prepared according to the literature procedure.[3] ^{1}**H NMR** (500 MHz, CD_2Cl_2): δ 8.28-8.21 (m, 3 H), 7.92 (d, *J* = 7.7 Hz, 2 H), 7.79 (d, *J* = 7.7 Hz, 2 H), 7.72-7.65 (m, 4 H), 4.61-4.49 (m, 4 H), 1.98 (p, *J* = 7.6 Hz, 4 H), 1.49-1.22 (m, 20 H), 0.86 (t, *J* = 6.8 Hz, 6 H); ^{13}**C NMR** (126 MHz, CD_2Cl_2): δ 138.19, 136.50, 135.88, 134.90, 132.96, 128.98, 128.15, 127.60 (q, *J* = 31.4 Hz), 121.42 (q, *J* = 277.6 Hz), 120.60 (q, *J* = 320.8 Hz), 116.08, 114.96, 112.98, 51.61, 31.99, 29.35, 29.3, 26.89, 22.93, 14.18.

The analytical data are in accordance with the reported literature.[6]

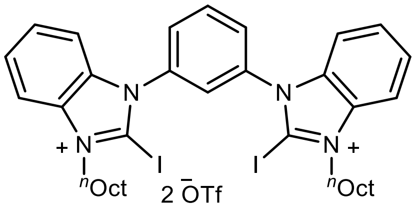

H

Catalyst **H** was prepared according to the literature procedure.[3] **^{1}H NMR** (600 MHz, CD_3CN): δ 7.95-7.92 (m, 1 H), 7.85-7.82 (m, 6 H), 7.73-7.72 (m, 1 H), 4.23 (t, J = 7.5 Hz, 4 H), 1.95-1.88 (m, 4 H), 1.44-1.31 (m, 20 H), 0.91-0.89 (m, 6 H); **^{13}C NMR** (151 MHz, CD_3CN): δ 138.50, 132.95, 130.88, 127.87, 127.10, 127.00, 121.87 (q, $^1J_{C\text{-}F}$ = 320.4 Hz), 101.39, 54.07, 32.41, 30.12, 29.71, 29.59, 26.73, 23.28, 14.32.

The analytical data are in accordance with the reported literature.[6]

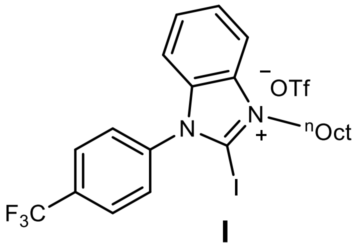

I

Catalyst **I** was prepared according to the literature procedure.[3] **^{1}H NMR** (500 MHz, CD_2Cl_2): δ 8.03 (d, J = 8.4 Hz, 2 H), 7.84 (d, J = 8.4 Hz, 1 H), 7.76 (d, J = 8.3 Hz, 2 H), 7.71-7.68 (m, 1 H), 7.62-7.59 (m, 1 H), 7.36 (d, J = 8.4 Hz, 1 H), 4.63-4.60 (m, 2 H), 2.02 (p, J = 7.8 Hz, 2 H), 1.56-1.50 (m, 2 H), 1.50-1.39 (m, 2 H), 1.37-1.28 (m, 6 H), 0.89 (t, J = 6.9 Hz, 3 H); **^{13}C NMR** (126 MHz, CD_2Cl_2): δ 137.73, 135.42, 134.28 (q, J = 33.52 Hz), 133.77, 129.26, 128.67 (q, J = 3.8 Hz), 128.57, 128.28, 123.83 (q, J = 273.4 Hz), 121.04 (q, J = 321.4 Hz), 113.73, 113.65, 112.78, 51.78, 32.26, 29.66, 29.64, 29.61, 27.31, 23.17, 14.40.

The analytical data are in accordance with the reported literature.[6]

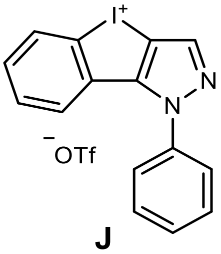

J

Catalyst **J** was prepared according to the literature procedure.[4] **^{1}H NMR** (600 MHz, DMSO): δ 8.31 (dd, J = 8.2, 1.3 Hz, 1H), 8.18 (s, 1H), 7.74-7.70 (m,3H), 7.69-7.66 (m, 2H), 7.65 (td, J = 7.6, 1.3 Hz, 1H), 7.61 (ddd, J = 8.9, 7.4, 1.7 Hz, 1H), 7.23 (dd, J = 7.7, 1.7 Hz, 1H); **^{13}C NMR** (151 MHz, DMSO): δ 147.89, 138.86, 138.73, 131.58, 130.59, 130.55, 130.42, 130.05, 128.02, 126.71, 126.37, 125.28, 120.72 (q, J = 322.4 Hz), 94.64.

The analytical data are in accordance with the reported literature.[7]

Catalysts **K** was prepared according to the literature procedure[8]: PPh_3 (3.9 mmol) was added to a solution of methyl trifluoromethanesulfonate (3.9 mmol) in dry CH_2Cl_2 (10.0 mL) and the reaction mixture was stirred for 3 h at room temperature and evaporated to give 1.65 g (100%) of salt K as white solid.

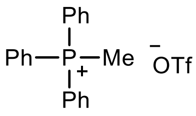

K

^{1}H NMR (500 MHz, $CDCl_3$): δ 7.82 – 7.76 (m, 3H), 7.71 – 7.61 (m, 13H), 2.91 (d, J = 13.4 Hz, 3H); **^{13}C NMR** (101 MHz, $CDCl_3$): δ 135.35 (d, *J* = 3.1 Hz), 133.20 (d, *J* = 10.6 Hz), 130.62 (d, *J* = 13.0 Hz), 119.07 (d, *J* = 88.8 Hz), 9.54 (d, *J* = 58.5 Hz); **^{19}F NMR** (377 MHz, $CDCl_3$) δ -78.26; ^{31}P NMR (162 MHz, $CDCl_3$) δ 21.50.

The analytical data are in accordance with the reported literature[8].

Synthesis of cyclopropanated glycosyl donors

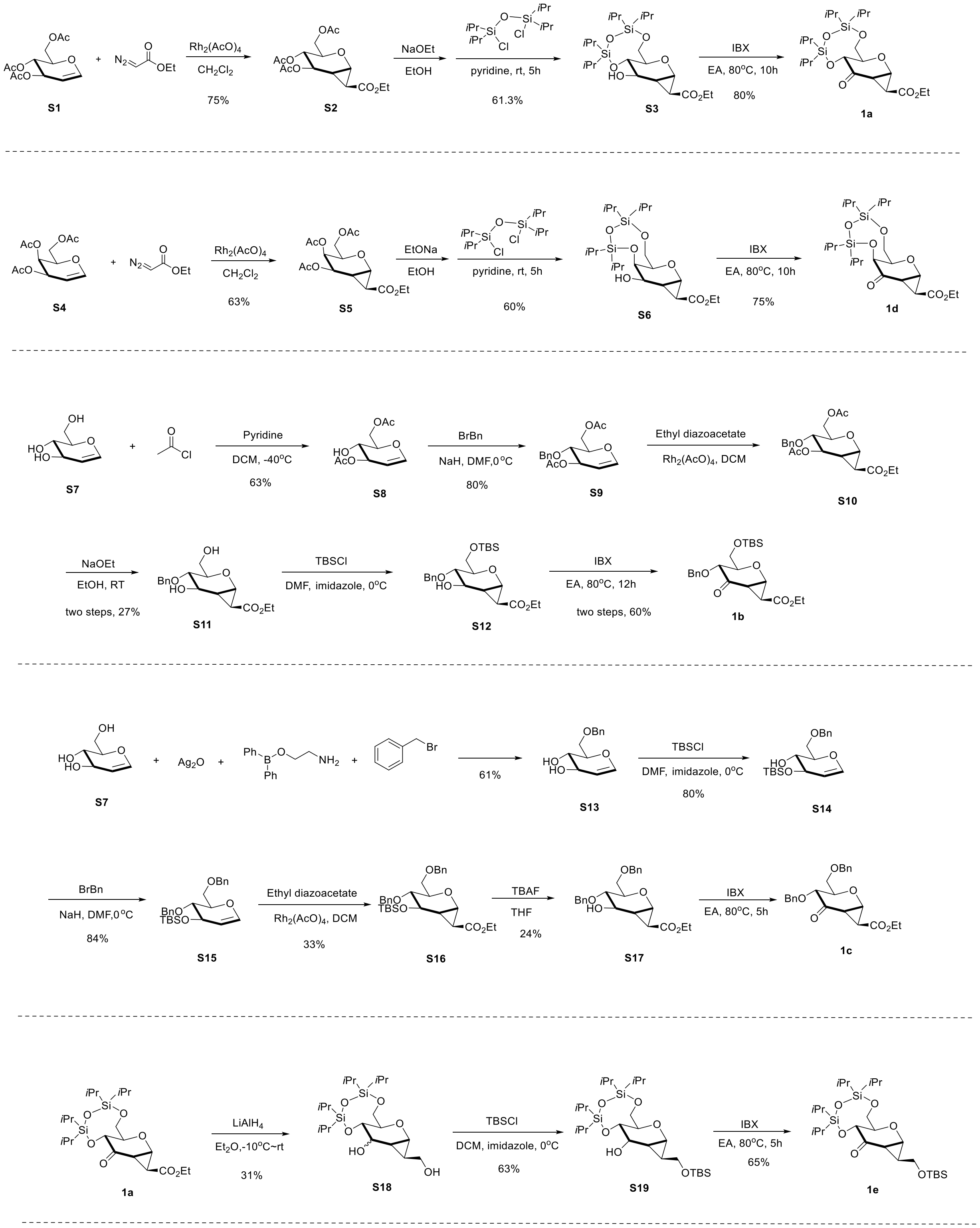

Supplementary Figure 1: Overview for the synthesis of the cyclopropanated sugar donor **1a-1e**

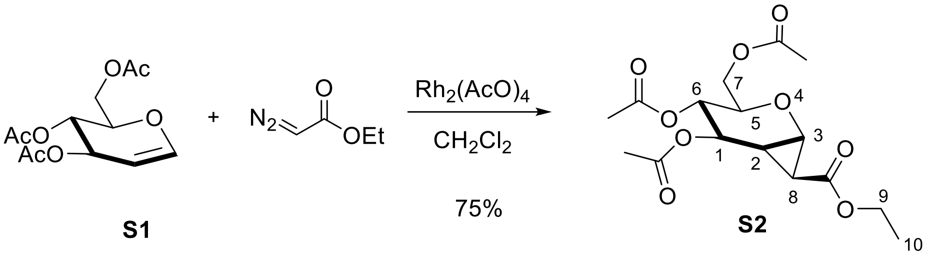

Compound **S2** was prepared according to the literature procedure.[9] A mixture of Tri-O-acetyl-D-glucal **S1** (15 g, 55.1 mmol, 1.0 equiv) and rhodium acetate dimer (243 mg, 0.551 mmol, 0.01 equiv) in dichloromethane (250 ml) was vigorously stirred under an argon atmosphere. Ethyl diazoacetate (11.6 ml, 110 mmol, 2.0 equiv) in dichloromethane (15 ml) was added dropwise to the mixture over the course of 14 hours via syringe pump, after which time TLC (hexanes/ethyl acetate 1:1) showed the reaction to be complete. The mixture was evaporated under reduced pressure to give a green syrup which was purified by flash chromatography. Elution with *n*-pentane/ethyl acetate (5:1~3:1) gave 15 g **S2** as light-yellow oil, 75% yield. (Compound **S2** contains a small amount of inseparable isomers.)

^{1}H NMR (600 MHz, $CDCl_3$) δ 5.08 (dd, *J* = 5.0, 1.7 Hz, 1H, H-1), 4.84 (t, *J* = 4.9 Hz, 1H, H-6), 4.46 (dd, *J* = 12.1, 7.5 Hz, 1H, H-7), 4.13-4.09 (m, 3H, H-7, H-9), 3.93 (dd, *J* = 7.1, 2.1 Hz, 1H, H-3), 3.85-3.82 (m, 1H, H-5), 2.09 (s, 3H, OAc), 2.08 (s, 3H, OAc), 2.07 (s, 3H, OAc), 2.05 (dd, *J* = 4.6, 2.4 Hz, 1H), 1.77-1.74 (m, 1H, H-8), 1.25 (t, *J* = 7.1 Hz, 3H, H-10). **^{13}C NMR** (151 MHz, $CDCl_3$) δ 170.77, 170.69, 169.63, 169.57, 72.08, 68.49, 67.00, 61.91, 61.17, 55.99, 24.71, 23.23, 21.03, 20.97, 20.90, 14.31. **ESI-HRMS**: Calculated for $C_{16}H_{22}O_9Na$ $(M+Na)^+$: 381.1156, Found: 381.1161.

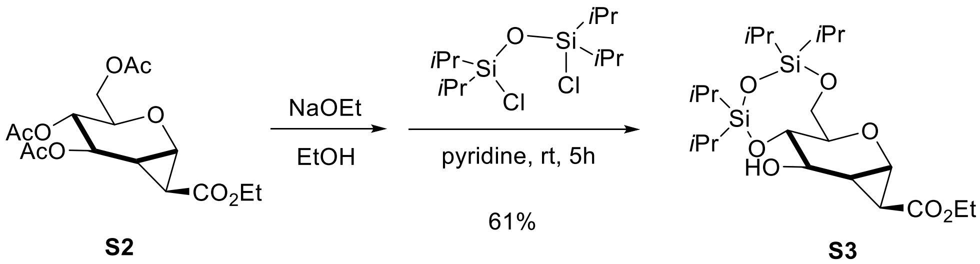

To a 250 ml round-bottomed flask fitted with a nitrogen inlet, a septum, and magnetic stir-bar was added **S2** (15.0 g, 41.8 mmol, 1.0 equiv) followed by dry EtOH (100 mL). Then NaOEt (142 mg, 2.1 mmol, 0.05 equiv) was added. The light-yellow solution was stirred at rt for overnight. The volatiles were removed under reduced pressures using rotary evaporation. The resulting yellow viscous reside was dissolved in $CHCl_3$ (100 mL) and once again concentrated under reduced pressure by rotary evaporation. This procedure was repeated two more times and the remaining volatiles were removed under high vacuum (0.06 mmHg) for approximately 2 h. Then dry pyridine (40 mL) was added to the crude yellow syrup, and the solution was cooled to –5 °C using an ice salt bath. 1,3-dichloro-1,1,3,3-tetraisopropyldisiloxane (13.4 mL, 41.8 mmol, 1.0 equiv) was added. The contents were warmed to rt and stirred for 5 h. The solvent was removed by rotary evaporation to afford a light-yellow oil. This solution was diluted with EtOAc (200 mL) and washed with HCl (1 M) until neutral, then the organic extracts were washed with brine (100 mL), dried over Na_2SO_4 and filtered. The crude reside was purified by column chromatography (*n*-pentane/EtOAc, 15:1), which afforded 12.2 g (61%) of **S3** as an oil.

^{1}H NMR (600 MHz, $CDCl_3$) δ 4.11 (q, *J* = 7.1 Hz, 2H), 4.06 (dd, *J* = 7.7, 2.7 Hz, 1H), 4.01 (dd, *J* = 12.4, 1.8 Hz, 1H), 3.85 (d, *J* = 8.5 Hz, 1H), 3.81 (dd, *J* = 12.4, 1.9 Hz, 1H), 3.73 (dd, *J* = 9.7, 8.3 Hz, 1H), 3.05 (dt, *J* = 9.7, 1.9 Hz, 1H), 2.30 (s, 1H), 1.95 (dd, *J* = 5.6, 2.7 Hz, 1H), 1.73-1.70 (m, 1H), 1.25 (t, *J* = 7.1 Hz, 3H), 1.12-1.02 (m, 28H). **^{13}C NMR** (151 MHz, $CDCl_3$) δ 171.86, 74.11, 73.24, 70.21, 60.99, 60.88, 59.98, 25.84, 22.54, 17.67, 17.44, 17.43, 17.40, 17.36, 17.22, 14.37, 13.65, 13.40, 12.70, 12.59. **ESI-HRMS**: Calculated for $C_{22}H_{43}O_7Si_2$ $(M+H)^+$: 475.2542, Found: 475.2540.

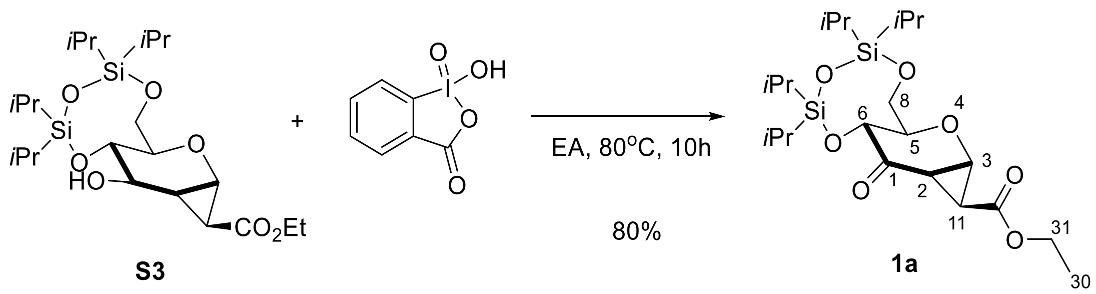

S3 (7.5 g, 15.8 mmol, 1.0 equiv) was dissolved in ethyl acetate (100 ml), and IBX (9.73 g, 34.7 mmol, 2.2 equiv) was added. The resulting suspension was immersed in an oil bath set to 80 °C and stirred for 10 h, the reaction was cooled to room temperature and filtered. The filter cake was washed with 3 × 30 mL of ethyl acetate, and the combined filtrates were concentrated and purified by column chromatography (*n*-pentane/EtOAc, 25:1~20:1), which afforded 6.0 g (80%) of **1a** as an oil.

^{1}H NMR (700 MHz, $CDCl_3$) δ 4.34 (dd, *J* = 6.3, 2.8 Hz, 1H, H-3), 4.25 (dd, *J* = 10.6, 0.7 Hz, 1H, H-6), 4.16-4.13 (m, 2H, H-31), 4.02 (dd, *J* = 12.5, 2.0 Hz, 1H, H-8), 3.93 (dd, *J* = 12.6, 1.5 Hz, 1H, H-8), 3.56 (d, *J* = 10.6 Hz, 1H, H-5), 2.75 (dd, *J* = 5.0, 2.7 Hz, 1H, H-2), 2.34 (ddd, *J* = 6.1, 5.0, 0.8 Hz, 1H, H-11), 1.25 (t, *J* = 7.1 Hz, 3H, H-30), 1.13 (d, *J* = 7.5 Hz, 3H), 1.10-0.96 (m, 25H). **^{13}C NMR** (176 MHz, $CDCl_3$) δ 199.73, 169.71, 72.61, 70.76, 61.66, 61.27, 60.51, 59.83, 29.25, 20.75, 17.38, 17.24, 17.19, 17.18, 17.11, 16.88, 14.33, 14.25, 13.63, 13.28, 12.60, 12.30. **ESI-HRMS**: Calculated for $C_{22}H_{41}O_7Si_2$ $(M+H)^+$: 473.2385, Found: 473.2385.

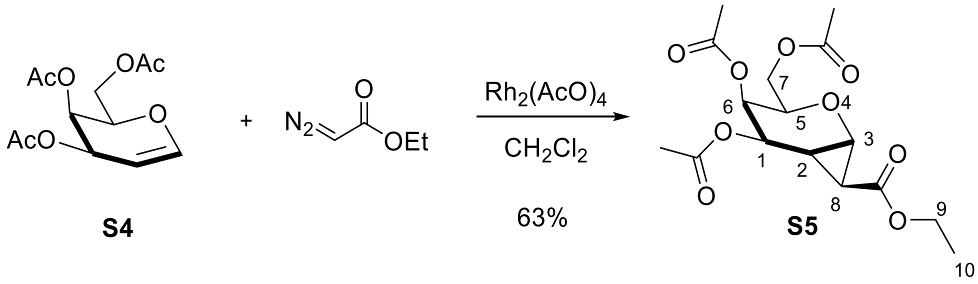

Compound **S5** was prepared according to the literature procedure.[9] A mixture of Tri-O-acetyl-D-Galactal **S4** (12 g, 44 mmol, 1.0 equiv) and rhodium acetate dimer (195 mg, 0.441 mmol, 0.01 equiv) in dichloromethane (250 ml) was vigorously stirred under an argon atmosphere. Ethyl diazoacetate (12 g, 105 mmol, 2.4 equiv) in dichloromethane (12 ml) was added dropwise to the mixture over the course of 14 hours via syringe pump, after which time TLC (hexanes/ethyl acetate 1:1) showed the reaction to be complete. The mixture was evaporated under reduced pressure to give a green syrup which was purified by flash chromatography. Elution with *n*-pentane/ethyl acetate (5:1~4:1) gave 10 g **S5** as light-yellow oil, 63% yield. (Compound **S5** contains a small amount of inseparable isomers.)

^{1}H NMR (600 MHz, $CDCl_3$) δ 5.63-5.62 (m, 0.4H), 5.49-5.48 (m, 0.4H), 5.18 (dd, *J* = 5.0, 1.7 Hz, 1H, H-1), 5.11 (t, *J* = 4.9 Hz, 1H, H-6), 4.36 (td, *J* = 6.7, 2.8 Hz, 0.4H), 4.33 (dd, *J* = 11.9, 8.0 Hz, 1H, H-7), 4.17-4.15 (m, 1.2 H), 4.12 (q, *J* = 7.1 Hz, 2H, H-9), 4.09-4.06 (m, 1.2H, H-7), 4.00-3.98 (m, 1H, H-3), 3.81-3.79 (m, 1H, H-5), 2.09 (s, 3H, OAc), 2.08 (s, 3H, OAc), 2.07 (s, 3H, OAc), 1.85-1.80 (m, 2H, H-8, H-2), 1.68-1.66 (m, 1H), 1.56-1.53 (m, 0.3H),1.25 (t, *J* = 7.1 Hz, 3H, H-10). **^{13}C NMR** (151 MHz, $CDCl_3$) δ 170.77, 170.69, 169.63, 169.57, 72.08, 68.49, 67.00, 61.91, 61.17, 55.99, 24.71, 23.23, 21.03, 20.97, 20.90, 14.31. **ESI-HRMS**: Calculated for $C_{16}H_{22}O_9Na$ $(M+Na)^+$: 381.1156, Found: 381.1161.

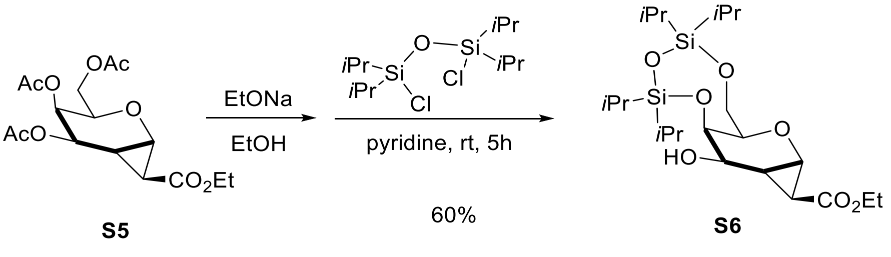

To a 250 ml round-bottomed flask fitted with a nitrogen inlet, a septum, and magnetic stir-bar was added **S5** (10.0 g, 27.9 mmol, 1.0 equiv) followed by dry EtOH (100 mL). Then NaOEt (95 mg, 1.4 mmol, 0.05 equiv) was added. The light-yellow solution was stirred at rt for overnight. The volatiles were removed under reduced pressures using rotary evaporation. The resulting yellow viscous reside was dissolved in $CHCl_3$ (100 mL) and once again concentrated under reduced pressure by rotary evaporation. This procedure was repeated two more times and the remaining volatiles were removed under high vacuum (0.06 mmHg) for approximately 2 h. Then dry pyridine (40 mL) was added to the crude yellow syrup, and the solution was cooled to –5 °C using an ice salt bath. 1,3-dichloro-1,1,3,3-tetraisopropyldisiloxane (8.9 mL, 27.9 mmol, 1.0 equiv) was added. The contents were warmed to rt and stirred for 5 h. The solvent was removed by rotary evaporation to afford a light-yellow oil. This solution was diluted with EtOAc (200 mL) and washed with HCl (1 M) until neutral, then the organic extracts were washed with brine (100 mL), dried over Na_2SO_4 and filtered. The crude reside was purified by column chromatography (*n*-pentane/EtOAc, 15:1), which afforded 8.0 g (60%) of **S6** as an oil.

^{1}H NMR (600 MHz, $CDCl_3$) δ 5.18 (dd, *J* = 3.6, 2.2 Hz, 1H), 5.11 (t, *J* = 3.4 Hz, 1H), 4.33 (dd, *J* = 11.9, 8.0 Hz, 1H), 4.12 (q, *J* = 7.1 Hz, 2H), 3.98 (dd, *J* = 7.1, 2.5 Hz, 1H), 3.80 (ddd, *J* = 7.8, 4.5, 3.1 Hz, 1H), 2.10 (s, 3H), 2.08 (s, 3H), 2.07 (s, 3H), 1.85-1.83 (m, 1H), 1.25 (t, *J* = 7.1 Hz, 3H). **^{13}C NMR** (151 MHz, $CDCl_3$) δ 170.93, 170.78, 170.01, 169.90, 71.05, 66.52, 64.74, 62.26, 61.17, 56.73, 25.10, 22.86, 20.93, 20.91, 20.76, 14.32. **ESI-HRMS**: Calculated for $C_{22}H_{43}O_7Si_2$ $(M+H)^+$: 475.2542, Found: 475.2540.

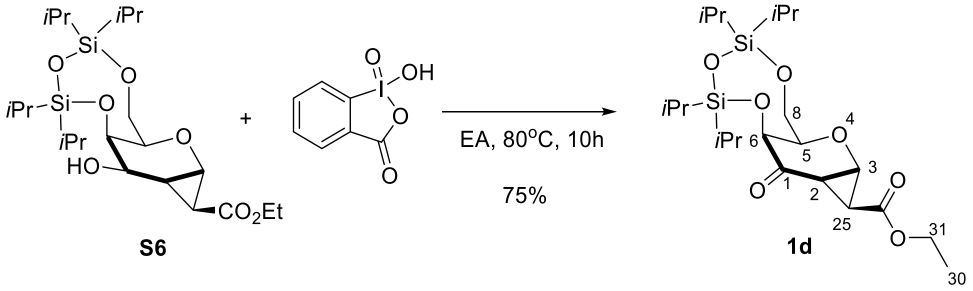

S6 (8.0 g, 16.8 mmol, 1.0 equiv) was dissolved in ethyl acetate (100 ml), and IBX (10.4 g, 37 mmol, 2.2 equiv) was added. The resulting suspension was immersed in an oil bath set to 80 °C and stirred for 12 h, the reaction was cooled to room temperature and filtered. The filter cake was washed with 3 × 30 mL of ethyl acetate, and the combined filtrates were concentrated and purified by column chromatography (*n*-pentane/EtOAc, 25:1~20:1), which afforded 6.0 g (75%) of **1d** as an oil.

^{1}H NMR (700 MHz, $CDCl_3$) δ 4.36 (dd, *J* = 6.4, 2.6 Hz, 1H, H-3), 4.14 (qd, *J* = 7.1, 1.5 Hz, 2H, H-31), 3.98 (d, *J* = 2.3 Hz, 1H, H-6), 3.92 (dd, *J* = 9.2, 3.1 Hz, 1H, H-8), 3.89-3.85 (m, 2H, H-8, H-5), 2.53 (dd, *J* = 5.2, 2.6 Hz, 1H, H-2), 2.44 (dd, *J* = 6.4, 5.2 Hz, 1H, H-25), 1.26 (t, *J* = 7.1 Hz, 3H, H-30), 1.08-1.01 (m, 28H). **^{13}C NMR** (176 MHz, $CDCl_3$) δ 198.04, 169.56, 74.72, 68.38, 61.60, 60.62, 60.13, 30.07, 24.81, 17.50, 17.44, 17.43, 17.41, 17.34, 17.32, 17.31, 17.10, 14.28, 13.66, 13.32, 13.18, 12.77. **ESI-HRMS**: Calculated for $C_{22}H_{41}O_7Si_2$ $(M+H)^+$: 473.2385, Found: 473.2391.

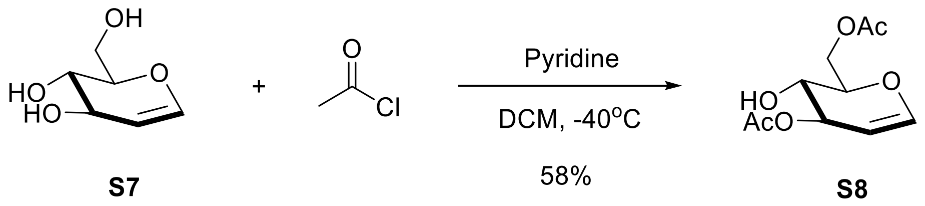

Compound **S8** was prepared according to the literature modified procedure.[10] To a solution of D-Glucal **S7** (9.79 g, 67 mmol, 1.0 equiv) in anhydrous CH_2Cl_2 (100 mL), pyridine (13.5 mL, 167.5 mmol, 2.5 equiv) was added under an argon atmosphere. After cooling to -40 °C, acetyl chloride (9.5 mL, 134 mmol, 2.0 equiv) in dichloromethane (15 ml) was added dropwise to the mixture over the course of 0.5 hours via syringe pump, and the mixture was stirred for 1 hour at the same temperature, then stirred for 2 h at room temperature. After consumption of starting material, the mixture was quenched with H_2O and organic materials were extracted and washed with HCl (1 M) until neutral, then the organic extracts

were washed with brine (100 mL), dried over Na_2SO_4 and filtered. The crude reside was purified by column chromatography (*n*-pentane/EtOAc, 6:1), which afforded 9.0 g (58%) of **S8** as an oil.

^{1}H NMR (500 MHz, $CDCl_3$) δ 6.42 (dd, *J* = 6.1, 1.6 Hz, 1H), 5.28 (ddd, *J* = 6.9, 2.6, 1.6 Hz, 1H), 4.72 (dd, *J* = 6.1, 2.5 Hz, 1H), 4.48 (dd, *J* = 12.3, 4.7 Hz, 1H), 4.39 (dd, *J* = 12.3, 2.5 Hz, 1H), 3.99 (ddd, *J* = 9.9, 4.7, 2.5 Hz, 1H), 3.84-3.82 (m, 1H), 3.74 (broad, 1H), 2.11 (s, 3H), 2.10 (s, 3H). **^{13}C NMR** (125 MHz, $CDCl_3$) δ 172.40, 171.61, 146.07, 99.44, 76.50, 73.02, 67.20, 62.65, 21.29, 20.95.

The analytical data are in accordance with the reported literature.[11]

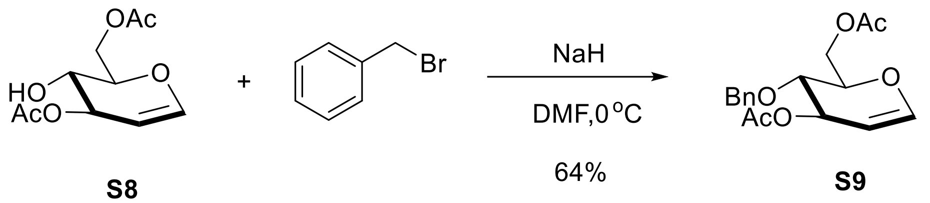

S8 **S9**

The suspension of NaH (1.758 g, 43.96 mmol, 1.1 equiv) in DMF (60 mL) was prepared and cooled down to 0 °C. **S8** (9.2 g, 39.96 mmol, 1.0 equiv) was added and obtained mixture was stirred at 0 °C for 30 min., then benzyl bromide (5.26 mL, 43.96 mmol, 1.1 equiv) was added dropwise. The cooling bath was removed and stirring was continued overnight. After reaction was completed, the reaction was quenched with NH_4Cl solution, water (150 ml) was added and solution was extracted with ethyl acetate (3 x 100 mL). Combined organic extracts were washed with water, then the organic extracts were washed with brine (100 mL), dried over Na_2SO_4 and filtered. The crude reside was purified by column chromatography (*n*-pentane/EtOAc, 10:1~5:1), which afforded 8.2 g (64%) of **S9** as an oil.

^{1}H NMR (500 MHz, $CDCl_3$) δ 7.36-7.28 (m, 5H), 6.41 (dd, *J* = 6.1, 1.4 Hz, 1H), 5.42 (ddd, *J* = 6.0, 3.2, 1.4 Hz, 1H), 4.80 (dd, *J* = 6.1, 3.1 Hz, 1H), 4.71 (d, *J* = 11.6 Hz, 1H), 4.64 (d, *J* = 11.6 Hz, 1H), 4.40-4.31 (m, 2H), 4.15 (ddd, *J* = 8.4, 5.5, 3.1 Hz, 1H), 3.81 (dd, *J* = 8.2, 5.9 Hz, 1H), 2.05 (s, 3H), 2.02 (s, 3H). **^{13}C NMR** (125 MHz, $CDCl_3$) δ 170.42, 170.26, 145.36, 137.40, 128.42, 128.41, 127.92, 127.88, 99.15, 74.88, 73.16, 72.69, 70.13, 62.12, 21.07, 20.68. **ESI-HRMS**: Calculated for $C_{17}H_{20}O_6Na$ $(M+Na)^+$: 343.1152, Found: 343.1155.

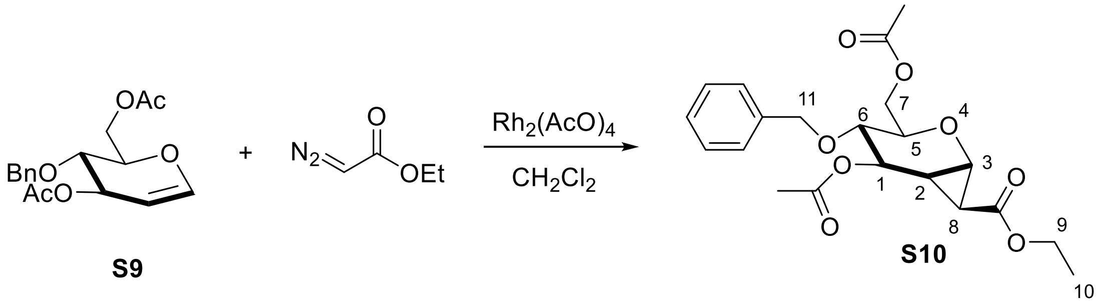

S9 **S10**

A mixture of **S9** (7.56 g, 23.6 mmol, 1.0 equiv) and rhodium acetate dimer (156 mg, 0.354 mmol, 0.015 equiv) in dichloromethane (250 ml) was vigorously stirred under an argon atmosphere. Ethyl diazoacetate (7.45 ml, 70.8 mmol, 3.0 equiv) in dichloromethane (12 ml) was added dropwise to the mixture over the course of 14 hours via syringe pump, after which time TLC showed the reaction to be complete. The mixture was evaporated under reduced pressure to give a green syrup which was purified by flash chromatography. Elution with *n*-pentane/ethyl acetate (10:1~3:1) gave **S10** as light-yellow oil. (Compound **S10** contains a small amount of inseparable isomers.)

^{1}H NMR (500 MHz, $CDCl_3$) δ 7.36-7.29 (m, 5H, Ar-H), 5.20 (dd, *J* = 4.7, 1.6 Hz, 1H, H-1), 4.66 (d, *J* = 11.8 Hz, 1H, H-11), 4.58 (d, *J* = 11.9 Hz, 1H, H-11), 4.37 (dd, *J* = 11.9, 7.3 Hz, 1H, H-7), 4.13-4.09 (m, 3H, H-7, H-9), 3.90 (dd, *J* = 7.1, 2.1 Hz, 1H, H-3), 3.85-3.81 (m, 1H, H-5), 3.44 (td, *J* = 4.8, 0.9 Hz, 1H, H-6), 2.22 (d, *J* = 2.1 Hz, 1H, H-2), 2.09 (s, 3H, OAc), 2.06 (s, 3H, OAc), 1.73 (t, *J* = 6.6 Hz, 1H, H-8), 1.25 (t, *J* = 7.2 Hz, 3H, H-10). **^{13}C NMR** (125 MHz, $CDCl_3$) δ 171.28, 170.79, 169.86, 137.35, 128.65, 128.46, 128.23, 128.16, 127.97, 73.70, 72.68, 72.32, 67.84, 62.59, 61.03, 56.19, 24.26, 23.58, 21.21, 20.98, 14.33. **ESI-HRMS**: Calculated for $C_{21}H_{27}O_8$ $(M+H)^+$: 407.1700, Found: 407.1703.

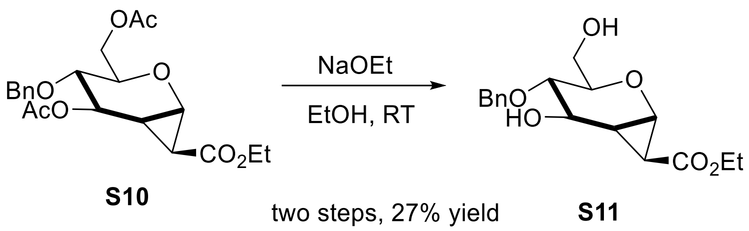

A mixture of **S10** (last step) in EtOH (80 ml) and a catalytic NaOEt was added to the mixture and the reaction was continued overnight, after which time TLC showed the reaction to be complete. The mixture was evaporated under reduced pressure and purified by flash chromatography. Elution with *n*-pentane/ethyl acetate (2:1) gave 2.05 g (27% yield, two steps) of **S11** as an oil.

^{1}H NMR (600 MHz, $CDCl_3$) δ 7.37-7.29 (m, H), 4.67 (s, 2H), 4.10 (qd, *J* = 7.1, 2.3 Hz, 2H), 4.02-4.01 (m, 1H), 3.97 (dd, *J* = 7.4, 2.3 Hz, 1H), 3.77 (t, *J* = 3.7 Hz, 2H), 3.50 (dt, *J* = 6.8, 4.1 Hz, 1H), 3.39 (t, *J* = 6.4 Hz, 1H), 3.19 (s, 1H), 2.58 (s, 1H), 2.07 (dd, *J* = 5.7, 2.3 Hz, 1H), 1.73 (ddd, *J* = 7.4, 5.7, 1.7 Hz, 1H), 1.24 (t, *J* = 7.1 Hz, 3H). **^{13}C NMR** (151 MHz, $CDCl_3$) δ 172.00, 137.92, 128.74, 128.17, 128.03, 77.67, 74.07, 73.40, 68.54, 62.48, 60.99, 58.21, 26.42, 23.91, 14.31. **ESI-HRMS**: Calculated for $C_{17}H_{23}O_6$ $(M+H)^+$: 323.1489, Found: 323.1494.

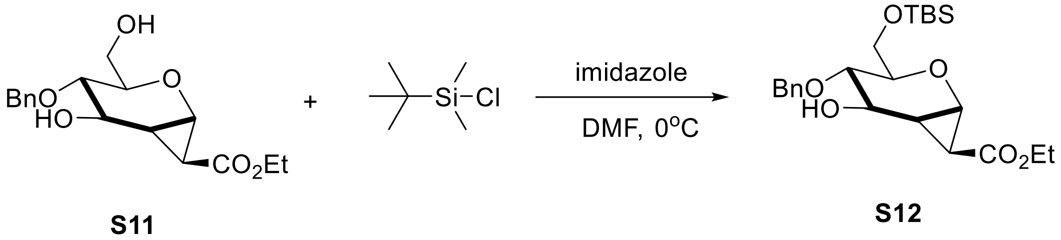

S11 (2.7 g, 8.38 mmol, 1.0 equiv) was dissolved in anhydrous DMF (20 ml) and cooled to 0 °C. Imidazole (1.14 g, 16.7 mmol, 2.0 equiv) and then tertbutyldimethylsilyl chloride (1.26 g, 8.38 mmol, 1.0 equiv) were added to the solution, and the mixture was stirred for 15 h, when TLC indicated complete consumption of starting material, water (80 ml) was added and solution was extracted with ethyl acetate (3 x 60 mL). Combined organic extracts were washed with water, then the organic extracts were washed with brine (100 mL), dried over Na_2SO_4 and filtered. The crude reside was purified by column chromatography (*n*-pentane/EtOAc, 8:1~6:1), which afforded **S12** as an oil.

^{1}H NMR (500 MHz, $CDCl_3$) δ 7.36-7.29 (m, 5H), 4.65 (s, 2H), 4.11-4.07 (m, 2H), 3.99-3.96 (m, 1H), 3.93 (dd, *J* = 7.3, 2.1 Hz, 1H), 3.87-3.78 (m, 2H), 3.63 (d, *J* = 6.5 Hz, 1H), 3.59-3.56 (m, 1H), 3.49 (t, *J* = 5.3 Hz, 1H), 2.08 (dd, *J* = 5.7, 2.0 Hz, 1H), 1.76 (td, *J* = 5.6, 2.9 Hz, 1H), 1.23 (t, *J* = 7.1 Hz, 3H), 0.92 (s, 9H), 0.12 (s, 3H), 0.10 (s, 3H). **^{13}C NMR** (126 MHz, $CDCl_3$) δ 171.86, 138.04, 128.61, 128.61, 127.97, 127.86, 78.22, 74.98, 72.82, 66.86, 64.03, 60.64, 58.03, 26.82, 25.89, 25.28, 18.33, 14.28, -5.22, -5.51. **ESI-HRMS**: Calculated for $C_{23}H_{37}O_6Si$ $(M+H)^+$: 437.2354, Found: 437.2353.

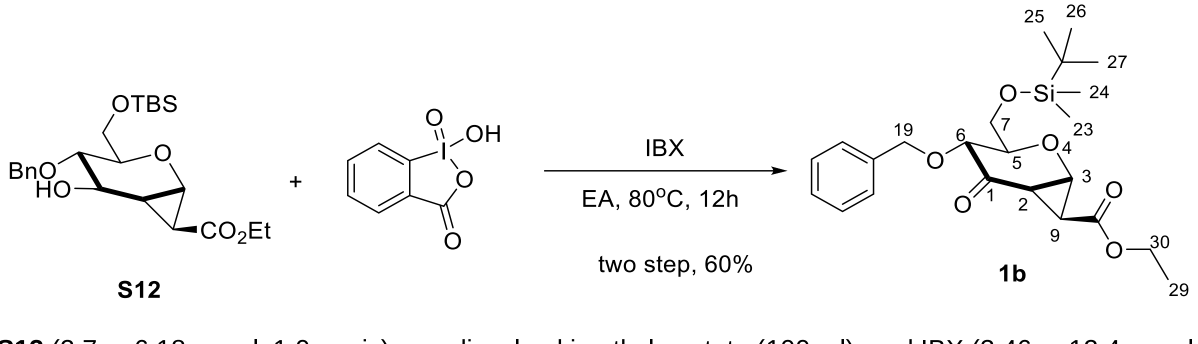

S12 (2.7 g, 6.18 mmol, 1.0 equiv) was dissolved in ethyl acetate (100 ml), and IBX (3.46 g, 12.4 mmol, 2.0 equiv) was added. The resulting suspension was immersed in an oil bath set to 80 °C and stirred for 12 h, the reaction was cooled to room temperature and filtered. The filter cake was washed with 3 × 30 mL of ethyl acetate, and the combined filtrates were concentrated and purified by column

chromatography (*n*-pentane/EtOAc, 25:1~20:1), which afforded 2.0 g (two steps, 60% yield) of **1b** as an oil.

^{1}H NMR (500 MHz, $CDCl_3$) δ 7.35-7.29 (m, 5H, Ar-H), 4.79 (d, *J* = 11.5 Hz, 1H, H-19), 4.51 (d, *J* = 11.4 Hz, 1H, H-19), 4.30 (dd, *J* = 6.5, 2.5 Hz, 1H, H-3), 4.13 (q, *J* = 7.1 Hz, 2H, H-30), 3.95 (q, *J* = 4.0 Hz, 1H, H-5), 3.78-3.75 (m, 3H, H-7, H-6), 2.97 (dd, *J* = 5.3, 2.5 Hz, 1H, H-2), 2.38 (ddd, *J* = 6.5, 5.2, 1.4 Hz, 1H, H-9), 1.26 (t, *J* = 7.1 Hz, 3H, H-29), 0.87 (s, 9H, H-25,26,27), 0.05 (s, 3H, H-23), 0.03 (s, 3H, H-24). **^{13}C NMR** (126 MHz, $CDCl_3$) δ 198.41, 169.55, 137.01, 128.60, 128.37, 128.19, 78.65, 78.14, 72.92, 63.31, 61.46, 61.41, 31.92, 28.63, 25.91, 18.40, 14.26, -5.44, -5.53. **ESI-HRMS**: Calculated for $C_{23}H_{35}O_6Si$ $(M+H)^+$: 435.2197, Found: 435.2198.

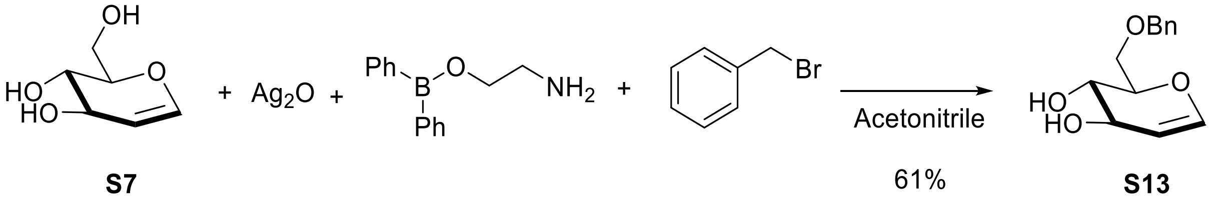

S7 **S13**

Compound **S13** was prepared according to the literature procedure.[12] Silver oxide (12.78 g, 55.15 mmol, 1.0 equiv) and 2-aminoethyl diphenylborinate (1.24 g, 5.52 mmol, 0.1 equiv) were added successively to a stirred solution of D-Glucal **S7** (8.06 g, 55.15 mmol, 1.0 equiv) diluted in acetonitrile (300 mL). Benzyl bromide (13.1 mL, 110.3 mmol, 2.0 equiv) was added dropwise via addition funnel, and the reaction mixture was stirred at rt for 12 h. Following reaction completion, the reaction mixture was filtered on a celite pad, washed with EtOAc, and then concentrated to remove most of the acetonitrile. EtOAc (300 mL) was added and then brine (300 mL) was used to wash the organic layer. The organic layer was dried over sodium sulfate, filtered, and concentrated to a residue. The crude product was purified by silica gel chromatography with 1:1 hexanes:EtOAc to yield 7.96 g (61%) of **S13** as an off-white solid.

^{1}H NMR (600 MHz, $CDCl_3$) δ 7.36-7.33 (m, 4H), 7.30-7.28 (m, 1H), 6.32 (dd, *J* = 6.1, 1.8 Hz, 1H), 4.69 (dd, *J* = 6.1, 2.2 Hz, 1H), 4.62 (d, *J* = 12.0 Hz, 1H), 4.56 (d, *J* = 12.0 Hz, 1H), 4.21 (d, *J* = 7.8 Hz, 1H), 3.88 (dt, *J* = 9.8, 3.9 Hz, 1H), 3.82-3.74 (m, 3H), 3.55 (s, 1H), 3.06 (s, 1H). **^{13}C NMR** (151 MHz, $CDCl_3$) δ 144.43, 137.72, 128.64, 128.05, 127.96, 102.86, 76.86, 73.90, 71.17, 69.85, 69.39.

The analytical data are in accordance with the reported literature.[12]

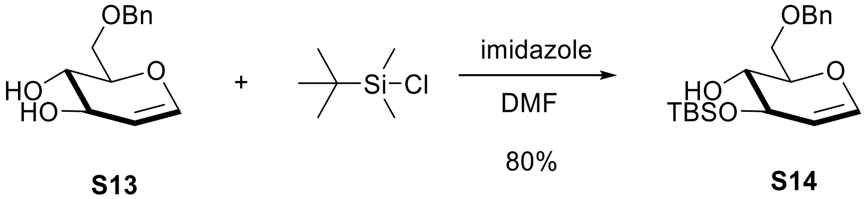

S13 **S14**

Compound **S14** was prepared according to the literature procedure.[12] A solution of **S13** (7.96 g, 33.69 mmol, 1.0 equiv) in anhydrous DMF (60 mL) was cooled to 0 °C with an ice bath under a stream of argon. Imidazole (4.59 g, 67.38 mmol, 2.0 equiv) and 4-dimethyl aminopyridine (205 mg, 1.68 mmol, 0.05 equiv) were added successively and allowed to stir for 5 mins. Next, *tert*butyldimethylsilyl chloride (5.08 g, 33.69 mmol, 1.0 equiv) was added and the reaction was stirred for 15 minutes before removing the ice bath. The reaction mixture was stirred for 12 h and reaction completion was determined through TLC analysis. The reaction mixture was diluted with diethyl ether (200 mL) and then quenched with brine (200 mL). The brine layer was extracted with diethyl ether (3 x 100 mL) and the combined organic extracts were washed with H_2O (2 x 300 mL) and brine (300 mL). The organic layer was dried over sodium sulfate, filtered, and concentrated to a residue. The crude product was purified by silica gel chromatography with 12:1 hexanes:EtOAc to yield 9.49 g (80%) of **S14** as a clear oil.

^{1}H NMR (500 MHz, $CDCl_3$) δ 7.39-7.35 (m, 4H), 7.29 (dt, *J* = 8.8, 4.4 Hz, 1H), 6.31 (dd, *J* = 6.2, 1.5 Hz, 1H), 4.65-4.57 (m, 3H), 4.22 (dt, *J* = 6.6, 2.0 Hz, 1H), 3.99 (ddd, *J* = 8.9, 5.4, 3.2 Hz, 1H), 3.84-3.75 (m,

3H), 2.35 (d, J = 4.2 Hz, 1H), 0.90 (s, 9H), 0.11 (s, 6H). **^{13}C NMR** (126 MHz, $CDCl_3$) δ 143.50, 137.97, 128.56, 127.94, 127.89, 103.64, 77.11, 73.75, 70.84, 69.94, 69.28, 26.01, 25.92, 18.21, -4.31, -4.42.

The analytical data are in accordance with the reported literature.[12]

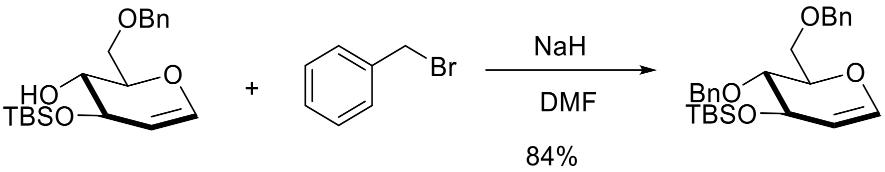

The suspension of NaH (1.25 g, 31.14 mmol, 1.15 equiv) in DMF (60 mL) was prepared and cooled down to 0 °C. **S14** (9.49 g, 27.07 mmol, 1.0 equiv) was added and obtained mixture was stirred at 0 °C for 30 min., then benzyl bromide (3.54 mL, 29.79 mmol, 1.1 equiv) was added dropwise. The cooling bath was removed and stirring was continued overnight. After reaction was completed, the reaction was quenched with NH_4Cl solution, water (150 ml) was added and solution was extracted with ethyl acetate (3 x 100 mL). Combined organic extracts were washed with water, then the organic extracts were washed with brine (100 mL), dried over Na_2SO_4 and filtered. The crude reside was purified by column chromatography (*n*-pentane/EtOAc, 10:1), which afforded 10.0 g (84%) of **S15** as an oil.

^{1}H NMR (500 MHz, $CDCl_3$) δ 7.26-7.17 (m, 7H), 6.26 (dd, J = 6.1, 1.4 Hz, 1H), 4.74 (d, J = 11.3 Hz, 1H), 4.58-4.57 (m, 2H), 4.49 (s, 2H), 4.28-4.26 (m, 1H), 4.00-3.97 (m, 1H), 3.70 (dd, J = 10.8, 5.6 Hz, 1H), 3.62 (dd, J = 10.8, 2.5 Hz, 1H), 3.58 (dd, J = 8.5, 6.0 Hz, 1H), 0.82 (s, 9H), 0.01 (d, J = 5.9 Hz, 6H). **^{13}C NMR** (126 MHz, $CDCl_3$) δ 143.61, 138.36, 138.22, 128.63, 128.60, 128.09, 128.04, 127.94, 127.86, 103.75, 76.89, 74.21, 73.71, 69.28, 68.91, 26.04, 18.21, -4.14, -4.41. **ESI-HRMS**: Calculated for $C_{26}H_{36}O_4SiNa$ $(M+Na)^+$: 463.2275, Found: 463.2270.

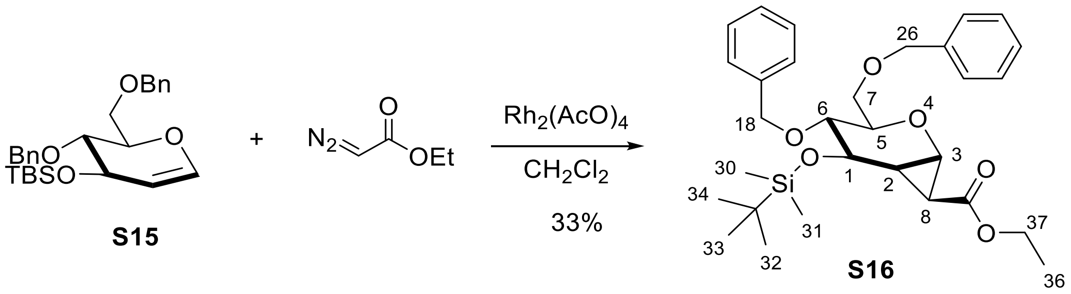

A mixture of **S15** (3.00 g, 6.81 mmol, 1.0 equiv) and rhodium acetate dimer (30 mg, 0.068 mmol, 0.01 equiv) in dichloromethane (60 ml) was vigorously stirred under an argon atmosphere. Ethyl diazoacetate (1.79 ml, 17 mmol, 2.5 equiv) in dichloromethane (4 ml) was added dropwise to the mixture over the course of 8 hours via syringe pump, after which time TLC showed the reaction to be complete. The mixture was evaporated under reduced pressure to give a green syrup which was purified by flash chromatography. Elution with *n*-pentane/ethyl acetate (10:1~3:1) gave **S16** a light-yellow oil 1.2 g, 33% yield. (Compound **S16** contains a small amount of inseparable isomers.)

^{1}H NMR (500 MHz, $CDCl_3$) δ 7.29-7.20 (m, 10H, Ar-H), 4.61 (d, J = 11.7 Hz, 1H, H-18), 4.53-4.50 (m, 3H, H-18, H-26), 4.07 (qd, J = 7.1, 3.2 Hz, 2H, H-37), 3.94 (dd, J = 5.6, 2.1 Hz, 1H, H-1), 3.86 (dd, J = 7.3, 2.1 Hz, 1H, H-3), 3.74 (ddd, J = 6.7, 5.3, 2.9 Hz, 1H, H-5), 3.73-3.66 (m, 1H, H-7), 3.43 (dd, J = 10.6, 3.0 Hz, 1H, H-7), 3.31 (t, J = 5.4 Hz, 1H, H-6), 2.00 (dd, J = 5.8, 2.0 Hz, 1H, H-2), 1.60 (ddd, J = 7.5, 5.7, 2.1 Hz, 1H, H-8), 1.20 (t, J = 7.1 Hz, 3H, H-36), 0.84 (s, 9H, H32-34), 0.05 (s, 3H, H-30), 0.00 (s, 3H, H-31). **^{13}C NMR** (126 MHz, $CDCl_3$) δ 171.83, 138.14, 138.06, 128.51, 128.49, 128.42, 128.32, 127.95, 127.88, 127.85, 127.76, 127.66, 77.50, 74.74, 73.49, 73.05, 69.36, 69.22, 60.68, 57.05, 27.82, 25.98, 25.83, 25.23, 17.98, 14.37, -4.67, -4.86. **ESI-HRMS**: Calculated for $C_{30}H_{43}O_6Si$ $(M+H)^+$: 527.2823, Found: 527.2821.

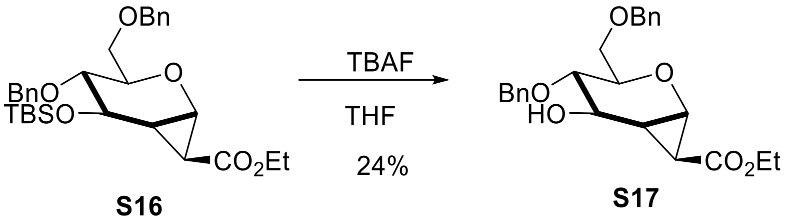

S16 **S17**

A solution of **S16** (1.3 g, 2.47 mmol, 1.0 equiv) in THF (12 mL) was cooled to 0 °C with an ice bath under a stream of argon. Tetra-n-butylammonium fluoride (1M in THF, 2.7 ml, 2.72 mmol, 1.1 equiv) was added dropwise and the solution was stirred at 0 °C, the reaction completion was determined through TLC analysis. The reaction mixture was poured into H_2O (50 mL) and extracted with diethyl ether (3 x 30 mL). The organic layer was dried over sodium sulfate, filtered, and concentrated to a residue. The crude product was purified by silica gel chromatography with 10:1 hexanes:EtOAc to yield 240 mg (24%) of **S17** as a clear oil.

^{1}H NMR (700 MHz, $CDCl_3$) δ 7.37-7.27 (m, 10H), 4.65-4.56 (m, 4H), 4.14-4.09 (m, 2H), 3.99-3.96 (m, 2H), 3.69 (dd, *J* = 8.4, 4.5 Hz, 2H), 3.67-3.65 (m, 1H), 3.45 (t, *J* = 5.5 Hz, 1H), 3.29 (d, *J* = 5.6 Hz, 1H), 2.08 (dd, *J* = 5.7, 2.1 Hz, 1H), 1.75 (ddd, *J* = 7.5, 5.6, 2.1 Hz, 1H), 1.25 (t, *J* = 7.2 Hz, 3H). **^{13}C NMR** (176 MHz, $CDCl_3$) δ 171.80, 137.94, 137.55, 128.54, 128.52, 127.91, 127.85, 127.83, 78.15, 73.89, 73.67, 72.93, 70.07, 67.93, 60.71, 57.78, 26.57, 24.76, 14.25. **ESI-HRMS**: Calculated for $C_{24}H_{29}O_6$ $(M+H)^+$: 413.1959, Found: 413.1959.

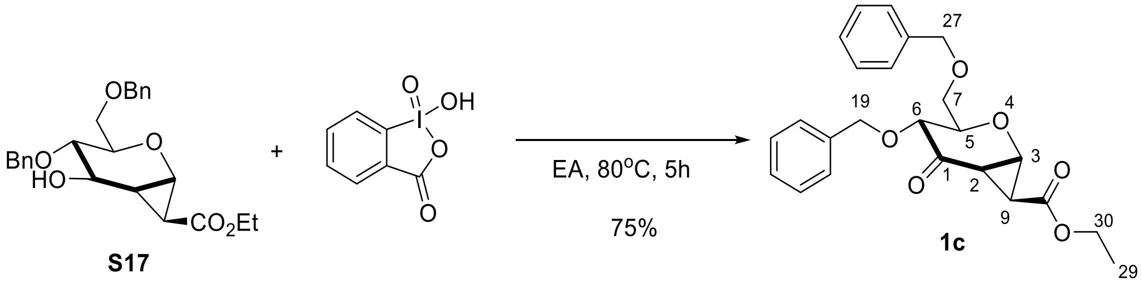

S17 (240 mg, 0.582 mmol, 1.0 equiv) was dissolved in ethyl acetate (15 ml), and IBX (358 mg, 1.28 mmol, 2.2 equiv) was added. The resulting suspension was immersed in an oil bath set to 80 °C and stirred for 5 h, the reaction was cooled to room temperature and filtered. The filter cake was washed with 3 × 10 mL of ethyl acetate, and the combined filtrates were concentrated and purified by column chromatography (*n*-pentane/EtOAc, 15:1), which afforded 180 mg (75% yield) of **1c** as an oil.

^{1}H NMR (500 MHz, $CDCl_3$) δ 7.37-7.27 (m, 10H, Ar-H), 4.82 (d, *J* = 11.4 Hz, 1H, H19), 4.55-4.47 (m, 3H, H-19, H-27), 4.33 (dd, *J* = 6.4, 2.6 Hz, 1H, H-3), 4.14 (qd, *J* = 7.1, 1.3 Hz, 2H, H-30), 4.04 (dt, *J* = 5.9, 4.0 Hz, 1H, H-5), 3.76 (dd, *J* = 5.8, 1.4 Hz, 1H, H-6), 3.63-3.57 (m, 2H, H-7), 2.98 (dd, *J* = 5.2, 2.6 Hz, 1H, H-2), 2.39 (ddd, *J* = 6.4, 5.1, 1.4 Hz, 1H, H-9), 1.26 (t, *J* = 7.1 Hz, 3H, H-29). **^{13}C NMR** (126 MHz, $CDCl_3$) δ 198.81, 169.43, 137.48, 136.91, 128.60, 128.43, 128.24, 128.01, 127.87, 77.70, 76.34, 73.78, 73.32, 69.28, 61.54, 61.24, 31.50, 27.12, 14.26. **ESI-HRMS**: Calculated for $C_{24}H_{27}O_6$ $(M+H)^+$: 411.1802, Found: 411.1803.

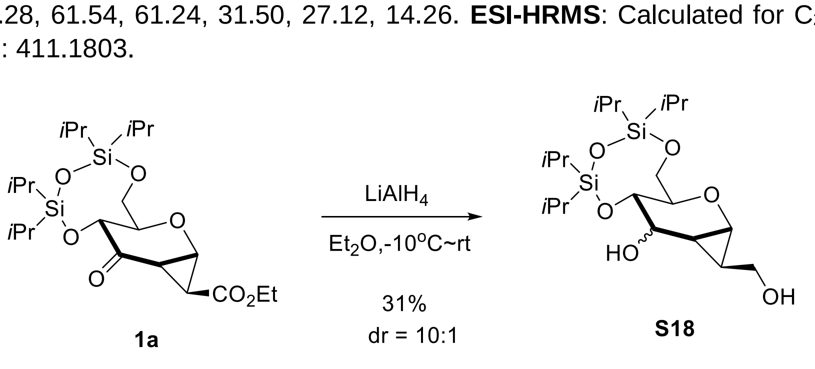

A solution of **1a** (1.0 g, 2.1 mmol, 1.0 equiv) in anhydrous Et_2O (10 mL) was cooled to -10 °C with an ice bath under a stream of argon. $LiAlH_4$ (79 mg, 2.1 mmol, 1.0 equiv) was added successively and

reaction completion was determined through TLC analysis. The reaction mixture was diluted with diethyl ether (30 mL) and then quenched with saturated ammonium chloride solution (20 mL). The saturated ammonium chloride layer was extracted with diethyl ether (3 x 20 mL) and the combined organic extracts were washed with H_2O (1 x 30 mL) and brine (30 mL). The organic layer was dried over sodium sulfate, filtered, and concentrated to a residue. The crude product was purified by silica gel to yield 280 mg (31%, dr = 10:1) of **S18** as oil.

^{1}H NMR (700 MHz, $CDCl_3$) δ 4.01 (dd, *J* = 12.3, 1.9 Hz, 1H), 3.80 (dd, *J* = 4.0, 2.1 Hz, 1H), 3.79 (d, *J* = 2.1 Hz, 1H), 3.70 (dd, *J* = 9.4, 8.4 Hz, 1H), 3.58 – 3.55 (m, 2H), 3.34 (dd, *J* = 11.5, 7.5 Hz, 1H), 3.09 (dt, *J* = 9.4, 2.1 Hz, 1H), 2.30 (br, 2H), 1.43 – 1.40 (m, 1H), 1.12 – 1.01 (m, 28H), 0.94 (td, *J* = 5.6, 2.8 Hz, 1H). **^{13}C NMR** (176 MHz, $CDCl_3$) δ 74.52, 74.28, 70.73, 63.74, 61.47, 56.43, 23.13, 20.75, 17.69, 17.44, 17.43, 17.41, 17.38, 17.36, 17.24, 13.65, 13.41, 12.71, 12.62. **ESI-HRMS**: Calculated for $C_{20}H_{41}O_6Si_2$ $(M+H)^+$: 433.2436, Found: 433.2433.

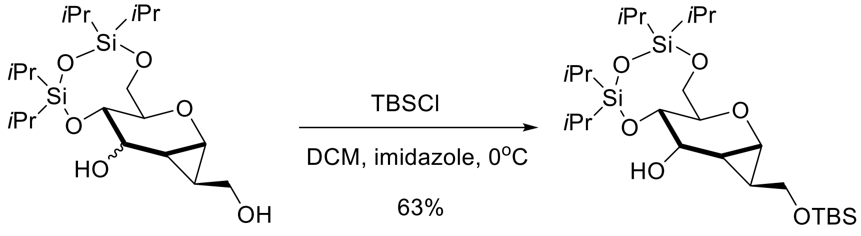

S18 **S19**

A solution of **S18** (280 mg, 0.647 mmol, 1.0 equiv) in anhydrous DCM (20 mL) was cooled to 0 °C with an ice bath under a stream of argon. Imidazole (70 mg, 0.78 mmol, 1.6 equiv) was added successively and allowed to stir for 5 mins. Next, *tert*butyldimethylsilyl chloride (70 mg, 1.04 mmol, 1.2 equiv) was added and the reaction was stirred for 15 minutes before removing the ice bath. The reaction mixture was stirred for 24 h and reaction completion was determined through TLC analysis. The reaction mixture was diluted with diethyl ether (20 mL) and then quenched with brine (20 mL). The brine layer was extracted with diethyl ether (3 x 10 mL) and the combined organic extracts were washed with H_2O (2 x 30 mL) and brine (30 mL). The organic layer was dried over sodium sulfate, filtered, and concentrated to a residue. The crude product was purified by silica gel chromatography to yield 223 mg (63%) of **S19** as a clear oil.

^{1}H NMR (700 MHz, $CDCl_3$) 4.01 (dd, *J* = 12.2, 1.8 Hz, 1H), 3.81 – 3.79 (m, 2H), 3.69 (dd, *J* = 9.5, 8.3 Hz, 1H), 3.58 (dd, *J* = 7.6, 3.2 Hz, 1H), 3.56 – 3.51 (m, 2H), 3.07 (dt, *J* = 9.5, 2.0 Hz, 1H), 2.27 (br, 1H), 1.31 – 1.28 (m, 1H), 1.13 – 1.01 (m, 28H), 0.96 – 0.93 (m, 1H), 0.86 (s, 9H), 0.02 (s, 6H). **^{13}C NMR** (176 MHz, $CDCl_3$) δ 74.52, 74.08, 71.01, 62.97, 61.45, 56.23, 26.04, 22.37, 20.01, 18.43, 17.72, 17.47, 17.44, 17.42, 17.40, 17.39, 17.38, 17.24, 13.66, 13.42, 12.72, 12.61, -5.07, -5.15. **ESI-HRMS**: Calculated for $C_{26}H_{55}O_6Si_3$ $(M+H)^+$: 547.3301, Found: 547.3300.

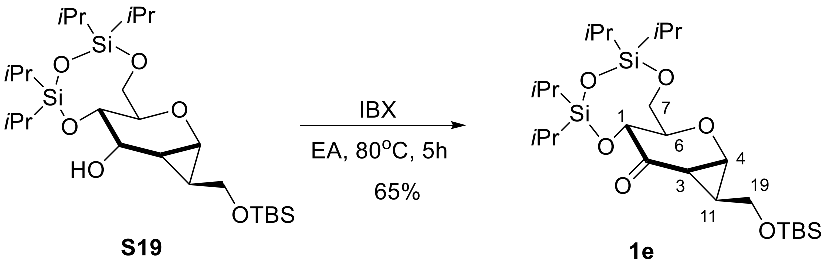

S19 **1e**

S19 (223 mg, 0.425 mmol, 1.0 equiv) was dissolved in ethyl acetate (15 ml), and IBX (357 mg, 1.28 mmol, 3.0 equiv) was added. The resulting suspension was immersed in an oil bath set to 80 °C and stirred for 5 h, the reaction was cooled to room temperature and filtered. The filter cake was washed with 3 × 10 mL of ethyl acetate, and the combined filtrates were concentrated and purified by column chromatography (*n*-pentane/EtOAc, 15:1), which afforded 150 mg (65% yield) of **1e** as an oil.

^{1}H NMR (600 MHz, $CDCl_3$) δ 4.15 (d, *J* = 10.4 Hz, 1H, H-6), 4.06 (dd, *J* = 5.9, 3.4 Hz, 1H, H-4), 4.01 (dd, *J* = 12.4, 2.0 Hz, 1H, H-7), 3.90 (d, *J* = 12.2 Hz, 1H, H-7), 3.76 (dd, *J* = 10.7, 3.9 Hz, 1H, H-19),

3.61 – 3.57 (m, 2H, H-19 & H-6), 2.22 (p, J = 4.2 Hz, 1H, H-11), 1.77 (t, J = 5.5 Hz, 1H, H-3), 1.16 – 1.00 (m, 28H), 0.83 (s, 9H), 0.00 (d, J = 2.6 Hz, 6H). **^{13}C NMR** (151 MHz, $CDCl_3$) δ 202.48, 71.86, 70.67, 61.52, 60.64, 57.35, 25.88, 25.50, 21.50, 18.28, 17.39, 17.38, 17.24, 17.19, 17.12, 16.83, 13.63, 13.27, 12.59, 12.23, -5.32, -5.38. **ESI-HRMS**: Calculated for $C_{26}H_{53}O_6Si_3$ $(M+H)^+$: 545.3145, Found: 545.3152.

NMR data

Ethyl(6a*R*,8*S*,9*R*,11a*R*)-2,2,4,4-tetraisopropyl-11-oxo-8-(((3a*R*,5*R*,5a*S*,8a*S*,8b*R*)-2,2,7,7-tetramethyltetrahydro-5H-bis([1,3]dioxolo)[4,5-b:4',5'-d]pyran-5-yl)methoxy)hexahydro-6H-oxepino[3,2-f][1,3,5,2,4]trioxadisilocine-9-carboxylate (3a)

The title product compound is prepared according to the general procedure **A** with 2 mol% catalyst **A** and 0.2 mmol cyclopropanated donor at 35 ˚C for 12 h and isolated by flash column chromatography (10:1 Pentane: Ethyl Acetate) giving **3a** as an oil (130 mg, 89% yield, α/β ratio > 20:1).

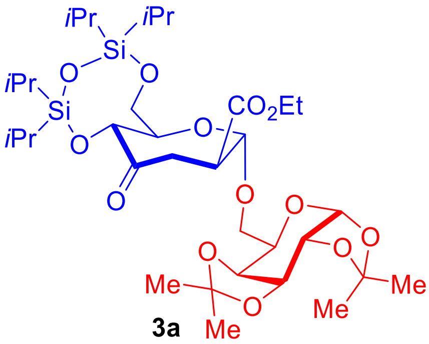

3a

^{1}H NMR (700 MHz, $CDCl_3$) δ 5.48 (d, *J* = 4.9 Hz, 1H), 5.26 (d, *J* = 6.3 Hz, 1H), 4.58 (dd, *J* = 7.9, 2.4 Hz, 1H), 4.45 (d, *J* = 9.7 Hz, 1H), 4.29 (dd, *J* = 4.9, 2.4 Hz, 1H), 4.18-4.10 (m, 4H), 3.94-3.90 (m, 2H), 3.86 (dd, J = 10.4, 7.0 Hz, 1H), 3.76 (dd, *J* = 10.4, 5.7 Hz, 1H), 2.86-2.79 (m, 2H), 2.60 (d, *J* = 13.1 Hz, 1H), 1.50 (s, 3H), 1.41 (s, 3H), 1.31 (d, *J* = 2.0 Hz, 6H), 1.24 (t, *J* = 7.1 Hz, 3H), 1.08-1.05 (m, 28H). **^{13}C NMR** (176 MHz, $CDCl_3$) δ 205.93, 171.37, 109.49, 108.73, 100.41, 96.41, 75.07, 71.14, 70.79, 70.62, 69.67, 67.15, 66.45, 63.32, 61.46, 45.32, 38.80, 26.16, 26.13, 25.04, 24.67, 17.47, 17.41, 17.37, 17.28, 17.26, 17.21, 17.10, 14.20, 13.41, 13.36, 12.55, 12.44. **ESI-HRMS**: Calculated for $C_{34}H_{61}O_{13}Si_2$ $(M+H)^+$: 733.36452, Found: 733.36616. $[\alpha]_D^{20}$ = -8.7 (c = 2.0, CH_2Cl_2).

Ethyl (2*S*,3*R*,6*R*,7*R*)-6-(benzyloxy)-7-(((tert-butyldimethylsilyl)oxy)methyl)-5-oxo-2-(((3a*R*,5*R*,5a*S*,8a*S*,8b*R*)-2,2,7,7-tetramethyltetrahydro-5H-bis([1,3]dioxolo)[4,5-b:4',5'-d]pyran-5-yl)methoxy)oxepane-3-carboxylate (3b)

The title product compound is prepared according to the general procedure **B** with 2 mol% catalyst **A** and 0.2 mmol cyclopropanated donor at rt for 4 h and isolated by flash column chromatography (10:1 Pentane: Ethyl Acetate) giving **3b** as an oil (125 mg, 90% yield, α/β ratio > 20:1).

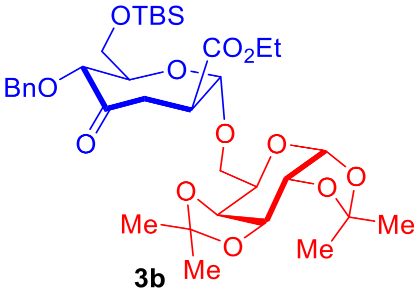

3b

^{1}H NMR (500 MHz, $CDCl_3$) δ 7.36-7.28 (m, 5H), 5.48 (d, *J* = 5.0 Hz, 1H), 5.04 (d, *J* = 8.6 Hz, 1H), 4.59-4.56 (m, 2H), 4.50 (d, *J* = 11.5 Hz, 1H), 4.29 (dd, *J* = 5.0, 2.4 Hz, 1H), 4.19-4.12 (m, 4H), 3.96 (d, *J* = 5.1 Hz, 1H), 3.91 (td, *J* = 6.5, 1.9 Hz, 1H), 3.81 (dd, *J* = 10.3, 6.5 Hz, 1H), 3.74-3.67 (m, 2H), 3.64-3.58 (m, 2H), 2.67-2.56 (m, 2H), 1.51 (s, 3H), 1.42 (s, 3H), 1.31 (s, 6H), 1.27 (t, *J* = 7.2 Hz, 3H), 0.88 (s, 9H), 0.06 (s, 6H). **^{13}C NMR** (126 MHz, $CDCl_3$) δ 206.06, 171.35, 137.37, 128.56, 128.13, 128.10, 109.32, 108.68, 101.63, 96.37, 85.66, 73.23, 71.80, 70.95, 70.69, 70.67, 67.03, 66.32, 64.12, 61.22, 44.93, 39.74, 26.20, 26.11, 26.06, 25.06, 24.58, 18.50, 14.26, -5.23. **ESI-HRMS**: Calculated for $C_{35}H_{55}O_{12}Si$ $(M+H)^+$: 695.3457, Found: 695.3461. $[\alpha]_D^{20}$ = +4.0 (c = 2.0, CH_2Cl_2).

Ethyl (2*S*,3*R*,6*R*,7*R*)-6-(benzyloxy)-7-((benzyloxy)methyl)-5-oxo-2-(((3a*R*,5*R*,5a*S*,8a*S*,8b*R*)-2,2,7,7-tetramethyltetrahydro-5H-bis([1,3]dioxolo)[4,5-b:4',5'-d]pyran-5-yl)methoxy)oxepane-3-carboxylate (3c)

The title product compound is prepared according to the general procedure **B** with 2 mol% catalyst **A** and 0.2 mmol cyclopropanated donor at rt for 7 h and isolated by flash column chromatography (10:1 Pentane: Ethyl Acetate) giving **3c** as an oil (123 mg, 92% yield, α/β ratio > 20:1).

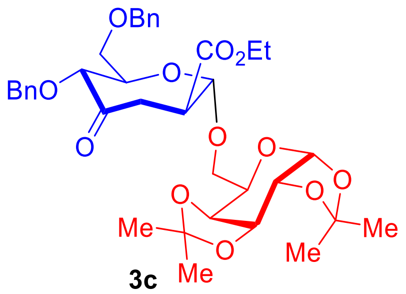

3c

^{1}H NMR (500 MHz, CD_2Cl_2) δ 7.36-7.27 (m, 10H), 5.45 (d, *J* = 5.0 Hz, 1H), 5.05 (d, *J* = 8.5 Hz, 1H), 4.61-4.44 (m, 5H), 4.29 (dd, *J* = 5.0, 2.4 Hz, 1H), 4.26 (t, *J* = 5.2 Hz, 1H), 4.18-4.13 (m, 3H), 3.96 (d, *J* = 5.6 Hz, 1H), 3.92 (td, *J* = 6.4, 1.9 Hz, 1H), 3.83 (dd, *J* = 10.3, 6.7 Hz, 1H), 3.66 (dd, *J* = 10.3, 6.1 Hz, 1H), 3.60 (dd, *J* = 10.2, 5.4 Hz, 1H), 3.57-3.50 (m, 2H), 2.67-2.59 (m, 2H), 1.49 (s, 3H), 1.39 (s, 3H), 1.31 (s, 6H), 1.27 (t, *J* = 7.1 Hz, 3H). **^{13}C NMR** (126 MHz, CD_2Cl_2) δ 205.77, 171.36, 138.56, 137.74, 128.75, 128.71, 128.38, 128.31, 128.02, 127.93, 109.49, 108.85, 101.92, 96.67, 85.68, 73.60, 73.35, 71.31, 71.15, 71.04, 70.96, 70.61, 67.44, 66.72, 61.53, 45.23, 39.98, 26.20, 26.17, 25.08, 24.62, 14.32. **ESI-HRMS**: Calculated for $C_{36}H_{46}O_{12}Na$ $(M+Na)^+$: 693.28815, Found: 693.28790. $[\alpha]_D^{20}$ = +9.9 (c = 2.0, CH_2Cl_2).

Ethyl (6a*R*,8*S*,9*R*,11a*R*)-2,2,4,4-tetraisopropyl-11-oxo-8-(((3a*S*,5a*R*,8a*R*,8b*S*)-2,2,7,7-tetramethyltetrahydro-3aH-bis([1,3]dioxolo)[4,5-b:4',5'-d]pyran-3a-yl)methoxy)hexahydro-6H-oxepino[3,2-f][1,3,5,2,4]trioxadisilocine-9-carboxylate (3d)

The title product compound is prepared according to the general procedure **A** with 2 mol% catalyst **A** and 0.2 mmol cyclopropanated donor at 35 ˚C for 12 h and isolated by flash column chromatography (10:1 Pentane: Ethyl Acetate) giving **3d** as an oil (120 mg, 82% yield, α/β ratio > 20:1).

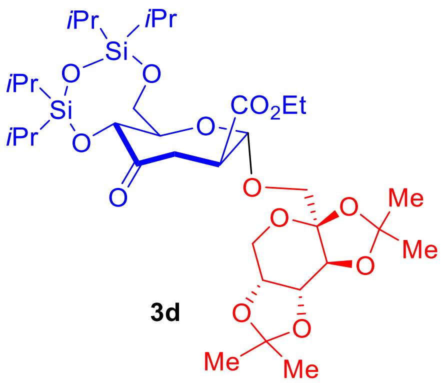

3d

^{1}H NMR (700 MHz, $CDCl_3$) δ 5.23 (d, *J* = 6.7 Hz, 1H), 4.57 (dd, *J* = 7.9, 2.7 Hz, 1H), 4.47 (d, *J* = 9.6 Hz, 1H), 4.33 (d, *J* = 2.6 Hz, 1H), 4.20 (d, *J* = 8.0 Hz, 1H), 4.19-4.16 (m, 2H), 4.11 (d, *J* = 11.8 Hz, 1H), 3.94-3.88 (m, 4H), 3.71 (d, *J* = 12.9 Hz, 1H), 3.56 (d, *J* = 10.3 Hz, 1H), 2.88-2.81 (m, 2H), 2.62 (d, *J* = 13.0 Hz, 1H), 1.51 (s, 3H), 1.45 (s, 3H), 1.34 (s, 3H), 1.32 (s, 3H), 1.26 (t, *J* = 7.2 Hz, 3H), 1.08-1.02 (m, 28H). **^{13}C NMR** (176 MHz, $CDCl_3$) δ 205.76, 171.23, 109.15, 108.86, 102.09, 100.97, 75.07, 71.07, 70.23, 69.93, 69.90, 63.29, 61.71, 61.30, 45.16, 38.80, 26.70, 26.11, 25.49, 24.19, 17.46, 17.41, 17.37,

17.27, 17.25, 17.18, 17.08, 14.14, 13.42, 13.34, 12.55, 12.38. **ESI-HRMS**: Calculated for $C_{34}H_{61}O_{13}Si_2$ $(M+H)^+$: 733.36452, Found: 733.36607. $[\alpha]_D^{20}$ = +5.0 (c = 1.0, CH_2Cl_2).

Ethyl (2*S*,3*R*,6*R*,7*R*)-6-(benzyloxy)-7-(((tert-butyldimethylsilyl)oxy)methyl)-5-oxo-2-(((3a*S*,5a*R*,8a*R*,8b*S*)-2,2,7,7-tetramethyltetrahydro-3aH-bis([1,3]dioxolo)[4,5-b:4',5'-d]pyran-3a-yl)methoxy)oxepane-3-carboxylate (3e)

The title product compound is prepared according to the general procedure **B** with 2 mol% catalyst **A** and 0.2 mmol cyclopropanated donor at rt for 4 h and isolated by flash column chromatography (10:1 Pentane: Ethyl Acetate) giving **3e** as an oil (121 mg, 87% yield, α/β ratio > 20:1).

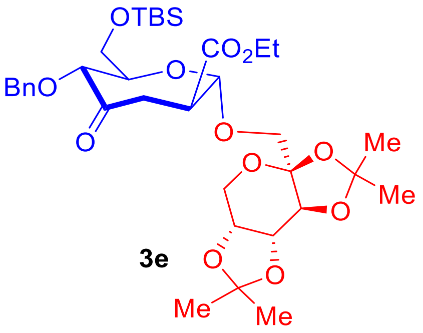

3e

^{1}H NMR (500 MHz, $CDCl_3$) δ 7.35-7.29 (m, 5H), 5.05 (d, *J* = 8.6 Hz, 1H), 4.59-4.56 (m, 2H), 4.51 (d, *J* = 11.6 Hz, 1H), 4.34 (d, *J* = 2.6 Hz, 1H), 4.21-4.14 (m, 4H), 4.01 (d, *J* = 5.0 Hz, 1H), 3.91-3.86 (m, 2H), 3.75-3.64 (m, 3H), 3.58 (dd, *J* = 10.5, 5.6 Hz, 1H), 3.49 (d, *J* = 10.5 Hz, 1H), 2.67-2.59 (m, 2H), 1.51 (s, 3H), 1.44 (s, 3H), 1.37 (s, 3H), 1.32 (s, 3H), 1.27 (t, *J* = 7.1 Hz, 3H), 0.88 (s, 9H), 0.05 (s, 6H). **^{13}C NMR** (126 MHz, $CDCl_3$) δ 205.79, 171.35, 137.38, 128.55, 128.09, 128.03, 109.05, 108.71, 102.19, 101.66, 85.59, 73.24, 71.83, 71.05, 70.21, 69.78, 69.34, 63.95, 61.43, 61.18, 44.80, 39.70, 26.74, 26.02, 25.43, 24.14, 18.46, 14.17, -5.24, -5.26. **ESI-HRMS**: Calculated for $C_{35}H_{54}O_{12}NaSi$ $(M+Na)^+$: 717.3277, Found: 717.3276. $[\alpha]_D^{20}$ = +19.05 (c = 2.0, CH_2Cl_2).

Ethyl (6a*R*,8*S*,9*R*,11a*R*)-2,2,4,4-tetraisopropyl-8-(((3a*R*,4*R*,6*R*,6a*R*)-6-methoxy-2,2-dimethyltetrahydrofuro[3,4-d][1,3]dioxol-4-yl)methoxy)-11-oxohexahydro-6H-oxepino[3,2-f][1,3,5,2,4]trioxadisilocine-9-carboxylate (3f)

The title product compound is prepared according to the general procedure **A** with 2 mol% catalyst **A** and 0.2 mmol cyclopropanated donor at 35 ˚C for 12 h and isolated by flash column chromatography (10:1 Pentane: Ethyl Acetate) giving **3f** as an oil (107 mg, 79% yield, α/β ratio > 20:1).

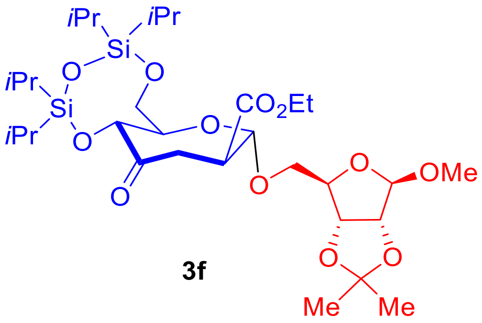

3f

^{1}H NMR (700 MHz, $CDCl_3$) δ 5.23 (d, *J* = 6.9 Hz, 1H), 4.92 (s, 1H), 4.60 (d, *J* = 6.0 Hz, 1H), 4.55 (d, *J* = 6.0 Hz, 1H), 4.45 (d, *J* = 9.6 Hz, 1H), 4.24 (dd, *J* = 9.1, 5.4 Hz, 1H), 4.17-4.14 (m, 3H), 3.89 (dd, *J* = 11.7, 2.4 Hz, 1H), 3.85 (dt, *J* = 9.6, 2.2 Hz, 1H), 3.81 (dd, *J* = 9.9, 5.4 Hz, 1H), 3.50 (t, *J* = 9.5 Hz, 1H), 3.28 (s, 3H), 2.87-2.84 (m, 1H), 2.81-2.77 (m, 1H), 2.63 (dd, *J* = 13.9, 1.9 Hz, 1H), 1.45 (s, 3H), 1.29 (s, 3H), 1.25 (t, *J* = 7.1 Hz, 3H), 1.08-1.02 (m, 28H). **^{13}C NMR** (176 MHz, $CDCl_3$) δ 205.50, 171.32,

112.50, 109.54, 101.24, 85.24, 84.82, 82.22, 70.06, 69.95, 63.48, 61.62, 55.18, 44.99, 38.75, 26.54, 25.06, 17.47, 17.40, 17.38, 17.34, 17.26, 17.20, 17.19, 17.07, 14.17, 13.41, 13.35, 12.48, 12.42. **ESI-HRMS**: Calculated for $C_{31}H_{57}O_{12}Si_2$ $(M+H)^+$: 677.33831, Found: 677.33977. $[\alpha]_D^{20}$ = -5.9 (c = 1.0, CH_2Cl_2).

Ethyl (2*S*,3*R*,6*R*,7*R*)-6-(benzyloxy)-7-(((tert-butyldimethylsilyl)oxy)methyl)-2-(((3a*R*,4*R*,6*R*,6a*R*)-6-methoxy-2,2-dimethyltetrahydrofuro[3,4-d][1,3]dioxol-4-yl)methoxy)-5-oxooxepane-3-carboxylate (3g)

The title product compound is prepared according to the general procedure **B** with 2 mol% catalyst **A** and 0.2 mmol cyclopropanated donor at rt for 4 h and isolated by flash column chromatography (10:1 Pentane: Ethyl Acetate) giving **3g** as an oil (107 mg, 84% yield, α/β ratio > 20:1).

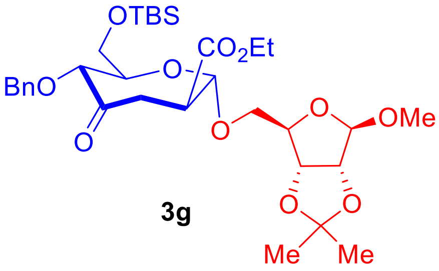

3g

^{1}H NMR (500 MHz, $CDCl_3$) δ 7.36-7.29 (m, 5H), 5.00 (d, *J* = 8.7 Hz, 1H), 4.92 (s, 1H), 4.61 (d, *J* = 6.0 Hz, 1H), 4.58-4.55 (m, 2H), 4.48 (d, *J* = 11.6 Hz, 1H), 4.27 (dd, *J* = 9.9, 5.2 Hz, 1H), 4.21-4.11 (m, 3H), 3.86-3.83 (m, 2H), 3.72-3.65 (m, 2H), 3.56 (dd, *J* = 10.7, 5.0 Hz, 1H), 3.37 (t, *J* = 9.8 Hz, 1H), 3.26 (s, 3H), 2.63-2.55 (m, 2H), 1.46 (s, 3H), 1.30-1.27 (m, 6H), 0.89 (s, 9H), 0.06 (s, 3H). **^{13}C NMR** (126 MHz, $CDCl_3$) δ 205.65, 171.46, 137.14, 128.62, 128.21, 128.14, 112.36, 109.37, 102.08, 85.69, 85.22, 84.70, 82.31, 73.18, 72.47, 69.42, 64.30, 61.35, 54.95, 44.59, 39.68, 26.51, 26.04, 25.02, 18.54, 14.22, -5.24, -5.28. **ESI-HRMS**: Calculated for $C_{32}H_{50}O_{11}NaSi$ $(M+Na)^+$: 661.3015, Found: 661.3014. $[\alpha]_D^{20}$ = +8.7 (c = 2.0, CH_2Cl_2).

Ethyl (6a*R*,8*S*,9*R*,11a*R*)-2,2,4,4-tetraisopropyl-11-oxo-8-(((2*R*,3*R*,4*S*,5*R*,6*S*)-3,4,5-tris(benzyloxy)-6-methoxytetrahydro-2H-pyran-2-yl)methoxy)hexahydro-6H-oxepino[3,2-f][1,3,5,2,4]trioxadisilocine-9-carboxylate (3h)

The title product compound is prepared according to the general procedure **A** with 2 mol% catalyst **A** and 0.2 mmol cyclopropanated donor at 35 ˚C for 12 h and isolated by flash column chromatography (10:1 Pentane: Ethyl Acetate) giving **3h** as an oil (159 mg, 85% yield, α/β ratio > 20:1).

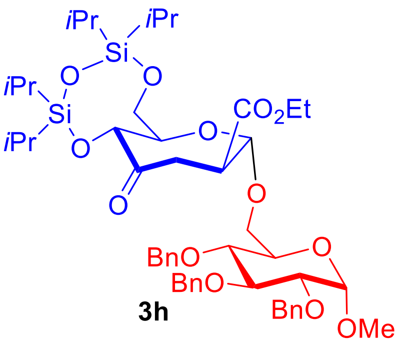

3h

^{1}H NMR (500 MHz, $CDCl_3$) δ 7.39-7.27 (m, 15H), 5.34 (d, *J* = 5.9 Hz, 1H), 5.01 (d, *J* = 10.9 Hz, 1H), 4.92 (d, *J* = 11.2 Hz, 1H), 4.84-4.79 (m, 2H), 4.70 (d, *J* = 12.2 Hz, 1H), 4.59-4.56 (m, 2H), 4.47 (d, *J* = 9.4 Hz, 1H), 4.11-4.07 (m, 2H), 4.05-3.98 (m, 2H), 3.84-3.79 (m, 4H), 3.76-3.73 (m, 1H), 3.49 (dd, *J* =

9.6, 3.6 Hz, 1H), 3.47-3.43 (m, 1H), 3.37 (s, 3H), 2.87-2.82 (m, 2H), 2.62-2.56 (m, 1H), 1.21 (t, *J* = 7.1 Hz, 3H), 1.11-1.04 (m, 28H). **^{13}C NMR** (126 MHz, $CDCl_3$) δ 205.88, 171.35, 138.80, 138.27, 138.20, 128.55, 128.50, 128.48, 128.18, 127.99, 127.67, 100.80, 97.91, 82.09, 79.86, 77.62, 75.78, 74.98, 74.92, 73.33, 70.16, 69.52, 66.93, 63.27, 61.47, 55.21, 45.26, 38.64, 17.48, 17.42, 17.38, 17.33, 17.28, 17.21, 17.19, 17.06, 14.15, 13.34, 13.30, 12.45, 12.35. **ESI-HRMS**: Calculated for $C_{50}H_{73}O_{13}Si_2$ $(M+H)^+$: 937.45842, Found: 937.46090. $[\alpha]_D^{20}$ = +47.65 (c = 2.0, CH_2Cl_2).

Ethyl (6a*R*,8*S*,9*R*,11a*R*)-2,2,4,4-tetraisopropyl-11-oxo-8-(((2*R*,3*R*,4*S*,5*R*,6*S*)-3,4,5,6-tetramethoxytetrahydro-2H-pyran-2-yl)methoxy)hexahydro-6H-oxepino[3,2-f][1,3,5,2,4]trioxa disilocine-9-carboxylate (3i)

The title product compound is prepared according to the general procedure **A** with 2 mol% catalyst **A** and 0.2 mmol cyclopropanated donor at 35 ˚C for 12 h and isolated by flash column chromatography (7:1 Pentane: Ethyl Acetate) giving **3i** as an oil (121 mg, 85% yield, α/β ratio > 20:1).

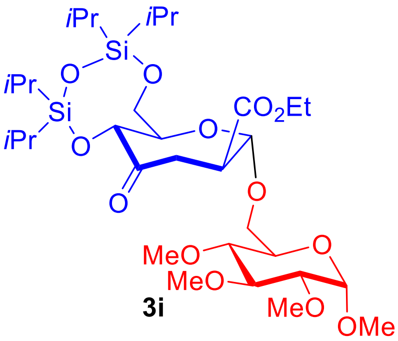

3i

^{1}H NMR (500 MHz, $CDCl_3$) δ 5.34 (d, *J* = 6.3 Hz, 1H), 4.70 (d, *J* = 3.5 Hz, 1H), 4.45 (d, *J* = 9.5 Hz, 1H), 4.17-4.11 (m, 3H), 3.91-3.85 (m, 2H), 3.80-3.79 (m, 2H), 3.57 (s, 3H), 3.56-3.55 (m, 1H), 3.48 (s, 3H), 3.47 (s, 3H), 3.44 (d, *J* = 9.2 Hz, 1H), 3.33 (s, 3H), 3.11 (dd, *J* = 9.6, 3.6 Hz, 1H), 2.99 (dd, *J* = 10.1, 8.8 Hz, 1H), 2.86-2.78 (m, 2H), 2.58 (d, *J* = 12.3 Hz, 1H), 1.24 (t, *J* = 7.1 Hz, 3H), 1.07-1.01 (m, 28H). **^{13}C NMR** (126 MHz, $CDCl_3$) δ 205.87, 171.33, 100.59, 97.23, 83.62, 81.86, 79.61, 75.02, 70.06, 69.58, 67.06, 63.30, 61.51, 60.91, 60.54, 59.03, 55.12, 45.29, 38.55, 17.45, 17.38, 17.34, 17.31, 17.22, 17.17, 17.14, 17.03, 14.15, 13.33, 13.32, 12.43, 12.31. **ESI-HRMS**: Calculated for $C_{32}H_{61}O_{13}Si_2$ $(M+H)^+$: 709.36452, Found: 709.36594. $[\alpha]_D^{20}$ = +60.1 (c = 2.0, CH_2Cl_2).

(2*R*,3*R*,4*S*,5*R*,6*S*)-2-((((6a*R*,8*S*,9*R*,11a*R*)-9-(ethoxycarbonyl)-2,2,4,4-tetraisopropyl-11-oxohexahydro-6H-oxepino[3,2-f][1,3,5,2,4]trioxadisilocin-8-yl)oxy)methyl)-6-methoxytetra hydro-2H-pyran-3,4,5-triyl tribenzoate (3j)

The title product compound is prepared according to the general procedure **A** with 2 mol% catalyst **A** and 0.2 mmol cyclopropanated donor at 35 ˚C for 18 h and isolated by flash column chromatography (7:1 Pentane: Ethyl Acetate) giving **3j** as an oil (163 mg, 85% yield, α/β ratio > 20:1).

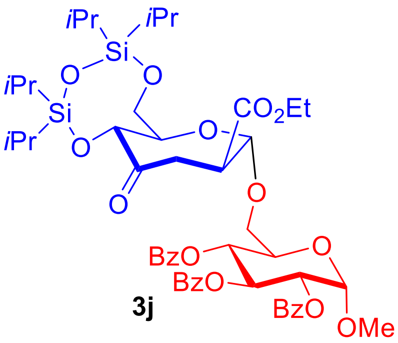

3j

^{1}H NMR (700 MHz, $CDCl_3$) δ 7.97 (dd, *J* = 8.4, 1.4 Hz, 2H), 7.92 (dd, *J* = 8.3, 1.4 Hz, 2H), 7.86-7.84 (m, 2H), 7.50 (td, *J* = 7.4, 3.2 Hz, 2H), 7.42-7.40 (m, 1H), 7.36 (q, *J* = 7.8 Hz, 4H), 7.29-7.26 (m, 2H), 6.11 (t, *J* = 9.9 Hz, 1H), 5.63 (t, *J* = 9.9 Hz, 1H), 5.32 (d, *J* = 6.7 Hz, 1H), 5.23 (dd, *J* = 10.2, 3.6 Hz, 1H), 5.17 (d, *J* = 3.6 Hz, 1H), 4.40 (d, *J* = 9.8 Hz, 1H), 4.23-4.18 (m, 3H), 3.97 (dd, *J* = 11.2, 4.9 Hz, 1H), 3.91-3.86 (m, 2H), 3.74 (dd, *J* = 11.3, 2.2 Hz, 1H), 3.62 (dd, *J* = 11.8, 2.1 Hz, 1H), 3.44 (s, 3H), 2.91-2.88 (m, 1H), 2.81 (dd, *J* = 14.0, 10.5 Hz, 1H), 2.64 (dd, *J* = 14.1, 1.8 Hz, 1H), 1.29 (t, *J* = 7.1 Hz, 3H), 1.08-1.01 (m, 24H), 0.95-0.93 (m, 4H). **^{13}C NMR** (176 MHz, $CDCl_3$) δ 205.62, 171.35, 165.98, 165.90, 165.13, 133.56, 133.47, 133.18, 130.04, 129.91, 129.77, 129.36, 129.18, 129.00, 128.58, 128.52, 128.38, 100.77, 97.07, 74.87, 72.22, 70.73, 69.67, 69.07, 68.57, 66.83, 63.38, 61.61, 55.70, 45.13, 38.59, 17.45, 17.39, 17.38, 17.32, 17.31, 17.21, 17.19, 17.06, 14.20, 13.36, 13.23, 12.41, 12.39. **ESI-HRMS**: Calculated for $C_{50}H_{67}O_{16}Si_2$ $(M+H)^+$: 979.39622, Found: 979.39940. $[\alpha]_D^{20}$ = +48.4 (c = 2.0, CH_2Cl_2).

Ethyl (6a*R*,8*R*,9*R*,11a*R*)-8-(((4a*S*,6*R*,7*S*,8*R*,8a*S*)-8-(benzyloxy)-6-methoxy-2 phenylhexahydro pyrano[3,2-d][1,3]dioxin-7-yl)oxy)-2,2,4,4-tetraisopropyl-11-oxohexahydro-6H-oxepino[3,2-f][1,3,5,2,4]trioxadisilocine-9-carboxylate (3k)

The title product compound is prepared according to the general procedure **A** with 2 mol% catalyst **A** and 0.2 mmol cyclopropanated donor at 35 ˚C for 12 h and isolated by flash column chromatography (10:1 Pentane: Ethyl Acetate) giving **3k** as an oil (139 mg, 82% yield, α/β ratio > 20:1).

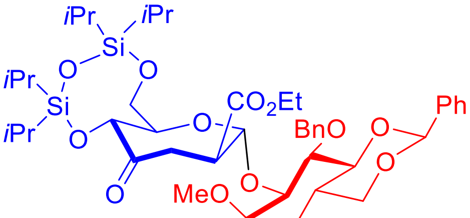

3k

^{1}H NMR (700 MHz, $CDCl_3$) δ 7.50 (dd, *J* = 7.8, 1.9 Hz, 2H), 7.39-7.36 (m, 3H), 7.29 (t, *J* = 6.0 Hz, 2H), 7.27-7.22 (m, 3H), 5.63 (s, 1H), 5.41 (d, *J* = 7.3 Hz, 1H), 4.92 (d, *J* = 3.5 Hz, 1H), 4.90 (d, *J* = 10.8 Hz, 1H), 4.58 (d, *J* = 10.8 Hz, 1H), 4.40 (d, *J* = 10.1 Hz, 1H), 4.32 (dd, *J* = 10.1, 4.7 Hz, 1H), 4.26-4.23 (m, 1H), 4.21-4.17 (m, 1H), 4.06 (dd, *J* = 9.7, 3.6 Hz, 1H), 3.98 (t, *J* = 9.4 Hz, 1H), 3.89-3.85 (m, 1H), 3.84 -3.77 (m, 3H), 3.70 (t, *J* = 9.3 Hz, 1H), 3.47-3.45 (m, 1H), 3.42 (s, 3H), 2.85-2.78 (m, 2H), 2.57 (d, *J* = 12.3 Hz, 1H), 1.31 (t, *J* = 7.0 Hz, 3H), 1.09-1.02 (m, 23H), 0.97 (d, *J* = 7.5 Hz, 3H), 0.90-0.88 (m, 2H). **^{13}C NMR** (176 MHz, $CDCl_3$) δ 205.51, 171.30, 138.47, 137.48, 129.00, 128.37, 128.31, 128.26, 127.77, 126.09, 101.39, 98.34, 97.63, 82.82, 75.63, 74.94, 74.67, 70.07, 69.24, 63.08, 62.43, 61.56, 55.30, 44.50, 39.54, 17.42, 17.39, 17.38, 17.36, 17.25, 17.21, 17.03, 14.16, 13.37, 13.05, 12.46, 12.36. **ESI-HRMS**: Calculated for $C_{43}H_{64}O_{13}NaSi_2$ $(M+Na)^+$: 867.3778, Found: 867.3777. $[\alpha]_D^{20}$ = +24.35 (c = 2.0, CH_2Cl_2).

Ethyl (6a*R*,8*R*,9*R*,11a*R*)-8-(((2*R*,4a*S*,6*R*,7*S*,8*R*,8a*S*)-7-(benzyloxy)-6-methoxy-2-phenylhexahydropyrano[3,2-d][1,3]dioxin-8-yl)oxy)-2,2,4,4-tetraisopropyl-11-oxohexahydro-6H-oxepino[3,2-f][1,3,5,2,4]trioxadisilocine-9-carboxylate (3l)

The title product compound is prepared according to the general procedure **A** with 2 mol% catalyst **A** and 0.2 mmol cyclopropanated donor at 35 ˚C for 16 h and isolated by flash column chromatography (15:1 Pentane: Ethyl Acetate) giving **3l** as an oil (135 mg, 80% yield, α/β ratio > 20:1).

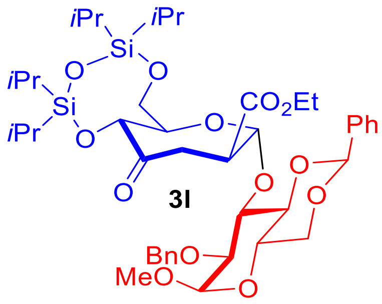

3l

^{1}H NMR (700 MHz, $CDCl_3$) δ 7.46-7.45 (m, 2H), 7.36-7.30 (m, 6H), 7.28-7.27 (m, 1H), 5.76 (d, *J* = 6.3 Hz, 1H), 5.46 (s, 1H), 4.67 (d, *J* = 3.6 Hz, 1H), 4.55 (q, *J* = 11.6 Hz, 2H), 4.46-4.42 (m, 2H), 4.24 (dd, *J* = 10.3, 4.9 Hz, 1H), 4.01-3.96 (m, 2H), 3.94 (dd, *J* = 11.9, 1.8 Hz, 1H), 3.82-3.76 (m, 2H), 3.72-3.67 (m, 2H), 3.54 (t, *J* = 9.4 Hz, 1H), 3.48 (dd, *J* = 9.5, 3.7 Hz, 1H), 3.38 (s, 3H), 2.81 (dd, *J* = 14.0, 9.8 Hz, 1H), 2.76-2.74 (m, 1H), 2.54 (dd, *J* = 14.1, 2.0 Hz, 1H), 1.06-0.99 (m, 28H). **^{13}C NMR** (176 MHz, $CDCl_3$) δ 206.82, 171.06, 137.75, 137.31, 129.12, 128.63, 128.34, 128.28, 128.20, 126.28, 101.59, 99.64, 98.56, 83.04, 78.30, 74.98, 73.42, 73.11, 69.19, 63.34, 61.94, 61.23, 55.37, 46.06, 38.23, 17.55, 17.48, 17.44, 17.41, 17.40, 17.28, 17.11, 14.14, 13.34, 13.24, 12.44, 12.40. **ESI-HRMS**: Calculated for $C_{43}H_{65}O_{13}Si_2$ $(M+H)^+$: 845.39582, Found: 845.39850. $[\alpha]_D^{20}$ = +25.4 (c = 2.0, CH_2Cl_2).

Ethyl (6a*R*,8*R*,9*R*,11a*R*)-8-(((2*R*,3*R*,4*R*,5*S*,6*S*)-2-allyl-4,5-bis(benzyloxy)-6-((benzyloxy)methyl) tetrahydro-2H-pyran-3-yl)oxy)-2,2,4,4-tetraisopropyl-11-oxohexahydro-6H-oxepino[3,2-f][1,3,5,2,4]trioxadisilocine-9-carboxylate (3m)

The title product compound is prepared according to the general procedure **A** with 2 mol% catalyst **A** and 0.2 mmol cyclopropanated donor at 35 ˚C for 12 h and isolated by flash column chromatography (10:1 Pentane: Ethyl Acetate) giving **3m** as an oil (155 mg, 82% yield, α/β ratio > 20:1).

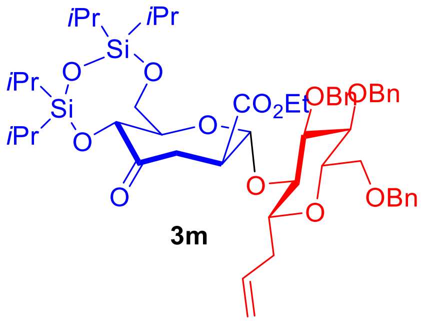

3m

^{1}H NMR (500 MHz, $CDCl_3$) δ 7.39-7.27 (m, 10H), 7.24-7.23 (m, 3H), 7.09-7.07 (m, 2H), 6.00-5.91 (m, 1H), 5.52 (d, *J* = 7.5 Hz, 1H), 5.17 (d, *J* = 17.2 Hz, 1H), 5.11 (d, *J* = 10.7 Hz, 1H), 4.98 (d, *J* = 11.5 Hz, 1H), 4.81 (dd, *J* = 11.0, 5.8 Hz, 2H), 4.66 (d, *J* = 12.4 Hz, 1H), 4.60 (d, *J* = 12.1 Hz, 2H), 4.37 (d, *J* = 10.0 Hz, 1H), 4.18 (q, *J* = 7.1 Hz, 2H), 3.95 (d, *J* = 12.0 Hz, 1H), 3.81 (t, *J* = 9.2 Hz, 1H), 3.77-3.68 (m, 4H), 3.55 (t, *J* = 9.0 Hz, 1H), 3.43-3.38 (m, 2H), 3.23 (td, *J* = 9.0, 2.9 Hz, 1H), 2.83 (dd, *J* = 13.3, 11.1 Hz, 1H), 2.73 (dd, *J* = 10.9, 7.8 Hz, 1H), 2.58 (dd, *J* = 13.8, 7.4 Hz, 1H), 2.51 (d, *J* = 13.3 Hz, 1H), 2.34–2.28 (m, 1H), 1.28 (t, *J* = 7.1 Hz, 3H), 1.06-1.01 (m, 24H), 0.93 (d, *J* = 2.3 Hz, 4H). **^{13}C NMR** (126 MHz, $CDCl_3$) δ 205.71, 171.47, 138.50, 138.34, 137.94, 134.71, 128.50, 128.47, 128.42, 128.09, 127.90, 127.69, 127.47, 127.04, 117.25, 99.76, 85.38, 79.72, 79.37, 79.28, 76.64, 75.65, 75.09, 74.85, 73.53, 70.25, 68.79, 63.43, 61.58, 45.14, 39.23, 36.11, 17.54, 17.48, 17.40, 17.33, 17.31, 17.26, 17.07, 14.18,

13.30, 13.10, 12.53, 12.47. **ESI-HRMS**: Calculated for $C_{52}H_{75}O_{12}Si_2$ $(M+H)^+$: 947.47916, Found: 947.48202. $[\alpha]_D^{20}$ = +9.9 (c = 2.0, CH_2Cl_2).

Ethyl (6a*R*,8*R*,9*R*,11a*R*)-8-(((2*S*,3*R*,4*R*,5*S*,6*S*)-2-allyl-4,5-bis(benzyloxy)-6-((benzyloxy)methyl)tetrahydro-2H-pyran-3-yl)oxy)-2,2,4,4-tetraisopropyl-11-oxohexahydro-6H-oxepino[3,2-f][1,3,5,2,4]trioxadisilocine-9-carboxylate (3n)

The title product compound is prepared according to the general procedure **A** with 2 mol% catalyst **A** and 0.2 mmol cyclopropanated donor at 35 ˚C for 12 h and isolated by flash column chromatography (10:1 Pentane: Ethyl Acetate) giving **3n** as an oil (153 mg, 81% yield, α/β ratio > 20:1).

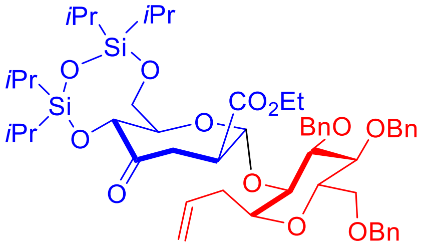

3n

^{1}H NMR (500 MHz, $CDCl_3$) δ 7.42-7.35 (m, 4H), 7.33-7.25 (m, 9H), 7.11-7.09 (m, 2H), 5.87-5.79 (m, 1H), 5.33 (d, *J* = 7.9 Hz, 1H), 5.15-5.10 (m, 2H), 4.90 (d, *J* = 11.5 Hz, 1H), 4.86 (d, *J* = 10.4 Hz, 1H), 4.74 (d, *J* = 11.5 Hz, 1H), 4.70 (d, *J* = 12.2 Hz, 1H), 4.56 (d, *J* = 2.3 Hz, 1H), 4.54 (d, *J* = 4.1 Hz, 1H), 4.38 (d, *J* = 10.2 Hz, 1H), 4.29-4.22 (m, 2H), 4.21-4.16 (m, 2H), 3.90 (d, *J* = 12.1 Hz, 1H), 3.78-3.63 (m, 6H), 3.41 (d, *J* = 12.8 Hz, 1H), 2.86 (t, *J* = 12.4 Hz, 1H), 2.68 (dd, *J* = 11.7, 7.9 Hz, 1H), 2.50 (d, *J* = 12.8 Hz, 1H), 2.47-2.42 (m, 1H), 2.22-2.15 (m, 1H), 1.29 (t, *J* = 7.2 Hz, 3H), 1.08-1.03 (m, 22H), 0.97-0.96 (m, 4H), 0.91-0.86 (m, 2H). **^{13}C NMR** (126 MHz, $CDCl_3$) δ 205.53, 171.40, 138.48, 138.21, 137.98, 134.42, 128.57, 128.51, 128.49, 128.18, 128.13, 127.97, 127.78, 127.58, 127.00, 117.31, 98.09, 81.20, 78.97, 75.70, 75.42, 74.64, 74.59, 73.65, 72.47, 71.23, 70.03, 68.93, 63.05, 61.74, 44.75, 39.69, 28.94, 17.55, 17.52, 17.41, 17.39, 17.30, 17.26, 17.05, 14.21, 13.36, 13.14, 12.53, 12.40. **ESI-HRMS**: Calculated for $C_{52}H_{75}O_{12}Si_2$ $(M+H)^+$: 947.47916, Found: 947.48206. $[\alpha]_D^{20}$ = +37.4 (c = 1.0, CH_2Cl_2).

Ethyl (6a*R*,8*R*,9*R*,11a*R*)-8-(((3a*R*,5*R*,6*S*,6a*R*)-5-((*R*)-2,2-dimethyl-1,3-dioxolan-4-yl)-2,2-dimethyltetrahydrofuro[2,3-d][1,3]dioxol-6-yl)oxy)-2,2,4,4-tetraisopropyl-11-oxohexahydro-6H-oxepino[3,2-f][1,3,5,2,4]trioxadisilocine-9-carboxylate (3o)

The title product compound is prepared according to the general procedure **A** with 2 mol% catalyst **A** and 0.2 mmol cyclopropanated donor at 35 ˚C for 12 h and isolated by flash column chromatography (10:1 Pentane: Ethyl Acetate) giving **3o** as an oil (114 mg, 76% yield, α/β ratio > 20:1).

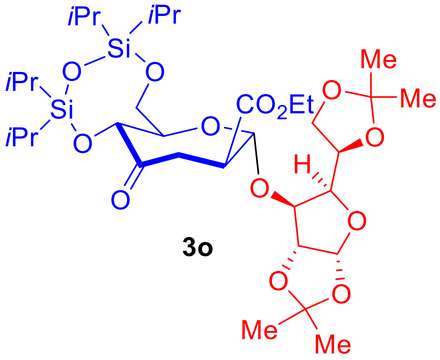

3o

^{1}H NMR (500 MHz, $CDCl_3$) δ 5.95 (d, *J* = 3.7 Hz, 1H), 5.29 (d, *J* = 6.2 Hz, 1H), 4.53 (d, *J* = 3.8 Hz, 1H), 4.43 (d, *J* = 9.6 Hz, 1H), 4.22 (dd, *J* = 7.3, 3.8 Hz, 1H), 4.15-4.06 (m, 4H), 3.92-3.77 (m, 4H), 3.67 (td, *J* = 7.1, 2.9 Hz, 1H), 2.82-2.77 (m, 2H), 2.59-2.54 (m, 1H), 1.45 (s, 3H), 1.29-1.28 (m, 9H), 1.22 (t, *J* =

7.1 Hz, 3H), 1.07-1.00 (m, 28H). **^{13}C NMR** (126 MHz, $CDCl_3$) δ 205.81, 171.23, 112.23, 106.45, 100.93, 100.04, 84.00, 79.26, 75.02, 74.93, 71.29, 69.64, 68.10, 63.29, 61.47, 45.01, 38.88, 27.22, 26.56, 24.00, 23.97, 17.44, 17.39, 17.34, 17.33, 17.21, 17.14, 17.02, 14.15, 13.34, 13.32, 12.51, 12.30 **ESI-HRMS**: Calculated for $C_{34}H_{61}O_{13}Si_2$ $(M+H)^+$: 733.36452, Found: 733.36649. $[\alpha]_D^{20}$ = +32.4 (c = 2.0, CH_2Cl_2).

Ethyl (6a*R*,8*R*,9*R*,11a*R*)-2,2,4,4-tetraisopropyl-8-(((4*R*,6*S*,7*S*,7a*R*)-4-methoxy-2,2,6-trimethyltetrahydro-4H-[1,3]dioxolo[4,5-c]pyran-7-yl)oxy)-11-oxohexahydro-6H-oxepino[3,2-f][1,3,5,2,4]trioxadisilocine-9-carboxylate (3p)

The title product compound is prepared according to the general procedure **A** with 2 mol% catalyst **A** and 0.2 mmol cyclopropanated donor at 35 ˚C for 16 h and isolated by flash column chromatography (10:1 Pentane: Ethyl Acetate) giving **3p** as an oil (113 mg, 82% yield, α/β ratio > 20:1).

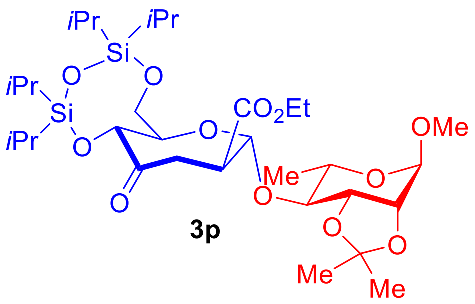

3p

^{1}H NMR (700 MHz, $CDCl_3$) δ 5.39 (d, *J* = 7.0 Hz, 1H), 4.80 (s, 1H), 4.50 (d, *J* = 9.0 Hz, 1H), 4.20 (dd, *J* = 11.5, 3.1 Hz, 1H), 4.18-4.13 (m, 2H), 4.11-4.09 (m, 1H), 4.08 (d, *J* = 4.7 Hz, 2H), 4.00 (dt, *J* = 8.9, 2.7 Hz, 1H), 3.57-3.54 (m, 1H), 3.50-3.48 (m, 1H), 3.34 (s, 3H), 2.98-2.95 (m, 1H), 2.79 (dd, *J* = 14.8, 10.1 Hz, 1H), 2.59 (dd, *J* = 14.8, 2.6 Hz, 1H), 1.54 (s, 3H), 1.31 (s, 3H), 1.25 (t, *J* = 7.2 Hz, 3H), 1.22 (d, *J* = 6.2 Hz, 3H), 1.08-1.03 (m, 28H). **^{13}C NMR** (176 MHz, $CDCl_3$) δ 205.75, 171.42, 109.28, 101.04, 98.15, 81.41, 75.90, 75.69, 70.95, 64.71, 62.96, 61.57, 54.99, 45.49, 38.72, 28.03, 26.23, 17.86, 17.53, 17.51, 17.40, 17.39, 17.35, 17.30, 17.22, 17.15, 14.18, 13.38, 13.37, 12.77, 12.50. **ESI-HRMS**: Calculated for $C_{32}H_{59}O_{12}Si_2$ $(M+H)^+$: 691.35396, Found: 691.35526. $[\alpha]_D^{20}$ = +14.6 (c = 2.0, CH_2Cl_2).

Ethyl (2*R*,3*R*,6*R*,7*R*)-6-(benzyloxy)-7-(((tert-butyldimethylsilyl)oxy)methyl)-2-(((4*R*,6*S*,7*S*,7a*R*)-4-methoxy-2,2,6-trimethyltetrahydro-4H-[1,3]dioxolo[4,5-c]pyran-7-yl)oxy)-5-oxooxepane-3-carboxylate (3q)

The title product compound is prepared according to the general procedure **B** with 2 mol% catalyst **A** and 0.2 mmol cyclopropanated donor at rt for 16 h and isolated by flash column chromatography (10:1 Pentane: Ethyl Acetate) giving **3q** as an oil (105 mg, 80% yield, α/β ratio > 20:1).

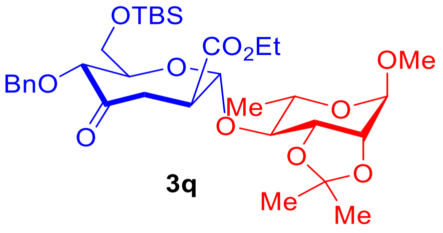

3q

^{1}H NMR (500 MHz, $CDCl_3$) δ 7.36-7.28 (m, 5H), 5.20 (d, *J* = 9.0 Hz, 1H), 4.80 (s, 1H), 4.56-4.50 (m, 2H), 4.23-4.11 (m, 4H), 4.06 (d, *J* = 5.9 Hz, 1H), 4.03 (d, *J* = 3.7 Hz, 1H), 3.91-3.82 (m, 2H), 3.56 (dt, *J* = 10.1, 7.2 Hz, 2H), 3.37-3.34 (m, 4H), 2.61-2.51 (m, 2H), 1.51 (s, 3H), 1.32 (s, 3H), 1.28 (t, *J* = 7.1 Hz, 3H), 1.21 (d, *J* = 6.2 Hz, 3H), 0.89 (s, 9H), 0.06 (s, 6H). **^{13}C NMR** (126 MHz, $CDCl_3$) δ 205.82, 171.59, 137.34, 128.59, 128.13, 128.12, 109.12, 101.89, 98.07, 86.50, 82.13, 77.28, 75.61, 73.10, 72.81, 64.37,

63.73, 61.27, 54.93, 45.38, 39.85, 28.12, 26.38, 26.02, 18.45, 17.85, 14.27, -5.20, -5.24. **ESI-HRMS**: Calculated for $C_{33}H_{52}O_{11}NaSi$ $(M+Na)^+$: 675.3171, Found: 675.3170. $[\alpha]_D^{20}$ = +22.8 (c = 1.0, CH_2Cl_2).

Ethyl (6a*R*,8*S*,9*R*,11a*R*)-2,2,4,4-tetraisopropyl-8-methoxy-11-oxohexahydro-6H-oxepino[3,2-f][1,3,5,2,4]trioxadisilocine-9-carboxylate (3r)

The title product compound is prepared according to the general procedure **A** with 2 mol% catalyst **A** and 0.2 mmol cyclopropanated donor at 35 ˚C for 7 h and isolated by flash column chromatography (15:1 Pentane: Ethyl Acetate) giving **3r** as an oil (71 mg, 70% yield, α/β ratio > 20:1).

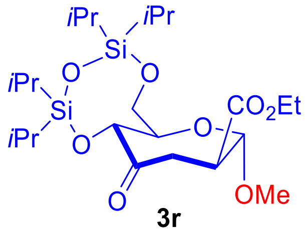

3r

¹H NMR (700 MHz, $CDCl_3$) δ 5.14 (d, *J* = 5.5 Hz, 1H), 4.47 (d, *J* = 9.6 Hz, 1H), 4.19-4.14 (m, 3H), 3.90 (dd, *J* = 11.7, 2.5 Hz, 1H), 3.83 (dt, *J* = 9.6, 2.2 Hz, 1H), 3.45 (s, 3H), 2.84-2.80 (m, 2H), 2.63-2.59 (m, 1H), 1.26 (t, *J* = 7.1 Hz, 3H), 1.09-1.03 (m, 28H). **¹³C NMR** (176 MHz, $CDCl_3$) δ 205.78, 171.43, 101.84, 75.13, 69.76, 63.51, 61.59, 56.42, 45.19, 38.84, 17.50, 17.45, 17.40, 17.38, 17.29, 17.25, 17.21, 17.10, 14.20, 13.42, 13.40, 12.56, 12.43. **ESI-HRMS**: Calculated for $C_{23}H_{44}O_8NaSi_2$ $(M+Na)^+$: 527.2467, Found: 527.2463. $[\alpha]_D^{20}$ = +15.0 (c = 1.0, CH_2Cl_2).

Ethyl (2*S*,3*R*,6*R*,7*R*)-6-(benzyloxy)-7-(((tert-butyldimethylsilyl)oxy)methyl)-2-methoxy-5-oxooxepane-3-carboxylate (3s)

The title product compound is prepared according to the general procedure **B** with 2 mol% catalyst **A** and 0.2 mmol cyclopropanated donor at rt for 3 h and isolated by flash column chromatography (15:1 Pentane: Ethyl Acetate) giving **3s** as an oil (73 mg, 78% yield, α/β ratio > 20:1).

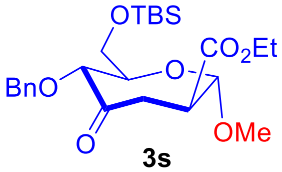

3s

¹H NMR (500 MHz, $CDCl_3$) δ 7.36-7.29 (m, 5H), 4.91 (d, *J* = 8.6 Hz, 1H), 4.59 (d, *J* = 11.6 Hz, 1H), 4.48 (d, *J* = 11.6 Hz, 1H), 4.18 (q, *J* = 7.1 Hz, 2H), 4.10 (q, *J* = 5.0 Hz, 1H), 3.86 (d, *J* = 5.0 Hz, 1H), 3.68 (dd, *J* = 10.7, 6.3 Hz, 1H), 3.64-3.57 (m, 2H), 3.41 (s, 3H), 2.66-2.56 (m, 2H), 1.28 (t, *J* = 7.2 Hz, 3H), 0.89 (s, 9H), 0.05 (d, *J* = 2.3 Hz, 6H). **¹³C NMR** (126 MHz, $CDCl_3$) δ 205.83, 171.50, 137.22, 128.60, 128.18, 128.12, 102.50, 85.63, 73.16, 72.06, 64.35, 61.33, 56.07, 44.87, 39.76, 26.01, 18.49, 14.24, -5.27. **ESI-HRMS**: Calculated for $C_{24}H_{38}O_7NaSi$ $(M+Na)^+$: 489.2279, Found: 489.2277. $[\alpha]_D^{20}$ = +36.8 (c = 1.0, CH_2Cl_2).

Ethyl (6a*R*,8*S*,9*S*,11a*R*)-8-ethoxy-2,2,4,4-tetraisopropyl-11-oxohexahydro-6H-oxepino[3,2-f][1,3,5,2,4]trioxadisilocine-9-carboxylate (3t)

The title product compound is prepared according to the general procedure **B** with 2 mol% catalyst **A** and 0.2 mmol cyclopropanated donor at rt for 7 h and isolated by flash column chromatography (15:1 Pentane: Ethyl Acetate) giving **3t** as an oil (81 mg, 79% yield, α/β ratio > 20:1).

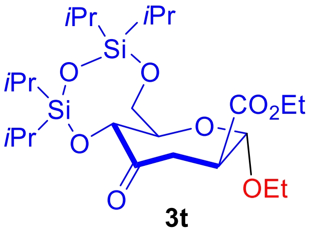

3t

^{1}H NMR (500 MHz, CD_2Cl_2) δ 5.19 (d, *J* = 6.8 Hz, 1H), 4.43 (d, *J* = 9.7 Hz, 1H), 4.17-4.10 (m, 3H), 3.85-3.78 (m, 2H), 3.59 (dq, *J* = 9.8, 7.0 Hz, 1H), 2.83-2.75 (m, 2H), 2.57-2.54 (m, 1H), 1.25 (t, *J* = 7.1 Hz, 3H), 1.18 (t, *J* = 7.1 Hz, 3H), 1.09-1.02 (m, 28H). **^{13}C NMR** (126 MHz, CD_2Cl_2) δ 205.94, 171.65, 100.46, 75.44, 69.90, 64.33, 63.67, 61.76, 45.57, 39.29, 17.50, 17.48, 17.43, 17.39, 17.32, 17.28, 17.25, 17.15, 15.04, 14.27, 13.64, 13.62, 12.99, 12.69. **ESI-HRMS**: Calculated for $C_{24}H_{47}O_8Si_2$ $(M+H)^+$: 519.28040, Found: 519.28100. $[\alpha]_D^{20}$ = +15.7 (c = 1.0, CH_2Cl_2).

Ethyl (6a*R*,8*S*,9*R*,11a*R*)-2,2,4,4-tetraisopropyl-8-(octyloxy)-11-oxohexahydro-6H-oxepino[3,2-f][1,3,5,2,4]trioxadisilocine-9-carboxylate (3u)

The title product compound is prepared according to the general procedure **A** with 2 mol% catalyst **A** and 0.2 mmol cyclopropanated donor at 35 ˚C for 12 h and isolated by flash column chromatography (15:1 Pentane: Ethyl Acetate) giving **3u** as an oil (99 mg, 82% yield, α/β ratio > 20:1).

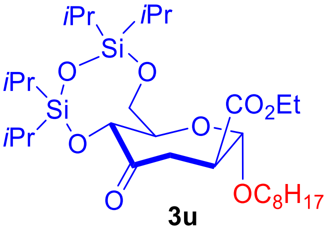

3u

^{1}H NMR (500 MHz, $CDCl_3$) δ 5.22 (d, *J* = 6.5 Hz, 1H), 4.46 (d, *J* = 9.6 Hz, 1H), 4.17-4.13 (m, 3H), 3.87-3.84 (m, 2H), 3.74-3.70 (m, 1H), 3.50 (dt, *J* = 9.7, 6.4 Hz, 1H), 2.84-2.78 (m, 2H), 2.64-2.58 (m, 1H), 1.54-1.50 (m, 3H), 1.28-1.23 (m, 13H), 1.09-1.05 (m, 28H), 0.86 (t, *J* = 6.9 Hz, 3H). **^{13}C NMR** (126 MHz, $CDCl_3$) δ 206.03, 171.54, 100.31, 75.14, 69.62, 68.88, 63.48, 61.49, 45.34, 38.73, 31.94, 29.47, 29.45, 29.35, 26.15, 22.77, 17.49, 17.42, 17.39, 17.35, 17.28, 17.22, 17.20, 17.08, 14.21, 13.38, 12.48, 12.39. **ESI-HRMS**: Calculated for $C_{30}H_{59}O_8Si_2$ $(M+H)^+$: 603.37430, Found: 603.37557. $[\alpha]_D^{20}$ = +20.7 (c = 1.0, CH_2Cl_2).

Ethyl (2*S*,3*R*,6*R*,7*R*)-6-(benzyloxy)-7-(((tert-butyldimethylsilyl)oxy)methyl)-2-(octyloxy)-5-oxooxepane-3-carboxylate (3v)

The title product compound is prepared according to the general procedure **B** with 2 mol% catalyst **A** and 0.2 mmol cyclopropanated donor at rt for 4 h and isolated by flash column chromatography (15:1 Pentane: Ethyl Acetate) giving **3v** as an oil (90 mg, 80% yield, α/β ratio > 20:1).

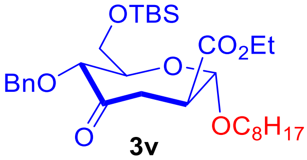

3v

^{1}H NMR (500 MHz, $CDCl_3$) δ 7.36-7.29 (m, 5H), 4.99 (d, *J* = 8.6 Hz, 1H), 4.58 (d, *J* = 11.6 Hz, 1H), 4.49 (d, *J* = 11.5 Hz, 1H), 4.17 (qd, *J* = 7.1, 1.9 Hz, 2H), 4.12 (q, *J* = 5.3 Hz, 1H), 3.89 (d, *J* = 4.9 Hz, 1H), 3.76-3.72 (m, 1H), 3.70-3.62 (m, 2H), 3.60-3.55 (m, 1H), 3.44-3.39 (m, 1H), 2.66-2.56 (m, 2H), 1.55-1.50 (m, 2H), 1.30-1.26 (m, 15H), 0.89-0.86 (m, 12H), 0.06-0.05 (m, 6H). **^{13}C NMR** (126 MHz, $CDCl_3$)

δ 206.07, 171.60, 137.29, 128.59, 128.15, 101.23, 85.87, 73.19, 71.93, 68.68, 64.36, 61.22, 44.98, 39.76, 31.95, 29.51, 29.46, 29.39, 26.19, 26.12, 26.03, 22.79, 18.51, 14.28, 14.23, -5.25. **ESI-HRMS**: Calculated for $C_{31}H_{53}O_7Si$ $(M+H)^+$: 565.35551, Found: 565.35659. $[\alpha]_D^{20}$ = +30.8 (c = 1.0, CH_2Cl_2).

Ethyl (6a*R*,8*S*,9*R*,11a*R*)-2,2,4,4-tetraisopropyl-8-(neopentyloxy)-11-oxohexahydro-6H-oxepino[3,2-f][1,3,5,2,4]trioxadisilocine-9-carboxylate (3w)

The title product compound is prepared according to the general procedure **A** with 2 mol% catalyst **A** and 0.2 mmol cyclopropanated donor at 35 ˚C for 12 h and isolated by flash column chromatography (15:1 Pentane: Ethyl Acetate) giving **3w** as an oil (101 mg, 90% yield, α/β ratio > 20:1).

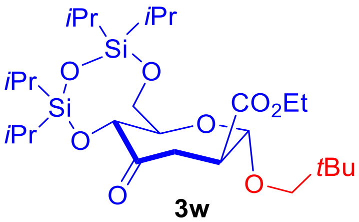

3w

^{1}H NMR (500 MHz, CD_2Cl_2) δ 5.12 (d, *J* = 6.8 Hz, 1H), 4.43 (d, *J* = 9.2 Hz, 1H), 4.17-4.10 (m, 3H), 3.85-3.82 (m, 2H), 3.46 (d, *J* = 8.9 Hz, 1H), 3.13 (d, *J* = 8.9 Hz, 1H), 2.86-2.78 (m, 2H), 2.55 (d, *J* = 12.0 Hz, 1H), 1.25 (t, *J* = 7.1 Hz, 3H), 1.10-1.03 (m, 28H), 0.89 (s, 9H). **^{13}C NMR** (126 MHz, CD_2Cl_2) δ 206.01, 171.80, 101.53, 79.45, 75.49, 70.00, 63.59, 61.72, 45.50, 39.26, 32.13, 26.60, 17.50, 17.49, 17.43, 17.40, 17.33, 17.28, 17.25, 17.16, 14.30, 13.64, 12.99, 12.71. **ESI-HRMS**: Calculated for $C_{27}H_{53}O_8Si_2$ $(M+H)^+$: 561.32735, Found: 561.32865. $[\alpha]_D^{20}$ = +20.3 (c = 2.0, CH_2Cl_2).

Ethyl (6a*R*,8*S*,9*R*,11a*R*)-8-isopropoxy-2,2,4,4-tetraisopropyl-11-oxohexahydro-6H-oxepino[3,2-f][1,3,5,2,4]trioxadisilocine-9-carboxylate (3x)

The title product compound is prepared according to the general procedure **A** with 2 mol% catalyst **A** and 0.2 mmol cyclopropanated donor at 35 ˚C for 24 h and isolated by flash column chromatography (15:1 Pentane: Ethyl Acetate) giving **3x** as an oil (81 mg, 76% yield, α/β ratio > 20:1).

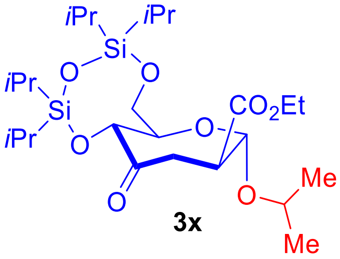

3x

^{1}H NMR (700 MHz, CD_2Cl_2) δ 5.25 (d, *J* = 7.0 Hz, 1H), 4.42 (d, *J* = 9.5 Hz, 1H), 4.17 (q, *J* = 7.0 Hz, 2H), 4.14-4.12 (m, 1H), 4.02 (p, *J* = 6.2 Hz, 1H), 3.88 (dt, *J* = 9.5, 2.4 Hz, 1H), 3.80 (dd, *J* = 11.7, 2.6 Hz, 1H), 2.81-2.74 (m, 2H), 2.55-2.53 (m, 1H), 1.26 (t, *J* = 7.1 Hz, 3H), 1.16 (d, *J* = 6.3 Hz, 3H), 1.12 (d, *J* = 6.0 Hz, 3H), 1.10-0.99 (m, 27H), 0.95-0.91 (m, 1H). **^{13}C NMR** (176 MHz, CD_2Cl_2) δ 205.92, 171.69, 98.56, 75.61, 70.11, 69.51, 63.81, 61.73, 45.80, 39.51, 23.35, 21.20, 17.54, 17.53, 17.45, 17.43, 17.37, 17.32, 17.28, 17.19, 14.33, 13.69, 13.68, 13.06, 12.76. **ESI-HRMS**: Calculated for $C_{25}H_{49}O_8Si_2$ $(M+H)^+$: 533.29605, Found: 533.29677. $[\alpha]_D^{20}$ = +18.0 (c = 1.0, CH_2Cl_2).

Ethyl (2*S*,3*R*,6*R*,7*R*)-6-(benzyloxy)-7-(((tert-butyldimethylsilyl)oxy)methyl)-2-isopropoxy-5-oxooxepane-3-carboxylate (3y)

The title product compound is prepared according to the general procedure **B** with 2 mol% catalyst **A** and 0.2 mmol cyclopropanated donor at rt for 12 h and isolated by flash column chromatography (15:1 Pentane: Ethyl Acetate) giving **3y** as an oil (74 mg, 75% yield, α/β ratio > 20:1).

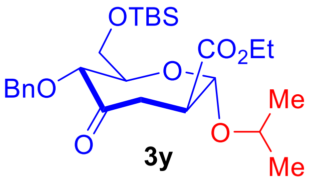

3y

^{1}H NMR (500 MHz, CD_2Cl_2) δ 7.37-7.30 (m, 5H), 5.07 (d, *J* = 8.8 Hz, 1H), 4.57-4.49 (m, 2H), 4.17-4.12 (m, 3H), 3.98-3.94 (m, 1H), 3.92 (d, *J* = 4.6 Hz, 1H), 3.70 (dd, *J* = 10.6, 5.6 Hz, 1H), 3.63-3.59 (m, 1H), 3.56 (dd, *J* = 10.6, 5.8 Hz, 1H), 2.61-2.54 (m, 2H), 1.26 (t, *J* = 7.1 Hz, 3H), 1.18-1.14 (m, 3H), 1.09 (d, *J* = 6.1 Hz, 3H), 0.90 (s, 9H), 0.07 (d, *J* = 2.3 Hz, 6H). **^{13}C NMR** (126 MHz, CD_2Cl_2) δ 206.11, 171.60, 137.87, 128.78, 128.34, 128.32, 99.41, 86.45, 73.36, 72.15, 69.66, 64.65, 61.41, 45.54, 40.13, 26.05, 23.50, 21.16, 18.66, 14.35, -5.31, -5.32. **ESI-HRMS**: Calculated for $C_{26}H_{42}O_7NaSi$ $(M+Na)^+$: 517.2592, Found: 517.2588. $[\alpha]_D^{20}$ = +39.8 (c = 1.0, CH_2Cl_2).

Ethyl (6a*R*,8*S*,9*R*,11a*R*)-8-(cyclohexyloxy)-2,2,4,4-tetraisopropyl-11-oxohexahydro-6H-oxepino[3,2-f][1,3,5,2,4]trioxadisilocine-9-carboxylate (3z)

The title product compound is prepared according to the general procedure **A** with 2 mol% catalyst **A** and 0.2 mmol cyclopropanated donor at 35 ˚C for 24 h and isolated by flash column chromatography (15:1 Pentane: Ethyl Acetate) giving **3z** as an oil (74 mg, 65% yield, α/β ratio > 20:1).

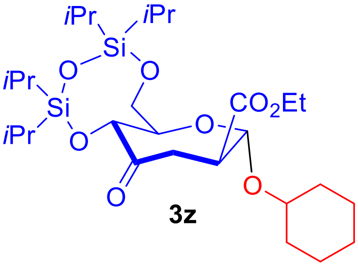

3z

^{1}H NMR (700 MHz, $CDCl_3$) δ 5.34 (d, *J* = 6.7 Hz, 1H), 4.46 (d, *J* = 9.5 Hz, 1H), 4.18-4.13 (m, 3H), 3.91 (dt, *J* = 9.5, 2.4 Hz, 1H), 3.81 (dd, *J* = 11.6, 2.6 Hz, 1H), 3.76-3.72 (m, 1H), 2.83-2.79 (m, 2H), 2.63-2.59 (m, 1H), 1.83-1.66 (m, 4H), 1.50-1.49 (m, 1H), 1.37-1.23 (m, 8H), 1.09-0.93 (m, 28H). **^{13}C NMR** (176 MHz, $CDCl_3$) δ 206.04, 171.63, 97.94, 75.27, 74.64, 69.76, 63.63, 61.45, 45.61, 38.97, 33.32, 31.17, 25.71, 24.09, 23.64, 17.53, 17.46, 17.40, 17.39, 17.31, 17.25, 17.22, 17.12, 14.26, 13.43, 12.53, 12.45. **ESI-HRMS**: Calculated for $C_{28}H_{53}O_8Si_2$ $(M+H)^+$: 573.32735, Found: 573.32826. $[\alpha]_D^{20}$ = +16.0 (c = 1.0, CH_2Cl_2).

Ethyl (6a*R*,8*R*,9*R*,11a*R*)-8-(tert-butoxy)-2,2,4,4-tetraisopropyl-11-oxohexahydro-6H-oxepino[3,2-f][1,3,5,2,4]trioxadisilocine-9-carboxylate (3aa)

The title product compound is prepared according to the general procedure **A** with 2 mol% catalyst **A** and 0.2 mmol cyclopropanated donor at 35 ˚C for 48 h and isolated by flash column chromatography (15:1 Pentane: Ethyl Acetate) giving **3aa** as an oil (66 mg, 60% yield, α/β ratio > 20:1).

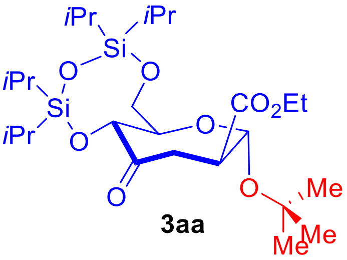

3aa

^{1}H NMR (500 MHz, $CDCl_3$) δ 5.47 (d, *J* = 6.8 Hz, 1H), 4.47 (d, *J* = 8.7 Hz, 1H), 4.19- 4.09 (m, 3H), 3.99-3.96 (m, 1H), 3.81 (dd, *J* = 11.5, 3.5 Hz, 1H), 2.95-2.91 (m, 1H), 2.81-2.75 (m, 1H), 2.63 (dd, *J* = 15.0, 3.0 Hz, 1H), 1.26 (t, *J* = 7.1 Hz, 3H), 1.22 (s, 9H), 1.09-1.03 (m, 28H). **^{13}C NMR** (126 MHz, $CDCl_3$) δ 206.30, 171.71, 94.87, 76.01, 75.72, 70.34, 63.51, 61.32, 46.53, 38.58, 28.74, 17.54, 17.38, 17.33, 17.29, 17.21, 17.16, 14.27, 13.43, 13.37, 12.70, 12.44. **ESI-HRMS**: Calculated for $C_{26}H_{51}O_8Si_2$ $(M+H)^+$: 547.31170, Found: 547.31268. $[\alpha]_D^{20}$ = +23.5 (c = 1.0, CH_2Cl_2).

Ethyl (2*R*,3*R*,6*R*,7*R*)-6-(benzyloxy)-2-(tert-butoxy)-7-(((tert-butyldimethylsilyl)oxy)methyl)-5-oxooxepane-3-carboxylate (3ab)

The title product compound is prepared according to the general procedure **B** with 2 mol% catalyst **A** and 0.2 mmol cyclopropanated donor at rt for 12 h and isolated by flash column chromatography (15:1 Pentane: Ethyl Acetate) giving **3ab** as an oil (66 mg, 65% yield, α/β ratio > 20:1).

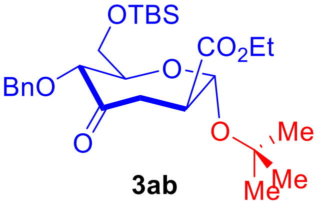

3ab

^{1}H NMR (500 MHz, CD_2Cl_2) δ 7.38-7.31 (m, 5H), 5.23 (d, *J* = 8.8 Hz, 1H), 4.52 (s, 2H), 4.21-4.09 (m, 3H), 3.97 (d, *J* = 4.1 Hz, 1H), 3.73-3.68 (m, 2H), 3.52 (dd, *J* = 10.4, 6.7 Hz, 1H), 2.60-2.53 (m, 2H), 1.27 (t, *J* = 7.1 Hz, 3H), 1.20 (s, 9H), 0.90 (s, 9H), 0.07 (s, 6H). **^{13}C NMR** (126 MHz, CD_2Cl_2) δ 206.29, 171.80, 137.93, 128.78, 128.34, 128.30, 96.46, 86.98, 75.53, 73.32, 72.20, 64.45, 61.33, 46.24, 40.18, 28.83, 26.06, 18.63, 14.38, -5.27. **ESI-HRMS**: Calculated for $C_{27}H_{44}O_7NaSi$ $(M+Na)^+$: 531.2749, Found: 531.2743. $[\alpha]_D^{20}$ = +31.2 (c = 1.0, CH_2Cl_2).

ethyl (6a*R*,8*S*,9*S*,11a*R*)-2,2,4,4-tetraisopropyl-8-(((1*R*,2*S*,5*R*)-2-isopropyl-5-methylcyclohexyl)oxy)-11-oxohexahydro-6H-oxepino[3,2-f][1,3,5,2,4]trioxadisilocine-9-carboxylate (3ac)

The title product compound is prepared according to the general procedure **A** with 2 mol% catalyst **A** and 0.2 mmol cyclopropanated donor at 35 ˚C for 18 h and isolated by flash column chromatography (15:1 Pentane: Ethyl Acetate) giving **3ac** as an oil (78 mg, 62% yield, α/β ratio > 20:1).

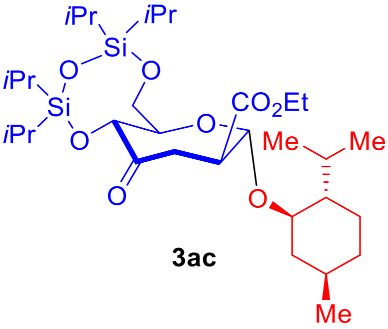

3ac

^{1}H NMR (700 MHz, $CDCl_3$) δ 5.31 (d, *J* = 6.8 Hz, 1H), 4.47 (d, *J* = 8.7 Hz, 1H), 4.15-4.10 (m, 3H), 3.98-3.96 (m, 1H), 3.87 (dd, *J* = 11.5, 3.5 Hz, 1H), 3.41 (td, *J* = 10.6, 4.3 Hz, 1H), 3.02-2.99 (m, 1H), 2.77 (dd, *J* = 15.1, 9.8 Hz, 1H), 2.60 (dd, *J* = 15.1, 2.8 Hz, 1H), 2.21-2.18 (m, 1H), 1.97-1.92 (m, 1H), 1.63-1.57 (m, 2H), 1.39-1.34 (m, 1H), 1.25 (t, *J* = 7.2 Hz, 3H), 1.09-1.02 (m, 28H), 0.98-0.93 (m, 3H), 0.89 (d, *J* = 6.6 Hz, 3H), 0.85 (d, *J* = 7.1 Hz, 3H), 0.70 (d, *J* = 7.0 Hz, 3H). **^{13}C NMR** (176 MHz, $CDCl_3$) δ 205.93, 171.71, 101.98, 81.54, 75.92, 70.72, 63.29, 61.43, 48.72, 45.92, 43.39, 38.55, 34.32, 31.66, 25.38, 23.03, 22.36, 21.24, 17.52, 17.49, 17.38, 17.29, 17.21, 17.15, 16.00, 14.15, 13.47, 13.40, 12.62, 12.48. **ESI-HRMS**: Calculated for $C_{32}H_{61}O_8Si_2$ $(M+H)^+$: 629.38995, Found: 629.39139. $[\alpha]_D^{20}$ = -1.1 (c = 1.0, CH_2Cl_2).

Ethyl (2*S*,3*R*,6*R*,7*R*)-6-(benzyloxy)-7-(((tert-butyldimethylsilyl)oxy)methyl)-2-(((1*R*,2*R*,5*R*)-2-isopropyl-5-methylcyclohexyl)oxy)-5-oxooxepane-3-carboxylate (3ad)

The title product compound is prepared according to the general procedure **B** with 2 mol% catalyst **A** and 0.2 mmol cyclopropanated donor at rt for 5 h and isolated by flash column chromatography (15:1 Pentane: Ethyl Acetate) giving **3ad** as an oil (104 mg, 88% yield, α/β ratio > 20:1).

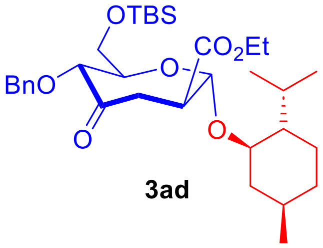

3ad

^{1}H NMR (500 MHz, $CDCl_3$) δ 7.36-7.28 (m, 5H), 5.06 (d, *J* = 8.8 Hz, 1H), 4.57-4.51 (m, 2H), 4.17-4.08 (m, 4H), 3.84-3.79 (m, 1H), 3.75 (dd, *J* = 10.3, 4.3 Hz, 1H), 3.54 (dd, *J* = 10.2, 7.5 Hz, 1H), 3.34-3.29 (m, 1H), 2.60 (dd, *J* = 16.8, 12.6 Hz, 1H), 2.50 (dd, *J* = 16.8, 4.0 Hz, 1H), 2.14-2.11 (m, 1H), 2.01-1.94 (m, 1H), 1.64-1.58 (m, 2H), 1.28 (t, *J* = 7.2 Hz, 3H), 0.99-0.79 (m, 20H), 0.68 (d, *J* = 7.0 Hz, 3H), 0.06 (s, 6H). **^{13}C NMR** (126 MHz, $CDCl_3$) δ 206.17, 171.85, 137.45, 128.55, 128.06, 103.12, 86.57, 80.88, 73.11, 72.10, 63.75, 61.21, 48.58, 45.38, 43.53, 40.00, 34.28, 31.63, 26.01, 24.92, 22.80, 22.47, 21.32, 18.46, 15.80, 14.17, -5.19, -5.21. **ESI-HRMS**: Calculated for $C_{33}H_{55}O_7Si$ $(M+H)^+$: 591.37116, Found: 591.37239. $[\alpha]_D^{20}$ = +10.4 (c = 2.0, CH_2Cl_2).

Ethyl (6a*R*,8*S*,9*R*,11a*R*)-8-(((3*S*,8*S*,9*S*,10*R*,13*R*,14*S*,17*R*)-10,13-dimethyl-17-((*R*)-6-methylheptan-2-yl)-2,3,4,7,8,9,10,11,12,13,14,15,16,17-tetradecahydro-1H-cyclopenta[a]phenanthren-3-yl)oxy)-2,2,4,4-tetraisopropyl-11-oxohexahydro-6H-oxepino[3,2-f][1,3,5,2,4]trioxadisilocine-9-carboxylate (3ae)

The title product compound is prepared according to the general procedure **A** with 2 mol% catalyst **A** and 0.2 mmol cyclopropanated donor at 35 ˚C for 24 h and isolated by flash column chromatography (15:1 Pentane: Ethyl Acetate) giving **3ae** as an oil (117 mg, 68% yield, α/β ratio > 20:1).

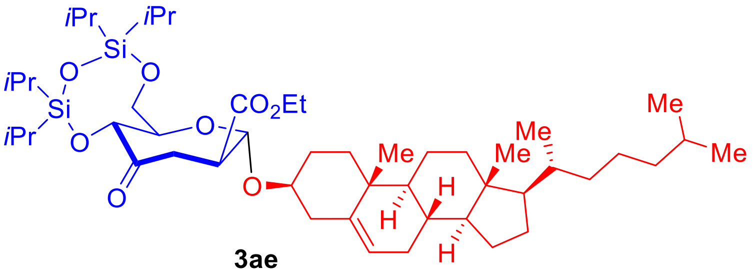

3ae

^{1}H NMR (500 MHz, $CDCl_3$) δ 5.35 (d, *J* = 6.7 Hz, 1H), 5.33-5.32 (m, 1H), 4.46 (d, *J* = 9.4 Hz, 1H), 4.19 -4.12 (m, 3H), 3.91 (dt, *J* = 9.4, 2.4 Hz, 1H), 3.82 (dd, *J* = 11.6, 2.7 Hz, 1H), 3.65-3.59 (m, 1H), 2.84-

2.77 (m, 2H), 2.62 (d, J = 11.9 Hz, 1H), 2.28 (t, J = 12.0 Hz, 1H), 2.19-2.15 (m, 1H), 2.01-1.81 (m, 2H), 1.59-1.32 (m, 11H), 1.25 (t, J = 7.2 Hz, 4H), 1.10-0.97 (m, 43H), 0.90 (d, J = 6.5 Hz, 5H), 0.85 (dd, J = 6.6, 2.3 Hz, 7H). **^{13}C NMR** (126 MHz, $CDCl_3$) δ 205.98, 171.50, 140.64, 122.00, 97.80, 76.38, 75.28, 69.85, 63.59, 61.46, 56.82, 56.23, 50.14, 45.49, 42.42, 39.96, 39.83, 39.63, 38.87, 36.91, 36.85, 36.29, 35.91, 32.03, 31.98, 28.36, 28.13, 27.58, 24.40, 23.94, 22.96, 22.69, 21.17, 19.48, 18.83, 17.53, 17.48, 17.39, 17.37, 17.30, 17.25, 17.21, 17.10, 14.26, 13.41, 13.39, 12.50, 12.40, 11.97. **ESI-HRMS**: Calculated for $C_{49}H_{87}O_8Si_2$ $(M+H)^+$: 859.59340, Found: 859.59480. $[\alpha]_D^{20}$ = +21.65 (c = 2.0, CH_2Cl_2).

Ethyl (6a*R*,8*R*,9*S*,11a*R*)-2,2,4,4-tetraisopropyl-8-((4-methoxyphenyl)thio)-11-oxohexahydro-6H-oxepino[3,2-f][1,3,5,2,4]trioxadisilocine-9-carboxylate (3af)

The title product compound is prepared according to the general procedure **B** with 2 mol% catalyst **A** and 0.2 mmol cyclopropanated donor at rt for 2 h and isolated by flash column chromatography (20:1 Pentane: Ethyl Acetate) giving **3af** as an oil (88 mg, 72% yield, α/β ratio > 20:1).

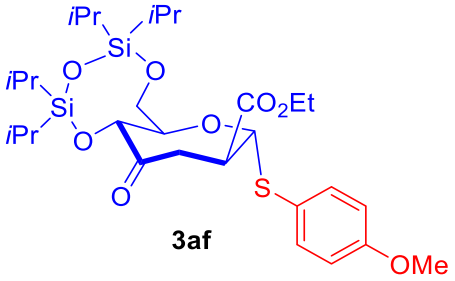

3af

^{1}H NMR (700 MHz, $CDCl_3$) δ 7.41-7.39 (m, 2H), 6.86-6.84 (m, 2H), 5.75 (d, J = 8.6 Hz, 1H), 4.56 (d, J = 9.0 Hz, 1H), 4.23-4.17 (m, 2H), 4.12 (dd, J = 11.6, 2.4 Hz, 1H), 3.80 (s, 3H), 3.77 (dd, J = 11.6, 3.1 Hz, 1H), 3.08-3.05 (m, 1H), 2.93 (dd, J = 14.7, 9.8 Hz, 1H), 2.67 (dd, J = 14.7, 2.8 Hz, 1H), 1.28 (t, J = 7.1 Hz, 3H), 1.10-0.95 (m, 28H). **^{13}C NMR** (176 MHz, $CDCl_3$) δ 205.45, 171.20, 160.02, 134.69, 123.70, 114.93, 89.30, 75.48, 72.60, 63.66, 61.84, 55.50, 45.93, 40.34, 17.51, 17.50, 17.37, 17.35, 17.31, 17.27, 17.22, 17.13, 14.22, 13.41, 13.40, 12.61, 12.46. **ESI-HRMS**: Calculated for $C_{29}H_{49}O_8Si_2S$ $(M+H)^+$: 613.26895, Found: 613.26953. $[\alpha]_D^{20}$ = +118.4 (c = 1.0, CH_2Cl_2).

Ethyl (2*R*,3*S*,6*R*,7*R*)-6-(benzyloxy)-7-(((tert-butyldimethylsilyl)oxy)methyl)-2-((4-methoxyphenyl)thio)-5-oxooxepane-3-carboxylate (3ag)

The title product compound is prepared according to the general procedure **B** with 2 mol% catalyst **A** and 0.2 mmol cyclopropanated donor at rt for 1 h and isolated by flash column chromatography (15:1 Pentane: Ethyl Acetate) giving **3ag** as an oil (96 mg, 83% yield, α/β ratio > 20:1).

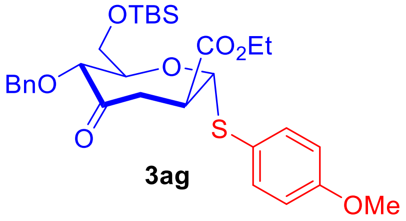

3ag

^{1}H NMR (500 MHz, $CDCl_3$) δ 7.42-7.40 (m, 2H), 7.36-7.30 (m, 5H), 6.86-6.81 (m, 2H), 5.47 (d, J = 11.0 Hz, 1H), 4.57 (q, J = 11.4 Hz, 2H), 4.40-4.37 (m, 1H), 4.23 (qd, J = 7.1, 2.4 Hz, 2H), 4.08 (d, J = 4.5 Hz, 1H), 3.79 (s, 3H), 3.78-3.70 (m, 2H), 3.51 (dd, J = 10.5, 6.1 Hz, 1H), 2.72-2.59 (m, 2H), 1.31 (t, J = 7.2 Hz, 3H), 0.88 (s, 9H), 0.02 (d, J = 6.6 Hz, 6H). **^{13}C NMR** (126 MHz, $CDCl_3$) δ 205.49, 171.17, 159.84, 137.25, 134.63, 128.63, 128.21, 128.20, 124.32, 114.69, 90.18, 85.68, 73.58, 73.31, 63.93, 61.60, 55.46, 45.52, 41.70, 26.01, 18.48, 14.26, -5.27. **ESI-HRMS**: Calculated for $C_{30}H_{42}O_7NaSi$ $(M+Na)^+$: 597.2313, Found: 597.2305. $[\alpha]_D^{20}$ = +146.4 (c = 2.0, CH_2Cl_2).

Ethyl (6a*R*,8*R*,9*S*,11a*R*)-8-(butylthio)-2,2,4,4-tetraisopropyl-11-oxohexahydro-6H-oxepino[3,2-f][1,3,5,2,4]trioxadisilocine-9-carboxylate (3ah)

The title product compound is prepared according to the general procedure **B** with 2 mol% catalyst **A** and 0.2 mmol cyclopropanated donor at rt for 3 h and isolated by flash column chromatography (20:1 Pentane: Ethyl Acetate) giving **3ah** as an oil (69 mg, 61% yield, α/β ratio > 20:1).

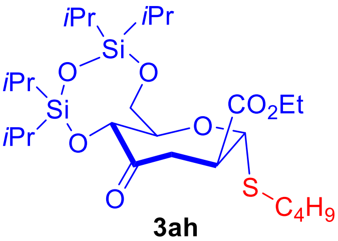

3ah

^{1}H NMR (700 MHz, $CDCl_3$) δ 5.58 (d, *J* = 8.5 Hz, 1H), 4.55 (d, *J* = 9.3 Hz, 1H), 4.20-4.06 (m, 3H), 4.07 (dt, *J* = 9.3, 2.5 Hz, 1H), 3.80 (dd, *J* = 11.7, 2.8 Hz, 1H), 2.93 (dd, *J* = 14.3, 9.9 Hz, 1H), 2.86-2.83 (m, 1H), 2.75-2.71 (m, 1H), 2.60-2.55 (m, 2H), 1.63-1.56 (m, 2H), 1.42-1.36 (m, 2H), 1.26 (t, *J* = 7.1 Hz, 3H), 1.09-1.04 (m, 28H), 0.90 (t, *J* = 7.4 Hz, 3H). **^{13}C NMR** (176 MHz, $CDCl_3$) δ 205.66, 171.35, 84.94, 75.30, 71.55, 63.75, 61.74, 46.05, 40.41, 31.82, 30.92, 22.00, 17.50, 17.47, 17.39, 17.37, 17.30, 17.28, 17.21, 17.12, 14.17, 13.75, 13.40, 13.39, 12.60, 12.45. **ESI-HRMS**: Calculated for $C_{26}H_{50}O_7NaSi_2S$ $(M+Na)^+$: 585.27163, Found: 585.27132. $[\alpha]_D^{20}$ = +69.6 (c = 1.0, CH_2Cl_2).

Ethyl (6a*R*,8*R*,9*R*,11a*R*)-2,2,4,4-tetraisopropyl-11-oxo-8-phenoxyhexahydro-6H-oxepino[3,2-f][1,3,5,2,4]trioxadisilocine-9-carboxylate (3ai)

The title product compound is prepared according to the general procedure **C** with 2 mol% catalyst **A** and 0.2 mmol cyclopropanated donor at 50 ˚C for 18 h and isolated by flash column chromatography (10:1 Pentane: Ethyl Acetate) giving **3ai** as an oil (82 mg, 72% yield, α/β ratio > 20:1).

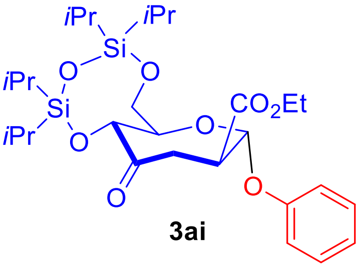

3ai

^{1}H NMR (700 MHz, $CDCl_3$) δ 7.31-7.29 (m, 2H), 7.11-7.09 (m, 2H), 7.04 (tt, *J* = 7.3, 1.1 Hz, 1H), 5.96 (d, *J* = 6.7 Hz, 1H), 4.54 (d, *J* = 9.4 Hz, 1H), 4.22-4.17 (m, 2H), 4.03 (dd, *J* = 11.7, 2.3 Hz, 1H), 3.97 (dt, *J* = 9.4, 2.4 Hz, 1H), 3.71 (dd, *J* = 11.7, 2.6 Hz, 1H), 3.20-3.17 (m, 1H), 2.92 (dd, *J* = 14.6, 10.0 Hz, 1H), 2.80 (dd, *J* = 14.6, 2.7 Hz, 1H), 1.26 (t, *J* = 7.2 Hz, 3H), 1.09-0.99 (m, 28H). **^{13}C NMR** (176 MHz, $CDCl_3$) δ 205.61, 171.02, 156.52, 129.73, 122.90, 117.01, 98.85, 75.26, 70.79, 63.25, 61.78, 45.31, 38.66, 17.47, 17.44, 17.39, 17.38, 17.30, 17.27, 17.20, 17.14, 14.23, 13.42, 13.34, 12.65, 12.46. **ESI-HRMS**: Calculated for $C_{28}H_{47}O_8Si_2$ $(M+H)^+$: 567.28040, Found: 567.28158. $[\alpha]_D^{20}$ = +42.9 (c = 1.0, CH_2Cl_2).

Ethyl (6a*R*,8*R*,9*R*,11a*R*)-2,2,4,4-tetraisopropyl-8-(4-methoxyphenoxy)-11-oxohexahydro-6H-oxepino[3,2-f][1,3,5,2,4]trioxadisilocine-9-carboxylate (3aj)

The title product compound is prepared according to the general procedure **A** with 2 mol% catalyst **A** and 0.2 mmol cyclopropanated donor at 35 ˚C for 12 h and isolated by flash column chromatography (10:1 Pentane: Ethyl Acetate) giving **3aj** as an oil (99 mg, 83% yield, α/β ratio > 20:1).

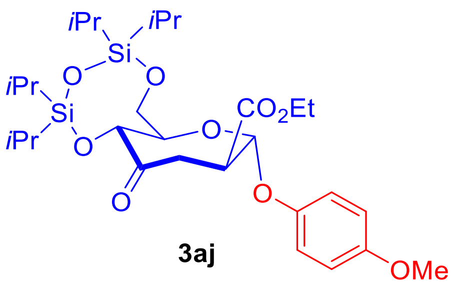

3aj

^{1}H NMR (500 MHz, $CDCl_3$) δ 7.03-7.00 (m, 2H), 6.83-6.80 (m, 2H), 5.85 (d, *J* = 6.8 Hz, 1H), 4.53 (d, *J* = 9.4 Hz, 1H), 4.19 (p, *J* = 7.2 Hz, 2H), 4.05 (dd, *J* = 11.7, 2.2 Hz, 1H), 3.97 (dt, *J* = 9.4, 2.4 Hz, 1H), 3.76 (s, 3H), 3.73 (dd, *J* = 11.7, 2.6 Hz, 1H), 3.16-3.12 (m, 1H), 2.90 (dd, *J* = 14.5, 10.0 Hz, 1H), 2.77 (dd, *J* = 14.5, 2.7 Hz, 1H), 1.26 (t, *J* = 7.1 Hz, 3H), 1.07-0.96 (m, 28H). **^{13}C NMR** (126 MHz, $CDCl_3$) δ 205.64, 171.05, 155.33, 150.46, 118.17, 114.70, 99.61, 75.18, 70.60, 63.22, 61.72, 55.71, 45.22, 38.61, 17.45, 17.41, 17.36, 17.34, 17.25, 17.23, 17.16, 17.09, 14.20, 13.36, 13.31, 12.56, 12.38. **ESI-HRMS**: Calculated for $C_{29}H_{49}O_9Si_2$ $(M+H)^+$: 597.29096, Found: 597.29187. $[\alpha]_D^{20}$ = +44.1 (c = 2.0, CH_2Cl_2).

Ethyl (2*R*,3*R*,6*R*,7*R*)-6-(benzyloxy)-7-(((tert-butyldimethylsilyl)oxy)methyl)-2-(4-methoxyphenoxy)-5-oxooxepane-3-carboxylate (3ak)

The title product compound is prepared according to the general procedure **B** with 2 mol% catalyst **A** and 0.2 mmol cyclopropanated donor at rt for 12 h and isolated by flash column chromatography (15:1 Pentane: Ethyl Acetate) giving **3ak** as an oil (87 mg, 78% yield, α/β ratio > 20:1).

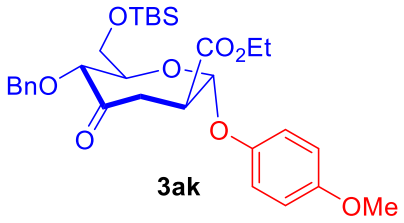

3ak

^{1}H NMR (500 MHz, $CDCl_3$) δ 7.37-7.31 (m, 5H), 7.01-6.97 (m, 2H), 6.81-6.78 (m, 2H), 5.60 (d, *J* = 8.7 Hz, 1H), 4.62 (d, *J* = 11.4 Hz, 1H), 4.54 (d, *J* = 11.5 Hz, 1H), 4.27 (q, *J* = 5.2 Hz, 1H), 4.20 (qd, *J* = 7.1, 2.0 Hz, 2H), 4.03 (d, *J* = 4.9 Hz, 1H), 3.96-3.91 (m, 1H), 3.76 (s, 3H), 3.67 (dd, *J* = 10.6, 5.1 Hz, 1H), 3.49 (dd, *J* = 10.6, 5.5 Hz, 1H), 2.73-2.65 (m, 2H), 1.28 (t, *J* = 7.1 Hz, 3H), 0.85 (s, 9H), -0.02 (d, *J* = 6.8 Hz, 6H). **^{13}C NMR** (126 MHz, $CDCl_3$) δ 205.65, 171.13, 155.34, 150.95, 137.27, 128.63, 128.21, 128.18, 118.66, 114.60, 101.05, 85.61, 73.37, 72.69, 63.90, 61.48, 55.78, 44.83, 39.78, 25.97, 25.93, 18.43, 14.30, -5.33. **ESI-HRMS**: Calculated for $C_{30}H_{42}O_8NaSi$ $(M+Na)^+$: 581.2541, Found: 581.2536. $[\alpha]_D^{20}$ = +79.1 (c = 1.0, CH_2Cl_2).

Ethyl (6a*R*,8*R*,9*R*,11a*R*)-8-(4-bromophenoxy)-2,2,4,4-tetraisopropyl-11-oxohexahydro-6H-oxepino[3,2-f][1,3,5,2,4]trioxadisilocine-9-carboxylate (3al)

The title product compound is prepared according to the general procedure **C** with 2 mol% catalyst **A** and 0.2 mmol cyclopropanated donor at 50 ˚C for 18 h and isolated by flash column chromatography (10:1 Pentane: Ethyl Acetate) giving **3al** as an oil (90 mg, 70% yield, α/β ratio > 20:1).

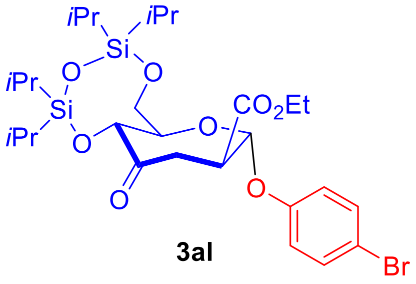

3al

^{1}H NMR (700 MHz, $CDCl_3$) δ 7.41-7.38 (m, 2H), 7.00-6.98 (m, 2H), 5.90 (d, *J* = 6.9 Hz, 1H), 4.52 (d, *J* = 9.4 Hz, 1H), 4.21-4.16 (m, 2H), 4.02 (dd, *J* = 11.7, 2.2 Hz, 1H), 3.92 (dt, *J* = 9.4, 2.4 Hz, 1H), 3.67 (dd, *J* = 11.8, 2.7 Hz, 1H), 3.17-3.15 (m, 1H), 2.90 (dd, *J* = 14.4, 10.3 Hz, 1H), 2.77 (dd, *J* = 14.4, 2.6 Hz, 1H), 1.25 (t, *J* = 7.1 Hz, 3H), 1.08-0.99 (m, 28H). **^{13}C NMR** (176 MHz, $CDCl_3$) δ 205.27, 170.81, 155.60, 132.63, 118.72, 115.33, 99.02, 75.15, 71.01, 63.16, 61.85, 45.06, 38.68, 17.45, 17.43, 17.36, 17.27, 17.24, 17.17, 17.11, 14.21, 13.40, 13.31, 12.64, 12.43. **ESI-HRMS**: Calculated for $C_{28}H_{46}O_8Si_2Br$ $(M+H)^+$: 645.19091, Found: 645.19223, 647.19005, Found: 647.19024. $[\alpha]_D^{20}$ = +45.5 (c = 2.0, CH_2Cl_2).

Ethyl (6a*R*,8*R*,9*R*,11a*R*)-8-(3,4-dichlorophenoxy)-2,2,4,4-tetraisopropyl-11-oxohexahydro-6H-oxepino[3,2-f][1,3,5,2,4]trioxadisilocine-9-carboxylate (3am)

The title product compound is prepared according to the general procedure **A** with 2 mol% catalyst **A** and 0.2 mmol cyclopropanated donor at 35 ˚C for 12 h and isolated by flash column chromatography (10:1 Pentane: Ethyl Acetate) giving **3am** as an oil (76 mg, 60% yield, α/β ratio > 20:1).

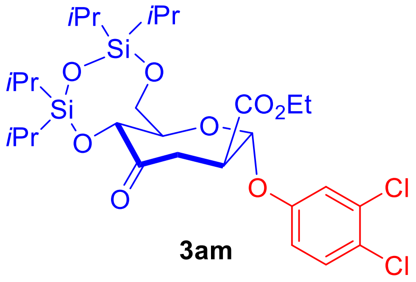

3am

^{1}H NMR (700 MHz, $CDCl_3$) δ 7.36 (d, *J* = 8.8 Hz, 1H), 7.21 (d, *J* = 2.8 Hz, 1H), 6.99 (dd, *J* = 8.9, 2.8 Hz, 1H), 5.90 (d, *J* = 7.1 Hz, 1H), 4.52 (d, *J* = 9.3 Hz, 1H), 4.22-4.16 (m, 2H), 4.03 (dd, *J* = 11.8, 2.2 Hz, 1H), 3.92-3.90 (m, 1H), 3.68 (dd, *J* = 11.8, 2.8 Hz, 1H), 3.18-3.15 (m, 1H), 2.88 (dd, *J* = 14.4, 10.5 Hz, 1H), 2.77 (dd, *J* = 14.4, 2.6 Hz, 1H), 1.26 (t, *J* = 7.1 Hz, 3H), 1.07-1.02 (m, 28H). **^{13}C NMR** (176 MHz, $CDCl_3$) δ 205.00, 170.67, 155.41, 133.22, 131.04, 126.42, 119.20, 116.48, 99.30, 75.16, 71.40, 63.15, 61.95, 44.87, 38.74, 17.45, 17.36, 17.35, 17.28, 17.25, 17.17, 17.11, 14.21, 13.41, 13.33, 12.67, 12.44. **ESI-HRMS**: Calculated for $C_{28}H_{45}O_8Si_2Cl_2$ $(M+H)^+$: 635.20245, Found: 635.20389. $[\alpha]_D^{20}$ = +39.0 (c = 1.0, CH_2Cl_2).

Ethyl (6aR,8R,9S,11aR)-2,2,4,4-tetraisopropyl-11-oxo-8-(4-(trifluoromethyl)phenoxy)hexahydro-6H-oxepino[3,2-f][1,3,5,2,4]trioxadisilocine-9-carboxylate (3an)

The title product compound is prepared according to the general procedure **A** with 5 mol% catalyst **A** and 0.2 mmol cyclopropanated donor at 45 ˚C for 48 h and isolated by flash column chromatography (10:1 Pentane: Ethyl Acetate) giving **3an** as an oil (63 mg, 50% yield, α/β ratio > 20:1).

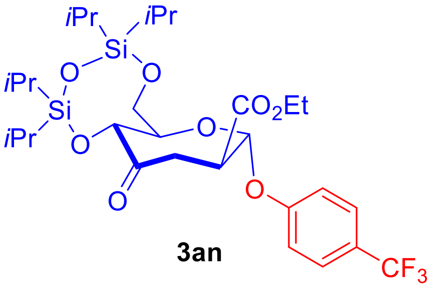

3an

^{1}H NMR (600 MHz, $CDCl_3$) δ 7.61 (d, *J* = 8.2 Hz, 2H), 7.23 (d, *J* = 8.3 Hz, 2H), 6.02 (d, *J* = 7.2 Hz, 1H), 4.51 (d, *J* = 9.5 Hz, 1H), 4.18 (qd, *J* = 7.1, 1.4 Hz, 2H), 4.01 (dd, *J* = 11.8, 2.2 Hz, 1H), 3.96 (dt, *J* = 9.5, 2.3 Hz, 1H), 3.62 (dd, *J* = 11.8, 2.5 Hz, 1H), 3.18 (ddd, *J* = 10.9, 7.2, 2.2 Hz, 1H), 2.92 (dd, *J* = 14.2, 10.9 Hz, 1H), 2.76 (dd, *J* = 14.2, 2.2 Hz, 1H), 1.25 (t, *J* = 7.1 Hz, 3H), 1.07 – 1.00 (m, 28H). **^{13}C NMR** (151 MHz, $CDCl_3$) δ 205.15, 170.94, 159.44, 127.46 (q, *J* = 3.7 Hz), 124.78 (q, *J* = 271.8 Hz), 124.96 (q, *J* = 32.7 Hz), 117.26, 99.29, 75.34, 71.52, 63.37, 62.22, 45.24, 39.18, 17.47, 17.43, 17.41, 17.35, 17.28, 17.24, 17.19, 14.27, 13.70, 13.58, 13.08, 12.76. **ESI-HRMS**: Calculated for $C_{29}H_{46}O_8F_3Si_2$ $(M+H)^+$: 635.2679, Found: 635.2690. $[\alpha]_D^{20}$ = +51.9 (c = 1.0, CH_2Cl_2).

Ethyl (6a*R*,8*R*,9*R*,11a*R*)-2,2,4,4-tetraisopropyl-8-(naphthalen-1-yloxy)-11-oxohexahydro-6H-oxepino[3,2-f][1,3,5,2,4]trioxadisilocine-9-carboxylate (3ao)

The title product compound is prepared according to the general procedure **A** with 2 mol% catalyst **A** and 0.2 mmol cyclopropanated donor at 35 ˚C for 12 h and isolated by flash column chromatography (10:1 Pentane: Ethyl Acetate) giving **3ao** as an oil (94 mg, 76% yield, α/β ratio > 20:1).

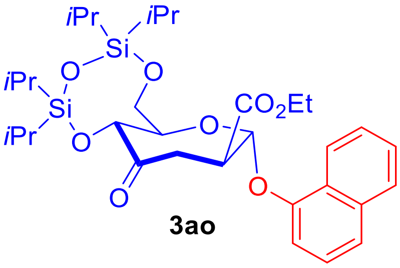

3ao

^{1}H NMR (700 MHz, $CDCl_3$) δ 8.13 (d, *J* = 7.8 Hz, 1H), 7.81 (d, *J* = 8.5 Hz, 1H), 7.53 (d, *J* = 8.2 Hz, 1H), 7.51-7.46 (m, 2H), 7.41 (t, *J* = 7.9 Hz, 1H), 7.35 (d, *J* = 7.6 Hz, 1H), 6.17 (d, *J* = 6.7 Hz, 1H), 4.61 (d, *J* = 9.3 Hz, 1H), 4.21-4.15 (m, 2H), 4.02 (dt, *J* = 9.3, 2.5 Hz, 1H), 3.98 (dd, *J* = 11.7, 2.2 Hz, 1H), 3.69 (dd, *J* = 11.8, 2.6 Hz, 1H), 3.43-3.40 (m, 1H), 3.04 (dd, *J* = 14.7, 9.9 Hz, 1H), 2.90 (dd, *J* = 14.7, 2.8 Hz, 1H), 1.23 (t, *J* = 7.1 Hz, 3H), 1.09-0.96 (m, 28H). **^{13}C NMR** (176 MHz, $CDCl_3$) δ 205.63, 171.05, 152.07, 134.64, 127.75, 126.62, 126.02, 125.77, 125.62, 122.34, 121.73, 108.76, 98.86, 75.33, 71.06, 63.21, 61.87, 45.47, 38.72, 17.43, 17.41, 17.38, 17.32, 17.26, 17.19, 17.17, 14.21, 13.43, 13.32, 12.67, 12.45. **ESI-HRMS**: Calculated for $C_{32}H_{49}O_8Si_2$ $(M+H)^+$: 617.29605, Found: 617.29757. $[\alpha]_D^{20}$ = +34.7 (c = 1.0, CH_2Cl_2).

Ethyl (6a*R*,8*S*,9*R*)-2,2,4,4-tetraisopropyl-11-oxo-8-(((3a*R*,5*R*,5a*S*,8a*S*,8b*R*)-2,2,7,7-tetramethyltetrahydro-5H-bis([1,3]dioxolo)[4,5-b:4',5'-d]pyran-5-yl)methoxy)hexahydro-6H-oxepino[3,2-f][1,3,5,2,4]trioxadisilocine-9-carboxylate (3ap)

The title product compound is prepared according to the general procedure **B** with 2 mol% catalyst **A** and 0.2 mmol cyclopropanated donor at rt for 2 h and isolated by flash column chromatography (10:1 Pentane: Ethyl Acetate) giving **3ap** as an oil (132 mg, 90% yield, α/β ratio > 20:1).

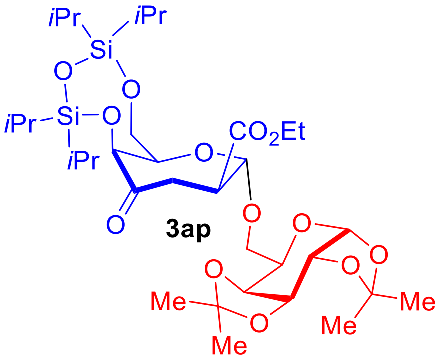

3ap

^{1}H NMR (700 MHz, $CDCl_3$) δ 5.50 (d, *J* = 5.0 Hz, 1H), 5.29 (d, *J* = 7.2 Hz, 1H), 4.58 (dd, *J* = 7.9, 2.5 Hz, 1H), 4.35 (dd, *J* = 2.1, 1.0 Hz, 1H), 4.29 (dd, *J* = 5.0, 2.4 Hz, 1H), 4.18 (dd, *J* = 7.9, 2.0 Hz, 1H), 4.15 (q, *J* = 7.1 Hz, 2H), 4.03 (ddd, *J* = 10.2, 5.7, 2.1 Hz, 1H), 3.91-3.88 (m, 2H), 3.83 (dd, *J* = 10.2, 5.6 Hz, 1H), 3.77 (dd, *J* = 9.6, 6.1 Hz, 1H), 3.62 (dd, *J* = 9.6, 7.4 Hz, 1H), 2.65 (ddd, *J* = 11.3, 7.2, 3.6 Hz, 1H), 2.39 (dd, *J* = 12.0, 3.6 Hz, 1H), 1.52 (s, 3H), 1.42 (s, 3H), 1.32 (s, 3H), 1.31 (s, 3H), 1.26 (t, *J* = 7.1 Hz, 3H), 1.13-1.01 (m, 25H), 0.95-0.93 (m, 3H). **^{13}C NMR** (176 MHz, $CDCl_3$) δ 206.85, 171.24, 109.32, 108.78, 100.66, 96.43, 76.28, 70.86, 70.75, 70.73, 69.73, 66.79, 66.20, 61.31, 59.33, 47.26, 37.27, 26.18, 26.12, 25.05, 24.66, 17.46, 17.42, 17.41, 17.38, 17.27, 17.24, 17.23, 17.20, 14.26, 13.34, 13.12, 12.73, 12.64. **ESI-HRMS**: Calculated for $C_{34}H_{60}O_{13}NaSi_2$ $(M+Na)^+$: 755.34647, Found: 755.34729. $[\alpha]_D^{20}$ = -0.5 (c = 1.0, CH_2Cl_2).

Ethyl (6a*R*,8*S*,9*R*)-2,2,4,4-tetraisopropyl-11-oxo-8-(((3a*S*,5a*R*,8a*R*,8b*S*)-2,2,7,7-tetramethyltetrahydro-3aH-bis([1,3]dioxolo)[4,5-b:4',5'-d]pyran-3a-yl)methoxy)hexahydro-6H-oxepino[3,2-f][1,3,5,2,4]trioxadisilocine-9-carboxylate (3aq)

The title product compound is prepared according to the general procedure **B** with 2 mol% catalyst **A** and 0.2 mmol cyclopropanated donor at rt for 2.5 h and isolated by flash column chromatography (10:1 Pentane: Ethyl Acetate) giving **3aq** as an oil (129 mg, 88% yield, α/β ratio > 20:1).

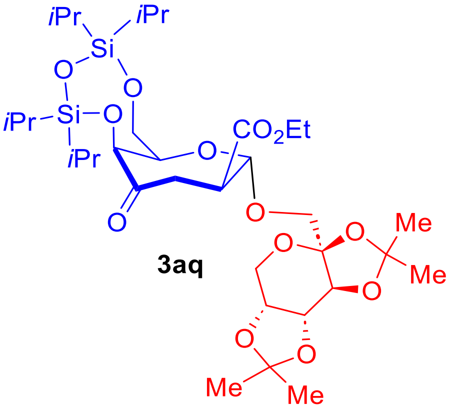

3aq

^{1}H NMR (600 MHz, $CDCl_3$) δ 5.26 (d, *J* = 7.4 Hz, 1H), 4.55 (dd, *J* = 7.9, 2.7 Hz, 1H), 4.34 (dd, *J* = 2.1, 1.1 Hz, 1H), 4.29 (d, *J* = 2.7 Hz, 1H), 4.20-4.14 (m, 2H), 4.02 (ddd, *J* = 9.9, 5.9, 2.0 Hz, 1H), 3.90-3.80 (m, 4H), 3.69 (d, *J* = 13.0 Hz, 1H), 3.41 (d, *J* = 10.0 Hz, 1H), 3.34 (t, *J* = 11.9 Hz, 1H), 2.66 (ddd, *J* = 12.0, 7.4, 3.4 Hz, 1H), 2.39 (dd, *J* = 11.7, 3.5 Hz, 1H), 1.49 (s, 3H), 1.42 (s, 3H), 1.34 (s, 3H), 1.30 (s, 3H), 1.25 (t, *J* = 7.2 Hz, 3H), 1.10-0.99 (m, 25H), 0.92 (d, *J* = 6.5 Hz, 3H). **^{13}C NMR** (151 MHz, $CDCl_3$) δ 206.69, 171.24, 108.94, 108.93, 108.86, 101.98, 100.76, 76.10, 71.05, 70.20, 69.78, 69.74, 69.65, 61.53, 61.17, 59.12, 47.26, 37.16, 26.68, 25.98, 25.37, 24.08, 17.41, 17.36, 17.32, 17.23, 17.21, 17.18, 17.17, 17.14, 14.09, 13.28, 13.06, 12.66, 12.59. **ESI-HRMS**: Calculated for $C_{34}H_{60}O_{13}NaSi_2$ $(M+Na)^+$: 755.3465, Found: 755.3464. $[\alpha]_D^{20}$ = +18.8 (c = 2.0, CH_2Cl_2).

Ethyl (6a*R*,8*S*,9*R*)-2,2,4,4-tetraisopropyl-8-(((3a*R*,4*R*,6*R*,6a*R*)-6-methoxy-2,2-dimethyltetrahydro furo[3,4-d][1,3]dioxol-4-yl)methoxy)-11-oxohexahydro-6H-oxepino[3,2-f][1,3,5,2,4]trioxadisilo cine-9-carboxylate (3ar)

The title product compound is prepared according to the general procedure **B** with 2 mol% catalyst **A** and 0.2 mmol cyclopropanated donor at rt for 3 h and isolated by flash column chromatography (10:1 Pentane: Ethyl Acetate) giving **3ar** as an oil (116 mg, 86% yield, α/β ratio > 20:1).

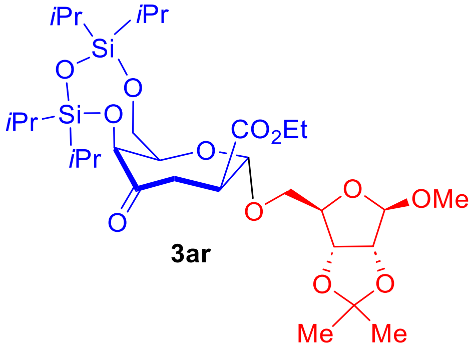

3ar

^{1}H NMR (700 MHz, $CDCl_3$) δ 5.26 (d, *J* = 7.3 Hz, 1H), 4.92 (s, 1H), 4.58 (d, *J* = 6.2 Hz, 1H), 4.54 (d, *J* = 6.0 Hz, 1H), 4.34 (s, 1H), 4.25 (dd, *J* = 8.9, 6.0 Hz, 1H), 4.18-4.13 (m, 2H), 3.99 (ddd, *J* = 10.2, 5.5, 2.0 Hz, 1H), 3.89 (t, *J* = 10.2 Hz, 1H), 3.79 (dd, *J* = 10.2, 5.5 Hz, 1H), 3.73 (dd, *J* = 9.7, 5.6 Hz, 1H), 3.37- 3.32 (m, 2H), 3.28 (s, 3H), 2.68-2.65 (m, 1H), 2.40 (dd, *J* = 12.4, 3.8 Hz, 1H), 1.45 (s, 3H), 1.28 (s, 3H), 1.26 (t, *J* = 7.2 Hz, 3H), 1.12-0.98 (m, 25H), 0.93 (d, *J* = 6.6 Hz, 3H). **^{13}C NMR** (176 MHz, $CDCl_3$) δ ^{13}C 206.55, 171.38, 112.48, 109.52, 101.04, 85.23, 84.74, 82.15, 76.13, 69.73, 69.69, 61.47, 59.37, 55.02, 46.85, 37.23, 26.53, 25.05, 17.47, 17.37, 17.36, 17.22, 17.19, 17.17, 17.15, 14.20, 13.32, 13.09, 12.69, 12.65. **ESI-HRMS**: Calculated for $C_{31}H_{57}O_{12}Si_2$ $(M+H)^+$: 677.33831, Found: 677.33944. $[\alpha]_D^{20}$ = +7.15 (c = 2.0, CH_2Cl_2).

Ethyl (6a*R*,8*S*,9*R*)-2,2,4,4-tetraisopropyl-11-oxo-8-(((2*R*,3*R*,4*S*,5*R*,6*S*)-3,4,5-tris(benzyloxy)-6-methoxytetrahydro-2H-pyran-2-yl)methoxy)hexahydro-6H-oxepino[3,2-f][1,3,5,2,4]trioxadisilo cine-9-carboxylate (3as)

The title product compound is prepared according to the general procedure **B** with 2 mol% catalyst **A** and 0.2 mmol cyclopropanated donor at rt for 3 h and isolated by flash column chromatography (8:1 Pentane: Ethyl Acetate) giving **3as** as an oil (159 mg, 85% yield, α/β ratio > 20:1).

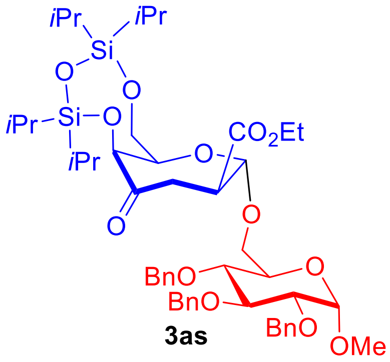

3as

^{1}H NMR (700 MHz, $CDCl_3$) δ 7.39-7.28 (m, 15H), 5.43 (d, *J* = 7.2 Hz, 1H), 5.01 (d, *J* = 11.0 Hz, 1H), 4.86 (dd, *J* = 10.9, 8.9 Hz, 2H), 4.80 (d, *J* = 12.1 Hz, 1H), 4.70 (d, *J* = 12.1 Hz, 1H), 4.61 (d, *J* = 10.8 Hz, 1H), 4.59 (d, *J* = 3.5 Hz, 1H), 4.40 (d, *J* = 2.2 Hz, 1H), 4.14-4.09 (m, 2H), 4.03-3.98 (m, 2H), 3.95 (t, *J* = 10.1 Hz, 1H), 3.82 (dd, *J* = 11.8, 4.4 Hz, 1H), 3.78 (dd, *J* = 9.9, 5.3 Hz, 1H), 3.71-3.68 (m, 2H), 3.53-3.48 (m, 2H), 3.44 (t, *J* = 11.7 Hz, 1H), 3.37 (s, 3H), 2.67 (ddd, *J* = 11.3, 7.2, 4.0 Hz, 1H), 2.39 (dd, *J* = 11.9, 3.9 Hz, 1H), 1.22 (t, *J* = 7.1 Hz, 3H), 1.14 (t, *J* = 7.3 Hz, 6H), 1.11-0.98 (m, 25H). **^{13}C NMR** (176 MHz, $CDCl_3$) δ 207.07, 171.35, 139.00, 138.34, 138.30, 128.55, 128.52, 128.49, 128.45, 128.17,

128.04, 127.98, 127.91, 127.85, 127.63, 100.53, 98.05, 82.04, 79.97, 77.63, 76.51, 75.71, 75.15, 73.41, 70.46, 70.05, 66.23, 61.31, 59.34, 55.17, 47.39, 37.24, 17.47, 17.40, 17.38, 17.37, 17.24, 17.22, 17.20, 17.19, 14.21, 13.35, 13.03, 12.71, 12.61. **ESI-HRMS**: Calculated for $C_{50}H_{73}O_{13}Si_2$ $(M+H)^+$: 937.45842, Found: 937.46110. $[\alpha]_D^{20}$ = +53.15 (c = 2.0, CH_2Cl_2).

Ethyl (6a*R*,8*S*,9*R*)-2,2,4,4-tetraisopropyl-11-oxo-8-(((2*R*,3*R*,4*S*,5*R*,6*S*)-3,4,5,6-tetramethoxytetrahydro-2H-pyran-2-yl)methoxy)hexahydro-6H-oxepino[3,2-f][1,3,5,2,4]trioxadisilocine-9-carboxylate (3at)

The title product compound is prepared according to the general procedure **B** with 2 mol% catalyst **A** and 0.2 mmol cyclopropanated donor at rt for 2.5 h and isolated by flash column chromatography (6:1 Pentane: Ethyl Acetate) giving **3at** as an oil (122 mg, 86% yield, α/β ratio > 20:1).

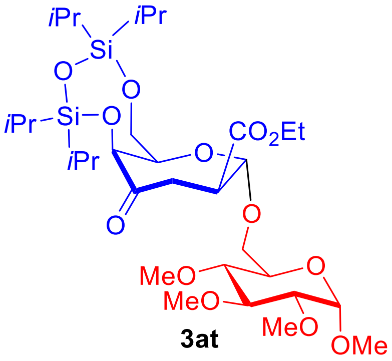

3at

¹H NMR (700 MHz, $CDCl_3$) δ 5.43 (d, *J* = 7.1 Hz, 1H), 4.72 (d, *J* = 3.6 Hz, 1H), 4.37 (d, *J* = 2.9 Hz, 1H), 4.16 (q, *J* = 7.1 Hz, 2H), 4.03-4.00 (m, 1H), 3.95 (t, *J* = 10.1 Hz, 1H), 3.82 (dd, *J* = 9.9, 5.3 Hz, 1H), 3.77 (dd, *J* = 11.6, 4.6 Hz, 1H), 3.66 (dd, *J* = 11.7, 1.7 Hz, 1H), 3.59 (s, 3H), 3.53-3.48 (m, 7H), 3.45 (t, *J* = 9.2 Hz, 1H), 3.41 (t, *J* = 11.6 Hz, 1H), 3.35 (s, 3H), 3.13 (dd, *J* = 9.6, 3.7 Hz, 1H), 3.06-3.04 (m, 1H), 2.68-2.64 (m, 1H), 2.38 (dd, *J* = 12.0, 4.3 Hz, 1H), 1.26 (t, *J* = 7.2 Hz, 3H), 1.12 (t, *J* = 6.8 Hz, 6H), 1.08-0.98 (m, 22H), 0.94 (d, *J* = 6.5 Hz, 3H). **¹³C NMR** (176 MHz, $CDCl_3$) δ 207.13, 171.34, 100.27, 97.44, 83.65, 81.82, 79.39, 76.63, 70.37, 70.17, 66.17, 61.33, 60.91, 60.57, 59.40, 59.08, 55.15, 47.39, 37.20, 17.47, 17.43, 17.40, 17.37, 17.22, 17.19, 17.19, 14.22, 13.35, 13.02, 12.76, 12.60. **ESI-HRMS**: Calculated for $C_{32}H_{61}O_{13}Si_2$ $(M+H)^+$: 709.36452, Found: 709.36634. $[\alpha]_D^{20}$ = +89.75 (c = 2.0, CH_2Cl_2).

Ethyl (6a*R*,8*R*,9*S*,11aS)-8-(((2*S*,6*S*,7*R*,8*S*)-7-(benzyloxy)-6-methoxy-2-phenylhexahydropyrano[3,2-d][1,3]dioxin-8-yl)oxy)-2,2,4,4-tetraisopropyl-11-oxohexahydro-6H-oxepino[3,2-f][1,3,5,2,4]trioxadisilocine-9-carboxylate (3au)

The title product compound is prepared according to the general procedure **B** with 2 mol% catalyst **A** and 0.2 mmol cyclopropanated donor at rt for 6 h and isolated by flash column chromatography (10:1 Pentane: Ethyl Acetate) giving **3au** as an oil (139 mg, 82% yield, α/β ratio > 20:1).

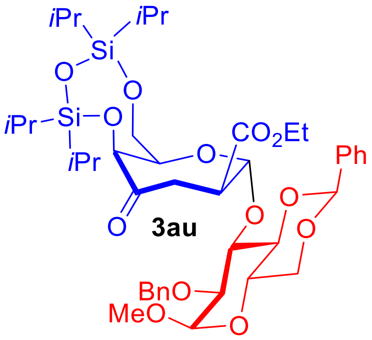

3au

^{1}**H NMR** (500 MHz, CD_2Cl_2) δ 7.47-7.45 (m, 2H), 7.40-7.28 (m, 8H), 5.63 (d, *J* = 6.5 Hz, 1H), 5.46 (s, 1H), 4.77 (d, *J* = 12.1 Hz, 1H), 4.59 (d, *J* = 3.6 Hz, 1H), 4.54 (d, *J* = 12.1 Hz, 1H), 4.25 (s, 1H), 4.23-4.15 (m, 3H), 4.01-3.95 (m, 2H), 3.85 (dd, *J* = 10.7, 7.1 Hz, 1H), 3.75 (ddd, *J* = 10.0, 7.4, 2.8 Hz, 2H), 3.67 (t, *J* = 10.2 Hz, 1H), 3.54 (t, *J* = 9.3 Hz, 1H), 3.46 (dd, *J* = 9.5, 3.6 Hz, 1H), 3.33 (s, 3H), 3.25 (dd, *J* = 12.4, 10.7 Hz, 1H), 2.58 (ddd, *J* = 10.7, 6.5, 2.6 Hz, 1H), 2.35 (ddd, *J* = 12.4, 2.7, 1.3 Hz, 1H), 1.12-1.00 (m, 27H), 0.96-0.95 (m, 4H). ^{13}**C NMR** (126 MHz, $CDCl_3$) δ 206.51, 171.13, 138.61, 137.83, 129.28, 128.79, 128.73, 128.50, 128.30, 126.55, 101.76, 100.35, 99.11, 83.35, 78.82, 75.72, 74.23, 73.47, 69.42, 67.81, 62.14, 61.44, 59.72, 55.46, 47.16, 37.73, 17.55, 17.50, 17.40, 17.39, 17.37, 17.35, 17.31, 17.16, 14.25, 13.63, 13.58, 13.06, 12.94. **ESI-HRMS**: Calculated for $C_{43}H_{65}O_{13}Si_2$ $(M+H)^+$: 845.39582, Found: 845.39850. $[\alpha]_D^{20}$ = +23.0 (c = 1.0, CH_2Cl_2).

Ethyl (6a*R*,8*R*,9*S*,11a*S*)-8-(((2*S*,6*S*,7*R*,8*S*)-8-(benzyloxy)-6-methoxy-2-phenylhexahydropyrano[3,2-d][1,3]dioxin-7-yl)oxy)-2,2,4,4-tetraisopropyl-11-oxohexahydro-6H-oxepino[3,2-f][1,3,5,2,4]trioxadisilocine-9-carboxylate (3av)

The title product compound is prepared according to the general procedure **B** with 2 mol% catalyst **A** and 0.2 mmol cyclopropanated donor at rt for 6 h and isolated by flash column chromatography (10:1 Pentane: Ethyl Acetate) giving **3av** as an oil (135 mg, 80% yield, α/β ratio > 20:1).

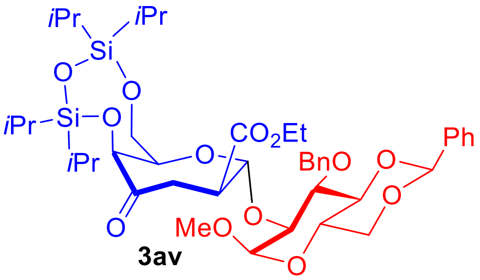

3av

^{1}**H NMR** (500 MHz, CD_2Cl_2) δ 7.47 (dd, *J* = 7.1, 2.4 Hz, 2H), 7.41-7.35 (m, 5H), 7.27-7.23 (m, 3H), 5.58 (s, 1H), 5.36 (d, *J* = 7.4 Hz, 1H), 4.87 (d, *J* = 3.4 Hz, 1H), 4.80 (d, *J* = 11.4 Hz, 1H), 4.74 (d, *J* = 11.3 Hz, 1H), 4.28-4.26 (m, 2H), 4.19 (qd, *J* = 7.1, 1.5 Hz, 2H), 4.06 (dd, *J* = 9.7, 6.2 Hz, 1H), 3.92-3.89 (m, 1H), 3.86 (d, *J* = 9.2 Hz, 1H), 3.83-3.78 (m, 2H), 3.73 (td, *J* = 10.1, 5.6 Hz, 2H), 3.63 (t, *J* = 9.2 Hz, 1H), 3.36 (s, 3H), 3.27 (t, *J* = 12.0 Hz, 1H), 2.61 (ddd, *J* = 12.0, 7.4, 2.1 Hz, 1H), 2.35 (dt, *J* = 11.8, 1.8 Hz, 1H), 1.30 (t, *J* = 7.2 Hz, 3H), 1.11-0.96 (m, 28H). ^{13}**C NMR** (126 MHz, CD_2Cl_2) δ 205.74, 171.45, 138.98, 138.07, 129.22, 128.84, 128.55, 128.51, 127.95, 126.49, 101.71, 98.65, 97.90, 82.58, 77.28, 76.02, 75.79, 75.56, 69.44, 68.87, 62.71, 61.87, 59.46, 55.39, 46.73, 38.00, 17.55, 17.47, 17.44, 17.43, 17.39, 17.34, 17.28, 17.18, 14.32, 13.58, 13.54, 13.09, 12.94. **ESI-HRMS**: Calculated for $C_{43}H_{65}O_{13}Si_2$ $(M+H)^+$: 845.39582, Found: 845.39551. $[\alpha]_D^{20}$ = +18.35 (c = 2.0, CH_2Cl_2).

Ethyl (6a*R*,8*R*,9*R*)-8-(((3a*R*,5*R*,6*S*,6a*R*)-5-((*R*)-2,2-dimethyl-1,3-dioxolan-4-yl)-2,2-dimethyltetrahydrofuro[2,3-d][1,3]dioxol-6-yl)oxy)-2,2,4,4-tetraisopropyl-11-oxohexahydro-6H-oxepino[3,2-f][1,3,5,2,4]trioxadisilocine-9-carboxylate (3aw)

The title product compound is prepared according to the general procedure **B** with 2 mol% catalyst **A** and 0.2 mmol cyclopropanated donor at rt for 6 h and isolated by flash column chromatography (10:1 Pentane: Ethyl Acetate) giving **3aw** as an oil (116 mg, 79% yield, α/β ratio > 20:1).

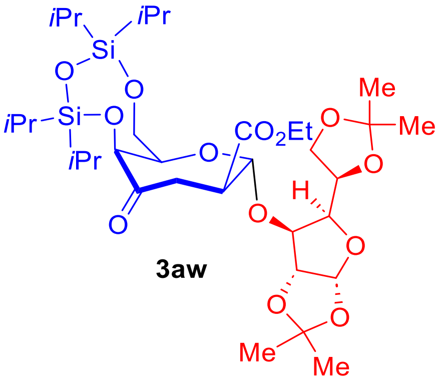

3aw

^{1}H NMR (600 MHz, $CDCl_3$) δ 5.97 (d, *J* = 3.7 Hz, 1H), 5.29 (d, *J* = 7.0 Hz, 1H), 4.55 (d, *J* = 3.7 Hz, 1H), 4.30 (s, 1H), 4.21 (dd, *J* = 6.9, 3.8 Hz, 1H), 4.18-4.12 (m, 3H), 4.02 (t, *J* = 8.1 Hz, 1H), 3.84 (d, *J* = 7.8 Hz, 2H), 3.74-3.71 (m, 2H), 3.69-3.66 (m, 1H), 3.33 (t, *J* = 11.7 Hz, 1H) 2.64 (ddd, *J* = 11.6, 7.0, 3.1 Hz, 1H), 2.38 (dd, *J* = 12.3, 2.6 Hz, 1H), 1.47 (s, 3H), 1.34 (s, 3H), 1.32 (s, 3H), 1.31 (s, 3H), 1.25 (t, *J* = 7.1 Hz, 3H), 1.11-1.00 (m, 25H), 0.94 (d, *J* = 6.0 Hz, 3H). **^{13}C NMR** (151 MHz, $CDCl_3$) δ 206.59, 171.30, 112.28, 106.52, 101.09, 100.01, 84.09, 79.53, 75.87, 75.08, 71.21, 68.87, 68.32, 61.38, 59.29, 46.78, 37.42, 27.27, 26.63, 23.96, 23.89, 17.47, 17.41, 17.40, 17.38, 17.36, 17.27, 17.24, 17.16, 14.25, 13.32, 13.16, 12.77, 12.67. **ESI-HRMS**: Calculated for $C_{34}H_{60}O_{13}NaSi_2$ $(M+Na)^+$: 755.34647, Found: 755.34620. $[\alpha]_D^{20}$ = +37.4 (c = 1.0, CH_2Cl_2).

Ethyl (6a*R*,8*R*,9*R*)-2,2,4,4-tetraisopropyl-8-(((4*R*,6*S*,7*S*,7a*R*)-4-methoxy-2,2,6-trimethyltetrahydro-4H-[1,3]dioxolo[4,5-c]pyran-7-yl)oxy)-11-oxohexahydro-6H-oxepino[3,2-f][1,3,5,2,4]trioxadisilocine-9-carboxylate (3ax)

The title product compound is prepared according to the general procedure **B** with 2 mol% catalyst **A** and 0.2 mmol cyclopropanated donor at rt for 12 h and isolated by flash column chromatography (10:1 Pentane: Ethyl Acetate) giving **3ax** as an oil (122 mg, 88% yield, α/β ratio > 20:1).

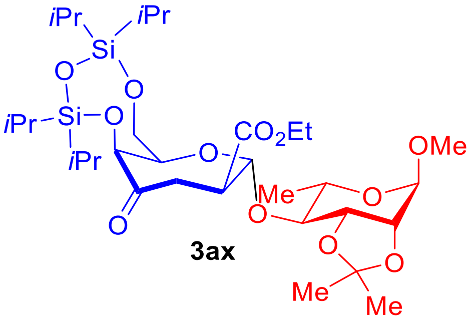

3ax

^{1}H NMR (500 MHz, $CDCl_3$) δ 5.46 (d, *J* = 7.2 Hz, 1H), 4.78 (s, 1H), 4.36 (s, 1H), 4.15 (q, *J* = 7.1 Hz, 2H), 4.11-4.06 (m, 4H), 3.86-3.81 (m, 1H), 3.53 (dd, *J* = 10.0, 6.2 Hz, 1H), 3.39 (t, *J* = 11.8 Hz, 1H), 3.33-3.30 (m, 4H), 2.66 (ddd, *J* = 11.2, 7.2, 3.7 Hz, 1H), 2.34 (dd, *J* = 12.0, 3.8 Hz, 1H), 1.52 (s, 3H), 1.32 (s, 3H), 1.27-1.19 (m, 6H), 1.12-1.01 (m, 28H), 0.93 (d, *J* = 6.2 Hz, 3H). **^{13}C NMR** (126 MHz, $CDCl_3$) δ 206.81, 171.56, 109.15, 100.52, 98.29, 81.26, 76.31, 75.75, 70.26, 64.48, 61.41, 59.05, 54.97, 47.52, 37.33, 27.94, 26.02, 18.04, 17.45, 17.42, 17.40, 17.37, 17.25, 17.21, 17.19, 14.21, 13.32, 13.07, 12.77, 12.54. **ESI-HRMS**: Calculated for $C_{32}H_{59}O_{12}Si_2$ $(M+H)^+$: 691.35396, Found: 691.35535. $[\alpha]_D^{20}$ = +25.0 (c = 1.0, CH_2Cl_2).

Ethyl (6a*R*,8*S*,9*R*)-8-(((3*S*,8*S*,9*S*,10*R*,13*R*,14*S*,17*R*)-10,13-dimethyl-17-((*R*)-6-methylheptan-2-yl)-2,3,4,7,8,9,10,11,12,13,14,15,16,17-tetradecahydro-1H-cyclopenta[a]phenanthren-3-yl)oxy)-2,2,4,4-tetraisopropyl-11-oxohexahydro-6H-oxepino[3,2-f][1,3,5,2,4]trioxadisilocine-9-carboxylate (3ay)

The title product compound is prepared according to the general procedure **B** with 2 mol% catalyst **A** and 0.2 mmol cyclopropanated donor at rt for 24 h and isolated by flash column chromatography (15:1 Pentane: Ethyl Acetate) giving **3ay** as an oil (107 mg, 62% yield, α/β ratio > 20:1).

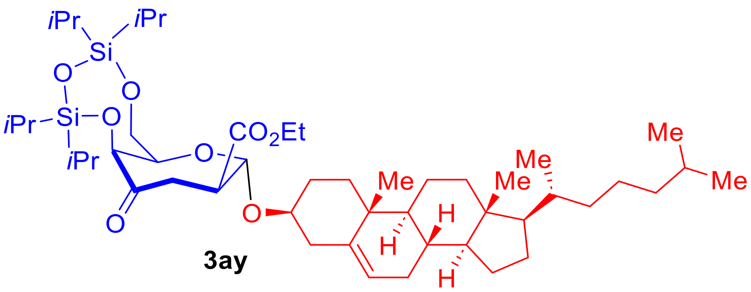

3ay

^{1}H NMR (700 MHz, $CDCl_3$) δ 5.39 (d, *J* = 7.4 Hz, 1H), 5.36 (d, *J* = 4.5 Hz, 1H), 4.34 (s, 1H), 4.17 (qq, *J* = 7.1, 3.7 Hz, 2H), 4.02 (ddd, *J* = 10.2, 5.6, 1.8 Hz, 1H), 3.86 (t, *J* = 10.2 Hz, 1H), 3.76 (dd, *J* = 10.2, 5.7 Hz, 1H), 3.45-3.40 (m, 1H), 3.36 (t, *J* = 11.8 Hz, 1H), 2.63-2.59 (m, 1H), 2.38 (dd, *J* = 11.9, 3.2 Hz, 1H), 2.30-2.23 (m, 2H), 2.02-1.96 (m, 2H), 1.86-1.80 (m, 3H), 1.59-1.33 (m, 11H), 1.27 (t, *J* = 7.2 Hz, 3H), 1.16-0.98 (m, 37H), 0.95 (d, *J* = 6.7 Hz, 3H), 0.91 (d, *J* = 6.5 Hz, 3H), 0.86 (dd, *J* = 6.6, 3.2 Hz, 6H). **^{13}C NMR** (176 MHz, $CDCl_3$) δ 206.82, 171.56, 140.76, 122.02, 98.07, 76.12, 69.27, 61.34, 59.65, 56.91, 56.31, 50.25, 47.24, 42.47, 40.13, 39.92, 39.67, 37.41, 37.12, 36.89, 36.34, 35.93, 32.06, 32.03, 28.38, 28.16, 27.70, 24.43, 23.97, 22.97, 22.71, 21.21, 19.50, 18.87, 17.53, 17.45, 17.43, 17.28, 17.24, 17.23, 17.19, 14.33, 13.37, 13.16, 12.75, 12.68, 12.00. **ESI-HRMS**: Calculated for $C_{49}H_{87}O_8Si_2$ $(M+H)^+$: 859.59340, Found: 859.59340. $[\alpha]_D^{20}$ = +18.5 (c = 1.0, CH_2Cl_2).

Ethyl (6a*R*,8*S*,9*R*)-2,2,4,4-tetraisopropyl-8-(((1*R*,2*R*,5*R*)-2-isopropyl-5-methylcyclohexyl)oxy)-11-oxohexahydro-6H-oxepino[3,2-f][1,3,5,2,4]trioxadisilocine-9-carboxylate (3az)

The title product compound is prepared according to the general procedure **B** with 2 mol% catalyst **A** and 0.2 mmol cyclopropanated donor at rt for 21 h and isolated by flash column chromatography (15:1 Pentane: Ethyl Acetate) giving **3az** as an oil (112 mg, 89% yield, α/β ratio > 20:1).

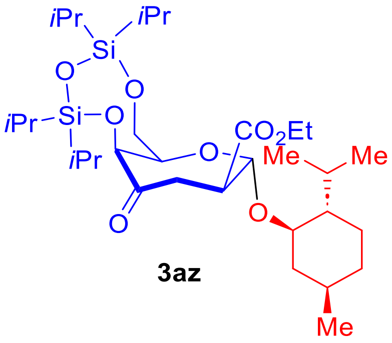

3az

^{1}H NMR (500 MHz, CD_2Cl_2) δ 5.33 (d, *J* = 7.4 Hz, 1H), 4.35 (s, 1H), 4.16-4.07 (m, 2H), 4.05-4.01 (m, 1H), 3.91 (t, *J* = 10.1 Hz, 1H), 3.82 (dd, *J* = 10.0, 5.5 Hz, 1H), 3.36 (t, *J* = 11.8 Hz, 1H), 3.25 (td, *J* = 10.6, 4.3 Hz, 1H), 2.61 (ddd, *J* = 11.3, 7.4, 3.5 Hz, 1H), 2.28 (dd, *J* = 12.2, 3.2 Hz, 1H), 2.15-2.11 (m, 1H), 1.96 (pd, *J* = 7.0, 2.5 Hz, 1H), 1.64-1.58 (m, 2H), 1.39-1.33 (m, 1H), 1.25 (t, *J* = 7.1 Hz, 3H), 1.20-1.02 (m, 25H), 0.95 (t, *J* = 7.1 Hz, 6H), 0.91 (d, *J* = 6.6 Hz, 3H), 0.85 (d, *J* = 7.0 Hz, 3H), 0.81 (dd, *J* = 12.3, 3.2 Hz, 1H), 0.69 (d, *J* = 6.9 Hz, 3H). **^{13}C NMR** (126 MHz, CD_2Cl_2) δ 206.82, 171.73, 102.12, 81.02, 76.66, 70.34, 61.61, 59.57, 48.93, 48.08, 44.00, 37.79, 34.59, 32.04, 25.24, 23.22, 22.42, 21.30, 17.53, 17.51, 17.45, 17.31, 17.26, 17.24, 16.02, 14.24, 13.65, 13.35, 13.09, 12.92. **ESI-HRMS**: Calculated for $C_{32}H_{61}O_8Si_2$ $(M+H)^+$: 629.38995, Found: 629.39151. $[\alpha]_D^{20}$ = +7.6 (c = 1.0, CH_2Cl_2).

Ethyl (6a*R*,8*S*,9*R*)-2,2,4,4-tetraisopropyl-8-methoxy-11-oxohexahydro-6H-oxepino[3,2-f][1,3,5,2,4]trioxadisilocine-9-carboxylate (3ba)

The title product compound is prepared according to the general procedure **B** with 2 mol% catalyst **A** and 0.2 mmol cyclopropanated donor at rt for 2 h and isolated by flash column chromatography (10:1 Pentane: Ethyl Acetate) giving **3ba** as an oil (74 mg, 73% yield, α/β ratio > 20:1).

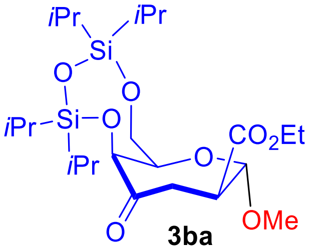

3ba

^{1}H NMR (500 MHz, $CDCl_3$) δ 5.17 (d, *J* = 7.2 Hz, 1H), 4.35 (s, 1H), 4.19-4.13 (m, 2H), 3.98 (td, *J* = 5.1, 1.9 Hz, 1H), 3.92 (t, *J* = 10.1 Hz, 1H), 3.80 (dd, *J* = 9.9, 5.3 Hz, 1H), 3.39-3.35 (m, 4H), 2.63-2.59 (m, 1H), 2.38 (dd, *J* = 12.2, 3.6 Hz, 1H), 1.25 (t, *J* = 7.1 Hz, 3H), 1.12-0.93 (m, 28H). **^{13}C NMR** (126 MHz, $CDCl_3$) δ 206.87, 171.39, 101.61, 76.23, 69.53, 61.40, 59.42, 56.17, 47.06, 37.22, 17.45, 17.36, 17.23, 17.20, 17.18, 17.16, 14.21, 13.32, 13.08, 12.67, 12.61. **ESI-HRMS**: Calculated for $C_{23}H_{45}O_8Si_2$ $(M+H)^+$: 505.26475, Found: 505.26525. $[\alpha]_D^{20}$ = +35.2 (c = 1.0, CH_2Cl_2).

Ethyl (6a*R*,8*S*,9*R*)-2,2,4,4-tetraisopropyl-8-(octyloxy)-11-oxohexahydro-6H-oxepino[3,2-f][1,3,5,2,4]trioxadisilocine-9-carboxylate (3bb)

The title product compound is prepared according to the general procedure **B** with 2 mol% catalyst **A** and 0.2 mmol cyclopropanated donor at rt for 3 h and isolated by flash column chromatography (15:1 Pentane: Ethyl Acetate) giving **3bb** as an oil (78 mg, 65% yield, α/β ratio > 20:1).

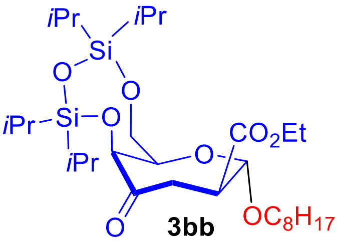

3bb

^{1}H NMR (700 MHz, $CDCl_3$) δ 5.26 (d, *J* = 7.2 Hz, 1H), 4.36 (s, 1H), 4.16 (q, *J* = 7.1 Hz, 2H), 3.99 (ddd, *J* = 10.3, 5.4, 2.1 Hz, 1H), 3.91 (t, *J* = 10.2 Hz, 1H), 3.78 (dd, *J* = 10.1, 5.5 Hz, 1H), 3.66 (dt, *J* = 9.5, 6.5 Hz, 1H), 3.40-3.34 (m, 2H), 2.64-2.61 (m, 1H), 2.39-2.37 (m, 1H), 1.54-1.49 (m, 2H), 1.30-1.24 (m, 13H), 1.12 (dd, *J* = 10.0, 7.0 Hz, 6H), 1.09-1.05 (m, 8H), 1.03-1.00 (m, 10H), 0.95-0.91 (m, 4H), 0.87 (t, *J* = 7.1 Hz, 3H). **^{13}C NMR** (176 MHz, $CDCl_3$) δ 207.04, 171.51, 100.23, 76.42, 69.70, 68.78, 61.29, 59.51, 47.27, 37.26, 31.94, 29.46, 29.42, 29.37, 26.12, 22.78, 17.48, 17.41, 17.40, 17.25, 17.23, 17.20, 17.20, 14.26, 14.21, 13.36, 13.10, 12.72, 12.65. **ESI-HRMS**: Calculated for $C_{30}H_{59}O_8Si_2$ $(M+H)^+$: 603.37430, Found: 603.37572. $[\alpha]_D^{20}$ = +19.7 (c = 1.0, CH_2Cl_2).

Ethyl (6a*R*,8*S*,9*S*,11a*S*)-2,2,4,4-tetraisopropyl-8-(neopentyloxy)-11-oxohexahydro-6H-oxepino [3,2-f][1,3,5,2,4]trioxadisilocine-9-carboxylate (3bc)

The title product compound is prepared according to the general procedure **B** with 2 mol% catalyst **A** and 0.2 mmol cyclopropanated donor at rt for 12 h and isolated by flash column chromatography (15:1 Pentane: Ethyl Acetate) giving **3bc** as an oil (76 mg, 68% yield, α/β ratio > 20:1).

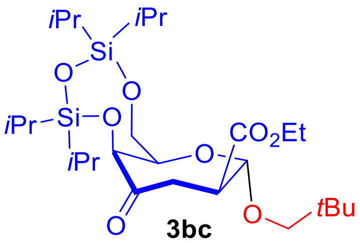

3bc

^{1}H NMR (500 MHz, $CDCl_3$) δ 5.22 (d, *J* = 7.2 Hz, 1H), 4.35 (s, 1H), 4.15 (q, *J* = 7.1 Hz, 2H), 3.97 (ddd, *J* = 10.3, 5.5, 2.0 Hz, 1H), 3.89 (t, *J* = 10.1 Hz, 1H), 3.77 (dd, *J* = 10.0, 5.6 Hz, 1H), 3.42-3.36 (m, 2H), 2.97 (d, *J* = 8.8 Hz, 1H), 2.67 (ddd, *J* = 11.2, 7.2, 3.7 Hz, 1H), 2.38 (dd, *J* = 11.9, 3.5 Hz, 1H), 1.25 (t, *J* = 7.1 Hz, 3H), 1.13-0.94 (m, 28H), 0.87 (s, 9H). **^{13}C NMR** (126 MHz, $CDCl_3$) δ 207.07, 171.57, 101.02, 79.30, 76.33, 69.48, 61.32, 59.50, 47.05, 37.19, 32.00, 26.63, 17.49, 17.41, 17.39, 17.26, 17.23, 17.21, 17.19, 14.27, 13.34, 13.11, 12.69, 12.62. **ESI-HRMS**: Calculated for $C_{27}H_{53}O_8Si_2$ $(M+H)^+$: 561.32735, Found: 561.32829. $[\alpha]_D^{20}$ = +45.6 (c = 1.0, CH_2Cl_2).

Ethyl (6a*R*,8*S*,9*R*)-8-isopropoxy-2,2,4,4-tetraisopropyl-11-oxohexahydro-6H-oxepino[3,2-f][1,3,5,2,4]trioxadisilocine-9-carboxylate (3bd)

The title product compound is prepared according to the general procedure **B** with 2 mol% catalyst **A** and 0.2 mmol cyclopropanated donor at rt for 36 h and isolated by flash column chromatography (15:1 Pentane: Ethyl Acetate) giving **3bd** as an oil (92 mg, 86% yield, α/β ratio > 20:1).

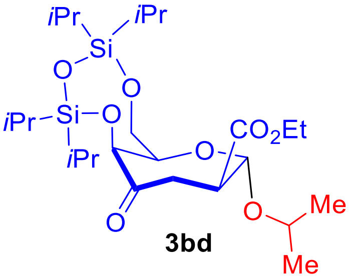

3bd

^{1}H NMR (500 MHz, CD_2Cl_2) δ 5.32 (d, *J* = 7.3 Hz, 1H), 4.34 (s, 1H), 4.14 (qd, *J* = 7.1, 4.5 Hz, 2H), 4.01 (ddd, *J* = 10.3, 5.6, 2.1 Hz, 1H), 3.90-3.83 (m, 2H), 3.76 (dd, *J* = 10.1, 5.6 Hz, 1H), 3.33 (t, *J* = 11.8 Hz, 1H), 2.57-2.53 (m, 1H), 2.35 (ddd, *J* = 11.9, 3.5, 1.2 Hz, 1H), 1.25 (t, *J* = 7.2 Hz, 3H), 1.16 (d, *J* = 6.3 Hz, 3H), 1.13-1.02 (m, 27H), 0.97-0.94 (m, 4H). **^{13}C NMR** (126 MHz, CD_2Cl_2) δ 206.92, 171.53, 98.25, 76.59, 69.75, 69.57, 61.55, 59.84, 47.82, 37.66, 23.50, 21.14, 17.53, 17.47, 17.45, 17.31, 17.29, 17.27, 17.24, 14.34, 13.63, 13.39, 13.02, 12.91. **ESI-HRMS**: Calculated for $C_{25}H_{48}O_8NaSi_2$ $(M+Na)^+$: 555.27799, Found: 555.27743. $[\alpha]_D^{20}$ = +35.5 (c = 1.0, CH_2Cl_2).

Ethyl (6a*R*,8*R*,9*R*)-2,2,4,4-tetraisopropyl-8-(4-methoxyphenoxy)-11-oxohexahydro-6H-oxepino[3,2-f][1,3,5,2,4]trioxadisilocine-9-carboxylate (3be)

The title product compound is prepared according to the general procedure **B** with 2 mol% catalyst **A** and 0.2 mmol cyclopropanated donor at rt for 5 h and isolated by flash column chromatography (10:1 Pentane: Ethyl Acetate) giving **3be** as an oil (87 mg, 73% yield, α/β ratio > 20:1).

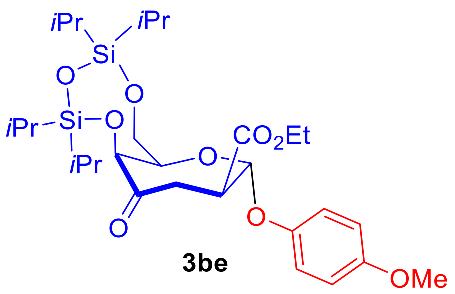

3be

^{1}H NMR (500 MHz, $CDCl_3$) δ 6.98-6.96 (m, 2H), 6.82-6.80 (m, 2H), 5.86 (d, *J* = 7.1 Hz, 1H), 4.37 (s, 1H), 4.20 (q, *J* = 7.1 Hz, 2H), 4.05-4.01 (m, 1H), 3.76 (s, 3H), 3.61 (t, *J* = 10.4 Hz, 1H), 3.48-3.42 (m, 2H), 2.91 (ddt, *J* = 11.2, 7.2, 3.9 Hz, 1H), 2.51 (dd, *J* = 12.1, 4.0 Hz, 2H), 1.27 (t, *J* = 7.1 Hz, 3H), 1.12 (dd, *J* = 11.0, 6.7 Hz, 6H), 1.03-0.94 (m, 22H). **^{13}C NMR** (126 MHz, $CDCl_3$) δ 206.73, 171.20, 155.72, 150.50, 119.54, 114.55, 100.47, 76.41, 70.18, 61.60, 59.07, 55.71, 46.93, 37.17, 17.40, 17.38, 17.32, 17.19, 17.17, 17.14, 14.27, 13.21, 13.03, 12.58. **ESI-HRMS**: Calculated for $C_{29}H_{49}O_9Si_2$ $(M+H)^+$: 597.29096, Found: 597.29194. $[\alpha]_D^{20}$ = +49.8 (c = 1.0, CH_2Cl_2).

Ethyl (6a*R*,8*R*,9*R*)-2,2,4,4-tetraisopropyl-8-(naphthalen-1-yloxy)-11-oxohexahydro-6H-oxepino[3,2-f][1,3,5,2,4]trioxadisilocine-9-carboxylate (3bf)

The title product compound is prepared according to the general procedure **B** with 2 mol% catalyst **A** and 0.2 mmol cyclopropanated donor at rt for 12 h and isolated by flash column chromatography (10:1 Pentane: Ethyl Acetate) giving **3bf** as an oil (94 mg, 76% yield, α/β ratio > 20:1).

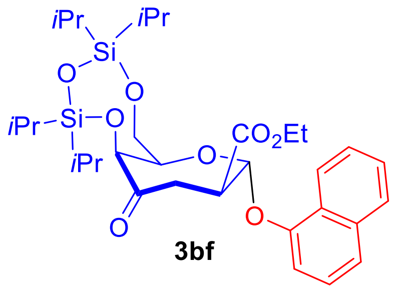

3bf

^{1}H NMR (700 MHz, $CDCl_3$) δ 8.17-8.16 (m, 1H), 7.81-7.80 (m, 1H), 7.55-7.53 (m, 1H), 7.50-7.48 (m, 2H), 7.38 (t, *J* = 7.9 Hz, 1H), 7.20 (d, *J* = 7.6 Hz, 1H), 6.18 (d, *J* = 6.7 Hz, 1H), 4.38 (s, 1H), 4.20 (qd, *J* = 7.2, 1.6 Hz, 2H), 4.05 (ddd, *J* = 10.2, 5.6, 1.8 Hz, 1H), 3.64 (t, *J* = 10.3 Hz, 1H), 3.59 (dd, *J* = 12.5, 10.6 Hz, 1H), 3.37-3.35 (m, 1H), 3.17 (ddd, *J* = 10.7, 6.7, 4.1 Hz, 1H), 2.65-2.63 (m, 1H), 1.25 (t, *J* = 7.1 Hz, 3H), 1.16-1.13 (m, 6H), 1.05-0.93 (m, 22H). **^{13}C NMR** (176 MHz, $CDCl_3$) δ 206.58, 171.08, 152.22, 134.68, 127.67, 126.59, 126.19, 125.83, 125.75, 122.77, 122.09, 110.07, 99.41, 76.31, 69.90, 61.74, 59.05, 46.78, 37.28, 17.43, 17.40, 17.37, 17.29, 17.24, 17.19, 17.18, 17.15, 14.24, 13.20, 13.14, 12.70, 12.60. **ESI-HRMS**: Calculated for $C_{32}H_{49}O_8Si_2$ $(M+H)^+$: 617.29605, Found: 617.29771. $[\alpha]_D^{20}$ = +40.2 (c = 1.0, CH_2Cl_2).

Ethyl (6a*R*,8*R*,9*S*)-2,2,4,4-tetraisopropyl-8-((4-methoxyphenyl)thio)-11-oxohexahydro-6H-oxepino[3,2-f][1,3,5,2,4]trioxadisilocine-9-carboxylate (3bg)

The title product compound is prepared according to the general procedure **B** with 2 mol% catalyst **A** and 0.2 mmol cyclopropanated donor at rt for 12 h and isolated by flash column chromatography (15:1 Pentane: Ethyl Acetate) giving **3bg** as an oil (75 mg, 61% yield, α/β ratio > 20:1).

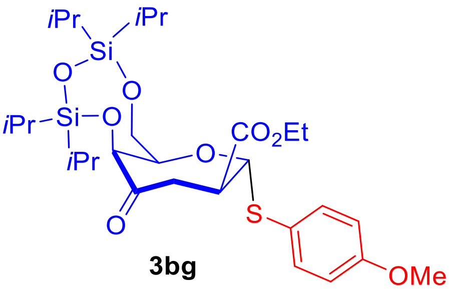

3bg

^{1}H NMR (700 MHz, $CDCl_3$) δ 7.43-7.41 (m, 2H), 6.86-6.85 (m, 2H), 5.65 (d, *J* = 9.8 Hz, 1H), 4.41 (d, *J* = 3.3 Hz, 1H), 4.24 (q, *J* = 7.1 Hz, 2H), 4.00 (ddd, *J* = 10.5, 5.4, 2.8 Hz, 1H), 3.80 (s, 3H), 3.59 (t, *J* = 11.5 Hz, 1H), 3.36 (t, *J* = 10.4 Hz, 1H), 3.31 (dd, *J* = 10.4, 5.5 Hz, 1H), 2.78 (ddd, *J* = 11.3, 9.7, 4.7 Hz, 1H), 2.44 (ddd, *J* = 11.5, 4.7, 1.0 Hz, 1H), 1.33 (t, *J* = 7.2 Hz, 3H), 1.12 (dd, *J* = 15.8, 7.3 Hz, 6H), 1.05-0.86 (m, 22H). **^{13}C NMR** (176 MHz, $CDCl_3$) δ 207.44, 171.32, 160.38, 136.28, 123.47, 114.68, 87.53, 72.86, 61.68, 58.77, 55.39, 48.27, 39.15, 17.47, 17.45, 17.35, 17.33, 17.26, 17.21, 17.19, 17.18, 14.28, 13.18, 12.83, 12.56, 12.51 **ESI-HRMS**: Calculated for $C_{29}H_{49}O_8Si_2S$ $(M+H)^+$: 613.26895, Found: 613.26930. $[\alpha]_D^{20}$ = +54.3 (c = 1.0, CH_2Cl_2).

(6aR,8S,9R,11aR)-9-(((tert-butyldimethylsilyl)oxy)methyl)-2,2,4,4-tetraisopropyl-8-(((3aR,5R,5aS,8aS,8bR)-2,2,7,7-tetramethyltetrahydro-5H-bis([1,3]dioxolo)[4,5-b:4',5'-d]pyran-5-yl)methoxy)tetrahydro-6H-oxepino[3,2-f][1,3,5,2,4]trioxadisilocin-11(8H)-one (3bh)

The title product compound is prepared according to the general procedure **B** with 2 mol% catalyst **A** and 0.2 mmol cyclopropanated donor at rt for 2 h and isolated by flash column chromatography (15:1 Pentane: Ethyl Acetate) giving **3bh** as an oil (27 mg, 17% yield).

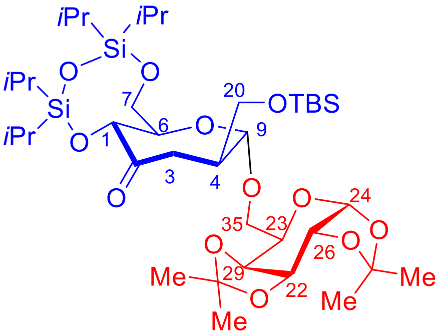

3bh

^{1}H NMR (700 MHz, $CDCl_3$) 5.47 (d, *J* = 5.0 Hz, 1H, H-24), 4.78 (d, *J* = 8.1 Hz, 1H, H-9), 4.60 (dd, *J* = 7.9, 2.5 Hz, 1H, H-22), 4.41 (d, *J* = 10.0 Hz, 1H, H-1), 4.31 (dd, *J* = 5.1, 2.5 Hz, 1H, H-26), 4.22 (dd, *J* = 7.9, 1.9 Hz, 1H, H-29), 4.10 (dd, *J* = 11.8, 2.0 Hz, 1H, H-7), 3.93 – 3.90 (m, 2H, H-7 & H-6), 3.87 – 3.85 (m, 2H, H-6 & H-35), 3.71 – 3.66 (m, 2H, H-35 & H-20), 3.60 (dd, *J* = 10.0, 4.9 Hz, 1H, H-20), 2.66 (t, *J* = 12.3 Hz, 1H, H-3), 2.35 (dd, *J* = 12.7, 1.3 Hz, 1H, H-3), 1.87 – 1.83 (m, 1H, H-4), 1.49 (s, 3H), 1.42 (s, 3H), 1.34 – 1.30 (m, 6H), 1.11 – 1.02 (m, 28H), 0.89 (s, 9H), 0.05 (d, *J* = 2.5 Hz, 6H). **^{13}C NMR** (176 MHz, $CDCl_3$) δ 207.92, 109.63, 108.88, 101.90, 96.77, 75.04, 71.49, 71.15, 71.03, 69.64, 67.25, 66.77, 64.36, 63.70, 41.82, 40.83, 26.29, 26.22, 26.02, 25.10, 24.78, 18.51, 17.56, 17.54, 17.48, 17.41, 17.34, 17.31, 17.20, 13.73, 13.63, 13.11, 12.74, -5.37, -5.38. **ESI-HRMS**: Calculated for $C_{38}H_{73}O_{12}Si_3$ $(M+H)^+$: 805.4404, Found: 805.4427. $[\alpha]_D^{20}$ = -2.3 (c = 1.0, CH_2Cl_2).

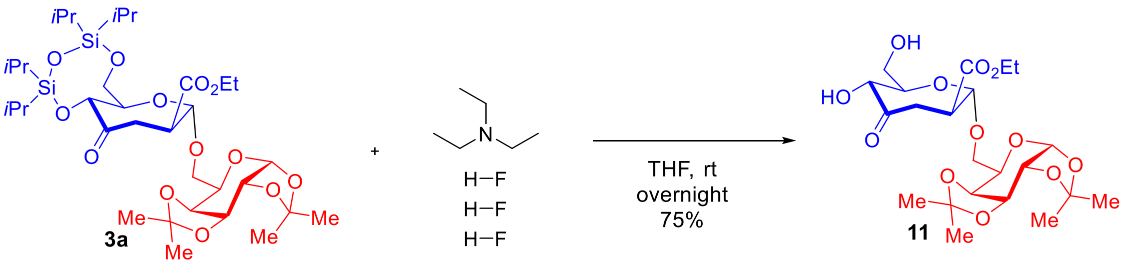

To a solution of **3a** (240 mg, 0.327 mmol) in 10 mL THF was added **$Et_3N·3HF$** (158 mg, 0.982 mmol). The reaction mixture was stirred at room temperature under Ar for overnight. Then the reaction mixture was concentrated in vacuo. The residue was purified through column chromatography to give **11** in 75% yield (121 mg, 0.247 mmol) as a colorless oil.

^{1}H NMR (500 MHz, CD_2Cl_2) δ 5.47 (d, *J* = 5.0 Hz, 1H), 5.26 (d, *J* = 6.5 Hz, 1H), 4.59 (dd, *J* = 7.9, 2.5 Hz, 1H), 4.31 (dd, *J* = 5.0, 2.4 Hz, 1H), 4.19-4.17 (m, 3H), 4.12 (d, *J* = 9.9 Hz, 1H), 3.95-3.88 (m, 3H), 3.81-3.70 (m, 4H), 2.94 (dd, *J* = 14.2, 10.0 Hz, 1H), 2.81 (ddd, *J* = 10.0, 6.6, 1.9 Hz, 1H), 2.73 (dd, *J* = 14.2, 1.9 Hz, 1H), 1.49 (s, 3H), 1.41 (s, 3H), 1.31 (s, 6H), 1.26 (t, *J* = 7.1 Hz, 3H). **^{13}C NMR** (126 MHz, CD_2Cl_2) δ 208.98, 171.12, 109.69, 108.99, 100.39, 96.63, 76.82, 71.44, 71.07, 70.86, 69.53, 67.75, 66.97, 64.00, 62.01, 45.41, 37.60, 26.14, 25.04, 24.59, 14.26. **ESI-HRMS**: Calculated for $C_{22}H_{35}O_{12}$ $(M+H)^+$: 491.21230, Found: 491.21269. $[\alpha]_D^{20}$ = -15.5 (c = 2.0, CH_2Cl_2).

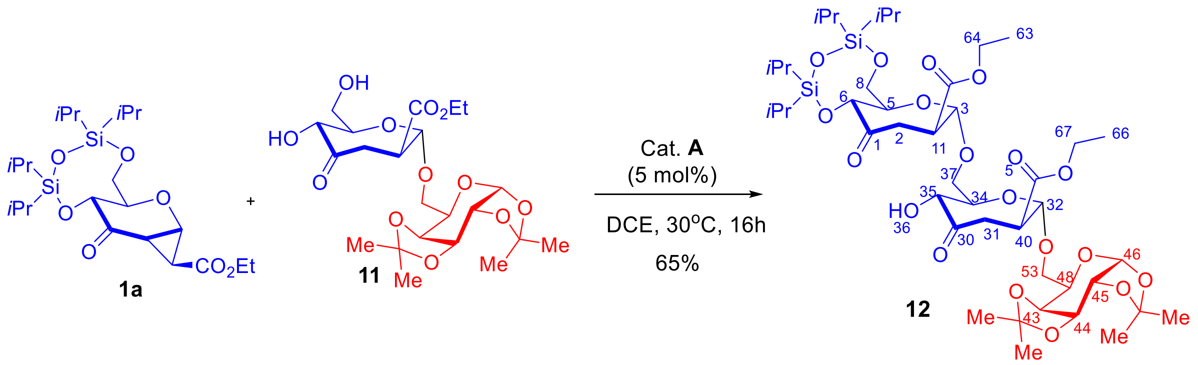

A mixture of catalyst **A** (5 mol%), glycosyl acceptor **1a** (0.1 mmol), glycal **11** (0.15 mmol) was dissolved in anhydrous DCE (0.6 mL) and sealed in a dry tube under argon. The mixture was stirred at 30 ˚C for 16 h. Afterwards, the solvent was removed under reduced pressure and the residue was analyzed by crude ^{1}H NMR and then subjected to flash column chromatography to give **12** in 65% yield (63 mg, 0.65 mmol, α/β ratio > 20:1) as a colorless oil.

^{1}H NMR (700 MHz, CD_2Cl_2) δ 5.47 (d, *J* = 5.0 Hz, 1H, H-46), 5.29 (d, *J* = 6.6 Hz, 1H, H-3), 5.21 (d, *J* = 6.8 Hz, 1H, H-32), 4.58 (dd, *J* = 7.9, 2.5 Hz, 1H, H-44), 4.43 (d, *J* = 9.8 Hz, 1H, H-6), 4.30 (dd, *J* = 5.0, 2.4 Hz, 1H, H-45), 4.18-4.14 (m, 6H, H-43, H-64, H-67, H-35), 4.10 (d, *J* = 11.9 Hz, 1H, H-8), 4.05 (dd, *J* = 10.6, 4.4 Hz, 1H, H-37), 3.97-3.92 (m, 2H, H-8, H-5), 3.91-3.89 (m, 1H, H-48), 3.88-3.84 (m, 3H, H-37, H-53, H-34), 3.69 (dd, *J* = 10.4, 4.7 Hz, 1H, H-53), 3.62 (d, *J* = 3.2 Hz, 1H, H-36), 2.94 (dd, *J* = 13.9, 10.6 Hz, 1H, H-31*ax*), 2.89-2.86 (m, 1H, H-11), 2.81 (dd, *J* = 13.9, 10.3 Hz, 1H, H-2*ax*), 2.76-2.73 (m, 1H, H-40), 2.69 (d, *J* = 14.6 Hz, 1H, H-31*eq*), 2.60 (d, *J* = 13.7 Hz, 1H, H-2*eq*), 1.48 (s, 3H), 1.41 (s, 3H), 1.31 (d, *J* = 6.3 Hz, 6H), 1.26 (t, *J* = 6.4 Hz, 6H), 1.09-1.00 (m, 28H). **^{13}C NMR** (176 MHz, CD_2Cl_2) δ 209.06, 206.02, 171.54, 171.16, 109.75, 108.86, 100.90, 100.48, 96.70, 75.86, 75.34, 71.58, 71.14, 70.93, 69.99, 68.93, 68.56, 67.64, 66.83, 63.51, 61.97, 61.82, 45.65, 45.29, 39.03, 37.96, 26.23, 26.20, 25.09, 24.58, 17.54, 17.52, 17.49, 17.44, 17.36, 17.33, 17.29, 17.18, 14.32, 14.29, 13.65, 13.01, 12.78. **ESI-HRMS**: Calculated for $C_{44}H_{74}O_{19}Si_2Na$ $(M+Na)^+$: 985.42550, Found: 985.42543. $[\alpha]_D^{20}$ = +3.8 (c = 1.0, CH_2Cl_2).

NMR data for control experiment

Control experiment on ester-truncated donor 4 (Related to Figure 3a in the manuscript)

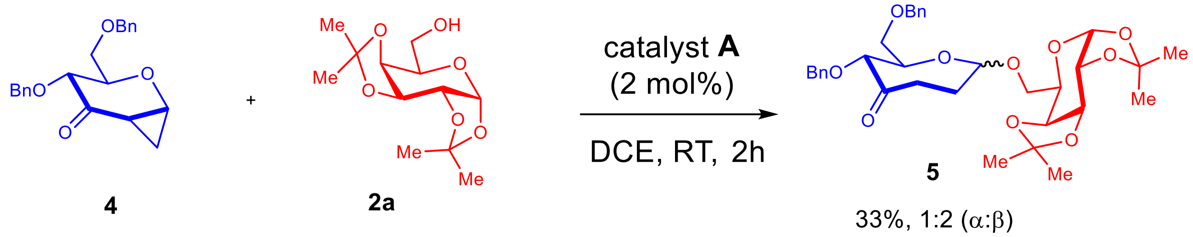

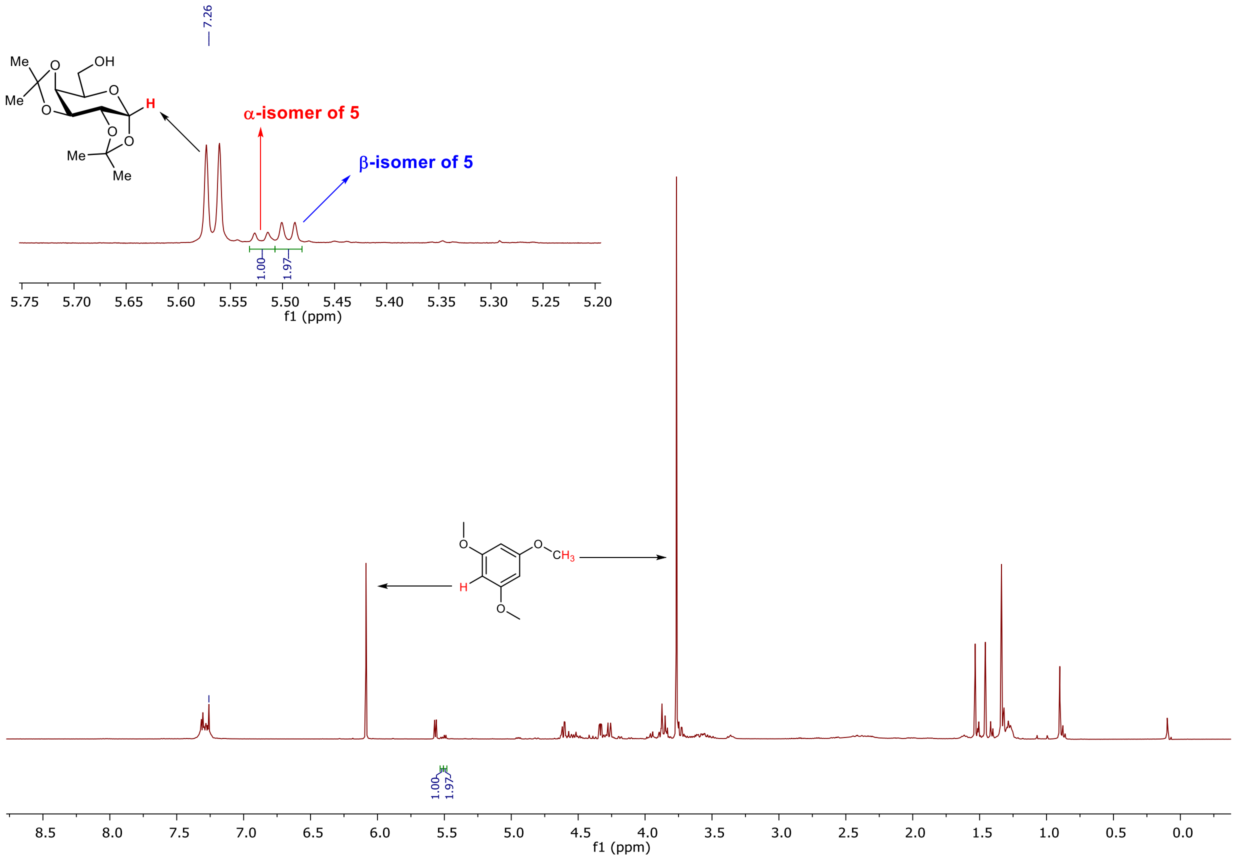

Supplementary Figure 2: ^{1}H NMR spectra for **compound 5** using **A** as catalyst

Control experiment on OTBS donor 1e

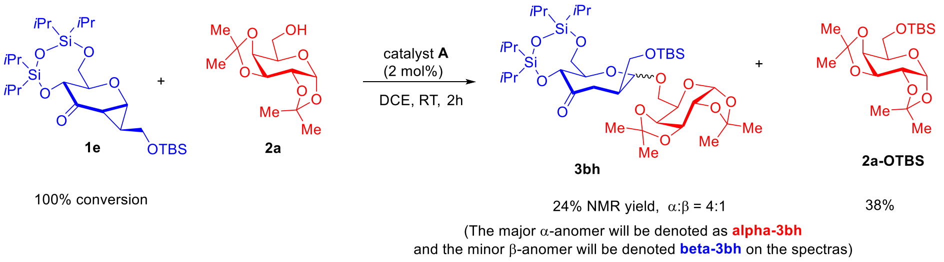

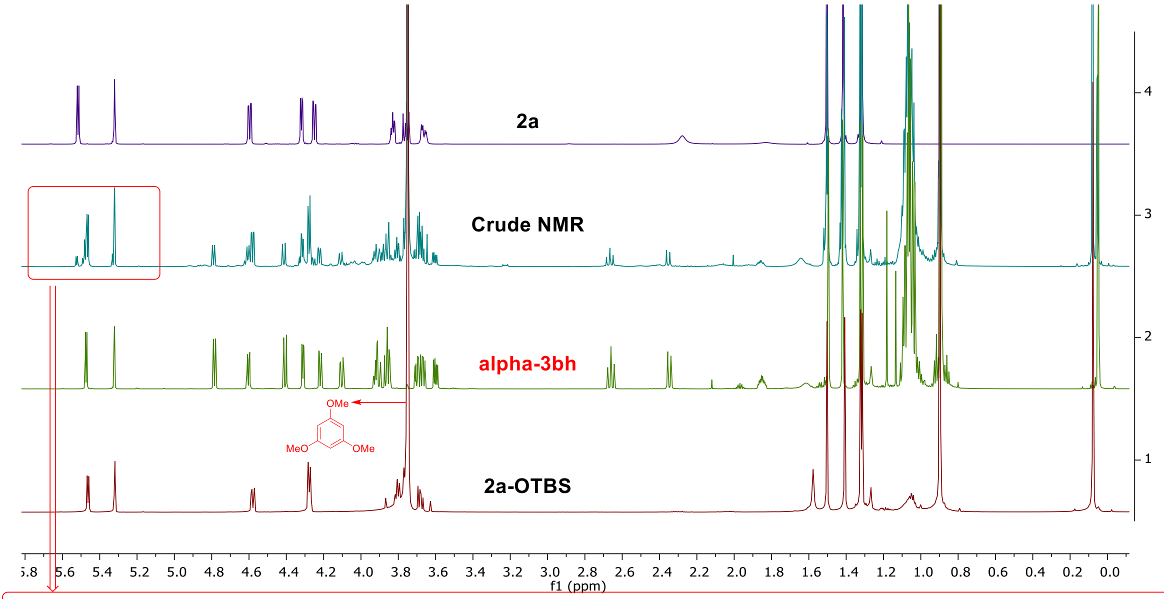

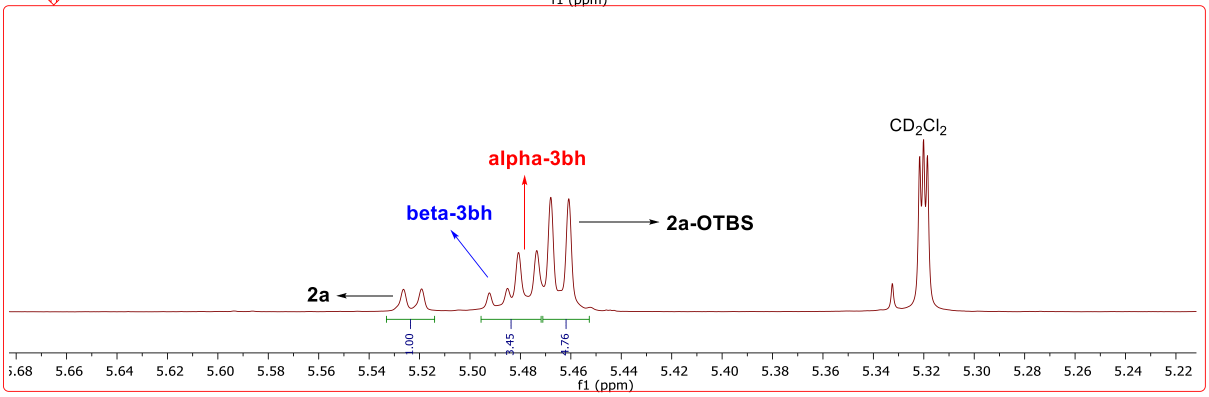

Zoomed-in graph of the chemical shift from 5.68 to 5.22.

Supplementary Figure 3-1: Crude ^{1}H NMR spectra for **compound 3bh**

Upon analysis of the crude NMR spectrum, an excess of compound **2a** was observed, along with the desired product **3bh** and the **2a-OTBS**. The ratios of these three compounds were found to be 1.0 : 3.4 : 4.8**.**

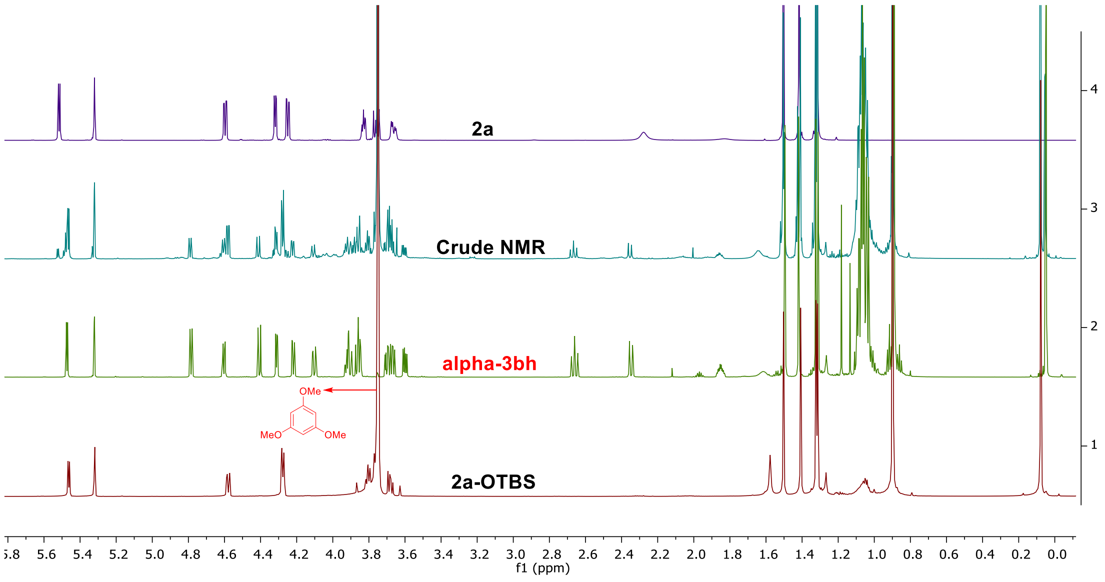

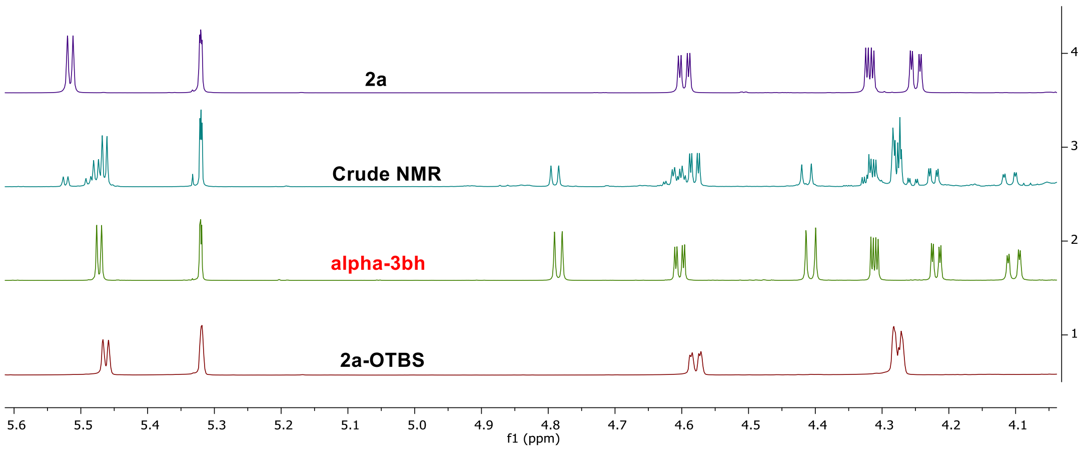

Zoomed-in graph of the chemical shift from 5.6 to 4.0.

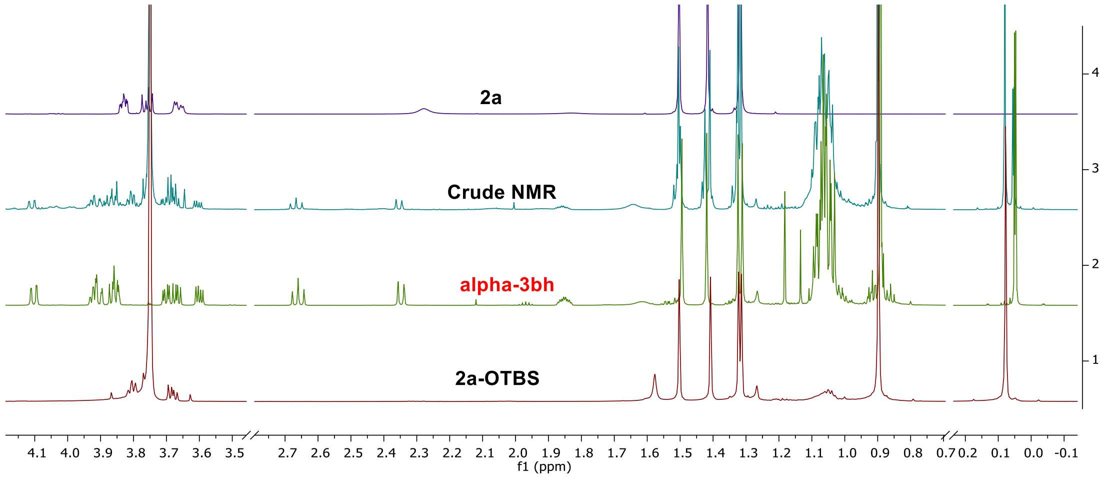

Zoomed-in graph of the chemical shift from 4.1 to 0.

Supplementary Figure 3-2: Crude ^{1}H NMR spectra for **compound 3bh**

Red ● represents alpha-3bh, while blue ★ represents the beta-3bh

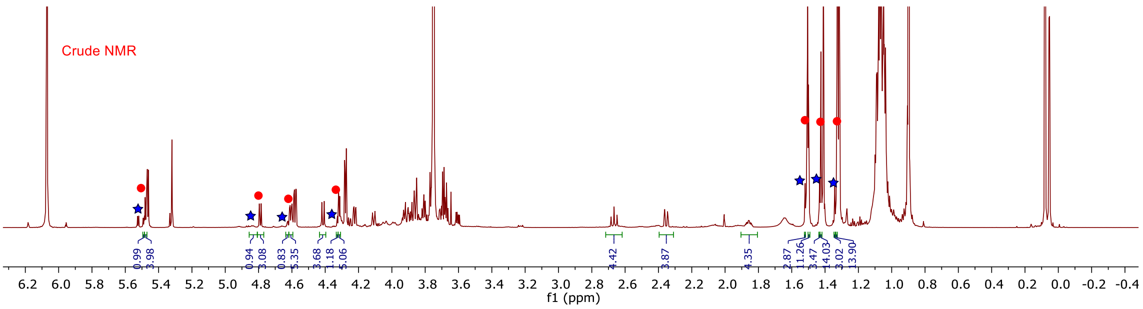

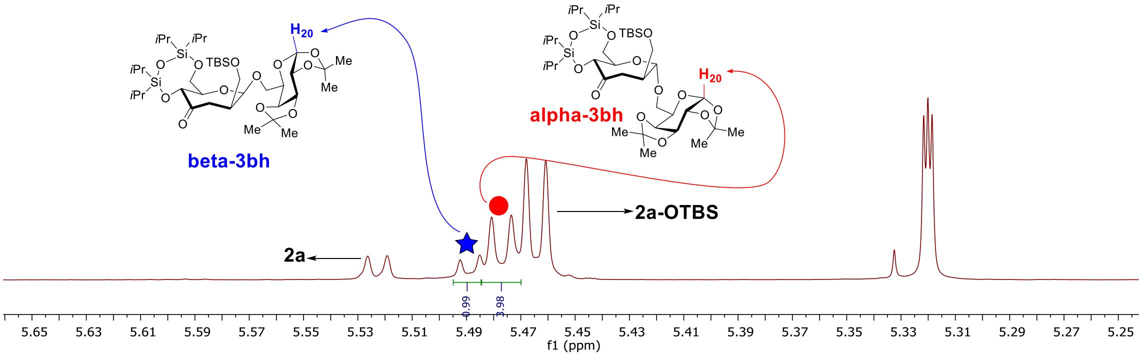

Zoomed-in graph of the chemical shift from 5.65 to 5.25.

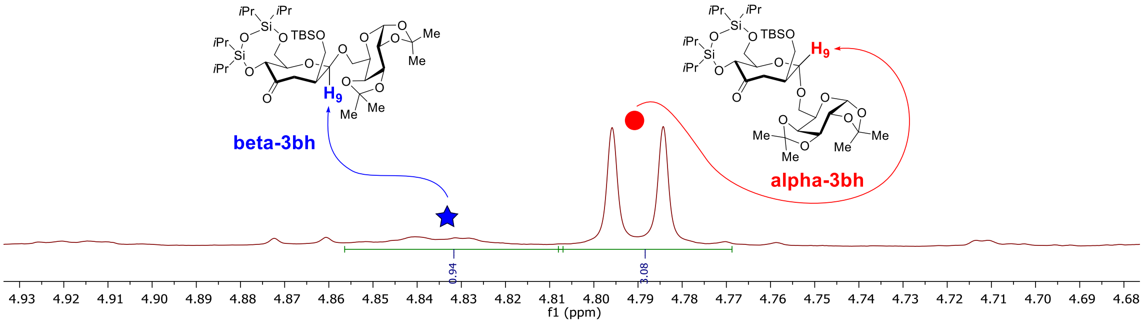

Zoomed-in graph of the chemical shift from 4.93 to 4.68.

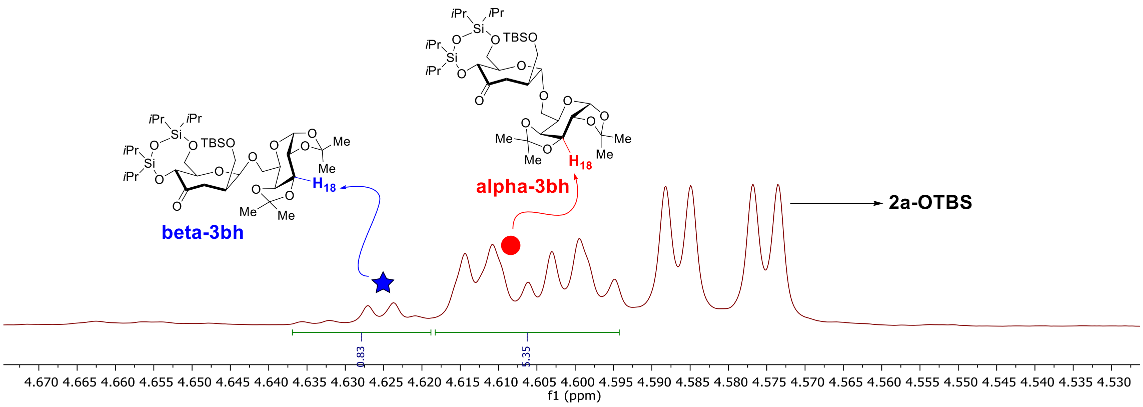

Zoomed-in graph of the chemical shift from 4.67 to 4.53.

Supplementary Figure 3-3: Crude ^{1}H NMR spectra for **compound 3bh**

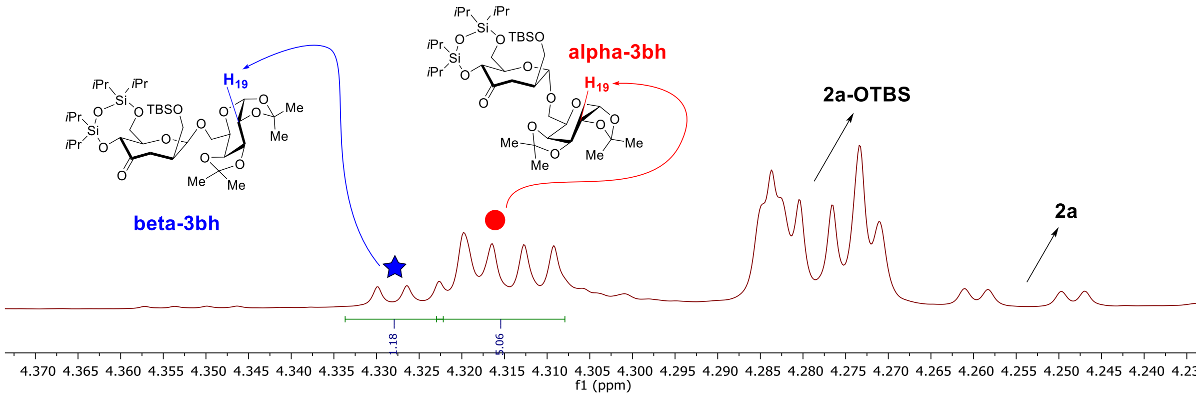

Zoomed-in graph of the chemical shift from 4.37 to 4.23.

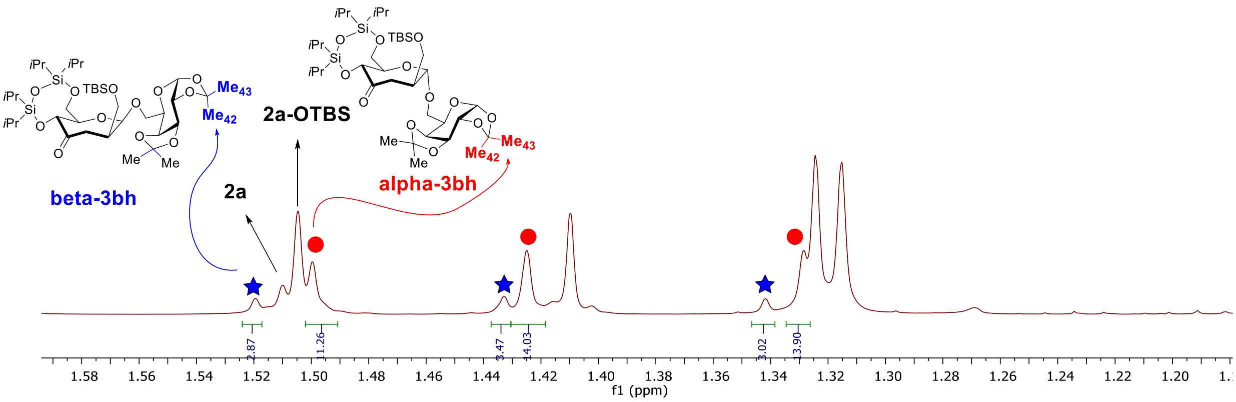

Zoomed-in graph of the chemical shift from 1.58 to 1.20.

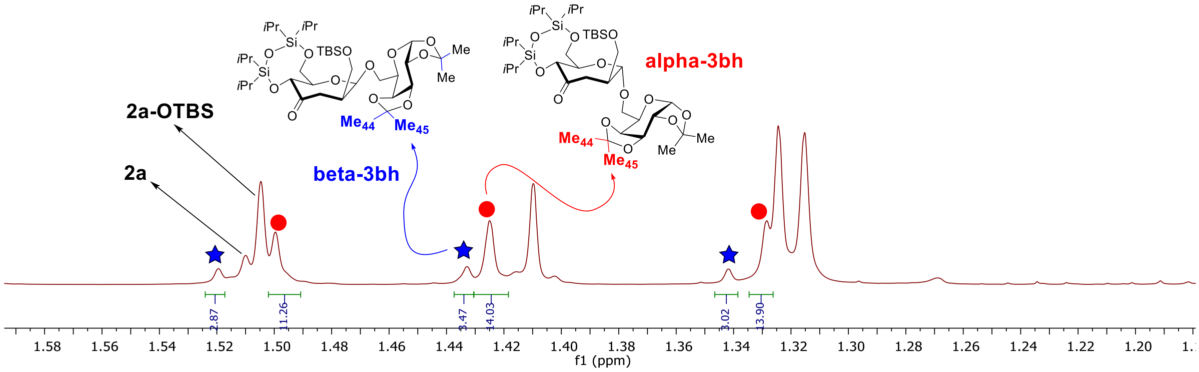

Zoomed-in graph of the chemical shift from 1.58 to 1.20.

Supplementary Figure 3-4: Crude ^{1}H NMR spectra for **compound 3bh**

In this control experiment with substrate **1e**, we are able to detect both the alpha and beta septanoside with a 4:1 ratio in ^{1}H crude NMR. Despite our best efforts, we were only able to isolate the alpha product of **3bh** after flash column chromatography but not the beta product. We surmise that the beta product is likely unstable to the purification conditions due to its sterically unfavorable 1,2-*cis* conformation. However, we are able to observe multiple discernable peaks of the beta product with consistent ratios to the alpha anomer over multiple different chemical shift regions in the crude ^{1}H NMR to give us confidence in the spectra interpretation.

1) The chemical shift signals in the 5.55-5.45 ppm range are assigned to H-20 (**Supplementary Figure 3-3**, top spectra). We can clearly observe four sets of signals. By comparing the spectra, we could easily assign the resonance at 5.44 ppm to **2a** and the resonance at 5.38 ppm to **2a-OTBS** (side product of reaction due to TBS migration). Additionally, we observed two sets of

doublets of the two septanoside anomers in an approximate 1:4 ratio. Since we isolated the major product alpha-**3bh**, we are able to distinguish the anomers in this region.

2) We are able to locate the anomeric proton peaks of the sepatanoside anomers H-9 in the 4.85-4.77 ppm region (**Supplementary Figure 3-3**, second spectra from bottom), where the diagnostic lower field beta anomeric proton of the septanoside[12] can be clearly distinguished.
3) The chemical shift signals in the 4.64-4.56 ppm range are assigned to H-18 (**Supplementary Figure 3-3**, bottom spectra). Similarly, the beta-**3bh** signal is observed at a lower field position. We noticed that the integral ratio is approximately 5:1 (alpha:beta), which is due to the presence of overlapping **2a** peaks in the alpha-**3bh** signal region.
4) The chemical shift signals in the 4.34-4.30 ppm range are assigned to H-19 (**Supplementary Figure 3-4**, top spectra). The beta-**3bh** signal is observed at a lower field position, and we can observe two sets of doublets of doublet (dd) peaks of both anomers with some overlap in an approximate ratio of 4:1 (alpha:beta).
5) The chemical shift signals in the 1.54-1.48 ppm range are assigned to one of the methyl groups in either Me-42 or Me-43. In this region, four signals can be observed. Excluding **2a** and **2a-OTBS**, we can observe approximately a 3:12 ratio (~1:4) of beta-**3bh** and alpha-**3bh**.
6) Similar to the previous case, the chemical shift signals in the 1.44-1.40 ppm range are assigned to one of the methyl groups of either Me-44 or Me-45. Here, we can observe approximately a 3:14 ratio (~1:4) of beta and alpha products. Meanwhile, the chemical shift signals in the 1.34-1.30 ppm range are assigned to the remaining isopropylidene methyl group signal in **3bh**.

Using catalyst A on ester containing donor 1c

(Related to Figure 3a in the manuscript)

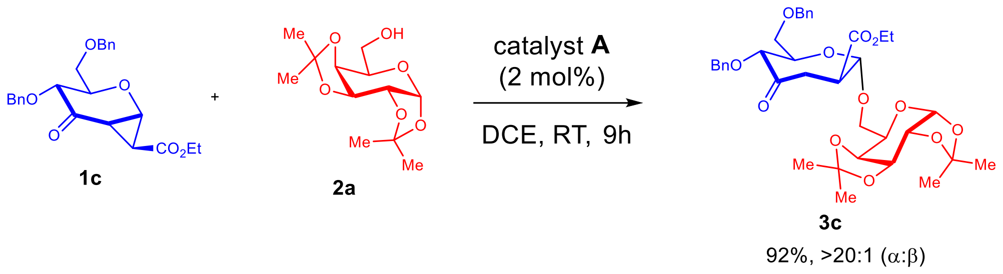

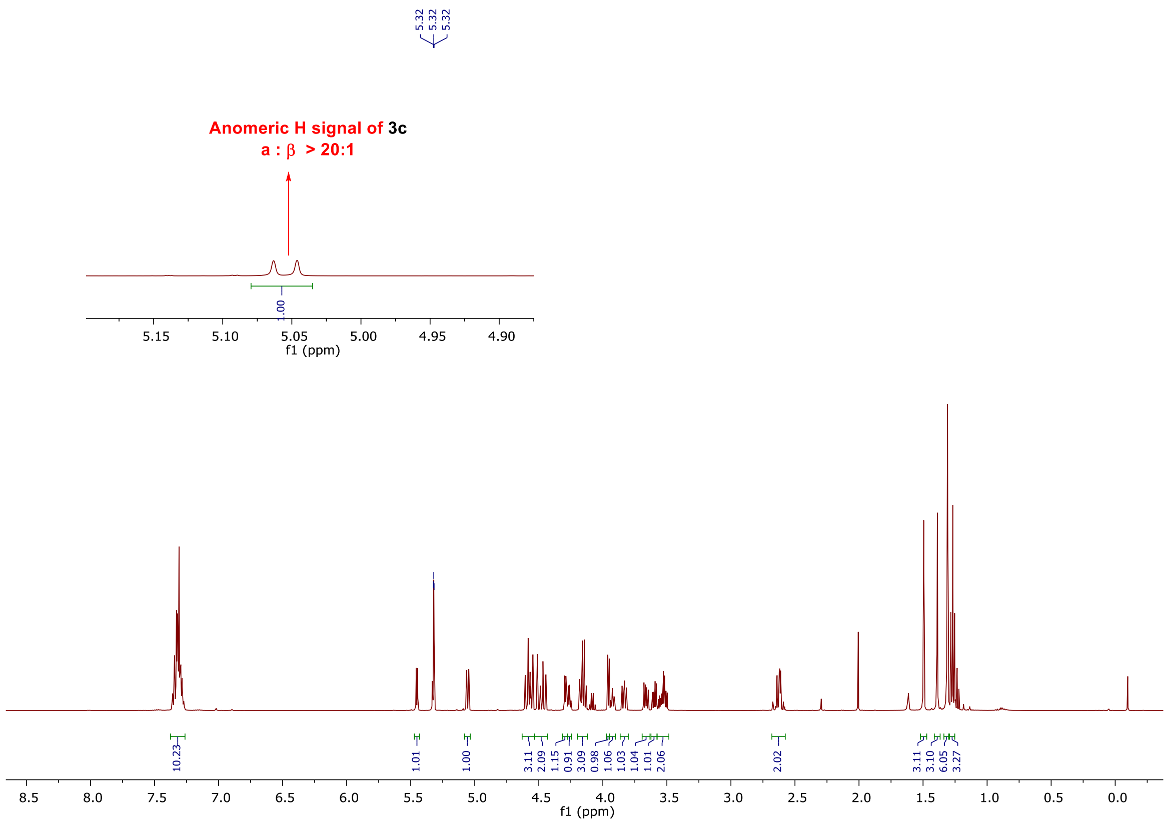

Supplementary Figure 3-5: ^{1}H NMR spectra for **compound 3c** using **A** as catalyst

Competitive experiment between thiophenol and representative secondary alcohol saccharide

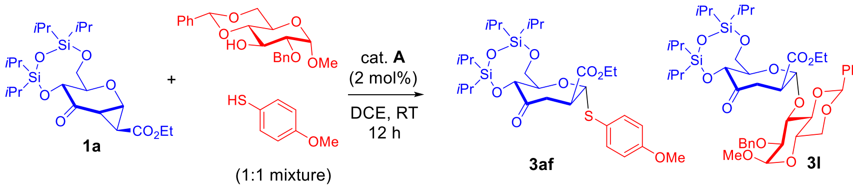

78% combined NMR yield

Ratio of **3af**:**3l** = 5/1 ≈ k_{SH}/k_{OH}

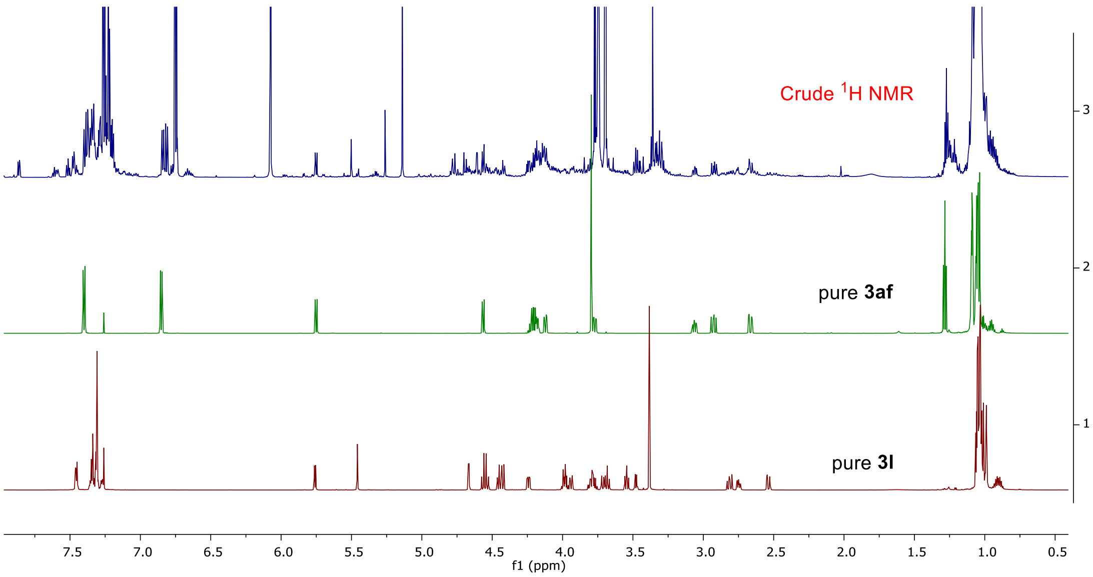

Zoomed in spectra between 5.675-5.810 ppm region of the above spectras

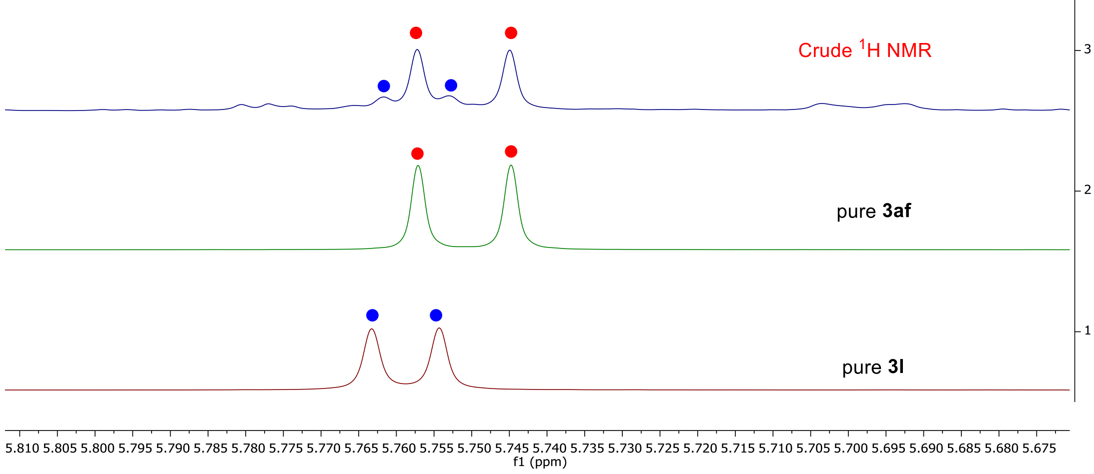

Calculation of 3al to 3al ratio based on integrals:

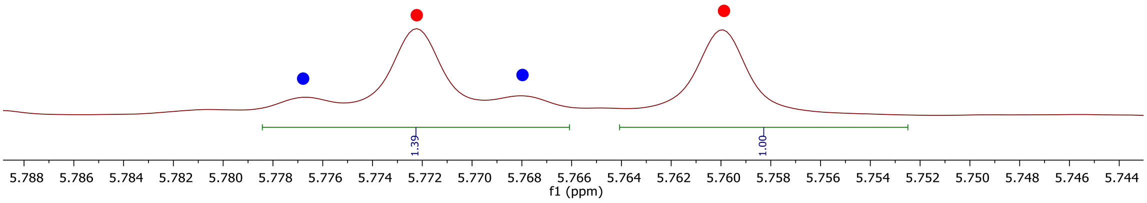

Supplementary Figure 3-6: Competitive experiment between thiophenol and secondary alcohol

Benchmarking our method with previously known TMSOTf promoted method

Using catalyst A

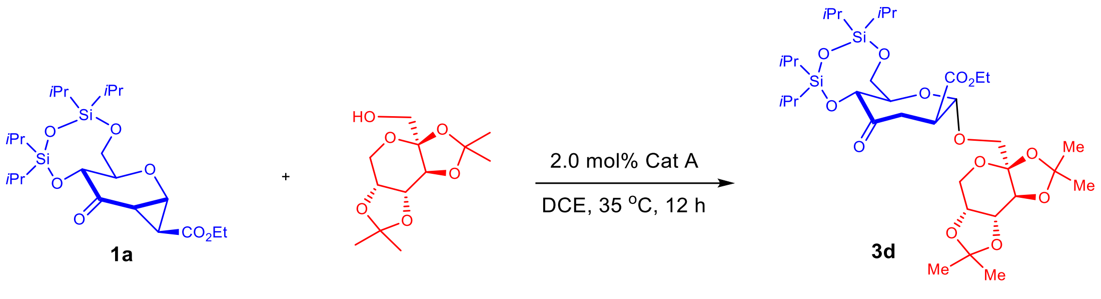

82%, >20:1 (α:β)

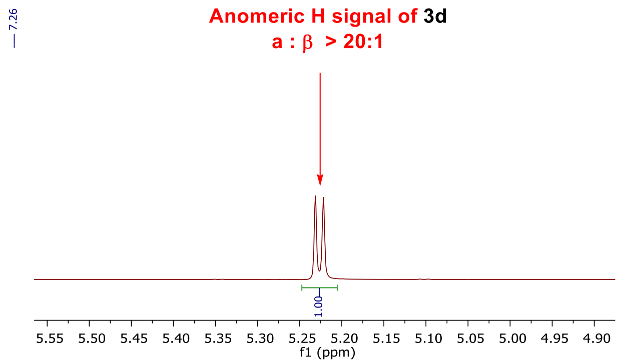

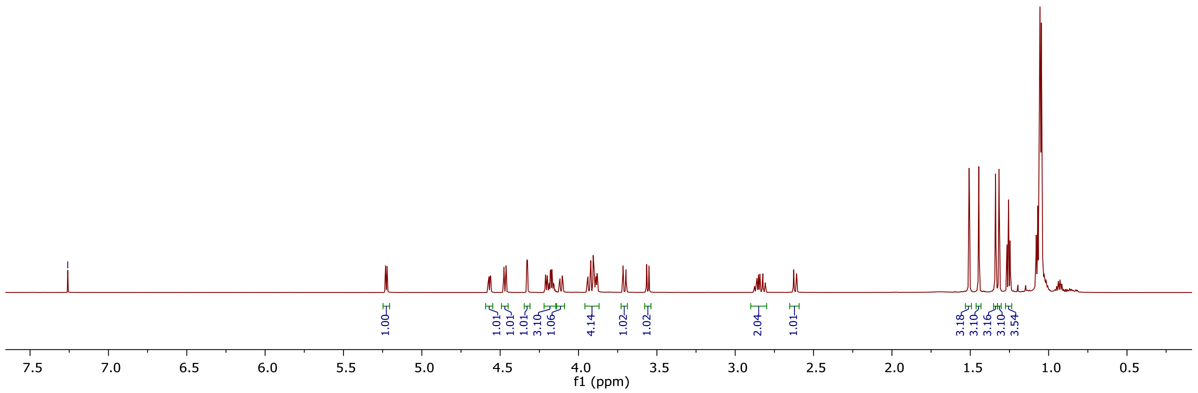

Supplementary Figure 4-1: ^{1}H NMR spectra for **compound 3d** using **A** as catalyst

Using previously known TMSOTf promoted septanosylation method[13]

Use the reference method

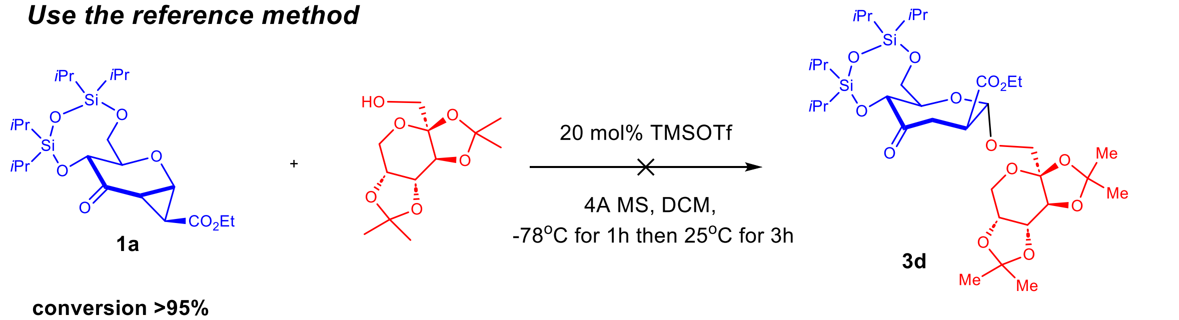

conversion >95%

Crude ^{1}H NMR suggests that the donor 1a was decomposed.

no desired product was detected

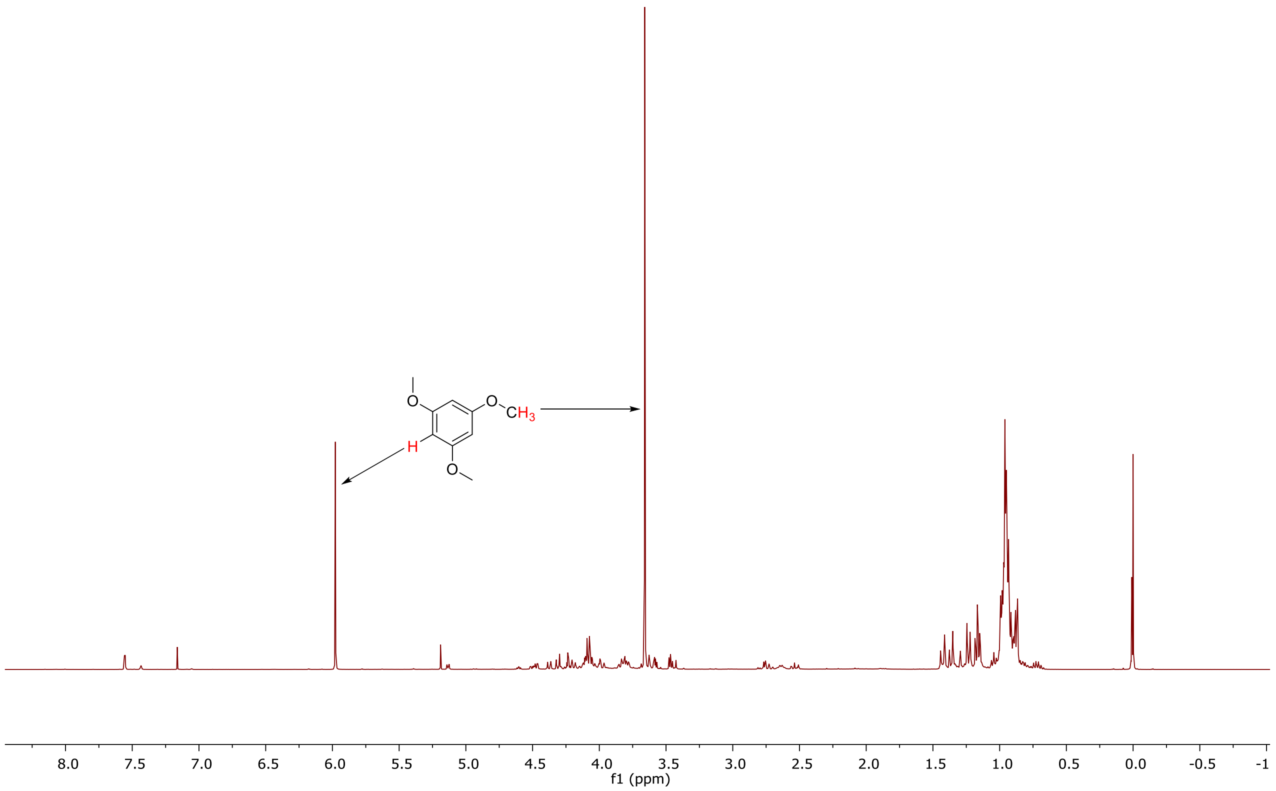

Supplementary Figure 4-2: Crude ^{1}H NMR spectra using **TMSOTf** as catalyst

NMR data for titration experiments

^{77}Se NMR shift on catalyst A with isopropanol

(Related to Figure 2a in the manuscript)

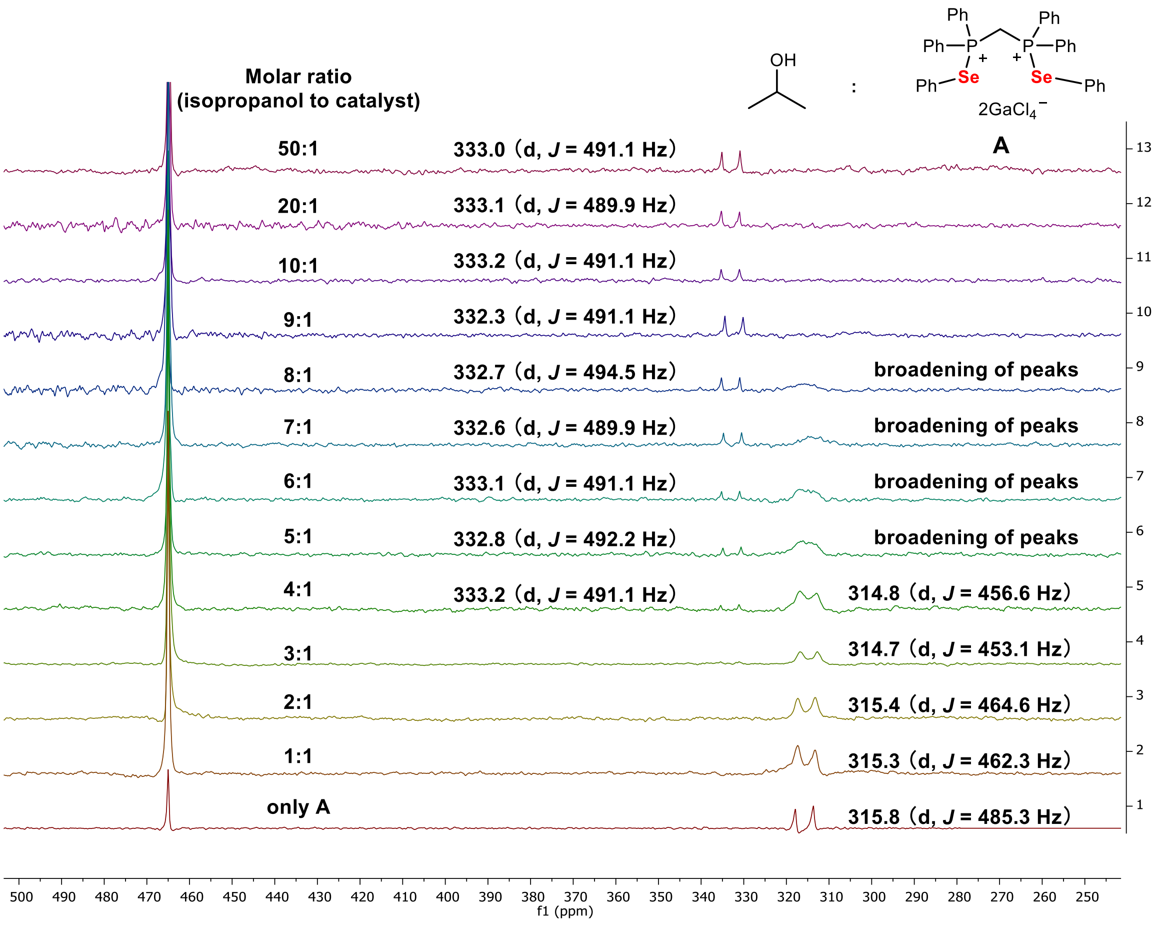

Supplementary Figure 5-1. ^{77}Se titration for catalyst **A** and isopropanol.

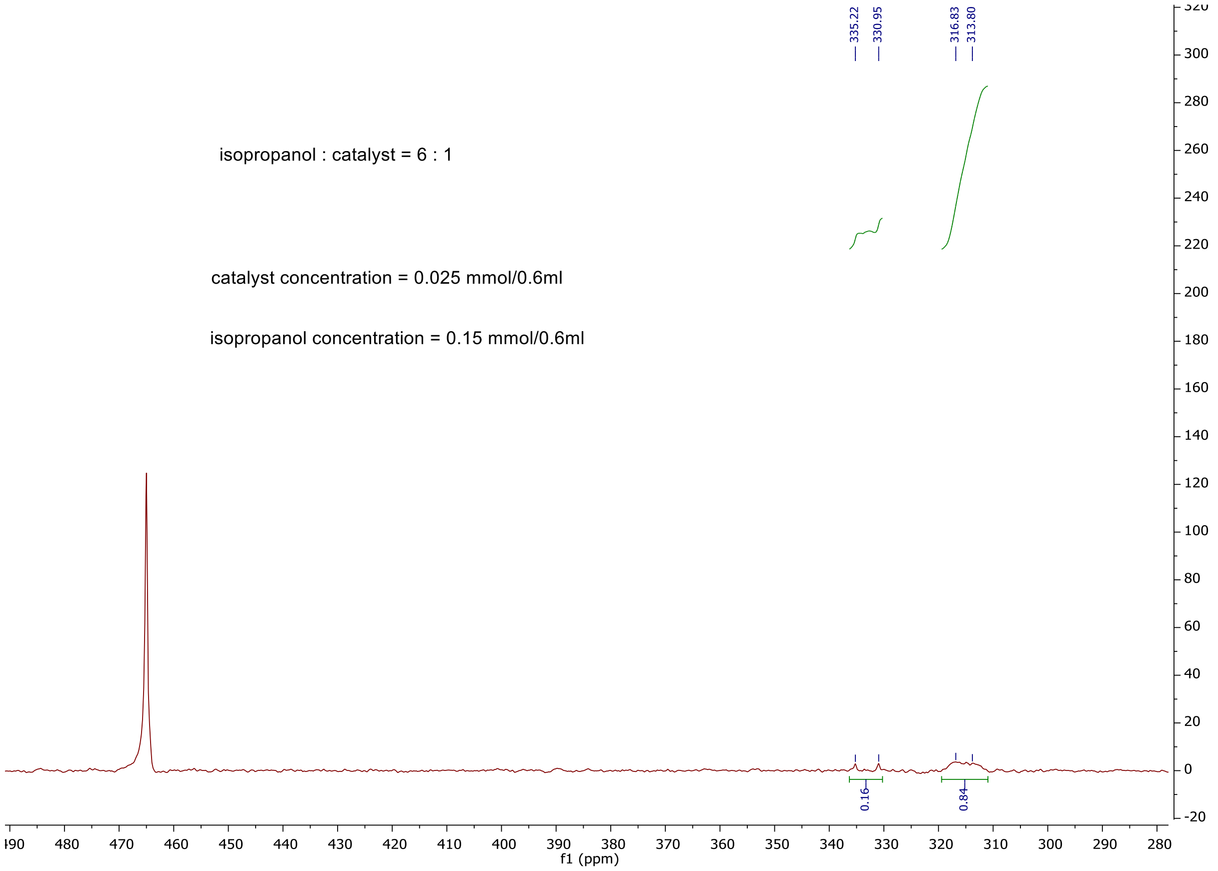

Supplementary Figure 5-2. ^{77}Se titration for catalyst **A** and isopropanol (6:1).

We have opted for a calculation involving isopropanol and the catalyst in a 6:1 ratio, as it offers a higher signal-to-noise ratio, in accordance with the following formula.[14,15,16]

$$K = \frac{[HG]}{[H][G]}$$

$$K = \frac{0.16*0.025}{[0.84*0.025]*[0.15-0.16*0.025]} = 1.3\mathrm{M}^{-1}$$

^{77}Se NMR shift on catalyst A with butanethiol

(Related to Figure 2g in the manuscript)

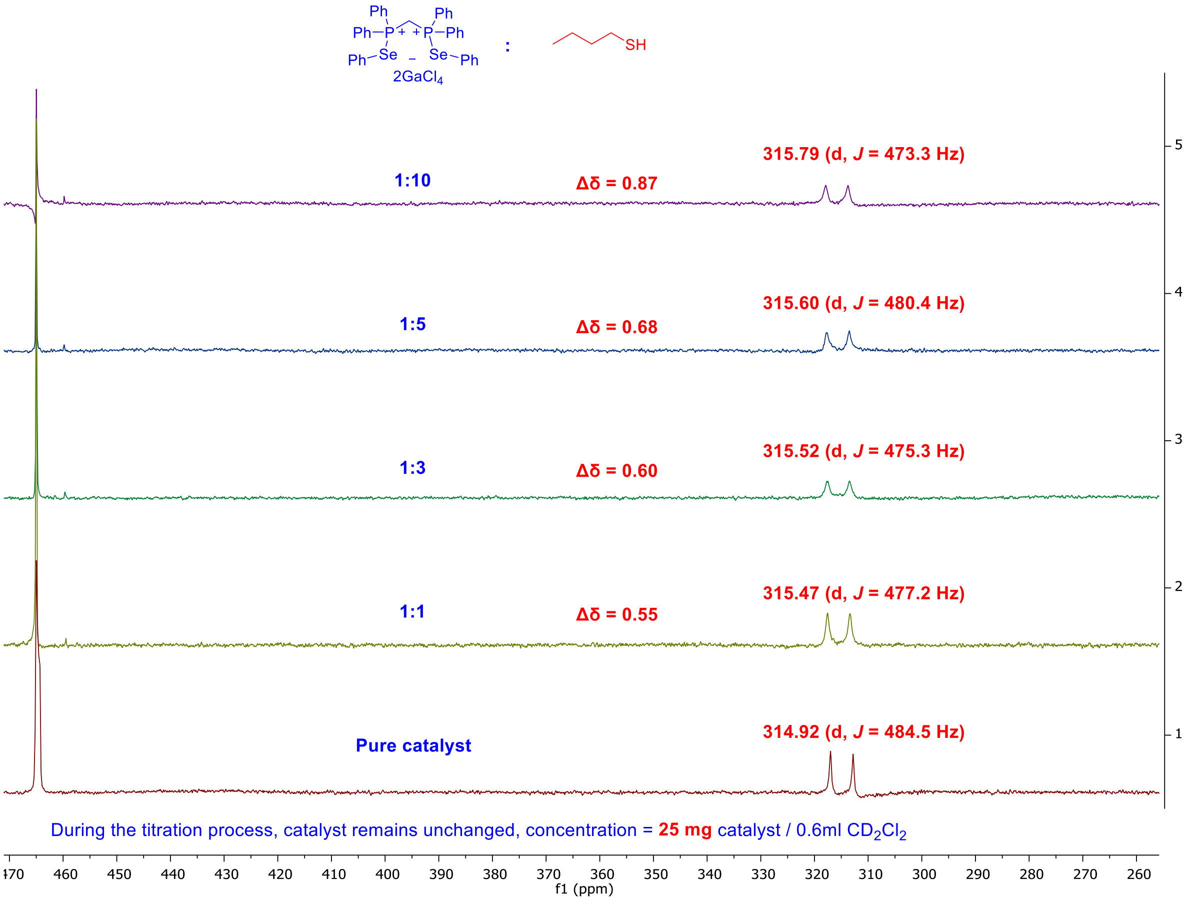

Supplementary Figure 5-3. ^{77}Se titration for catalyst **A** and butanethiol.

^{31}P NMR shift of catalyst A

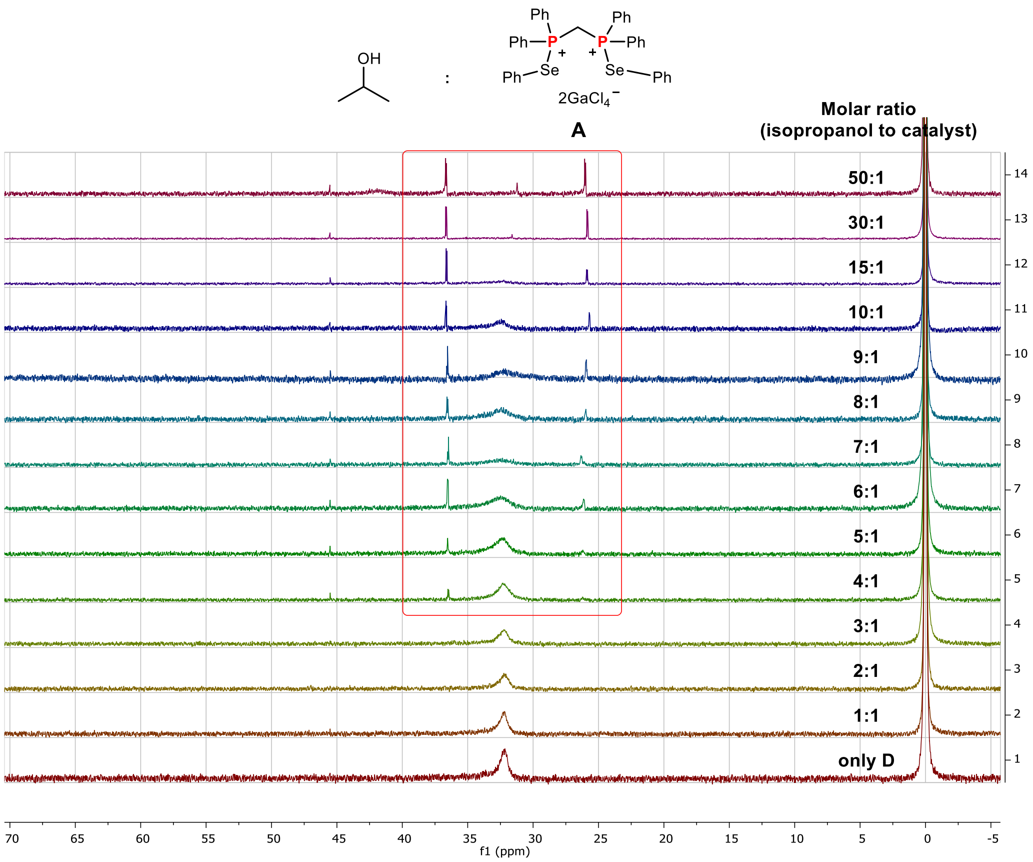

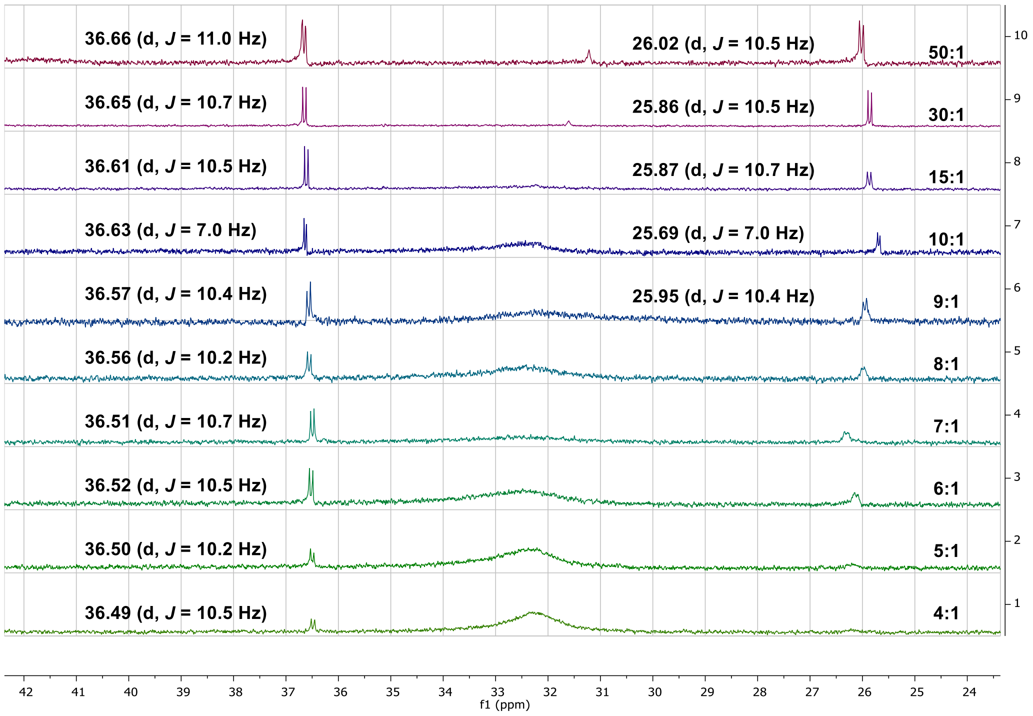

Supplementary Figure 6. ^{31}P titration of catalyst **A** with isopropanol.

(See Supplementary Discussion)

^{77}Se NMR shift on catalyst A with 1a

(Related to Figure 2b in the manuscript)

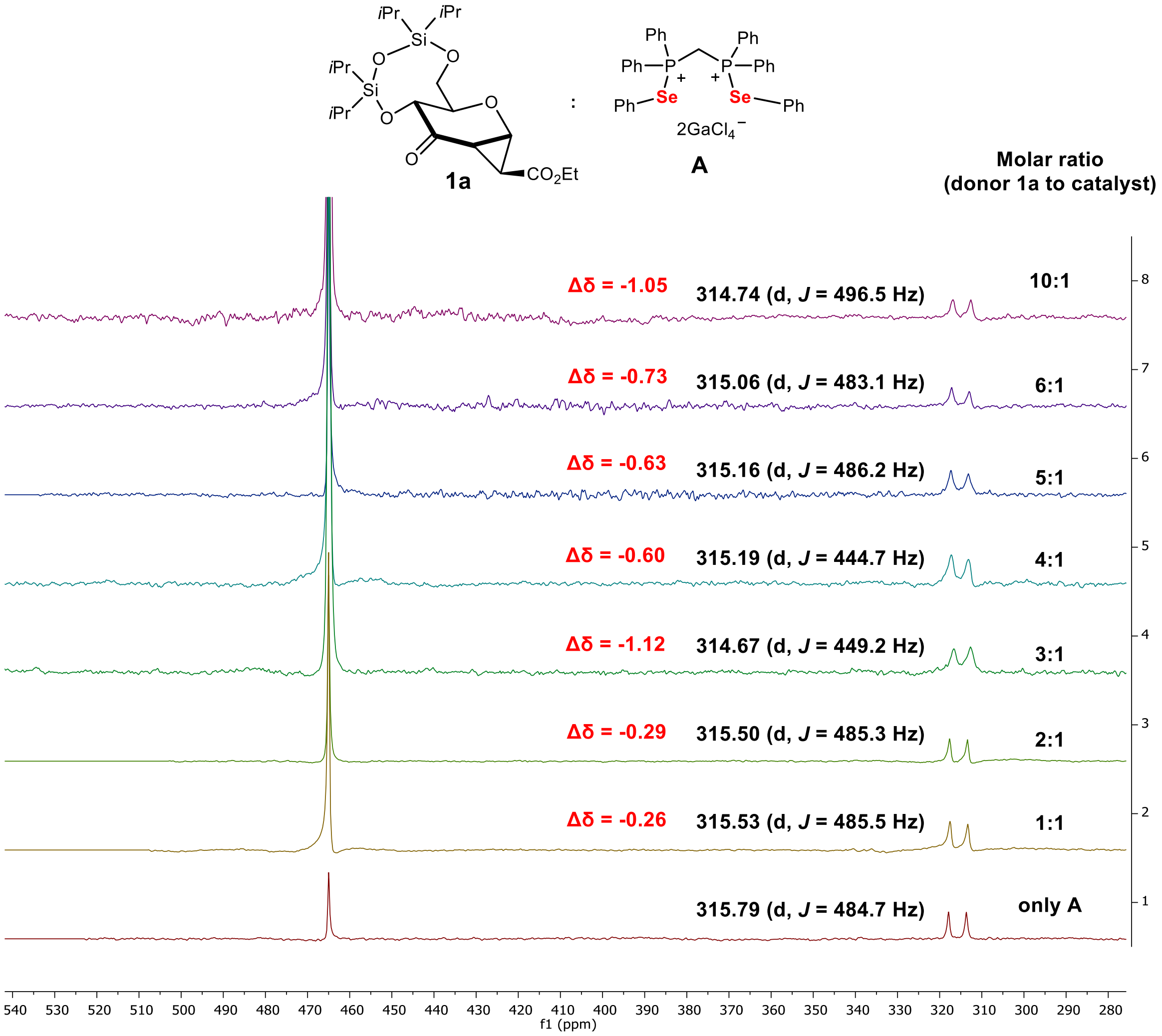

Supplementary Figure 7. ^{77}Se titration of catalyst **A** with donor **1a**.

^{13}C NMR shift of carbonyl group and ester group on donor 1a with catalyst (Related to Figure 2b in the manuscript)

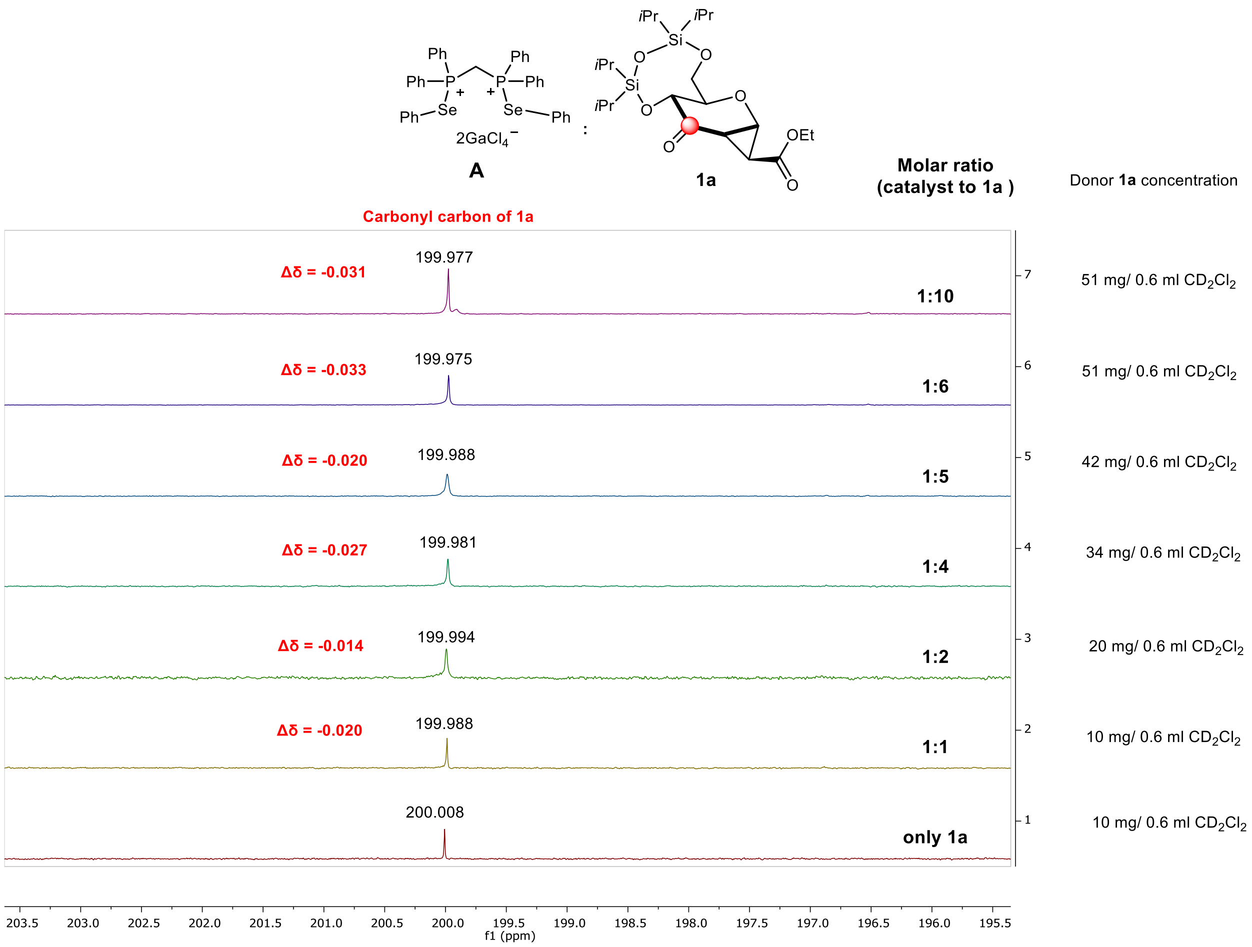

Supplementary Figure 8-1. ^{13}C titration of catalyst **A** with donor **1a**.

Taking into consideration that the concentration of the donor continuously varies during the titration process mentioned above, in order to eliminate possible errors that could result from concentration changes, we repeated the ^{13}C titration under constant donor concentrations (Three different donor concentrations were selected as presented Supplementary Figures 8-2 to 8-4). We noted that a reproducible upfield shift of 0.010-0.014 ppm can still be observed consistently, indicating that a weak but non-negligible ChB between the ketone and the catalyst is operative in the binary titration.

The specific experimental results are as follows.

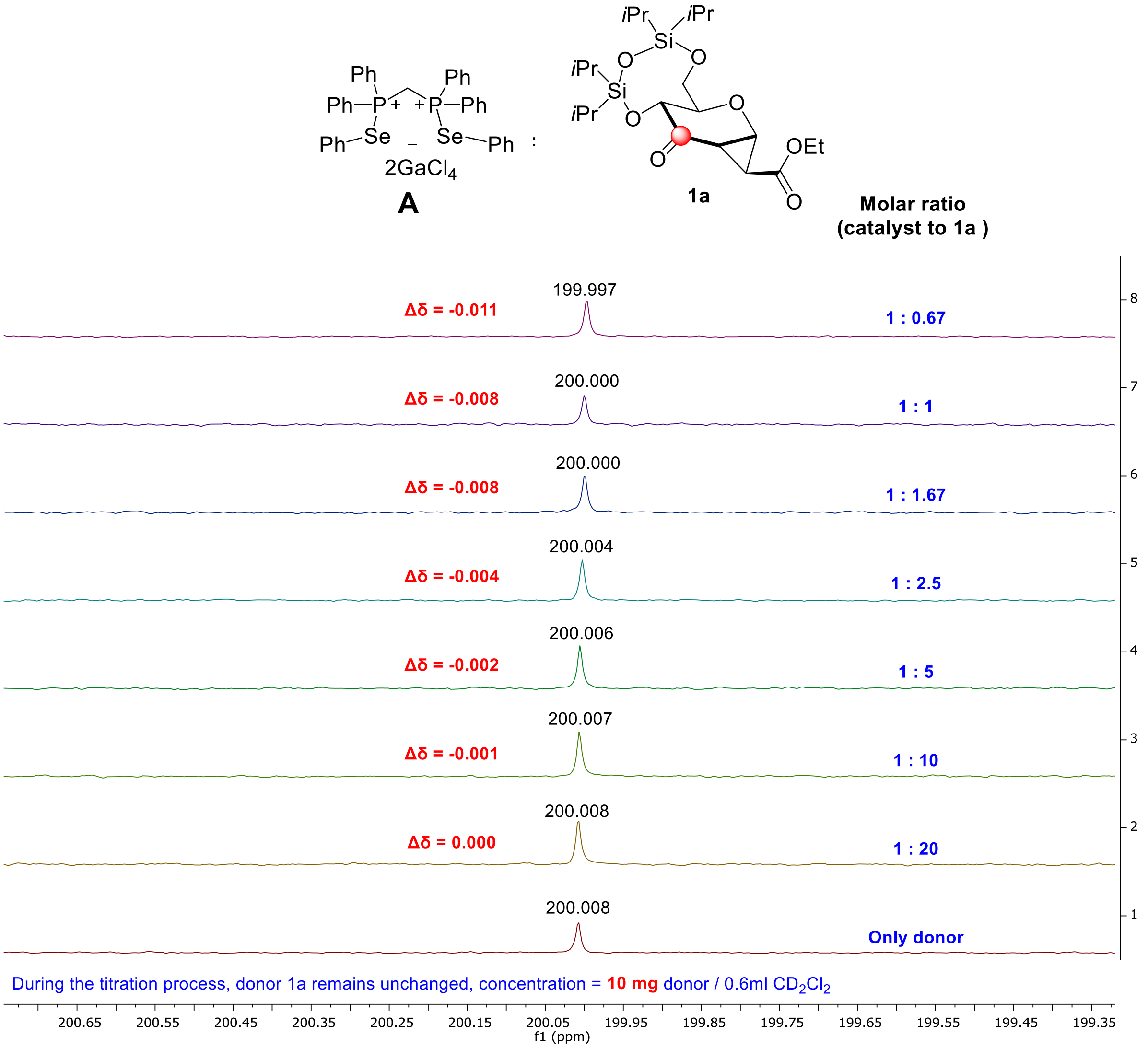

Supplementary Figure 8-2. ^{13}C titration of catalyst **A** with donor **1a**.

During the titration process, the volume of CD_2Cl_2 is maintained at 0.6 ml. Since a maximum of 30 mg catalyst can be dissolved in 0.6 ml of CD_2Cl_2, the catalyst-to-donor ratio can only be titrated to 1:2 when the donor concentration is 30 mg/0.6 mL CD_2Cl_2.

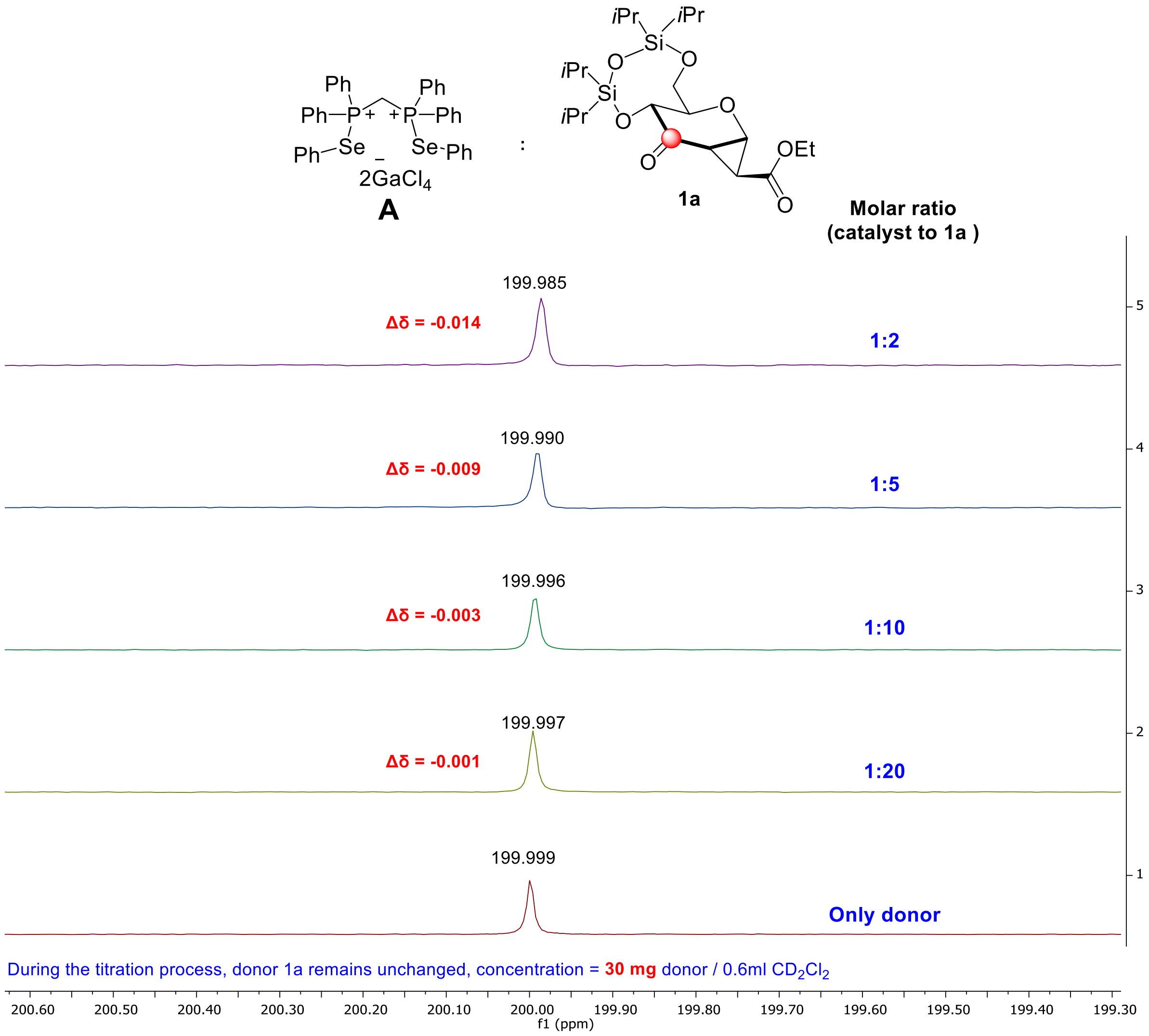

Supplementary Figure 8-3. ^{13}C titration of catalyst **A** with donor **1a**.

During the titration process, the volume of CD_2Cl_2 is maintained at 0.6 ml. Since a maximum of 30 mg catalyst can be dissolved in 0.6 ml of CD_2Cl_2, when the donor concentration is 60 mg/0.6 mL CD_2Cl_2, the catalyst-to-donor ratio can only be titrated to 1:5.

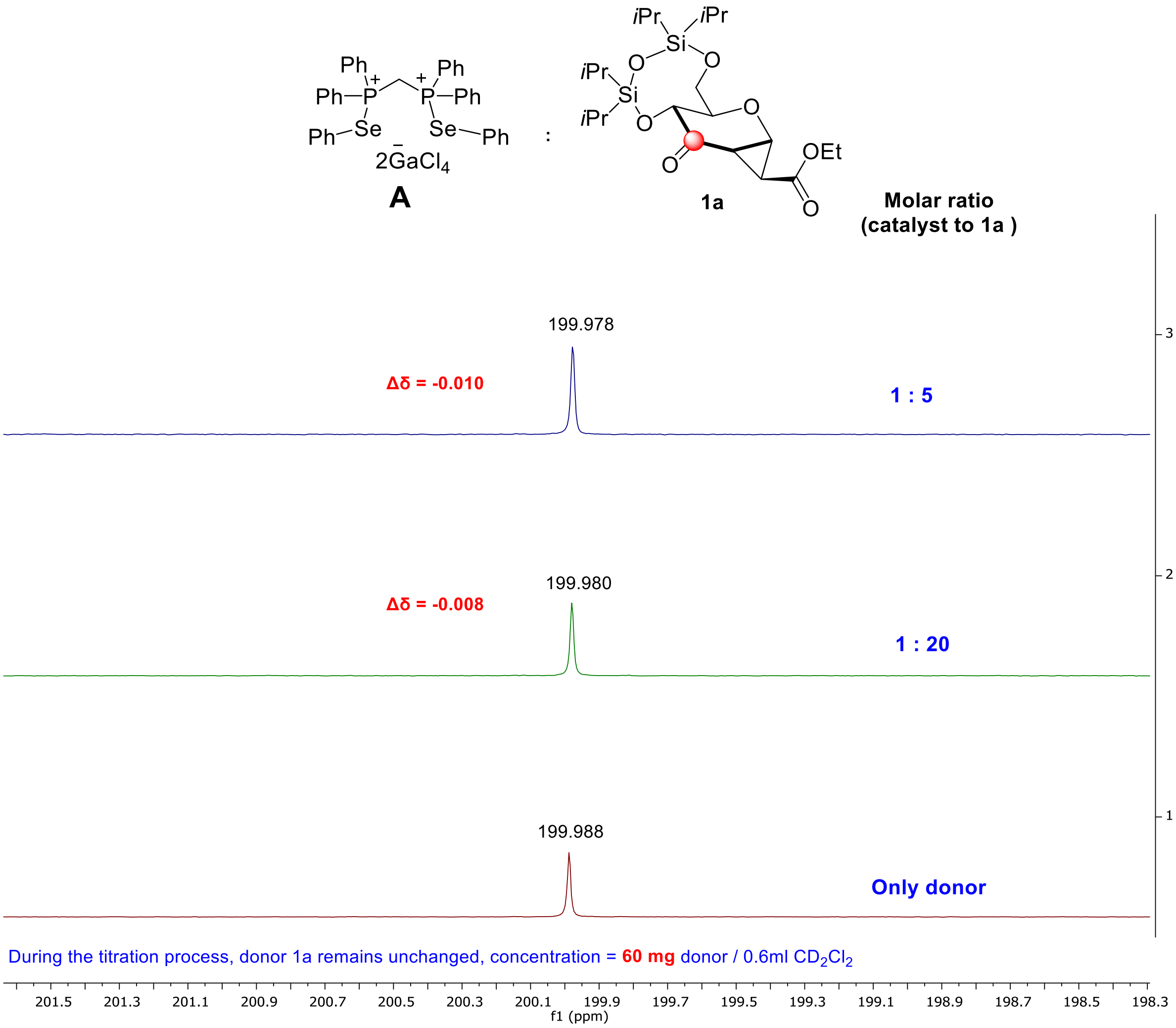

Supplementary Figure 8-4. ^{13}C titration of catalyst **A** with donor **1a**.

^{13}C NMR shift of carbonyl carbon of donor 1a with isopropanol

(Related to Figure 2c in the manuscript)

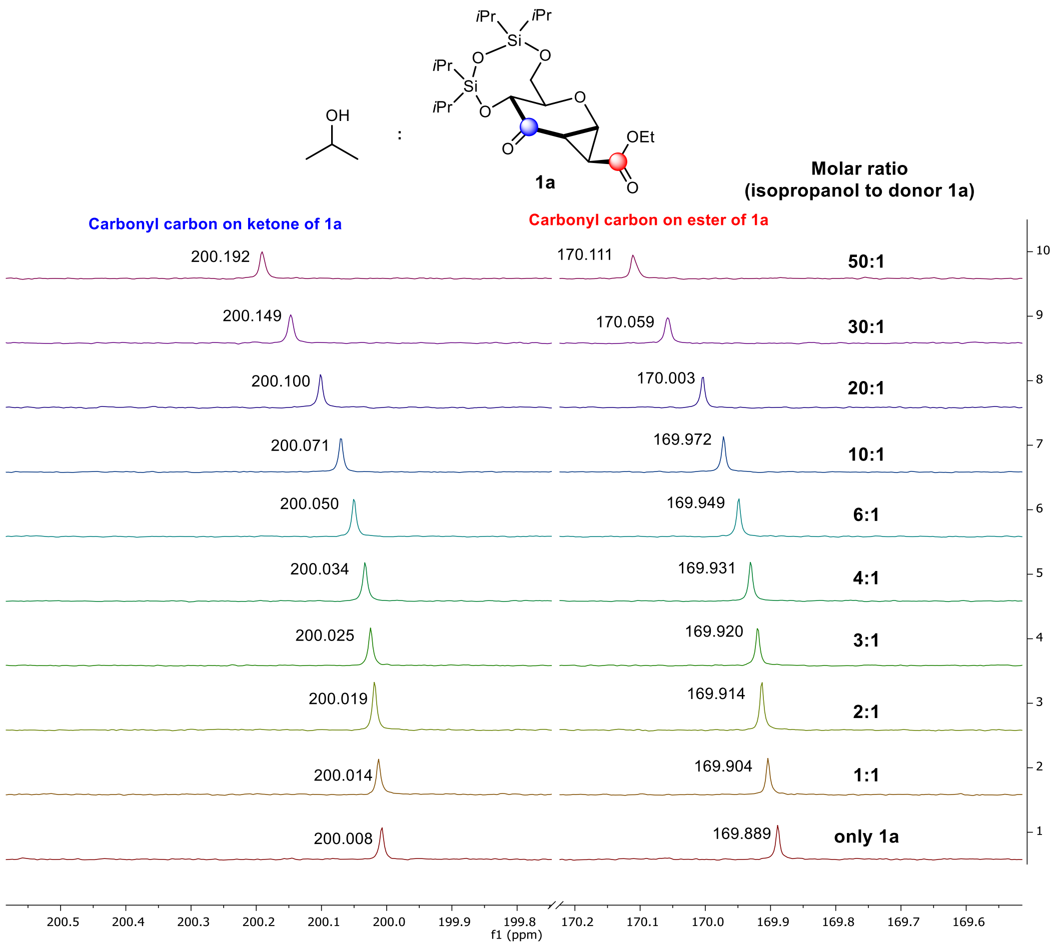

Entry	Concentration of **1a**	Concentration of isopropanol	Ketone δ /ppm	Ester δ /ppm
1	0.05	0	200.008	169.889
2	0.05	0.05	200.014	169.904
3	0.05	0.10	200.019	169.914
4	0.05	0.15	200.025	169.920
5	0.05	0.20	200.034	169.931
6	0.05	0.30	200.050	169.949
7	0.05	0.50	200.071	169.972
8	0.05	1.00	200.100	170.003
9	0.05	2.00	200.149	170.059
10	0.05	3.00	200.192	170.111

Supplementary Figure 9-1. ^{13}C titration of donor **1a** with isopropanol.

For the determination of the binding constants, the ^{13}C shifts of the carbon of the host (donor **1a**) was recorded (experiment conducted in CD_2Cl_2 solvent). The collected data was fitted using the bindfit program[14,17,18]. For the calculations of the binding constants (K in M^{-1}) a 1:2 stoichiometry was selected as the optimal stoichiometry for fitting. K_{11} and K_{12} are the two respective rate constants defined within a 1:2 host to guest stoichiometry model as seen in the equations below.[14]

The **1 : 2** host–guest system

$$\mathbf{H} + \mathbf{G} \underset{}{\overset{K_{11}}{\rightleftharpoons}} \mathbf{HG} \qquad \mathbf{HG} + \mathbf{G} \underset{}{\overset{K_{12}}{\rightleftharpoons}} \mathbf{HG_2}$$

$$K_{11} = \frac{[\mathrm{HG}]}{[\mathrm{H}][\mathrm{G}]} \qquad K_{12} = \frac{[\mathrm{HG_2}]}{[\mathrm{H}][\mathrm{HG}]}$$

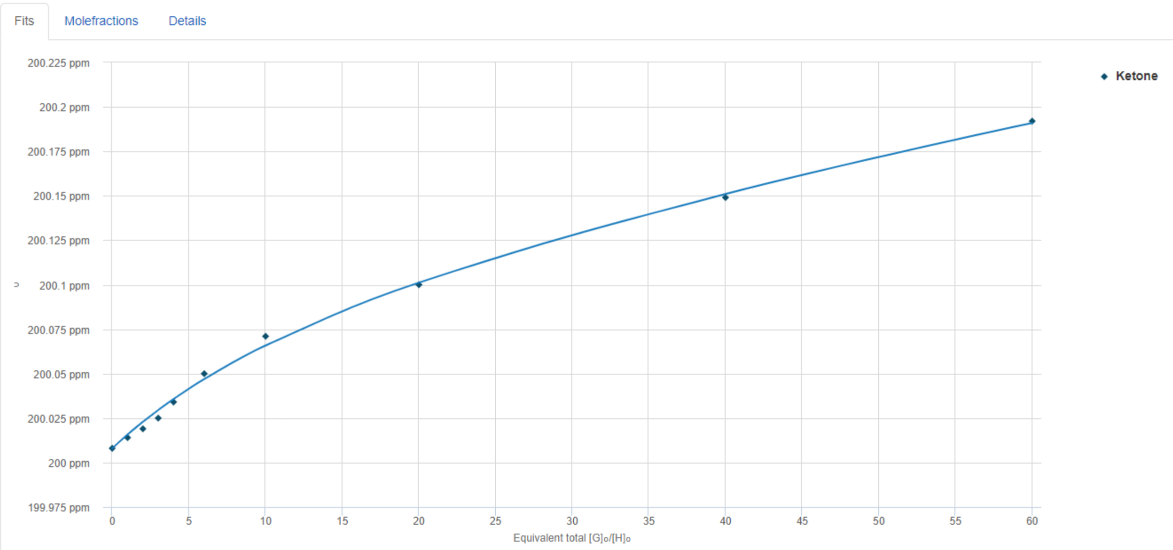

Supplementary Figure 9-2: Titration of donor **1a** with isopropanol in CD_2Cl_2, K_{11} = 1.20 M^{-1}; K_{12}=1.39e-9 M^{-1} (Ketone carbonyl titration, NMR 1:2, method: L-BFGS-B)

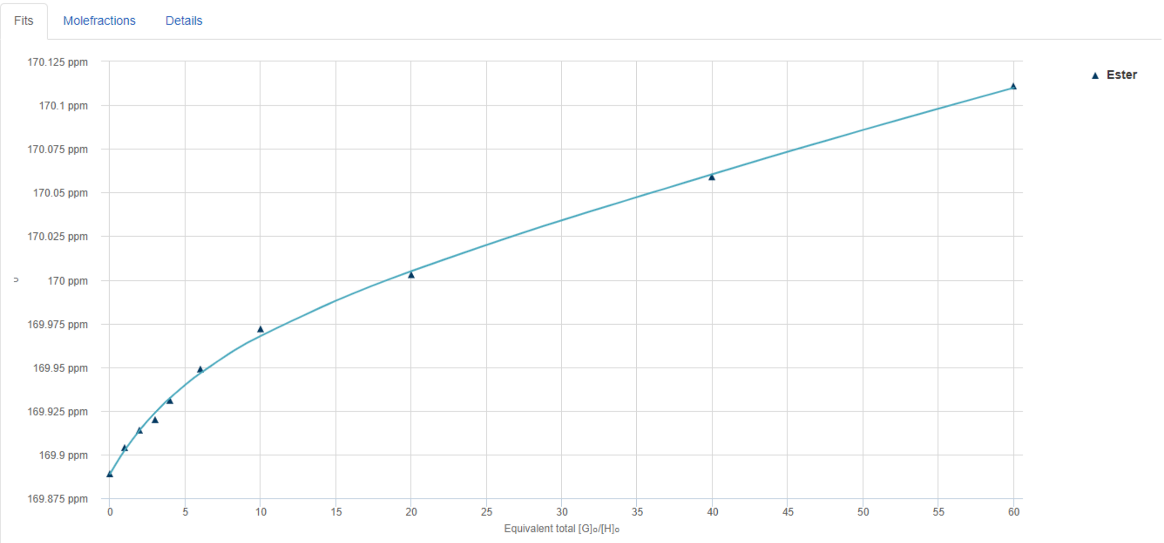

Supplementary Figure 9-3: Titration of donor **1a** with isopropanol in CD_2Cl_2, K_{11} = 3.23 M^{-1}; K_{12}= 3.98e-9 M^{-1} (Ester carbonyl titration, NMR 1:2, method: L-BFGS-B)

^{13}C NMR shift of carbonyl carbon of donor 1a with butanethiol

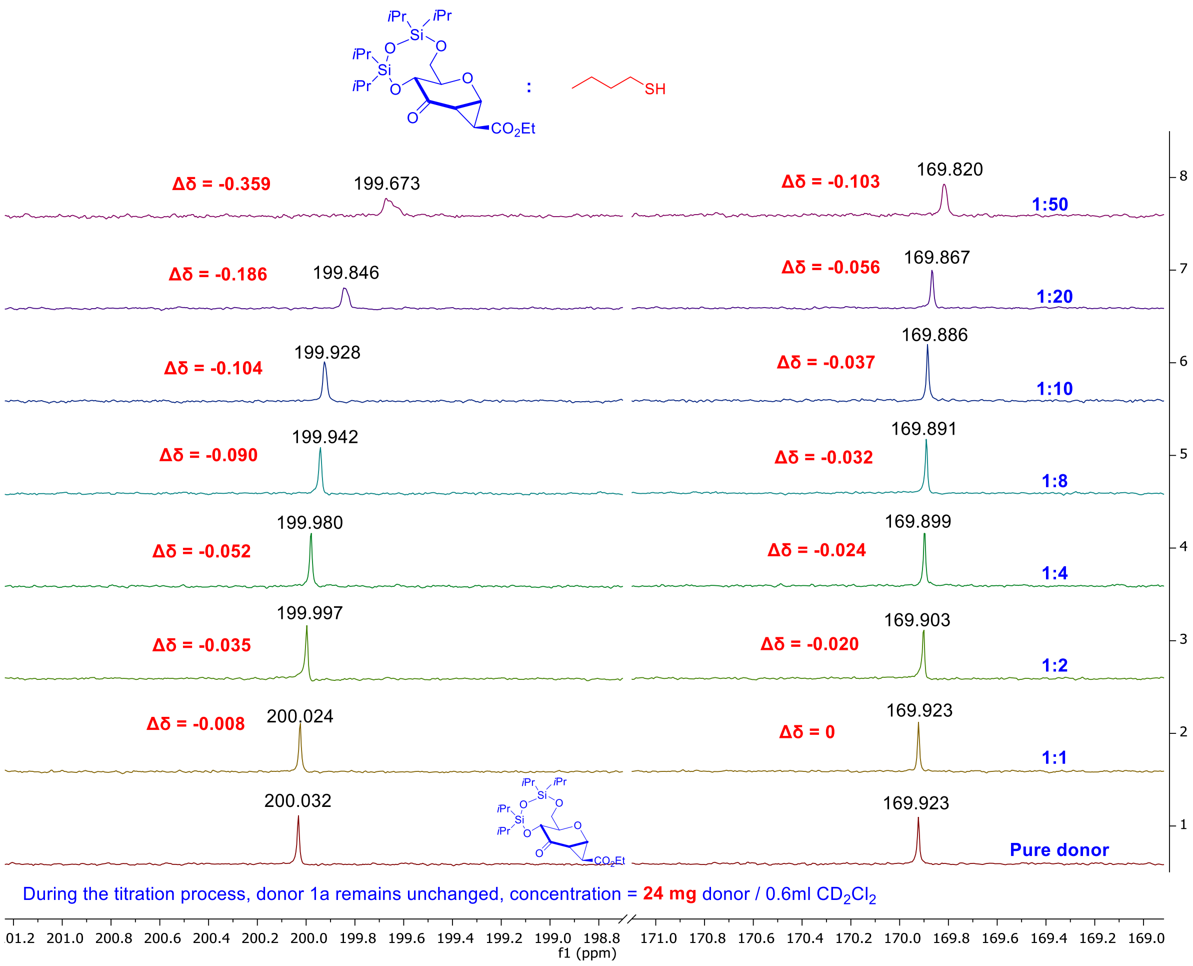

Supplementary Figure 9-4. ^{13}C titration of donor **1a** with butanethiol.

^{13}C NMR monitoring of standard reaction

Procedure: To a dry NMR tube was charged cyclopropanated donor **1a** (47.3 mg, 0.1 mmol), catalyst **A** (2.2 mg, 0.002 mmol) and isopropanol (6 mg, 0.01 mmol, 1eq). CD_2Cl_2 was then added in order to reach a total volume of 0.5 mL. Afterwards, the tube was sealed with cap and measured on the NMR spectrometer at room temperature by recording ^{13}C-NMR spectra at different time.

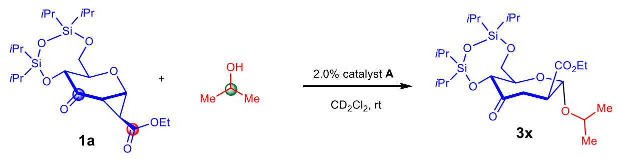

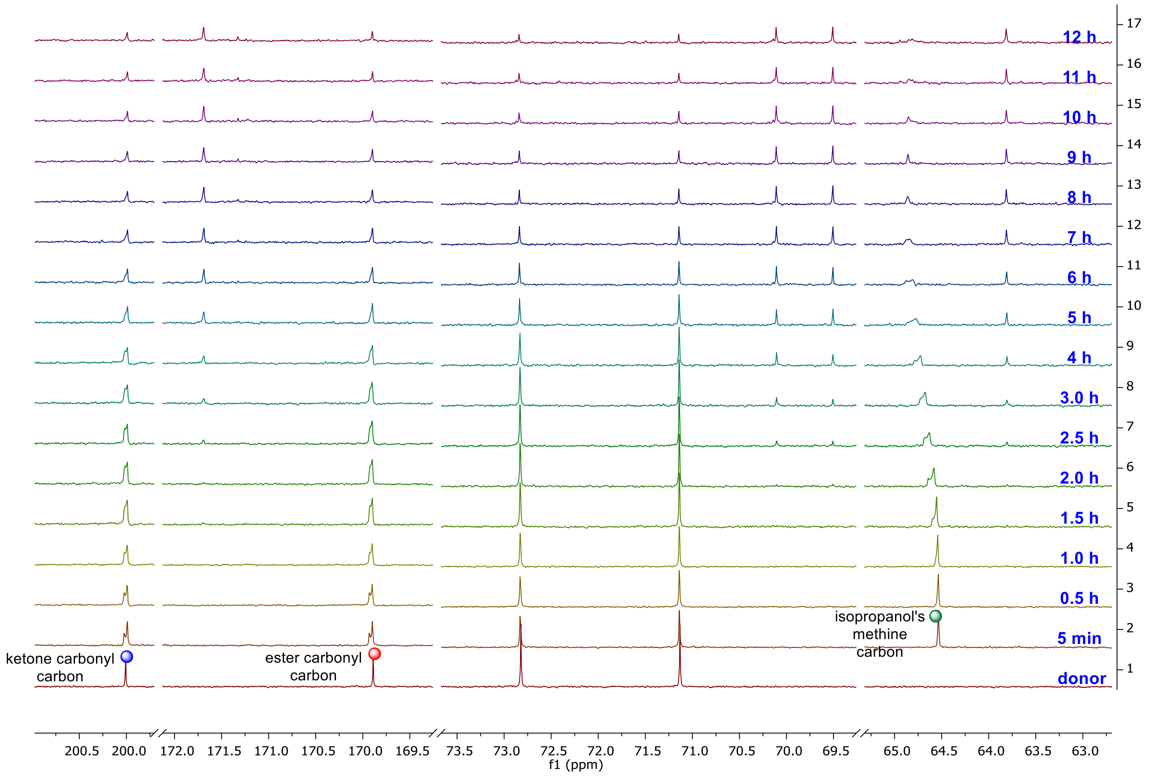

Supplementary Figure 10-1. ^{13}C-NMR monitoring of donor **1a** with isopropanol.

^{13}C NMR in-situ poisoning of complex peaks with PPh_3 and TBAC

Procedure:To a dry NMR tube, isopropanol (6.0 mg, 0.01 mmol), catalyst **A** (2.2 mg, 0.002 mmol), cyclopropanated donor **1a** (47.3 mg, 0.1 mmol), CD_2Cl_2 was added in order to reach a total volume of 500 µL. Afterwards, the tube was sealed with cap and measured on the NMR spectrometer room temperature by recording ^{13}C spectra after 1 hour, then add PPh_3 or TBAC (0.1eq, 0.01 mmol) in the reaction and measured ^{13}C-NMR at once and after 18h.

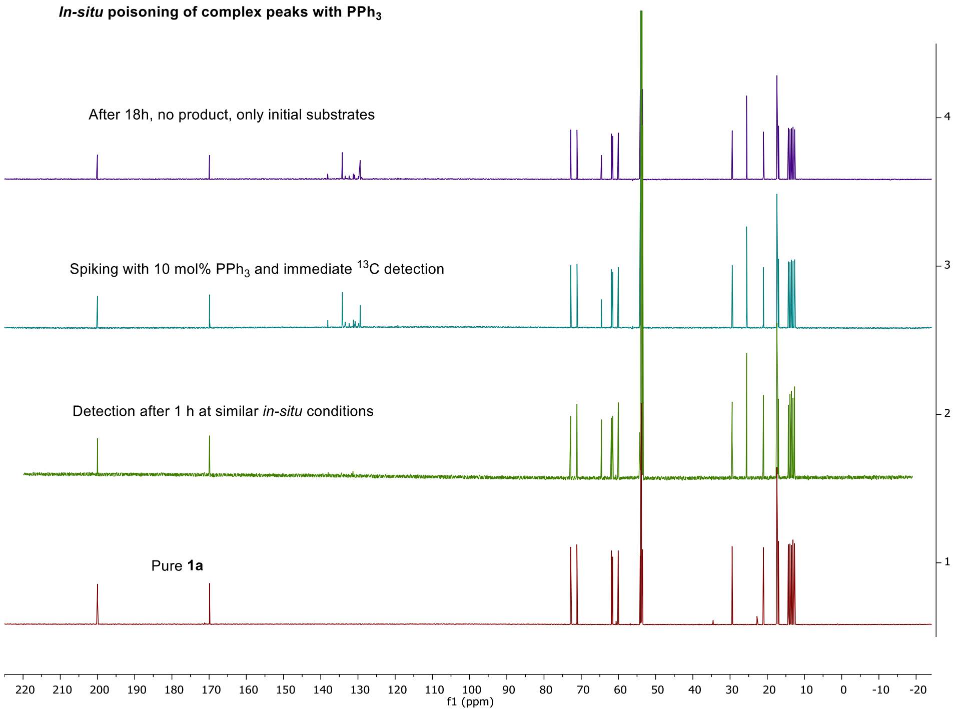

Supplementary Figure 10-2. ^{13}C NMR in-situ poisoning of complex peaks with PPh_3.

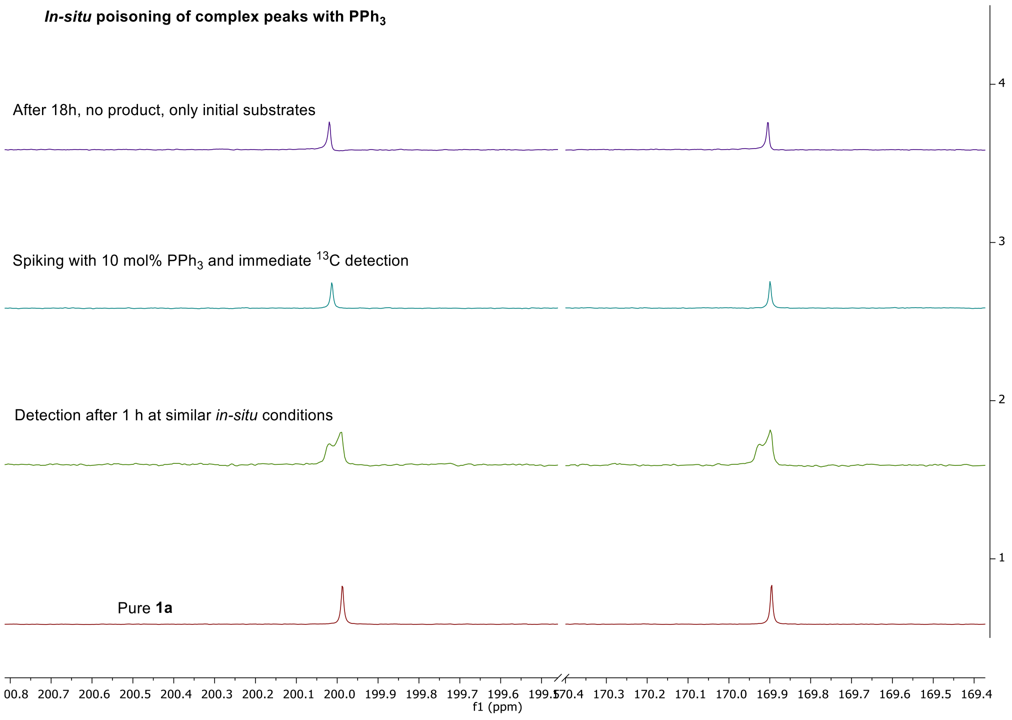

Supplementary Figure 10-3. Zoomed-in graph of the chemical shift of ester and ketone signal in **1a**.

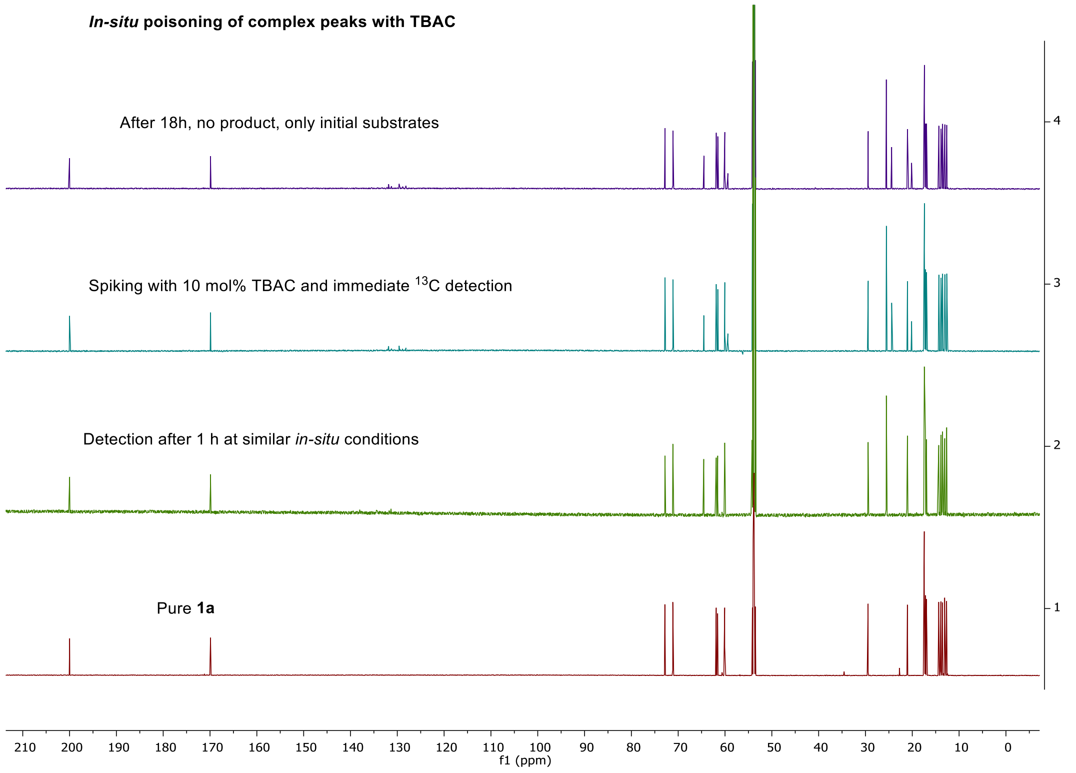

Supplementary Figure 10-4. ^{13}C NMR in-situ poisoning of complex peaks with TBAC.

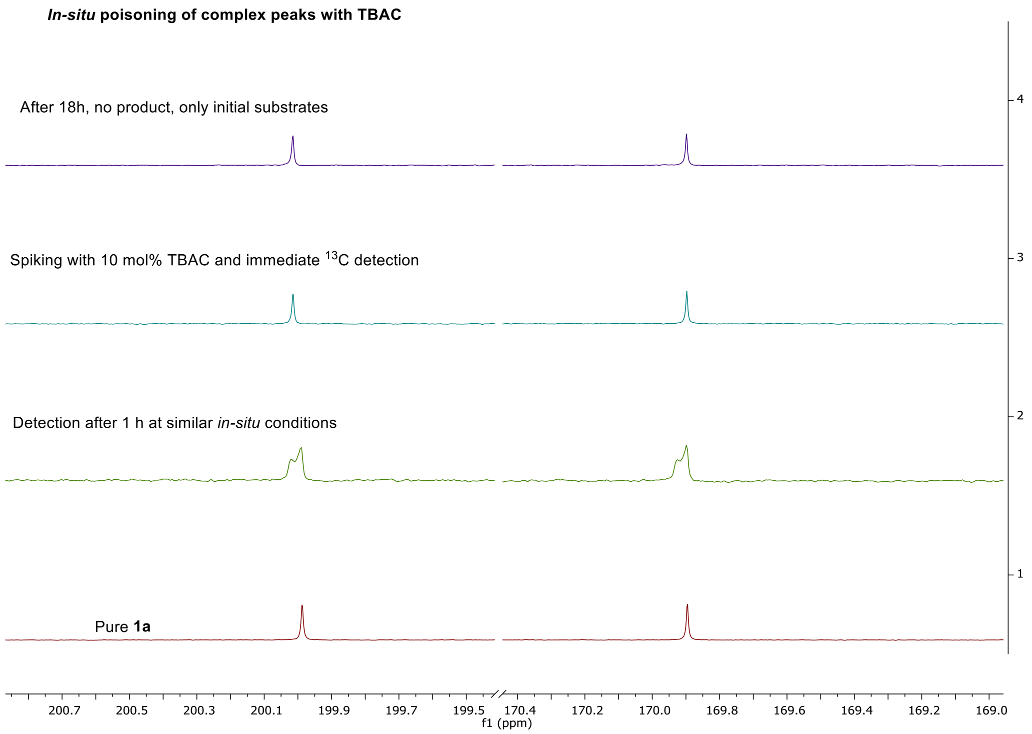

Supplementary Figure 10-5. Zoomed-in graph of the chemical shift of ester and ketone signal in **1a**.

^{77}Se-NMR monitoring of standard reaction

Procedure: To a dry NMR tube was charged cyclopropanated donor **1a** (47.3 mg, 0.1 mmol), catalyst **A** (2.2 mg, 0.002 mmol) and isopropanol (6 mg, 0.01 mmol, 1eq). CD_2Cl_2 was then added in order to reach a total volume of 0.5 mL. Afterwards, the tube was sealed with cap and measured on the NMR spectrometer at room temperature by recording ^{77}Se-NMR spectra every 1 hour. Each ^{77}Se-NMR spectrum acquisition involves 1000 scans and takes approximately 40 minutes. (See **Supplementary Discussion 2**)

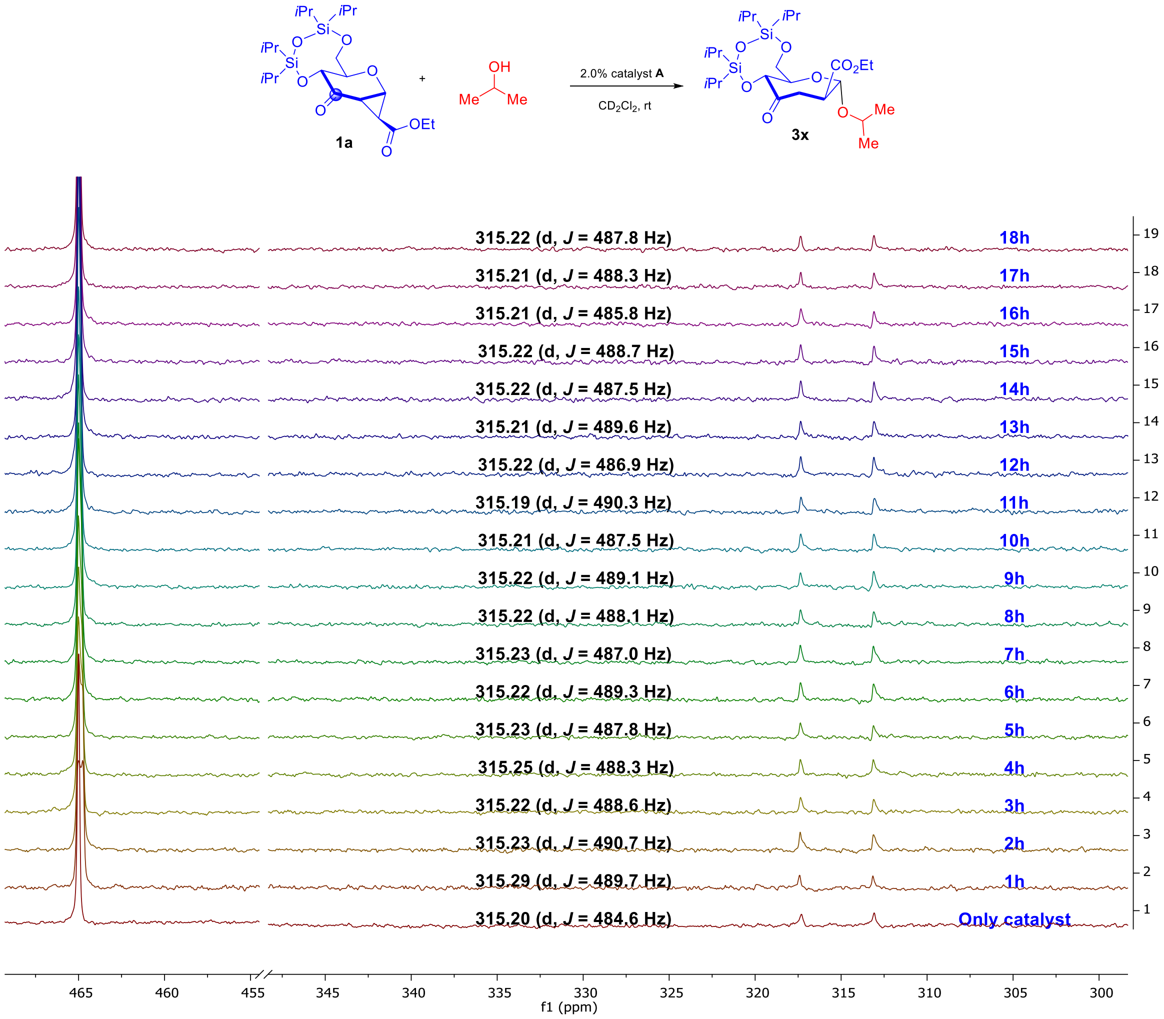

Supplementary Figure 10-6. ^{77}Se-NMR monitoring of donor **1a** with isopropanol.

^{13}C NMR shift of carbonyl carbon of donor 1a with trifluoroacetate

Procedure: To an NMR tube filled with donor **1a** (36 mg, 0.076 mmol) was added varying amounts of trifluoroacetate (Note: normally donor:trifluoroacetate molar ratio are 20:1, 10:1, 1:1). Varying amounts of CD_2Cl_2 were added in order to reach a total volume of 0.6 ml. The detected ^{13}C shifts of donor **1a** are shown respectively.

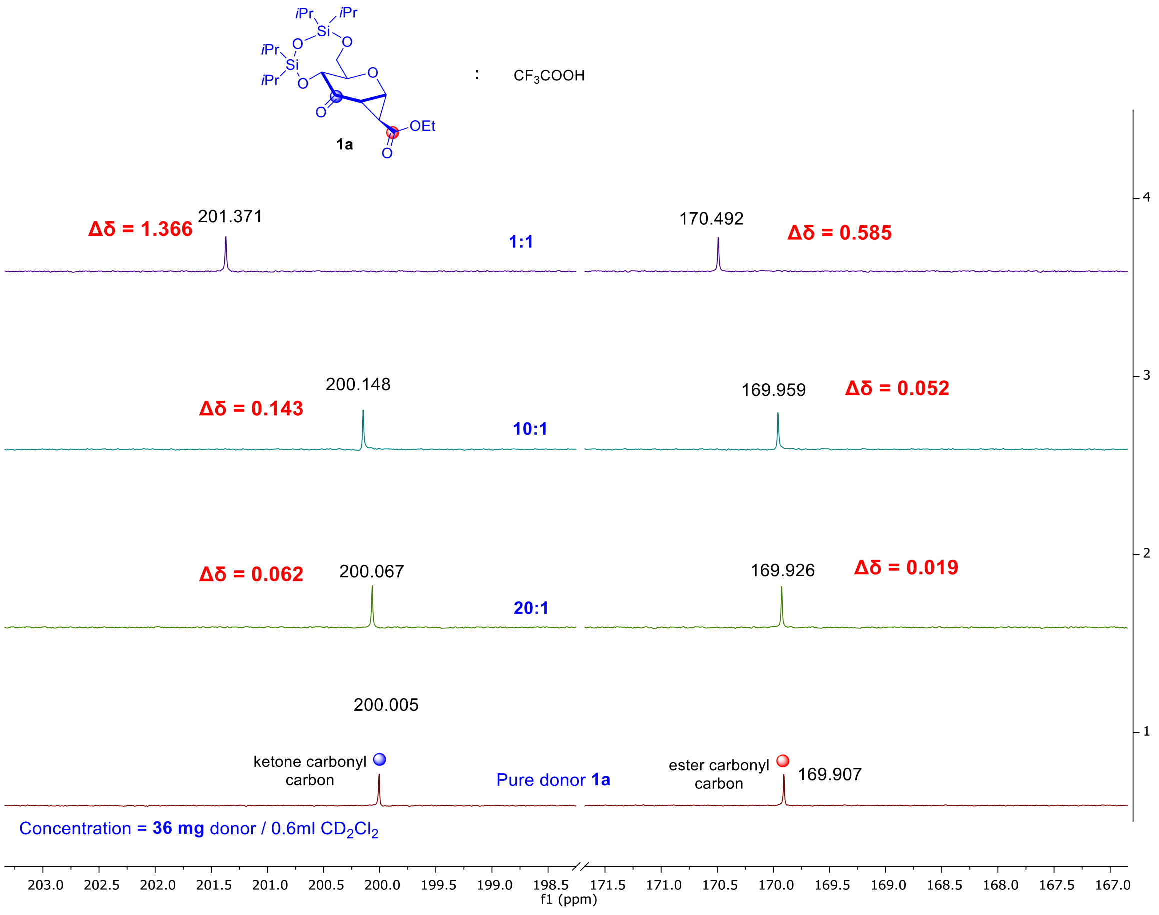

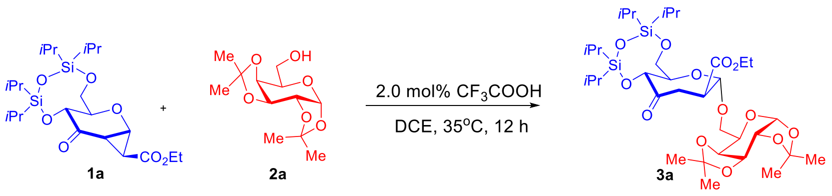

No **3a** was detected in crude ^{1}H-NMR

Supplementary Figure 11. ^{13}C titration of donor **1a** with trifluoroacetate and control experiment.

1H NMR shift of hydroxyl signal on isopropanol

Procedure: 0.5 mL of CD_2Cl_2 and 1.0 equivalent isopropanol were added to the NMR tube and the ^{1}H-NMR spectra was measured; 3.0 equivalents of donor **1a** was dissolved in 90 µl of CD_2Cl_2, subsequently 0.5 equivalent of **1a** was added to the NMR tube for each titration measurement (15 µl) and then the ^{1}H-NMR was immediately recorded.

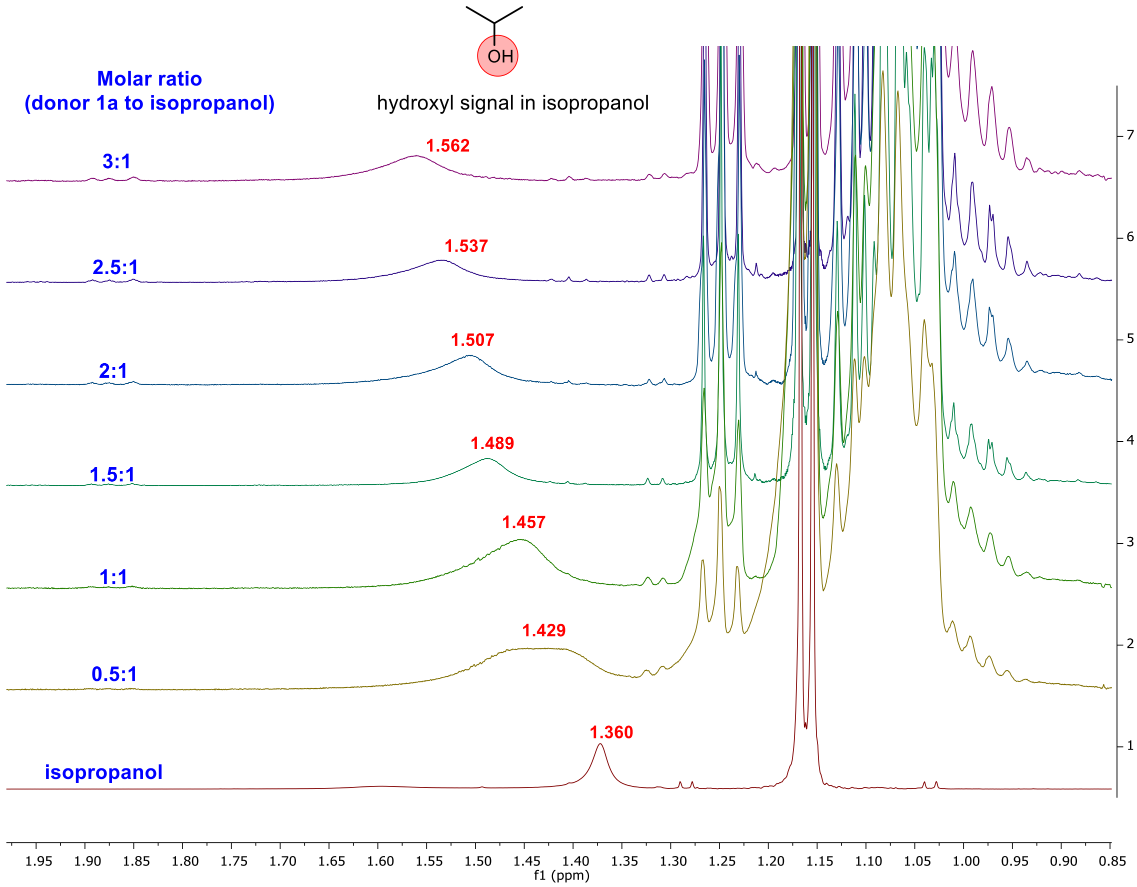

Supplementary Figure 12-1. ^{1}H titration of isopropanol with donor **1a**.

^{1}H NMR shift of hydroxyl signal on isopropanol

Procedure: 0.5 mL of CD_2Cl_2 and 1.0 equivalent isopropanol were added to the NMR tube and the ^{1}H-NMR spectra was measured; 2.0 mol% catalyst **A** was dissolved in 20 µl of CD_2Cl_2 for ^{1}H-NMR measurement; 3.0 equivalents of donor **1a** was dissolved in 90 µl of CD_2Cl_2, 0.5 equivalent of **1a** was subsequently added for each titration measurement (15 µl) and then the ^{1}H-NMR spectra was immediately recorded.

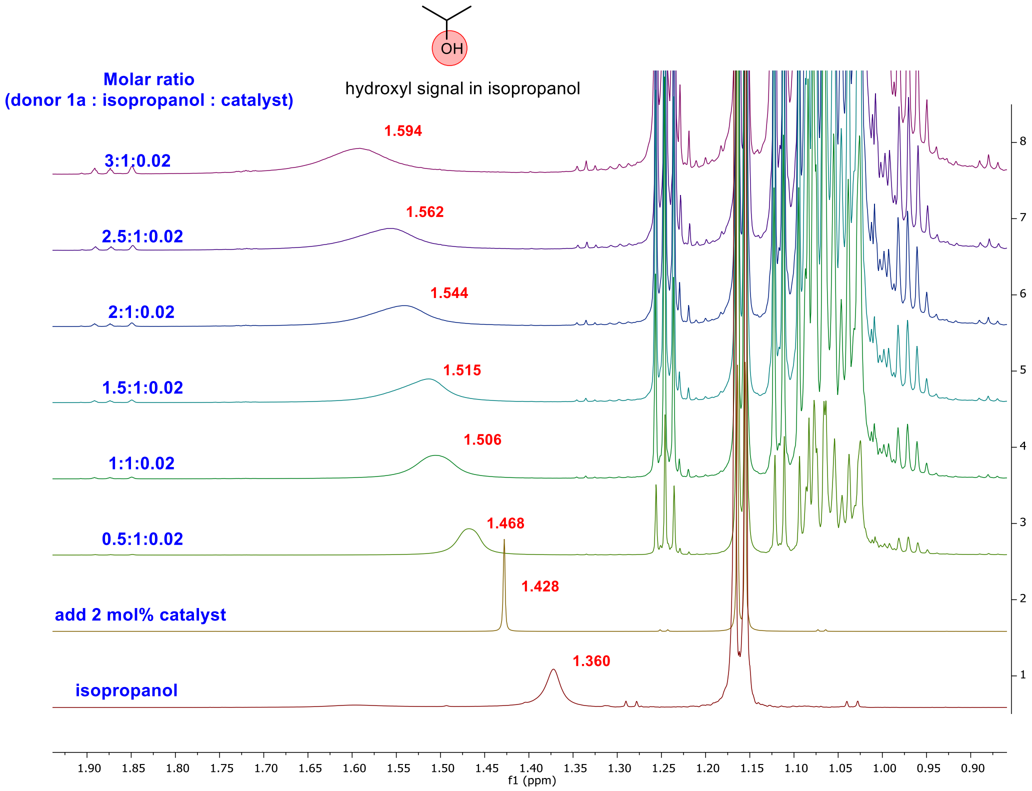

Supplementary Figure 12-2. ^{1}H titration of isopropanol, catalyst **A** with donor **1a**.

Supplementary Table 17 chemical shift difference between three-component titration with an analogous two-component titration

Donor: isopropanol			Donor: isopropanol: catalyst			chemical shift difference
Entry	ratio	chemical shift (ppm)	Entry	ratio	chemical shift (ppm)	δ ppm
1	3:1	1.562	9	3:1:2%	1.594	0.032
2	2.5:1	1.537	10	2.5:1:2%	1.562	0.025
3	2:1	1.507	11	2:1:2%	1.544	0.037
4	1.5:1	1.489	12	1.5:1:2%	1.515	0.026
5	1:1	1.457	13	1:1:2%	1.506	0.049
6	0.5:1	1.429	14	0.5:1:2%	1.468	0.039
7	/	/	15	0:1:2%	1.428	0.068
8	isopropanol	1.360	16	isopropanol	1.360	0

^{13}C NMR shift of carbonyl carbon of donor 1a and methine carbon of isopropanol

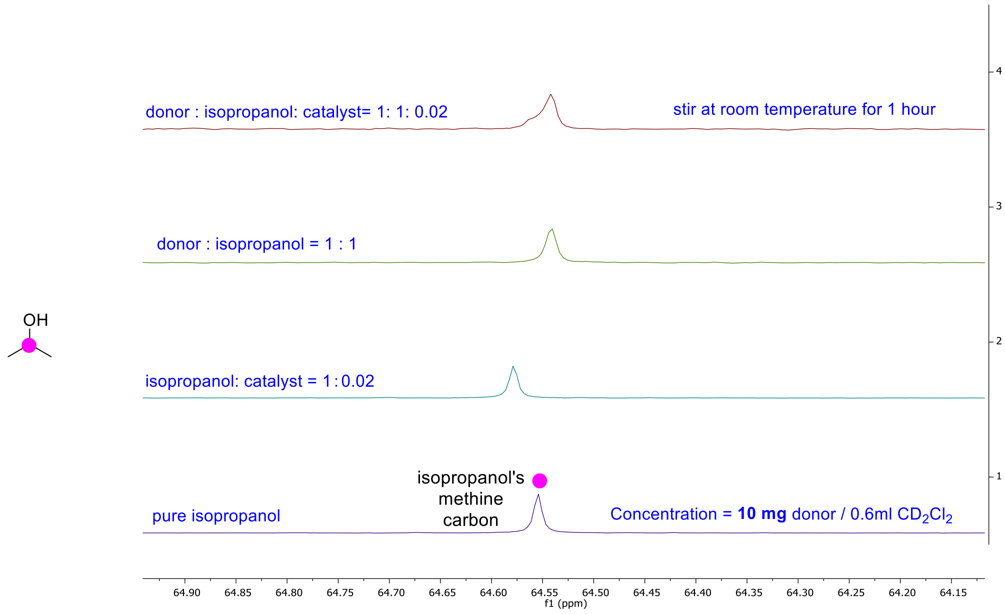

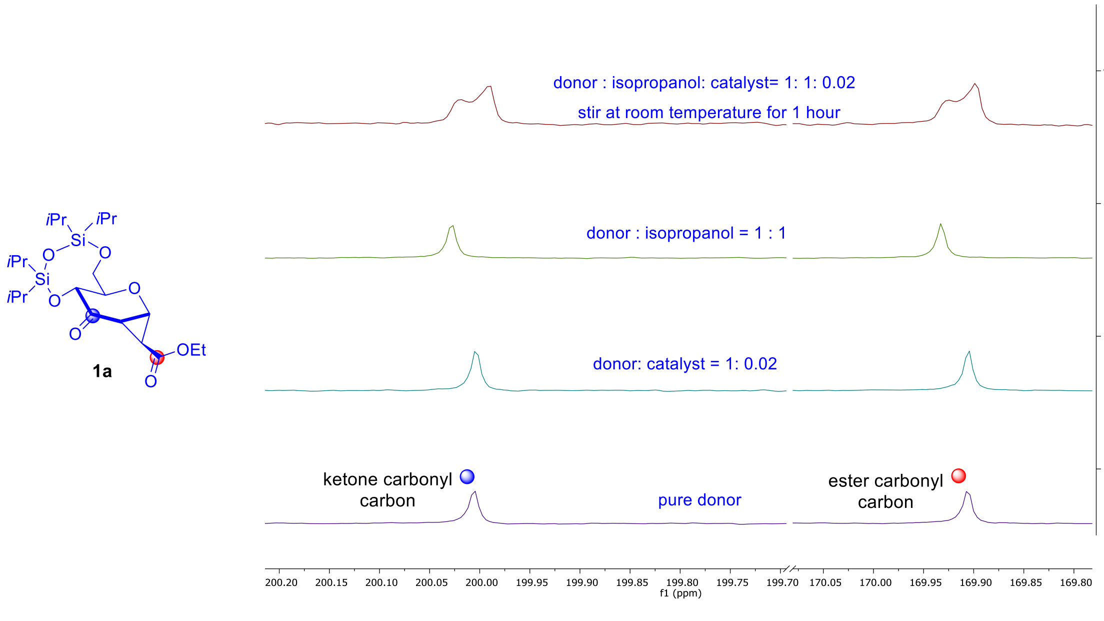

Concentration = **47 mg** donor / 0.6ml CD_2Cl_2

Supplementary Figure 13. Comparing ^{13}C NMR in donor **1a** and isopropanol.

Deuterated experiments

(Related to Figure 3d in the manuscript)

NMR data for deuterated experiment using isopropanol-d_1

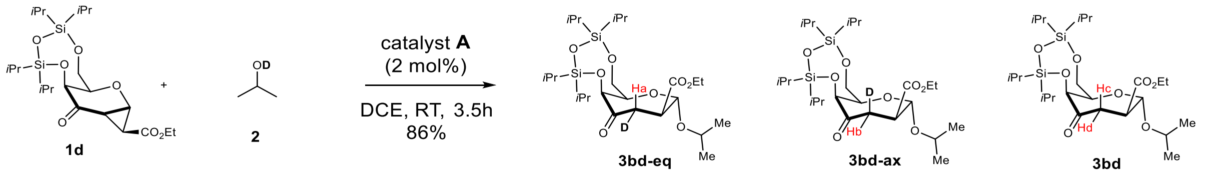

1 : 3.1 : 1.33
(due to residual H_2O)

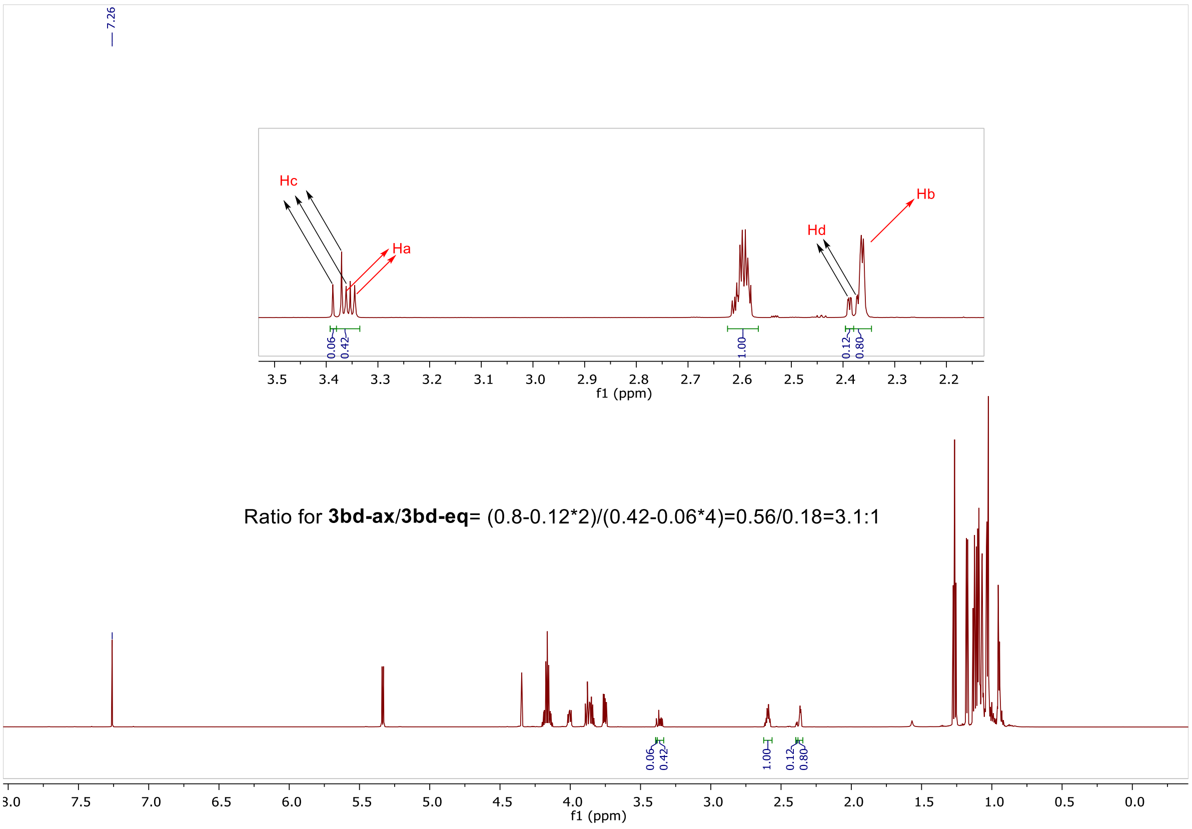

Supplementary Figure 14: ^{1}H NMR spectra for **compound 3bd**

NMR monitoring data for kinetics studies

NMR monitoring under standard conditions

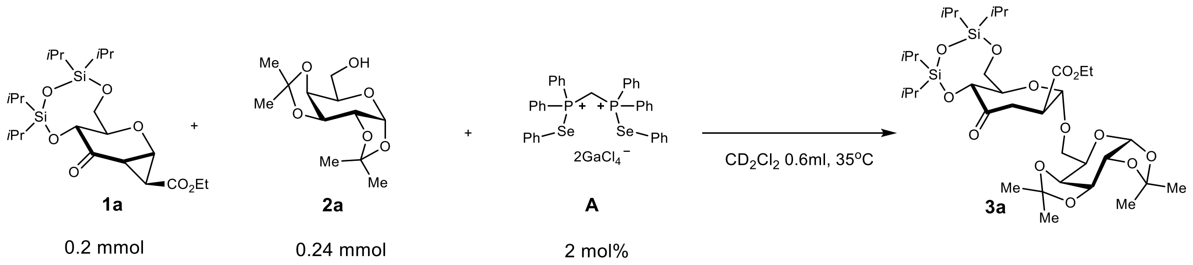

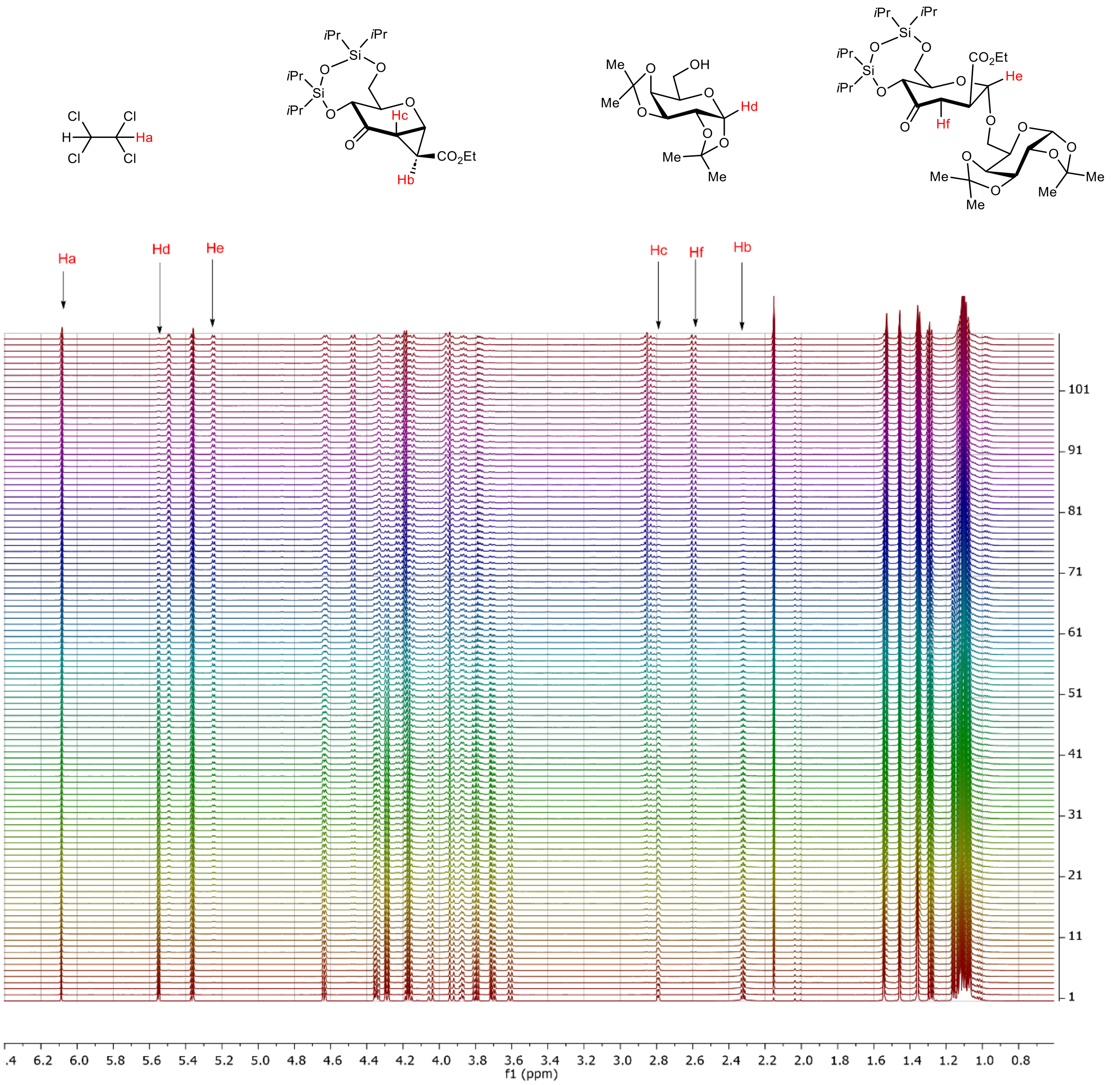

Supplementary Figure 15: Stacked ^{1}H NMR spectra for the monitoring under standard conditions

Supplementary table 18: Concentration for **1a**, **2a**, **3a** calculated by ^{1}H NMR analysis for the monitoring under standard conditions

Time/h	[**Donor-1a**]/M	[**Acceptor-2a**]/M	[**3a**]/M
0	0.196313	0.269505	0.002693
0.166667	0.196313	0.269505	0.005815
0.333333	0.193059	0.266665	0.006923
0.5	0.193542	0.261778	0.011049
0.666667	0.188434	0.260465	0.010903
0.833333	0.187697	0.256544	0.014101
1	0.184992	0.252749	0.016253
1.166667	0.182318	0.250596	0.018136
1.333333	0.180412	0.246764	0.020771
1.5	0.17756	0.243112	0.022917
1.666667	0.176309	0.24046	0.024326
1.833333	0.174822	0.236737	0.027843
2	0.172088	0.234127	0.029223
2.166667	0.170613	0.230945	0.031569
2.333333	0.168814	0.228548	0.032848
2.5	0.166636	0.224033	0.037299
2.666667	0.16484	0.221923	0.037934
2.833333	0.163417	0.218862	0.040542
3	0.161377	0.216057	0.042995
3.166667	0.15916	0.213332	0.044903
3.333333	0.156271	0.211601	0.046365
3.5	0.154858	0.208975	0.048684
3.666667	0.153284	0.206339	0.050651
3.833333	0.15144	0.20436	0.052694
4	0.150255	0.201667	0.054414
4.166667	0.152045	0.193337	0.060307
4.333333	0.147239	0.196639	0.05925
4.5	0.144052	0.194453	0.061238
4.666667	0.143579	0.191665	0.063307
4.833333	0.139816	0.189858	0.064706
5	0.139313	0.186093	0.067788
5.166667	0.136596	0.182331	0.070733
5.333333	0.134338	0.181188	0.072031
5.5	0.132318	0.179182	0.074187
5.666667	0.133149	0.170248	0.078981
5.833333	0.131543	0.1671	0.082242
6	0.126307	0.170271	0.080902
6.166667	0.126863	0.161987	0.085948
6.333333	0.124424	0.159414	0.088408
6.5	0.122514	0.157769	0.089977
6.666667	0.117046	0.158699	0.09094

6.833333	0.117744	0.152093	0.094852
7	0.115644	0.148871	0.097562
7.166667	0.113144	0.146393	0.100237
7.333333	0.11019	0.143592	0.102034
7.5	0.108875	0.140533	0.104455
7.666667	0.106344	0.13768	0.106441
7.833333	0.103821	0.135045	0.109349
8	0.101741	0.132375	0.111934
8.166667	0.099563	0.130161	0.113617
8.333333	0.096462	0.126732	0.116087
8.5	0.09471	0.124881	0.118278
8.666667	0.092294	0.1215	0.120772
8.833333	0.089551	0.119609	0.123166
9	0.088057	0.115501	0.125677
9.166667	0.08539	0.114113	0.127429
9.333333	0.082874	0.111453	0.129692
9.5	0.080615	0.109353	0.132012
9.666667	0.077482	0.109104	0.134053
9.833333	0.076936	0.102589	0.136579
10	0.074381	0.101016	0.138631
10.16667	0.072106	0.098319	0.14056
10.33333	0.069451	0.096639	0.142648
10.5	0.067343	0.093437	0.14509
10.66667	0.065555	0.090856	0.146812
10.83333	0.063531	0.087815	0.14946
11	0.061611	0.085699	0.151462
11.16667	0.058749	0.083568	0.153005
11.33333	0.057005	0.080725	0.155739
11.5	0.05445	0.078707	0.156743
11.66667	0.05312	0.076118	0.159726
11.83333	0.051018	0.074516	0.161073
12	0.049086	0.071082	0.163572
12.16667	0.046967	0.069768	0.16528
12.33333	0.044669	0.068516	0.166657
12.5	0.042725	0.066399	0.168056
12.66667	0.040862	0.06451	0.170291
12.83333	0.03945	0.061681	0.172114
13	0.037651	0.060201	0.173419
13.16667	0.036345	0.057955	0.175143
13.33333	0.034309	0.056315	0.176192
13.5	0.033101	0.054304	0.178582
13.66667	0.029569	0.055967	0.178859
13.83333	0.026168	0.054552	0.180098
14	0.028434	0.050541	0.181313

14.16667	0.026288	0.050373	0.182216
14.33333	0.026366	0.047506	0.18314
14.5	0.023068	0.048865	0.184176
14.66667	0.023266	0.046426	0.184812
14.83333	0.023399	0.04393	0.186186
15	0.02169	0.043923	0.186407
15.16667	0.019457	0.044742	0.186879
15.33333	0.020579	0.041638	0.18743
15.5	0.019971	0.040652	0.188263
15.66667	0.018916	0.040025	0.188148
15.83333	0.018215	0.038973	0.189213
16	0.017537	0.038573	0.188838
16.16667	0.016911	0.038704	0.188386
16.33333	0.01641	0.037362	0.189645
16.5	0.015555	0.037628	0.189793
16.66667	0.015872	0.036193	0.190644
16.83333	0.015643	0.035732	0.190302
17	0.01495	0.035524	0.190675
17.16667	0.014141	0.036801	0.189276
17.33333	0.014334	0.035341	0.190565
17.5	0.013184	0.036279	0.188835
17.66667	0.012726	0.036515	0.188962
17.83333	0.01342	0.035093	0.189852
18	0.013316	0.034728	0.189519
18.16667	0.01309	0.034513	0.190024

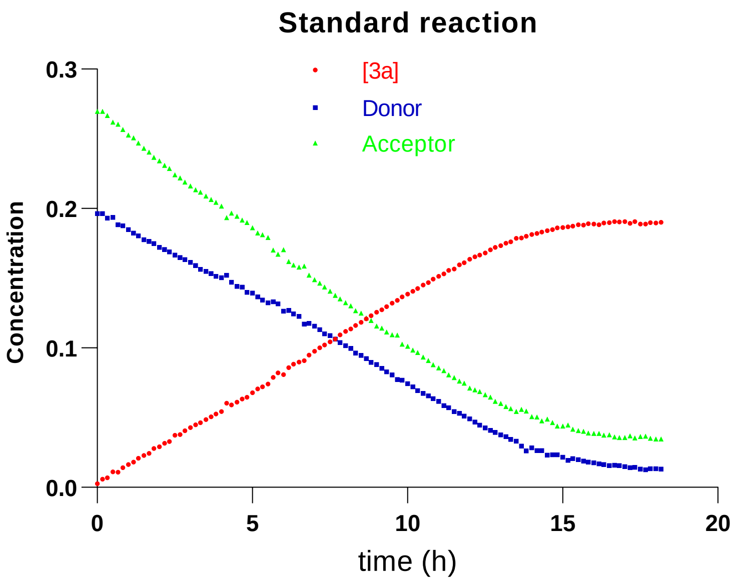

Supplementary Figure 16: Temporal kinetics profile for the monitoring under standard conditions

Concentration dependence studies

Donor 1a concentration dependence (Related to Figure 3e in the manuscript)

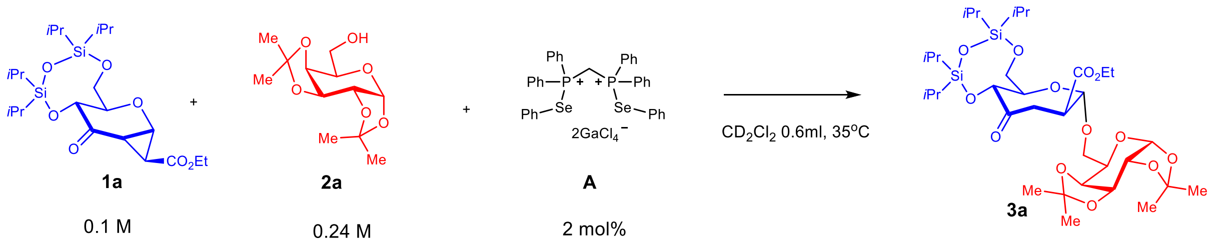

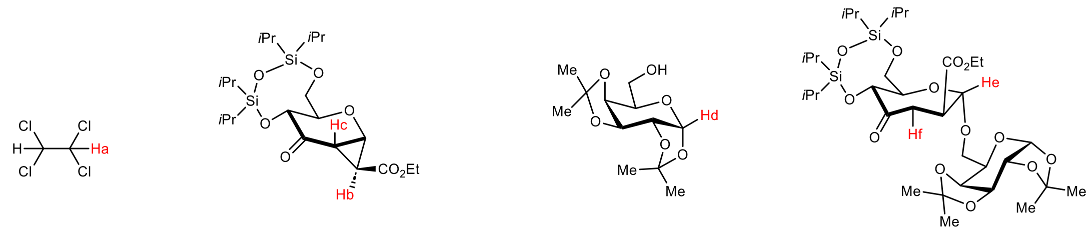

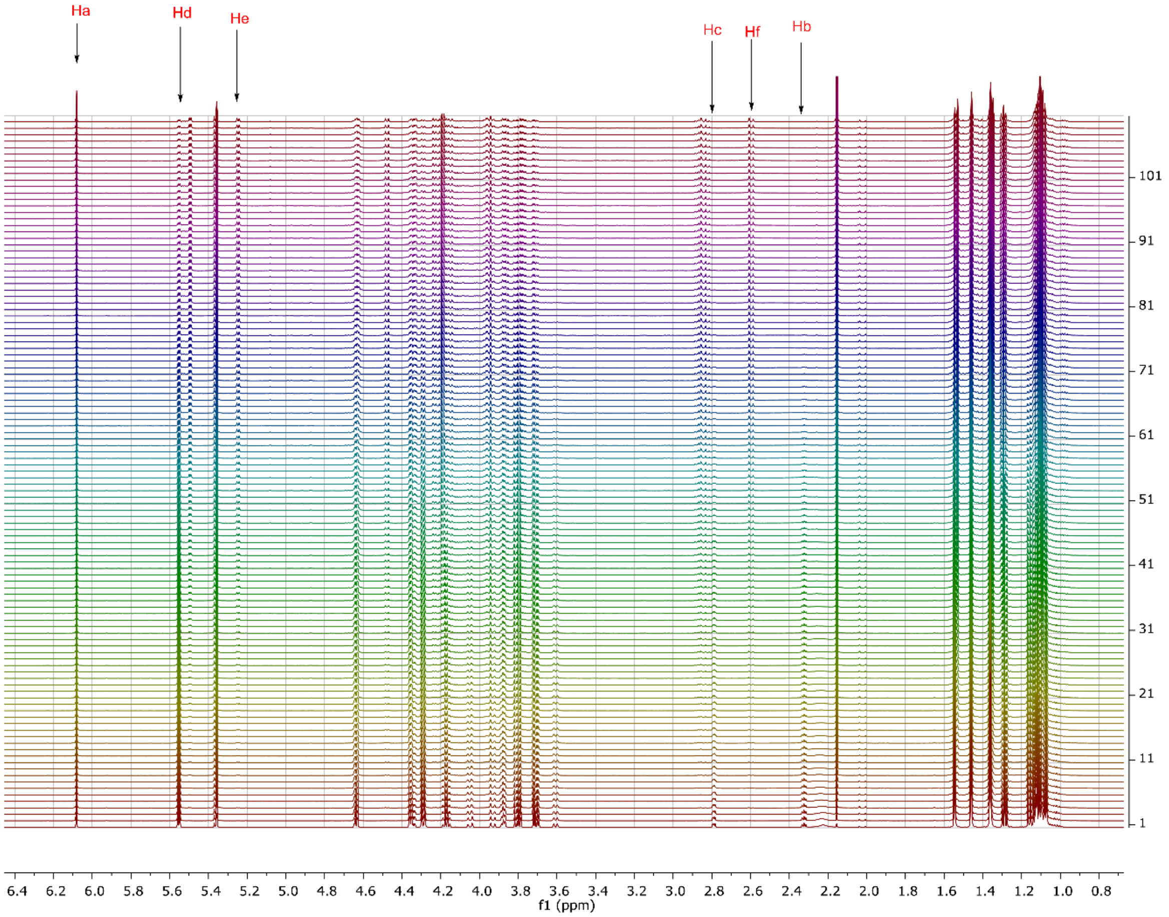

Supplementary Figure 17: Stacked ^{1}H spectra for the experiment of entry [**0.1M**]

Donor 1a concentration dependence (Related to Figure 3e in the manuscript)

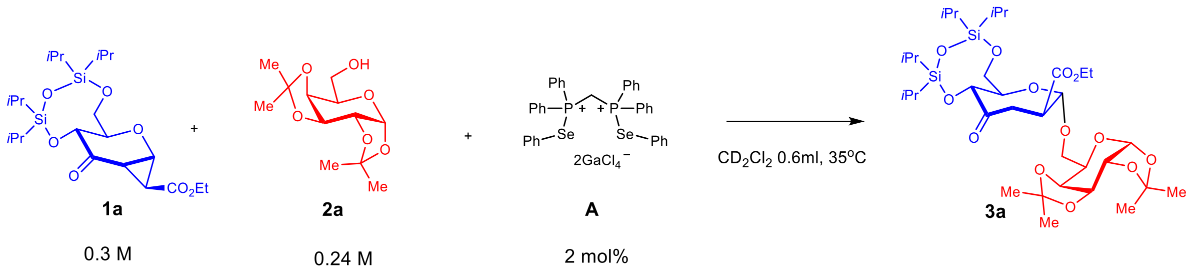

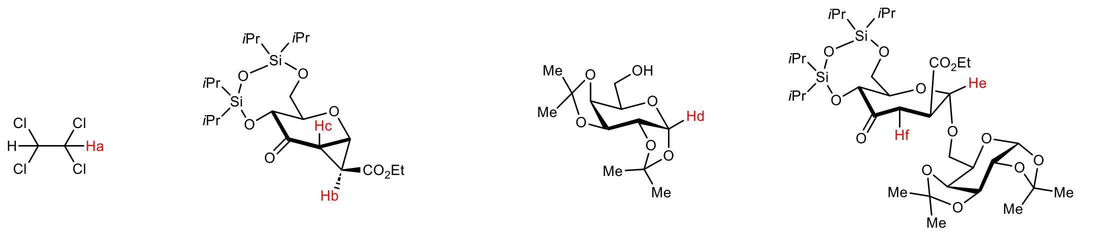

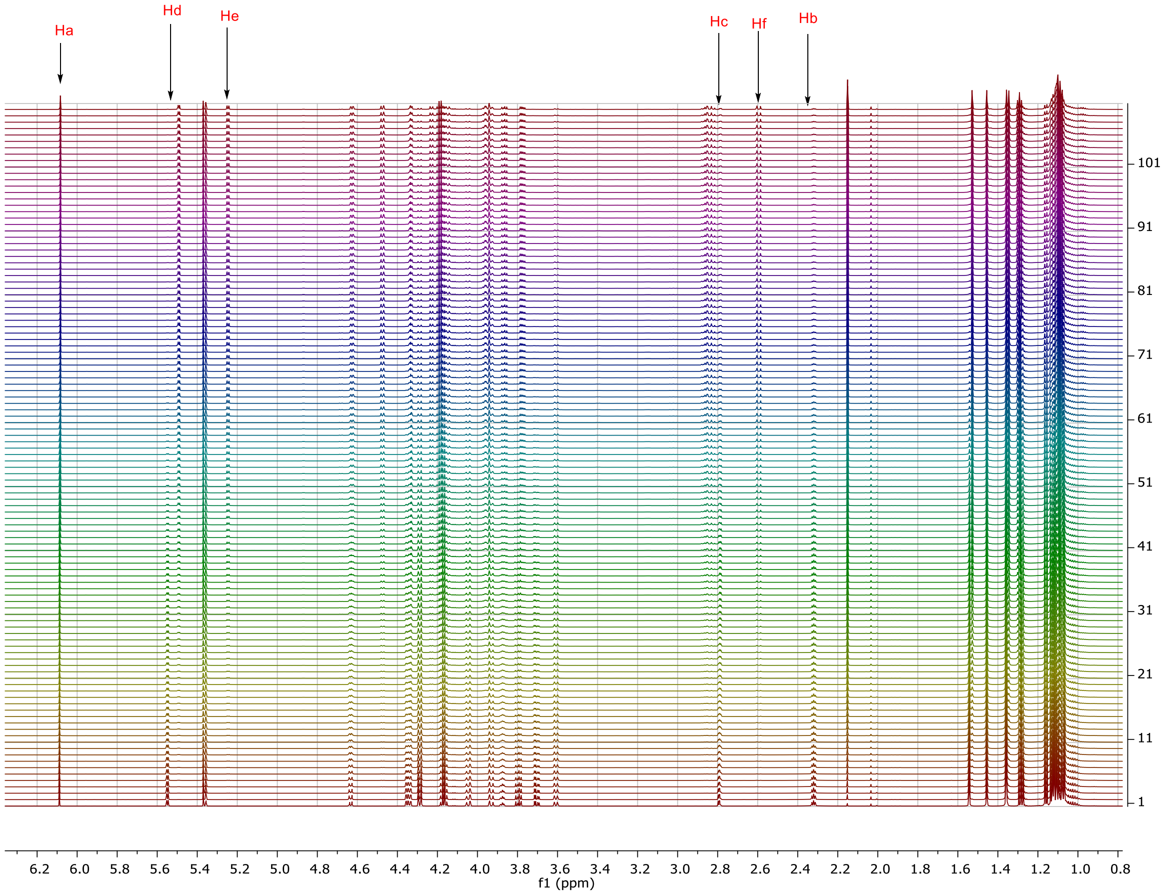

Supplementary Figure 18: Stacked ^{1}H spectra for the experiment of entry [**0.3M**]

Supplementary table 19: Concentration for **3a** calculated by ^{1}H NMR analysis for varying the donor concentration experiment

Time/h	**3a** (**1a** =0.1M)	**3a** (**1a** =0.2M)	**3a** (**1a** =0.3M)
0	0.003675	0.002693	0.003915
0.166667	0.004065	0.005815	0.006435
0.333333	0.005188	0.006923	0.009342
0.5	0.007175	0.011049	0.012217
0.666667	0.00851	0.010903	0.015604
0.833333	0.01124	0.014101	0.018762
1	0.012014	0.016253	0.021348
1.166667	0.013654	0.018136	0.024045
1.333333	0.014808	0.020771	0.027201
1.5	0.016125	0.022917	0.030034
1.666667	0.017209	0.024326	0.033172
1.833333	0.018499	0.027843	0.035966
2	0.019825	0.029223	0.038468
2.166667	0.021355	0.031569	0.041577
2.333333	0.022458	0.032848	0.044247
2.5	0.023392	0.037299	0.047127
2.666667	0.024524	0.037934	0.049933
2.833333	0.025953	0.040542	0.053158
3	0.027061	0.042995	0.056001
3.166667	0.028263	0.044903	0.058887
3.333333	0.029333	0.046365	0.061777
3.5	0.030352	0.048684	0.064472
3.666667	0.03183	0.050651	0.067221
3.833333	0.032534	0.052694	0.069872
4	0.033992	0.054414	0.073192
4.166667	0.035139	0.060307	0.076081
4.333333	0.036862	0.05925	0.079055
4.5	0.037886	0.061238	0.082113
4.666667	0.03931	0.063307	0.085607
4.833333	0.040393	0.064706	0.08858
5	0.041851	0.067788	0.091646
5.166667	0.042822	0.070733	0.095081
5.333333	0.044179	0.072031	0.098406
5.5	0.045471	0.074187	0.101616
5.666667	0.047503	0.078981	0.105041
5.833333	0.04834	0.082242	0.108527
6	0.049618	0.080902	0.112422
6.166667	0.051419	0.085948	0.116103
6.333333	0.052568	0.088408	0.119801
6.5	0.053784	0.089977	0.123805
6.666667	0.055301	0.09094	0.12761

6.833333	0.056735	0.094852	0.131157
7	0.057834	0.097562	0.135208
7.166667	0.059743	0.100237	0.138871
7.333333	0.06103	0.102034	0.142732
7.5	0.06289	0.104455	0.146249
7.666667	0.064246	0.106441	0.150051
7.833333	0.065657	0.109349	0.153416
8	0.066677	0.111934	0.156526
8.166667	0.068088	0.113617	0.159631
8.333333	0.069744	0.116087	0.163126
8.5	0.070995	0.118278	0.165967
8.666667	0.072335	0.120772	0.169137
8.833333	0.073965	0.123166	0.171789
9	0.075378	0.125677	0.174842
9.166667	0.076402	0.127429	0.177304
9.333333	0.077972	0.129692	0.179693
9.5	0.079064	0.132012	0.181937
9.666667	0.080599	0.134053	0.184244
9.833333	0.081508	0.136579	0.186216
10	0.083014	0.138631	0.188665
10.16667	0.084324	0.14056	0.190427
10.33333	0.085232	0.142648	0.192534
10.5	0.086284	0.14509	0.194074
10.66667	0.087593	0.146812	0.19577
10.83333	0.088666	0.14946	0.197634
11	0.089473	0.151462	0.198681
11.16667	0.090724	0.153005	0.20047
11.33333	0.091496	0.155739	0.201833
11.5	0.092737	0.156743	0.202983
11.66667	0.092824	0.159726	0.204114
11.83333	0.093878	0.161073	0.205195
12	0.094693	0.163572	0.205981
12.16667	0.095342	0.16528	0.206635
12.33333	0.095949	0.166657	0.207396
12.5	0.096387	0.168056	0.208176
12.66667	0.097244	0.170291	0.208598
12.83333	0.097589	0.172114	0.209394
13	0.098341	0.173419	0.209781
13.16667	0.0985	0.175143	0.210138
13.33333	0.098723	0.176192	0.2105
13.5	0.099106	0.178582	0.211035
13.66667	0.099321	0.178859	0.211206
13.83333	0.09969	0.180098	0.211653
14	0.099839	0.181313	0.211566

14.16667	0.100194	0.182216	0.212075
14.33333	0.100489	0.18314	0.212323
14.5	0.100345	0.184176	0.21226
14.66667	0.100227	0.184812	0.212279
14.83333	0.100669	0.186186	0.212352
15	0.100429	0.186407	0.212556
15.16667	0.100223	0.186879	0.212539
15.33333	0.100487	0.18743	0.212797
15.5	0.100423	0.188263	0.212787
15.66667	0.100372	0.188148	0.212812
15.83333	0.100146	0.189213	0.213052
16	0.100247	0.188838	0.212873
16.16667	0.099923	0.188386	0.212846
16.33333	0.099744	0.189645	0.212741
16.5	0.099495	0.189793	0.212832
16.66667	0.099218	0.190644	0.212829
16.83333	0.099339	0.190302	0.212928
17	0.098883	0.190675	0.213151
17.16667	0.098987	0.189276	0.212632
17.33333	0.098779	0.190565	0.212431
17.5	0.098372	0.188835	0.212694
17.66667	0.098406	0.188962	0.212501
17.83333	0.097866	0.189852	0.212249
18	0.098219	0.189519	0.212675
18.16667	0.09794	0.190024	0.212632

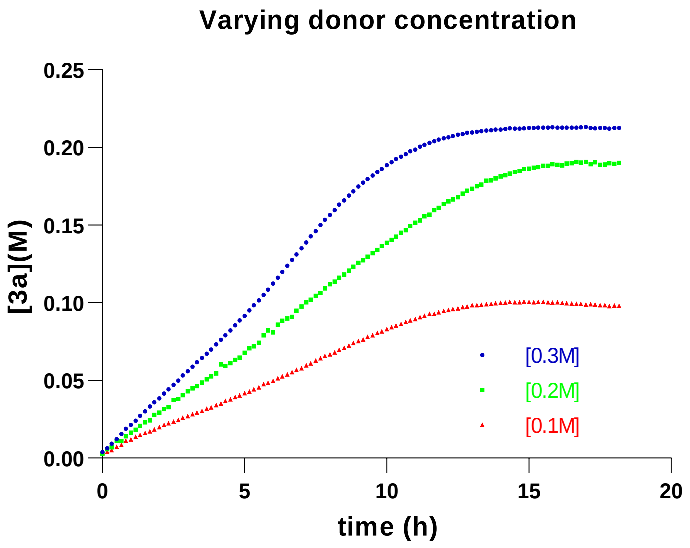

Supplementary Figure 19. Overlapped profile for the donor **1a** concentration dependence experiments

Acceptor 2a concentration dependence (Related to Figure 3e in the manuscript)

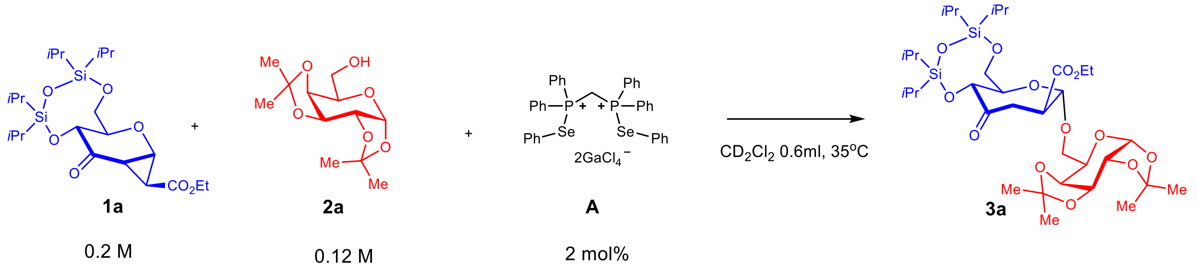

0.2 M 0.12 M 2 mol%

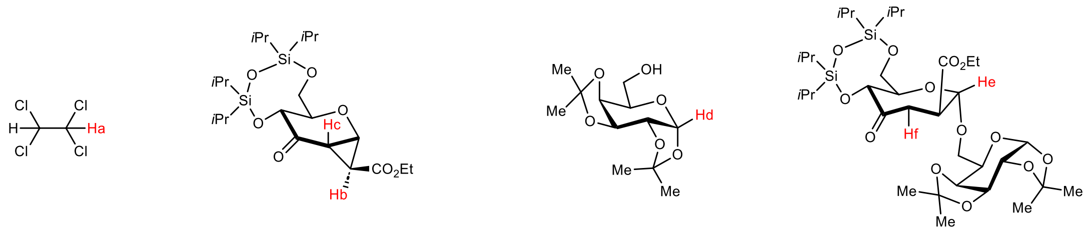

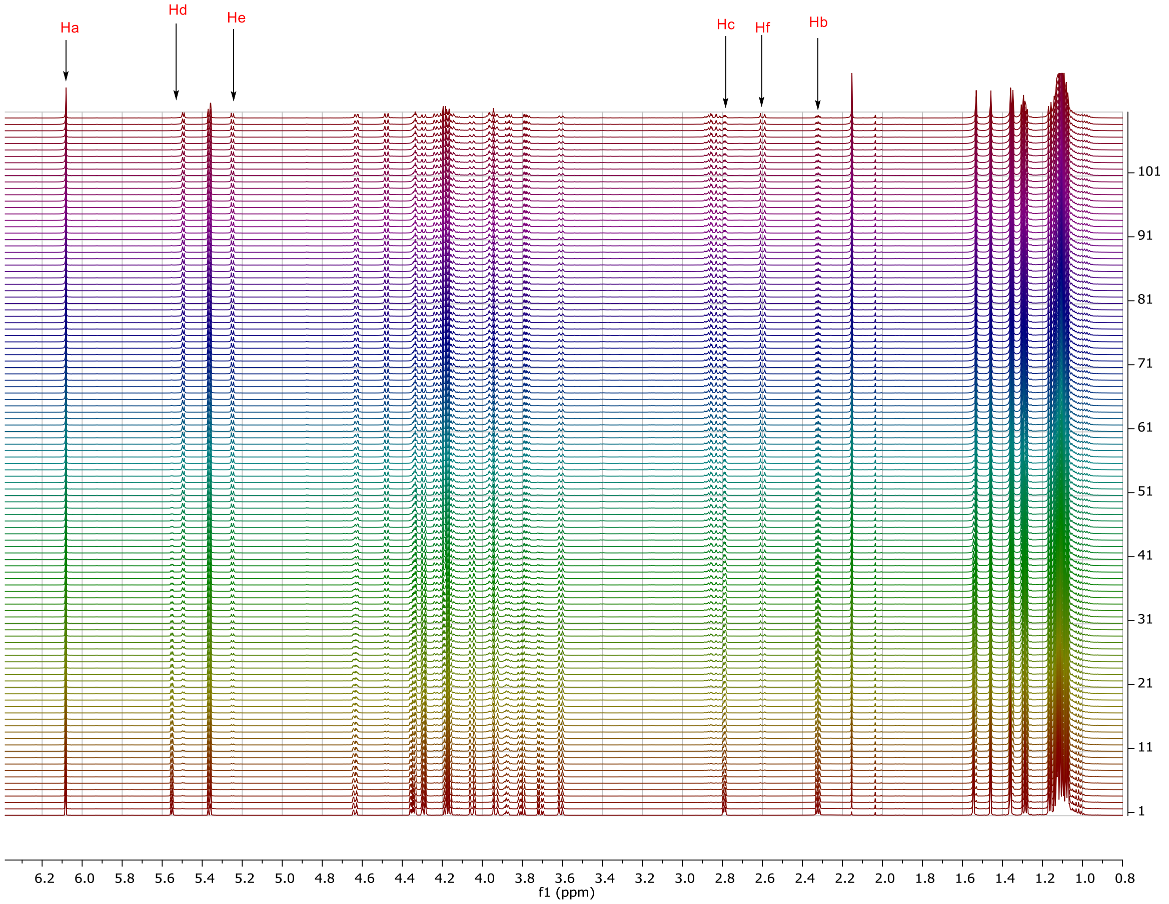

Supplementary Figure 20: Stacked ^{1}H spectra for the experiment of entry [**0.12M**]

Acceptor 2a concentration dependence (Related to Figure 3e in the manuscript)

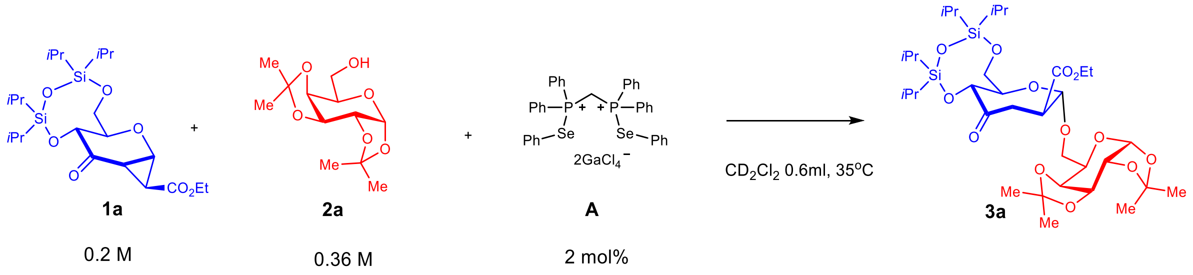

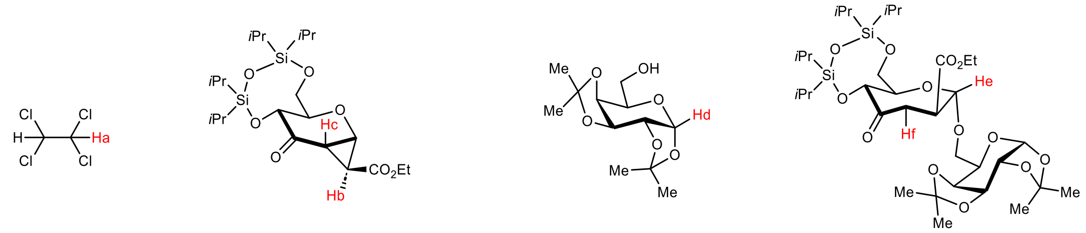

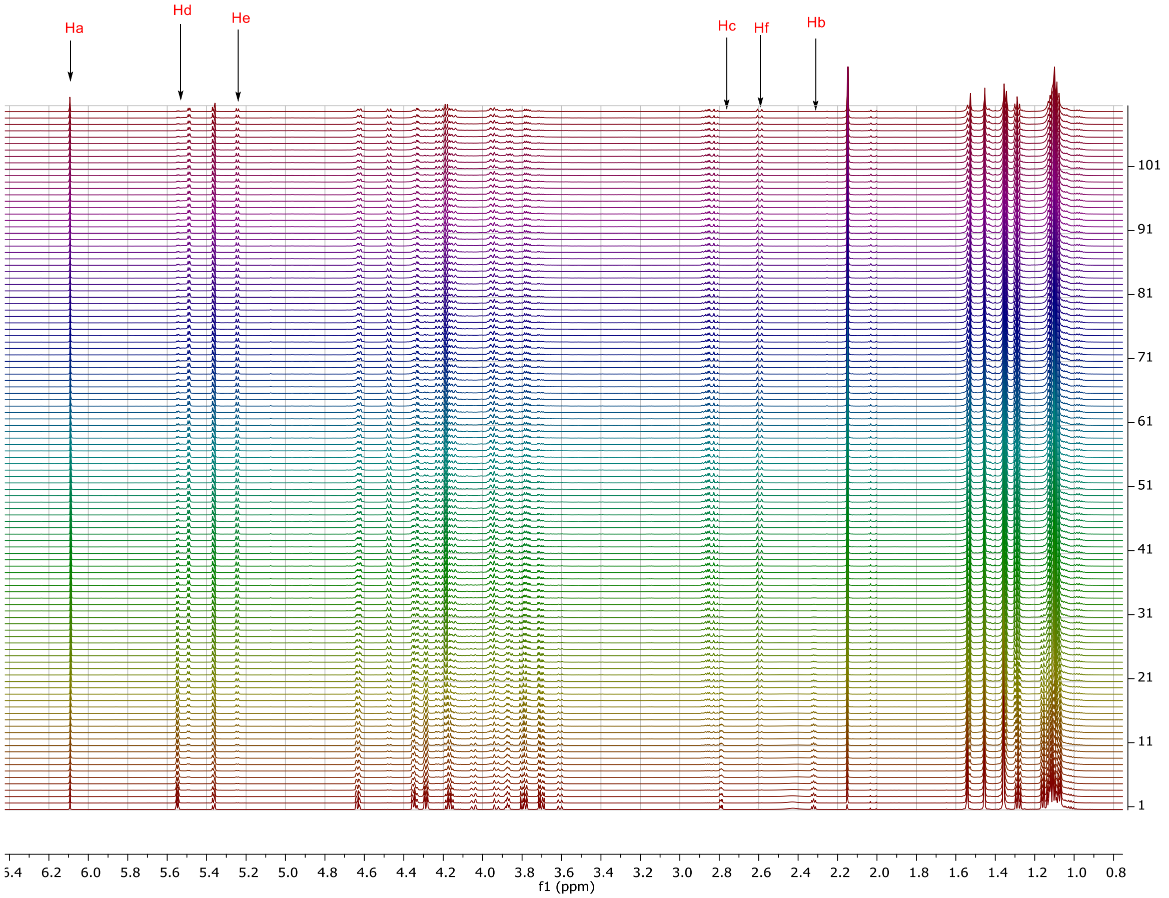

Supplementary Figure 21: Stacked ^{1}H spectra for the experiment of entry [**0.36M**]

Supplementary table 20: Concentration for **3a** calculated by ^{1}H NMR analysis for varying the acceptor concentration experiment

Time/h	**3a** (**2a** =0.12M)	**3a** (**2a** =0.24M)	**3a** (**2a** =0.36M)
0	0.00378158	0.002693315	0.010801235
0.166667	0.005194977	0.005815301	0.014027014
0.333333	0.007543195	0.006922694	0.022183033
0.5	0.009792059	0.011049305	0.028964458
0.666667	0.012279422	0.010902869	0.03597927
0.833333	0.014082536	0.014100672	0.04350344
1	0.016466272	0.016253043	0.050977353
1.166667	0.018187644	0.018135625	0.056196482
1.333333	0.020414153	0.020771339	0.063447916
1.5	0.022062186	0.022917311	0.071018687
1.666667	0.023699255	0.024326221	0.07933508
1.833333	0.025652934	0.027842826	0.087009973
2	0.027549399	0.0292227	0.091548646
2.166667	0.029340497	0.031568781	0.101091163
2.333333	0.031006092	0.032847511	0.108407605
2.5	0.032768416	0.037298716	0.116051309
2.666667	0.034493834	0.037934091	0.123456267
2.833333	0.036524693	0.040541705	0.131121126
3	0.038368999	0.042994623	0.138416943
3.166667	0.040319968	0.044903386	0.144826507
3.333333	0.042154641	0.046365301	0.151694141
3.5	0.043746009	0.048684171	0.158808443
3.666667	0.045794263	0.050651327	0.165500622
3.833333	0.047461593	0.052693564	0.170433733
4	0.0498106	0.054413637	0.176015862
4.166667	0.051746416	0.060306879	0.180134416
4.333333	0.053881056	0.059250209	0.184360134
4.5	0.056261173	0.061238217	0.189771461
4.666667	0.058341923	0.063307282	0.192771878
4.833333	0.060848429	0.064705746	0.196208088
5	0.062831199	0.067787836	0.198707449
5.166667	0.065623179	0.070732764	0.201386288
5.333333	0.067572029	0.072030589	0.203131362
5.5	0.070008015	0.074186652	0.206636801
5.666667	0.072418923	0.078981146	0.208314368
5.833333	0.074591892	0.082241548	0.208668058
6	0.076567667	0.080902151	0.211658157
6.166667	0.078574802	0.085947529	0.213086234
6.333333	0.08090684	0.088408329	0.214568892
6.5	0.082426662	0.089977303	0.214921487
6.666667	0.084485905	0.090940455	0.215217141

6.833333	0.086296396	0.094851701	0.21676764
7	0.087977927	0.097562138	0.217586944
7.166667	0.089608649	0.100236937	0.217767487
7.333333	0.090898986	0.102033933	0.218300977
7.5	0.09228213	0.104455337	0.218573682
7.666667	0.094046956	0.106441389	0.218558526
7.833333	0.09480366	0.109348543	0.218833952
8	0.096676746	0.111934228	0.219416252
8.166667	0.097053978	0.113617089	0.219559789
8.333333	0.098699669	0.11608739	0.219266341
8.5	0.099058716	0.118278441	0.220208347
8.666667	0.100324857	0.120771894	0.220576895
8.833333	0.100716521	0.123165839	0.219908811
9	0.101247292	0.12567651	0.219509985
9.166667	0.102222116	0.127428865	0.224737818
9.333333	0.102073912	0.129692024	0.219791175
9.5	0.103025569	0.132011854	0.224318179
9.666667	0.103252541	0.134052549	0.224447946
9.833333	0.10307438	0.13657861	0.224632197
10	0.103885594	0.138630982	0.223917689
10.16667	0.103862448	0.14055975	0.222861174
10.33333	0.104392538	0.142647833	0.223142152
10.5	0.104016384	0.145089615	0.217293993
10.66667	0.104001484	0.146811555	0.215747143
10.83333	0.104442535	0.149459836	0.215762956
11	0.105011656	0.151462326	0.22068371
11.16667	0.104684767	0.153004562	0.221157789
11.33333	0.105973988	0.155739275	0.220343716
11.5	0.104292751	0.156743071	0.214741759
11.66667	0.104696653	0.15972629	0.218988175
11.83333	0.104264446	0.161072693	0.218002604
12	0.106120525	0.163572397	0.21312816
12.16667	0.104849572	0.165279565	0.211858065
12.33333	0.104580625	0.166657117	0.212028297
12.5	0.104781213	0.168055595	0.215018906
12.66667	0.105574573	0.170291383	0.215684296
12.83333	0.10614532	0.172114337	0.21087399
13	0.10566062	0.173419462	0.20917135
13.16667	0.1046751	0.175143086	0.212821211
13.33333	0.104302757	0.17619212	0.207319055
13.5	0.105611839	0.178581896	0.206916705
13.66667	0.105601062	0.178858772	0.212744041
13.83333	0.105119163	0.180098259	0.20776713
14	0.103922608	0.18131303	0.20779292

14.16667	0.104084961	0.182216364	0.206958291
14.33333	0.105235204	0.183140352	0.2064291
14.5	0.103988898	0.184176102	0.205319124
14.66667	0.103945479	0.184812222	0.205133029
14.83333	0.103905749	0.186186472	0.204504339
15	0.105025035	0.186406949	0.203587126
15.16667	0.10513795	0.186878681	0.203575565
15.33333	0.104910505	0.18742963	0.203417284
15.5	0.105595518	0.18826344	0.202329774
15.66667	0.103031716	0.188148209	0.202363087
15.83333	0.104639838	0.189213466	0.202135068
16	0.104204511	0.188837826	0.201608379
16.16667	0.10427097	0.188385857	0.201048298
16.33333	0.103710317	0.189644661	0.200368921
16.5	0.102997327	0.189793257	0.199911271
16.66667	0.103967864	0.190644111	0.200315683
16.83333	0.103974867	0.190301721	0.199656388
17	0.103552161	0.190675263	0.198299881
17.16667	0.104463532	0.189276301	0.198490462
17.33333	0.10305636	0.190564981	0.197982293
17.5	0.103109956	0.188834713	0.198034271
17.66667	0.103559747	0.188962371	0.196952793
17.83333	0.102371318	0.18985169	0.197206597
18	0.102230147	0.189519288	0.196486334
18.16667	0.100038577	0.190023506	0.195991157

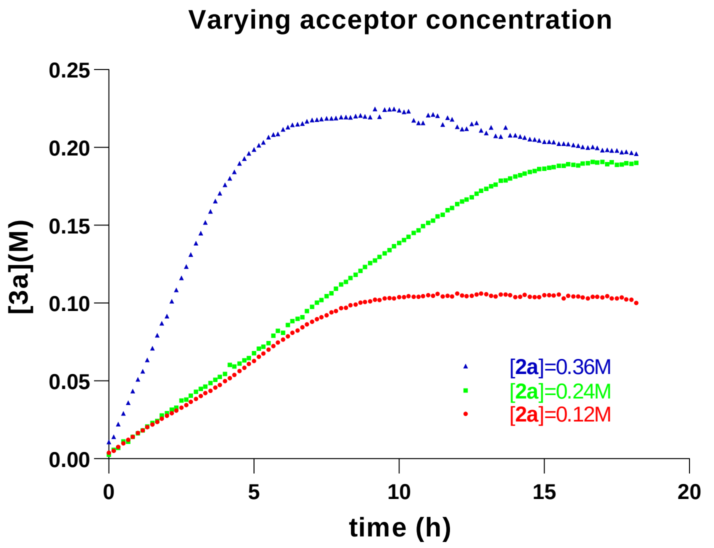

Supplementary Figure 22. Overlapped profile for the acceptor **2a** concentration dependence experiments

Catalyst A concentration dependence (Related to Figure 3e in the manuscript)

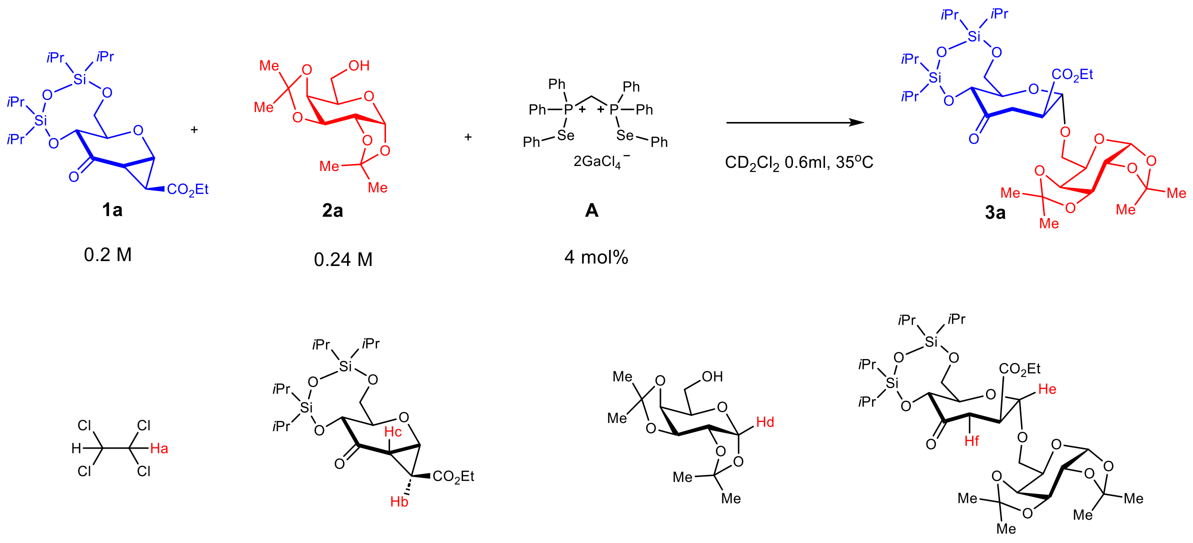

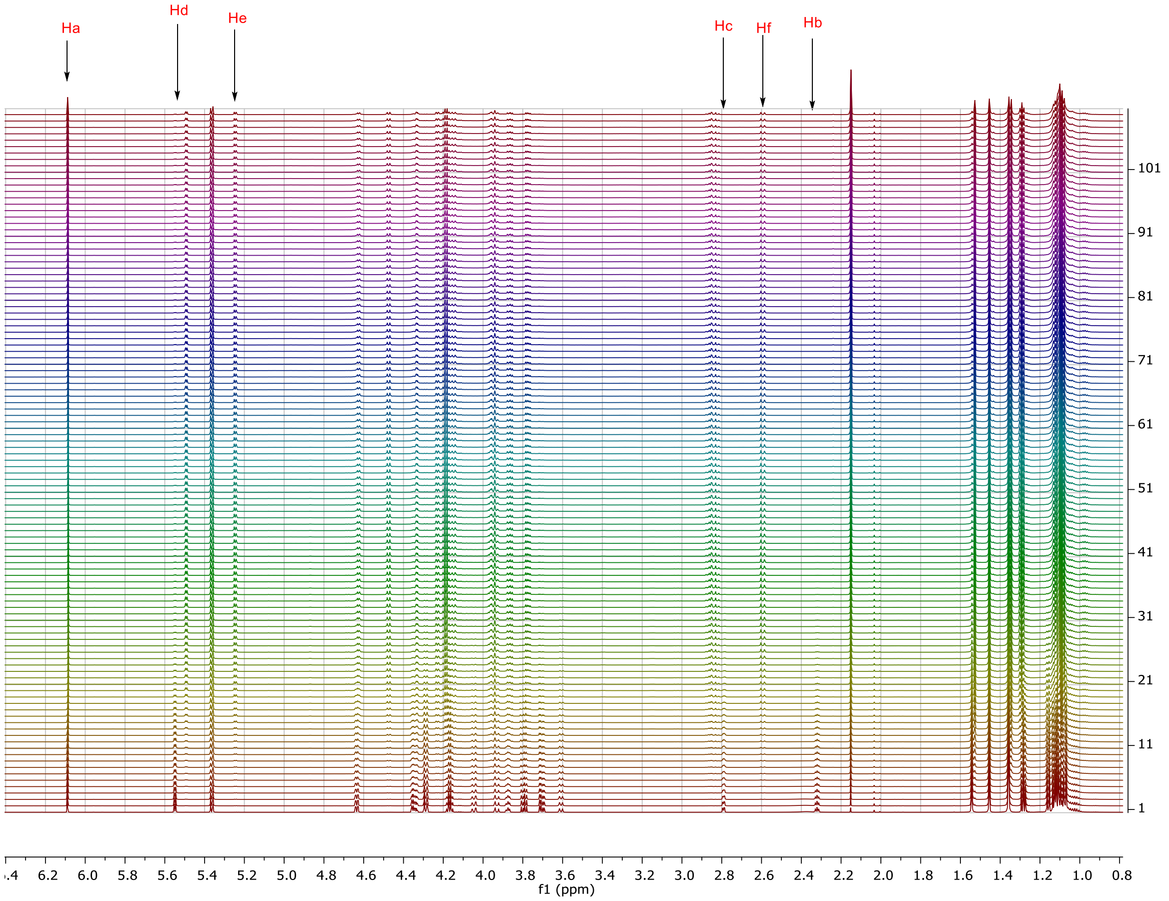

Supplementary Figure 23: Stacked ^{1}H spectra for the experiment of entry [**4 mol%**]

Catalyst concentration dependence (Related to Figure 3e in the manuscript)

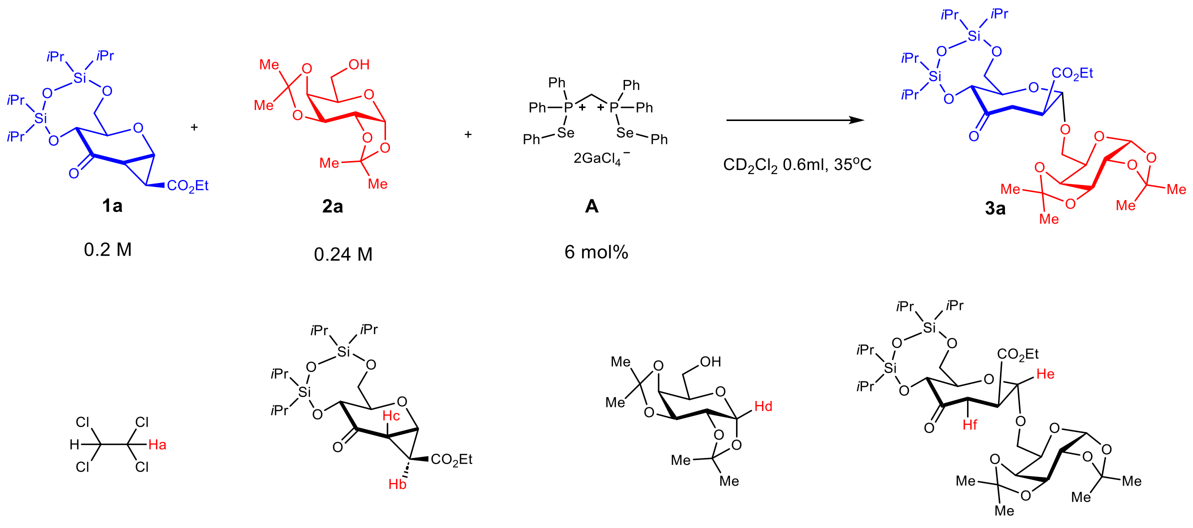

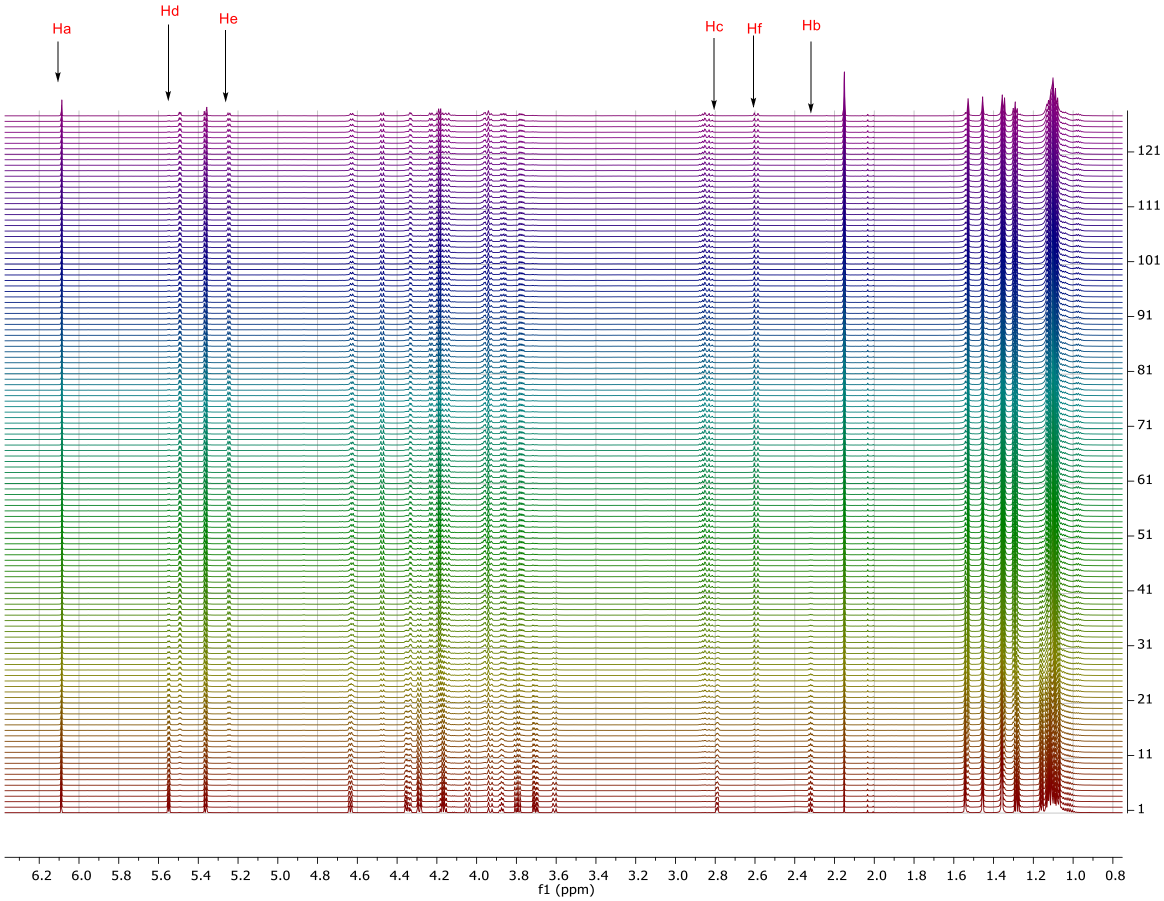

Supplementary Figure 24: Stacked ^{1}H spectra for the experiment of entry [**6 mol%**]

Supplementary table 21: Concentration for **3a** calculated by ^{1}H NMR analysis for varying the catalyst concentration experiment

Time/h	**3a** (**A** =2.0 mol%)	**3a** (**A** =4.0 mol%)	**3a** (**A** =6.0 mol%)
0	0.004225373	0.002693315	0.008194797
0.083333			0.010771854
0.166667	0.008582359	0.005815301	0.01416618
0.25			0.01711825
0.333333	0.012485092	0.006922694	0.021272352
0.416667			0.025311106
0.5	0.017800438	0.011049305	0.028401702
0.583333			0.032862105
0.666667	0.022500265	0.010902869	0.037098575
0.75			0.04209618
0.833333	0.028679623	0.014100672	0.046355356
0.916667			0.051177726
1	0.037587508	0.016253043	0.056101461
1.083333			0.061797527
1.166667	0.042438339	0.018135625	0.066421344
1.25			0.071885958
1.333333	0.050022659	0.020771339	0.077472369
1.416667			0.083326757
1.5	0.05524466	0.022917311	0.089178573
1.583333			0.095717597
1.666667	0.061316276	0.024326221	0.101368129
1.75			0.107353703
1.833333	0.070106945	0.027842826	0.11385403
1.916667			0.118670855
2	0.080431347	0.0292227	0.124611979
2.083333			0.129585118
2.166667	0.087936158	0.031568781	0.134261351
2.25			0.141018793
2.333333	0.097689786	0.032847511	0.144920777
2.416667			0.148913443
2.5	0.106316461	0.037298716	0.152678178
2.583333			0.15393044
2.666667	0.117968852	0.037934091	0.156658759
2.75			0.159889873
2.833333	0.126866967	0.040541705	0.162218923
2.916667			0.164087865
3	0.135994183	0.042994623	0.166203999
3.083333			0.167922656
3.166667	0.143944323	0.044903386	0.169722838
3.25			0.170803234
3.333333	0.14833363	0.046365301	0.172280166

3.416667			0.173189252
3.5	0.154130871	0.048684171	0.174394226
3.583333			0.176490072
3.666667	0.159241724	0.050651327	0.177062802
3.75			0.177599766
3.833333	0.163479273	0.052693564	0.178800789
3.916667			0.179750576
4	0.166962454	0.054413637	0.179928503
4.083333			0.179917213
4.166667	0.169616507	0.060306879	0.18063904
4.25			0.179490903
4.333333	0.172183016	0.059250209	0.18002502
4.416667			0.179707098
4.5	0.173238762	0.061238217	0.180036063
4.583333			0.17948795
4.666667	0.175183367	0.063307282	0.17995882
4.75			0.179686606
4.833333	0.175915688	0.064705746	0.1798389
4.916667			0.179857519
5	0.176696026	0.067787836	0.179464962
5.083333			0.179317484
5.166667	0.177975479	0.070732764	0.17956933
5.25			0.180568563
5.333333	0.17825759	0.072030589	0.179145841
5.416667			0.178788612
5.5	0.178027116	0.074186652	0.178535358
5.583333			0.180427988
5.666667	0.17856111	0.078981146	0.180253774
5.75			0.17872008
5.833333	0.178520822	0.082241548	0.179539131
5.916667			0.179692339
6	0.178410506	0.080902151	0.177315615
6.083333			0.177667859
6.166667	0.178695308	0.085947529	0.177628793
6.25			0.177683563
6.333333	0.180411609	0.088408329	0.176248167
6.416667			0.177708981
6.5	0.177401558	0.089977303	0.176701742
6.583333			0.176974552
6.666667	0.177015355	0.090940455	0.176577706
6.75			0.176888764
6.833333	0.176577214	0.094851701	0.176877153
6.916667			0.175750111
7	0.179263647	0.097562138	0.176581374

7.083333			0.175907008
7.166667	0.175799406	0.100236937	0.174062655
7.25			0.175029979
7.333333	0.175250109	0.102033933	0.174128409
7.416667			0.17396018
7.5	0.178751333	0.104455337	0.17436292
7.583333			0.172909052
7.666667	0.177509443	0.106441389	0.173785502
7.75			0.172291867
7.833333	0.17387204	0.109348543	0.17336977
7.916667			0.173568755
8	0.17352954	0.111934228	0.173610818
8.083333			0.172665208
8.166667	0.173419901	0.113617089	0.172186381
8.25			0.172358875
8.333333	0.172150516	0.11608739	0.17188676
8.416667			0.171191191
8.5	0.17196098	0.118278441	0.171148891
8.583333			0.170858927
8.666667	0.174682729	0.120771894	0.17060116
8.75			0.170194416
8.833333	0.168371468	0.123165839	0.169874739
8.916667			0.169159783
9	0.168543417	0.12567651	0.169867364
9.083333			0.169497319
9.166667	0.170204526	0.127428865	0.168789573
9.25			0.168069576
9.333333	0.166527102	0.129692024	0.167029669
9.416667			0.168184256
9.5	0.165893583	0.132011854	0.166441639
9.583333			0.167441092
9.666667	0.166729032	0.134052549	0.166144915
9.75			0.165355698
9.833333	0.170379466	0.13657861	0.166187959
9.916667			0.166168429
10	0.164679318	0.138630982	0.166177876
10.08333			0.165900929
10.16667	0.163819037	0.14055975	0.165152859
10.25			0.164142386
10.33333	0.161773454	0.142647833	0.164412095
10.41667			0.164815162
10.5	0.163220016	0.145089615	0.163308829
10.58333			0.162607655
10.66667	0.162047401	0.146811555	0.162609926

10.75			0.162962616
10.83333	0.166150093	0.149459836	0.16297405
10.91667			0.161521763
11	0.161209095	0.151462326	0.162190805
11.08333			0.161121055
11.16667	0.159035005	0.153004562	0.160560215
11.25			0.160143547
11.33333	0.162749682	0.155739275	0.159627665
11.41667			0.159737722
11.5	0.163466335	0.156743071	0.15963012
11.58333			0.159109593
11.66667	0.162524014	0.15972629	0.158811685
11.75			0.157920574
11.83333	0.162048919	0.161072693	0.158762637
11.91667			0.157581211
12	0.155763269	0.163572397	0.157667022
12.08333			0.157616151
12.16667	0.16094927	0.165279565	0.156457206
12.25			0.155511128
12.33333	0.16002505	0.166657117	
12.5	0.154656173	0.168055595	
12.66667	0.15298463	0.170291383	
12.83333	0.15360235	0.172114337	
13	0.157885527	0.173419462	
13.16667	0.152551902	0.175143086	
13.33333	0.151007315	0.17619212	
13.5	0.150630233	0.178581896	
13.66667	0.153304165	0.178858772	
13.83333	0.150656861	0.180098259	
14	0.152067397	0.18131303	
14.16667	0.151485991	0.182216364	
14.33333	0.148437077	0.183140352	
14.5	0.148415379	0.184176102	
14.66667	0.152200651	0.184812222	
14.83333	0.151466061	0.186186472	
15	0.142249513	0.186406949	
15.16667	0.148367945	0.186878681	
15.33333	0.149693497	0.18742963	
15.5	0.147627068	0.18826344	
15.66667	0.14719066	0.188148209	
15.83333	0.146291827	0.189213466	
16	0.142792886	0.188837826	
16.16667	0.141777268	0.188385857	
16.33333	0.140020901	0.189644661	

16.5	0.145720226	0.189793257	
16.66667	0.140721294	0.190644111	
16.83333	0.1398925	0.190301721	
17	0.13982068	0.190675263	
17.16667	0.137846759	0.189276301	
17.33333	0.136962039	0.190564981	
17.5	0.135277446	0.188834713	
17.66667	0.134556212	0.188962371	
17.83333	0.134796397	0.18985169	
18	0.136737997	0.189519288	
18.16667	0.136056449	0.190023506	

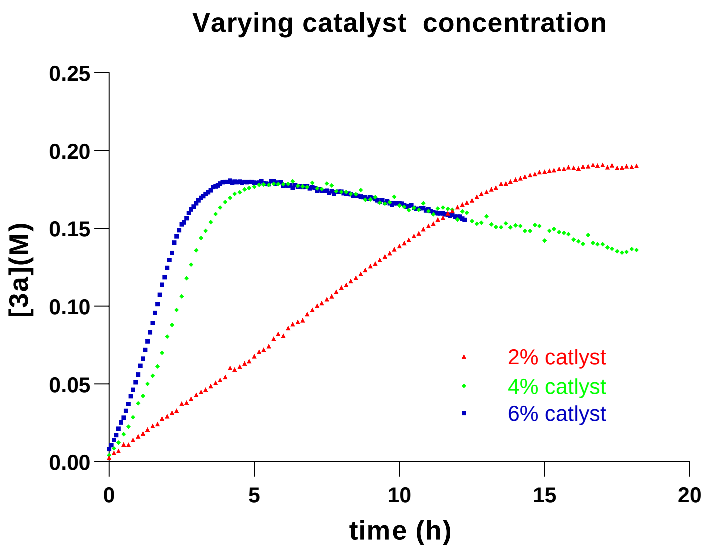

Supplementary Figure 25. Overlapped profile for the catalyst **A** concentration dependence experiments

Kinetic Analysis

Burés method to determine order with respect to catalyst **A**[19,20].

Supplementary Table 22. Concentration for **3a** & Product formation over time multiplied by [catalyst] to the 1.4 power.

	2.0 mol% catalyst **A**		4.0 mol% catalyst **A**		6.0 mol% catalyst **A**	
Time/min	t[cat]$^{1.4}$	[**3a**]	t[**cat**]$^{1.4}$	[**3a**]	t[**cat**]$^{1.4}$	[**3a**]
0	0	0.002693315	0	0.004225373	0	0.008194797
10	0.004394242	0.005815301	0.011596475	0.008582359	0.020457556	0.01416618
20	0.008788484	0.006922694	0.023192949	0.012485092	0.040915112	0.021272352
30	0.013182727	0.011049305	0.034789424	0.017800438	0.061372667	0.028401702
40	0.017576969	0.010902869	0.046385898	0.022500265	0.081830223	0.037098575
50	0.021971211	0.014100672	0.057982373	0.028679623	0.102287779	0.046355356
60	0.026365453	0.016253043	0.069578848	0.037587508	0.122745335	0.056101461
70	0.030759695	0.018135625	0.081175322	0.042438339	0.143202891	0.066421344
80	0.035153937	0.020771339	0.092771797	0.050022659	0.163660446	0.077472369
90	0.03954818	0.022917311	0.104368272	0.05524466	0.184118002	0.089178573
100	0.043942422	0.024326221	0.115964746	0.061316276	0.204575558	0.101368129
110	0.048336664	0.027842826	0.127561221	0.070106945	0.225033114	0.11385403
120	0.052730906	0.0292227	0.139157695	0.080431347	0.24549067	0.124611979
130	0.057125148	0.031568781	0.15075417	0.087936158	0.265948225	0.134261351
140	0.06151939	0.032847511	0.162350645	0.097689786	0.286405781	0.144920777
150	0.065913633	0.037298716	0.173947119	0.106316461	0.306863337	0.152678178
160	0.070307875	0.037934091	0.185543594	0.117968852	0.327320893	0.156658759
170	0.074702117	0.040541705	0.197140069	0.126866967	0.347778449	0.162218923
180	0.079096359	0.042994623	0.208736543	0.135994183	0.368236004	0.166203999
190	0.083490601	0.044903386	0.220333018	0.143944323	0.38869356	0.169722838
200	0.087884843	0.046365301	0.231929492	0.14833363	0.409151116	0.172280166
210	0.092279086	0.048684171	0.243525967	0.154130871	0.429608672	0.174394226
220	0.096673328	0.050651327	0.255122442	0.159241724	0.450066228	0.177062802
230	0.10106757	0.052693564	0.266718916	0.163479273	0.470523783	0.178800789
240	0.105461812	0.054413637	0.278315391	0.166962454	0.490981339	0.179928503
250	0.109856054	0.060306879	0.289911865	0.169616507	0.511438895	0.18063904
260	0.114250297	0.059250209	0.30150834	0.172183016		
270	0.118644539	0.061238217	0.313104815	0.173238762		
280	0.123038781	0.063307282	0.324701289	0.175183367		
290	0.127433023	0.064705746	0.336297764	0.175915688		
300	0.131827265	0.067787836	0.347894239	0.176696026		
310	0.136221507	0.070732764	0.359490713	0.177975479		
320	0.14061575	0.072030589	0.371087188	0.17825759		
330	0.145009992	0.074186652	0.382683662	0.178027116		
340	0.149404234	0.078981146	0.394280137	0.17856111		
350	0.153798476	0.082241548	0.405876612	0.178520822		
360	0.158192718	0.080902151	0.417473086	0.178410506		
370	0.16258696	0.085947529	0.429069561	0.178695308		

380	0.166981203	0.088408329	0.440666036	0.180411609
390	0.171375445	0.089977303		
400	0.175769687	0.090940455		
410	0.180163929	0.094851701		
420	0.184558171	0.097562138		
430	0.188952413	0.100236937		
440	0.193346656	0.102033933		
450	0.197740898	0.104455337		
460	0.20213514	0.106441389		
470	0.206529382	0.109348543		
480	0.210923624	0.111934228		
490	0.215317866	0.113617089		
500	0.219712109	0.11608739		
510	0.224106351	0.118278441		
520	0.228500593	0.120771894		
530	0.232894835	0.123165839		
540	0.237289077	0.12567651		
550	0.24168332	0.127428865		
560	0.246077562	0.129692024		
570	0.250471804	0.132011854		
580	0.254866046	0.134052549		
590	0.259260288	0.13657861		
600	0.26365453	0.138630982		
610	0.268048773	0.14055975		
620	0.272443015	0.142647833		
630	0.276837257	0.145089615		
640	0.281231499	0.146811555		
650	0.285625741	0.149459836		
660	0.290019983	0.151462326		
670	0.294414226	0.153004562		
680	0.298808468	0.155739275		
690	0.30320271	0.156743071		
700	0.307596952	0.15972629		
710	0.311991194	0.161072693		
720	0.316385436	0.163572397		
730	0.320779679	0.165279565		
740	0.325173921	0.166657117		
750	0.329568163	0.168055595		
760	0.333962405	0.170291383		
770	0.338356647	0.172114337		
780	0.34275089	0.173419462		
790	0.347145132	0.175143086		
800	0.351539374	0.17619212		
810	0.355933616	0.178581896		

820	0.360327858	0.178858772
830	0.3647221	0.180098259
840	0.369116343	0.18131303
850	0.373510585	0.182216364
860	0.377904827	0.183140352
870	0.382299069	0.184176102
880	0.386693311	0.184812222
890	0.391087553	0.186186472
900	0.395481796	0.186406949
910	0.399876038	0.186878681
920	0.40427028	0.18742963
930	0.408664522	0.18826344
940	0.413058764	0.188148209
950	0.417453006	0.189213466
960	0.421847249	0.188837826
970	0.426241491	0.188385857
980	0.430635733	0.189644661
990	0.435029975	0.189793257
1000	0.439424217	0.190644111
1010	0.443818459	0.190301721
1020	0.448212702	0.190675263
1030	0.452606944	0.189276301
1040	0.457001186	0.190564981
1050	0.461395428	0.188834713
1060	0.46578967	0.188962371
1070	0.470183913	0.18985169
1080	0.474578155	0.189519288
1090	0.478972397	0.190023506

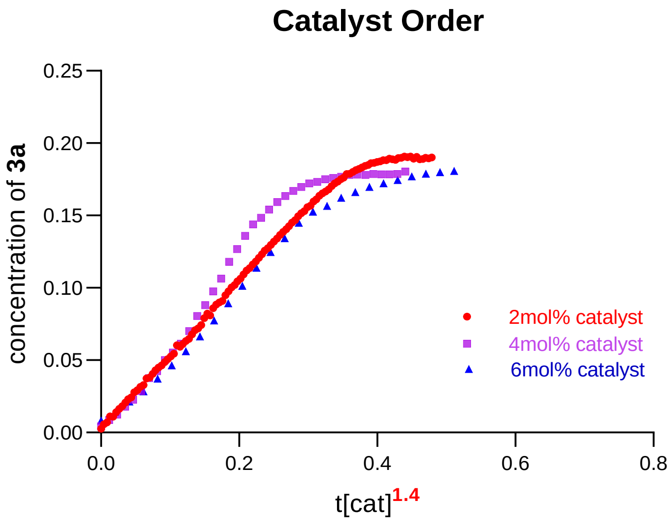

Supplementary Figure 26. Product formation over time multiplied by [**catalyst A**] to the 1.4 power. Graphical overlay represents a 1.4 dependence.

Supplementary Table 23. Initial rate of donor **1a**

Time/min	[Donor **1a**]$_{initial}$ (0.1M)	[Donor **1a**]$_{initial}$ (0.2M)	[Donor **1a**]$_{initial}$ (0.3M)
10	0.1	0.2	0.3
20	0.097894266	0.197820553	0.297233254
30	0.097132583	0.19720526	0.294521251
40	0.095912876	0.19360887	0.29178559
50	0.097165922	0.192396838	0.2883384
60	0.096252231	0.190554587	0.28642977
70	0.095042838	0.187688105	0.283332312
80	0.094193431	0.186544821	0.280327108

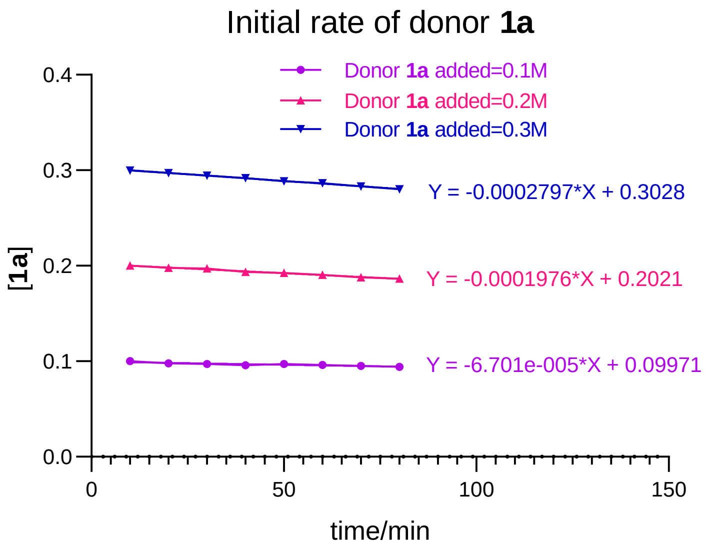

Supplementary Figure 27. Initial rate of donor **1a**.

Supplementary Table 24. Simple linear regression of Initial donor rate.

	[Donor **1a**]$_{initial}$ (0.1M)	[Donor **1a**]$_{initial}$ (0.2M)	[Donor **1a**]$_{initial}$ (0.3M)
**Best-fit values**			
Slope	-6.701e-005	-0.0001976	-0.0002797
Y-intercept	0.09971	0.2021	0.3028
X-intercept	1488	1023	1083
1/slope	-14922	-5060	-3575
**Std. Error**			
Slope	1.213e-005	9.041e-006	4.242e-006
Y-intercept	0.0006126	0.0004565	0.0002142
**95% Confidence Intervals**			
Slope	-9.670e-005 to -3.733e-005	-0.0002198 to -0.0001755	-0.0002901 to -0.0002693
Y-intercept	0.09822 to 0.1012	0.2010 to 0.2032	0.3023 to 0.3034
X-intercept	1045 to 2635	924.2 to 1146	1046 to 1123

Goodness of Fit			
R squared	0.8357	0.9876	0.9986
Sy.x	0.0007862	0.0005859	0.0002749
**Is slope significantly non-zero?**			
F	30.52	477.8	4346
DFn, DFd	1, 6	1, 6	1, 6
P value	0.0015	<0.0001	<0.0001
Deviation from zero?	Significant	Significant	Significant
Equation	Y = -6.701e-005*X + 0.09971	Y = -0.0001976*X + 0.2021	Y = -0.0002797*X + 0.3028
**Data**			
Number of X values	8	8	8
Maximum number of Y replicates	1	1	1
Total number of values	8	8	8
Number of missing values	0	0	0

Supplementary Table 25. Determination of order with respect to donor **1a**.

Entry	Concentration	$[\mathbf{1a}]^{0.6}$	Reaction rate
1	0.1M	0.251188643	-0.00006701 mmol/min
2	0.2M	0.380730788	-0.0001976 mmol/min
3	0.3M	0.485593375	-0.0002979 mmol/min

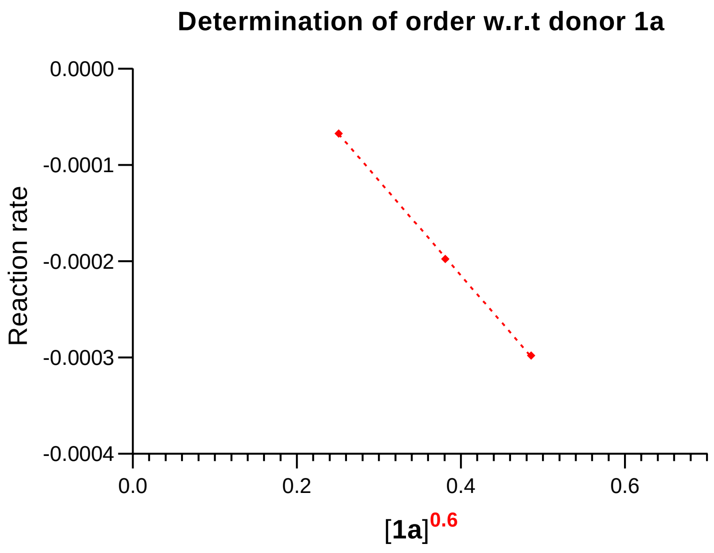

Supplementary Figure 28. Determination of order w.r.t **1a** using initial rate method

Supplementary Table 26. Initial rate of acceptor **2a**.

Time/min	[**Acceptor 2a**]$_{initial}$ (0.12M)	[**Acceptor 2a**]$_{initial}$ (0.24M)	[**Acceptor 2a**]$_{initial}$ (0.36M)
10	0.12	0.24	0.36
20	0.115903043	0.216138398	0.348679516
30	0.113486408	0.212176776	0.338207519
40	0.109921916	0.211112399	0.327894504
50	0.107328748	0.207934753	0.317683553

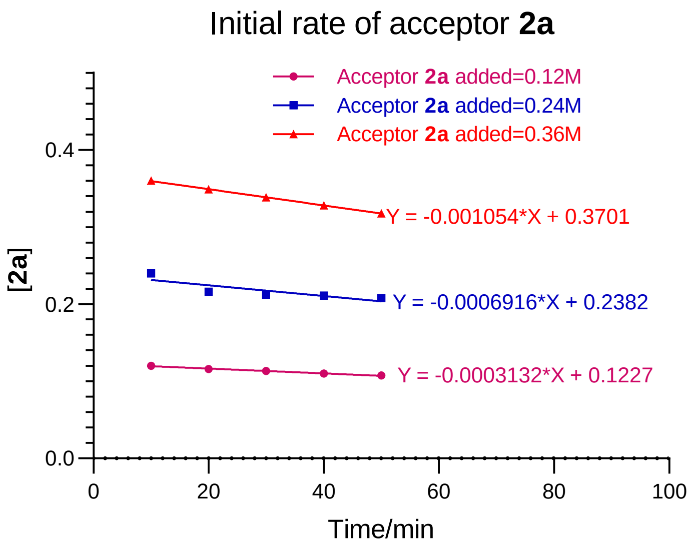

Supplementary Figure 29. Initial rate of acceptor **2a**.

Supplementary Table 27. Simple linear regression of Initial acceptor rate.

	[**Acceptor 2a**]$_{initial}$ (0.12M)	[**Acceptor 2a**]$_{initial}$ (0.24M)	[**Acceptor 2a**]$_{initial}$ (0.36M)
**Best-fit values**			
Slope	-0.0003132	-0.0006916	-0.001054
Y-intercept	0.1227	0.2382	0.3701
X-intercept	391.8	344.5	351.1
1/slope	-3192	-1446	-948.6
**Std. Error**			
Slope	1.469e-005	0.0002520	1.245e-005
Y-intercept	0.0004871	0.008357	0.0004131
**95% Confidence Intervals**			
Slope	-0.0003600 to -0.0002665	-0.001493 to 0.0001103	-0.001094 to -0.001015
Y-intercept	0.1212 to 0.1243	0.2116 to 0.2648	0.3688 to 0.3714
X-intercept	344.8 to 455.3	175.2 to +infinity	339.4 to 363.7
Goodness of Fit			
R squared	0.9934	0.7152	0.9996

Sy.x	0.0004644	0.007968	0.0003939
**Is slope significantly non-zero?**			
F	455.0	7.533	7164
DFn, DFd	1, 3	1, 3	1, 3
P value	0.0002	0.0711	<0.0001
Deviation from zero?	Significant	Not Significant	Significant
Equation	Y = -0.0003132*X + 0.1227	Y = -0.0006916*X + 0.2382	Y = -0.001054*X + 0.3701
**Data**			
Number of X values	5	5	5
Maximum number of Y replicates	1	1	1
Total number of values	5	5	5
Number of missing values	0	0	0

Supplementary Table 28. Determination of order with respect to acceptor **2a**.

Entry	**Concentration**	**[2a]**^{1}	**Reaction rate**
1	0.12M	0.12	-0.0003132 mmol/min
2	0.24M	0.24	-0.0006916 mmol/min
3	0.36M	0.36	-0.001054 mmol/min

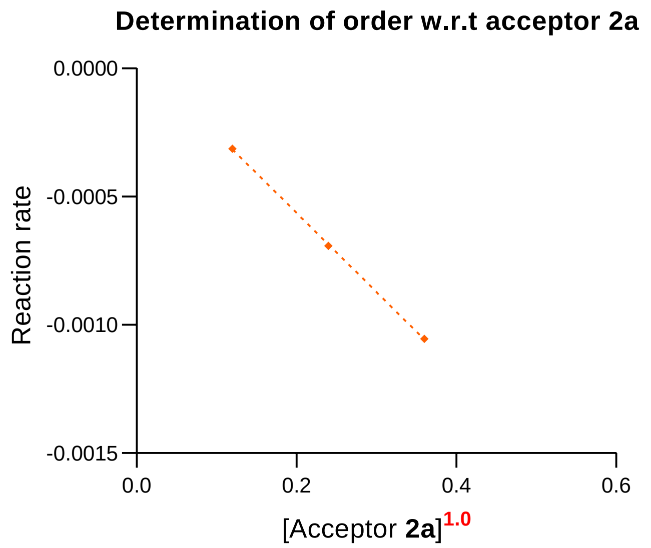

Supplementary Figure 30. Determination of order w.r.t to acceptor **2a** using the initial rate method.

Repetition of the kinetic study to ensure reproducibility

NMR monitoring under standard conditions

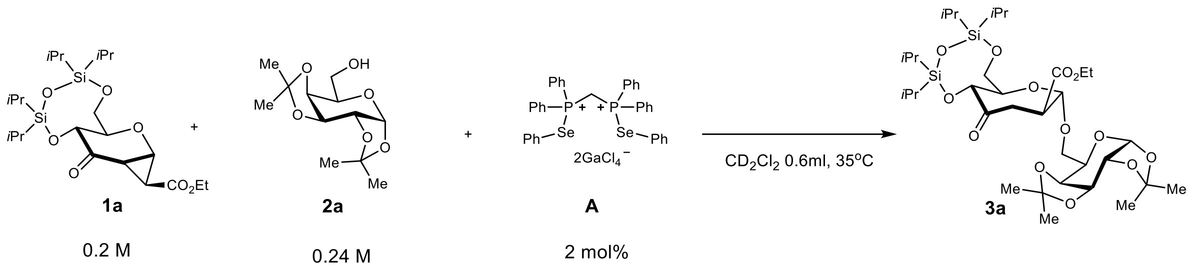

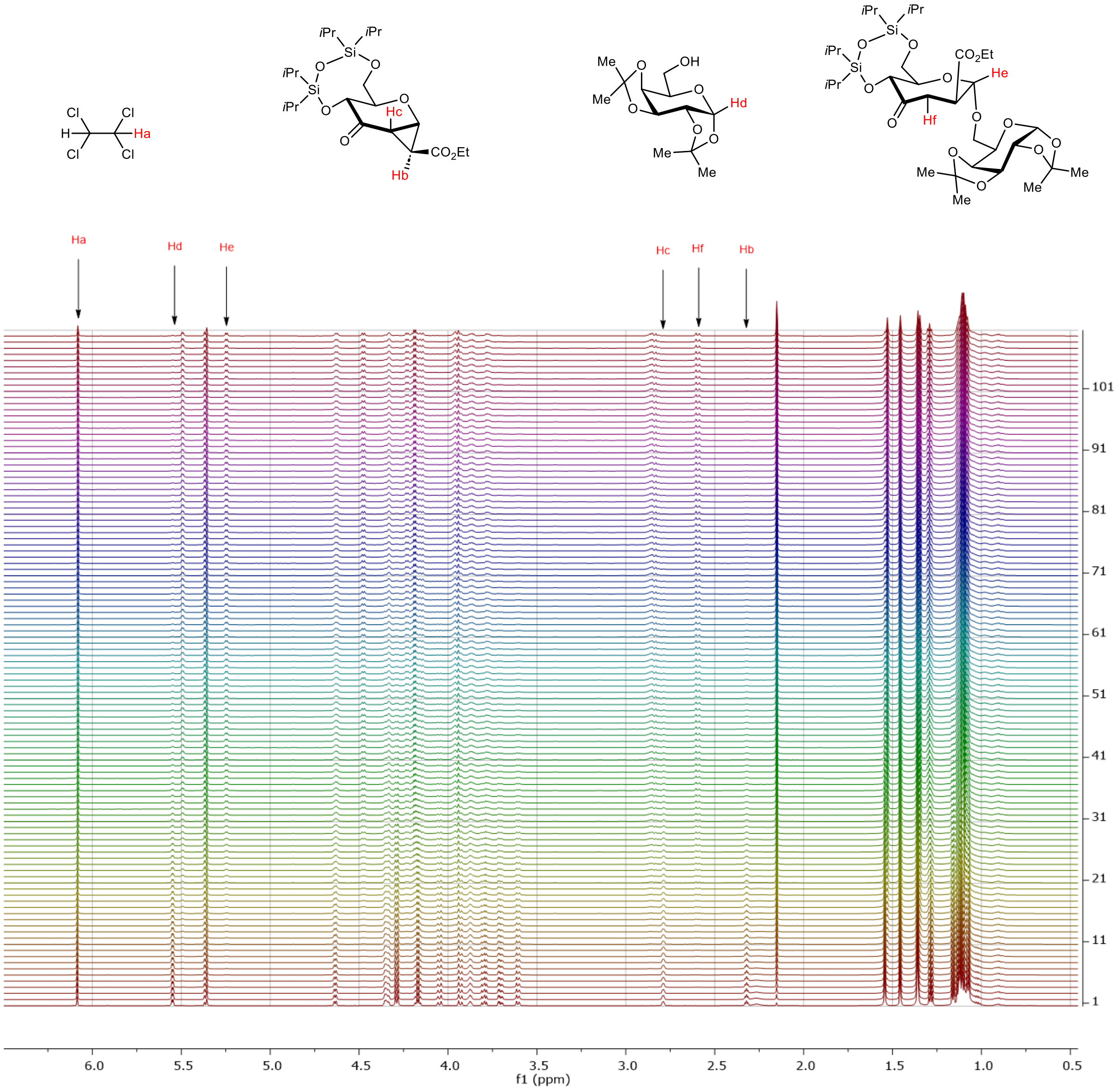

Supplementary Figure 31: Stacked ^{1}H NMR spectra for the monitoring under standard conditions

Supplementary table 29: Concentration for **1a**, **2a**, **3a** calculated by ^{1}H NMR analysis for the monitoring under standard conditions

time [h]:	[**Donor-1a**]/M	[**Acceptor-2a**]/M	[**3a**]/M

0	0.200000000	0.269014567	0.002877461
0.166667	0.199790125	0.268598554	0.006856125
0.333333	0.197871346	0.265697173	0.008173072
0.5	0.1936049024	0.262853199	0.010921073
0.666667	0.192406146	0.259984416	0.015147411
0.833333	0.191328136	0.256369479	0.017863953
1	0.187762122	0.254367971	0.019250704
1.166667	0.186541247	0.251119785	0.021795787
1.333333	0.184393187	0.247968343	0.024576874
1.5	0.181340382	0.246146547	0.026177615
1.666667	0.179740744	0.243736098	0.028764664
1.833333	0.177114797	0.240689518	0.030922779
2	0.173491816	0.238168545	0.033009183
2.166667	0.170571028	0.235941057	0.035214174
2.333333	0.168620925	0.233382185	0.037772687
2.5	0.165486125	0.231393669	0.039588412
2.666667	0.164067565	0.228588652	0.041895022
2.833333	0.160839721	0.226883458	0.044029231
3	0.159223214	0.224045686	0.046495556
3.166667	0.155893873	0.220245374	0.049294512
3.333333	0.152388796	0.220146963	0.050361402
3.5	0.149554239	0.217472361	0.052443007
3.666667	0.146681024	0.212852709	0.056156011
3.833333	0.143274271	0.209647488	0.058461205
4	0.139681885	0.207549073	0.060861611
4.166667	0.136444792	0.204528509	0.063394777
4.333333	0.133413981	0.203363048	0.064804783
4.5	0.129588514	0.199473578	0.067974404
4.666667	0.126271329	0.198476254	0.069777058
4.833333	0.122448205	0.195502195	0.072397068
5	0.118622436	0.190753408	0.075578111
5.166667	0.114574874	0.188392473	0.078040772
5.333333	0.111217409	0.185178742	0.080878753
5.5	0.107892468	0.181049189	0.083970351
5.666667	0.104355127	0.178254031	0.086676823
5.833333	0.100076782	0.175127381	0.089473312
6	0.096677614	0.171632817	0.092579762
6.166667	0.092819072	0.167975223	0.096102498
6.333333	0.089212968	0.164435177	0.098993293
6.5	0.085560858	0.160595726	0.102042371
6.666667	0.083046725	0.156588349	0.105684267
6.833333	0.079145279	0.153079434	0.108824009
7	0.076313304	0.149477623	0.112175797
7.166667	0.073492616	0.146960252	0.114737474
7.333333	0.070966637	0.143409159	0.118283448
7.5	0.067897526	0.138402299	0.121888325
7.666667	0.065415854	0.134566284	0.125224047
7.833333	0.062239877	0.131271356	0.128355625
8	0.059973377	0.127509863	0.131123735
8.166667	0.056856396	0.124131493	0.134177342
8.333333	0.054702765	0.121418951	0.136975526
8.5	0.051931638	0.116652827	0.140152922
8.666667	0.050376121	0.114223446	0.142771477
8.833333	0.047492321	0.111288343	0.145199205
9	0.045358152	0.108889662	0.147733379
9.166667	0.043600548	0.105234647	0.150365093
9.333333	0.040942761	0.102016643	0.152667809
9.5	0.039095808	0.098272021	0.154770457

9.666667	0.037110137	0.096168074	0.156976621
9.833333	0.035032704	0.092850702	0.159200366
10	0.033995243	0.089984626	0.161339605
10.16667	0.031419935	0.086527734	0.163317822
10.33333	0.029481986	0.085280651	0.165145979
10.5	0.027870653	0.082451699	0.166802745
10.66667	0.026364479	0.080233363	0.168619212
10.83333	0.025480206	0.077913801	0.170429976
11	0.023783464	0.075184774	0.171629528
11.16667	0.023019852	0.072860203	0.173243119
11.33333	0.021141684	0.069544871	0.174960049
11.5	0.020042634	0.068123696	0.176352073
11.66667	0.019206858	0.066853188	0.177286789
11.83333	0.018168874	0.063400179	0.178871578
12	0.017377912	0.061535956	0.179828414
12.16667	0.016507734	0.059416314	0.181041474
12.33333	0.015490779	0.057689183	0.181908984
12.5	0.014927144	0.056754643	0.182381045
12.66667	0.013814716	0.054284753	0.183272981
12.83333	0.012817278	0.052958631	0.184313354
13	0.012125735	0.051926199	0.184894951
13.16667	0.011655482	0.050589706	0.185354853
13.33333	0.010792031	0.049043326	0.186040962
13.5	0.010290277	0.047857249	0.186415605
13.66667	0.009332909	0.047076714	0.186816696
13.83333	0.008907346	0.045099031	0.187684882
14	0.008262371	0.045012677	0.187641493
14.16667	0.007860345	0.043088552	0.188288312
14.33333	0.007144812	0.042305315	0.188554956
14.5	0.006744111	0.040693852	0.188856595
14.66667	0.006262333	0.039486931	0.189153781
14.83333	0.005490864	0.039190969	0.189239476
15	0.004941463	0.038072647	0.189796386
15.16667	0.004330915	0.037357894	0.189927396
15.33333	0.004517753	0.036711418	0.190206567
15.5	0.003675961	0.035470733	0.190337591
15.66667	0.003488215	0.034999266	0.190668149
15.83333	0.003557243	0.033934891	0.190833922
16	0.003479367	0.033155142	0.191057723
16.16667	0.003421835	0.032465743	0.191174611
16.33333	0.003916423	0.032105738	0.191200071
16.5	0.003853681	0.031510326	0.191394707
16.66667	0.003952671	0.030198272	0.191598254
16.83333	0.003708202	0.030329478	0.191847309
17	0.003967564	0.02961026	0.191866505
17.16667	0.003610385	0.029099956	0.191923852
17.33333	0.003310319	0.027695801	0.192141138
17.5	0.003208165	0.027120586	0.192323344
17.66667	0.003591862	0.027589826	0.192320787
17.83333	0.003333293	0.026090034	0.192498527
18	0.003222761	0.026018201	0.192397131
18.16667	0.002981703	0.026109903	0.192462222

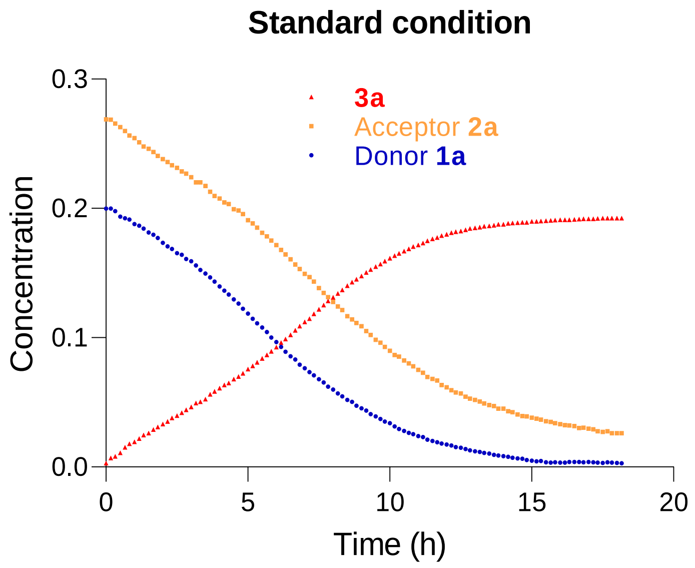

Supplementary Figure 32: Temporal kinetics profile for the monitoring under standard conditions

Concentration dependence studies

Donor 1a concentration dependence

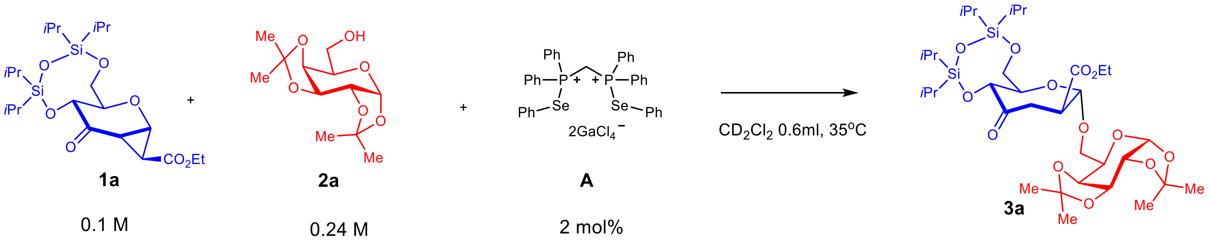

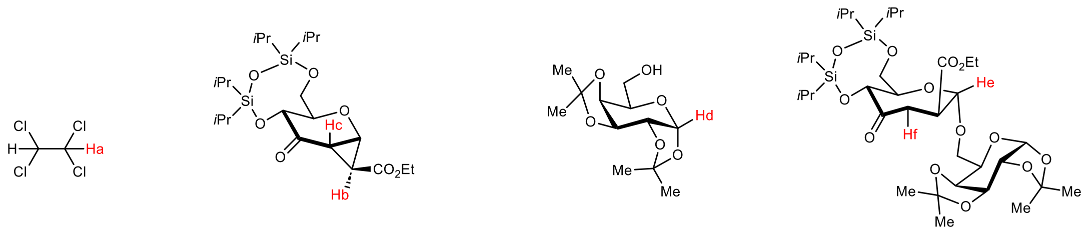

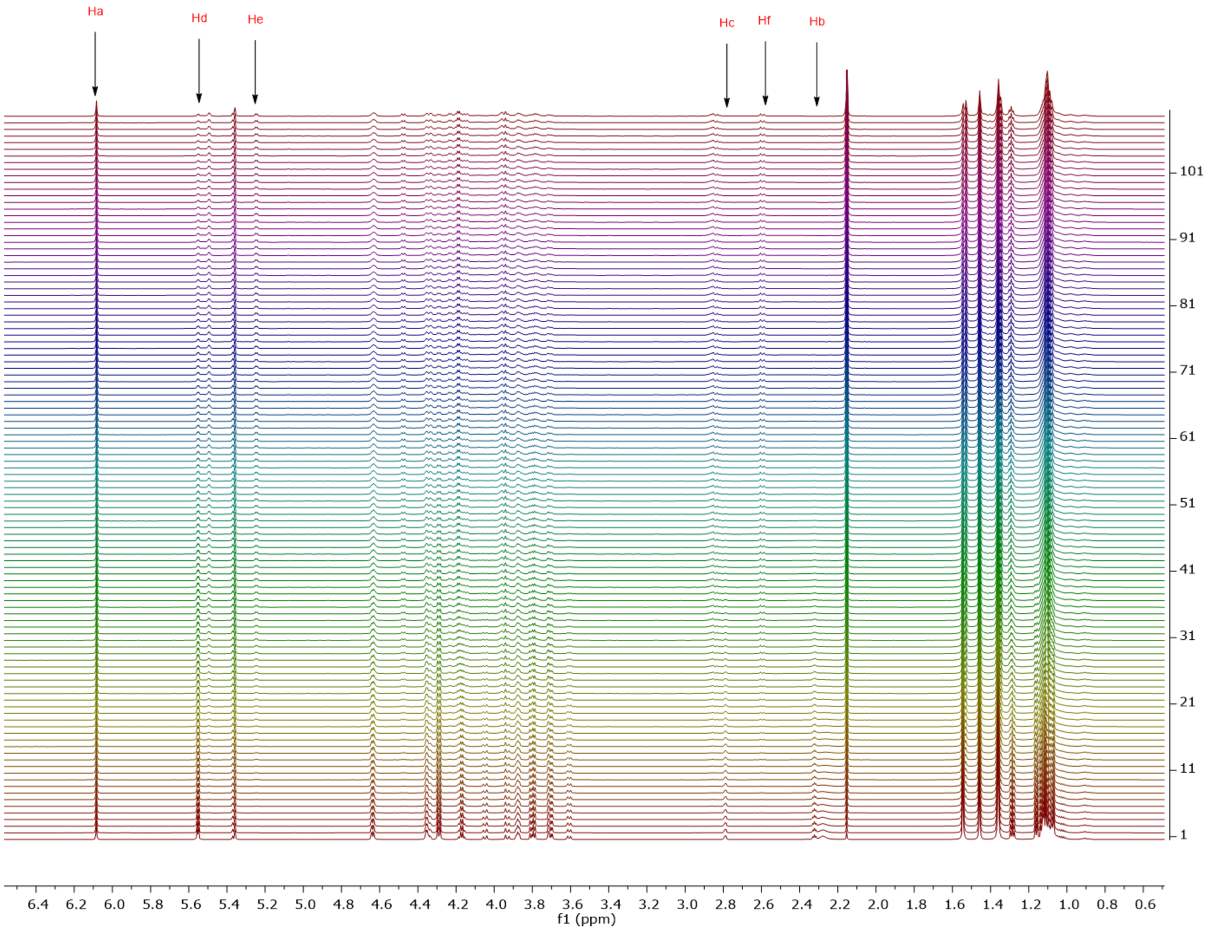

Supplementary Figure 33: Stacked ^{1}H spectra for the experiment of entry [**0.1M**]

Donor 1a concentration dependence

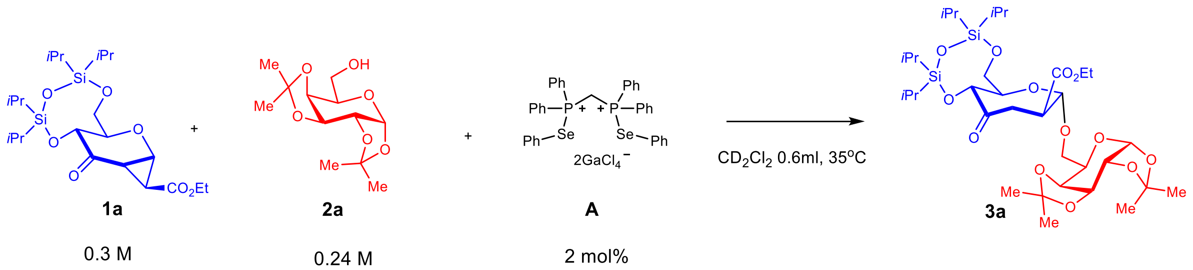

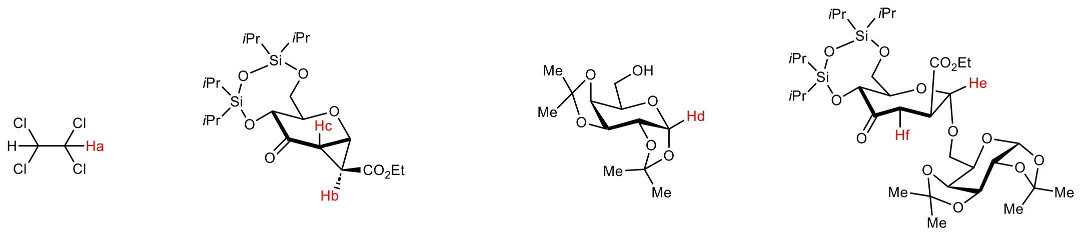

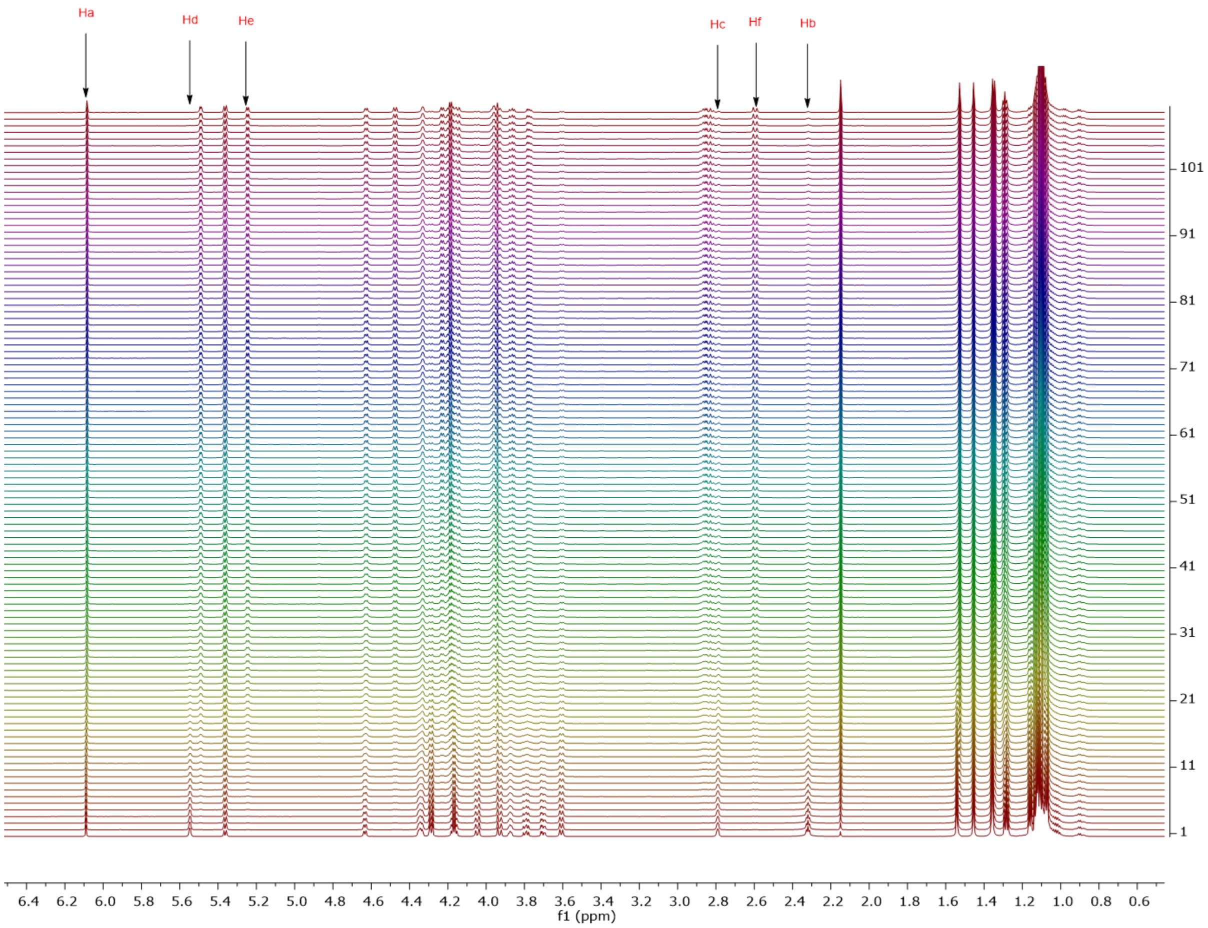

Supplementary Figure 34: Stacked ^{1}H spectra for the experiment of entry [**0.3M**]

Supplementary table 30: Concentration for **3a** calculated by ^{1}H NMR analysis for varying the donor concentration experiment

time [h]:	**3a** (**1a**=0.1M)	**3a** (**1a**=0.2M)	**3a** (**1a**=0.3M)
0	0.003548903	0.002877461	0.003516897
0.166667	0.004651858	0.006856125	0.008379708
0.333333	0.005472654	0.008173072	0.00998931
0.5	0.007091695	0.010921073	0.013347978
0.666667	0.009461287	0.015147411	0.018513503
0.833333	0.010997268	0.017863953	0.021833721
1	0.012977724	0.019250704	0.023528638
1.166667	0.014640089	0.021795787	0.026639296
1.333333	0.015711213	0.024576874	0.030038402
1.5	0.016090542	0.026177615	0.031994862
1.666667	0.017442377	0.028764664	0.035156812
1.833333	0.018985895	0.030922779	0.037794507
2	0.020040596	0.033009183	0.040344557
2.166667	0.021974203	0.035214174	0.043039546
2.333333	0.022597532	0.037772687	0.046166618
2.5	0.023740242	0.039588412	0.048385837
2.666667	0.024884273	0.041895022	0.051205027
2.833333	0.025994383	0.044029231	0.053813503
3	0.027000281	0.046495556	0.056827901
3.166667	0.028359204	0.049294512	0.060248848
3.333333	0.029800173	0.050361402	0.061552824
3.5	0.030905622	0.052443007	0.064097008
3.666667	0.031878345	0.056156011	0.068635124
3.833333	0.033201903	0.058461205	0.071452584
4	0.034152514	0.060861611	0.074386413
4.166667	0.035552059	0.063394777	0.077482505
4.333333	0.036146578	0.064804783	0.079205846
4.5	0.037689541	0.067974404	0.083079827
4.666667	0.038544985	0.069777058	0.085283071
4.833333	0.039682754	0.072397068	0.088485305
5	0.041176074	0.075578111	0.092373247
5.166667	0.042063971	0.078040772	0.095383166
5.333333	0.043944548	0.080878753	0.098851809
5.5	0.044600705	0.083970351	0.102630427
5.666667	0.046279983	0.086676823	0.105938339
5.833333	0.046862499	0.089473312	0.109356267
6	0.048602069	0.092579762	0.113153042
6.166667	0.049745622	0.096102498	0.117458609
6.333333	0.050808574	0.098993293	0.120991803
6.5	0.052399679	0.102042371	0.124718453
6.666667	0.053564333	0.105684267	0.129169659
6.833333	0.055524903	0.108824009	0.133007122
7	0.056656682	0.112175797	0.137103752
7.166667	0.058046332	0.114737474	0.140234691
7.333333	0.059130285	0.118283448	0.144568659
7.5	0.060527069	0.121888325	0.148974619
7.666667	0.061820554	0.125224047	0.153051613
7.833333	0.063038777	0.128355625	0.156879098
8	0.064136494	0.131123735	0.160262342
8.166667	0.065435472	0.134177342	0.163994527
8.333333	0.066899404	0.136975526	0.167414532
8.5	0.068292908	0.140152922	0.171298016
8.666667	0.069728086	0.142771477	0.174498472

8.833333	0.070831863	0.145199205	0.177465695
9	0.071982751	0.147733379	0.180563018
9.166667	0.073117243	0.150365093	0.183779558
9.333333	0.074392286	0.152667809	0.186593989
9.5	0.075660481	0.154770457	0.189163892
9.666667	0.077304285	0.156976621	0.191860313
9.833333	0.078089945	0.159200366	0.194578225
10	0.079237046	0.161339605	0.197192851
10.16667	0.080418905	0.163317822	0.199610672
10.33333	0.081651424	0.165145979	0.201845085
10.5	0.082450878	0.166802745	0.203870022
10.66667	0.083313447	0.168619212	0.206090145
10.83333	0.084365332	0.170429976	0.208303304
11	0.085212731	0.171629528	0.209769423
11.16667	0.086260654	0.173243119	0.211741591
11.33333	0.087208041	0.174960049	0.213840062
11.5	0.087987198	0.176352073	0.215541423
11.66667	0.088728632	0.177286789	0.216683853
11.83333	0.089540076	0.178871578	0.218620818
12	0.090222473	0.179828414	0.219790284
12.16667	0.090585491	0.181041474	0.221272912
12.33333	0.091328455	0.181908984	0.222333203
12.5	0.091950129	0.182381045	0.222910167
12.66667	0.092773897	0.183272981	0.224000309
12.83333	0.092761579	0.184313354	0.225271877
13	0.093292326	0.184894951	0.225982718
13.16667	0.09369133	0.185354853	0.226544821
13.33333	0.093868459	0.186040962	0.227383397
13.5	0.094534935	0.186415605	0.227841295
13.66667	0.094818471	0.186816696	0.228331517
13.83333	0.095122547	0.187684882	0.229392632
14	0.095251784	0.187641493	0.229339602
14.16667	0.095690783	0.188288312	0.230130159
14.33333	0.095625710	0.188554956	0.230456057
14.5	0.095772465	0.188856595	0.230824727
14.66667	0.095974793	0.189153781	0.231187955
14.83333	0.096027166	0.189239476	0.231292693
15	0.096018354	0.189796386	0.231973361
15.16667	0.096080837	0.189927396	0.232133483
15.33333	0.096388489	0.190206567	0.232474693
15.5	0.096382405	0.190337591	0.232634833
15.66667	0.096489881	0.190668149	0.233038849
15.83333	0.096435673	0.190833922	0.233241463
16	0.096508132	0.191057723	0.233514995
16.16667	0.096400394	0.191174611	0.233657857
16.33333	0.095817925	0.191200071	0.233688975
16.5	0.095768693	0.191394707	0.233926864
16.66667	0.095627745	0.191598254	0.234175643
16.83333	0.095411983	0.191847309	0.234480044
17	0.095374918	0.191866505	0.234503507
17.16667	0.095382951	0.191923852	0.234573597
17.33333	0.095341393	0.192141138	0.234839168
17.5	0.095036563	0.192323344	0.235061864
17.66667	0.095189203	0.192320787	0.235058739
17.83333	0.094798689	0.192498527	0.235275978
18	0.093834945	0.192397131	0.235151889
18.16667	0.095079638	0.192462222	0.235231605

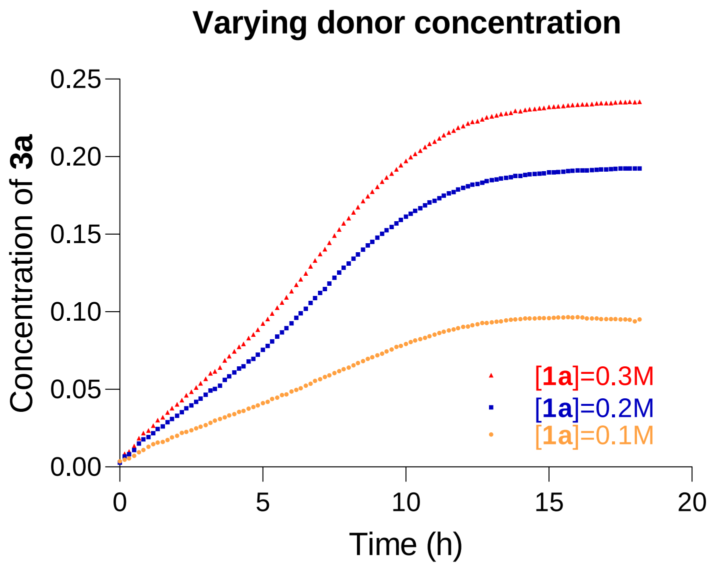

Supplementary Figure 35. Overlapped profile for the donor **1a** concentration dependence experiments

Acceptor 2a concentration dependence

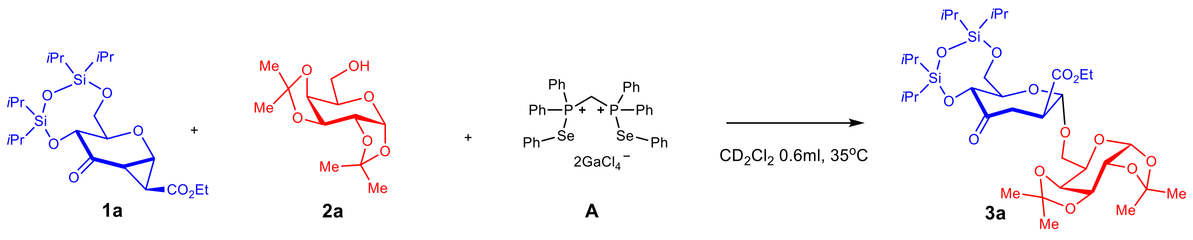

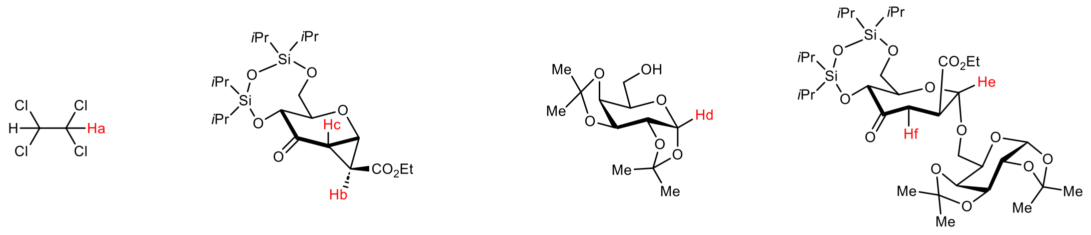

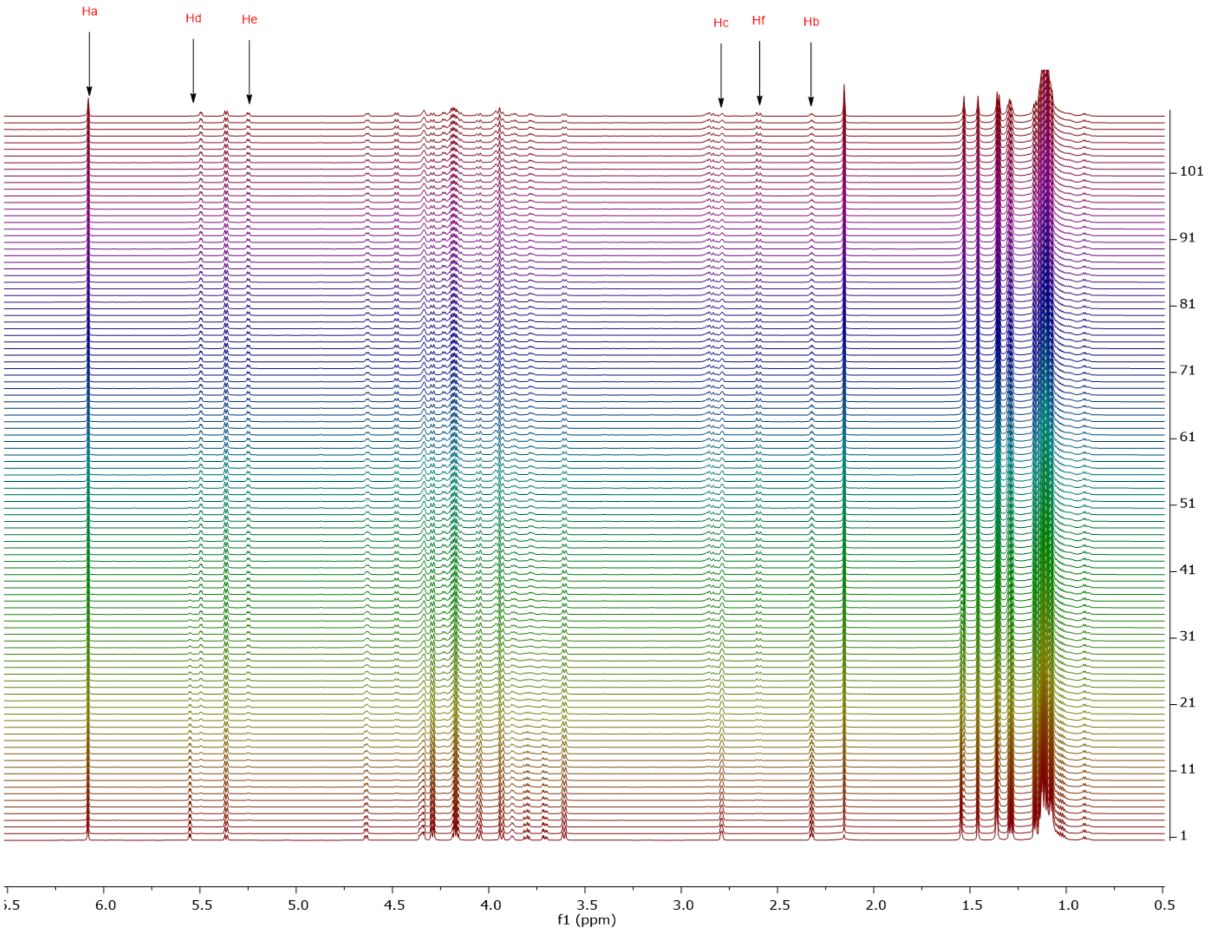

Supplementary Figure 36: Stacked ^{1}H spectra for the experiment of entry [**0.12M**]

Acceptor 2a concentration dependence

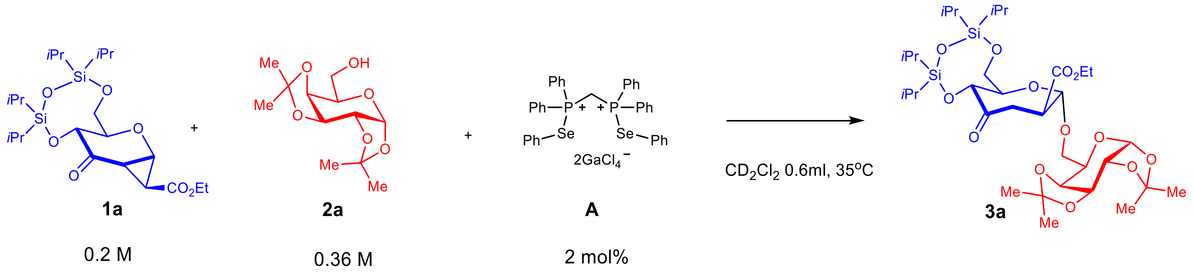

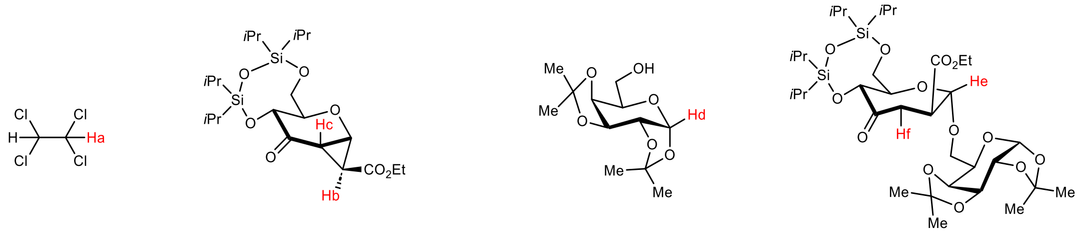

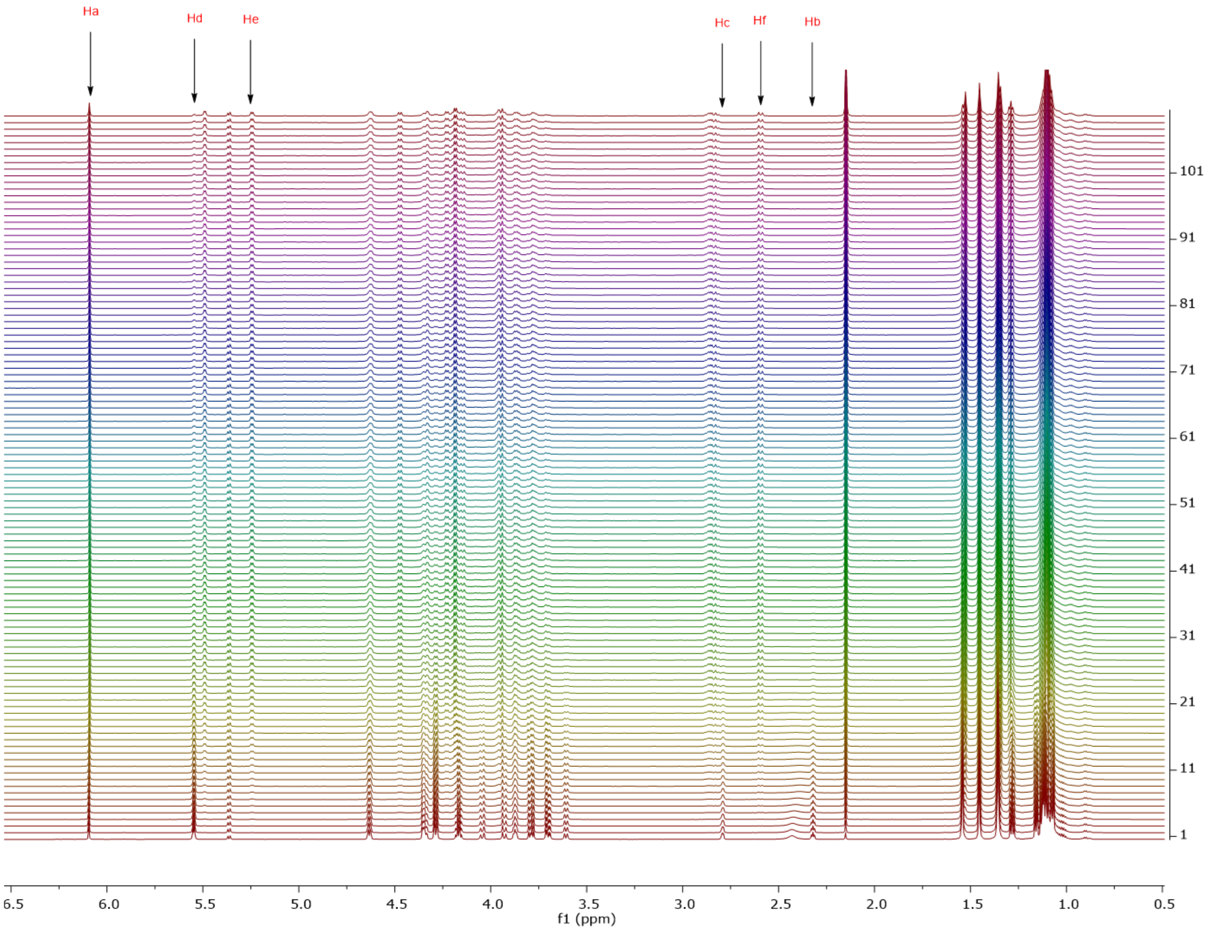

Supplementary Figure 37: Stacked ^{1}H spectra for the experiment of entry [**0.36M**]

Supplementary table 31: Concentration for **3a** calculated by ^{1}H NMR analysis for varying the acceptor concentration experiment

**time [h]:**	**3a** (**2a** =0.12M)	**3a** (**2a** =0.24M)	**3a** (**2a** =0.36M)
0	0.003007851	0.002877461	0.00782546
0.166667	0.004492538	0.006856125	0.010166341
0.333333	0.006338044	0.008173072	0.014813402
0.5	0.008637574	0.010921073	0.018192889
0.666667	0.011815352	0.015147411	0.023890072
0.833333	0.013641543	0.017863953	0.031383506
1	0.014596332	0.019250704	0.038342044
1.166667	0.016061597	0.021795787	0.045892592
1.333333	0.018542859	0.024576874	0.053389992
1.5	0.020726023	0.026177615	0.062271563
1.666667	0.021594245	0.028764664	0.070469331
1.833333	0.023111266	0.030922779	0.079151624
2	0.025415452	0.033009183	0.089362076
2.166667	0.027780643	0.035214174	0.097241953
2.333333	0.028663313	0.037772687	0.107415427
2.5	0.031079375	0.039588412	0.115676411
2.666667	0.032772973	0.041895022	0.124123074
2.833333	0.034162699	0.044029231	0.134171711
3	0.036298554	0.046495556	0.141886334
3.166667	0.037951482	0.049294512	0.149832263
3.333333	0.039921973	0.050361402	0.157595222
3.5	0.043325681	0.052443007	0.163508926
3.666667	0.046099164	0.056156011	0.169814461
3.833333	0.047860652	0.058461205	0.176366425
4	0.049797542	0.060861611	0.182119323
4.166667	0.052002487	0.063394777	0.186800174
4.333333	0.054170481	0.064804783	0.190944163
4.5	0.057276398	0.067974404	0.19402779
4.666667	0.059380109	0.069777058	0.197643234
4.833333	0.062020297	0.072397068	0.199368376
5	0.064591085	0.075578111	0.202469214
5.166667	0.067928118	0.078040772	0.204837347
5.333333	0.069682926	0.080878753	0.205585239
5.5	0.072909868	0.083970351	0.208716202
5.666667	0.075211384	0.086676823	0.208580899
5.833333	0.077796114	0.089473312	0.210242544
6	0.080178691	0.092579762	0.210707397
6.166667	0.082779625	0.096102498	0.212655125
6.333333	0.085041675	0.098993293	0.212026596
6.5	0.087376658	0.102042371	0.21306004
6.666667	0.089517817	0.105684267	0.212801542
6.833333	0.092252466	0.108824009	0.212528116
7	0.094002283	0.112175797	0.214546539
7.166667	0.095330822	0.114737474	0.21385739
7.333333	0.098170453	0.118283448	0.214793607
7.5	0.098512514	0.121888325	0.213986011
7.666667	0.100877765	0.125224047	0.214628069
7.833333	0.104031784	0.128355625	0.214670691
8	0.104118547	0.131123735	0.215303238
8.166667	0.105969115	0.134177342	0.213487683
8.333333	0.107088315	0.136975526	0.216014418
8.5	0.107113534	0.140152922	0.214707175
8.666667	0.109767355	0.142771477	0.214615589
8.833333	0.110476949	0.145199205	0.215338365

9	0.110835667	0.147733379	0.214321022
9.166667	0.110357343	0.150365093	0.213073166
9.333333	0.110788173	0.152667809	0.214491501
9.5	0.111372021	0.154770457	0.214567783
9.666667	0.112444328	0.156976621	0.214167615
9.833333	0.112281303	0.159200366	0.213824919
10	0.113328126	0.161339605	0.212872378
10.16667	0.113577796	0.163317822	0.212754149
10.33333	0.113381818	0.165145979	0.213448286
10.5	0.114274153	0.166802745	0.211840983
10.66667	0.114248693	0.168619212	0.212177865
10.83333	0.114831792	0.170429976	0.213345133
11	0.114418022	0.171629528	0.211543965
11.16667	0.114401632	0.173243119	0.214561907
11.33333	0.114886789	0.174960049	0.213651824
11.5	0.115512822	0.176352073	0.212348172
11.66667	0.115153244	0.177286789	0.210034256
11.83333	0.116571386	0.178871578	0.210657202
12	0.114722026	0.179828414	0.211259079
12.16667	0.115166319	0.181041474	0.212046504
12.33333	0.114690891	0.181908984	0.211145721
12.5	0.116732578	0.182381045	0.210777507
12.66667	0.115334529	0.183272981	0.210913681
12.83333	0.115038687	0.184313354	0.209691996
13	0.115259334	0.184894951	0.207082918
13.16667	0.116132031	0.185354853	0.209462693
13.33333	0.116759852	0.186040962	0.209394589
13.5	0.116226682	0.186415605	0.208728809
13.66667	0.115142613	0.186816696	0.209904498
13.83333	0.114733033	0.187684882	0.209866051
14	0.116173023	0.187641493	0.209628603
14.16667	0.116161168	0.188288312	0.207901508
14.33333	0.115631079	0.188554956	0.207076188
14.5	0.114314869	0.188856595	0.207210319
14.66667	0.114493458	0.189153781	0.209649321
14.83333	0.115758724	0.189239476	0.208002399
15	0.114387788	0.189796386	0.207816183
15.16667	0.114440776	0.189927396	0.206827341
15.33333	0.114854828	0.190206567	0.206303171
15.5	0.113882512	0.190337591	0.206772463
15.66667	0.114224739	0.190668149	0.206852806
15.83333	0.113920579	0.190833922	0.207412348
16	0.114115893	0.191057723	0.206289584
16.16667	0.114754515	0.191174611	0.204539892
16.33333	0.114431002	0.191200071	0.205895786
16.5	0.114207032	0.191394707	0.204485698
16.66667	0.113926426	0.191598254	0.204902852
16.83333	0.113997652	0.191847309	0.205684038
17	0.112793292	0.191866505	0.20547295
17.16667	0.113082232	0.191923852	0.204994995
17.33333	0.111948482	0.192141138	0.204658486
17.5	0.113261173	0.192323344	0.204770311
17.66667	0.112022449	0.192320787	0.204937852
17.83333	0.110383558	0.192498527	0.204175984
18	0.111858653	0.192397131	0.204898959
18.16667	0.111358093	0.192462222	0.203434265

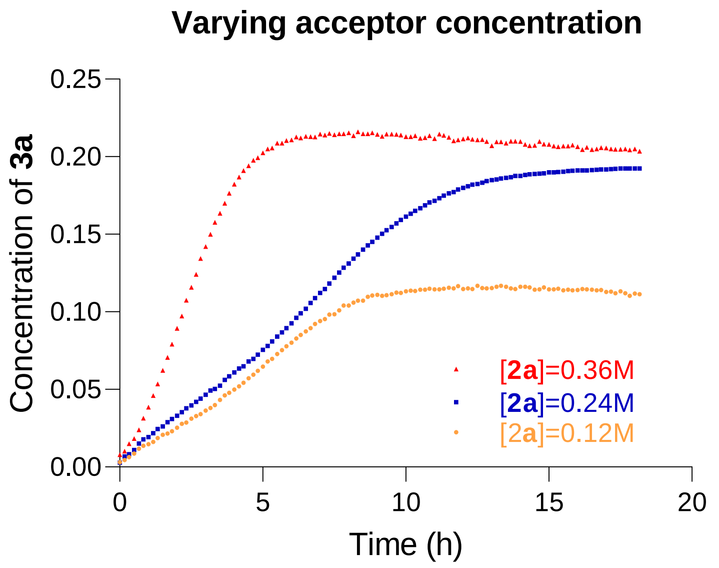

Supplementary Figure 38. Overlapped profile for the acceptor **2a** concentration dependence experiments

Catalyst A concentration dependence

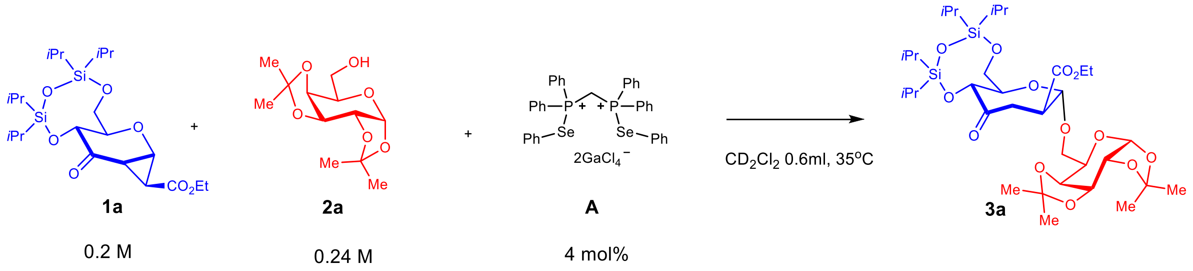

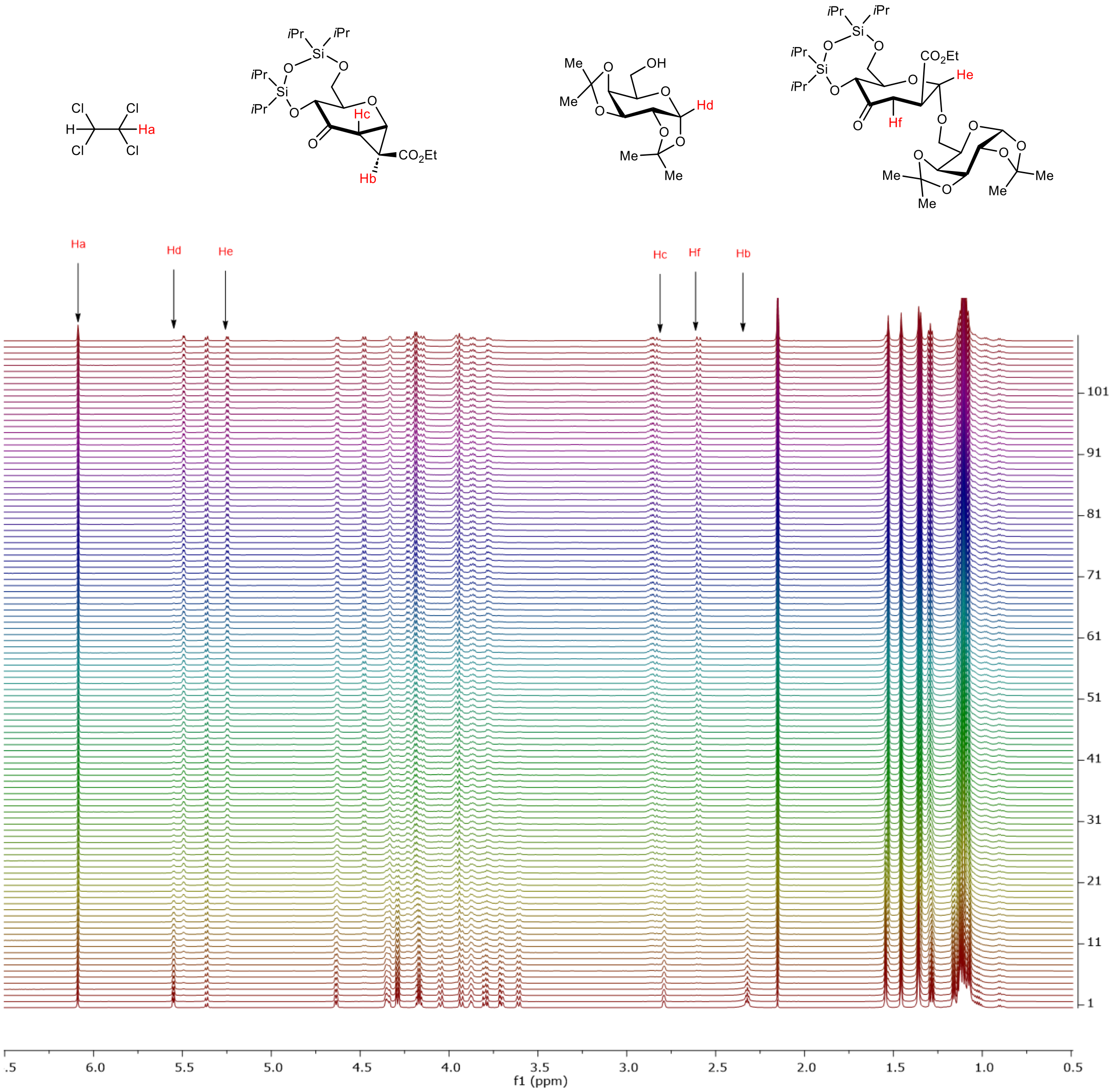

Supplementary Figure 39: Stacked ^{1}H spectra for the experiment of entry [**4 mol%**]

Catalyst concentration dependence

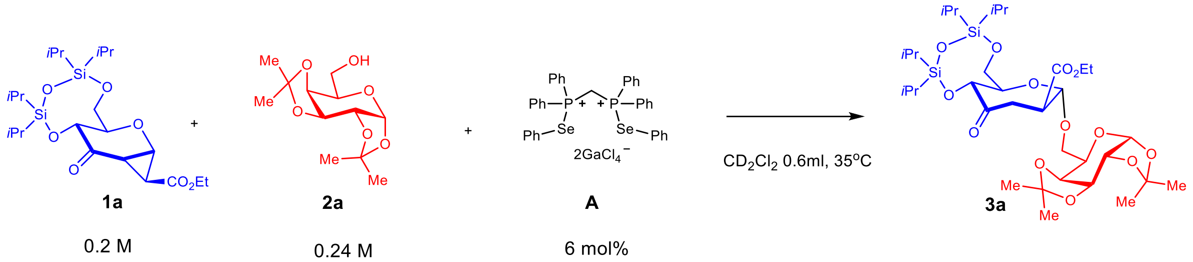

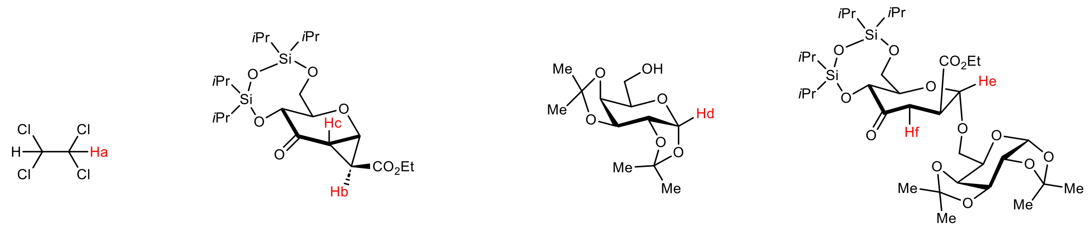

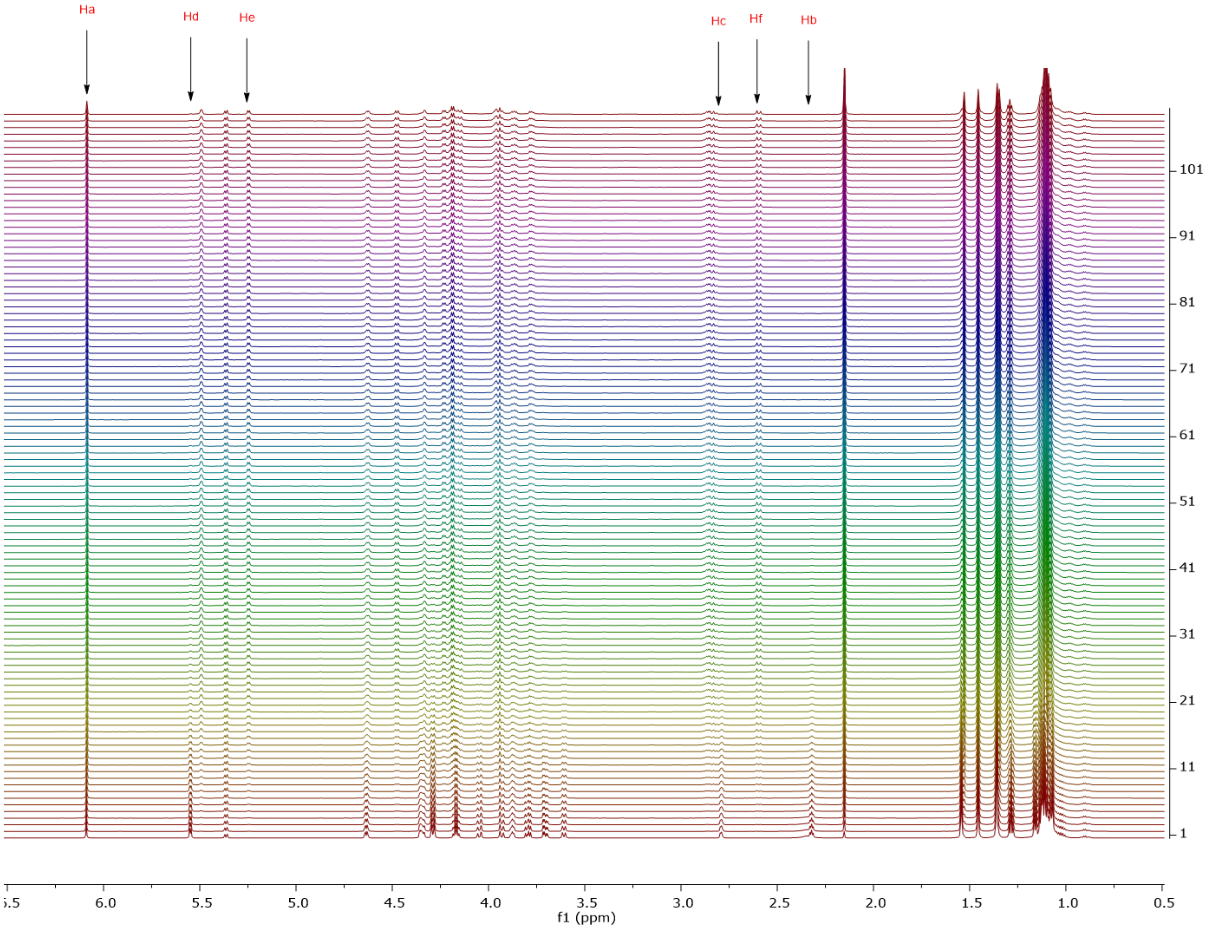

Supplementary Figure 40: Stacked ^{1}H spectra for the experiment of entry [**6 mol%**]

Supplementary table 32: Concentration for **3a** calculated by ^{1}H NMR analysis for varying the catalyst concentration experiment.

**time [h]:**	**3a** (**A** =2.0 mol%)	**3a** (**A** =4.0 mol%)	**3a** (**A** =6.0 mol%)
0	0.002877461	0.004256138	0.007524821
0.166667	0.006856125	0.006478352	0.009171493
0.333333	0.008173072	0.010025586	0.015808047
0.5	0.010921073	0.014107994	0.028066163
0.666667	0.015147411	0.020251045	0.035924981
0.833333	0.017863953	0.024189337	0.044395788
1	0.019250704	0.030446603	0.051586121
1.166667	0.021795787	0.034764052	0.064400439
1.333333	0.024576874	0.042610717	0.075668021
1.5	0.026177615	0.046962803	0.087800217
1.666667	0.028764664	0.055808444	0.100120893
1.833333	0.030922779	0.067485308	0.111618645
2	0.033009183	0.071700093	0.122819058
2.166667	0.035214174	0.080445812	0.129959277
2.333333	0.037772687	0.089245923	0.139735988
2.5	0.039588412	0.103187563	0.147969614
2.666667	0.041895022	0.108271607	0.154749514
2.833333	0.044029231	0.117224461	0.160491366
3	0.046495556	0.126206735	0.164481043
3.166667	0.049294512	0.133248171	0.168515177
3.333333	0.050361402	0.141474182	0.172061064
3.5	0.052443007	0.147390811	0.174013345
3.666667	0.056156011	0.152900892	0.176922467
3.833333	0.058461205	0.157299871	0.177519783
4	0.060861611	0.161103301	0.179390208
4.166667	0.063394777	0.164206215	0.182248002
4.333333	0.064804783	0.167259093	0.182991844
4.5	0.067974404	0.169487311	0.184313029
4.666667	0.069777058	0.171166514	0.185180133
4.833333	0.072397068	0.172859538	0.184459595
5	0.075578111	0.174855302	0.185951332
5.166667	0.078040772	0.175676191	0.183087148
5.333333	0.080878753	0.176498977	0.184292523
5.5	0.083970351	0.176906322	0.182753161
5.666667	0.086676823	0.177610207	0.183618742
5.833333	0.089473312	0.177949937	0.182335482
6	0.092579762	0.178488805	0.182614138
6.166667	0.096102498	0.178411102	0.181404321
6.333333	0.098993293	0.177868993	0.181260577
6.5	0.102042371	0.177542839	0.180960424
6.666667	0.105684267	0.177316176	0.179667271
6.833333	0.108824009	0.177313681	0.179582424
7	0.112175797	0.177379309	0.178525176
7.166667	0.114737474	0.177225663	0.178272086
7.333333	0.118283448	0.176997158	0.177588768
7.5	0.121888325	0.176681249	0.177728507
7.666667	0.125224047	0.176136268	0.176892114
7.833333	0.128355625	0.175715342	0.176142419
8	0.131123735	0.174951967	0.176600815
8.166667	0.134177342	0.174280607	0.176178569
8.333333	0.136975526	0.174292046	0.175097483
8.5	0.140152922	0.173717477	0.175441611
8.666667	0.142771477	0.173097483	0.174698808
8.833333	0.145199205	0.173173476	0.174052281

9	0.147733379	0.173013573	0.173398414
9.166667	0.150365093	0.170860797	0.173284998
9.333333	0.152667809	0.172129126	0.172412312
9.5	0.154770457	0.171557147	0.172248228
9.666667	0.156976621	0.170474507	0.171467558
9.833333	0.159200366	0.170828646	0.171940173
10	0.161339605	0.169720142	0.170352163
10.16667	0.163317822	0.169453732	0.169633897
10.33333	0.165145979	0.169798525	0.168281685
10.5	0.166802745	0.167825606	0.168109534
10.66667	0.168619212	0.167627536	0.167800574
10.83333	0.170429976	0.165459203	0.166800574
11	0.171629528	0.166302952	0.165637372
11.16667	0.173243119	0.166834901	0.164909645
11.33333	0.174960049	0.163787235	0.163501521
11.5	0.176352073	0.162951703	0.164544881
11.66667	0.177286789	0.162217591	0.162766557
11.83333	0.178871578	0.161905065	0.161892689
12	0.179828414	0.161675132	0.159584465
12.16667	0.181041474	0.160677483	0.161541624
12.33333	0.181908984	0.162779693	0.160743578
12.5	0.182381045	0.162429855	0.160998339
12.66667	0.183272981	0.162688003	0.159357528
12.83333	0.184313354	0.161501311	0.158739432
13	0.184894951	0.159617207	0.158753567
13.16667	0.185354853	0.160818913	0.158121171
13.33333	0.186040962	0.160396414	0.157485297
13.5	0.186415605	0.160258666	0.157161241
13.66667	0.186816696	0.159412111	0.157981572
13.83333	0.187684882	0.158172948	0.155720858
14	0.187641493	0.158597839	0.155677989
14.16667	0.188288312	0.157812746	0.155944642
14.33333	0.188554956	0.156560281	0.154675198
14.5	0.188856595	0.155842091	0.156544611
14.66667	0.189153781	0.154142357	0.155122756
14.83333	0.189239476	0.153607439	0.153419649
15	0.189796386	0.153146757	0.153204263
15.16667	0.189927396	0.153583826	0.152921061
15.33333	0.190206567	0.152459655	0.153907002
15.5	0.190337591	0.151643697	0.151839927
15.66667	0.190668149	0.153125962	0.151975573
15.83333	0.190833922	0.152377363	0.151159992
16	0.191057723	0.152491087	0.149676412
16.16667	0.191174611	0.152832462	0.150308237
16.33333	0.191200071	0.152653291	0.150386495
16.5	0.191394707	0.151695718	0.150817764
16.66667	0.191598254	0.150555177	0.149952524
16.83333	0.191847309	0.150151675	0.150137852
17	0.191866505	0.150898953	0.148533612
17.16667	0.191923852	0.150119187	0.148642993
17.33333	0.192141138	0.149983279	0.147513343
17.5	0.192323344	0.150429568	0.145702741
17.66667	0.192320787	0.150645146	0.145974641
17.83333	0.192498527	0.149384663	0.145545449
18	0.192397131	0.149093401	0.145552999
18.16667	0.192462222	0.149125604	0.145289445

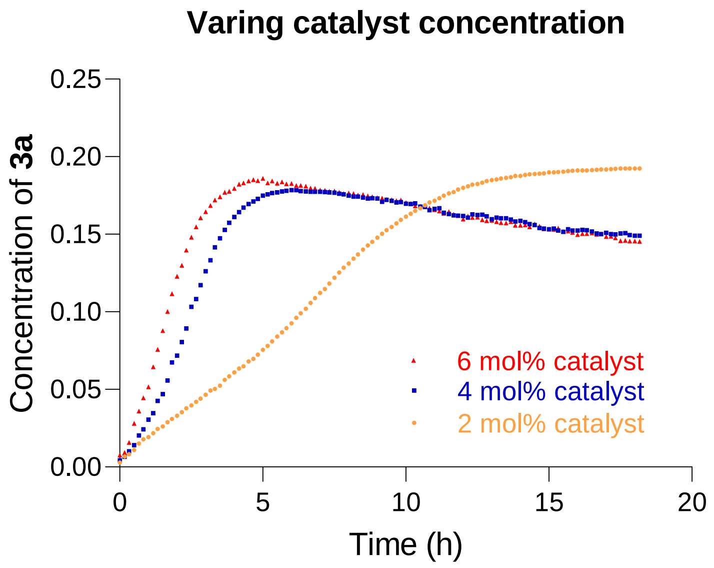

Supplementary Figure 41. Overlapped profile for the catalyst **A** concentration dependence experiments

Kinetic Analysis (Repetition)

Burés method to determine order with respect to catalyst **A**[19,20].

Supplementary Table 33. Concentration for **3a** & Product formation over time multiplied by [catalyst] to the 1.2 power.

	2.0 mol% catalyst **A**		4.0 mol% catalyst **A**		6.0 mol% catalyst **A**	
Time/min	t[cat]$^{1.2}$	[**3a**]	t[**cat**]$^{1.2}$	[**3a**]	t[**cat**]$^{1.2}$	[**3a**]
0	0	0.002877461	0	0.004256138	0	0.007524821
10	0.013257816	0.006856125	0.030458463	0.006478352	0.049547015	0.009171493
20	0.026515632	0.008173072	0.060916926	0.010025586	0.09909403	0.015808047
30	0.039773448	0.010921073	0.091375389	0.014107994	0.148641045	0.028066163
40	0.053031264	0.015147411	0.121833852	0.020251045	0.19818806	0.035924981
50	0.06628908	0.017863953	0.152292315	0.024189337	0.247735075	0.044395788
60	0.079546896	0.019250704	0.182750778	0.030446603	0.29728209	0.051586121
70	0.092804712	0.021795787	0.213209241	0.03476405	0.346829105	0.064400439
80	0.106062529	0.024576874	0.243667704	0.042610717	0.39637612	0.075668021
90	0.119320345	0.026177615	0.274126167	0.046962803	0.445923135	0.087800217
100	0.132578161	0.028764664	0.30458463	0.055808444	0.49547015	0.100120893
110	0.145835977	0.030922779	0.335043093	0.067485308	0.545017165	0.111618645
120	0.159093793	0.033009183	0.365501556	0.071700093	0.59456418	0.122819058
130	0.172351609	0.035214174	0.395960019	0.080445812	0.644111195	0.129959277
140	0.185609425	0.037772687	0.426418482	0.089245923	0.69365821	0.139735988
150	0.198867241	0.039588412	0.456876945	0.103187563	0.743205225	0.147969614
160	0.212125057	0.041895022	0.487335408	0.108271607	0.79275224	0.154749514
170	0.225382873	0.04402923	0.517793871	0.117224461	0.842299255	0.160491366
180	0.238640689	0.046495556	0.548252334	0.126206735	0.89184627	0.164481043
190	0.251898505	0.049294512	0.578710797	0.133248171	0.941393285	0.168515177
200	0.265156321	0.050361402	0.60916926	0.141474182	0.9909403	0.172061064
210	0.278414137	0.052443007	0.639627723	0.14739081	1.040487315	0.174013345
220	0.291671954	0.056156011	0.670086186	0.152900892	1.09003433	0.176922467
230	0.30492977	0.058461205	0.700544649	0.157299871	1.139581345	0.177519783
240	0.318187586	0.060861611	0.731003112	0.161103301	1.18912836	0.179390208
250	0.331445402	0.063394777	0.761461575	0.164206215	1.238675375	0.182248002
260	0.344703218	0.064804783	0.791920039	0.167259093	1.28822239	0.182991844
270	0.357961034	0.067974404	0.822378502	0.169487311	1.337769405	0.184313029
280	0.37121885	0.069777058	0.852836965	0.171166514	1.38731642	0.185180133
290	0.384476666	0.072397068	0.883295428	0.172859538	1.436863435	0.184459595
300	0.397734482	0.075578111	0.913753891	0.174855302	1.48641045	0.185951332
310	0.410992298	0.078040772	0.944212354	0.175676191	1.535957465	0.183087148
320	0.424250114	0.080878753	0.974670817	0.176498977		
330	0.43750793	0.08397035	1.00512928	0.176906322		
340	0.450765746	0.086676823	1.035587743	0.177610207		
350	0.464023562	0.08947331	1.066046206	0.177949937		
360	0.477281378	0.092579762	1.096504669	0.178488805		
370	0.490539195	0.096102498	1.126963132	0.178411102		

380	0.503797011	0.098993293	1.157421595	0.177868993
390	0.517054827	0.10204237	1.187880058	0.177542839
400	0.530312643	0.105684267	1.218338521	0.177316176
410	0.543570459	0.108824009	1.248796984	0.17731368
420	0.556828275	0.112175797	1.279255447	0.177379309
430	0.570086091	0.114737474	1.30971391	0.17722566
440	0.583343907	0.118283448	1.340172373	0.176997158
450	0.596601723	0.121888325	1.370630836	0.176681249
460	0.609859539	0.125224047	1.401089299	0.176136268
470	0.623117355	0.128355625	1.431547762	0.17571534
480	0.636375171	0.131123735	1.462006225	0.174951967
490	0.649632987	0.13417734	1.492464688	0.174280607
500	0.662890803	0.136975526	1.522923151	0.174292046
510	0.67614862	0.140152922	1.553381614	0.173717477
520	0.689406436	0.142771477		
530	0.702664252	0.145199205		
540	0.715922068	0.147733379		
550	0.729179884	0.150365093		
560	0.7424377	0.152667809		
570	0.755695516	0.154770457		
580	0.768953332	0.15697662		
590	0.782211148	0.159200366		
600	0.795468964	0.161339605		
610	0.80872678	0.163317822		
620	0.821984596	0.165145979		
630	0.835242412	0.166802745		
640	0.848500228	0.16861921		
650	0.861758045	0.170429976		
660	0.875015861	0.171629528		
670	0.888273677	0.173243119		
680	0.901531493	0.174960049		
690	0.914789309	0.176352073		
700	0.928047125	0.177286789		
710	0.941304941	0.178871578		
720	0.954562757	0.179828414		
730	0.967820573	0.181041474		
740	0.981078389	0.181908984		
750	0.994336205	0.182381045		
760	1.007594021	0.183272982		
770	1.020851837	0.184313354		
780	1.034109653	0.184894951		
790	1.047367469	0.185354853		
800	1.060625286	0.186040962		
810	1.073883102	0.186415605		

820	1.087140918	0.186816696
830	1.100398734	0.187684881
840	1.11365655	0.187641493
850	1.126914366	0.188288312
860	1.140172182	0.188554956
870	1.153429998	0.188856595
880	1.166687814	0.189153781
890	1.17994563	0.189239476
900	1.193203446	0.189796386
910	1.206461262	0.189927396
920	1.219719078	0.190206567
930	1.232976894	0.190337591
940	1.246234711	0.190668149
950	1.259492527	0.190833922
960	1.272750343	0.191057723
970	1.286008159	0.191174612
980	1.299265975	0.191200071
990	1.312523791	0.191394707
1000	1.325781607	0.191598254
1010	1.339039423	0.191847309
1020	1.352297239	0.191866505
1030	1.365555055	0.191923852
1040	1.378812871	0.192141138
1050	1.392070687	0.192323344
1060	1.405328503	0.192320787
1070	1.418586319	0.192498527
1080	1.431844135	0.192397012
1090	1.445101952	0.192462222

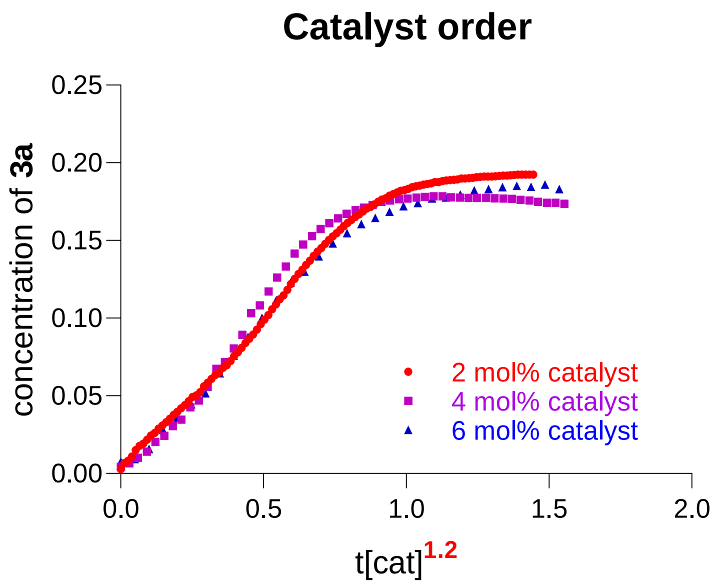

Supplementary Figure 42. Product formation over time multiplied by [**catalyst A**] to the 1.2 power. Graphical overlay represents a 1.2 dependence.

Supplementary Table 34. Initial rate of donor **1a**

Time/min	[**Donor 1a**]$_{initial}$ (0.1M)	[**Donor 1a**]$_{initial}$ (0.2M)	[**Donor 1a**]$_{initial}$ (0.3M)
10	0.1	0.2	0.3
20	0.097881	0.199790	0.297143
30	0.097126	0.197871	0.294621
40	0.095912	0.193605	0.291947
50	0.097261	0.192406	0.288328
60	0.096252	0.191328	0.286395
70	0.095048	0.187762	0.283295
80	0.094201	0.186541	0.280288

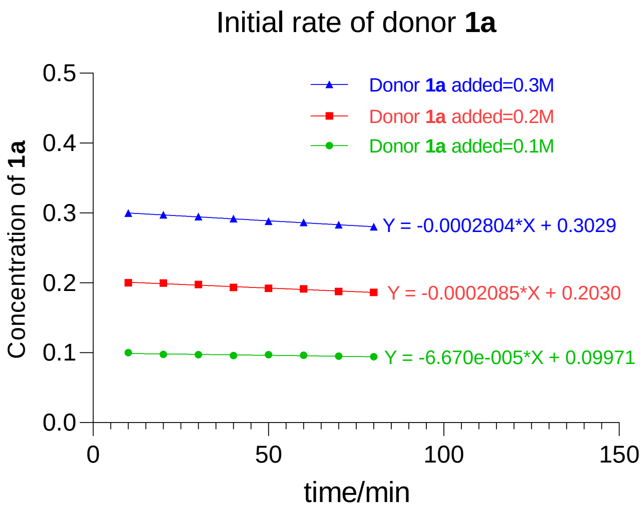

Supplementary Figure 43. Initial rate of donor **1a**.

Supplementary Table 35. Simple linear regression of Initial donor rate.

	[**Donor 1a**]$_{initial}$ (0.1M)	[**Donor 1a**]$_{initial}$ (0.2M)	[**Donor 1a**]$_{initial}$ (0.3M)
Best-fit values			
Slope	-6.670e-005	-0.0002085	-0.0002804
Y-intercept	0.09971	0.2030	0.3029
X-intercept	1495	973.6	1080
1/slope	-14992	-4795	-3567
Std. Error			
Slope	1.242e-005	1.452e-005	4.674e-006
Y-intercept	0.0006273	0.0007335	0.0002360
95% Confidence Intervals			
Slope	-9.710e-005 to -3.631e-005	-0.0002441 to -0.0001730	-0.0002918 to -0.0002689
Y-intercept	0.09818 to 0.1012	0.2013 to 0.2048	0.3023 to 0.3034
X-intercept	1041 to 2709	838.4 to 1164	1040 to 1124
Goodness of Fit			

R squared	0.8278	0.9717	0.9983
Sy.x	0.0008050	0.0009413	0.0003029
Is slope significantly non-zero?			
F	28.83	206.2	3599
DFn, DFd	1, 6	1, 6	1, 6
P value	0.0017	<0.0001	<0.0001
Deviation from zero?	Significant	Significant	Significant
Equation	Y = -6.670e-005*X + 0.09971	Y = -0.0002085*X + 0.2030	Y = -0.0002804*X + 0.3029
Data			
Number of X values	9	9	9
Maximum number of Y replicates	1	1	1
Total number of values	8	8	8
Number of missing values	1	1	1

Supplementary Table 36. Determination of order with respect to donor **1a**.

Entry	Concentration	$[\mathbf{1a}]^{0.6}$	Reaction rate
1	0.1M	0.251188643	-0.0000667mmol/min
2	0.2M	0.380730788	-0.0002085 mmol/min
3	0.3M	0.485593375	-0.0002804 mmol/min

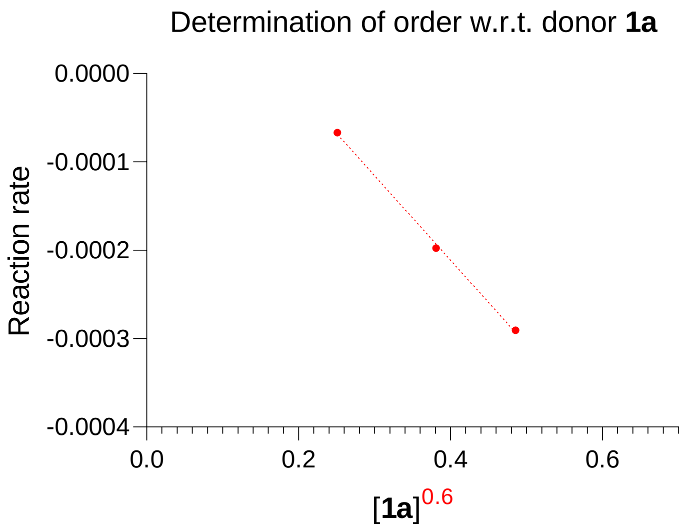

Supplementary Figure 44. Determination of order w.r.t **1a** using initial rate method

Supplementary Table 37. Initial rate of acceptor **2a**.

Time/min	[**Acceptor 2a**]$_{initial}$ (0.12M)	[**Acceptor 2a**]$_{initial}$ (0.24M)	[**Acceptor 2a**]$_{initial}$ (0.36M)
10	0.12	0.24	0.36
20	0.116021	0.216298	0.348709
30	0.113503	0.210916	0.338196
40	0.110216	0.210936	0.327904
50	0.107321	0.207993	0.317735

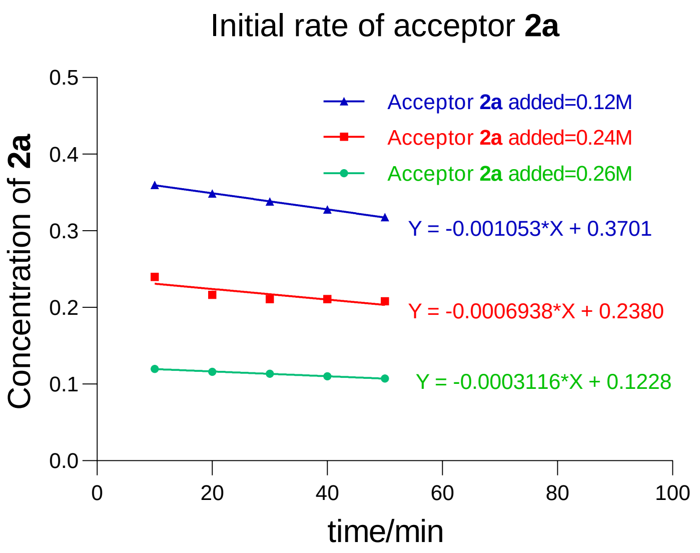

Supplementary Figure 45. Initial rate of acceptor **2a**.

Supplementary Table 38. Simple linear regression of Initial acceptor rate.

	[**Acceptor 2a**]$_{initial}$ (0.12M)	[**Acceptor 2a**]$_{initial}$ (0.12M)	[**Acceptor 2a**]$_{initial}$ (0.12M)
Best-fit values			
Slope	-0.0003116	-0.0006938	-0.001053
Y-intercept	0.1228	0.2380	0.3701
X-intercept	393.9	343.1	351.4
1/slope	-3209	-1441	-949.4
Std. Error			
Slope	1.181e-005	0.0002600	1.265e-005
Y-intercept	0.0003917	0.008624	0.0004195
95% Confidence Intervals			
Slope	-0.0003492 to -0.0002740	-0.001521 to 0.0001338	-0.001094 to -0.001013
Y-intercept	0.1215 to 0.1240	0.2106 to 0.2655	0.3688 to 0.3714
X-intercept	354.7 to 443.9	172.4 to +infinity	339.5 to 364.1
Goodness of Fit			
R squared	0.9957	0.7035	0.9996

Sy.x	0.0003734	0.008223	0.0004000
Is slope significantly non-zero?			
F	696.4	7.119	6936
DFn, DFd	1, 3	1, 3	1, 3
P value	0.0001	0.0758	<0.0001
Deviation from zero?	Significant	Not Significant	Significant
Equation	Y = -0.0003116*X + 0.1228	Y = -0.0006938*X + 0.2380	Y = -0.001053*X + 0.3701
Data			
Number of X values	5	5	5
Maximum number of Y replicates	1	1	1
Total number of values	5	5	5
Number of missing values	0	0	0

Supplementary Table 39. Determination of order with respect to acceptor **2a**.

Entry	**Concentration**	[**2a**]^1	**Reaction rate**
1	0.12M	0.12	-0.0003116 mmol/min
2	0.24M	0.24	-0.0006938 mmol/min
3	0.36M	0.36	-0.001053 mmol/min

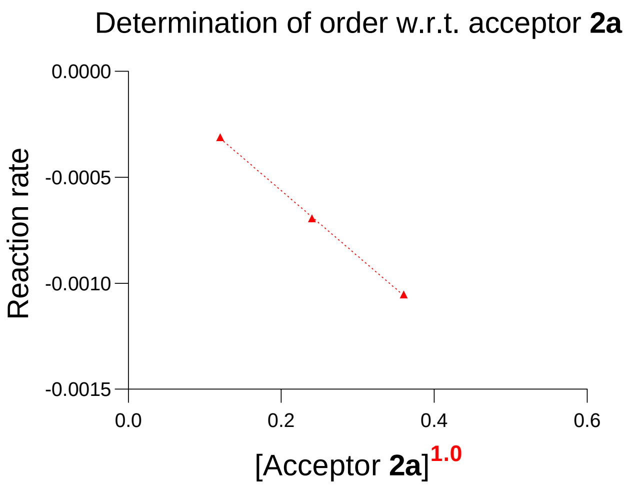

Supplementary Figure 46. Determination of order w.r.t to acceptor **2a** using the initial rate method.

^{13}C KIE measurements

We conducted our ^{13}C KIE experiments according to reported procedures.[21] Our experimental KIEs were subsequently computed by the equation below. Using ^{1}H-NMR analysis of the concentrated crude

mixture with 1,3,5-trimethoxybenzene as the internal standard, the fractional conversion of **1a**, F is then calculated. The ratios of the anomeric C1 and C5 carbon integrals in the substrate and product, denoted as R_0 and R_p respectively were obtained using the quantitative ^{13}C-NMR technique at natural abundance. The C5 atom integral was used as the reference atom.

The ^{13}C-KIE and inversion recovery experiments were performed on a Bruker Avance NEO 600 MHz spectrometer equipped with a 5 mm TMO cryoprobe (Bruker BioSpin GmbH, Rheinstetten, Germany). The inversion recovery experiment with inverse gated proton decoupling was used to determine the T_1 relaxation times of carbons C1 and C5. A 90° pulse of 12 µs and a relaxation delay of 60 s were used. The data showed that the T_1 relaxation times for both carbons were shorter than 1 second.

The quantitative ^{13}C spectra were acquired with inverse gated proton decoupling using a 30° flip angle (90° pulse: 12 µs), a relaxation delay of 30 s, an acquisition time of 2.2 s, 64k datapoints and 512 scans. The transmitter frequency offset was placed in the middle to ensure equal excitation of both carbons and a sweep width of 100 ppm was chosen. Each experiment was repeated three times.

The glycosylation experiments were performed as follows:

To an oven dried dram vial purged with an argon balloon was charged catalyst **A** (4.48 mg, 0.004 mmol, 2 mol%), cyclopropanated sugar donor **1a** (0.2 mmol, 1 equiv.), glycosyl acceptor **2a** (0.4 mmol, 2 equiv.) and then dry DCE (1 mL) was added. The dram vial was sealed and the mixture was immersed in a 35 °C heating block stirred for 2.5 h. The reaction mixture was filtered over a short silica plug and flushed with 5-10 mL of DCM. The filtrate was then evaporated and the determination of the conversion is by ^{1}H-NMR analysis of this concentrated crude mixture with 1,3,5-trimethoxybenzene as the internal standard. The crude mixture is subsequently loaded onto silica gel and subjected to flash column chromatography for purification.

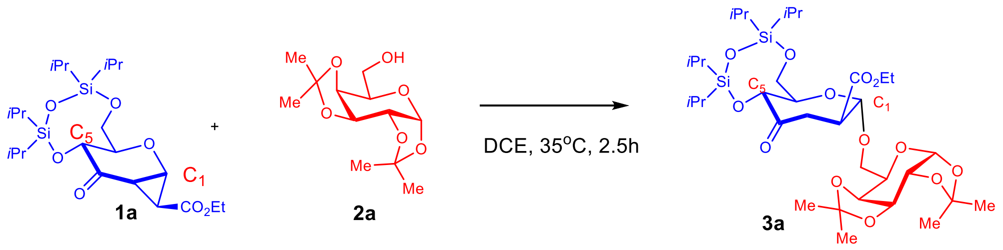

$$\mathrm{KIE} = \frac{\ln(1\text{-}F)}{\ln\left(1\text{-}F\,\frac{R_P}{R_0}\right)}$$

Run	F	R0	Rp	KIE
1	0.42230	1.022839	1.017839	1.0065
2	0.48393	1.022839	1.017551	1.0074
3	0.40664	1.022839	1.021990	1.0011

Average KIE = 1.005

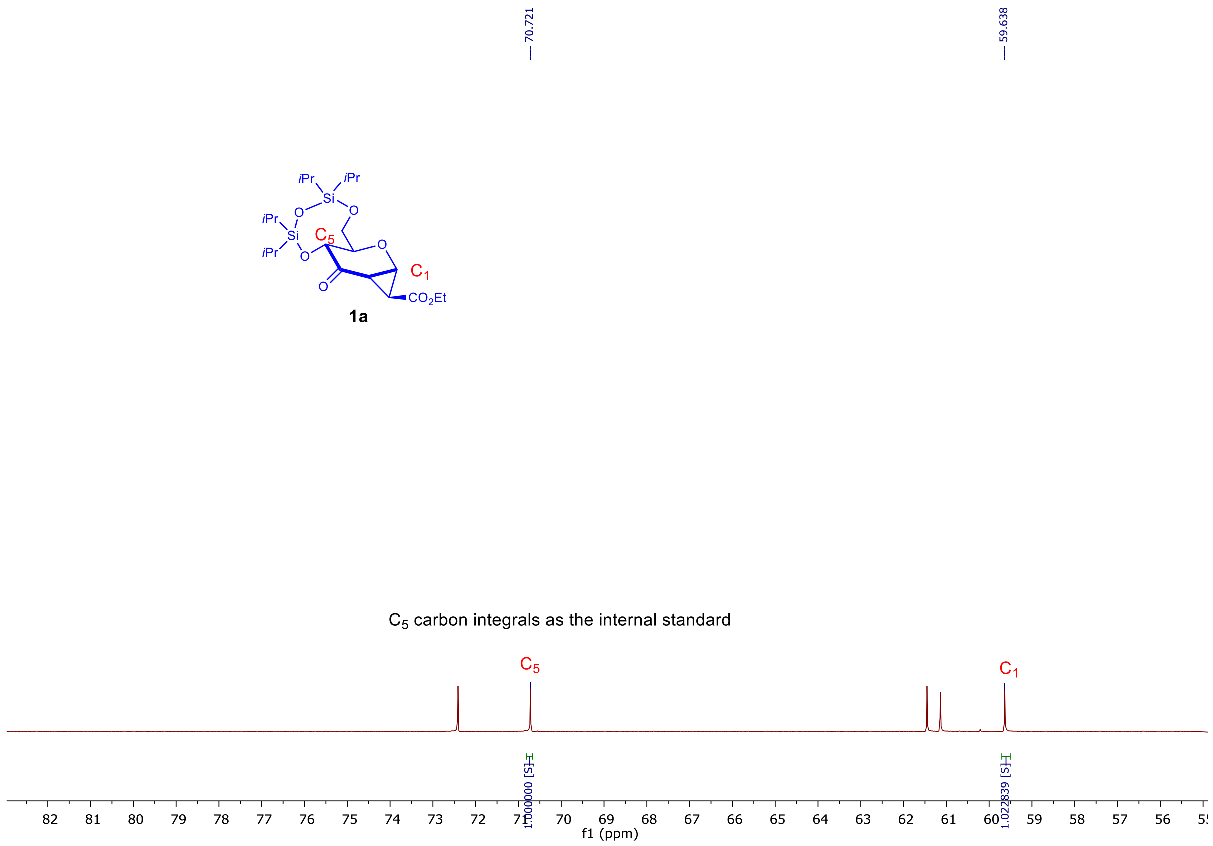

Supplementary Figure 47: Integration of 1st carbon in donor **1a**

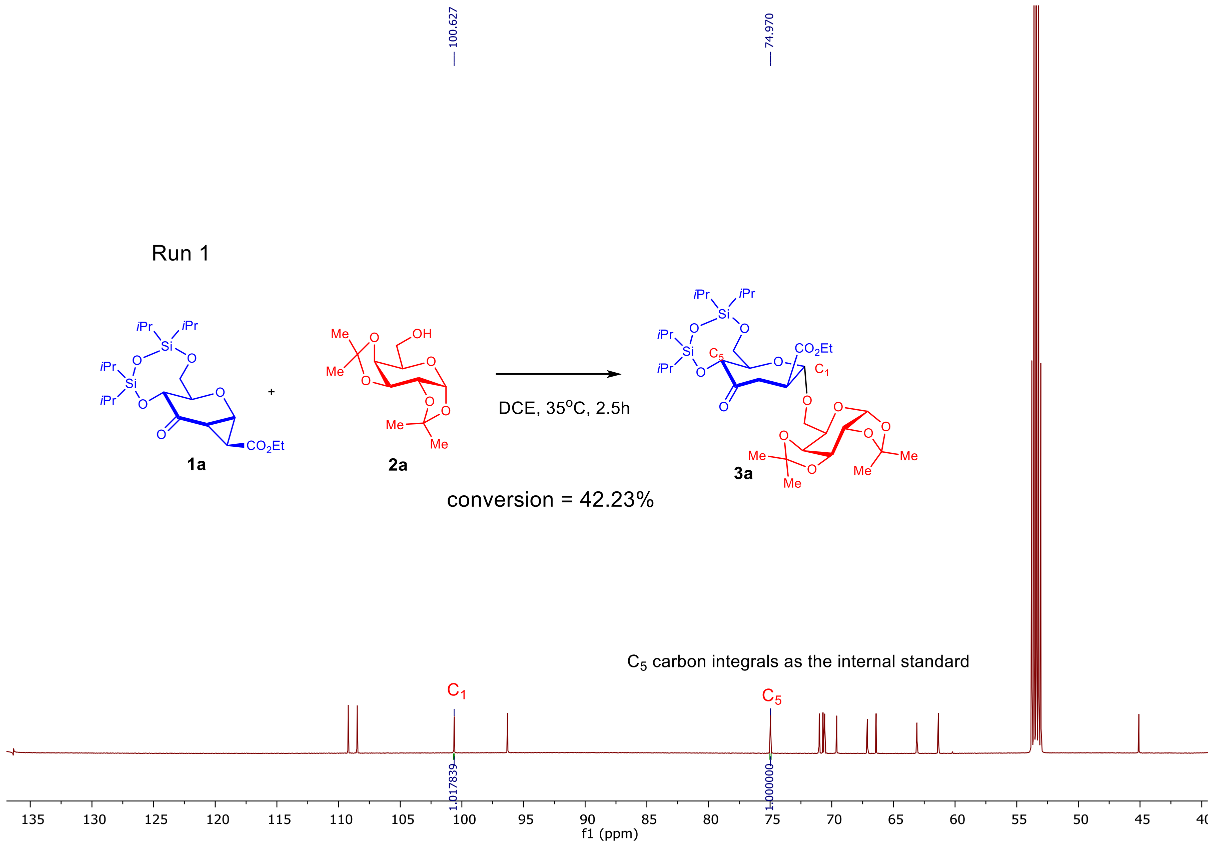

Supplementary Figure 48: Integration of 1st carbon in **3a** (run 1)

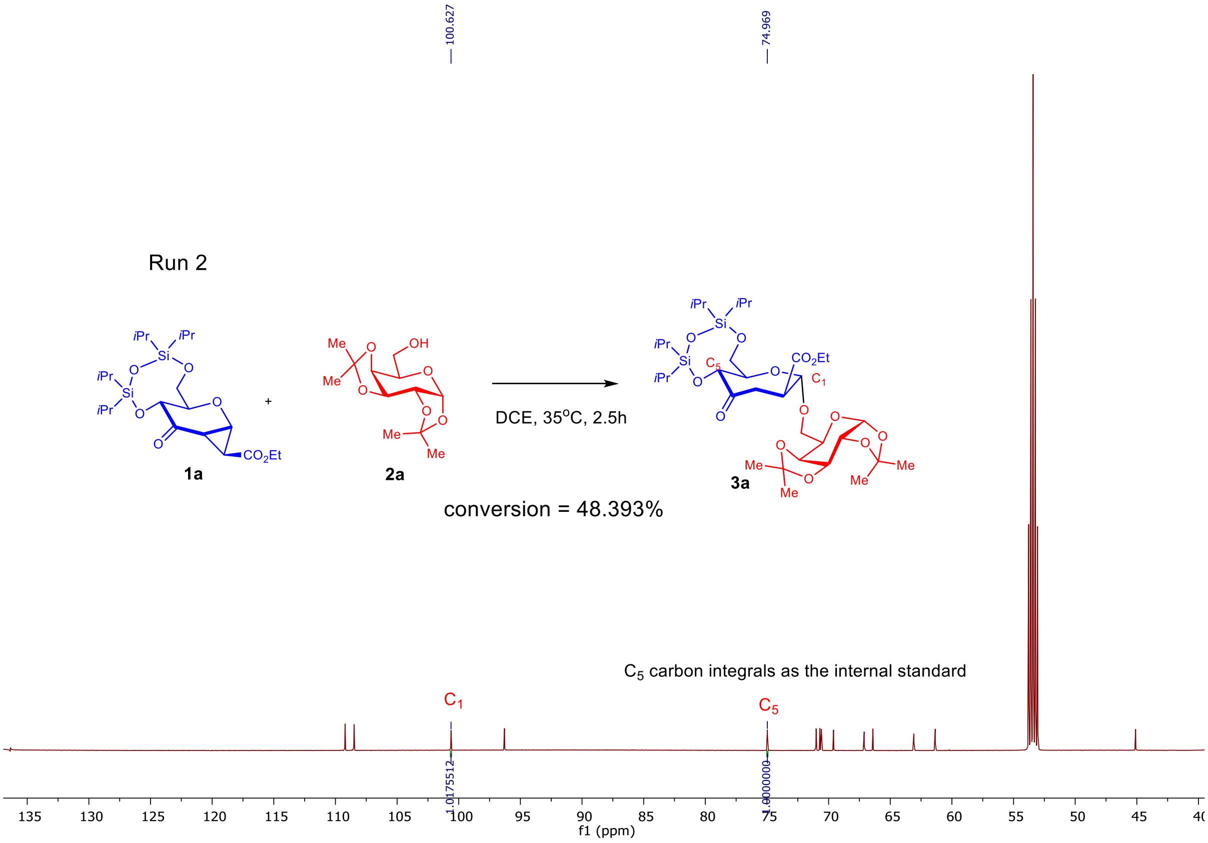

Supplementary Figure 49: Integration of 1st carbon in **3a** (run 2)

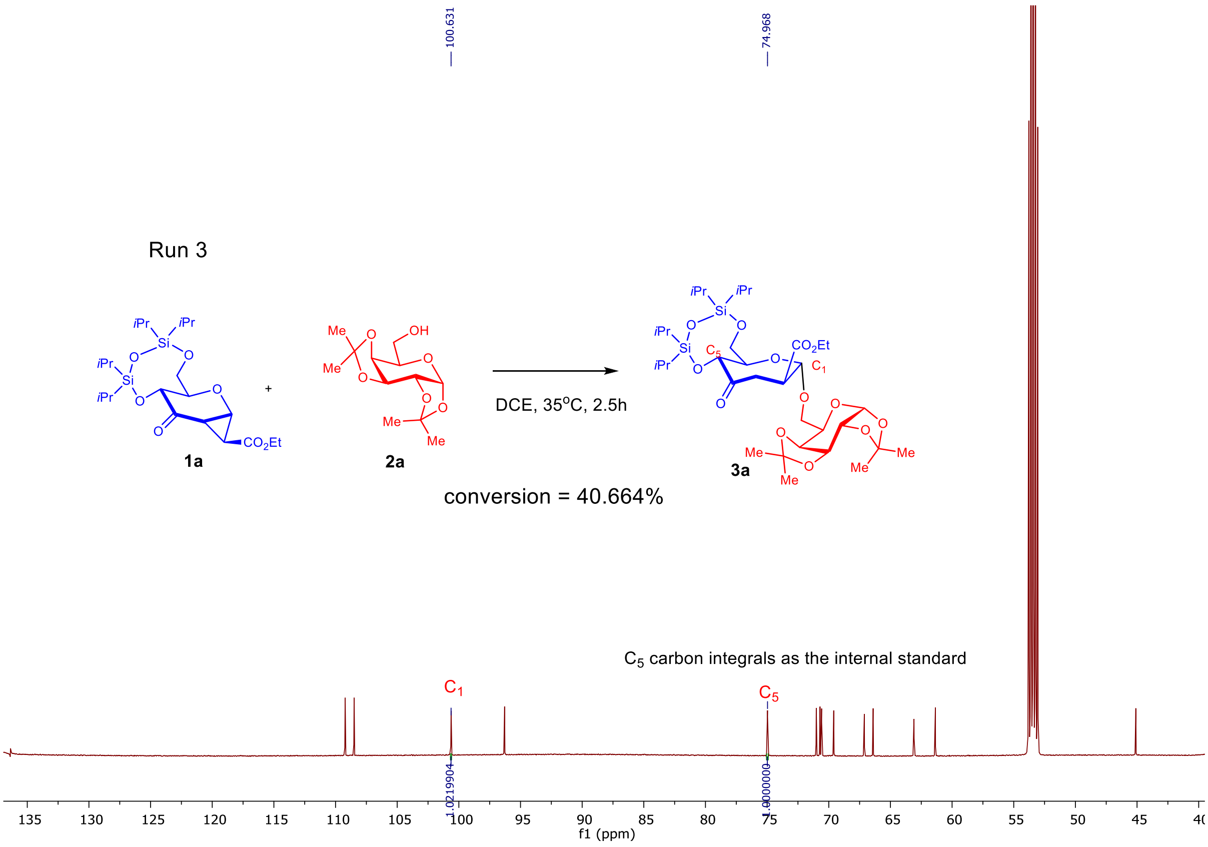

Supplementary Figure 50: Integration of 1st carbon in **3a** (run 3)

Computational Details

All quantum chemical calculations were performed with the developmental version of the Orca 5.0.4 program package,[22-23] and its interface to the open sourced stand-alone xTB code.[24] All molecular geometries were preliminarily optimized using the Grimme's semi-empirical GFN2-xTB hamilitonian[24] with the analytical linearized Poisson-Boltzmann (ALPB) implicit solvation model for CH_2Cl_2.[25] The iMTD-GC workflow of the open source CREST[26] code developed by Grimme et al was subsequently used at the same level of theory to search for the ensemble of energetically lowest lying conformers for the catalyst **A**-glycosyl donor-glycosyl acceptor complex **9**.

After scrutinizing multiple plausible conformers, the energetically lowest conformer from CREST was determined to be geometrically representative, which was then further optimized (utilizing TIGHTSCF settings) at a higher level using the meta-hybrid M06-2X functional[27] developed by Trular et al. with tight integration grids (DEFGRID3 setting in Orca), corrected using using the D3(0) dispersion model (the zero-damping variant recovers the long-range contribution, which is deficient in M06-2X),[28-29] and using the def2-SVP basis set.[30] The M06-2X functional has been demonstrated in benchmark studies[31-32] to be well suited in describing chalcogen bonding and non-covalent interactions in general.

The default Gaussian charge scheme with a scaled vdW-type cavity of the conductor-like polarizable continuum (CPCM) solvation model for 1,2-dicholoroethane (ε (dielectric constant)=10.36 and μ (refractive index)= 1.4448) as implemented in Orca was applied.[33-34] The resolution-of-identity approximation[35] for Coulomb integrals and COSX numerical integration for HF exchange (RIJCOSX) was applied along with the appropriate corresponding auxiliary basis sets.[36-37] Harmonic frequencies calculations at 298.15 K were computed at the same level of theory and used to verify the nature of the optimized stationary point as minima (no imaginary frequencies, threshold set at $i\omega < 10\ \mathrm{cm^{-1}}$) based on the modified ideal gas-rigid rotor-harmonic oscillator (RRHO) model.[38] Spectator counteranions were excluded in the computations.

The independent gradient model based on the Hirshfeld partition of molecular density (IGMH analysis)[39] by Lu et al. was performed to reveal the inter-fragment noncovalent isosurfaces, colored by the mapped function sign(λ_2)ρ, using algorithms implemented by Lu et al. in Multiwfn 3.8.[40] IGMH isosurfaces were then rendered using VMD 1.9.4a51.

CYLView[41] rendering of stationary points

The cartesian coordinates (in Å) of the optimized stationary point (minima) of complex **9** at the M06-2X-D3(0)/def2-SVP/CPCM(1,2-DCE) level of theory is provided. The geometry was rendered using CYLView20. (Red atoms = oxygen, grey atoms = carbon, yellow atoms = phosphorus, purple atoms = selenium, light orange atoms = silicon, white atoms = hydrogen). NCIs are denoted in dotted lines and the distances are labelled in Å.

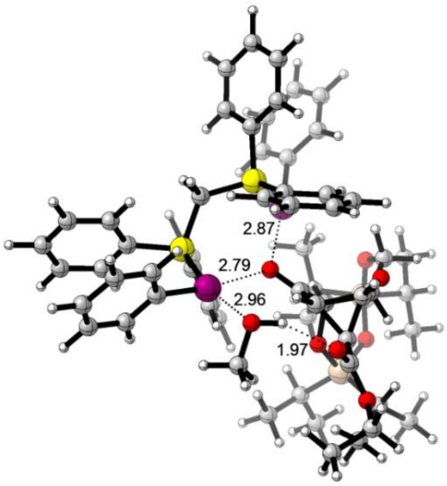

Encounter Complex 9

150			
C	3.63476609443541	4.07691567511775	1.06911451216994
O	3.56931823979110	2.96175339850730	1.92982728207562
H	2.64768439473929	4.33604001497753	0.64553150386965
H	4.30727445975796	3.82466321389039	0.23597516126120
H	4.04210502245827	4.96772151681495	1.57595372304792
H	2.95524763046179	3.19329098079954	2.64478408740061
H	-2.02803084256210	-0.68878208474938	-4.67847670924557
H	1.13533805871799	-6.23705693948077	4.26534345053469
C	-1.13200233027936	-0.72224144050192	-4.05555512208921
H	-1.18325065538045	1.39083115314537	-3.60933300237396
H	1.08954023282148	-8.03818107733231	2.54918025008380
C	-0.66436279421116	0.44536424958869	-3.45045149895384
H	-0.80857070683397	-2.83998119215816	-4.35802744436150
C	1.12401872501734	-5.98406968166603	3.20458036764829
C	-0.45343631752060	-1.92890234259406	-3.87444338313688
C	1.10090980420046	-6.99152952292109	2.24089473958907
C	1.12816369348646	-4.64398227058174	2.81818009059825
C	0.46870289071174	0.41196706139901	-2.63907884806524
H	1.13744691567192	-3.86114615932180	3.57832480816112
C	0.68095123522567	-1.97405078218350	-3.06785656155221
C	1.09514756763633	-6.66762898313455	0.88122985107380
H	2.25970709133011	-3.24893944985492	-0.96520095629507
H	0.82491906126685	1.32374362134251	-2.15465743264940
H	1.07744737671535	-7.45559387647862	0.12719856968457
C	1.12986995298136	-0.80303199043742	-2.43880652439007
H	1.20260646832806	-2.92540773628896	-2.93612082220207
H	3.27740861118181	-2.54336411522637	0.28740535964641
C	1.11810916829349	-4.32291781310154	1.45511531897946
C	2.34341654018714	-2.39147172726236	-0.27803620263623
C	1.10381609437179	-5.33508120552045	0.48325215834928
H	4.42378196832030	1.87990000894255	-2.66193910065760
P	2.57534938108541	-0.92550097516062	-1.36266143689045
H	1.07553508108589	-5.09593441438316	-0.58254290070745
P	0.99307769734218	-2.59858473469254	0.94380775767252
H	6.82703671147058	2.14654874386642	-3.25247639440335
H	-0.80961200325922	-1.60164027088236	2.98414058639789
H	3.08855163252014	-0.64662715163071	-4.20596622071062
C	5.20094389256028	1.63417055122917	-1.93550979749111
C	6.54793597376448	1.78308509851934	-2.26212759631989
C	3.99631601520491	-1.02628773046042	-3.73280827020869
C	4.01916206899442	-1.32294463076419	-2.36426411938954
C	0.19785954893995	-1.25973362677072	3.23184797076553
Se	-1.07943597861730	-2.27260319446788	0.08919834140967
C	1.27375879029469	-1.54778508900338	2.38277470395301
C	5.15067918196522	-1.21266877939694	-4.49243231157653
Se	3.01667874349348	0.93746769659771	-0.16420652618592
C	4.85549557689332	1.14938552776142	-0.67043394142379
C	5.19158097139786	-1.79381961382360	-1.75447725685220
H	5.13542675201894	-0.98597301935685	-5.55912238725806
C	7.53518318898192	1.47712424220742	-1.32166310329984
H	5.22914040216088	-2.00123817609923	-0.68295637075648
H	-0.41790786242426	-0.30498529569400	5.05663602347740
C	0.42101642456010	-0.53028505284122	4.39665517342304
H	8.58813964105837	1.59973691841754	-1.58060529197564
H	-2.01003703049415	-4.00258519906927	2.36046396267589
C	6.31793628716513	-1.67991356435037	-3.88890056174745
C	6.33795389721533	-1.97249147806924	-2.52253284191158
C	2.56538822720990	-1.10534570148694	2.69198608485949
C	5.83543932537471	0.85321503715675	0.28049869568213

C	-1.72457601783878	-4.07456461140551	0.21562439925725
C	7.18028845598079	1.02485648720794	-0.04989205810550
C	-2.09677033926148	-4.60582786748073	1.45418023828603
H	7.22050695447075	-1.81812602022266	-4.48634729961923
H	3.41599867083418	-1.32905239507161	2.04780922438172
H	7.25192522438119	-2.33544620573523	-2.05057119826455
C	1.70367545819637	-0.06896111200168	4.69947874777717
C	2.77186758142738	-0.35282882228893	3.84776718966807
H	5.54781734870938	0.48934030463364	1.26890079664614
H	7.95163714884737	0.79660462746064	0.68716846692881
C	-1.84817009381472	-4.82380081690704	-0.95800713461393
C	-2.56179712959326	-5.91834536333043	1.51850028872124
H	-1.55776960134899	-4.39215963670129	-1.91833166036849
H	1.87160157636993	0.51701010086381	5.60464018404273
H	-2.84086329211690	-6.34588614856593	2.48280511762714
H	3.77447915688031	0.00794200394030	4.08140694956618
C	-2.33388404399049	-6.12952043884222	-0.88455325081509
C	-2.67813204792920	-6.67839567389717	0.35173969999865
H	-2.43393584091877	-6.72106314813184	-1.79574605472617
H	-3.04663859709472	-7.70403395123412	0.40685505981578
H	0.85109770808072	6.41601740738081	4.10038819303882
C	0.38244647915728	6.69775939042234	3.14884301277201
H	-0.42220861080975	7.41623144556092	3.34097315230919
H	0.90805028024650	7.48686376418642	1.20863751869911
C	1.39396452392636	7.24024421065403	2.16268902805204
O	-0.28728166888301	5.54175093604319	2.60782108717138
H	-0.28373795923445	2.06949220382816	3.97007505090132
H	1.84935186610631	8.15359729138655	2.56875555403533
C	-0.73173838955039	2.07163478557216	2.97516455872885
C	0.30481870825282	4.36857760978405	2.72712815175393
H	2.19054123453232	6.50695705399321	1.98013901170728
O	1.37065610848180	4.18901663411599	3.27568093189148
O	-1.88280372266428	1.31783996894383	2.93668336854489
H	1.31049330593372	1.95403244598542	2.01410364083991
C	0.23949892456291	1.99417694052424	1.80459759661750
C	-0.51716422139074	3.27916375229366	2.12949894190274
H	-4.34244637729208	0.26450053920711	2.58134016967832
H	-2.91445233379770	-0.76173463926595	2.29819828342980
C	-2.63649691203186	1.28407510553591	1.73354112022804
C	-3.53506907886578	0.03877423776823	1.86549390234797
C	-0.29565617734777	1.10096075581541	0.77437327866531
H	-1.29897188169845	3.60101569178403	1.44377448383100
O	0.36043531625667	0.20818949951031	0.26640833170184
H	-3.25753254405207	2.19340885988828	1.68942219355713
C	-1.77181045942448	1.25247804063596	0.43580077292699
H	-7.25722609275522	-1.51898538276122	-1.38616741654850
H	-6.64548020208992	-1.93956962646482	0.23149709457117
H	-6.82110258076348	0.49207597921110	0.90593902153973
O	-4.03647258582382	-0.42535204748653	0.64500006641768
C	-7.26347778062479	-1.21878747492654	-0.32601289527931
H	-2.04414726015922	0.37231441731298	-0.16397120088857
H	-2.96726509157653	6.15896636881974	-1.26631388695822
O	-1.88991633832101	2.41684318353919	-0.34597809225490
C	-6.75586728493225	0.21930459982027	-0.16395585034617
C	-2.76013360211651	5.77930352985111	-0.25353264909688
H	-8.29937680778161	-1.31223495558635	0.03525822721095
Si	-4.93072314760349	0.34556231627773	-0.54984669890200
H	-1.70143455791322	5.47545427583926	-0.21767079813859
H	-3.66453560896064	-2.27971822068426	-1.16290081678314
H	-8.65740126142234	1.16787125022822	-0.65336423171546

H	-2.88964590254603	6.61984257346840	0.44608548187357
C	-7.60183050502092	1.21699683056757	-0.96368860779317
H	-5.44783164792087	5.36170419257093	-1.00692531805855
Si	-3.35407309511793	3.07848502223599	-0.89457052548091
O	-4.53600061806437	1.96695099512463	-0.53038223329420
H	-5.16179712614008	-2.43195926637395	-2.10141202867977
C	-4.17226432895793	-1.94988344174907	-2.08198340055907
H	-4.32188478789575	3.93378111703061	-4.48038132433680
C	-3.70911092761429	4.62776033476904	0.10246376763777
H	-7.25289128619088	2.25012355247333	-0.82118861709838
C	-5.18015478282797	5.05311852556512	0.01526520948513
C	-4.29837850450174	-0.42371035181318	-2.14166686688284
H	-7.56627621028778	0.99581364423106	-2.04195711409293
C	-4.37190833470020	3.98722076467302	-3.38160771015602
H	-5.37415033297699	5.91012843463112	0.67901436485208
H	-5.33135468009573	3.54729581355821	-3.06620027684751
H	-3.60118835538499	-2.33033477539898	-2.94529920947013
H	-3.27176393950906	-0.00850367653947	-2.21518160648927
C	-3.17134234385788	3.27462852423367	-2.74735921056738
H	-3.50906926972703	4.33449748181932	1.15012645068399
H	-5.85383779826368	4.23491123830826	0.30806824019518
H	-3.16804568631267	2.22648909601994	-3.10314129412707
H	-5.20293548817732	1.12326187335230	-3.42017224302896
C	-5.08713401102369	0.02895162173166	-3.37588084296795
H	-4.38494237503388	5.05298092448872	-3.10394440391667
H	-4.58639061547451	-0.29356053007564	-4.30293729272403
C	-1.84264691206885	3.92194042830305	-3.15979548853354
H	-0.98360489678223	3.45311725575094	-2.65628403235277
H	-6.09622580908319	-0.41296703505938	-3.37615071429744
H	-1.69078561003635	3.83635466091486	-4.24762272254534
H	-1.82923435433088	4.99321092627181	-2.91047646886358

IGMH analysis of complex 9[39]

(Green isosurfaces denotes intermolecular weak van der Waals interactions)

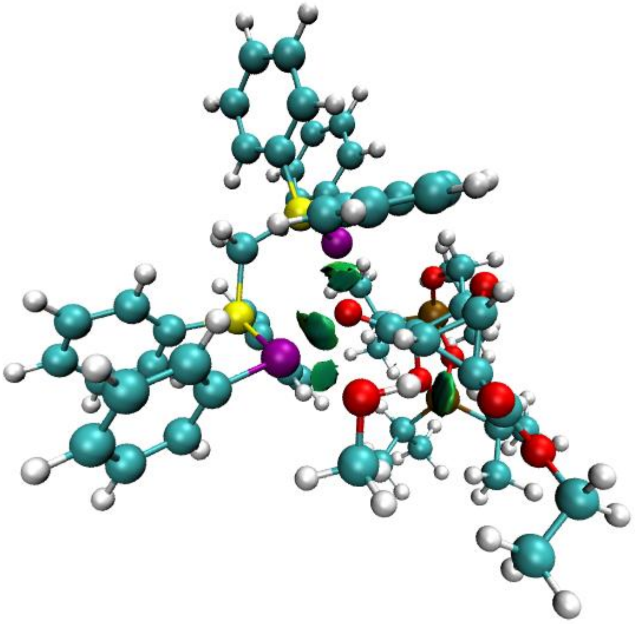

(Red atoms = oxygen, cyan atoms = carbon, yellow atoms = phosphorus, purple atoms = selenium, light orange atoms = silicon, white atoms = hydrogen)

Perspective of DFT optimized geometry of complex 9 when the glycosyl acceptor oxygen is superimposed with anomeric carbon

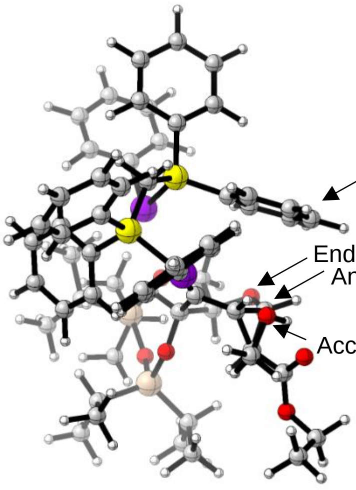

In this perspective of complex **9**, the C1 anomeric carbon of the glycosyl donor is superimposed with the glycosyl acceptor's oxygen. This superimposition offers insight that an envisaged direct attack path from the hydroxyl oxygen toward the anomeric carbon to construct the α-glycosidic linkage is not likely hindered by the cyclopropane's C-H bonds.

One could also envision from this view the donation of the lone pair of electrons from the hydroxyl's oxygen into the σ* orbital of the endocyclic O-C1 bond is feasible, which should be pointing out of the plane and pointing toward the hydroxyl's oxygen.

Further, the orientation of the glycosyl acceptor in this modelled ternary complex is below the pyranoside plane. Note worthily, one of the phenyl rings of the catalyst is shielding the β-face of the glycsoyl donor in the DFT model. The steric hindrance imposed by this top-face shielding is likely preventing upward folding of the *exo*-substituent, favoring the front face S_Ni pathway instead.

Supplementary Discussion

Supplementary Discussion 1

In our ^{31}P NMR titration, while we noticed the formation of a new major downfield doublet peak at 36.5 ppm when we titrated catalyst **A** against isopropanol till a ratio of 1:7. At extremely high concentrations of isopropanol especially beyond 1:15 ratio (**Supplementary Figure 6**), we observed the formation of another major new doublet at around 26 ppm, suggesting the existence of another noncovalent supramolecular species. We surmise that the ancillary pnictogen bond between the catalyst's phosphonium ion and acceptor is concentration dependent, and different supramolecular species at high concentrations could likely arise due to dynamic interchanging between the monodentate and bi-dentate complexes, or due to formation of oligomeric species.

We further conducted a control experiment using a phosphonium salt without selenium (**Supplementary Table 1**, Entry 26) at standard conditions. The absence of any conversion in this control experiment substantiates that purely pnictogen bonding interactions of a P-O nature is unlikely a dominant factor contributing to the catalysis.

Supplementary Discussion 2

In our opinion, the *in-situ* ^{77}Se NMR data (**Supplementary Figure 10-6**) is inconclusive as there are substantial limitations in the technique for complex detection:

a) There is significant peak broadening during the *in-situ* detection which raises the possibility of peak coalescence, obscuring putative complex peaks. This limitation is common and a known occurrence in the usage of ^{77}Se NMR in the discrimination of diastereotopic peaks. (Duddeck, H., ^{77}Se NMR Spectroscopy and Its Applications in Chemistry. In Annual Reports on NMR Spectroscopy, Academic

Press: 2004; Vol. 52, pp 148). Quoted from the reference literature is the following "*Large diastereomeric dispersions can often be seen at the* ^{77}*Se signal although line broadening due to coalescence may occur.*"

b) The possibility of fast exchange at the ^{77}Se NMR time scale due to the weak reversible interactions holding the ternary complex. This weak interaction postulate is supported by the very low chemical shift perturbation already observed in *in-situ* ^{13}C NMR (~0.1 ppm as mentioned in the manuscript **Figure 2d**). The fast exchange between the ternary complex and its component monomers allows free rotation about the C_2 axis of the catalyst. This swaps the position of the seleniums and averages out the selenium resonances when the decomplexation is faster than the ^{77}Se NMR time scale.

c) Diastereotopic splitting is sensitive to the distance of the selenium nuclei from the chirality centers.

We would like to point out that this distance effect by drawing attention to our ^{1}H NMR spectra inspection on our chiral glycosyl donor and septanoside products (see two examples in the figures below), ^{1}H nuclei only experiences diastereotopic splitting when they are in near proximity to the chirality centers of the sugar; further protons nuclei (> 3 bonds) such as those positioned on the ethyl ester chain, or on the aglycon were observed to be chemically equivalent.

In the top example below, the diastereotopic methylene protons H_7 attached to carbon 7 can be well discerned due to proximity to the stereocentre at C6, but distal protons such as those on C22 and C23 (H_{22} and H_{23}) consistently showed chemical equivalence.

In the bottom example, methylene protons (H_{21}) attached to carbon 21 are well splitted into their diastereotopic counterparts, but observed chemical equivalence were noted on the distal methylene protons, H_{22}, H_{23} and H_{24}.

Note: These are covalent bonds that have much shorter distances than noncovalent interactions (For comparison purpose, a C-C bond is on average 1.54 Å, while selenium-oxygen interactions in our DFT computed complex is almost twice this length).

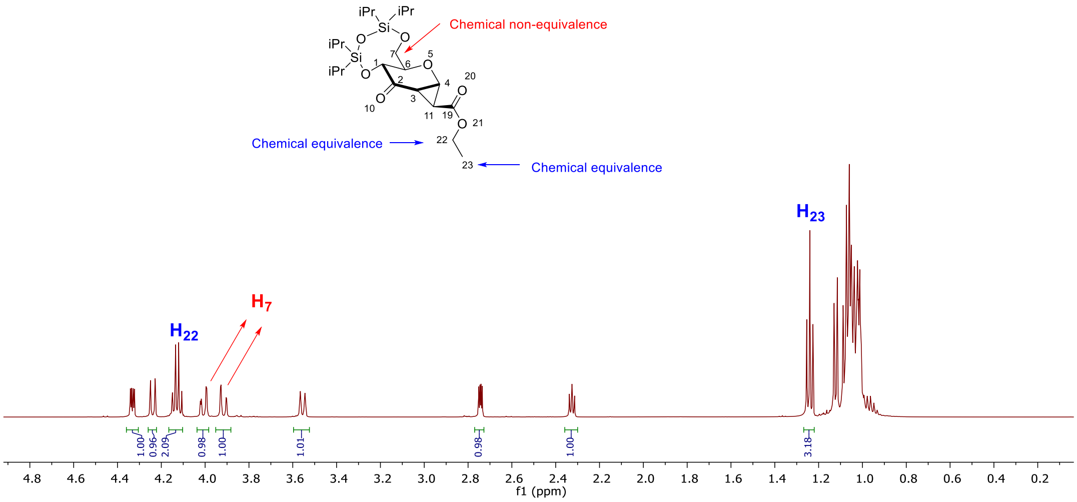

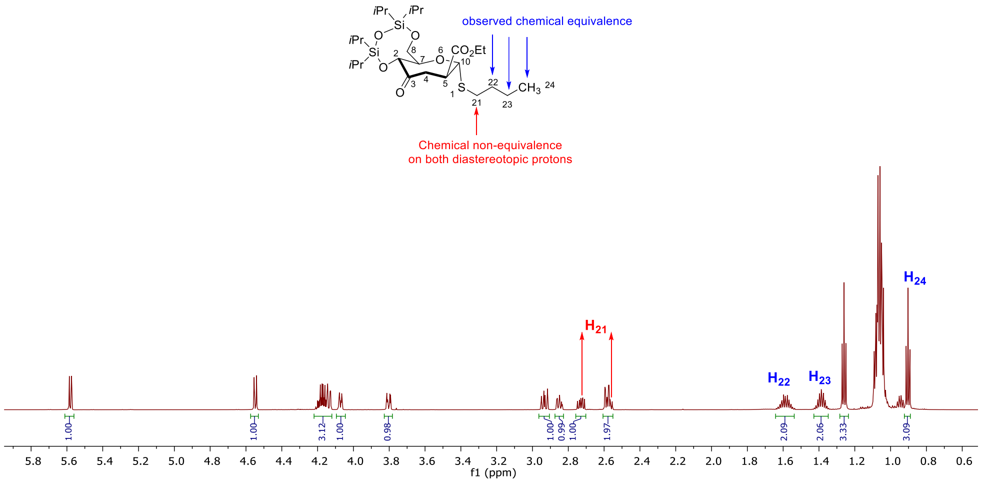

Further, the sensitivity of Se nuclei is much lower than that of H nuclei due to the lower gyromagetic ratio mentioned, which increases the propensity of the observed chemical equivalence of the two Se signals in the catalyst.

Hence, we posit that a larger distance imposed by a weak reversible non-covalent interaction between the selenium and the substrate's ketone oxygen (the ketone's oxygen is still 2 covalent bond distance away from the nearest chirality center), together with the above factors, could potentially explain an overall observed chemical equivalence of the selenium resonances. All these issues hinder meaningful ternary complex interpretation through the diastereotopic effect using *in-situ* ^{77}Se NMR spectroscopy.

References:

1) Dolomanov, O.V.; Bourhis, L.J.; Gildea, R.J.; Howard, J.A.K.; Puschmann, H. OLEX2: a complete structure solution, refinement and analysis program. *J. Appl. Cryst*. **2009**, *42*, 339-341.

2) Sheldrick, G.M. SHELXT – Integrated space-group and crystal structure determination. *Acta Cryst.*, **2015**, *A71*, 3-8.

3) Sheldrick, G.M. A short history of SHELX. *Acta Cryst*., **2008**, *A64*, 112-122.

4) Wang, W.; Zhu, H.; Liu, S.; Zhao, Z.; Zhang, L.; Hao, J.; Wang, Y. Chalcogen−Chalcogen Bonding Catalysis Enables Assembly of Discrete Molecules. *J. Am. Chem. Soc.* **2019**, *141*, 9175−9179.

5) Kong, X.; Zhou, P-P.; Wang, Y. Chalcogen···π Bonding Catalysis. *Angew. Chem. Int. Ed.* **2021***, 60,* 9395–9400.

6) Xu, C.; Loh, C. C. J. A Multistage Halogen Bond Catalyzed Strain-Release Glycosylation Unravels New Hedgehog Signaling Inhibitors. *J. Am. Chem. Soc.* **2019**, *141*, 5381−5391.

7) Boelke, A.; Kuczmera, J. J.; Lork, E.; Nachtsheim, B. J. N-Heterocyclic Iod(az)olium Salts-Potent Halogen-Bond Donors in Organocatalysis. *Chem. Eur. J.* **2021**, *27*, 13128–13134.

8) Tolstikova, L. L., Bel'skikh, A. V., Shainyan, B. A., *Russian Journal of General Chemistry*, **2011**, *81*, 474-480.

9) Hoberg, J. O.; Claffey, D. J. Cyclopropanation of Unsaturated Sugars with Ethyl Diazoacetate. *Tetrahedron Lett*., **1996**, *37*, 2533-2536.

10) Grieco, P. A.; Speake, J. S. Studies directed toward the total synthesis of scytophycin C: Synthesis of the C(1)-C(18) fragment of scytophycin C. *Tetrahedron Lett*., **1998**, *39*, 1275-1278.

11) Calveras, J.; Nagai, Y.; Sultana, I.; Ueda, Y.; Higashi, T.; Shoji, M.; Sugai, T. New chemo-enzymatic route toward N-acetylneuraminic acid derivatives with alkyl groups at C-7 hydroxyl group. *Tetrahedron* **2010**, *66*, 4284-4291.

12) Dowgiallo, M. G.; Miller, B. C.; Kassu, M.; Smith, K. P.; Fetigan, A. D.; Guo, J.J.; Kirby, J. E.; Manetsch, R. The convergent total synthesis and antibacterial profile of the natural product streptothricin F. *Chem. Sci*., **2022**, *13*, 3447-3453.

13) Sridhar, P. R., Venukumar, P. A Ring Expansion-Glycosylation Strategy toward the Synthesis of Septano-oligosaccharides. *Org. Lett*., **2012**, *14*, 5558-5561.

14) Thordarson, P. Determining association constants from titration experiments in supramolecular chemistry. *Chem. Soc. Rev.* **2011**, *40*, 1305–1323.

15) Roger S. Macomber, An introduction to NMR titration for studying rapid reversible complexation, *J. Chem. Educ.* **1992**, *69*, 375-378.

16) Mike P. Williamson, Using chemical shift perturbation to characterise ligand binding*, Progress in Nuclear Magnetic Resonance Spectroscopy*, **2013**, *73*, 1-16.

17) http://supramolecular.org

18) Brynn Hibbert, D.; Thordarson, P. The death of the Job plot, transparency, open science and online tools, uncertainty estimation methods and other developments in supramolecular chemistry data analysis. *Chem. Commun*. **2016**, *52*, 12792–12805.

19) Burés, J. A Simple Graphical Method to Determine the Order in Catalyst. *Angew. Chem. Int. Ed*. **2016**, *55*, 2028-2031.

20) Nielsen, C. D.-T.; Burés, J. Visual kinetic analysis, *Chem. Sci*., **2019**, *10*, 348-353.

21) a) Nishi, N., Sueoka, K., Iijima, K., Sawa, R., Takahashi, D., Toshima, K. Stereospecific β-L-Rhamnopyranosylation through an S_Ni-Type Mechanism by Using Organoboron Reagents. *Angew. Chem. Int. Ed.* **2018**, *57*, 13858-13862; b) Tanaka, M.; Nakagawa, A.; Nishi, N.; Iijima, K.; Sawa, R.; Takahashi, D. & Toshima, K. Boronic-Acid-Catalyzed Regioselective and 1,2-*cis*-Stereoselective Glycosylation of Unprotected Sugar Acceptors *via* S_Ni-Type Mechanism. *J. Am. Chem. Soc.* **2018**, *140*, 3644-3651.

22) Neese, F. The ORCA program system*. Wiley Interdiscip. Rev. Comput. Mol. Sci.* **2012**, *2*, 73-78.

23) Nesse, F. Software update: The ORCA program system—Version 5.0. *WIREs Comput. Mol. Sci.* **2022**, 5, e1606.

24) Bannwarth, C.; Ehlert, S.; Grimme, S. GFN2-xTB — An Accurate and Broadly Parametrized Self-Consistent Tight-Binding Quantum Chemical Method with Multipole Electrostatics and Density-Dependent Dispersion Contributions. *J. Chem. Theory Comput.* **2019**, *15*, 1652-1671.

25) Ehlert, S.; Stahn, M.; Spicher, S.; Grimme, S. Robust and Efficient Implicit Solvation Model for Fast Semiempirical Methods. *J. Chem. Theory Comput.* **2021**, *17*, 4250-4261.

26) Pracht, P.; Bohle, F.; Grimme, S. Automated exploration of the low-energy chemical space with fast quantum chemical methods. *Phys. Chem. Chem. Phys.* **2020**, *22*, 7169-7192.

27) Zhao, Y.; Truhlar, D. G., The M06 suite of density functionals for main group thermochemistry, thermochemical kinetics, noncovalent interactions, excited states, and transition elements: two new functionals and systematic testing of four M06-class functionals and 12 other functionals. *Theor. Chem. Acc.* **2008**, *120*, 215-241.

28) Grimme, S.; Antony, J.; Ehrlich, S.; Krieg, H. A consistent and accurate *ab initio* parametrization of density functional dispersion correction (DFT-D) for the 94 elements H-Pu. *J. Chem. Phys.* **2010**, *132*, 154104.

29) Grimme, S.; Hansen, A.; Brandenburg, J. G.; Bannwarth, C., Dispersion-Corrected Mean-Field Electronic Structure Methods. *Chem. Rev.* **2016**, *116*, 5105-5154.

30) Weigend, F.; Ahlrichs, R., Balanced basis sets of split valence, triple zeta valence and quadruple zeta valence quality for H to Rn: Design and assessment of accuracy. *Phys. Chem. Chem. Phys.* **2005,** *7*, 3297-3305.

31) de Azevedo Santos, L.; Ramalho, T. C.; Hamlin, T. A.; Bickelhaupt, F. M., Chalcogen bonds: Hierarchical ab initio benchmark and density functional theory performance study. *J. Comput. Chem.* **2021,** *42*, 688-698.

32) Bauzá, A.; Alkorta, I.; Frontera, A.; Elguero, J., On the Reliability of Pure and Hybrid DFT Methods for the Evaluation of Halogen, Chalcogen, and Pnicogen Bonds Involving Anionic and Neutral Electron Donors. *J. Chem. Theory Comput.* **2013,** *9*, 5201-5210.

33) Garcia-Ratés, M.; Nesse, F. Effect of the Solute Cavity on the Solvation Energy and its Derivatives within the Framework of the Gaussian Charge Scheme. *J. Comput. Chem.* **2020**, *41*, 922-939.

34) Barone, V.; Cossi, M. Potential energy surfaces for the low-lying ^2A‘ and ^2A’ States of HO_2: Use of the diatomics in molecules model to fit *ab initio* data**.** *J. Phys. Chem. A* **1998**, *102*, 1995.

35) F. Neese, F. Wennmohs, A. Hansen, U. Becker. Efficient, approximate and parallel Hartree–Fock and hybrid DFT calculations. A ‘chain-of-spheres’ algorithm for the Hartree–Fock exchange. *Chem. Phys.* **2009**, *356*, 98-109.

36) K. Eichkorn, O. Treutler, H. Oehm, M. Häser, R. Ahlrichs. Auxiliary basis sets to approximate Coulomb potentials *Chem. Phys.* **1995**, *242*, 652-660.

37) K. Eichkorn, F. Weigend, O. Treutler, R. Ahlrichs. Auxiliary basis sets for main row atoms and transition metals and their use to approximate Coulomb potentials. *Theor. Chem. Acc.* **1997**, *97*, 119-124.

38) Grimme, S. Supramolecular Binding Thermodynamics by Dispersion-Corrected Density Functional Theory. *Chem. Eur. J.* **2012**, *18*, 9955-9964.

39) Lu, T.; Chen, Q., Independent gradient model based on Hirshfeld partition: A new method for visual study of interactions in chemical systems. *J. Comput. Chem.* **2022**, *43*, 539-555.

40) T. Lu, F. Chen, Multiwfn: A Multifunctional Wavefunction Analyzer, *J. Comput. Chem.* **2012**, *33*, 580-592.

41) CYLview20; Legault, C. Y., Université de Sherbrooke, **2020** (http://www.cylview.org)

NMR Spectra

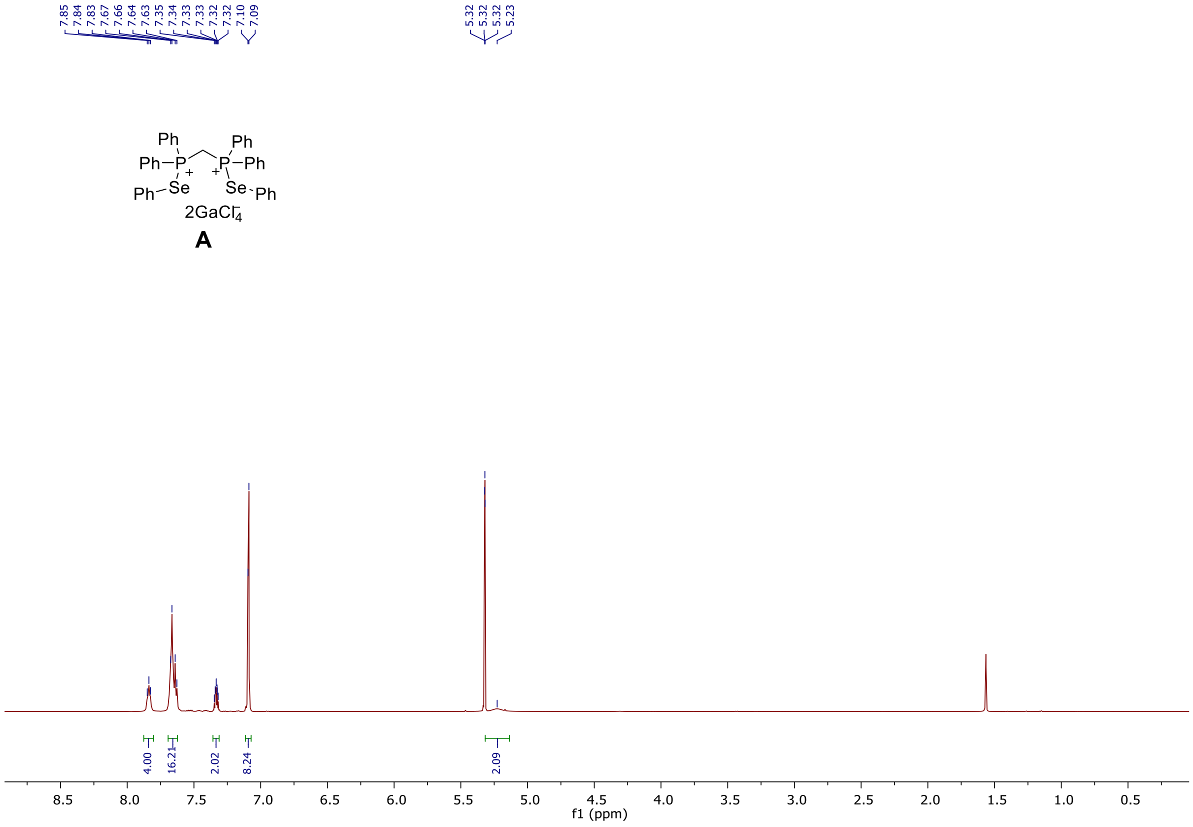

Supplementary Figure 51: ^{1}H spectra for **A**

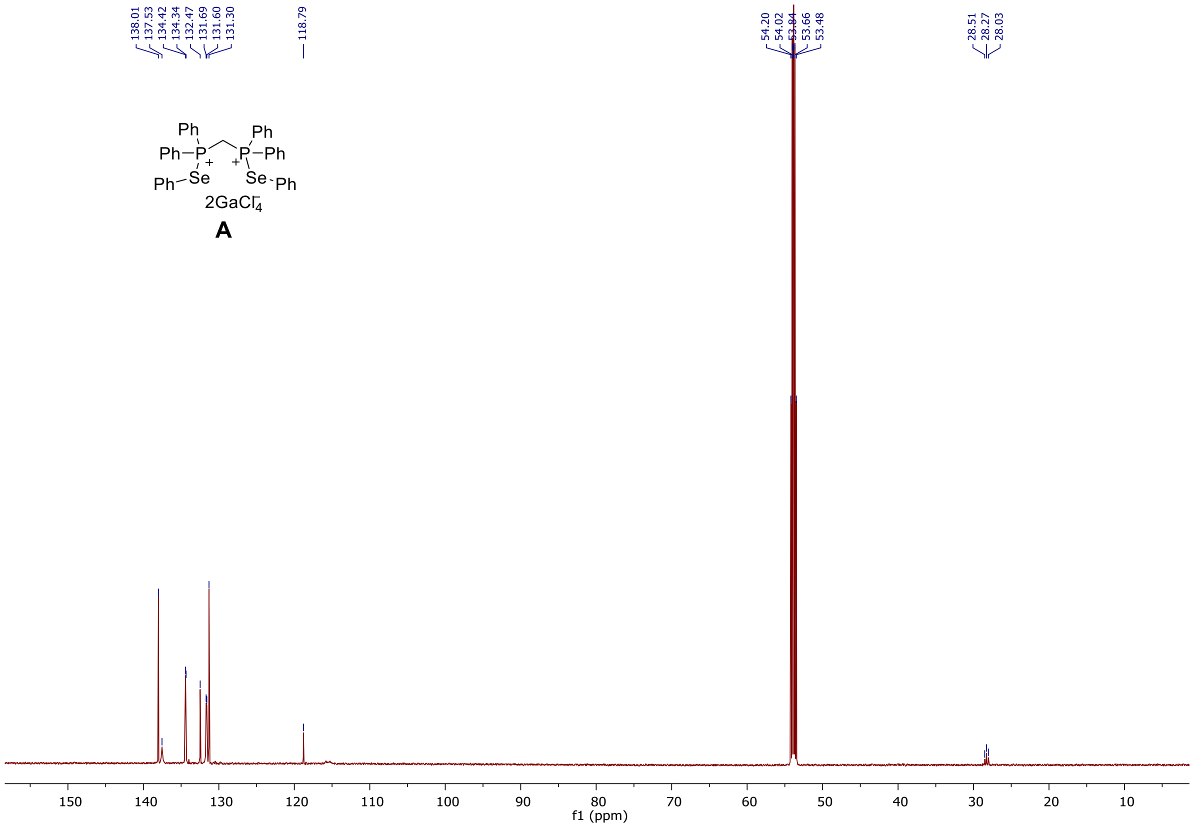

Supplementary Figure 52: ^{13}C spectra for **A**

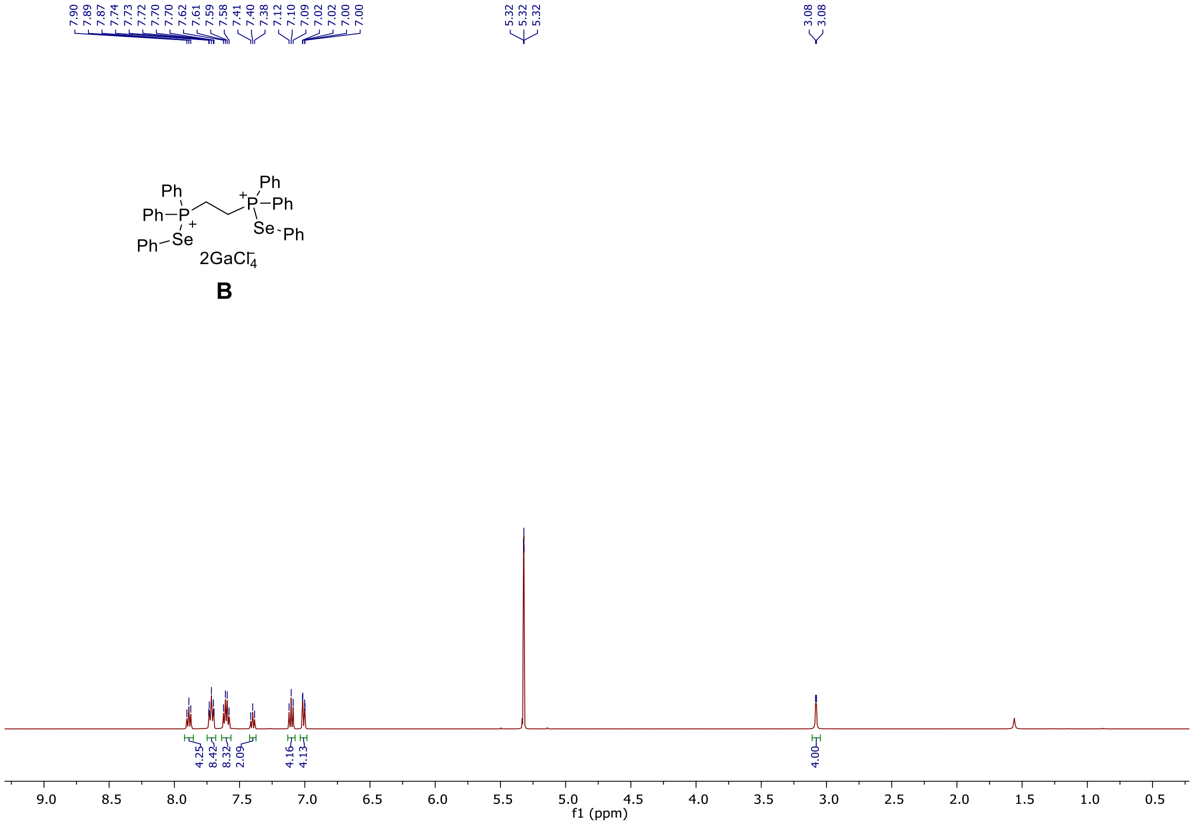

Supplementary Figure 53: ^{1}H spectra for **B**

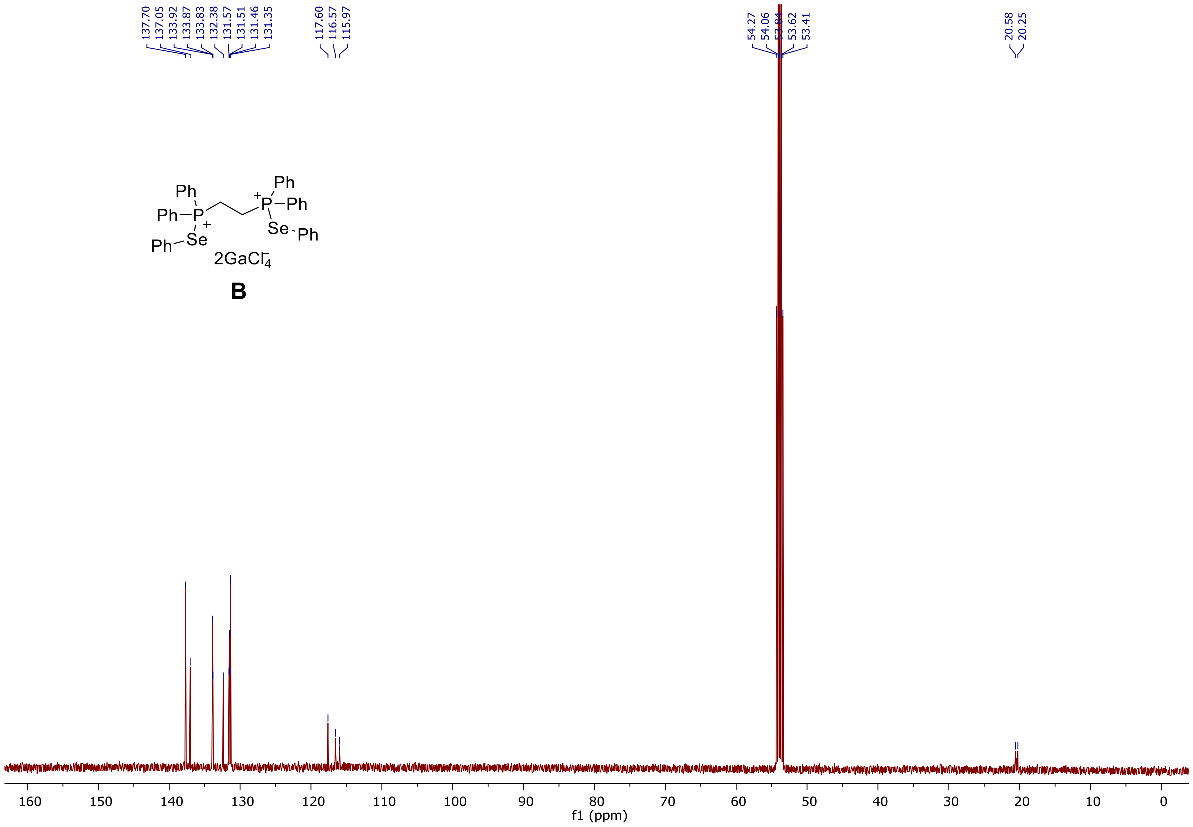

Supplementary Figure 54: ^{13}C spectra for **B**

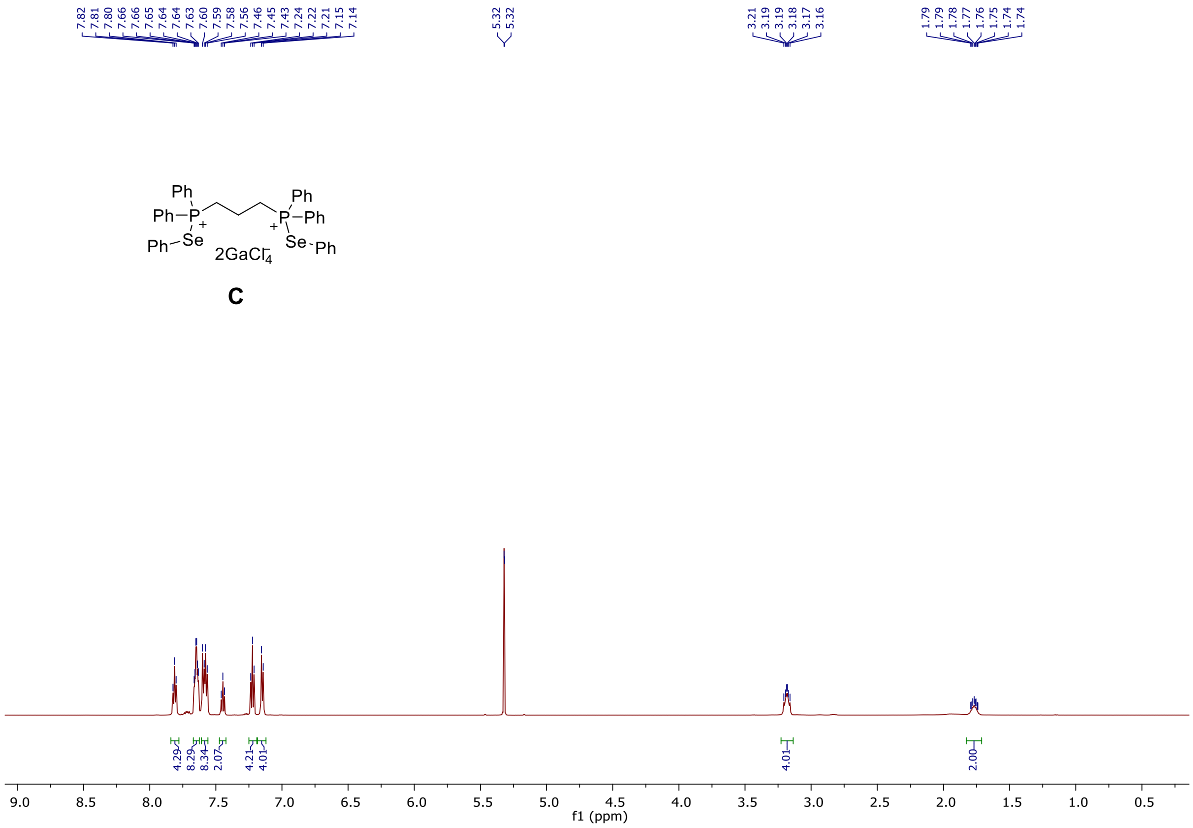

Supplementary Figure 55: ^{1}H spectra for **C**

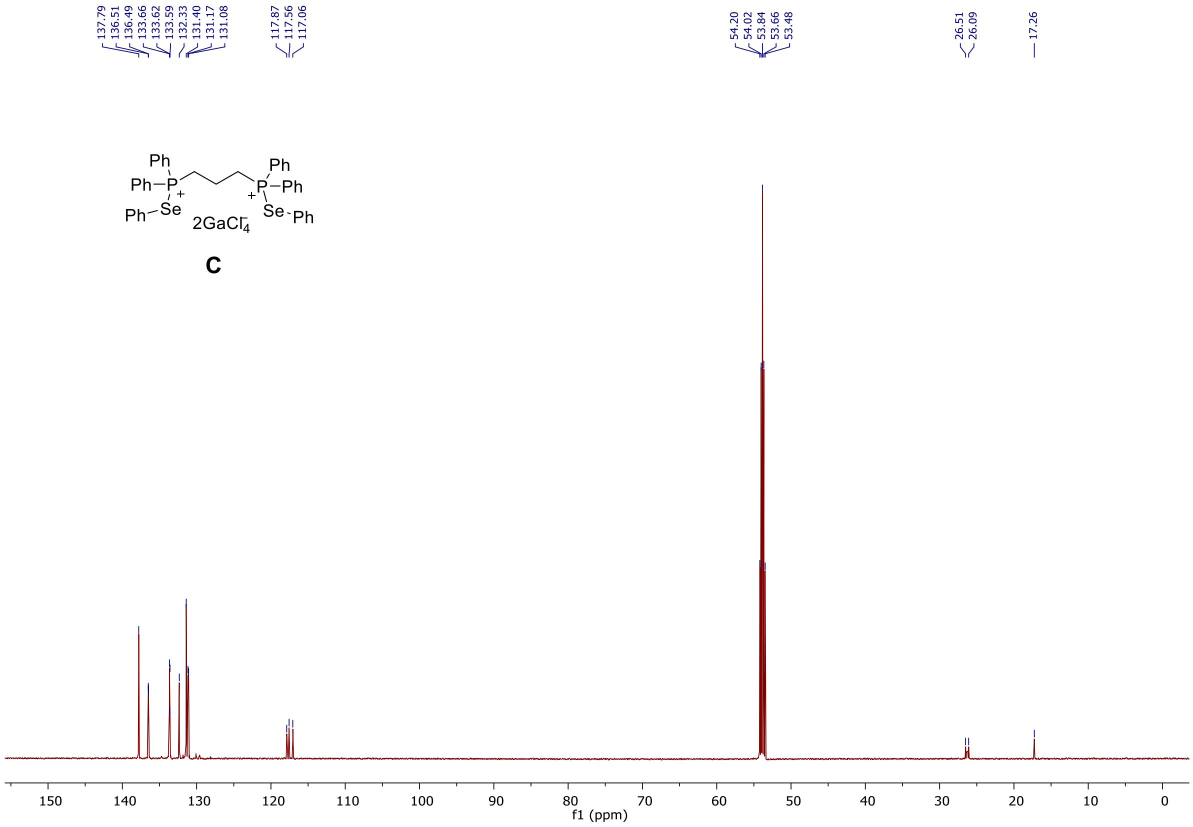

Supplementary Figure 56: ^{13}C spectra for **C**

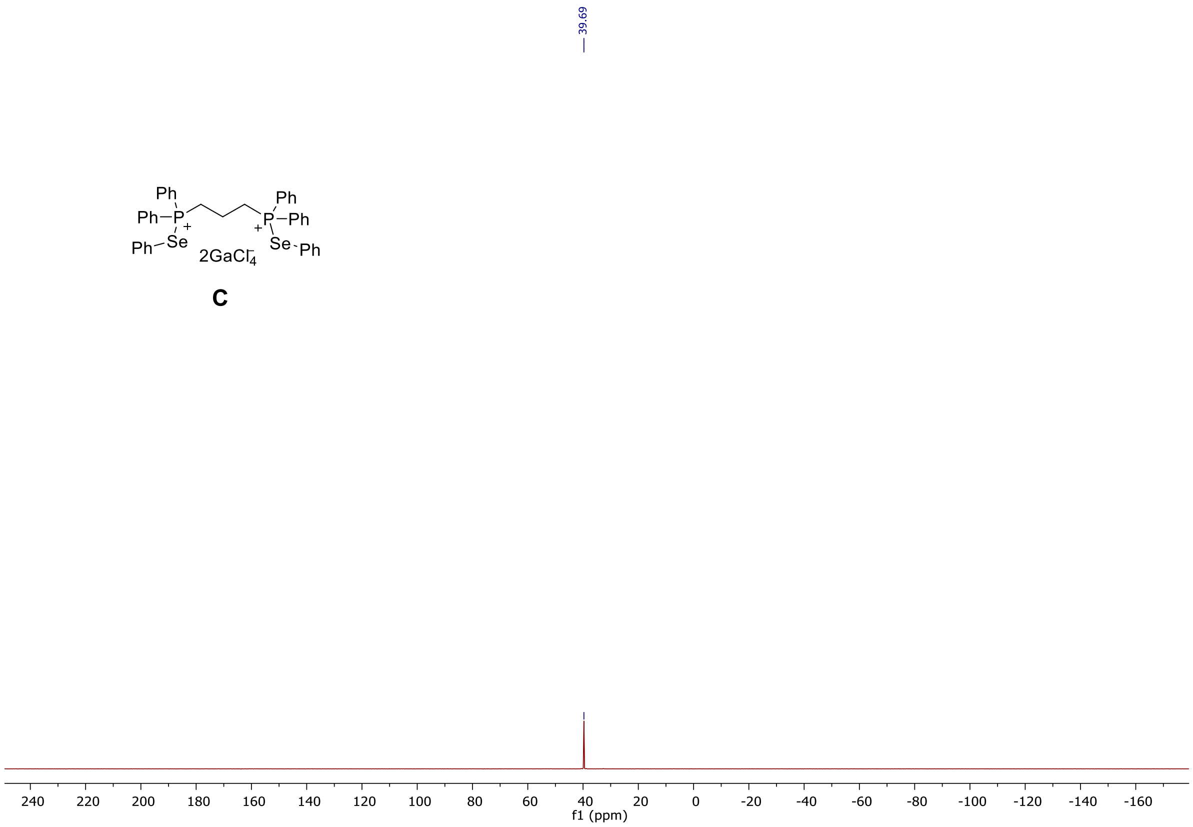

Supplementary Figure 57: ^{31}P spectra for **C**

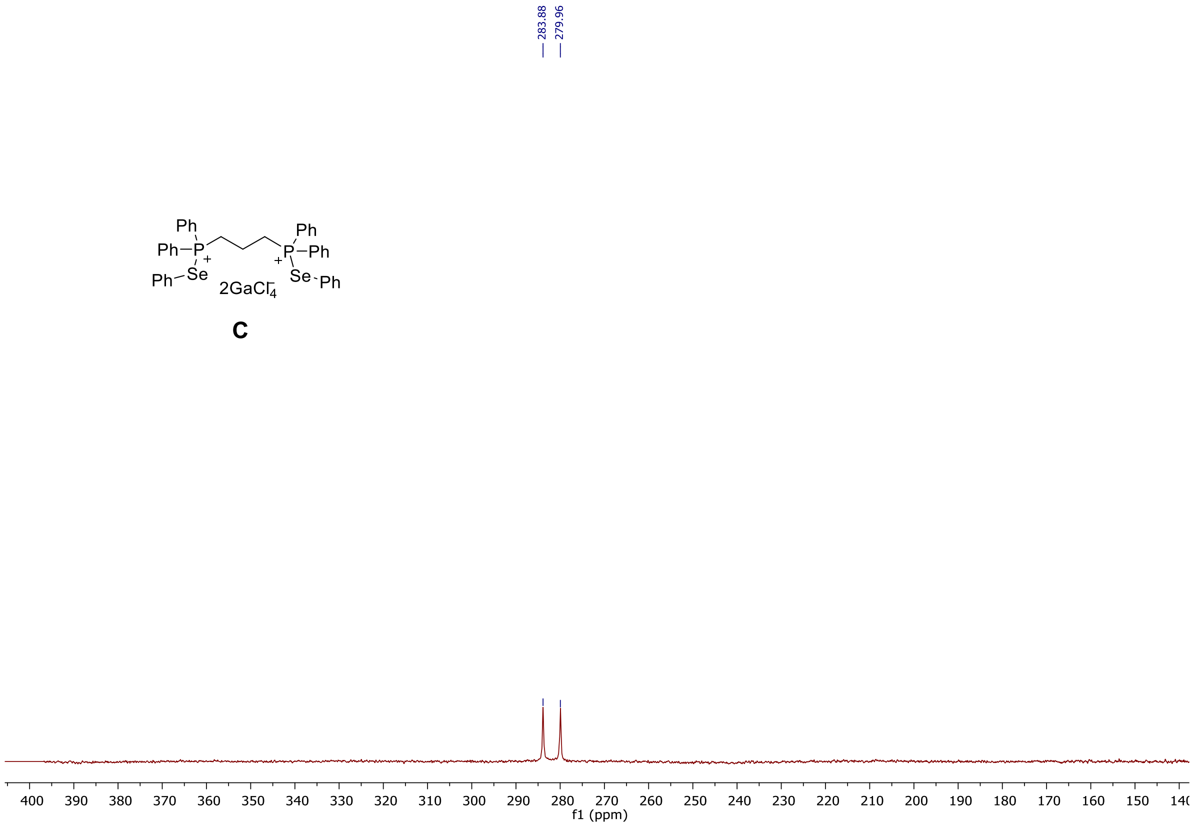

Supplementary Figure 58: ^{77}Se spectra for **C**

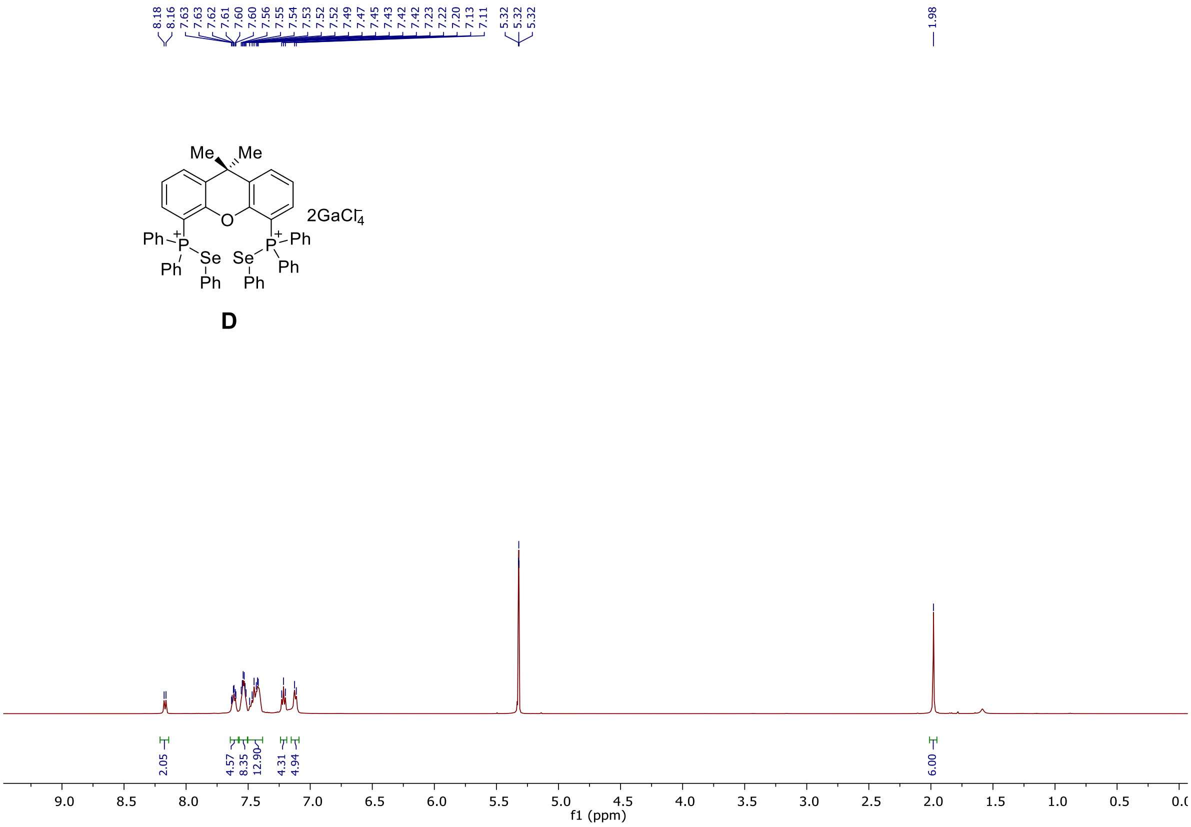

Supplementary Figure 59: ^{1}H spectra for **D**

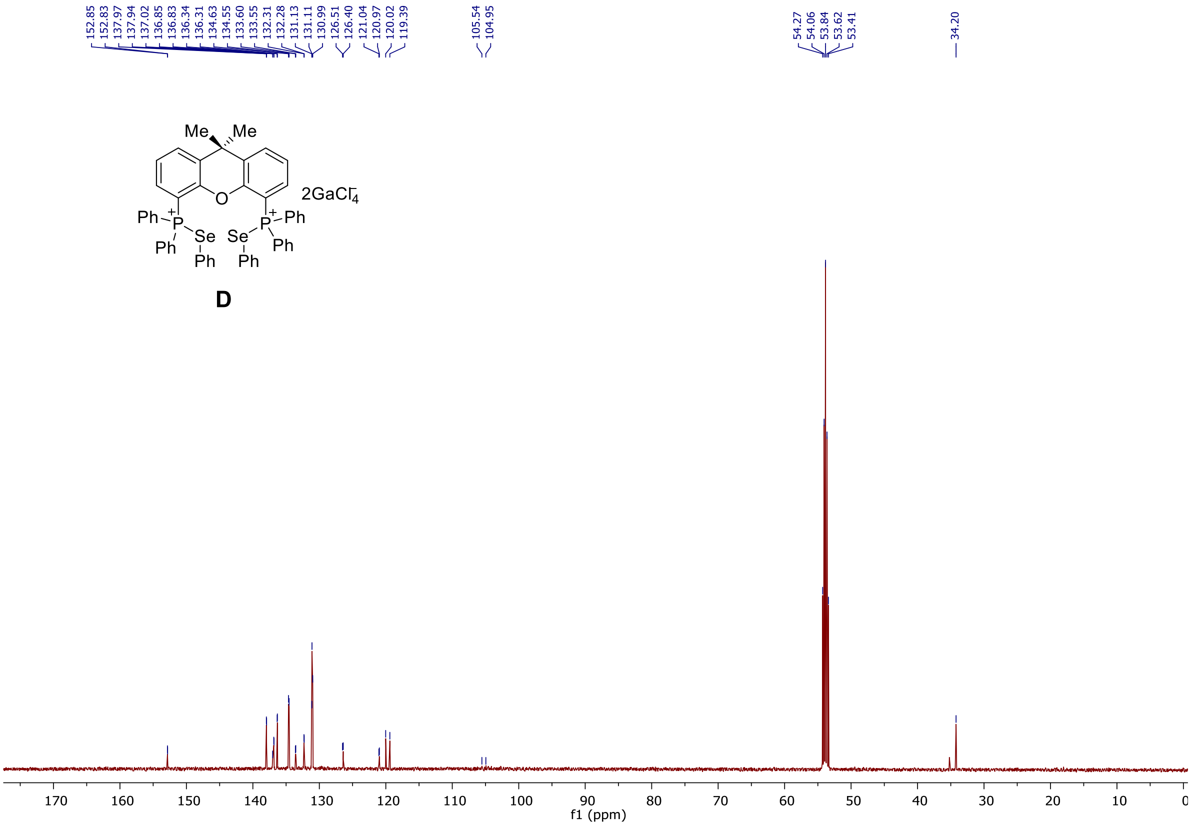

Supplementary Figure 60: ^{13}C spectra for **D**

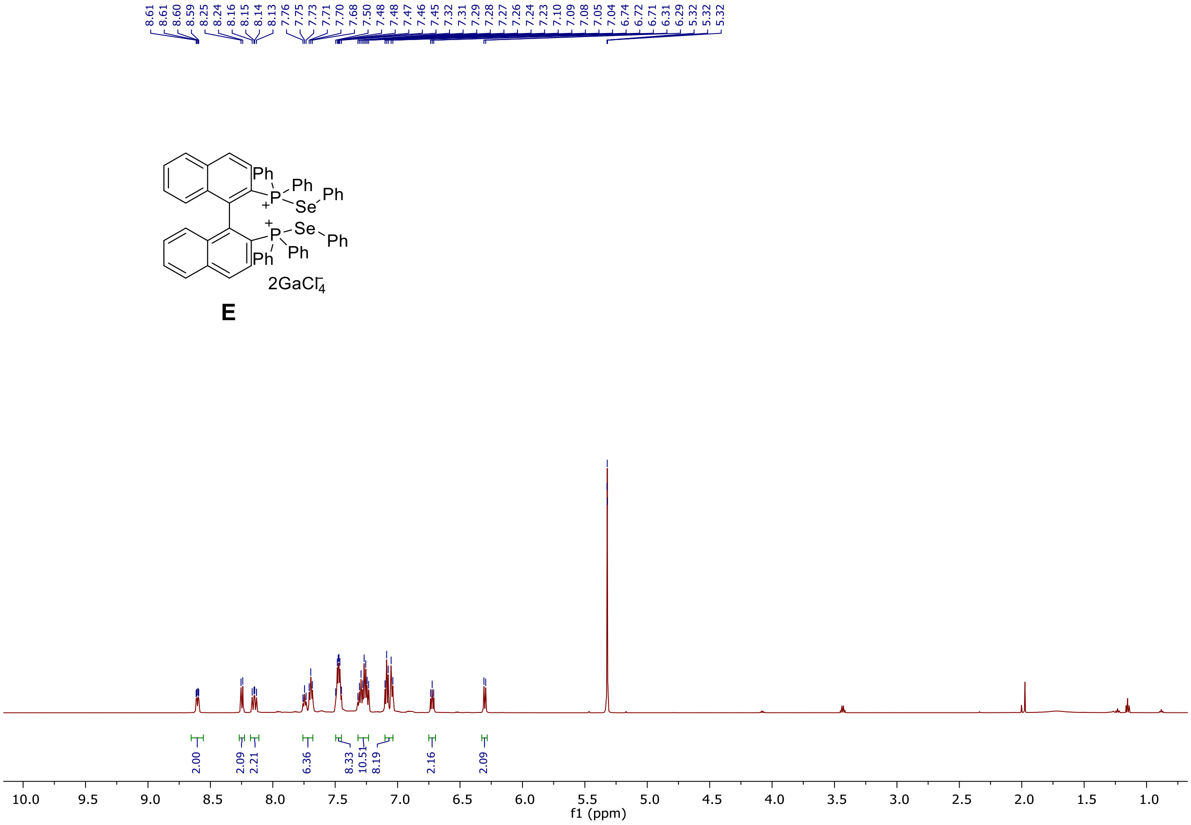

Supplementary Figure 61: ^{1}H spectra for **E**

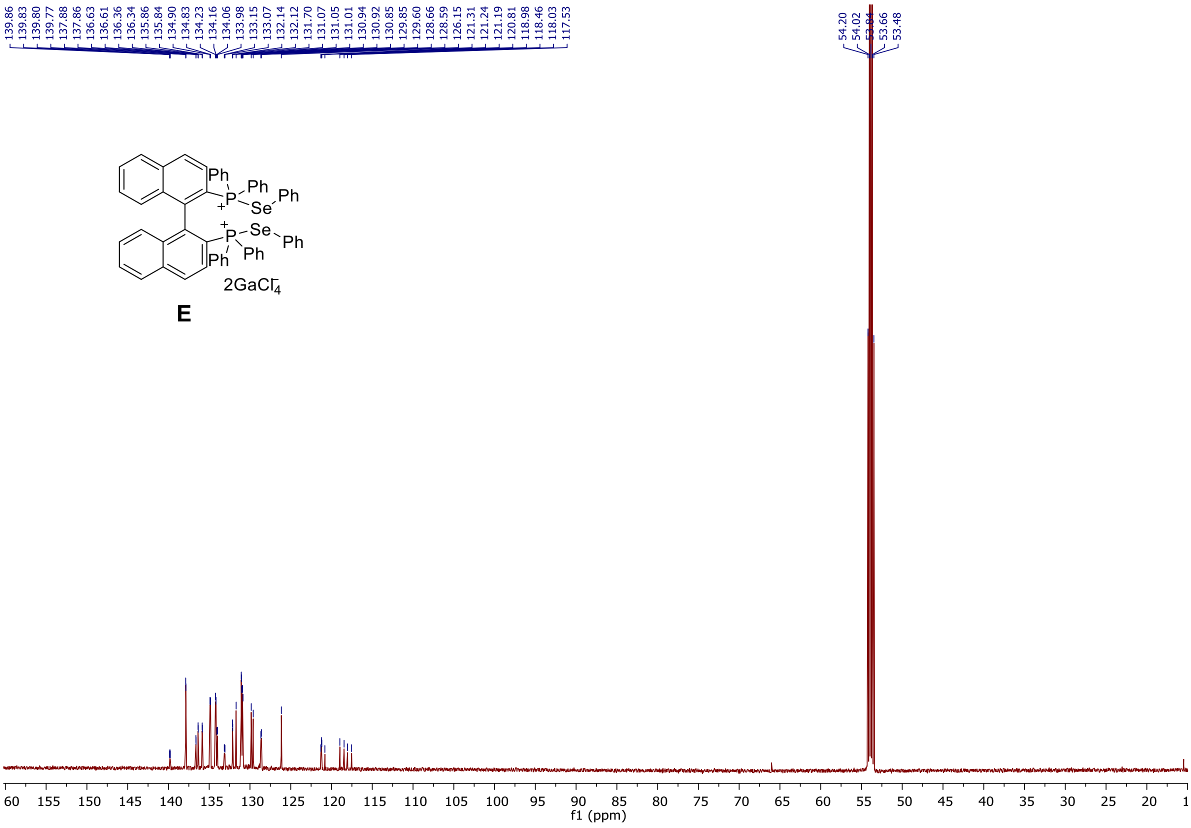

Supplementary Figure 62: ^{13}C spectra for **E**

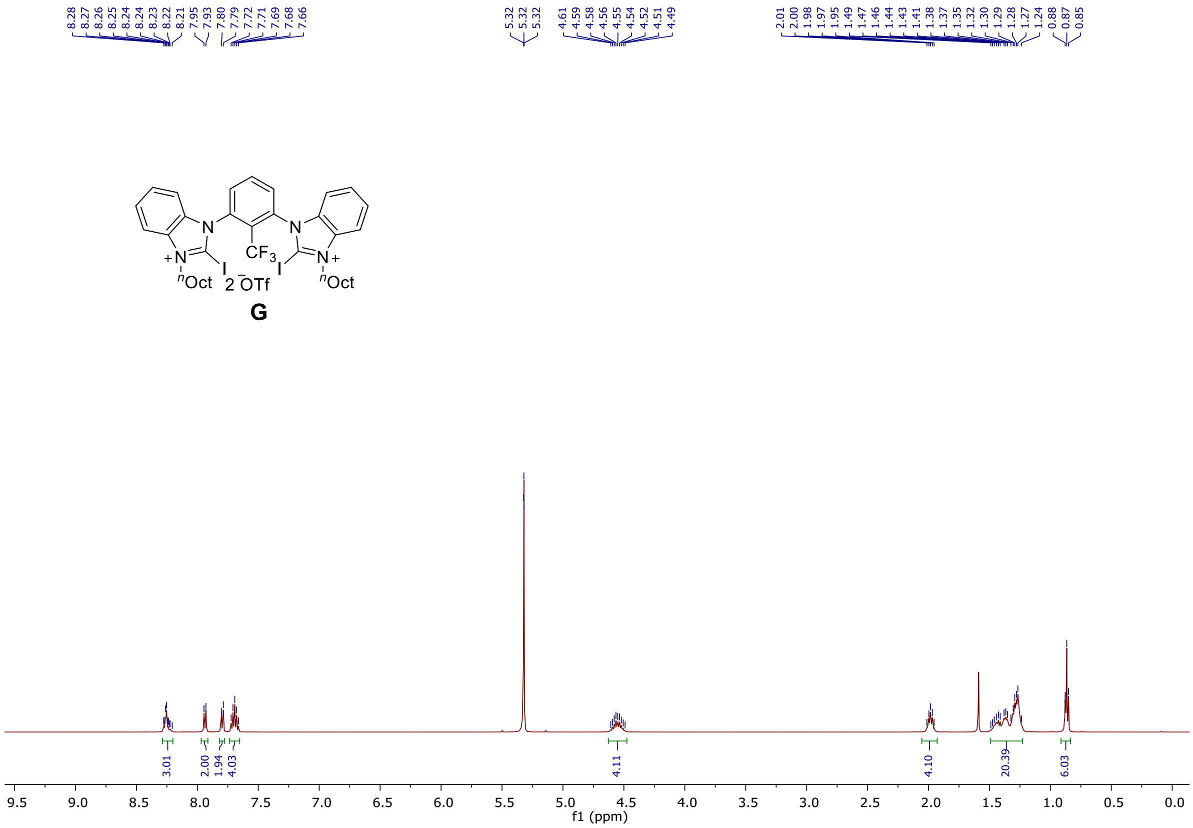

Supplementary Figure 63: ^{1}H spectra for **G**

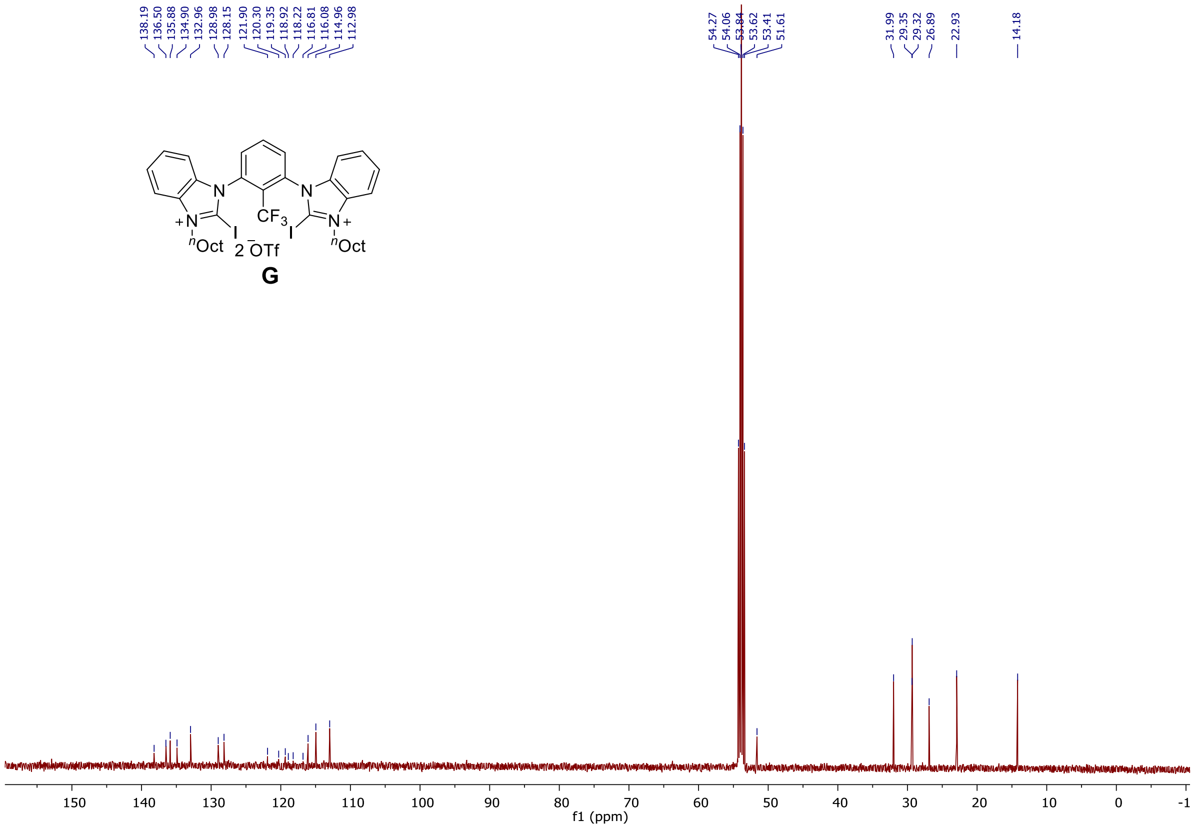

Supplementary Figure 64: ^{13}C spectra for **G**

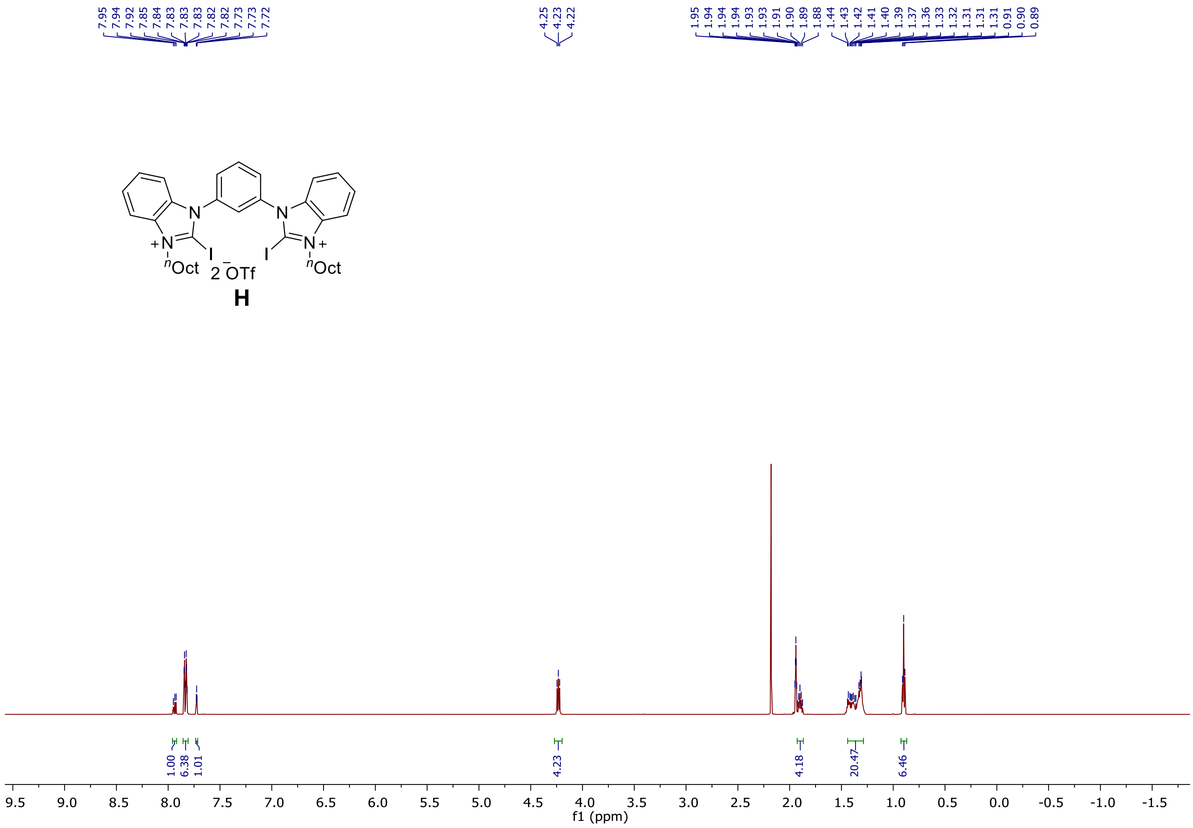

Supplementary Figure 65: ^{1}H spectra for **H**

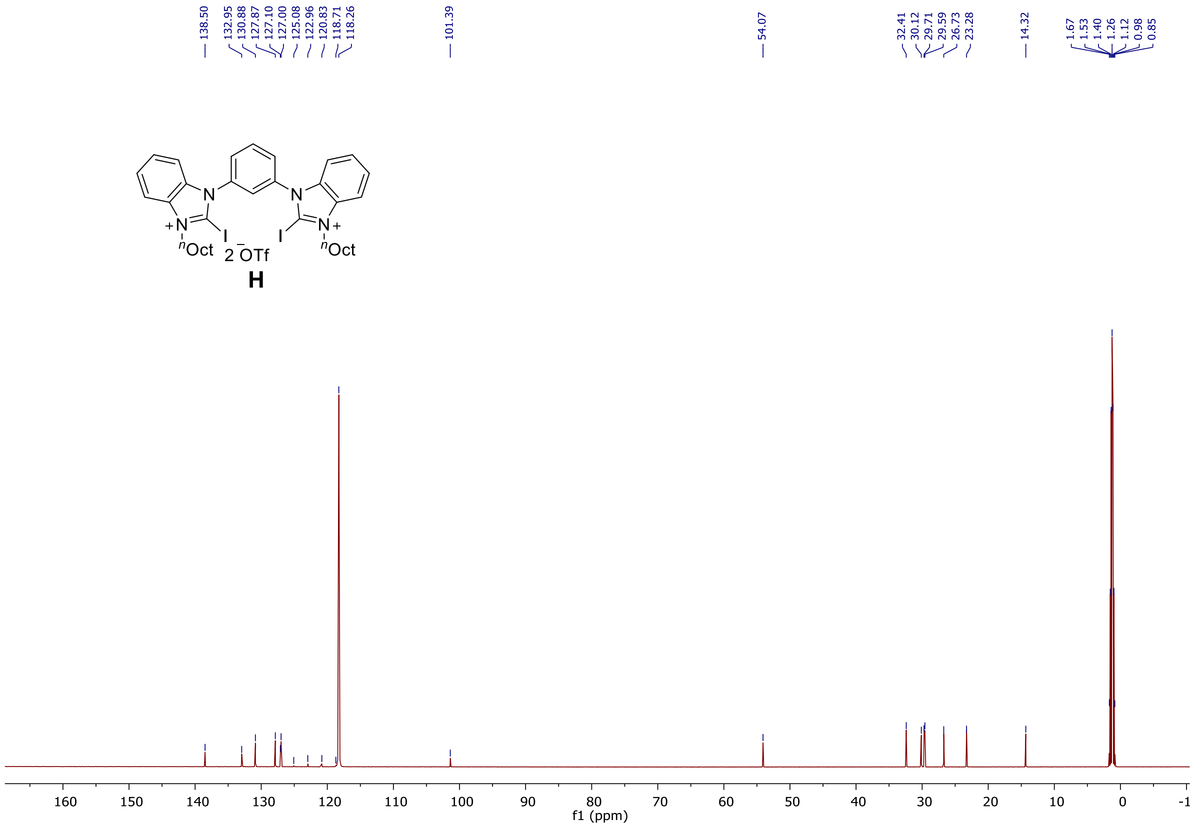

Supplementary Figure 66: ^{13}C spectra for **H**

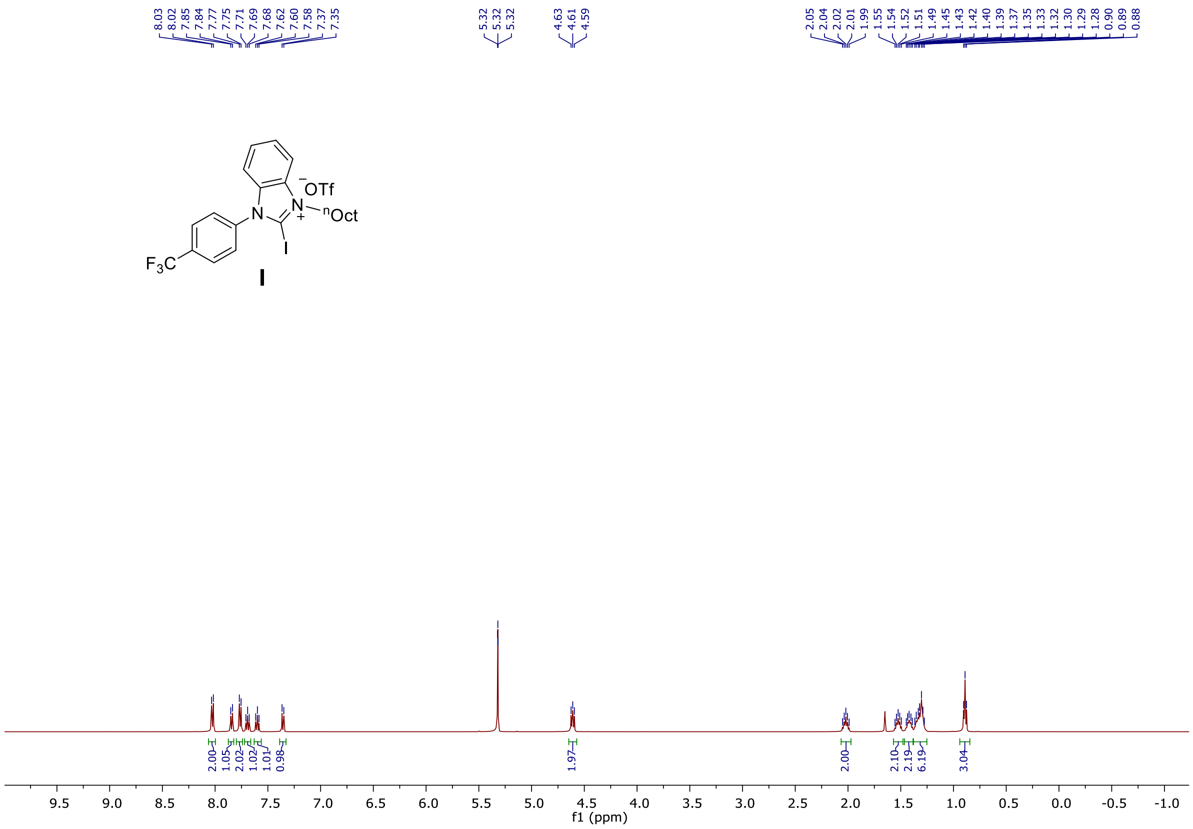

Supplementary Figure 67: ^{1}H spectra for **I**

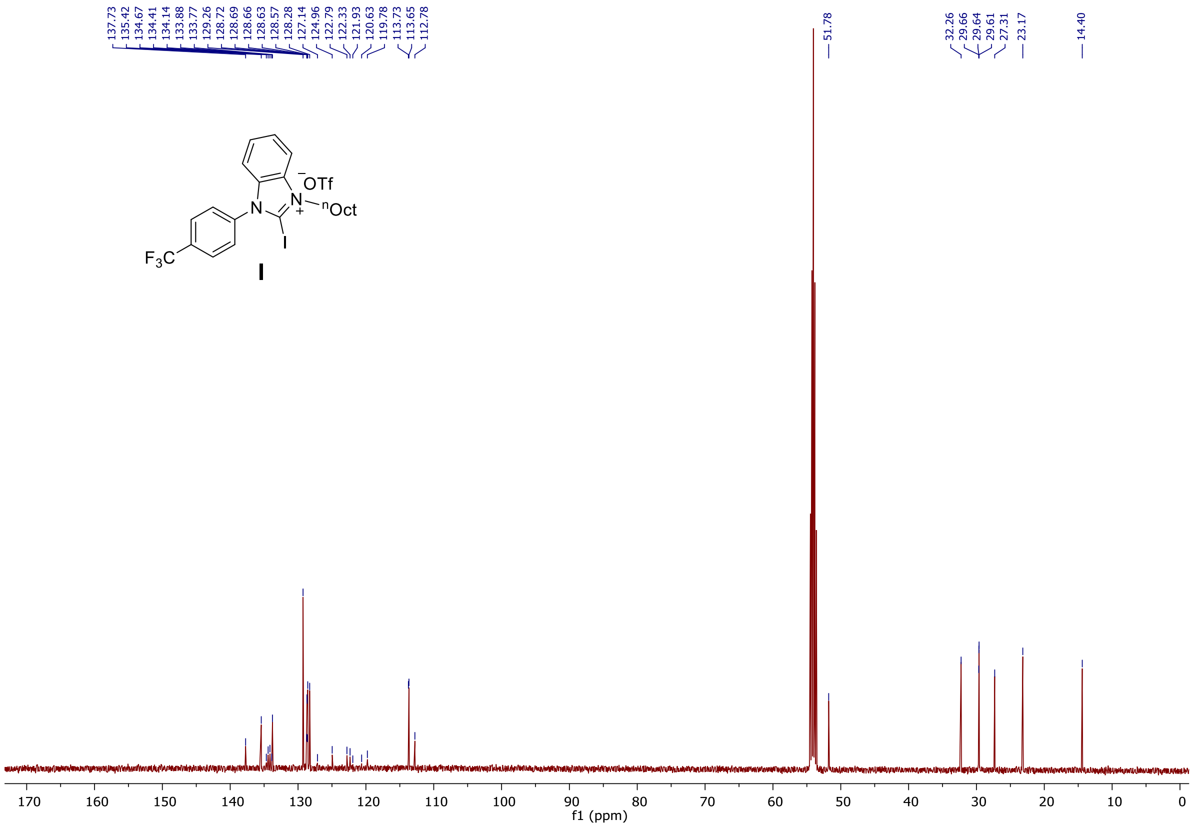

Supplementary Figure 68: ^{13}C spectra for **I**

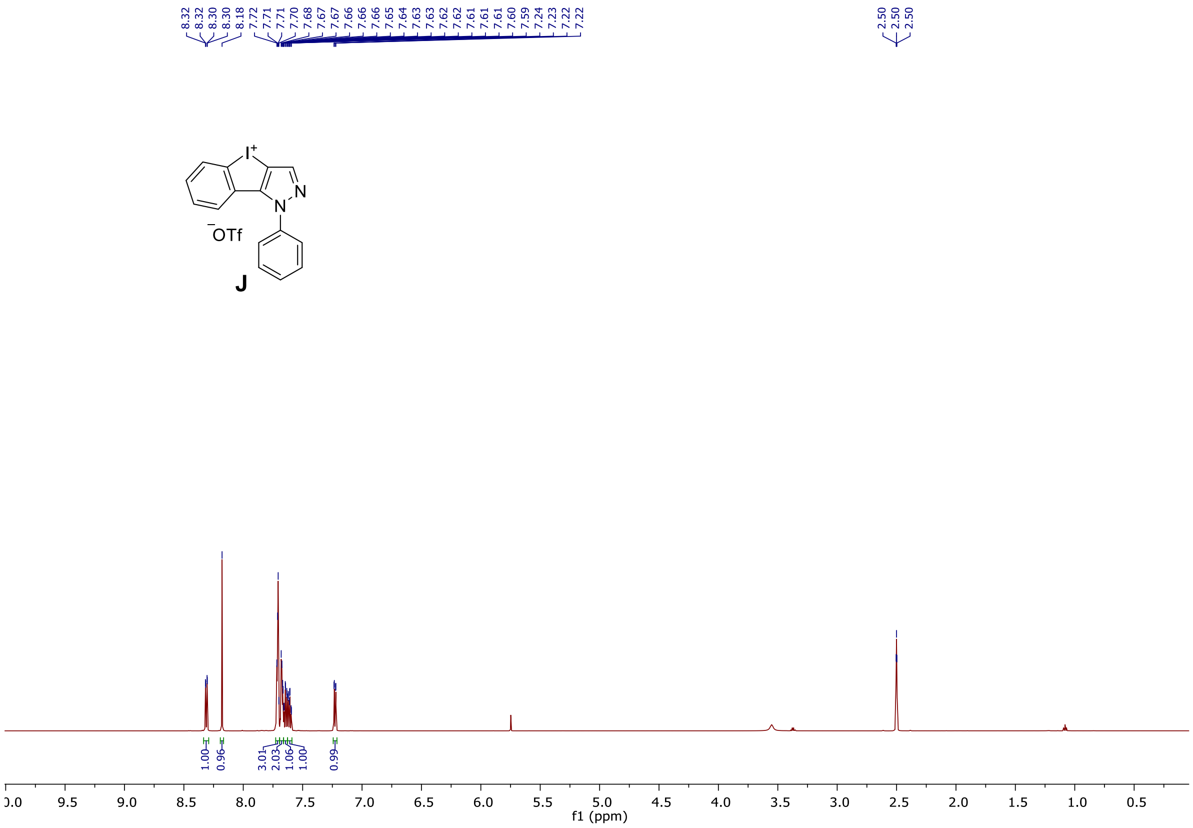

Supplementary Figure 69: ^{1}H spectra for **J**

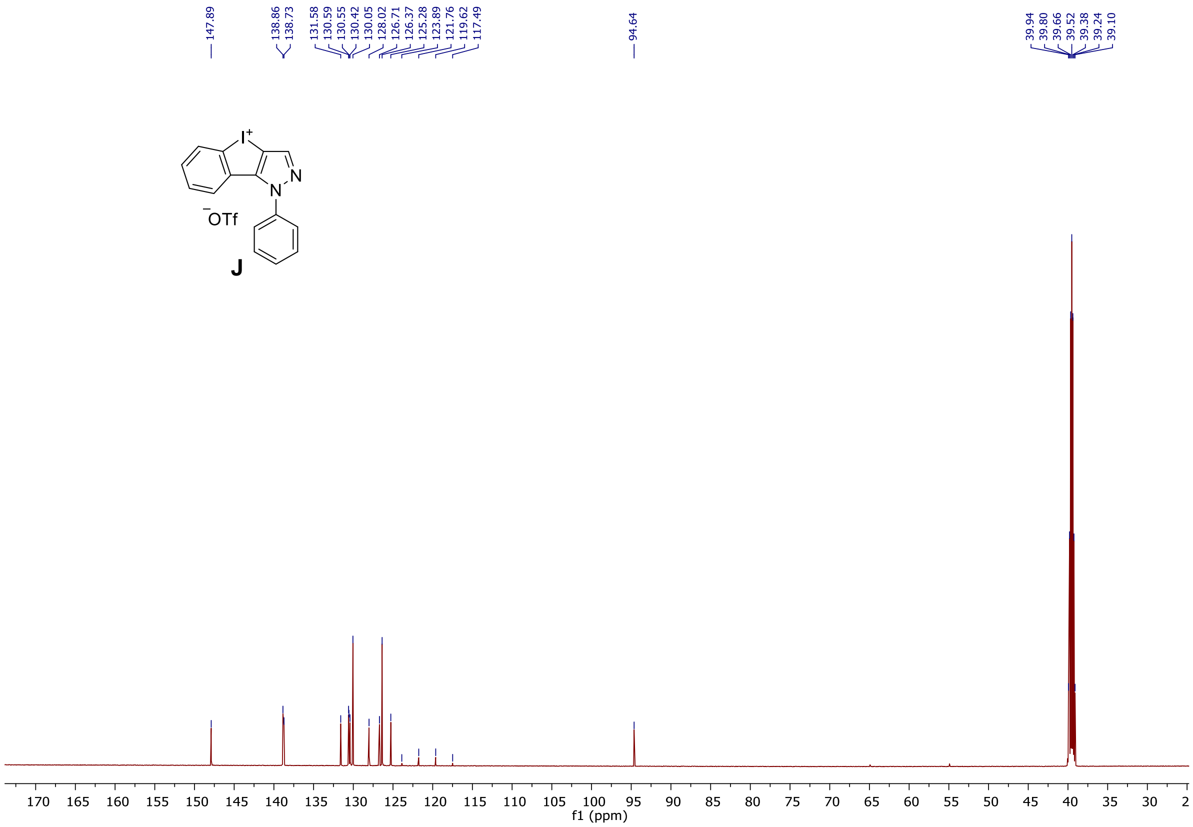

Supplementary Figure 70: ^{13}C spectra for **J**

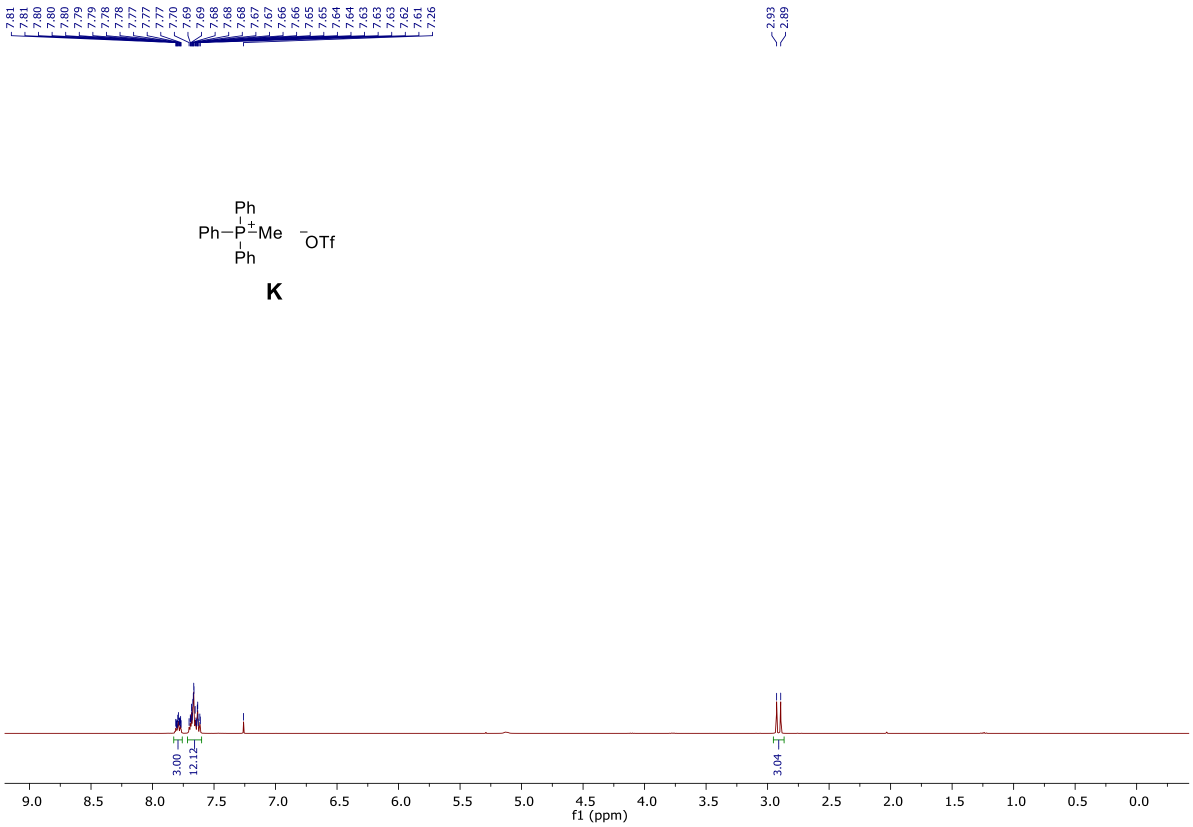

Supplementary Figure 71: ^{1}H spectra for **K**

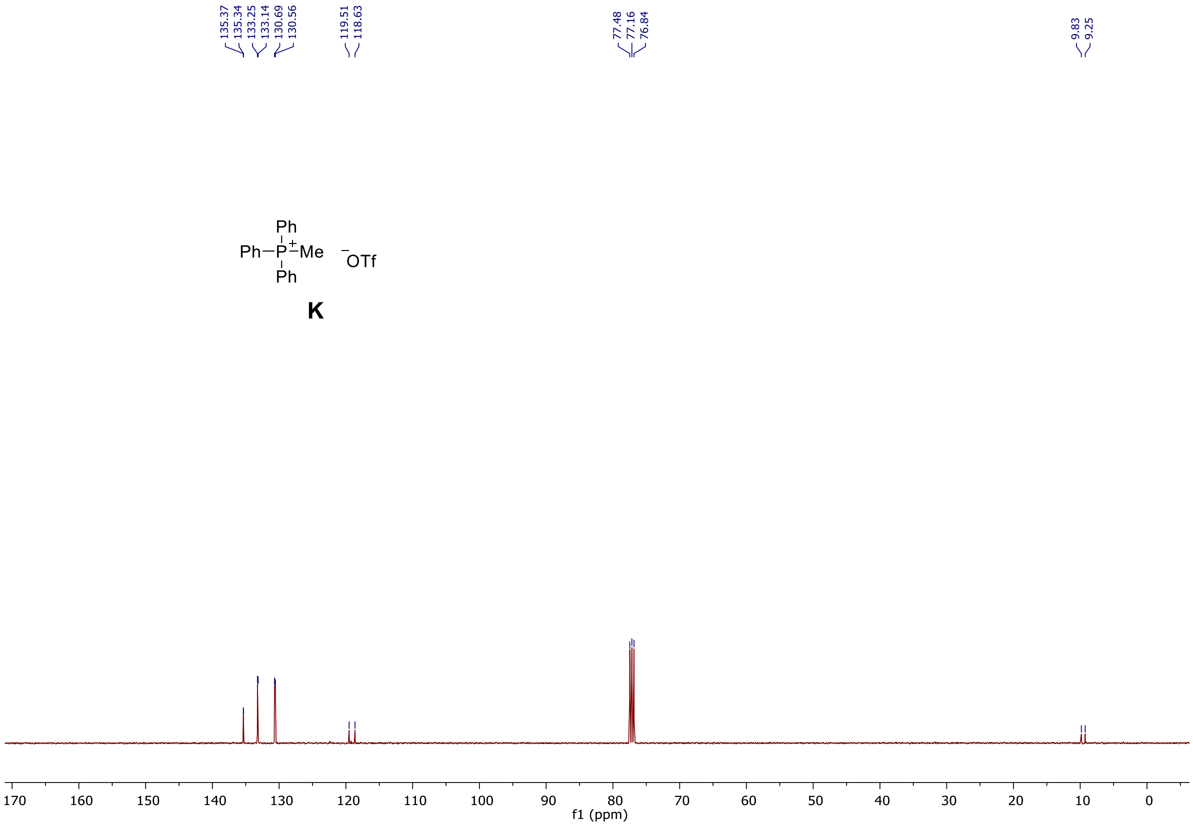

Supplementary Figure 72: ^{13}C spectra for **K**

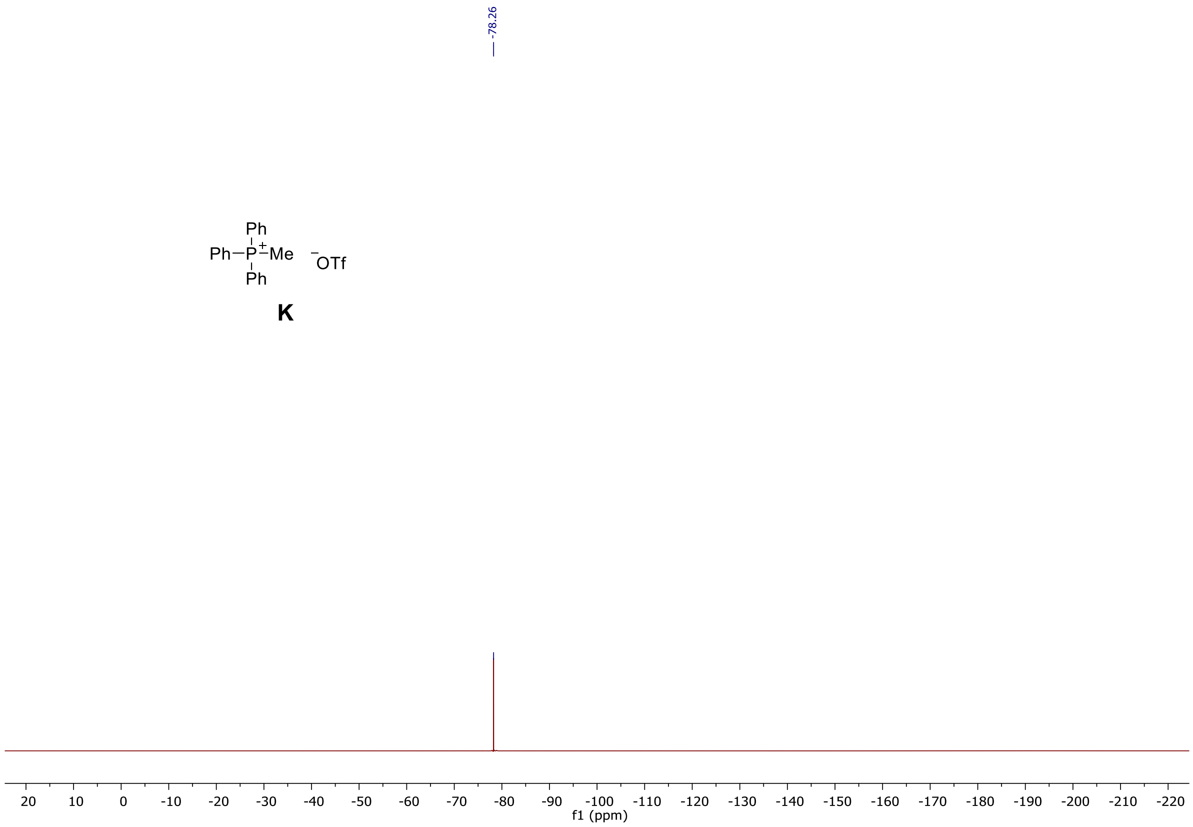

Supplementary Figure 73: ^{19}F spectra for **K**

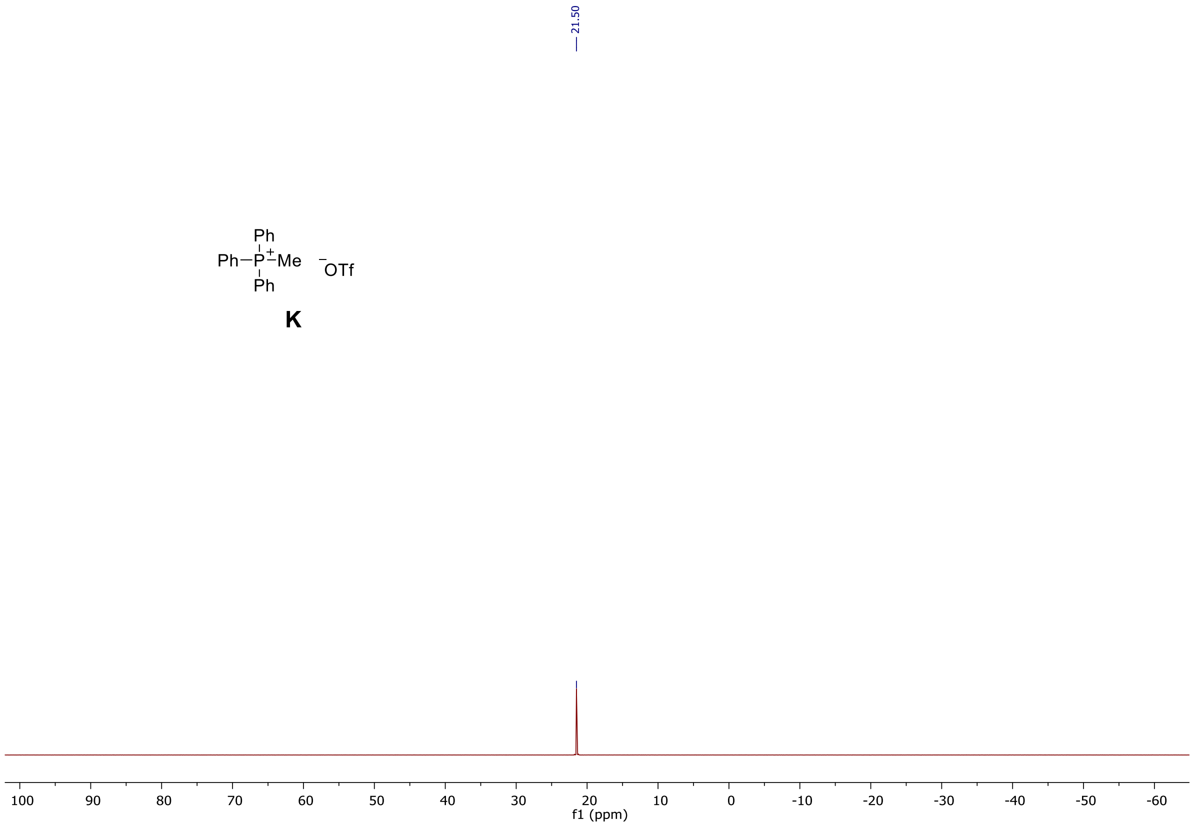

Supplementary Figure 74: ^{31}P spectra for **K**

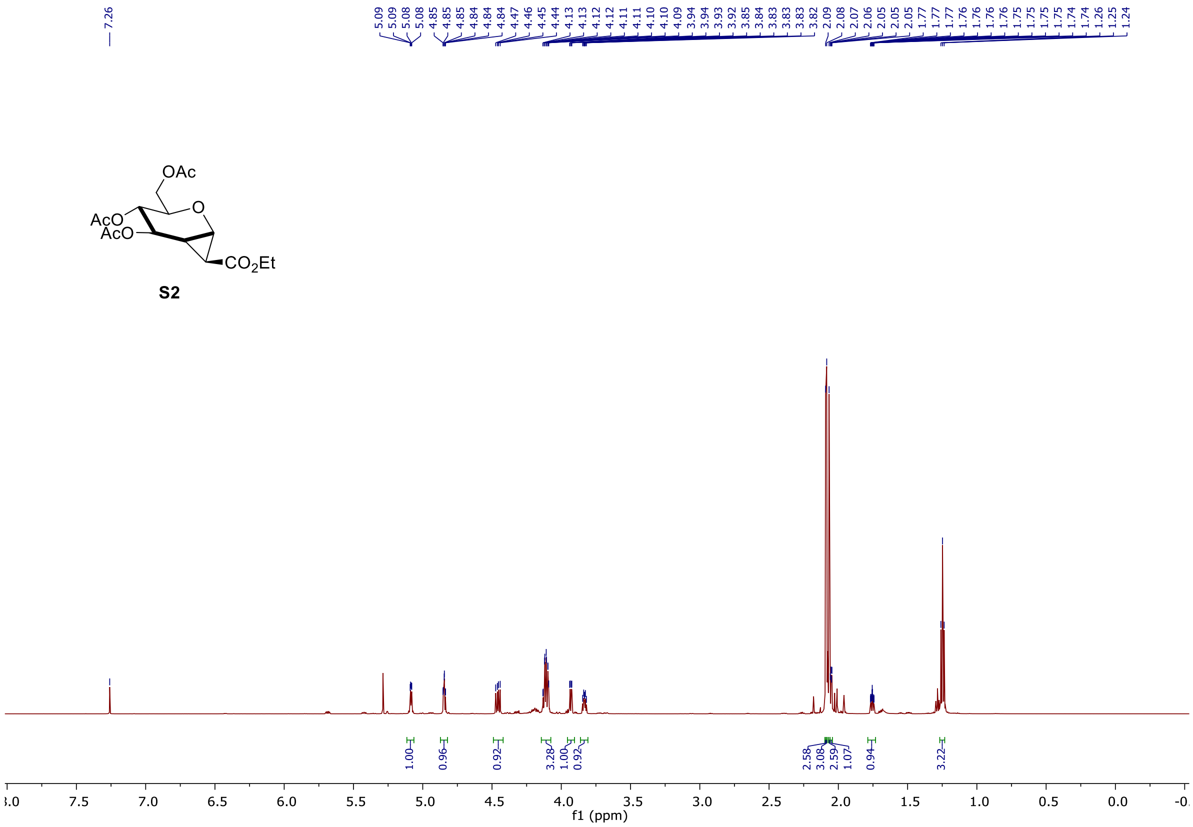

Supplementary Figure 75: ^{1}H spectra for **S2**

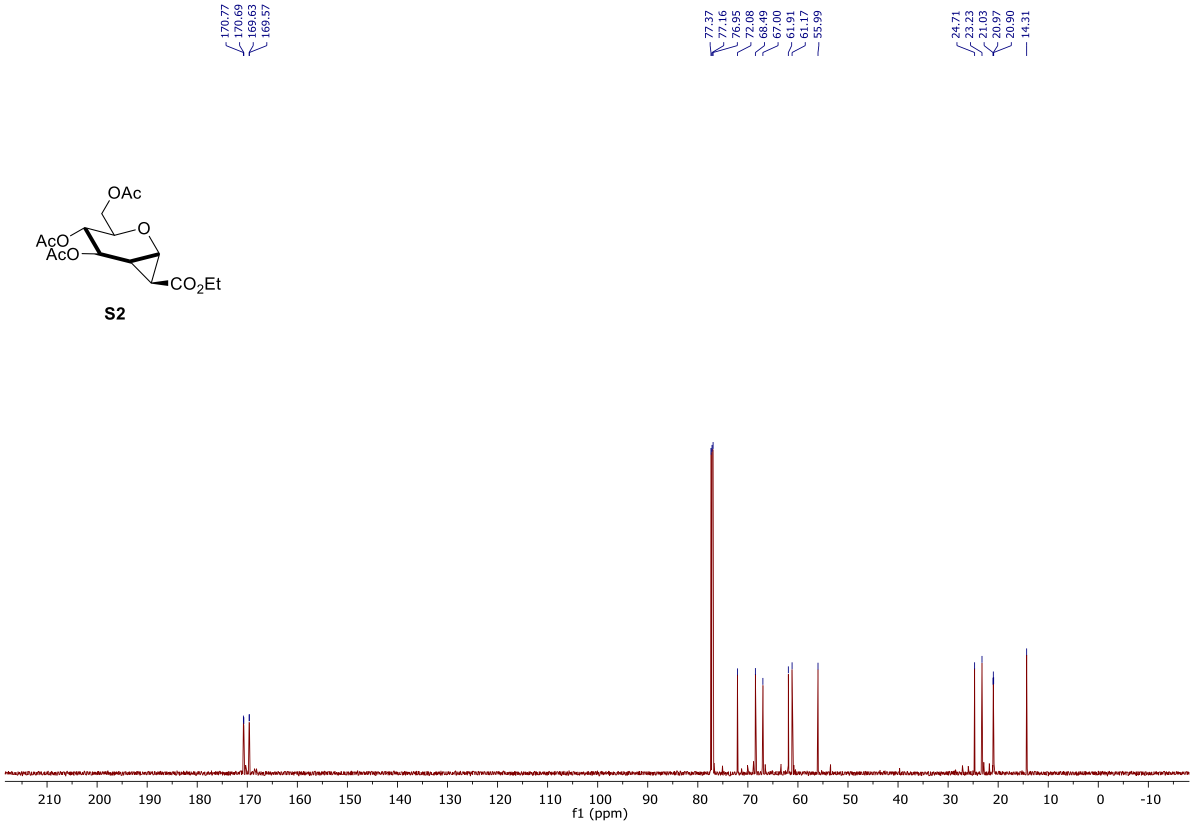

Supplementary Figure 76: ^{13}C spectra for **S2**

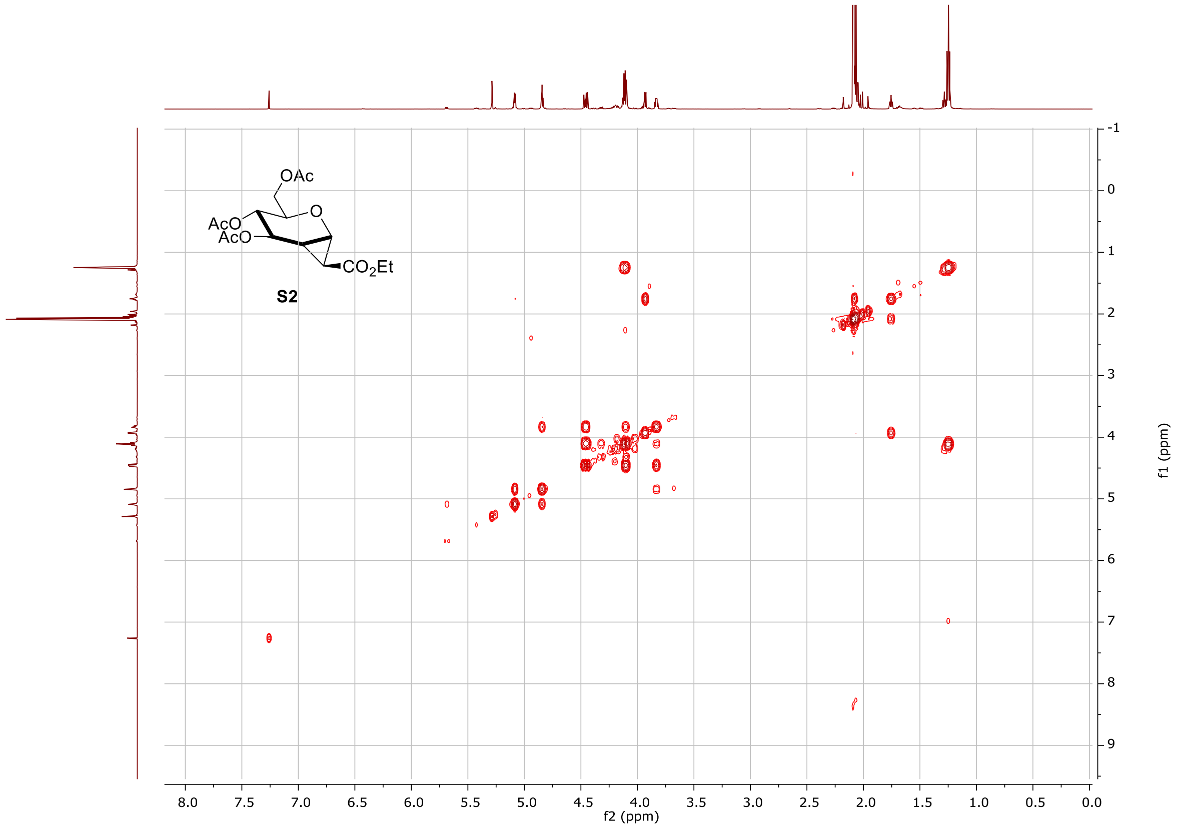

Supplementary Figure 77: COSY spectra for compound **S2**

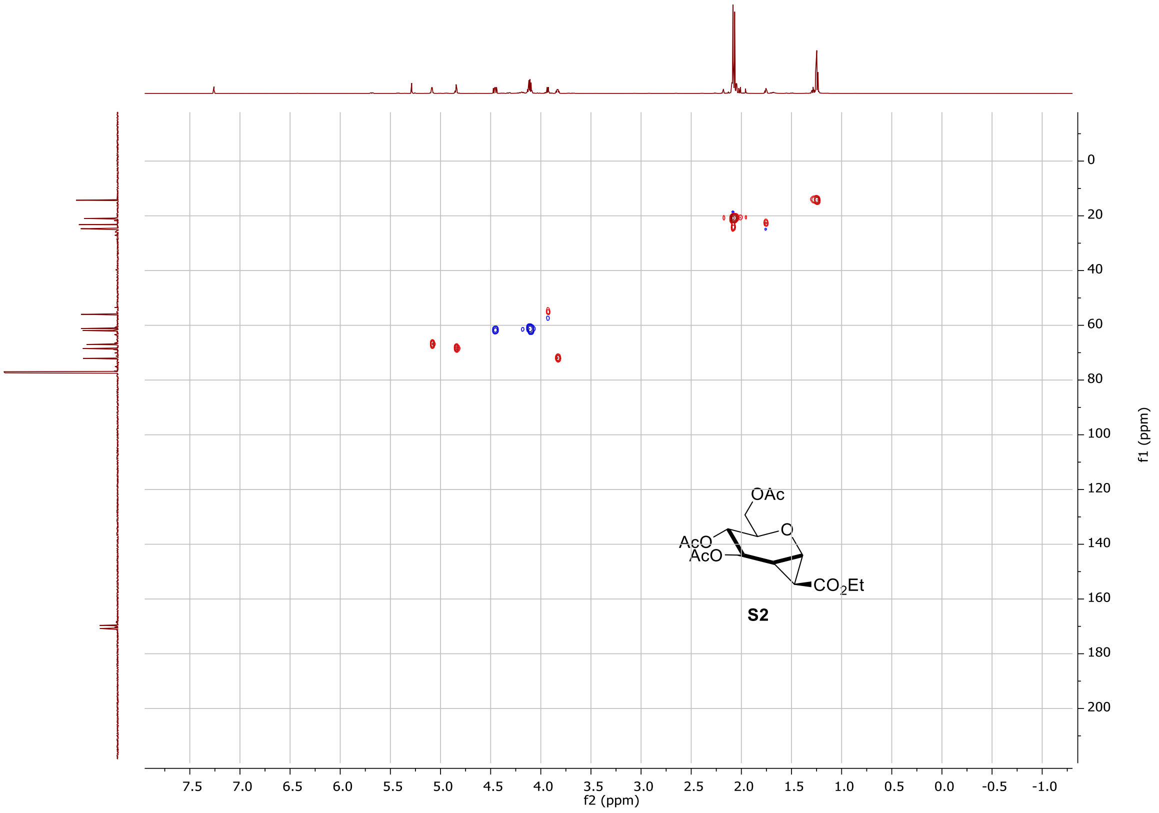

Supplementary Figure 78: HSQC spectra for compound **S2**

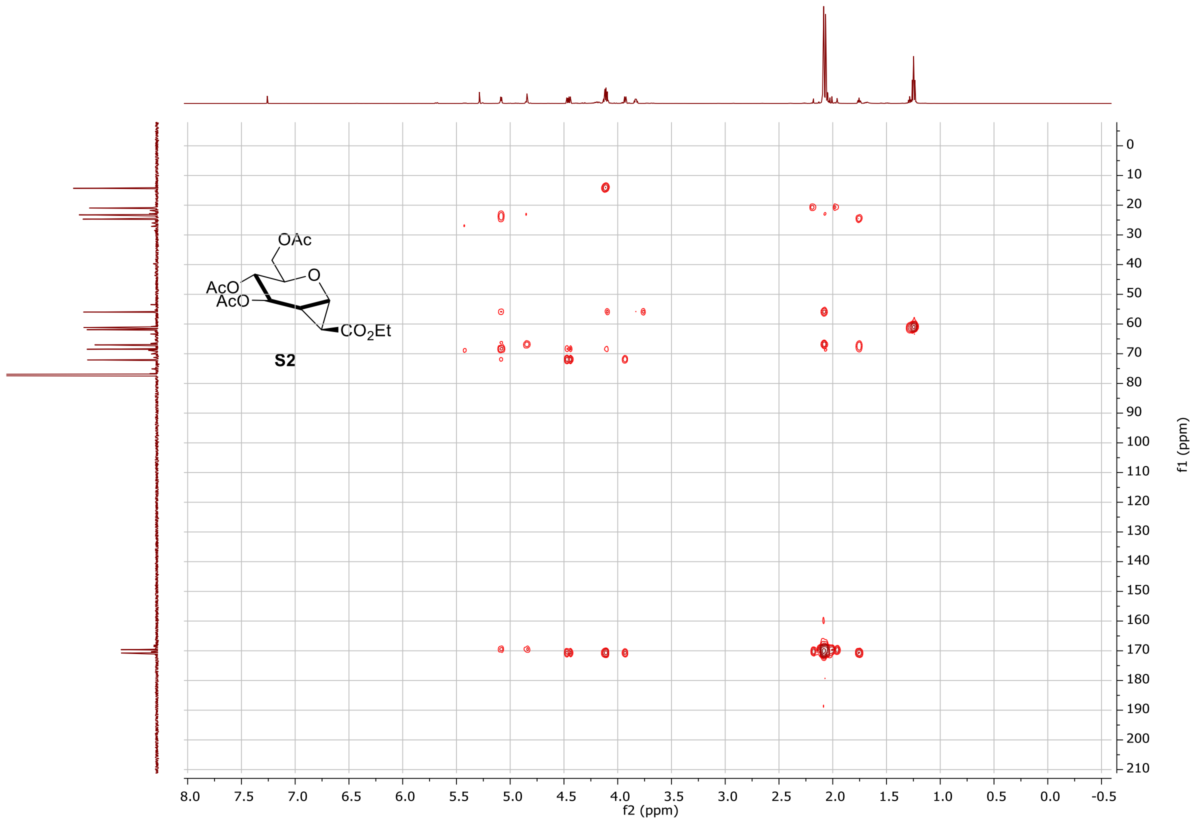

Supplementary Figure 79: HMBC spectra for compound **S2**

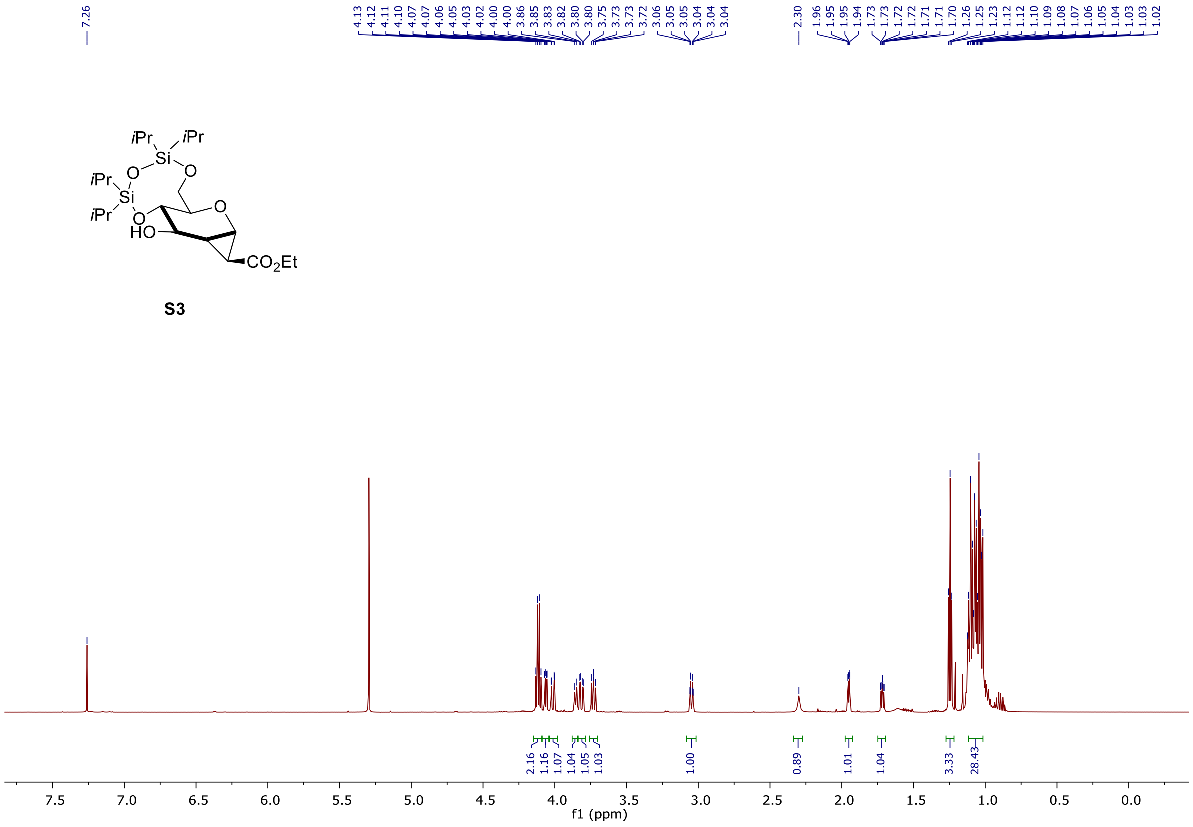

Supplementary Figure 80: ^{1}H spectra for **S3**

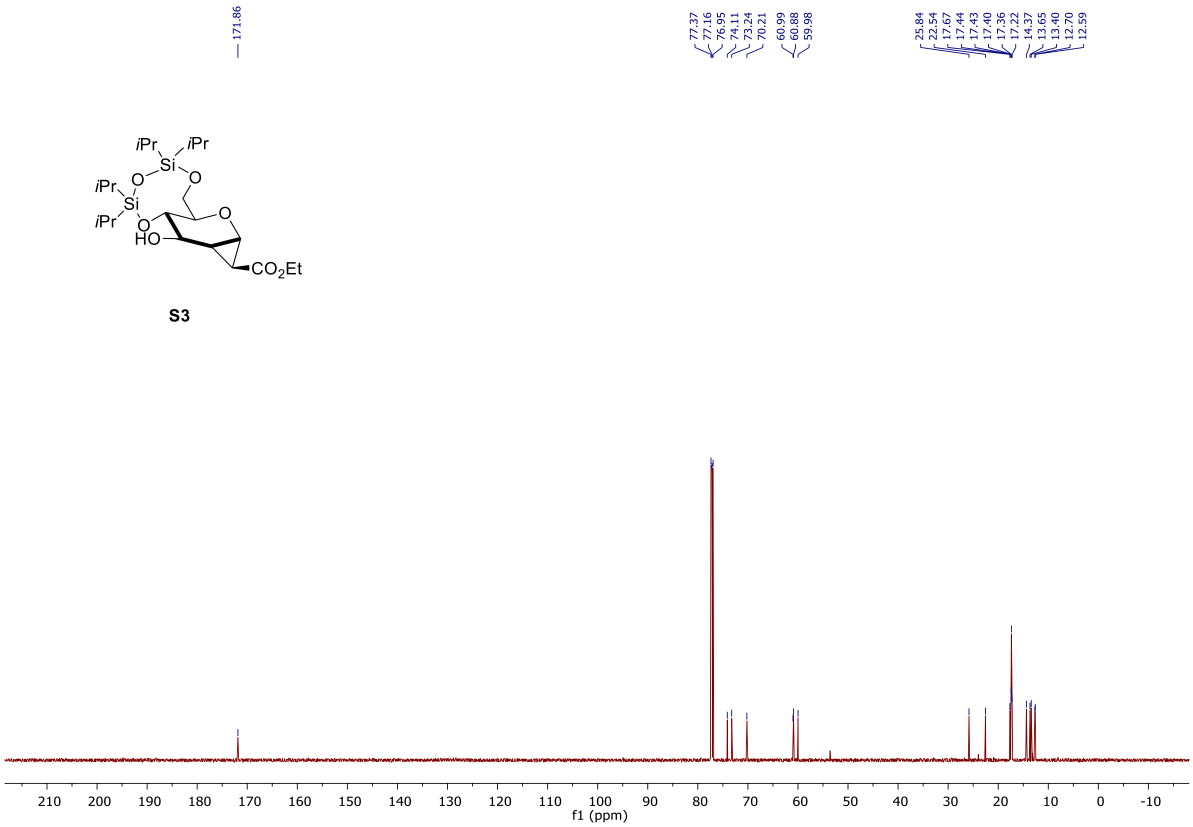

Supplementary Figure 81: ^{13}C spectra for **S3**

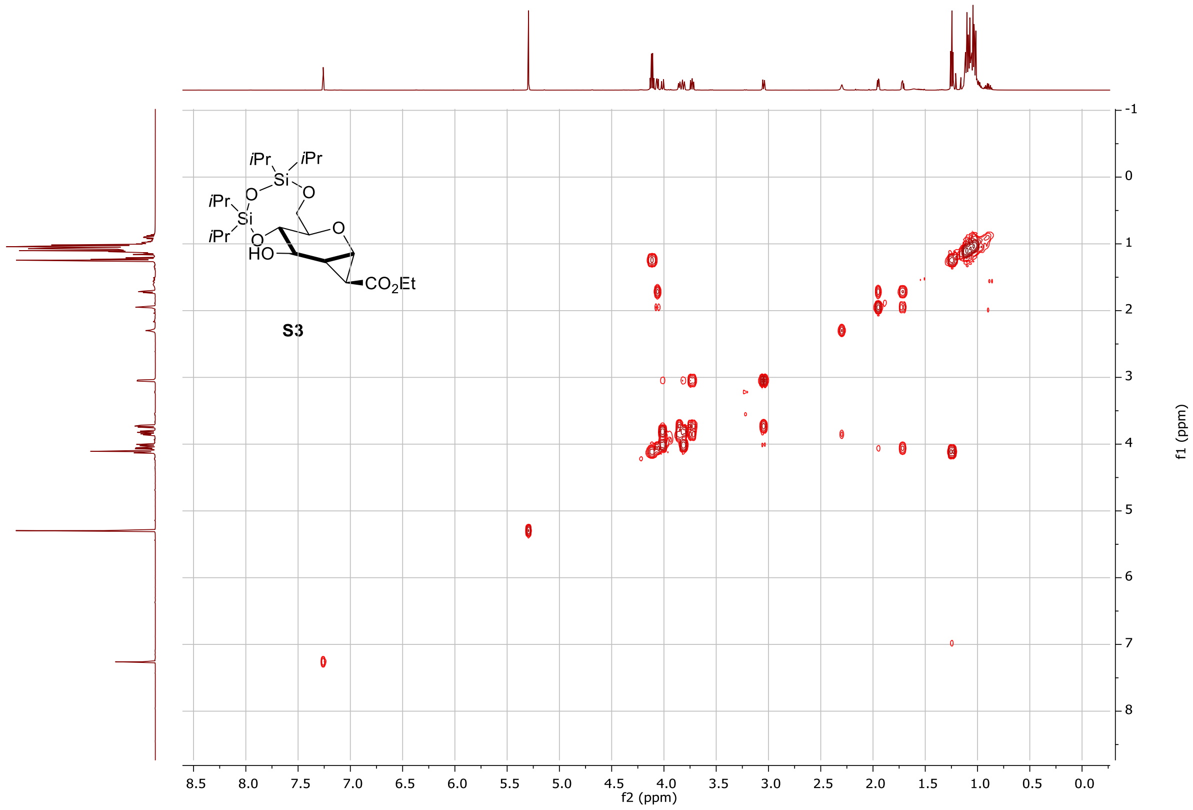

Supplementary Figure 82: COSY spectra for compound **S3**

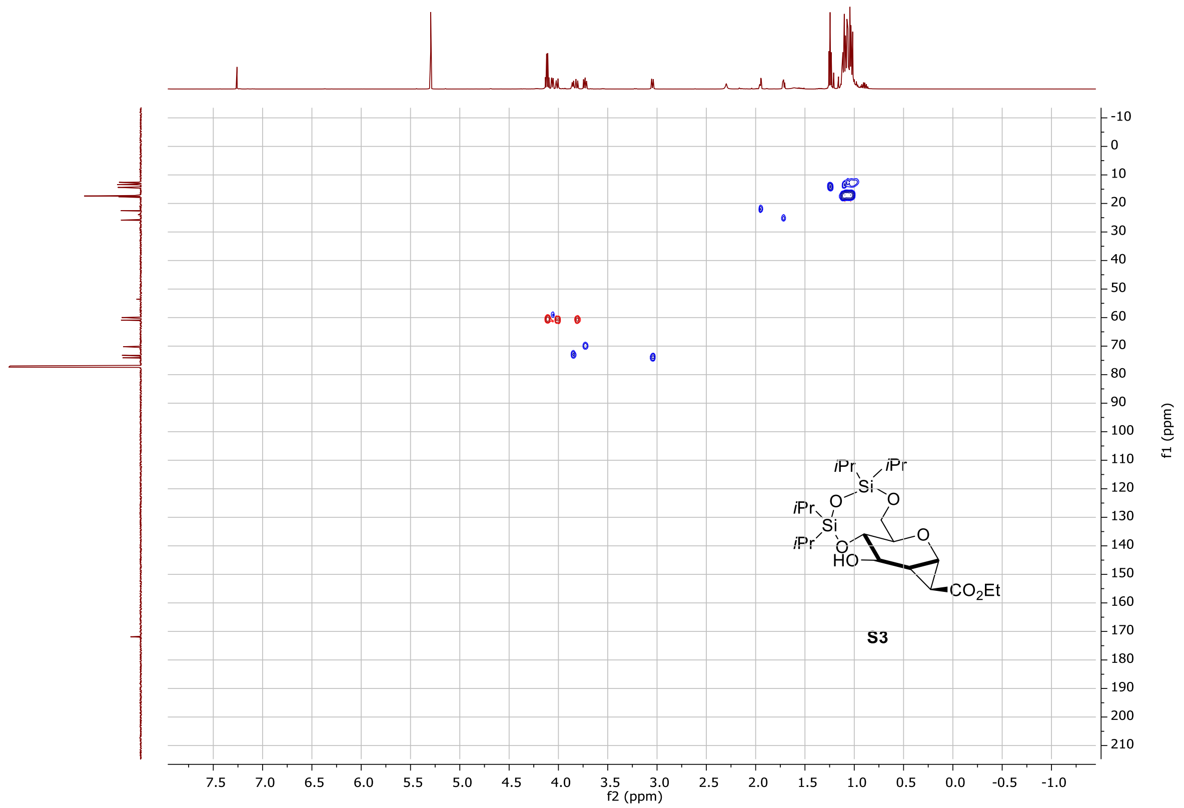

Supplementary Figure 83: HSQC spectra for compound **S3**

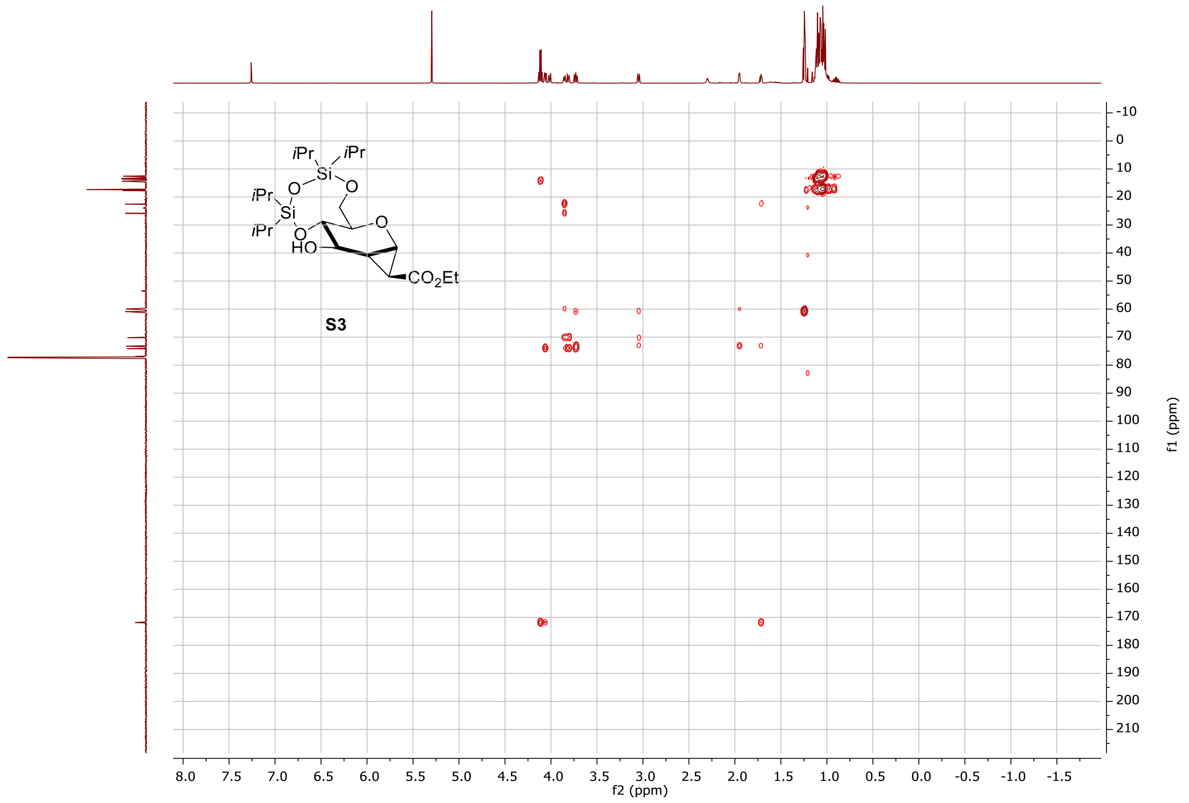

Supplementary Figure 84: HMBC spectra for compound **S3**

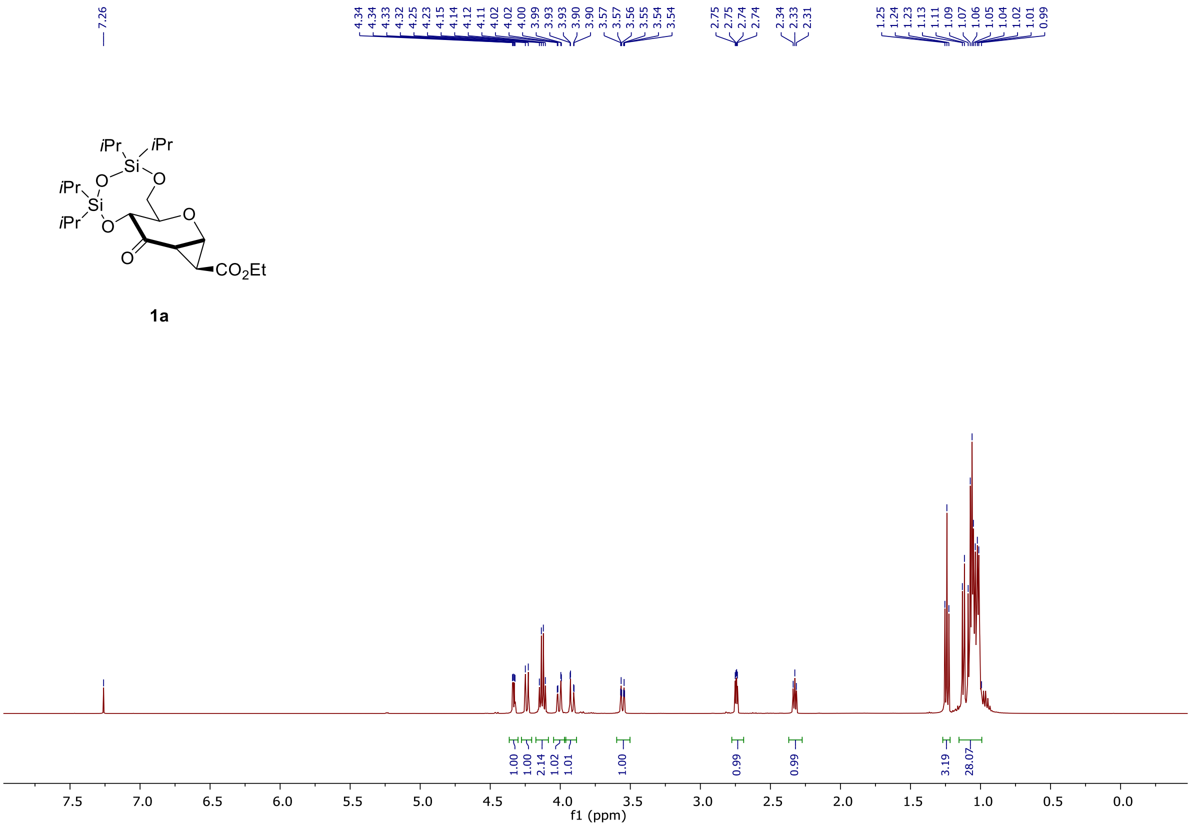

Supplementary Figure 85: ^{1}H spectra for **1a**

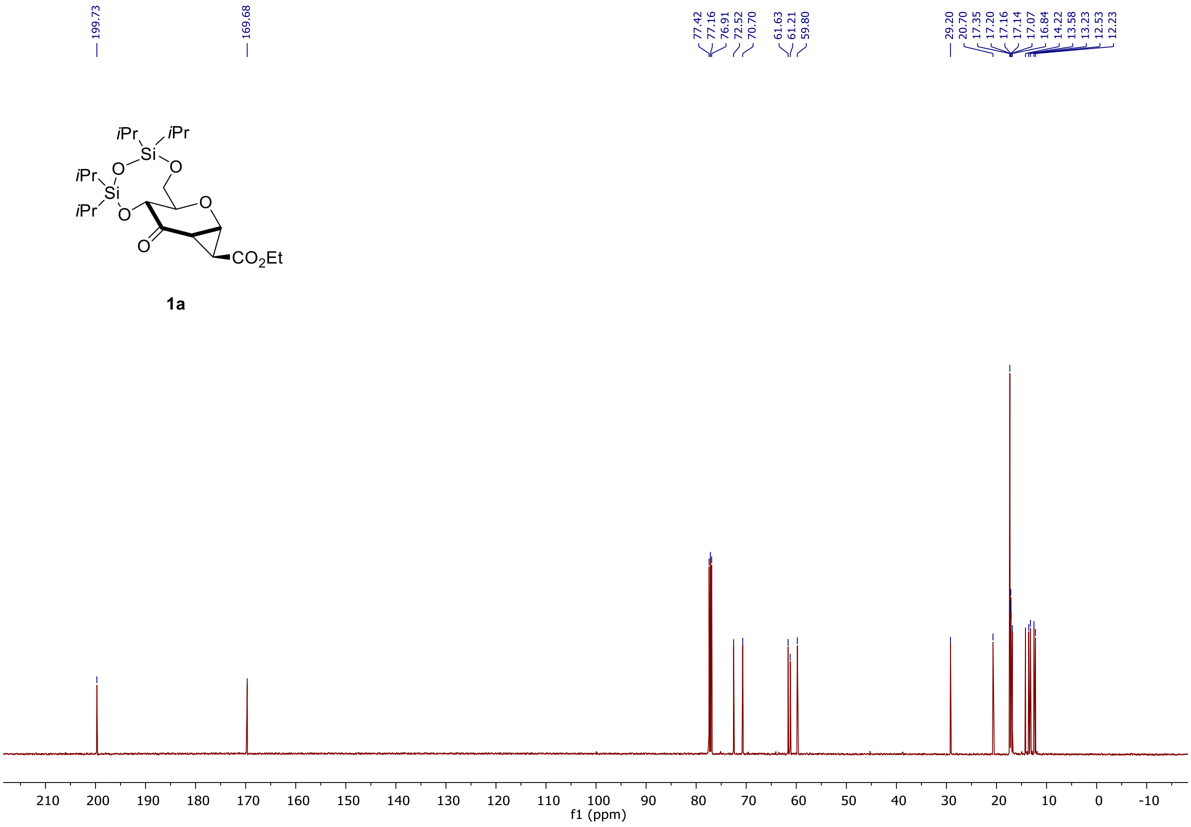

Supplementary Figure 86: ^{13}C spectra for **1a**

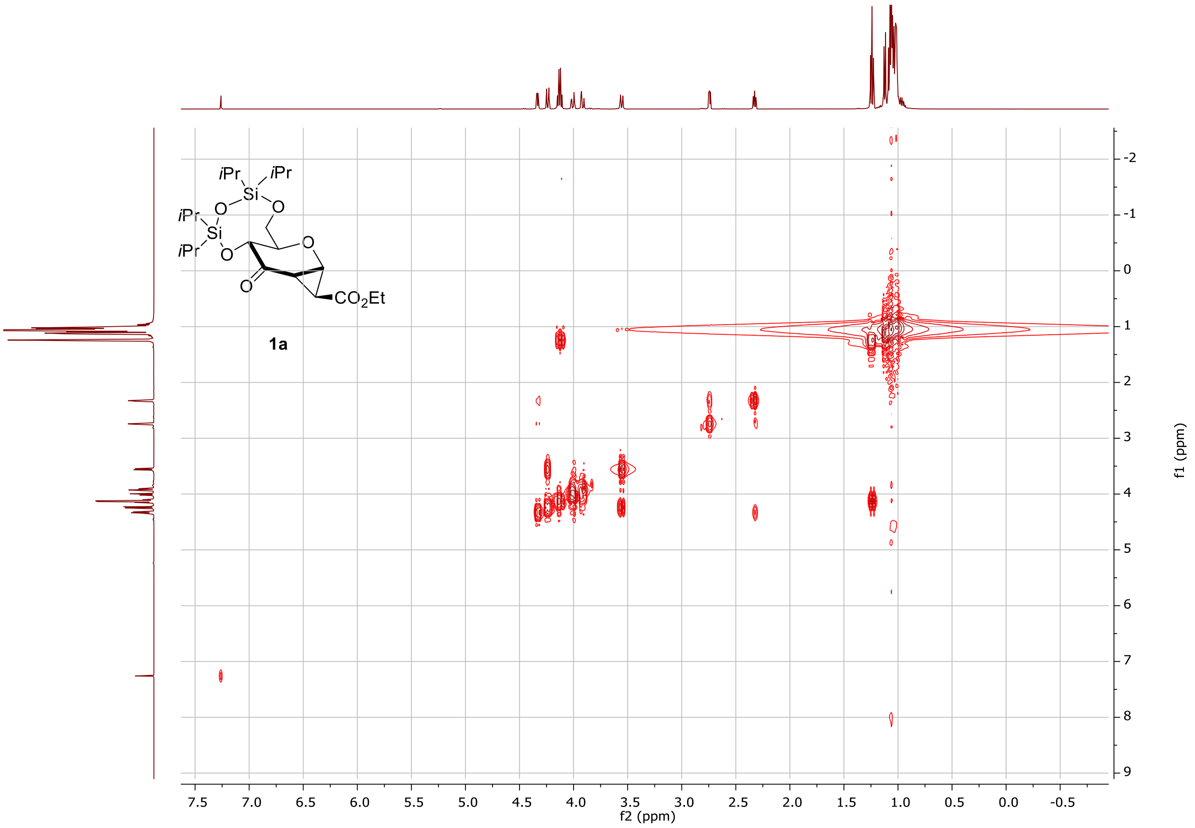

Supplementary Figure 87: COSY spectra for compound **1a**

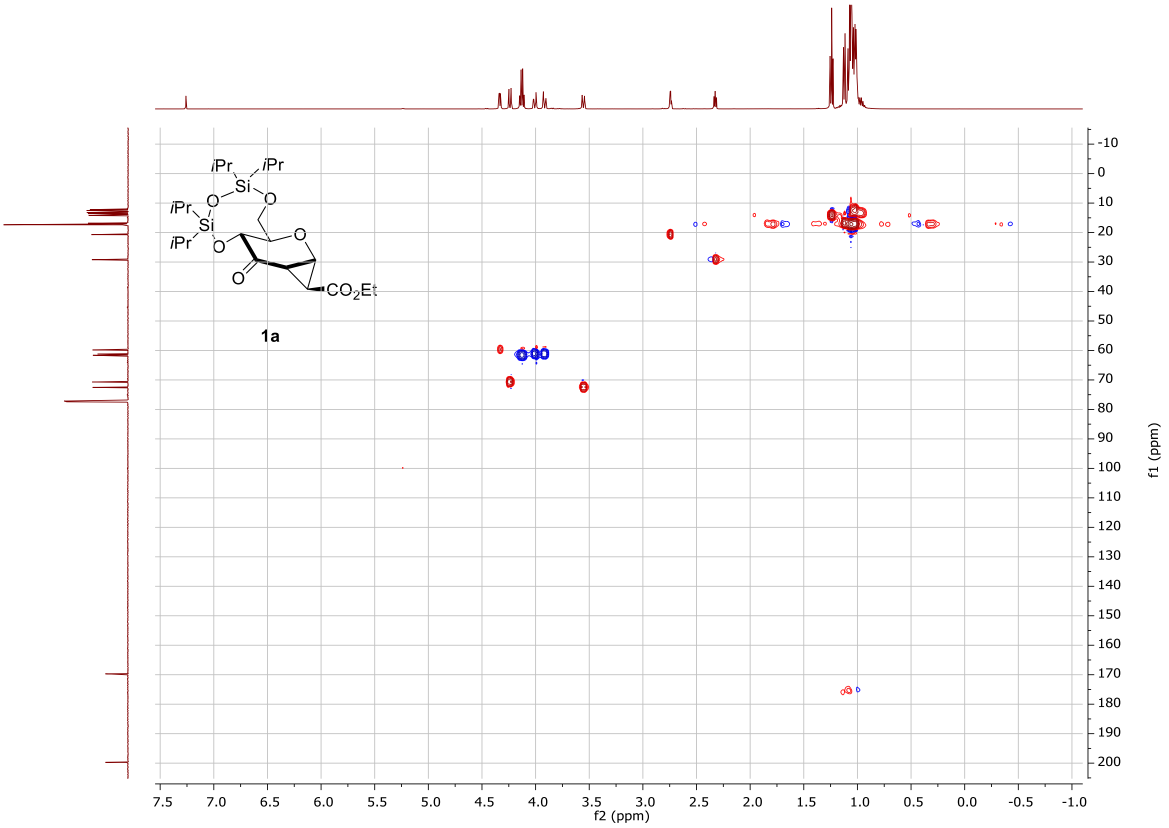

Supplementary Figure 88: HSQC spectra for compound **1a**

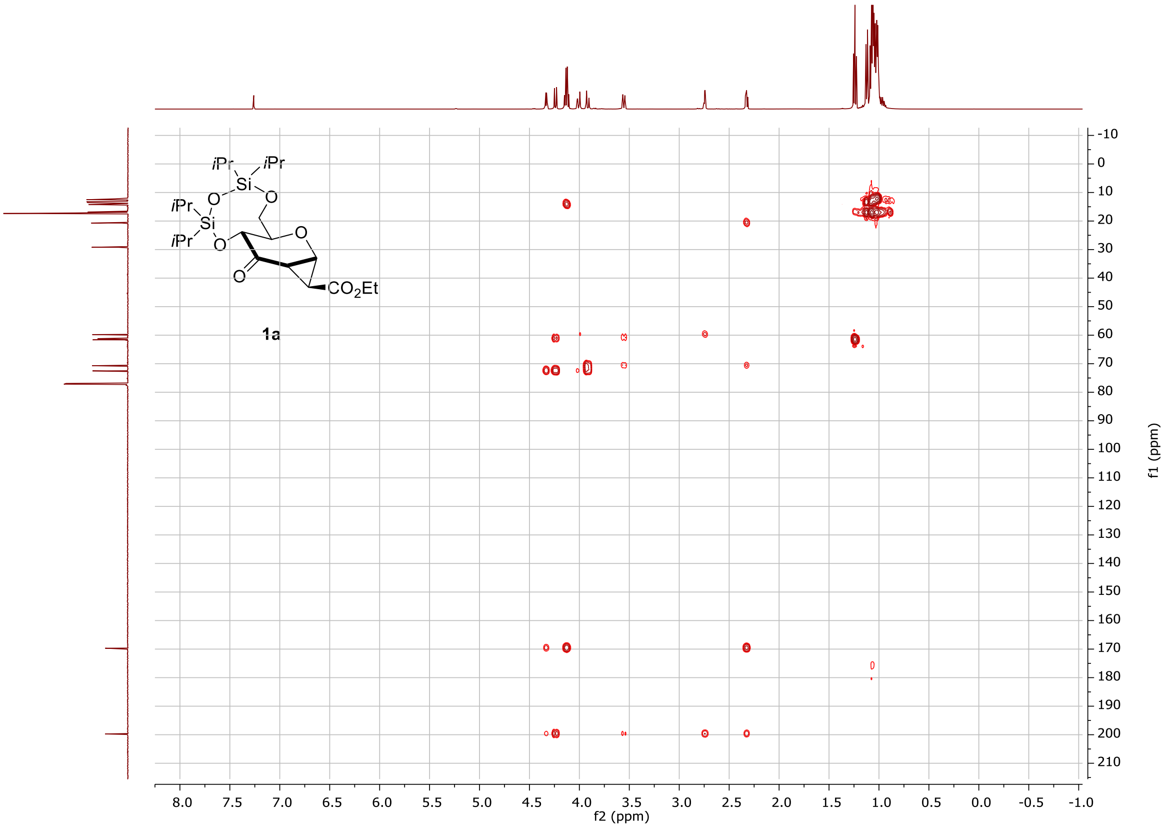

Supplementary Figure 89: HMBC spectra for compound **1a**

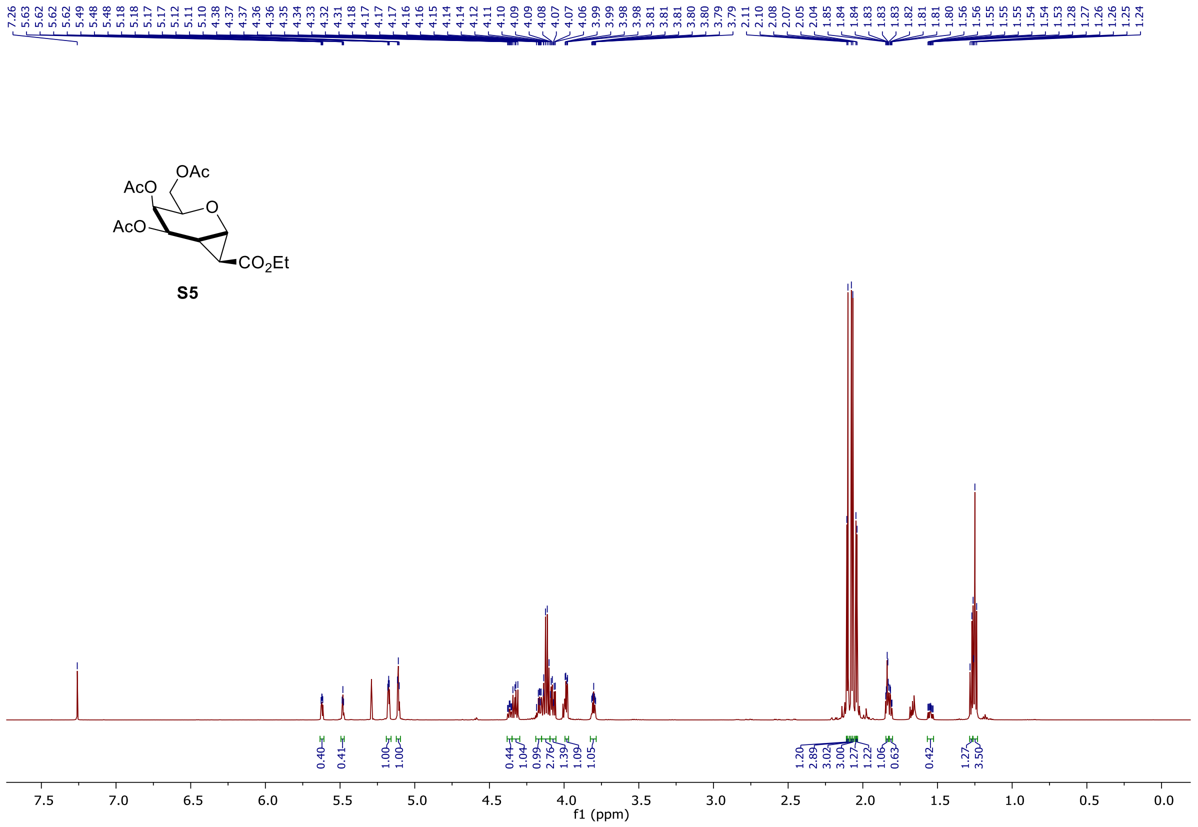

Supplementary Figure 90: ^{1}H spectra for **S5**

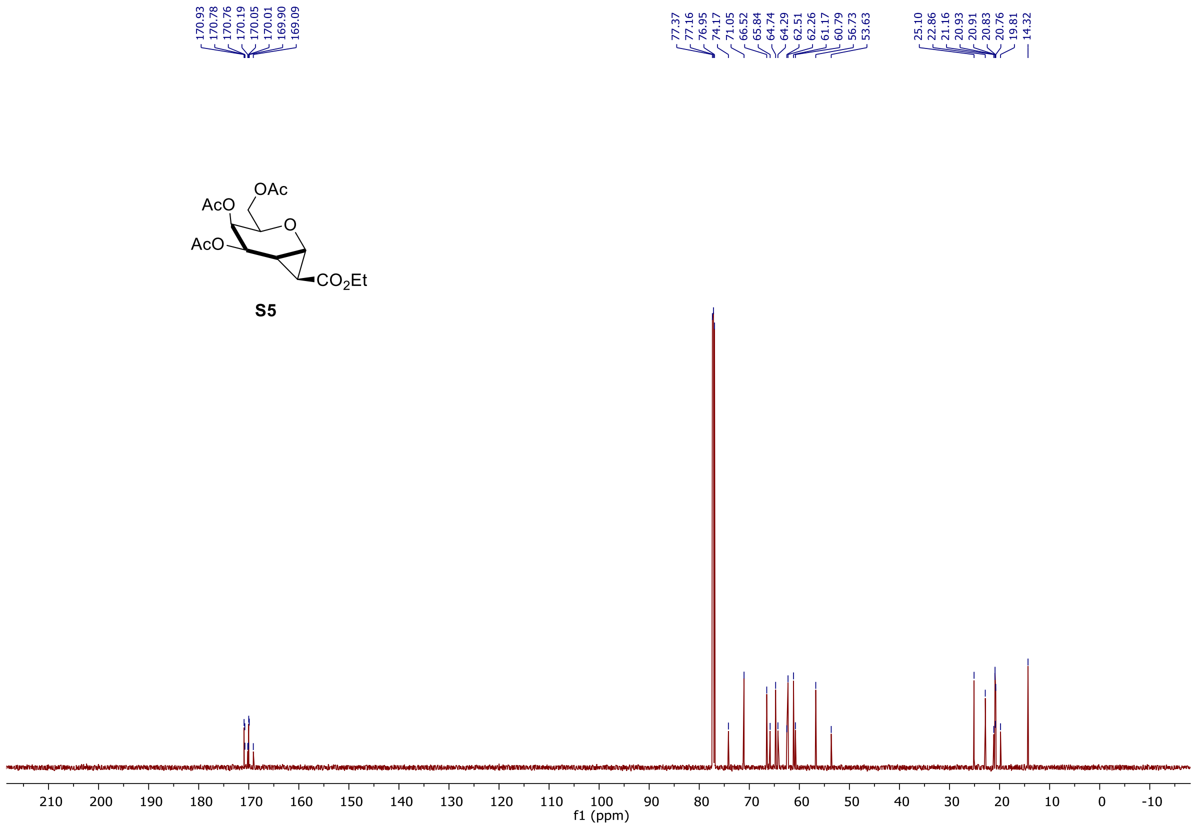

Supplementary Figure 91: ^{13}C spectra for **S5**

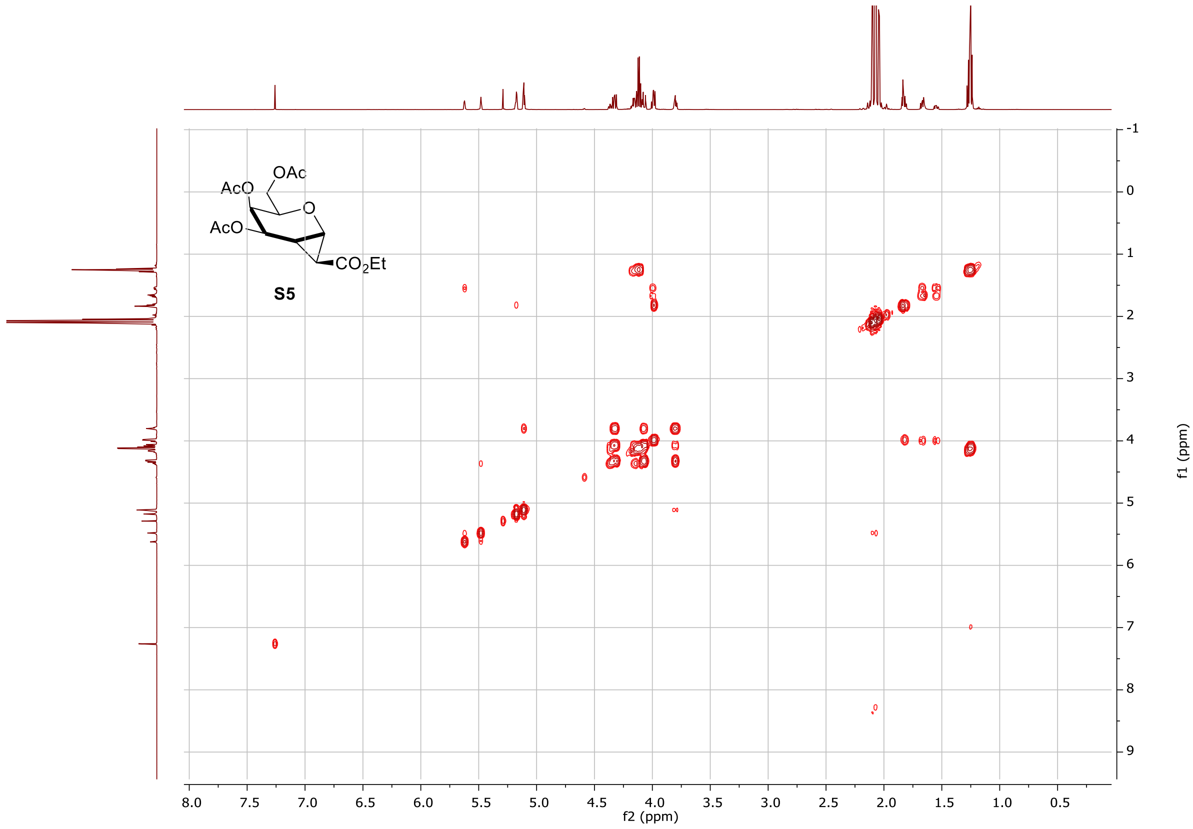

Supplementary Figure 92: COSY spectra for compound **S5**

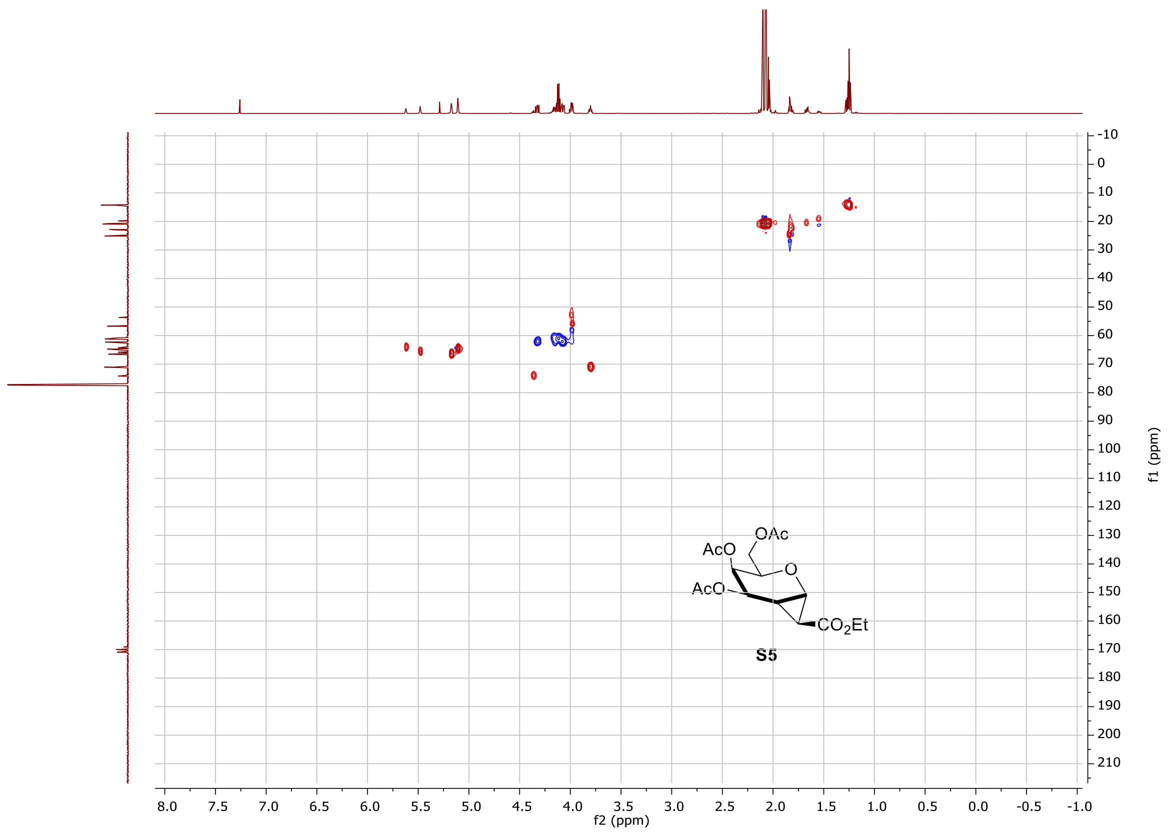

Supplementary Figure 93: HSQC spectra for compound **S5**

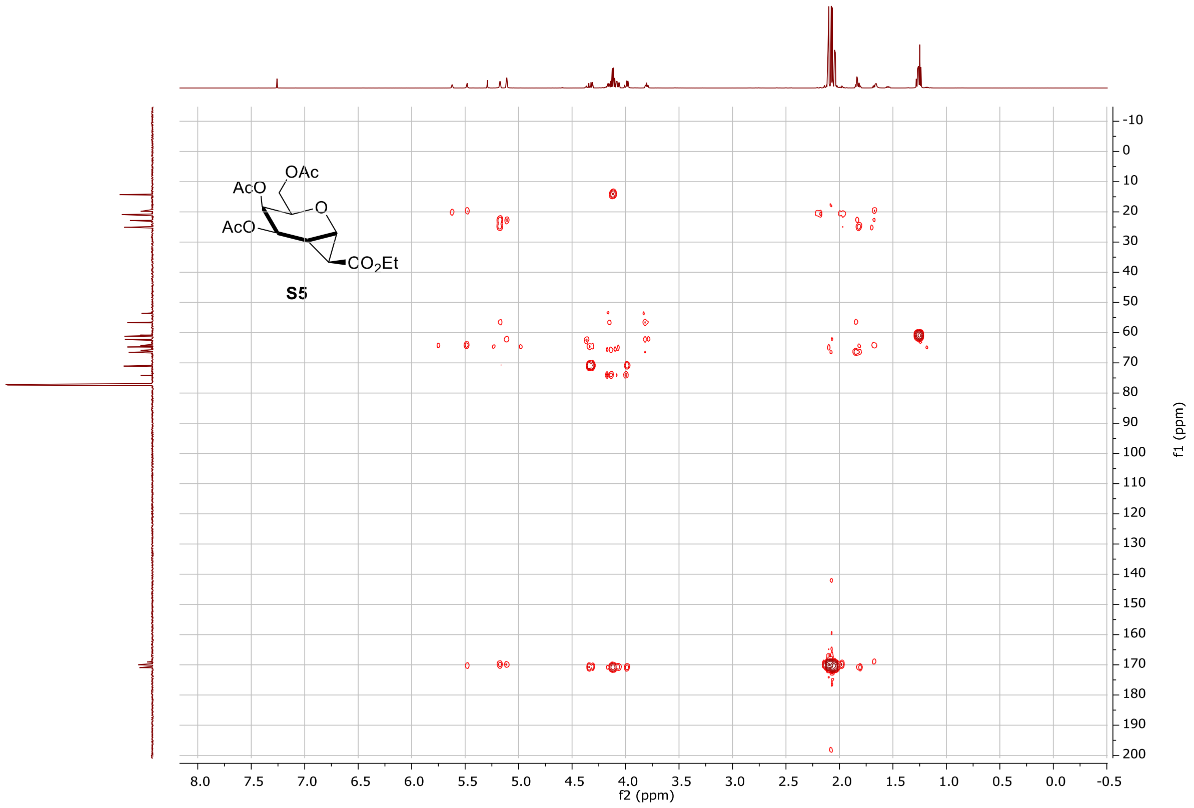

Supplementary Figure 94: HMBC spectra for compound **S5**

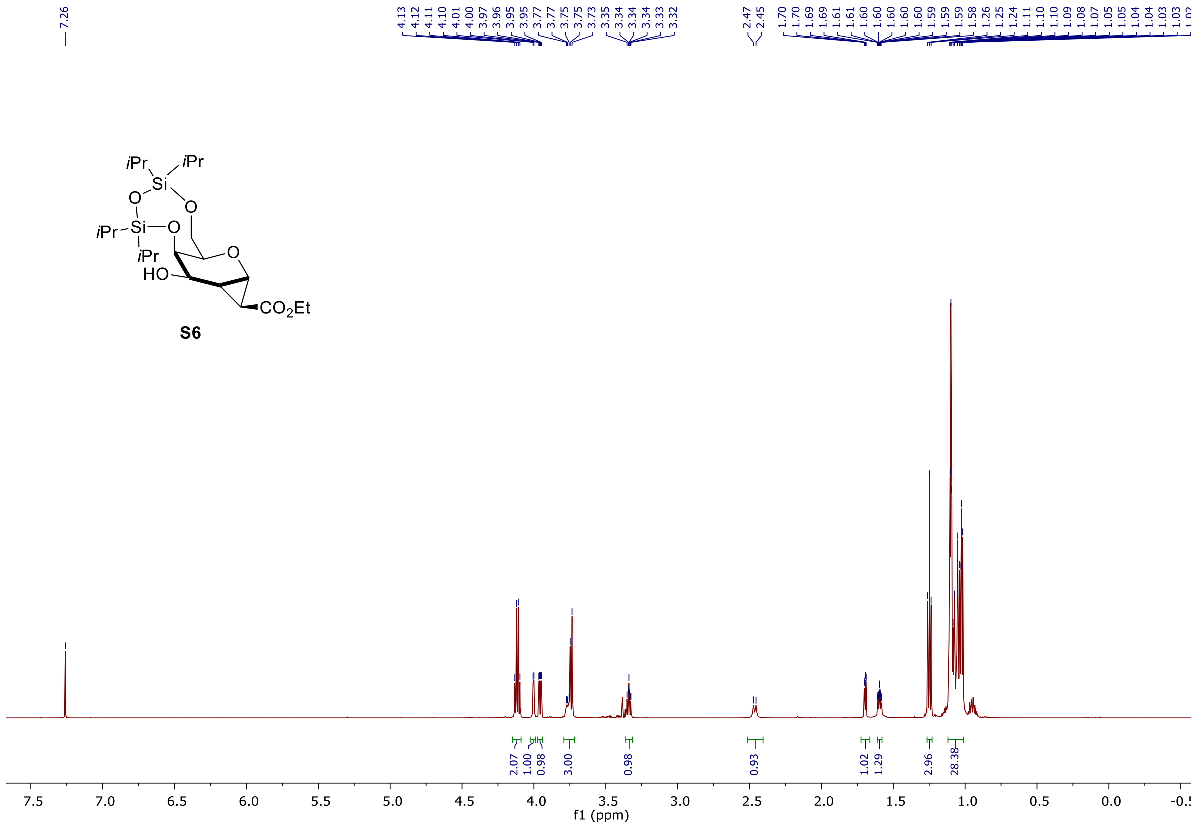

Supplementary Figure 95: ^{1}H spectra for **S6**

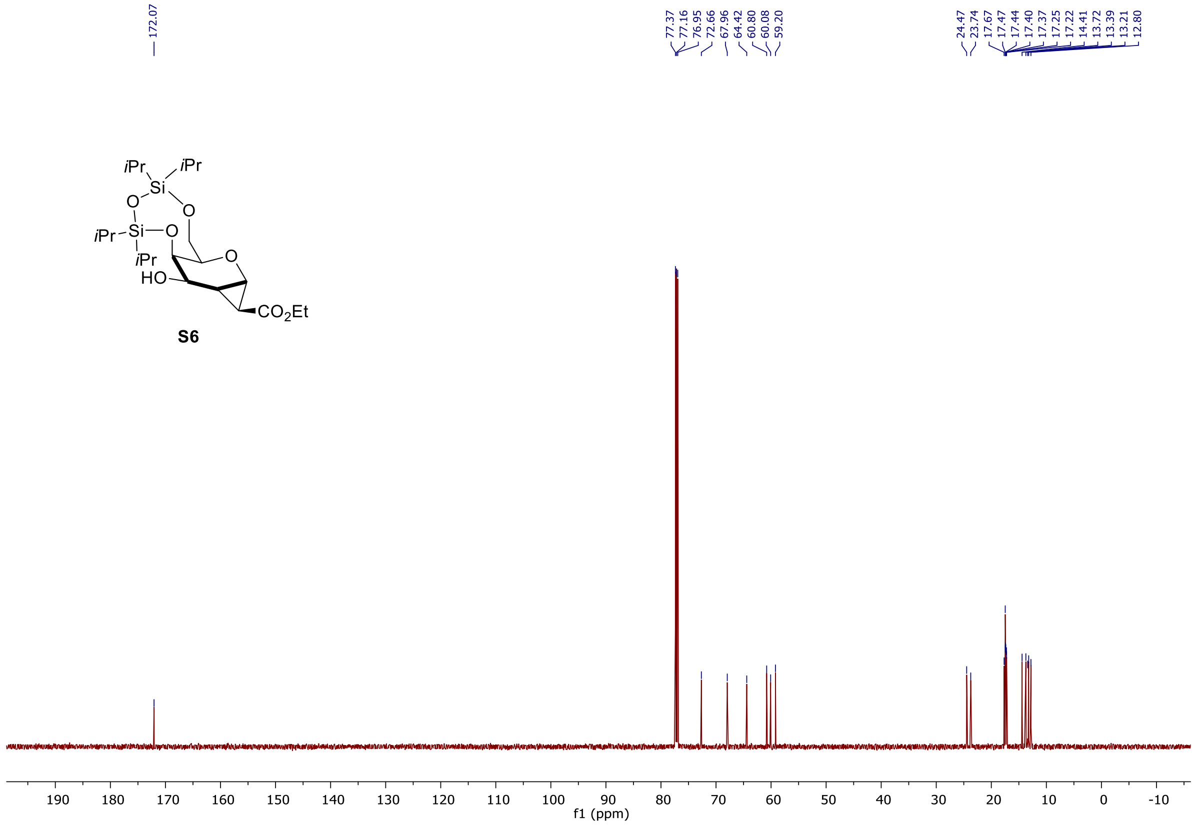

Supplementary Figure 96: ^{13}C spectra for **S6**

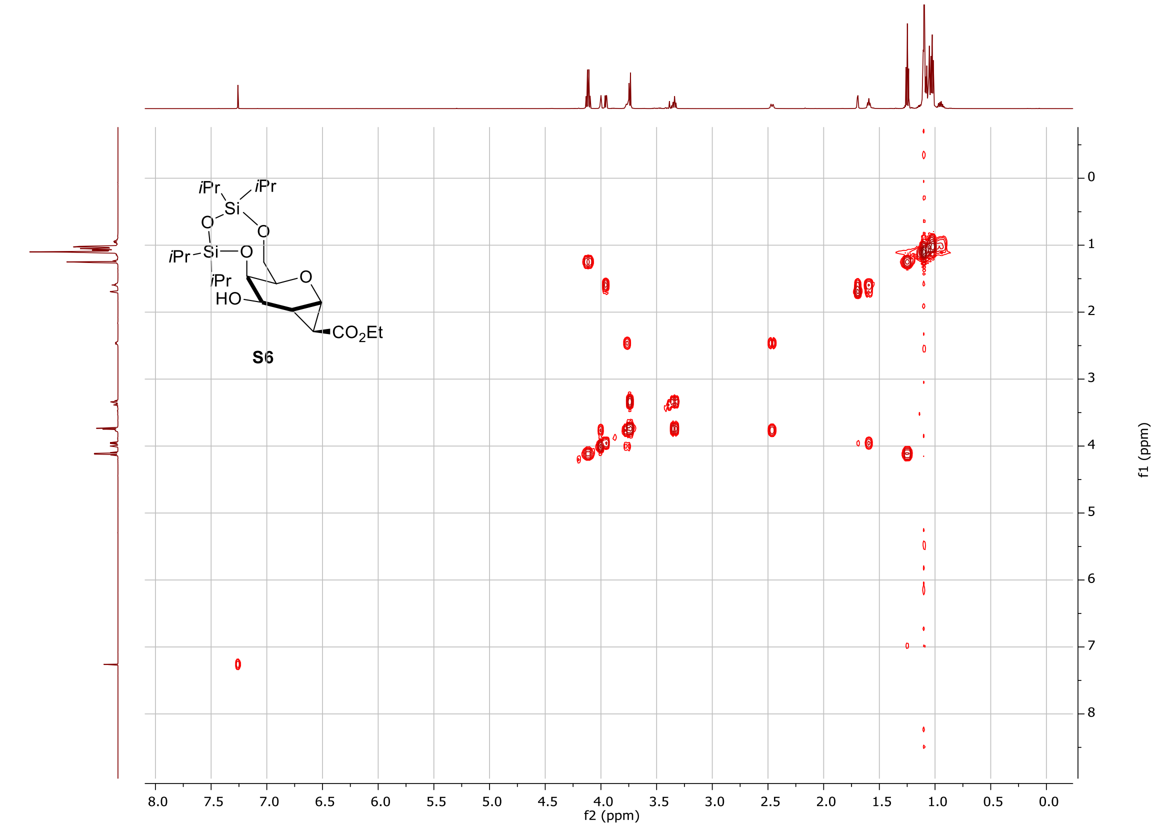

Supplementary Figure 97: COSY spectra for compound **S6**

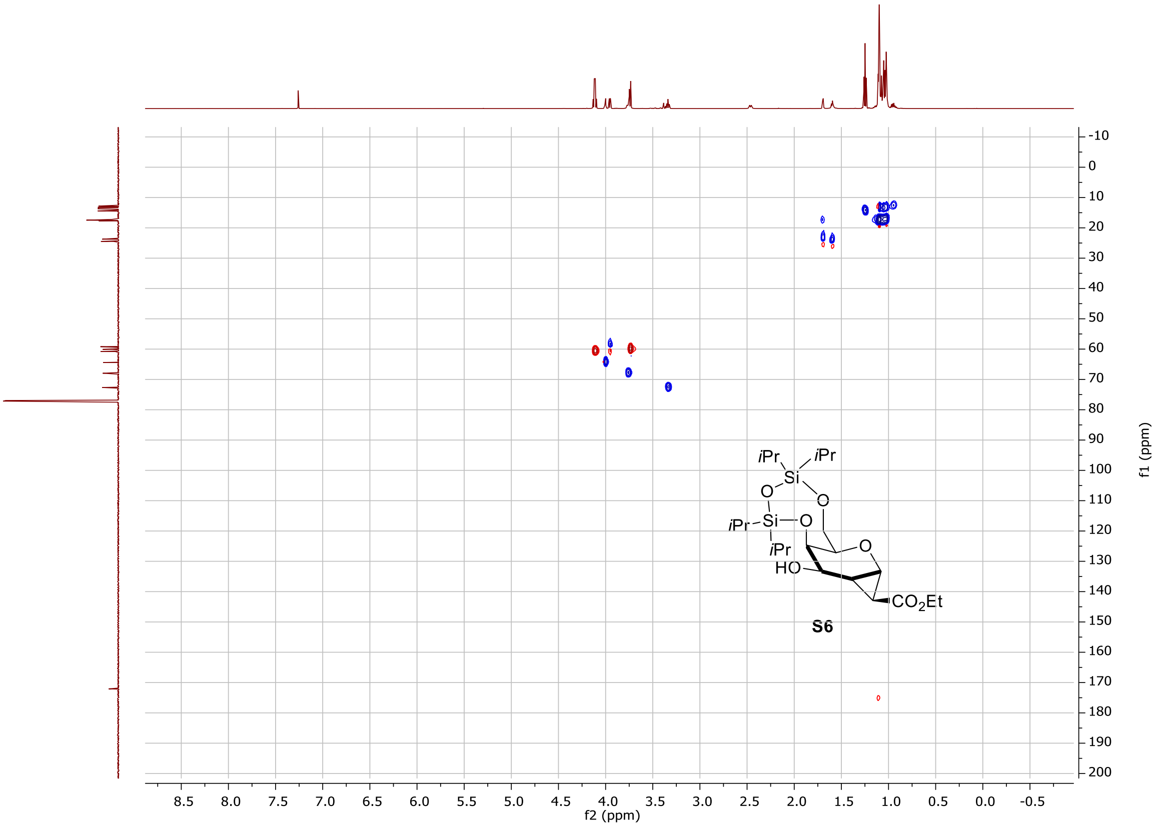

Supplementary Figure 98: HSQC spectra for compound **S6**

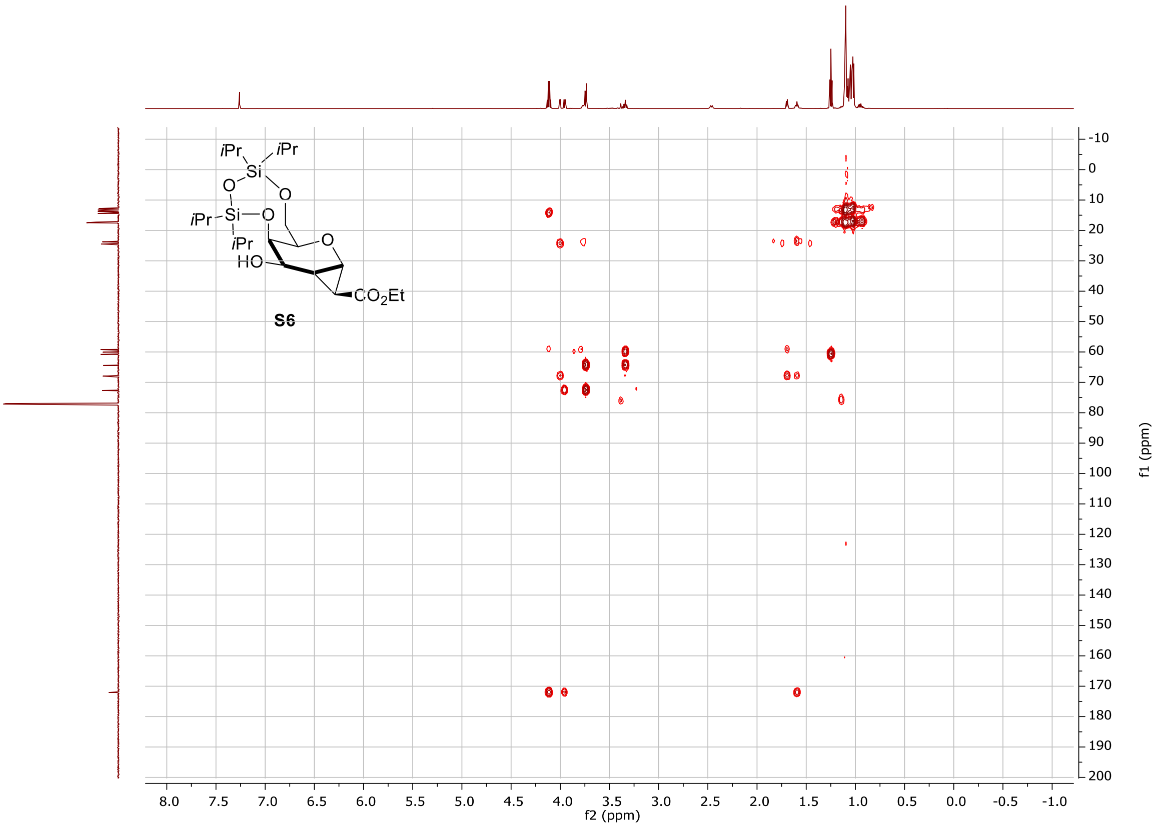

Supplementary Figure 99: HMBC spectra for compound **S6**

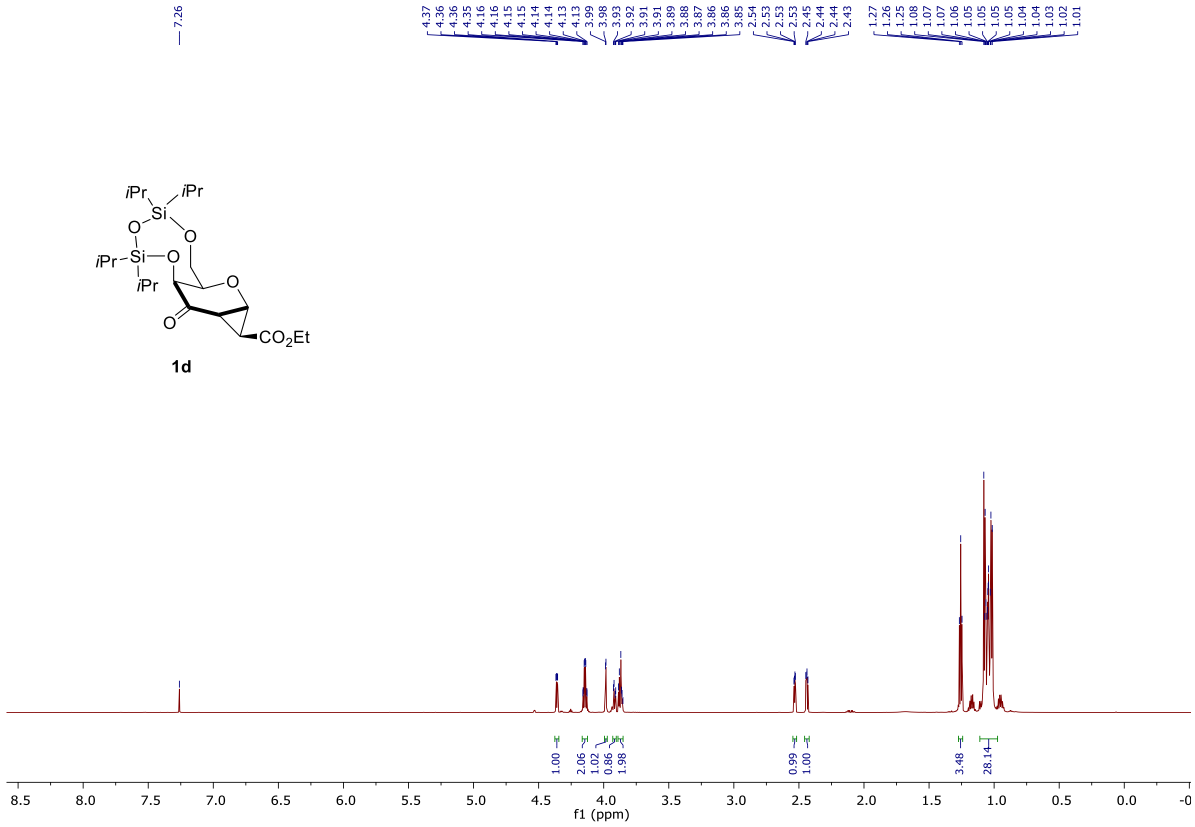

Supplementary Figure 100: ^{1}H spectra for **1d**

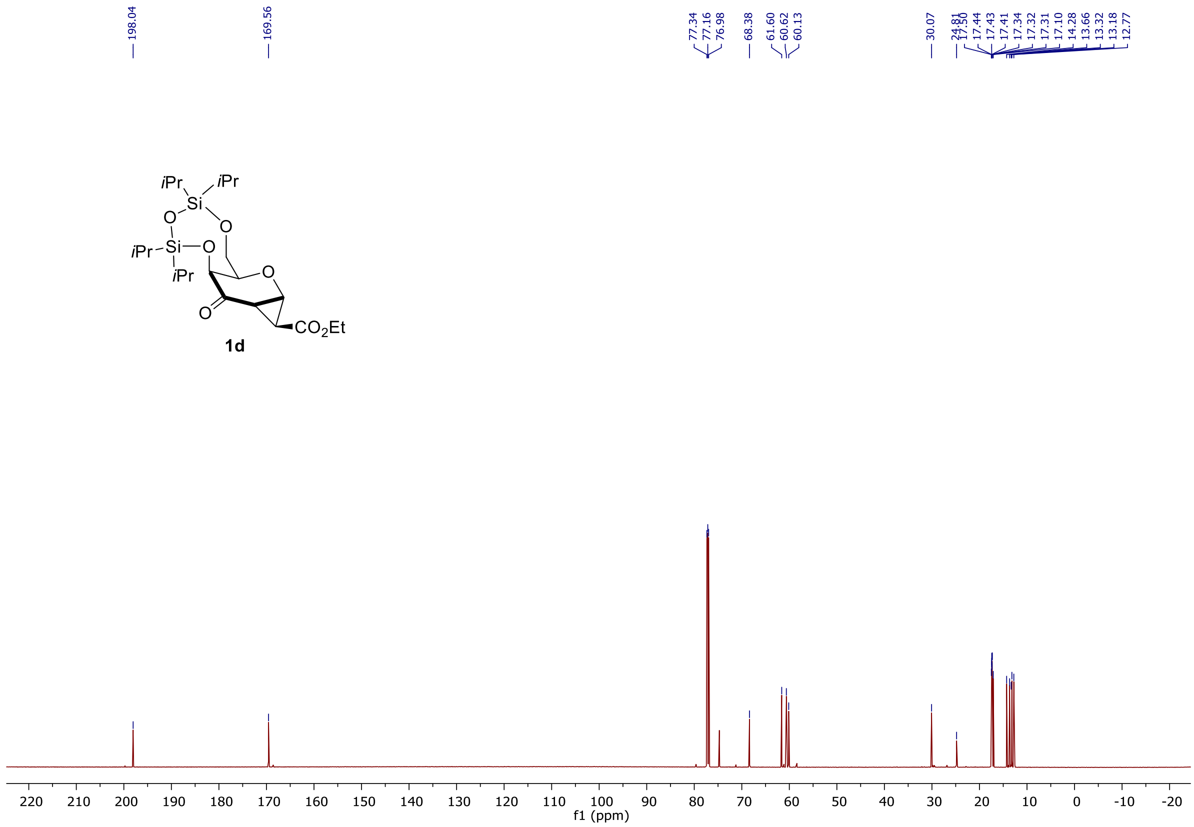

Supplementary Figure 101: ^{13}C spectra for **1d**

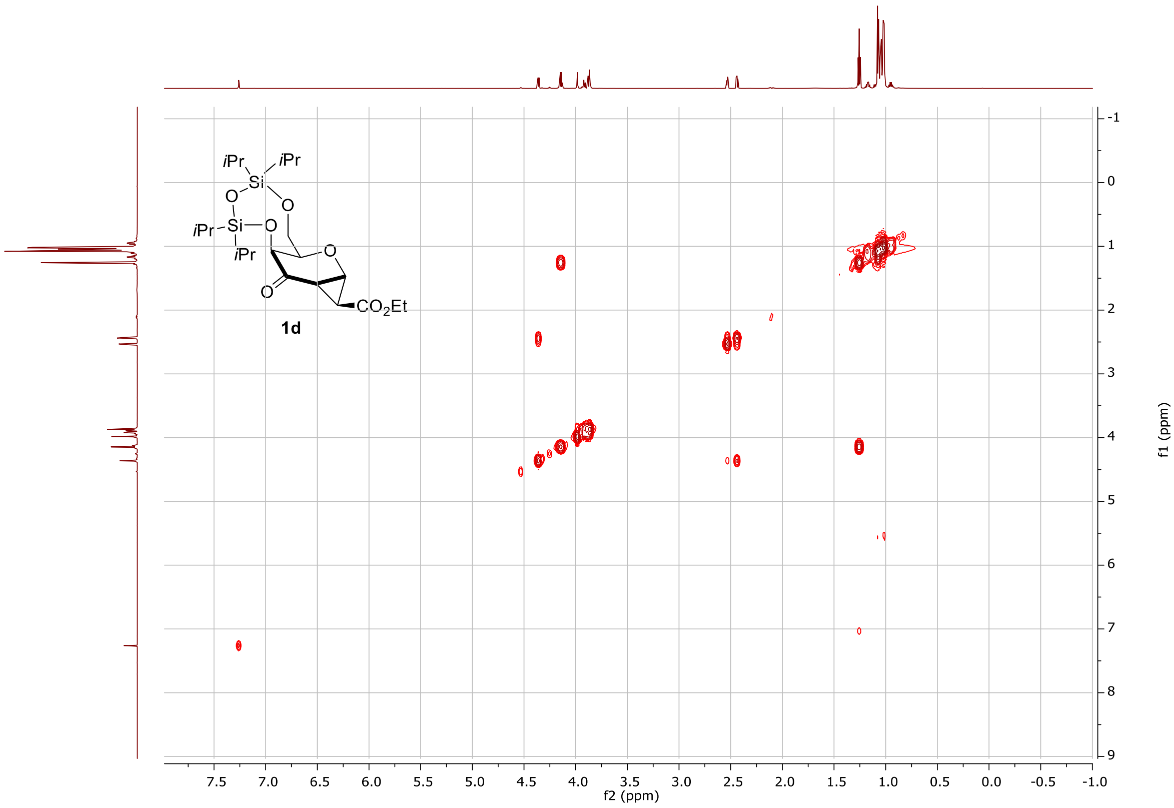

Supplementary Figure 102: COSY spectra for compound **1d**

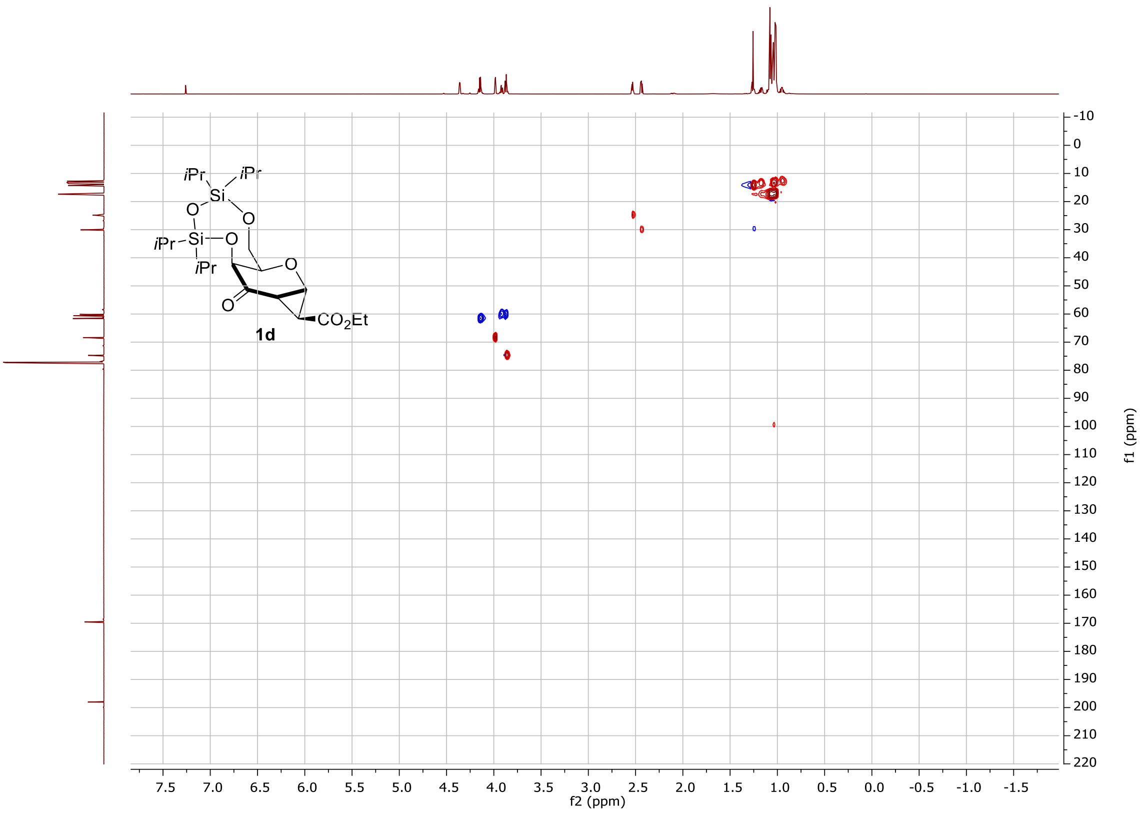

Supplementary Figure 103: HSQC spectra for compound **1d**

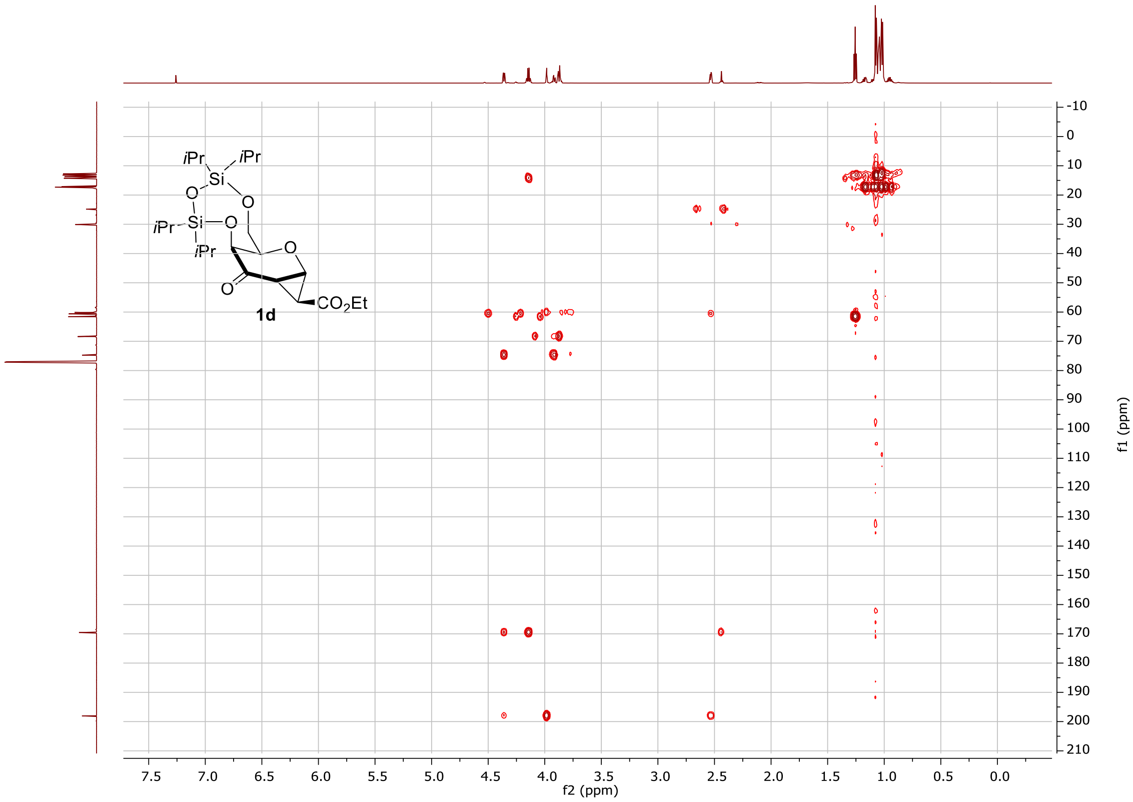

Supplementary Figure 104: HMBC spectra for compound **1d**

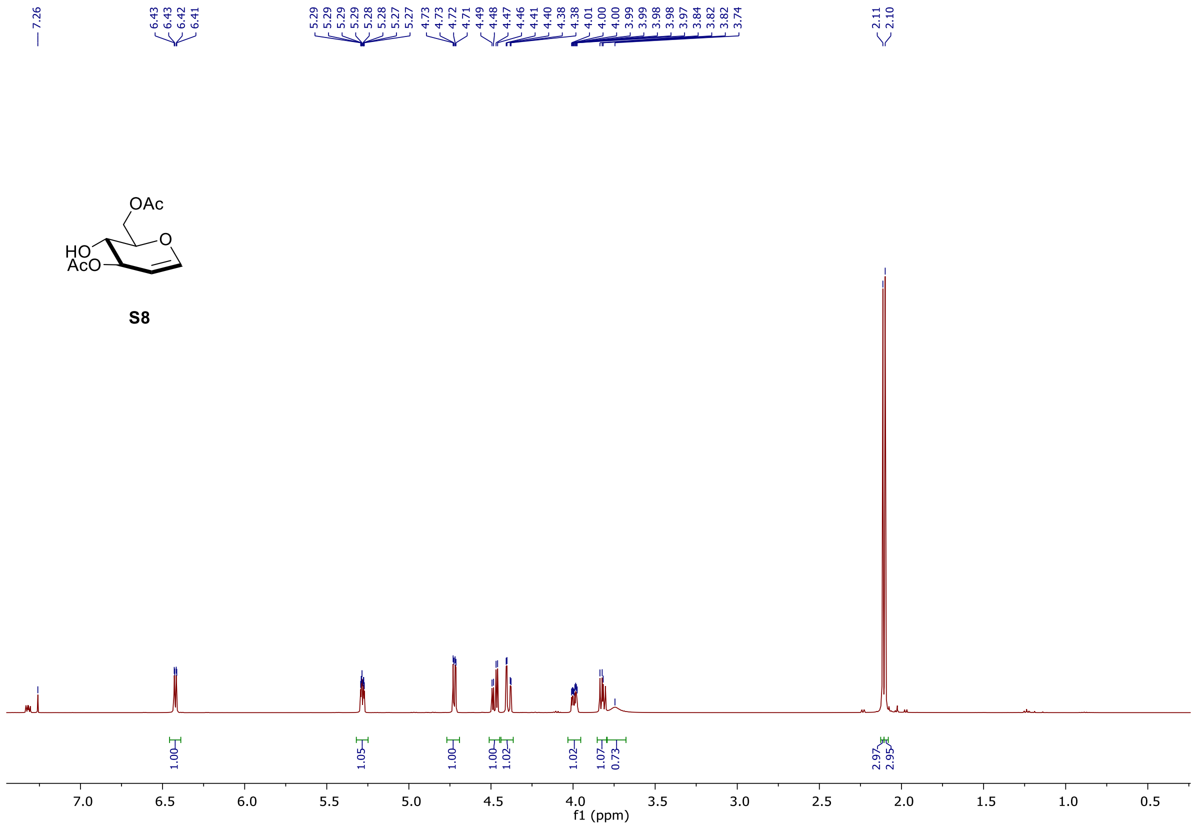

Supplementary Figure 105: ^{1}H spectra for **S8**

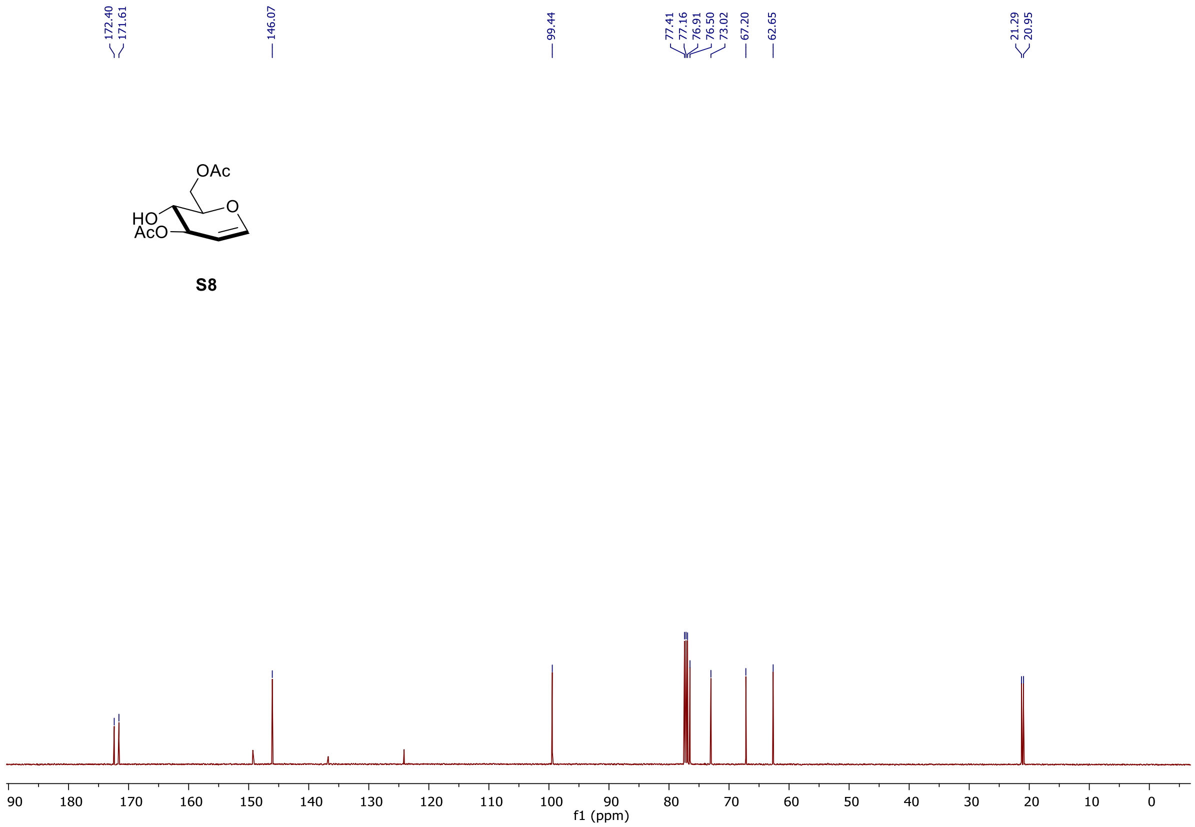

Supplementary Figure 106: ^{13}C spectra for **S8**

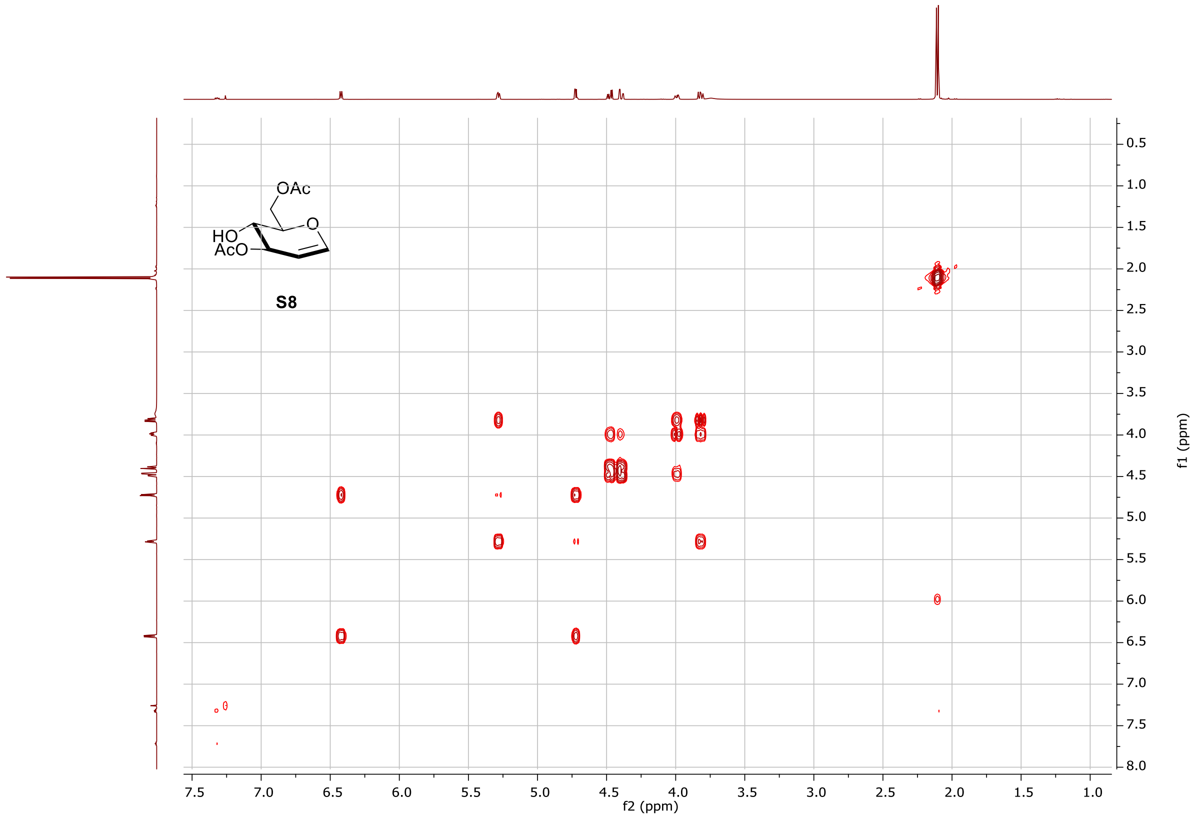

Supplementary Figure 107: COSY spectra for compound **S8**

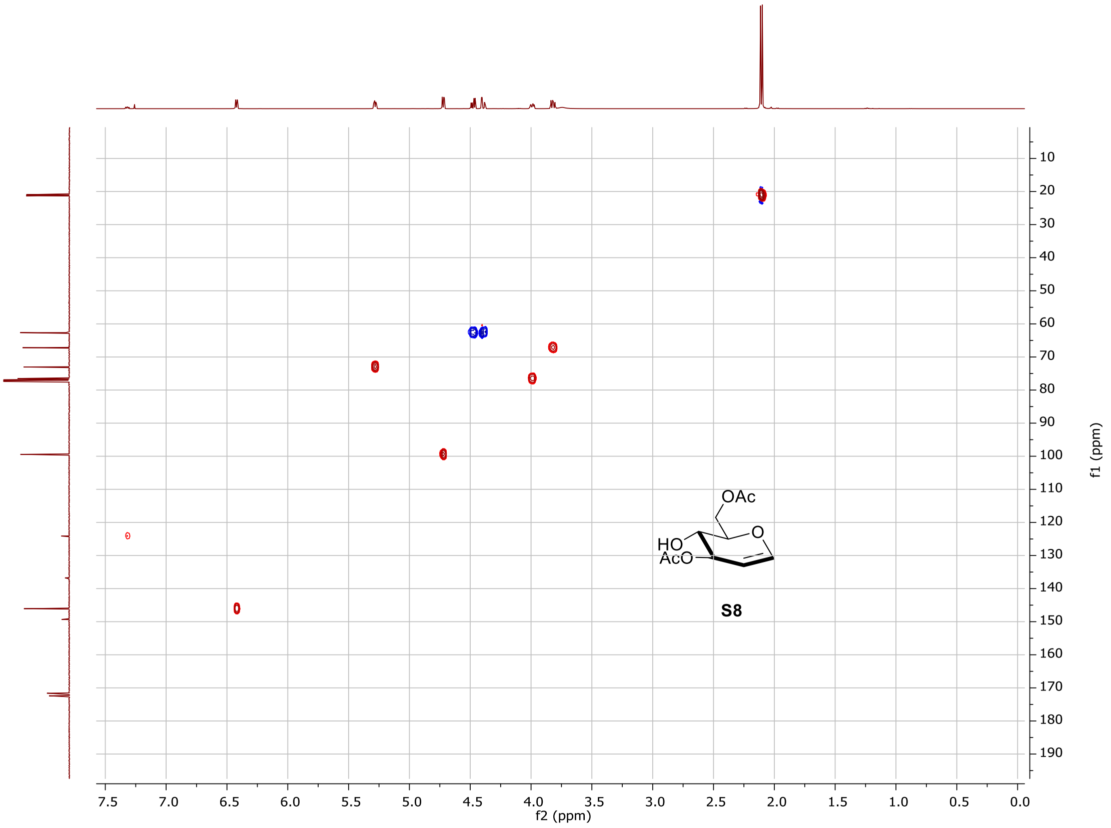

Supplementary Figure 108: HSQC spectra for compound **S8**

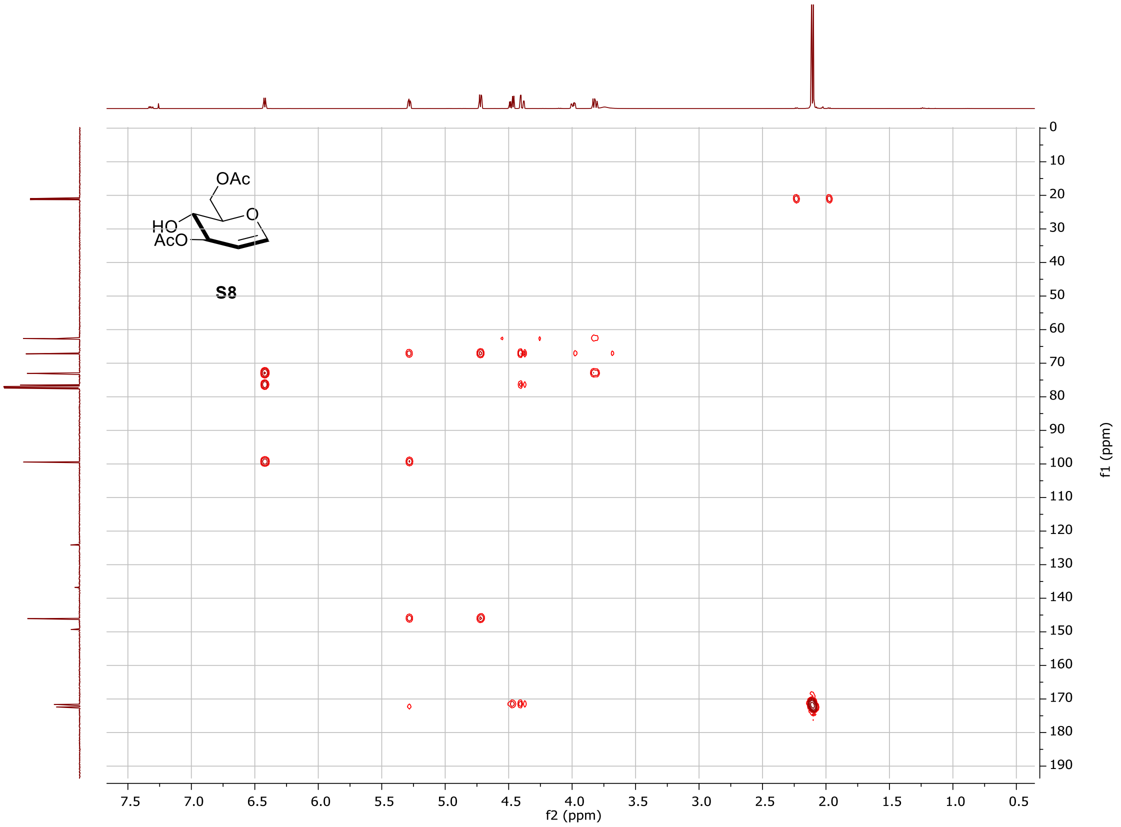

Supplementary Figure 109: HMBC spectra for compound **S8**

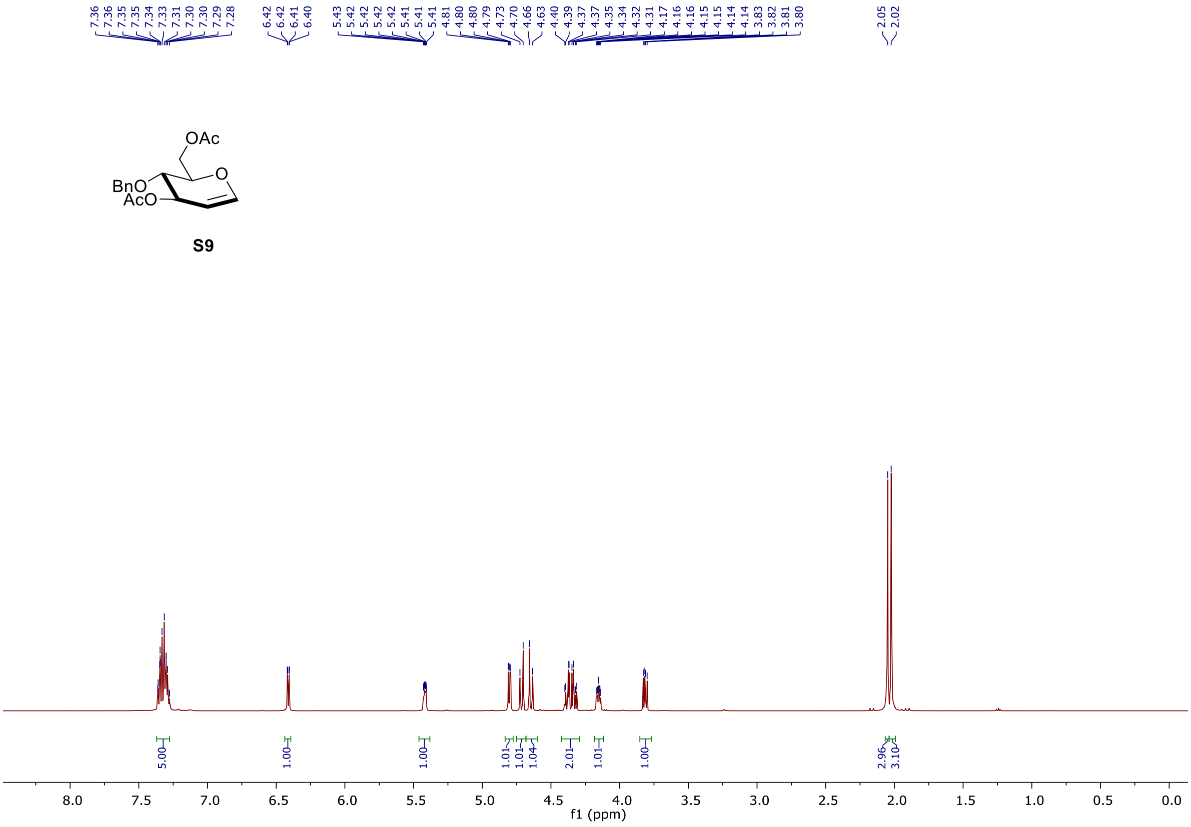

Supplementary Figure 110: ^{1}H spectra for **S9**

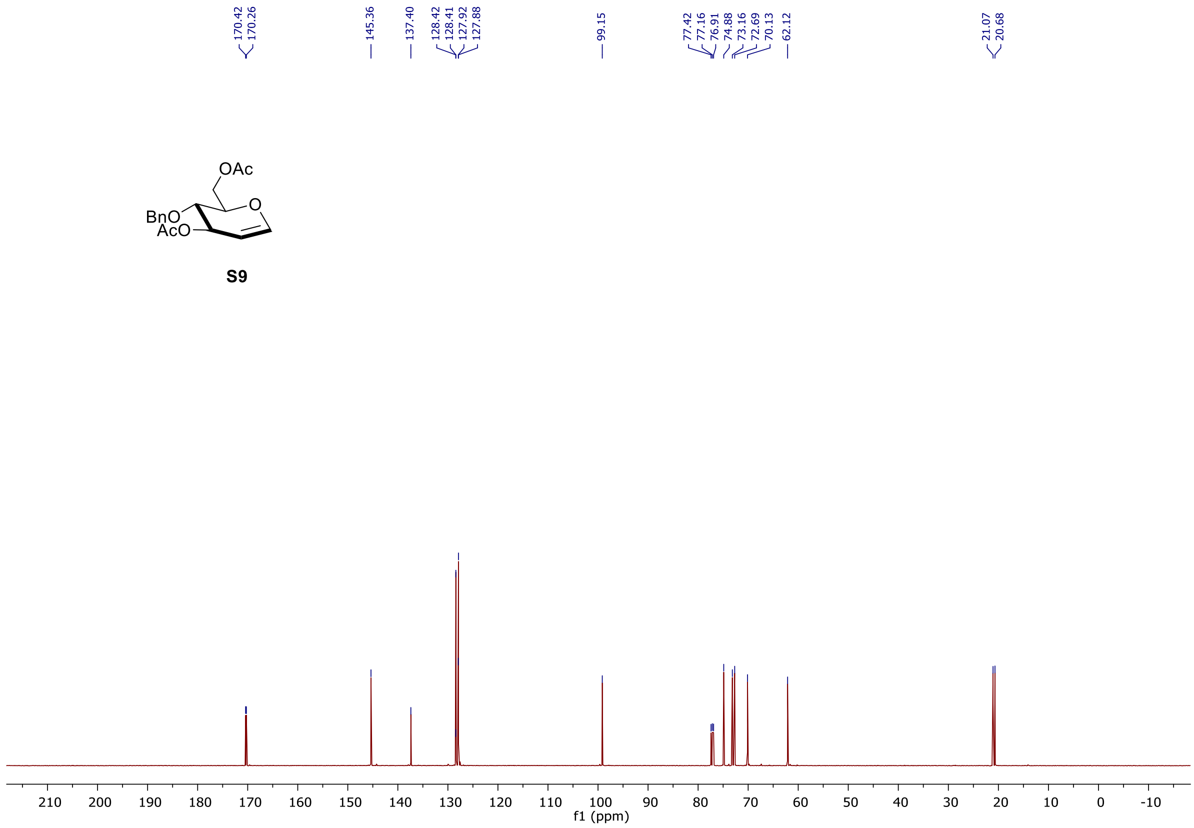

Supplementary Figure 111: ^{13}C spectra for **S9**

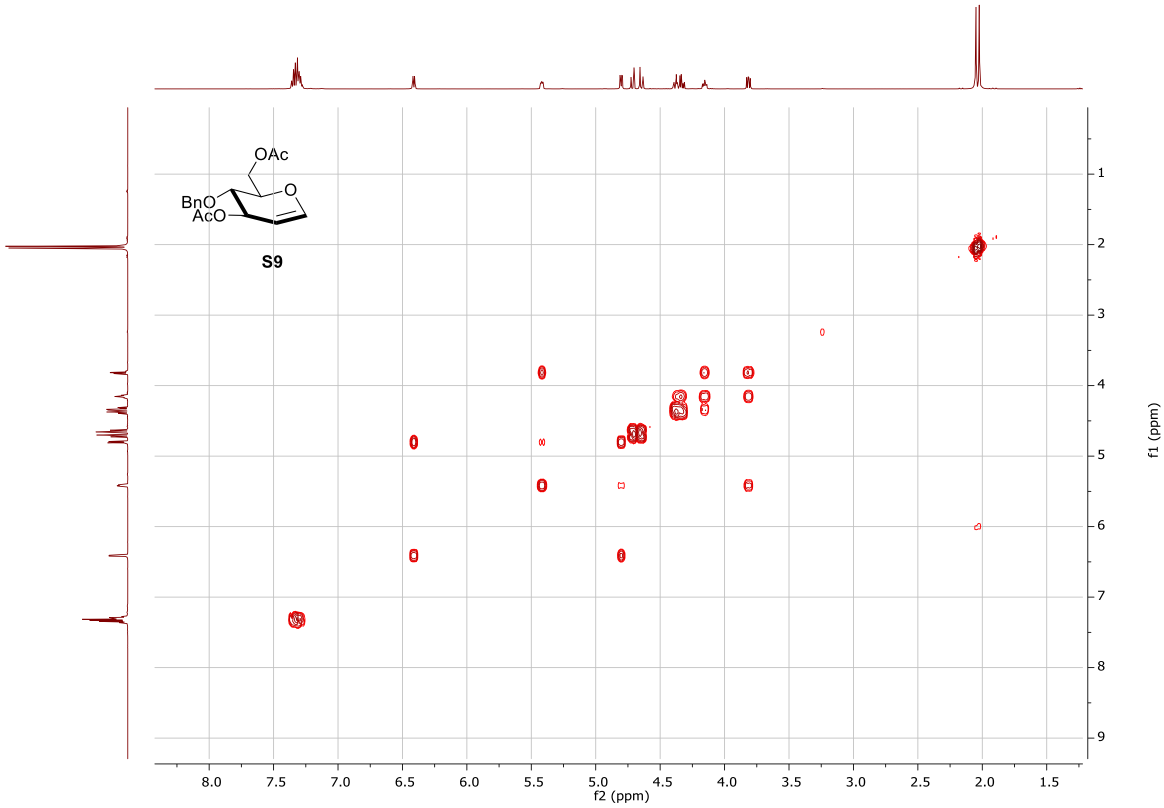

Supplementary Figure 112: COSY spectra for compound **S9**

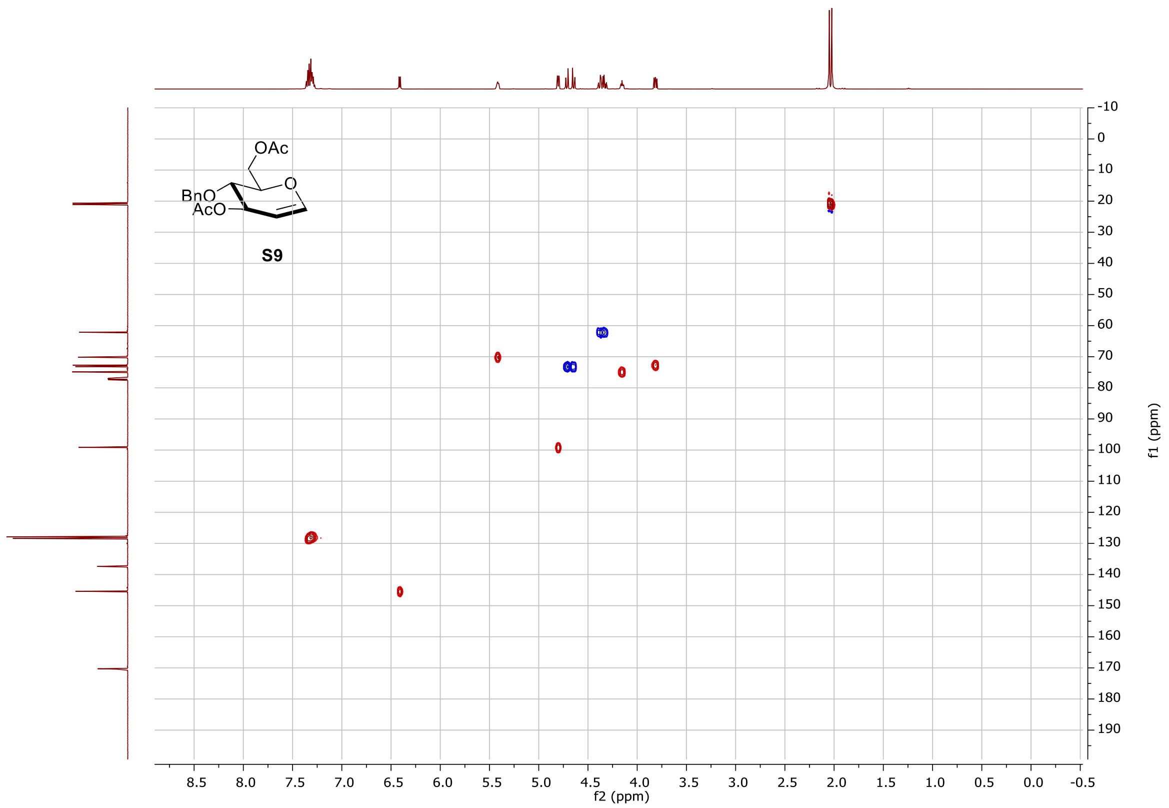

Supplementary Figure 113: HSQC spectra for compound **S9**

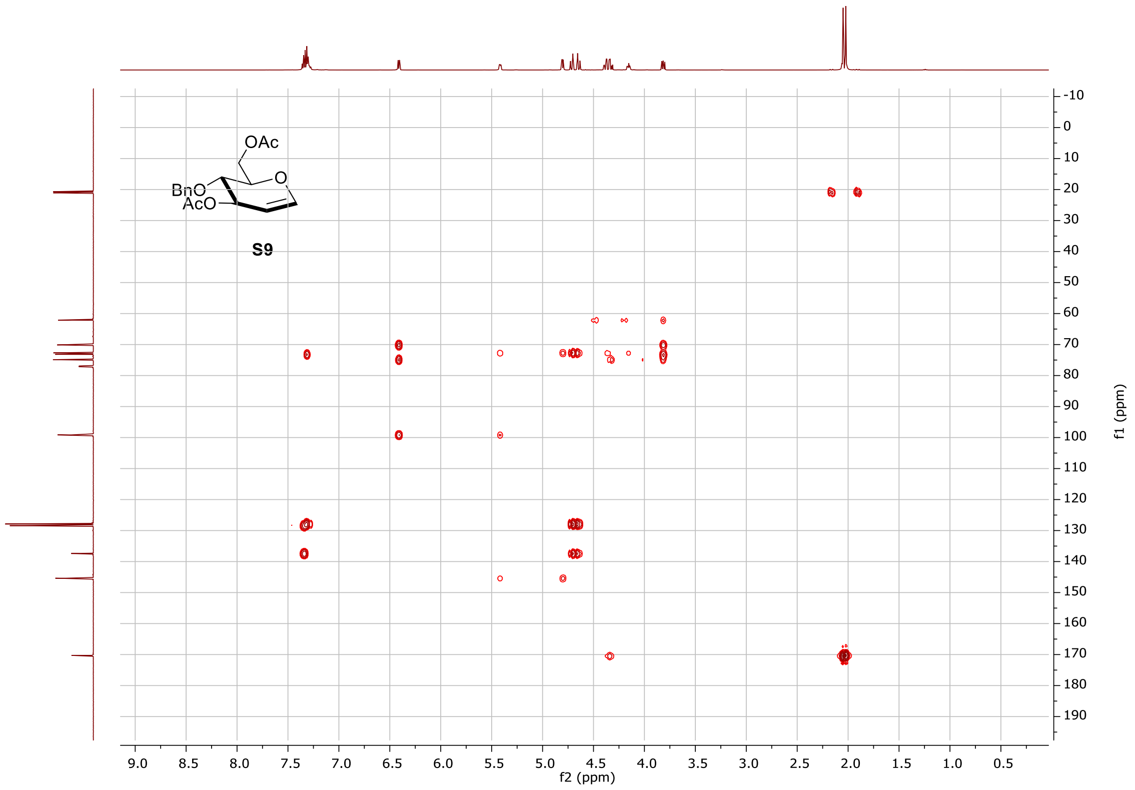

Supplementary Figure 114: HMBC spectra for compound **S9**

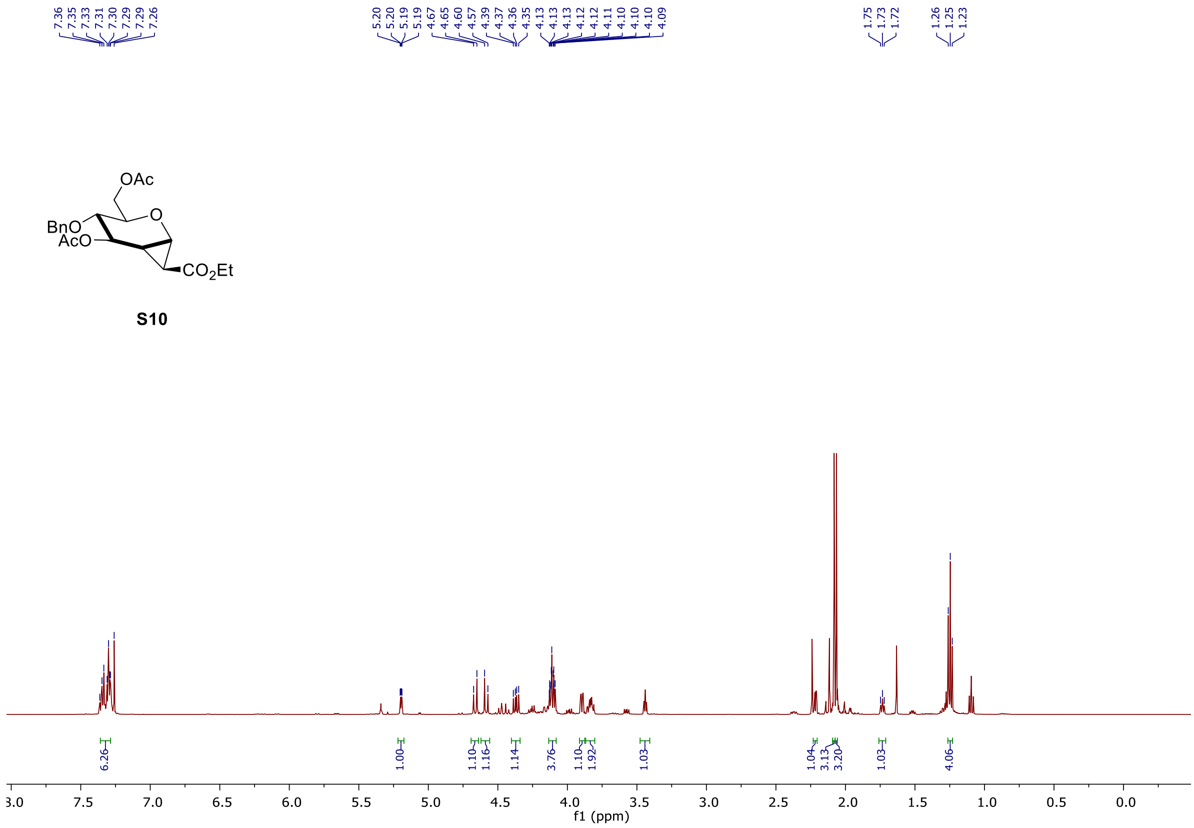

Supplementary Figure 115: ^{1}H spectra for **S10**

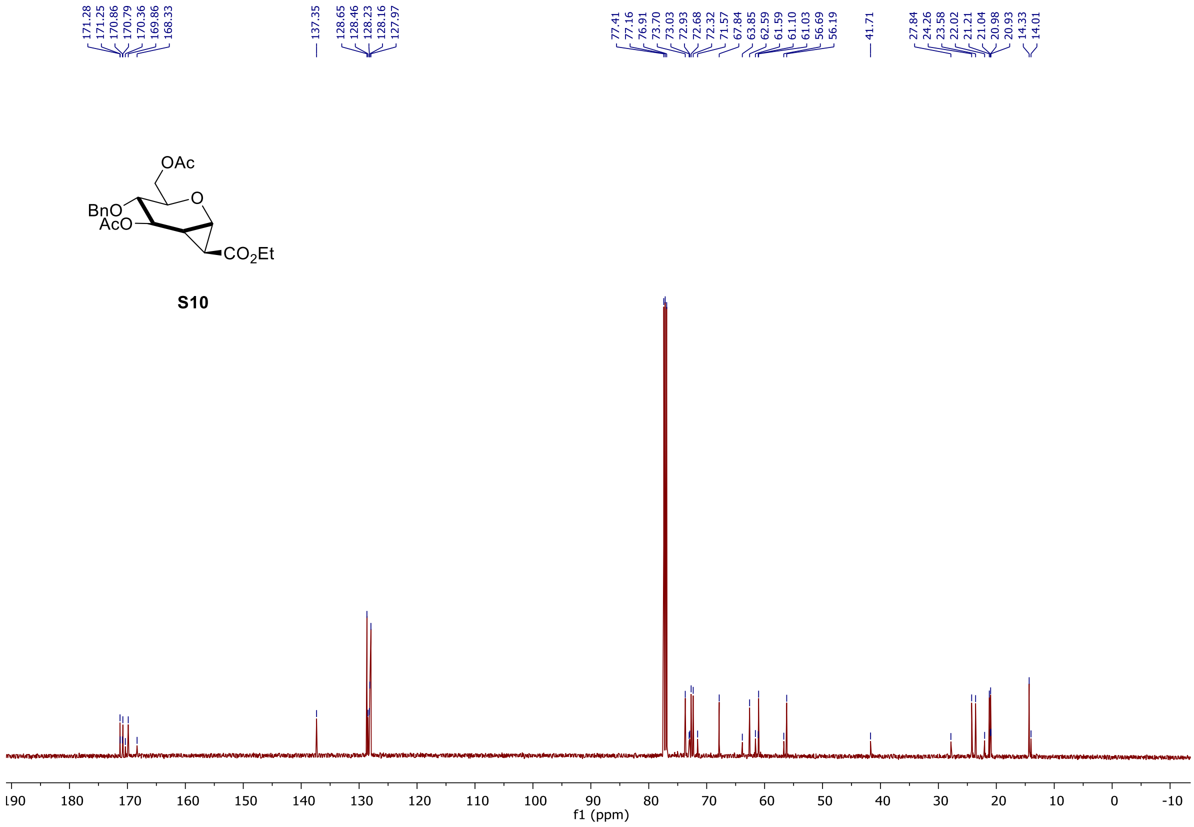

Supplementary Figure 116: ^{13}C spectra for **S10**

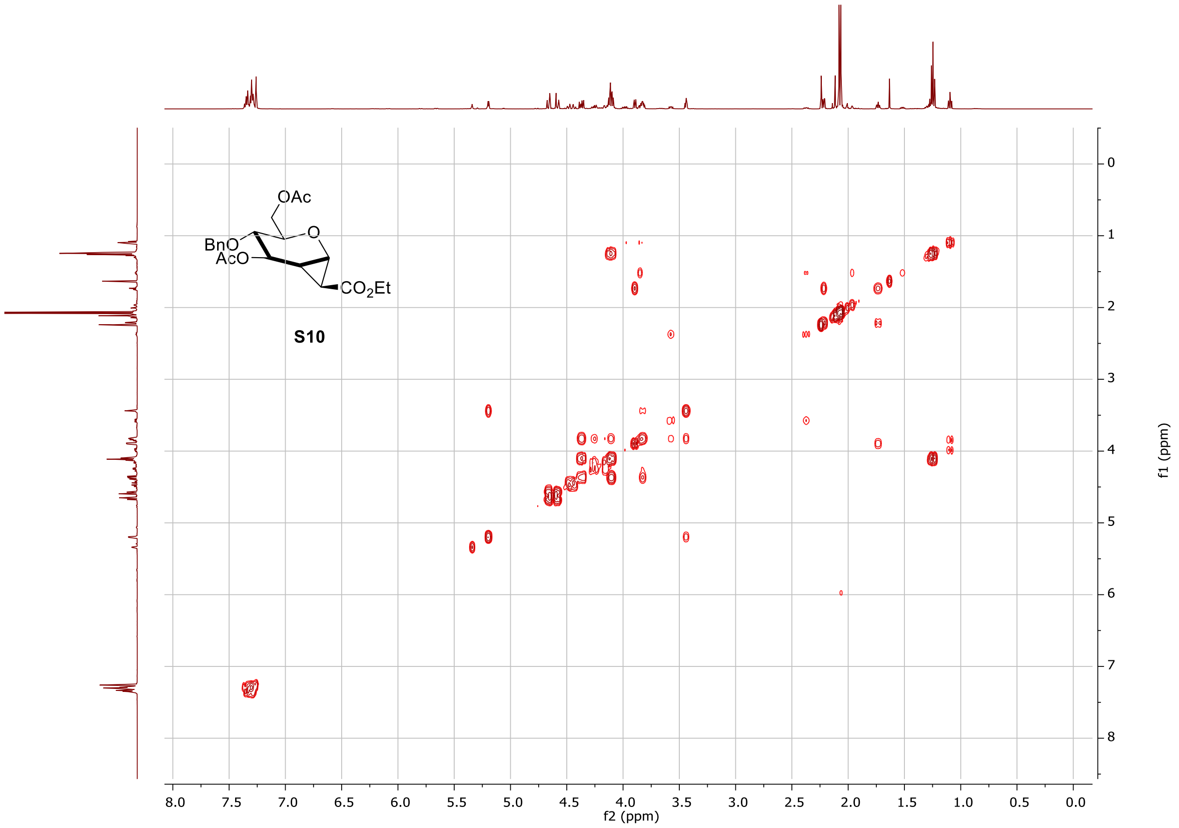

Supplementary Figure 117: COSY spectra for compound **S10**

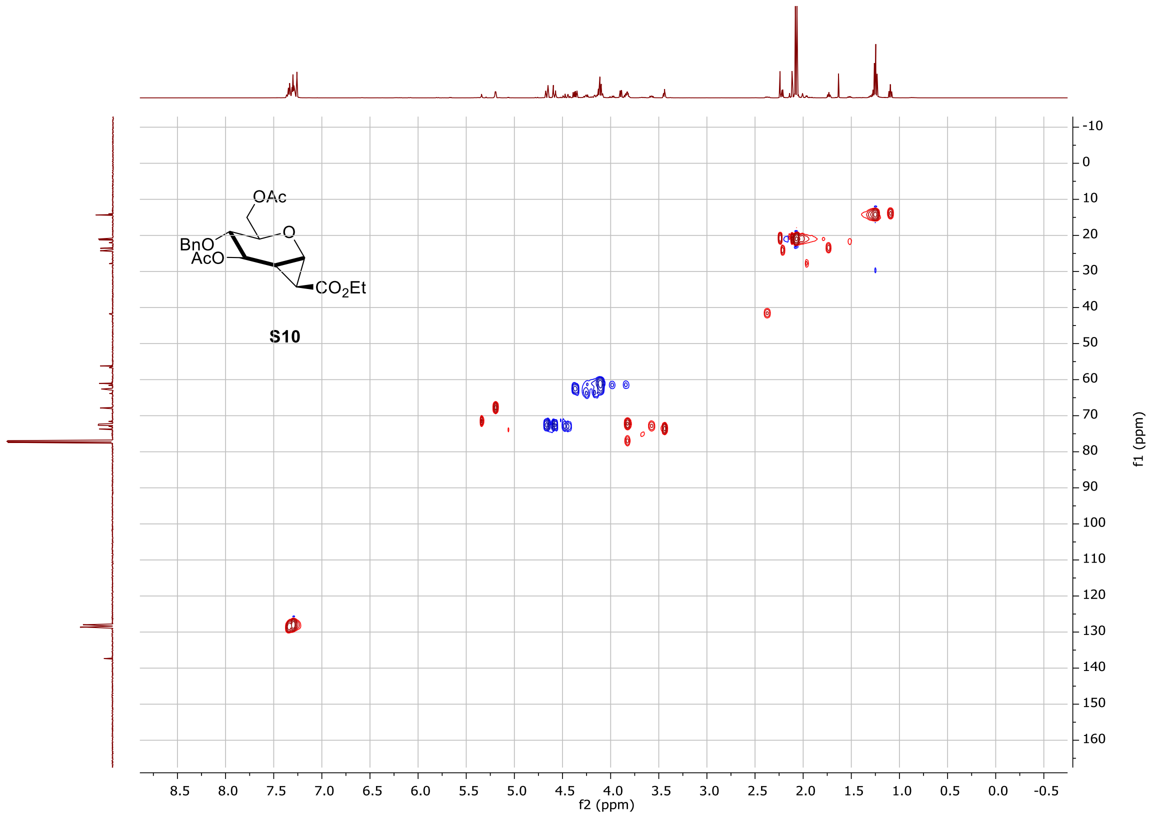

Supplementary Figure 118: HSQC spectra for compound **S10**

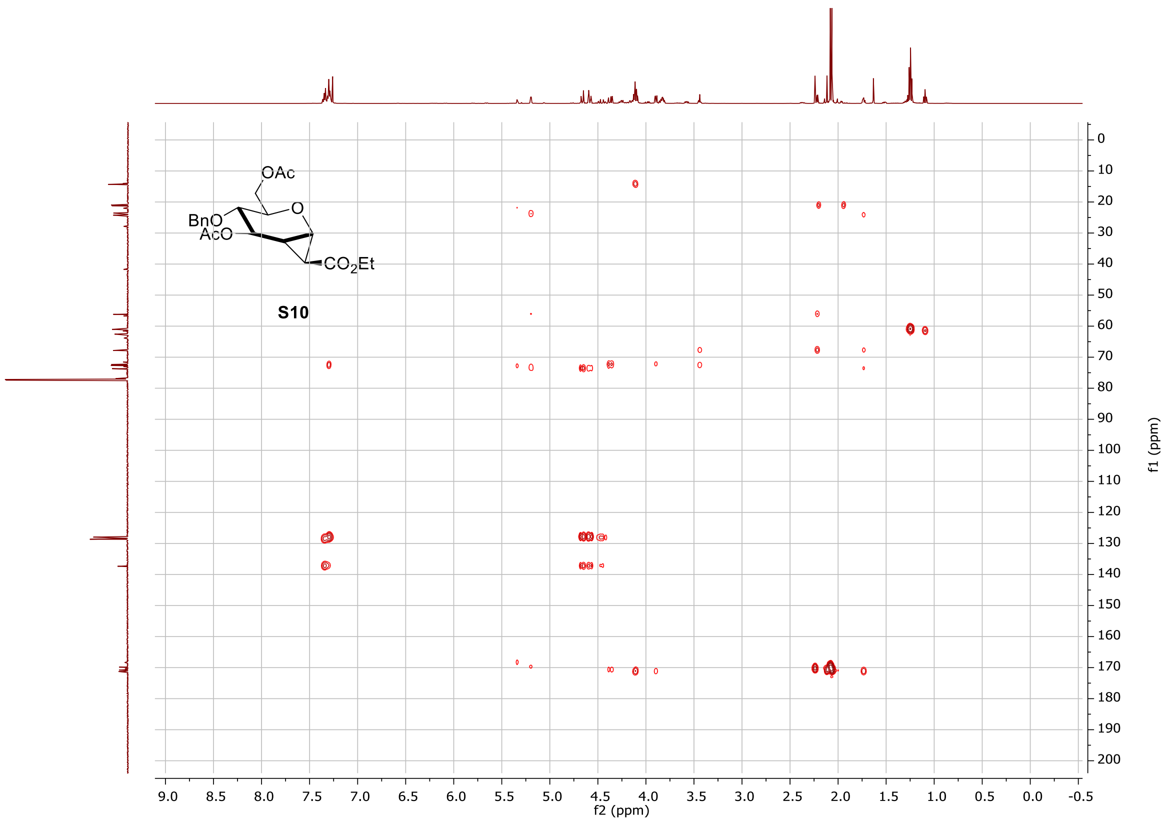

Supplementary Figure 119: HMBC spectra for compound S10

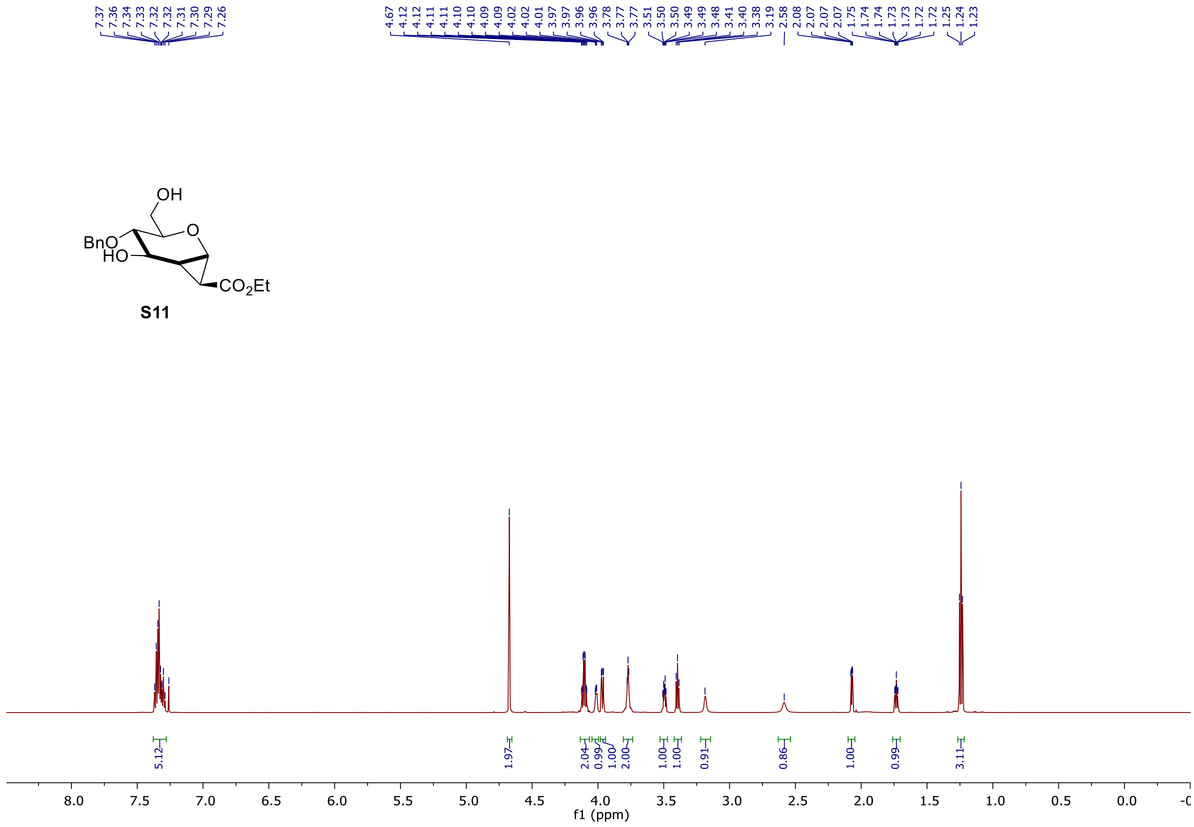

Supplementary Figure 120: ^{1}H spectra for S11

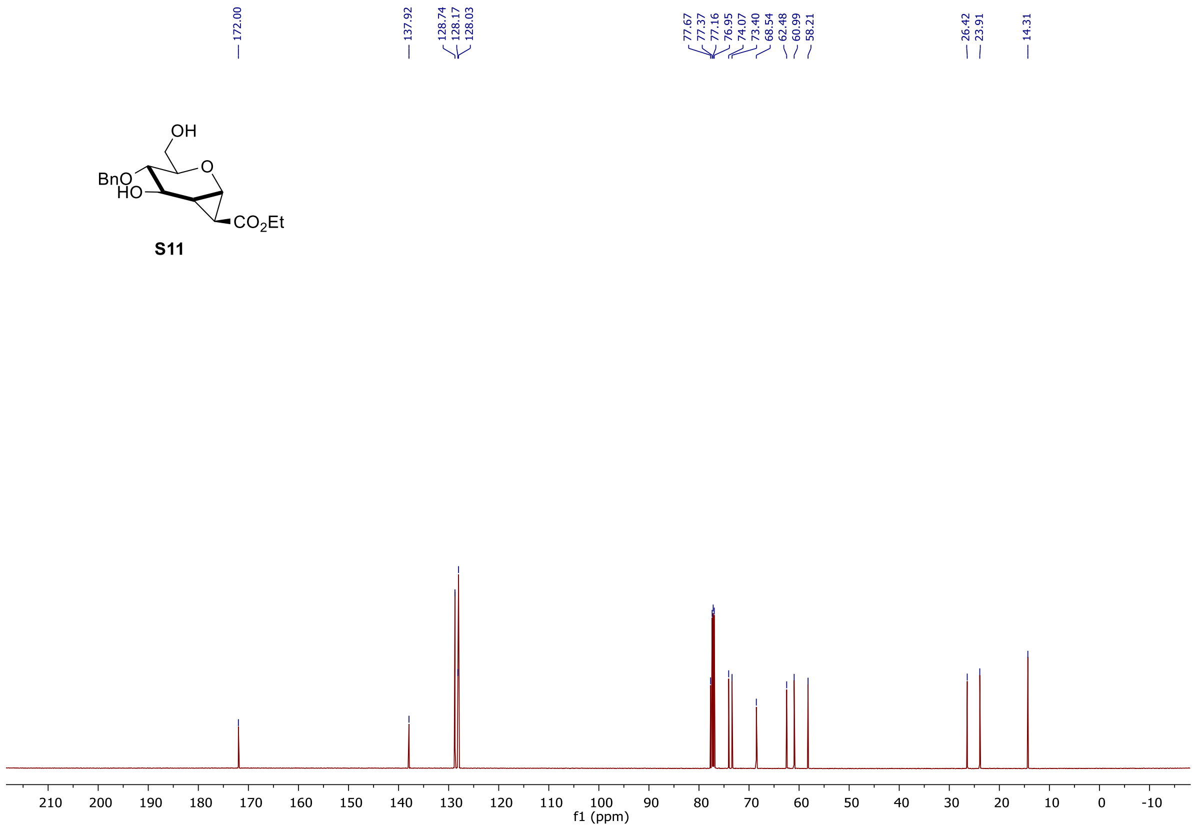

Supplementary Figure 121: ^{13}C spectra for **S11**

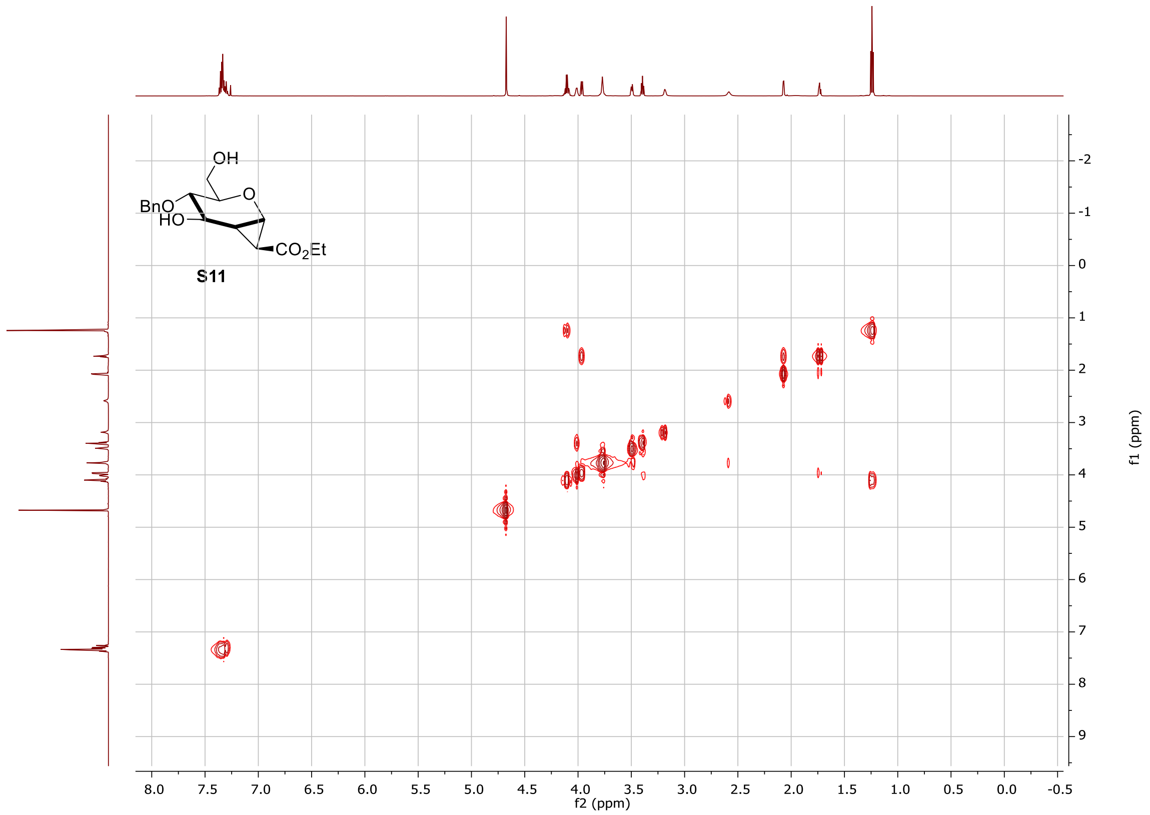

Supplementary Figure 122: COSY spectra for compound **S11**

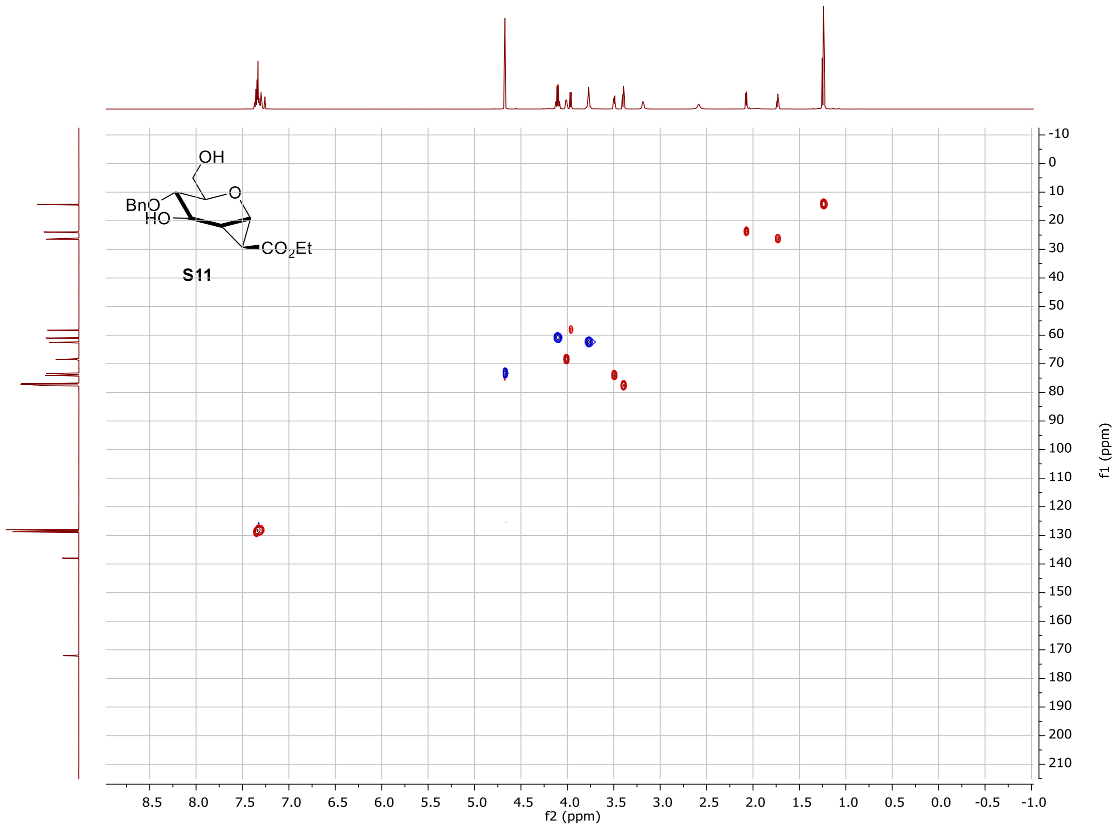

Supplementary Figure 123: HSQC spectra for compound **S11**

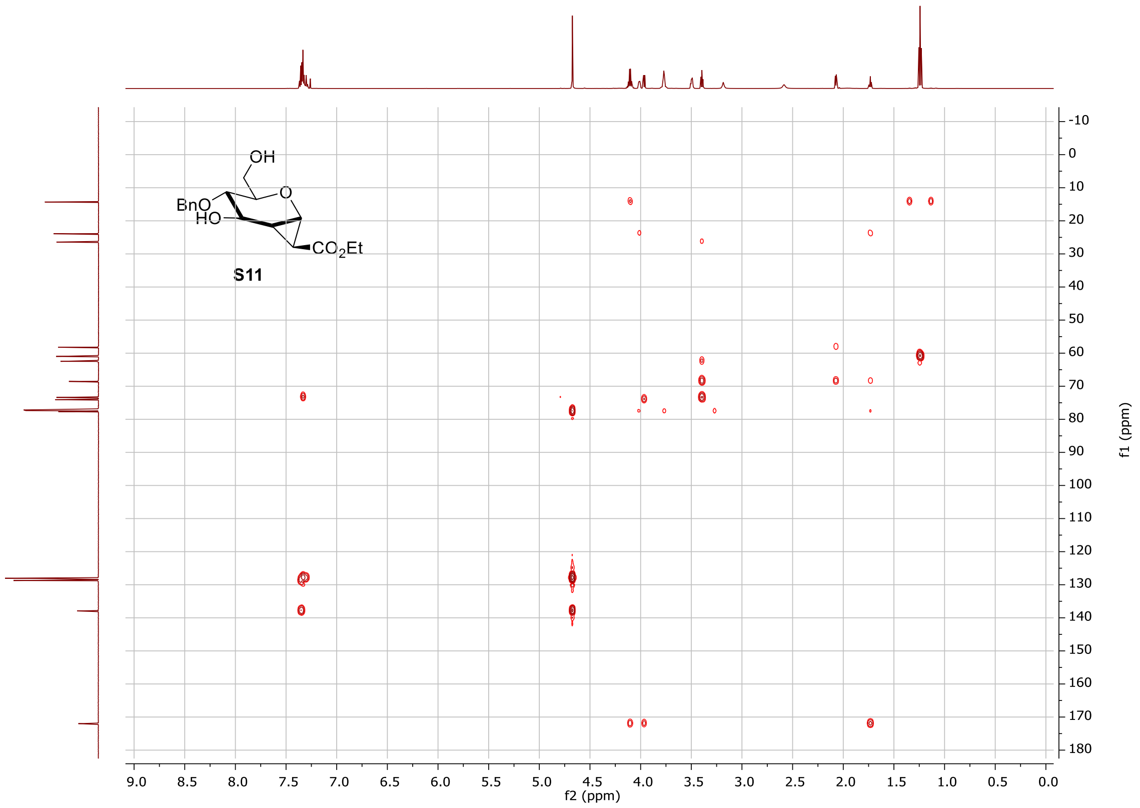

Supplementary Figure 124: HMBC spectra for compound **S11**

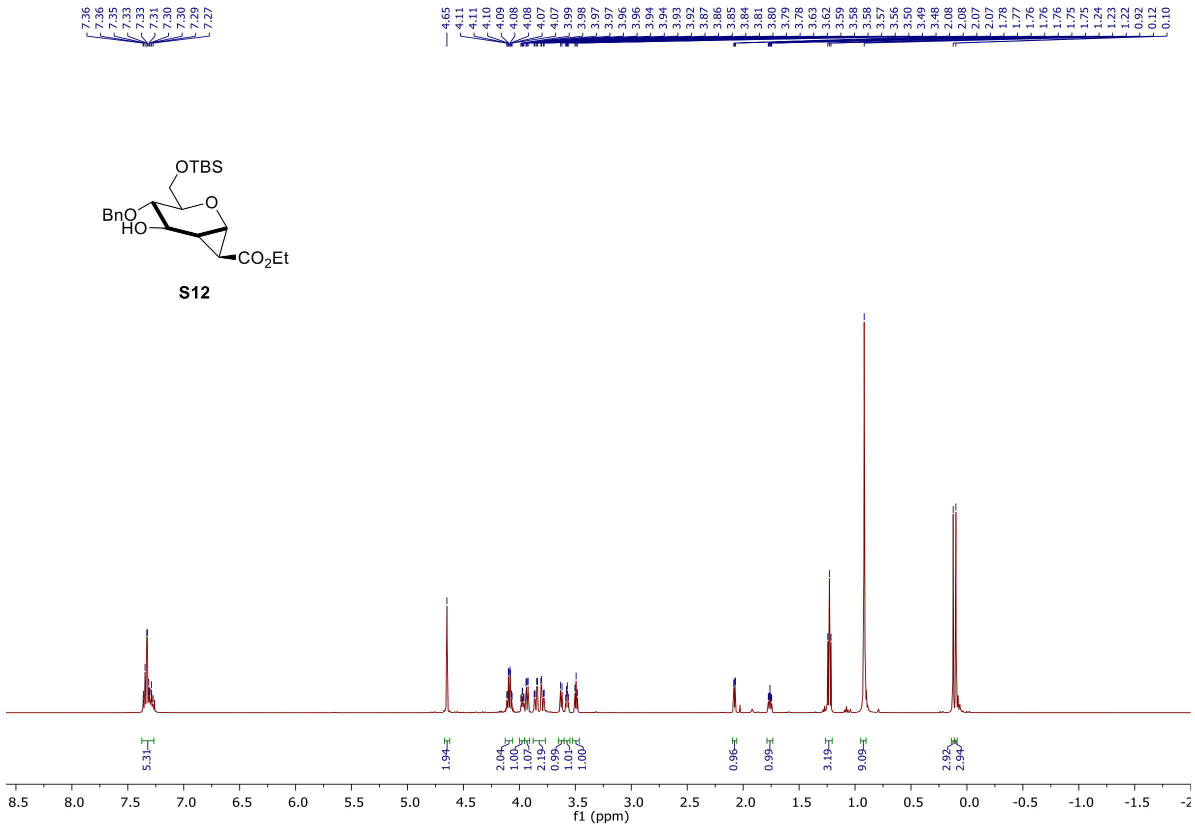

Supplementary Figure 125: ^{1}H spectra for **S12**

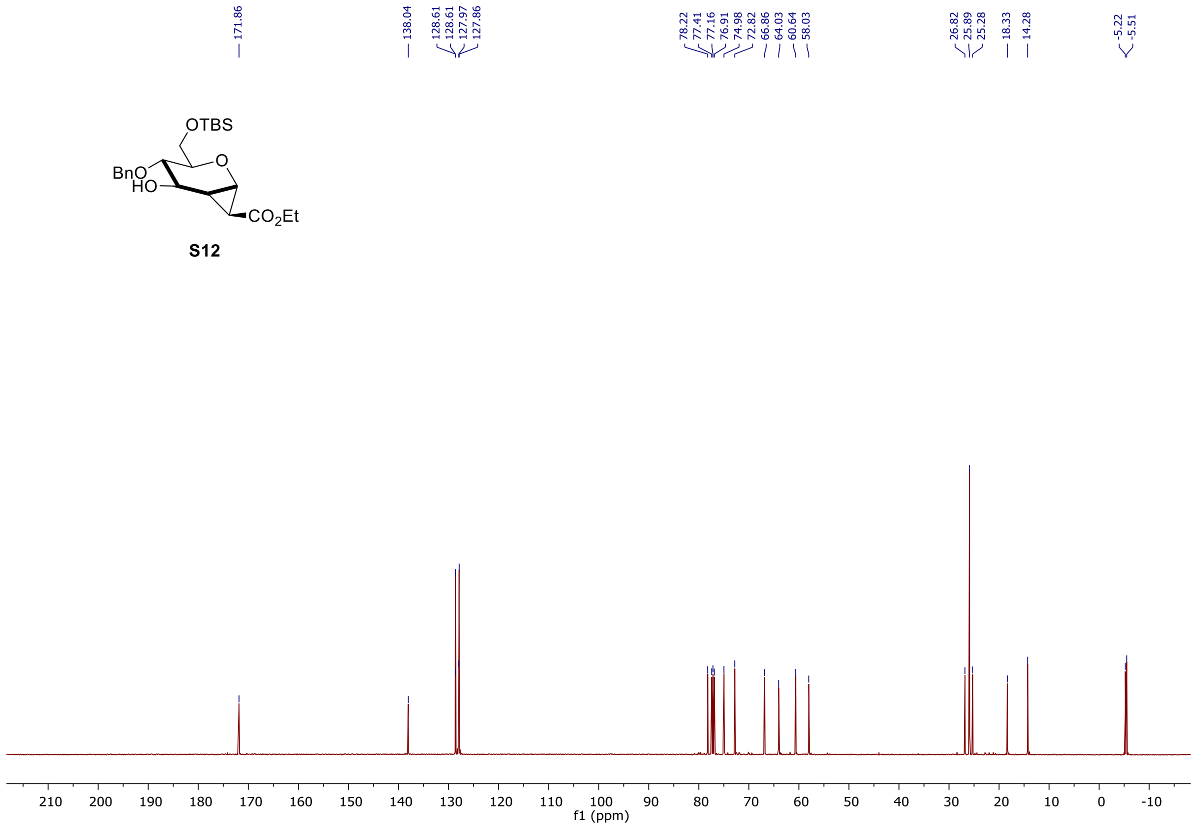

Supplementary Figure 126: ^{13}C spectra for **S12**

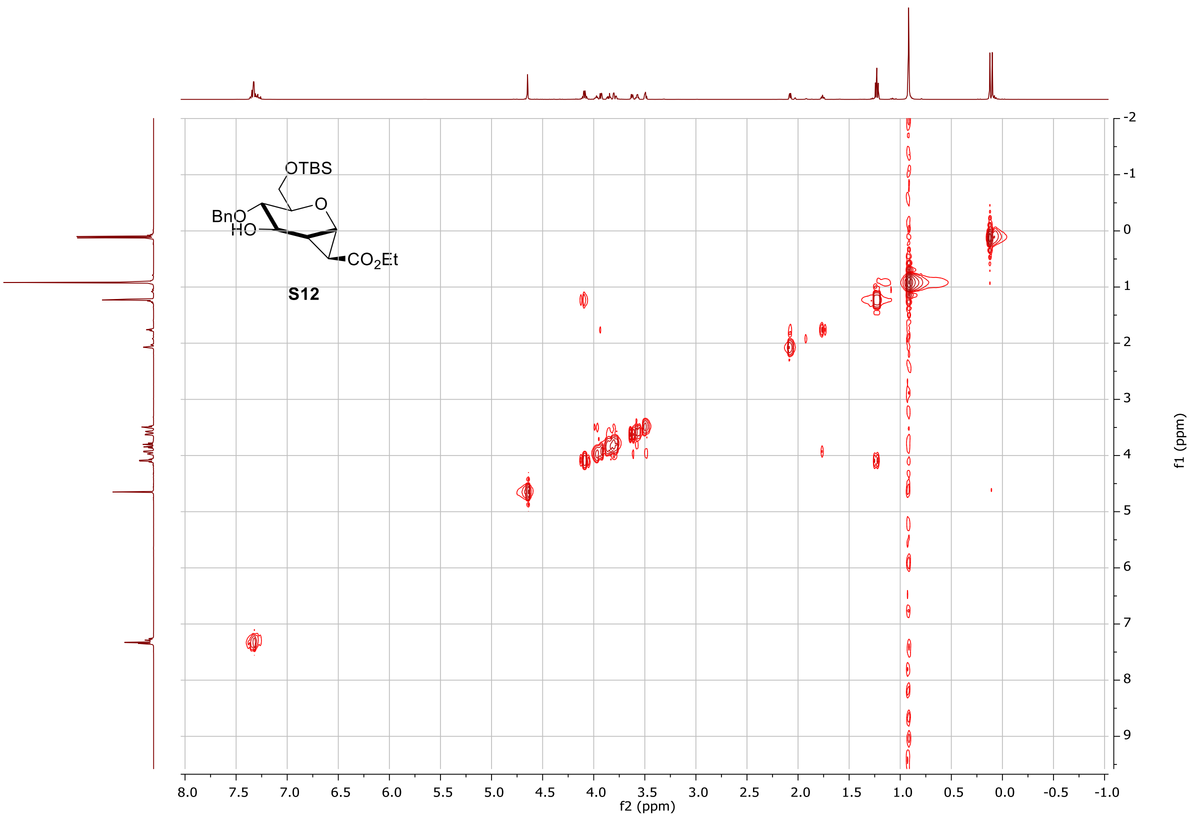

Supplementary Figure 127: COSY spectra for compound **S12**

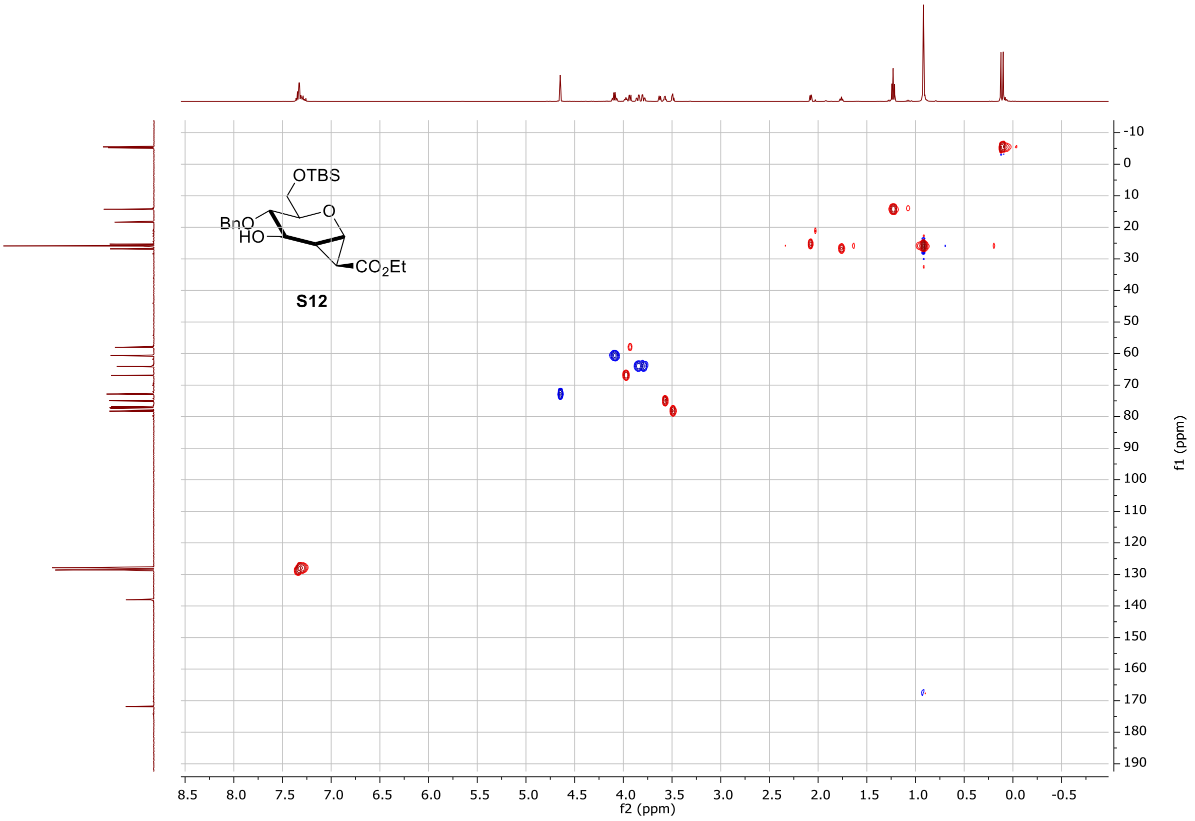

Supplementary Figure 128: HSQC spectra for compound **S12**

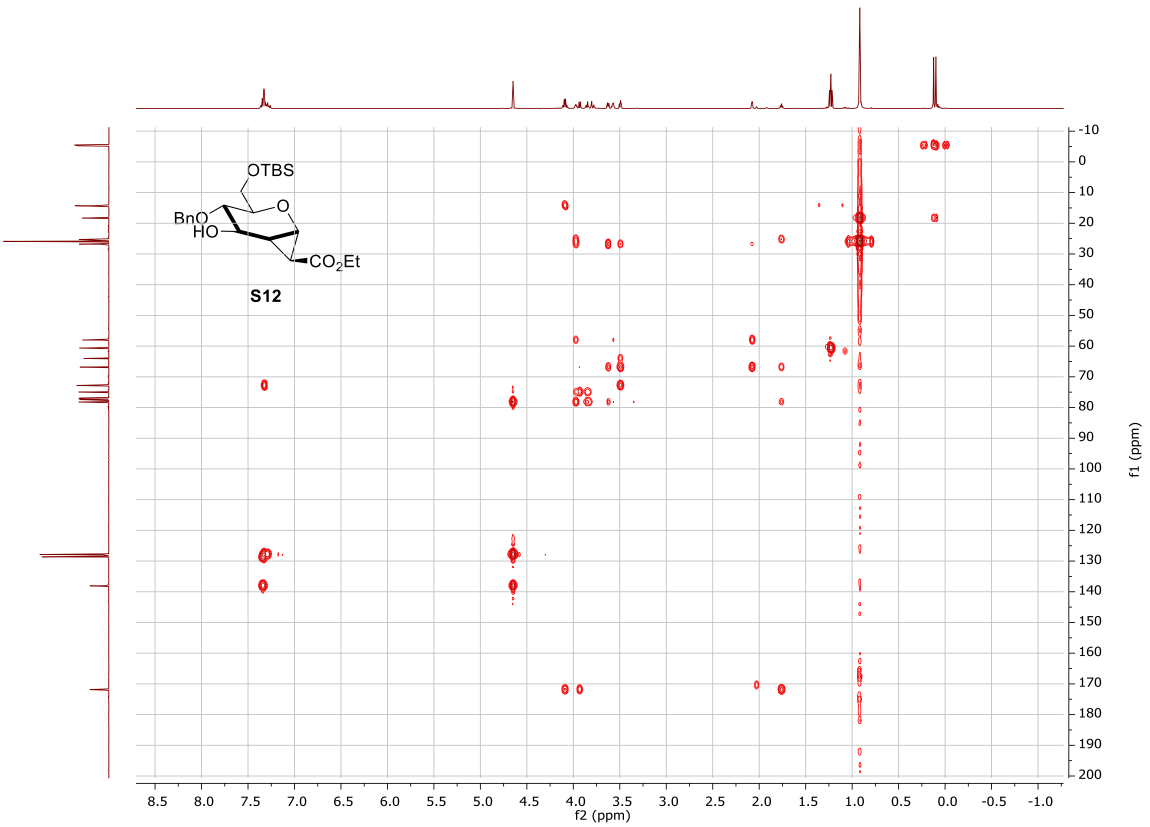

Supplementary Figure 129: HMBC spectra for compound **S12**

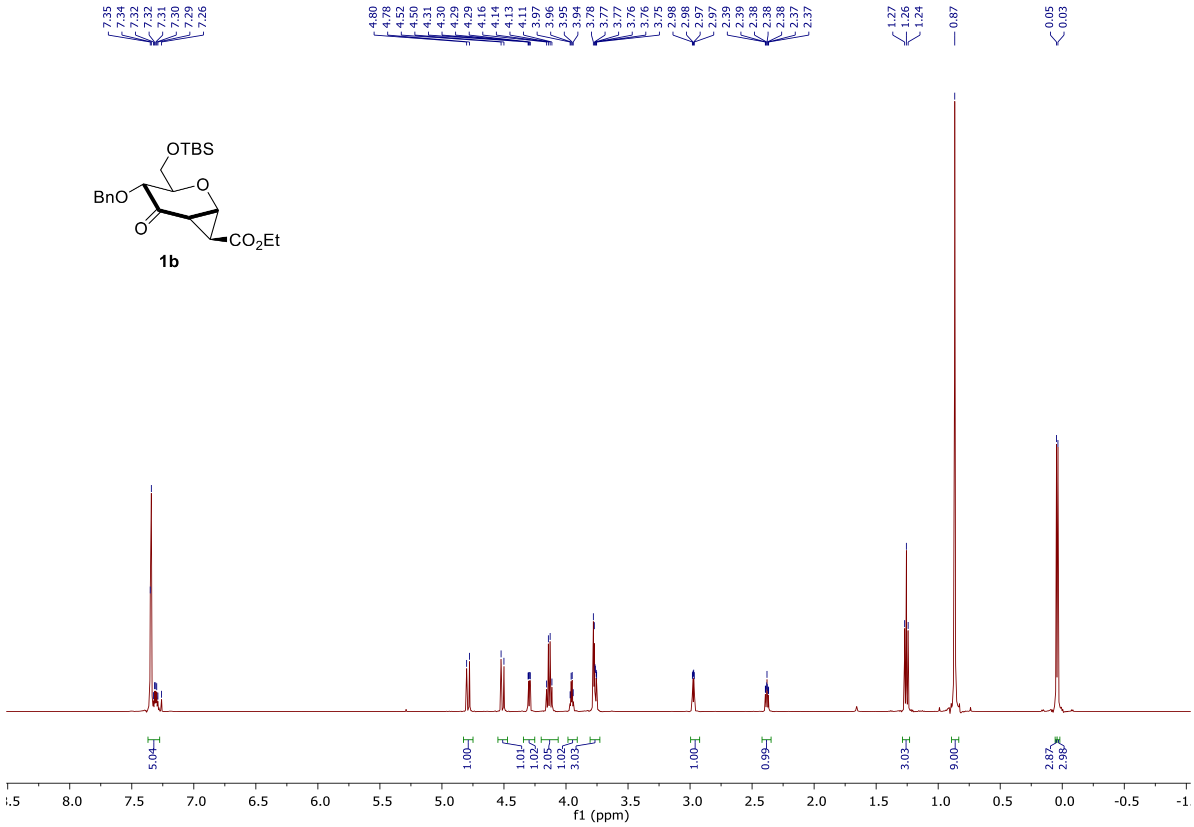

Supplementary Figure 130: ^{1}H spectra for **1b**

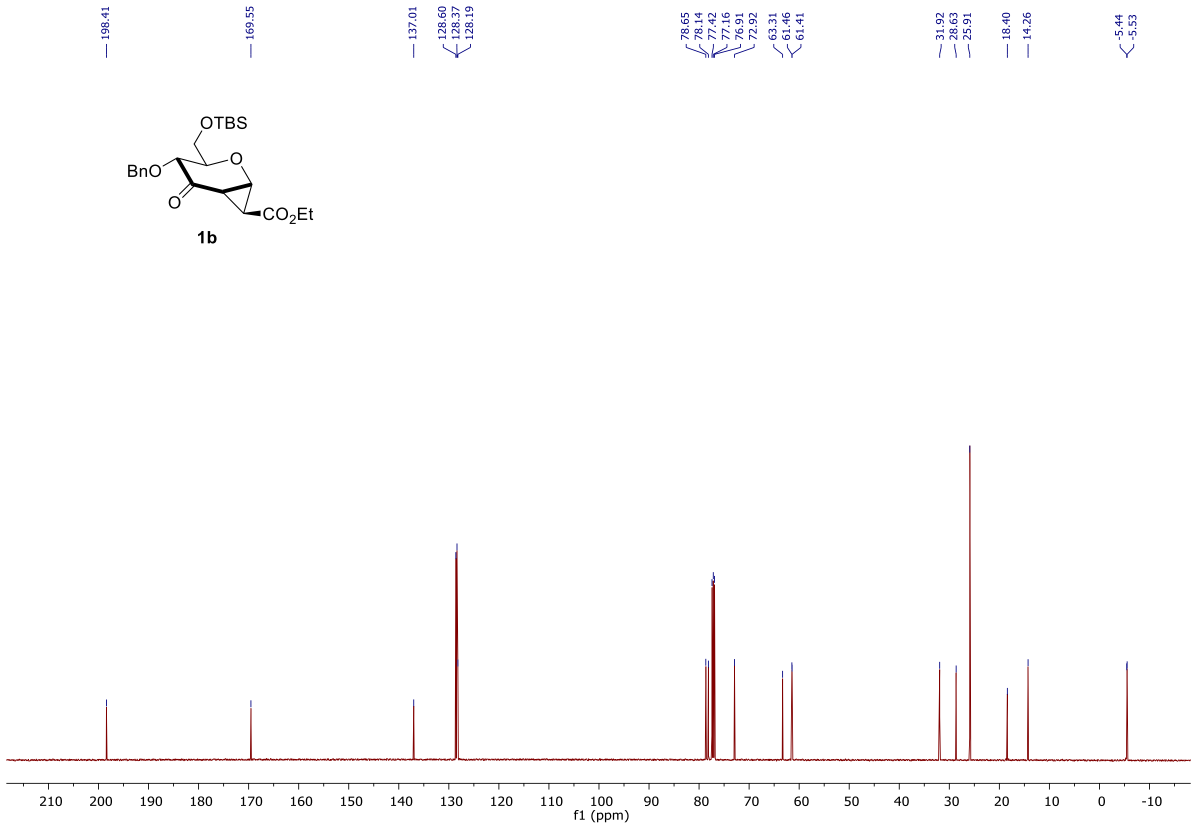

Supplementary Figure 131: ^{13}C spectra for **1b**

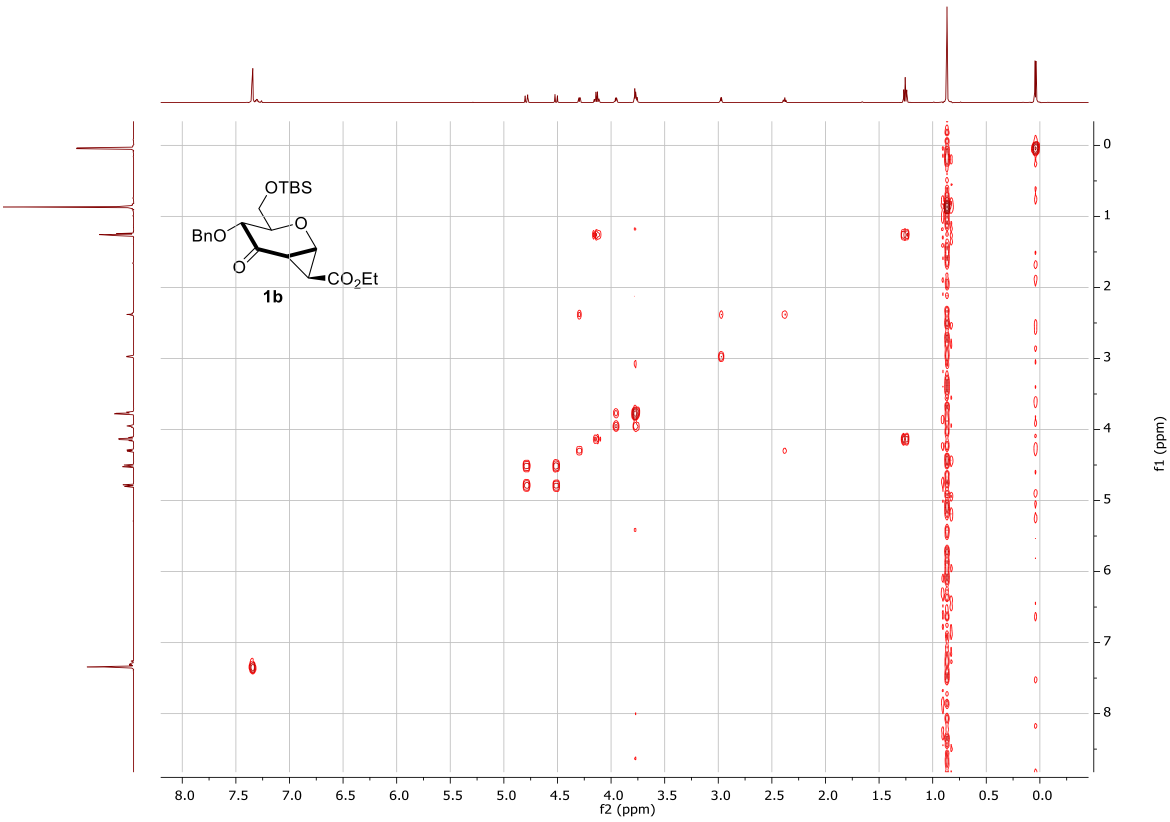

Supplementary Figure 132: COSY spectra for compound **1b**

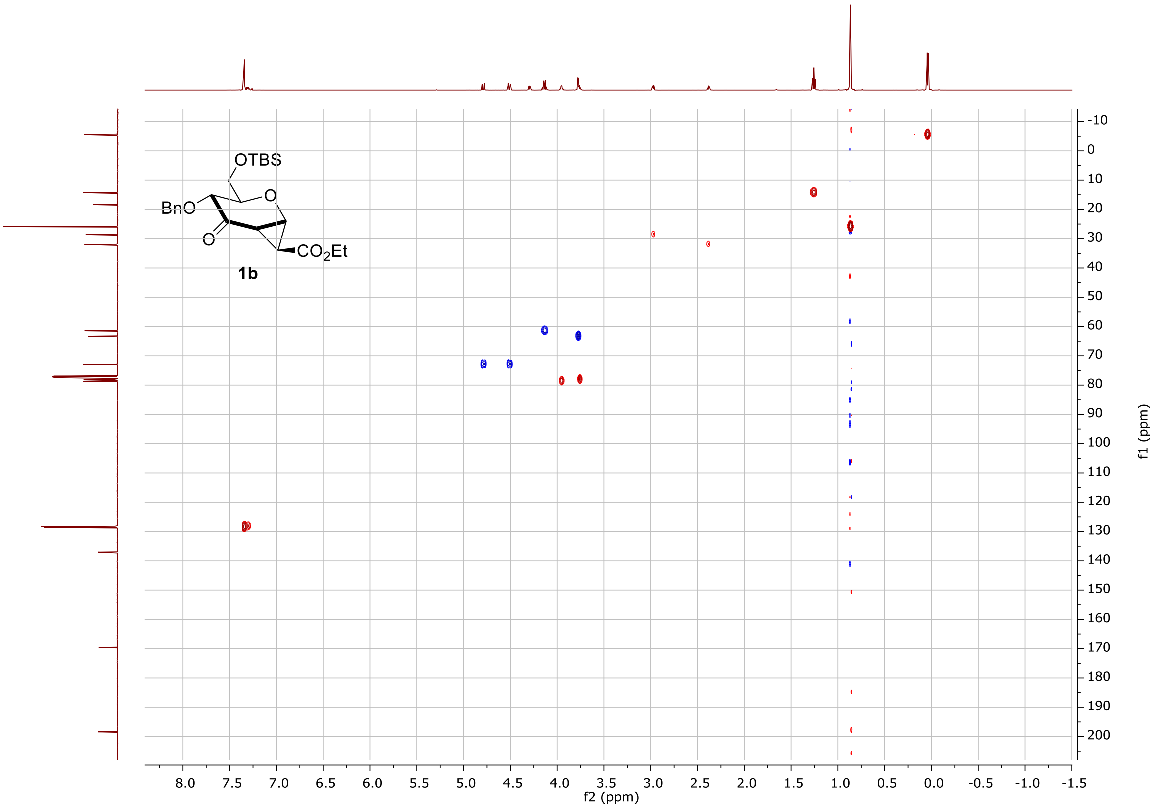

Supplementary Figure 133: HSQC spectra for compound **1b**

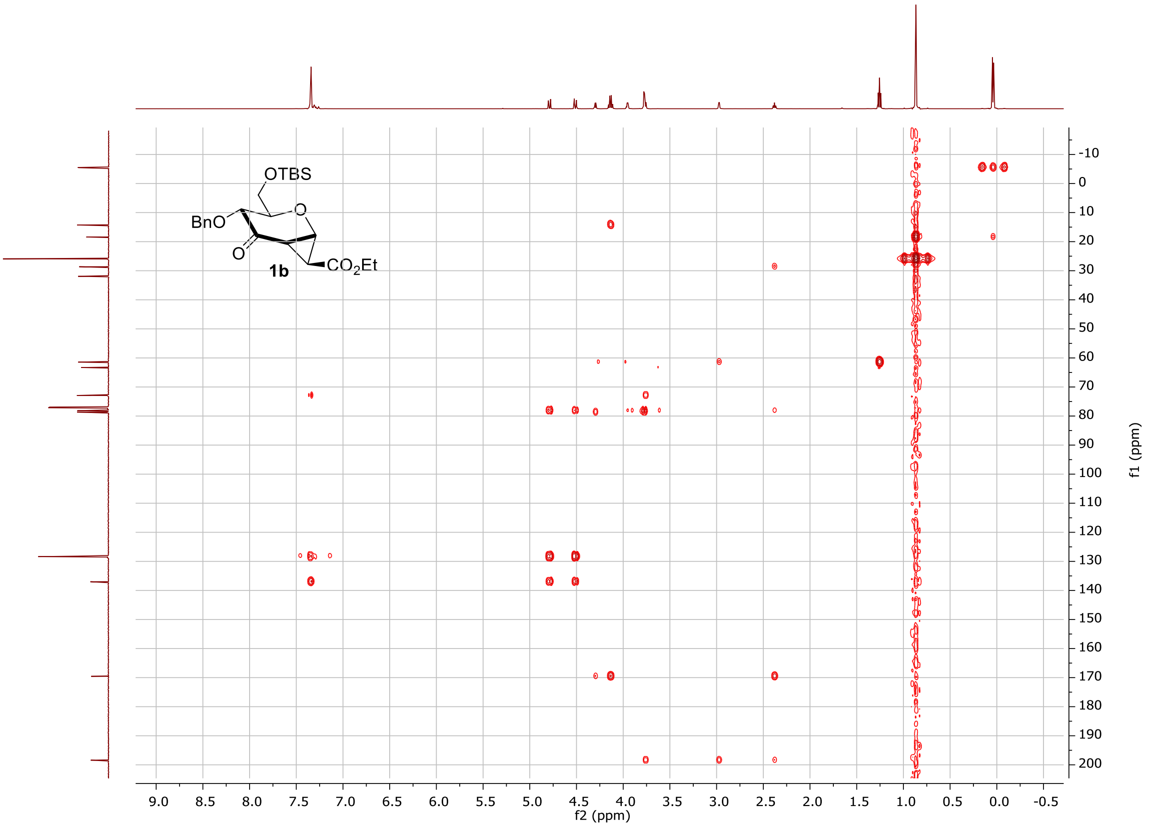

Supplementary Figure 134: HMBC spectra for compound **1b**

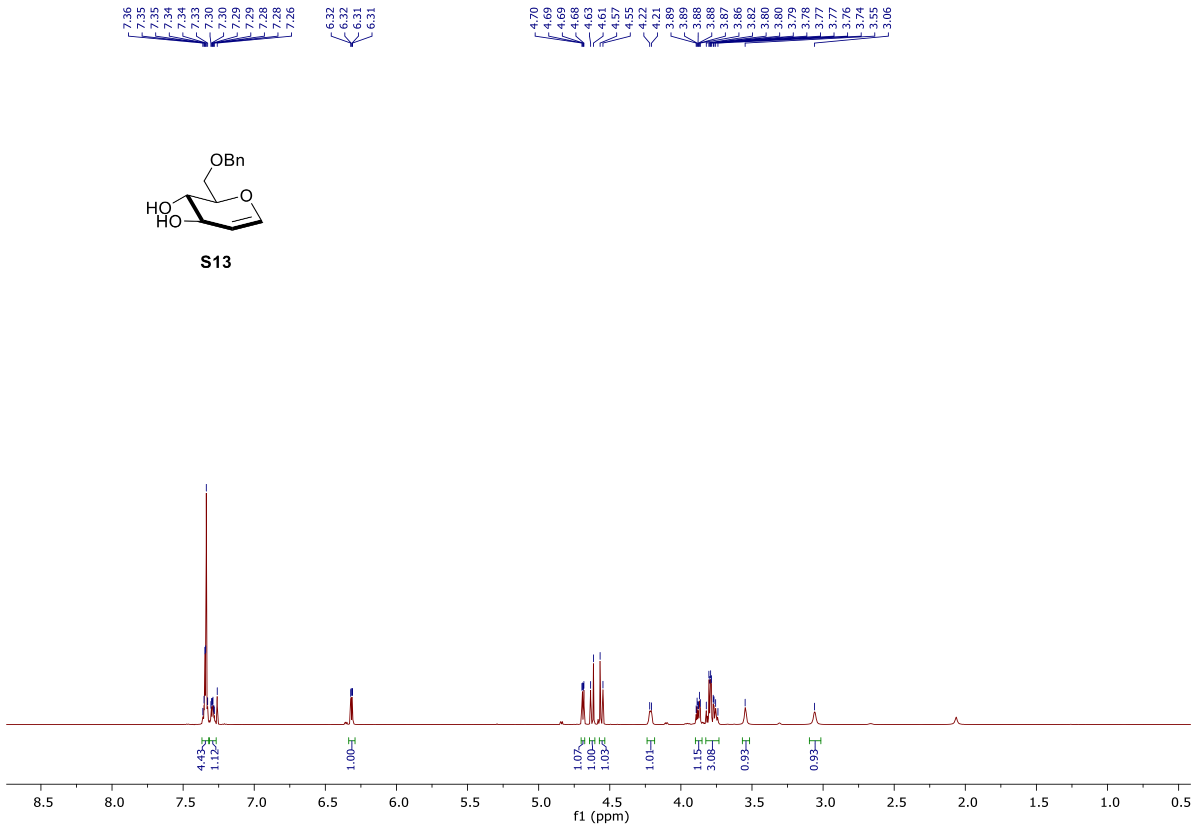

Supplementary Figure 135: ^{1}H spectra for **S13**

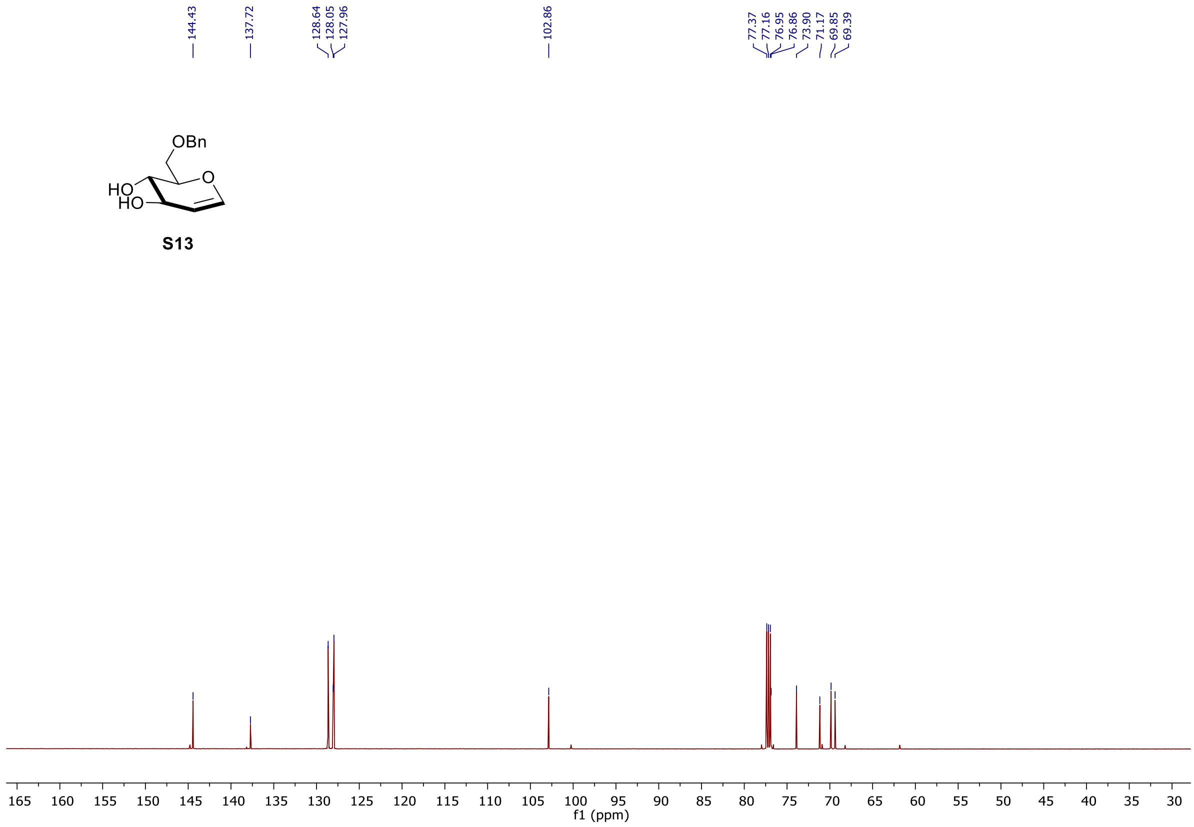

Supplementary Figure 136: ^{13}C spectra for **S13**

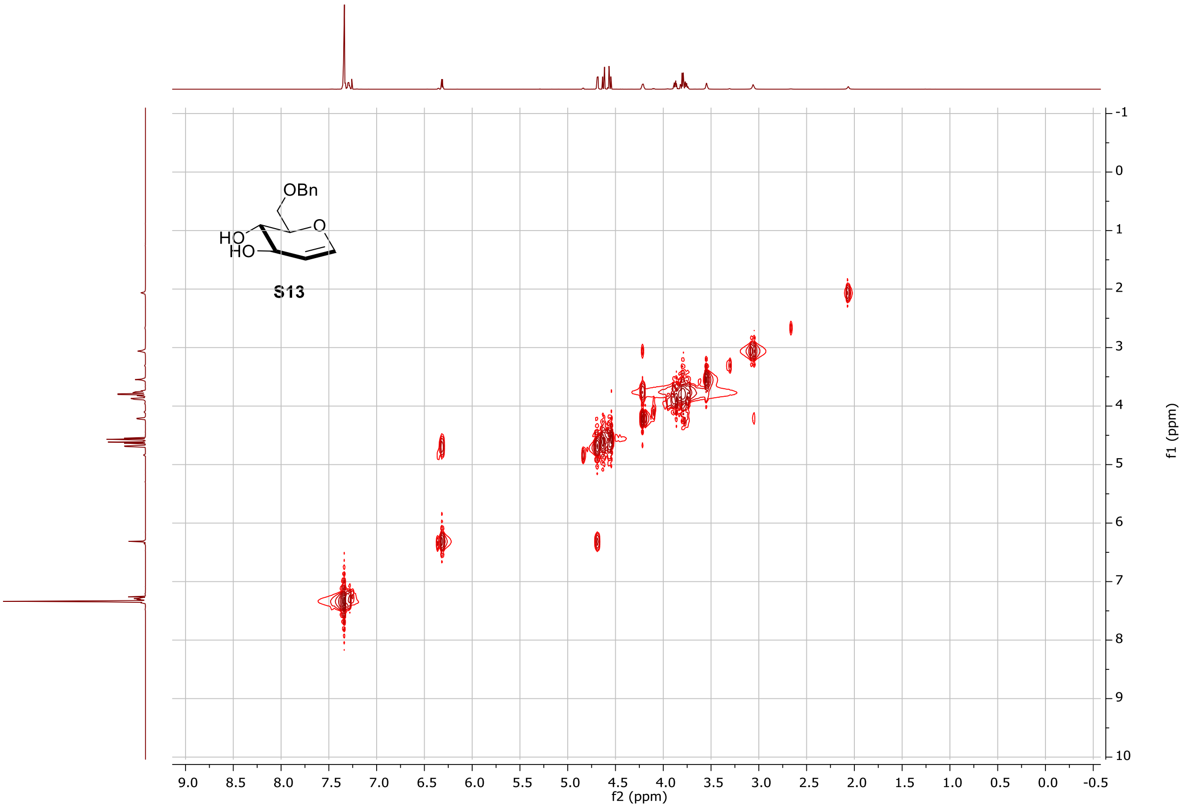

Supplementary Figure 137: COSY spectra for compound **S13**

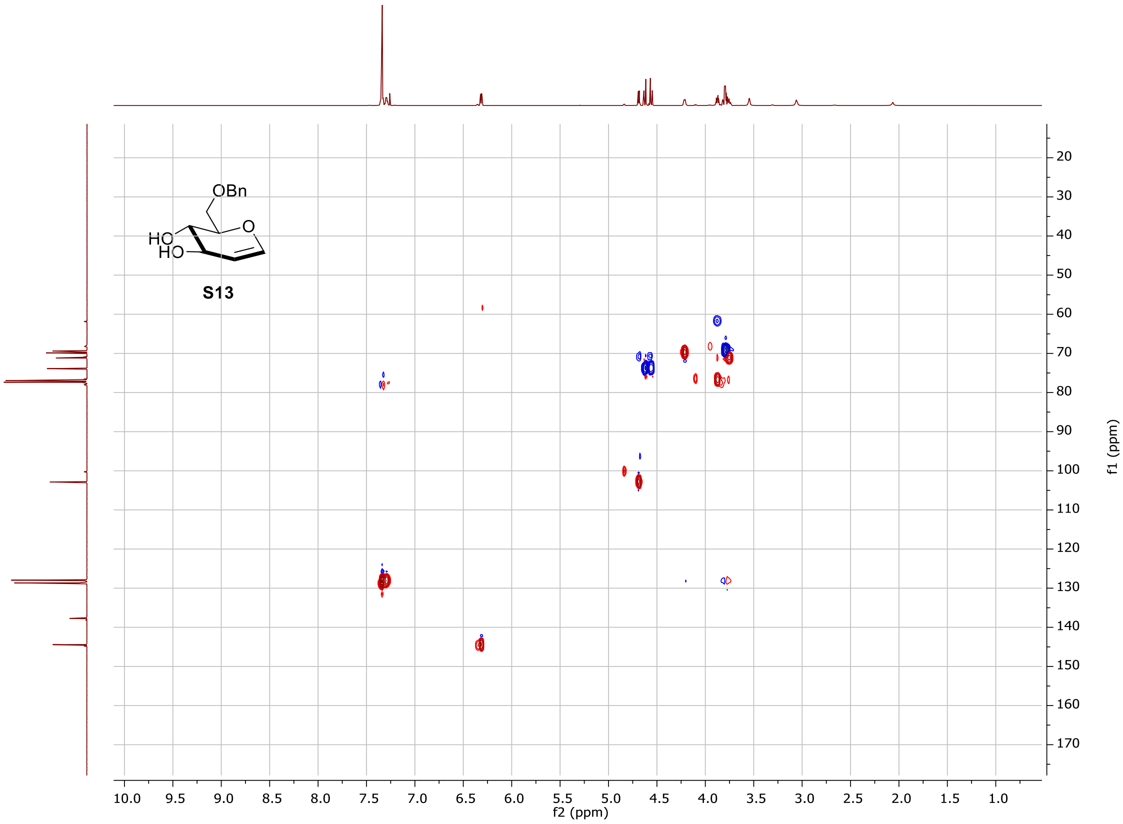

Supplementary Figure 138: HSQC spectra for compound **S13**

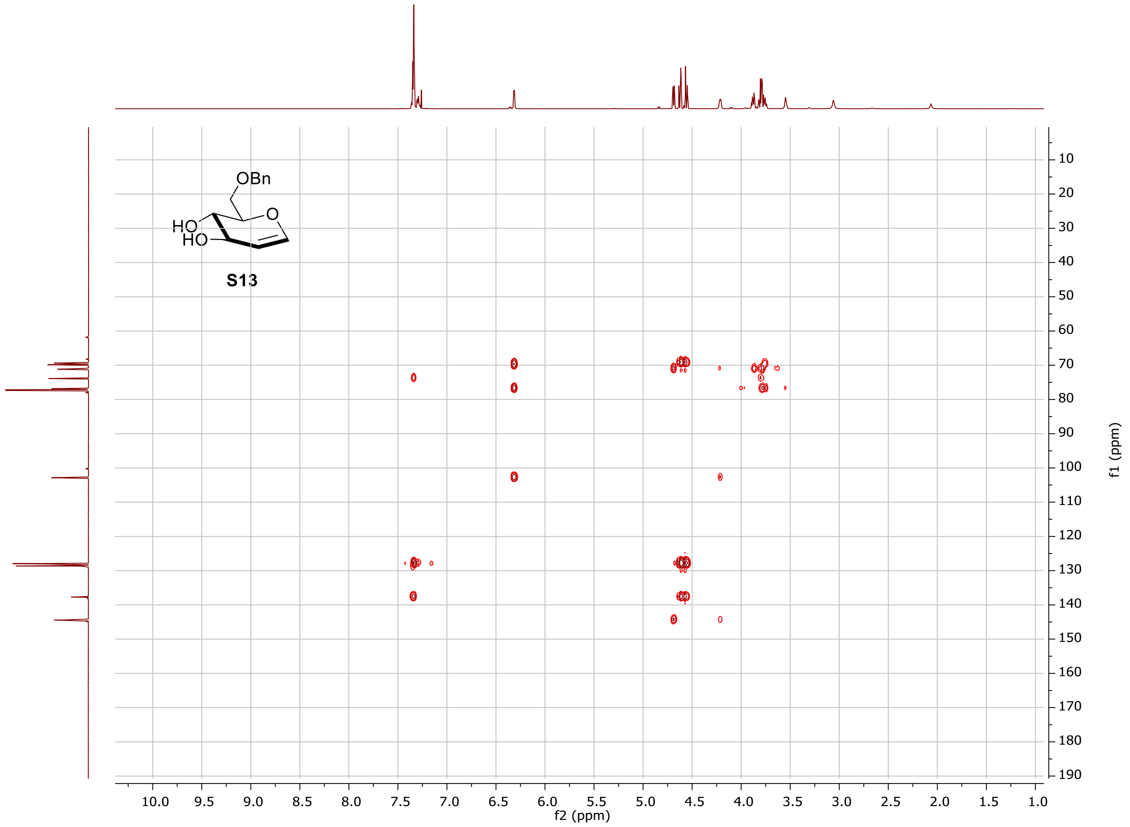

Supplementary Figure 139: HMBC spectra for compound **S13**

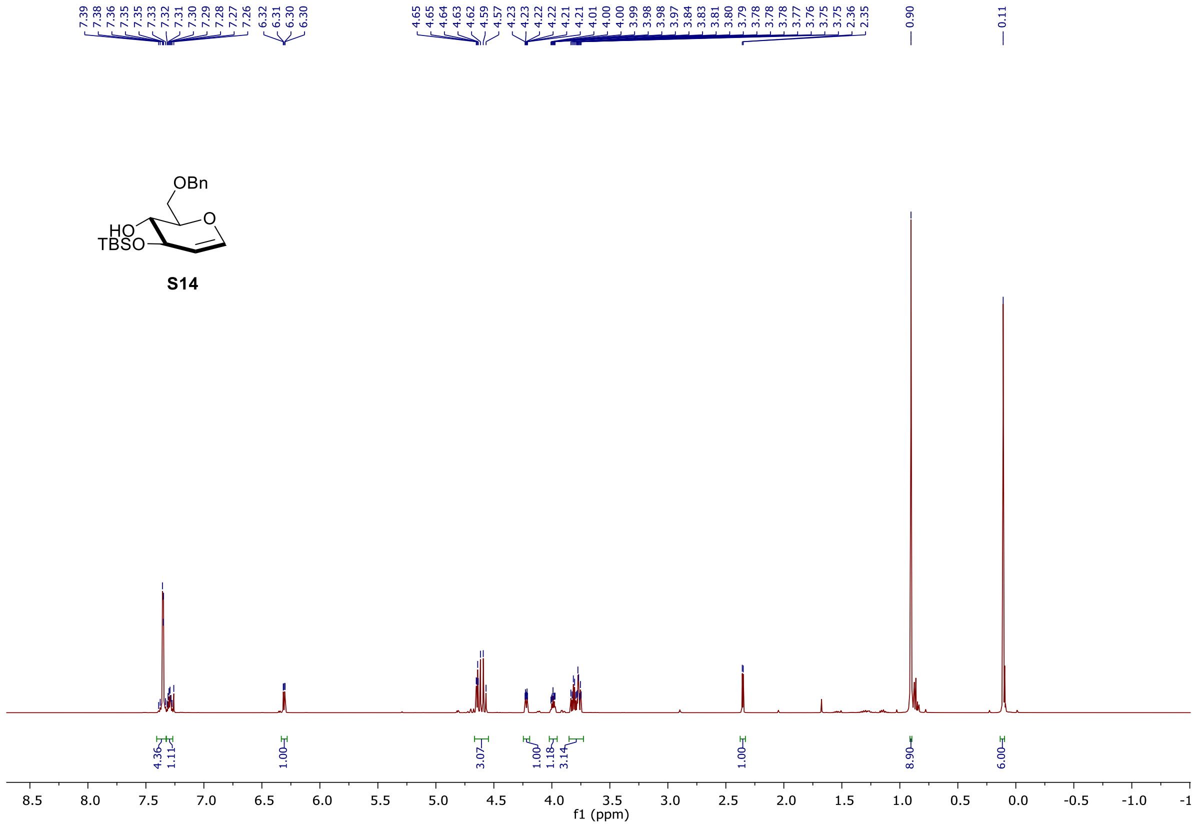

Supplementary Figure 140: ^{1}H spectra for **S14**

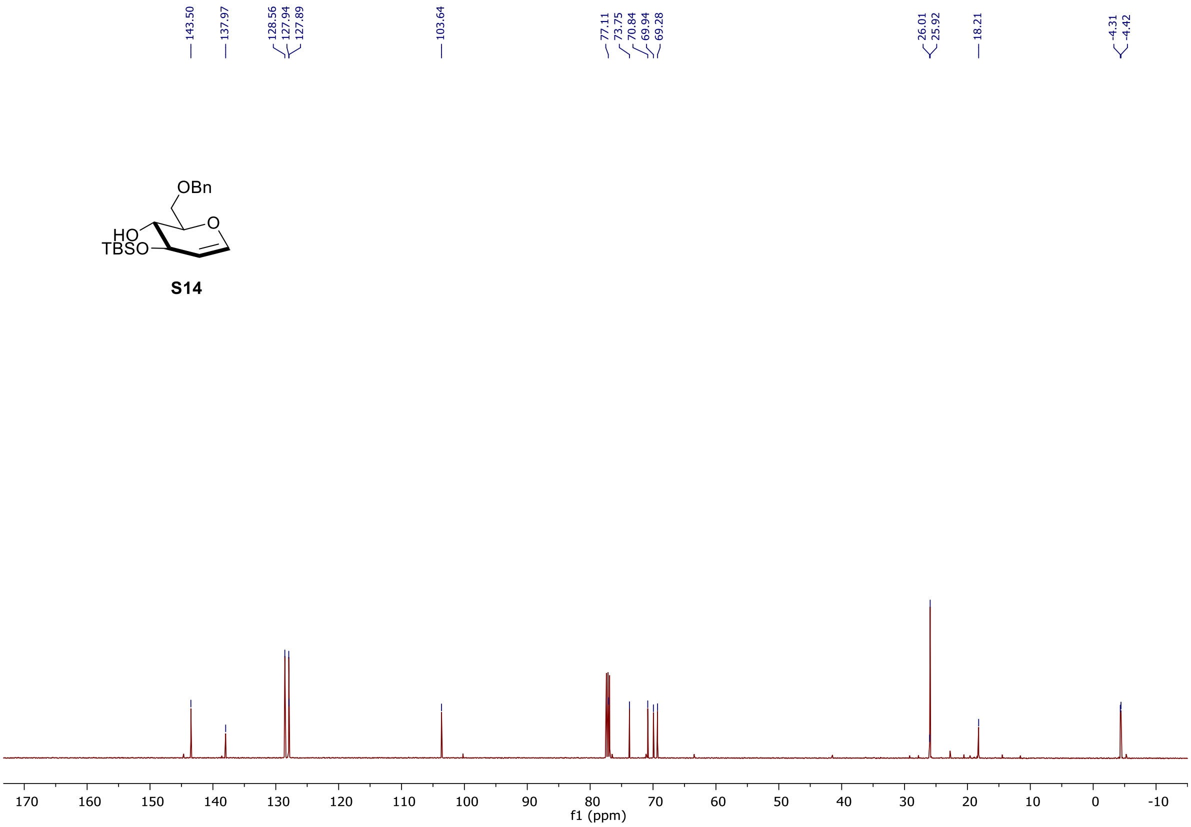

Supplementary Figure 141: ^{13}C spectra for **S14**

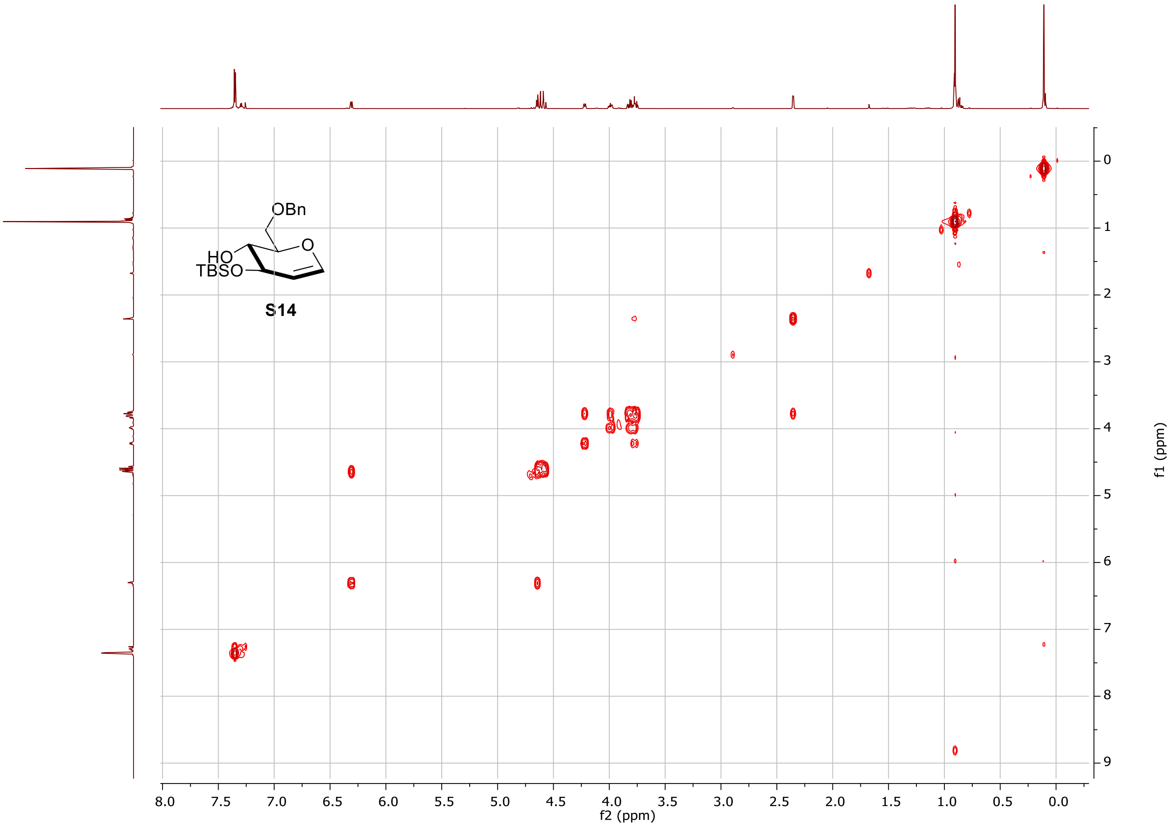

Supplementary Figure 142: COSY spectra for compound **S14**

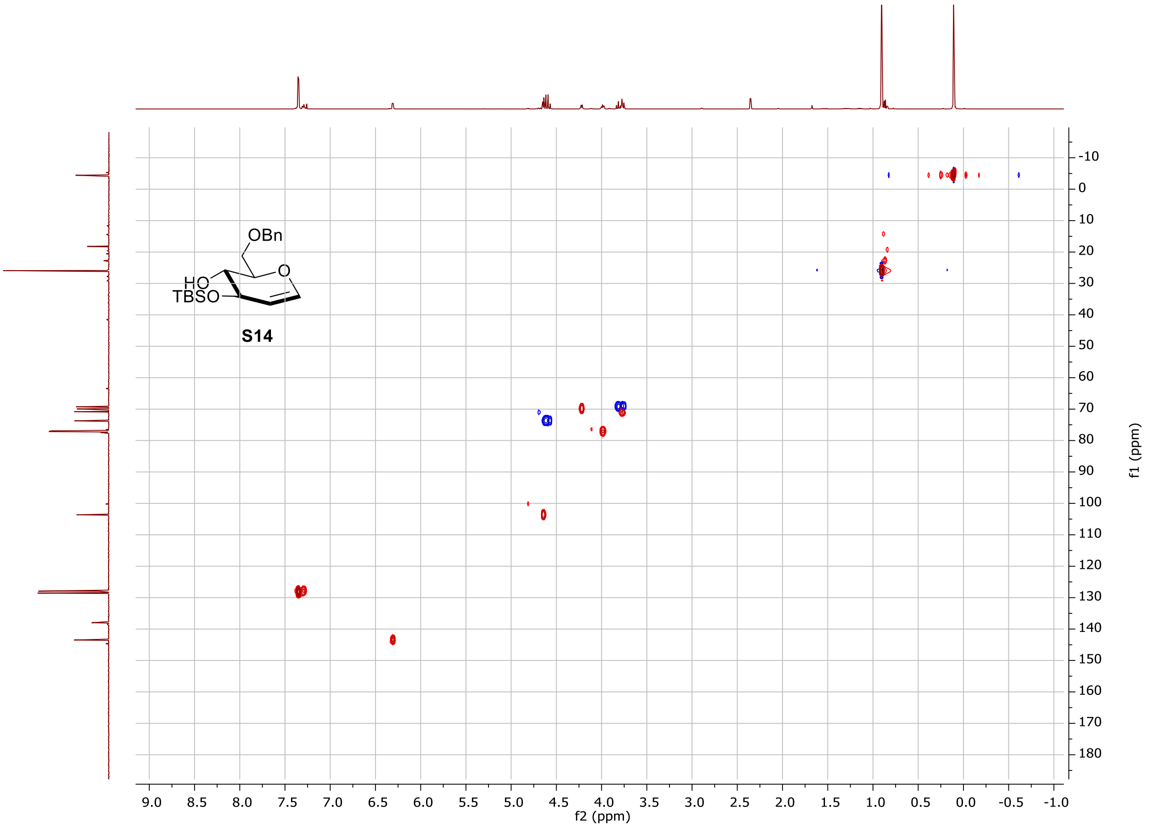

Supplementary Figure 143: HSQC spectra for compound **S14**

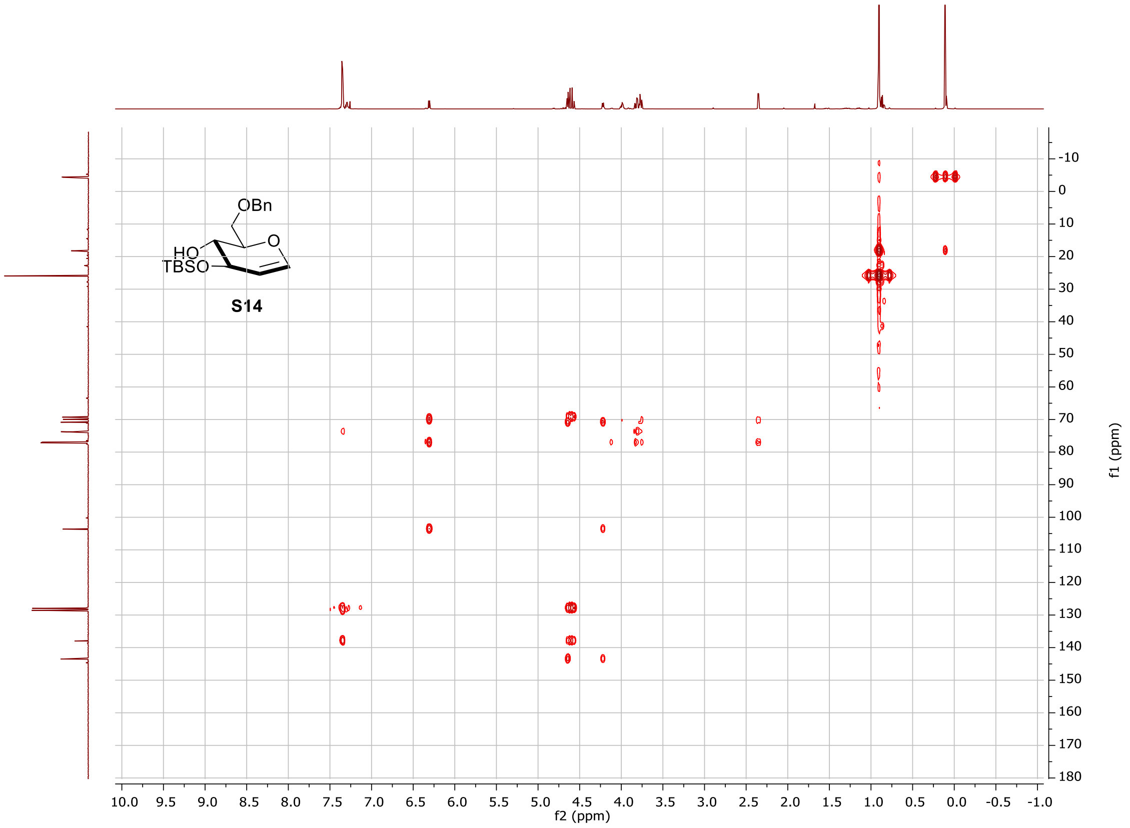

Supplementary Figure 144: HMBC spectra for compound **S14**

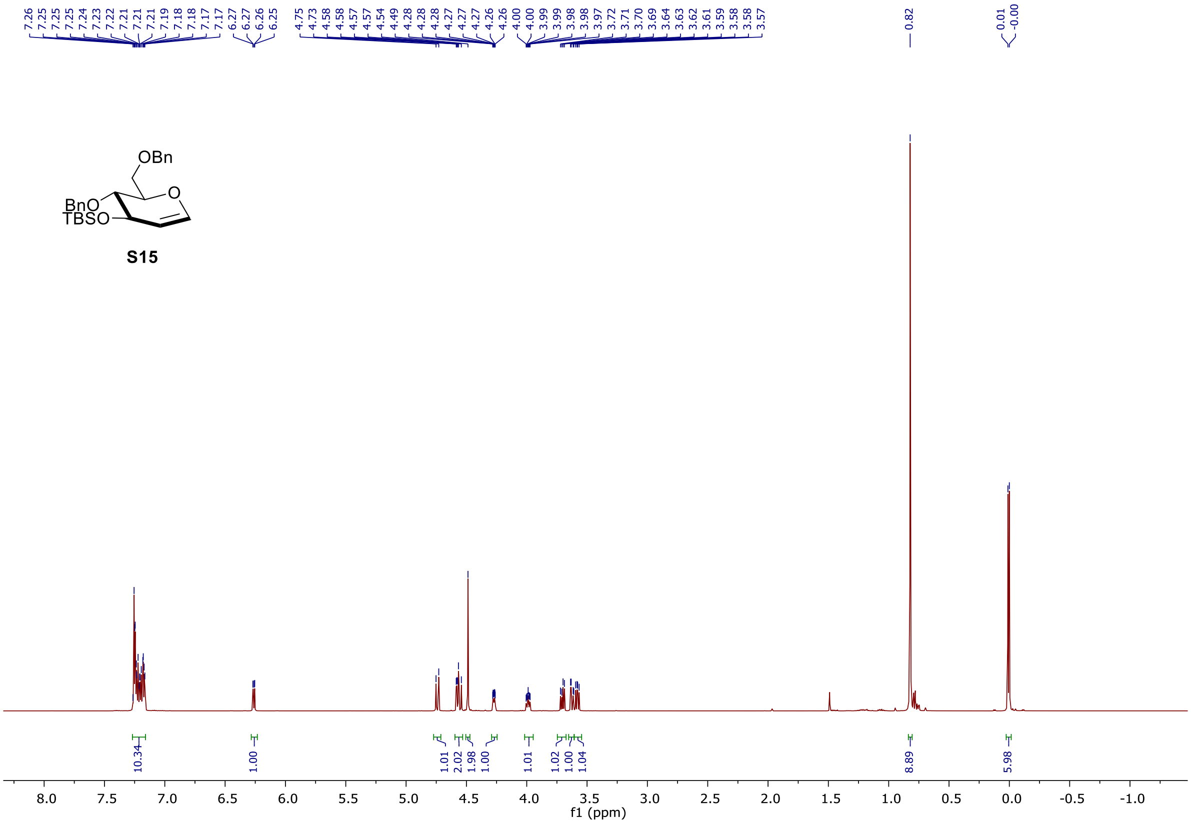

Supplementary Figure 145: ^{1}H spectra for **S15**

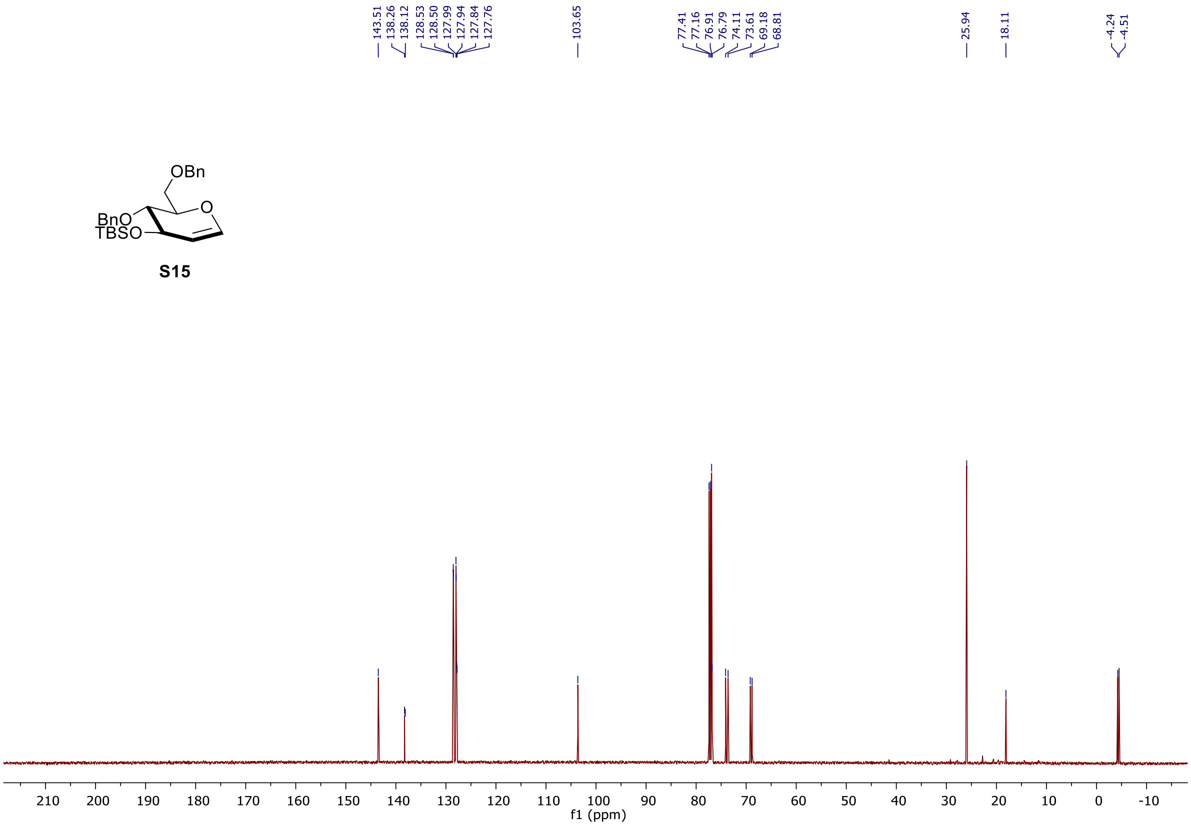

Supplementary Figure 146: ^{13}C spectra for **S15**

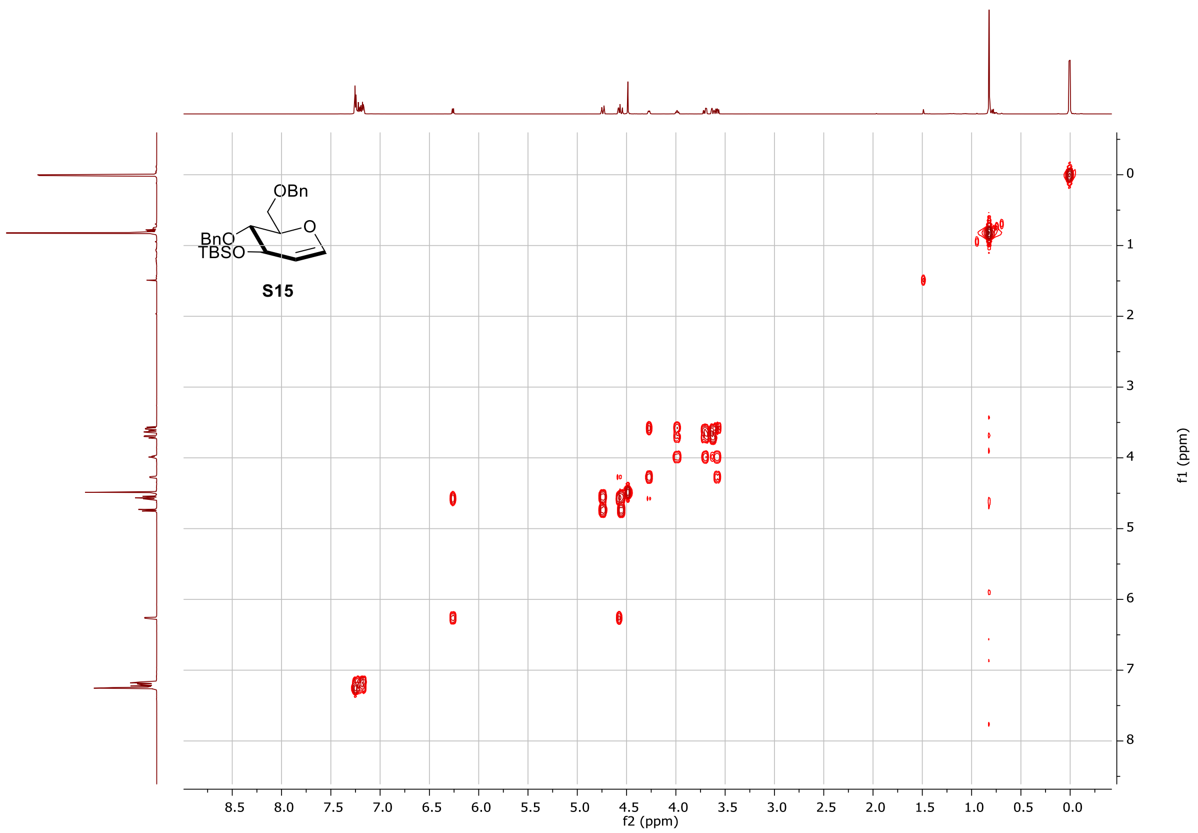

Supplementary Figure 147: COSY spectra for compound **S15**

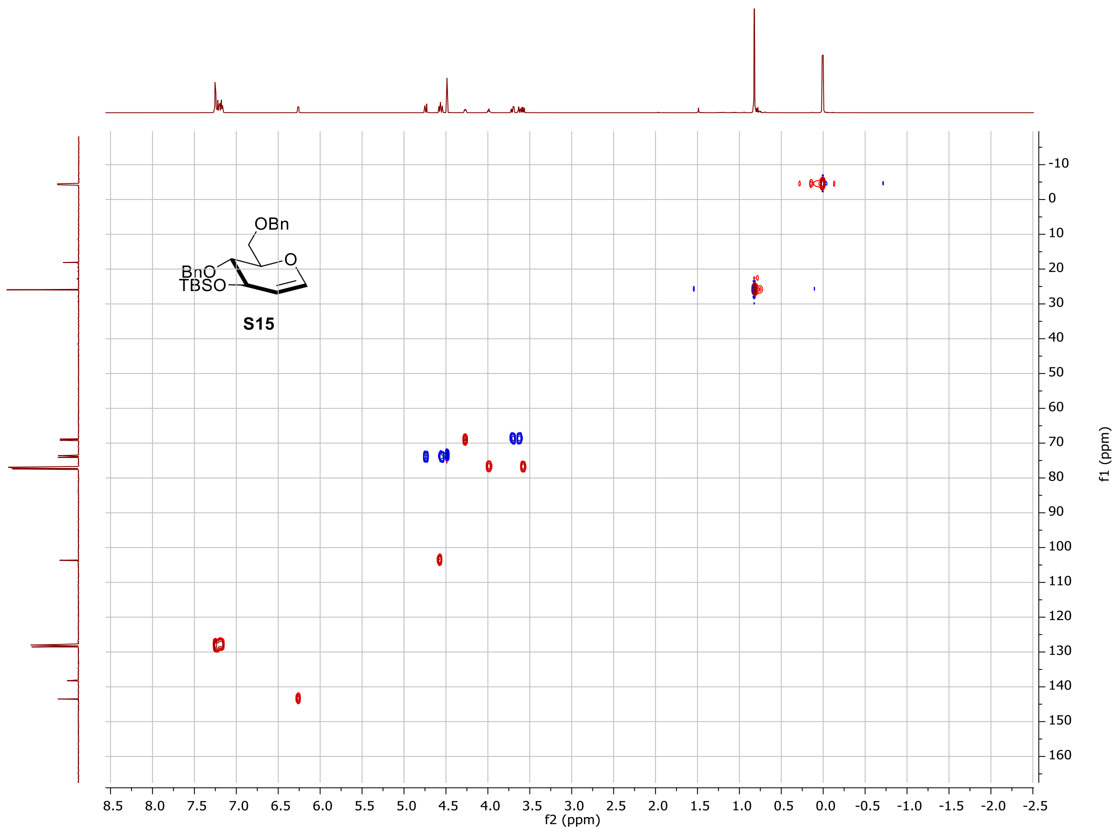

Supplementary Figure 148: HSQC spectra for compound **S15**

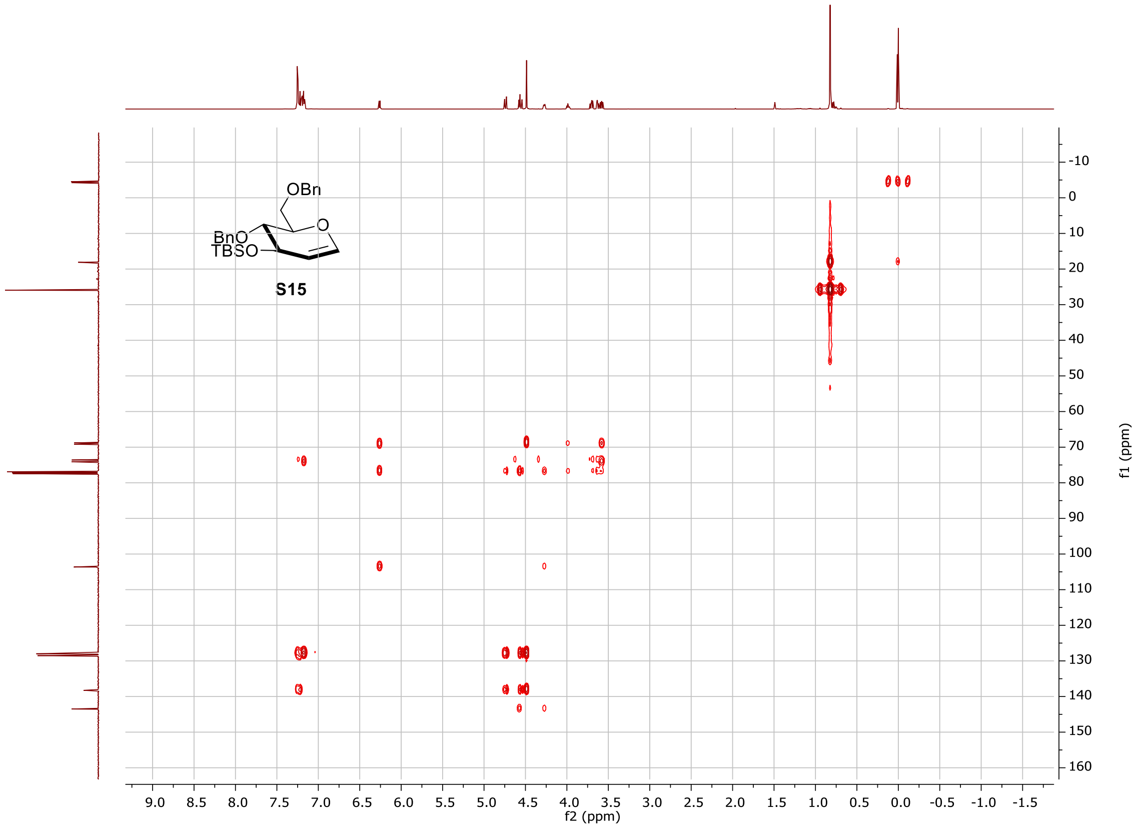

Supplementary Figure 149: HMBC spectra for compound **S15**

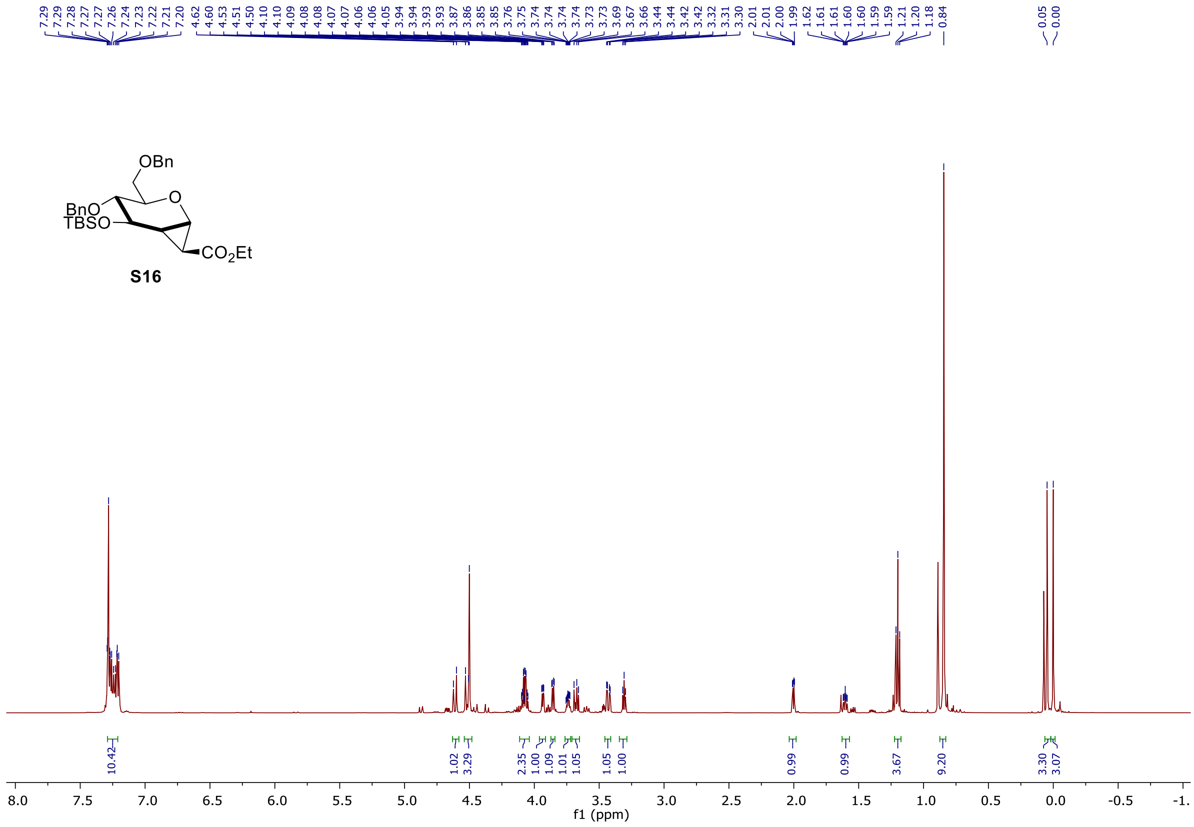

Supplementary Figure 150: ^{1}H spectra for **S16**

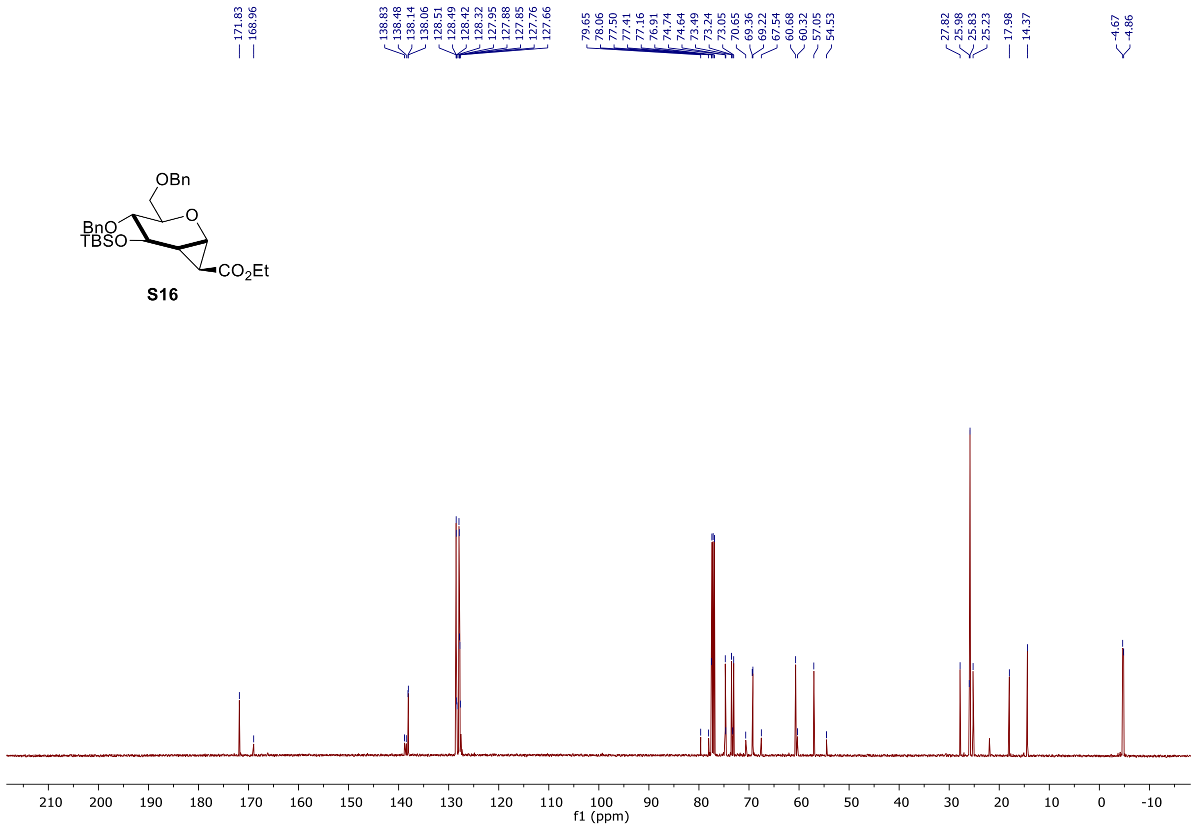

Supplementary Figure 151: ^{13}C spectra for **S16**

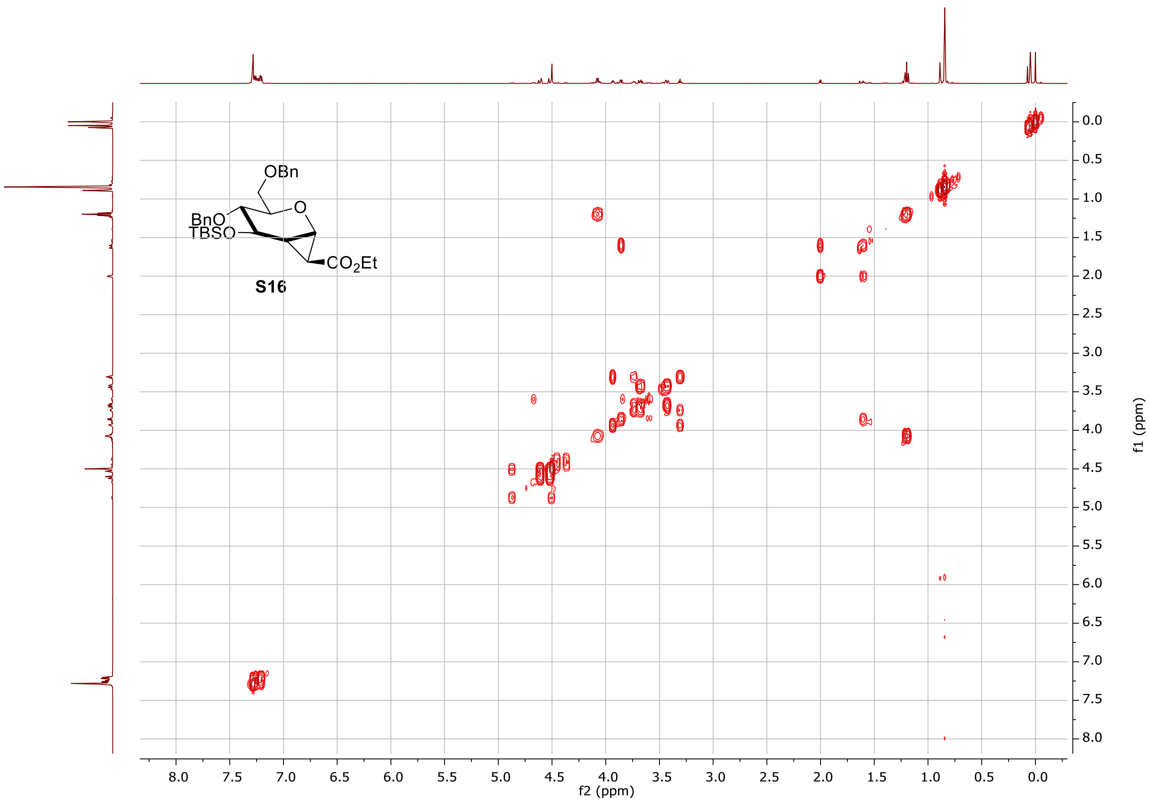

Supplementary Figure 152: COSY spectra for compound **S16**

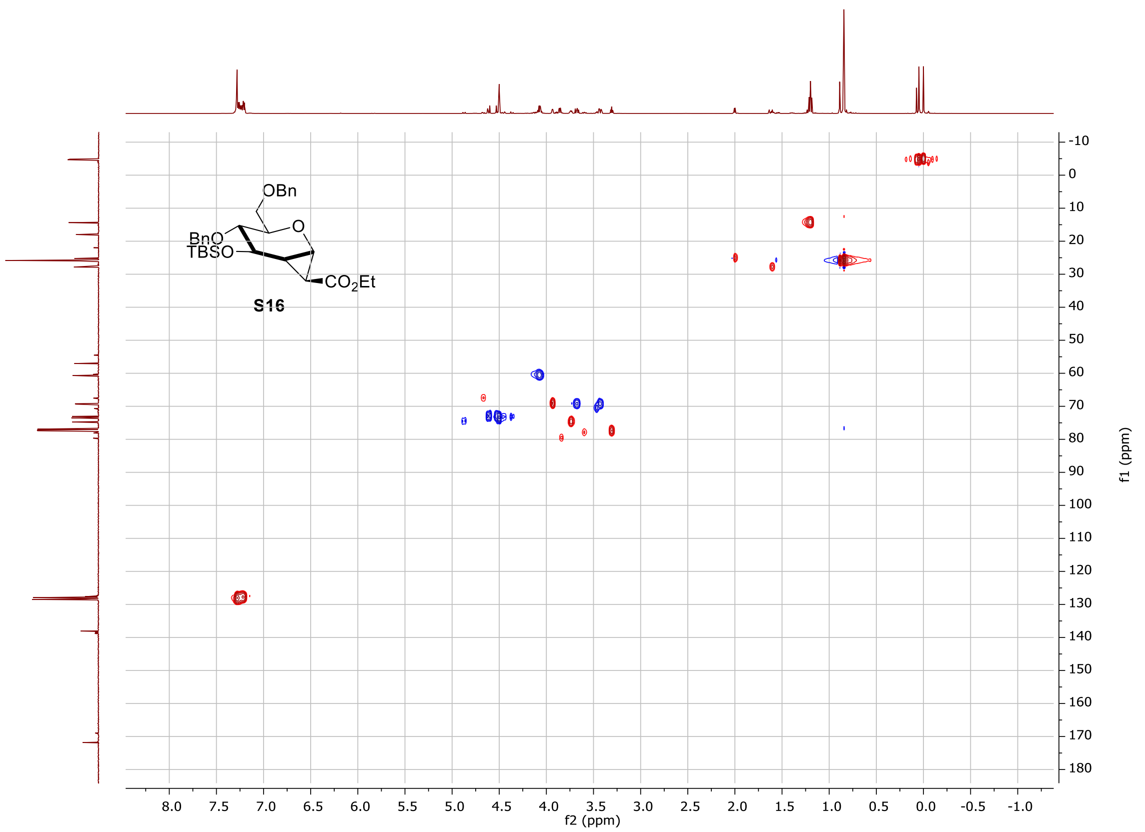

Supplementary Figure 153: HSQC spectra for compound **S16**

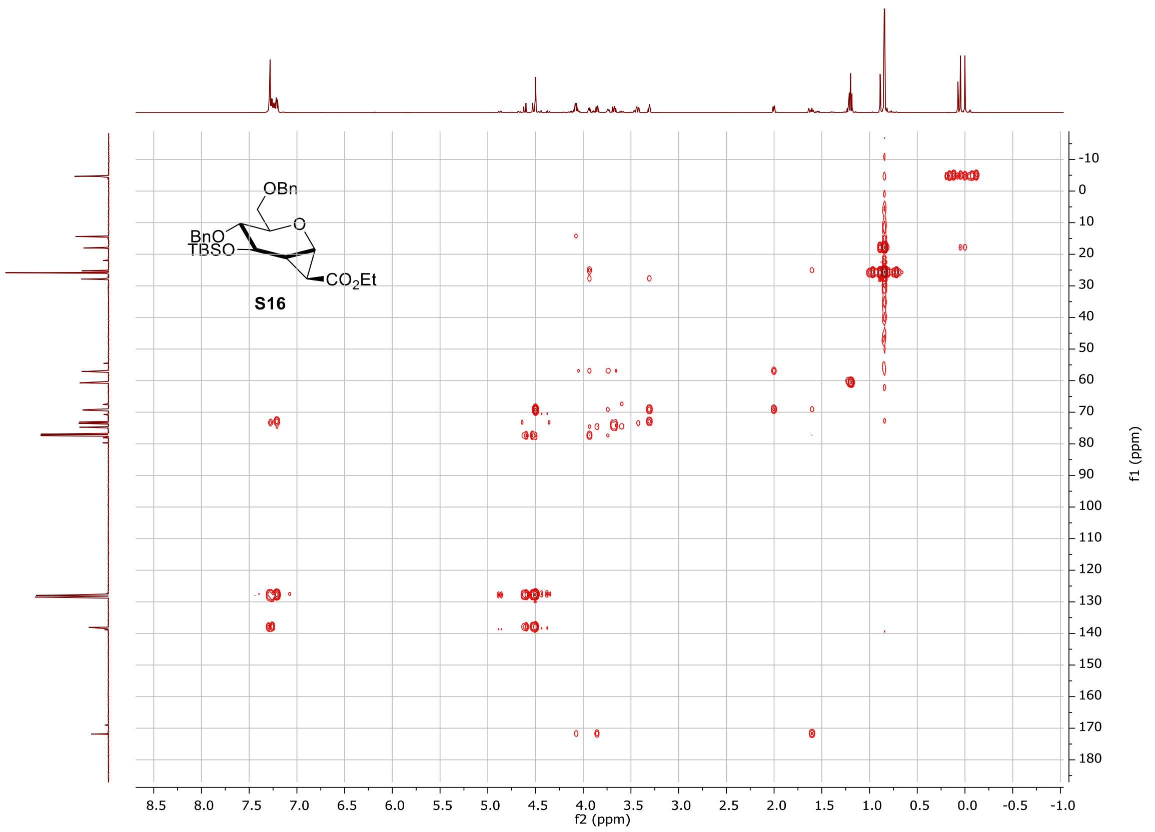

Supplementary Figure 154: HMBC spectra for compound **S16**

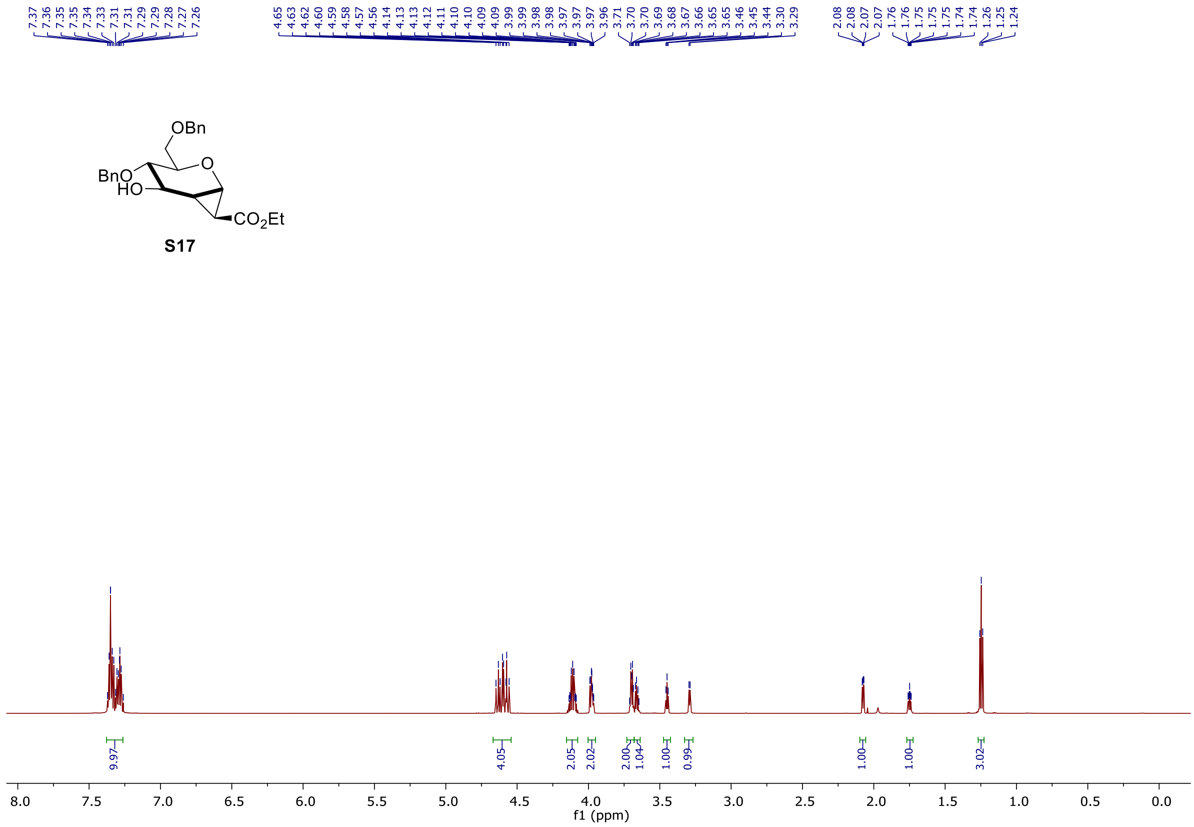

Supplementary Figure 155: ^{1}H spectra for **S17**

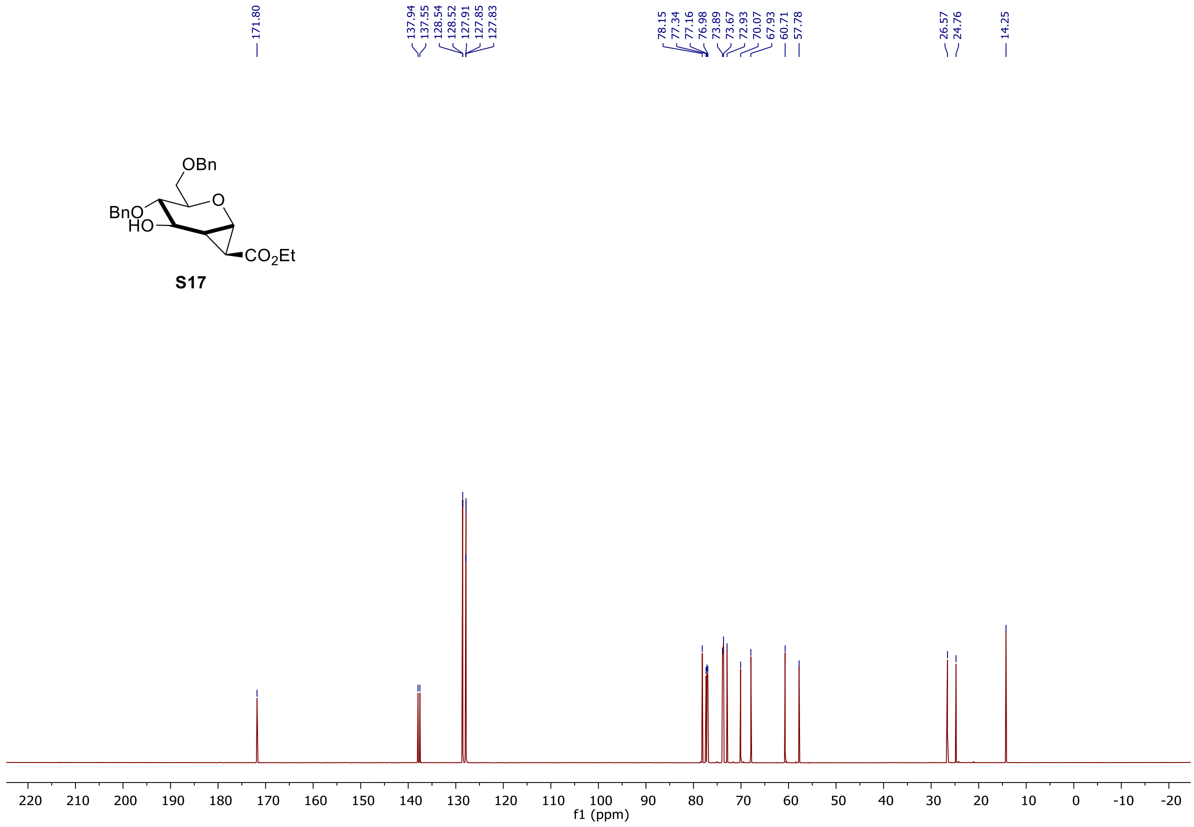

Supplementary Figure 156: ^{13}C spectra for **S17**

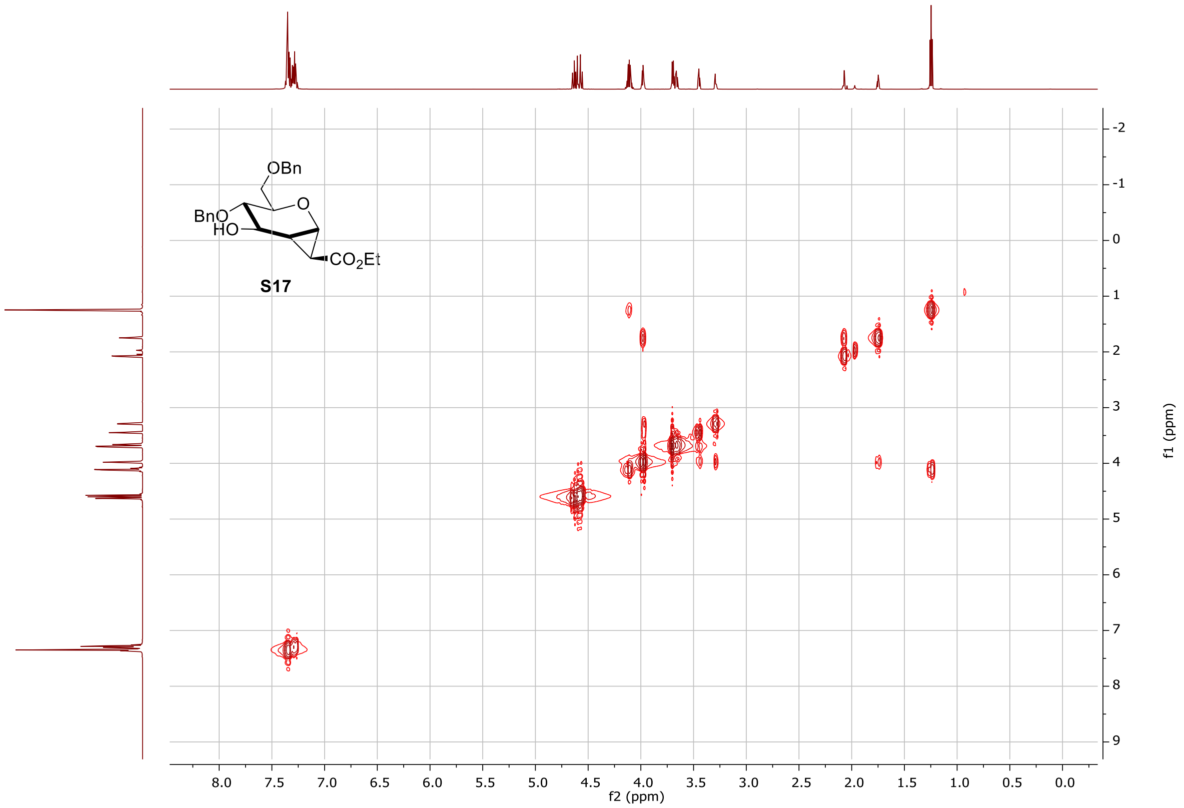

Supplementary Figure 157: COSY spectra for compound **S17**

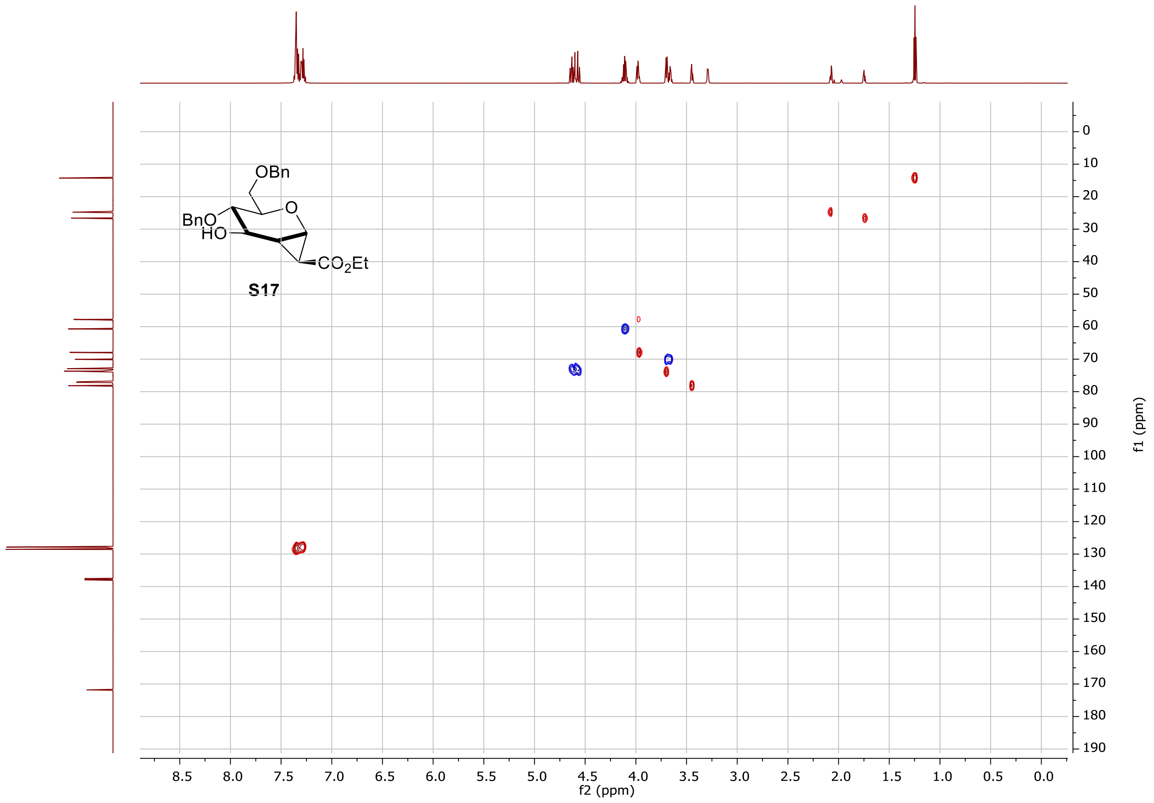

Supplementary Figure 158: HSQC spectra for compound **S17**

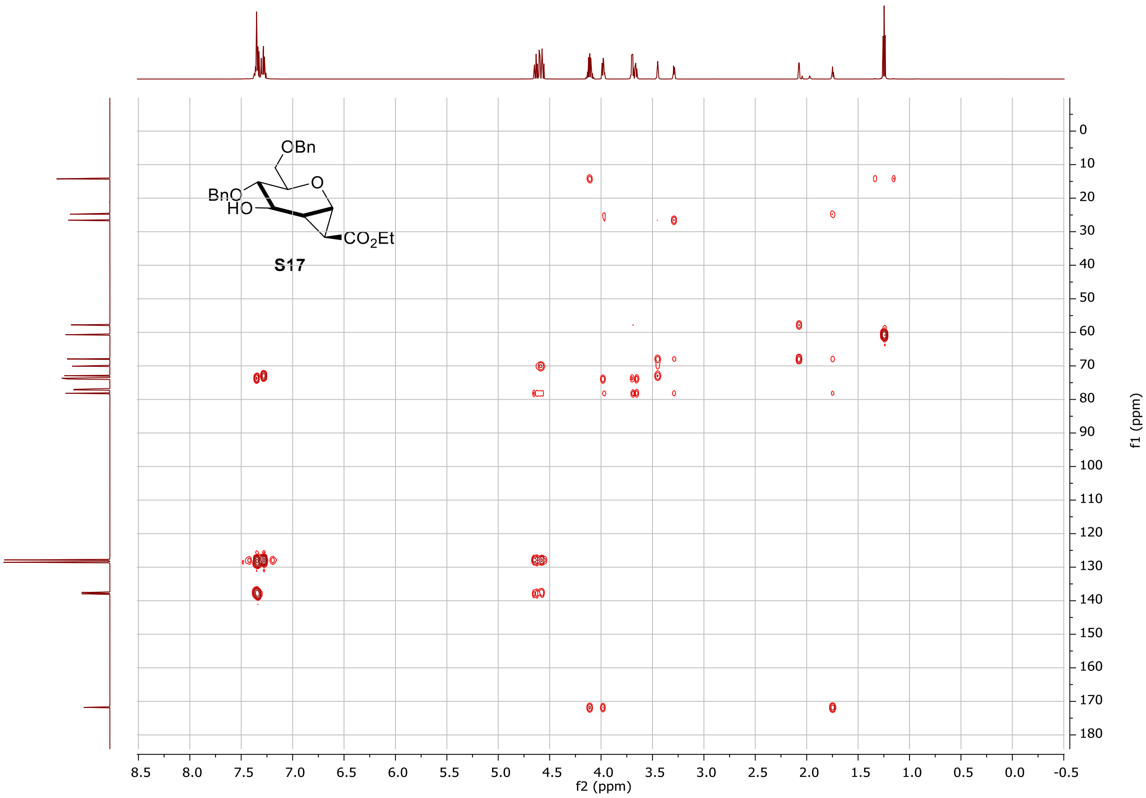

Supplementary Figure 159: HMBC spectra for compound **S17**

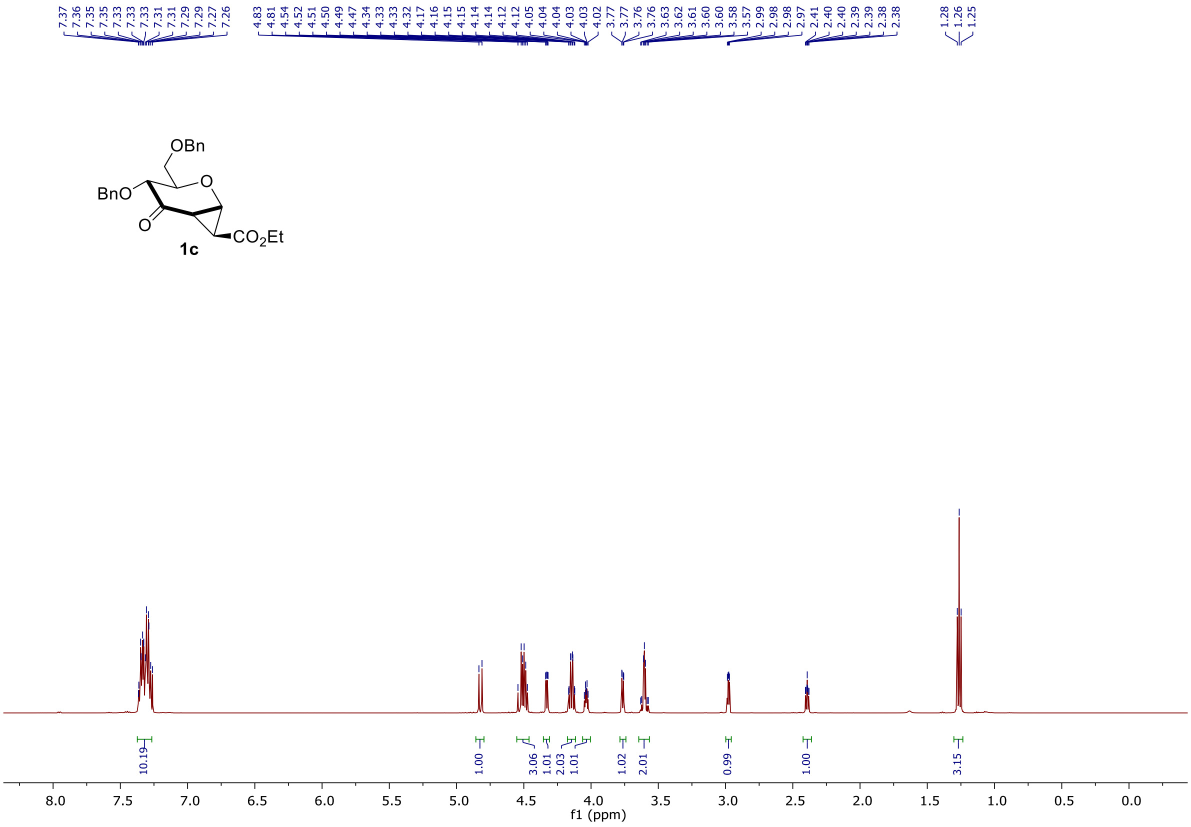

Supplementary Figure 160: ^{1}H spectra for **1c**

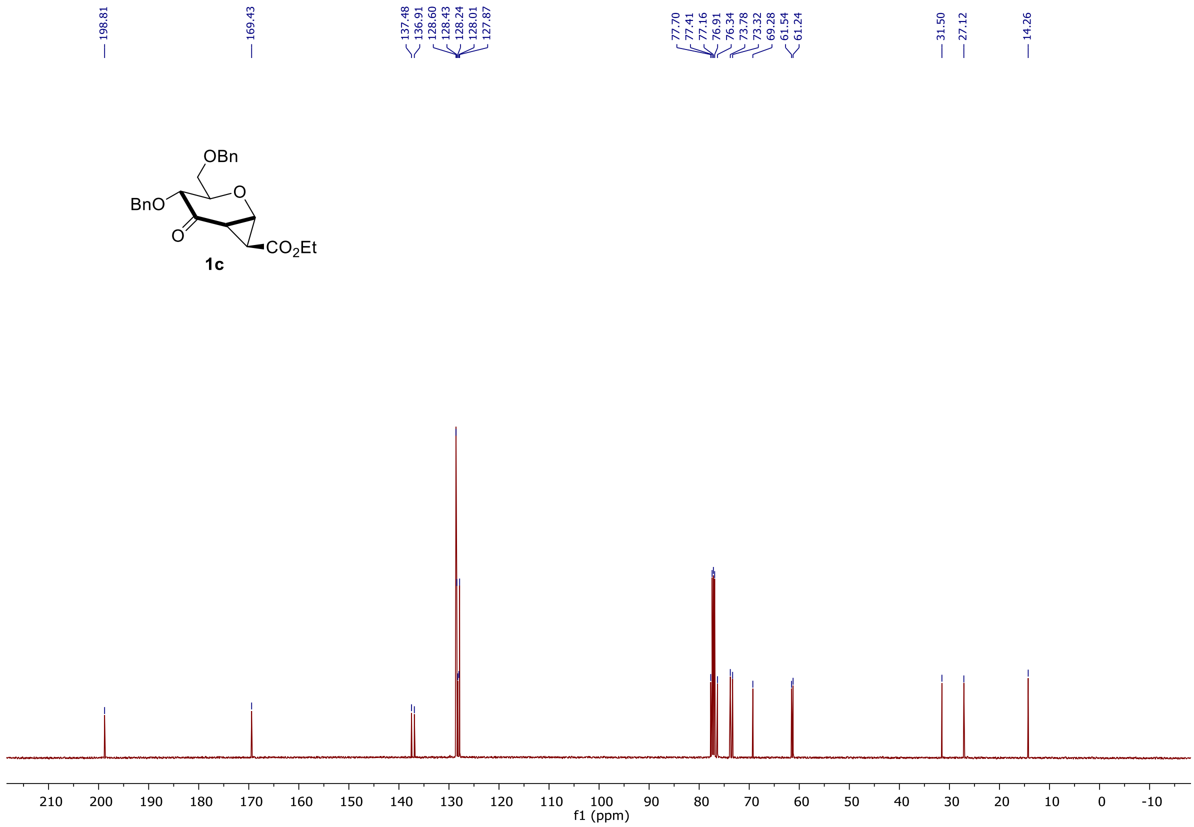

Supplementary Figure 161: ^{13}C spectra for **1c**

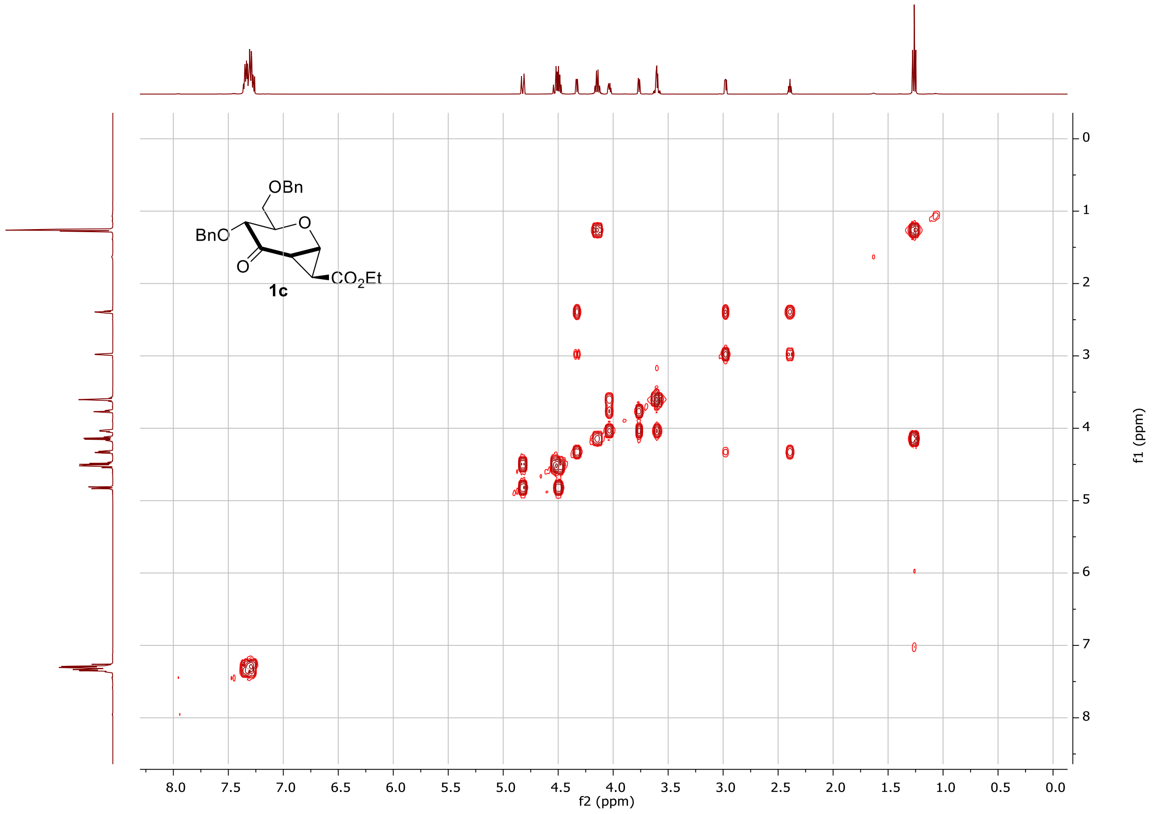

Supplementary Figure 162: COSY spectra for compound **1c**

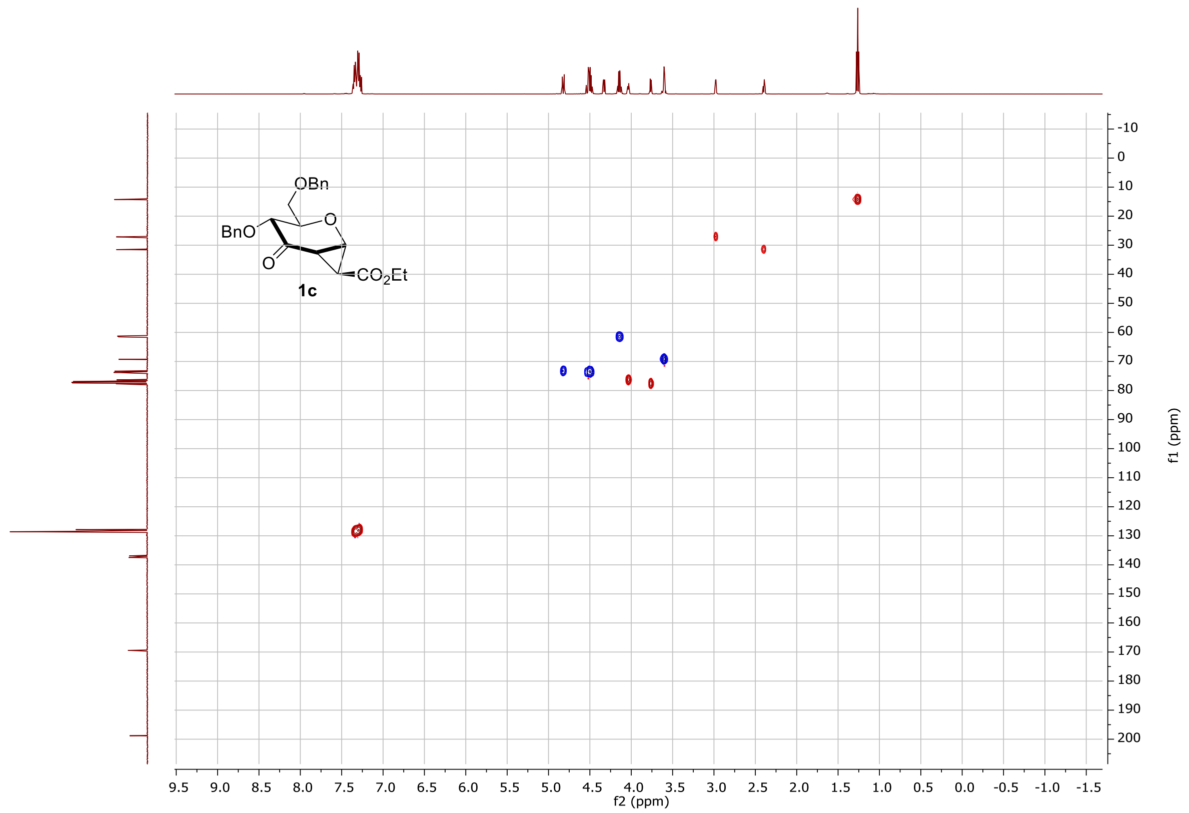

Supplementary Figure 163: HSQC spectra for compound **1c**

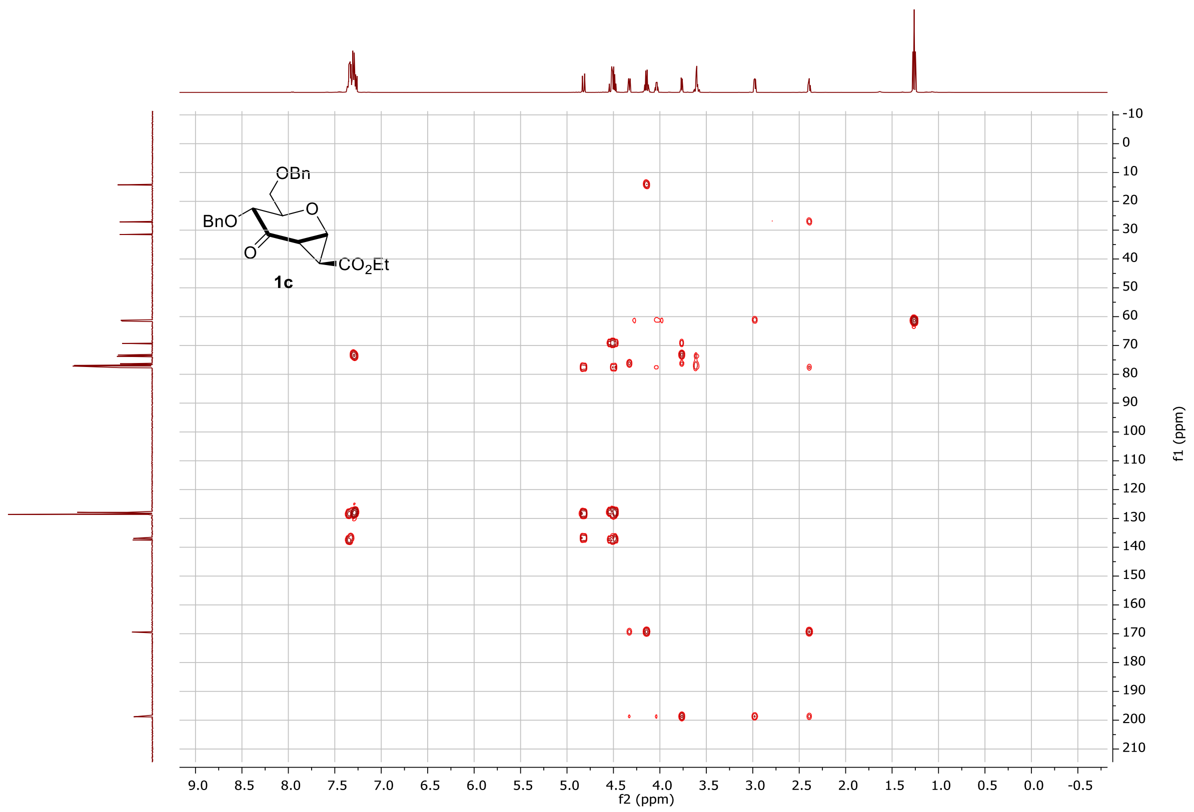

Supplementary Figure 164: HMBC spectra for compound **1c**

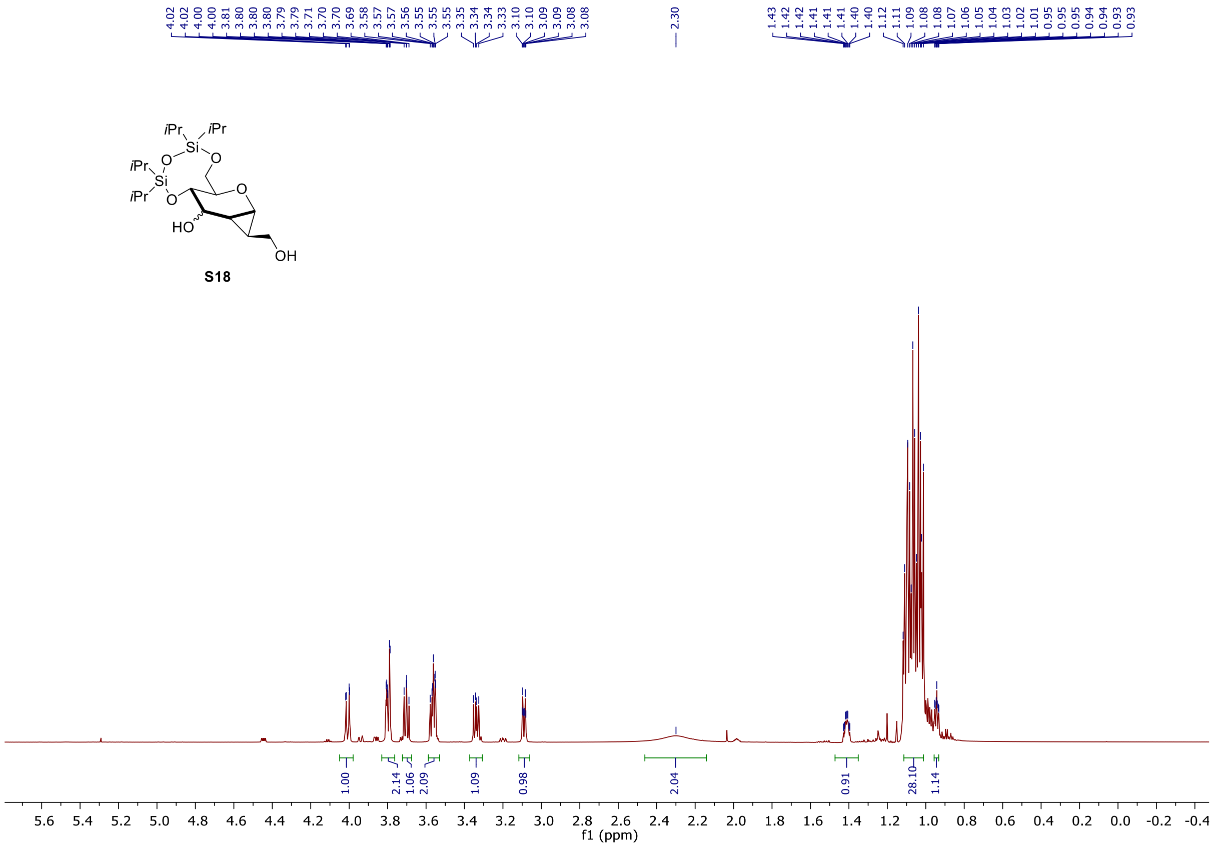

Supplementary Figure 165: ^{1}H spectra for **S18**

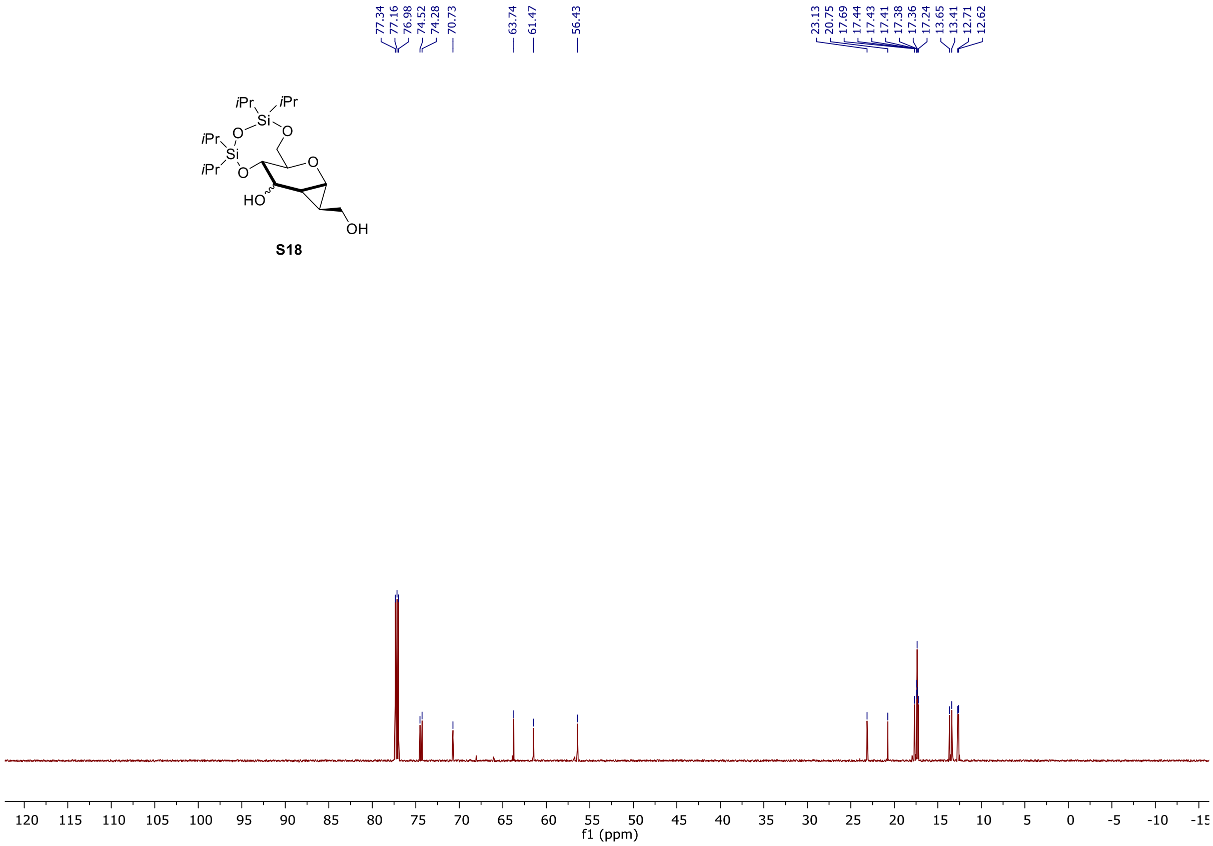

Supplementary Figure 166: ^{13}C spectra for **S18**

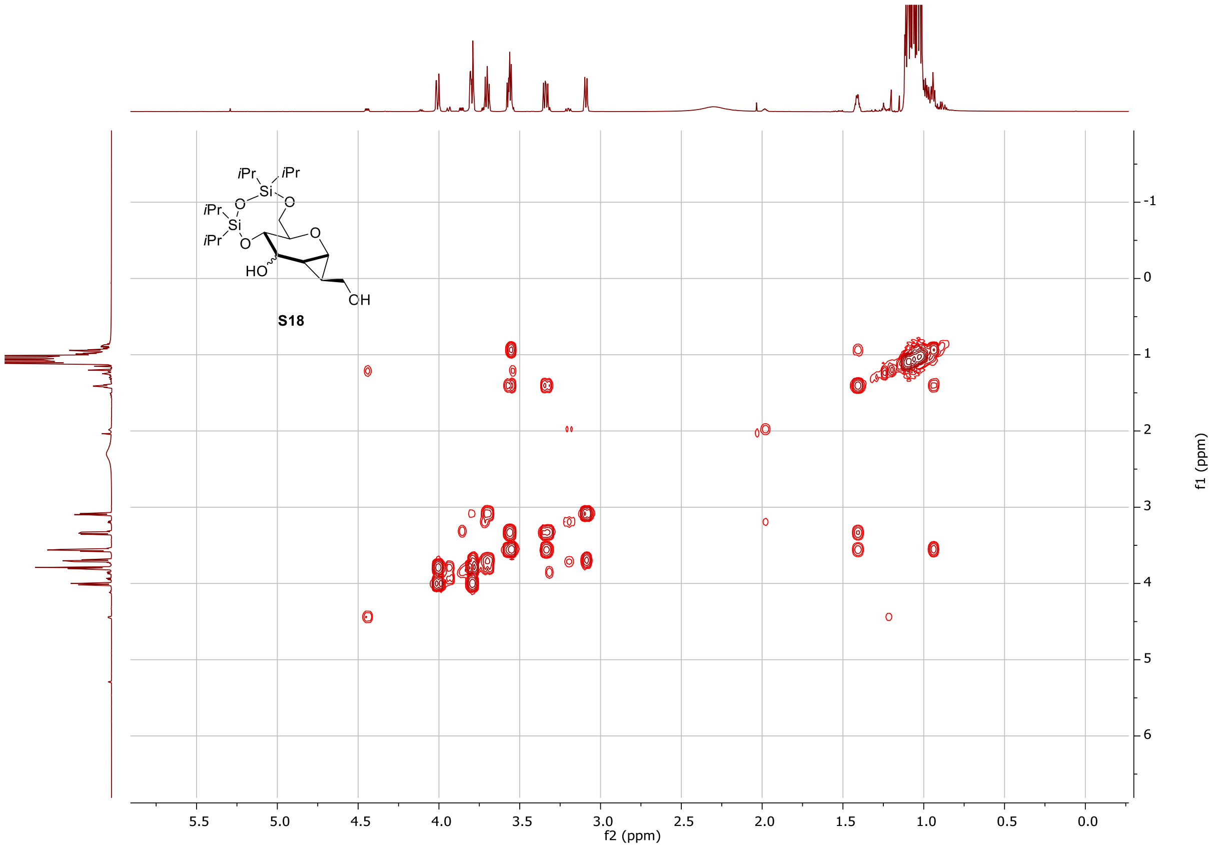

Supplementary Figure 167: COSY spectra for compound **S18**

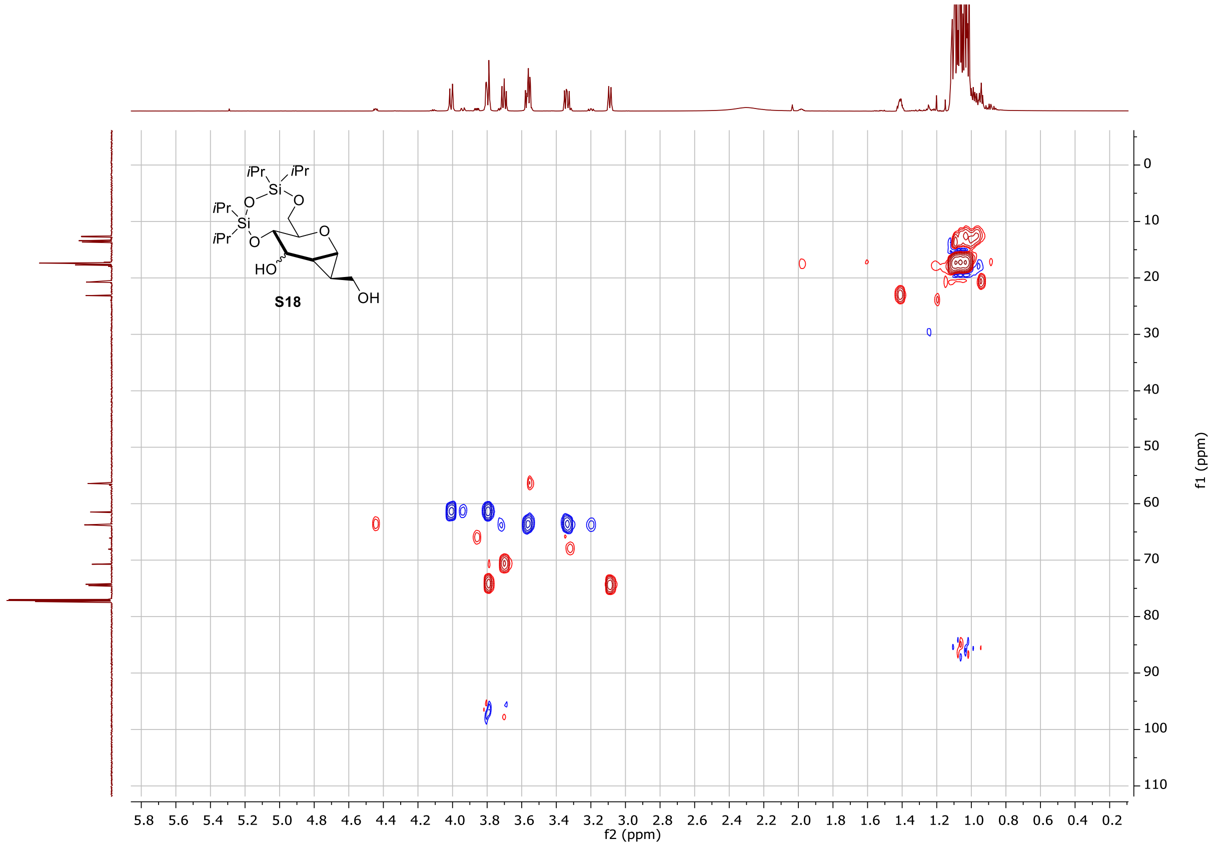

Supplementary Figure 168: HSQC spectra for compound **S18**

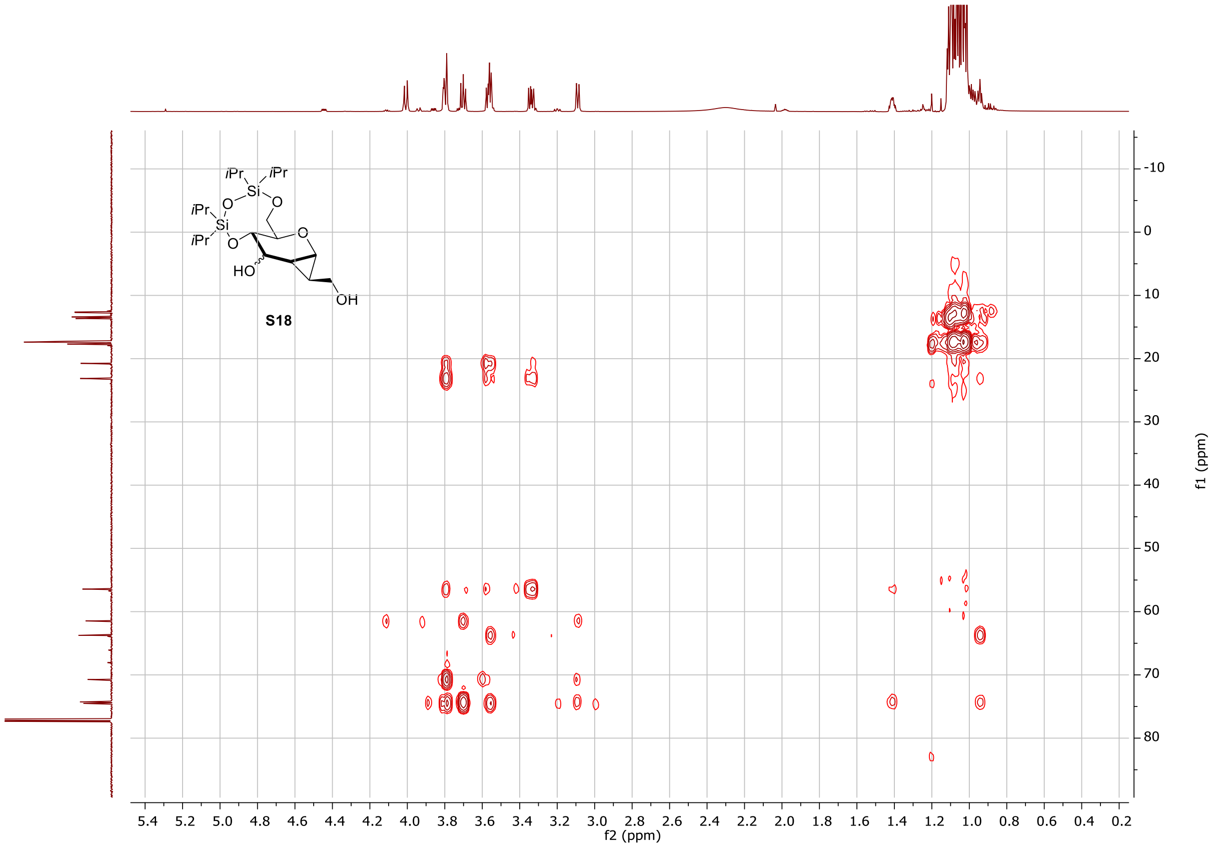

Supplementary Figure 169: HMBC spectra for compound **S18**

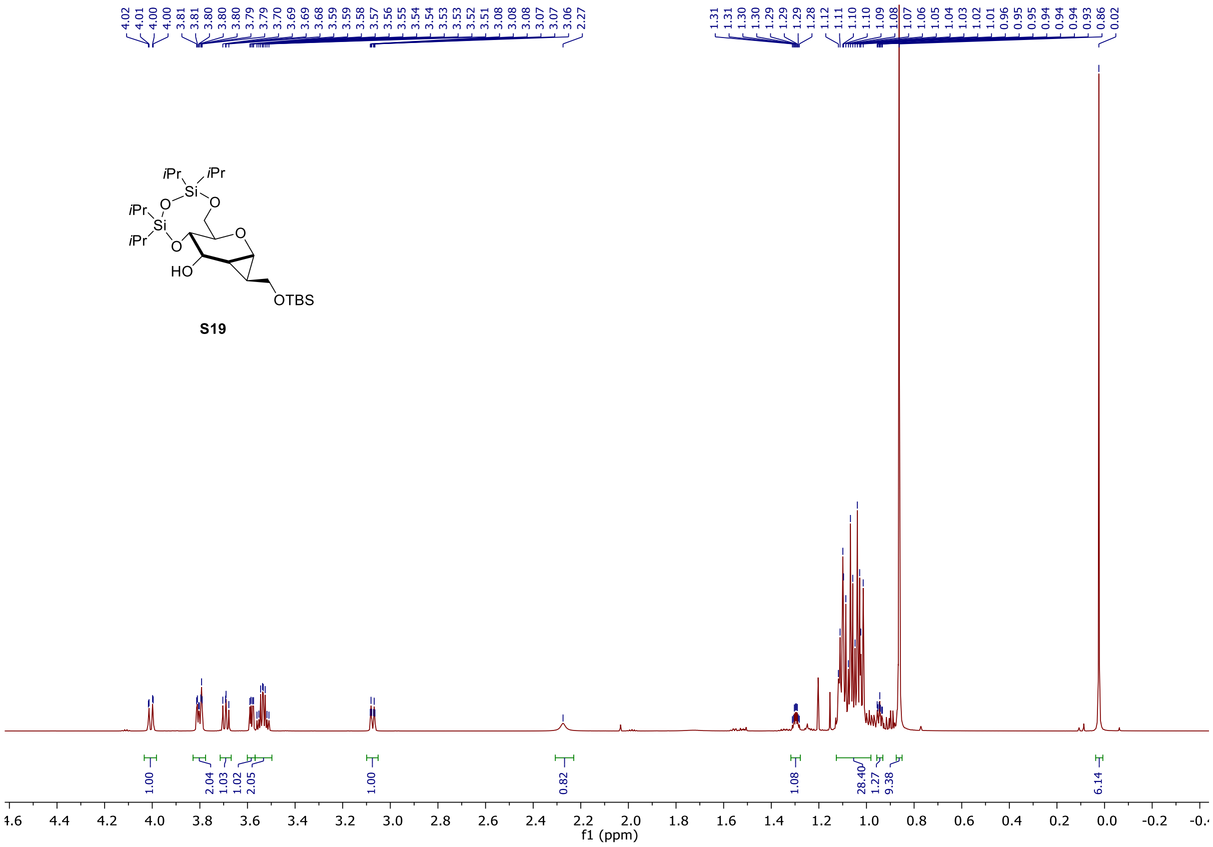

Supplementary Figure 170: ^{1}H spectra for **S19**

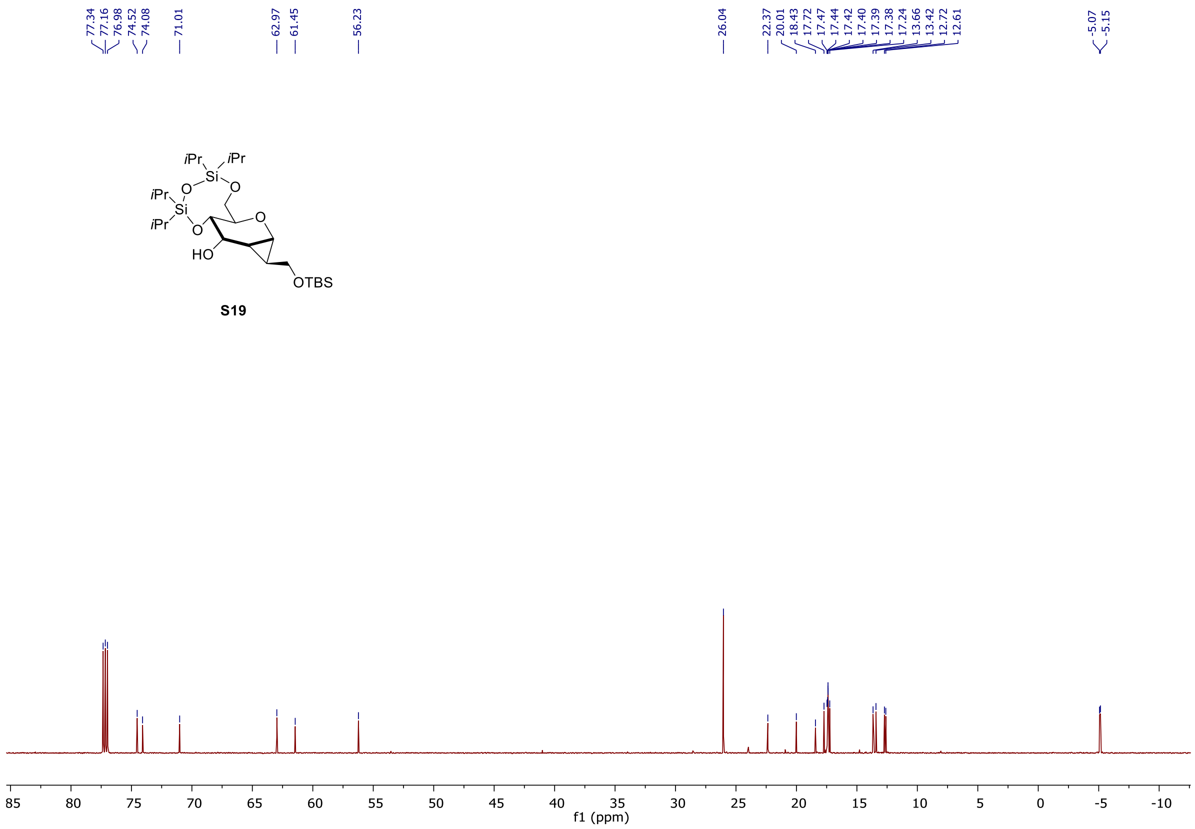

Supplementary Figure 171: ^{13}C spectra for **S19**

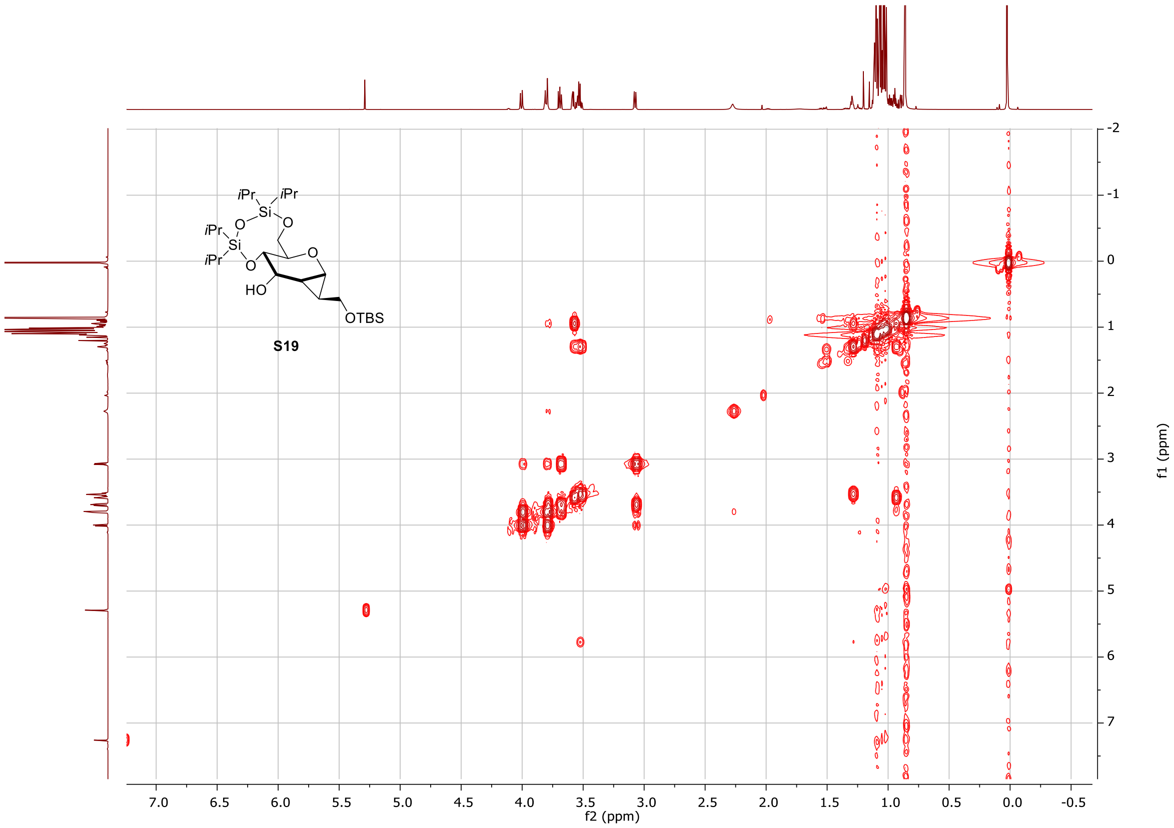

Supplementary Figure 172: COSY spectra for compound **S19**

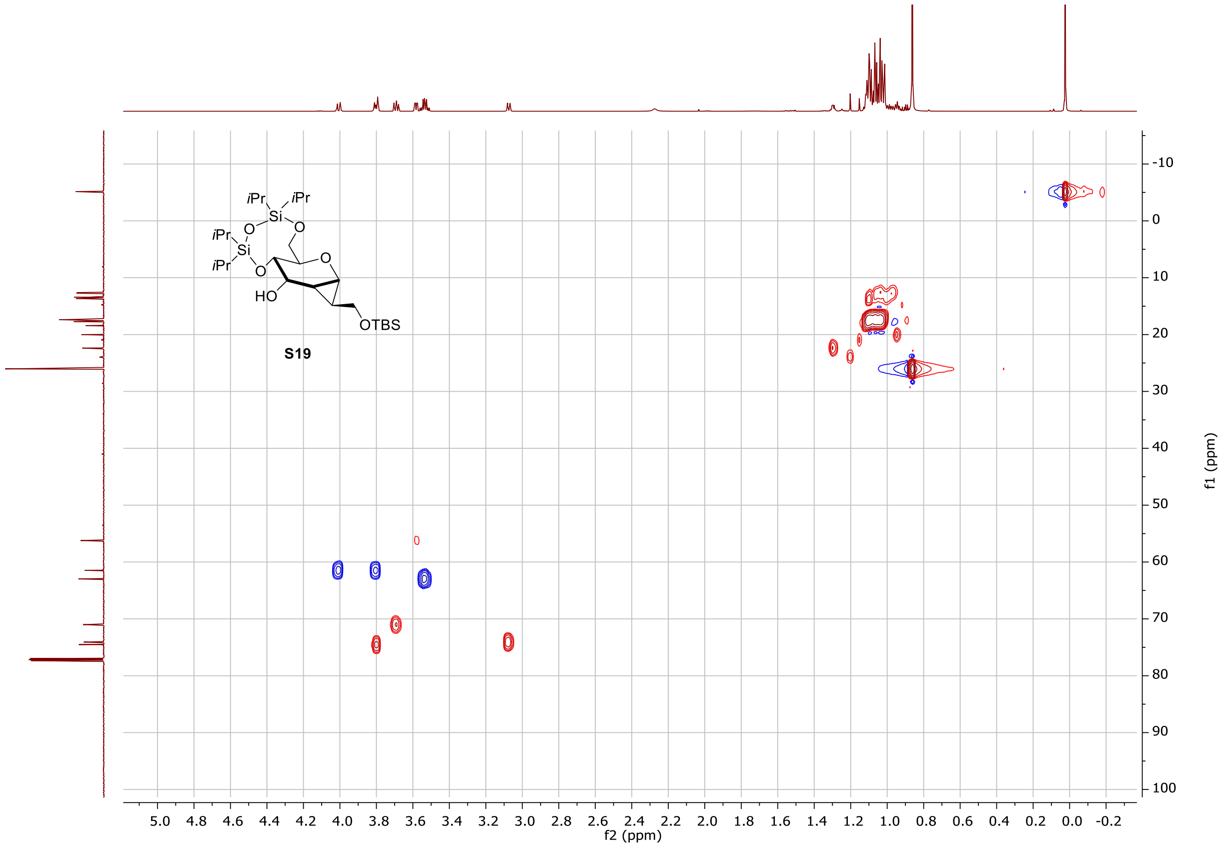

Supplementary Figure 173: HSQC spectra for compound **S19**

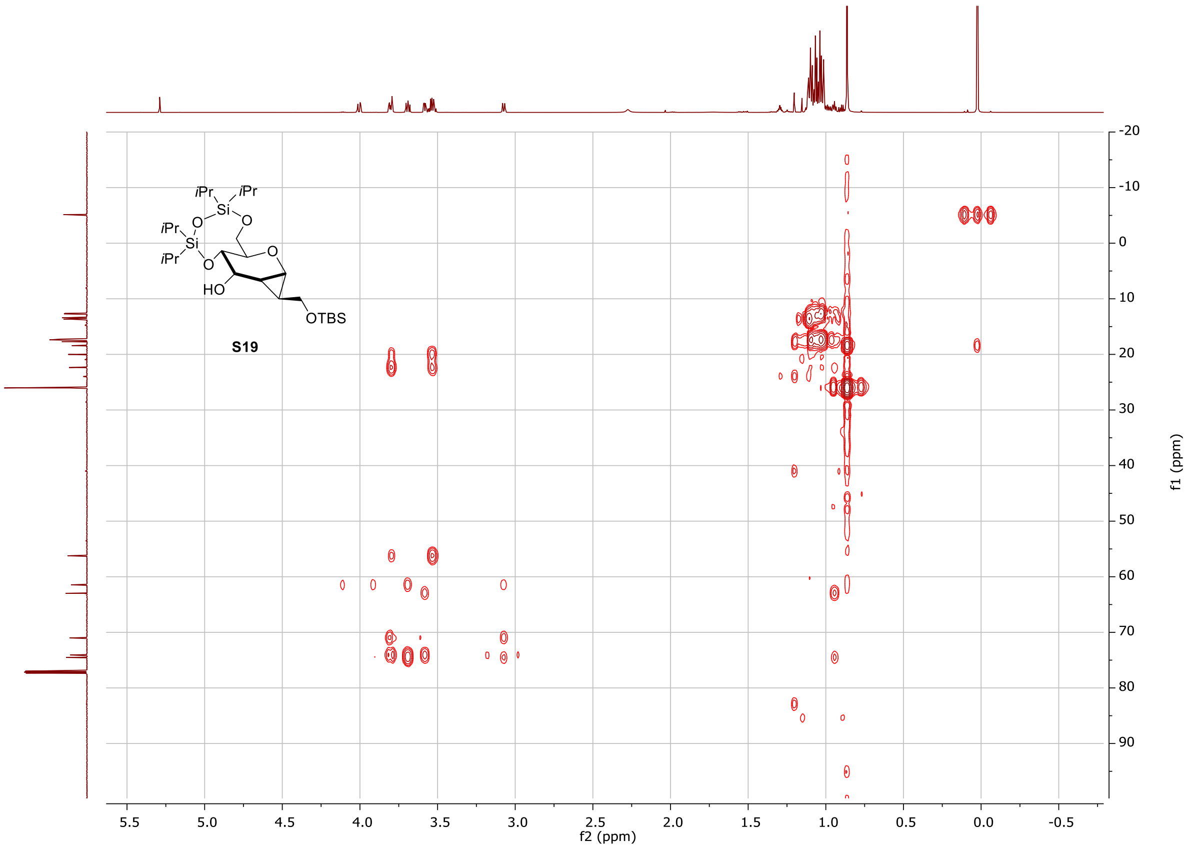

Supplementary Figure 174: HMBC spectra for compound **S19**

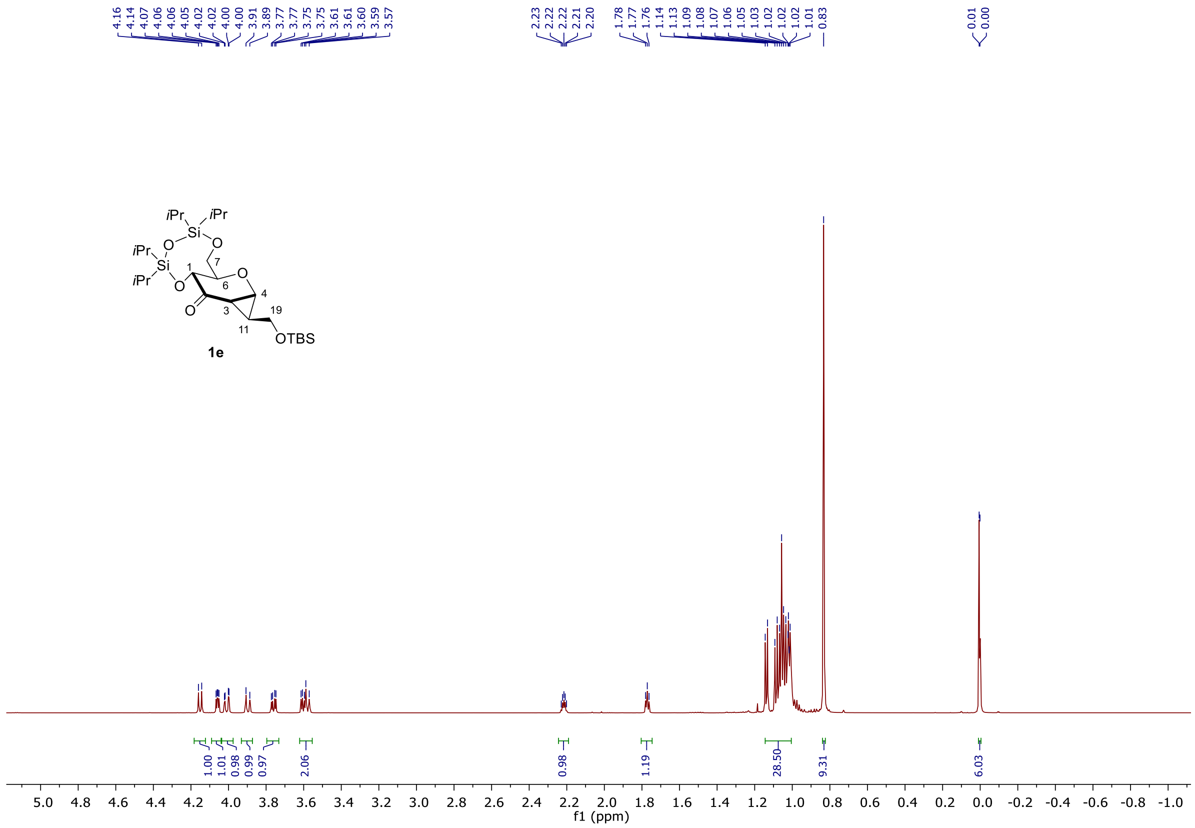

Supplementary Figure 175: ^{1}H spectra for **1e**

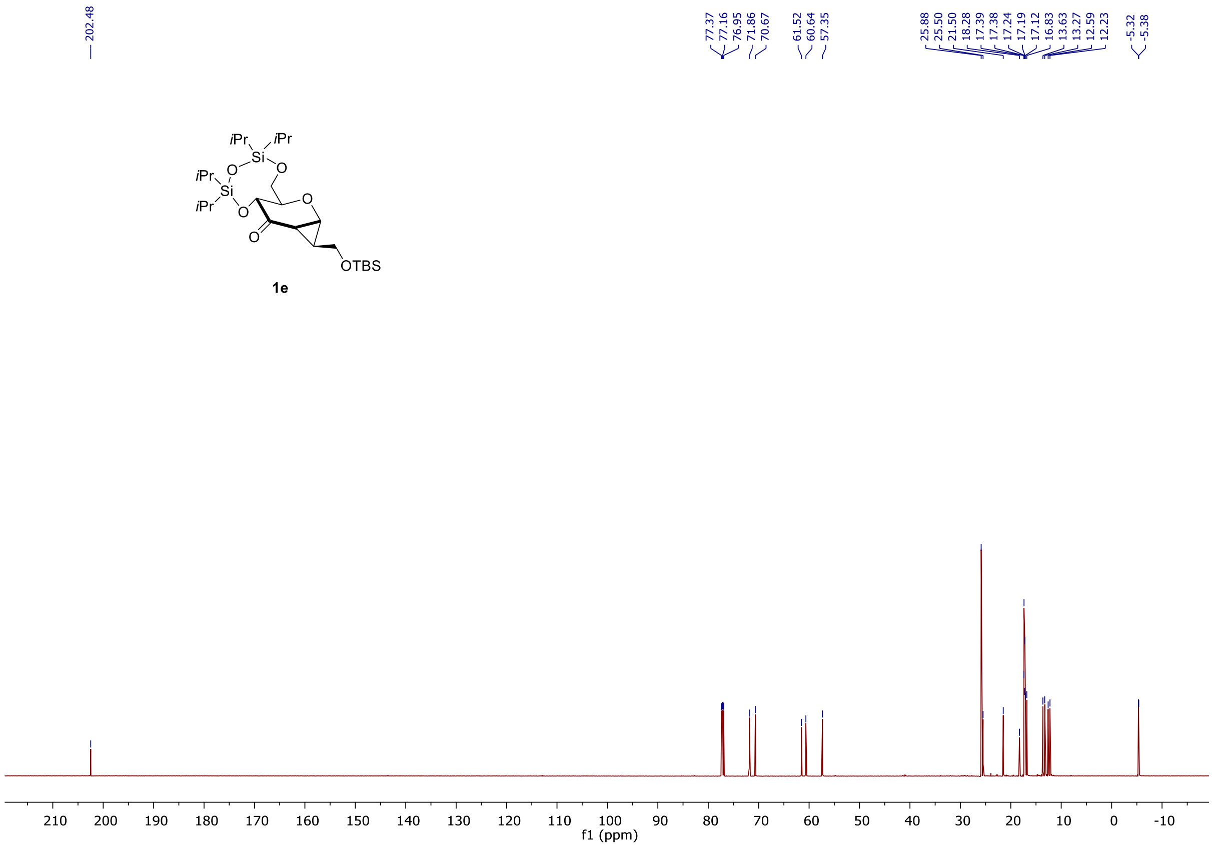

Supplementary Figure 176: ^{13}C spectra for **1e**

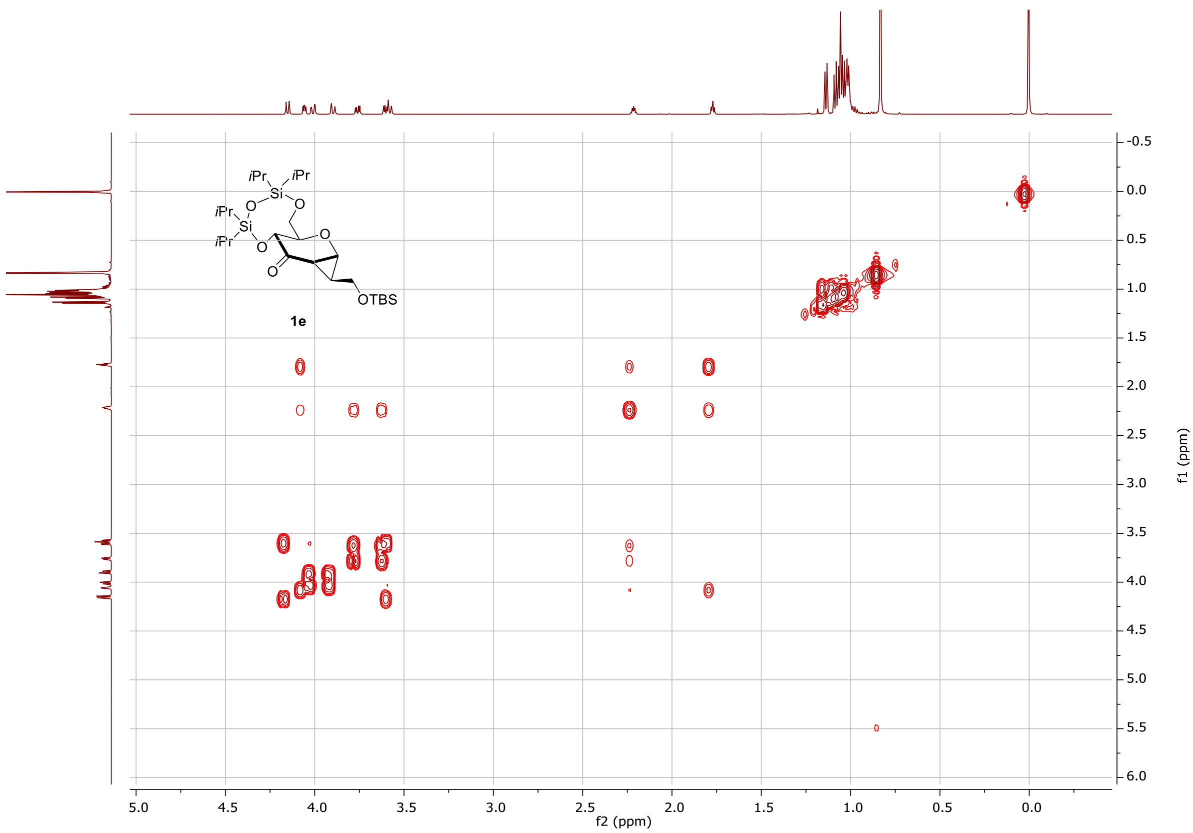

Supplementary Figure 177: COSY spectra for compound **1e**

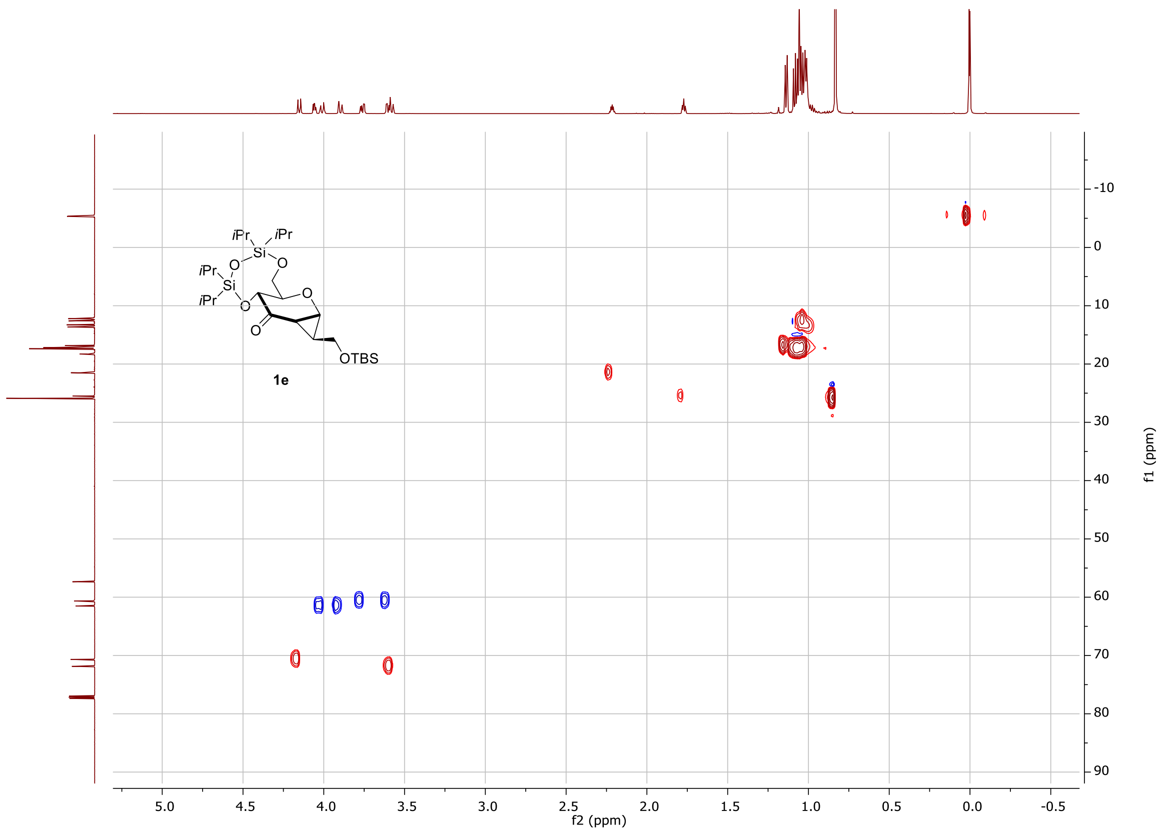

Supplementary Figure 178: HSQC spectra for compound **1e**

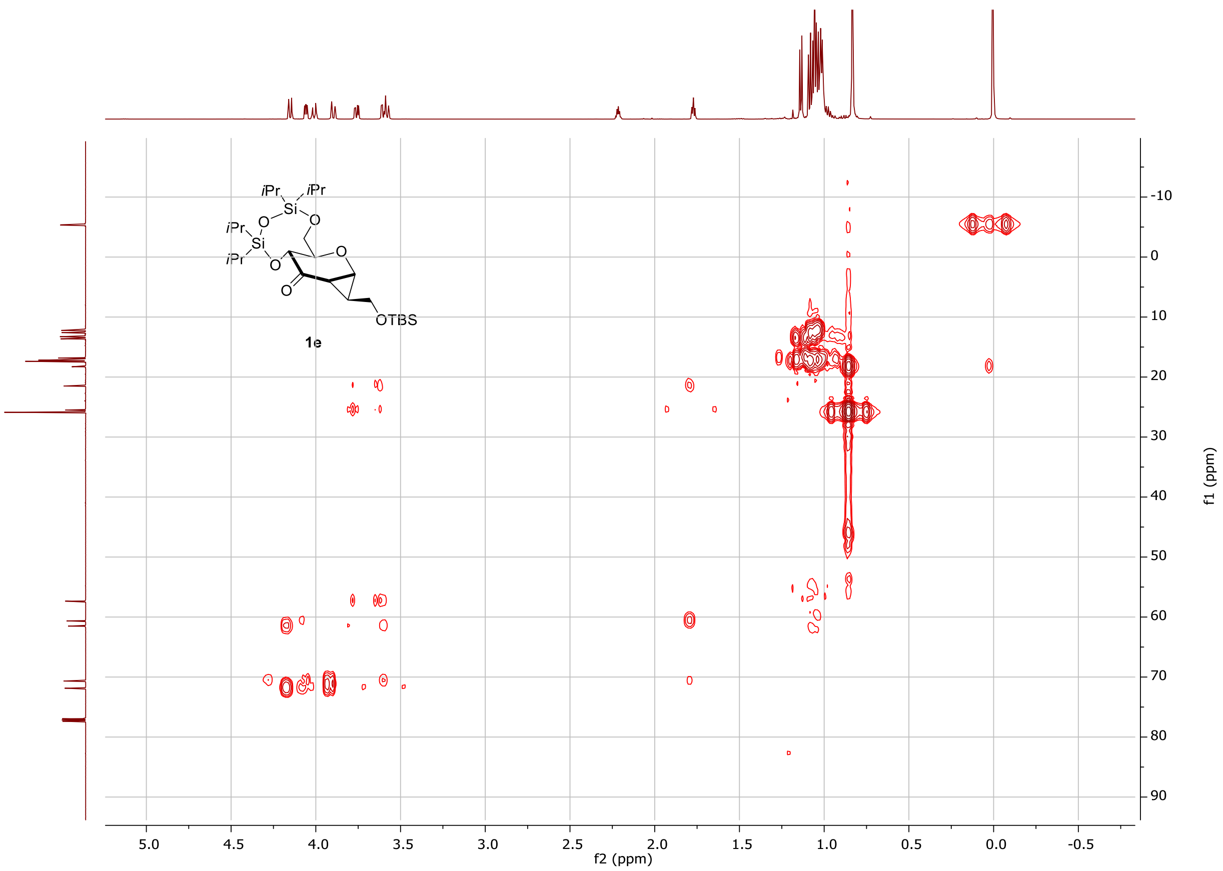

Supplementary Figure 179: HMBC spectra for compound **1e**

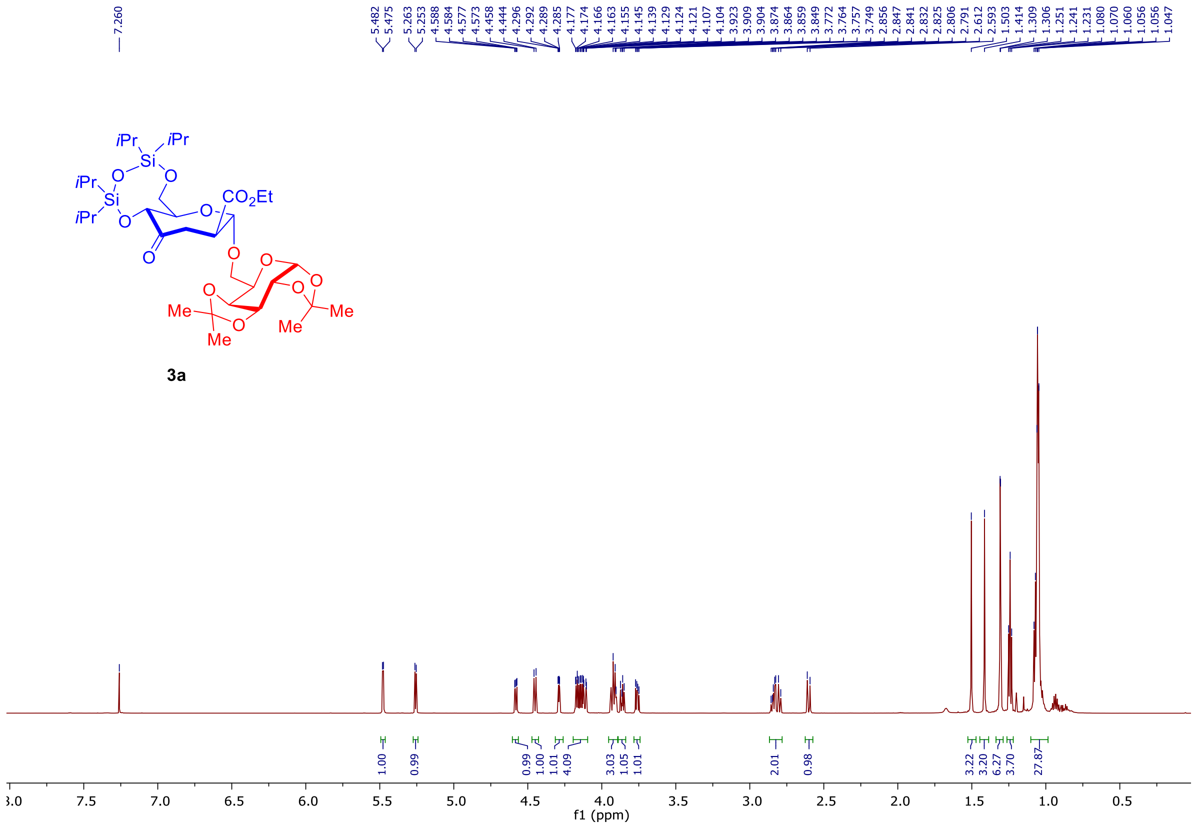

Supplementary Figure 180: ^{1}H spectra for **3a**

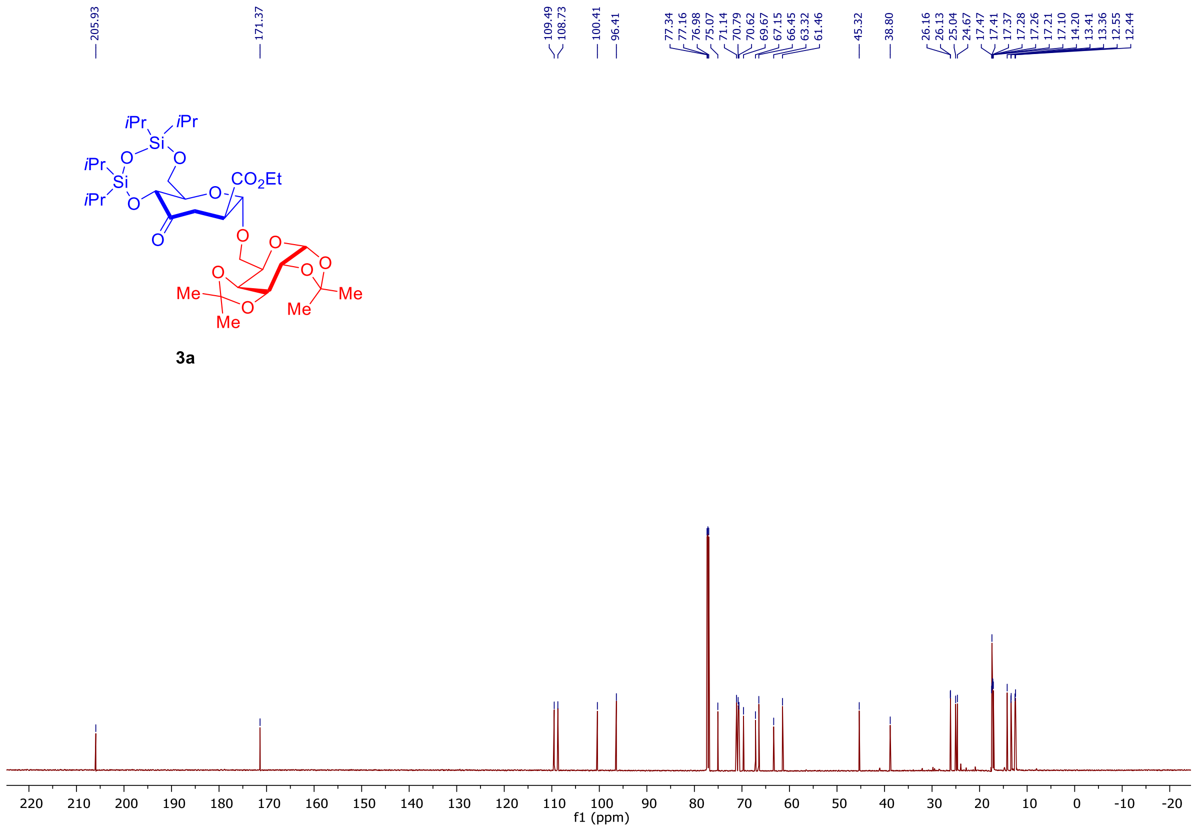

Supplementary Figure 181: ^{13}C spectra for **3a**

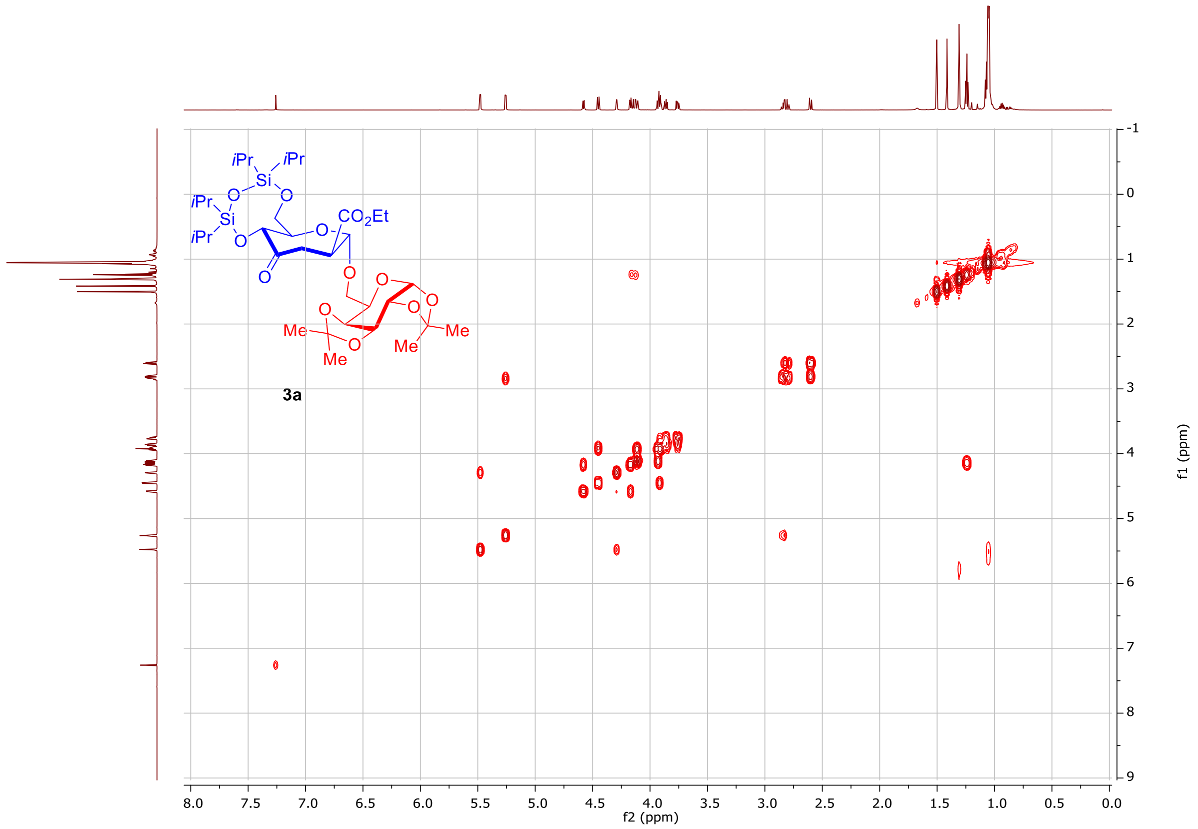

Supplementary Figure 182: COSY spectra for compound **3a**

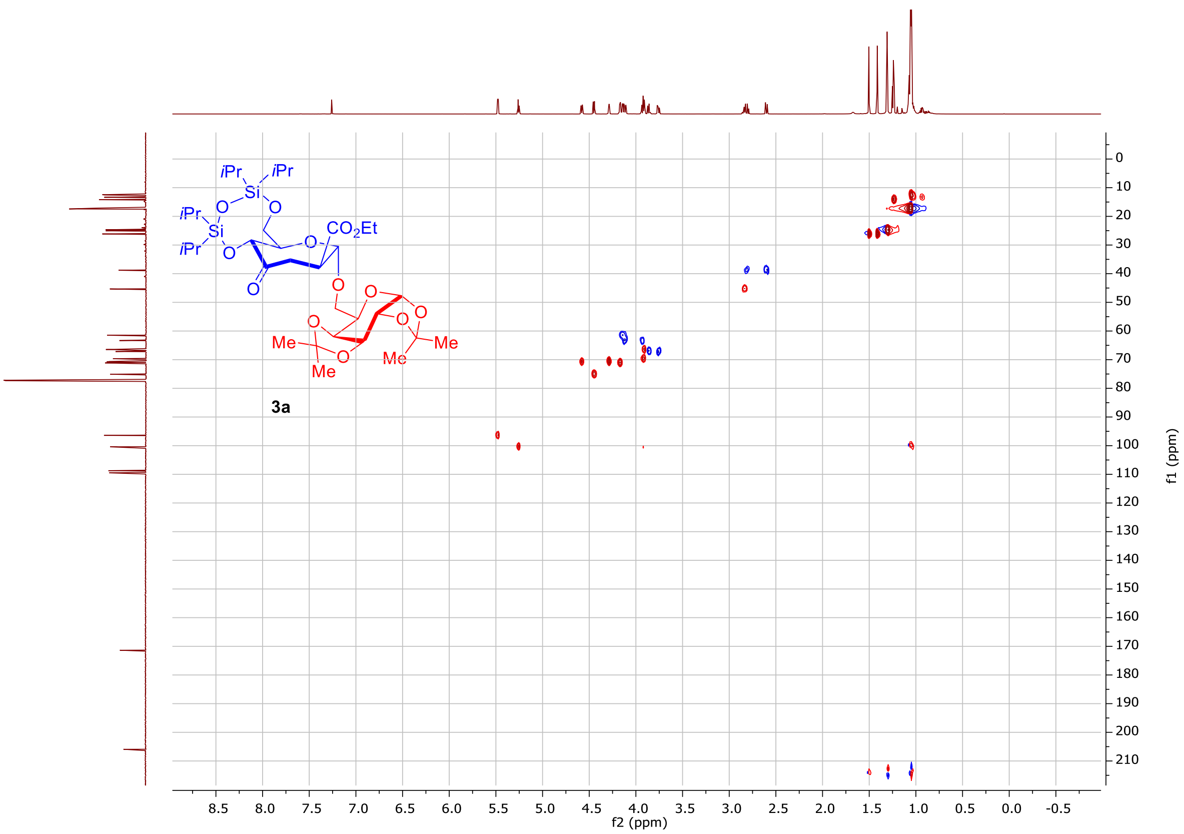

Supplementary Figure 183: HSQC spectra for compound **3a**

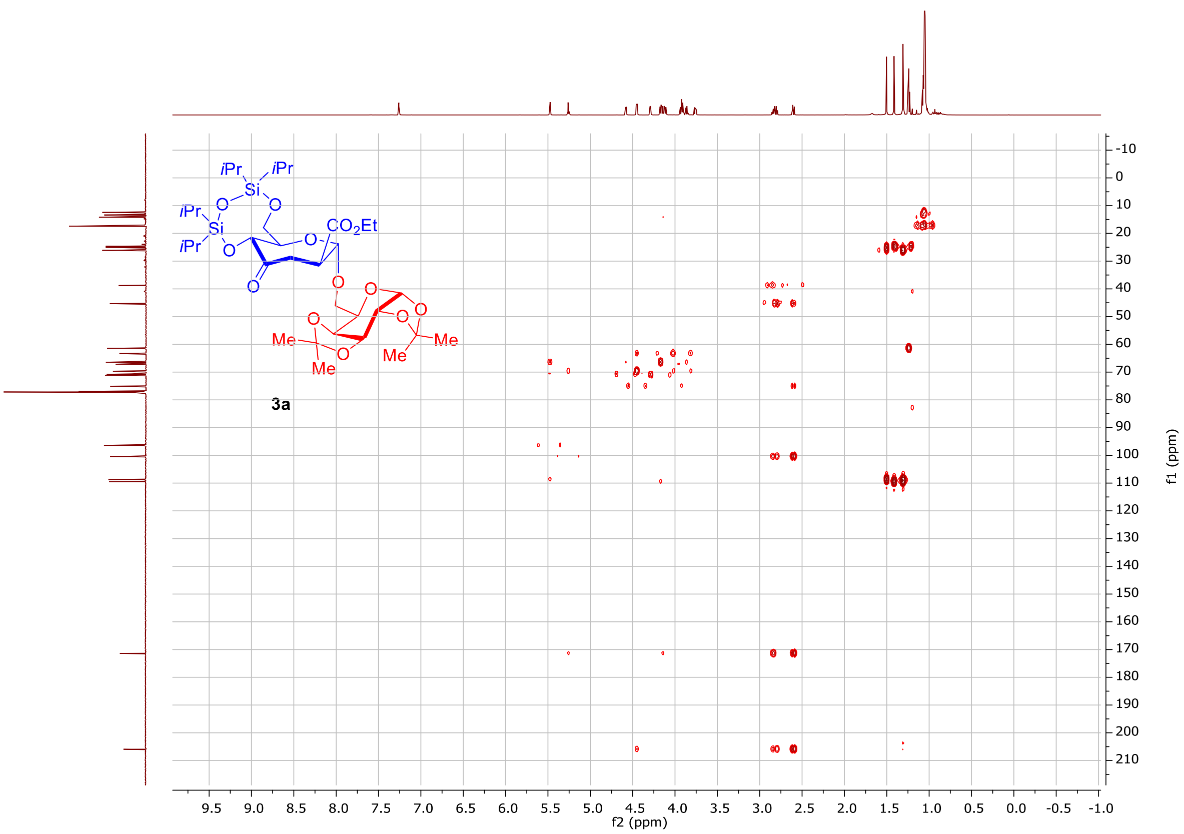

Supplementary Figure 184: HMBC spectra for compound **3a**

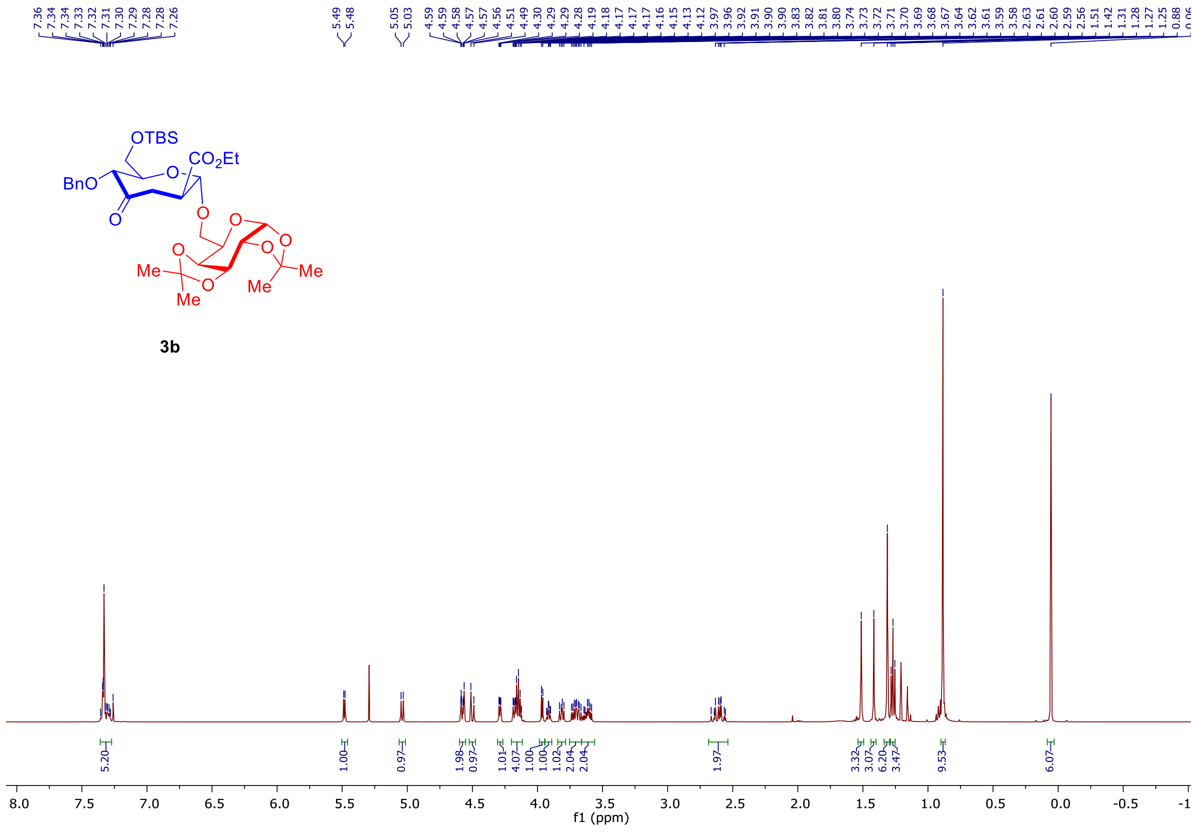

Supplementary Figure 185: ^{1}H spectra for **3b**

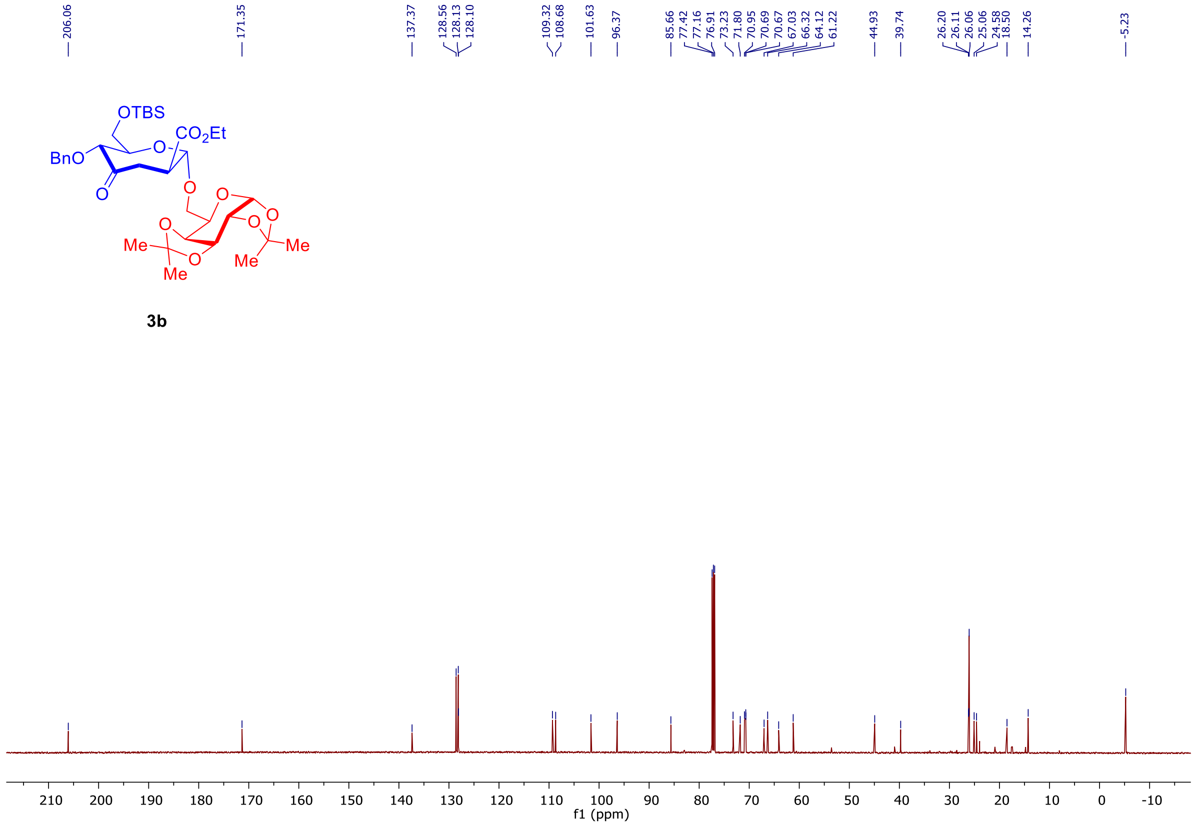

Supplementary Figure 186: ^{13}C spectra for **3b**

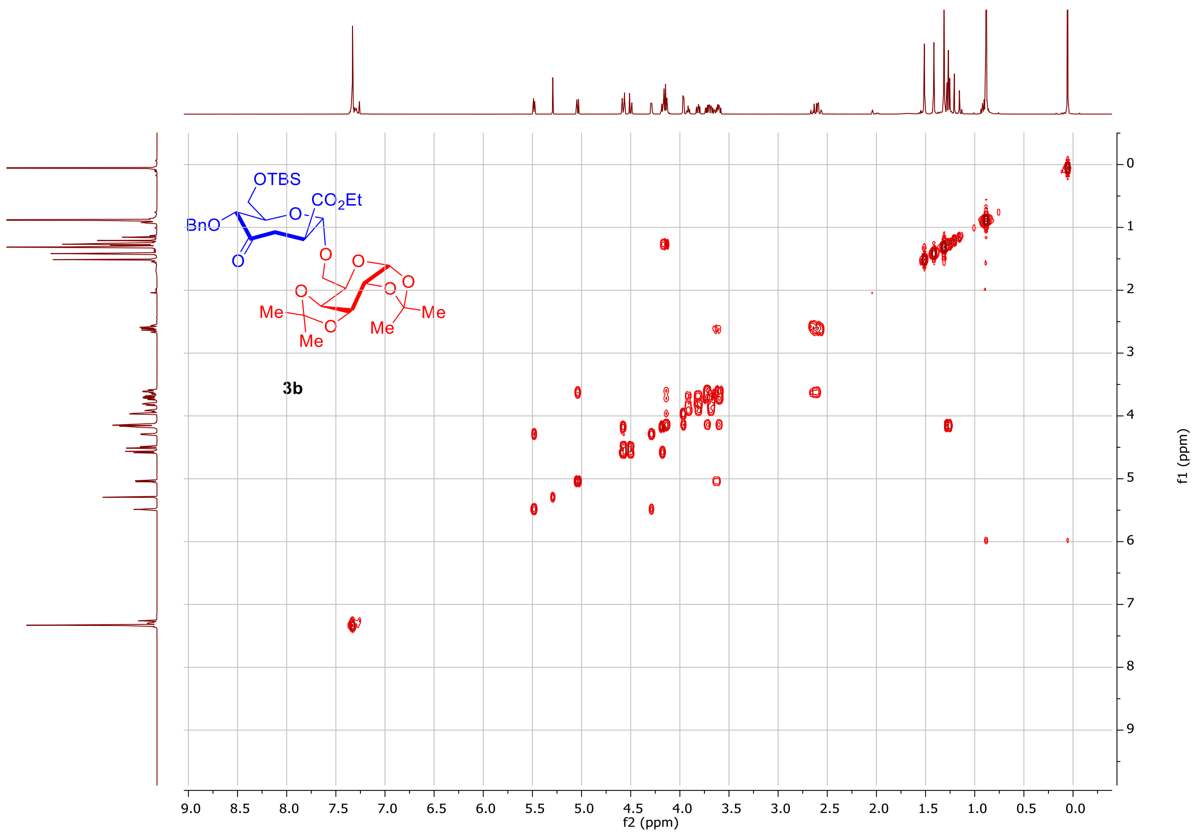

Supplementary Figure 187: COSY spectra for compound **3b**

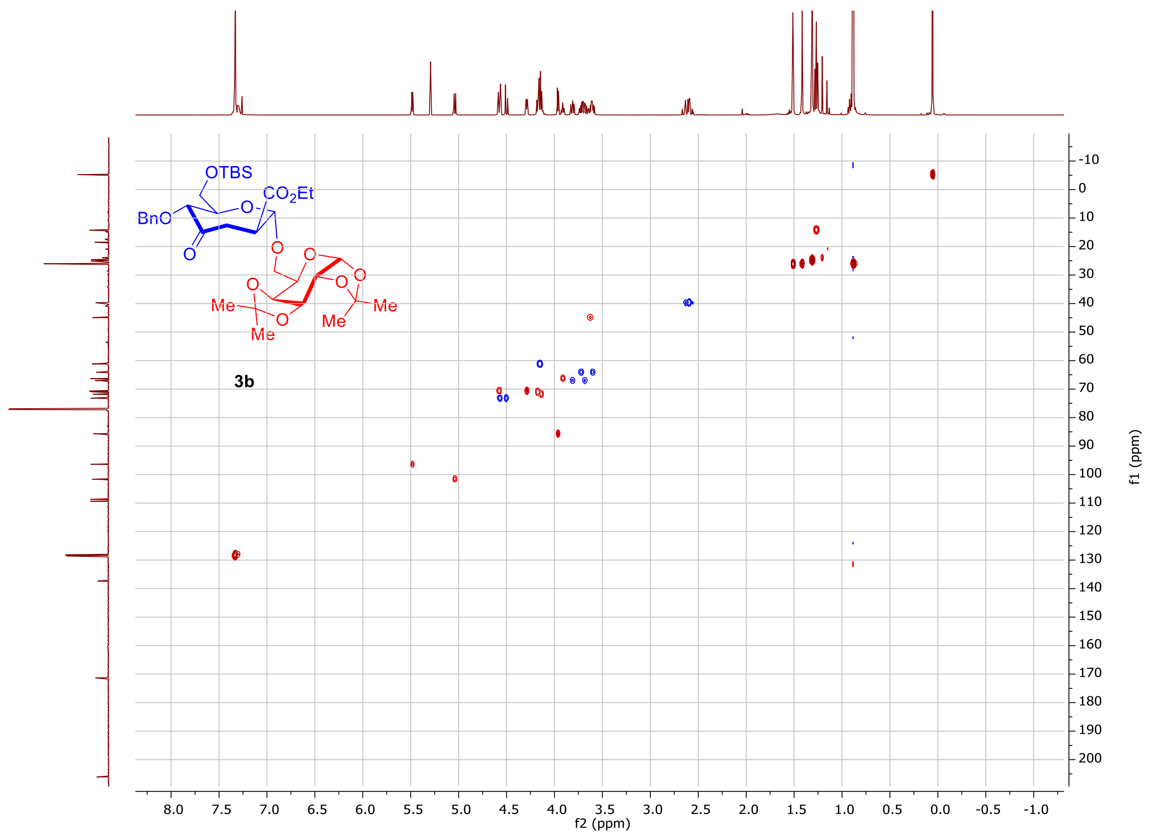

Supplementary Figure 188: HSQC spectra for compound **3b**

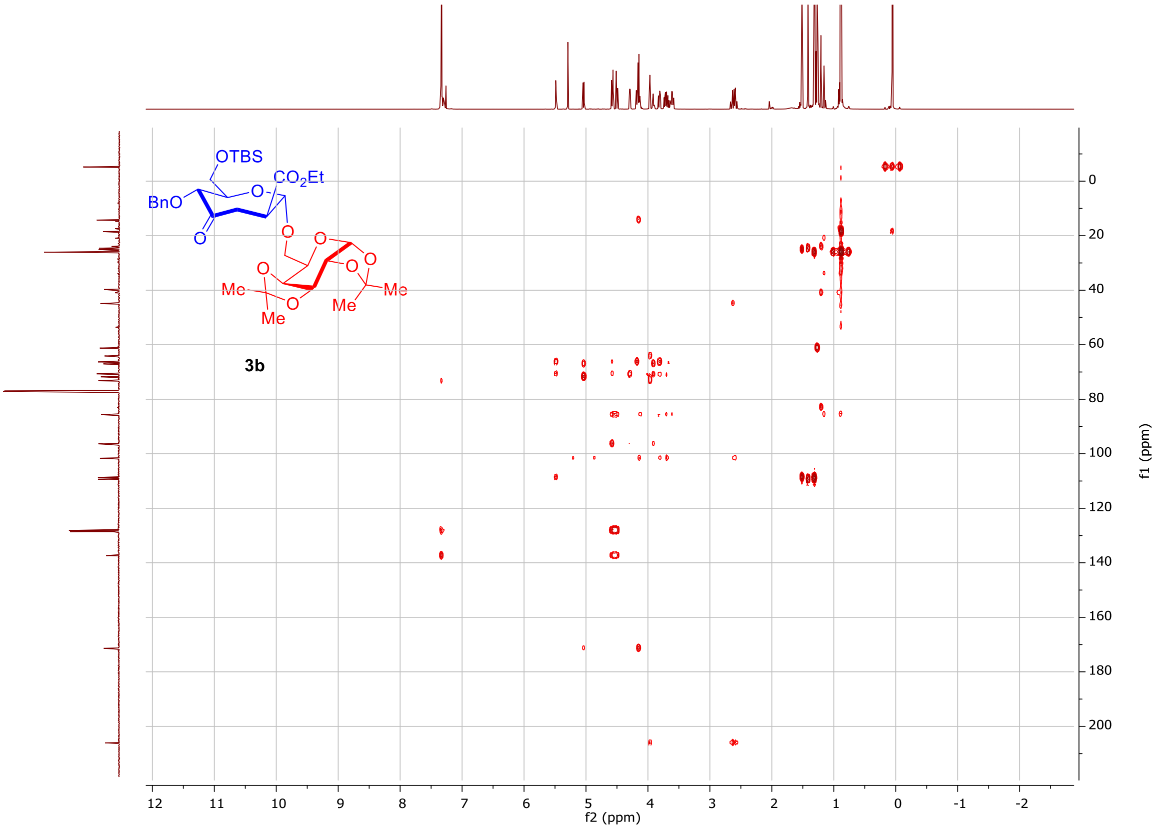

Supplementary Figure 189: HMBC spectra for compound **3b**

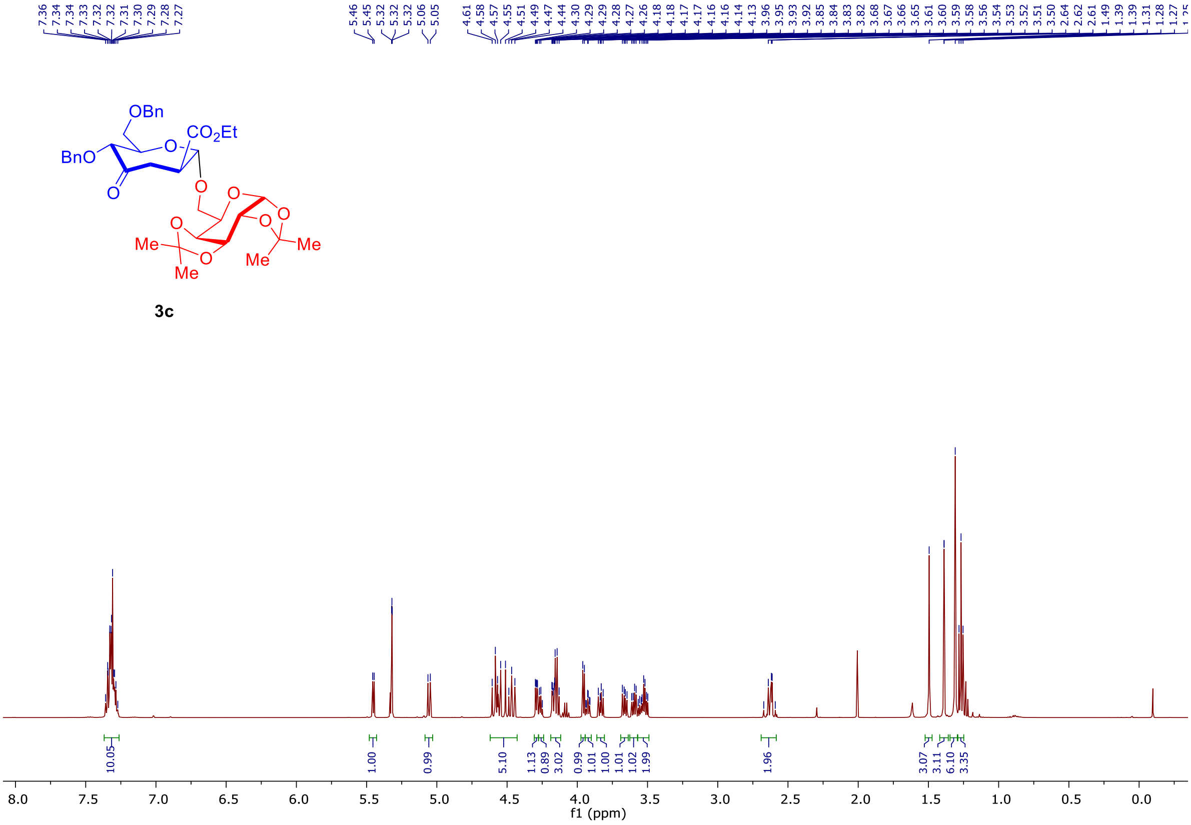

Supplementary Figure 190: ^{1}H spectra for **3c**

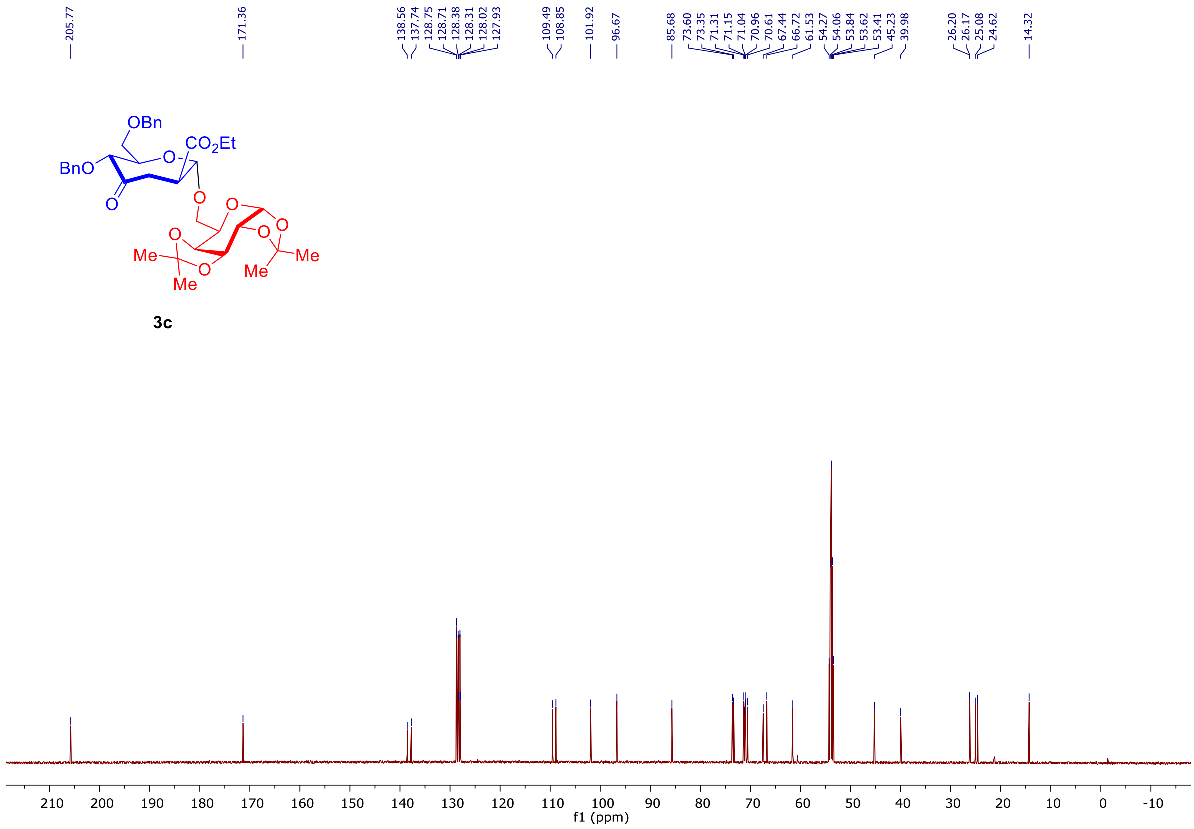

Supplementary Figure 191: ^{13}C spectra for **3c**

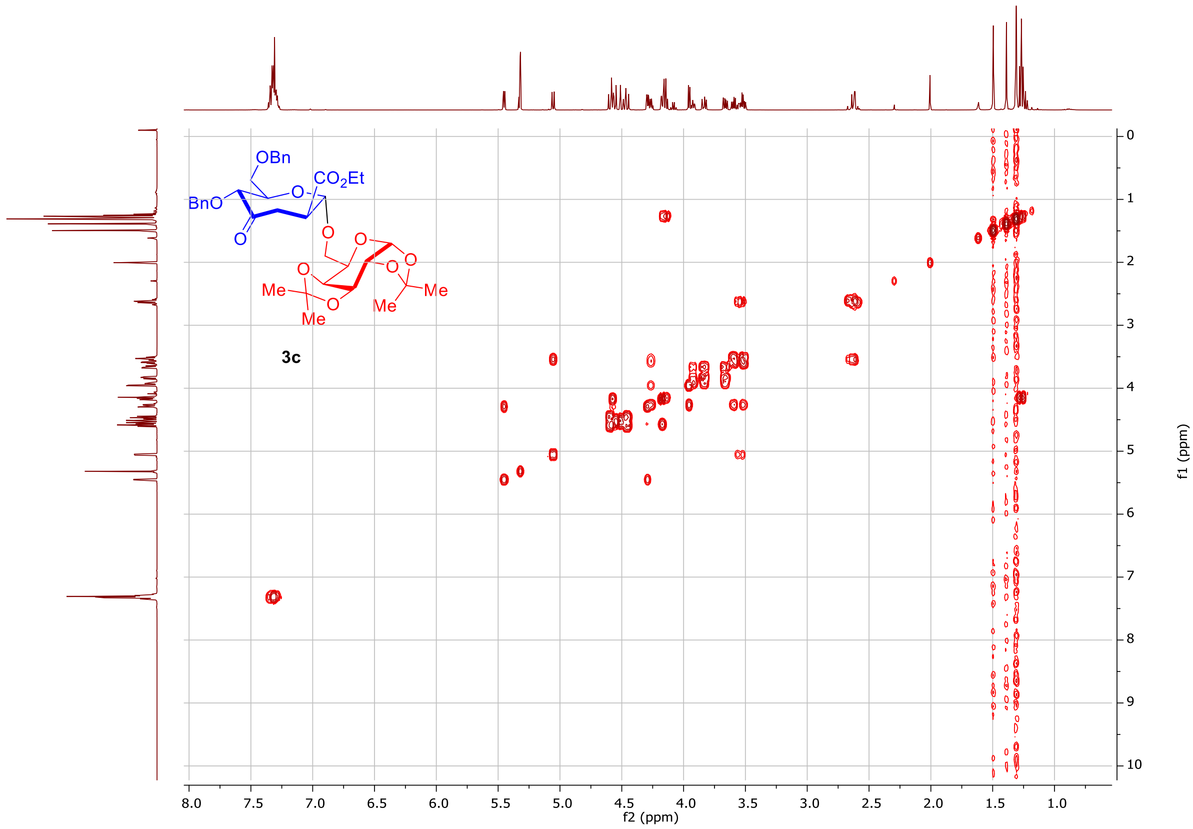

Supplementary Figure 192: COSY spectra for compound **3c**

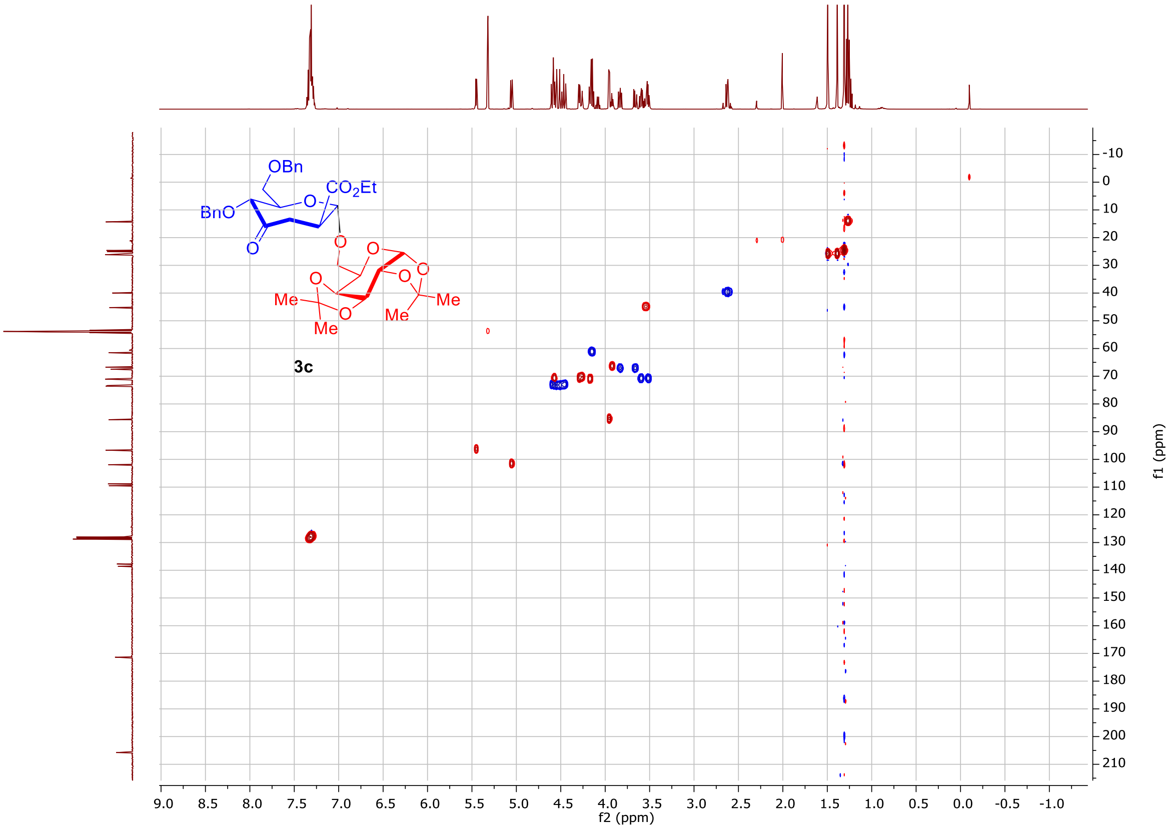

Supplementary Figure 193: HSQC spectra for compound **3c**

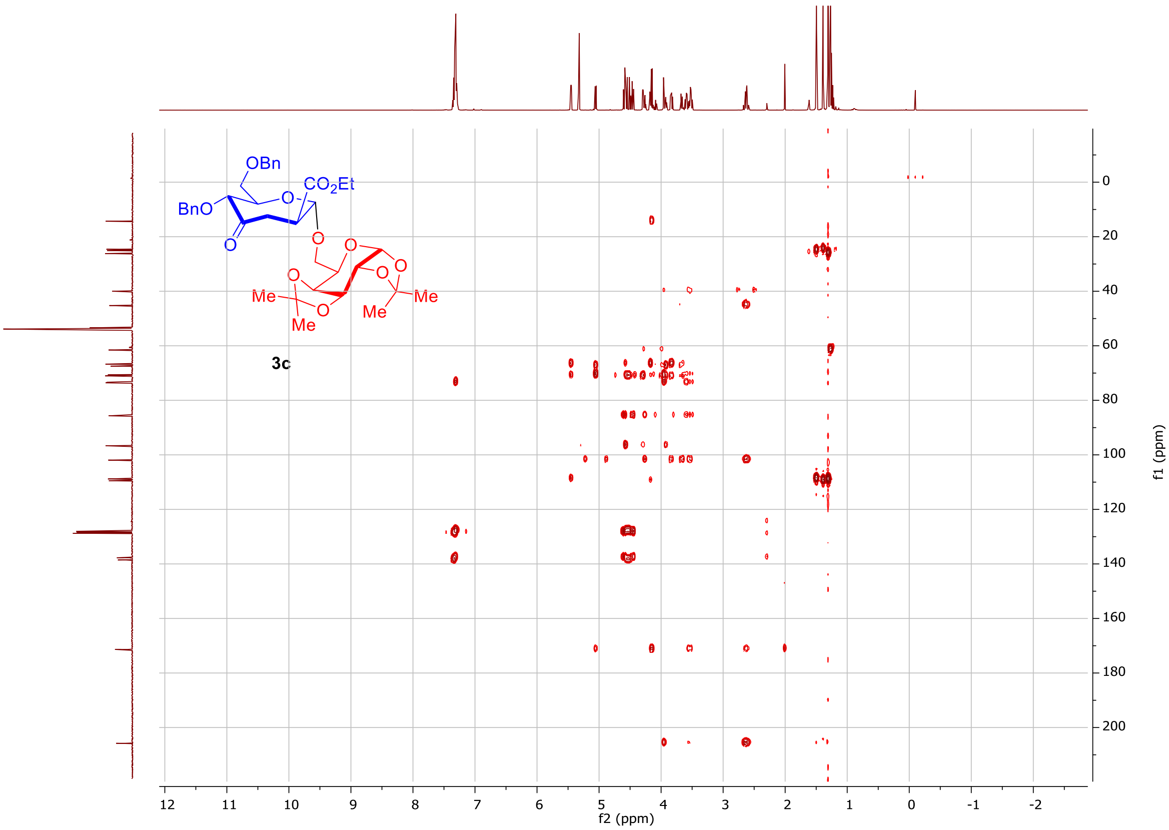

Supplementary Figure 194: HMBC spectra for compound **3c**

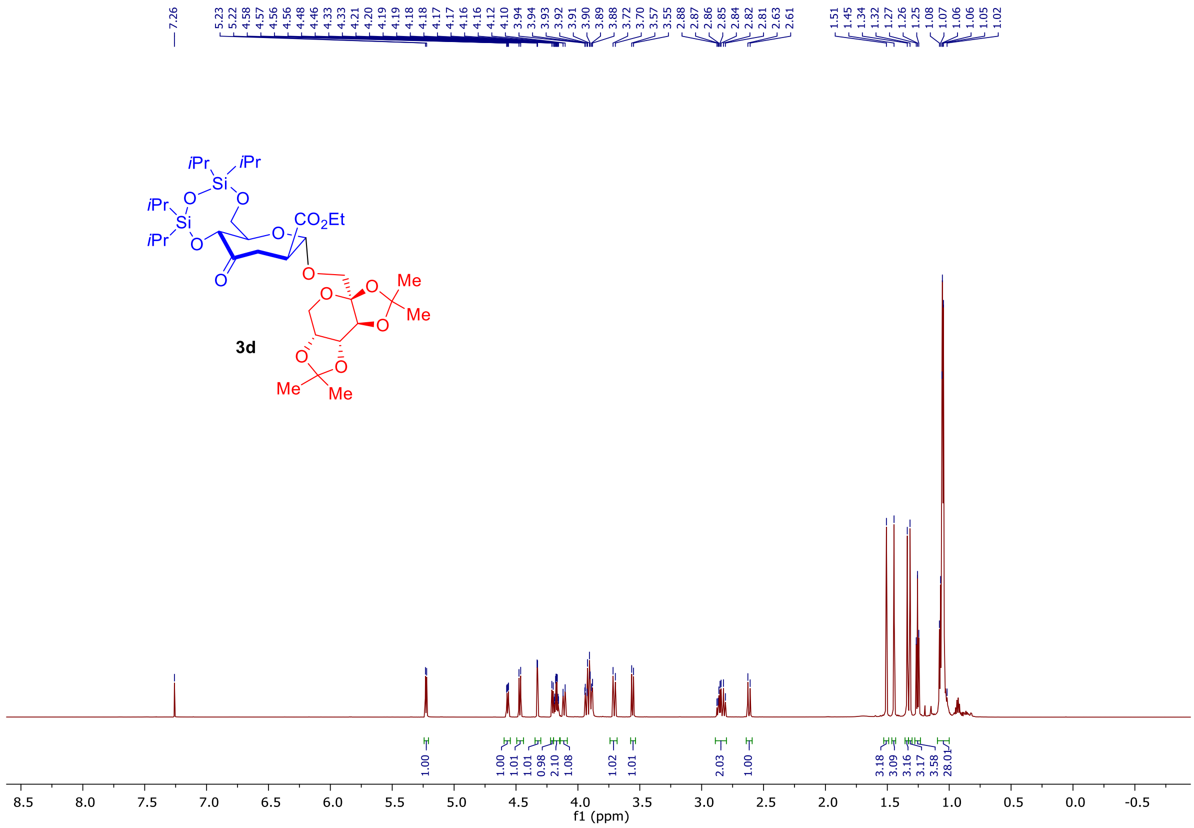

Supplementary Figure 195: ^{1}H spectra for **3d**

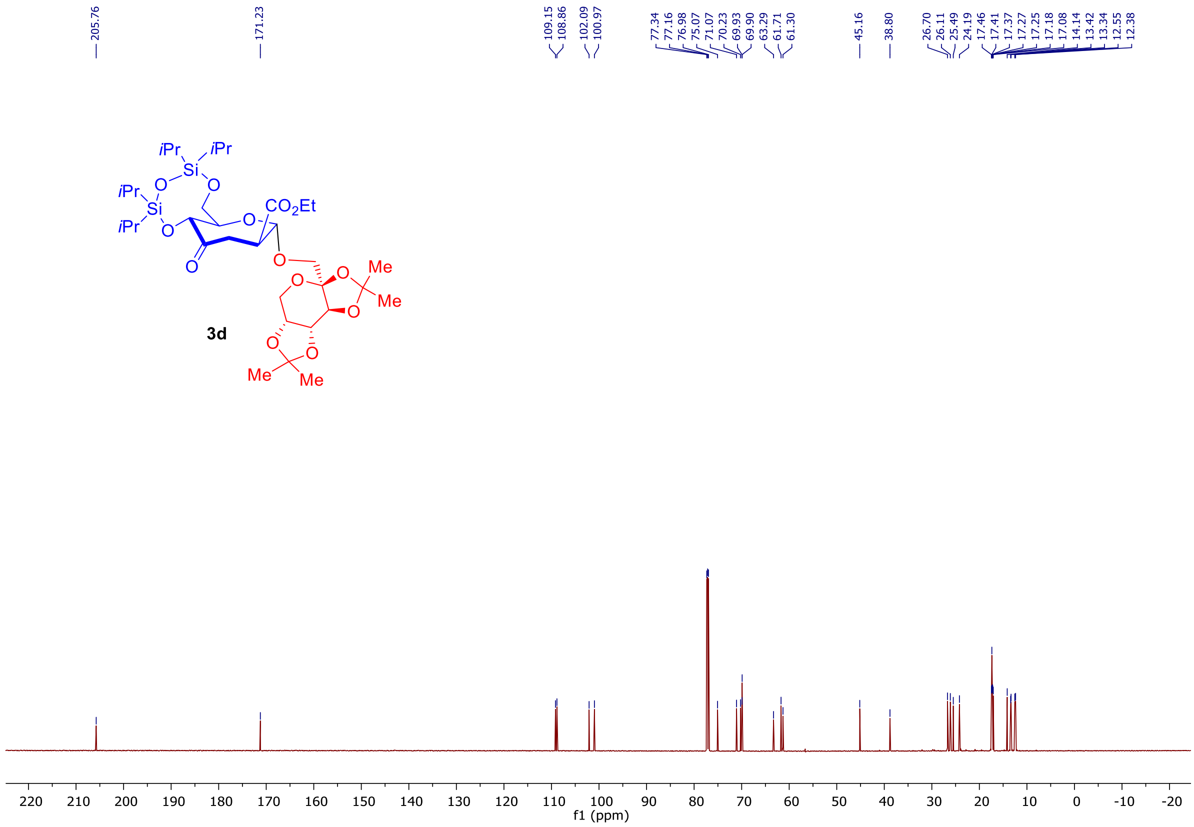

Supplementary Figure 196: ^{13}C spectra for **3d**

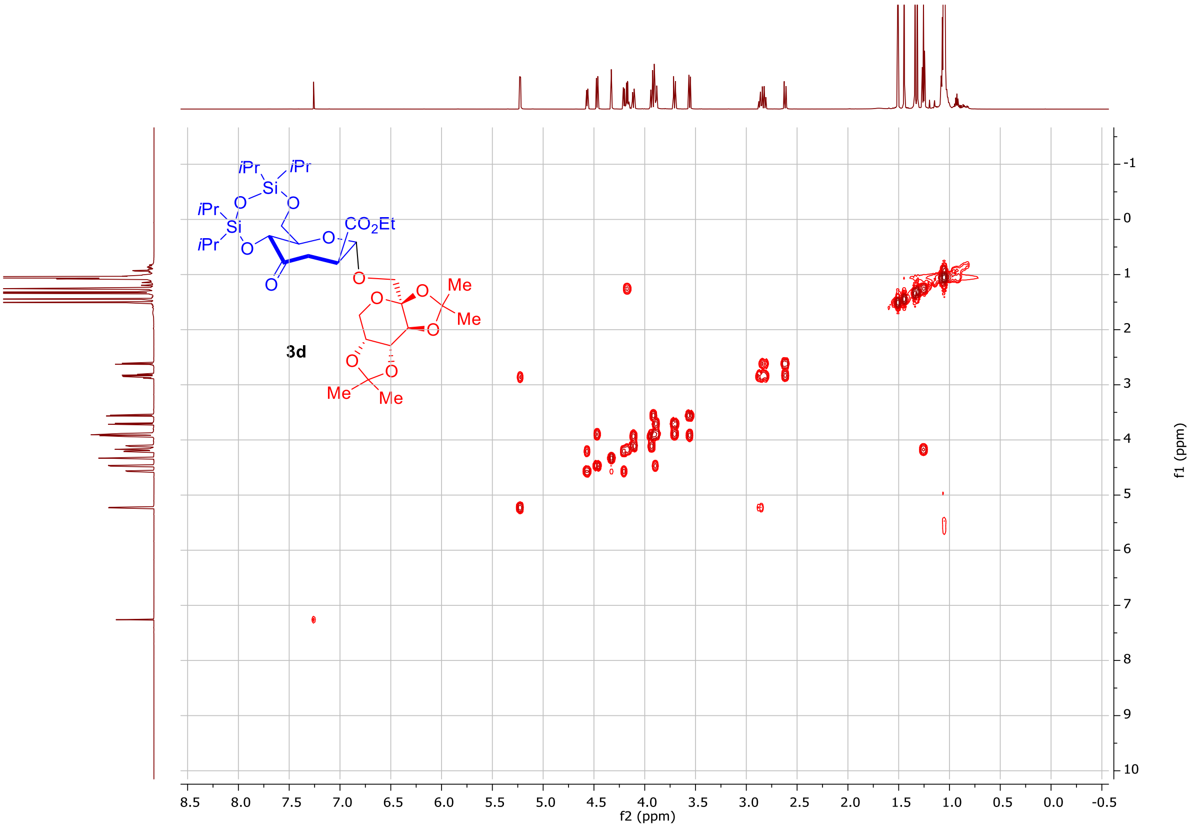

Supplementary Figure 197: COSY spectra for compound **3d**

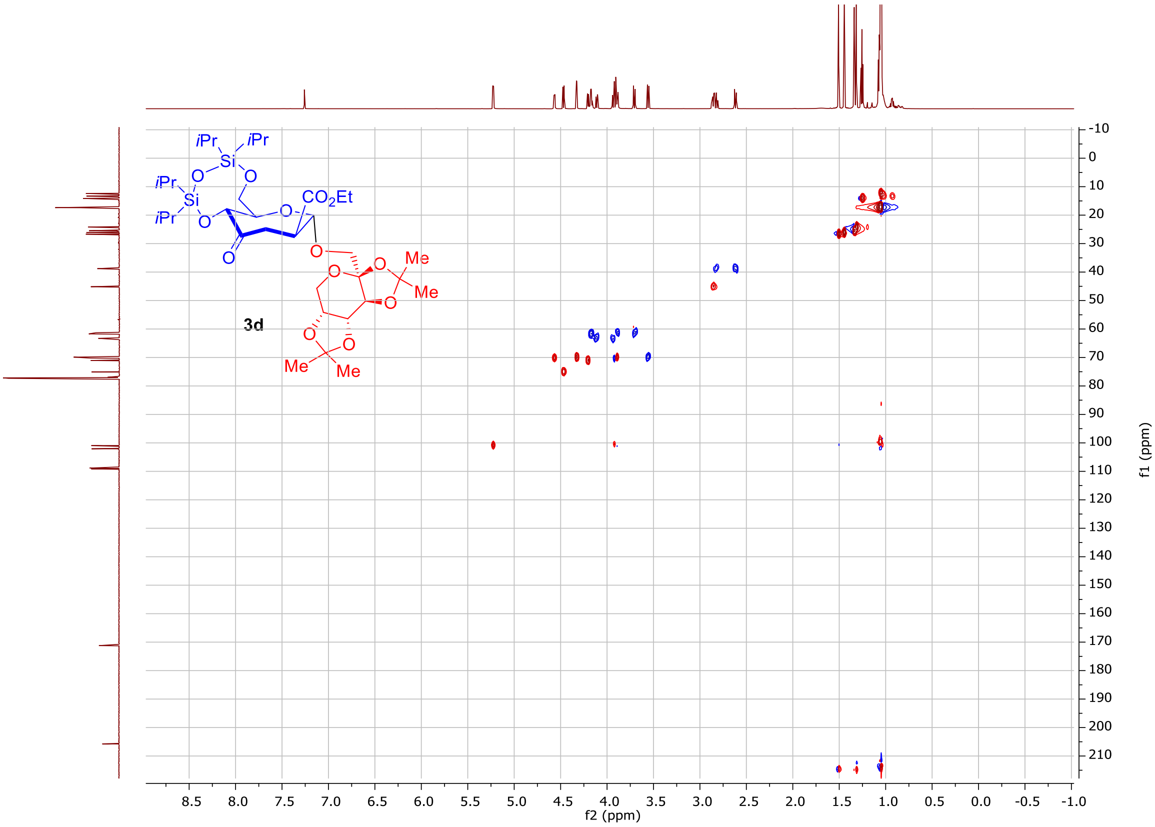

Supplementary Figure 198: HSQC spectra for compound **3d**

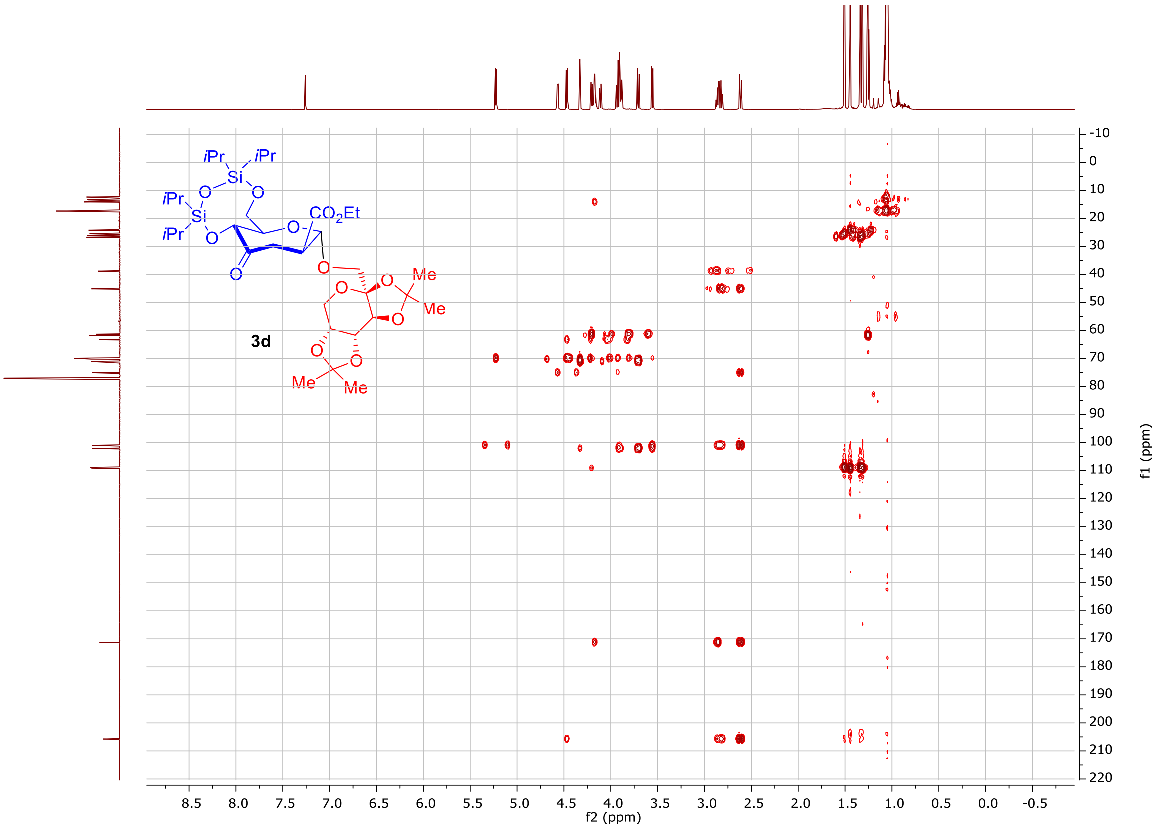

Supplementary Figure 199: HMBC spectra for compound **3d**

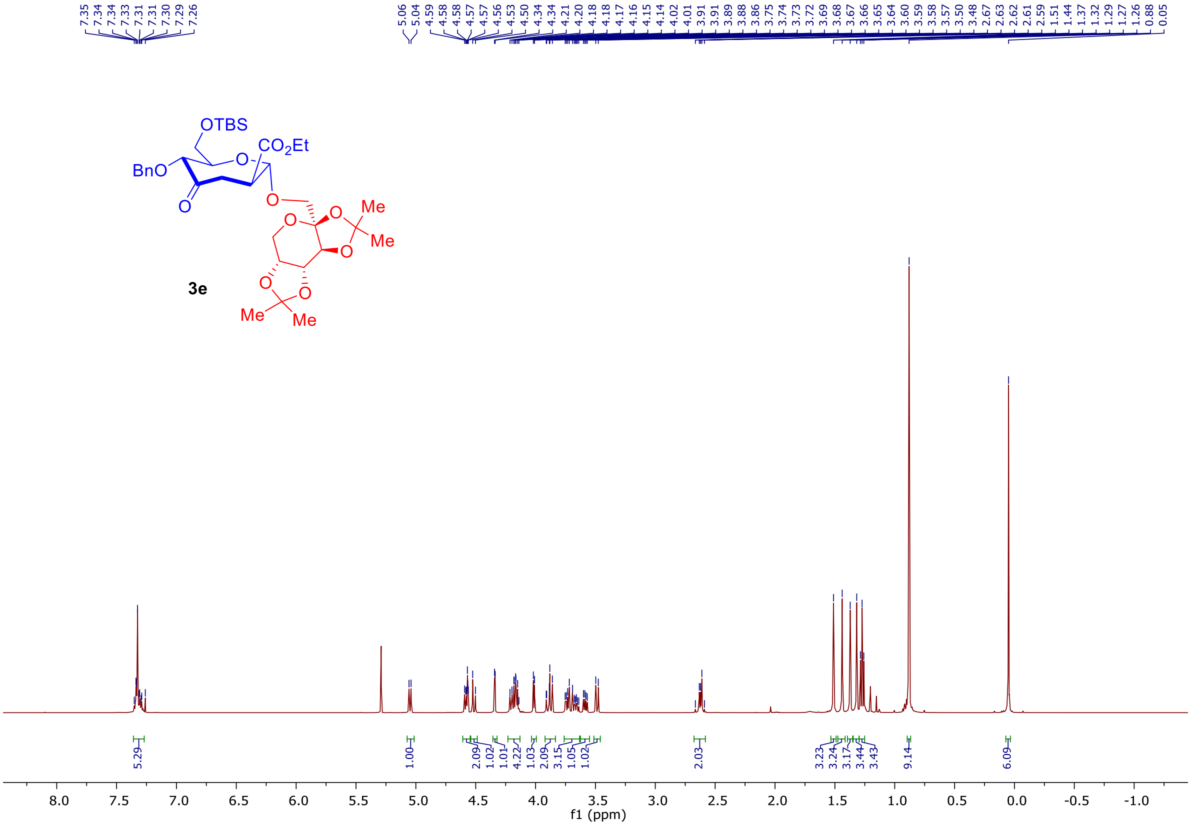

Supplementary Figure 200: ^{1}H spectra for **3e**

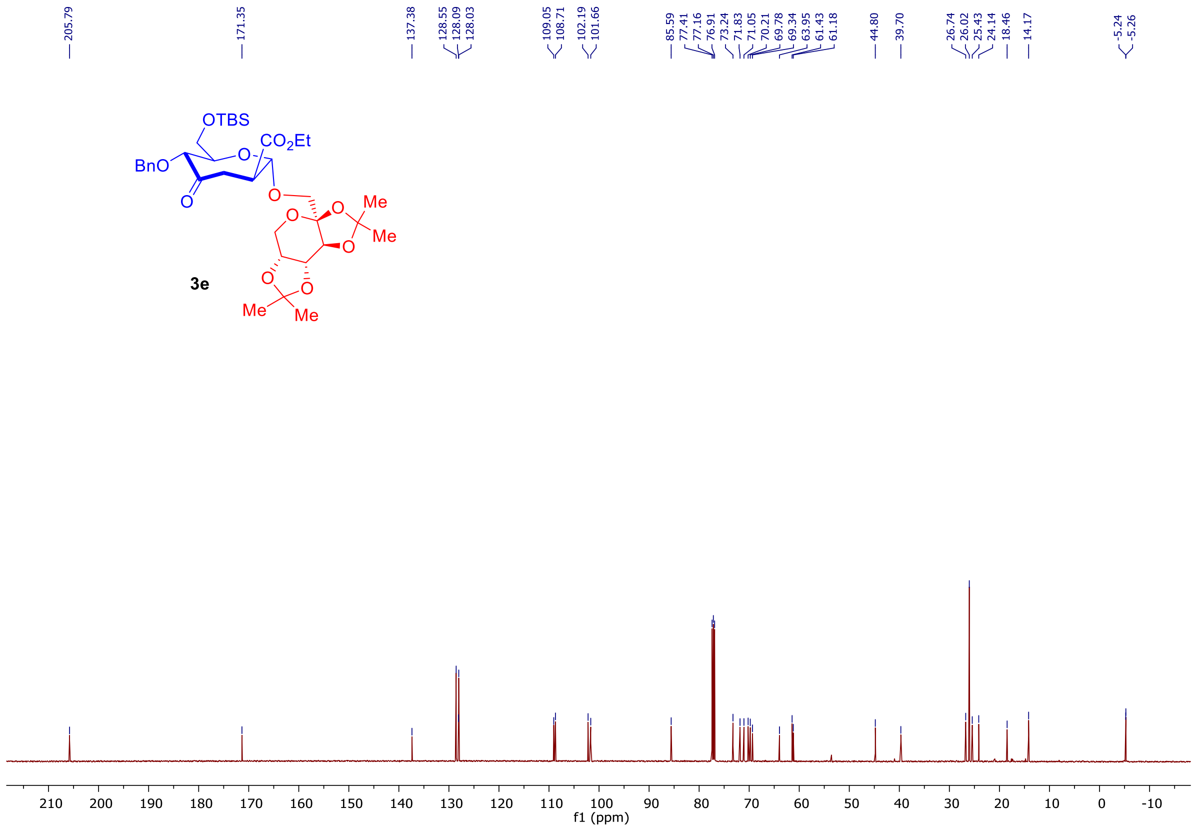

Supplementary Figure 201: ^{13}C spectra for **3e**

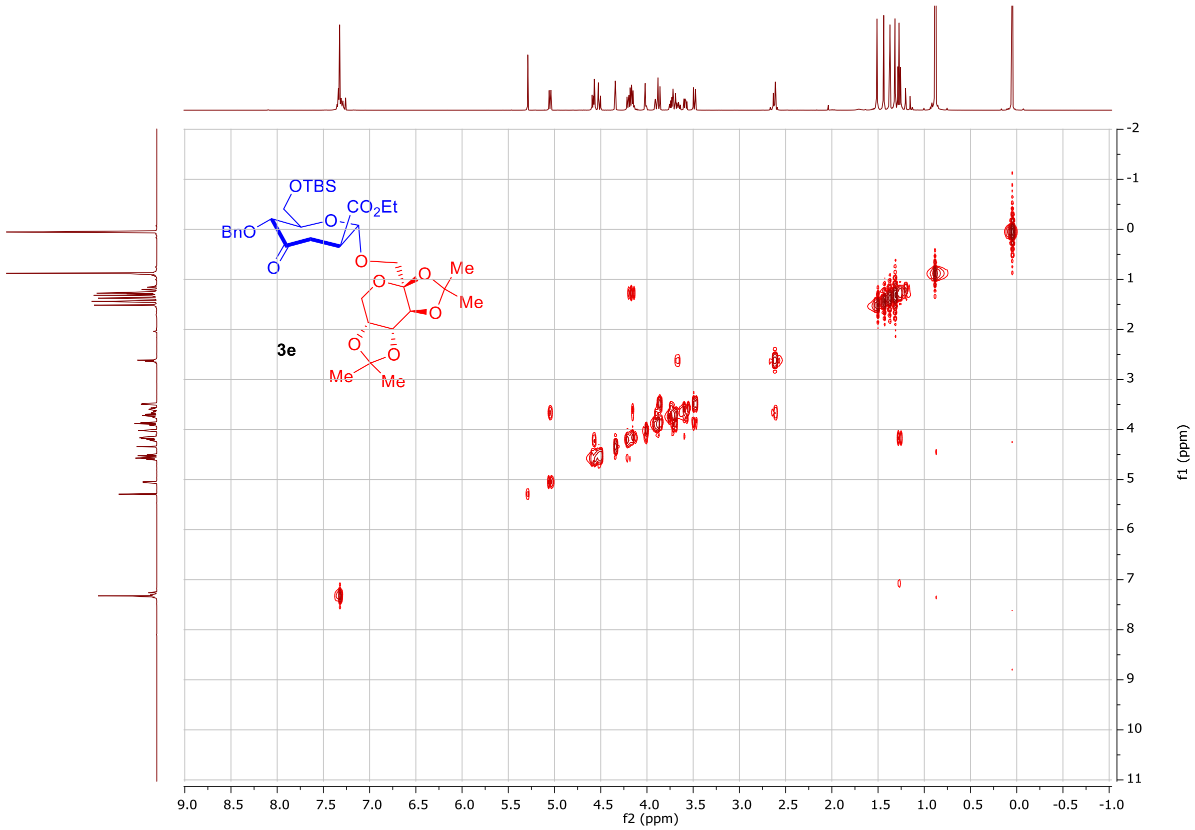

Supplementary Figure 202: COSY spectra for compound **3e**

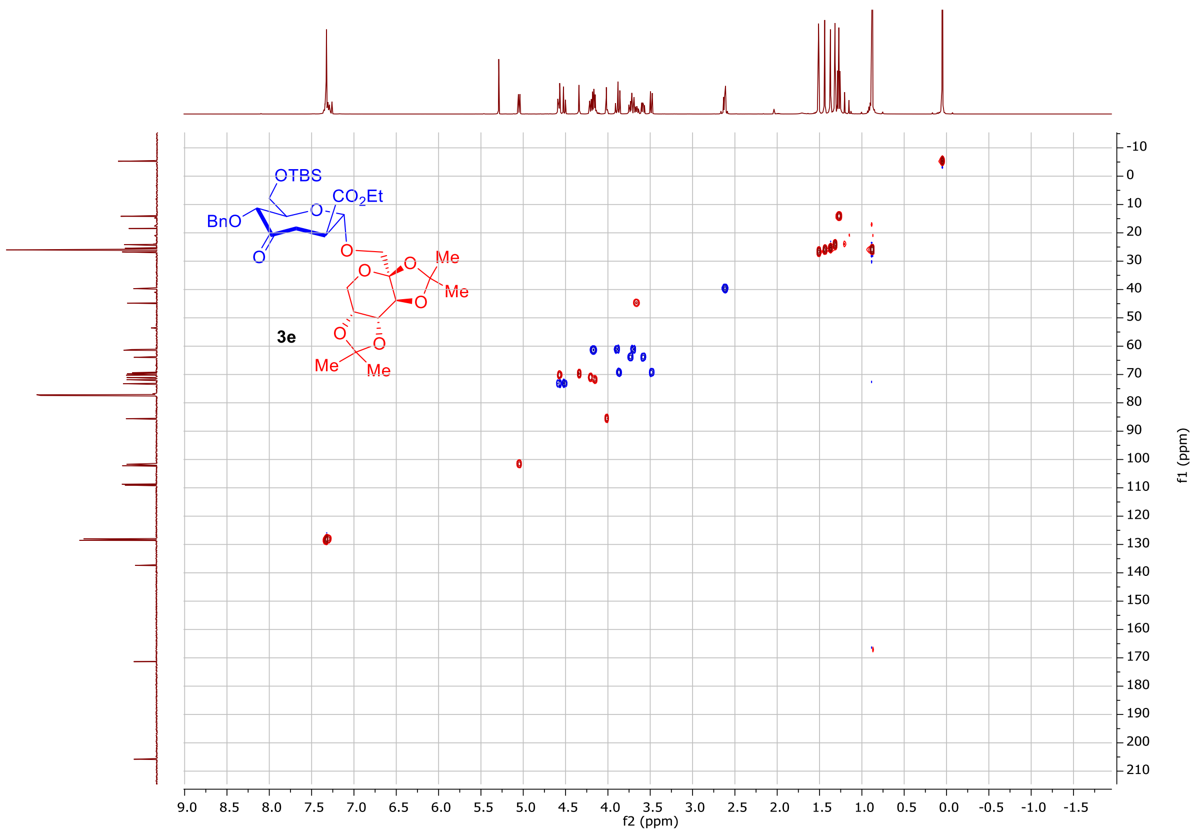

Supplementary Figure 203: HSQC spectra for compound **3e**

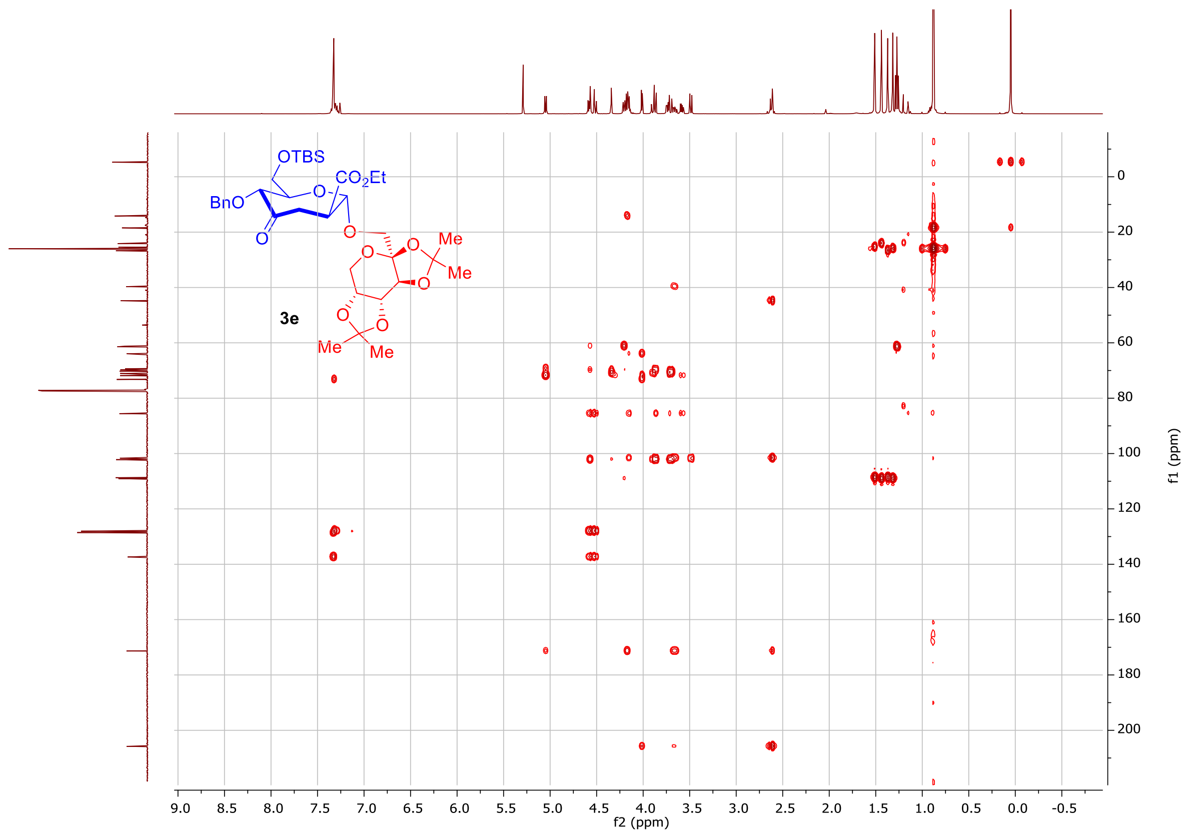

Supplementary Figure 204: HMBC spectra for compound **3e**

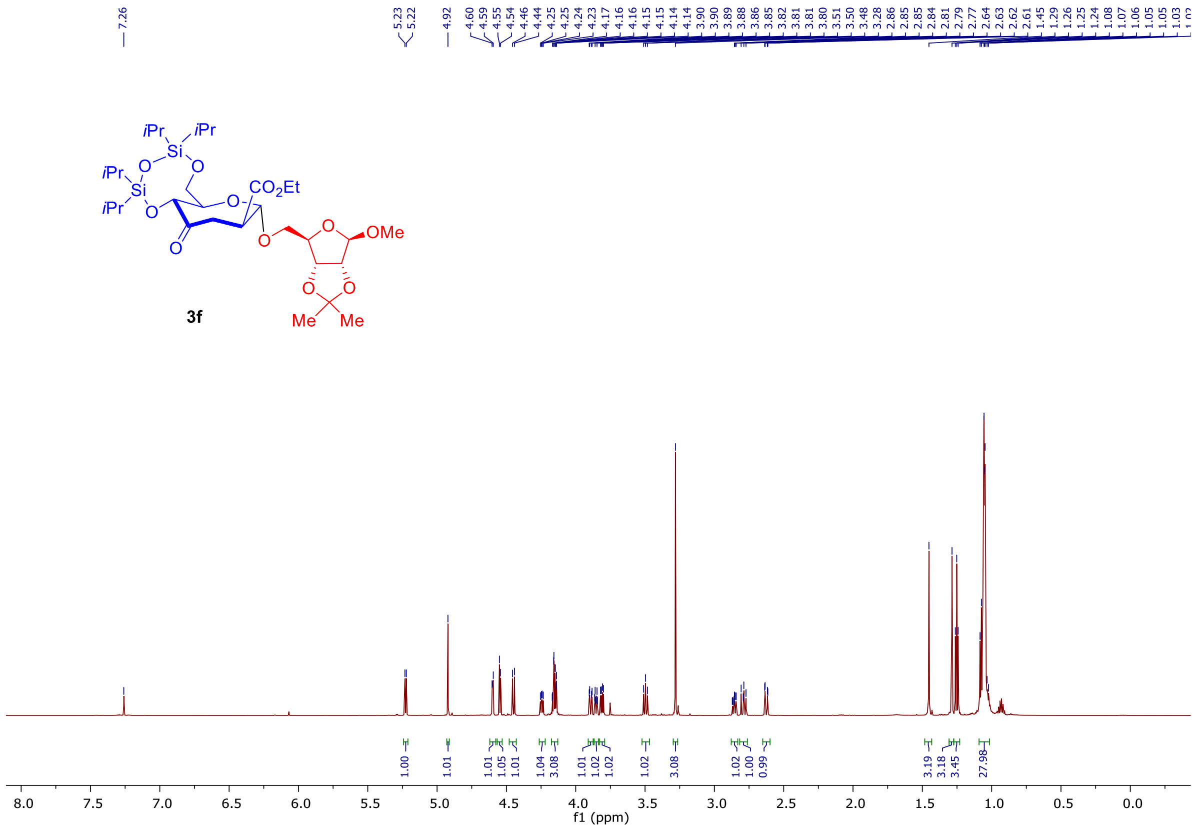

Supplementary Figure 205: ^{1}H spectra for **3f**

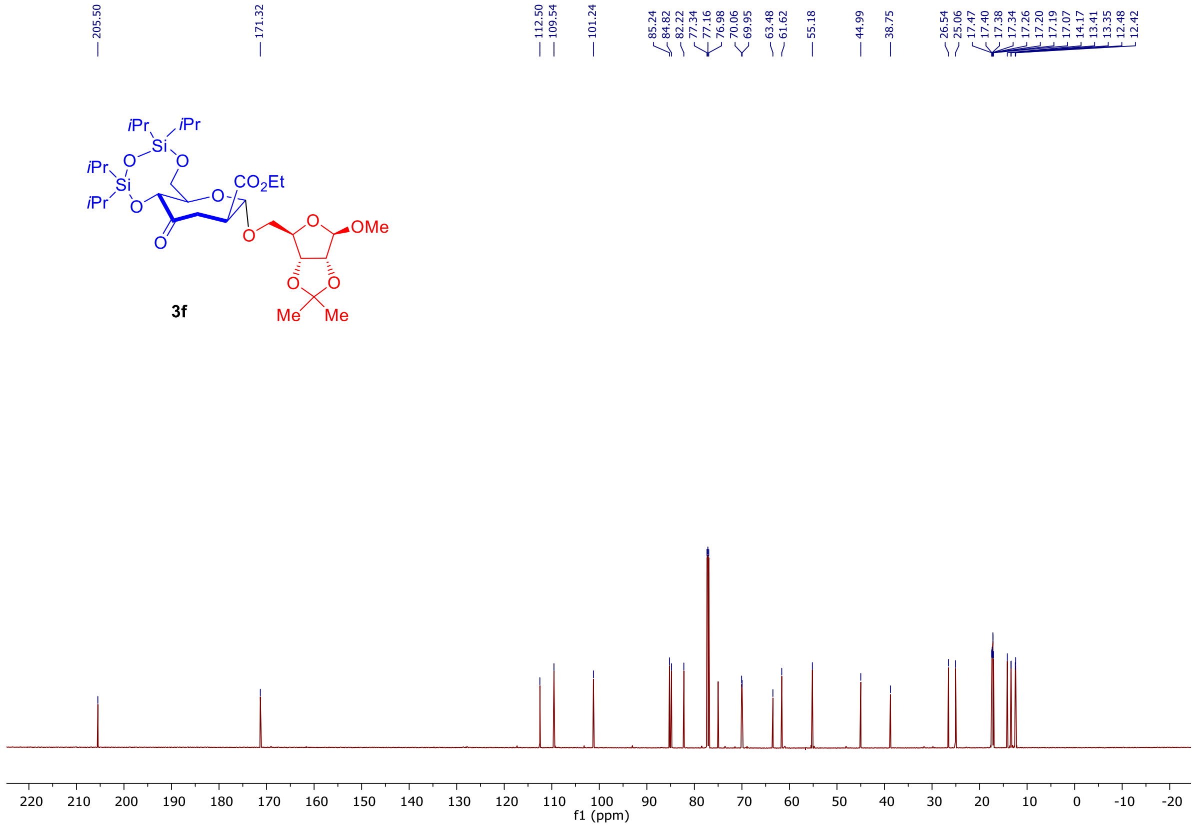

Supplementary Figure 206: ^{13}C spectra for **3f**

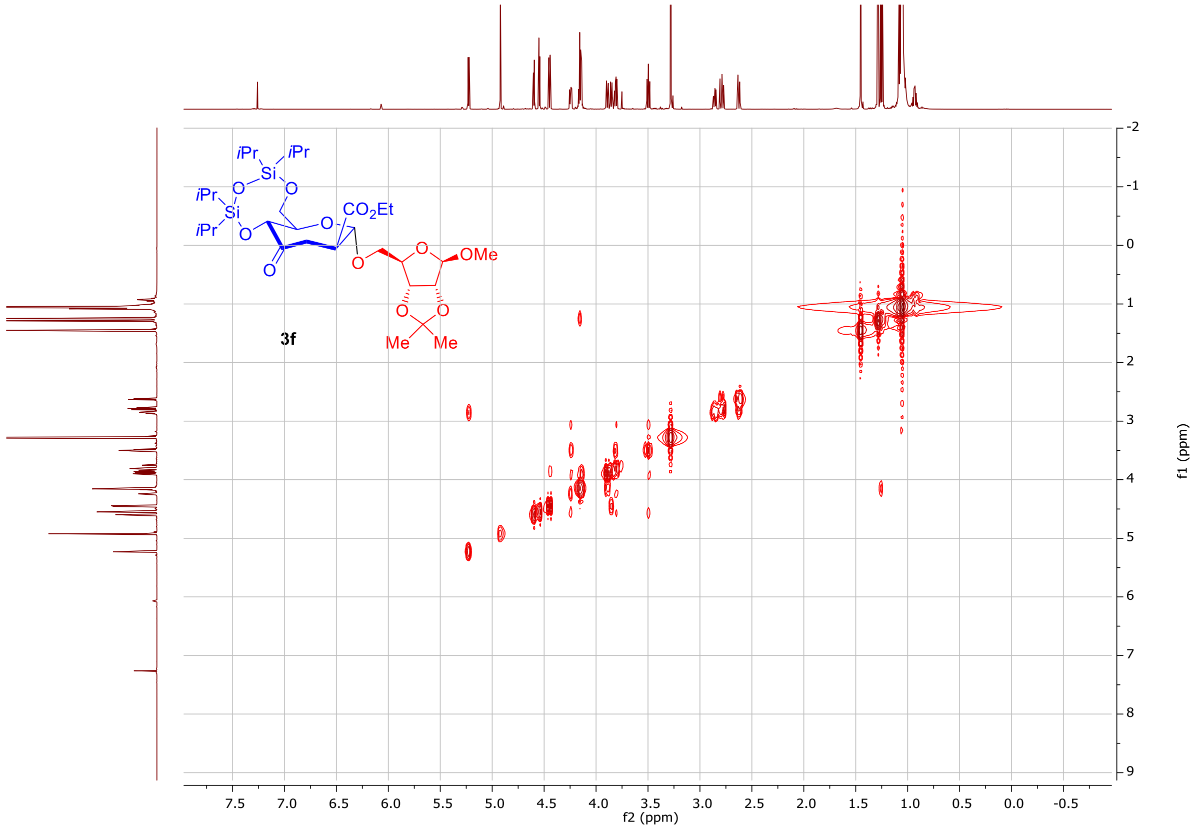

Supplementary Figure 207: COSY spectra for compound **3f**

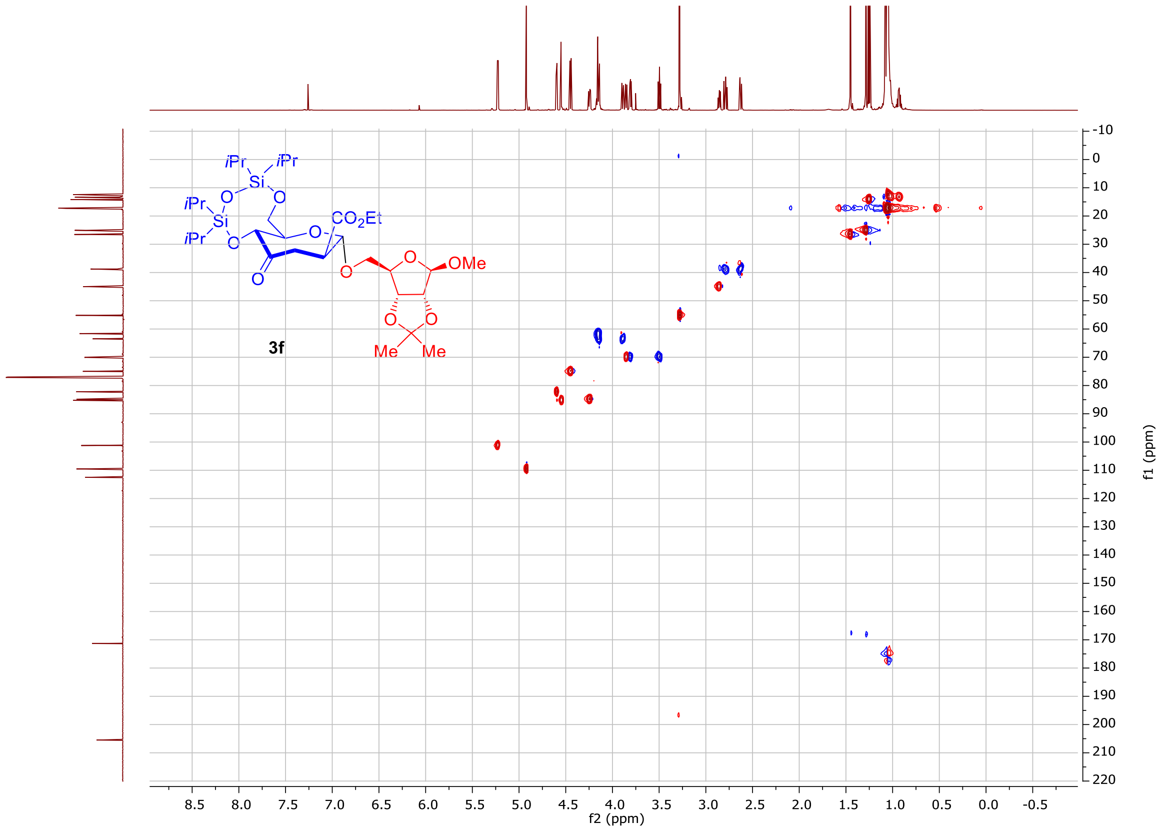

Supplementary Figure 208: HSQC spectra for compound **3f**

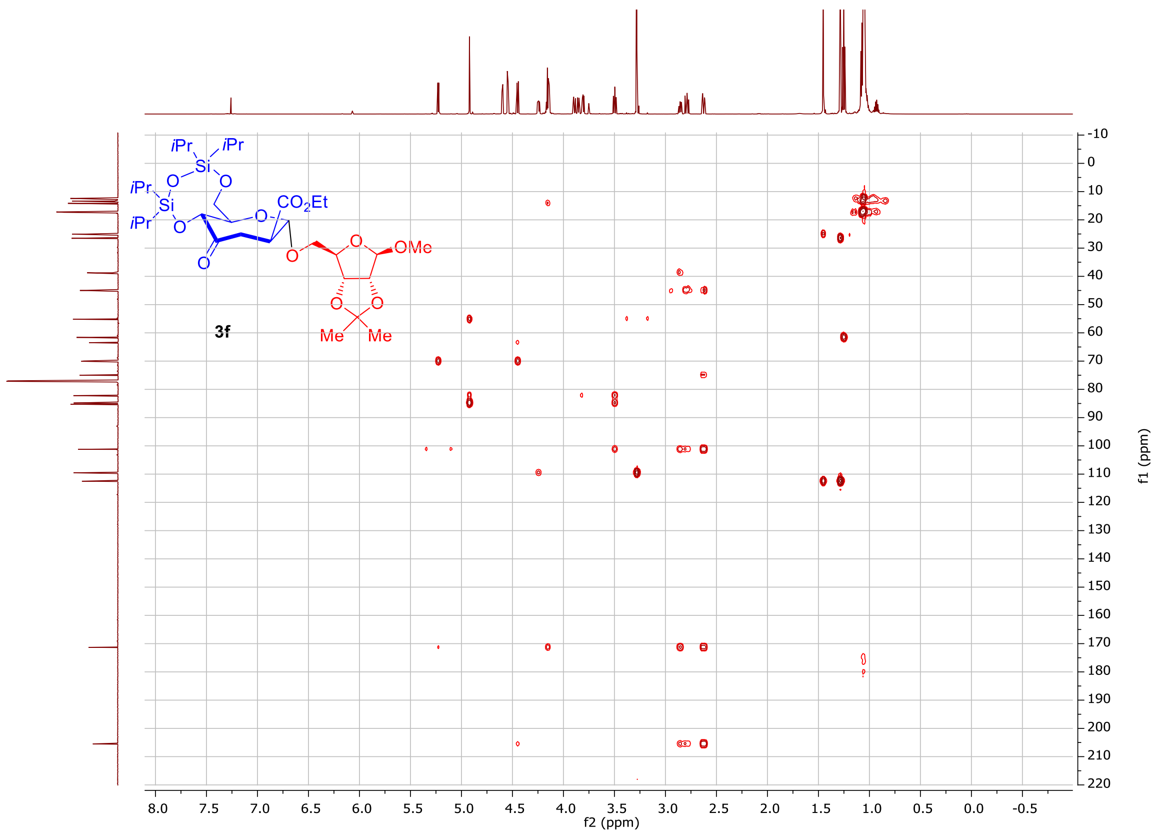

Supplementary Figure 209: HMBC spectra for compound **3f**

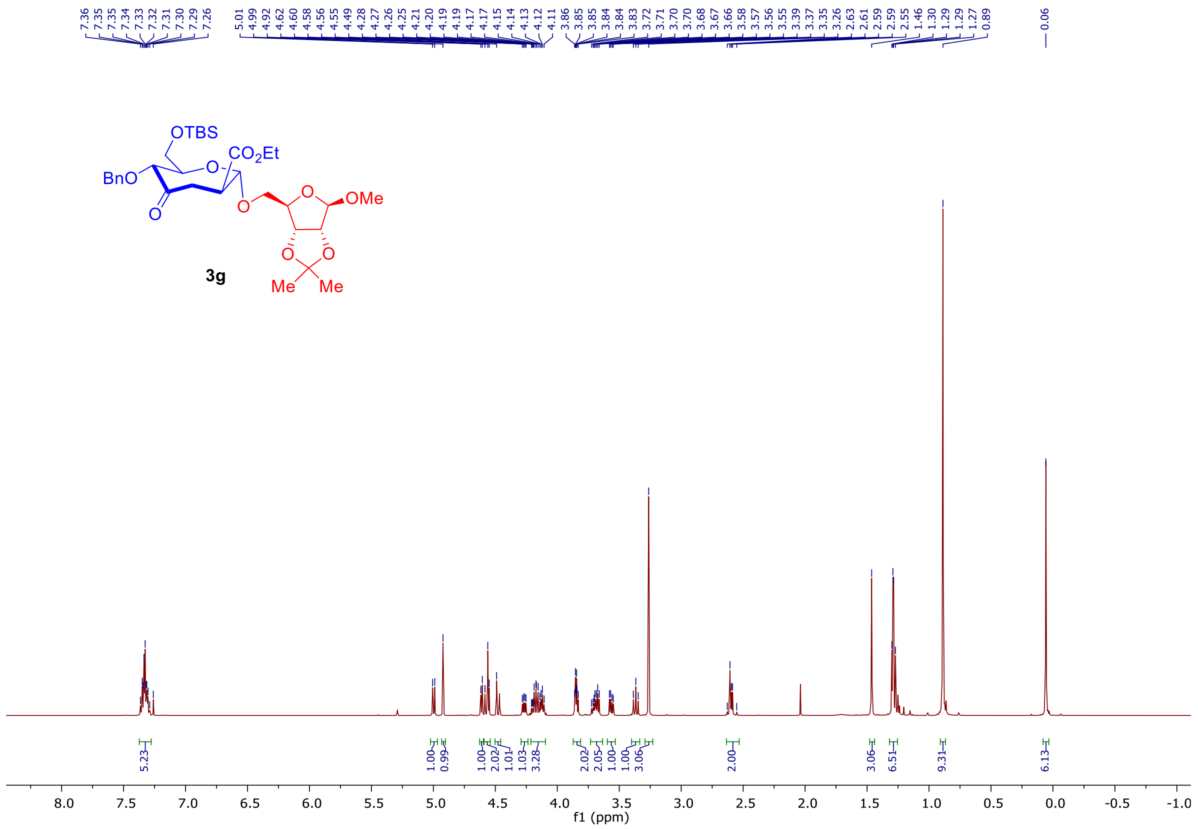

Supplementary Figure 210: ^{1}H spectra for **3g**

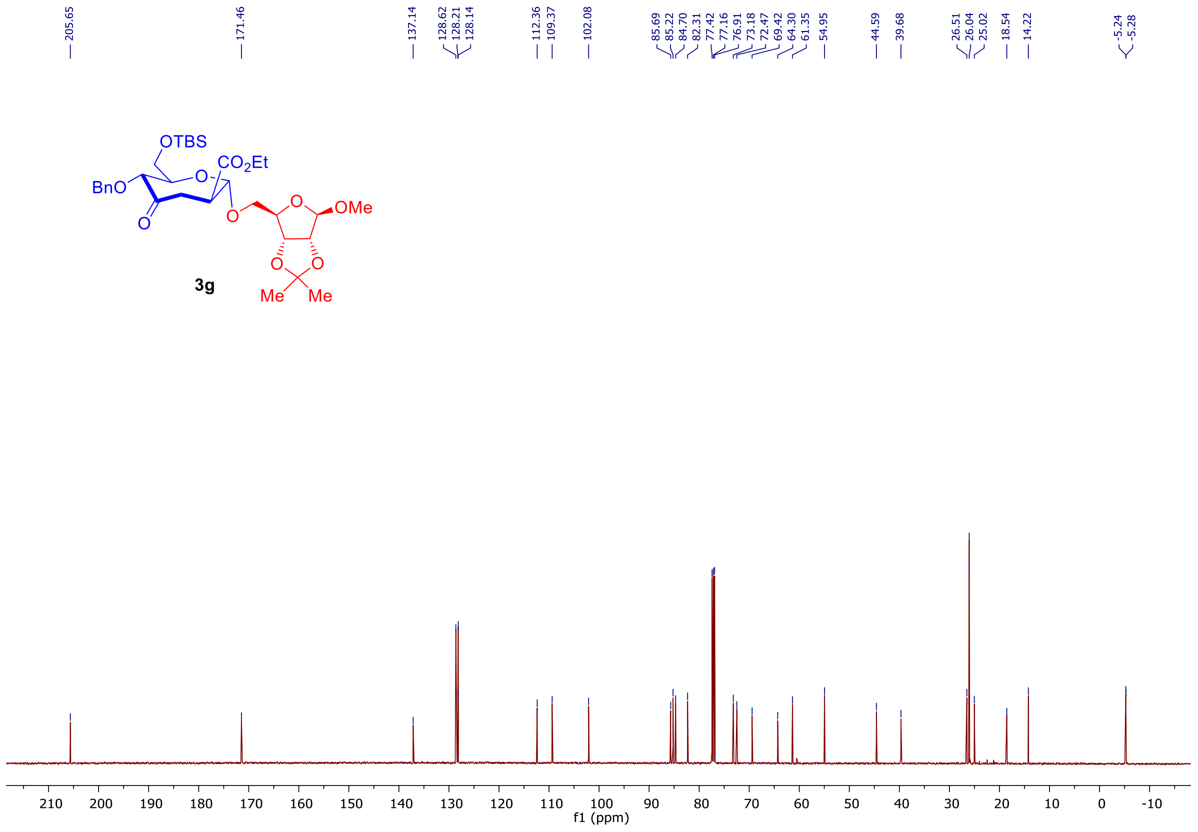

Supplementary Figure 211: ^{13}C spectra for **3g**

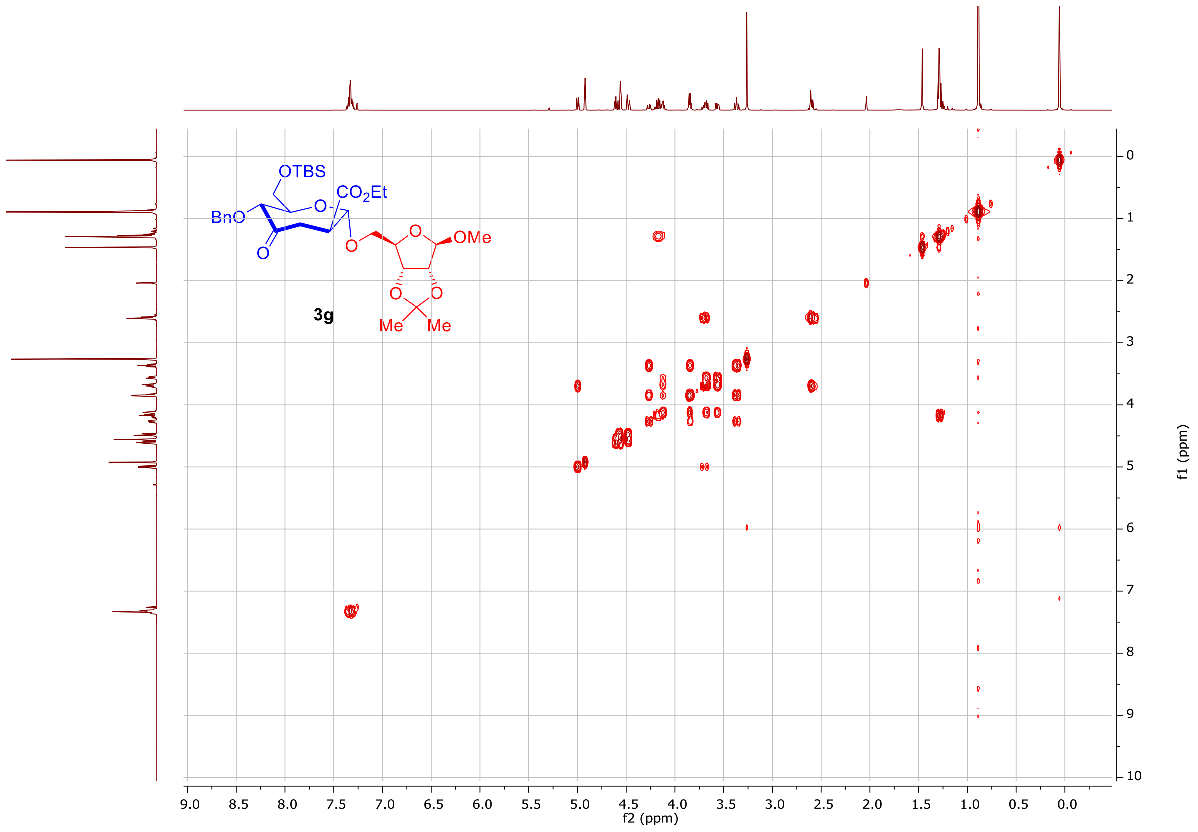

Supplementary Figure 212: COSY spectra for compound **3g**

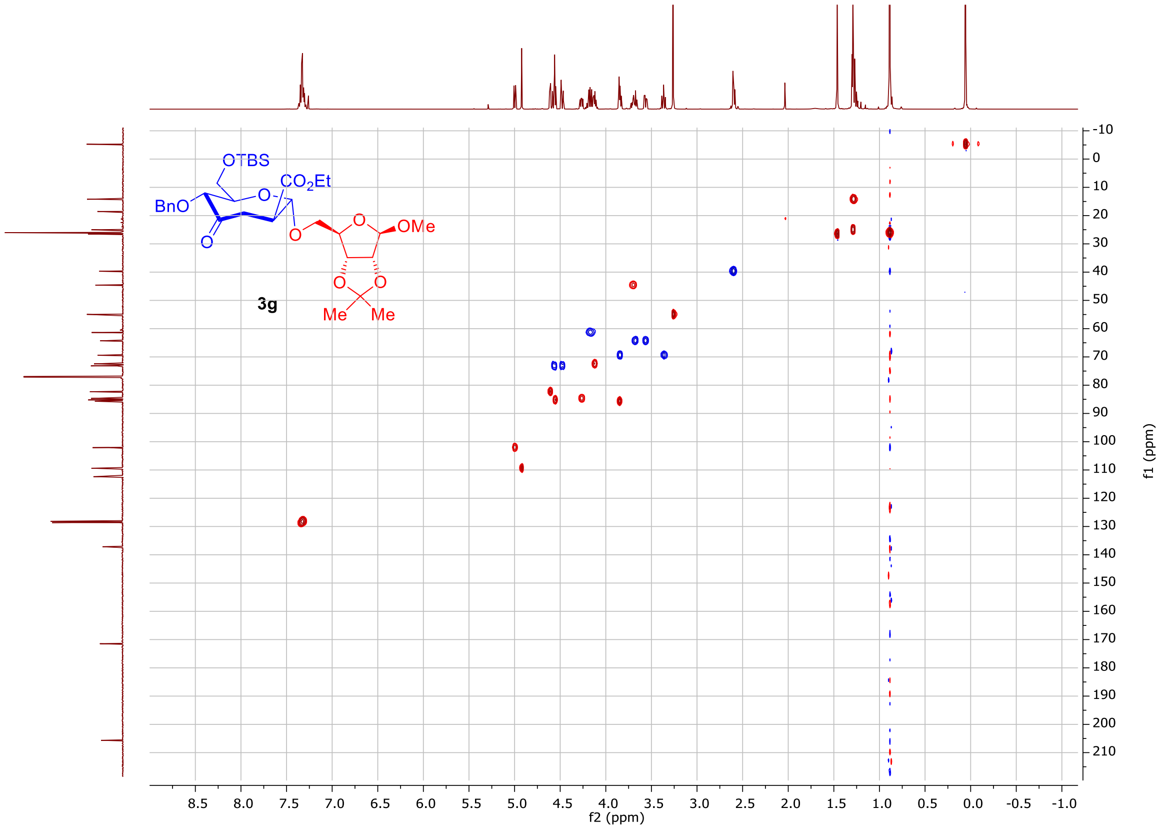

Supplementary Figure 213: HSQC spectra for compound **3g**

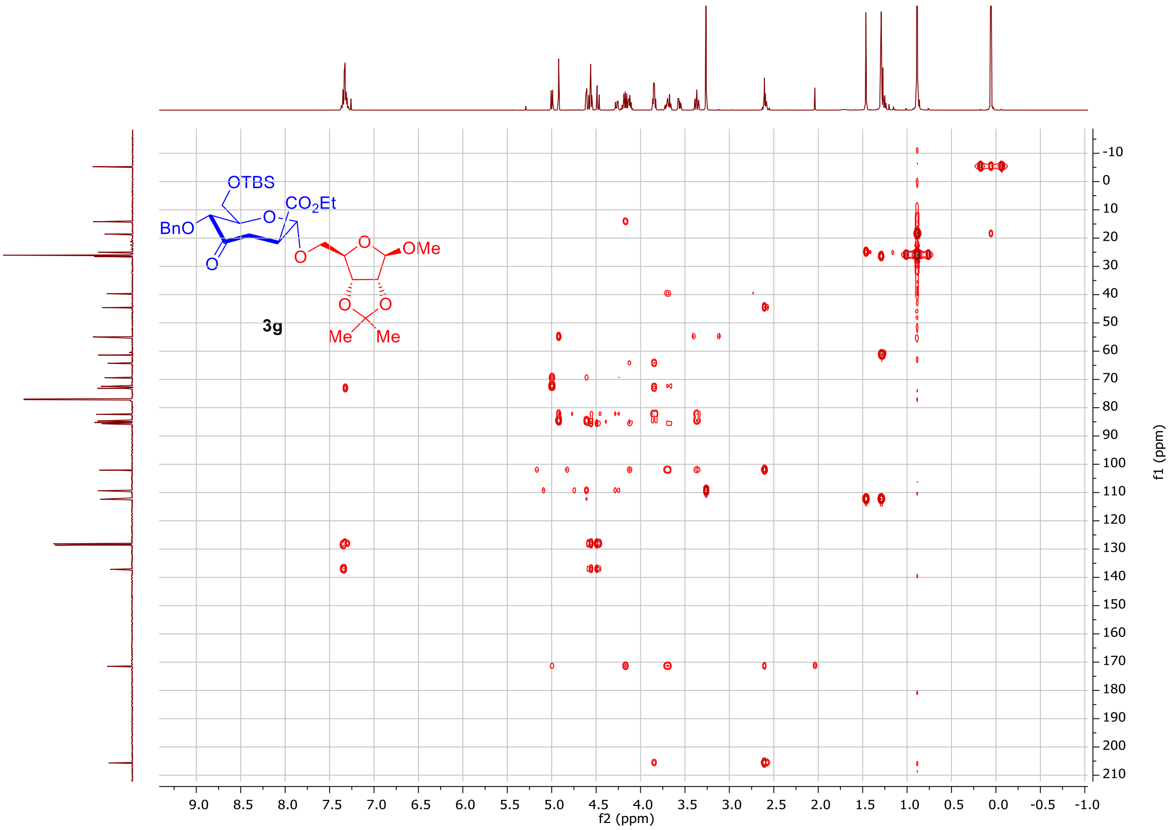

Supplementary Figure 214: HMBC spectra for compound **3g**

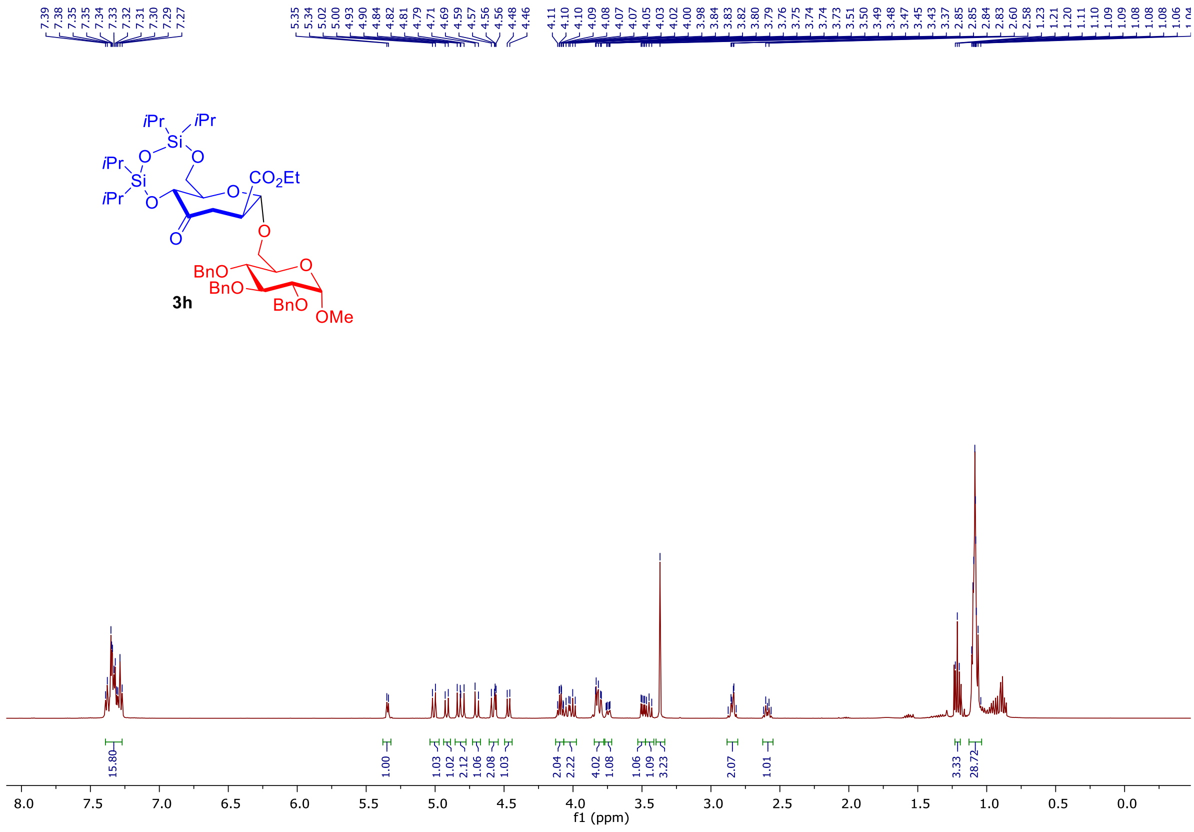

Supplementary Figure 215: ^{1}H spectra for **3h**

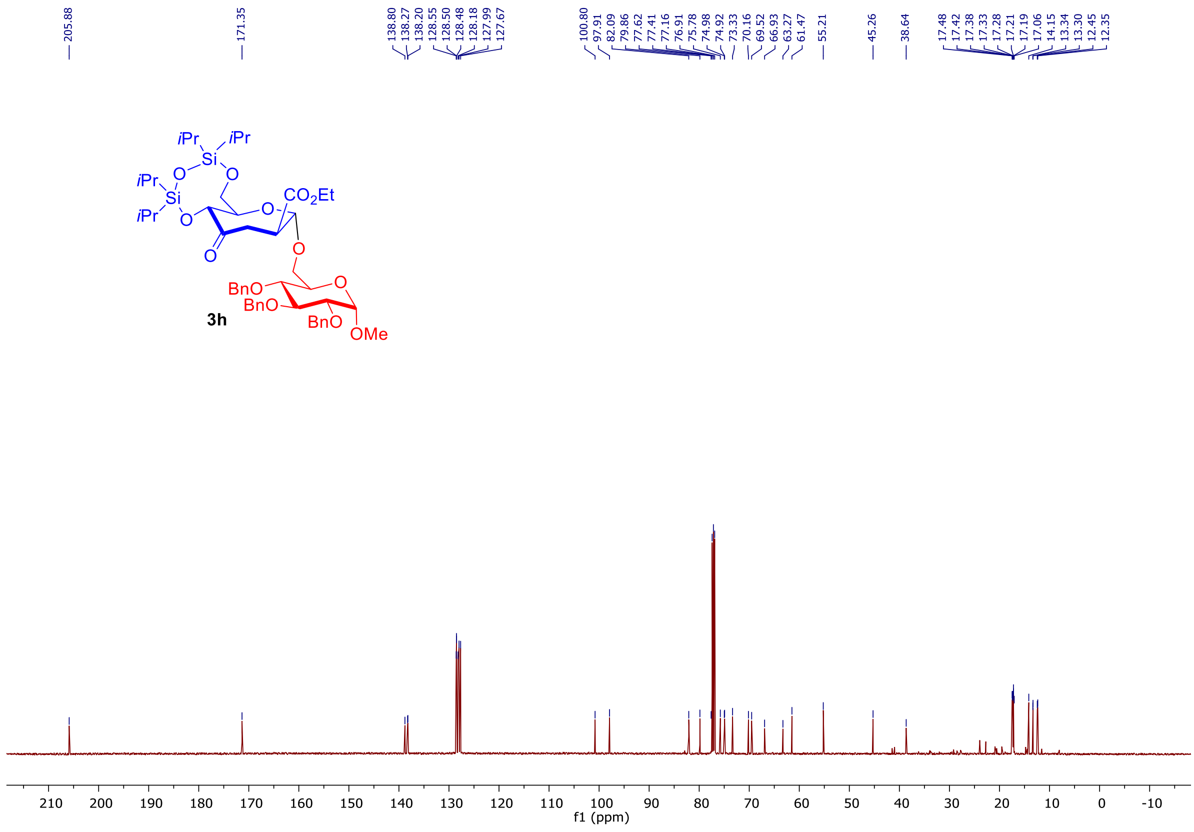

Supplementary Figure 216: ^{13}C spectra for **3h**

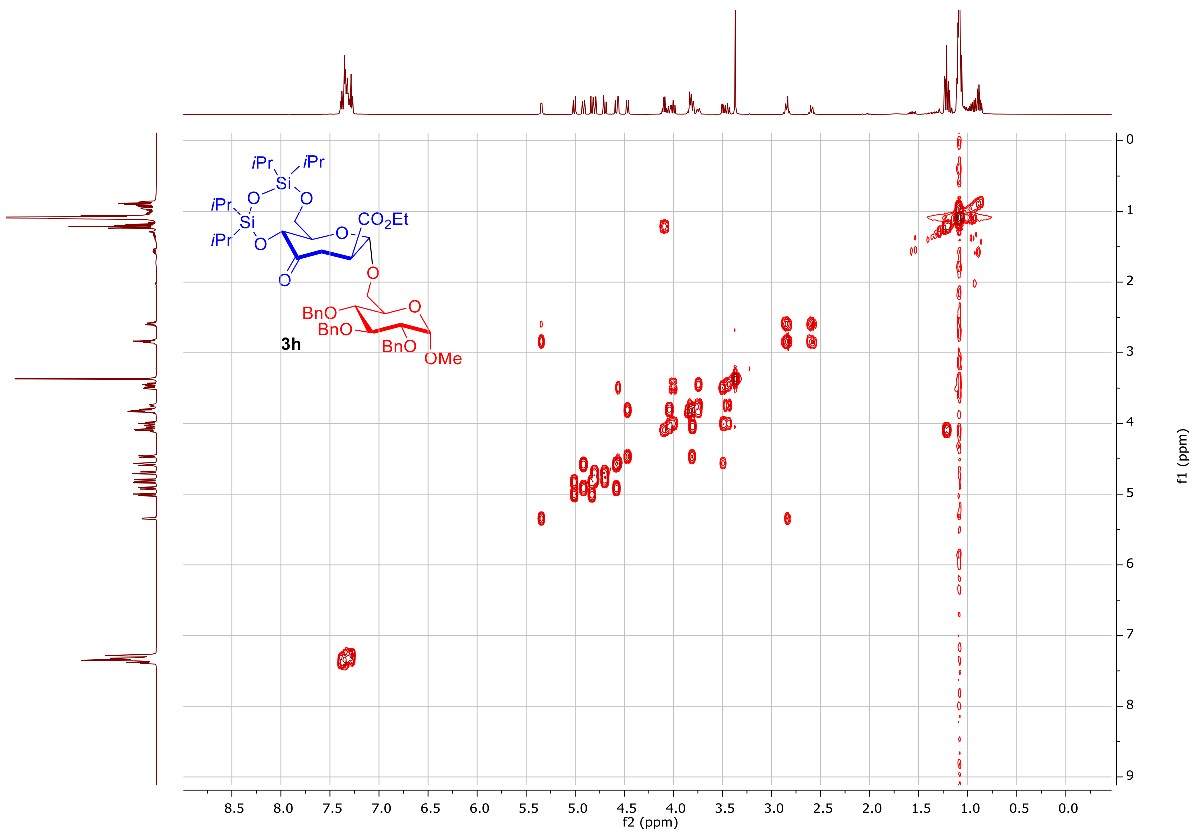

Supplementary Figure 217: COSY spectra for compound **3h**

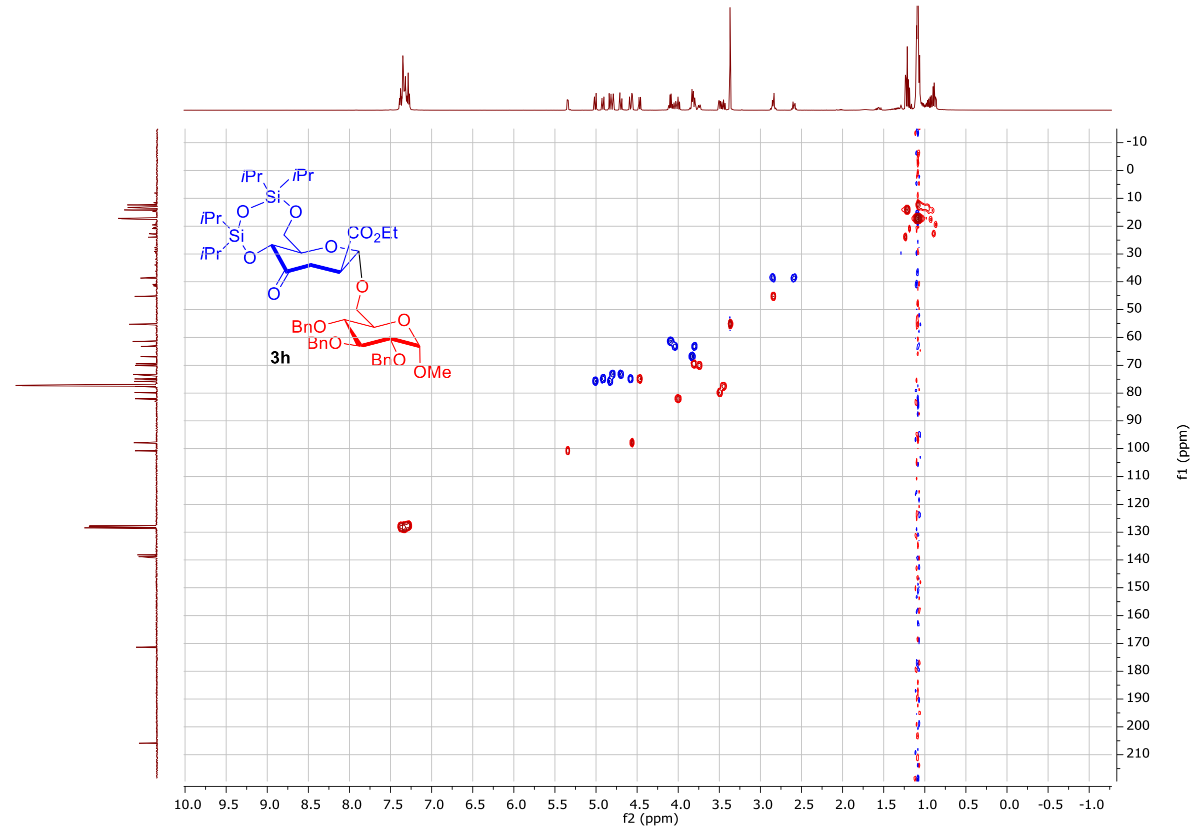

Supplementary Figure 218: HSQC spectra for compound **3**h

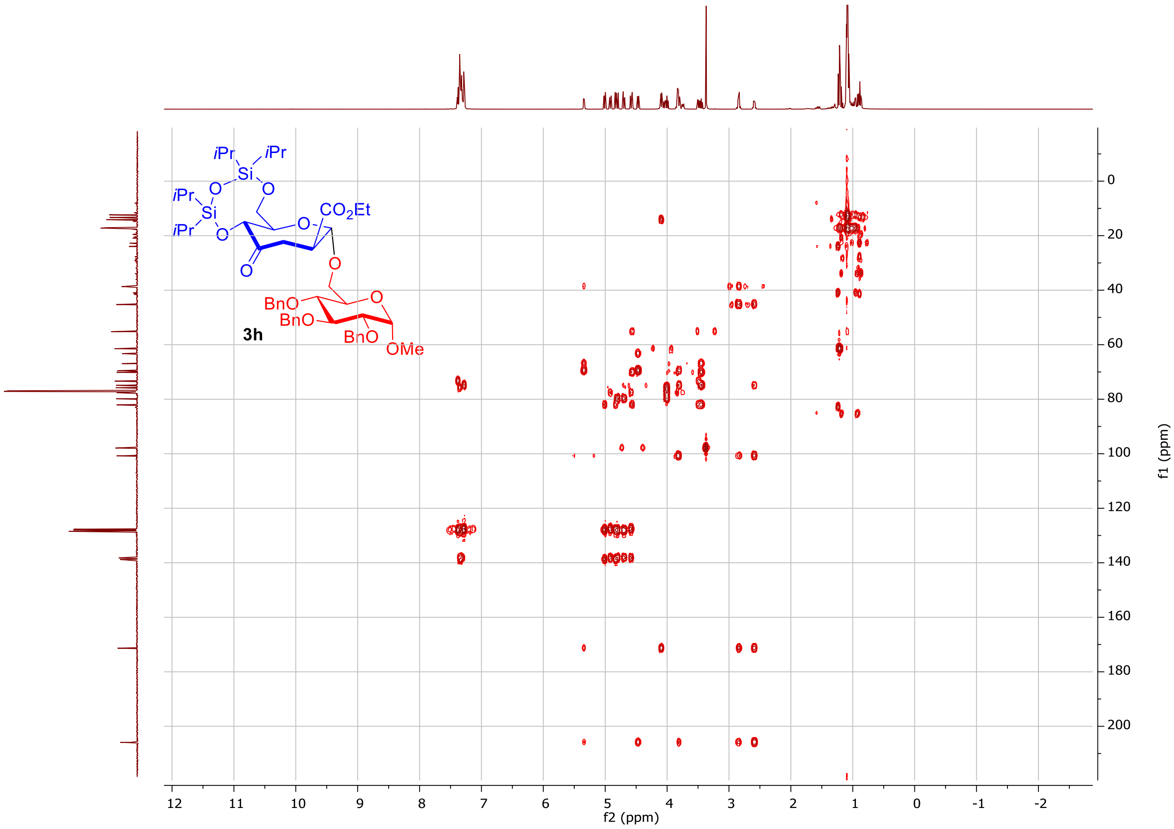

Supplementary Figure 219: HMBC spectra for compound **3h**

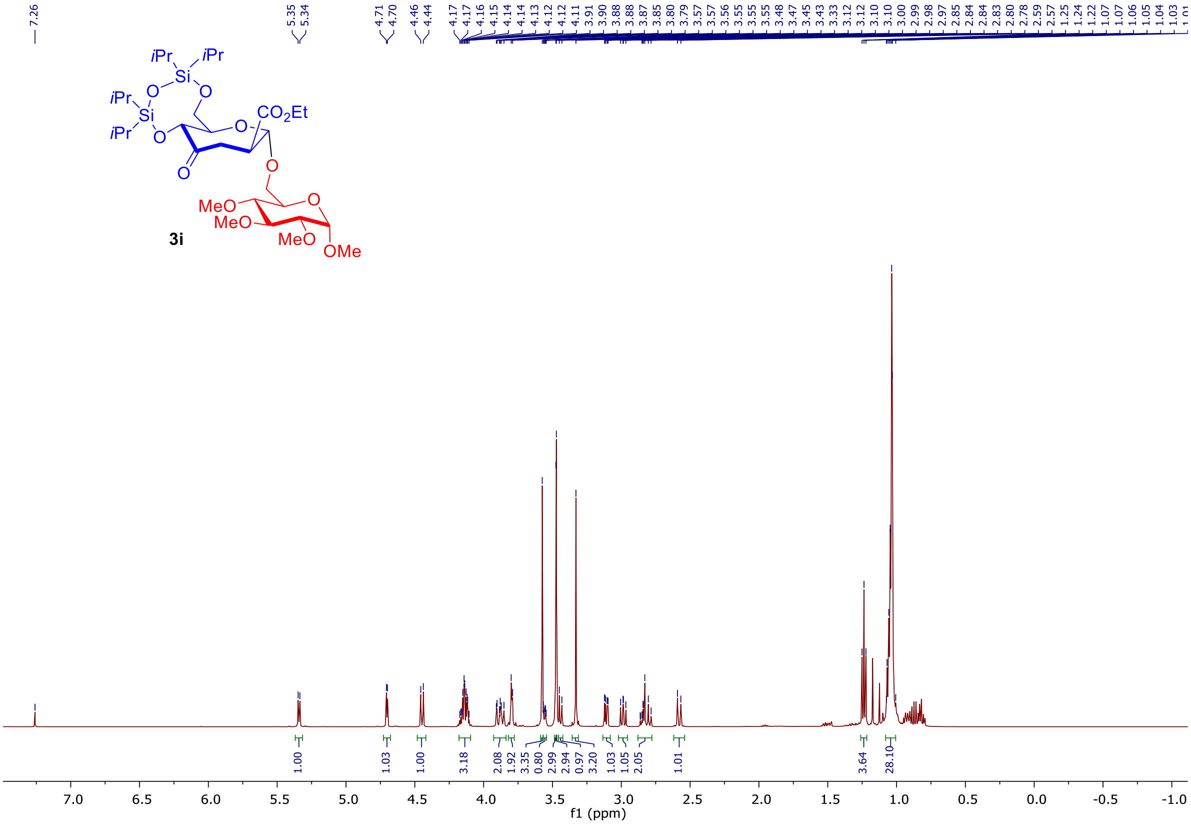

Supplementary Figure 220: ^{1}H spectra for **3i**

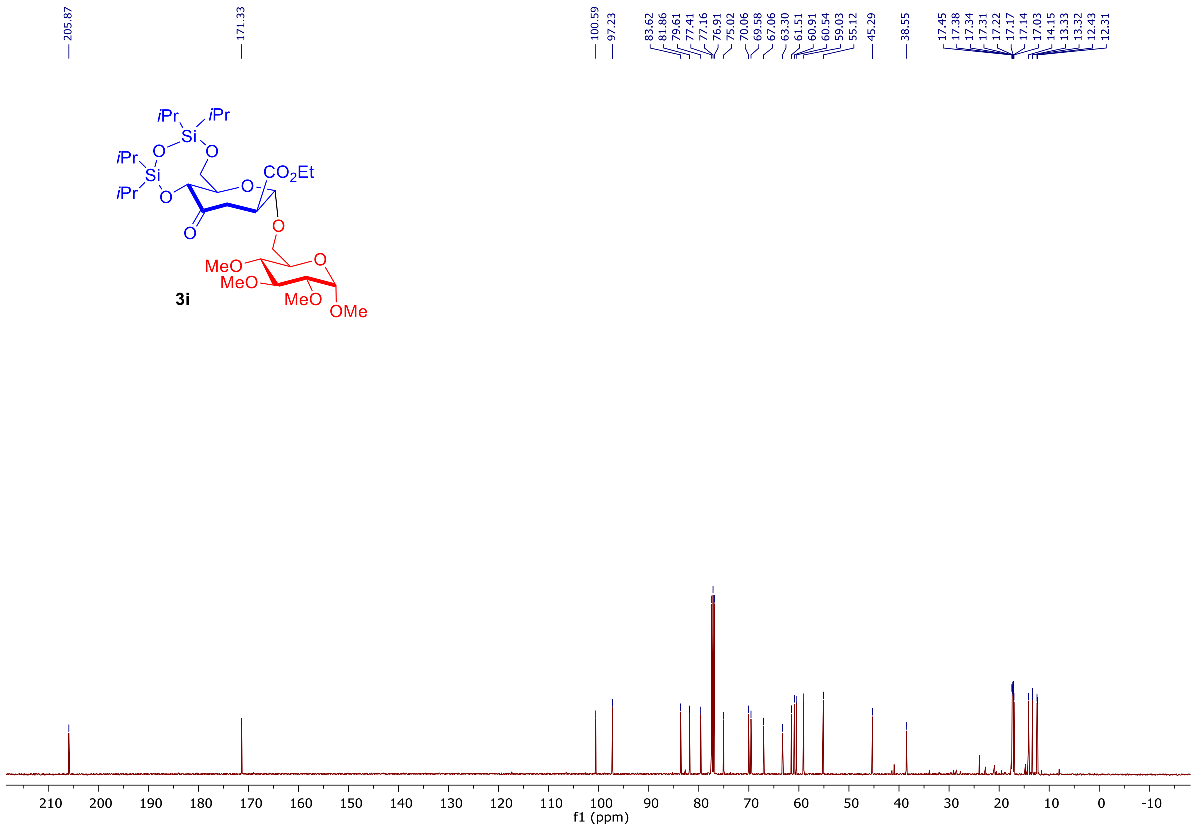

Supplementary Figure 221: ^{13}C spectra for **3i**

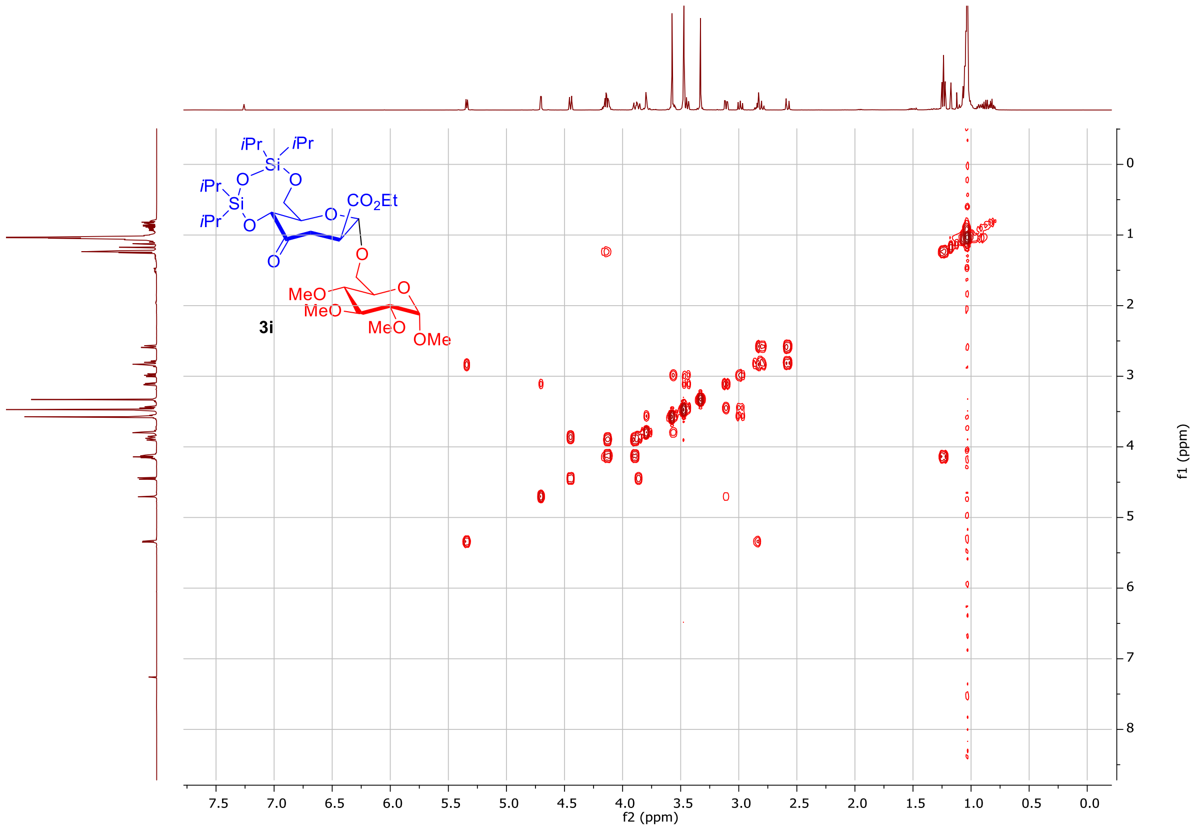

Supplementary Figure 222: COSY spectra for compound **3i**

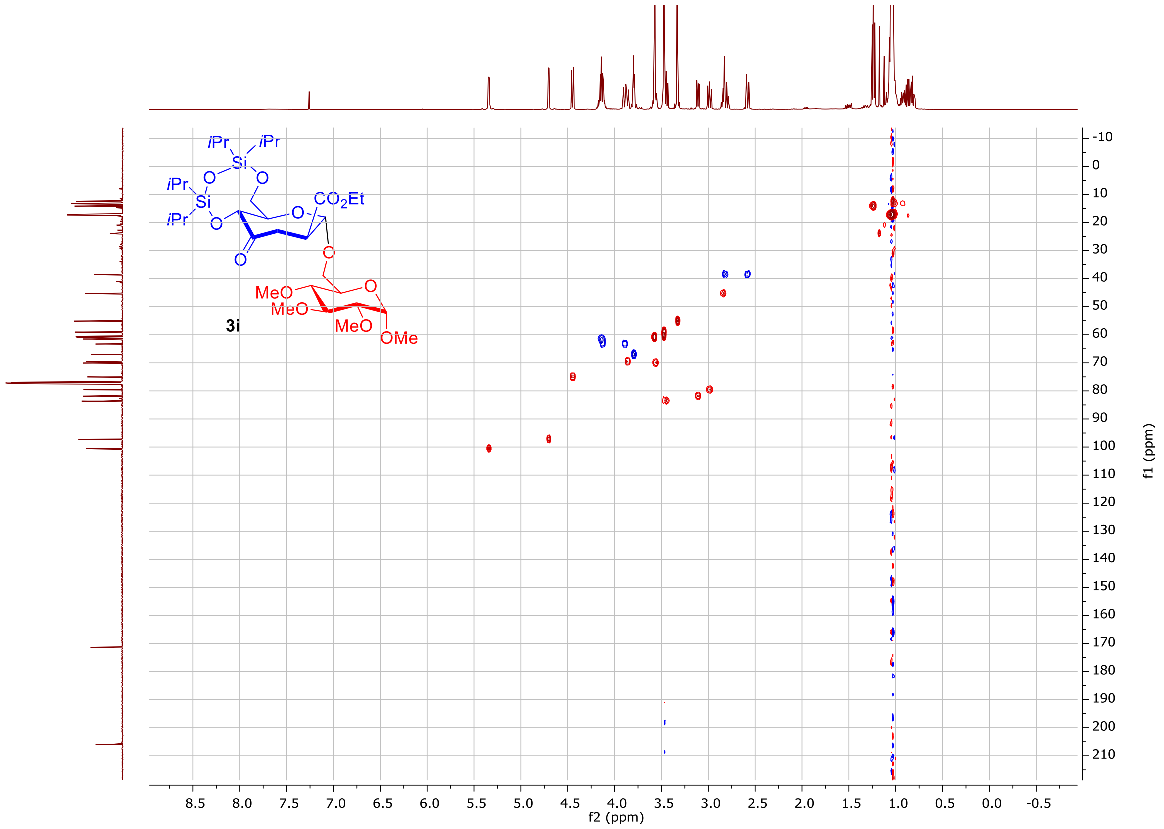

Supplementary Figure 223: HSQC spectra for compound **3i**

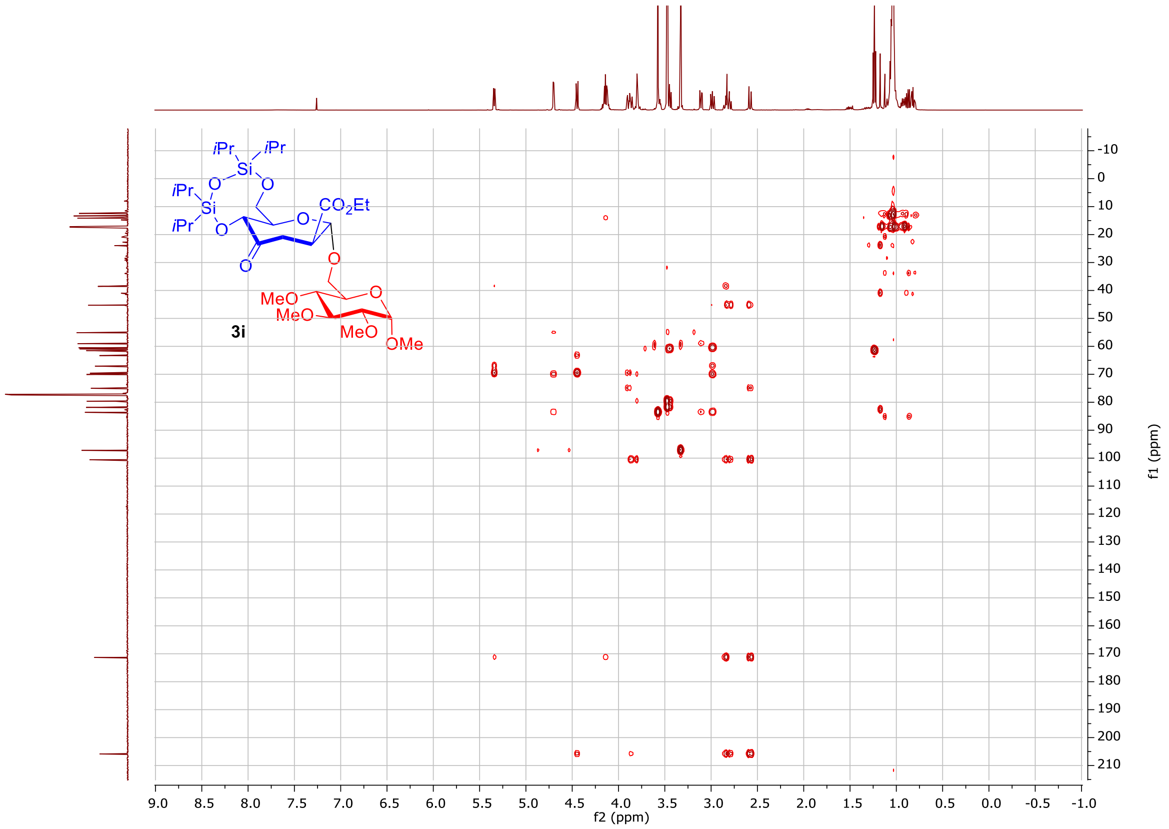

Supplementary Figure 224: HMBC spectra for compound **3i**

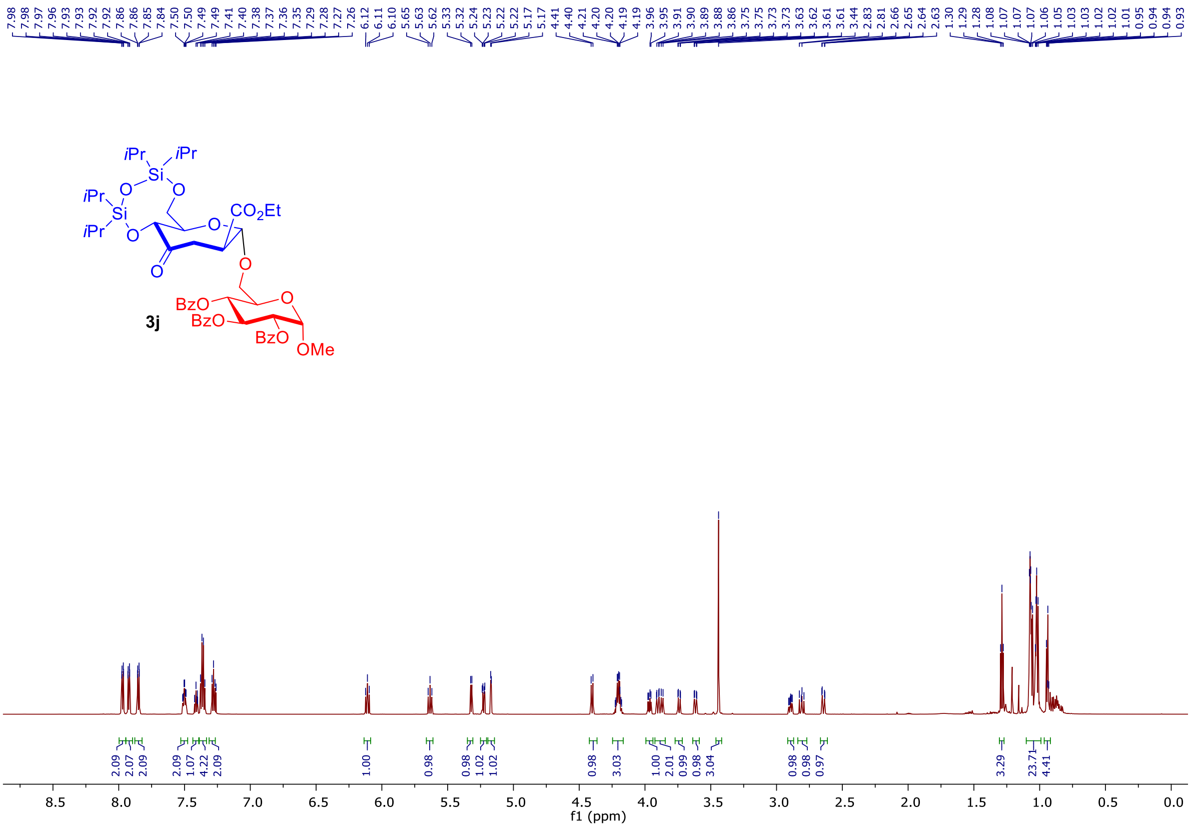

Supplementary Figure 225: ^{1}H spectra for **3j**

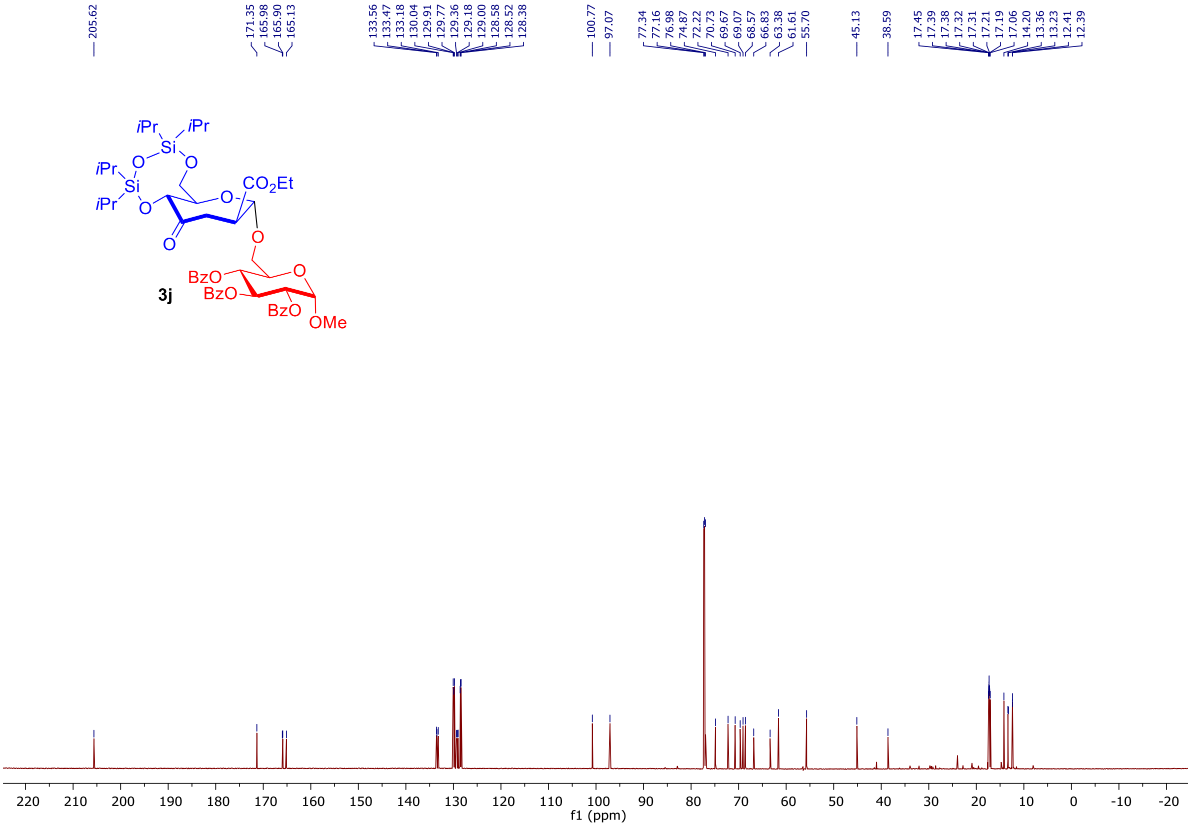

Supplementary Figure 226: ^{13}C spectra for **3j**

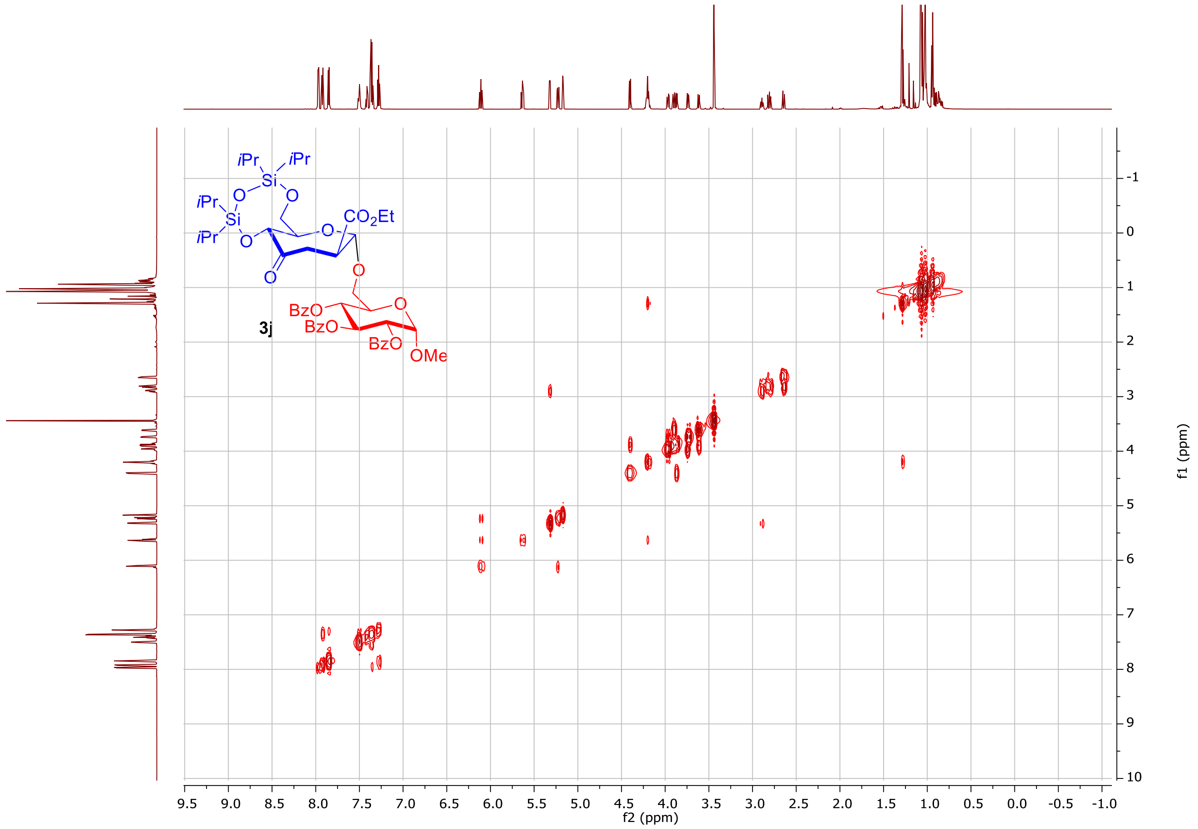

Supplementary Figure 227: COSY spectra for compound **3j**

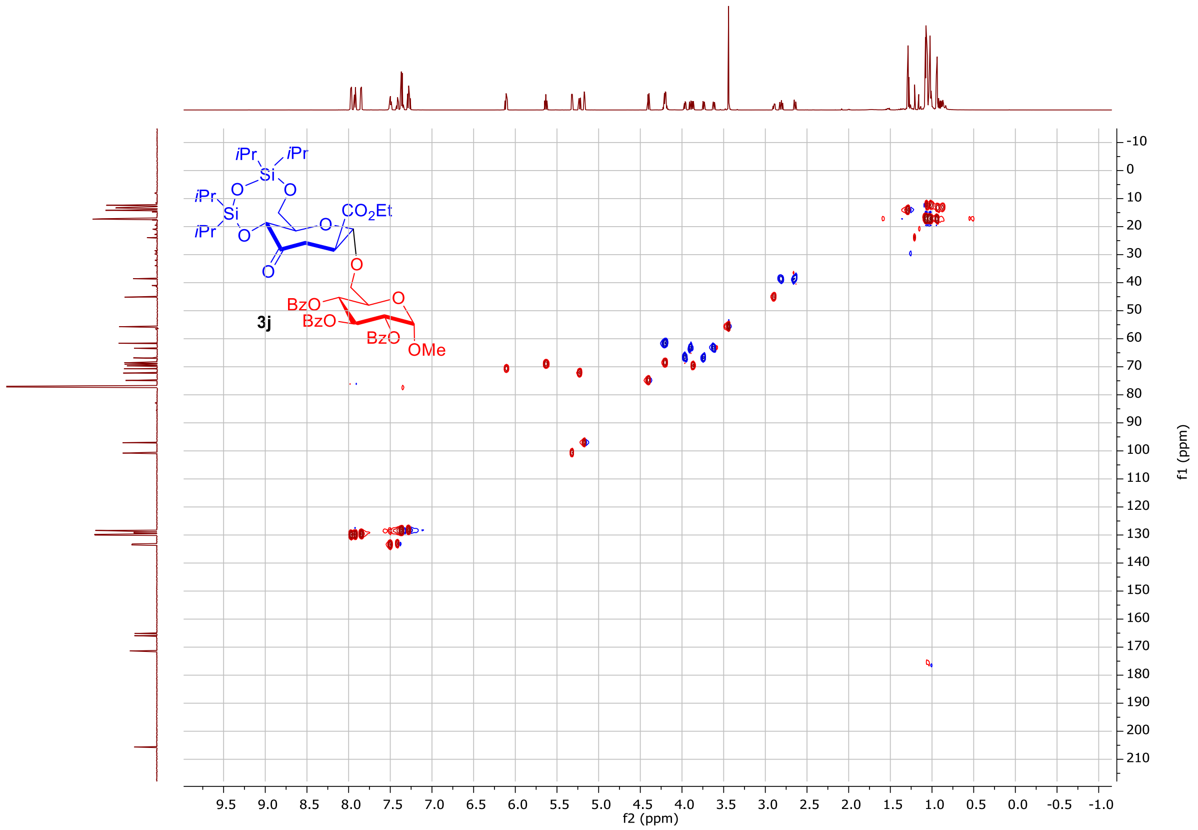

Supplementary Figure 228: HSQC spectra for compound **3j**

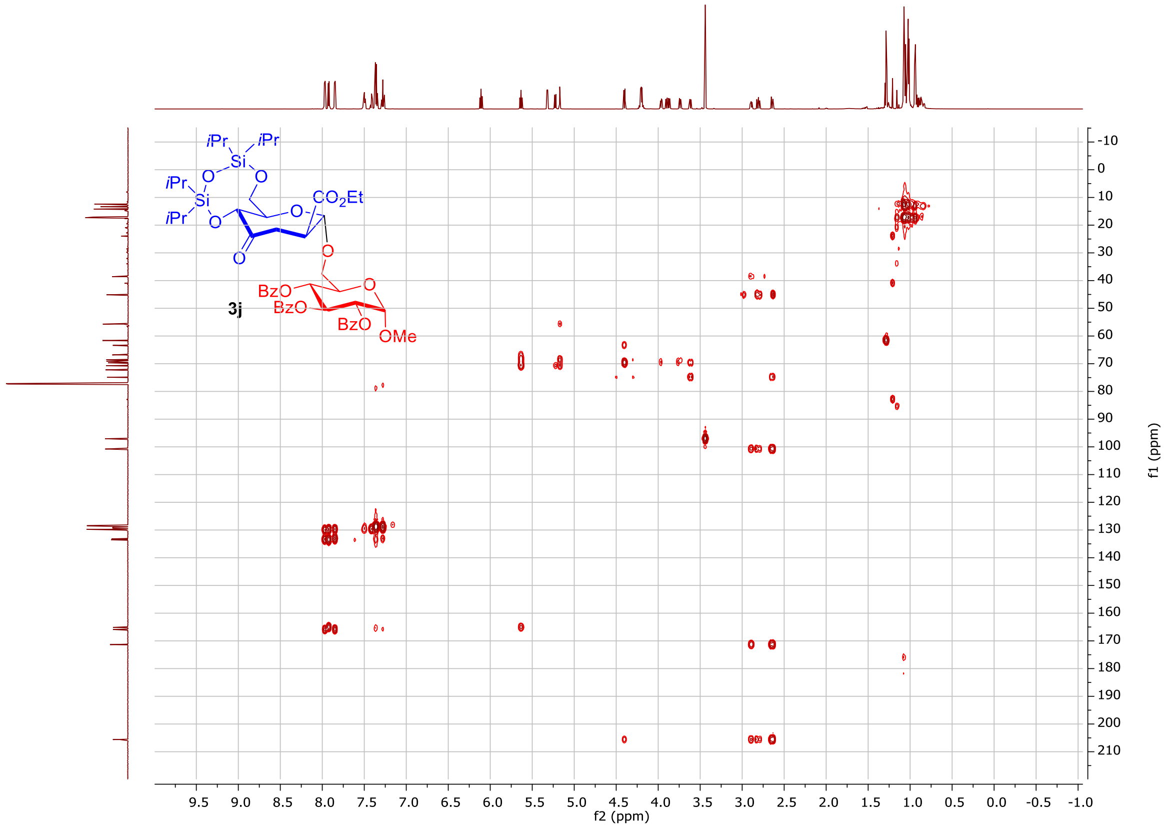

Supplementary Figure 229: HMBC spectra for compound **3j**

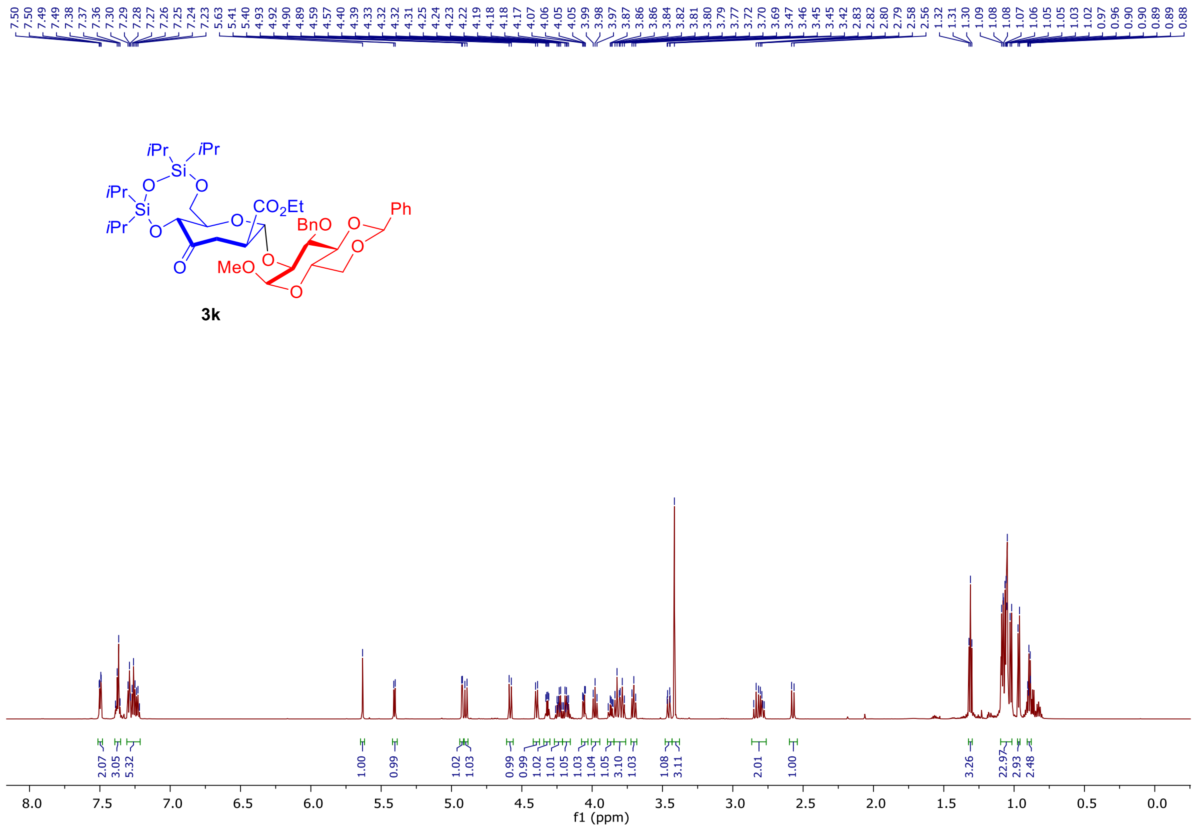

Supplementary Figure 230: ^{1}H spectra for **3k**

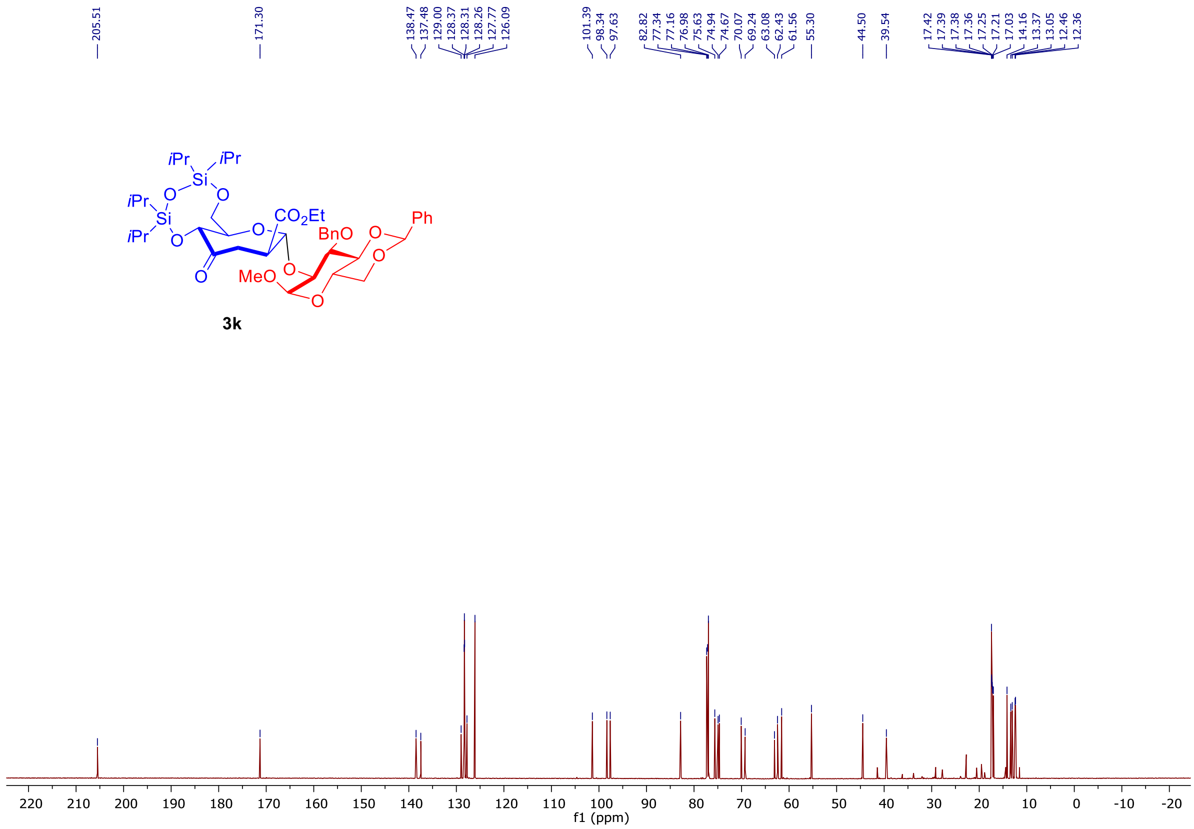

Supplementary Figure 231: ^{13}C spectra for **3k**

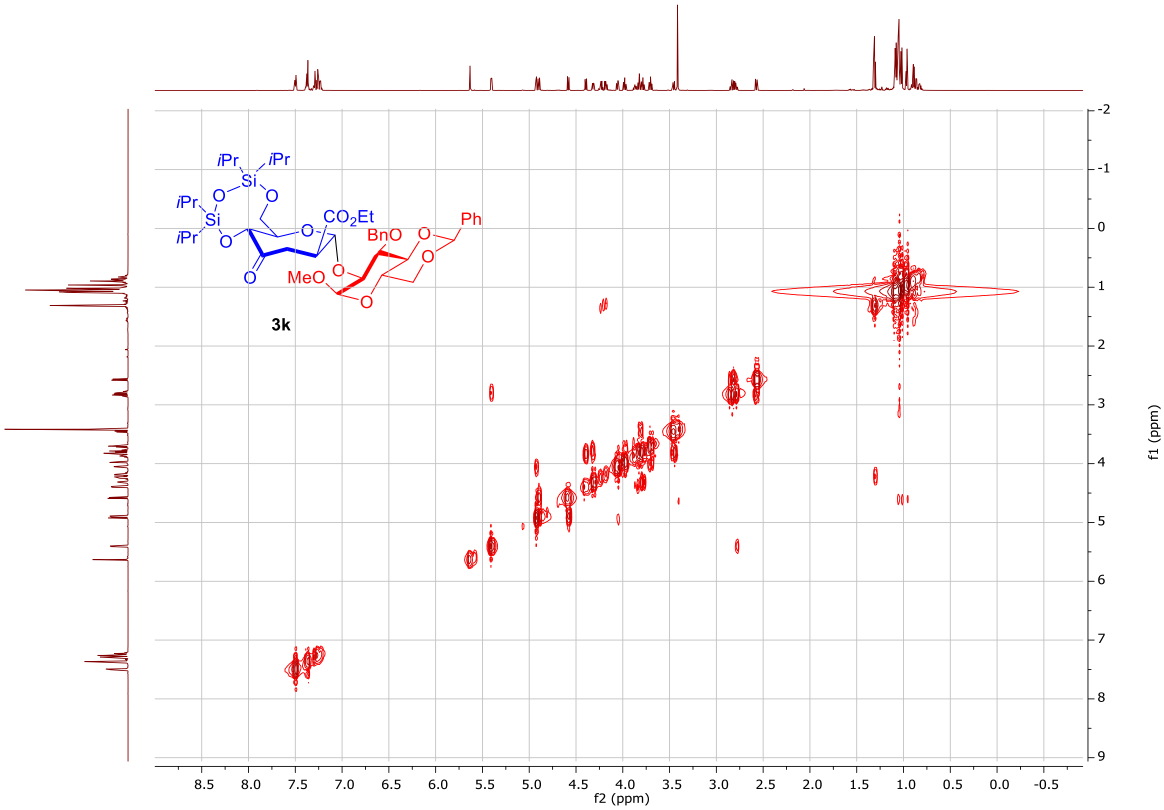

Supplementary Figure 232: COSY spectra for compound **3k**

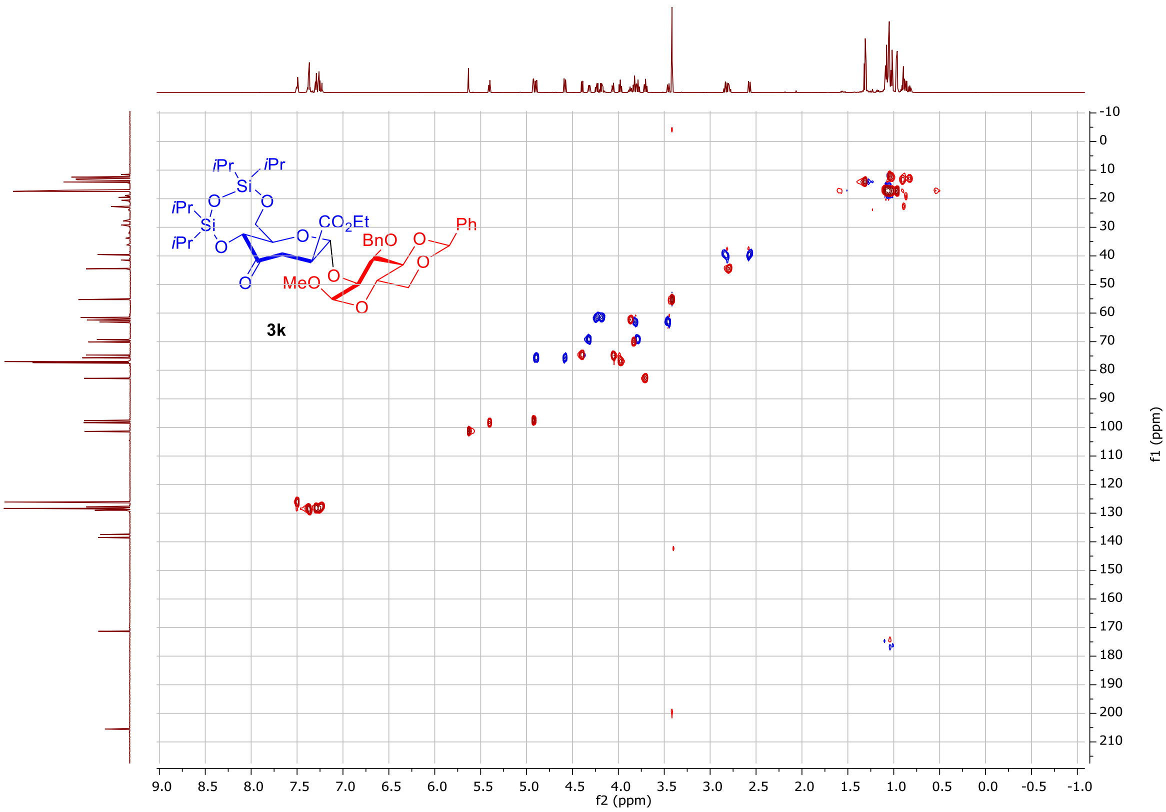

Supplementary Figure 233: HSQC spectra for compound **3k**

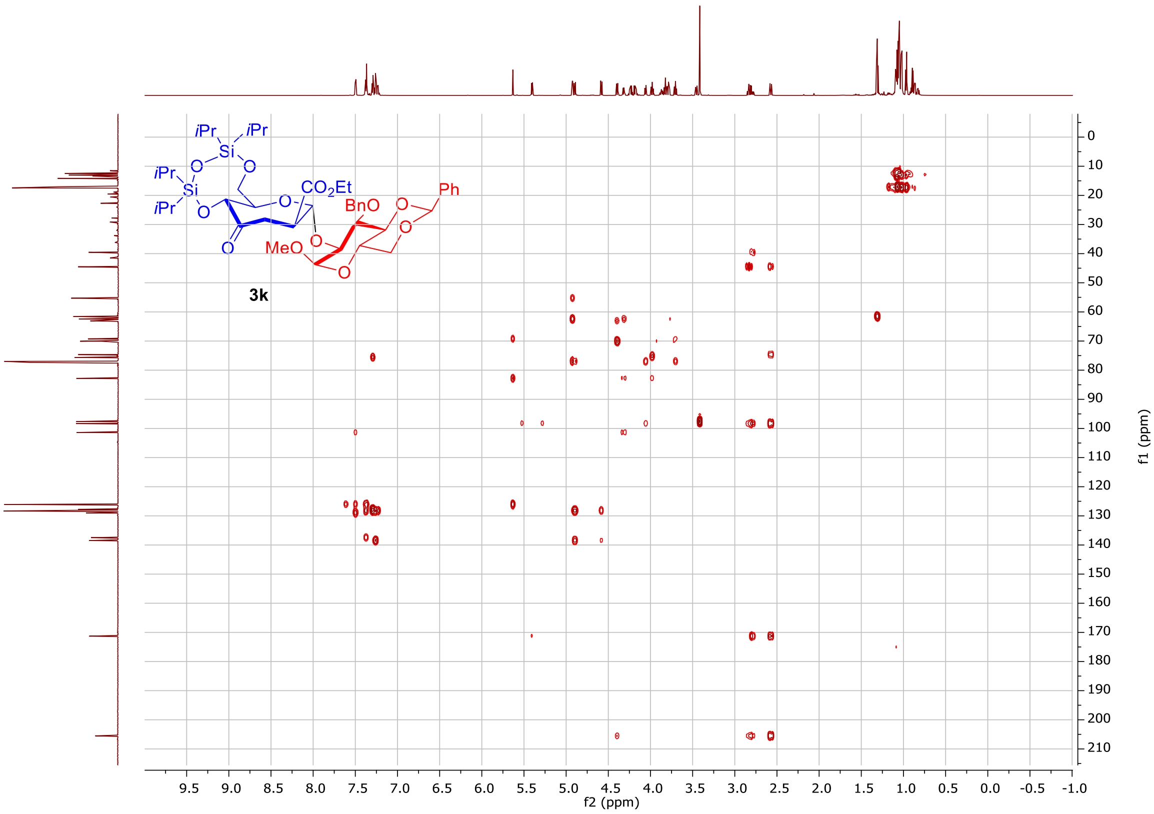

Supplementary Figure 234: HMBC spectra for compound **3k**

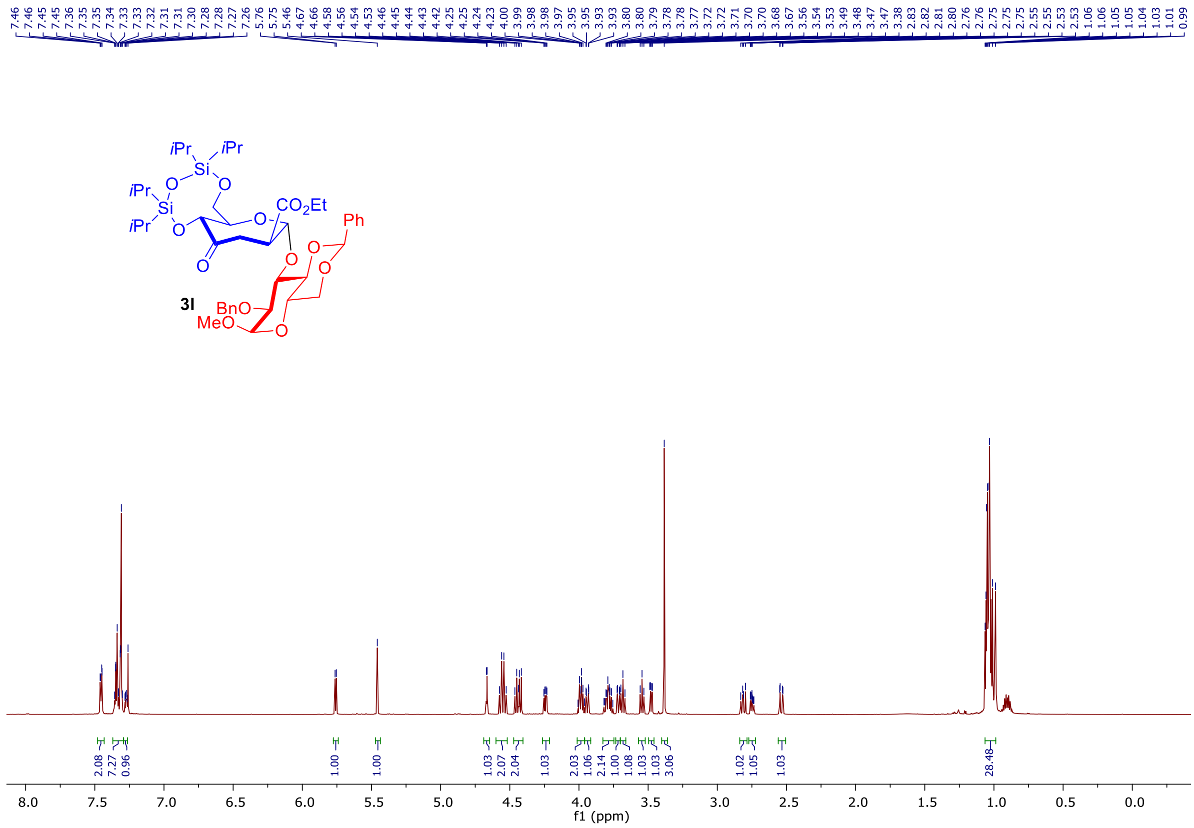

Supplementary Figure 235: ^{1}H spectra for **3l**

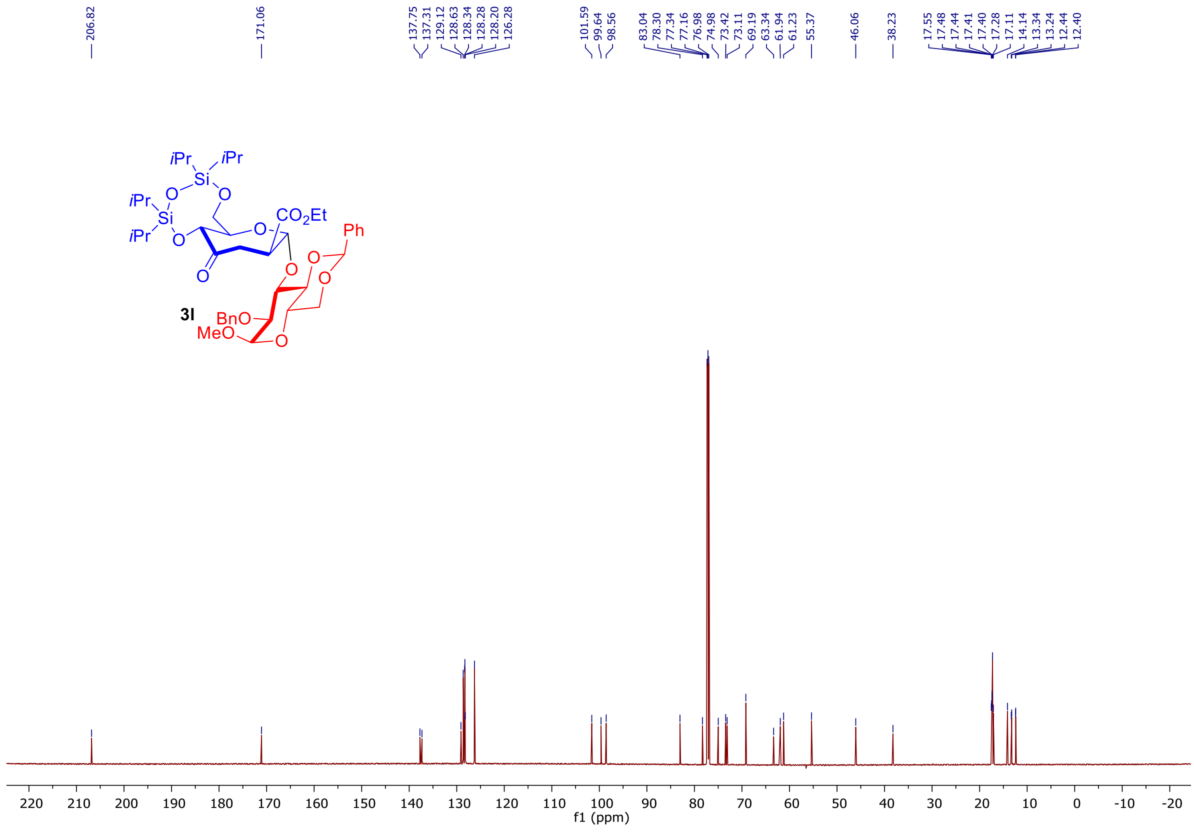

Supplementary Figure 236: ^{13}C spectra for **3l**

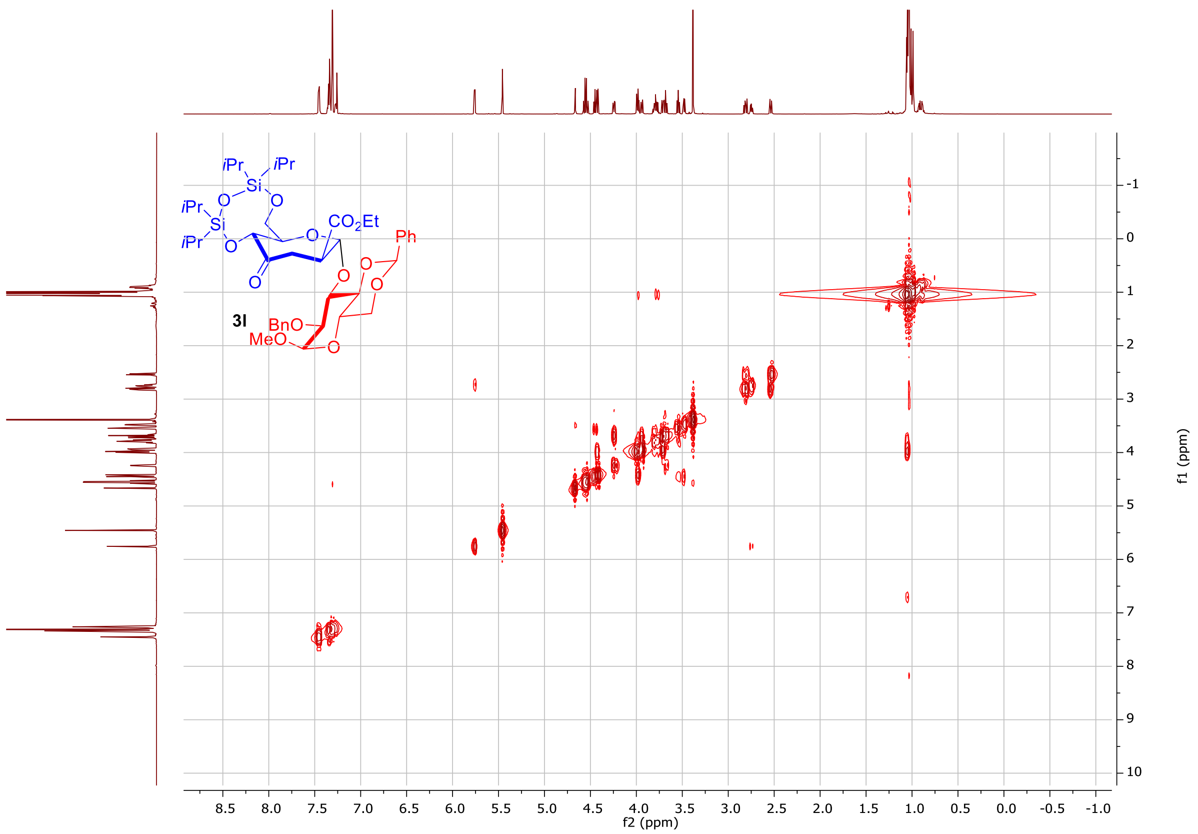

Supplementary Figure 237: COSY spectra for compound **3l**

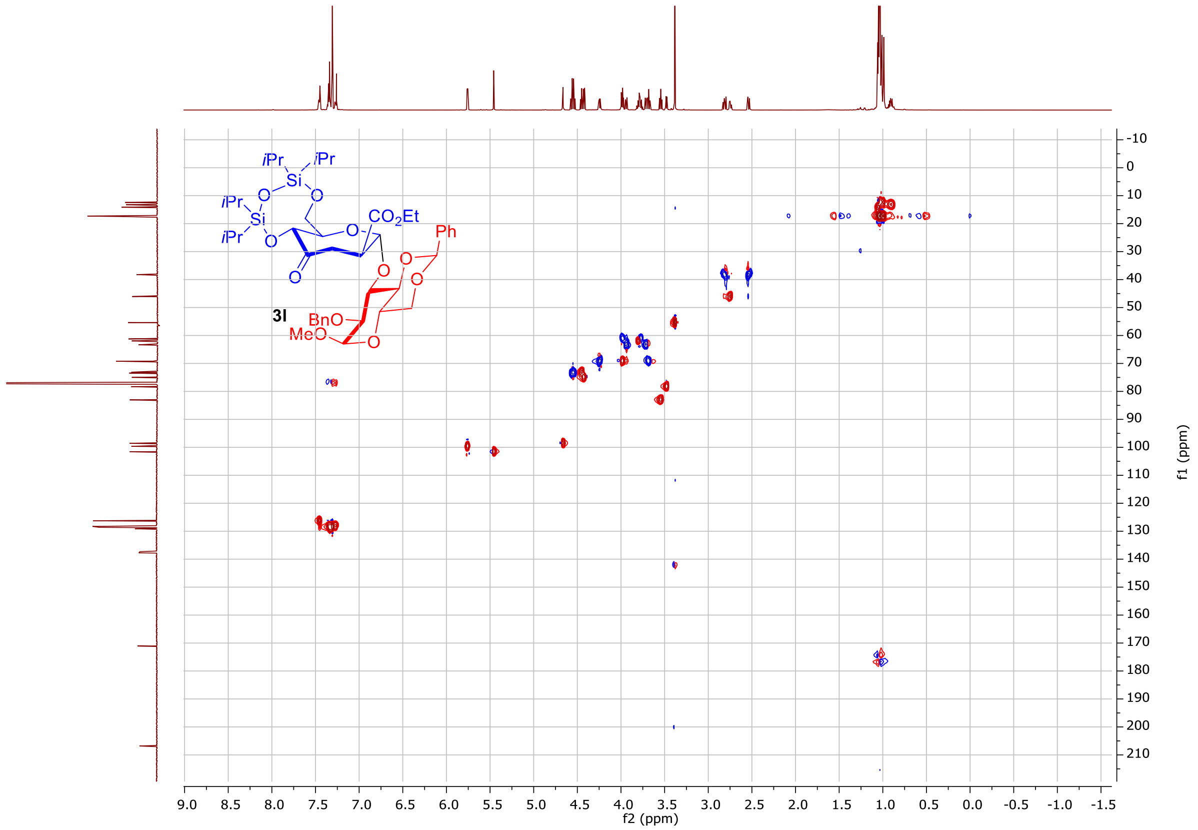

Supplementary Figure 238: HSQC spectra for compound **3l**

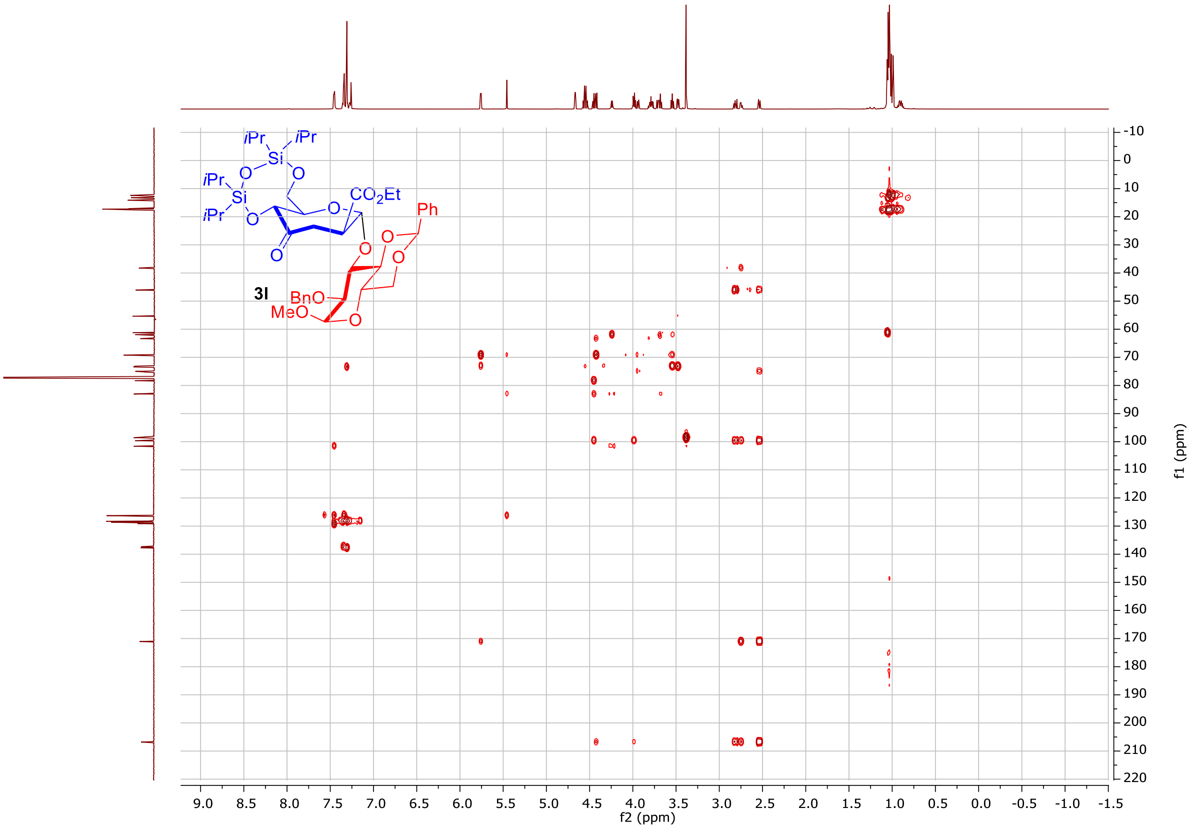

Supplementary Figure 239: HMBC spectra for compound **3l**

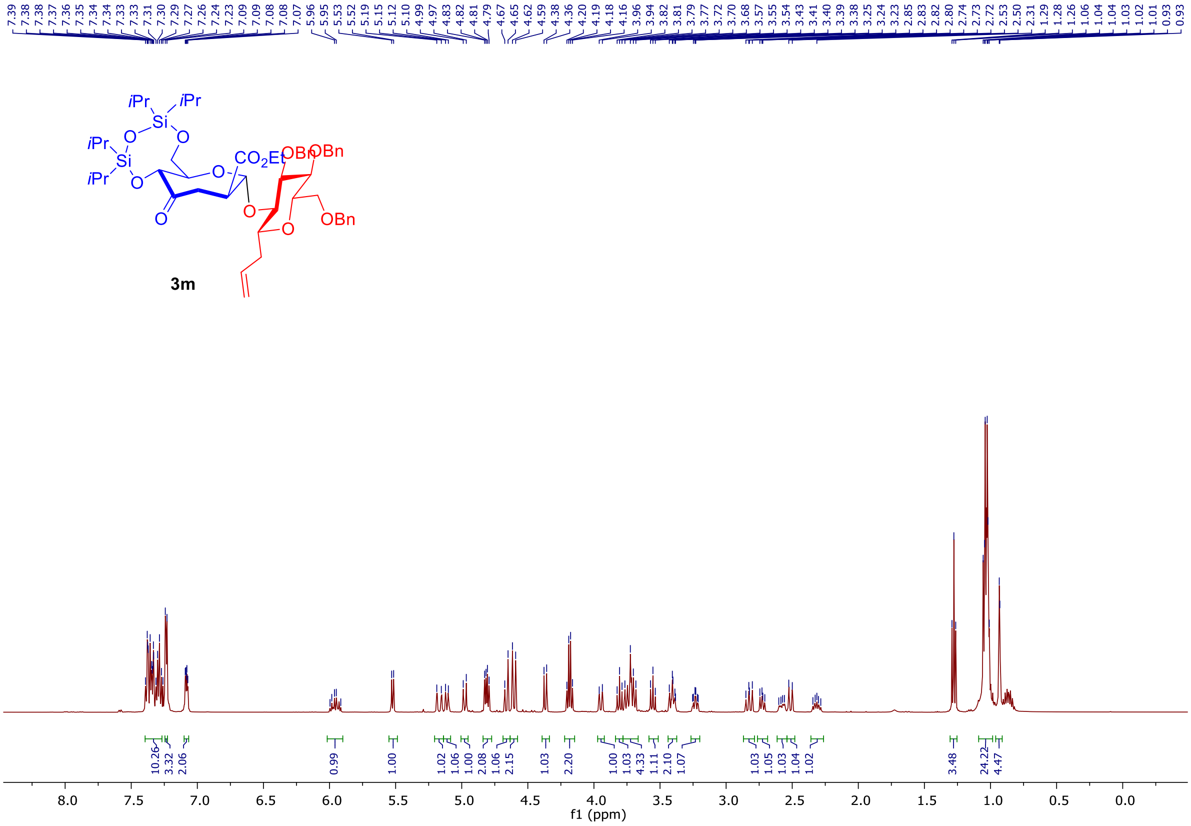

Supplementary Figure 240: ^{1}H spectra for **3m**

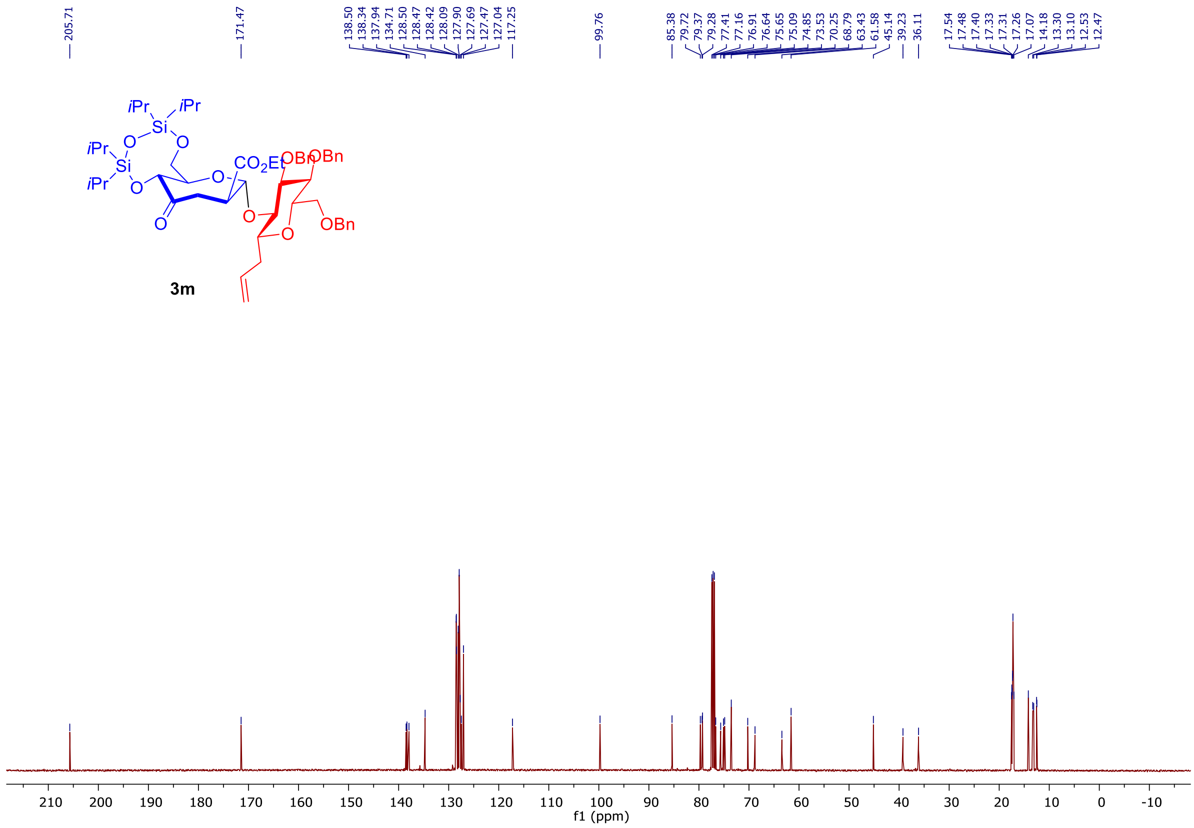

Supplementary Figure 241: ^{13}C spectra for **3m**

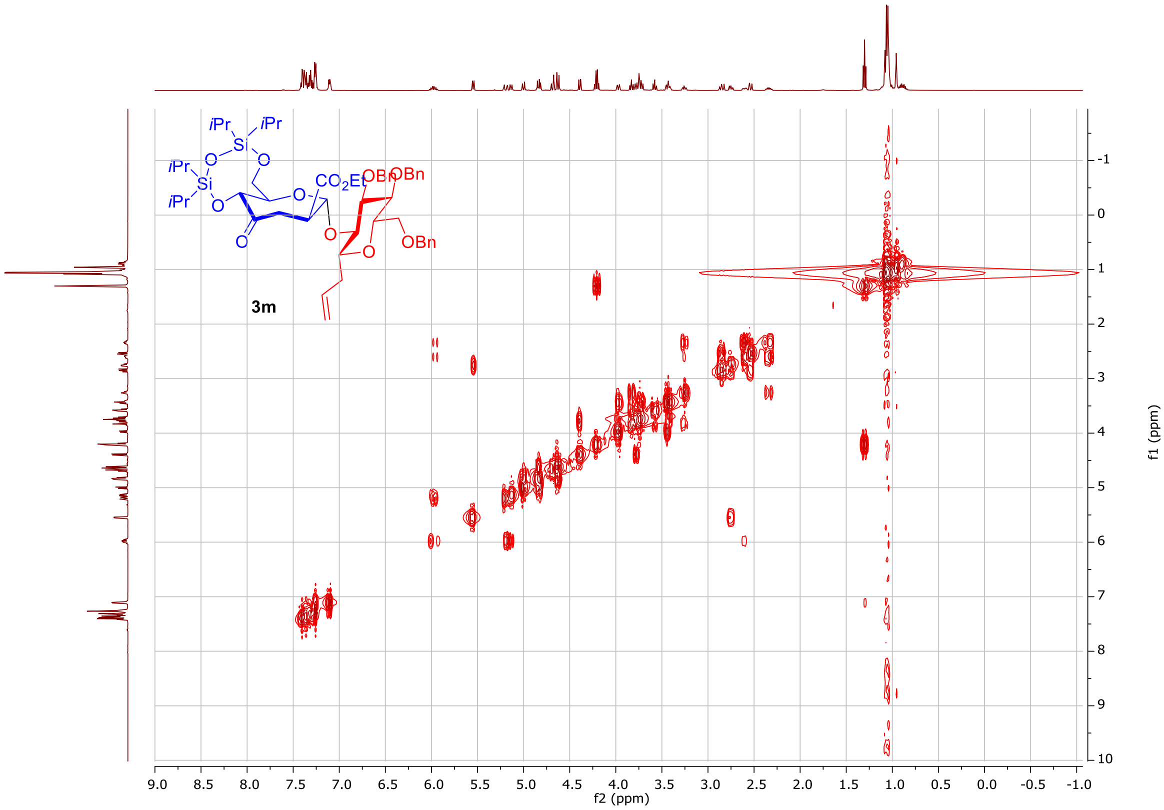

Supplementary Figure 242: COSY spectra for compound **3m**

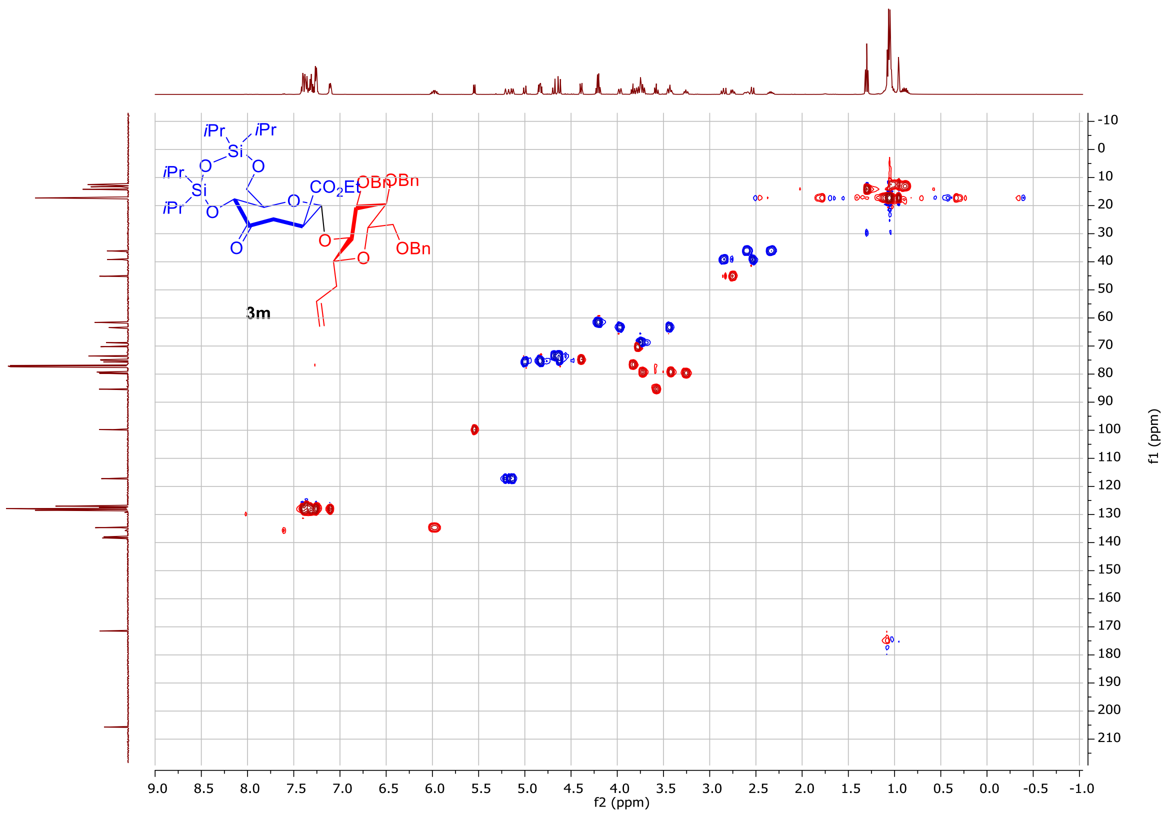

Supplementary Figure 243: HSQC spectra for compound **3m**

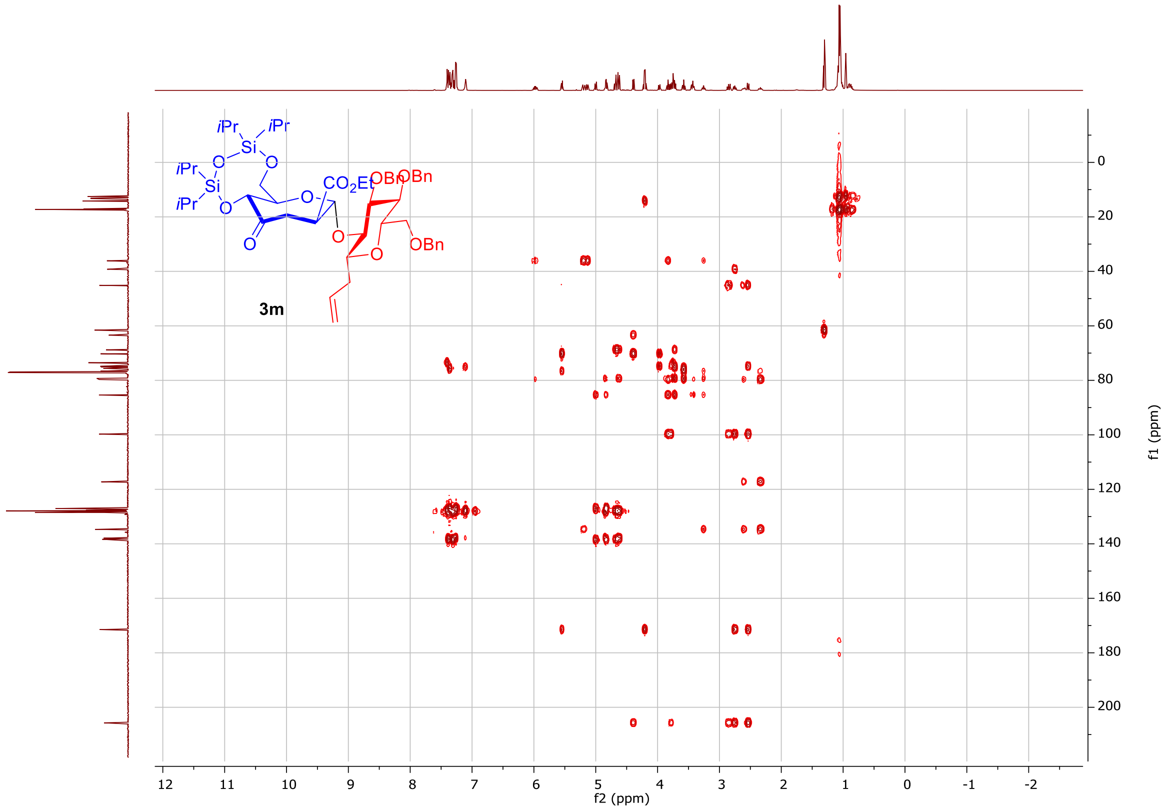

Supplementary Figure 244: HMBC spectra for compound **3m**

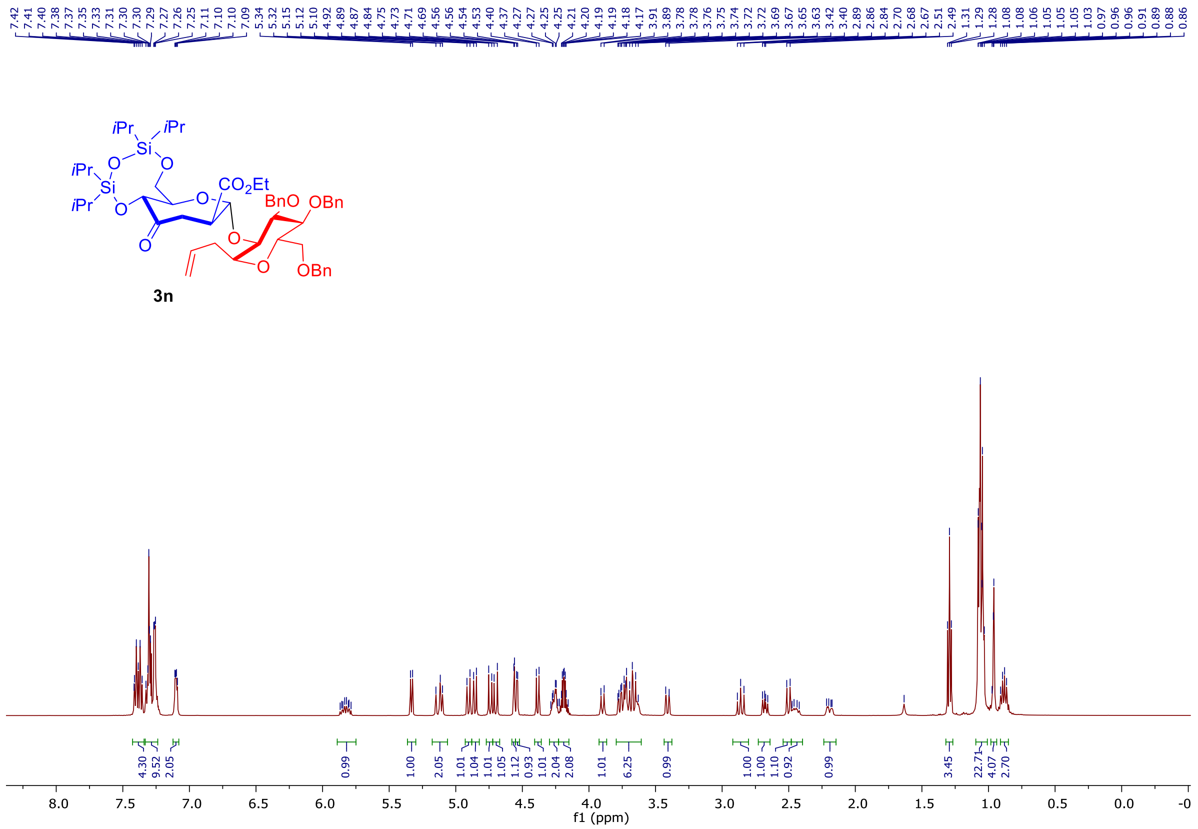

Supplementary Figure 245: ^{1}H spectra for **3n**

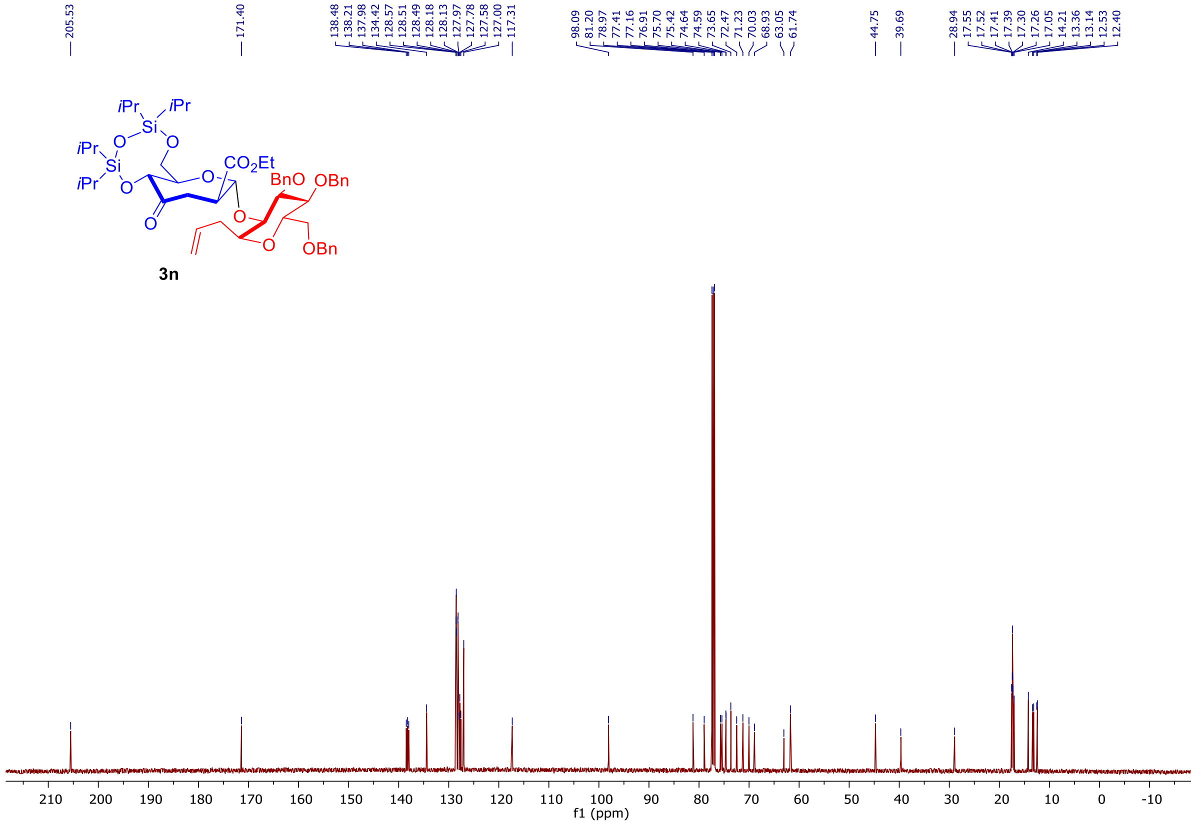

Supplementary Figure 246: ^{13}C spectra for **3n**

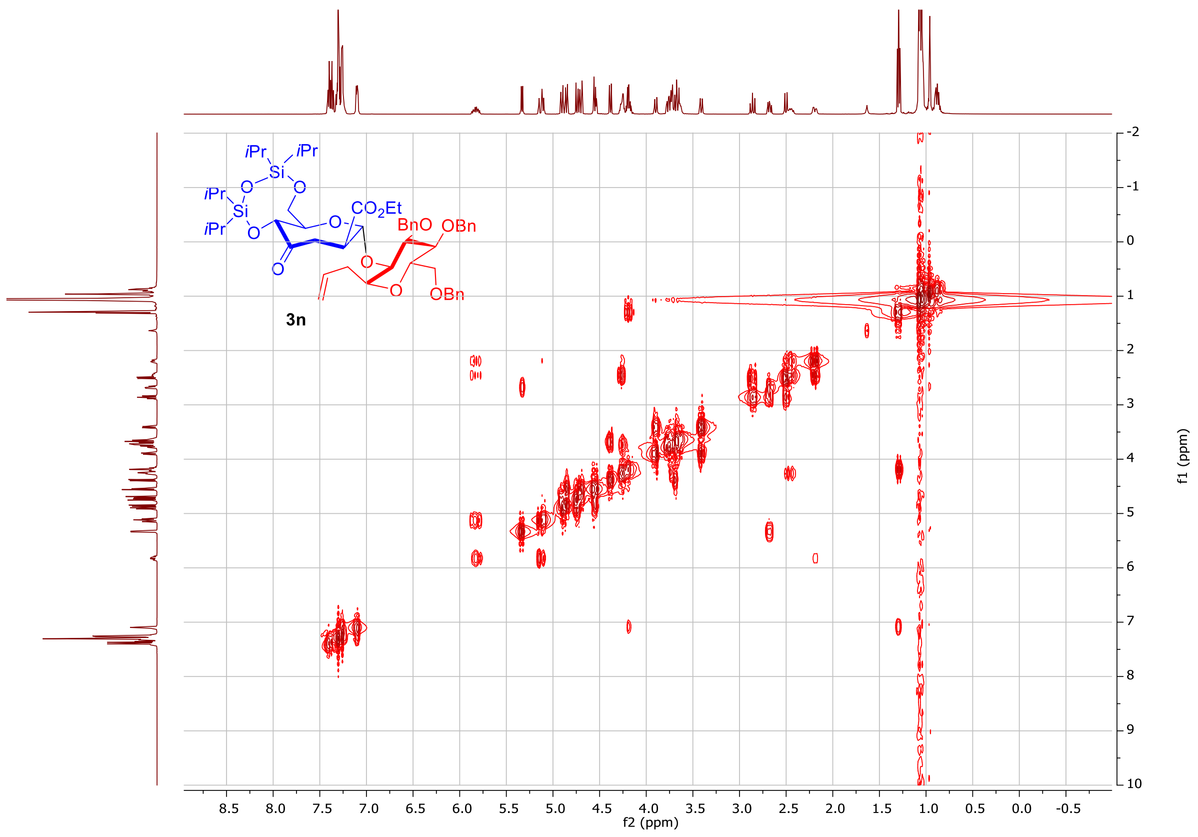

Supplementary Figure 247: COSY spectra for compound **3n**

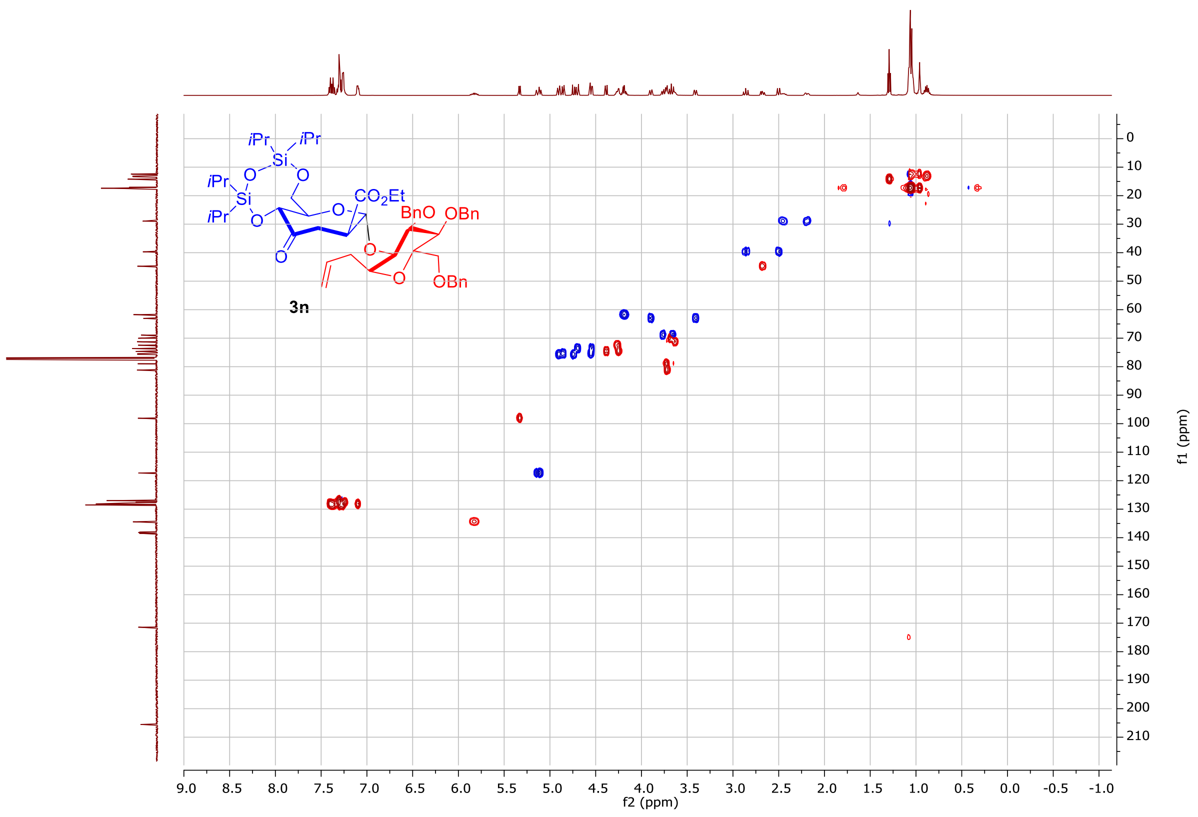

Supplementary Figure 248: HSQC spectra for compound **3n**

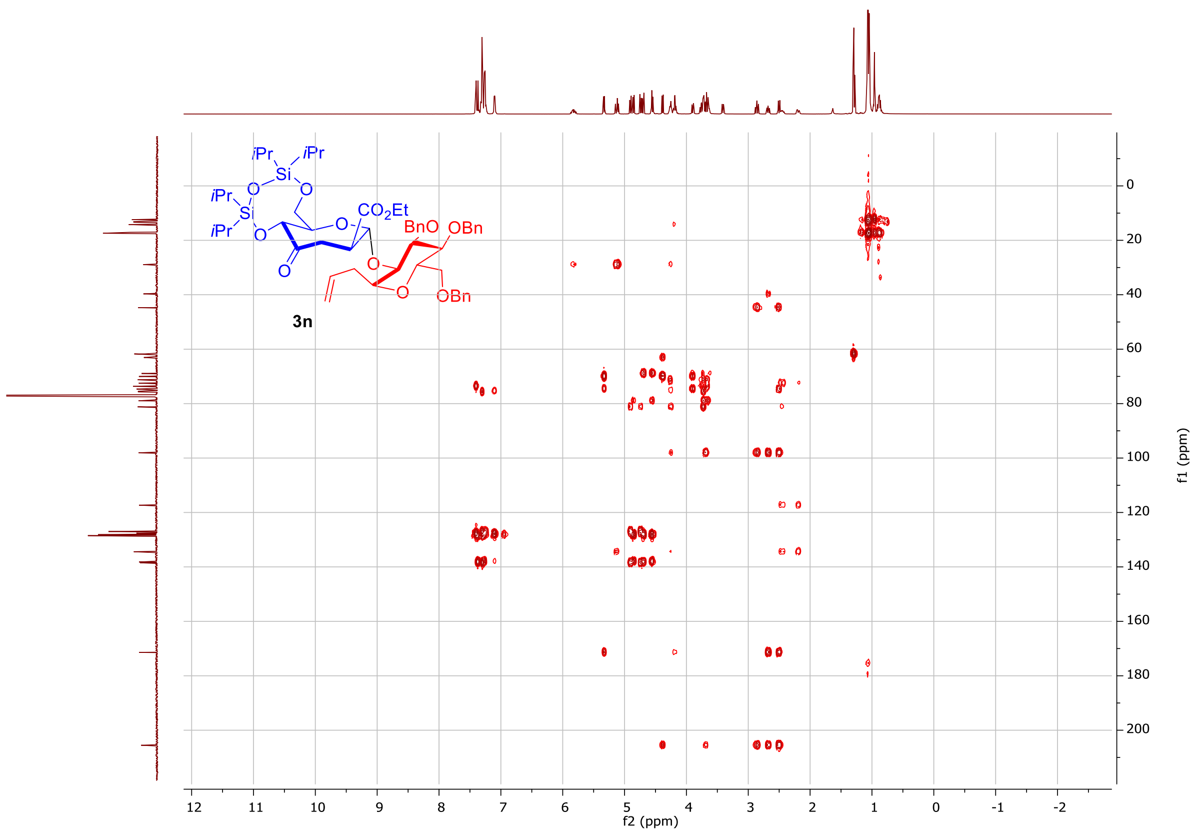

Supplementary Figure 249: HMBC spectra for compound **3n**

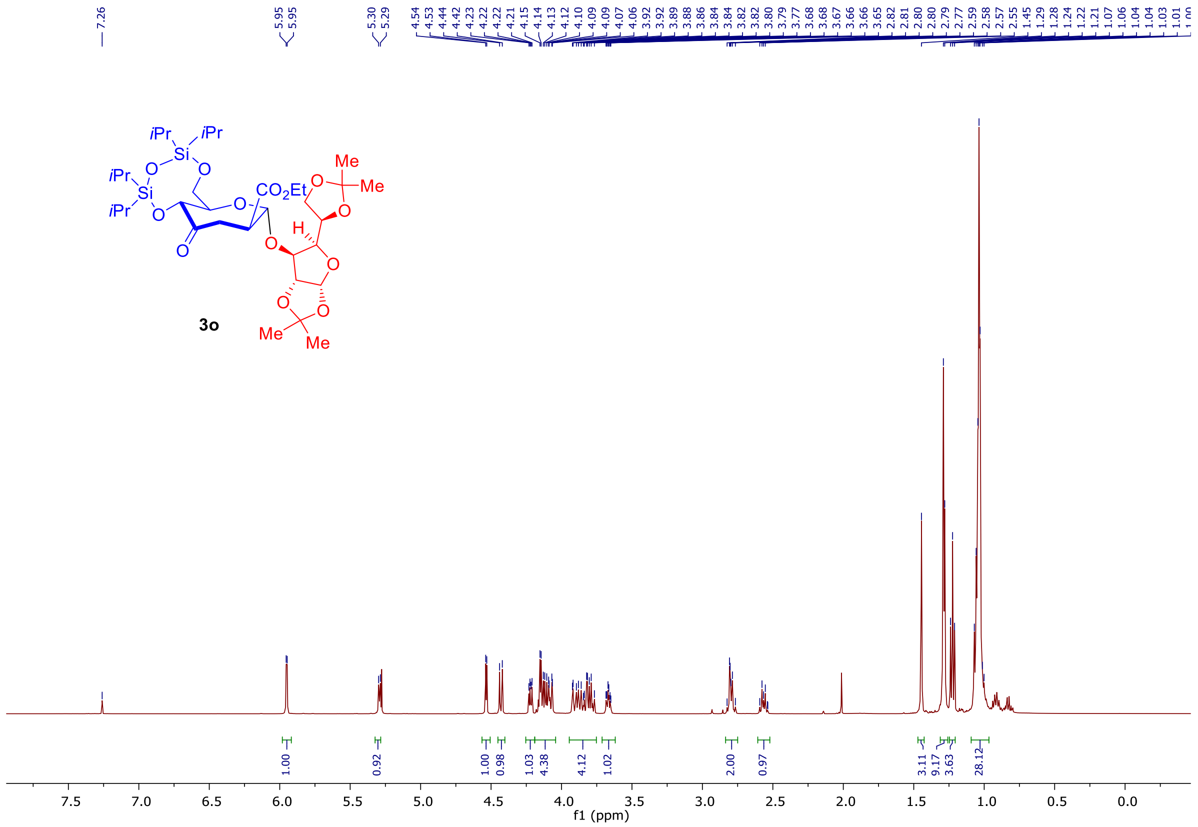

Supplementary Figure 250: ^{1}H spectra for **3o**

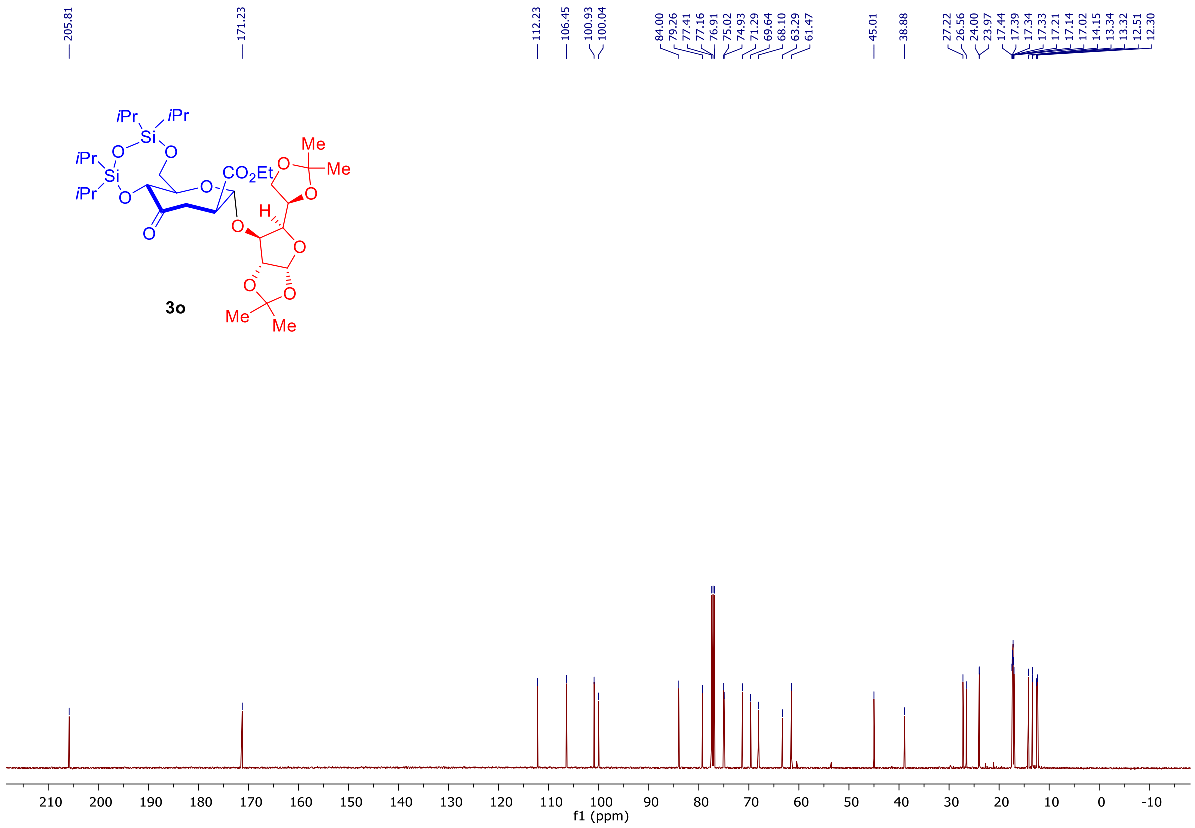

Supplementary Figure 251: ^{13}C spectra for **3o**

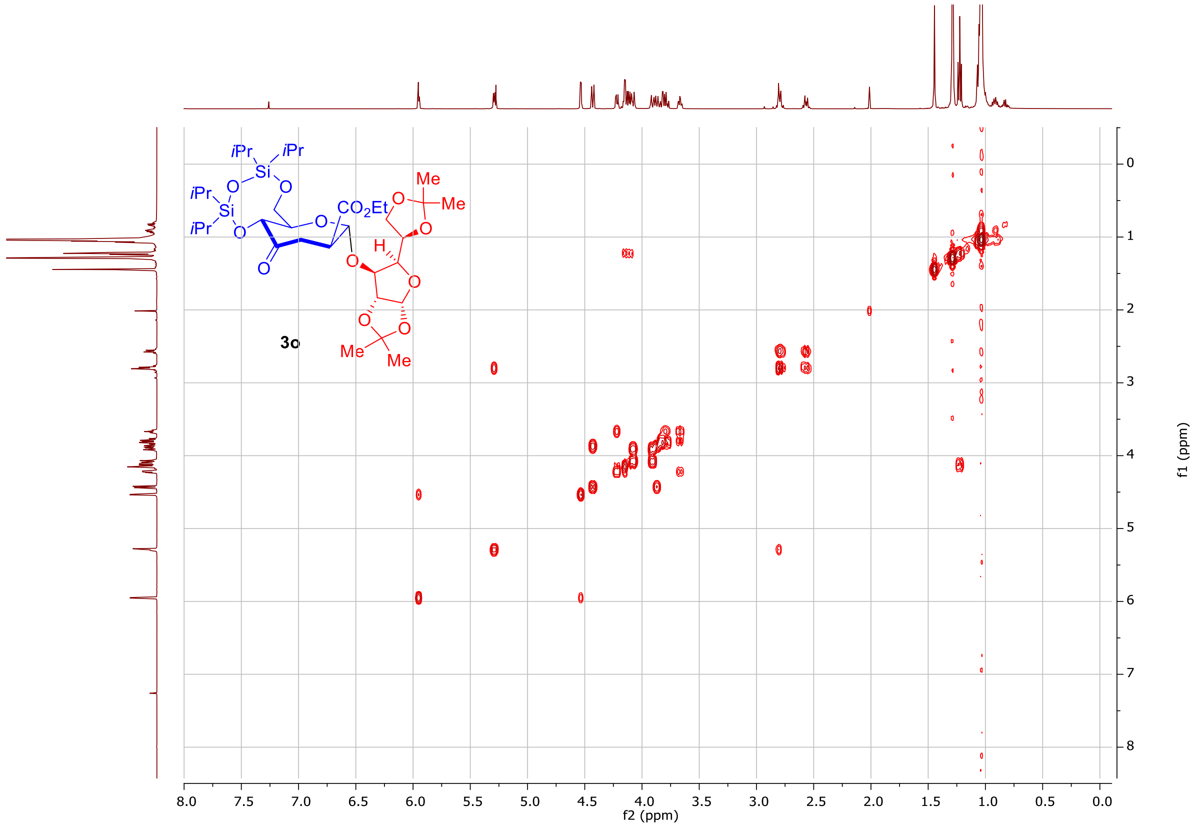

Supplementary Figure 252: COSY spectra for compound **3o**

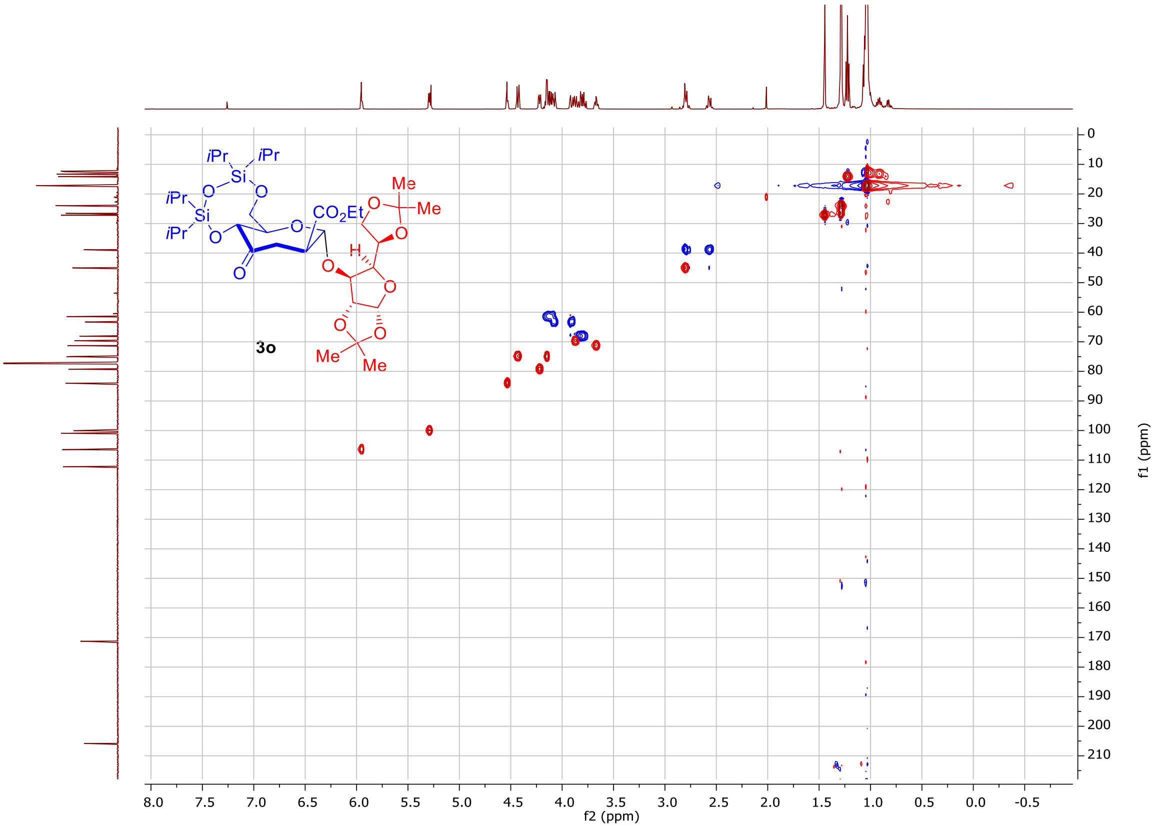

Supplementary Figure 253: HSQC spectra for compound **3o**

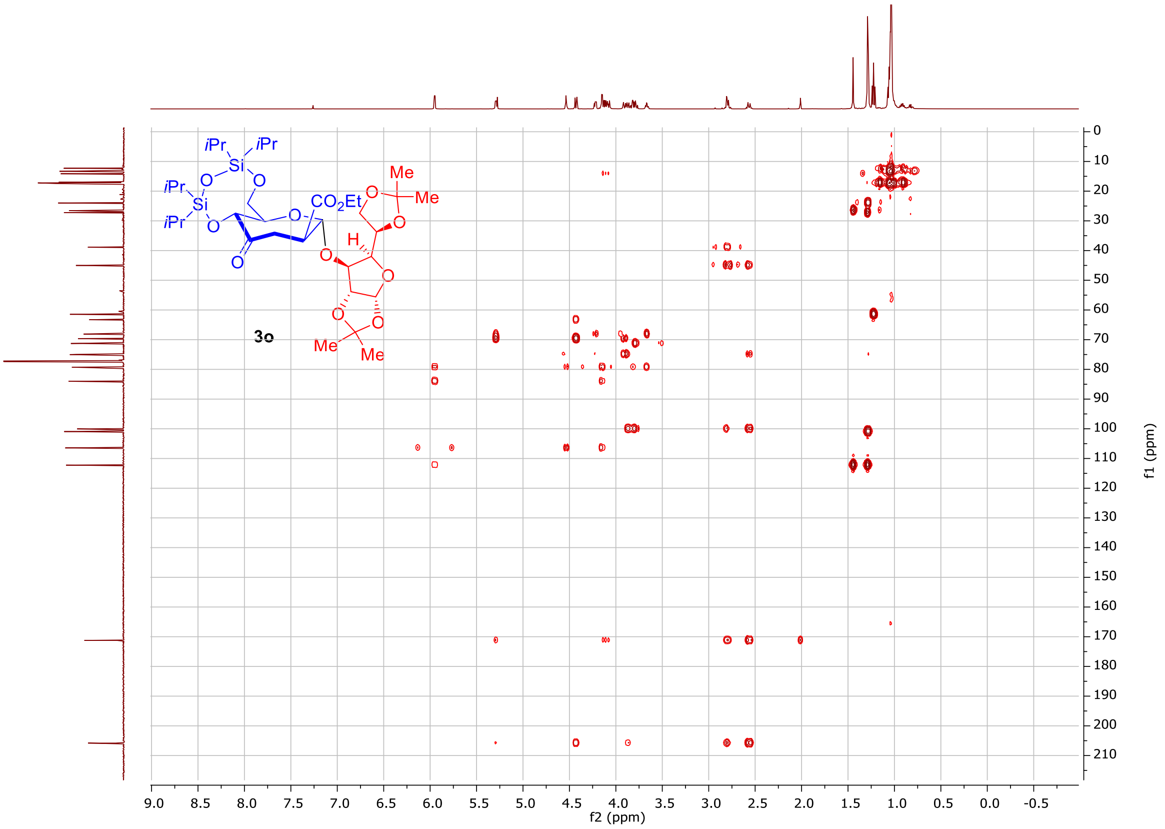

Supplementary Figure 254: HMBC spectra for compound **3o**

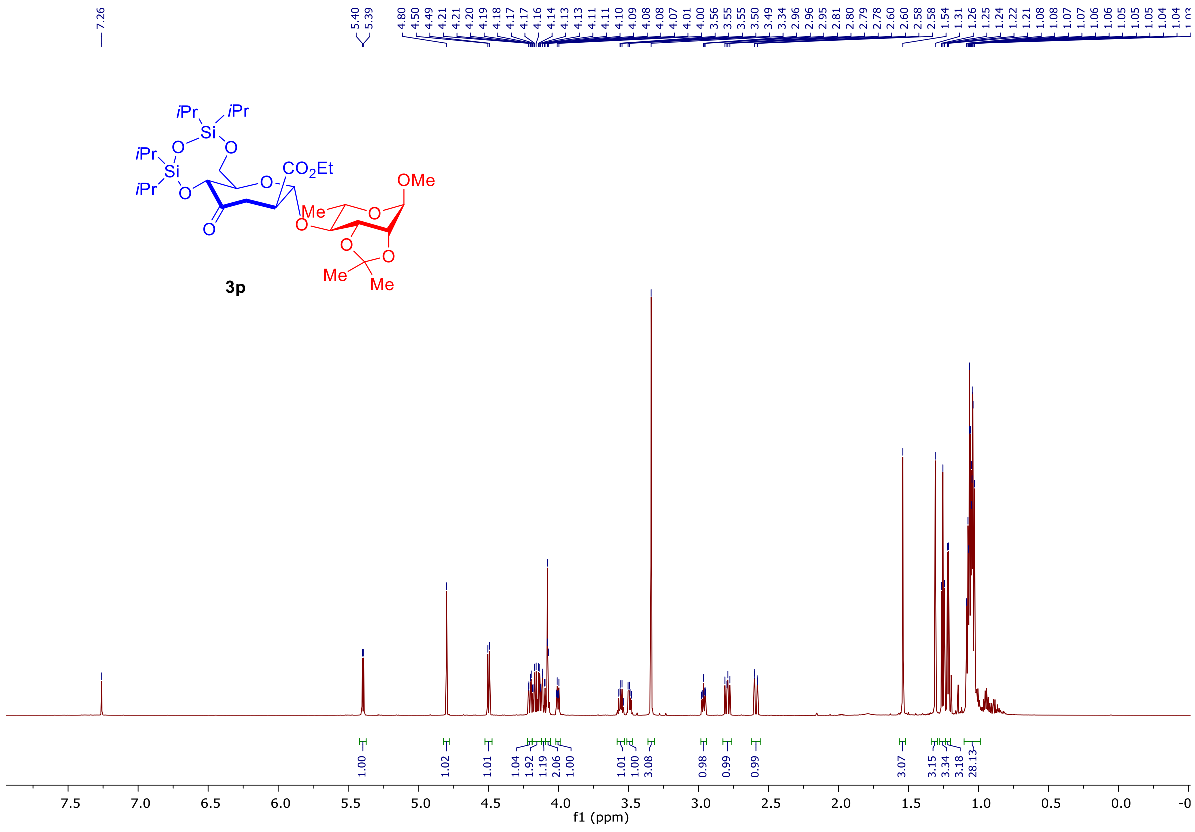

Supplementary Figure 255: ^{1}H spectra for **3p**

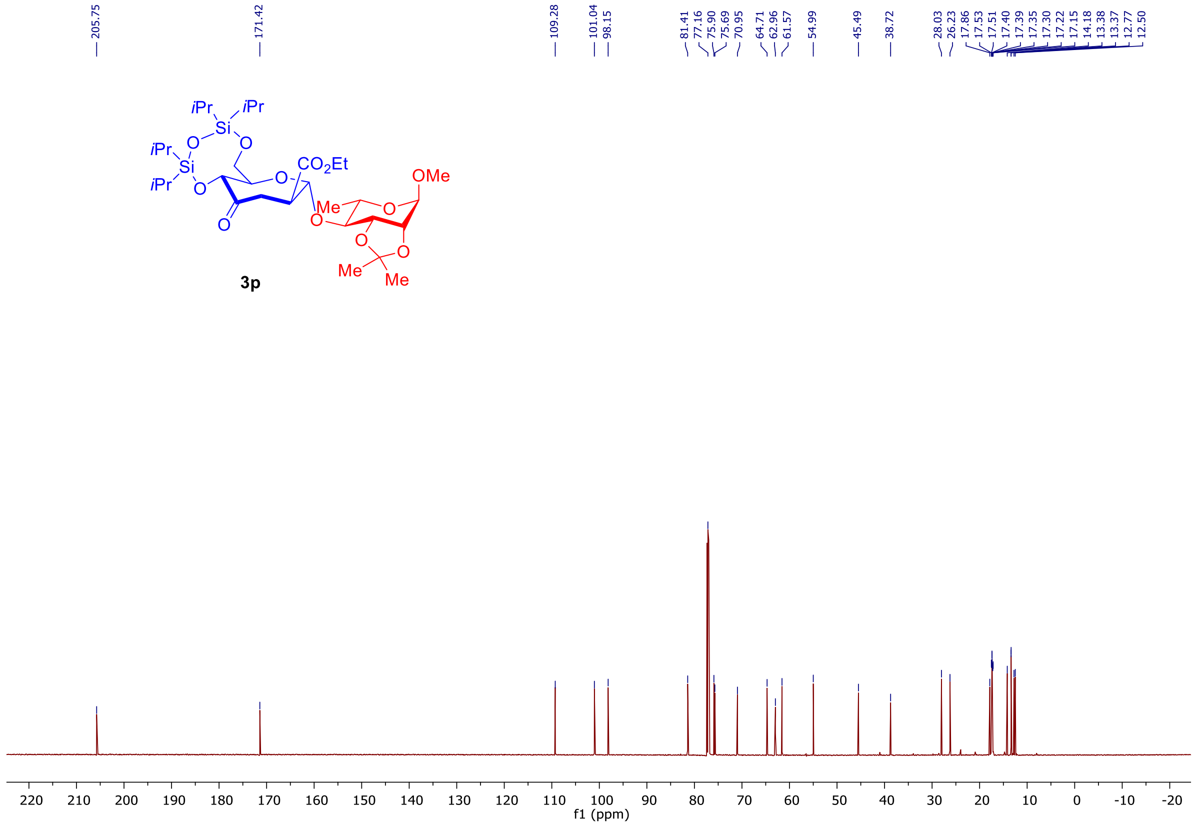

Supplementary Figure 256: ^{13}C spectra for **3p**

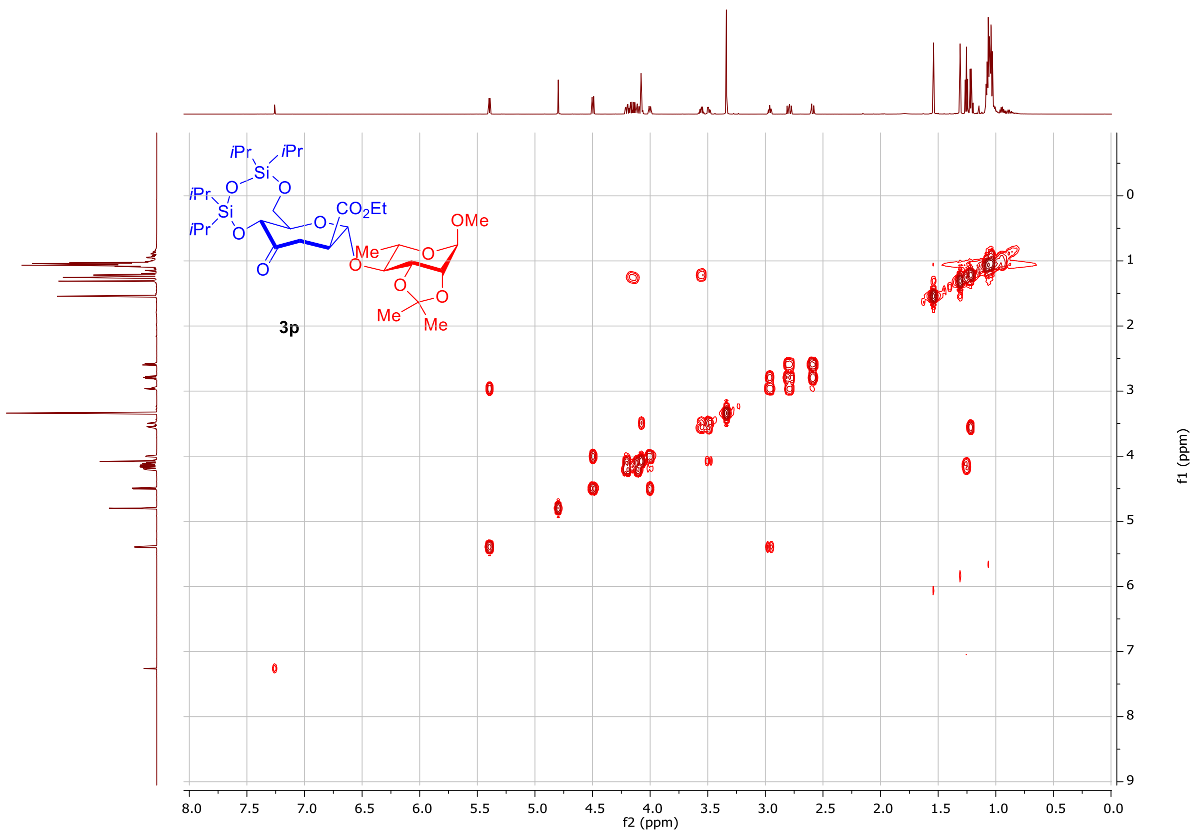

Supplementary Figure 257: COSY spectra for compound **3p**

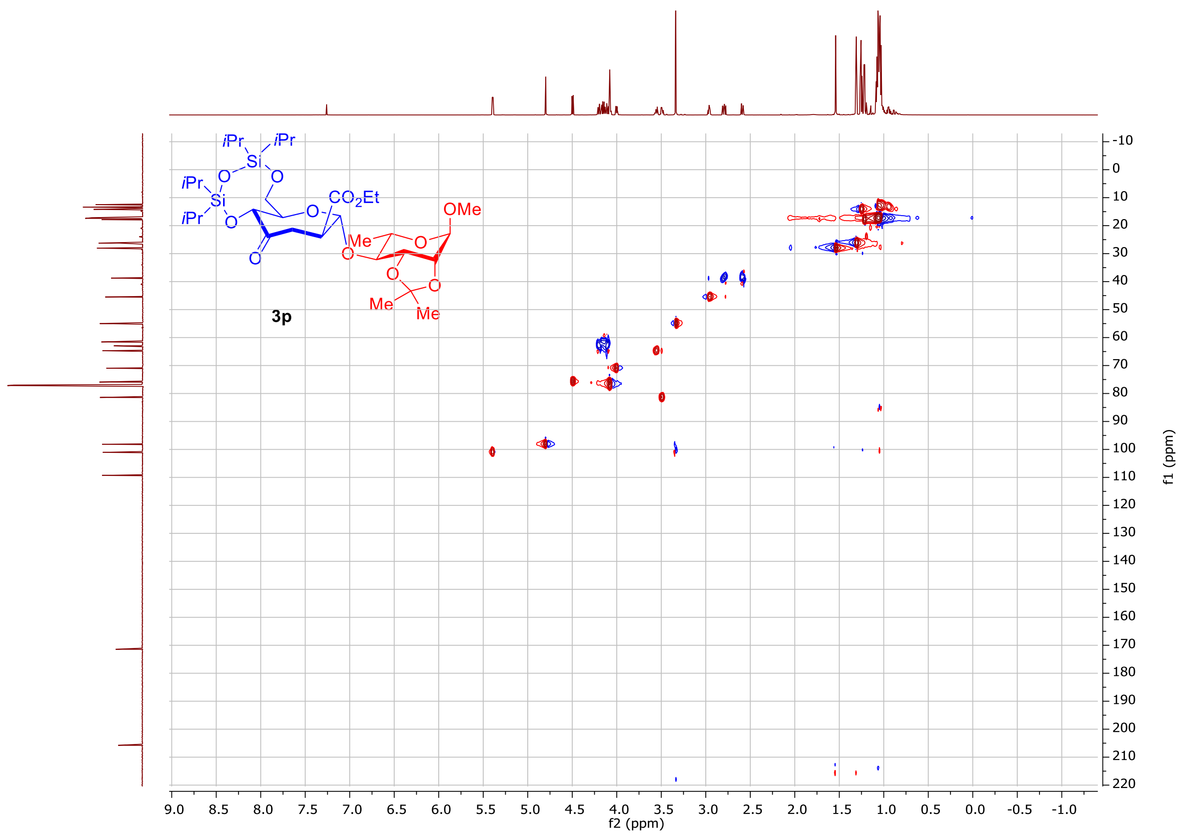

Supplementary Figure 258: HSQC spectra for compound **3p**

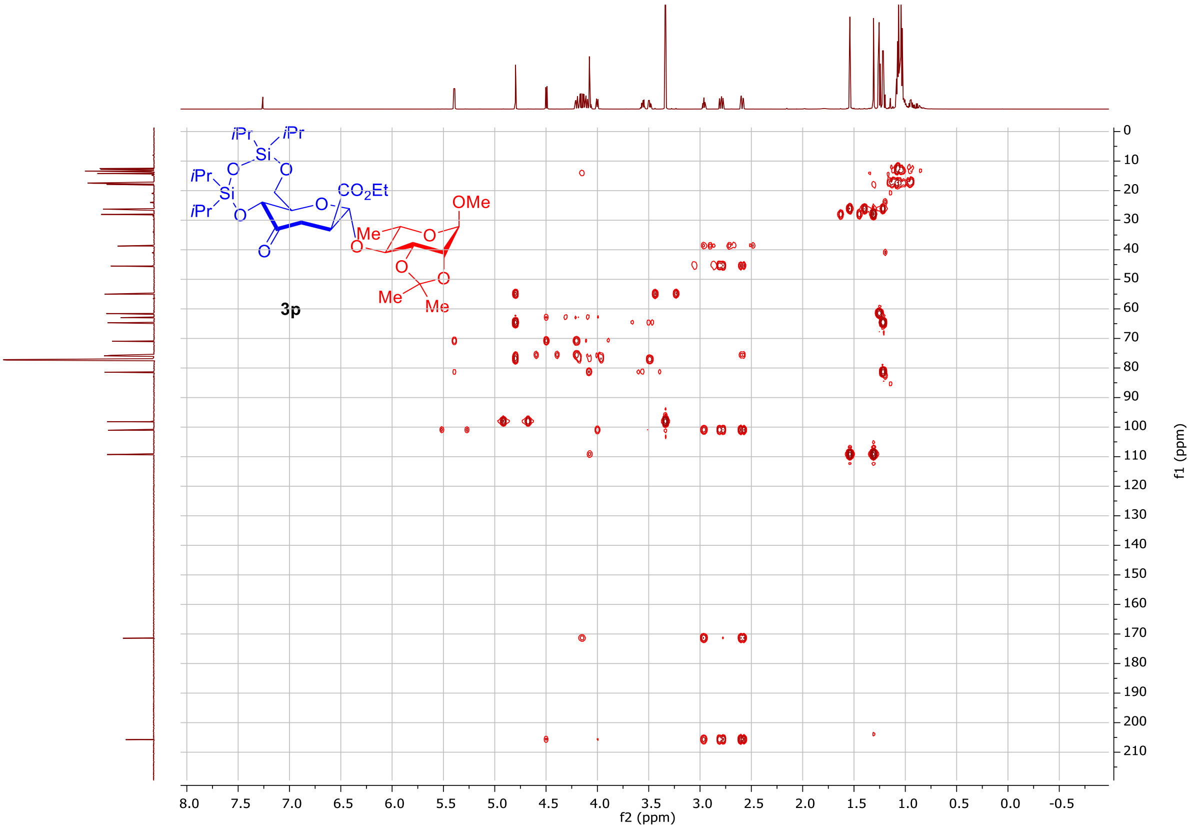

Supplementary Figure 259: HMBC spectra for compound **3p**

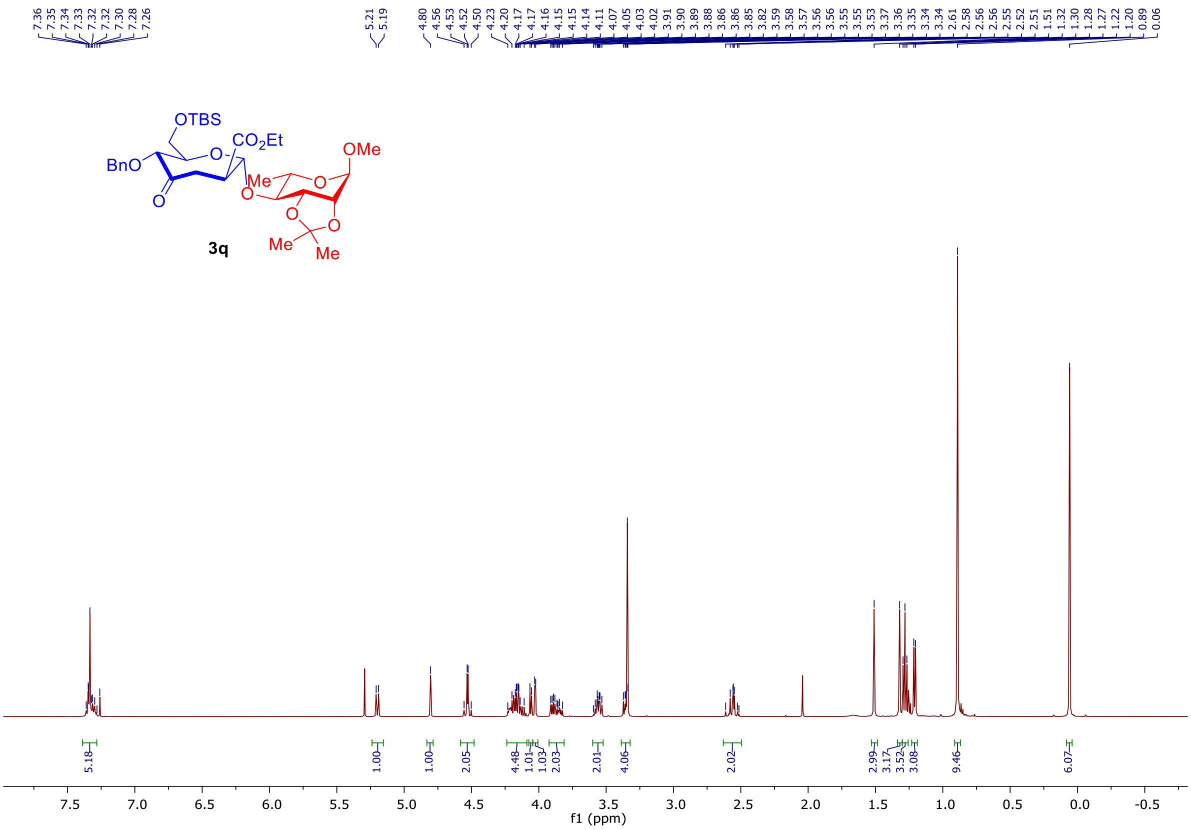

Supplementary Figure 260: ^{1}H spectra for **3q**

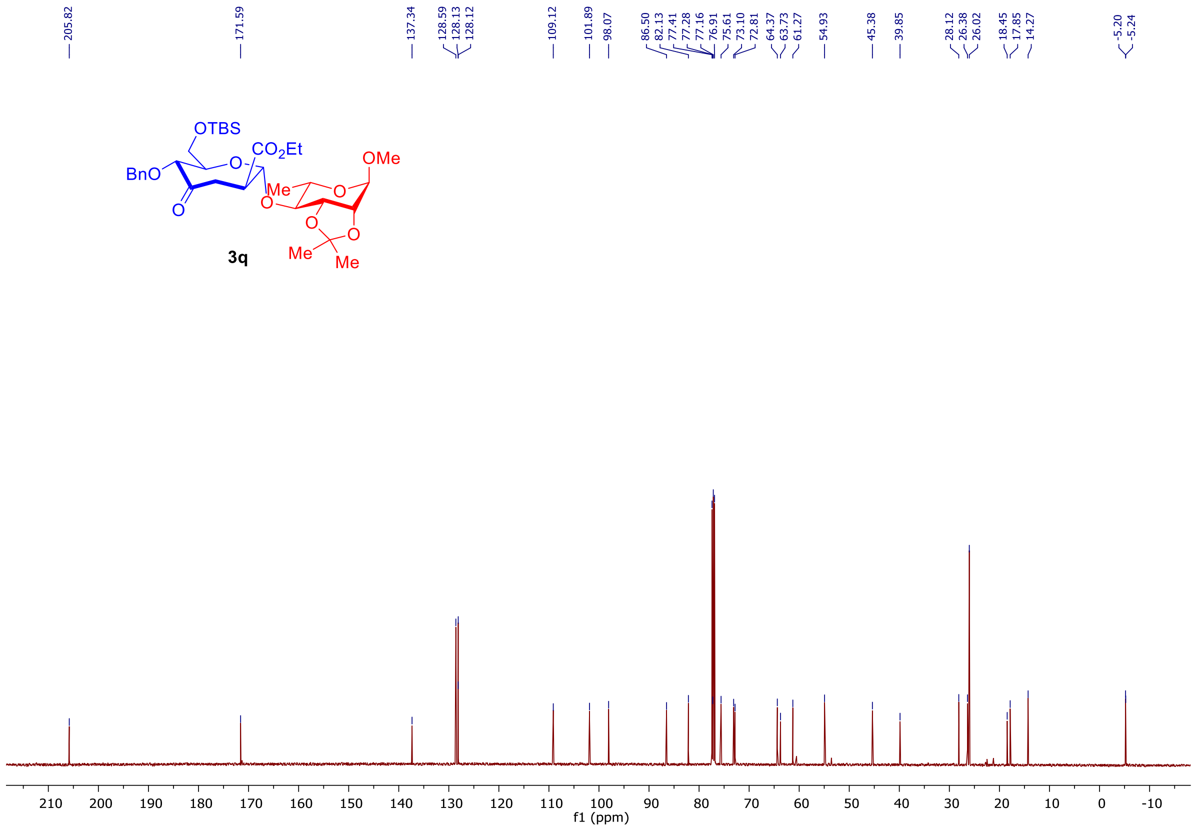

Supplementary Figure 261: ^{13}C spectra for **3q**

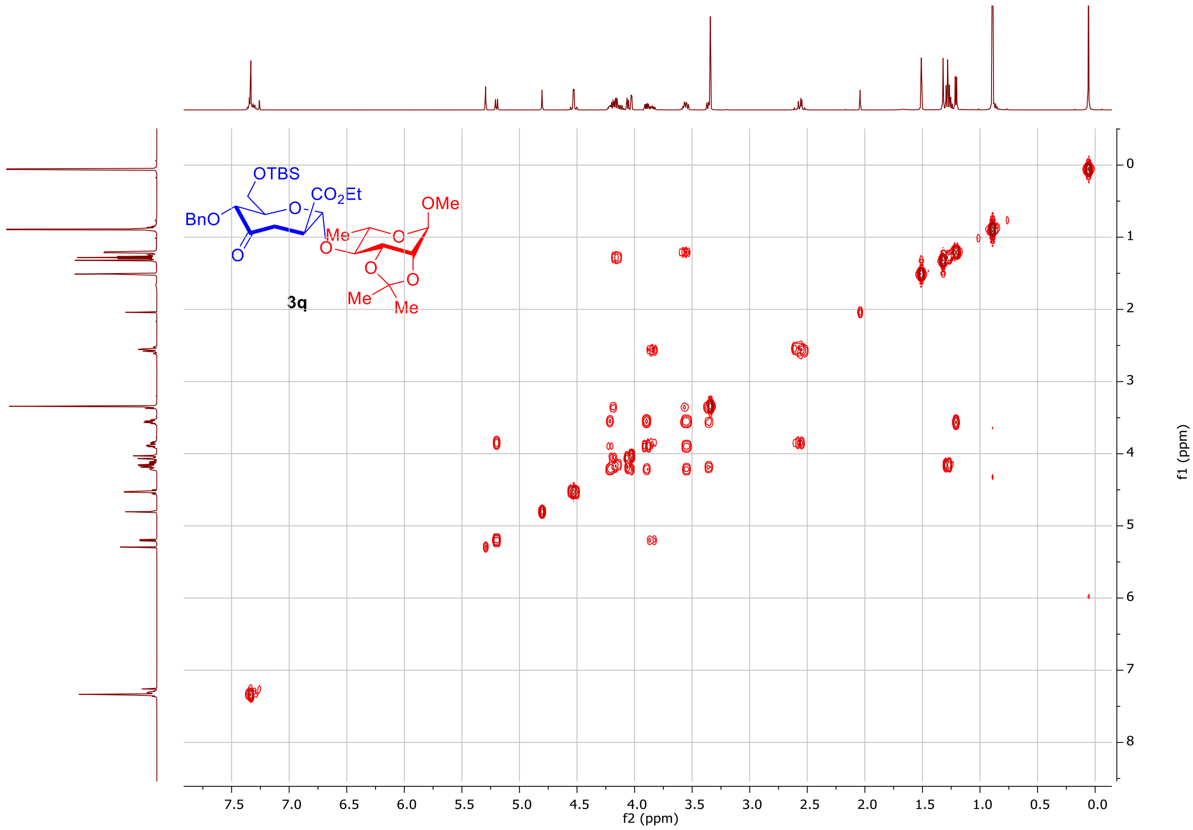

Supplementary Figure 262: COSY spectra for compound **3q**

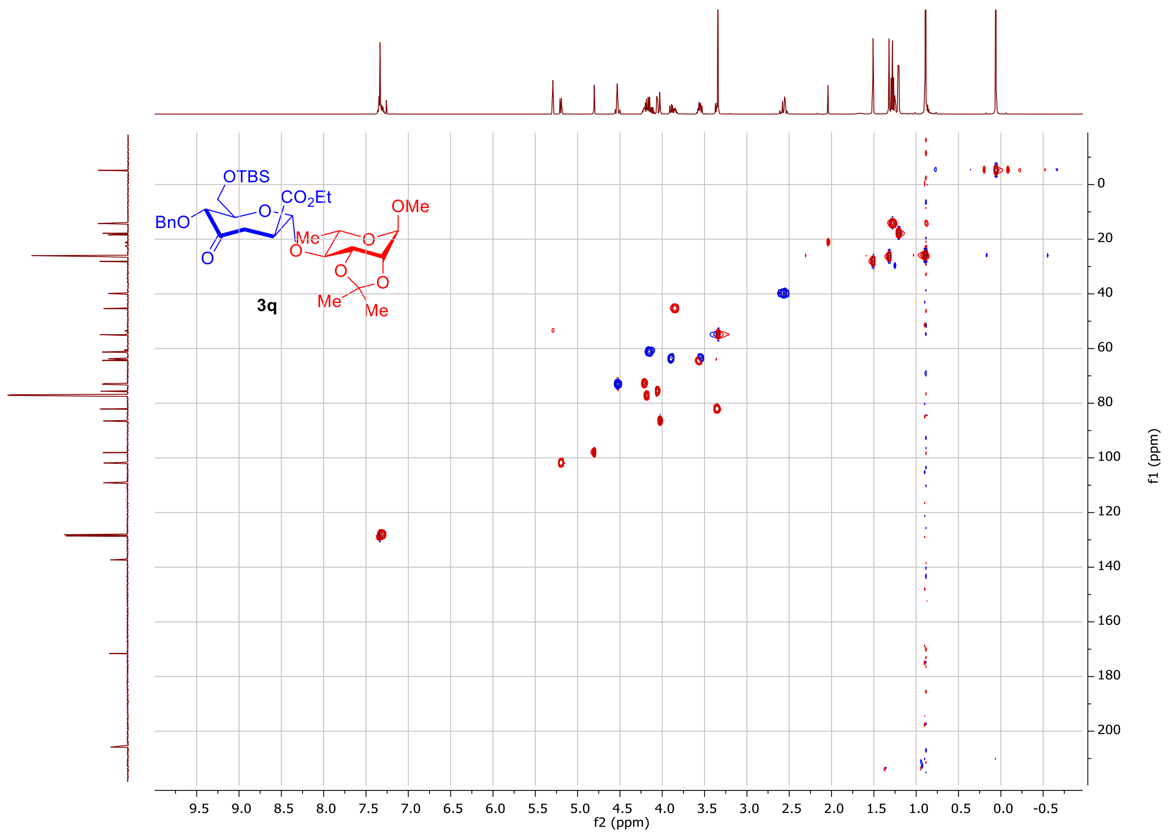

Supplementary Figure 263: HSQC spectra for compound **3q**

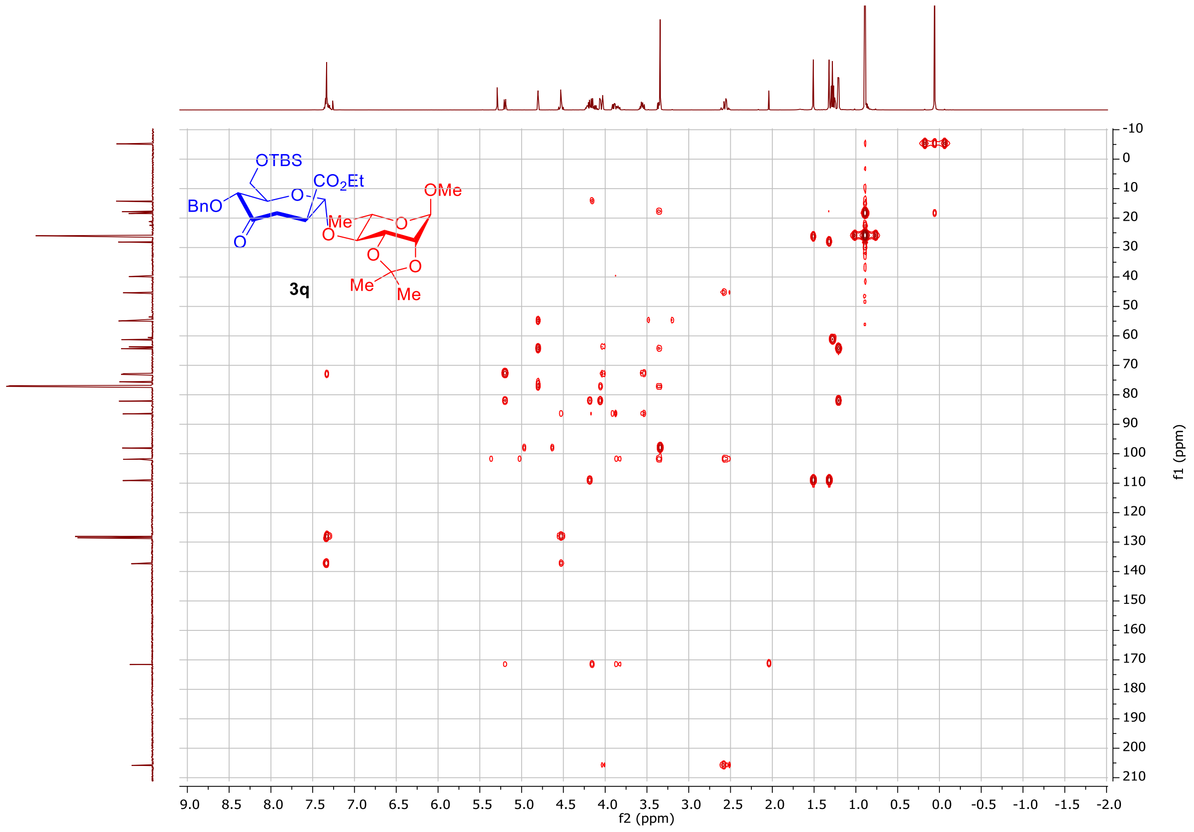

Supplementary Figure 264: HMBC spectra for compound **3q**

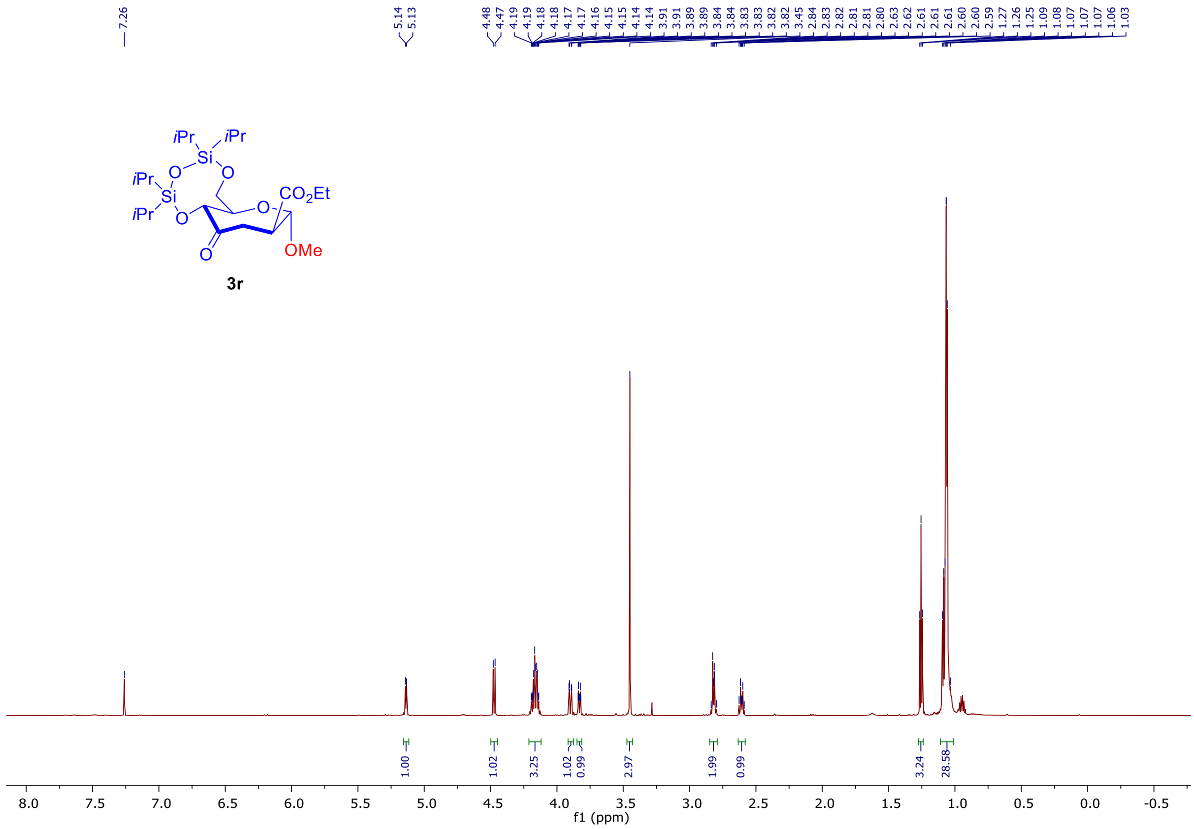

Supplementary Figure 265: ^{1}H spectra for **3r**

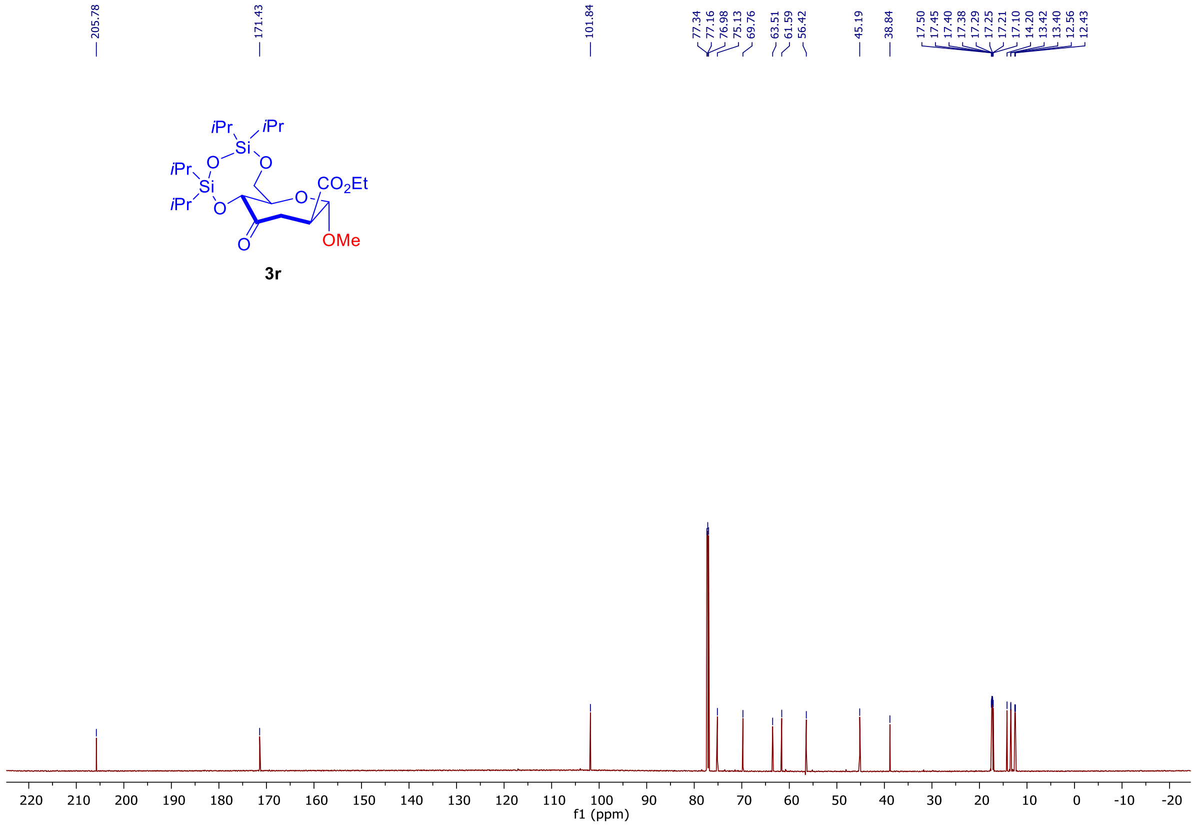

Supplementary Figure 266: ^{13}C spectra for **3r**

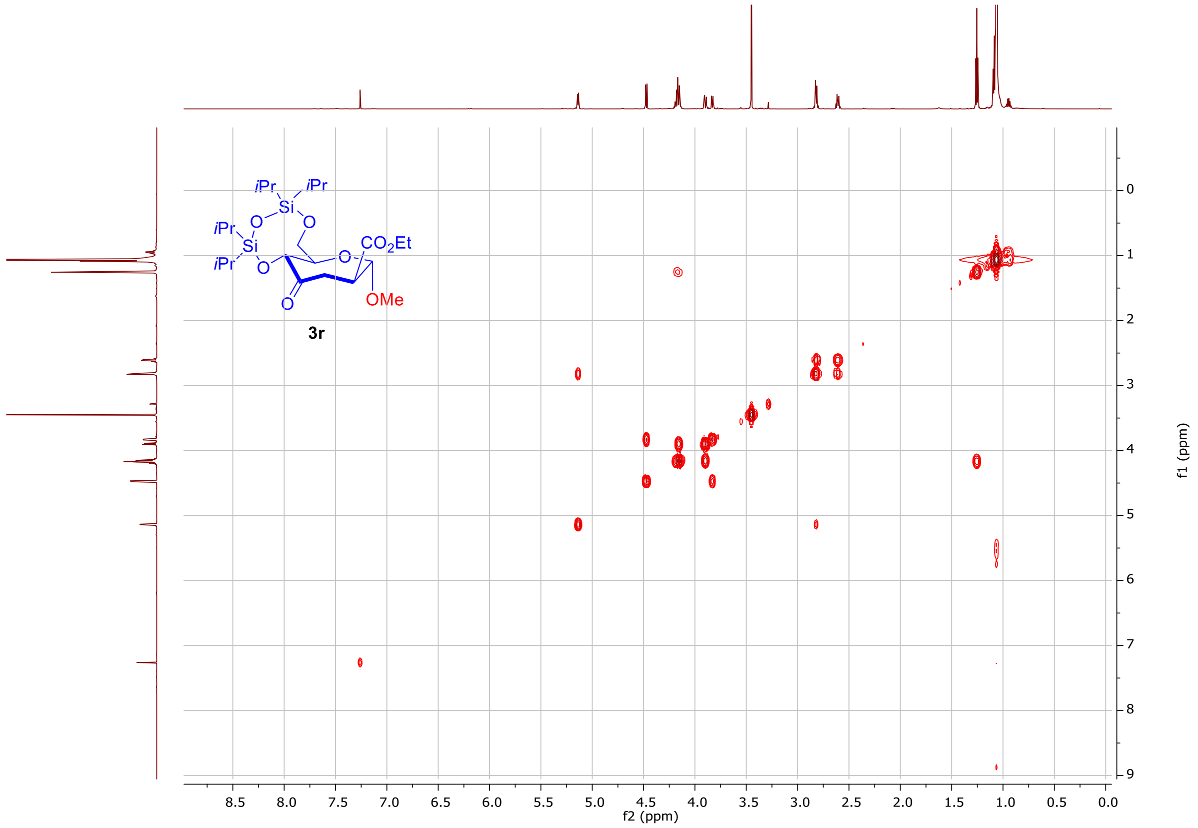

Supplementary Figure 267: COSY spectra for compound **3r**

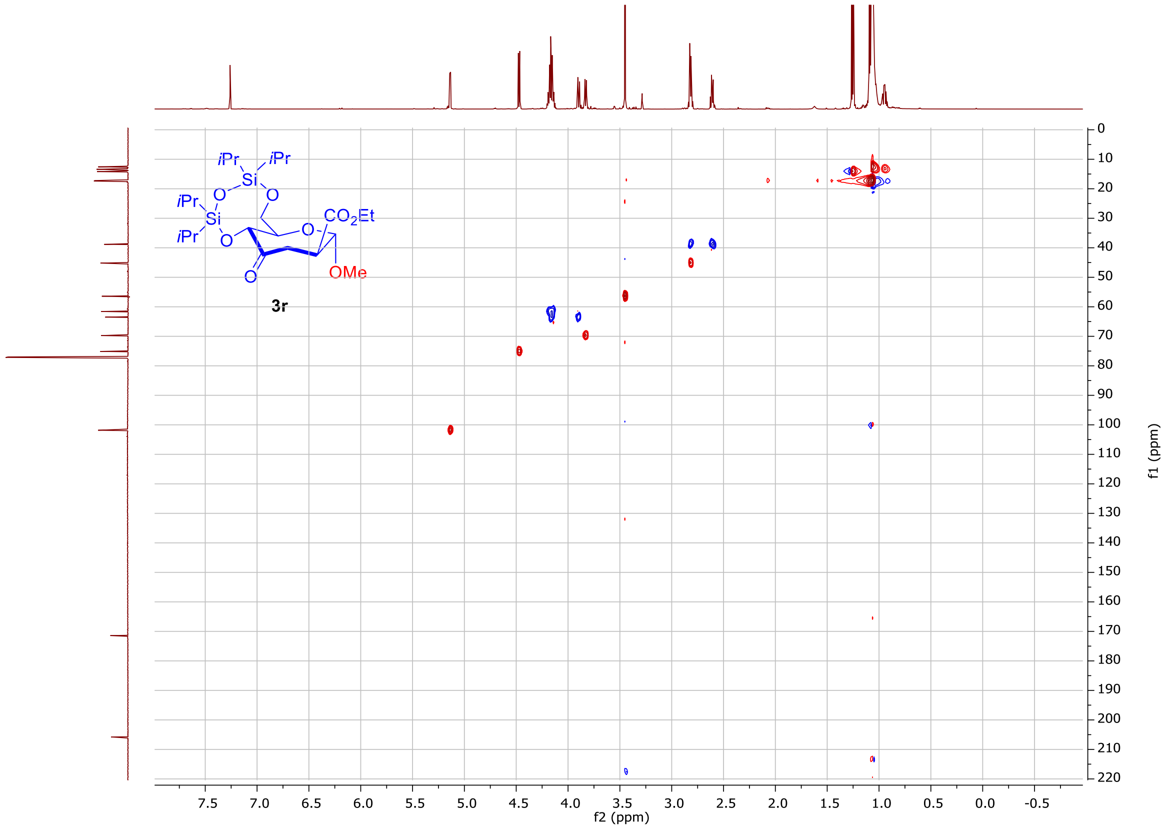

Supplementary Figure 268: HSQC spectra for compound **3r**

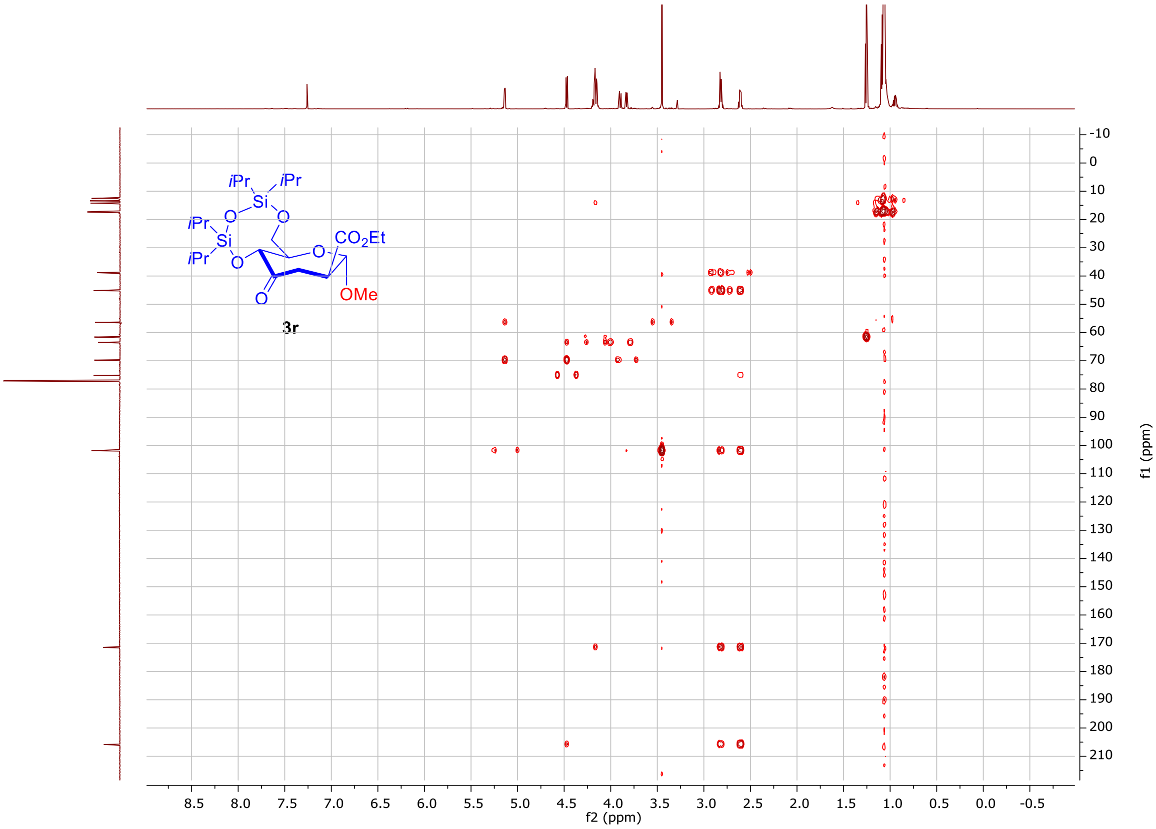

Supplementary Figure 269: HMBC spectra for compound **3r**

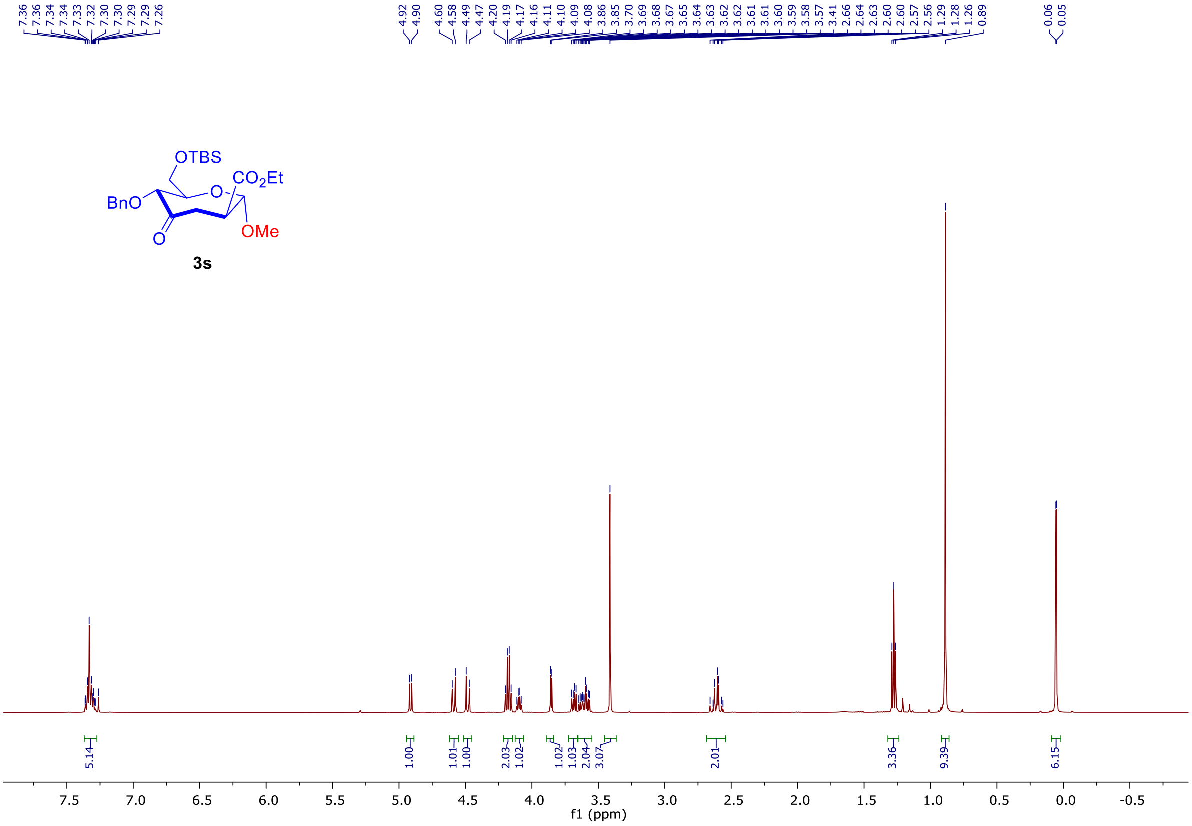

Supplementary Figure 270: ^{1}H spectra for **3s**

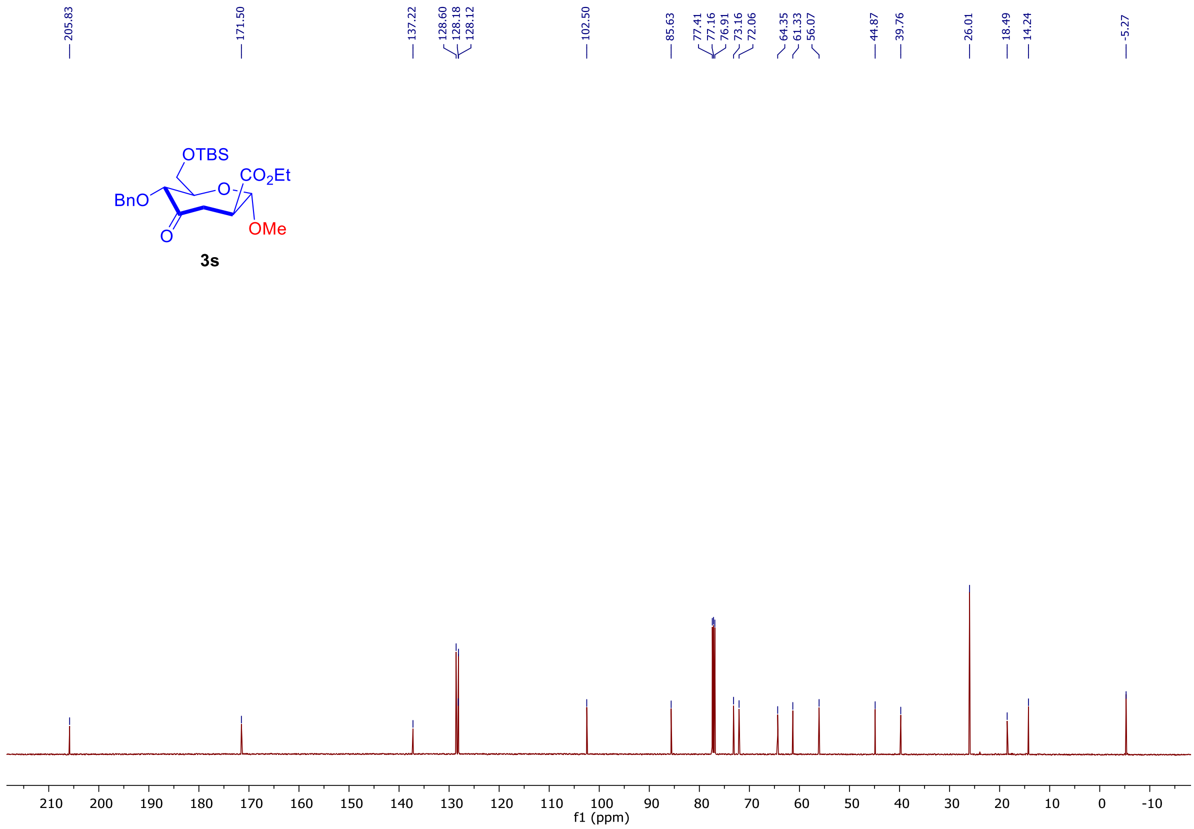

Supplementary Figure 271: ^{13}C spectra for **3s**

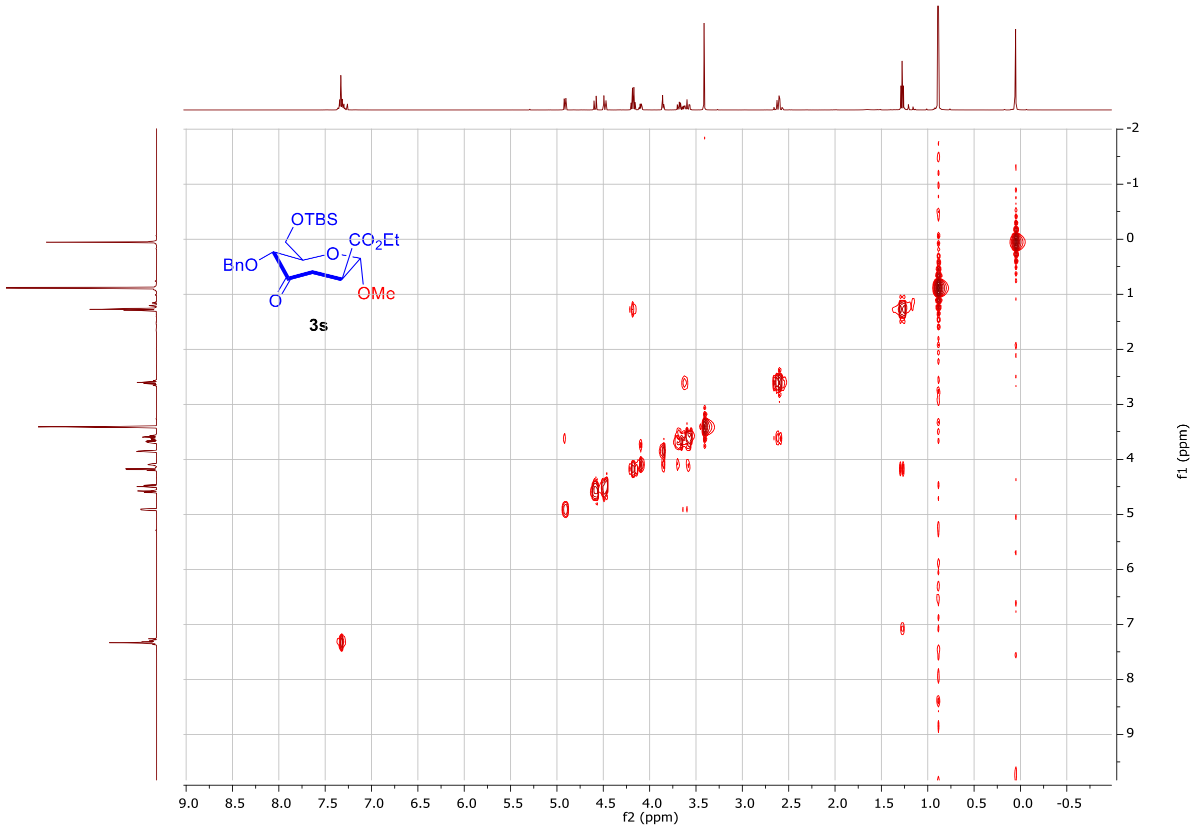

Supplementary Figure 272: COSY spectra for compound **3s**

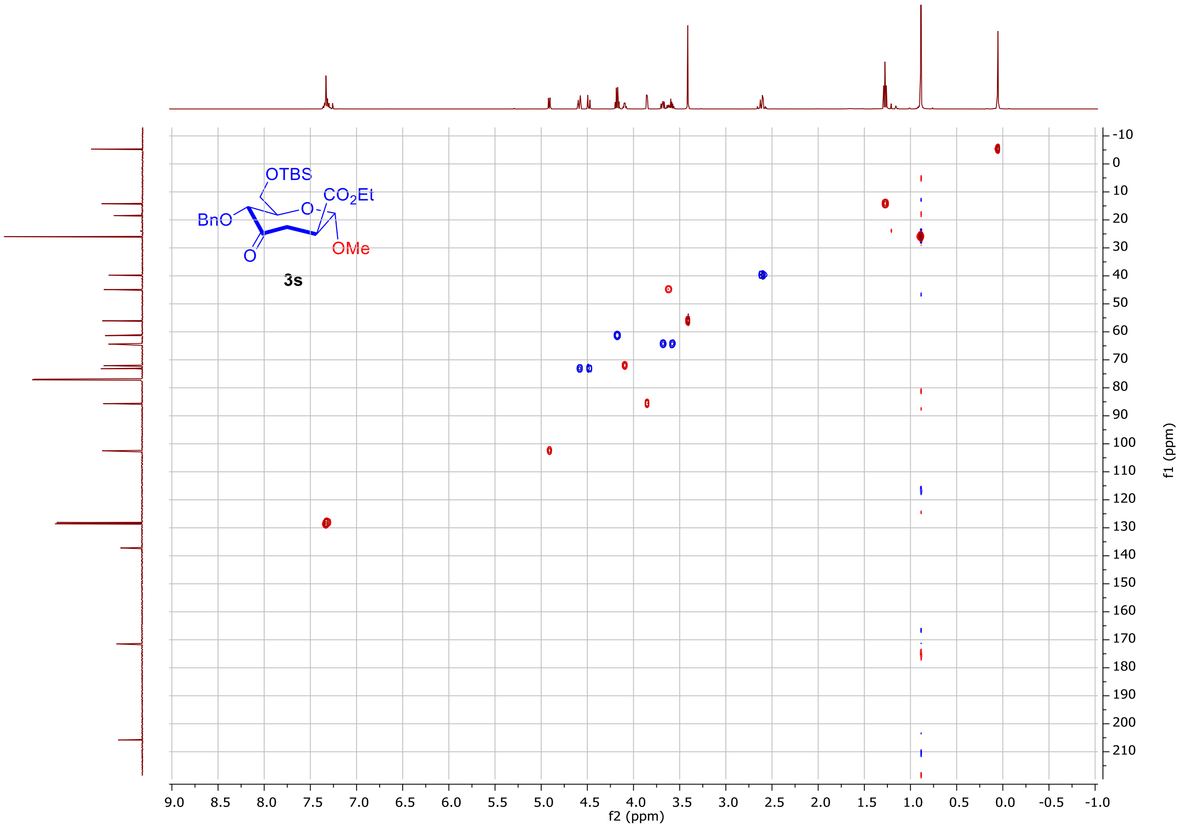

Supplementary Figure 273: HSQC spectra for compound **3s**

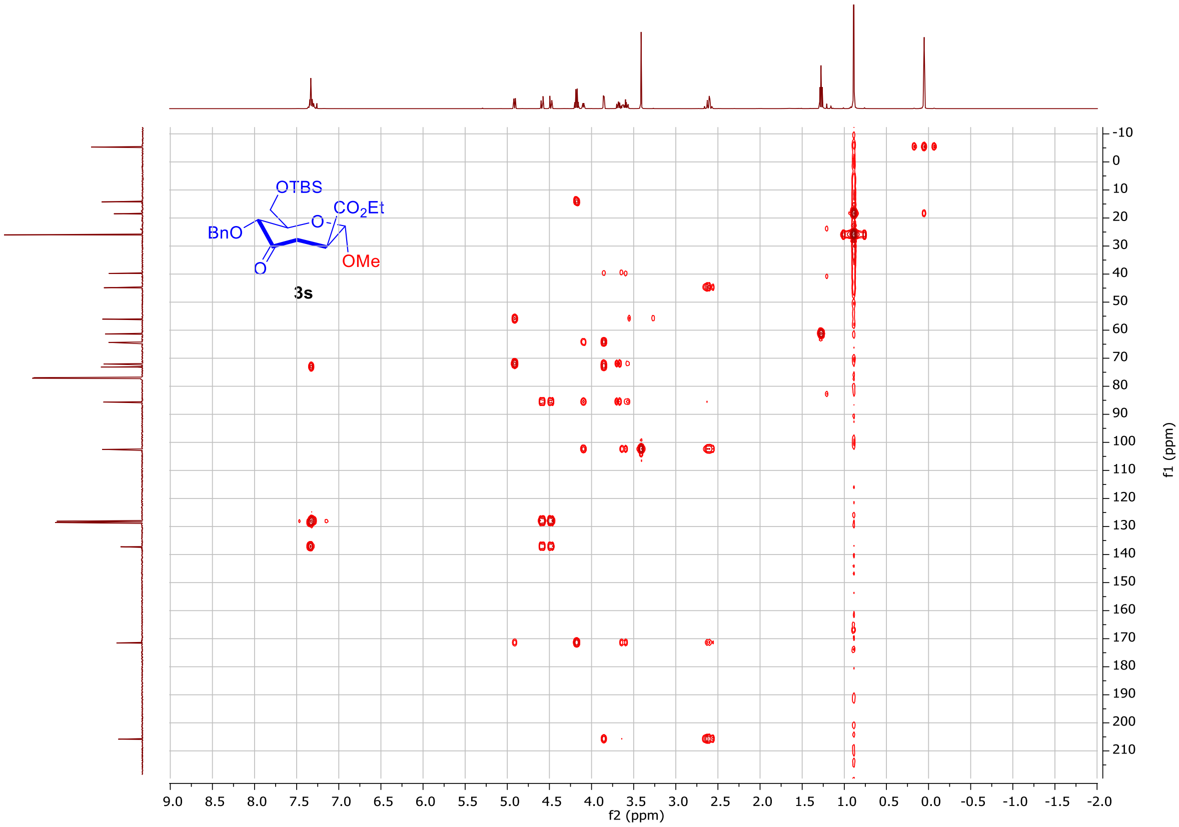

Supplementary Figure 274: HMBC spectra for compound **3s**

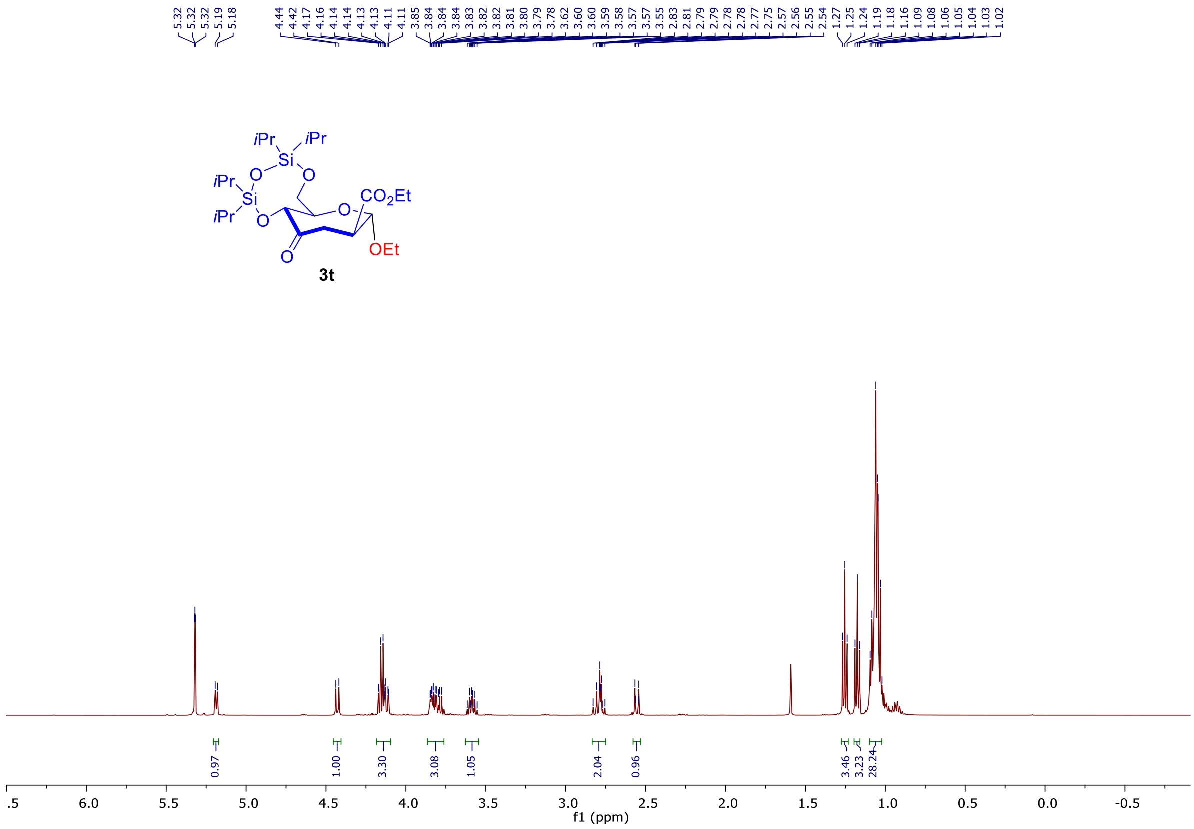

Supplementary Figure 275: ^{1}H spectra for **3t**

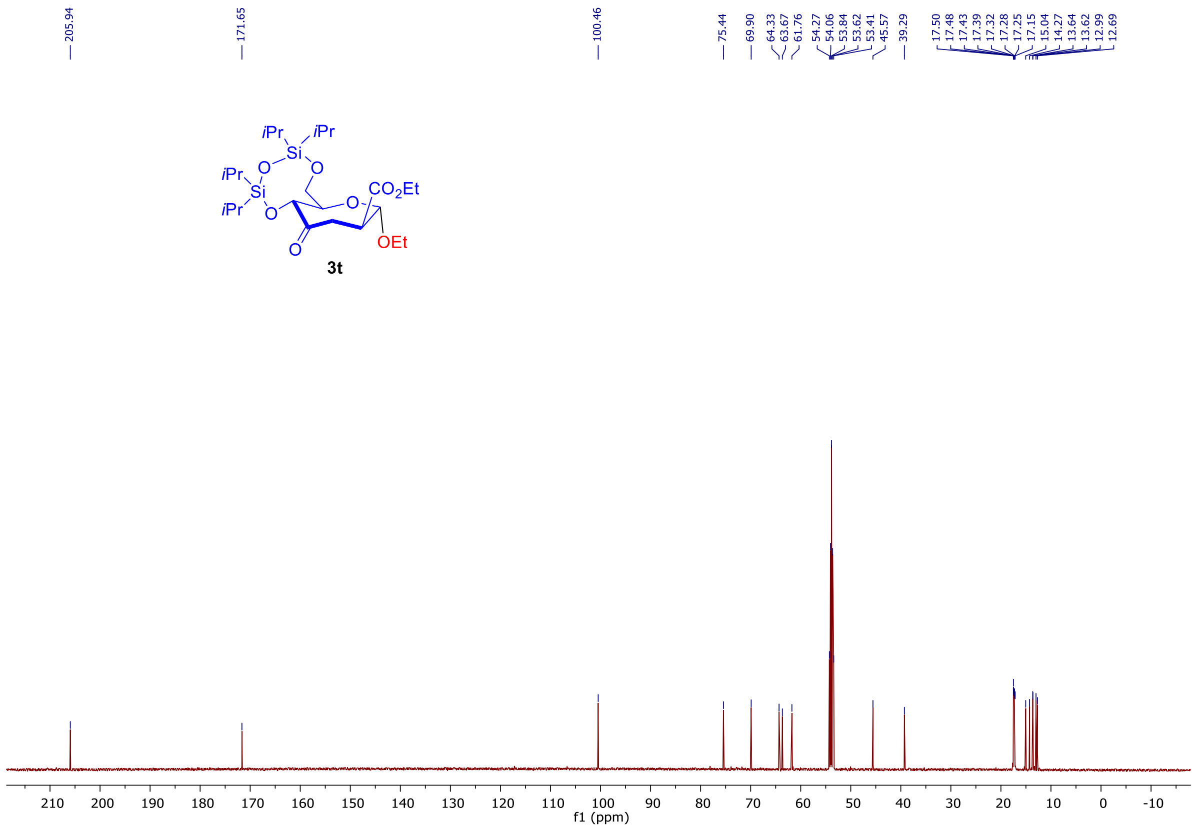

Supplementary Figure 276: ^{13}C spectra for **3t**

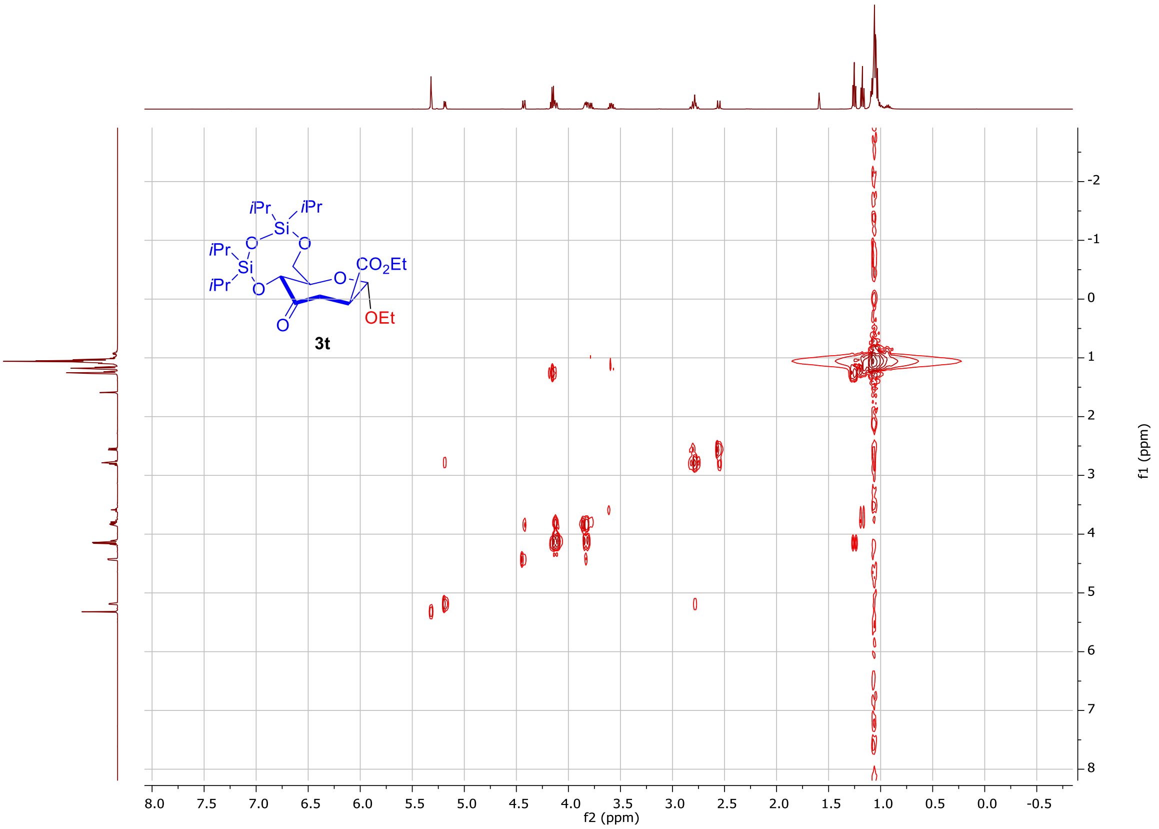

Supplementary Figure 277: COSY spectra for compound **3t**

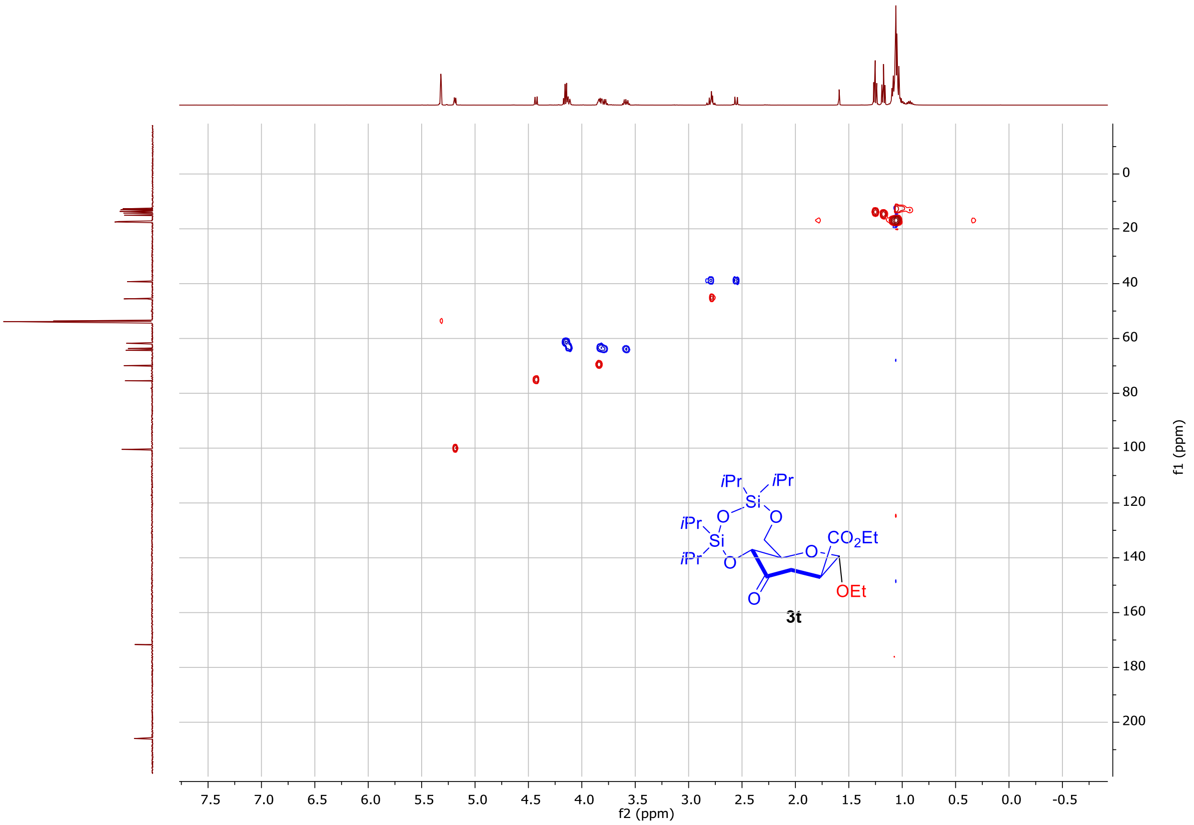

Supplementary Figure 278: HSQC spectra for compound **3t**

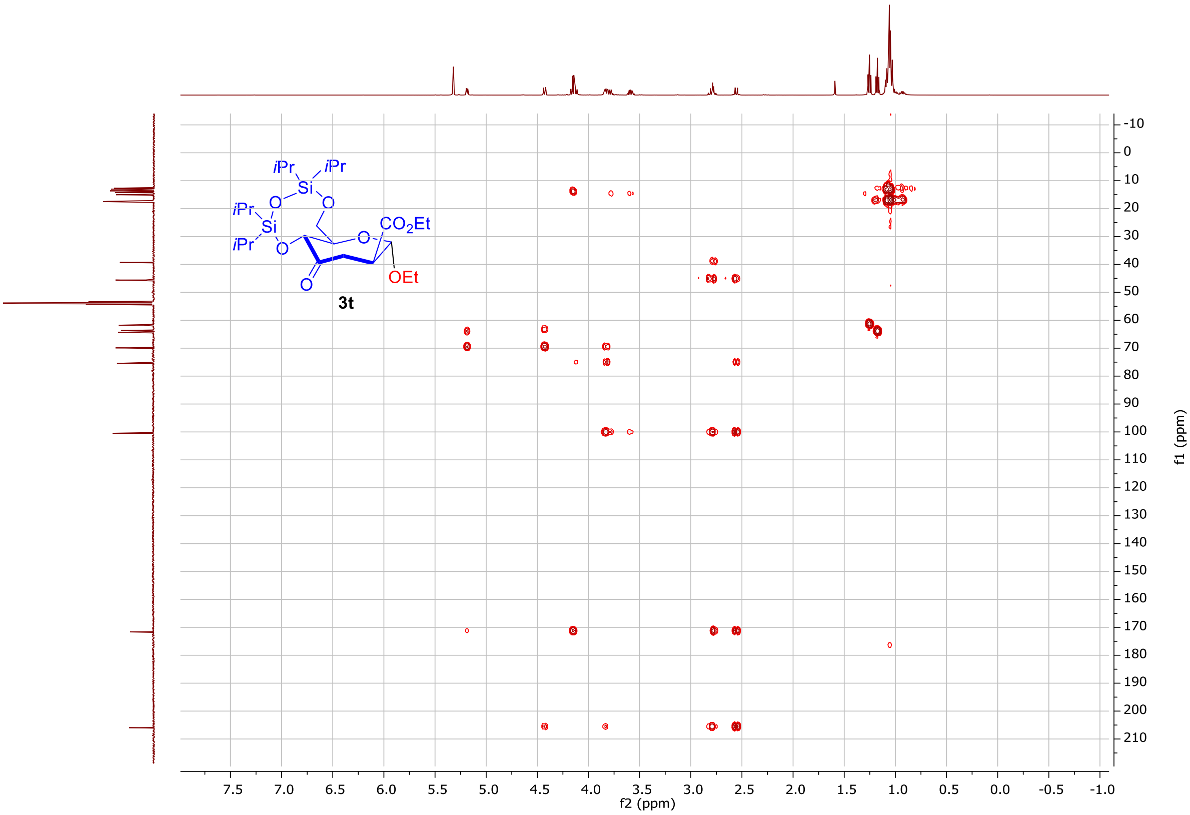

Supplementary Figure 279: HMBC spectra for compound **3t**

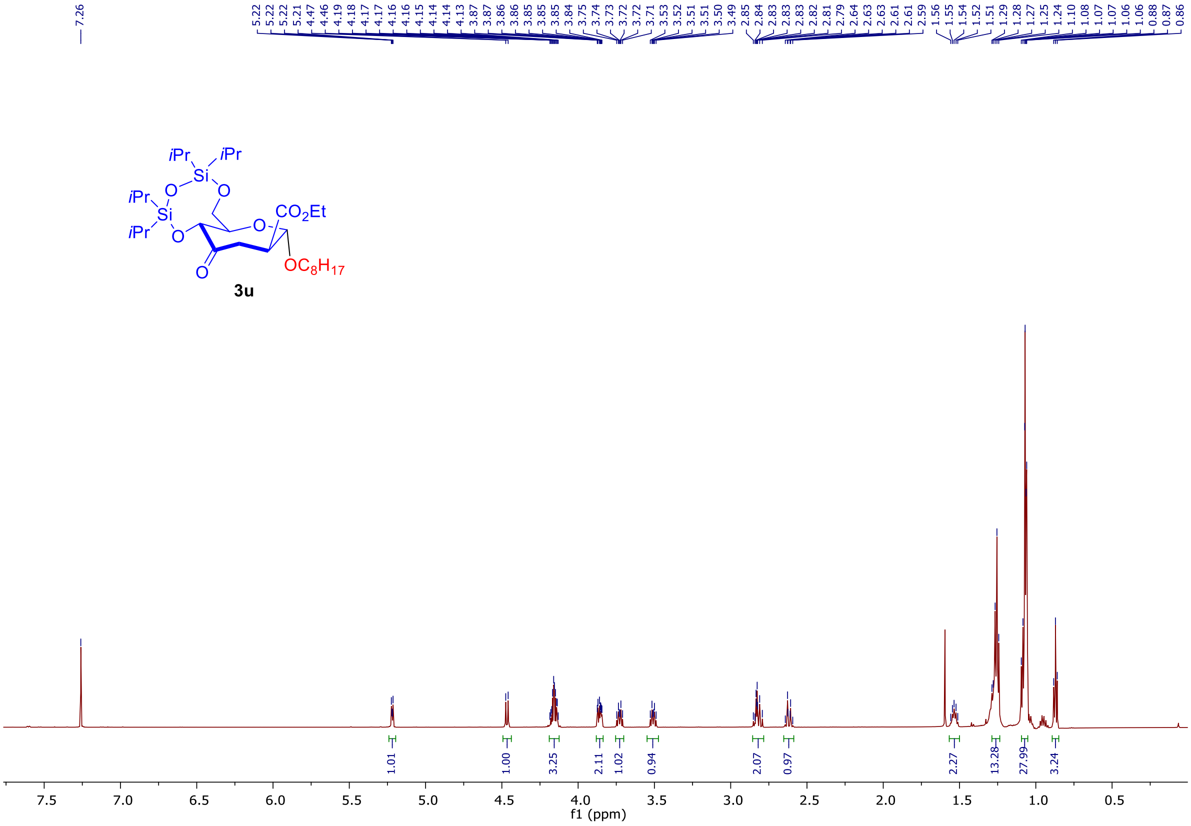

Supplementary Figure 280: ^{1}H spectra for **3u**

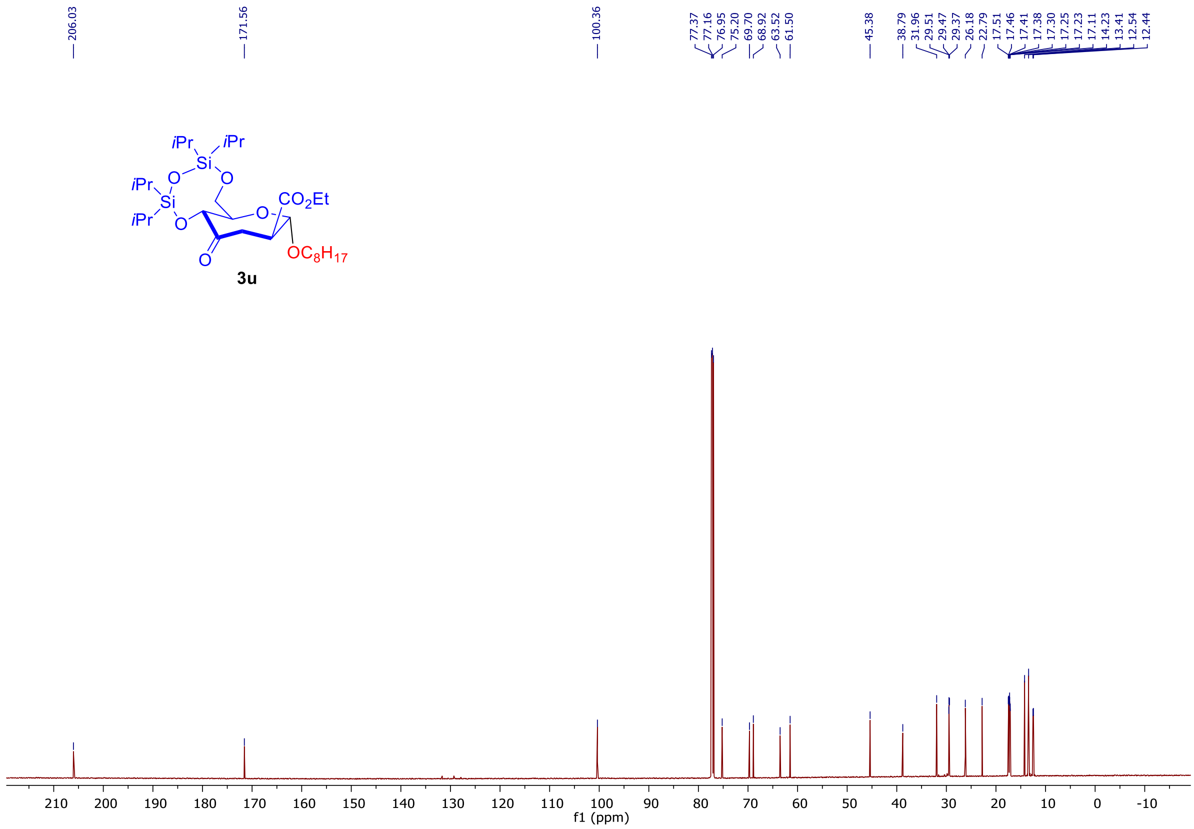

Supplementary Figure 281: ^{13}C spectra for **3u**

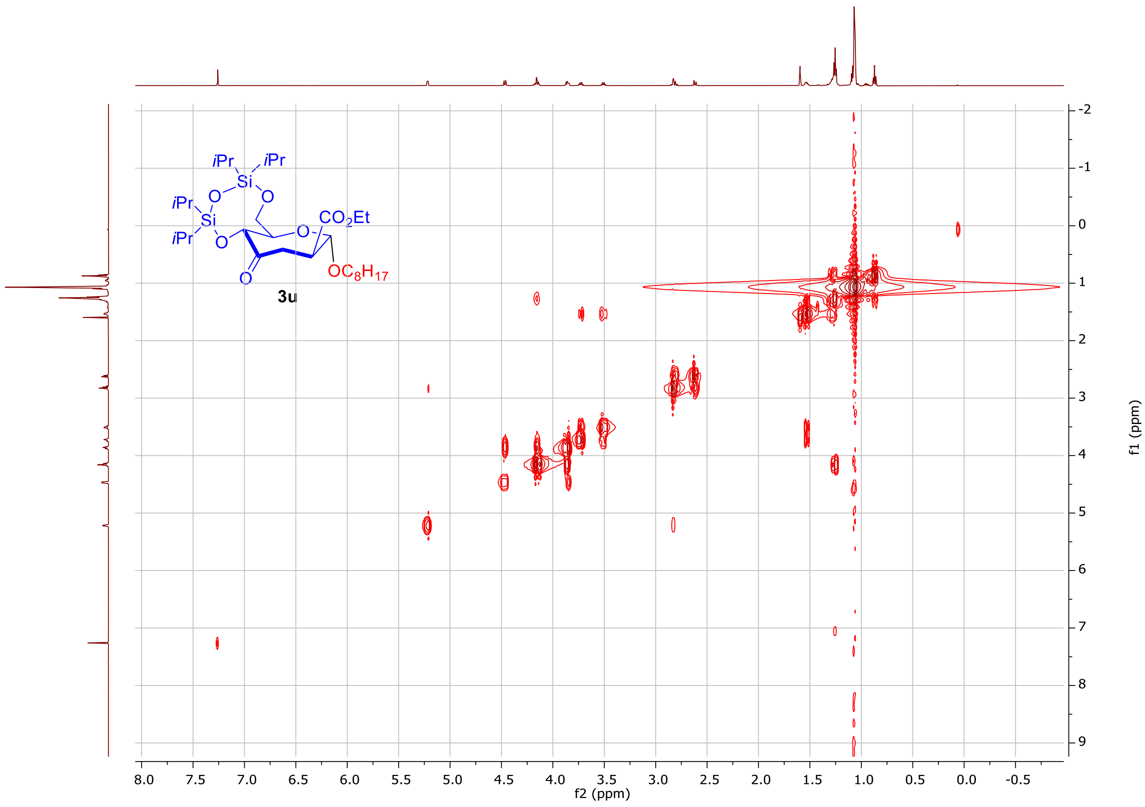

Supplementary Figure 282: COSY spectra for compound **3u**

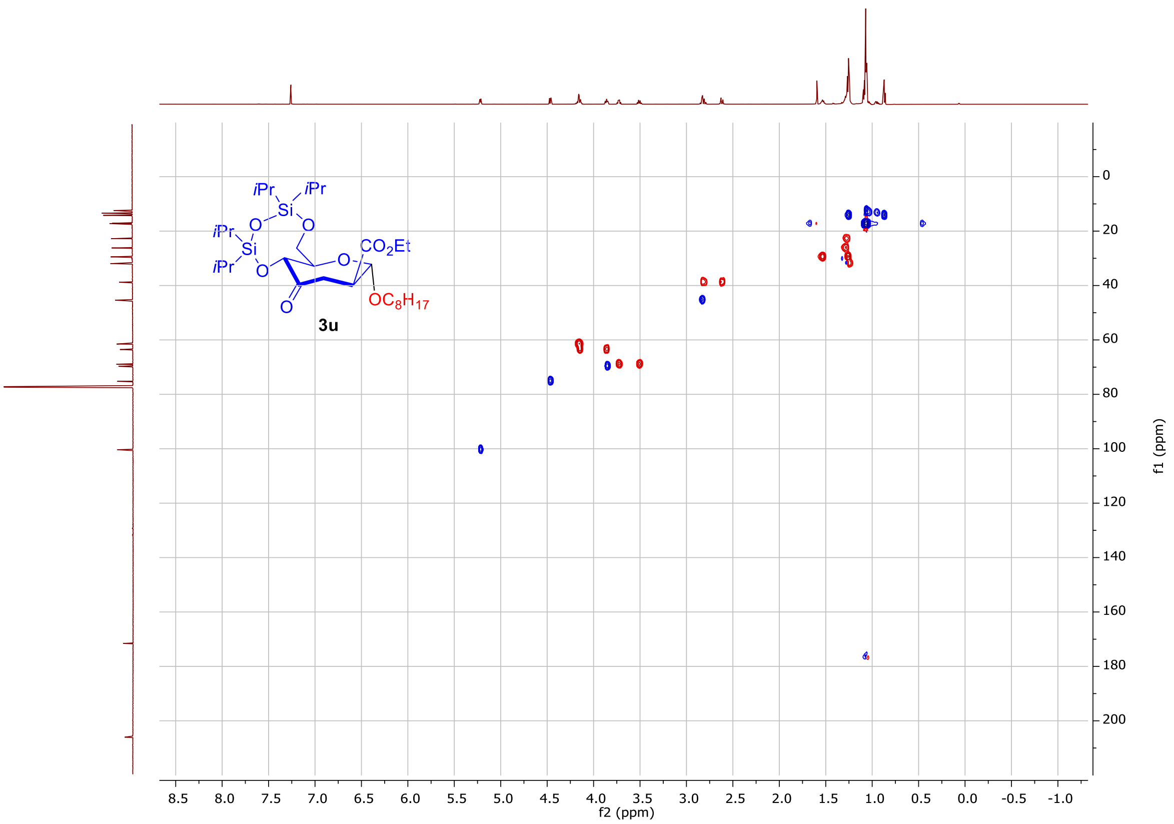

Supplementary Figure 283: HSQC spectra for compound **3u**

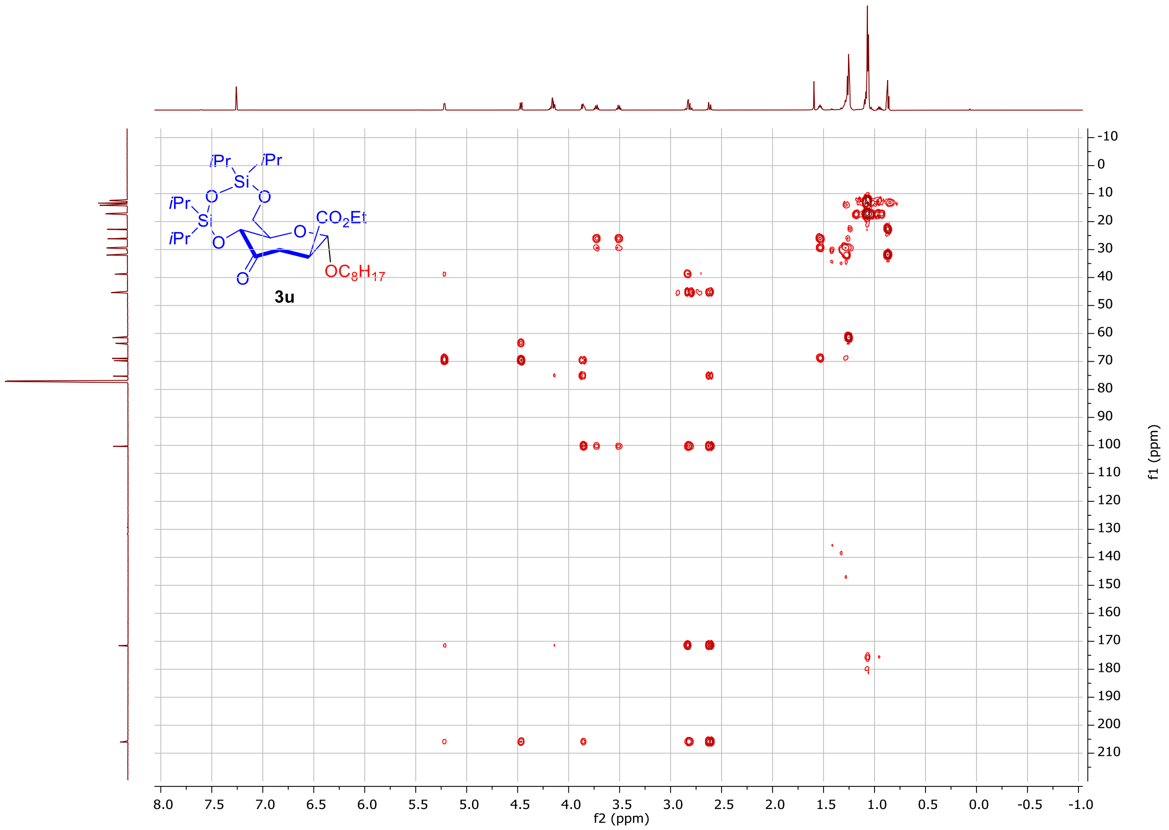

Supplementary Figure 284: HMBC spectra for compound **3u**

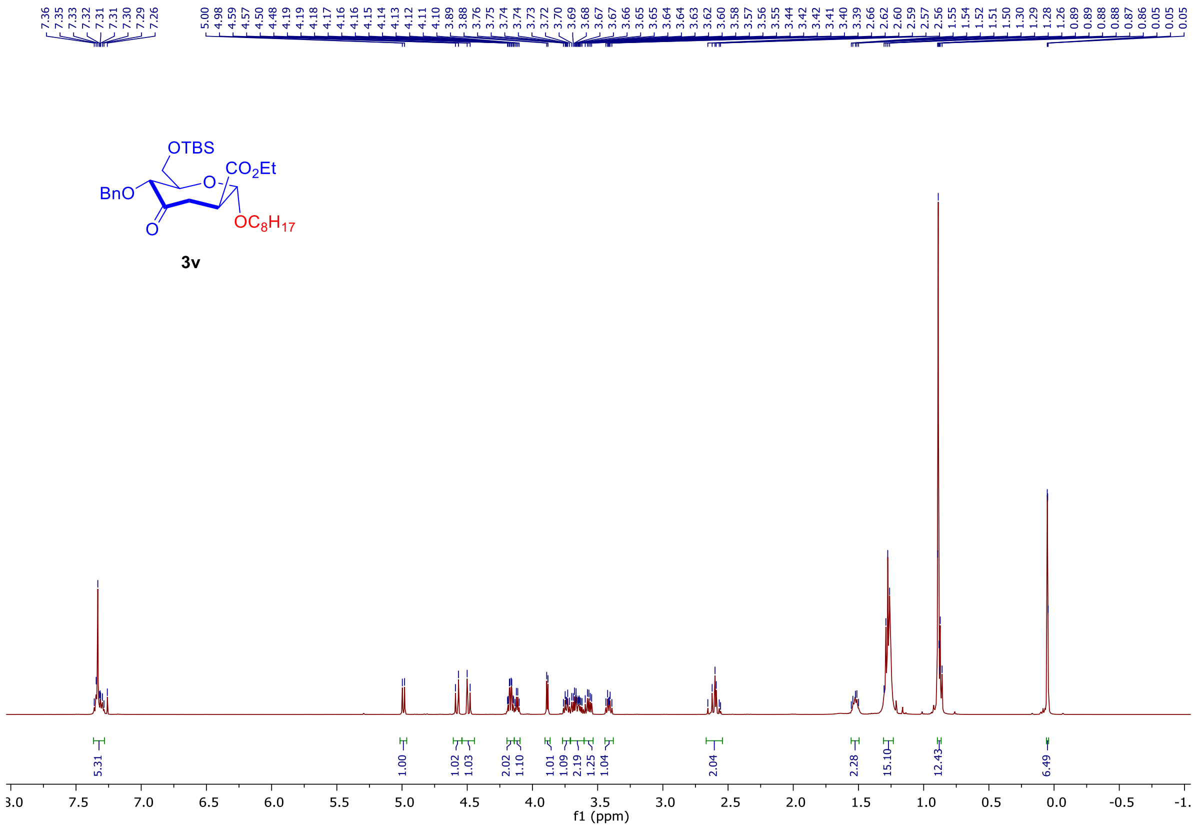

Supplementary Figure 285: ^{1}H spectra for **3v**

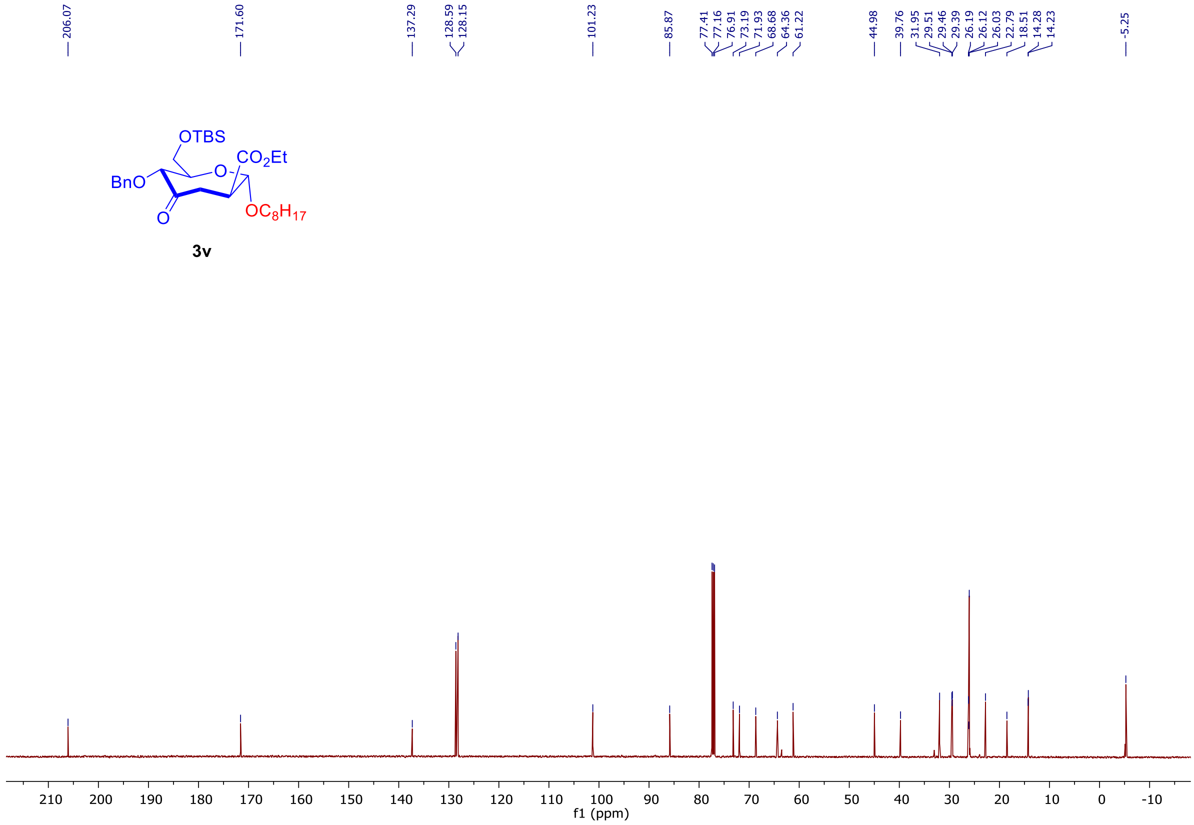

Supplementary Figure 286: ^{13}C spectra for **3v**

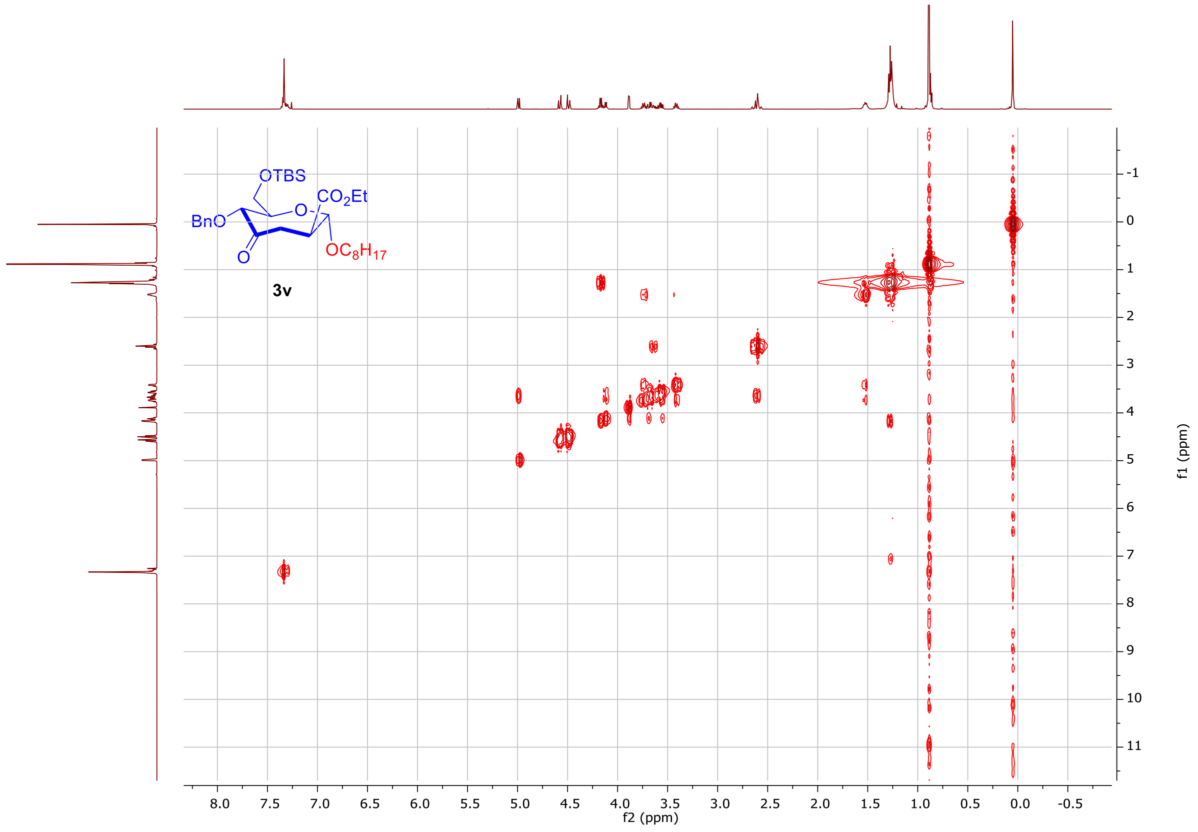

Supplementary Figure 287: COSY spectra for compound **3v**

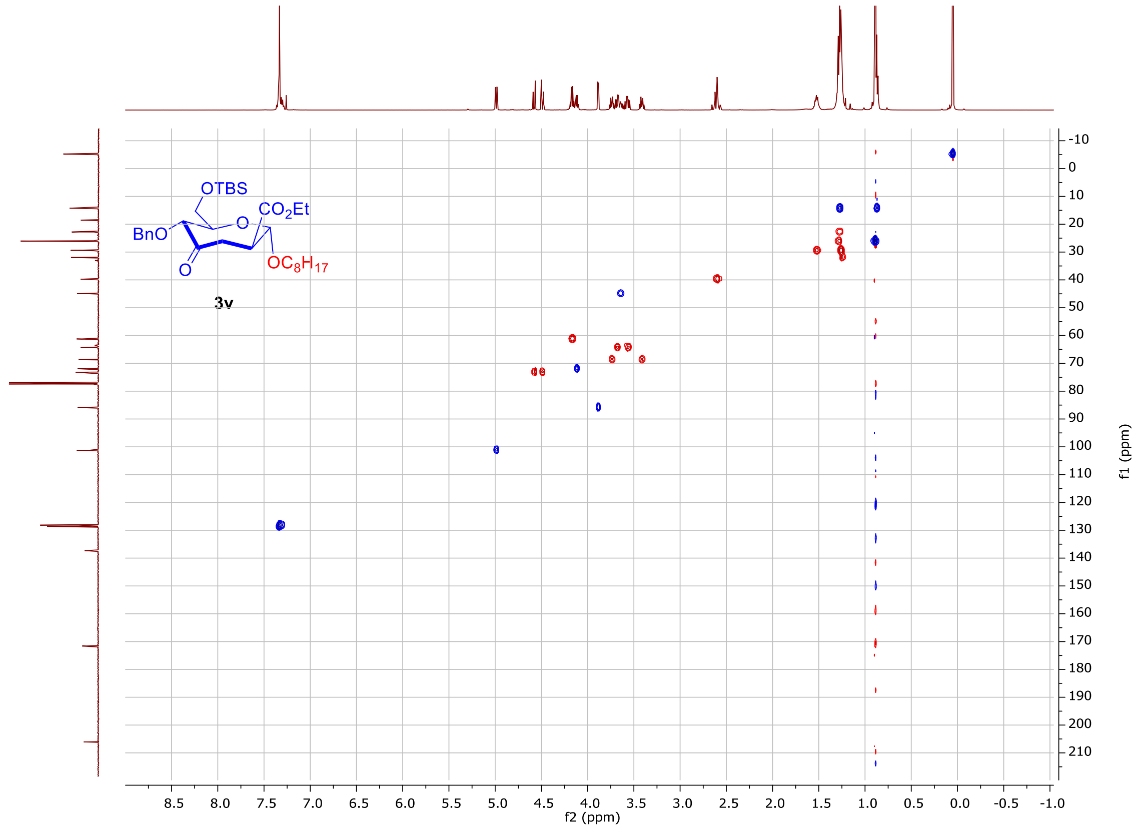

Supplementary Figure 288: HSQC spectra for compound **3v**

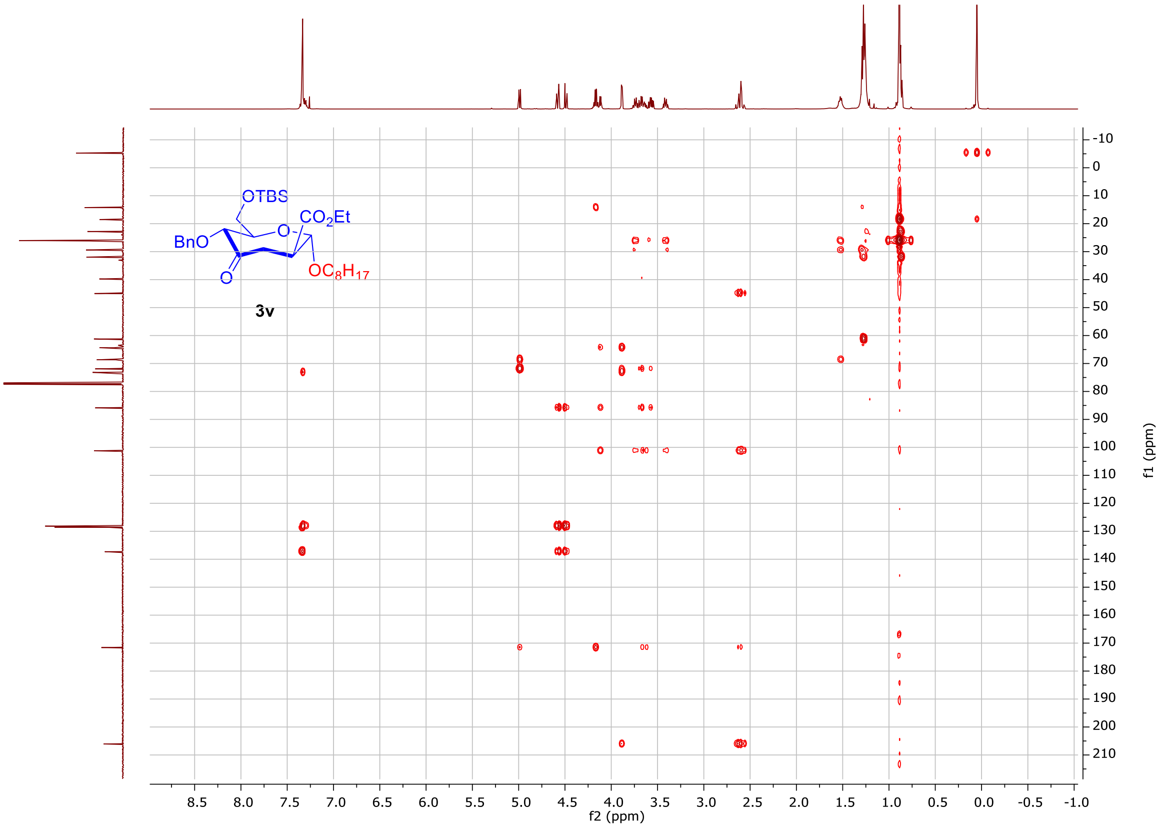

Supplementary Figure 289: HMBC spectra for compound **3v**

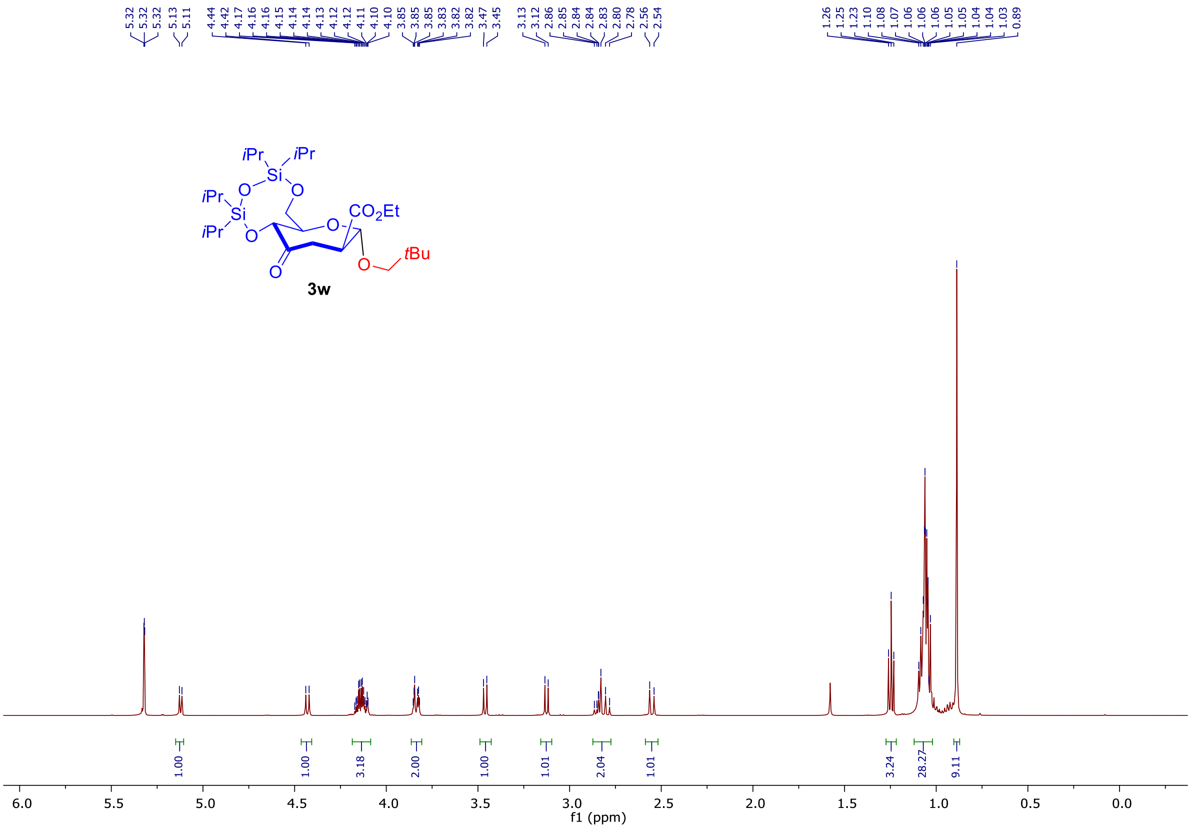

Supplementary Figure 290: ^{1}H spectra for **3w**

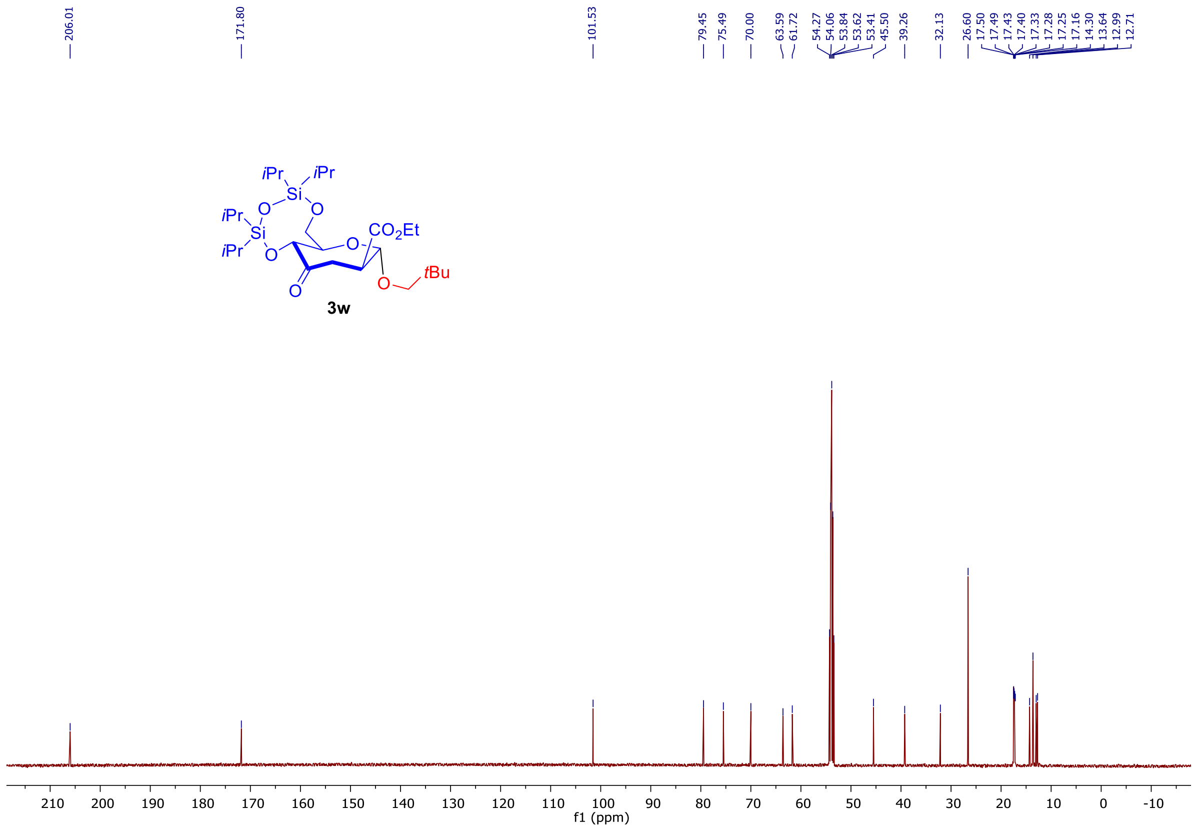

Supplementary Figure 291: ^{13}C spectra for **3w**

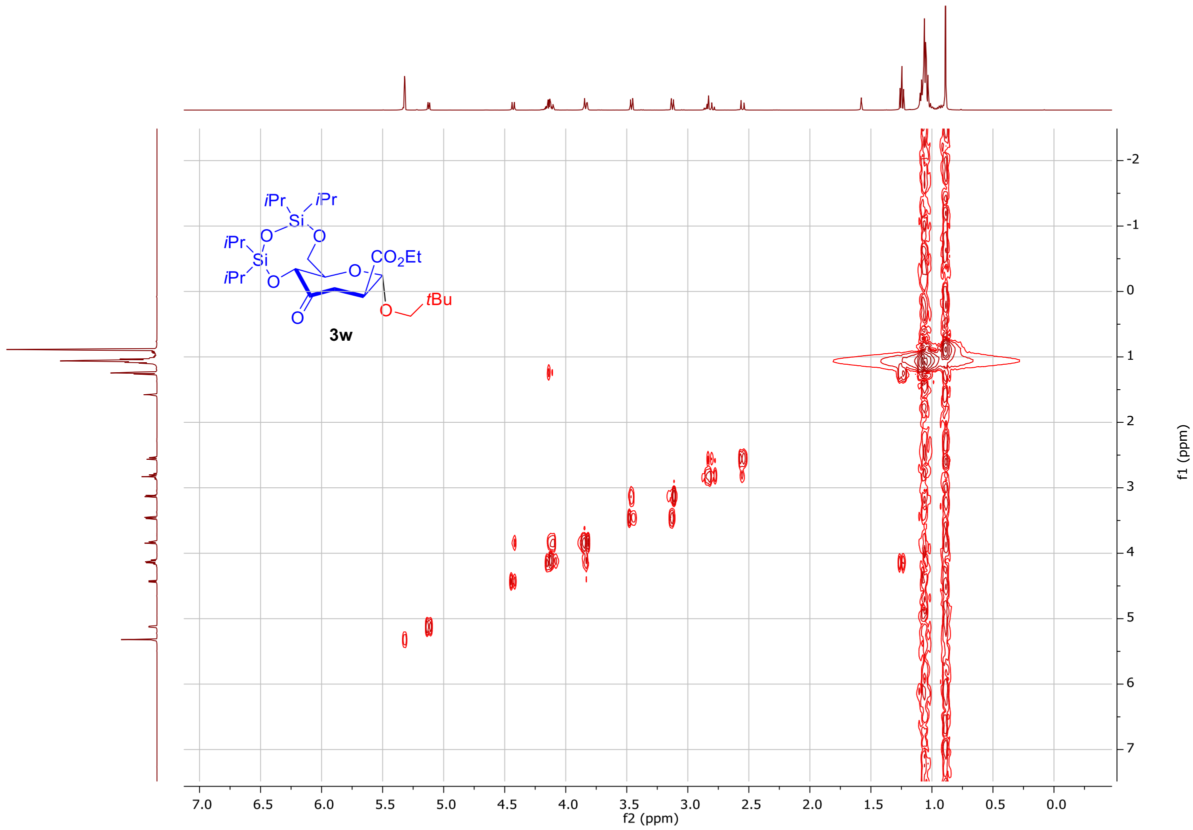

Supplementary Figure 292: COSY spectra for compound **3w**

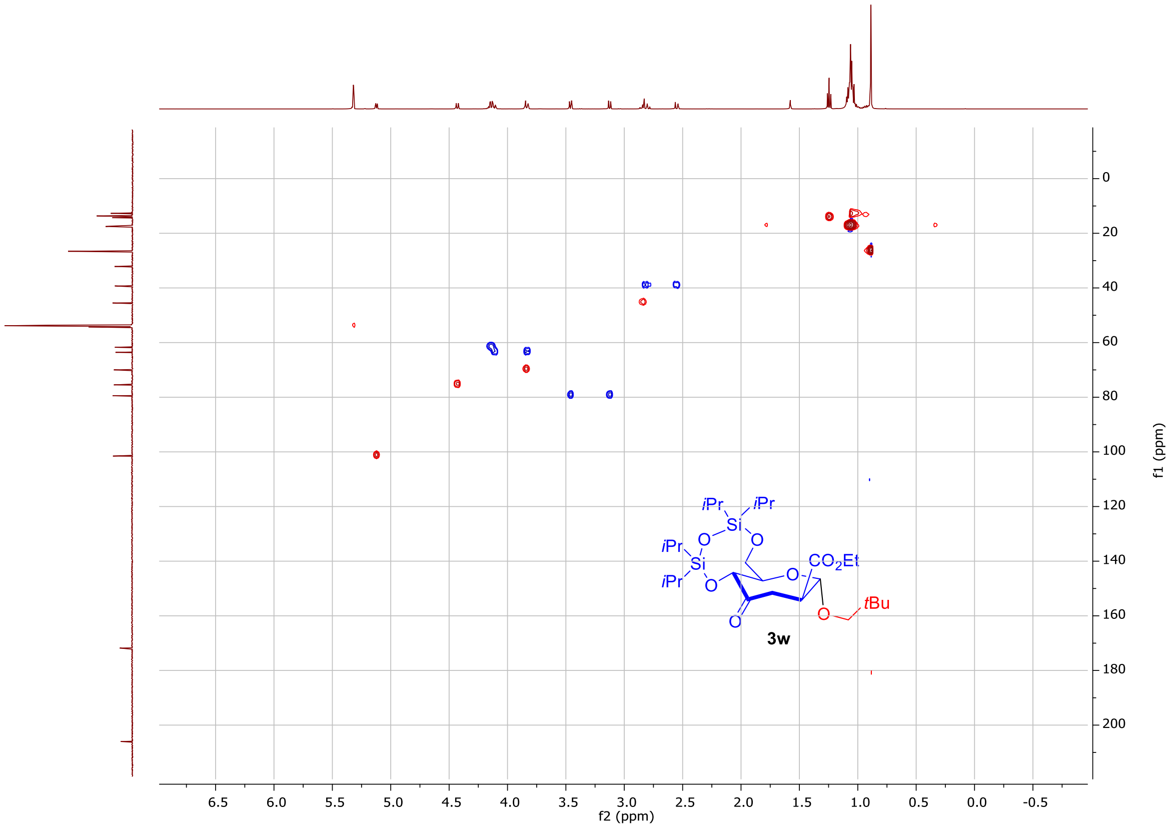

Supplementary Figure 293: HSQC spectra for compound **3w**

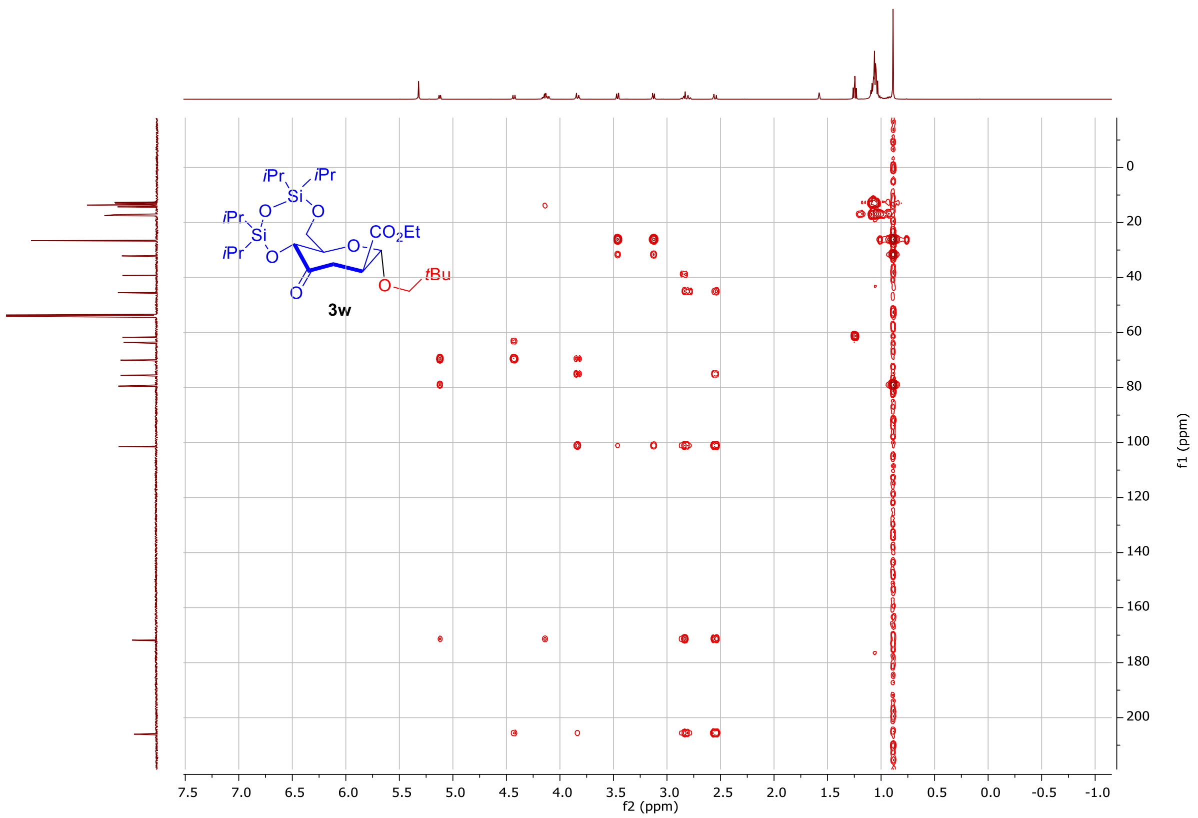

Supplementary Figure 294: HMBC spectra for compound **3w**

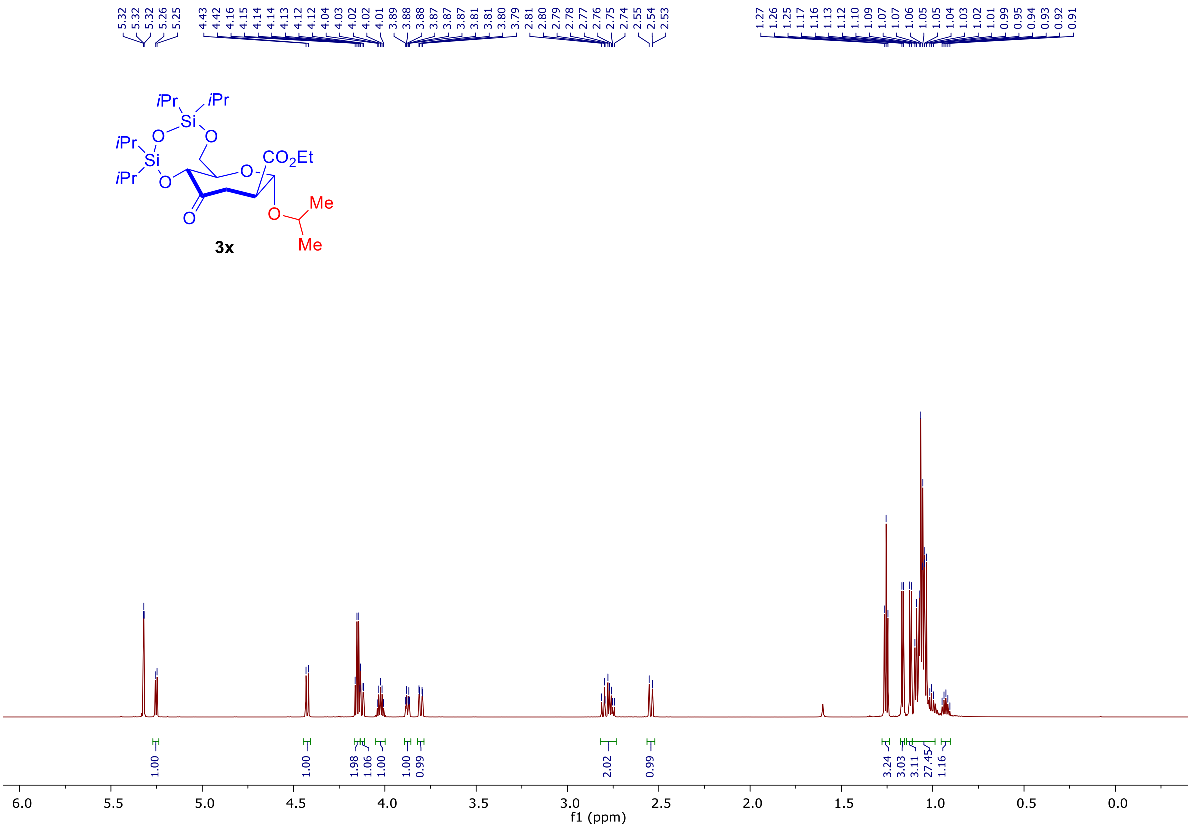

Supplementary Figure 295: ^{1}H spectra for **3x**

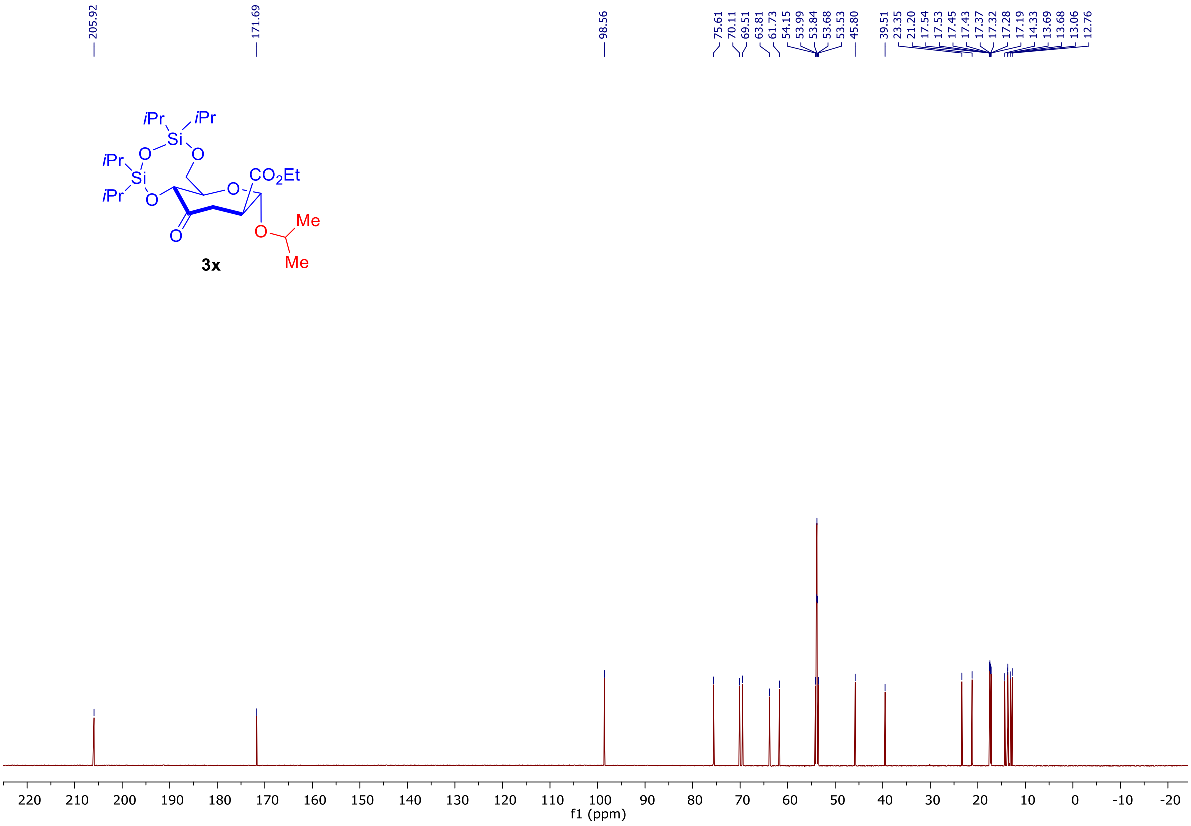

Supplementary Figure 296: ^{13}C spectra for **3x**

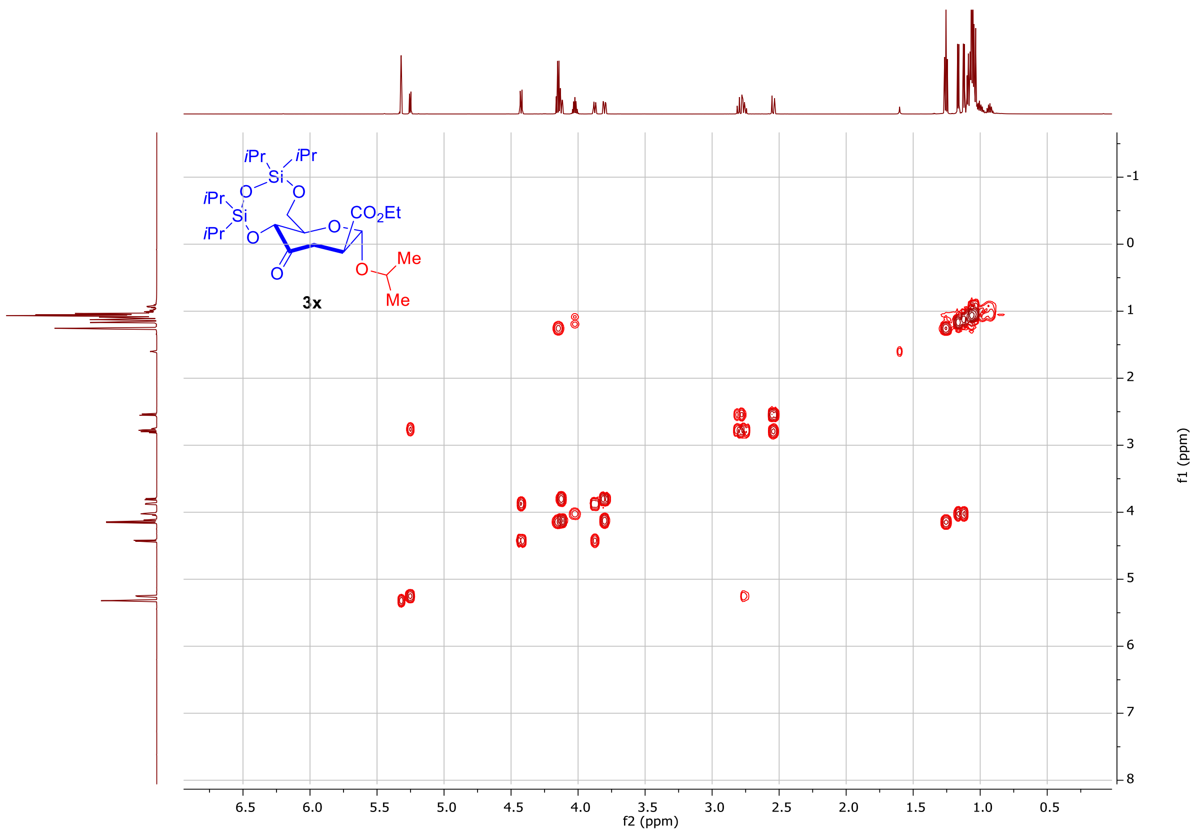

Supplementary Figure 297: COSY spectra for compound **3x**

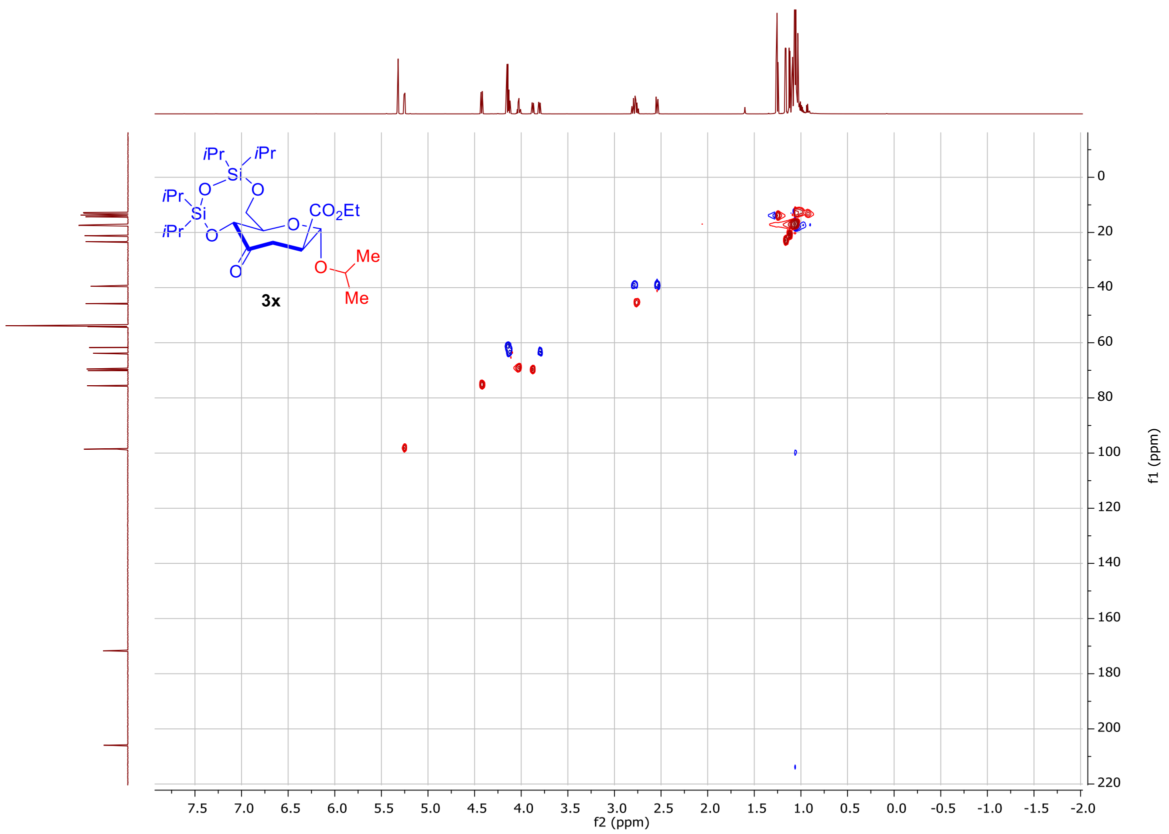

Supplementary Figure 298: HSQC spectra for compound **3x**

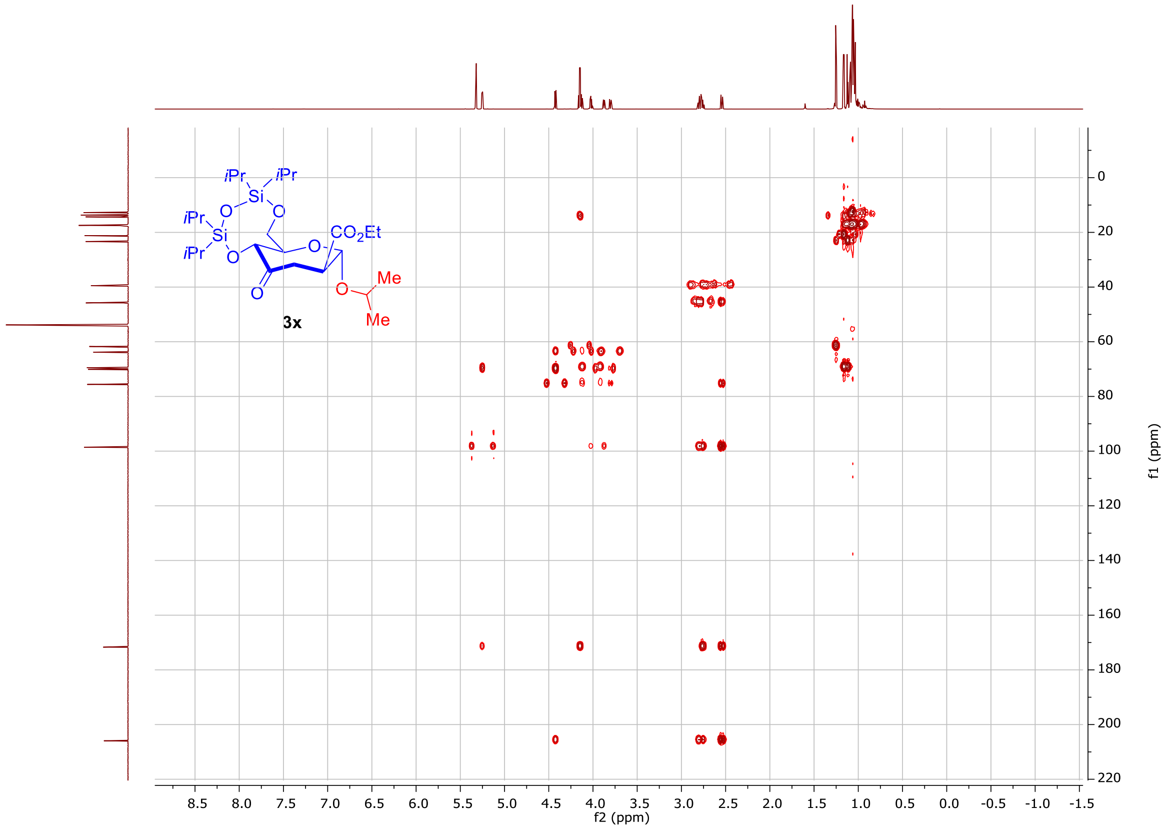

Supplementary Figure 299: HMBC spectra for compound **3x**

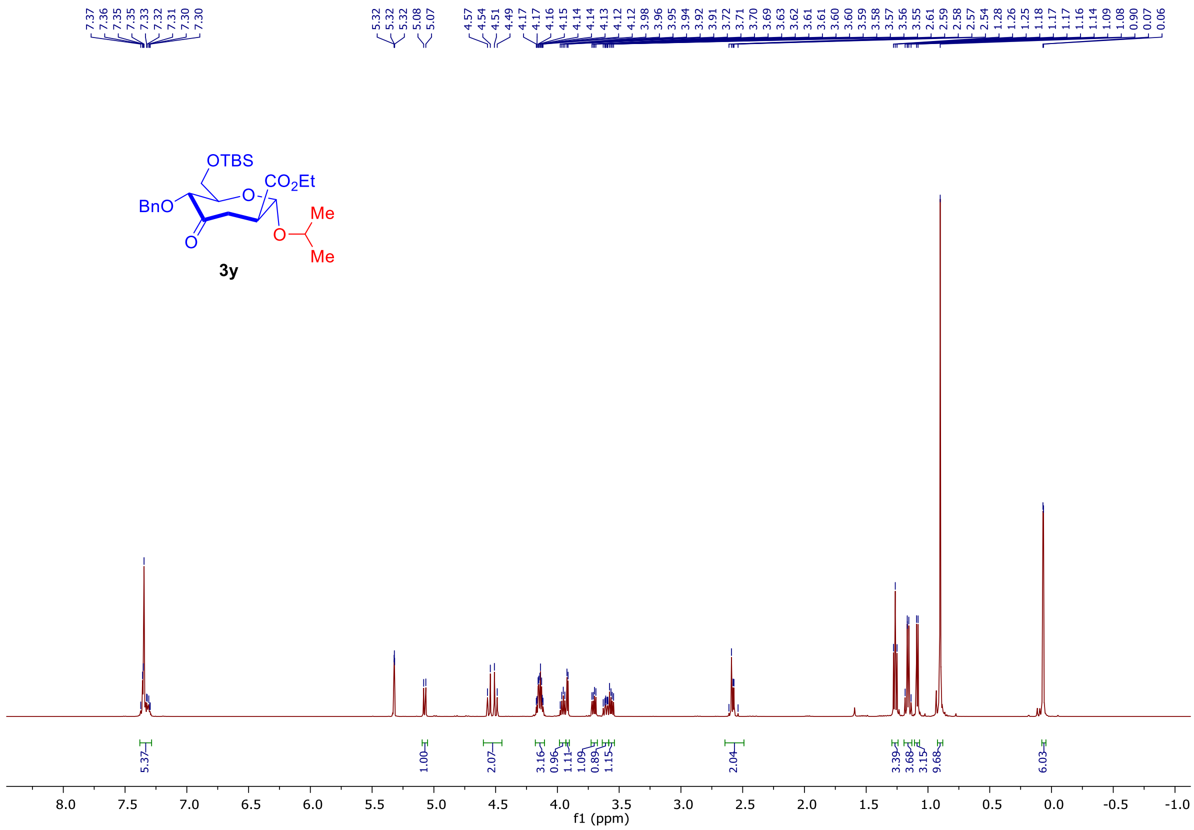

Supplementary Figure 300: ^{1}H spectra for **3y**

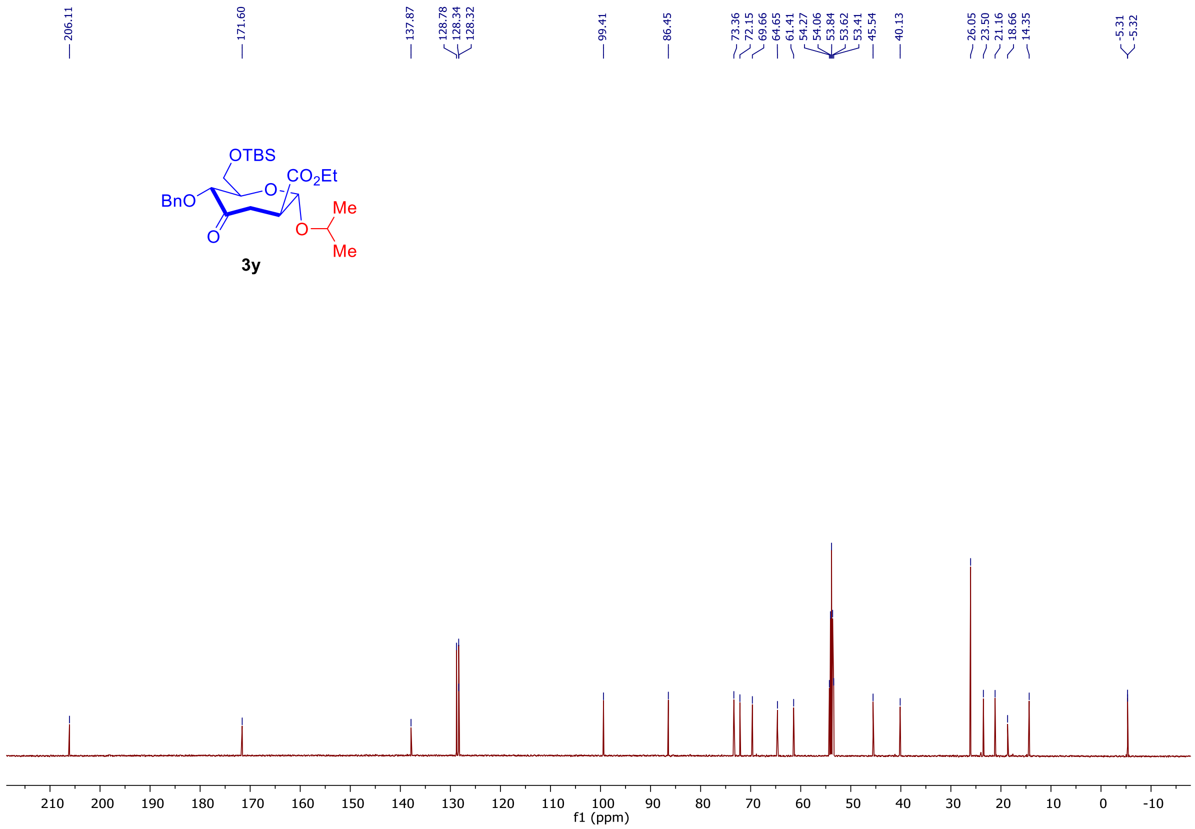

Supplementary Figure 301: ^{13}C spectra for **3y**

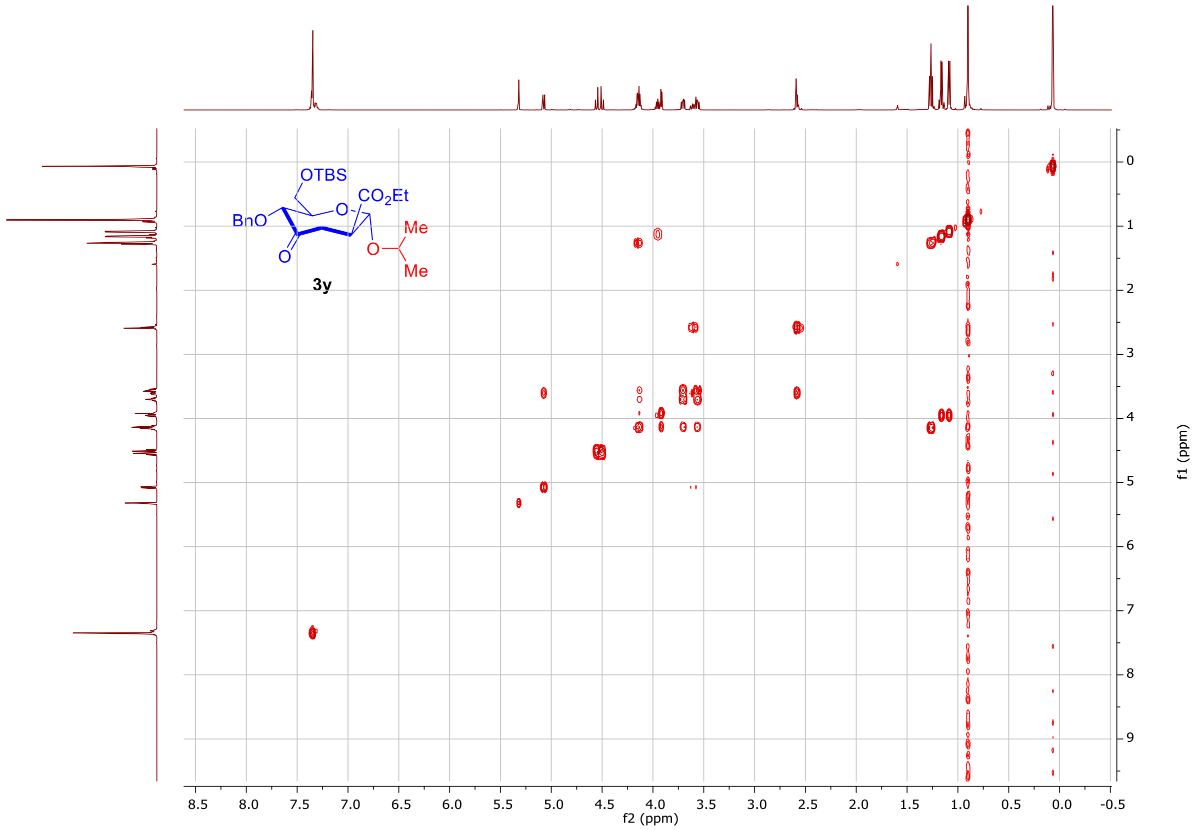

Supplementary Figure 302: COSY spectra for compound **3y**

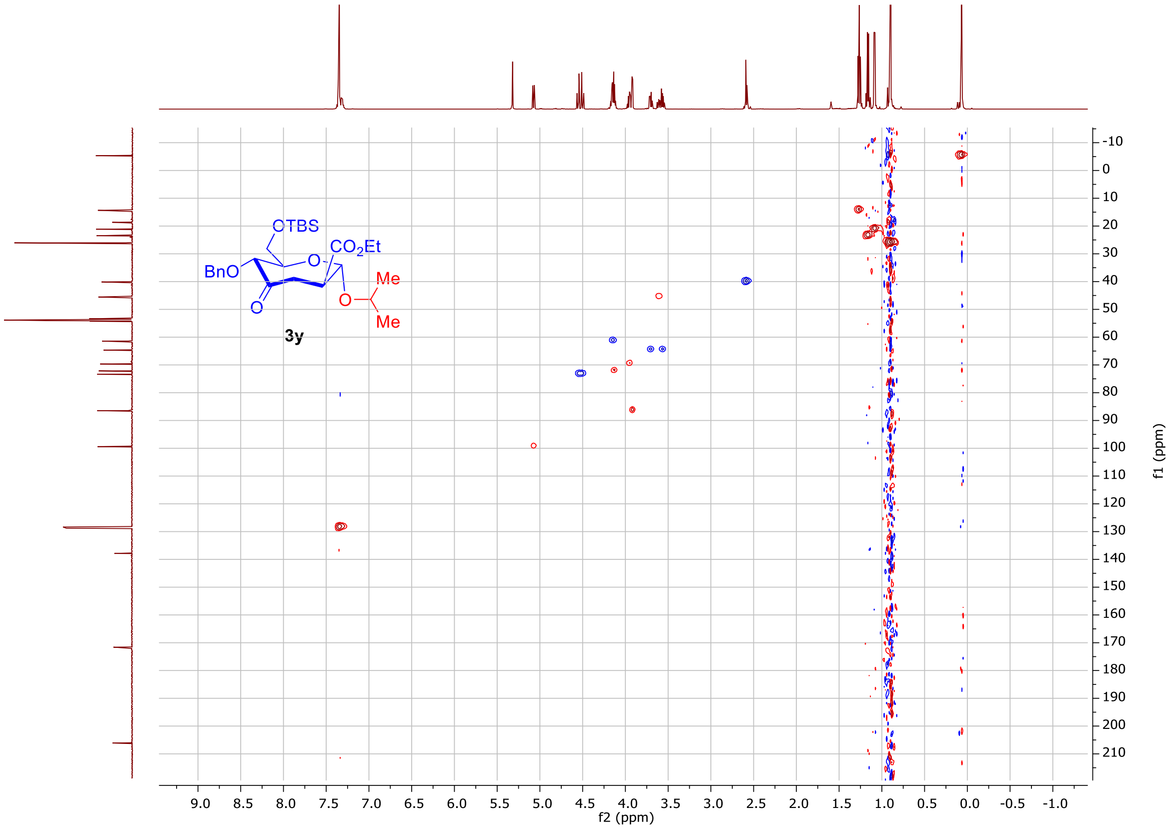

Supplementary Figure 303: HSQC spectra for compound **3y**

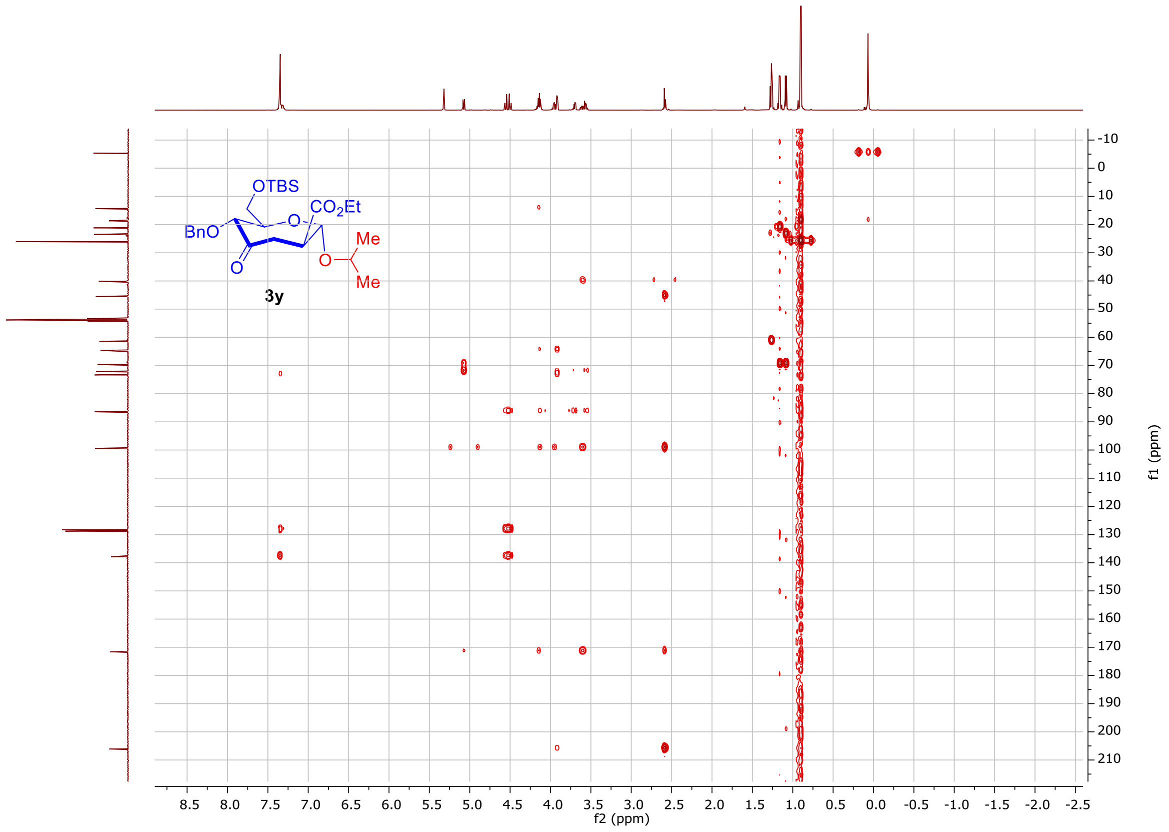

Supplementary Figure 304: HMBC spectra for compound **3y**

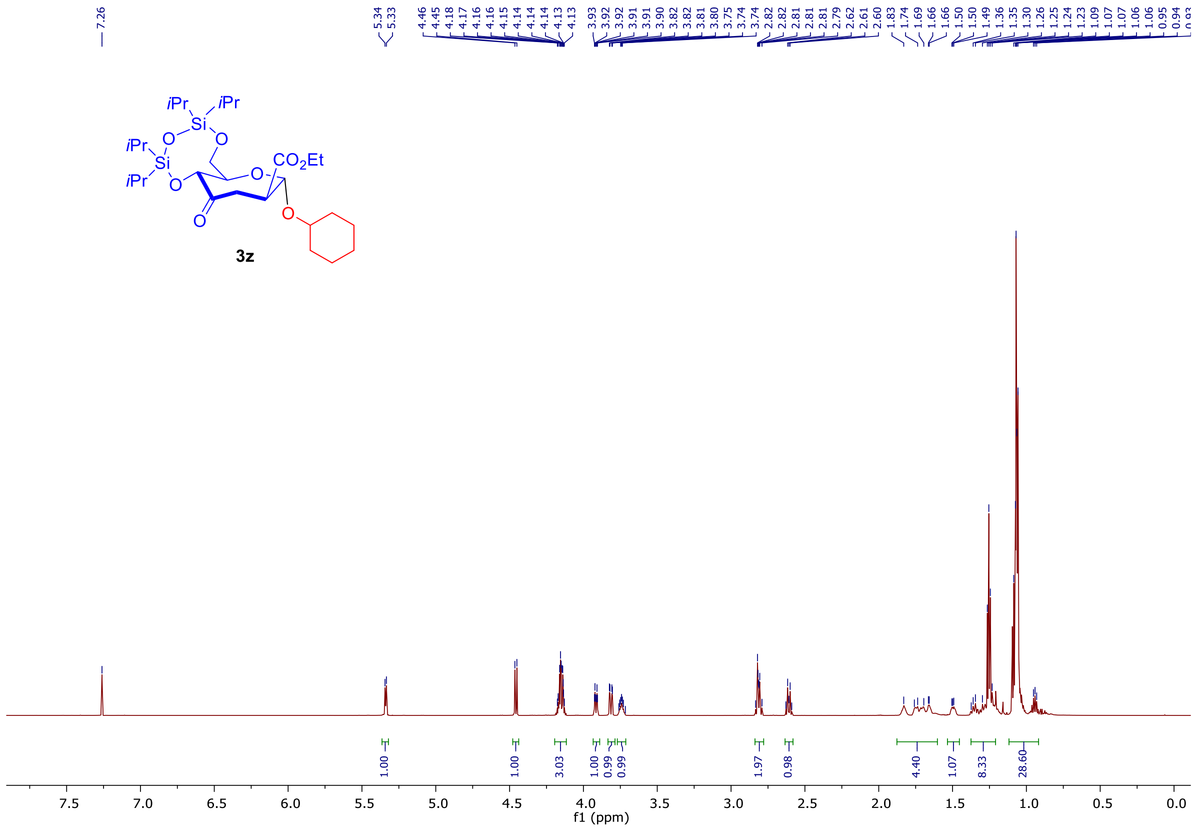

Supplementary Figure 305: ^{1}H spectra for **3z**

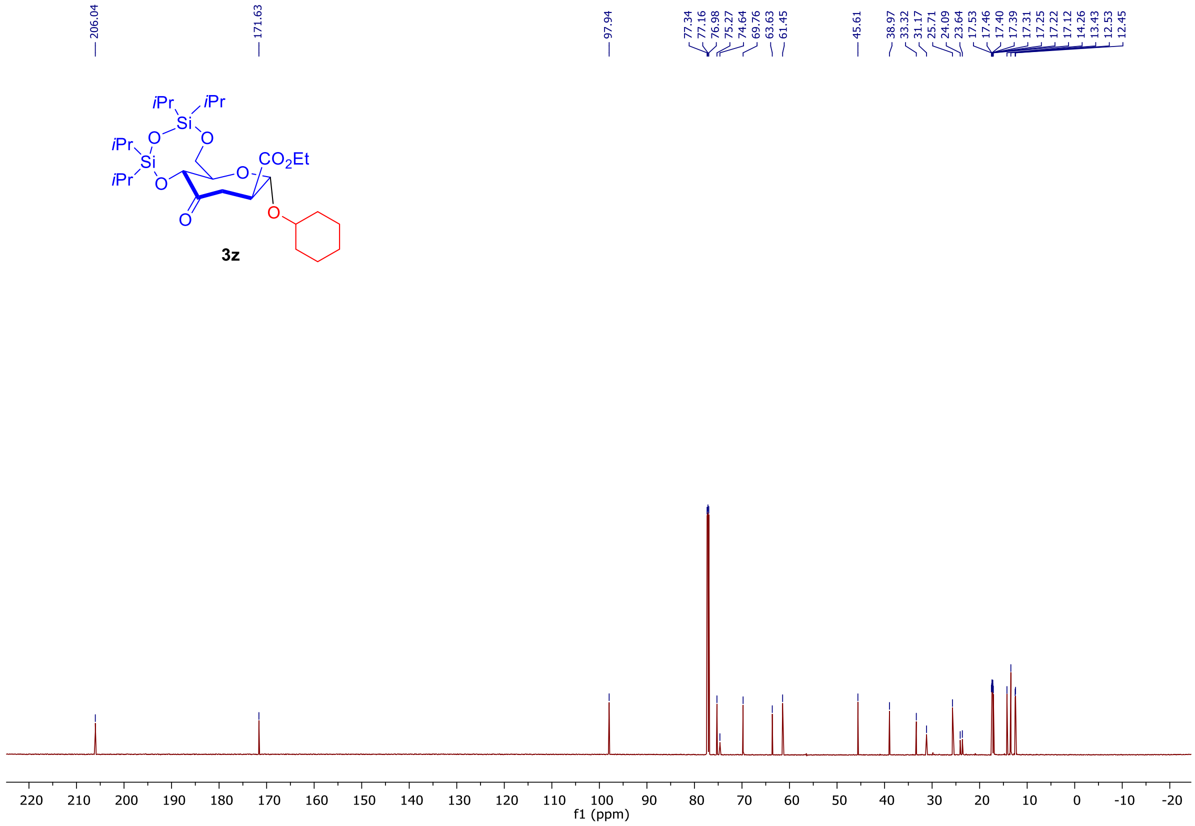

Supplementary Figure 306: ^{13}C spectra for **3z**

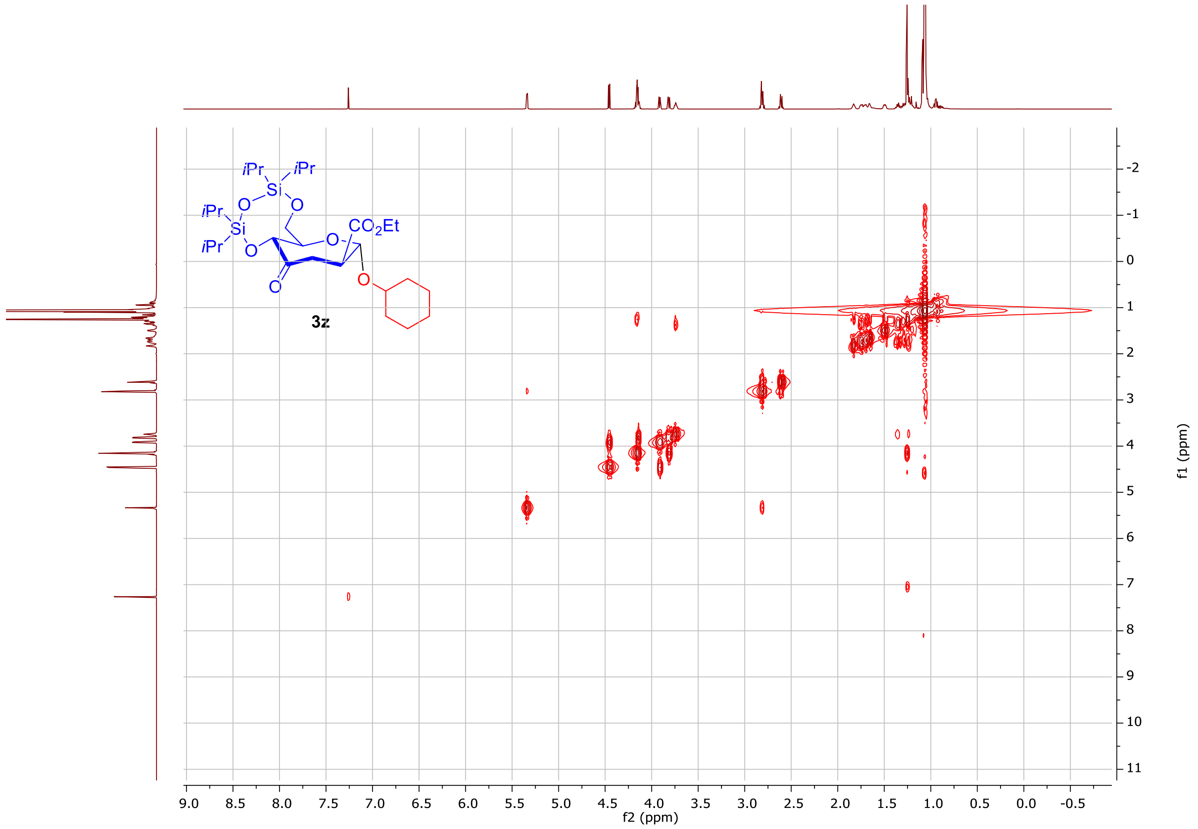

Supplementary Figure 307: COSY spectra for compound **3z**

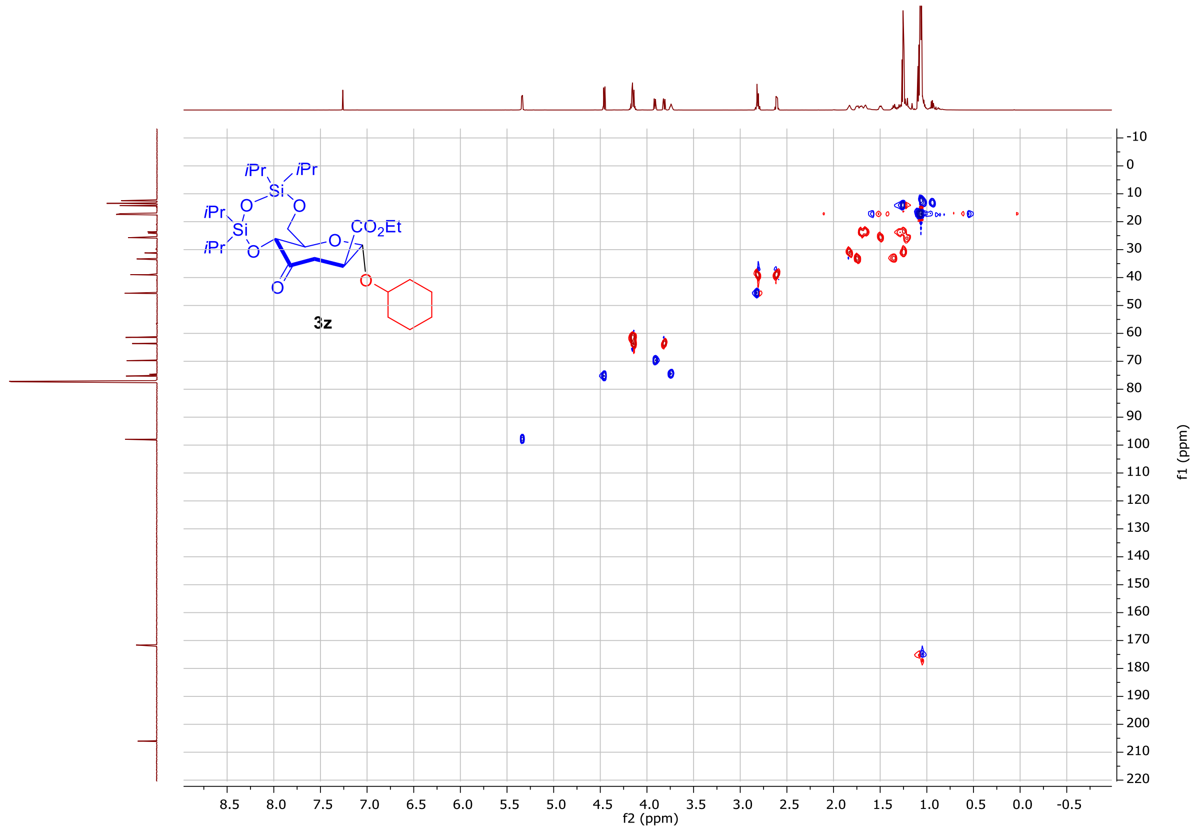

Supplementary Figure 308: HSQC spectra for compound **3z**

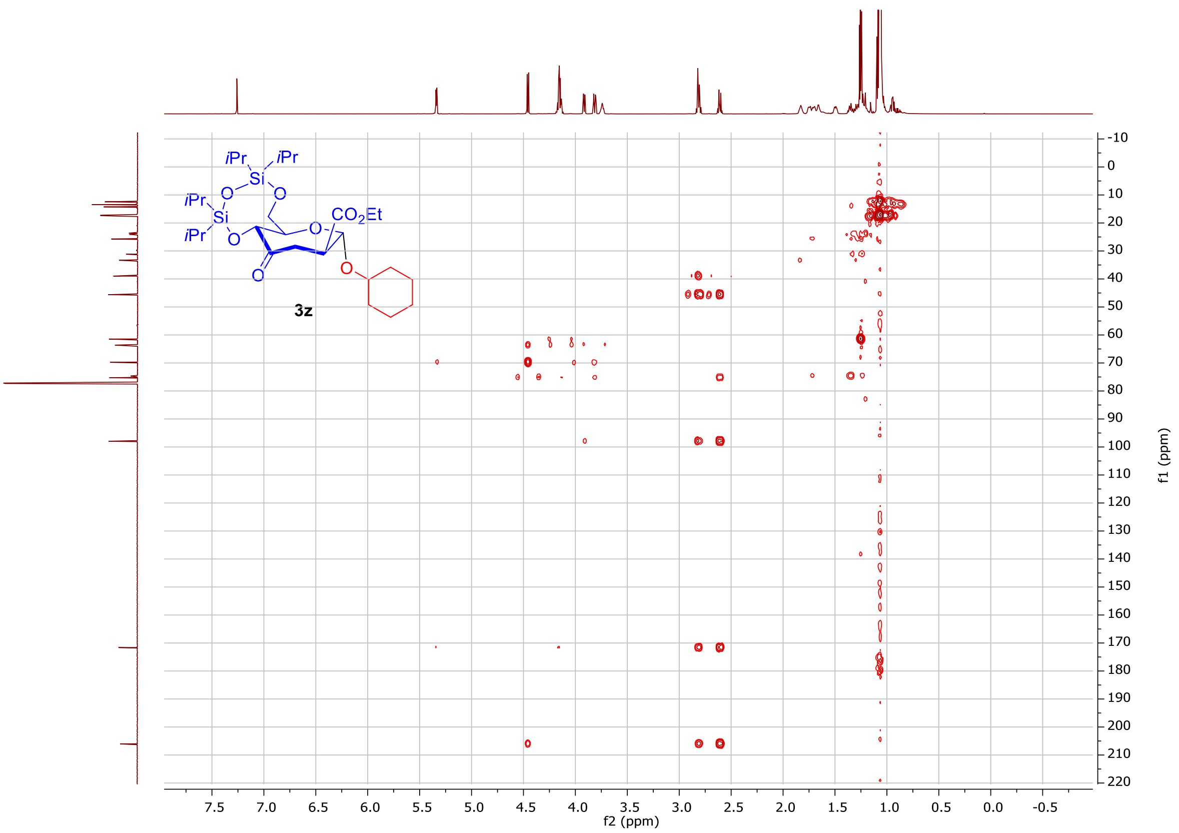

Supplementary Figure 309: HMBC spectra for compound **3z**

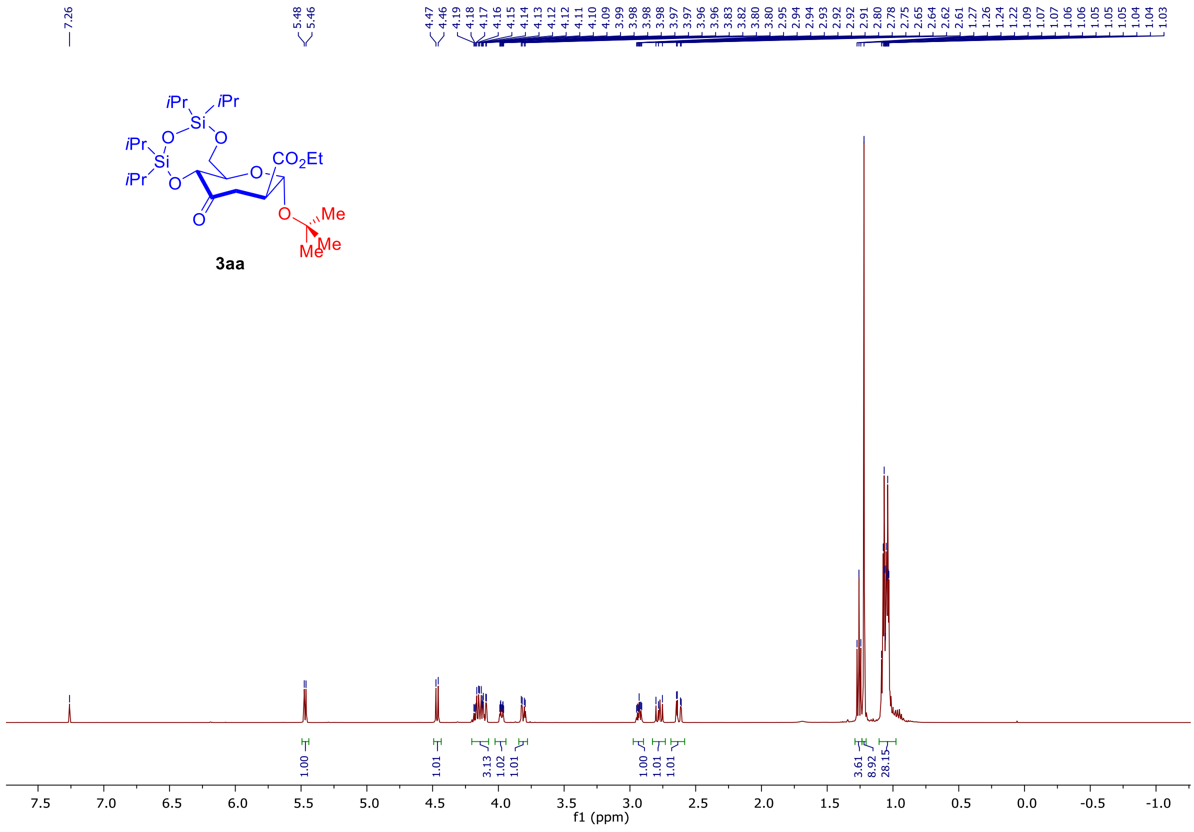

Supplementary Figure 310: ^{1}H spectra for **3aa**

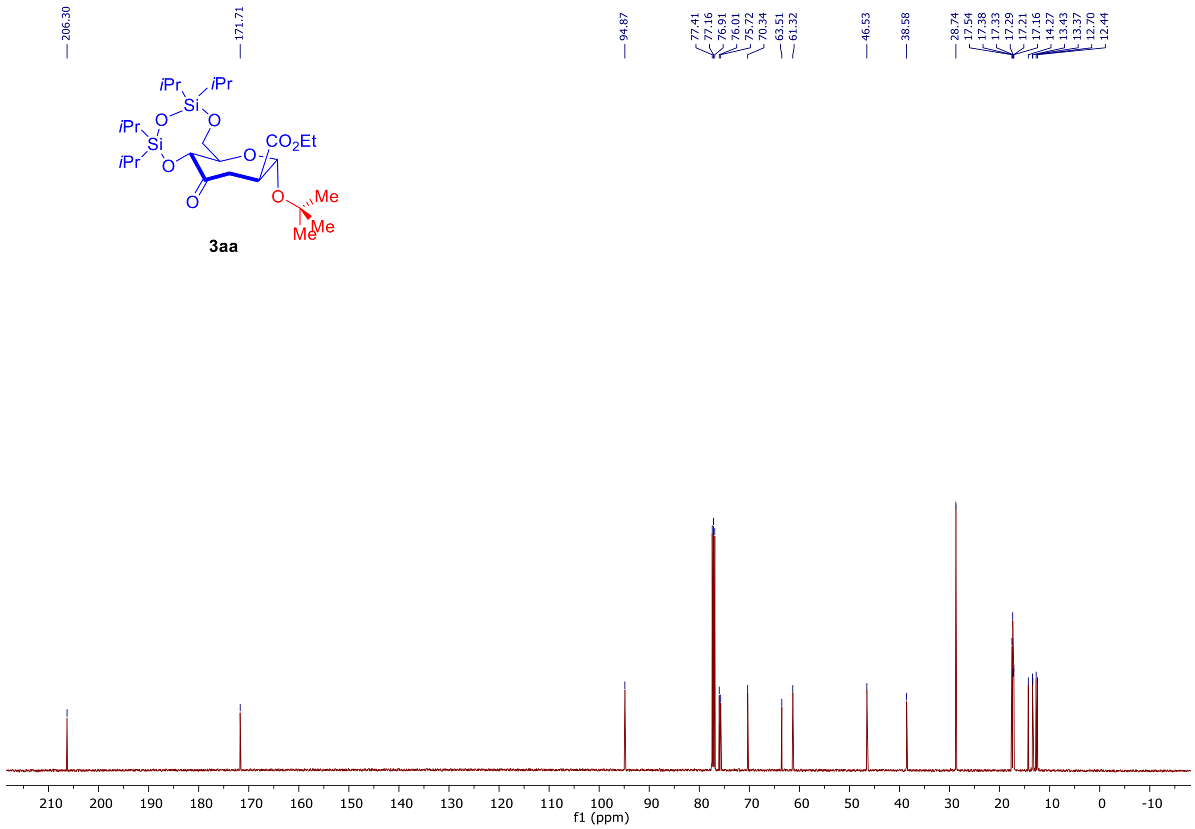

Supplementary Figure 311: ^{13}C spectra for **3aa**

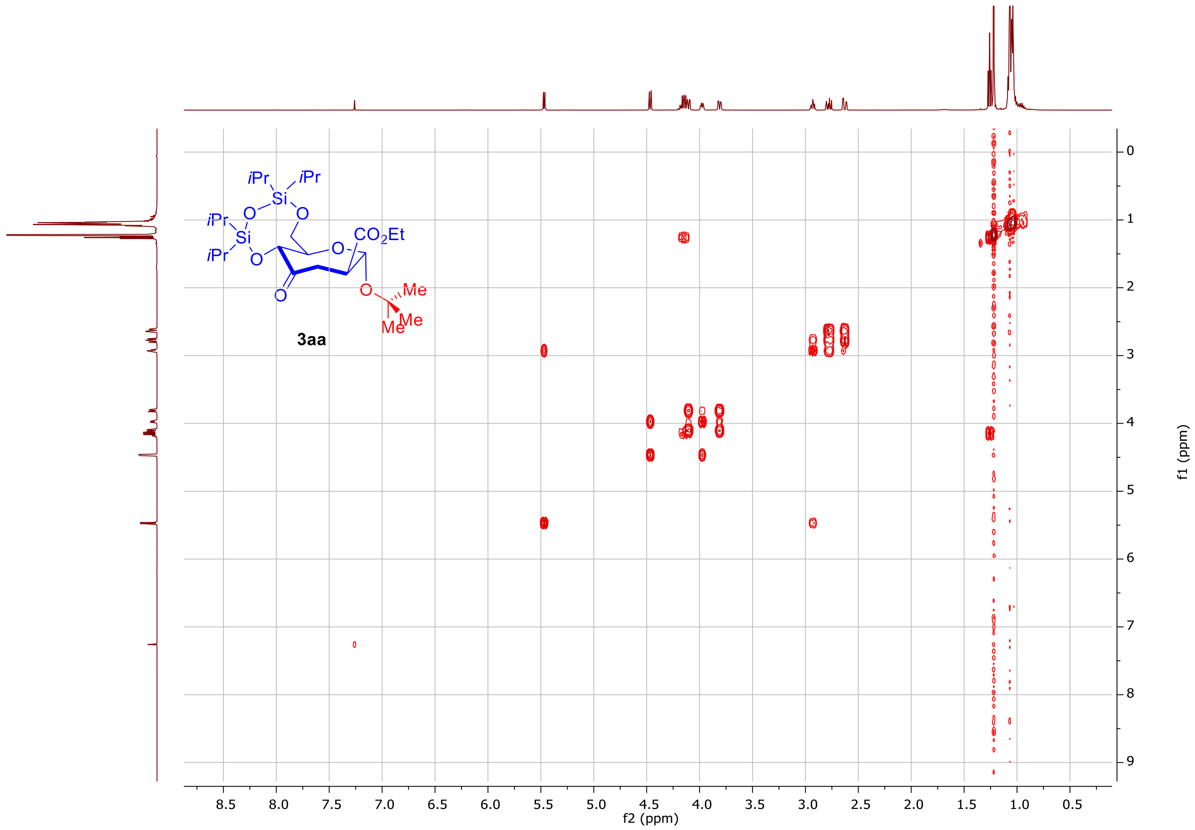

Supplementary Figure 312: COSY spectra for compound **3aa**

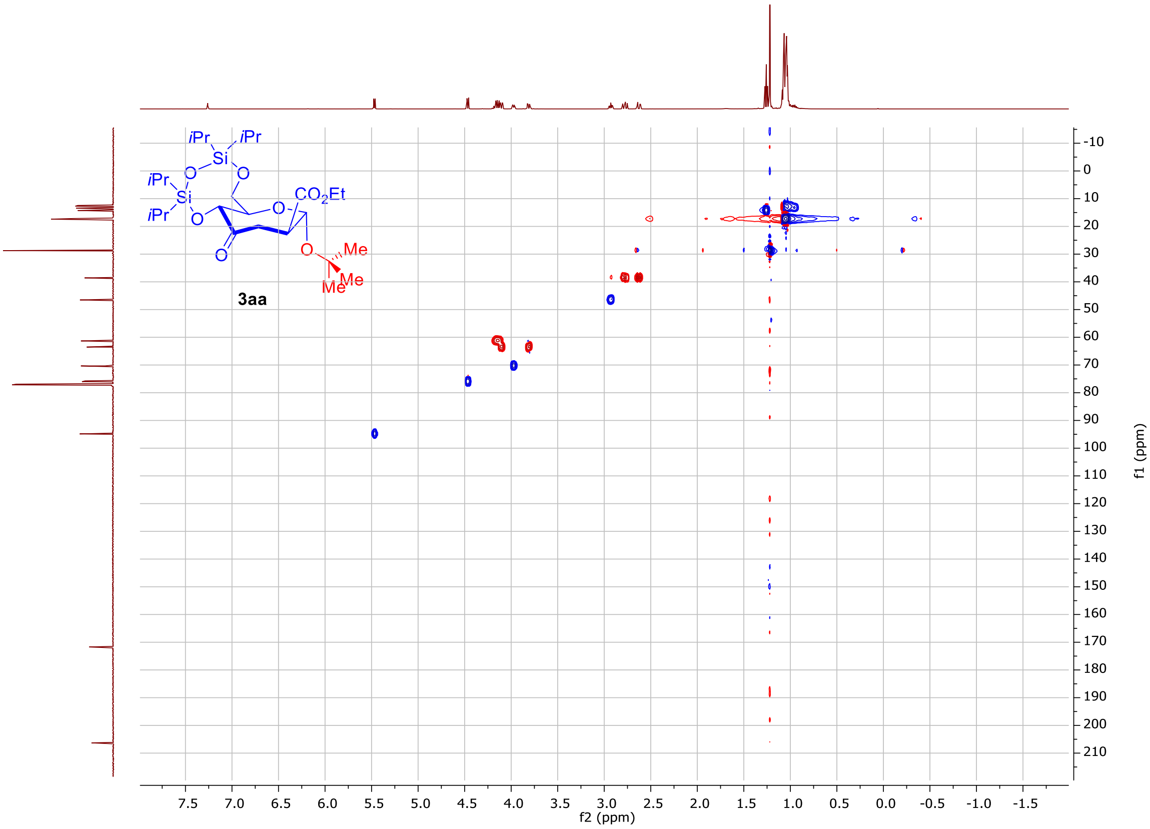

Supplementary Figure 313: HSQC spectra for compound **3aa**

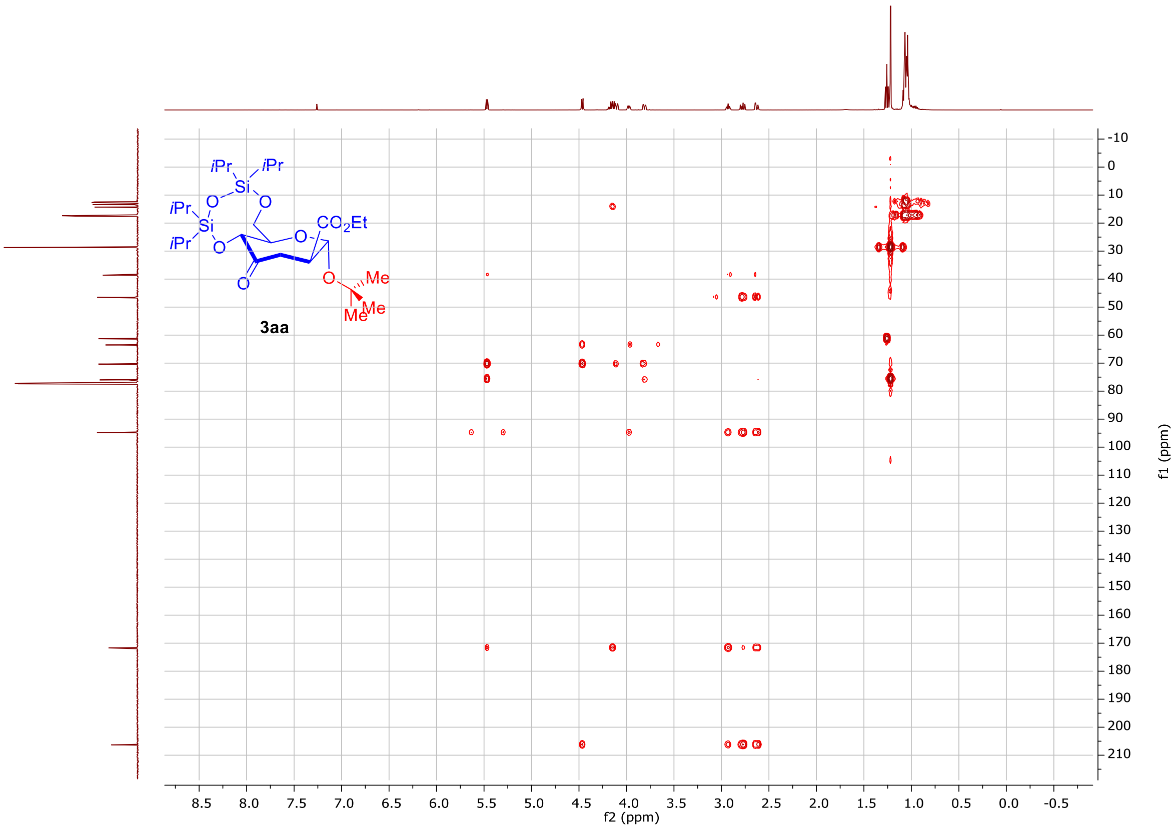

Supplementary Figure 314: HMBC spectra for compound **3aa**

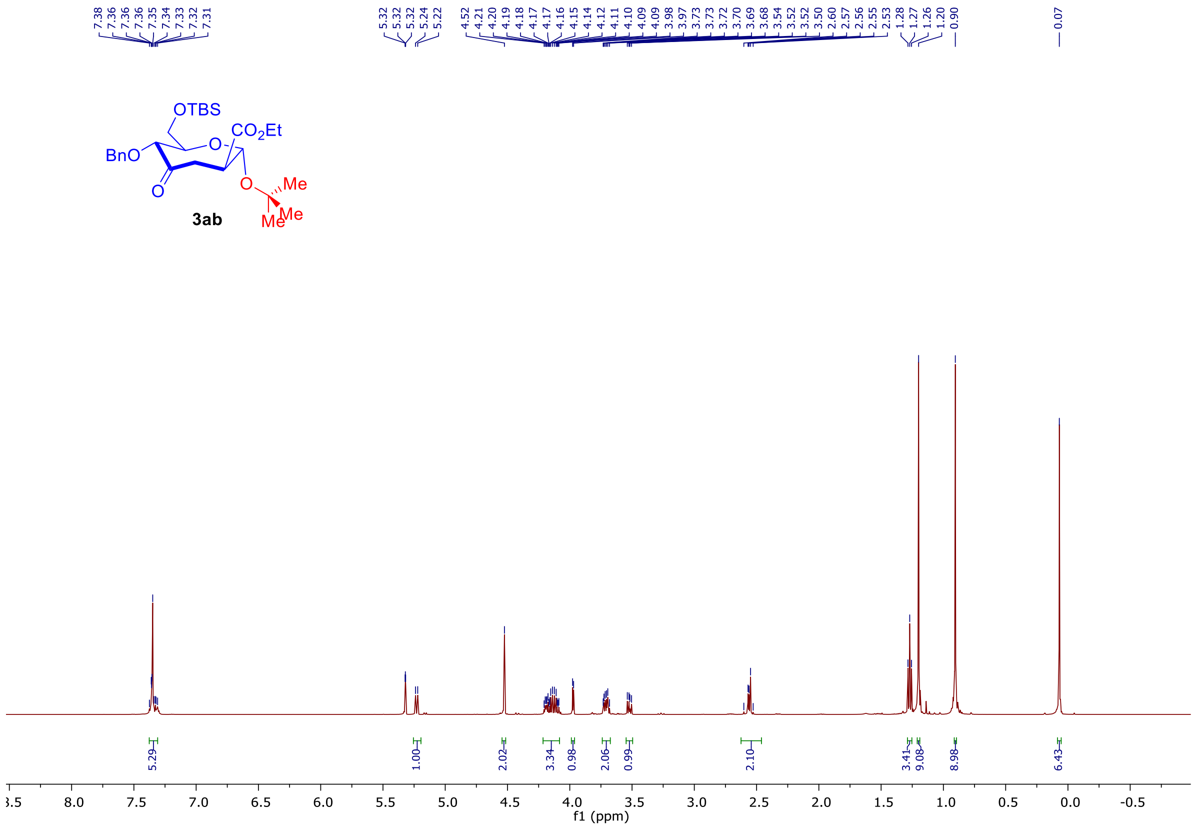

Supplementary Figure 315: ^{1}H spectra for **3ab**

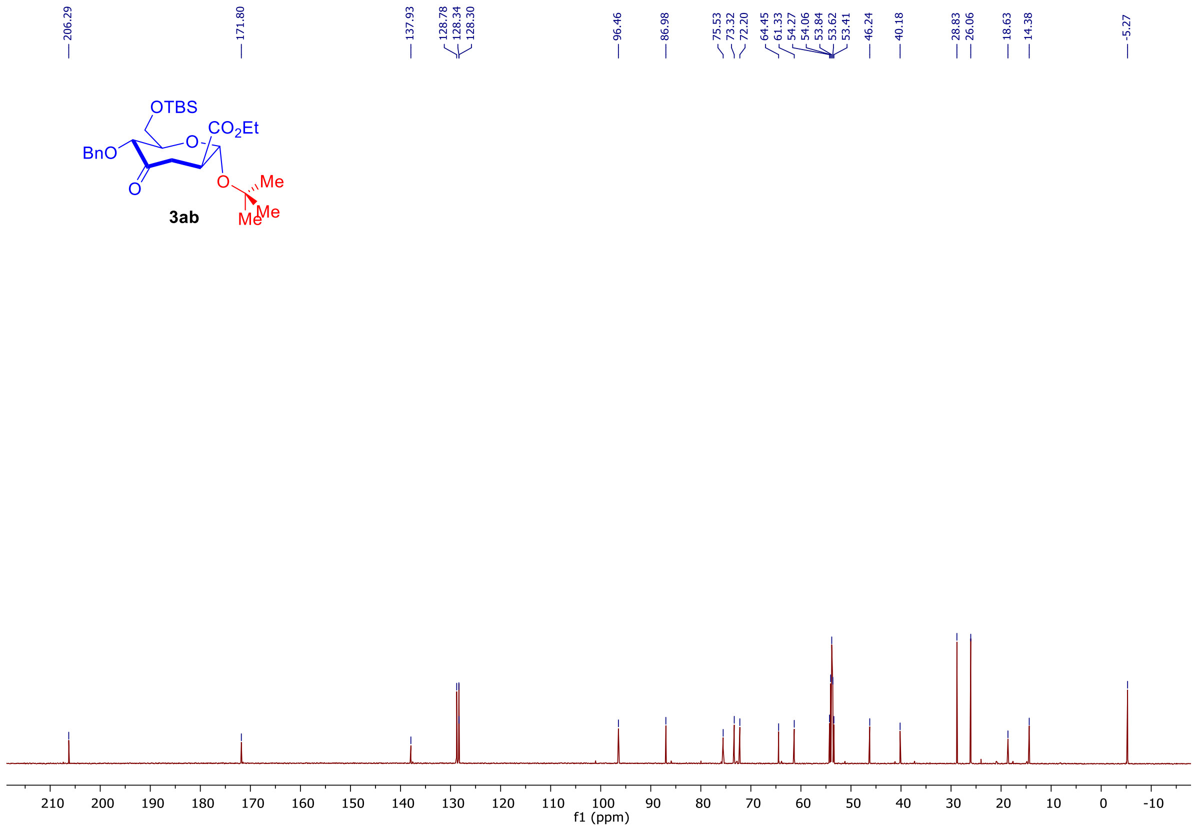

Supplementary Figure 316: ^{13}C spectra for **3ab**

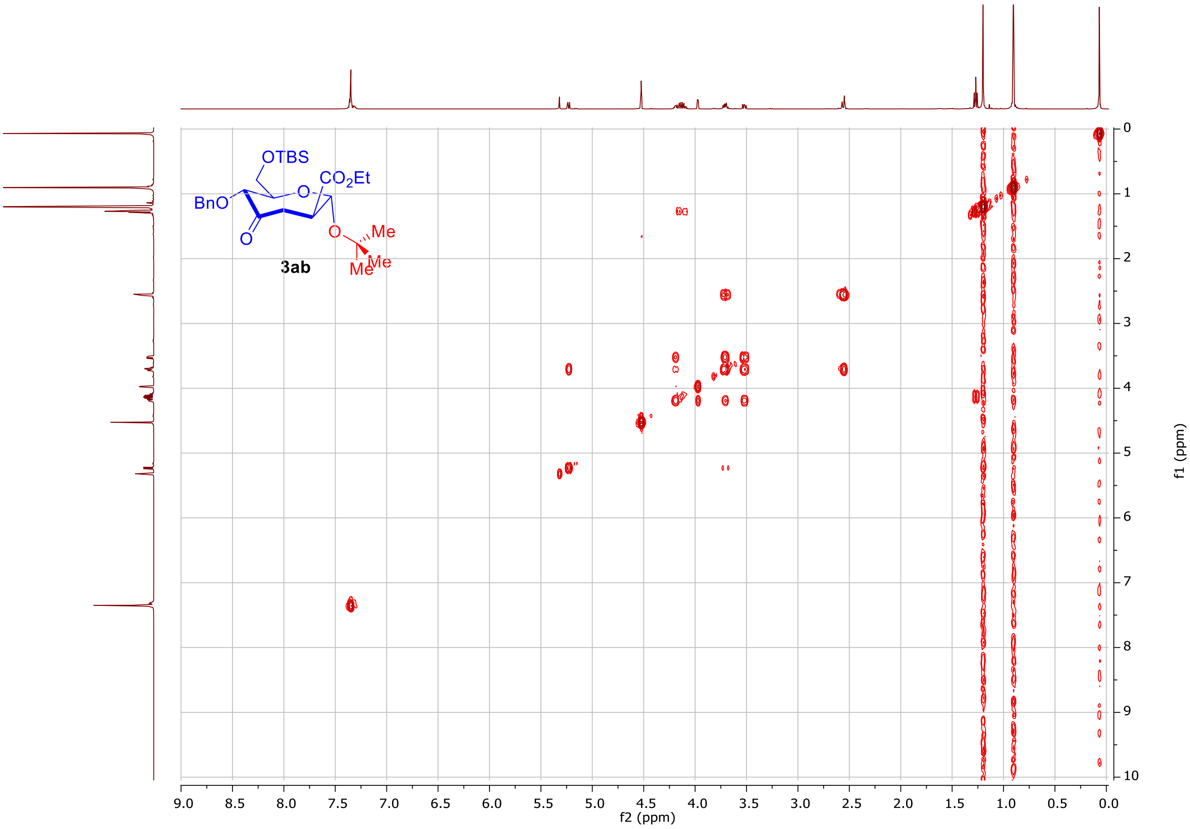

Supplementary Figure 317: COSY spectra for compound **3ab**

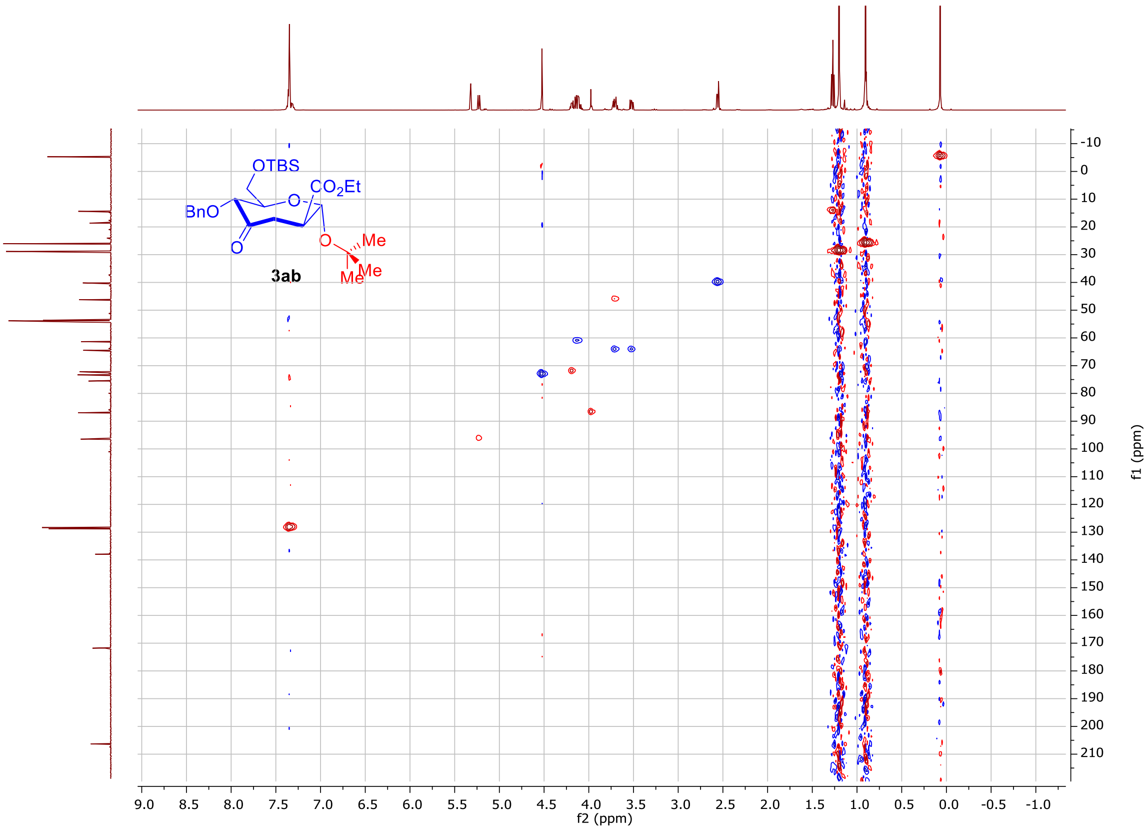

Supplementary Figure 318: HSQC spectra for compound **3ab**

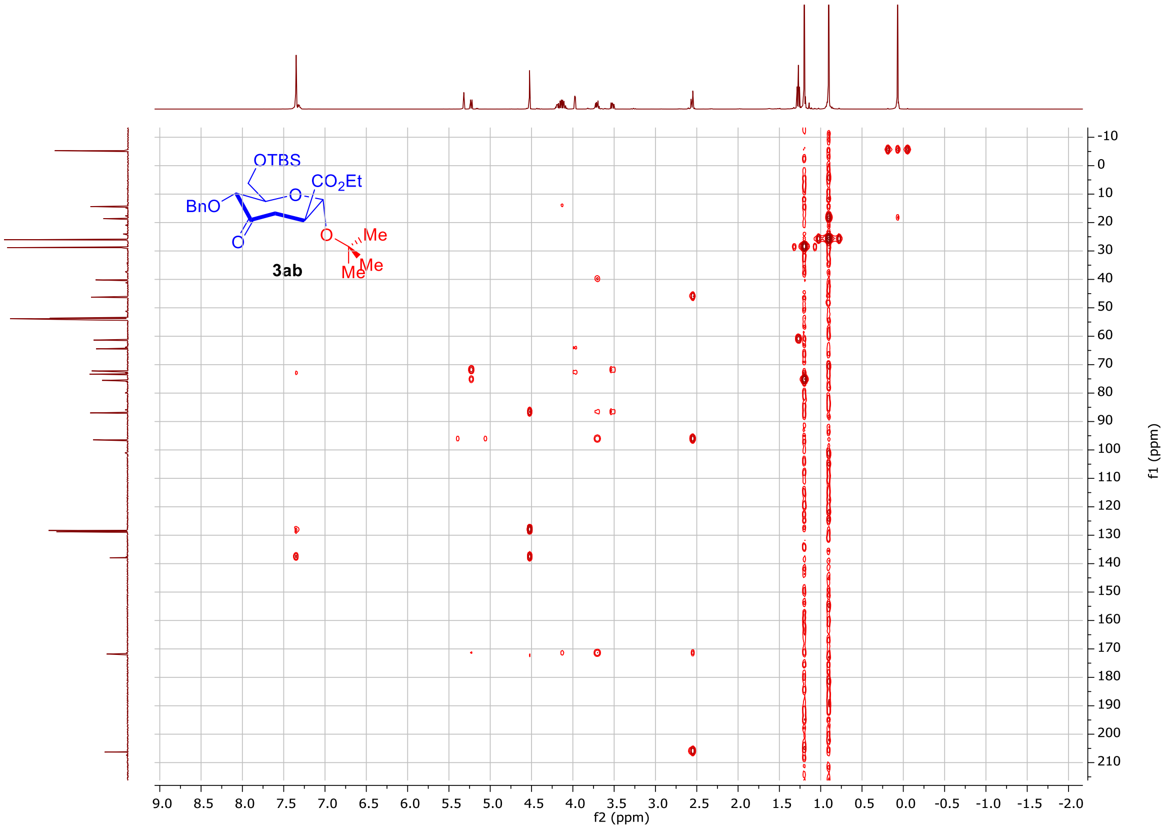

Supplementary Figure 319: HMBC spectra for compound **3ab**

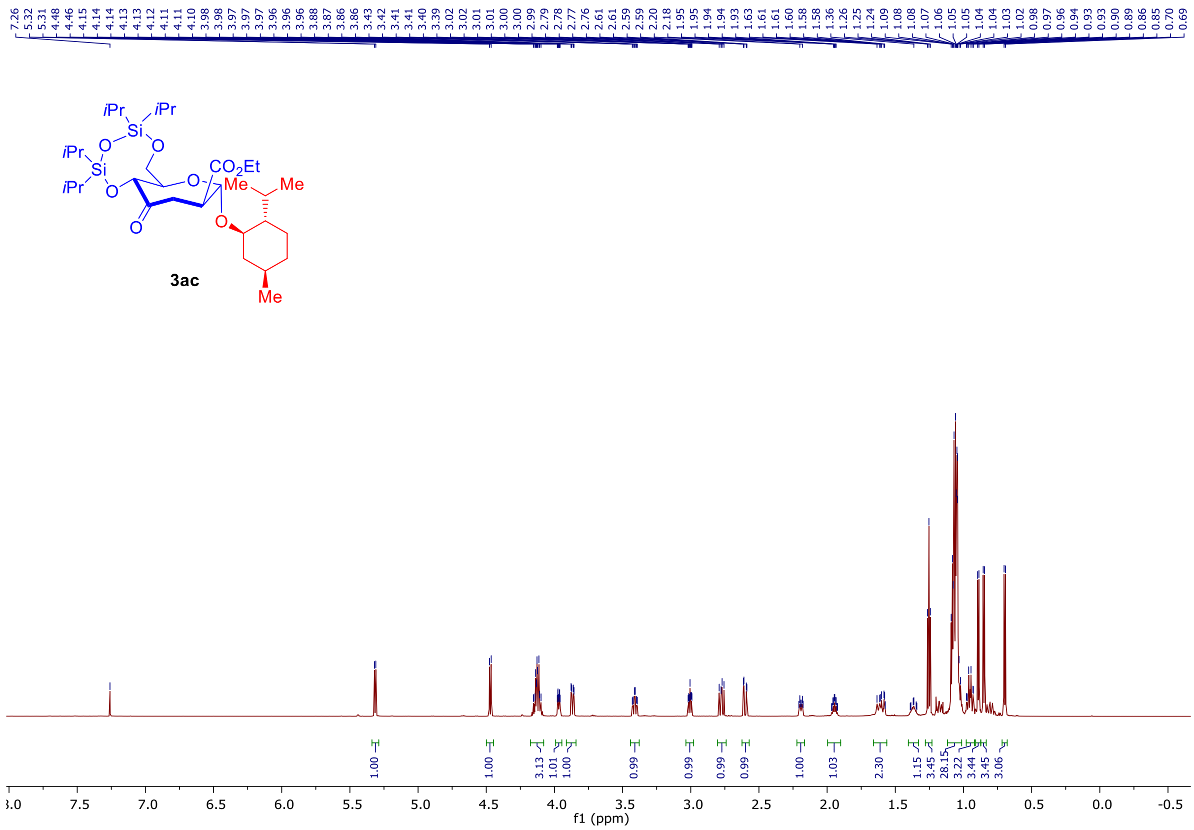

Supplementary Figure 320: ^{1}H spectra for **3ac**

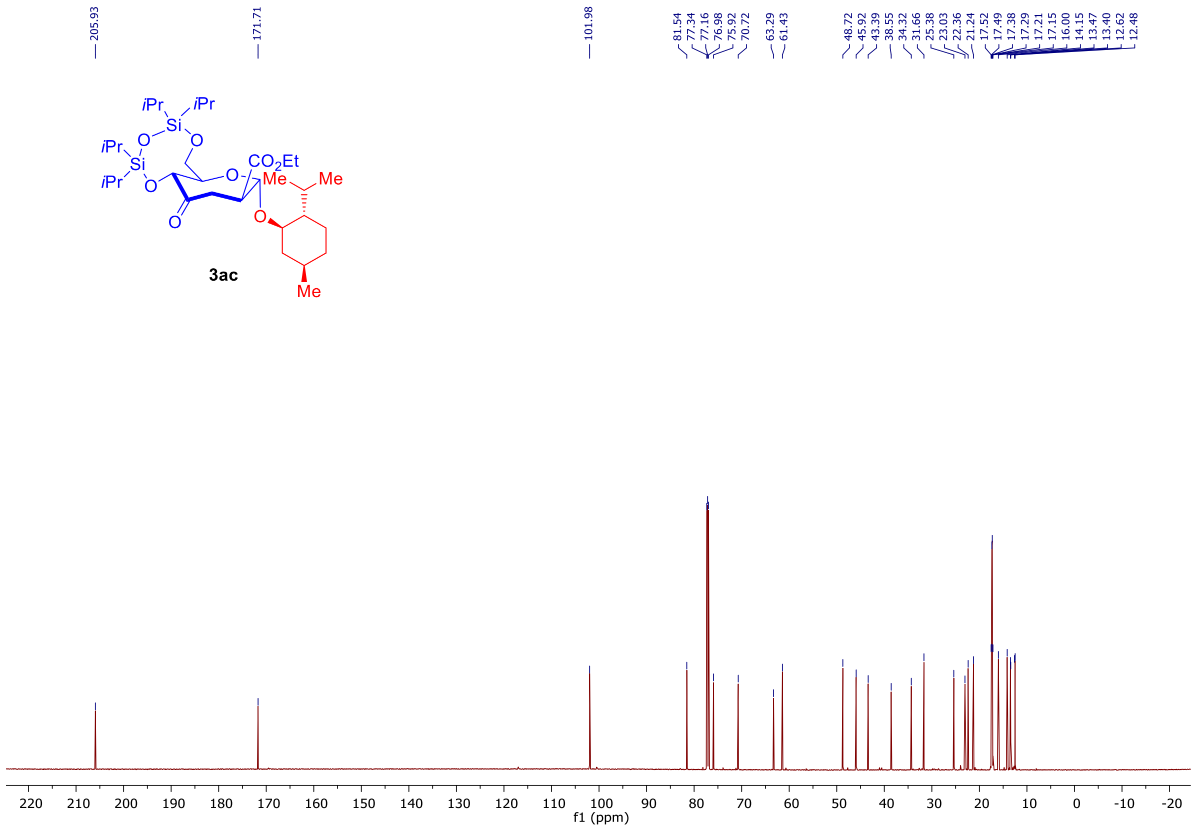

Supplementary Figure 321: ^{13}C spectra for **3ac**

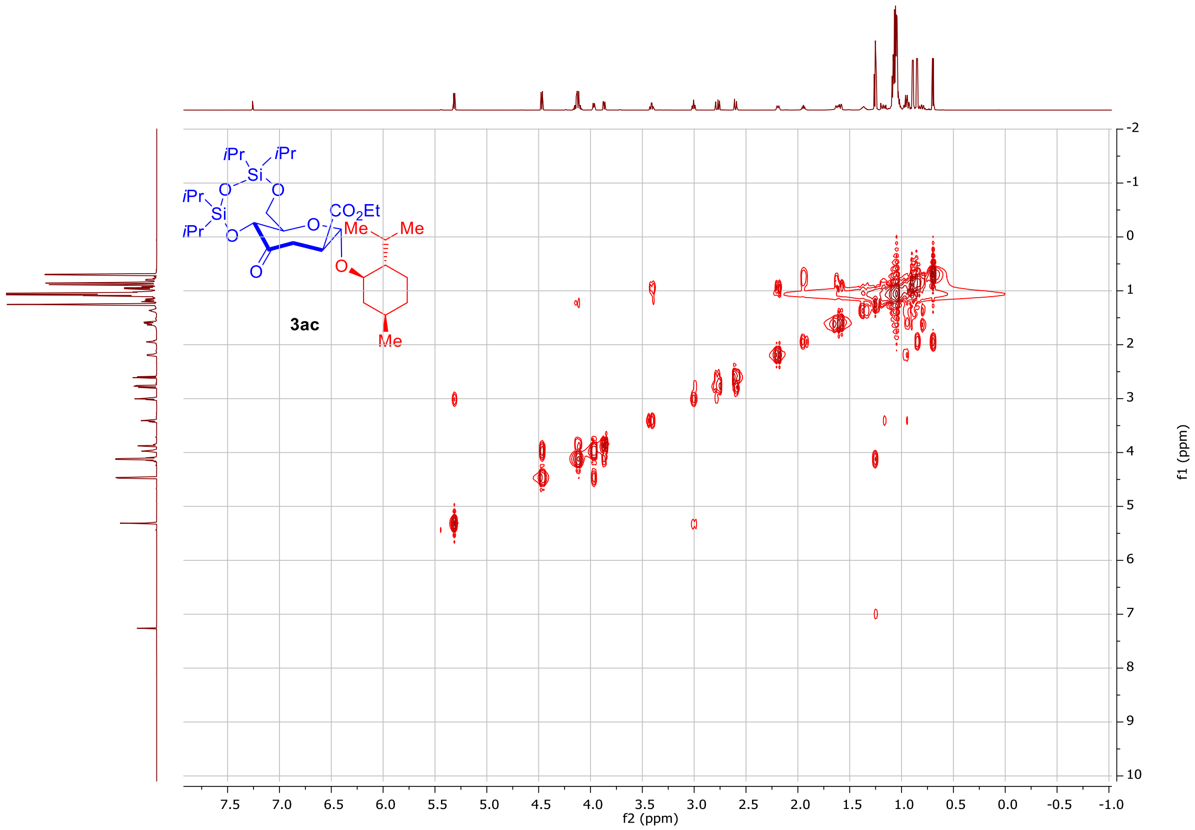

Supplementary Figure 322: COSY spectra for compound **3ac**

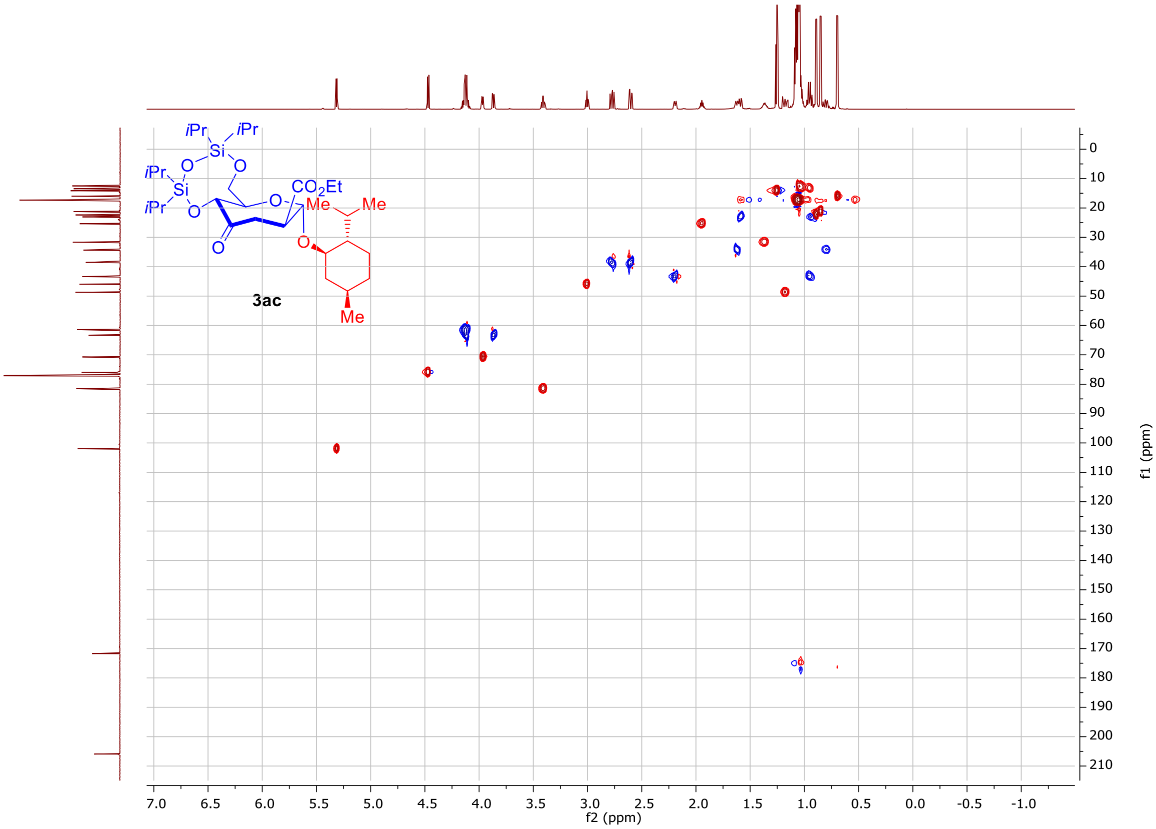

Supplementary Figure 323: HSQC spectra for compound **3ac**

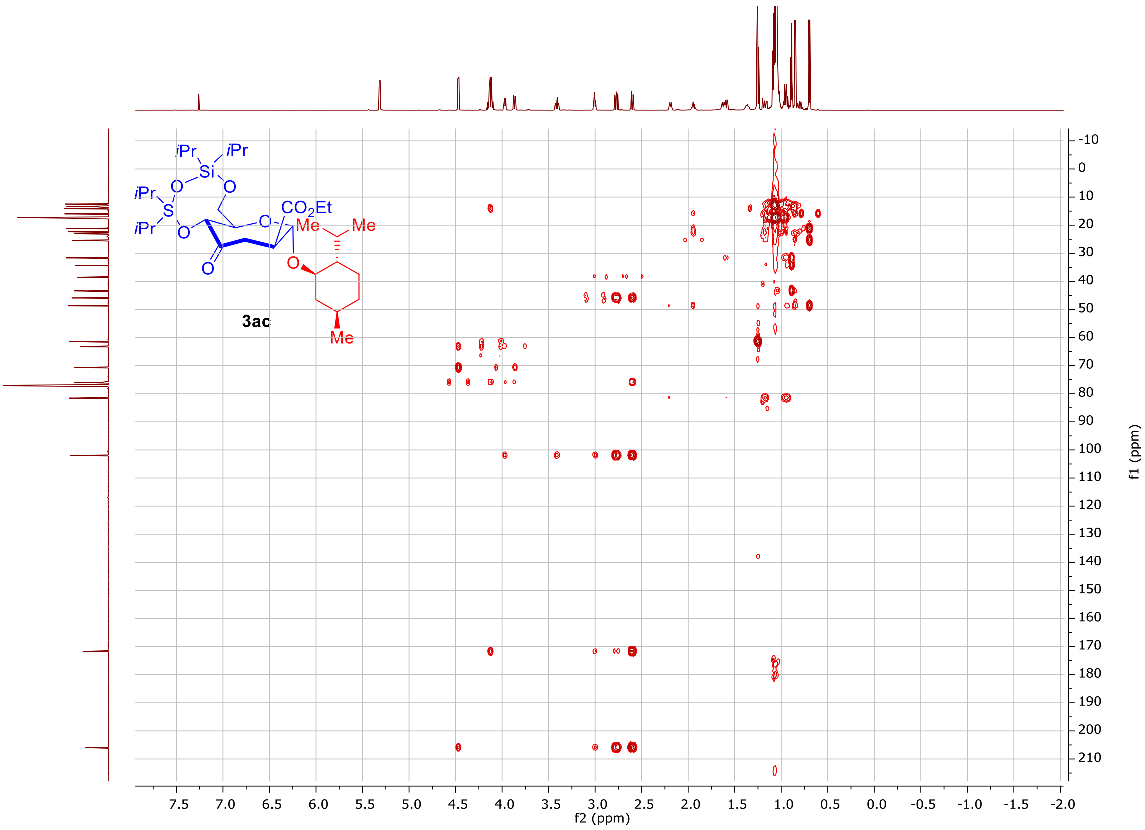

Supplementary Figure 324: HMBC spectra for compound **3ac**

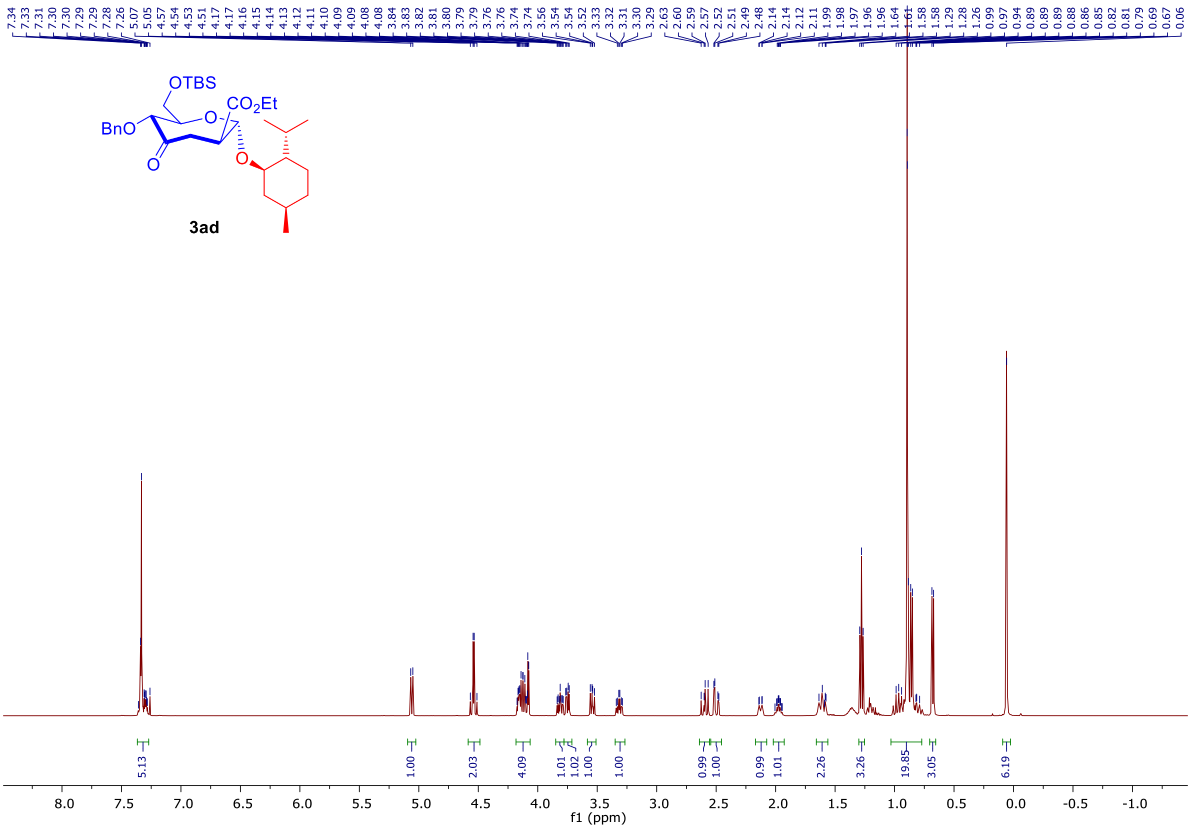

Supplementary Figure 325: ^{1}H spectra for **3ad**

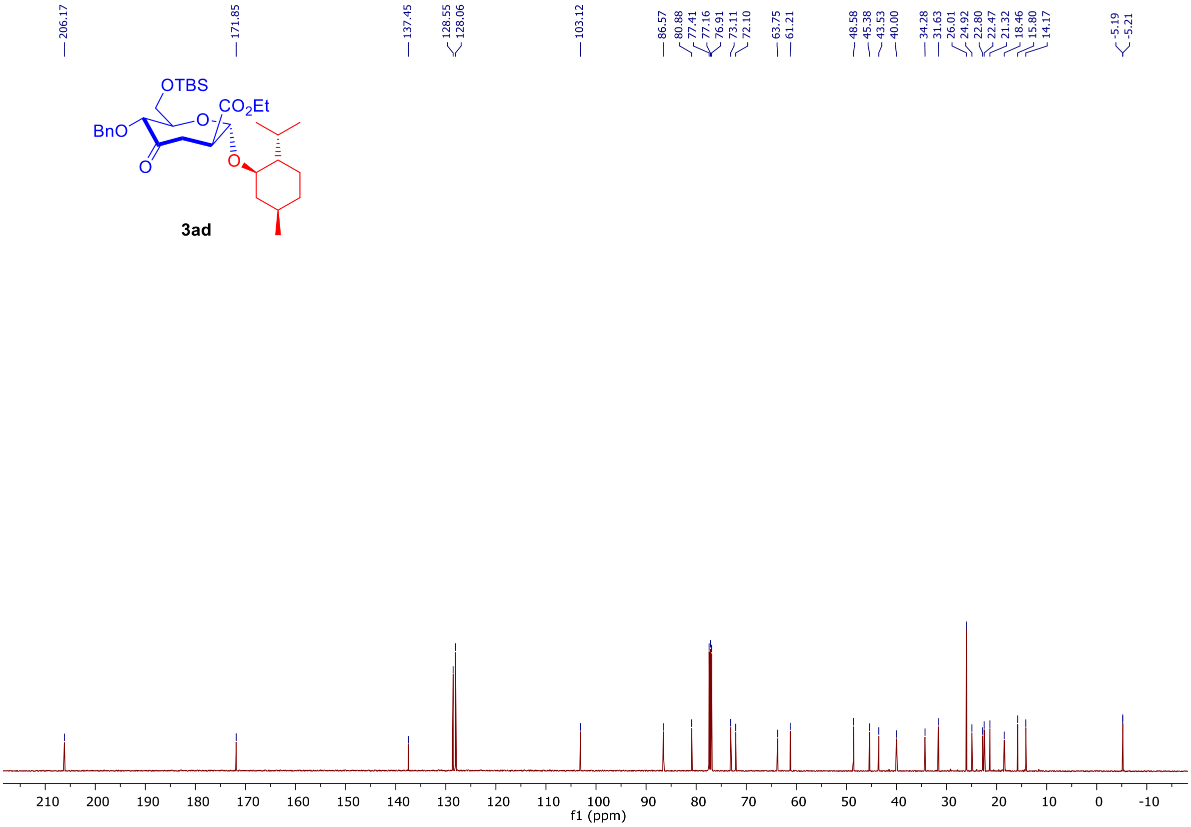

Supplementary Figure 326: ^{13}C spectra for **3ad**

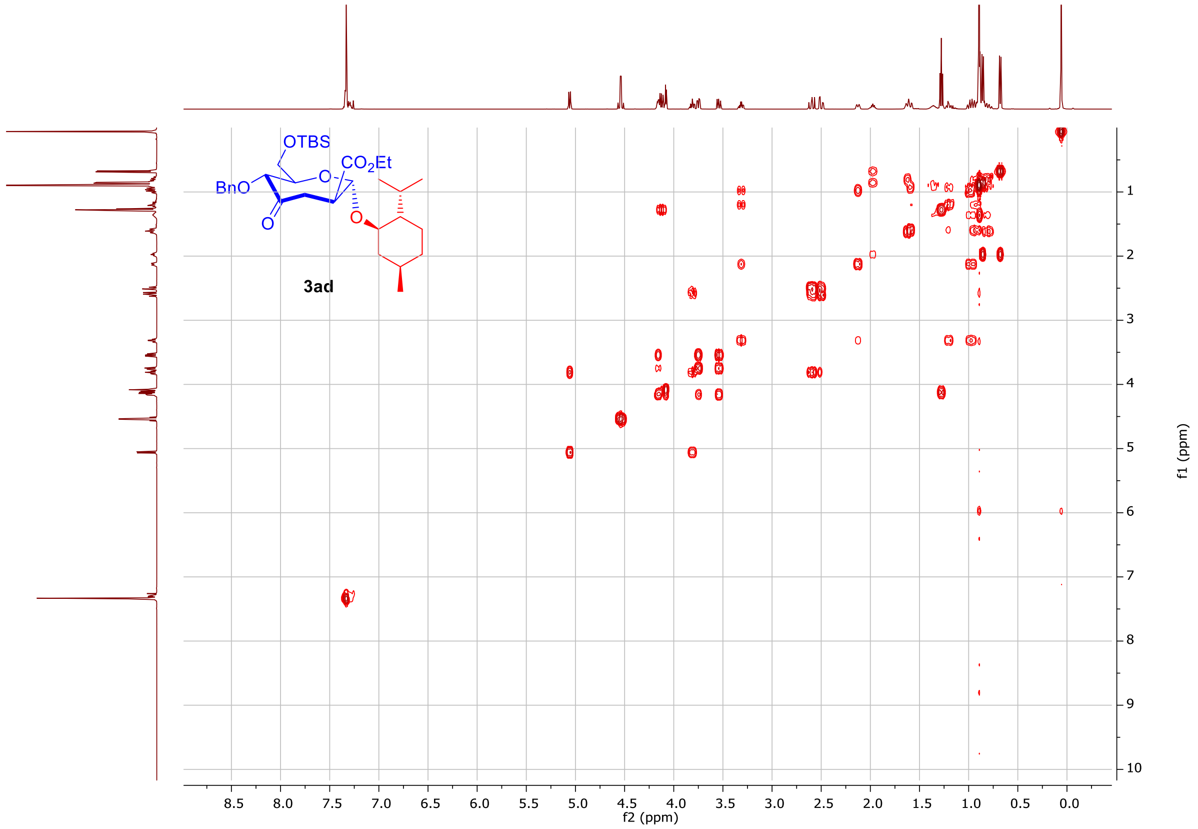

Supplementary Figure 327: COSY spectra for compound **3ad**

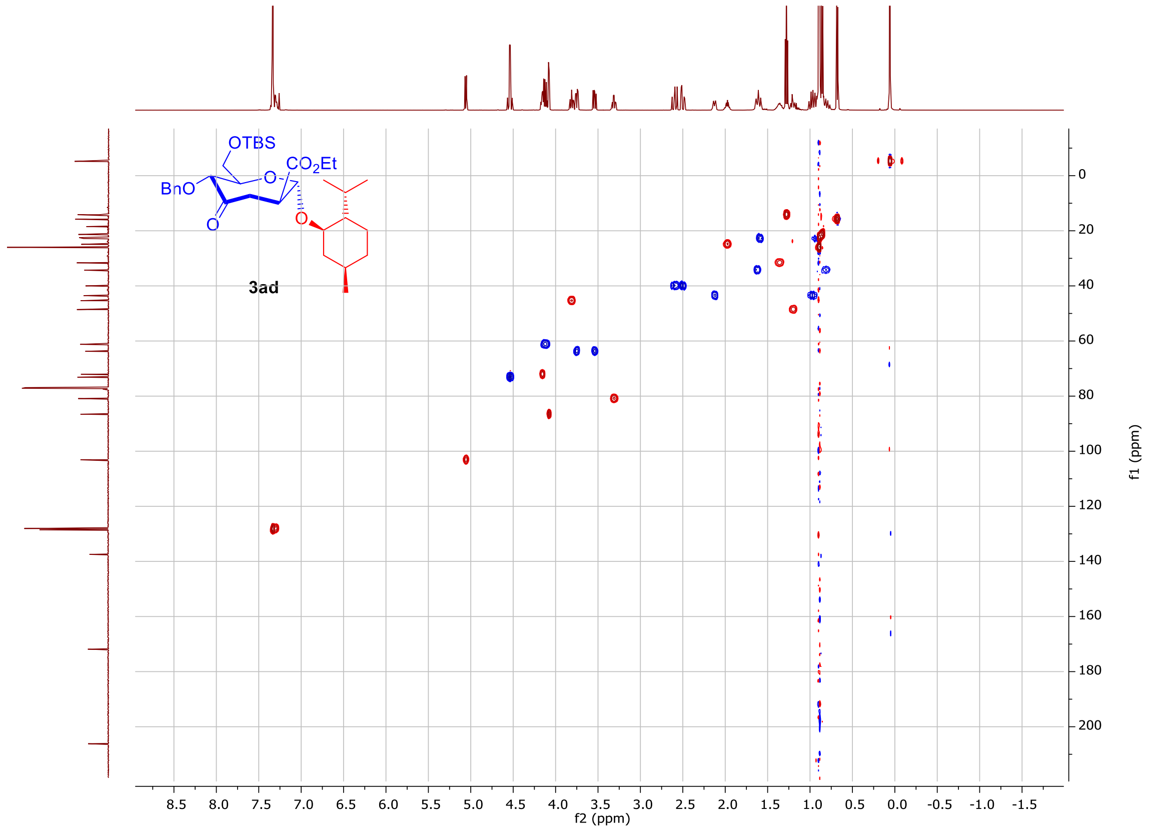

Supplementary Figure 328: HSQC spectra for compound **3ad**

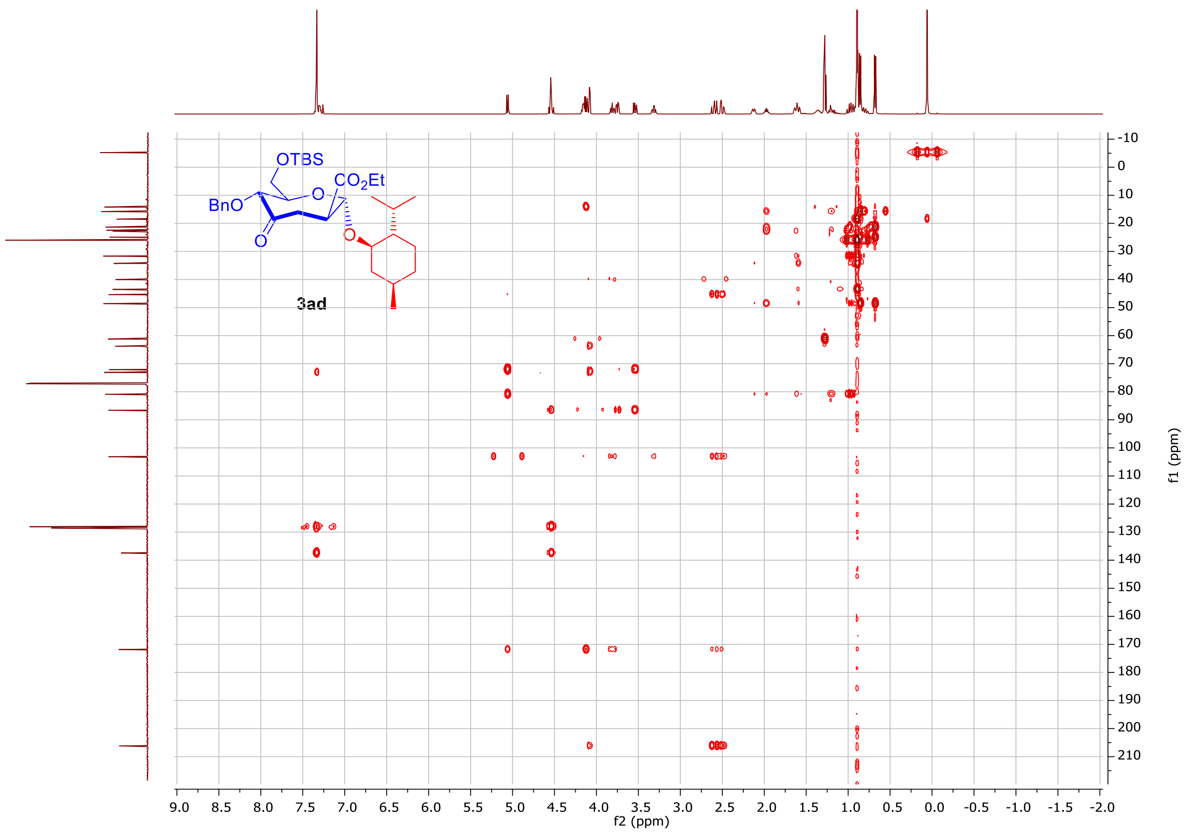

Supplementary Figure 329: HMBC spectra for compound **3ad**

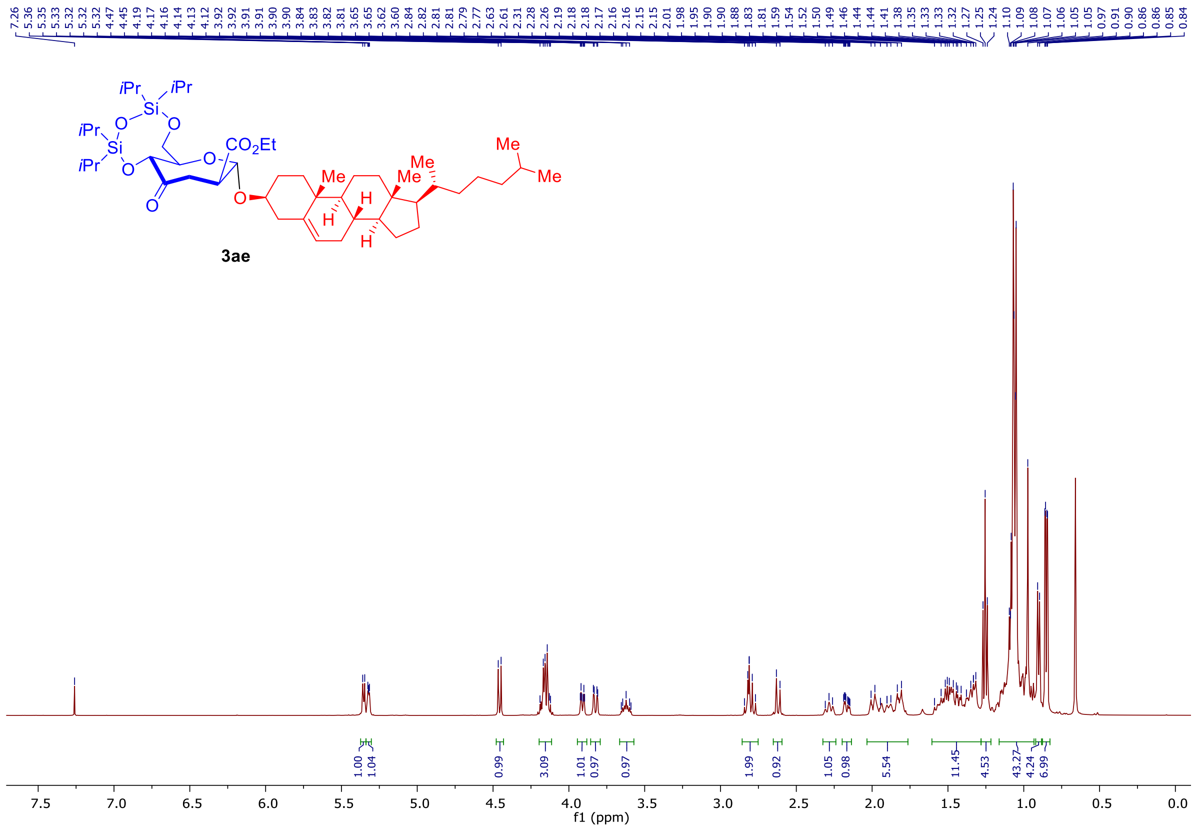

Supplementary Figure 330: ^{1}H spectra for **3ae**

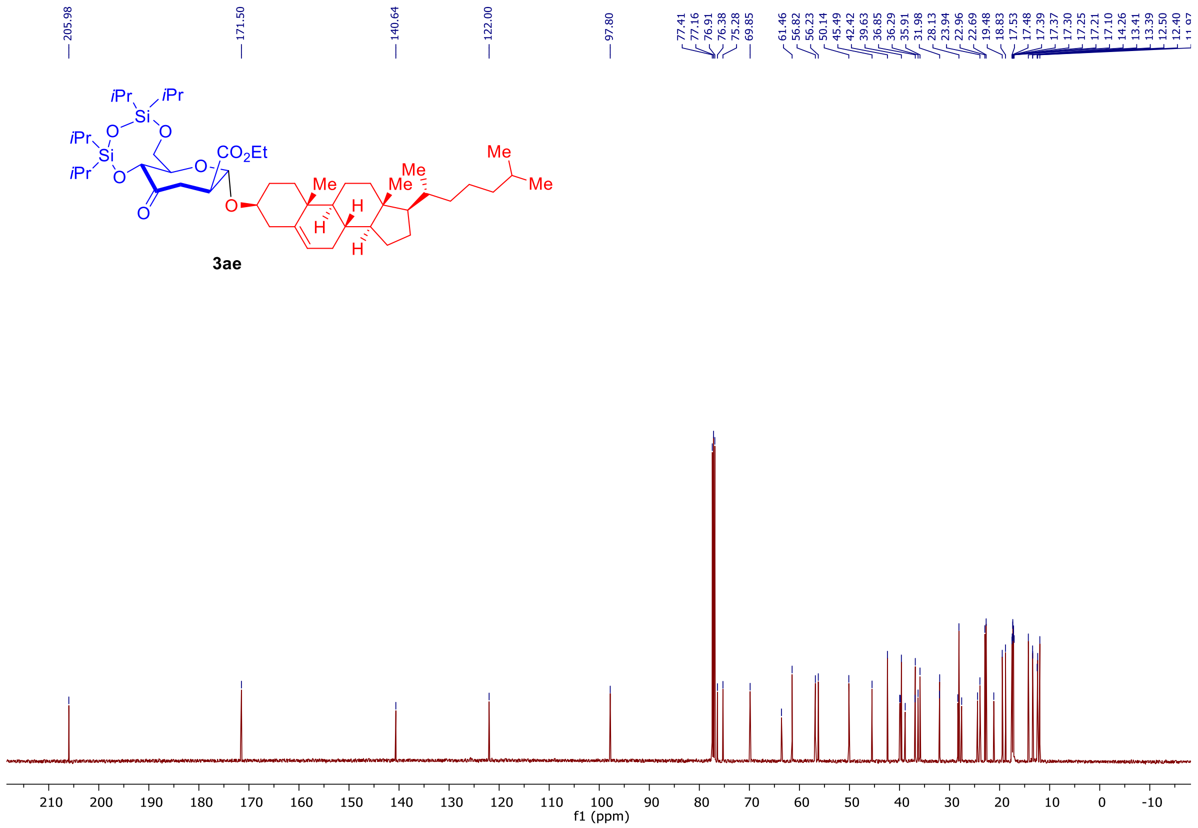

Supplementary Figure 331: ^{13}C spectra for **3ae**

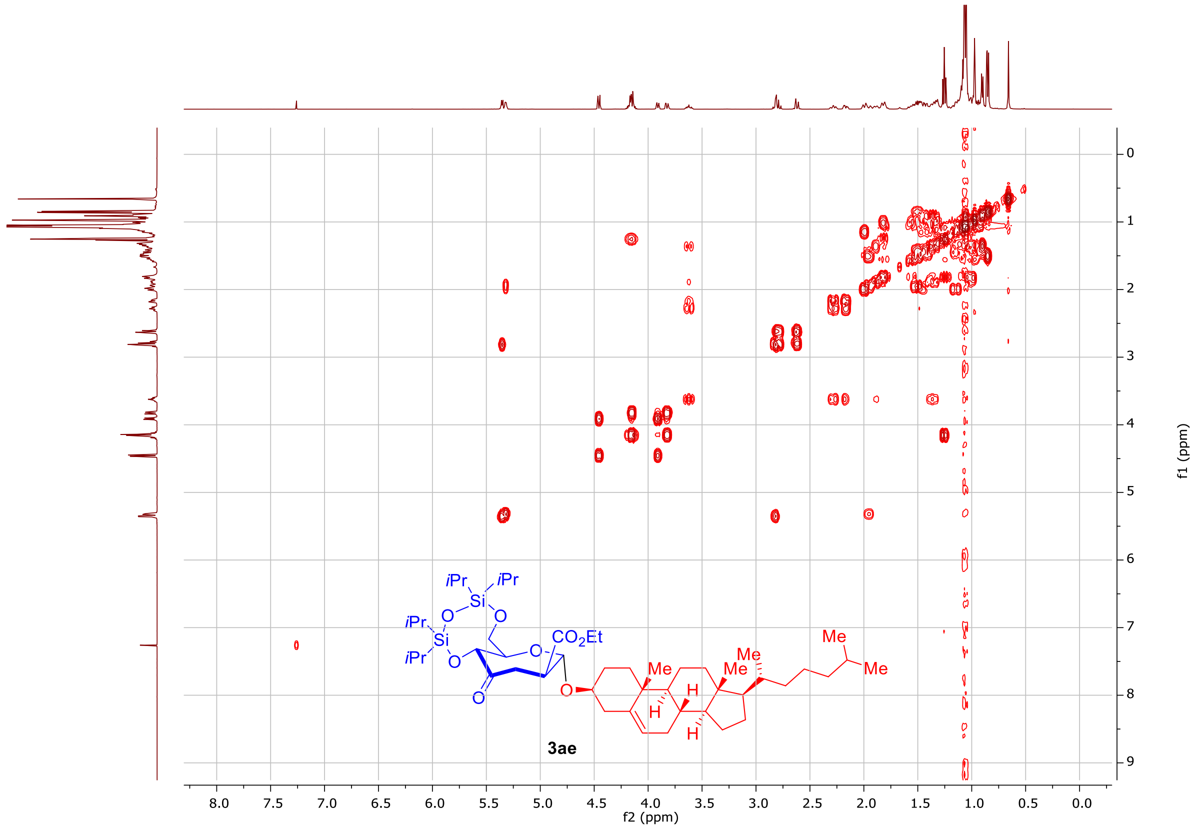

Supplementary Figure 332: COSY spectra for compound **3ae**

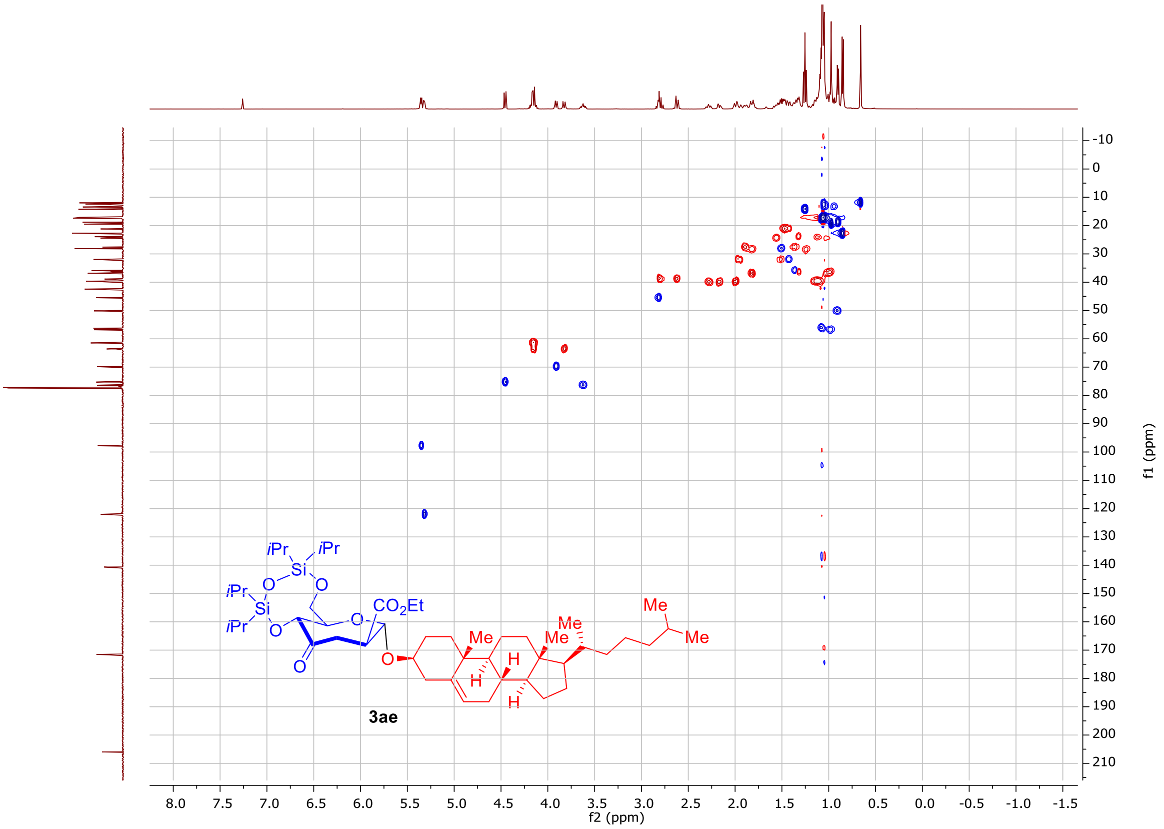

Supplementary Figure 333: HSQC spectra for compound **3ae**

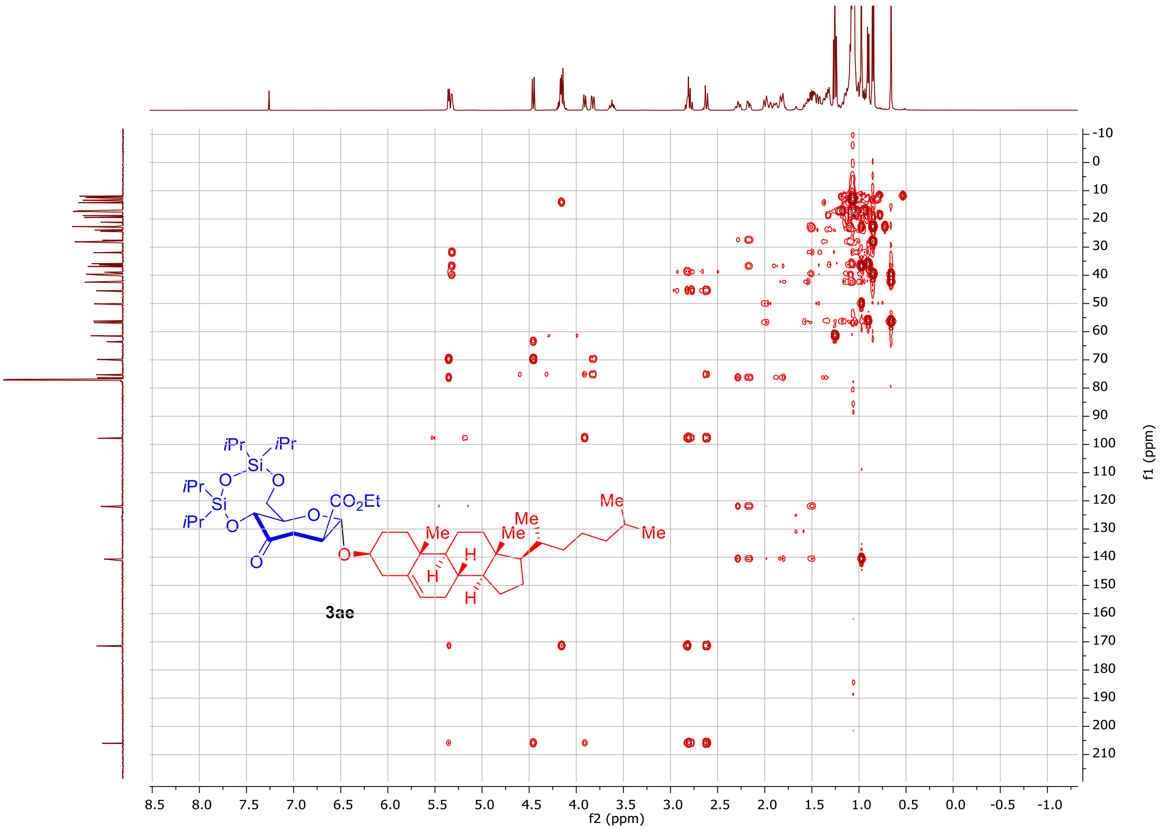

Supplementary Figure 334: HMBC spectra for compound **3ae**

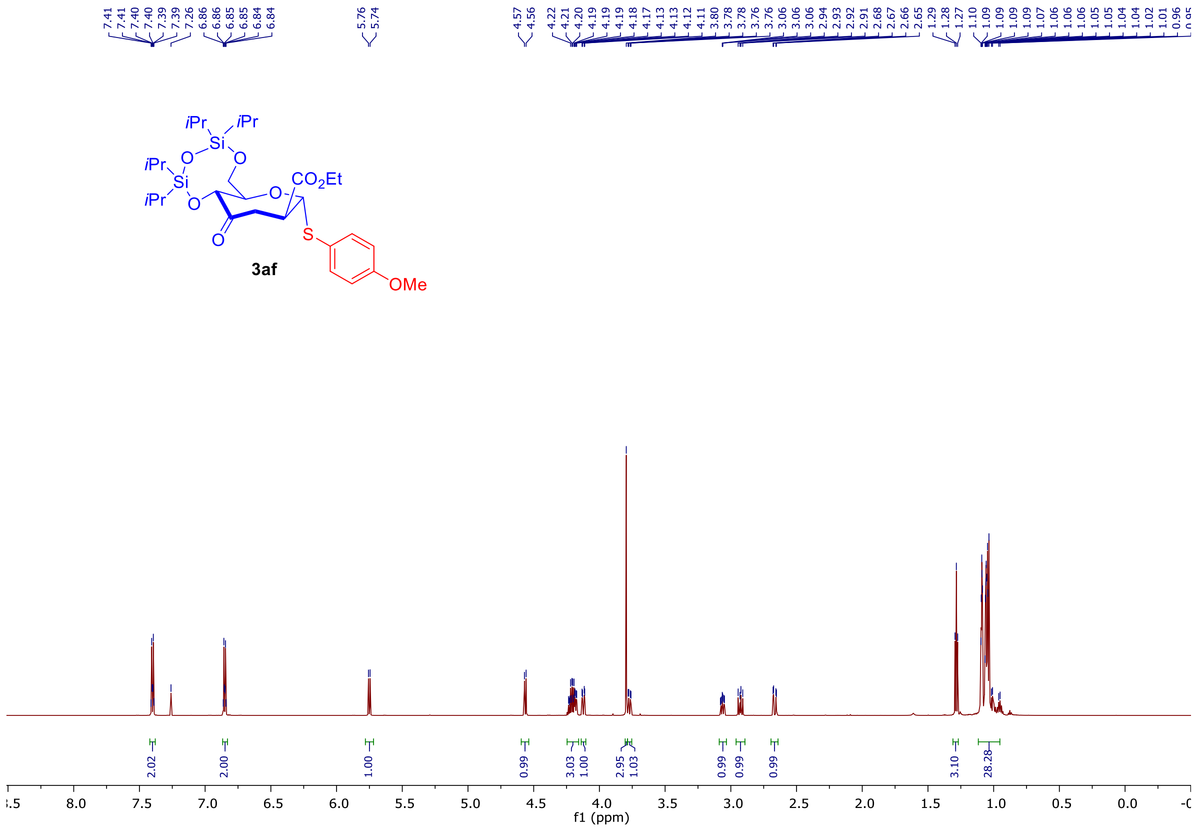

Supplementary Figure 335: ^{1}H spectra for **3af**

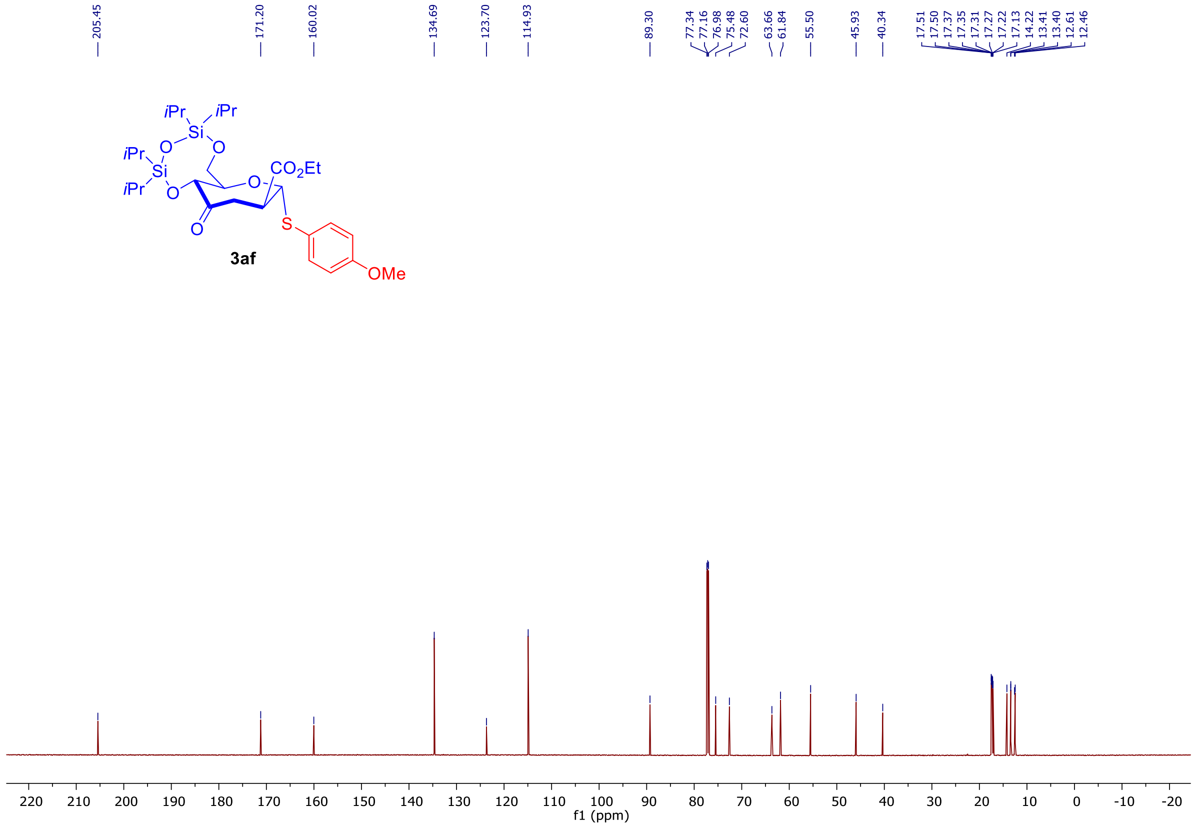

Supplementary Figure 336: ^{13}C spectra for **3af**

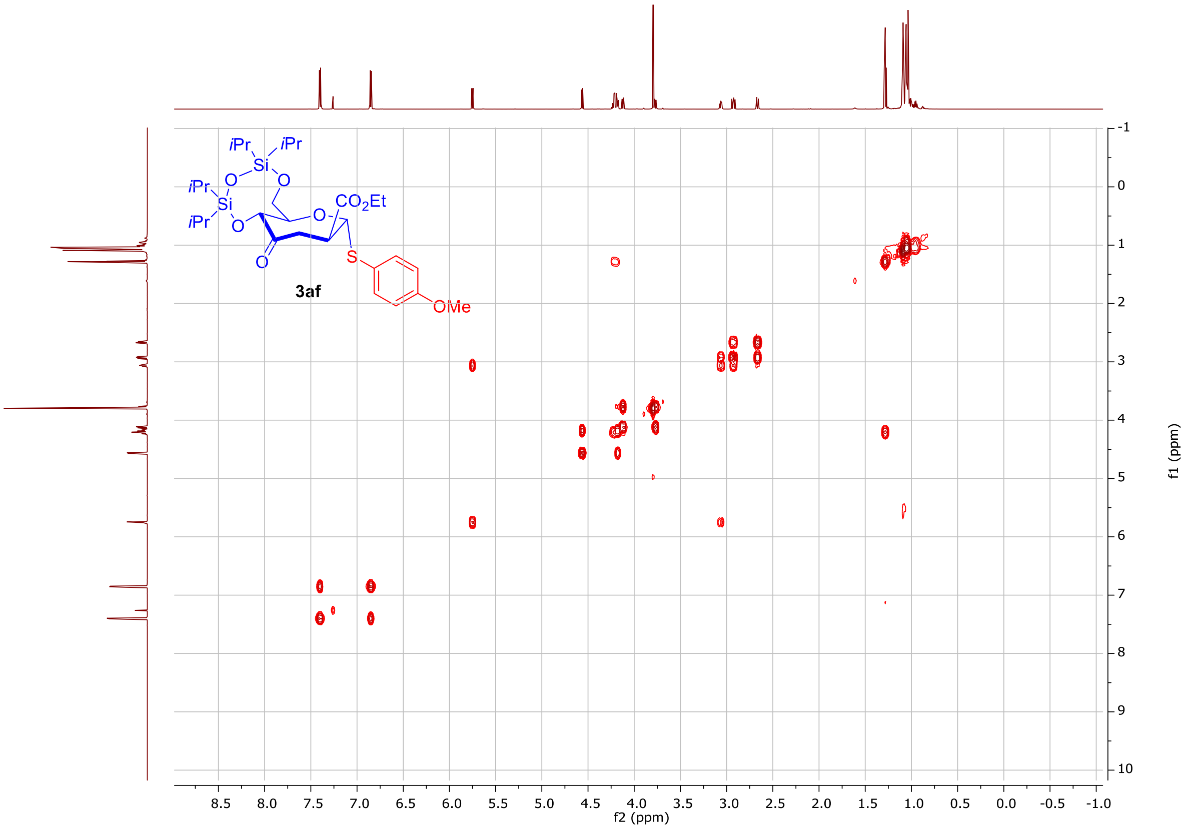

Supplementary Figure 337: COSY spectra for compound **3af**

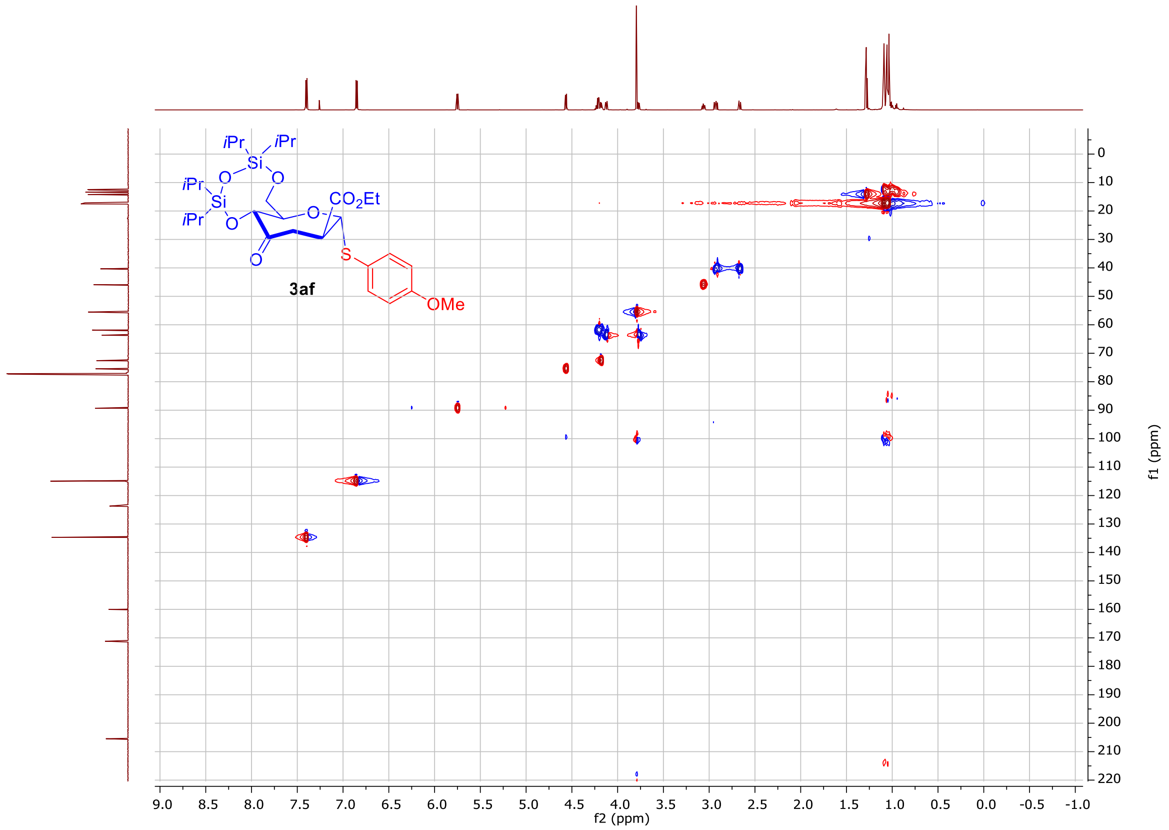

Supplementary Figure 338: HSQC spectra for compound **3af**

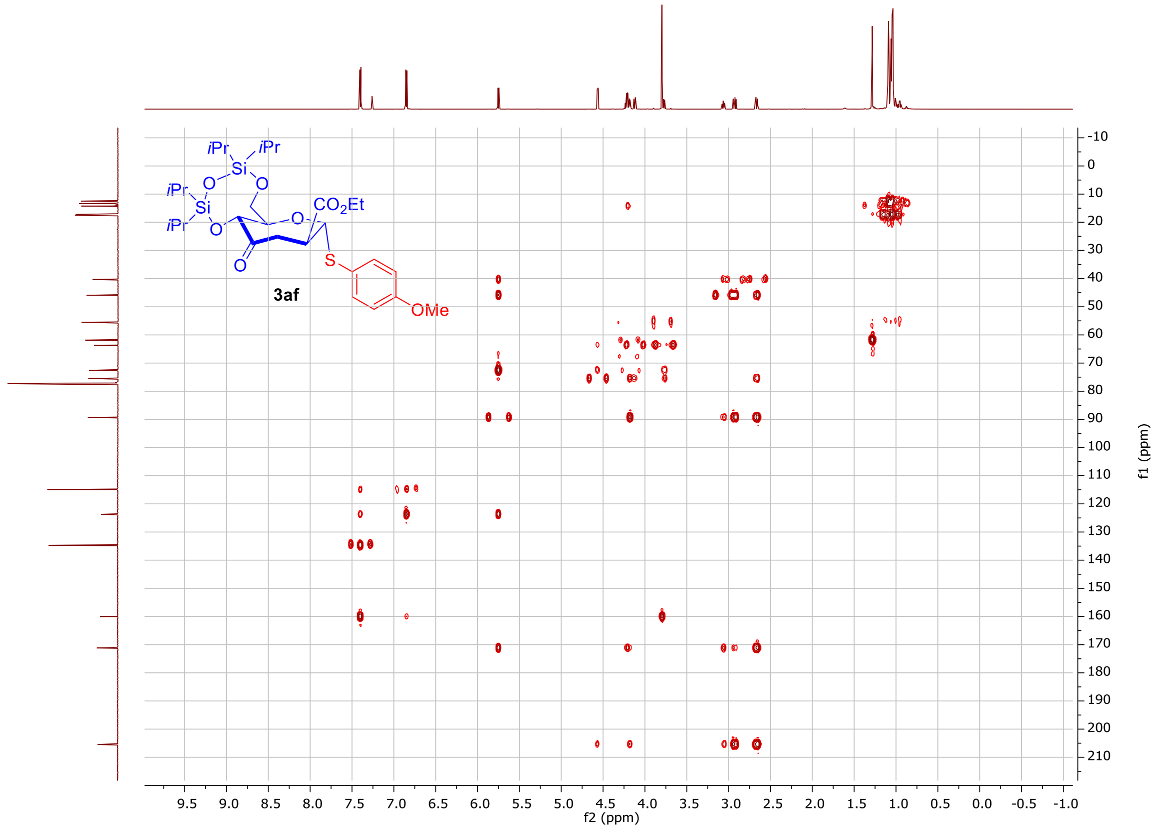

Supplementary Figure 339: HMBC spectra for compound **3af**

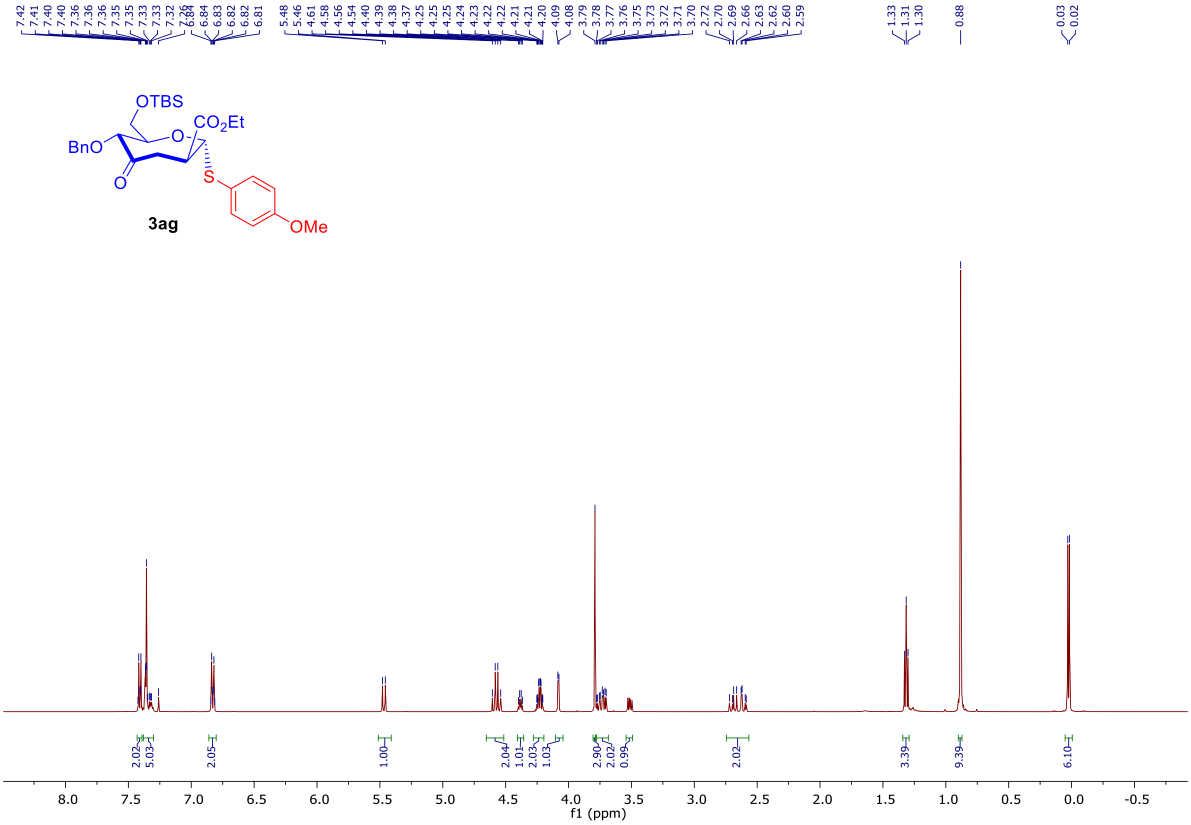

Supplementary Figure 340: ^{1}H spectra for **3ag**

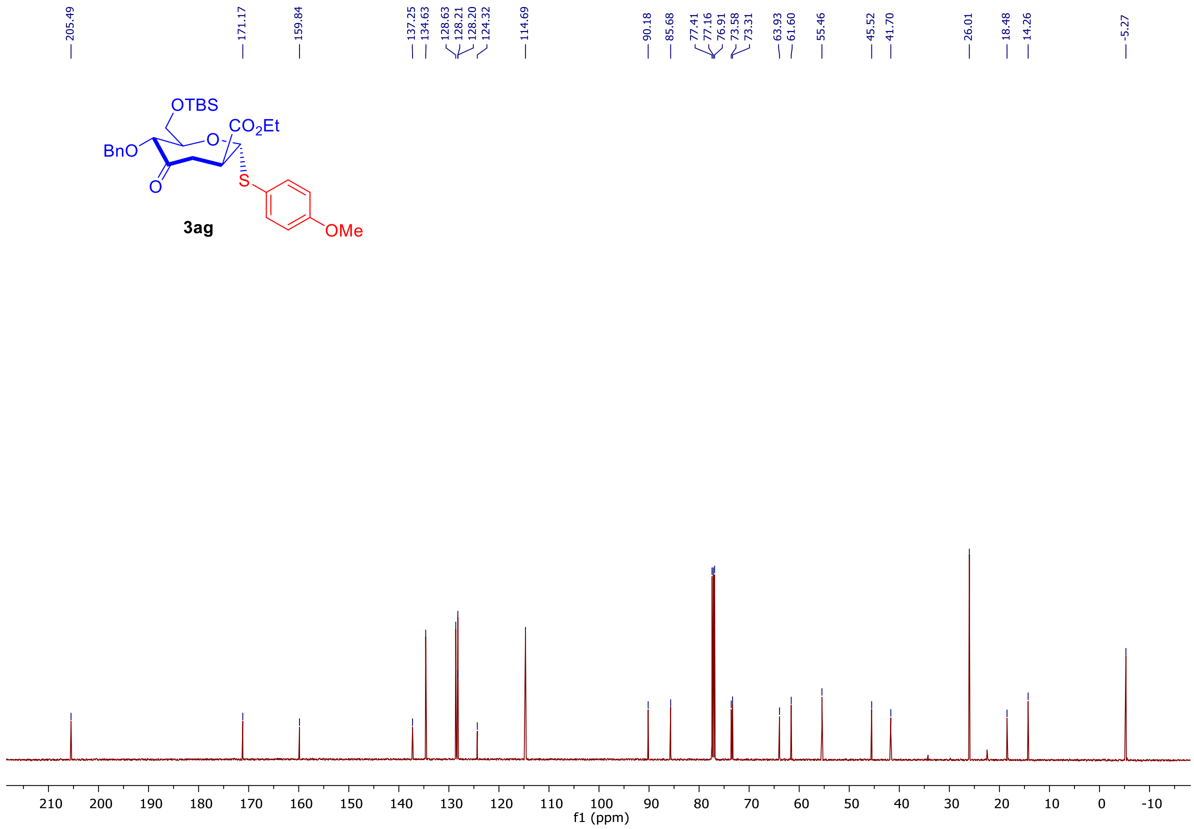

Supplementary Figure 341: ^{13}C spectra for **3ag**

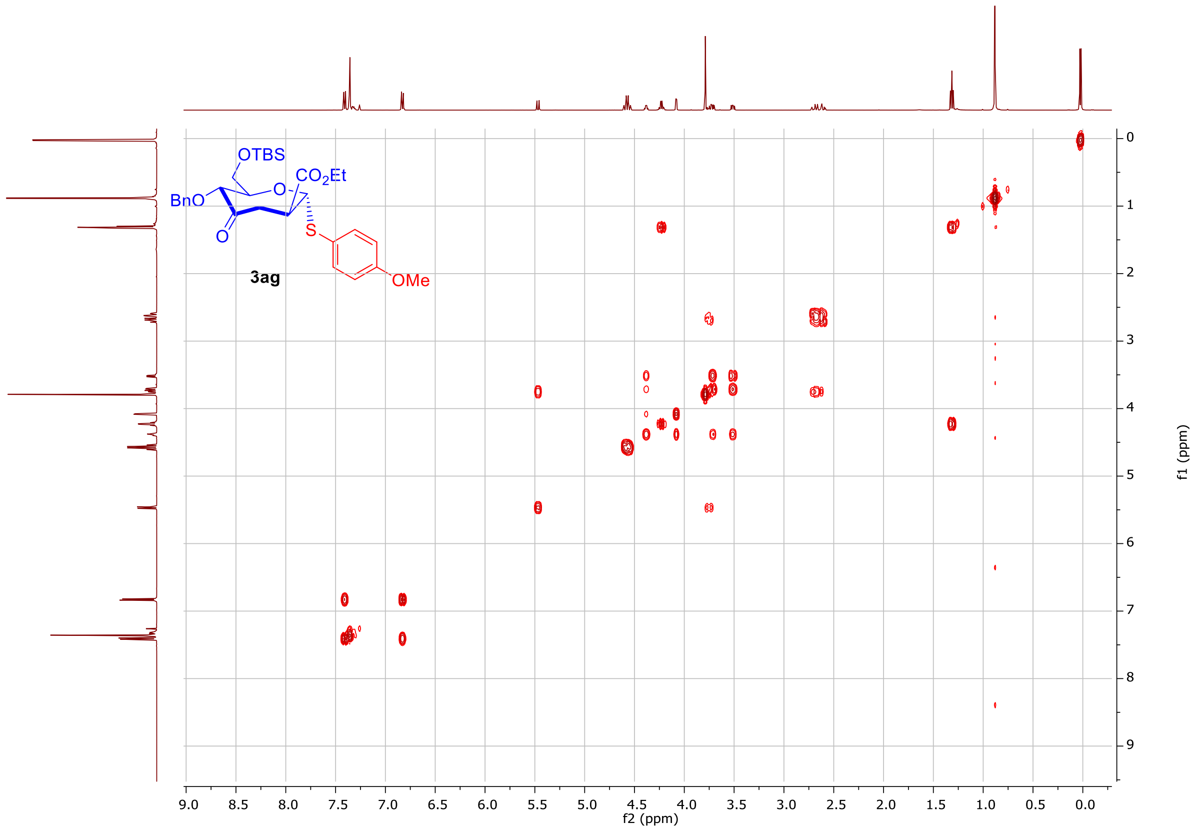

Supplementary Figure 342: COSY spectra for compound **3ag**

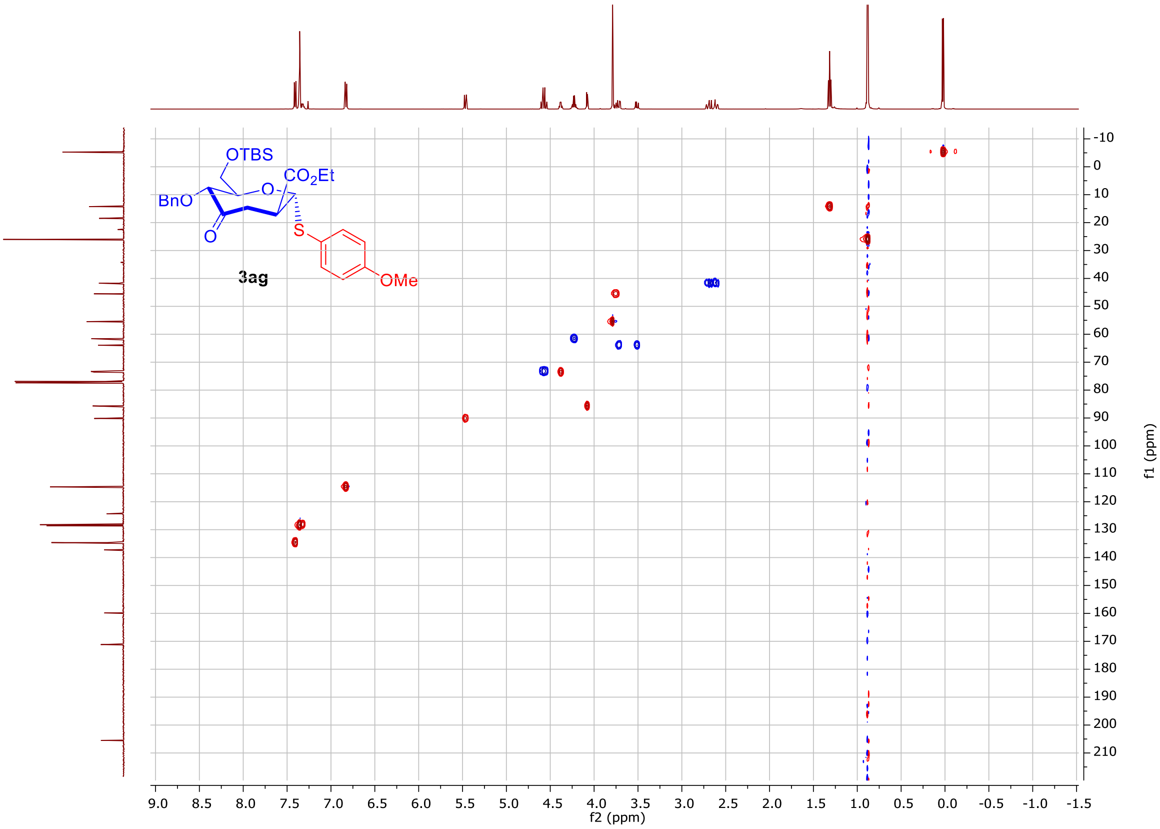

Supplementary Figure 343: HSQC spectra for compound **3ag**

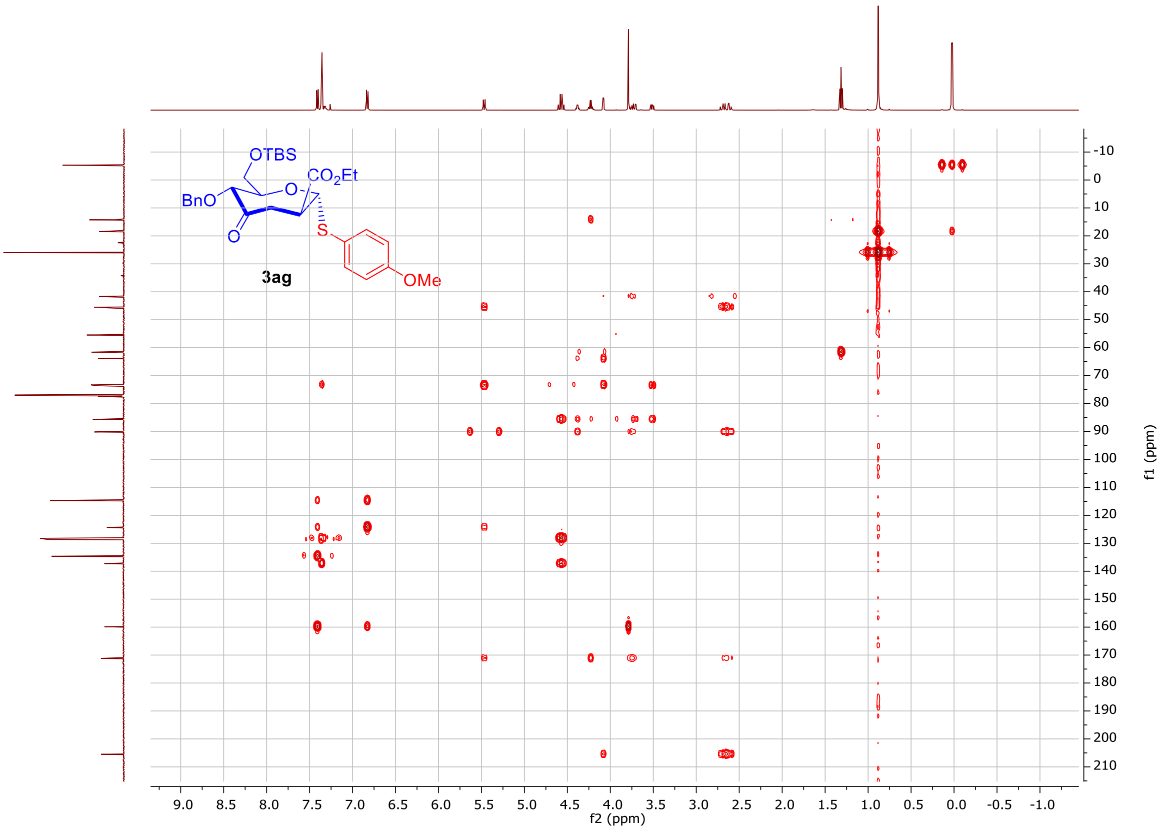

Supplementary Figure 344: HMBC spectra for compound **3ag**

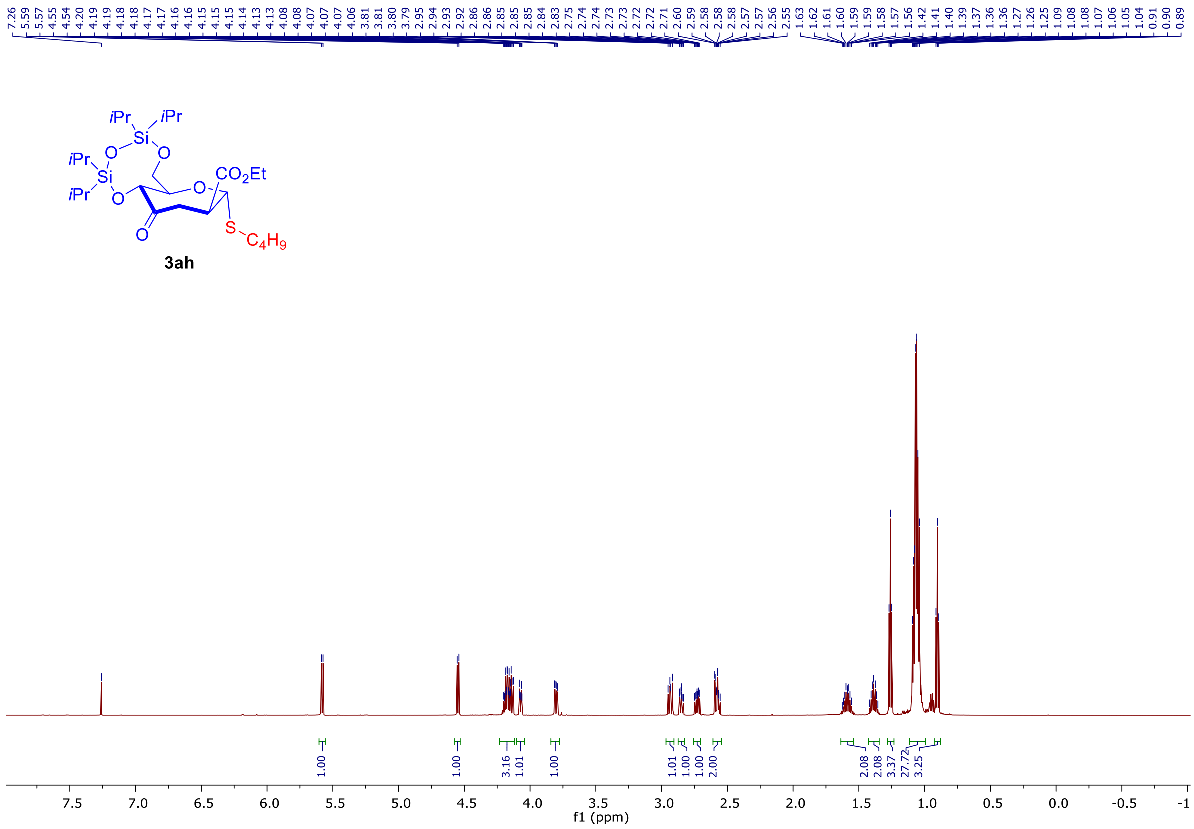

Supplementary Figure 345: ^{1}H spectra for **3ah**

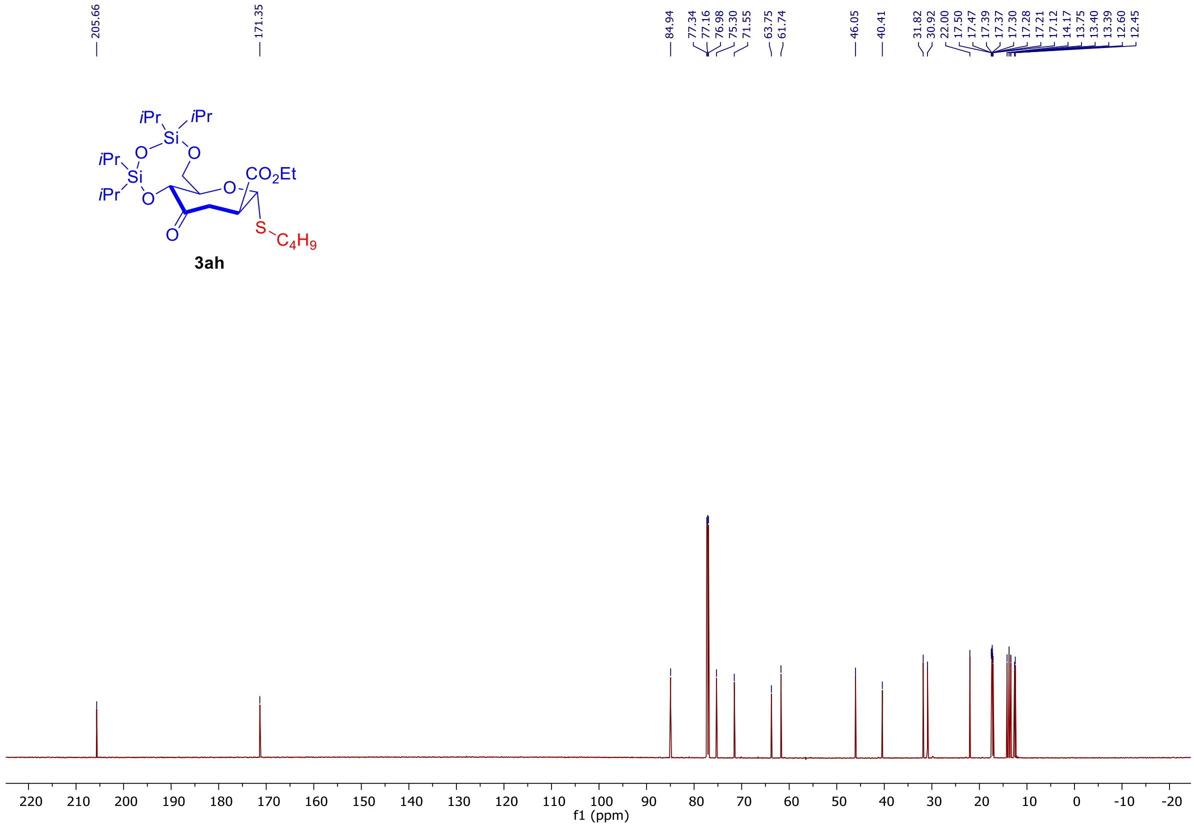

Supplementary Figure 346: ^{13}C spectra for **3ah**

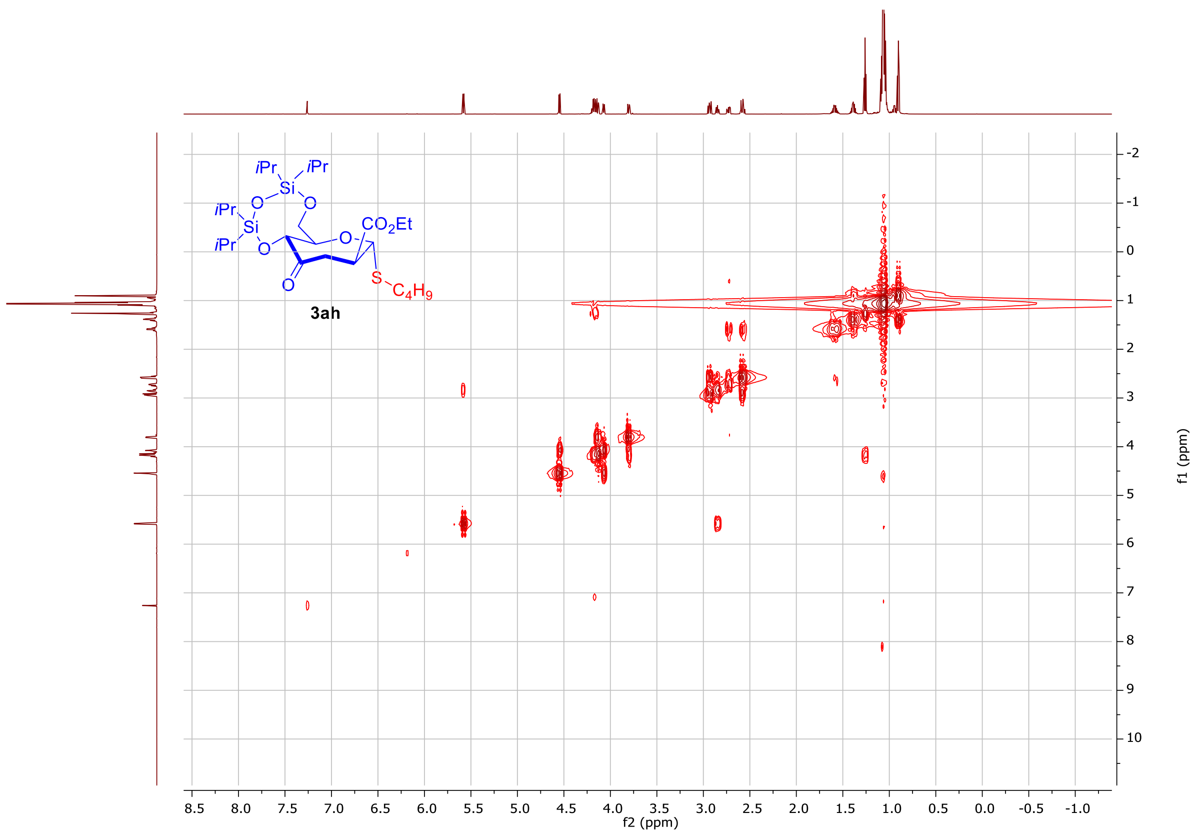

Supplementary Figure 347: COSY spectra for compound **3ah**

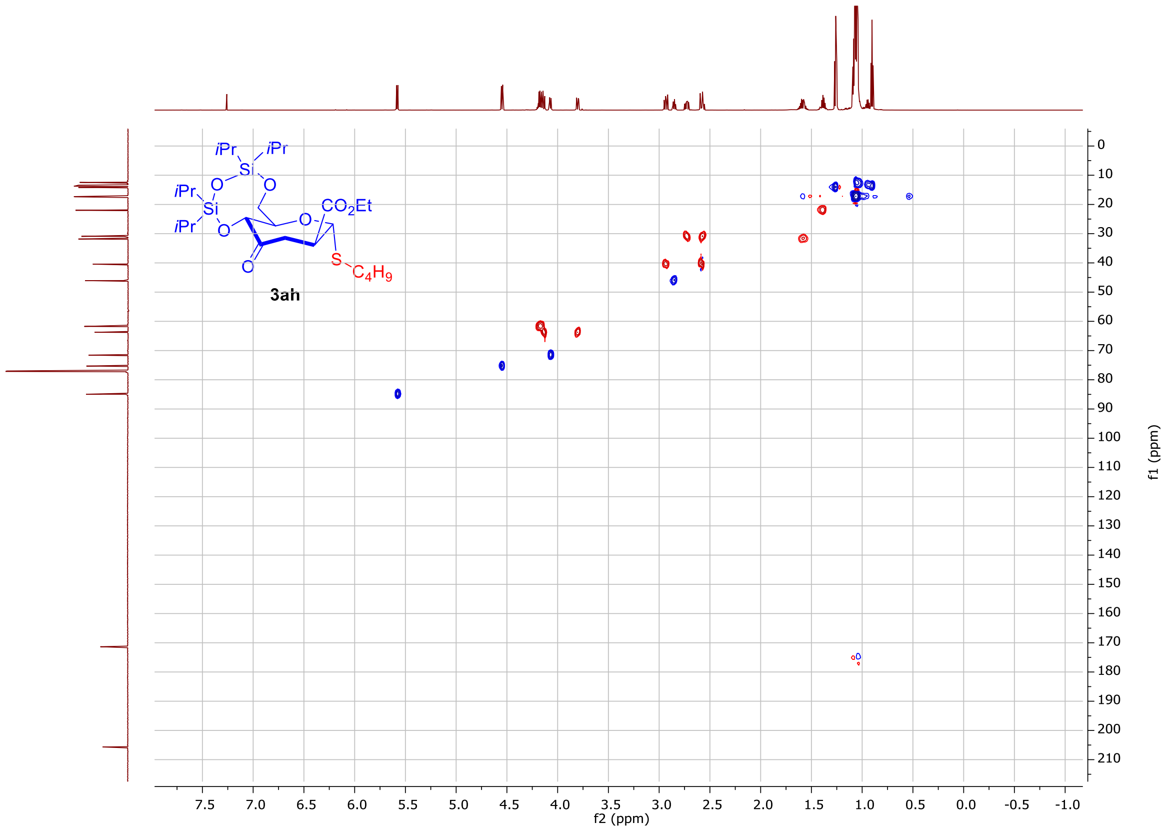

Supplementary Figure 348: HSQC spectra for compound **3ah**

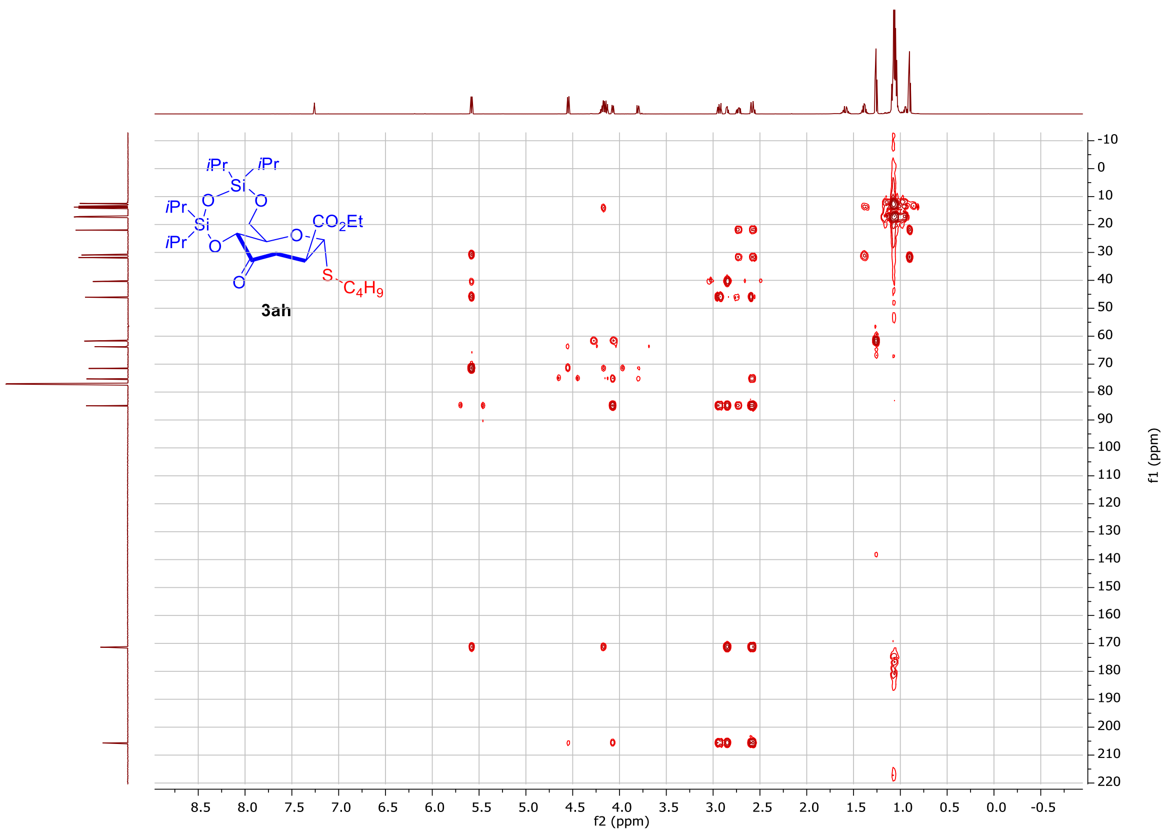

Supplementary Figure 349: HMBC spectra for compound **3ah**

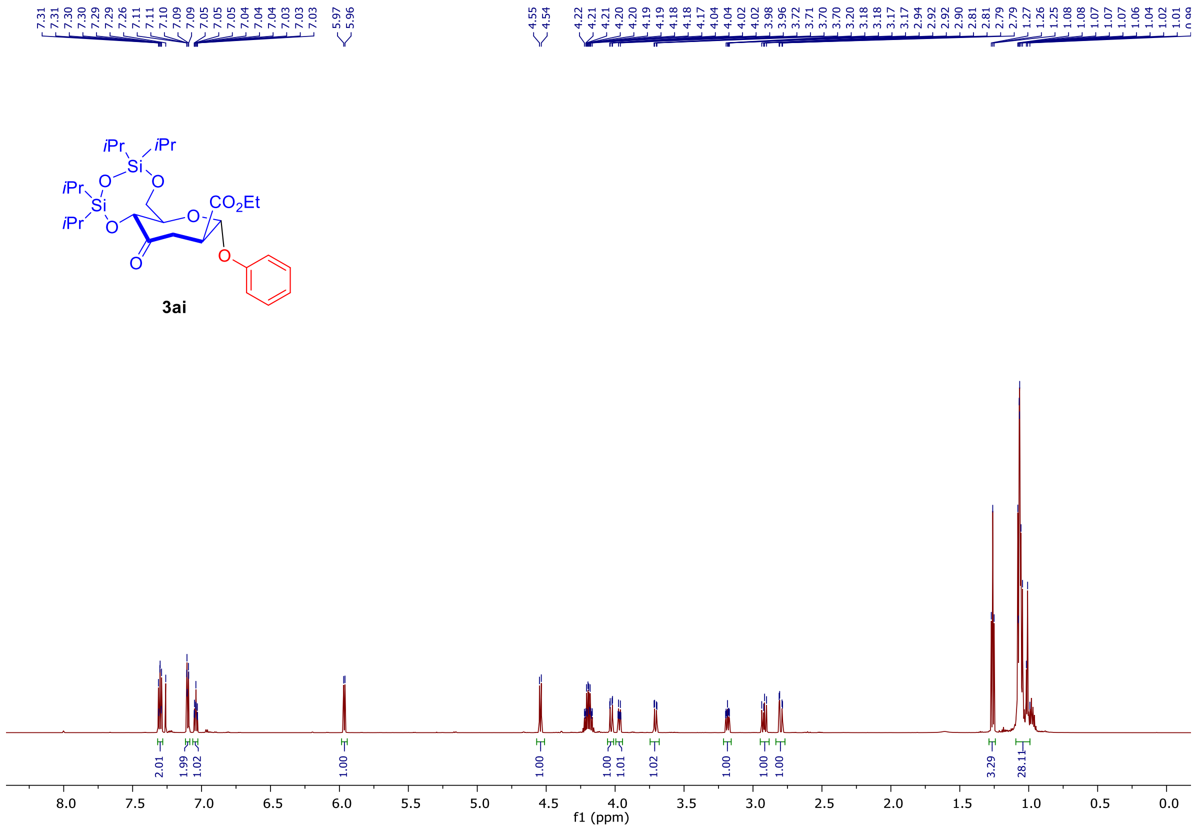

Supplementary Figure 350: ^{1}H spectra for **3ai**

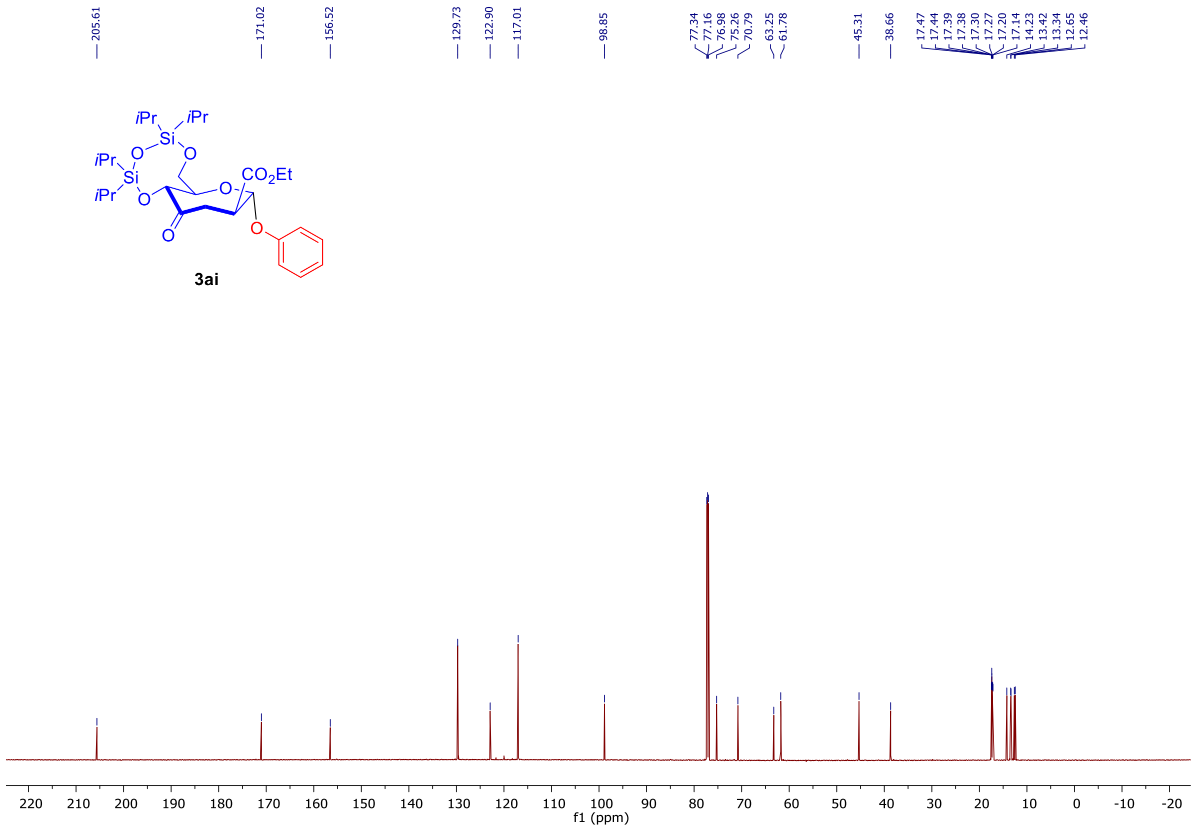

Supplementary Figure 351: ^{13}C spectra for **3ai**

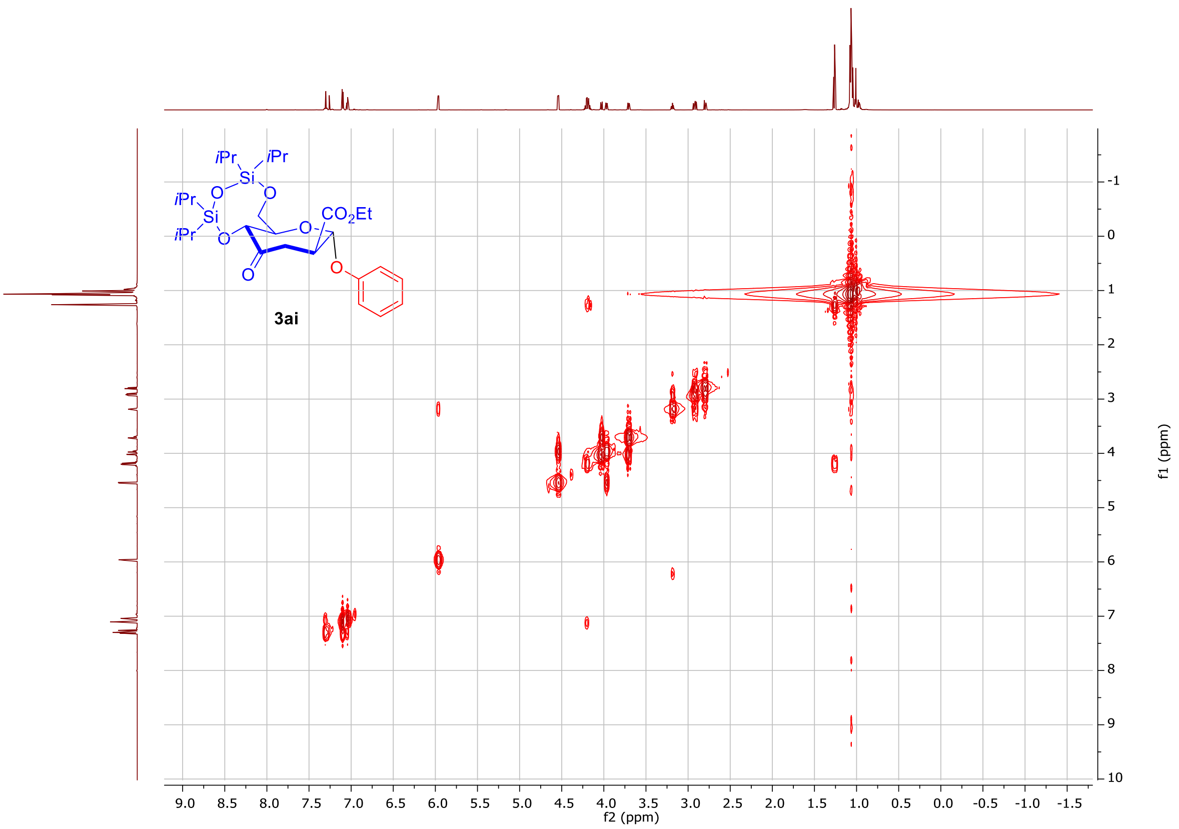

Supplementary Figure 352: COSY spectra for compound **3ai**

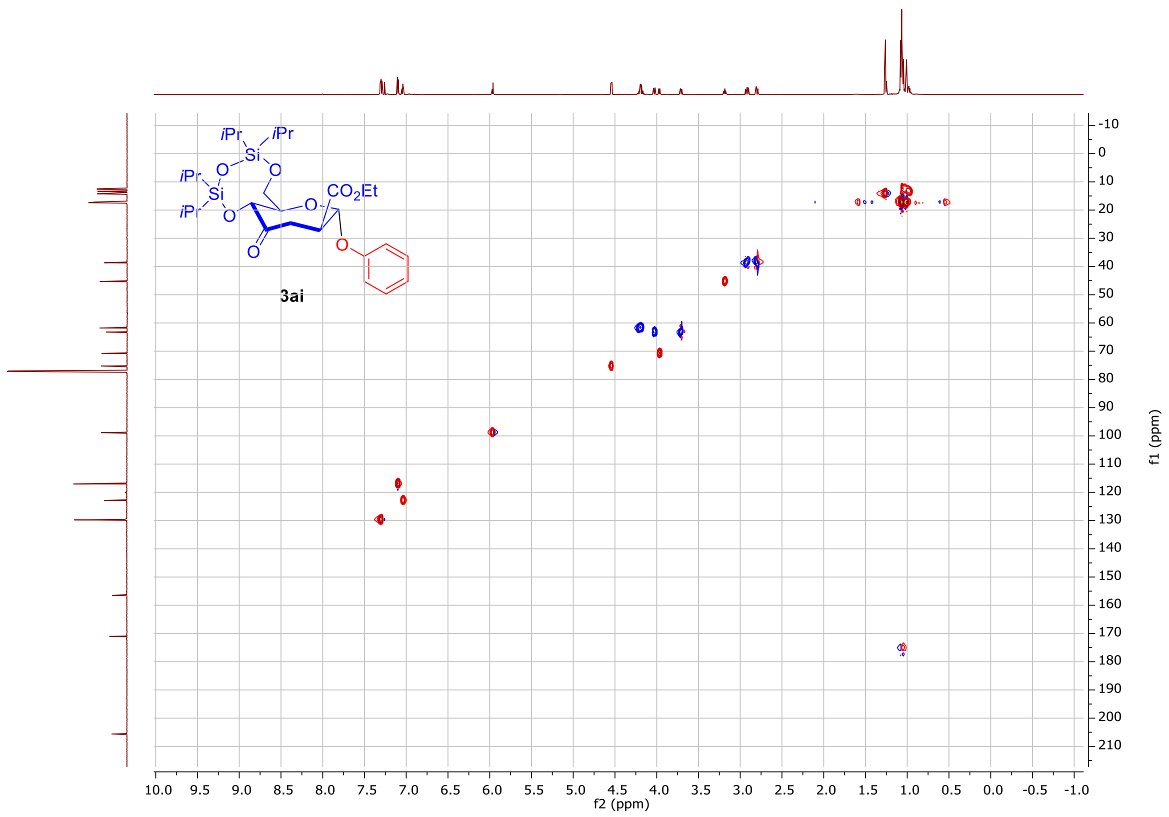

Supplementary Figure 353: HSQC spectra for compound **3ai**

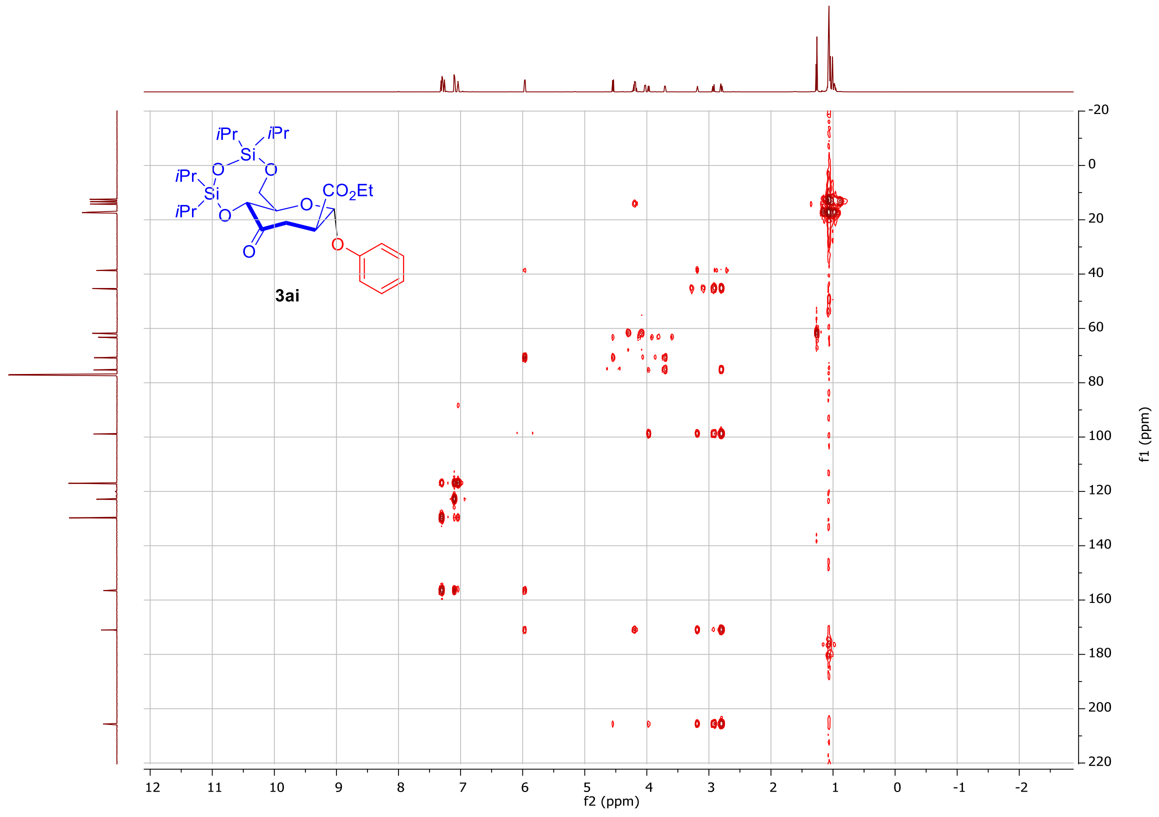

Supplementary Figure 354: HMBC spectra for compound **3ai**

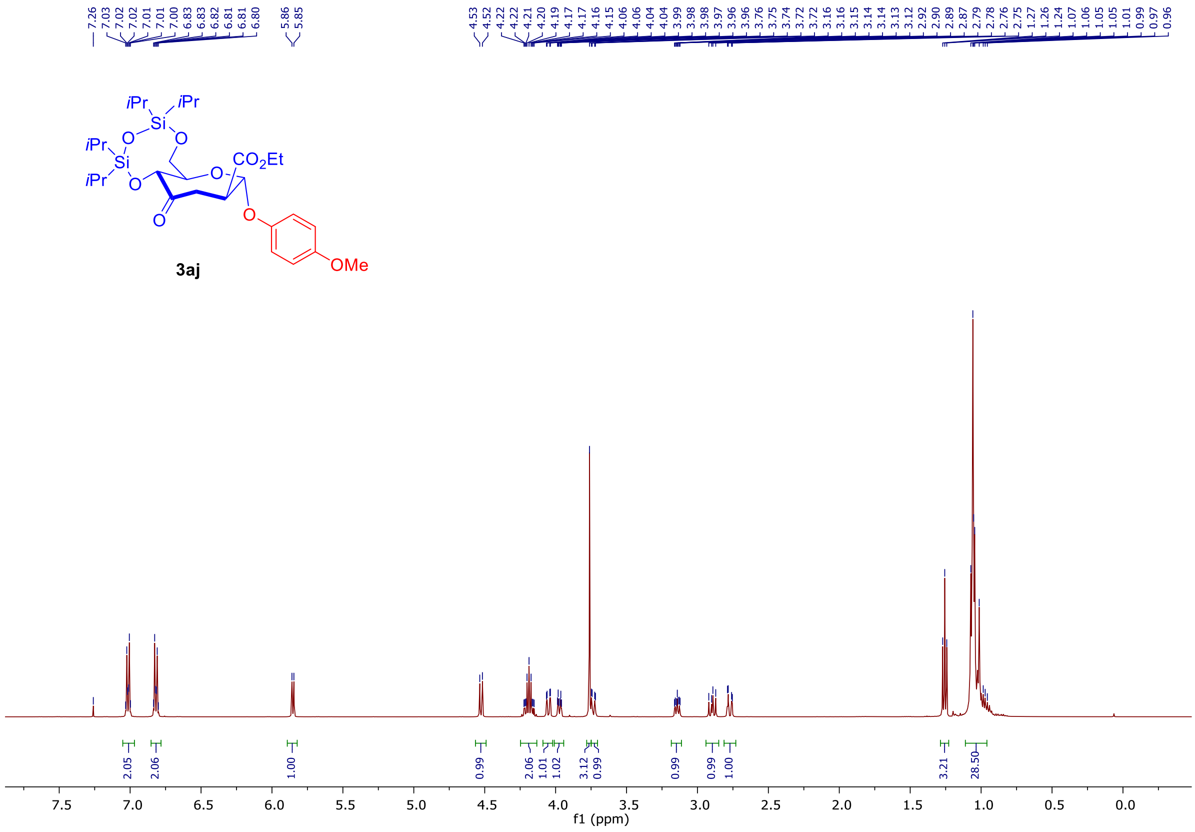

Supplementary Figure 355: ^{1}H spectra for **3aj**

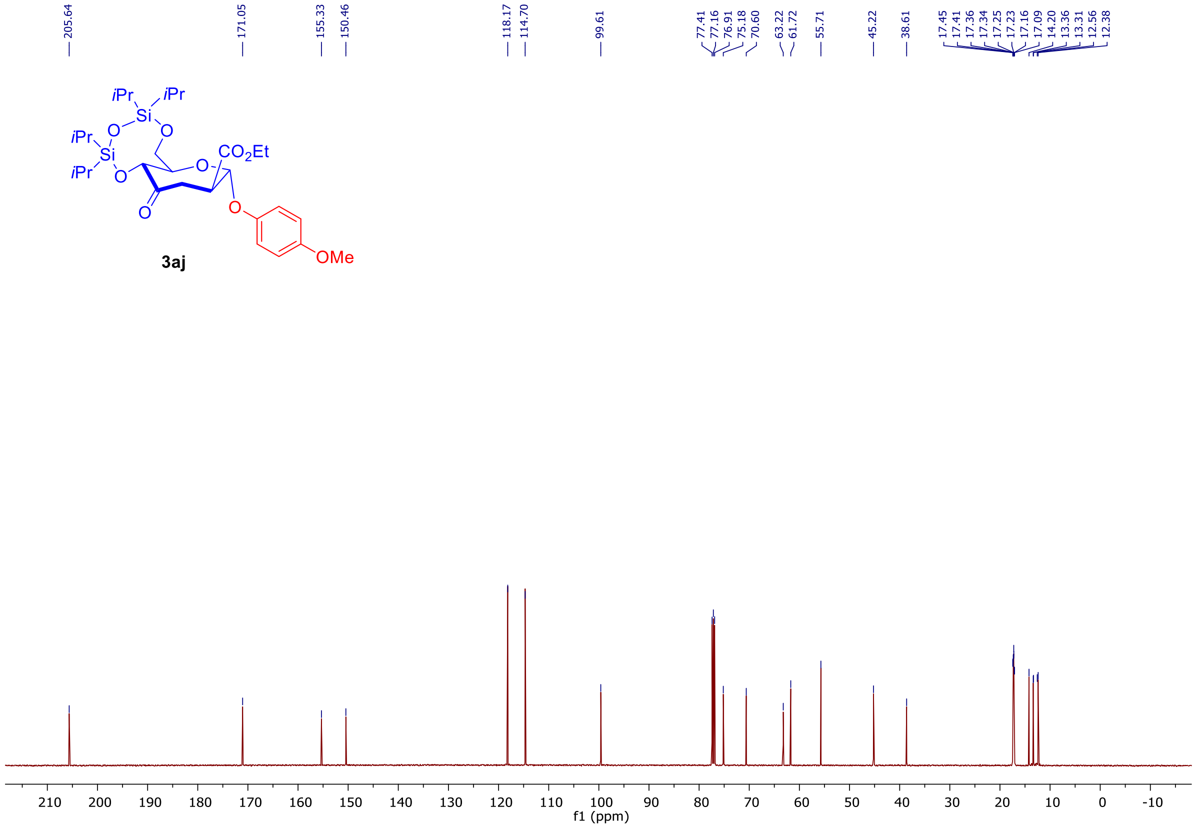

Supplementary Figure 356: ^{13}C spectra for **3aj**

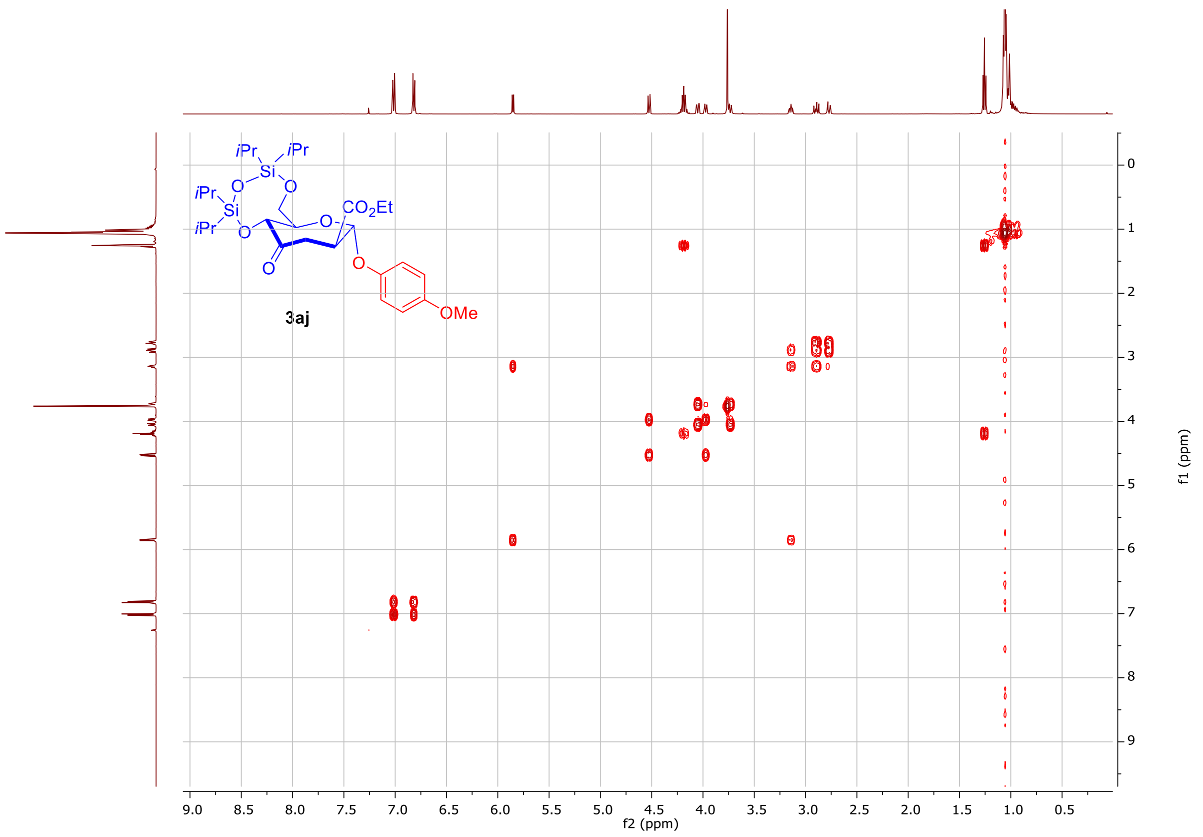

Supplementary Figure 357: COSY spectra for compound **3aj**

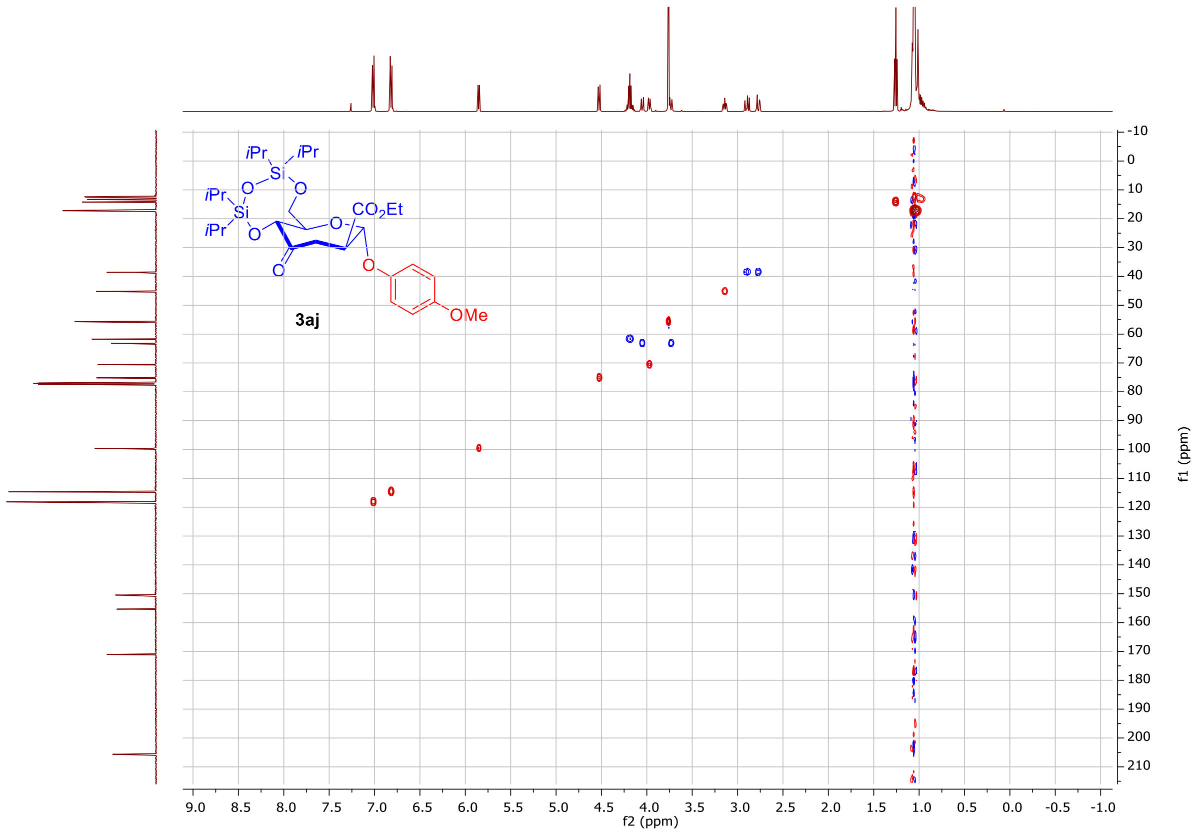

Supplementary Figure 358: HSQC spectra for compound **3aj**

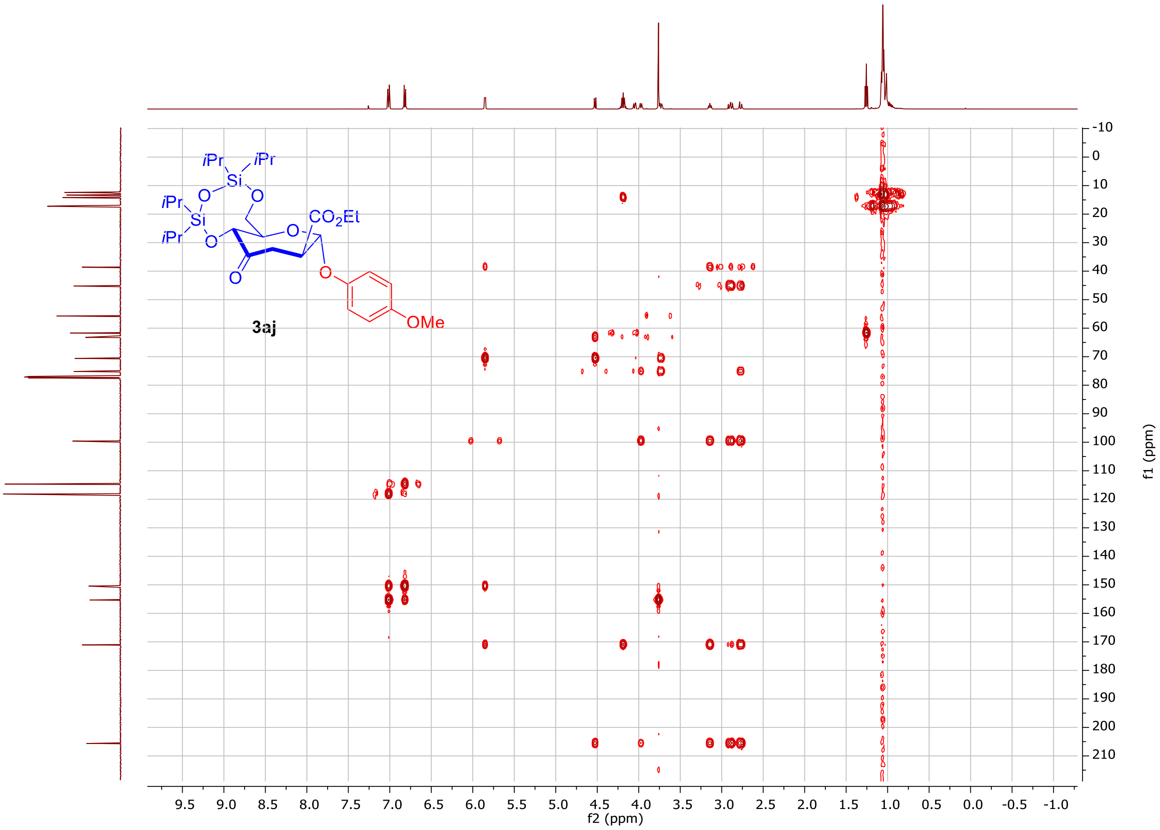

Supplementary Figure 359: HMBC spectra for compound **3aj**

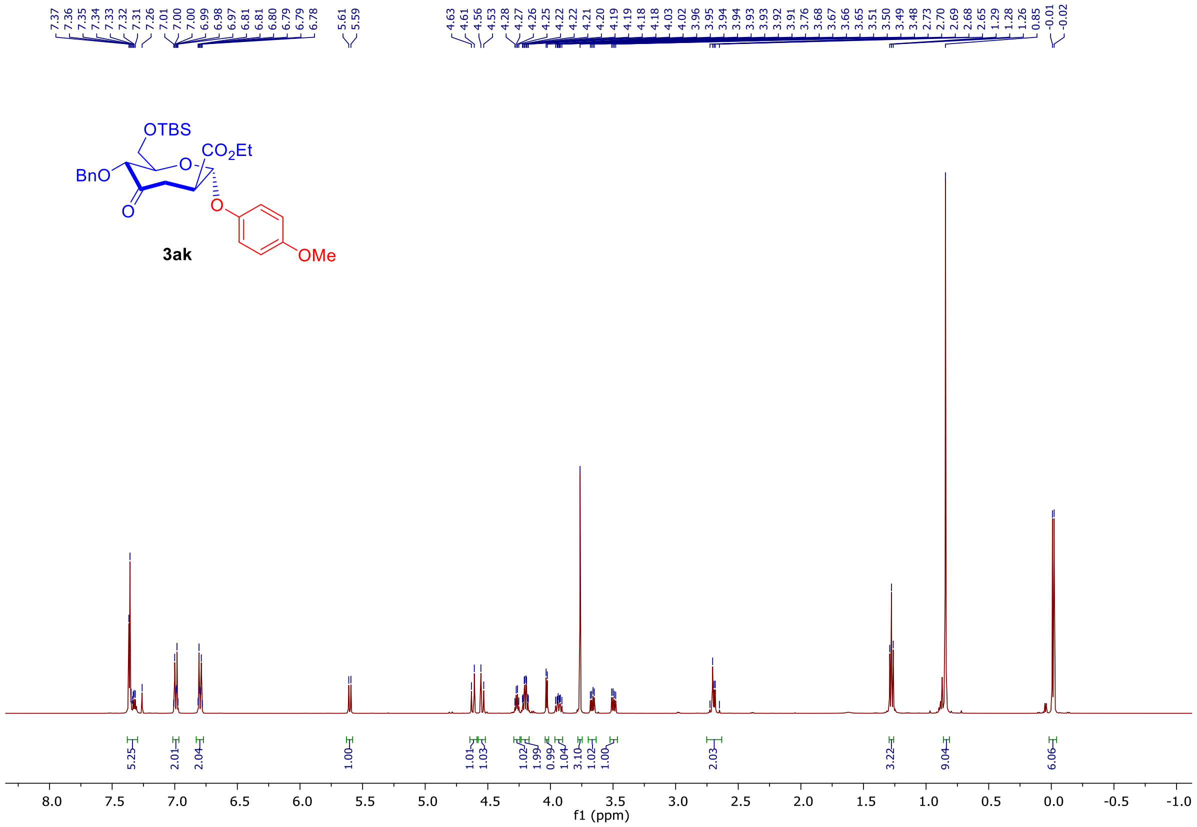

Supplementary Figure 360: ^{1}H spectra for **3ak**

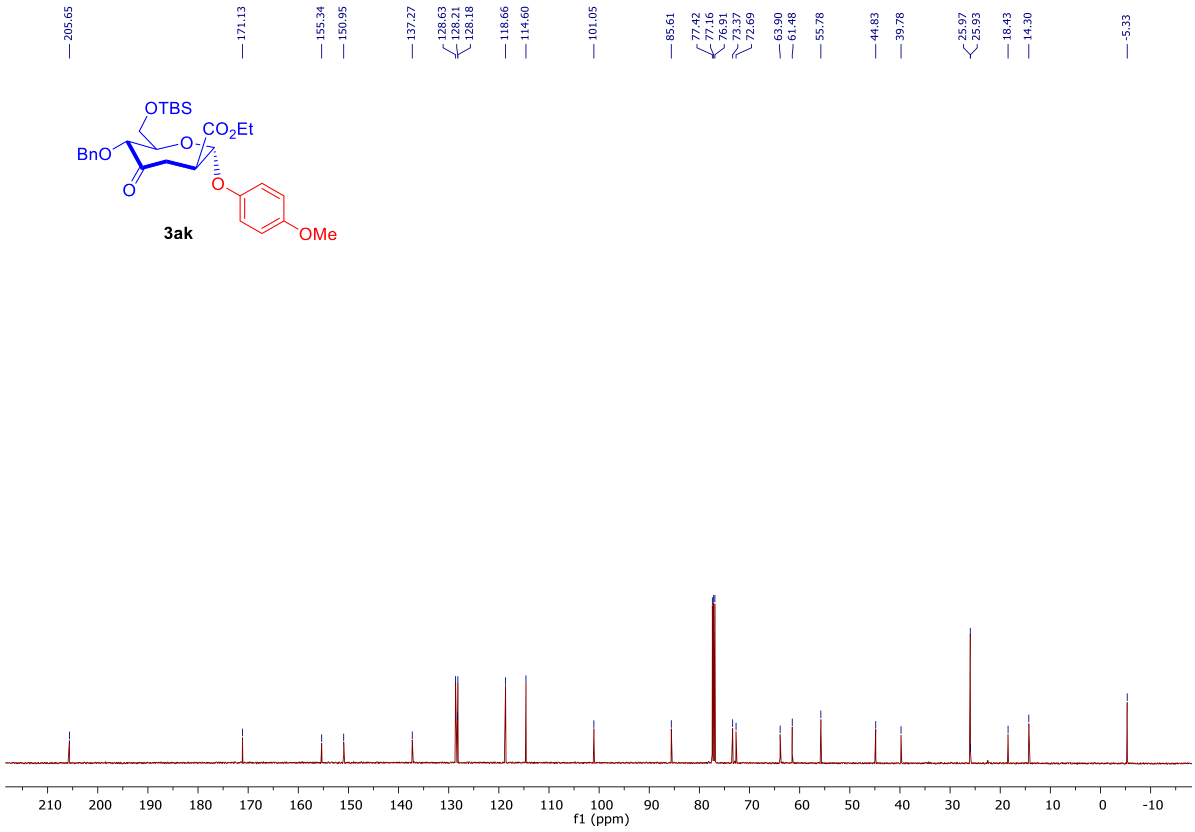

Supplementary Figure 361: ^{13}C spectra for **3ak**

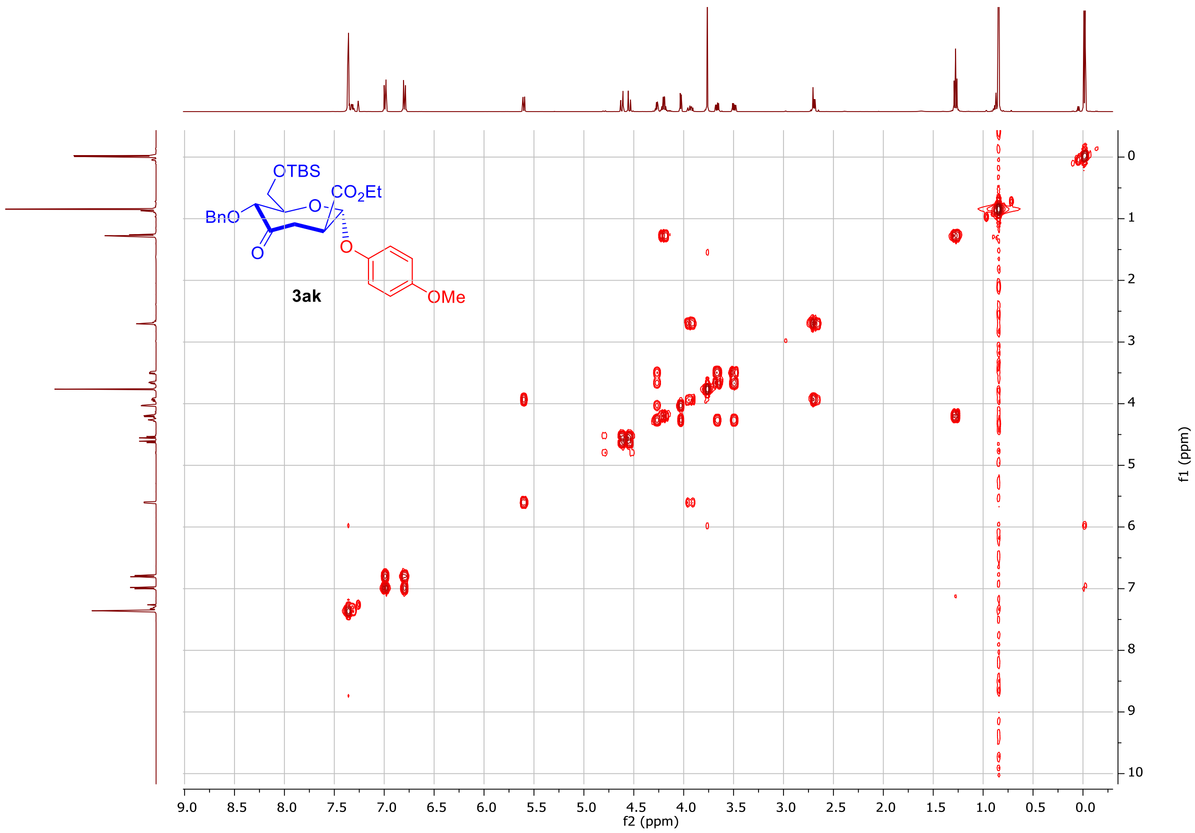

Supplementary Figure 362: COSY spectra for compound **3ak**

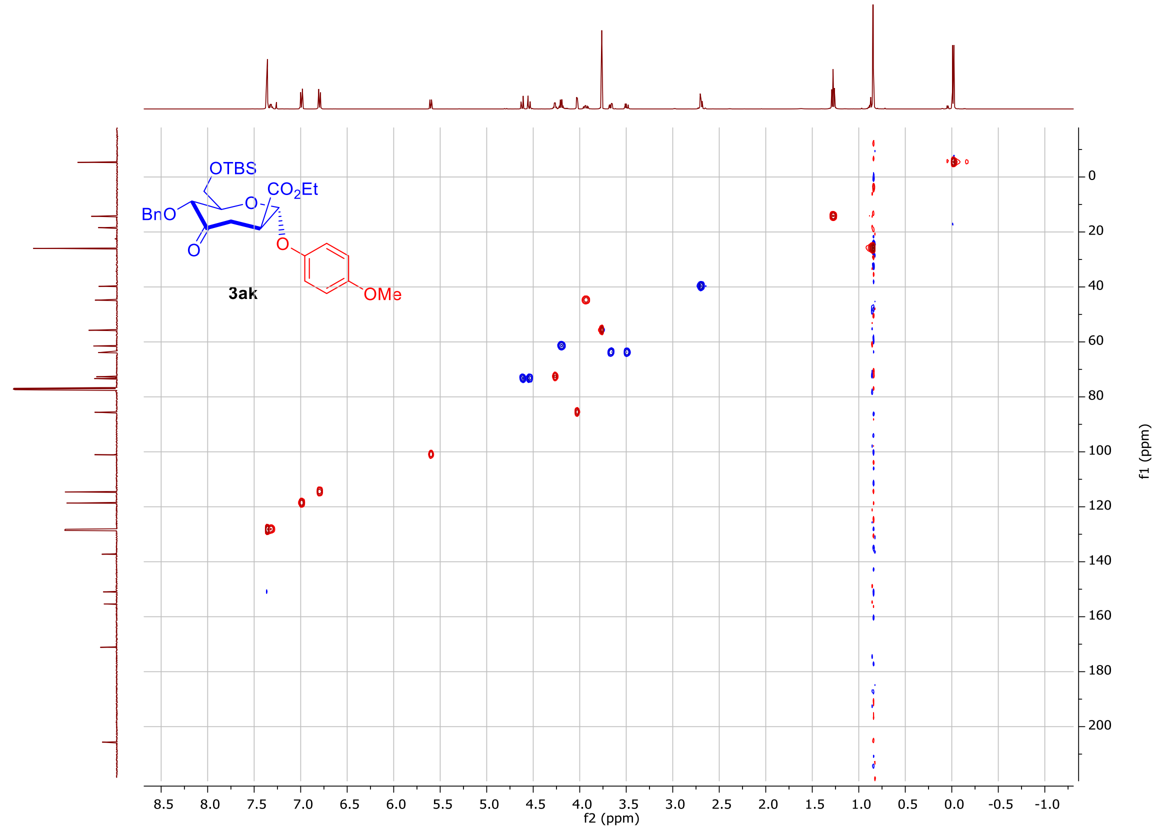

Supplementary Figure 363: HSQC spectra for compound **3ak**

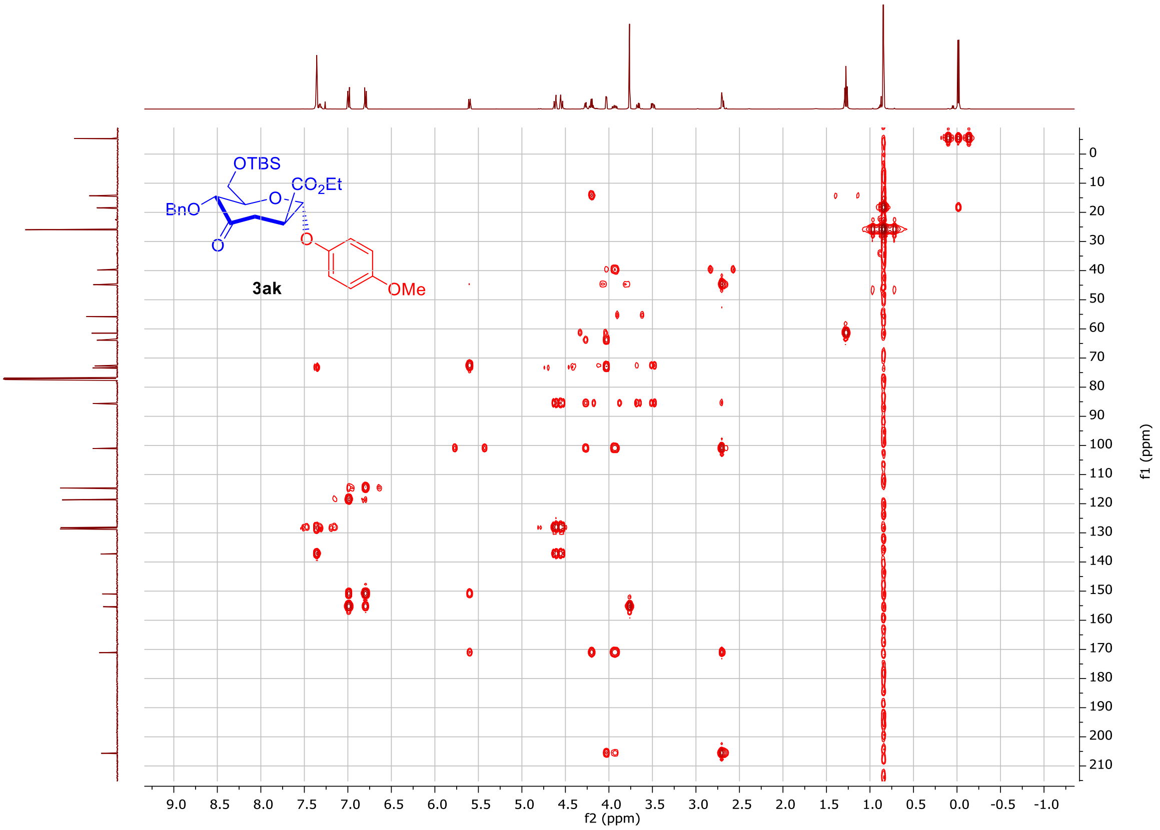

Supplementary Figure 364: HMBC spectra for compound **3ak**

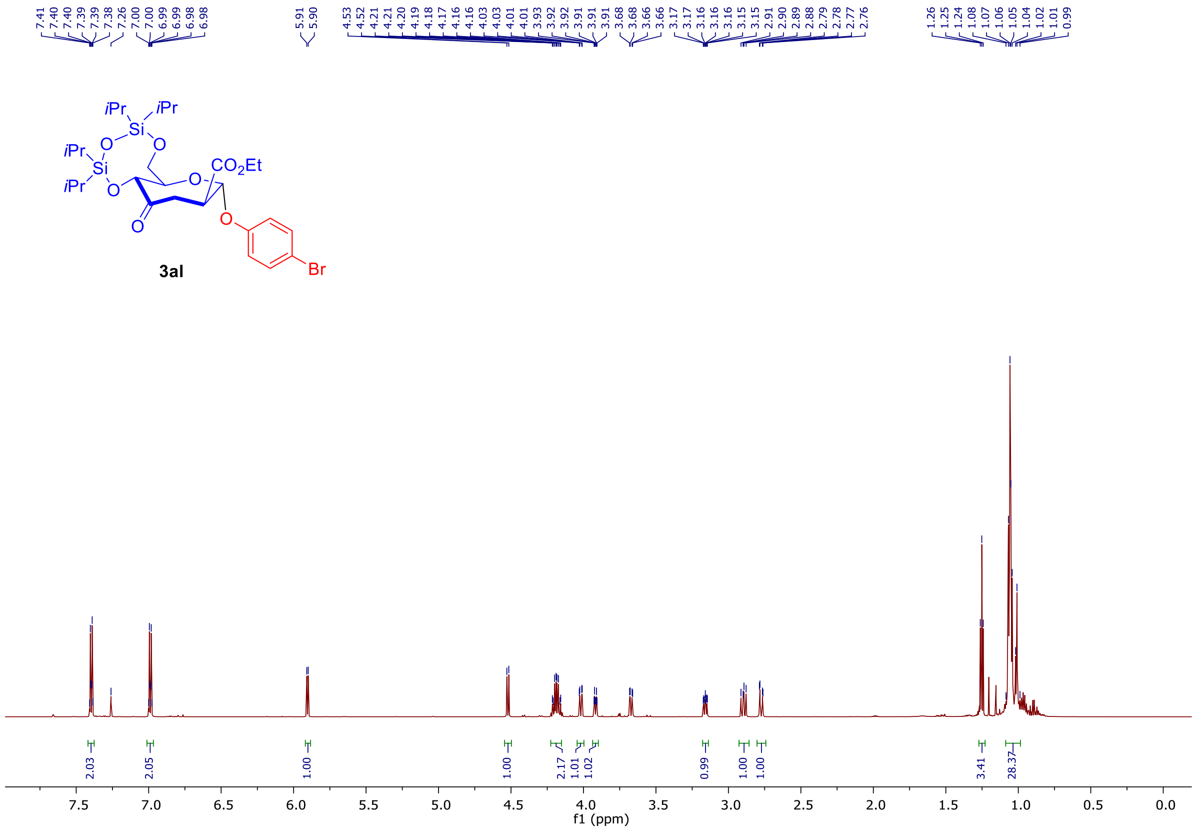

Supplementary Figure 365: ^{1}H spectra for **3al**

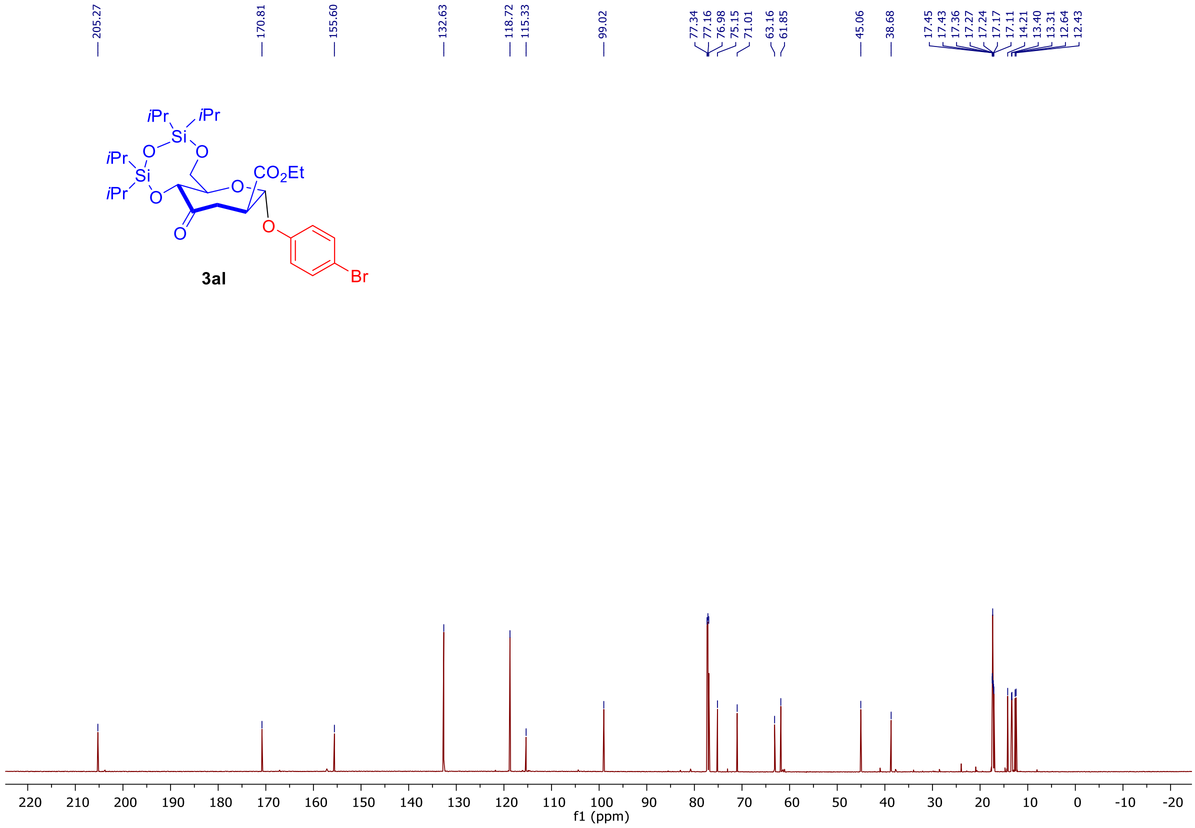

Supplementary Figure 366: ^{13}C spectra for **3al**

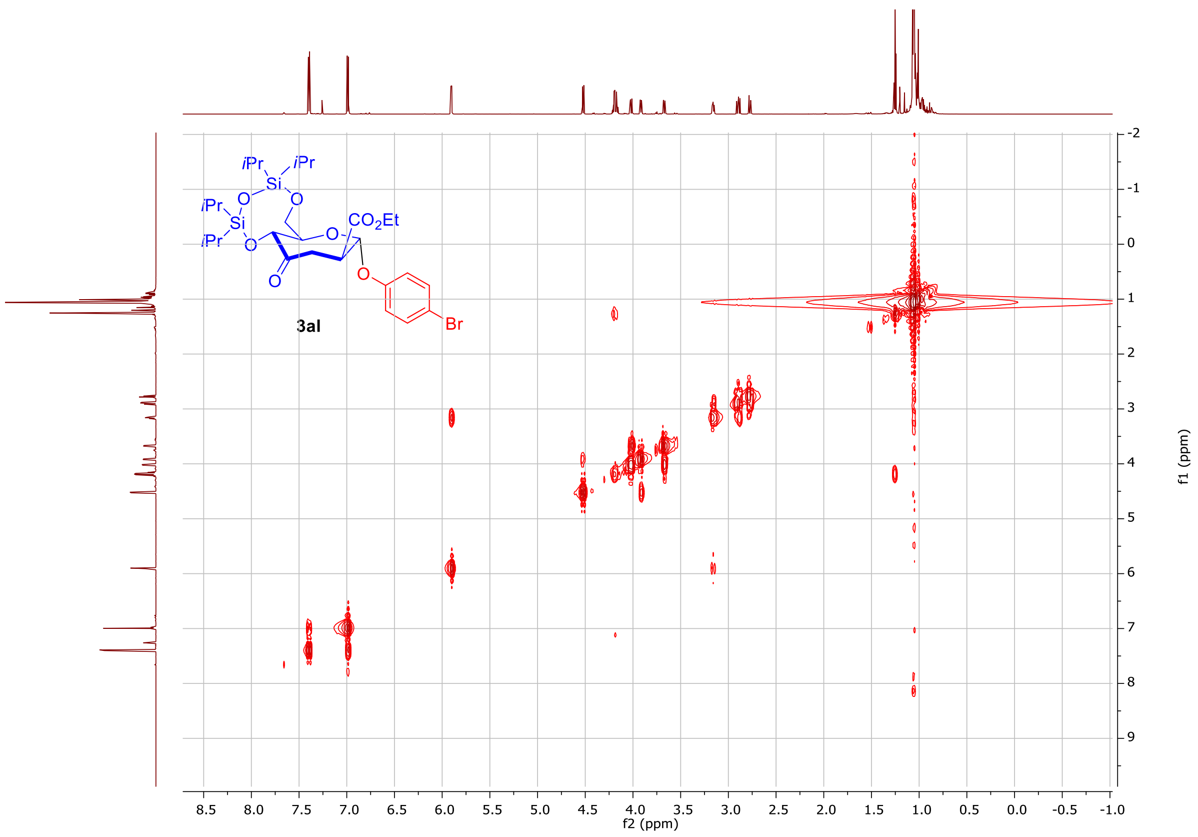

Supplementary Figure 367: COSY spectra for compound **3al**

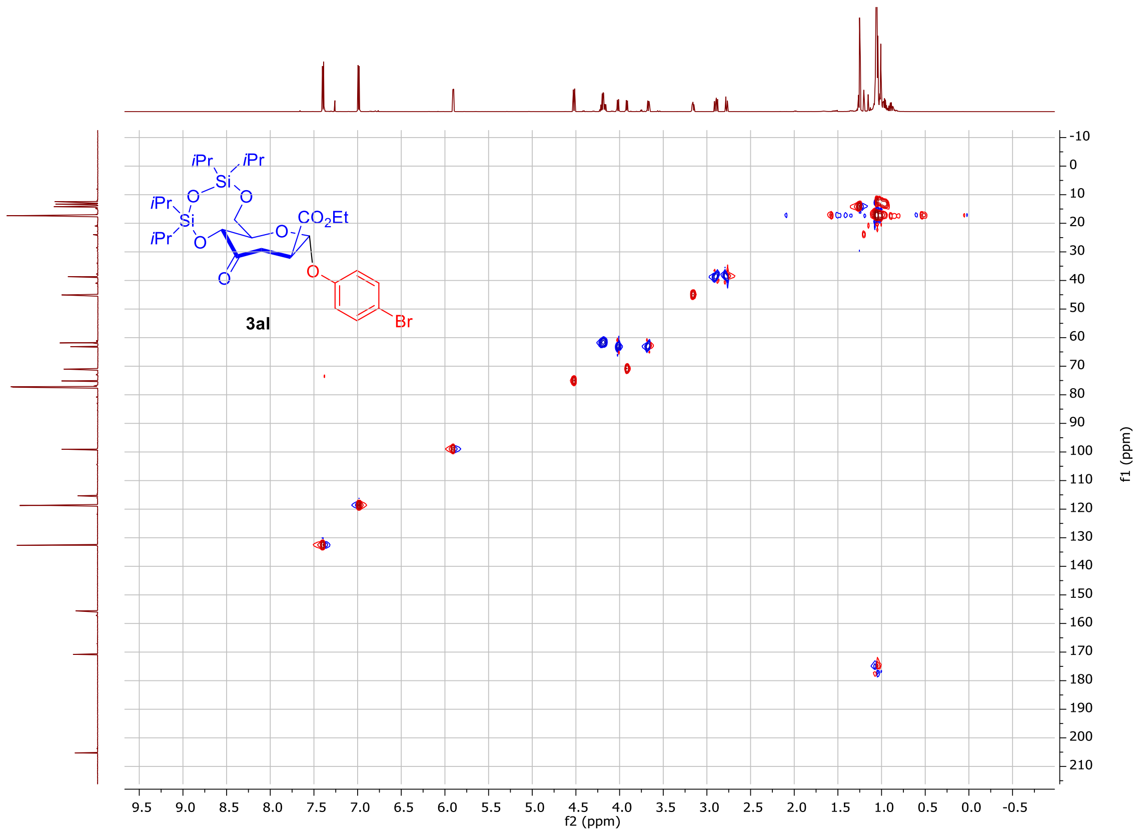

Supplementary Figure 368: HSQC spectra for compound **3al**

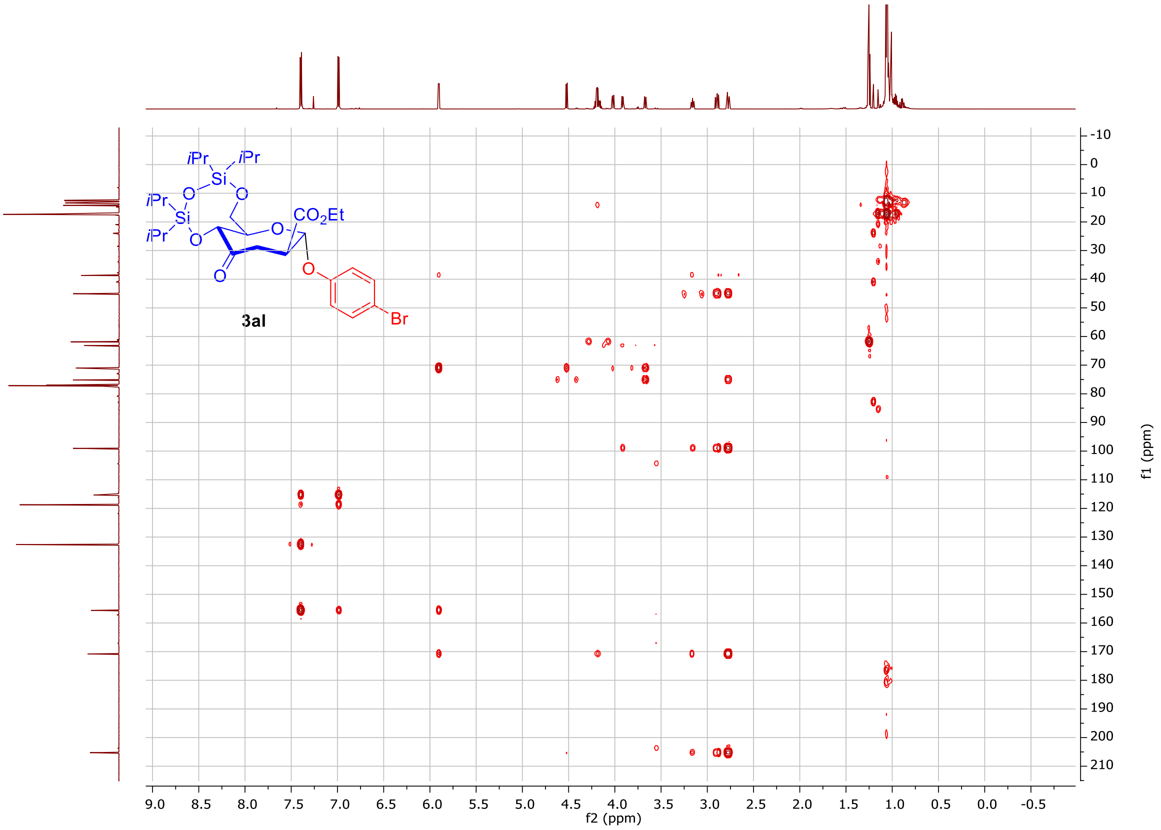

Supplementary Figure 369: HMBC spectra for compound **3al**

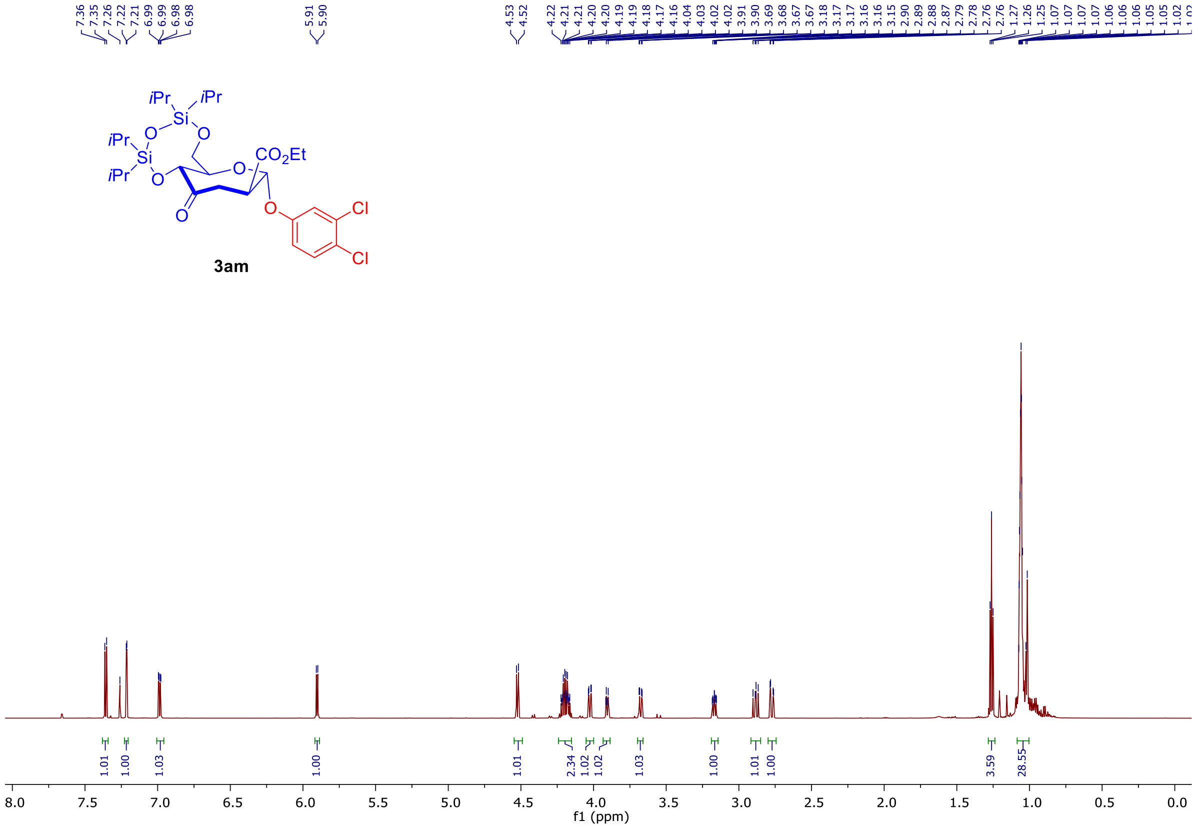

Supplementary Figure 370: ^{1}H spectra for **3am**

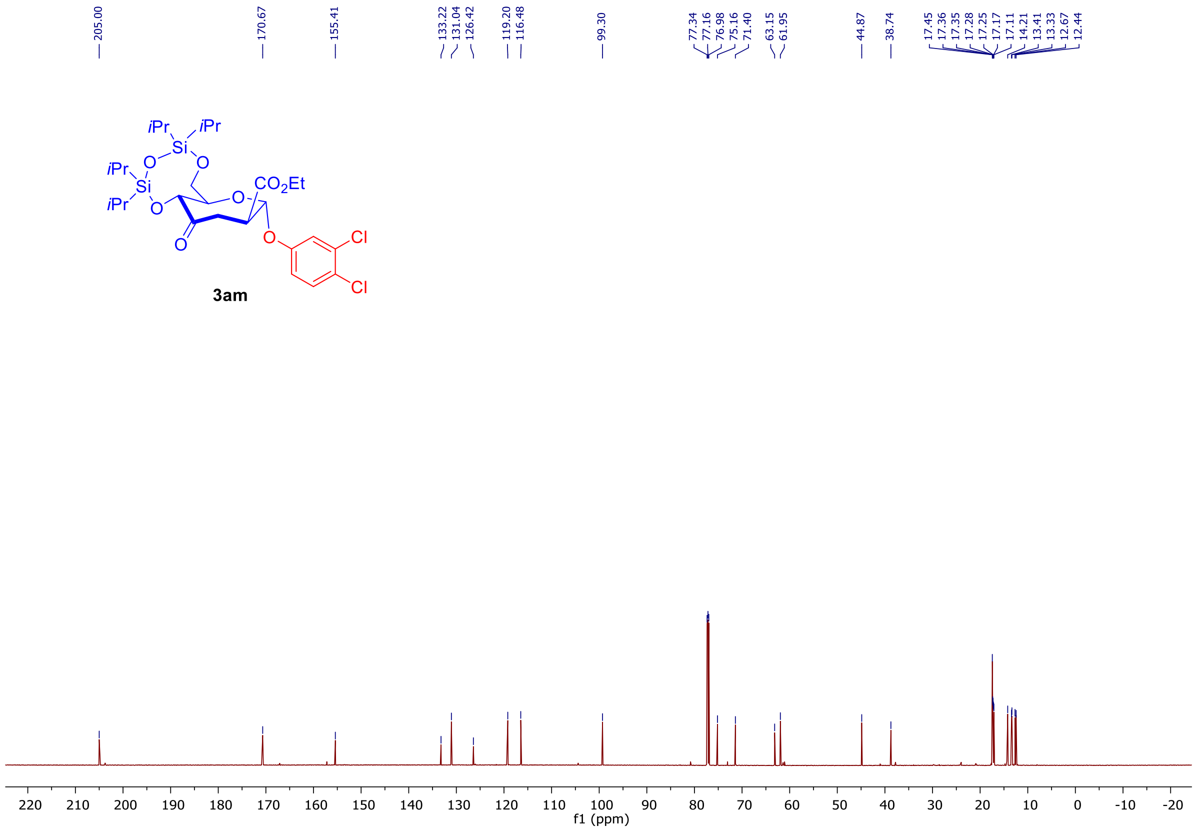

Supplementary Figure 371: ^{13}C spectra for **3am**

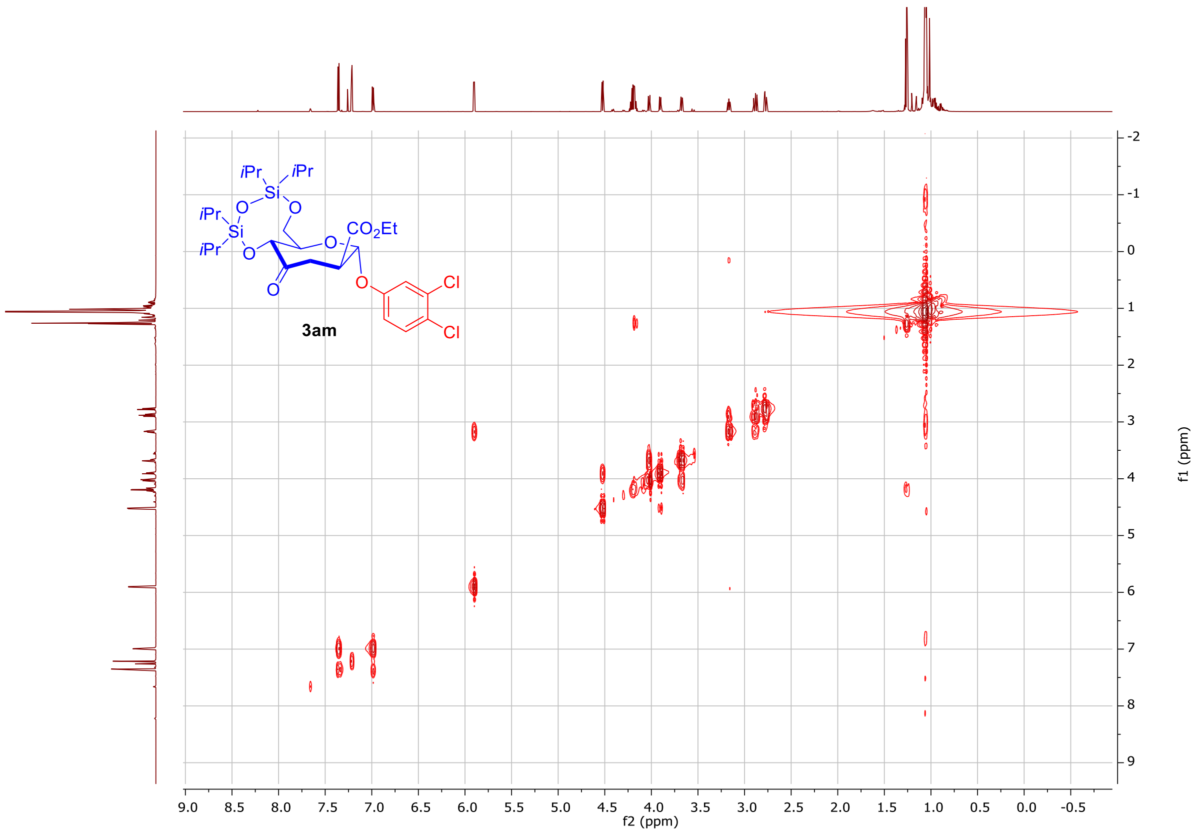

Supplementary Figure 372: COSY spectra for compound **3am**

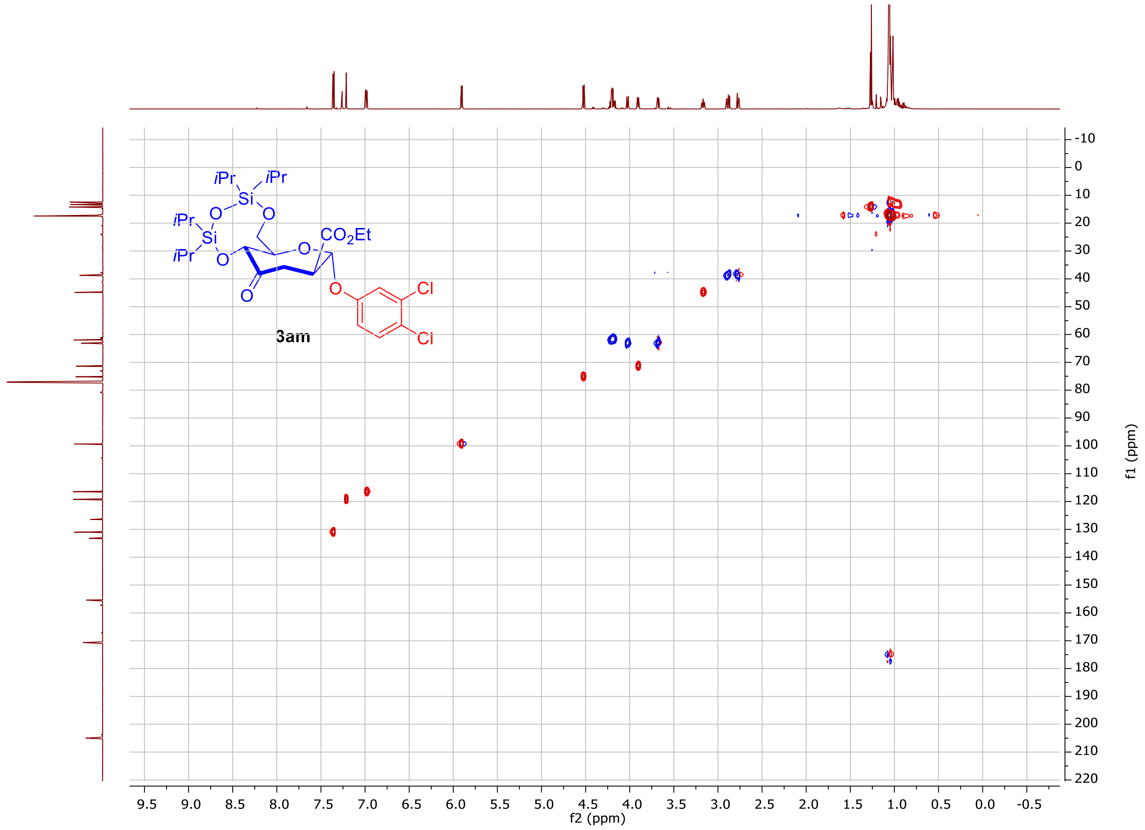

Supplementary Figure 373: HSQC spectra for compound **3am**

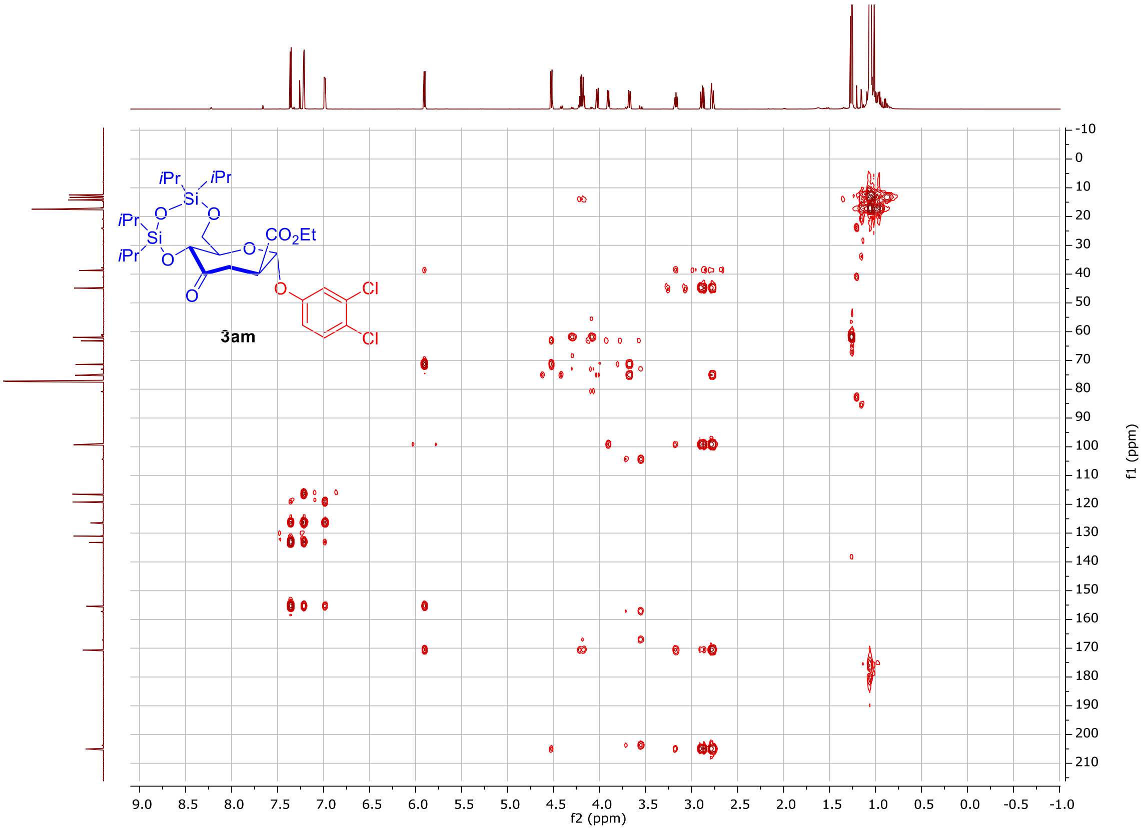

Supplementary Figure 374: HMBC spectra for compound **3am**

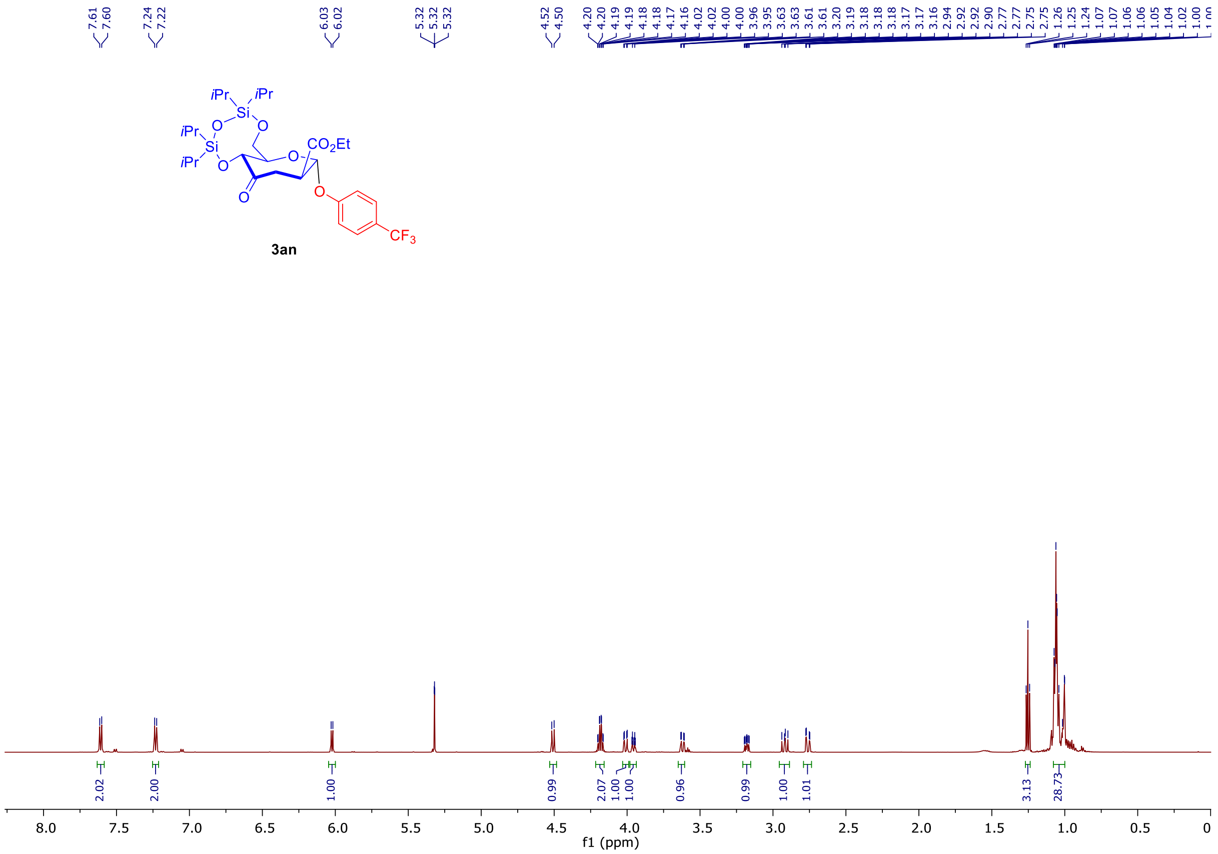

Supplementary Figure 375: ^{1}H spectra for compound **3an**

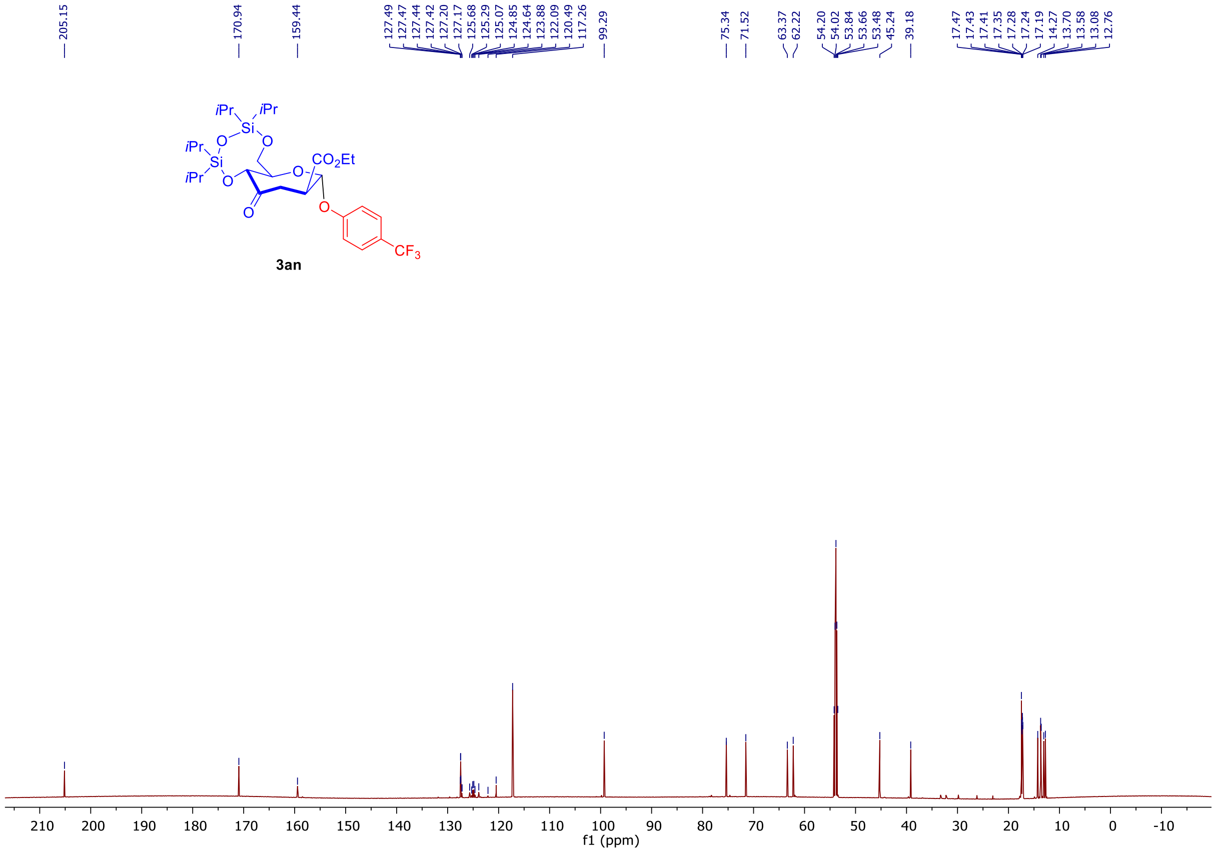

Supplementary Figure 376: ^{13}C spectra for compound **3an**

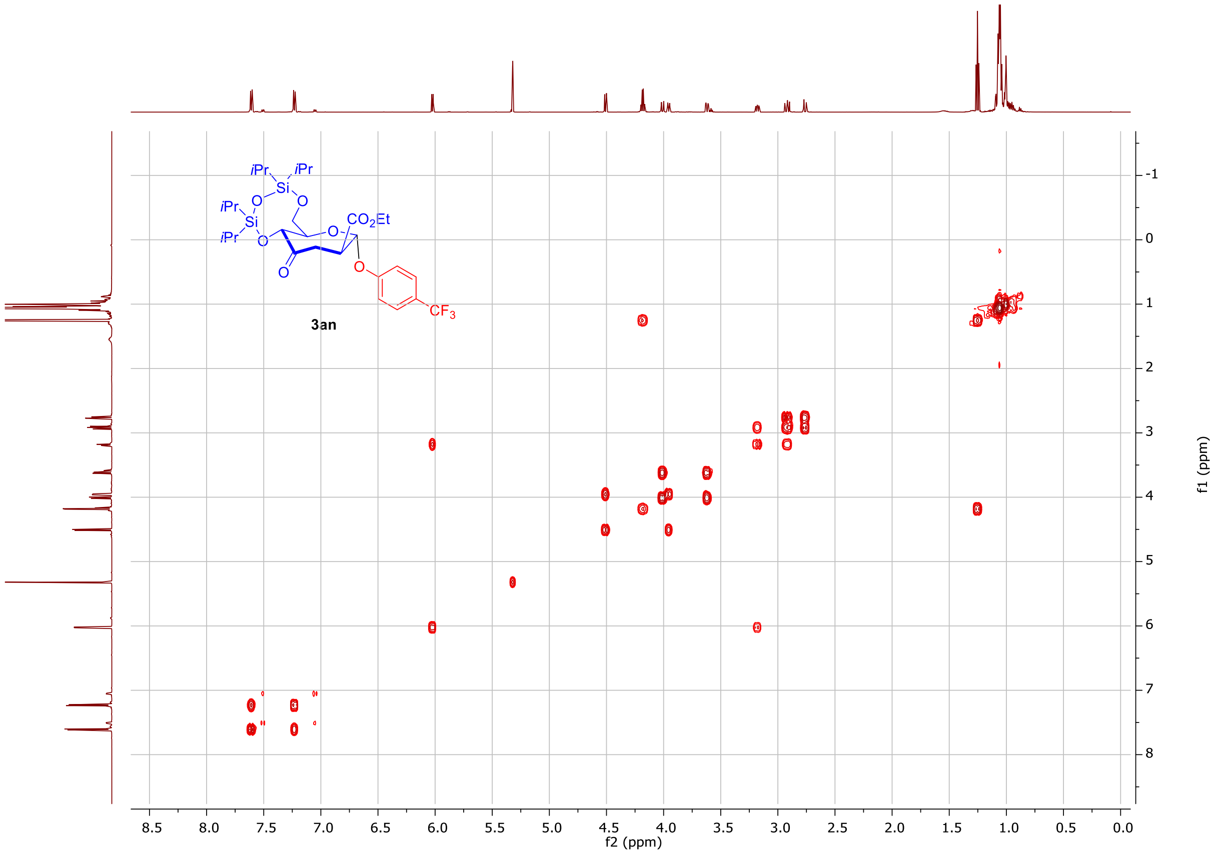

Supplementary Figure 377: COSY spectra for compound **3an**

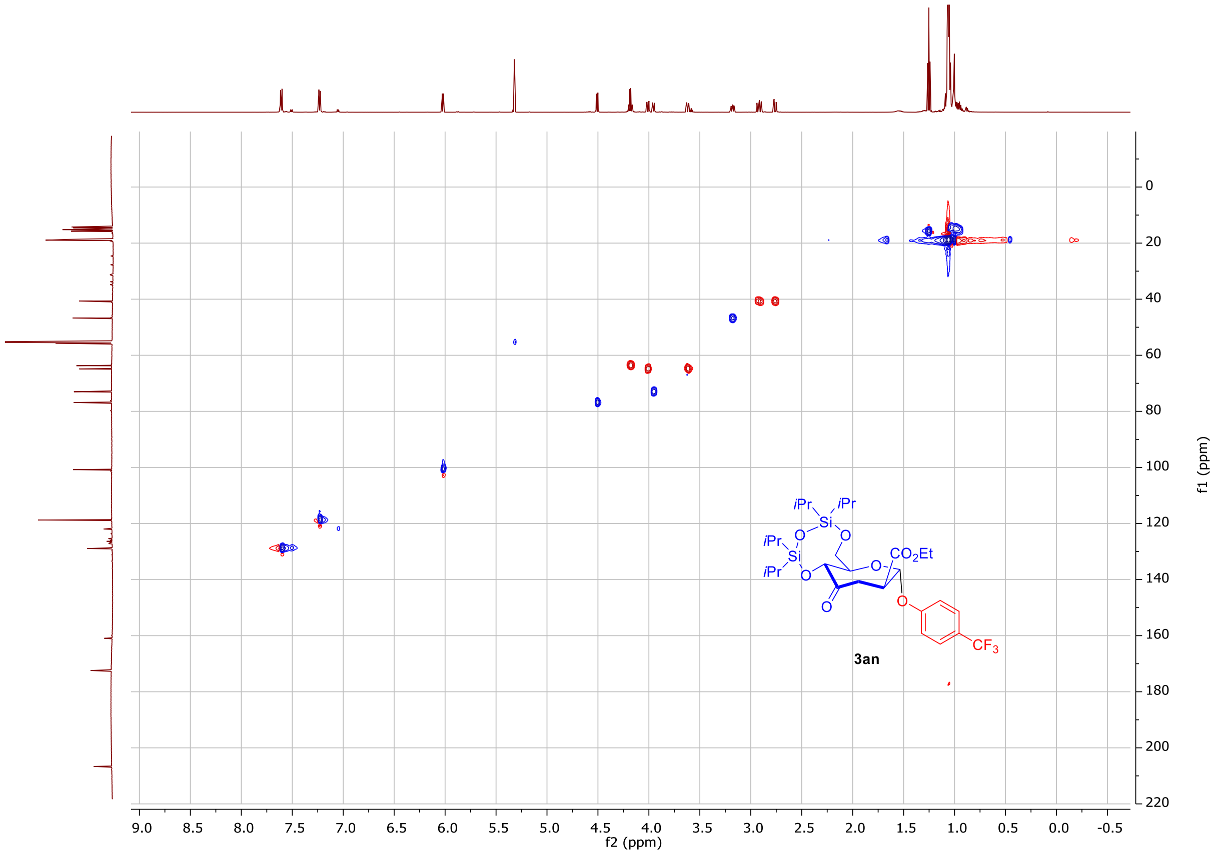

Supplementary Figure 378: HSQC spectra for compound **3an**

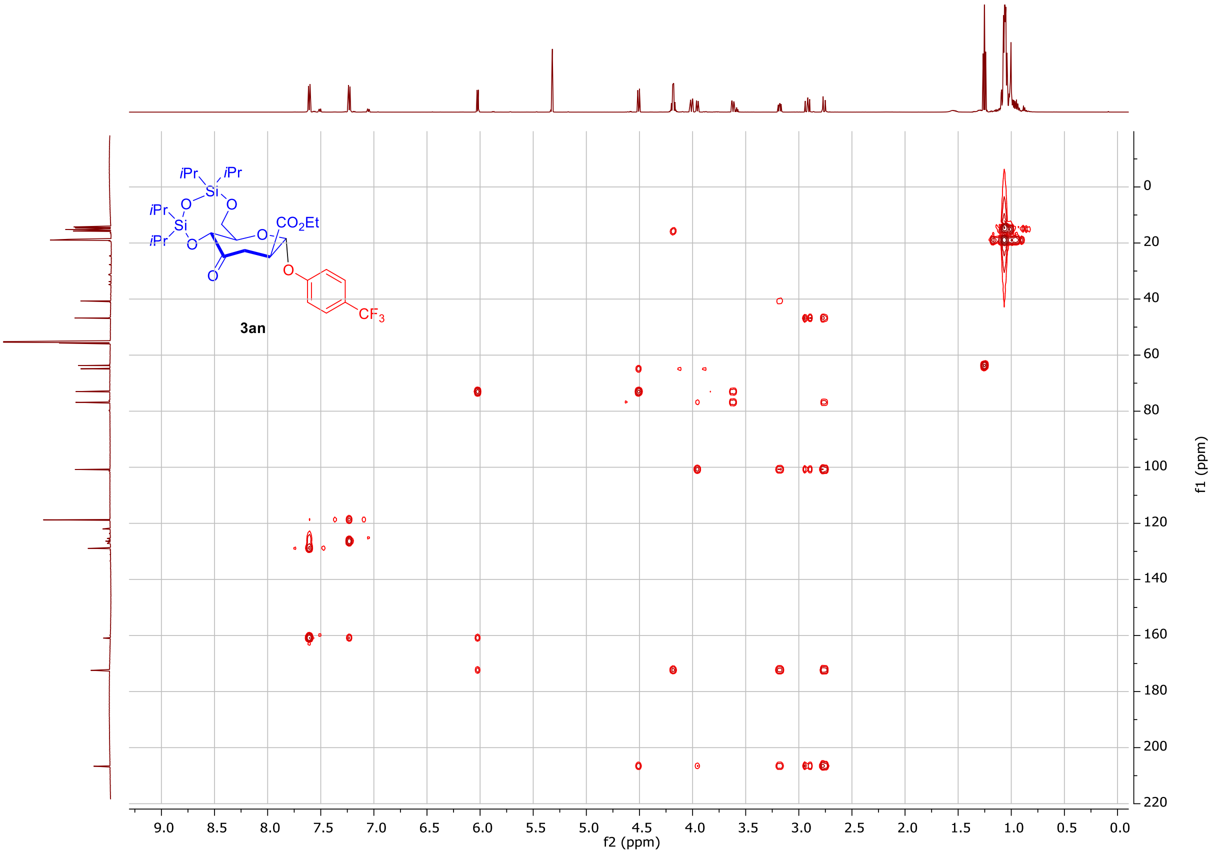

Supplementary Figure 379: HMBC spectra for compound **3an**

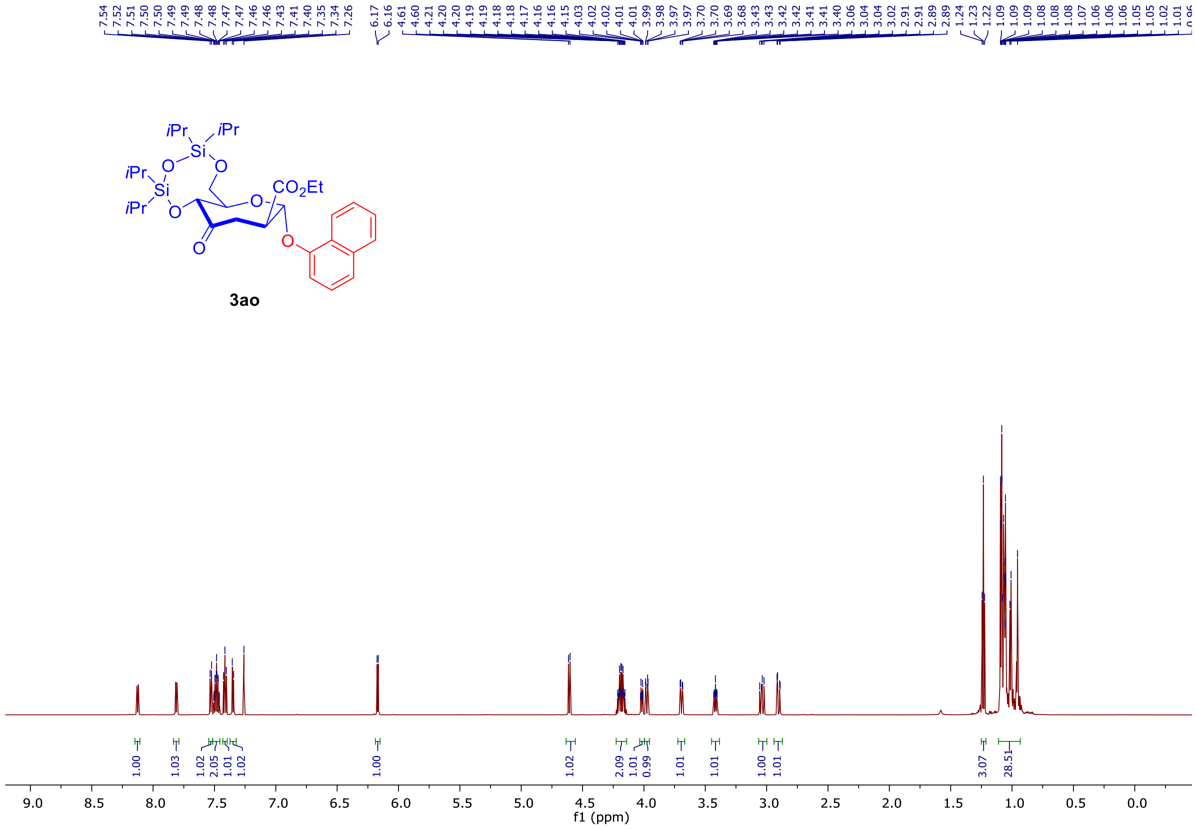

Supplementary Figure 380: ^{1}H spectra for **3ao**

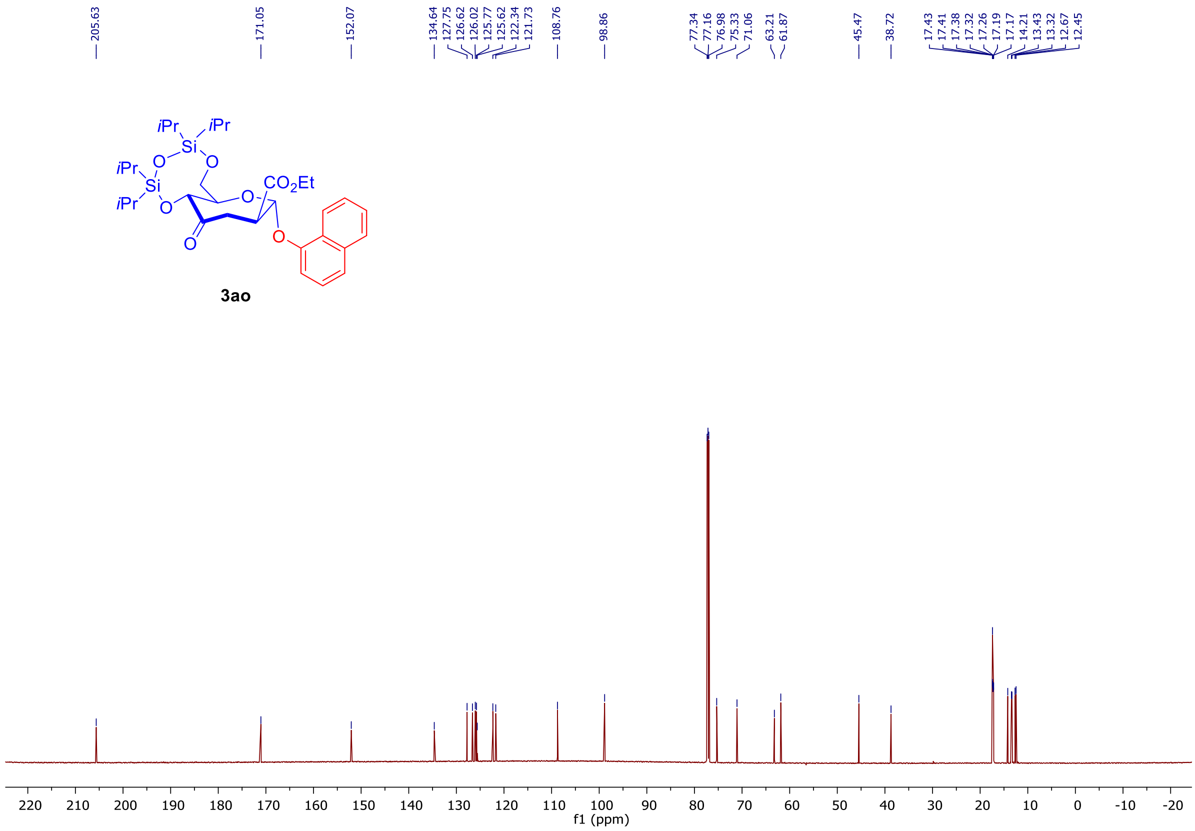

Supplementary Figure 381: ^{13}C spectra for **3ao**

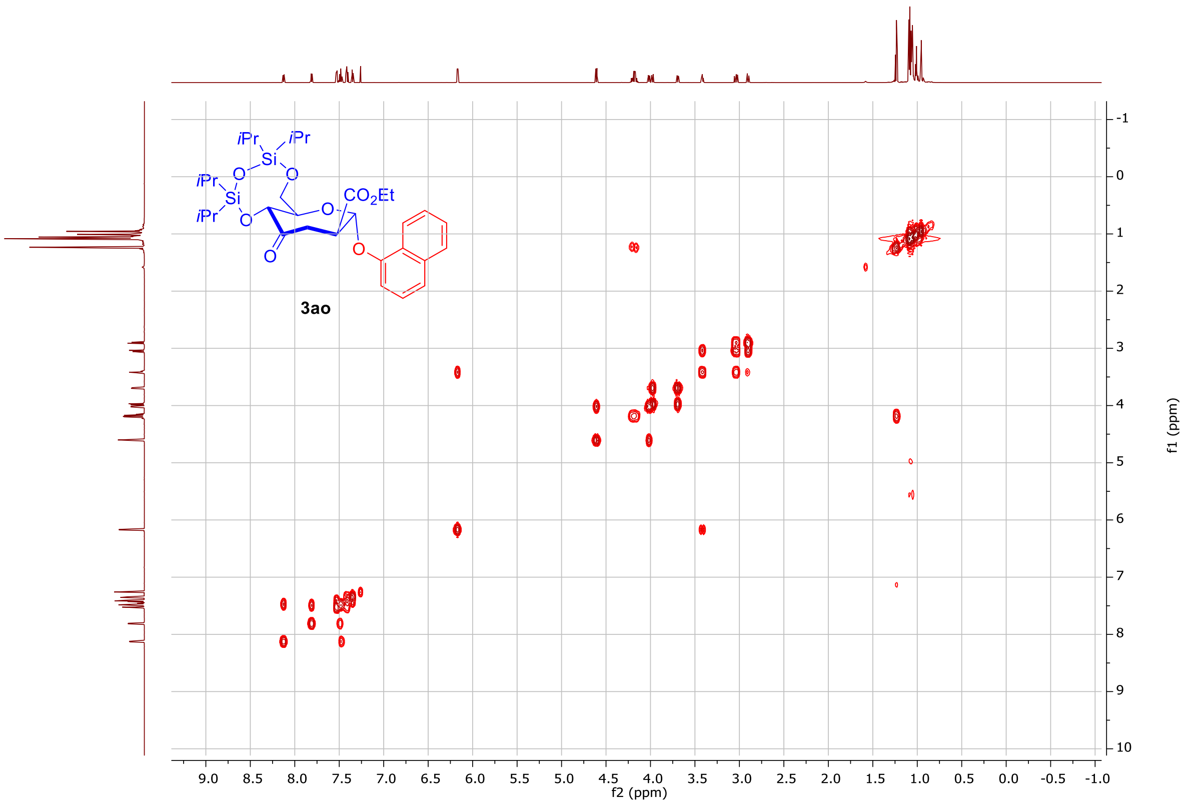

Supplementary Figure 382: COSY spectra for compound **3ao**

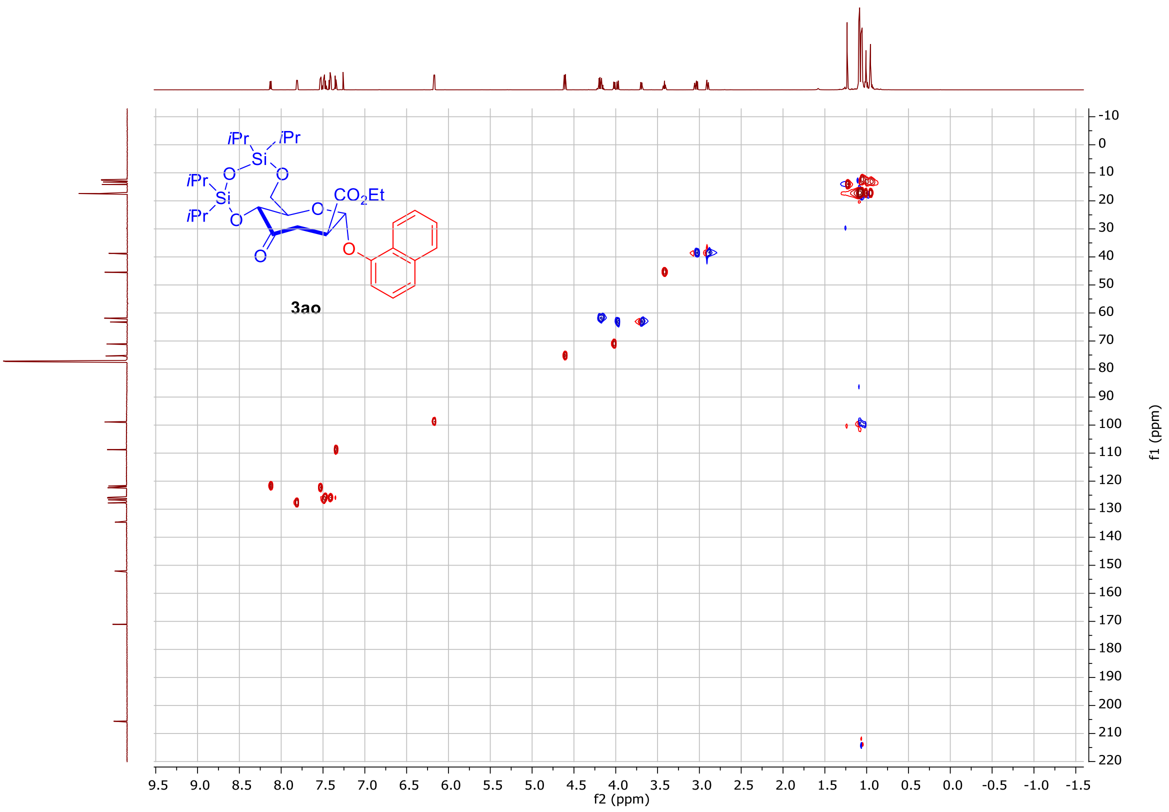

Supplementary Figure 383: HSQC spectra for compound **3ao**

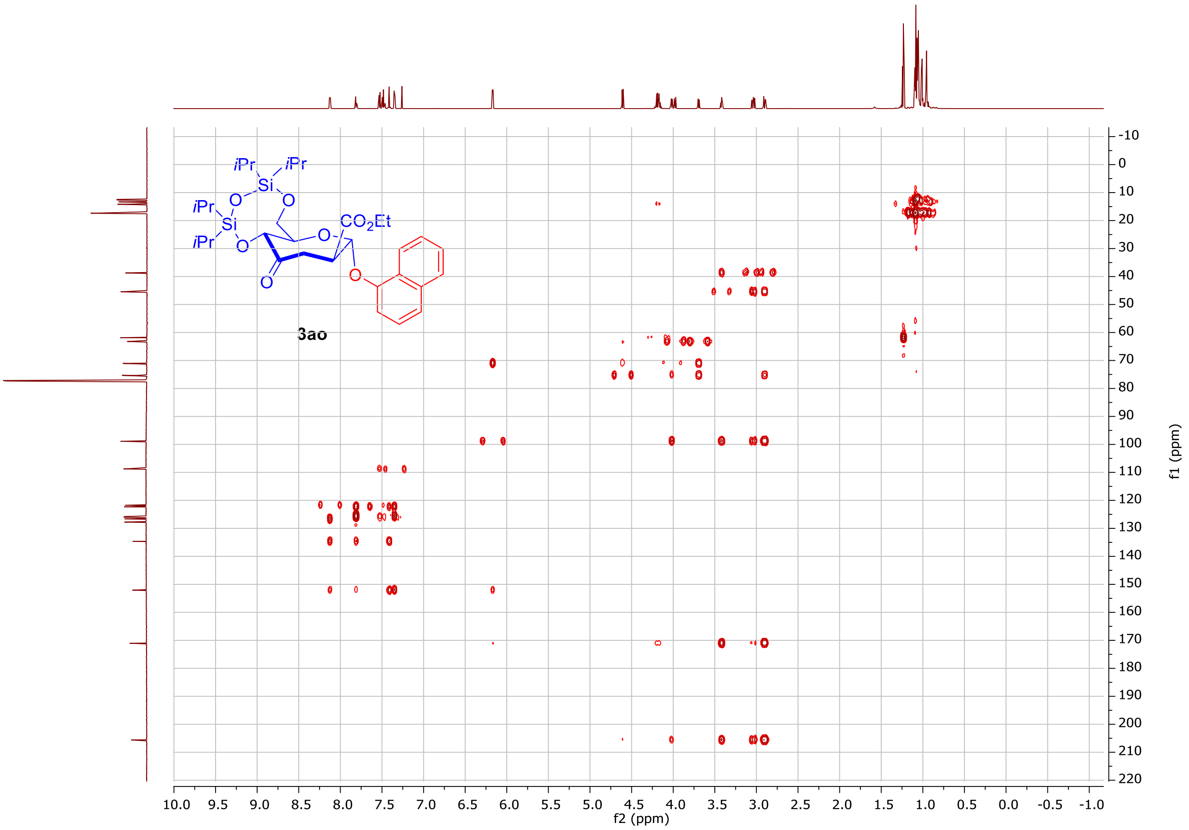

Supplementary Figure 384: HMBC spectra for compound **3ao**

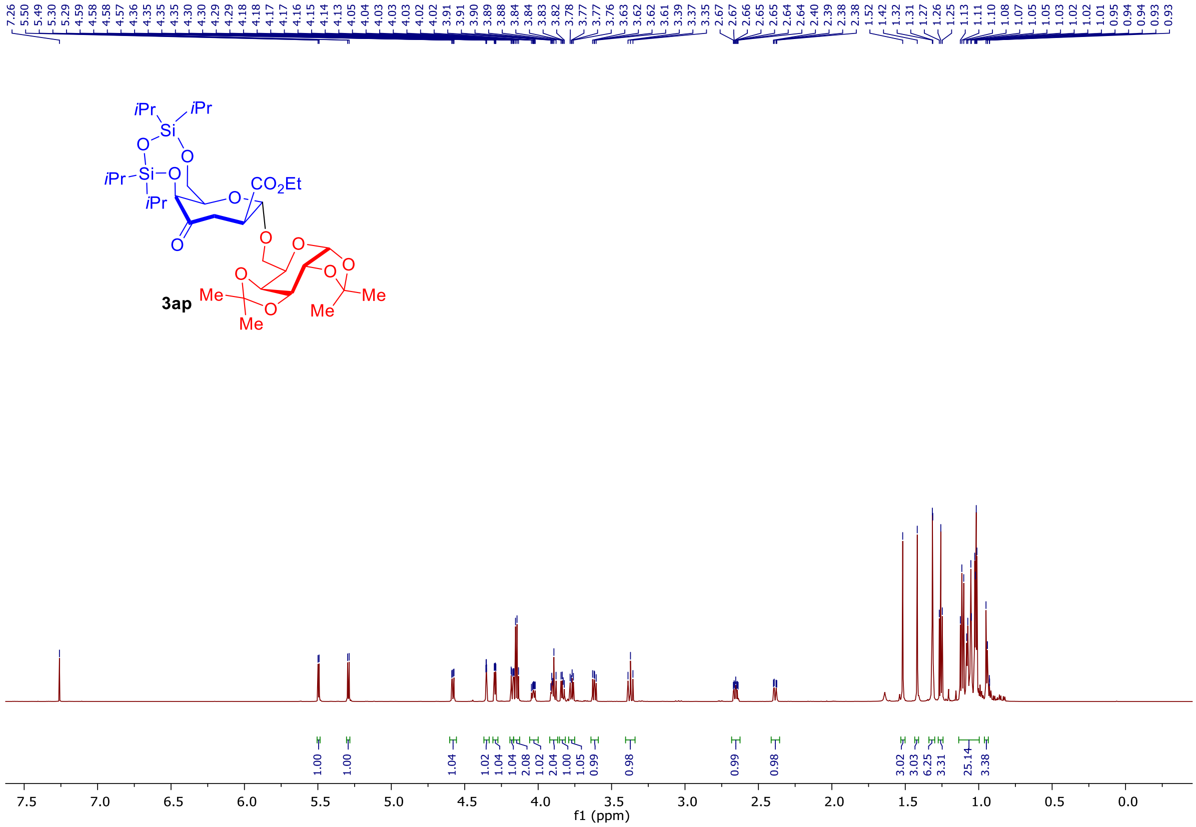

Supplementary Figure 385: ^{1}H spectra for **3ap**

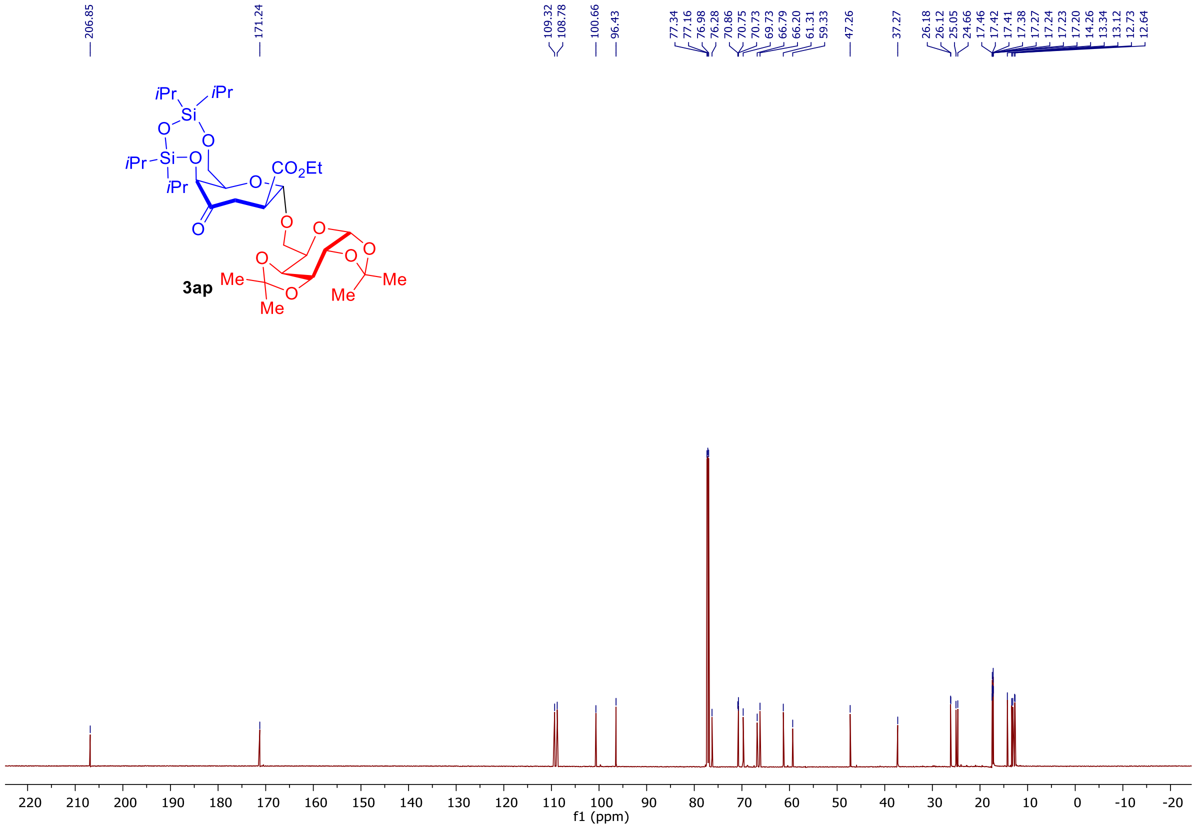

Supplementary Figure 386: ^{13}C spectra for **3ap**

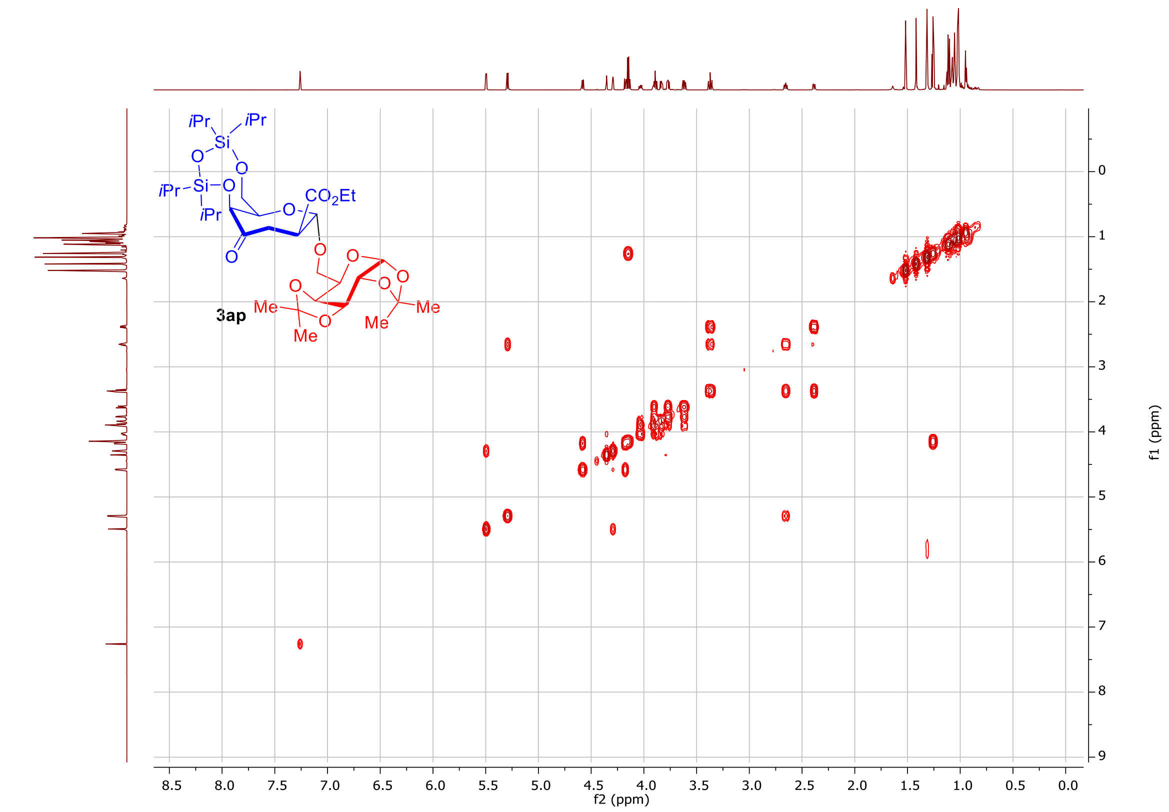

Supplementary Figure 387: COSY spectra for compound **3ap**

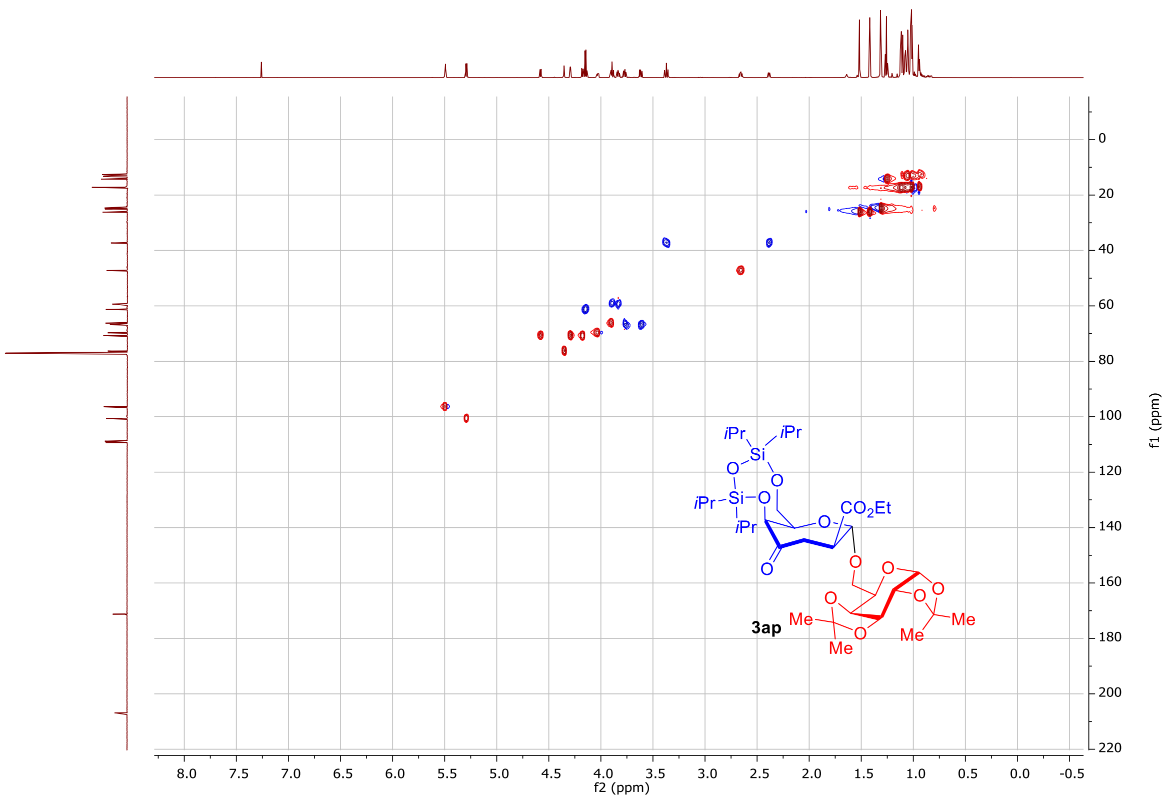

Supplementary Figure 388: HSQC spectra for compound **3ap**

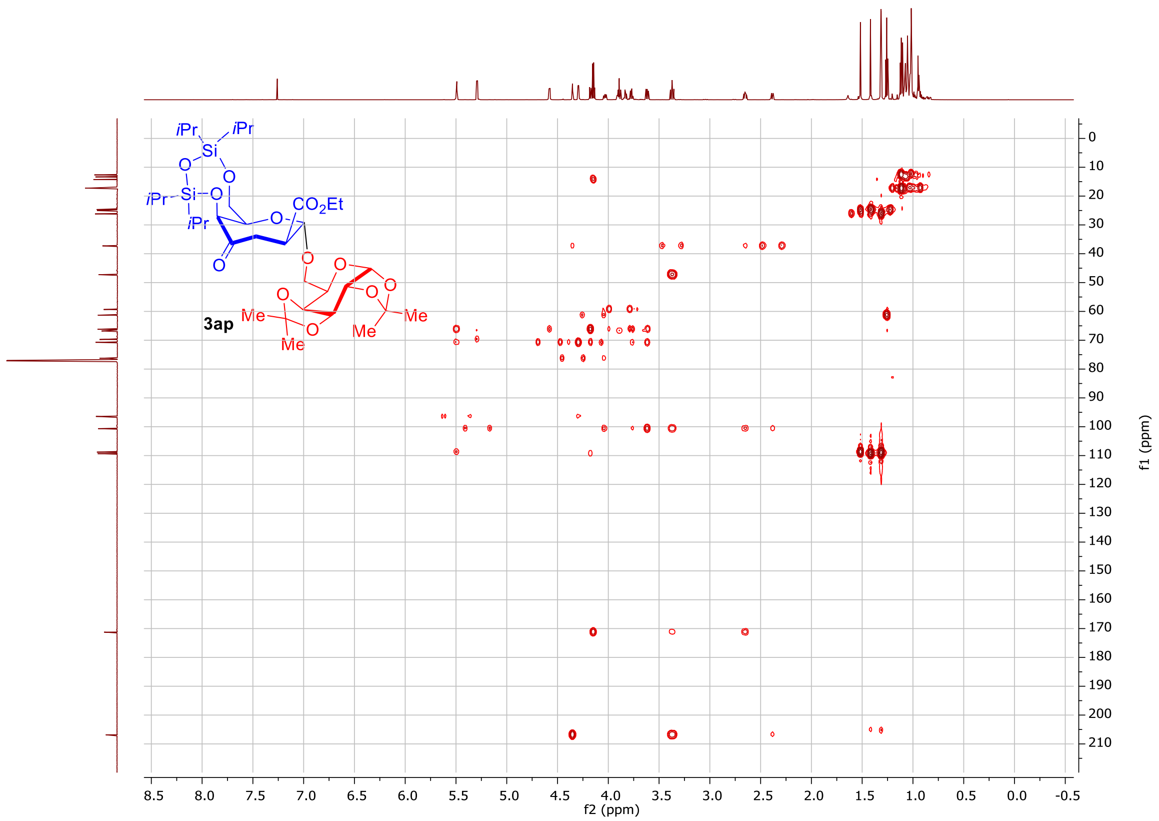

Supplementary Figure 389: HMBC spectra for compound **3ap**

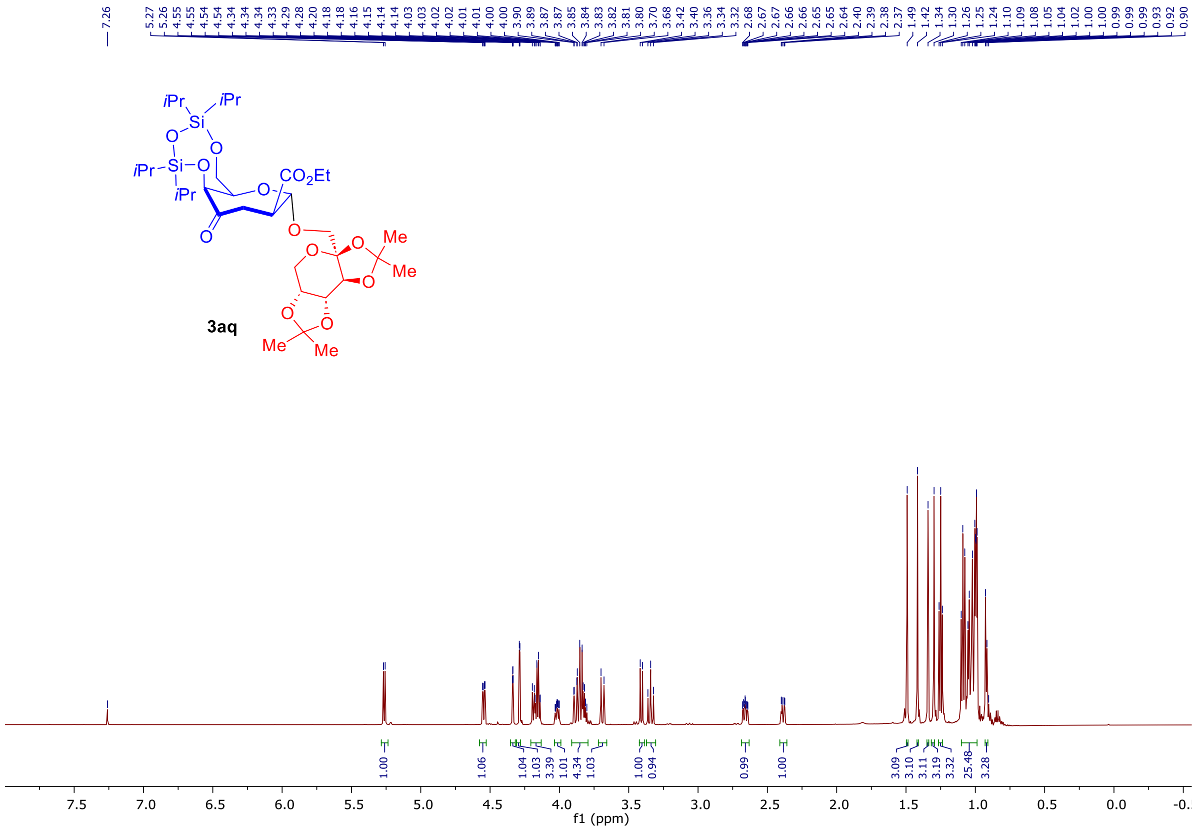

Supplementary Figure 390: ^{1}H spectra for **3aq**

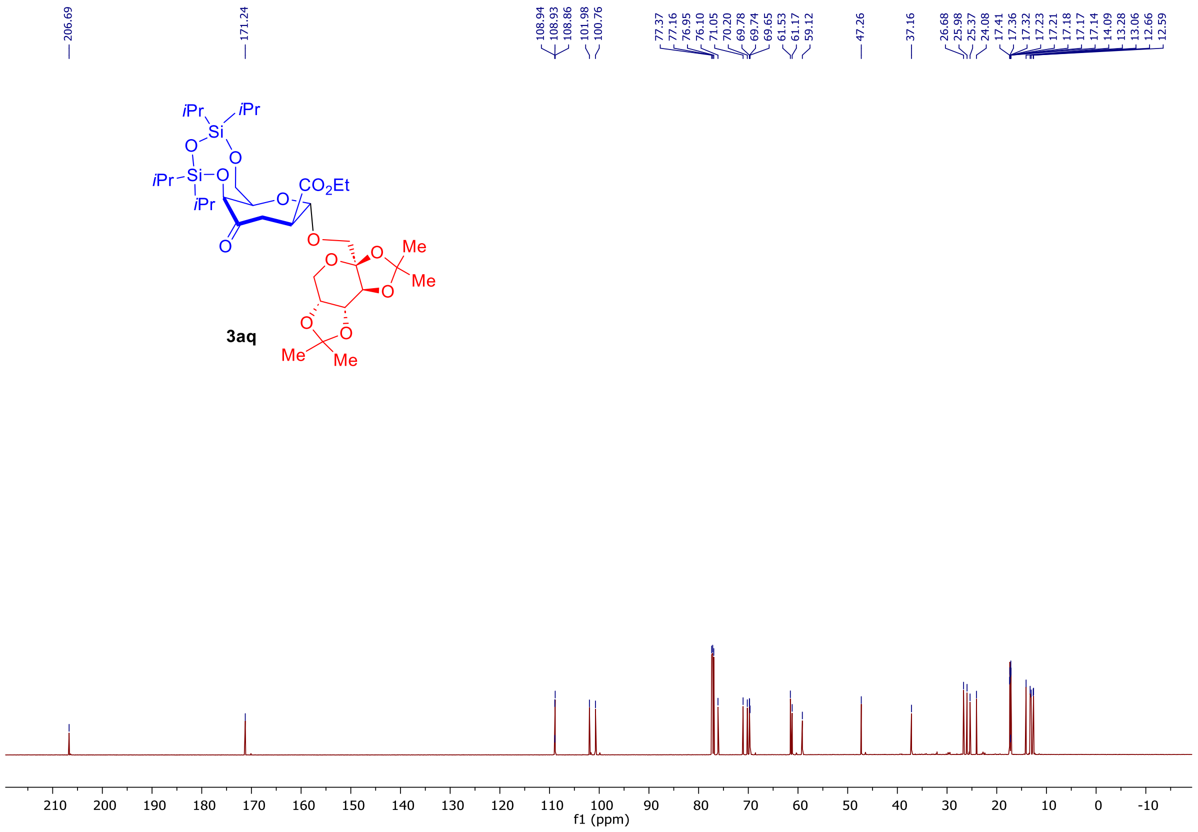

Supplementary Figure 391: ^{13}C spectra for **3aq**

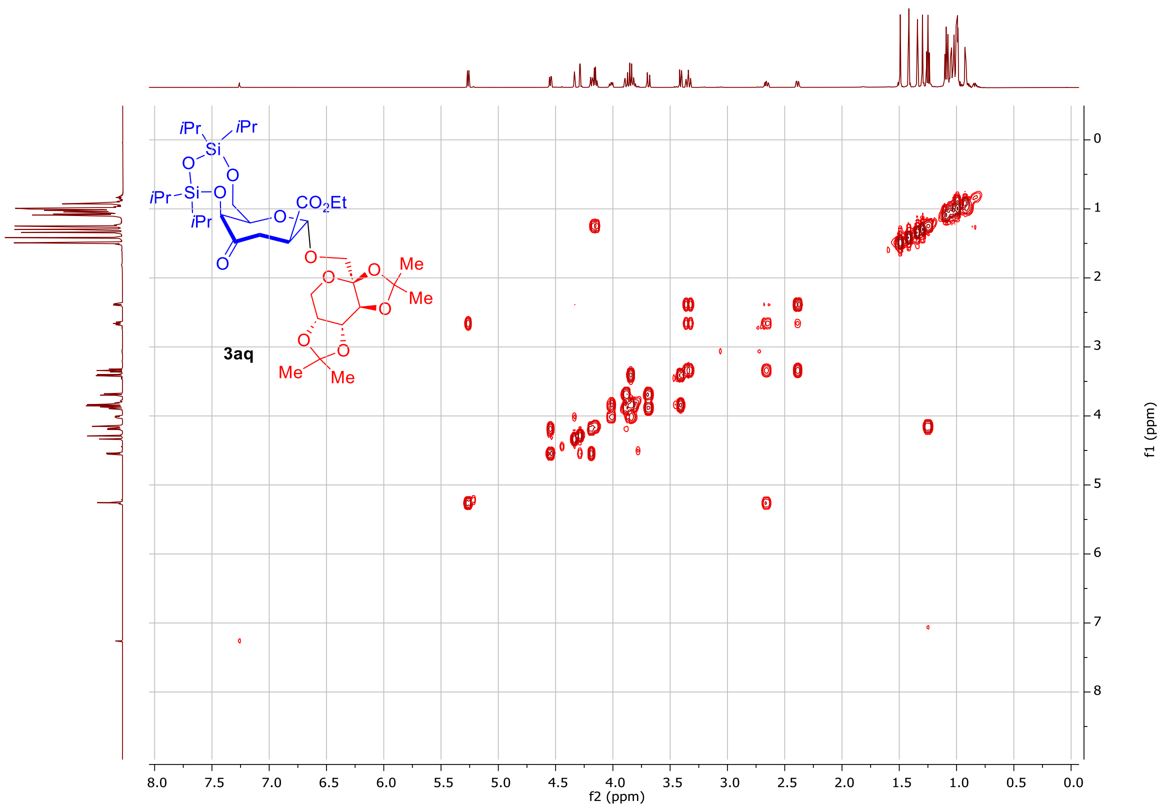

Supplementary Figure 392: COSY spectra for compound **3aq**

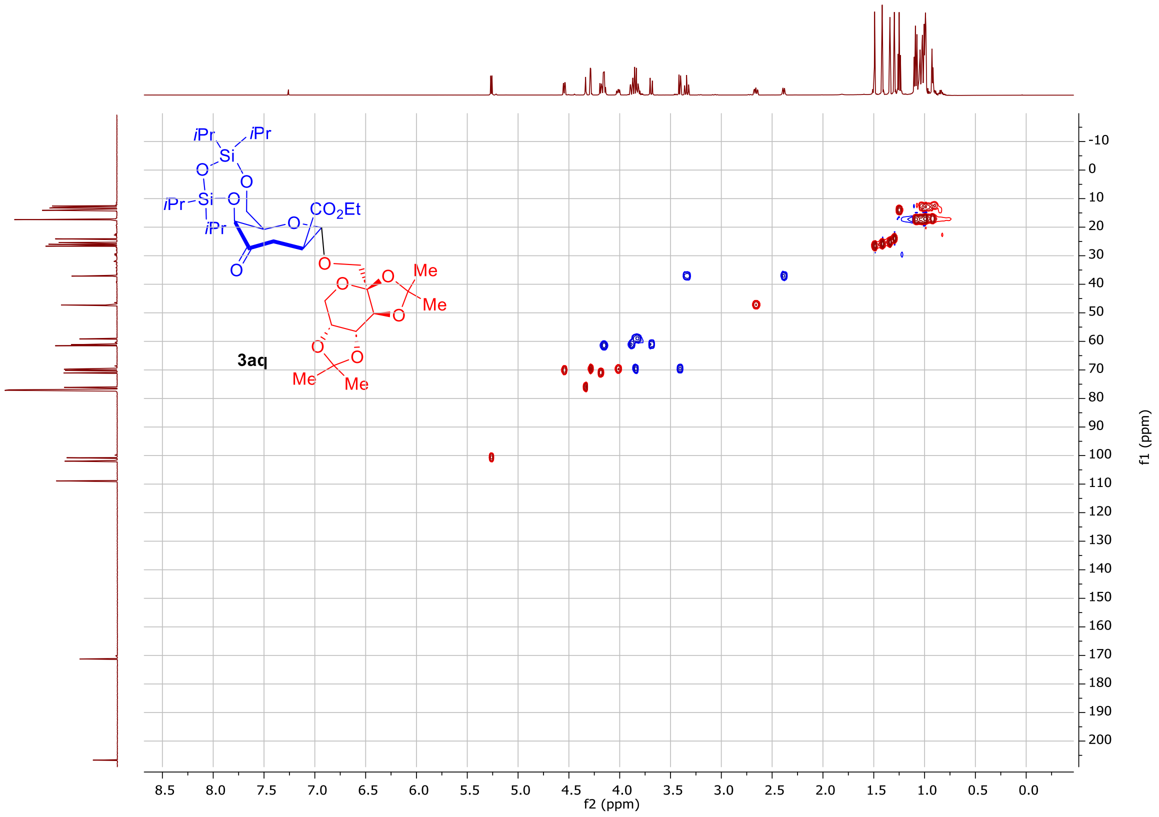

Supplementary Figure 393: HSQC spectra for compound **3aq**

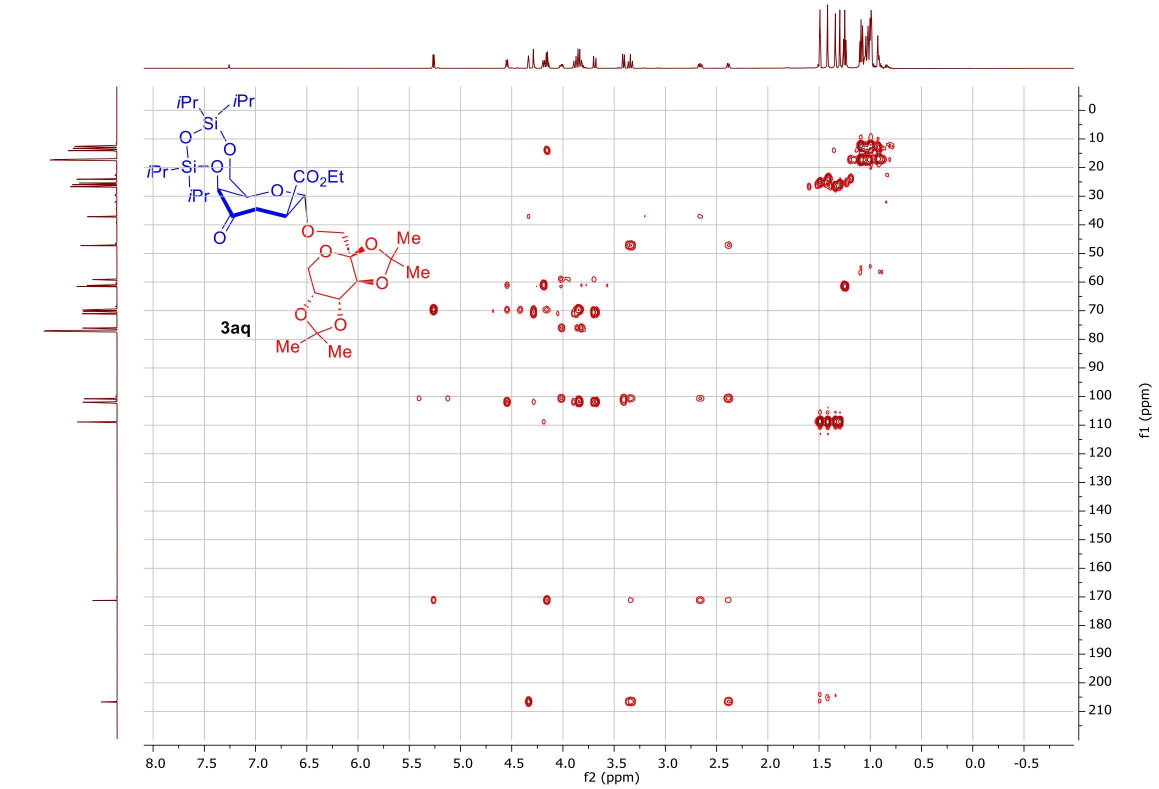

Supplementary Figure 394: HMBC spectra for compound **3aq**

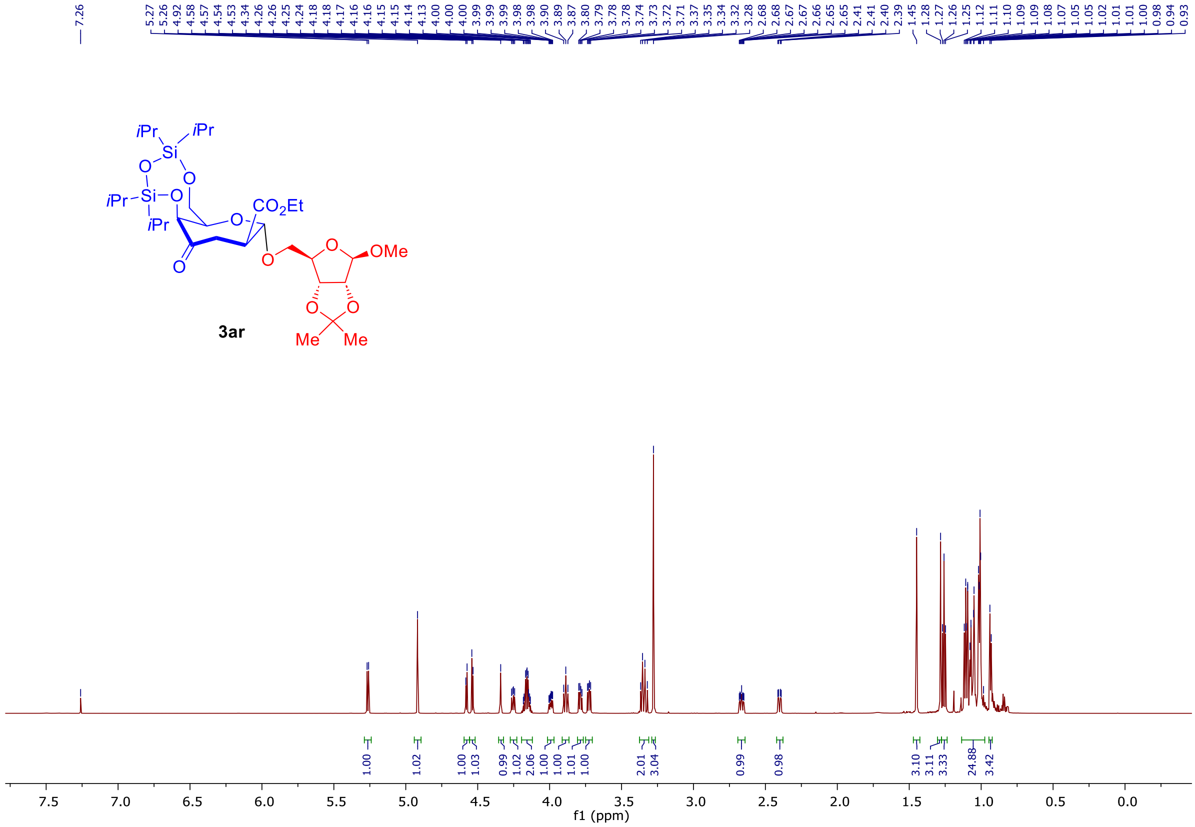

Supplementary Figure 395: ^{1}H spectra for **3ar**

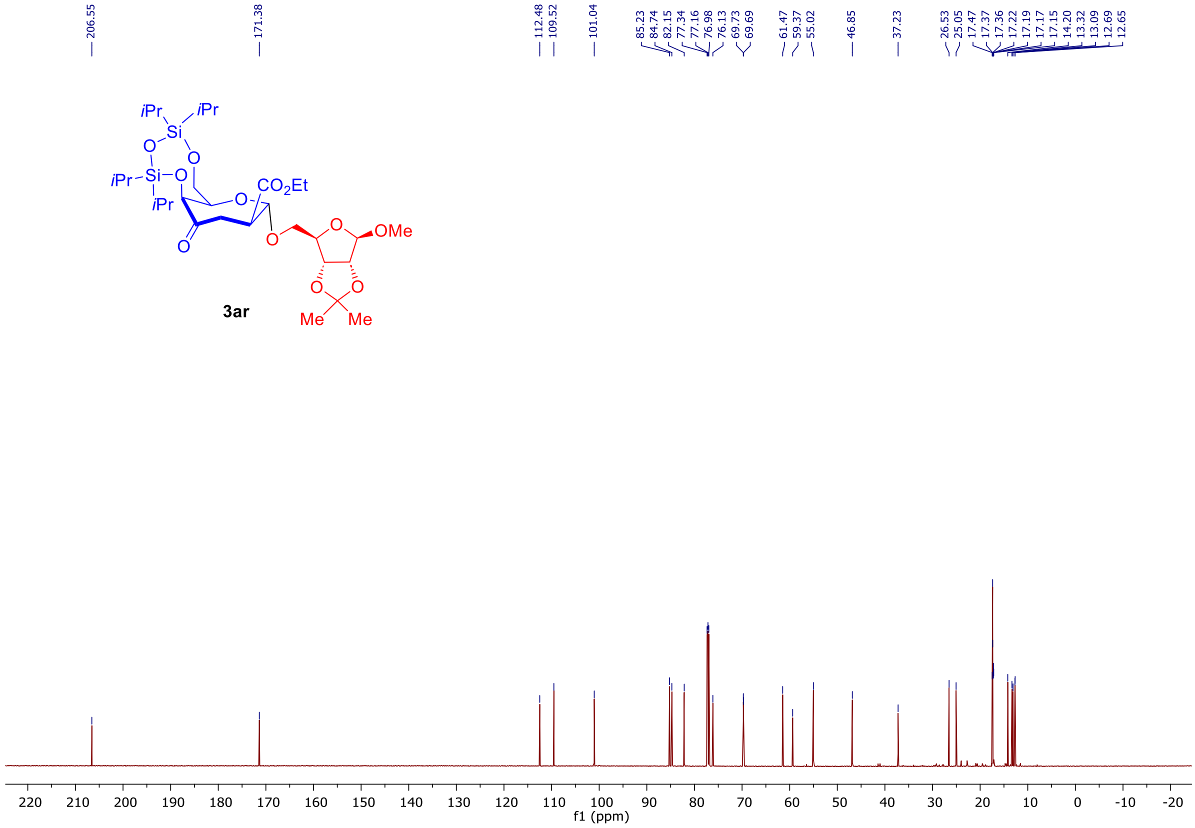

Supplementary Figure 396: ^{13}C spectra for **3ar**

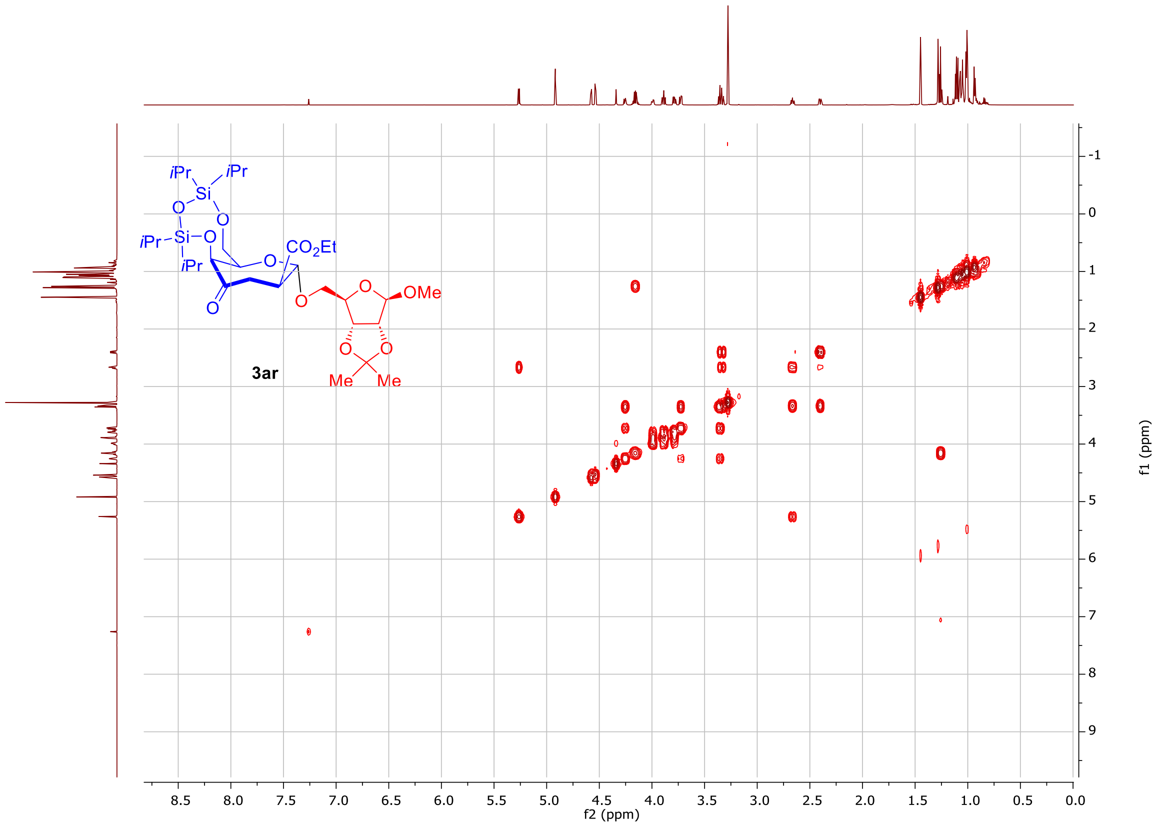

Supplementary Figure 397: COSY spectra for compound **3ar**

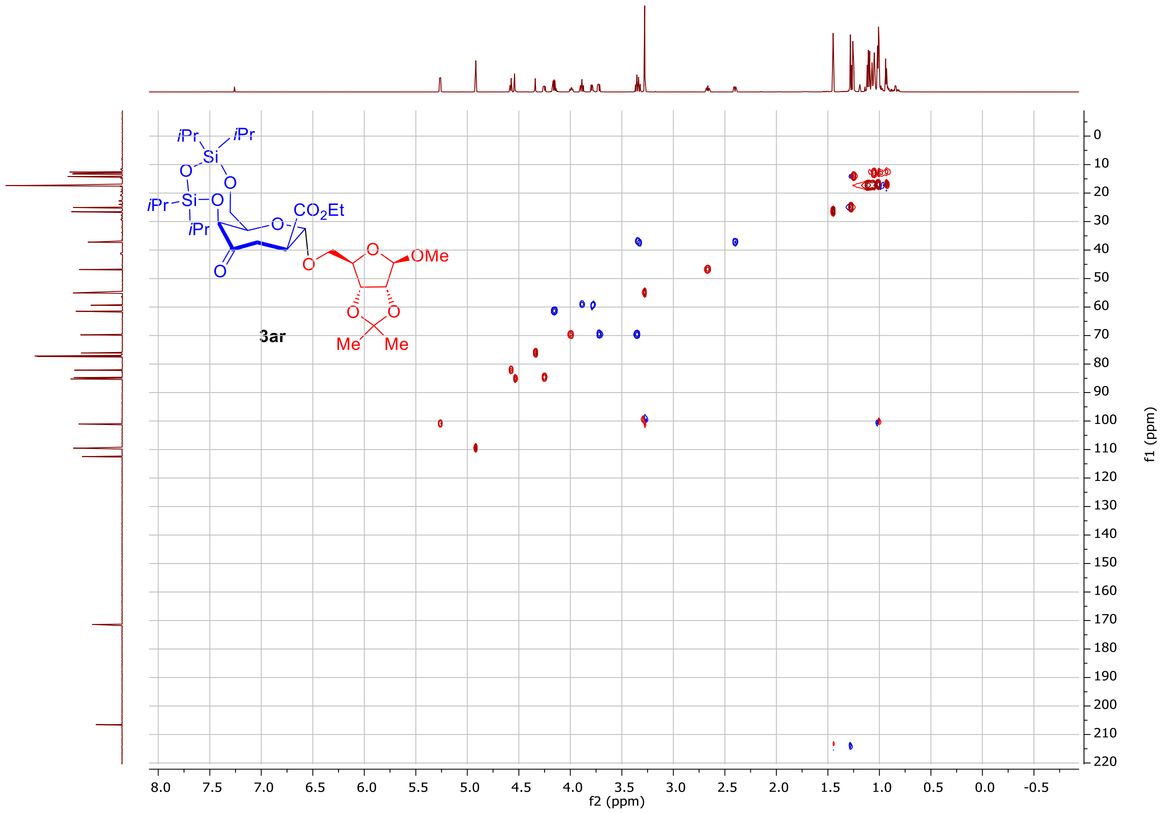

Supplementary Figure 398: HSQC spectra for compound **3ar**

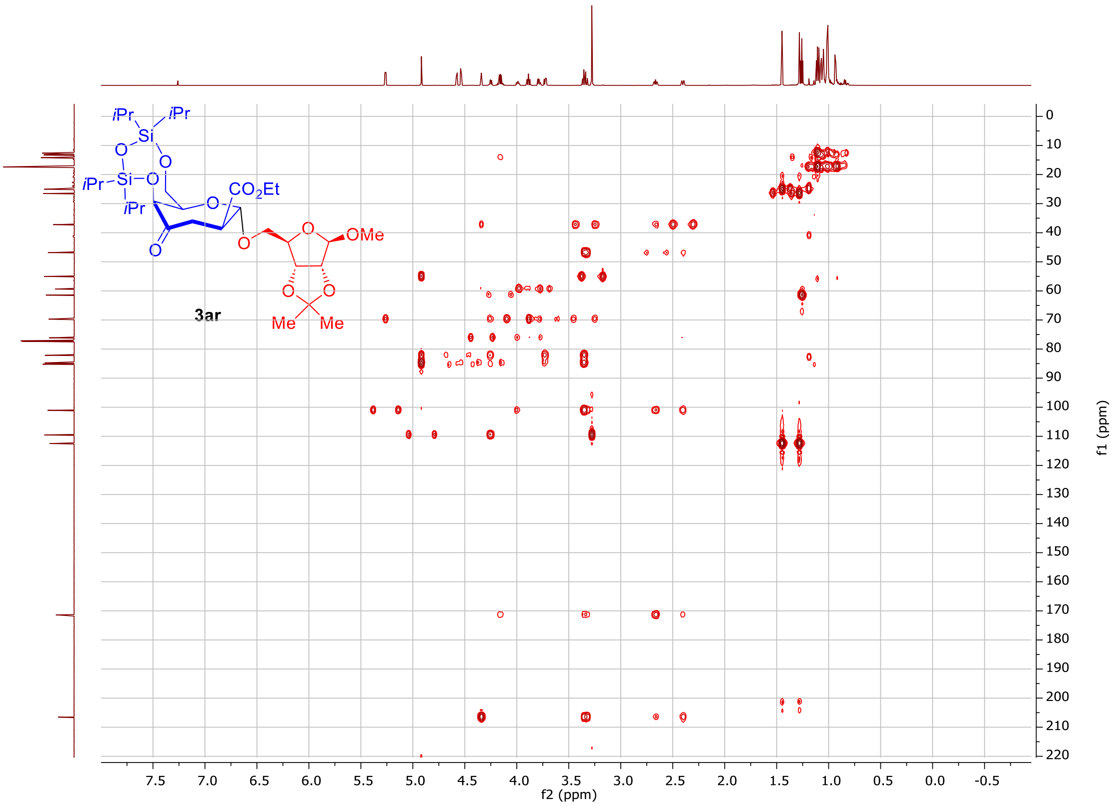

Supplementary Figure 399: HMBC spectra for compound **3ar**

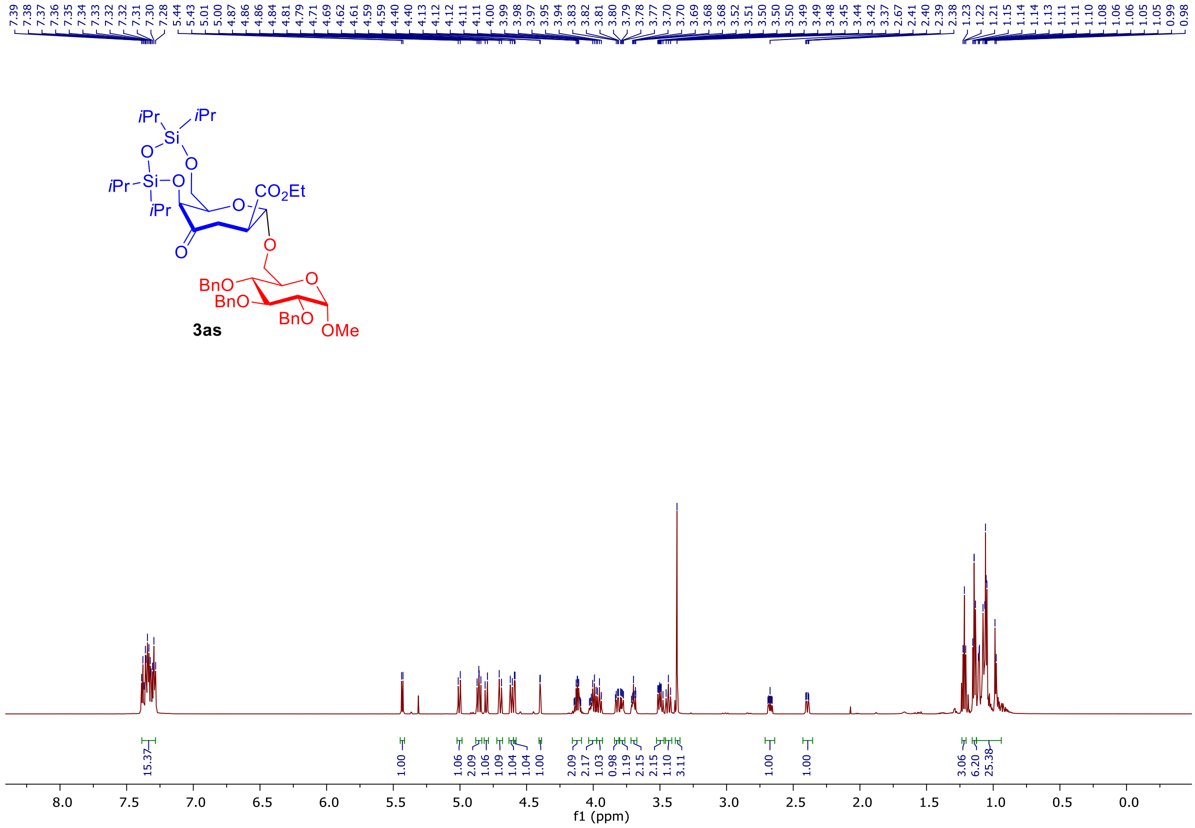

Supplementary Figure 400: ^{1}H spectra for **3as**

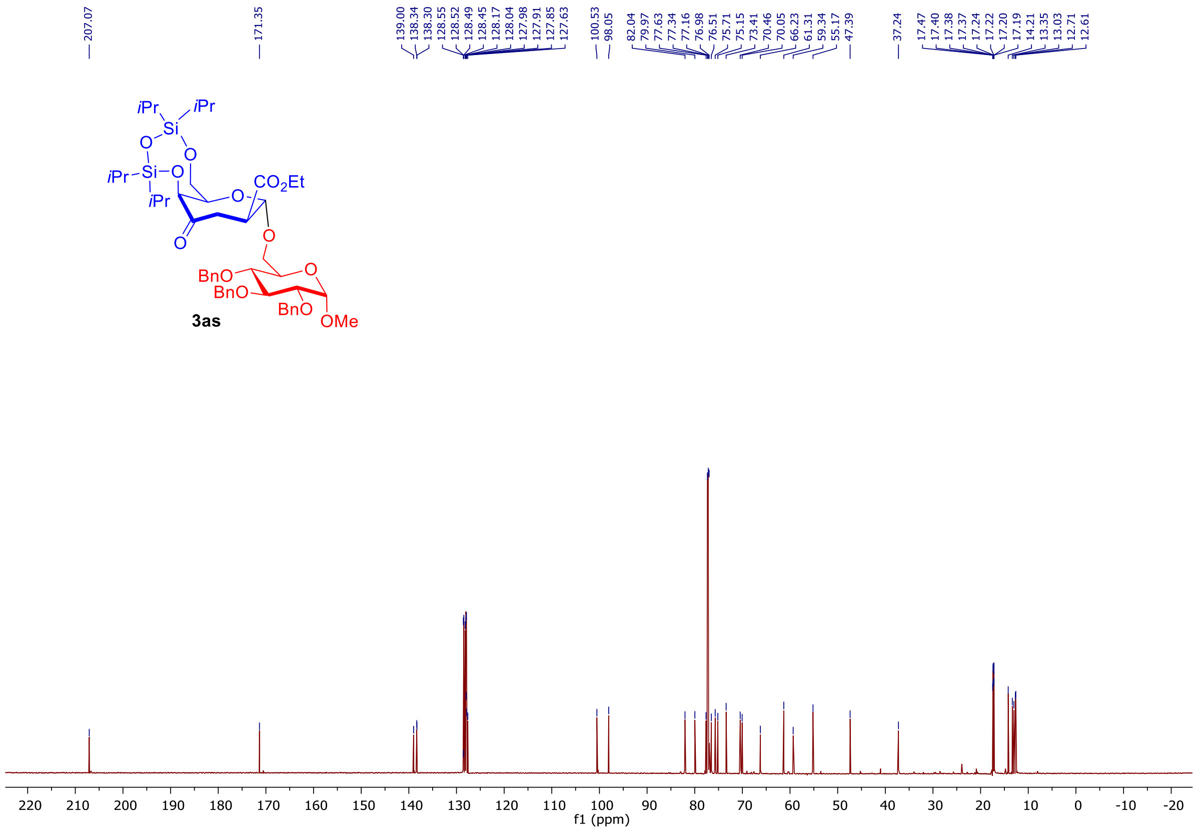

Supplementary Figure 401: ^{13}C spectra for **3as**

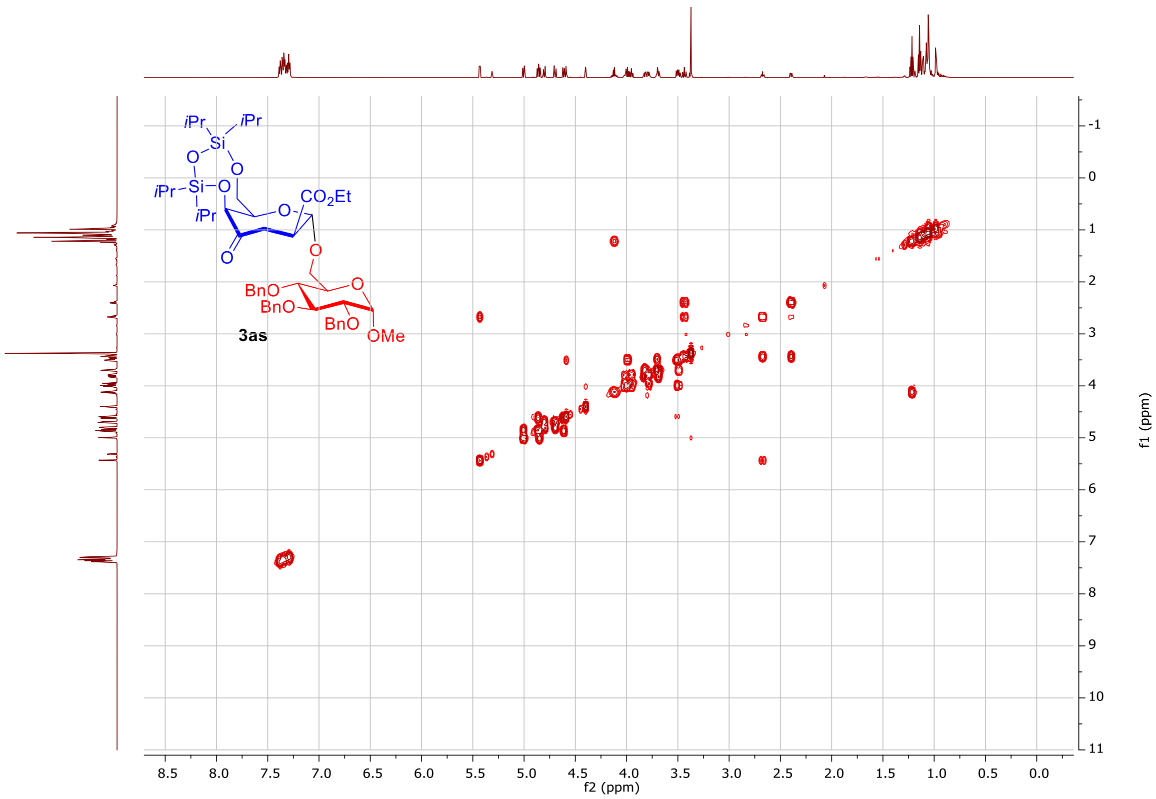

Supplementary Figure 402: COSY spectra for compound **3as**

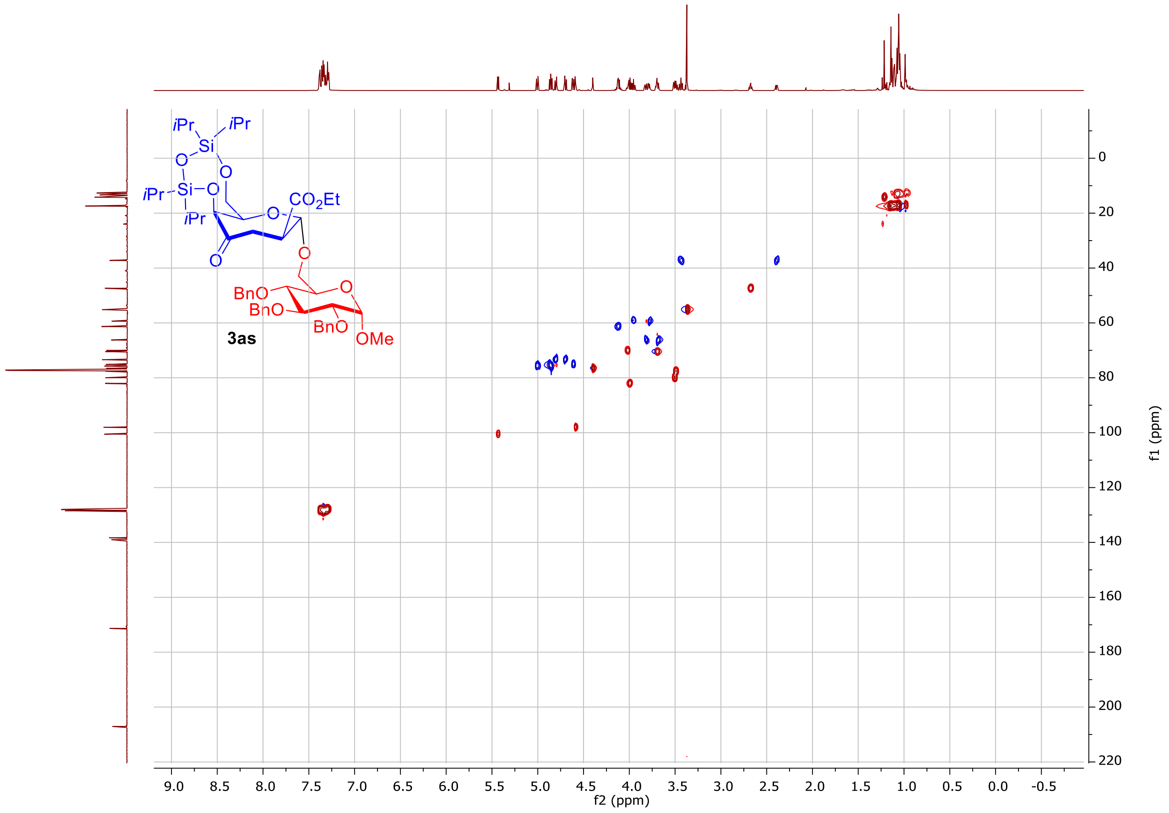

Supplementary Figure 403: HSQC spectra for compound **3as**

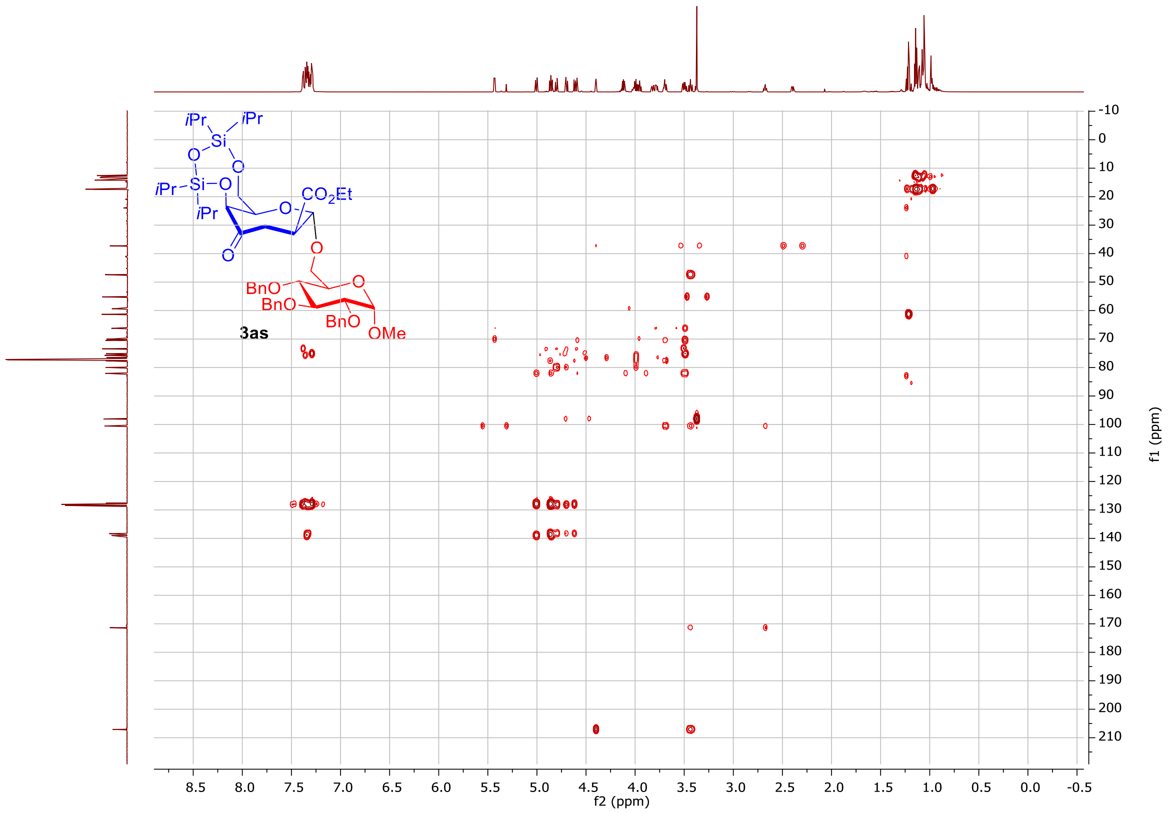

Supplementary Figure 404: HMBC spectra for compound **3as**

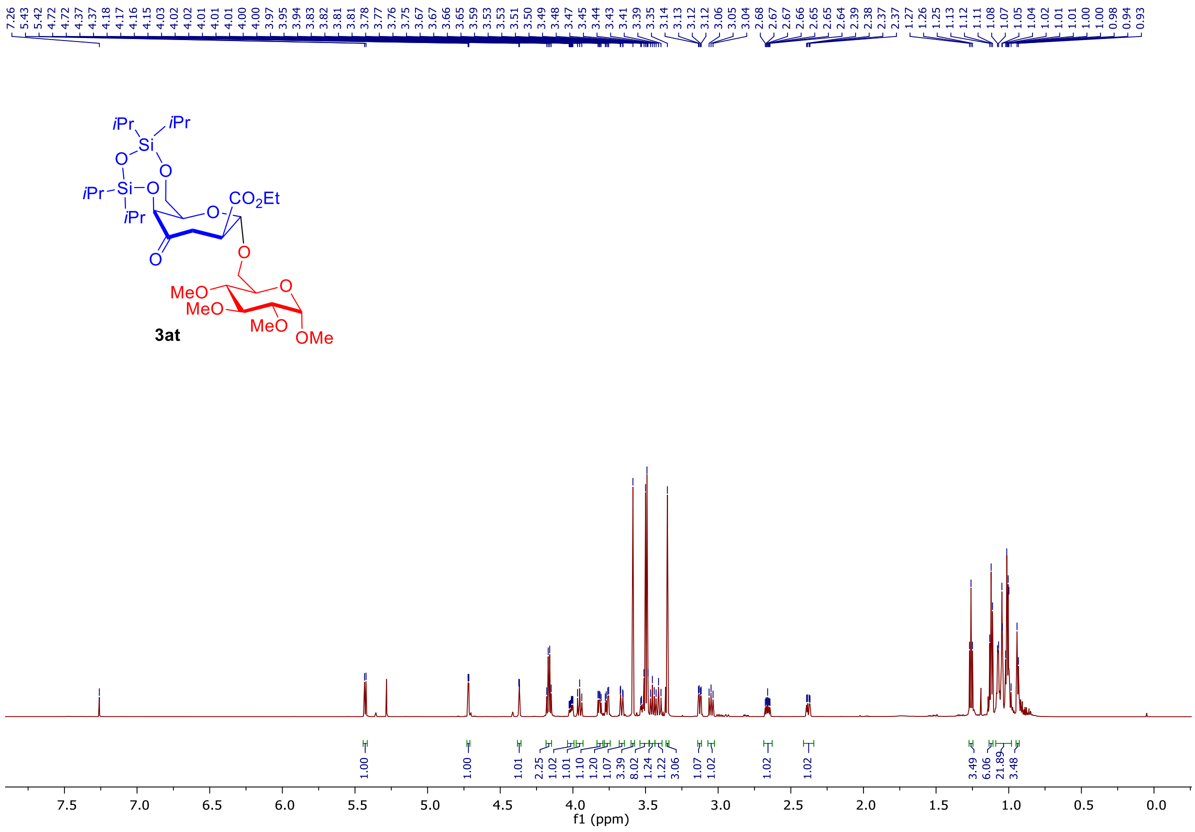

Supplementary Figure 405: ^{1}H spectra for **3at**

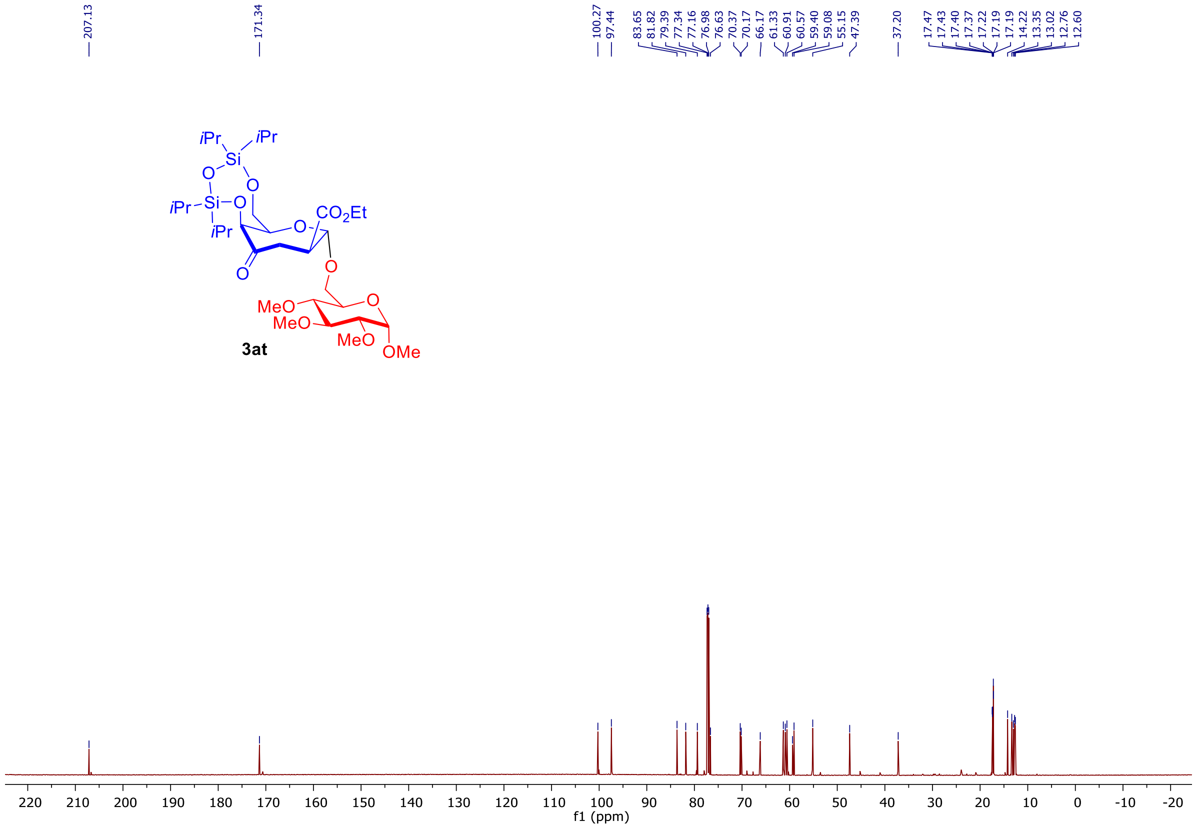

Supplementary Figure 406: ^{13}C spectra for **3at**

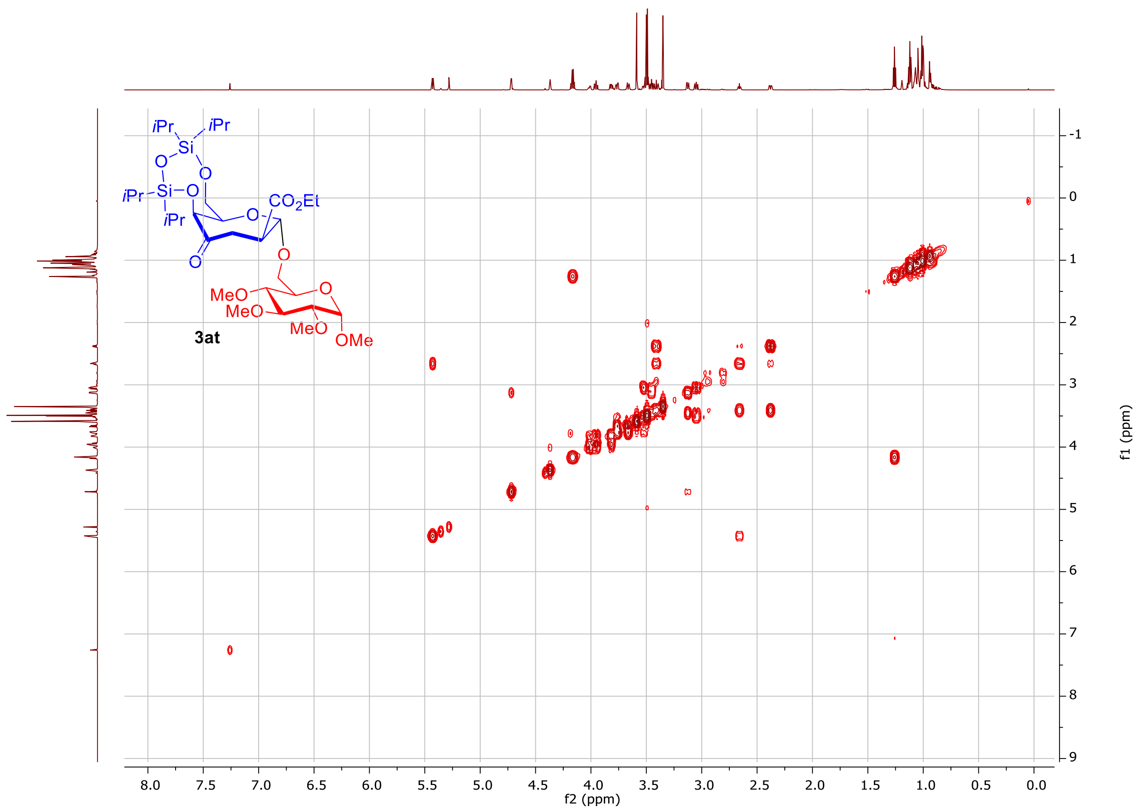

Supplementary Figure 407: COSY spectra for compound **3at**

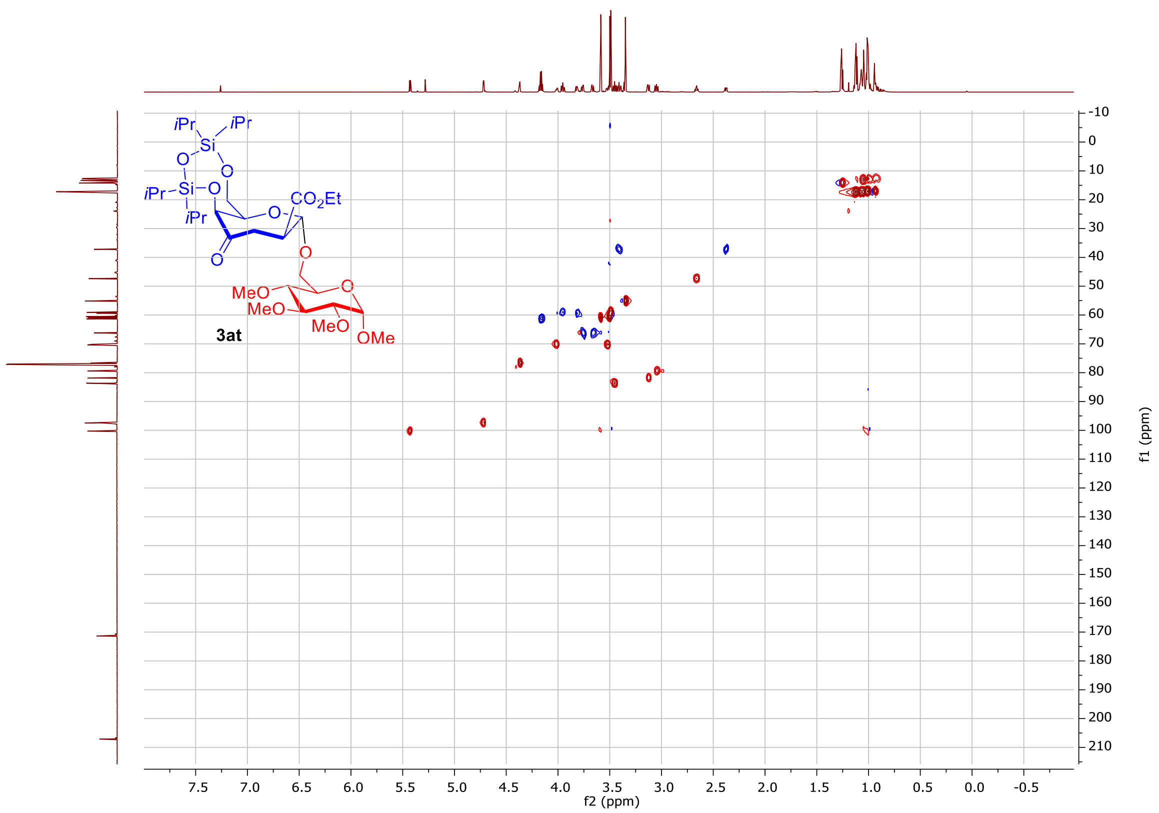

Supplementary Figure 408: HSQC spectra for compound **3at**

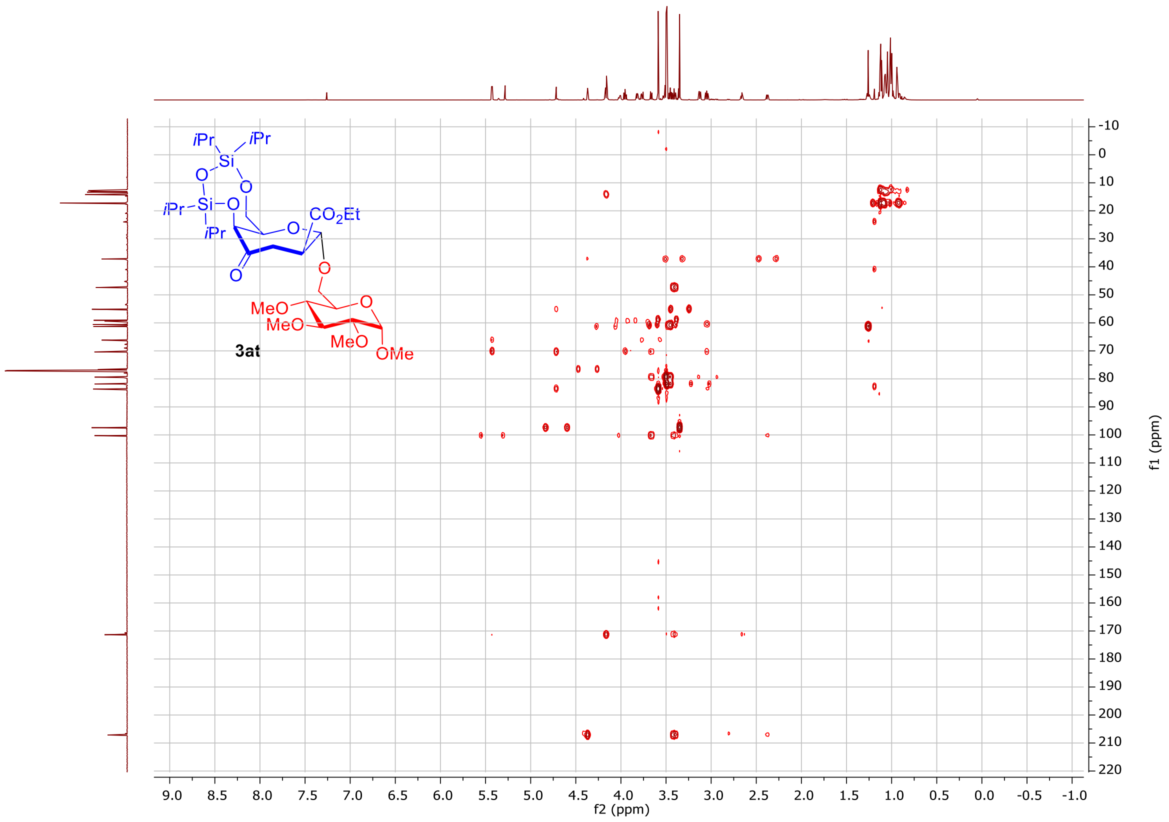

Supplementary Figure 409: HMBC spectra for compound **3at**

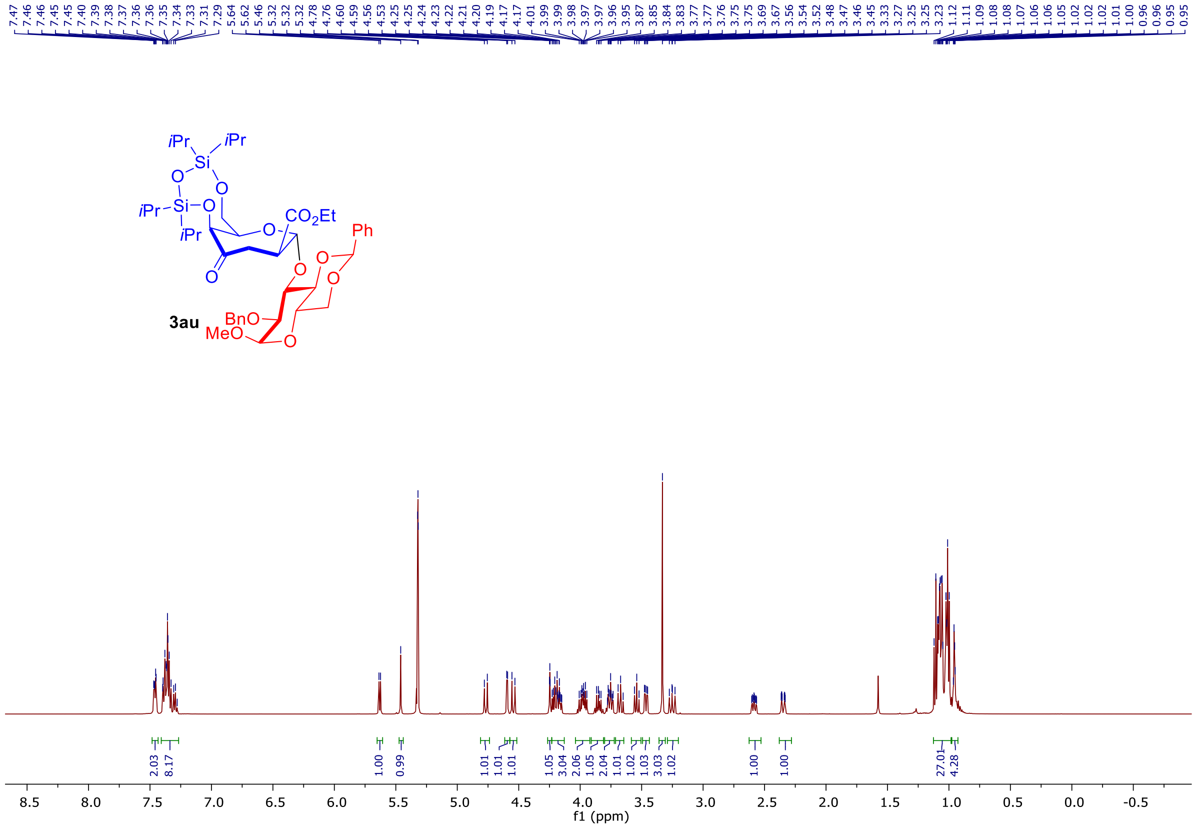

Supplementary Figure 410: ^{1}H spectra for **3au**

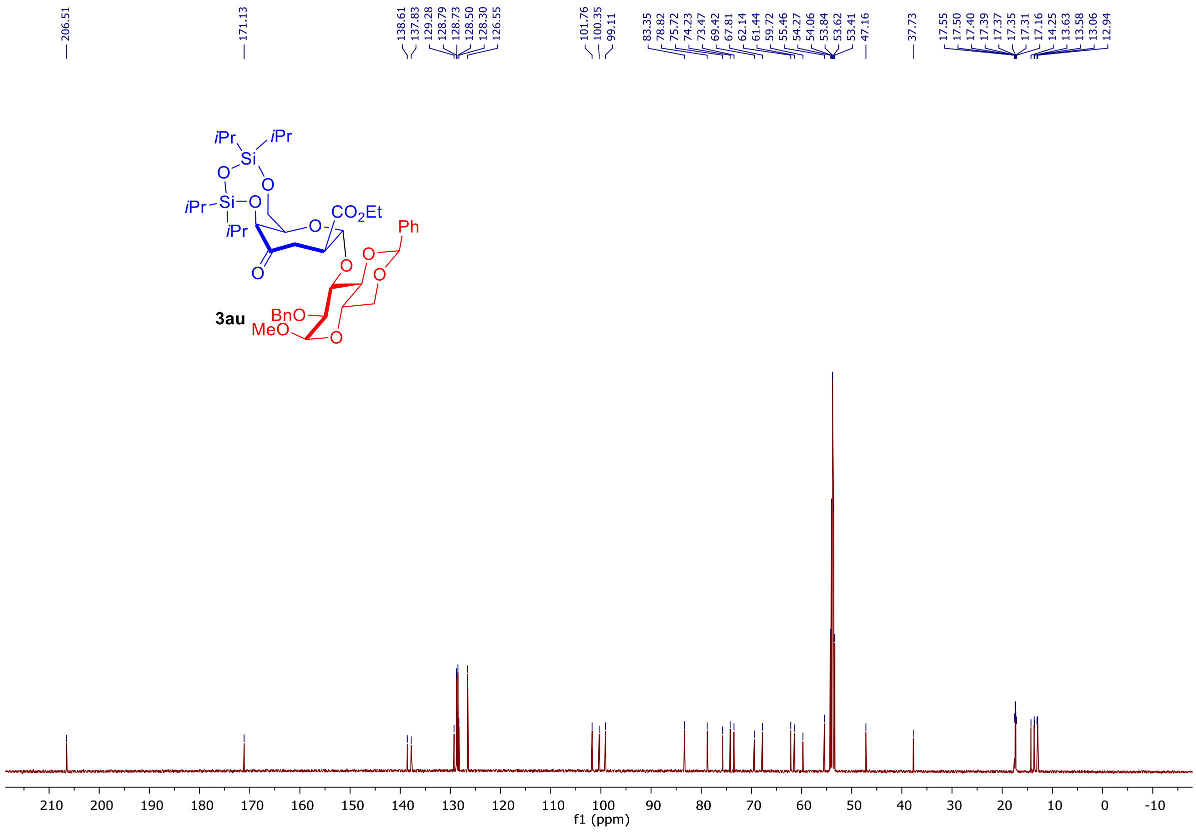

Supplementary Figure 411: ^{13}C spectra for **3au**

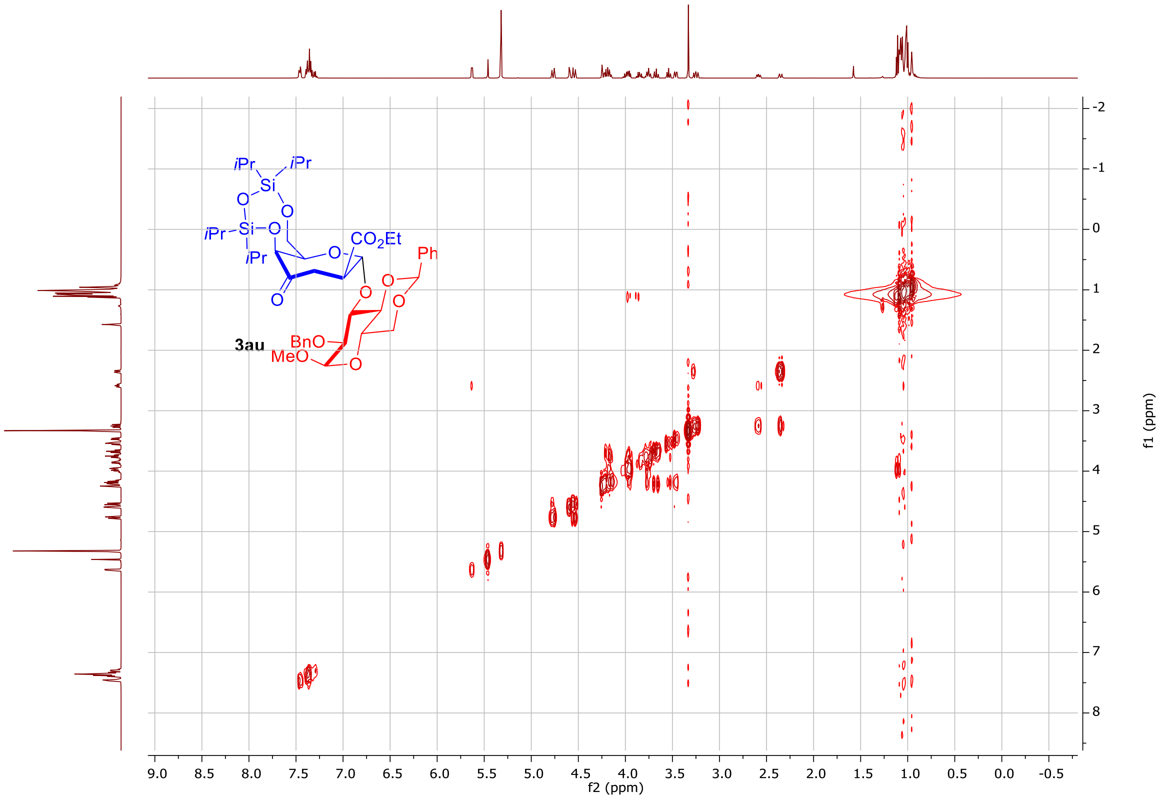

Supplementary Figure 412: COSY spectra for compound **3au**

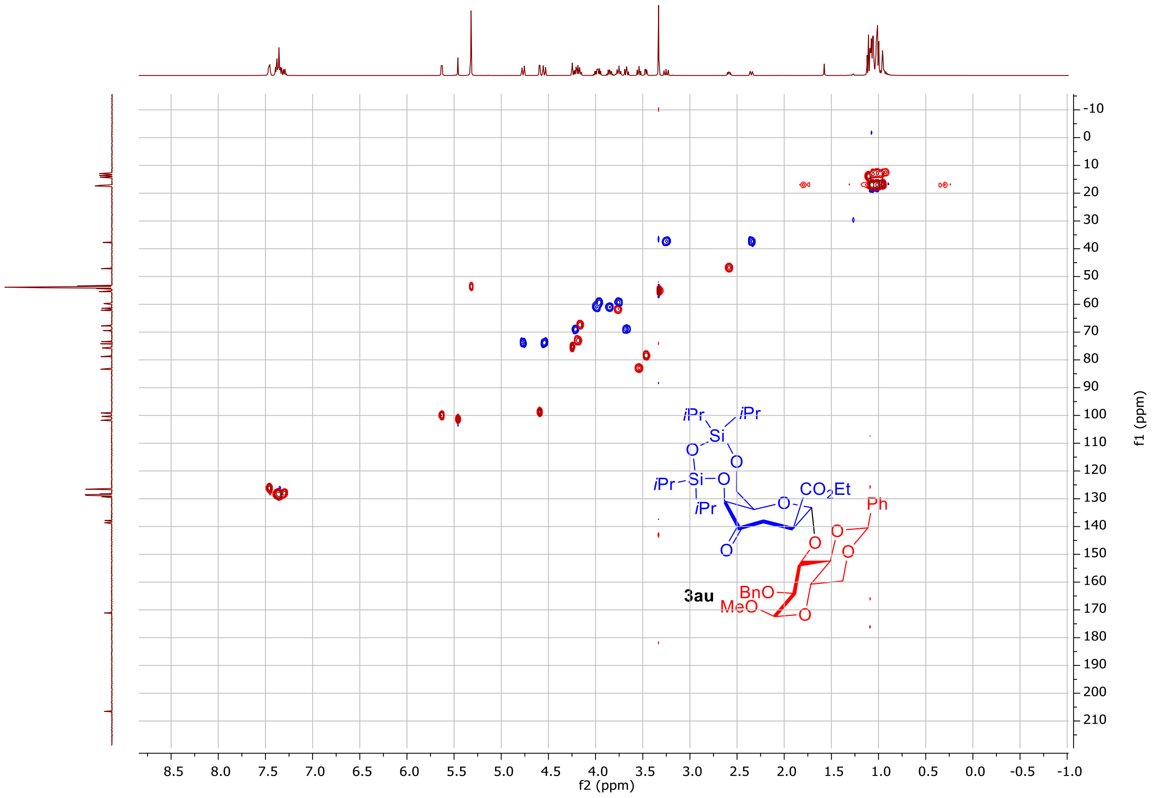

Supplementary Figure 413: HSQC spectra for compound **3au**

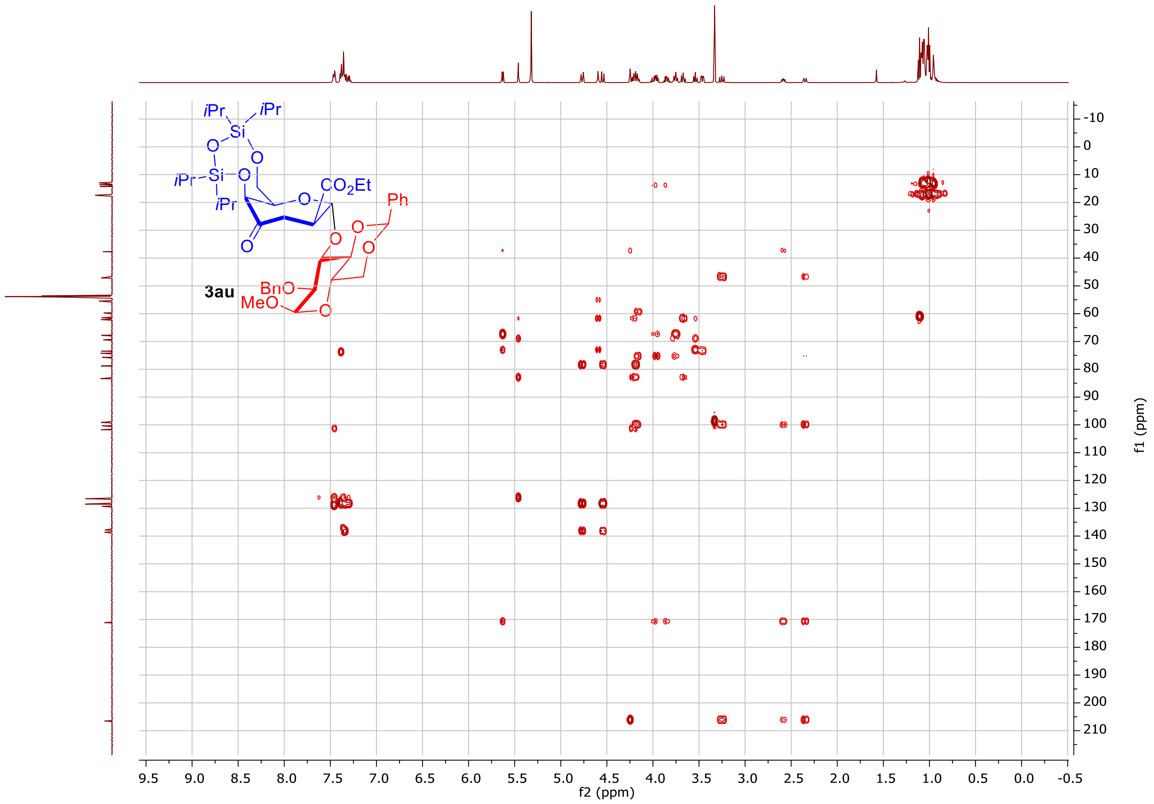

Supplementary Figure 414: HMBC spectra for compound **3au**

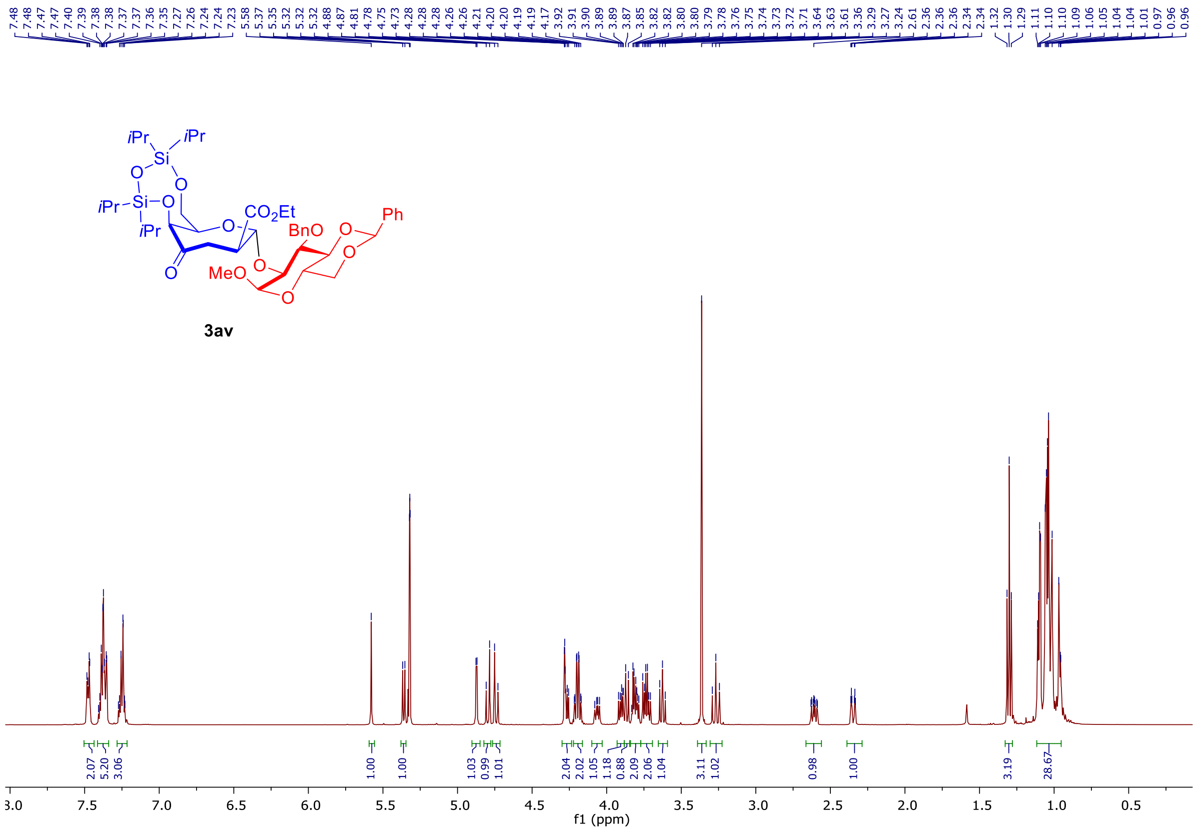

Supplementary Figure 415: ^{1}H spectra for **3av**

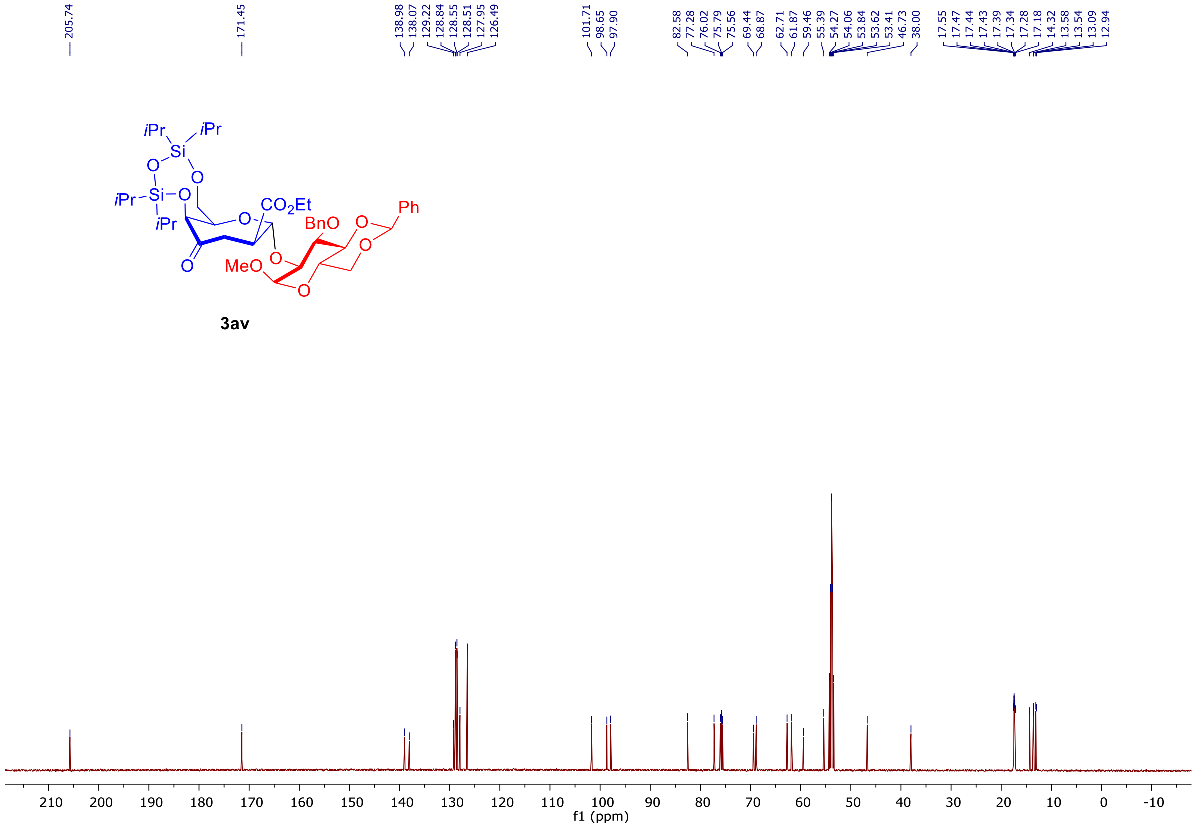

Supplementary Figure 416: ^{13}C spectra for **3av**

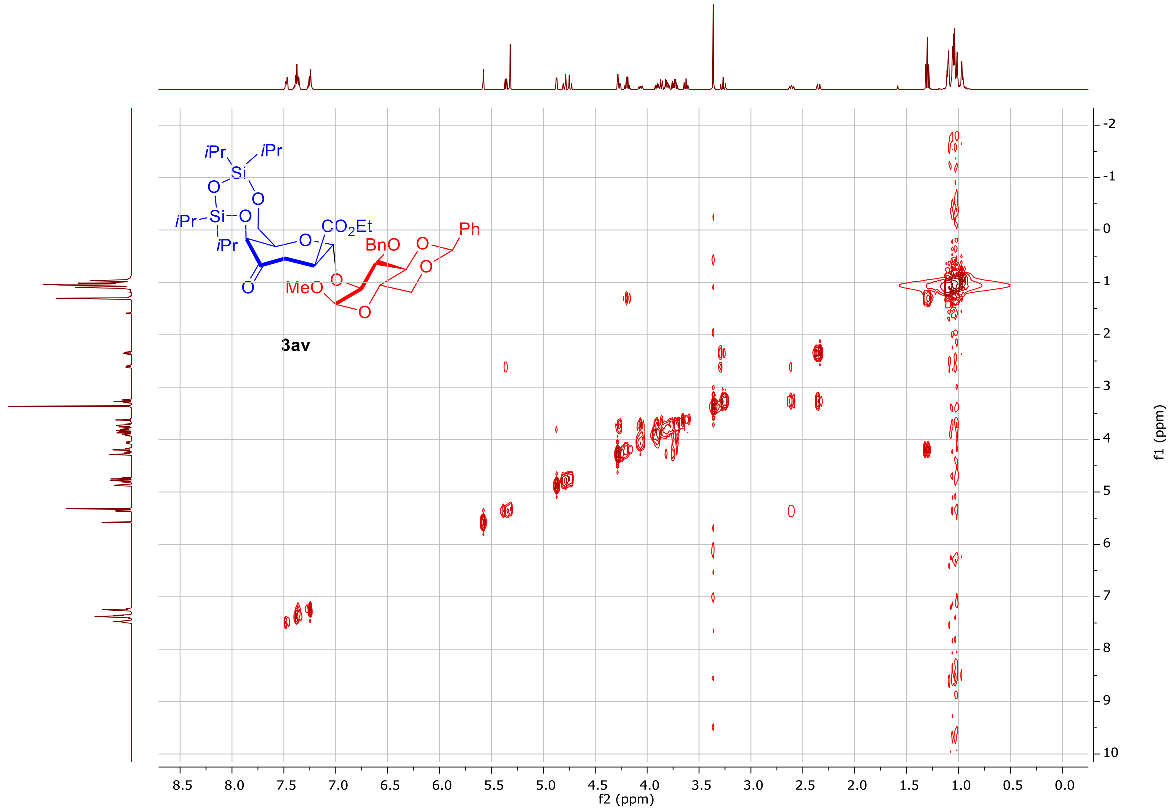

Supplementary Figure 417: COSY spectra for compound **3av**

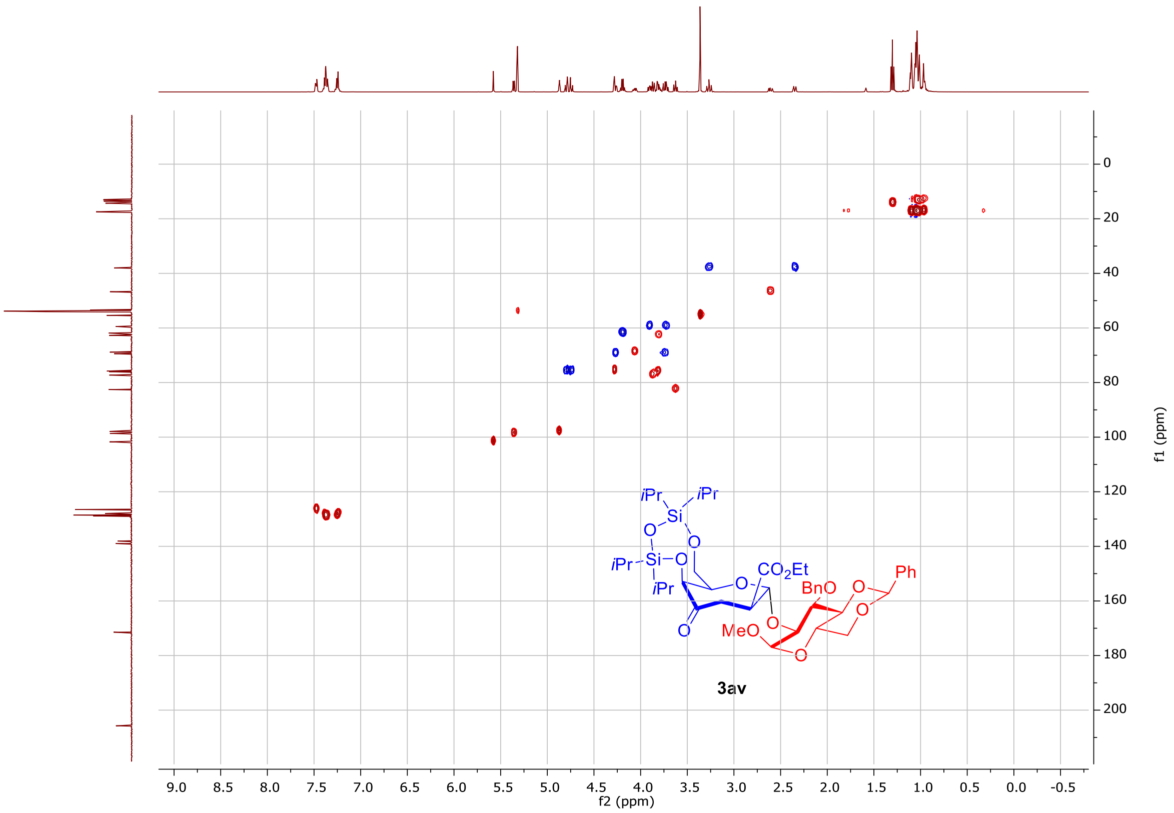

Supplementary Figure 418: HSQC spectra for compound **3av**

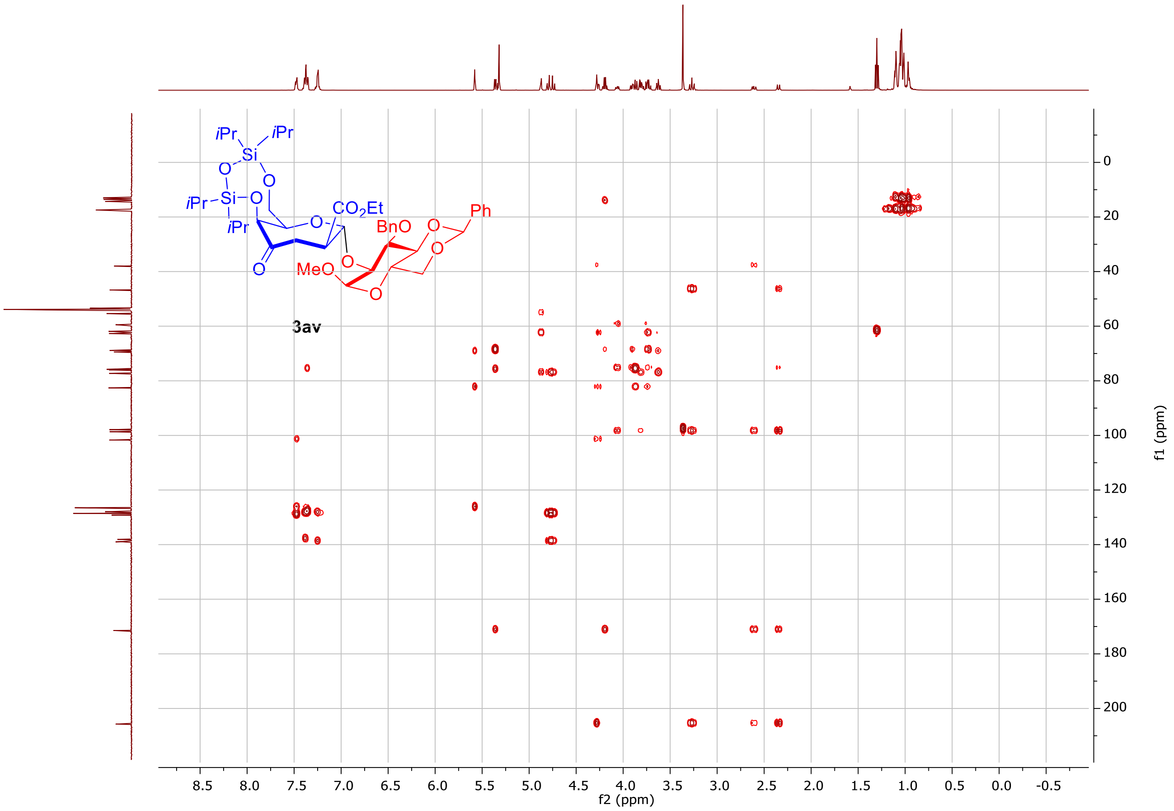

Supplementary Figure 419: HMBC spectra for compound **3av**

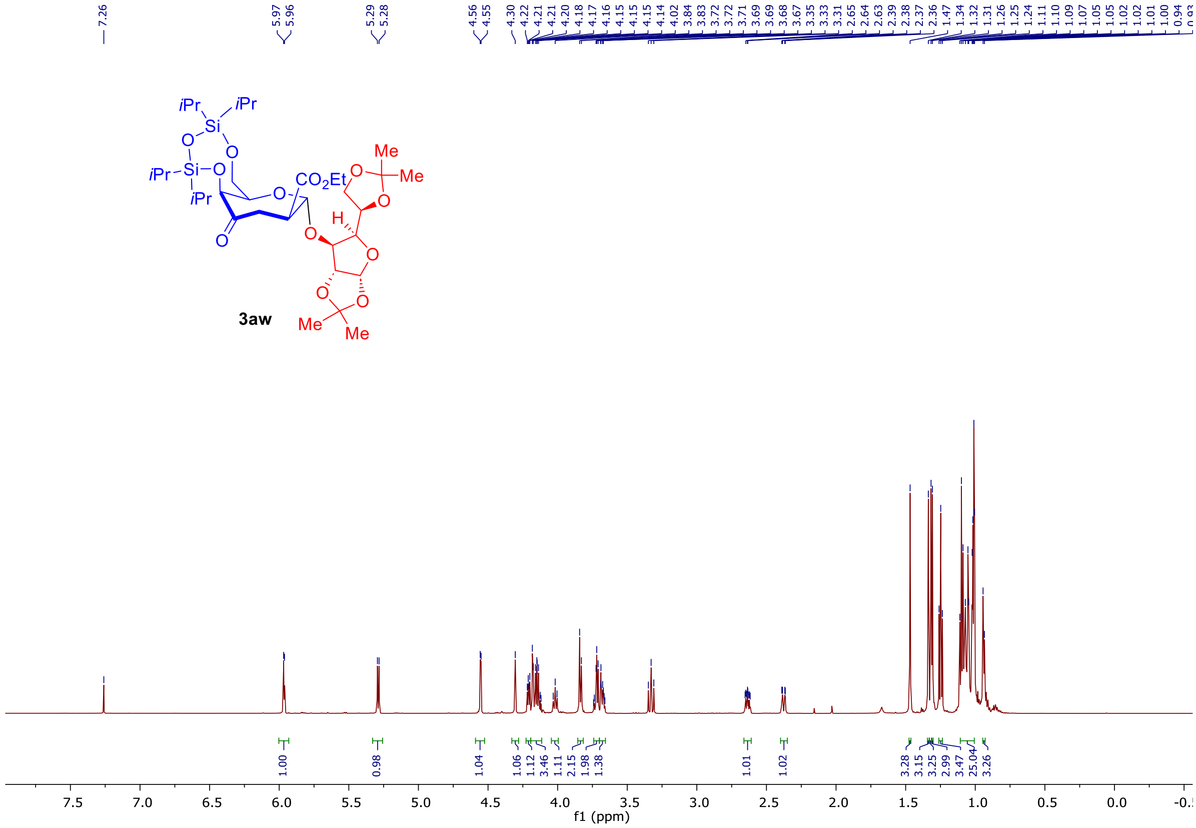

Supplementary Figure 420: ^{1}H spectra for **3aw**

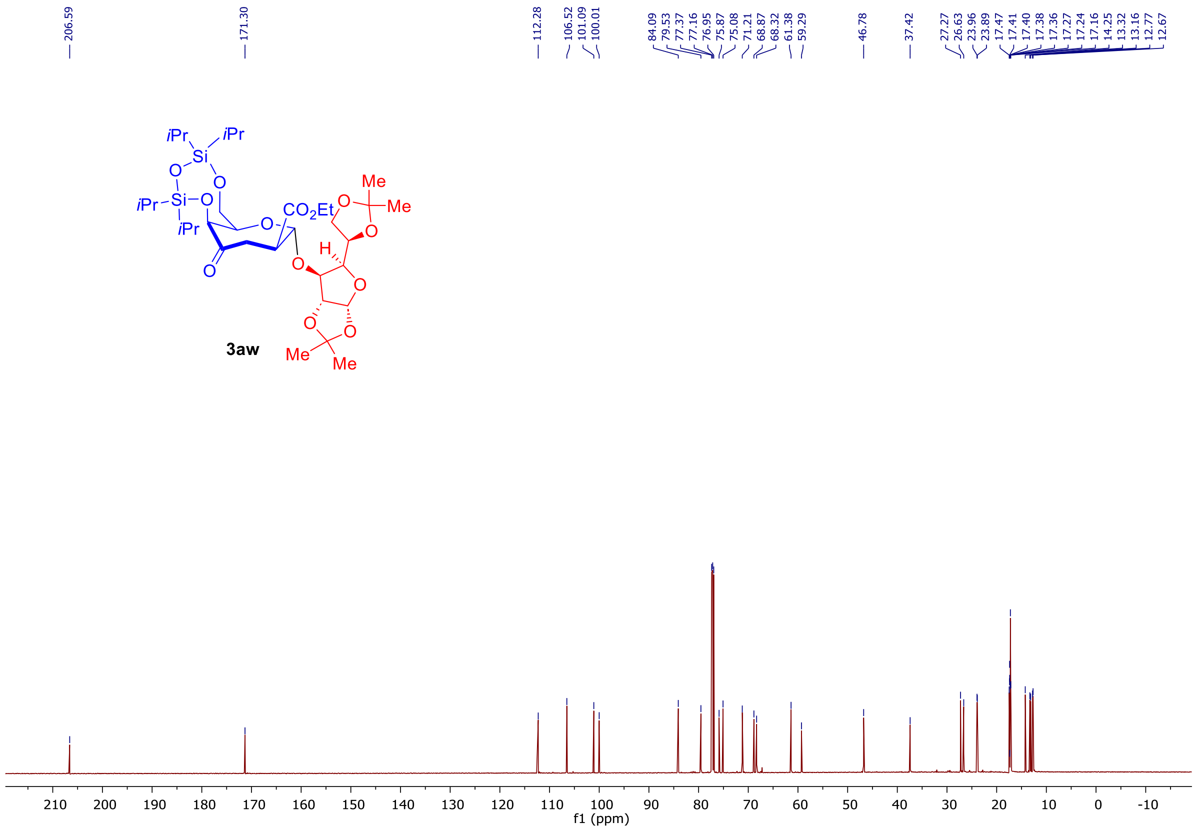

Supplementary Figure 421: ^{13}C spectra for **3aw**

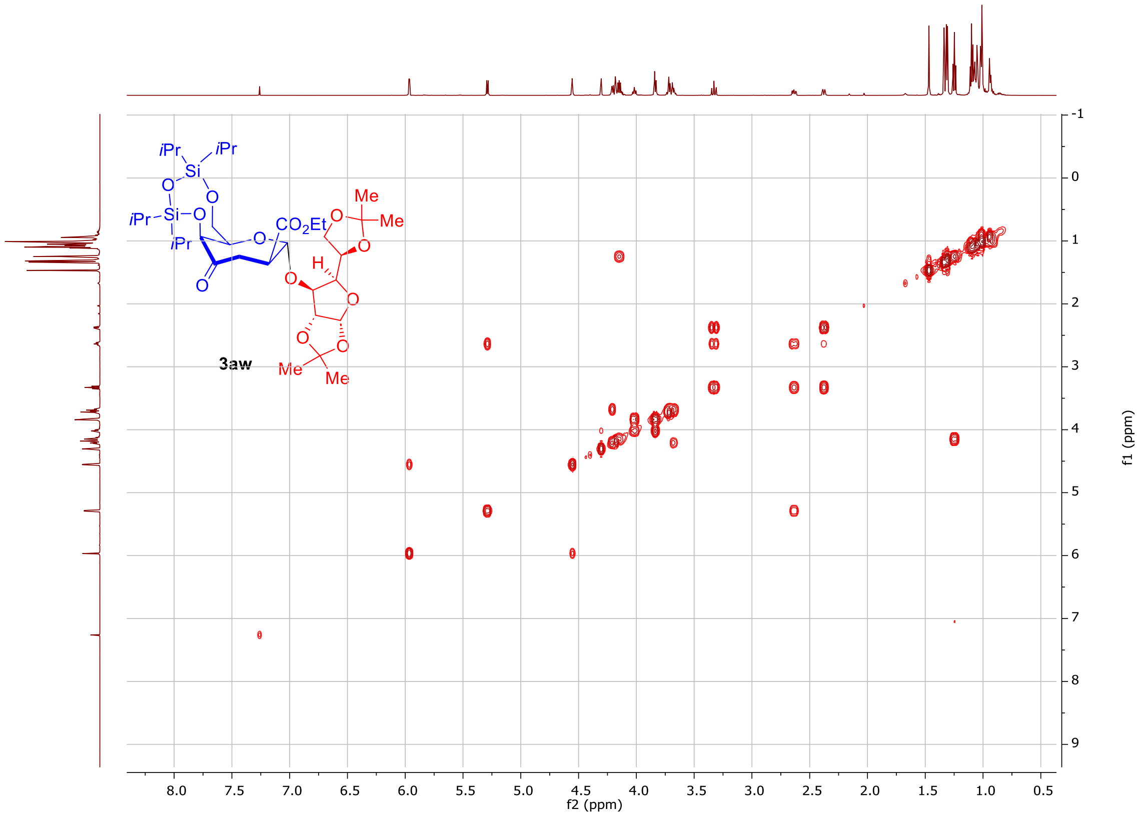

Supplementary Figure 422: COSY spectra for compound **3aw**

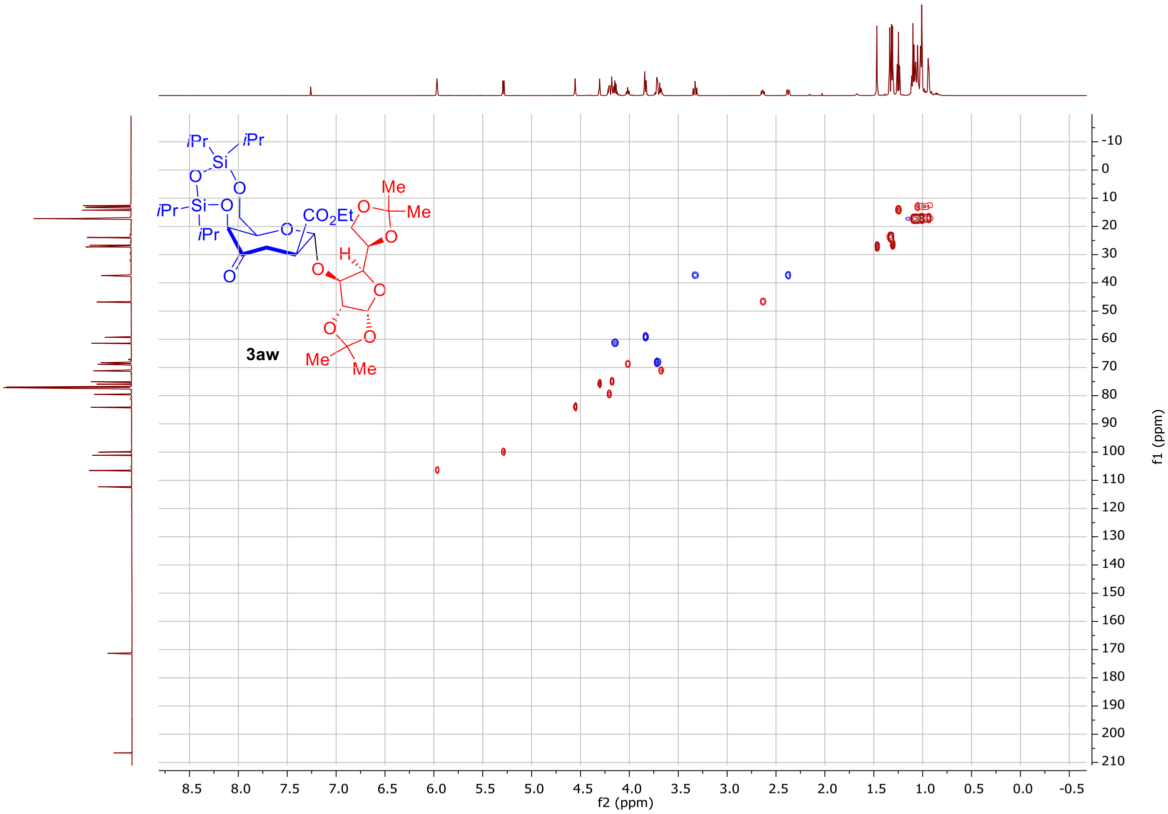

Supplementary Figure 423: HSQC spectra for compound **3aw**

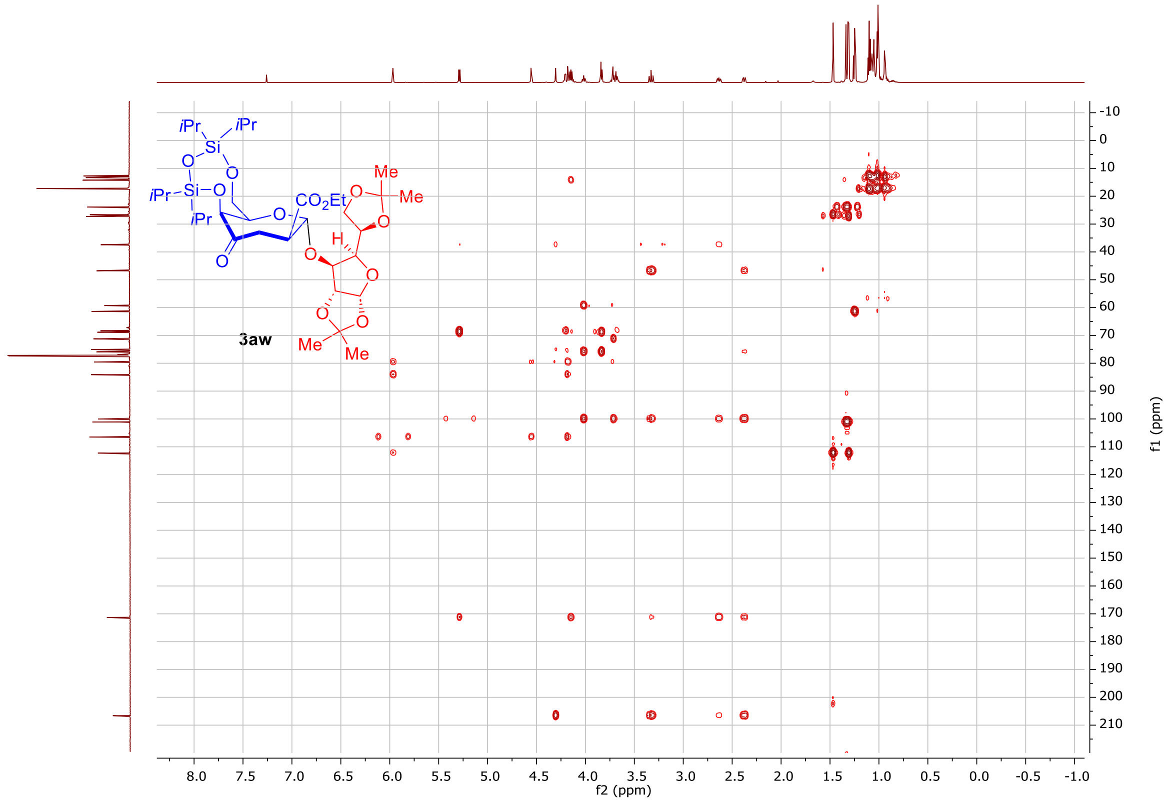

Supplementary Figure 424: HMBC spectra for compound **3aw**

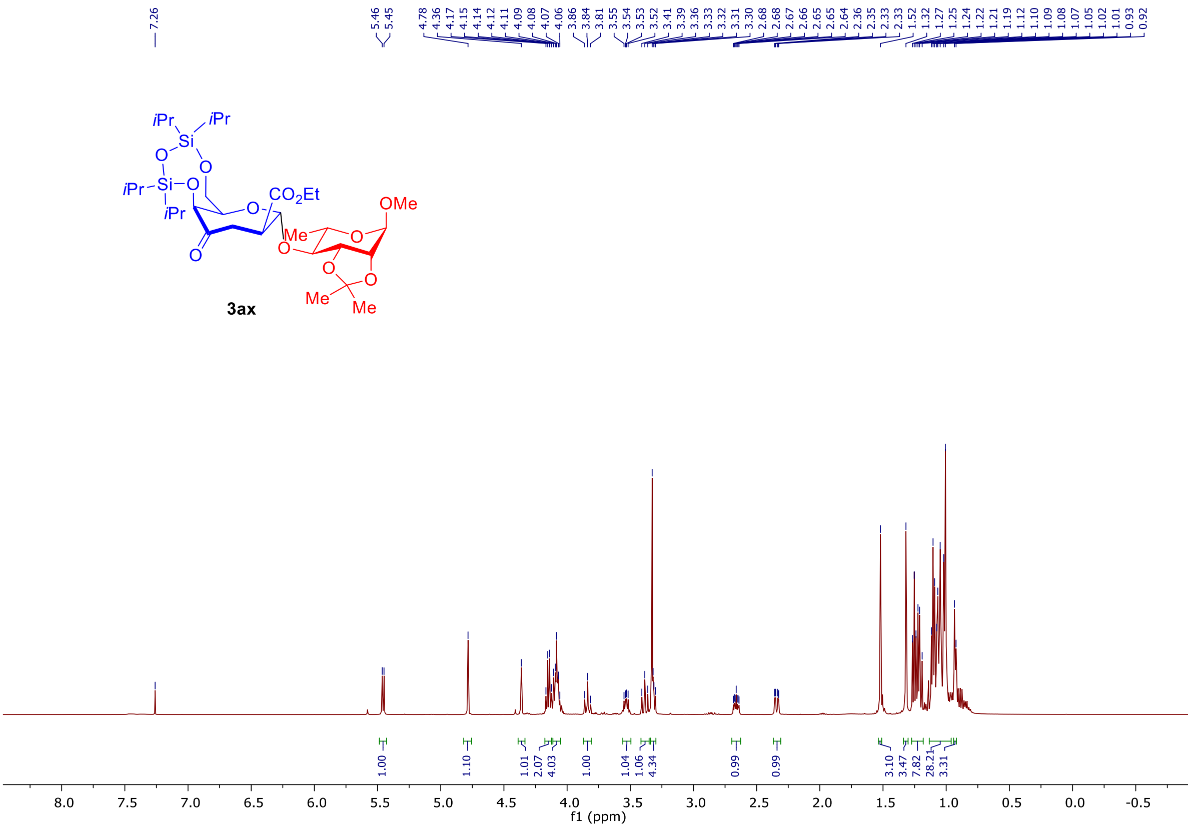

Supplementary Figure 425: ^{1}H spectra for **3ax**

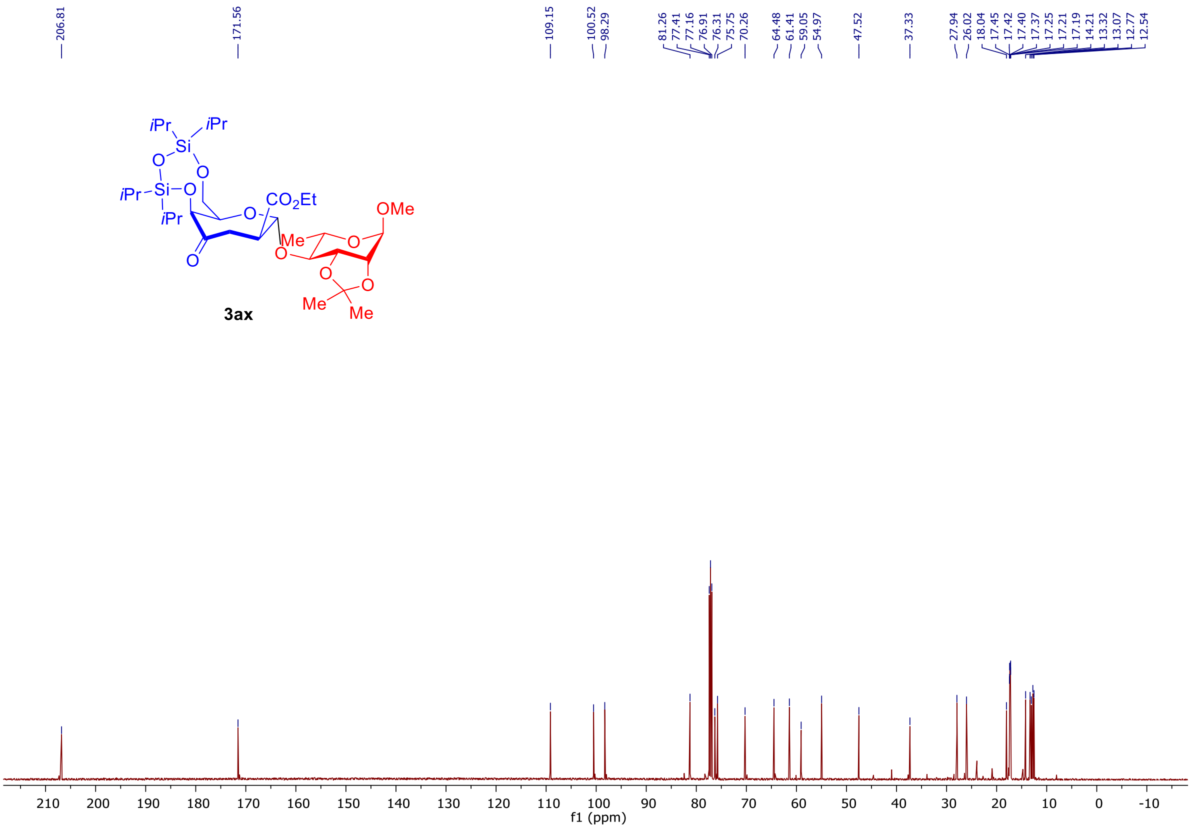

Supplementary Figure 426: ^{13}C spectra for **3ax**

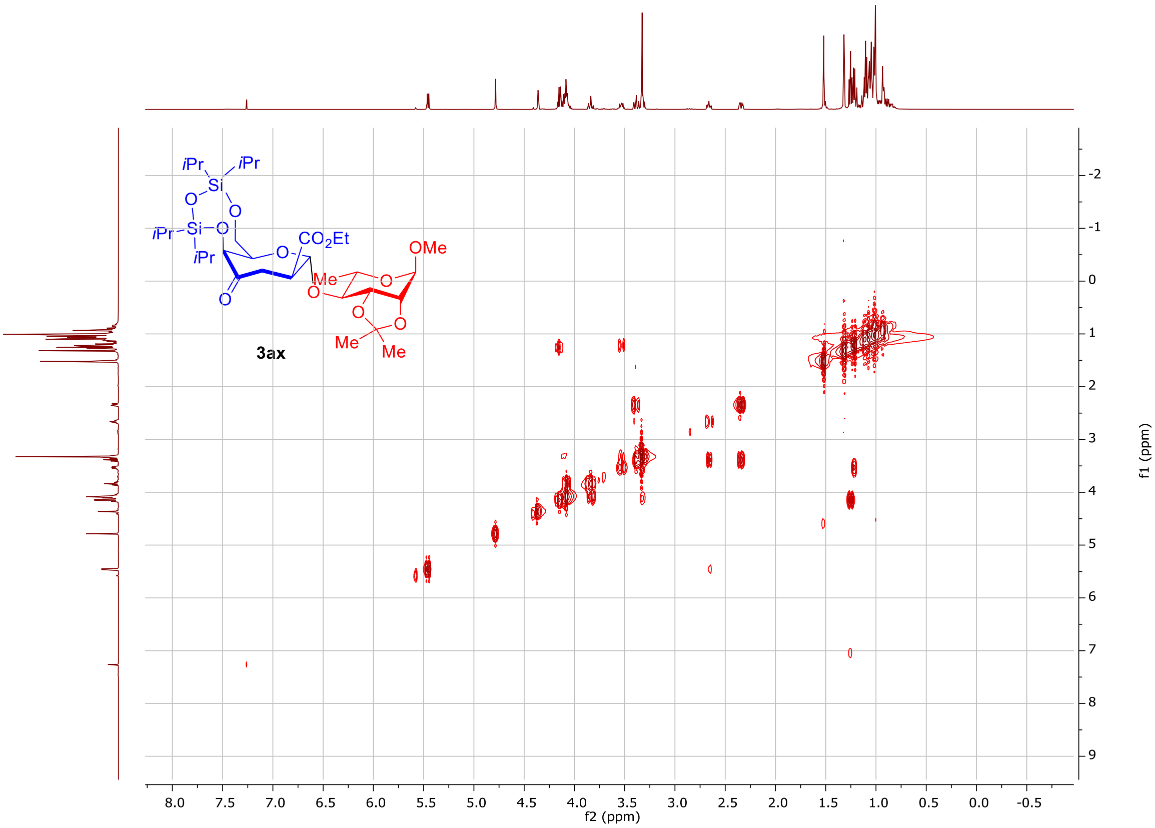

Supplementary Figure 427: COSY spectra for compound **3ax**

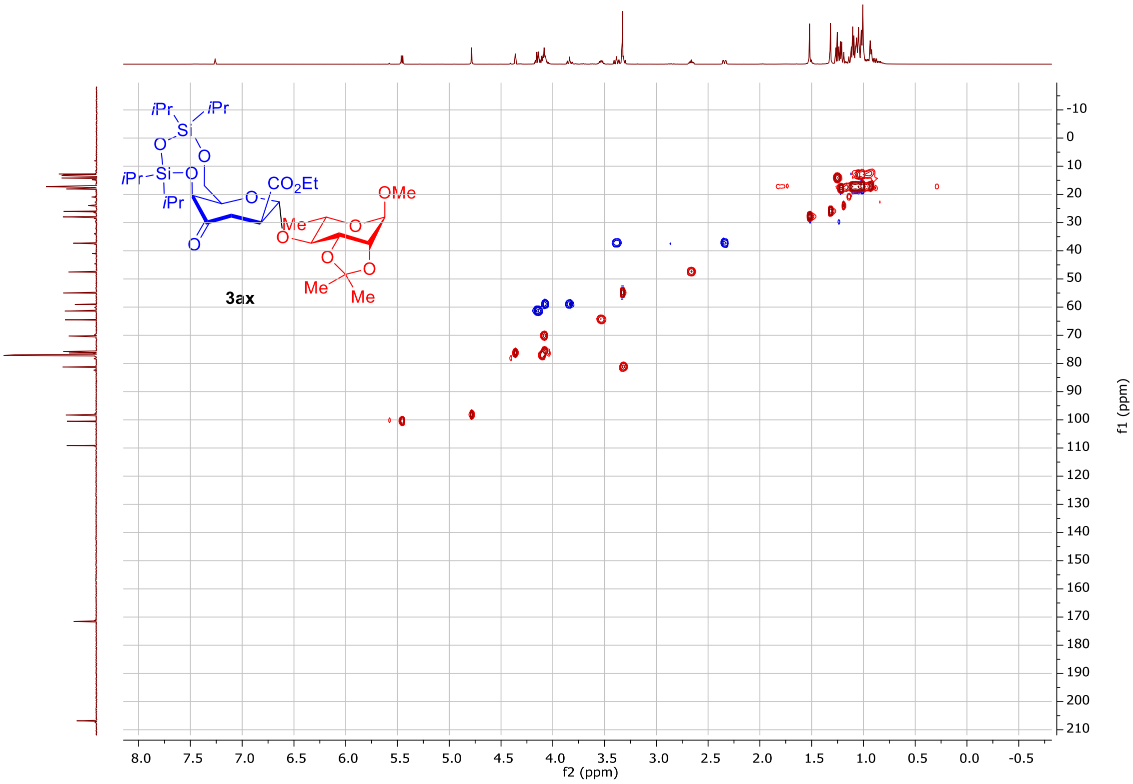

Supplementary Figure 428: HSQC spectra for compound **3ax**

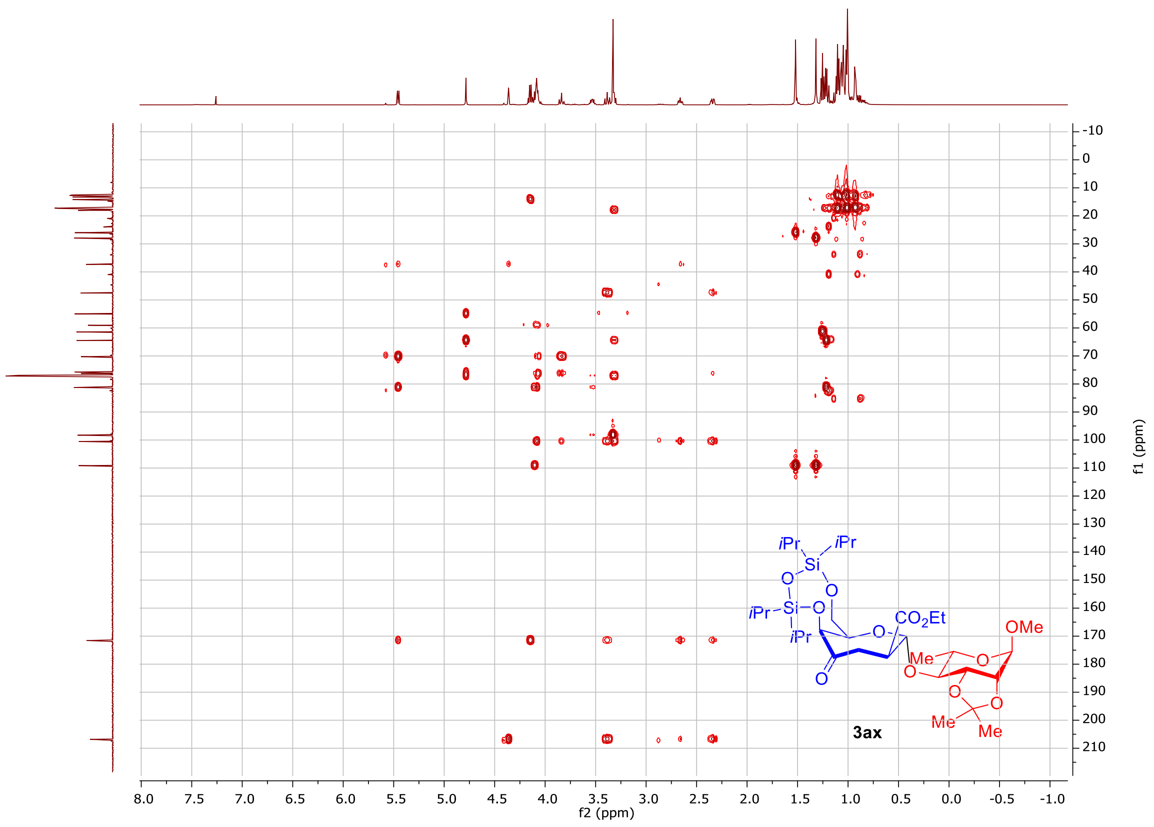

Supplementary Figure 429: HMBC spectra for compound **3ax**

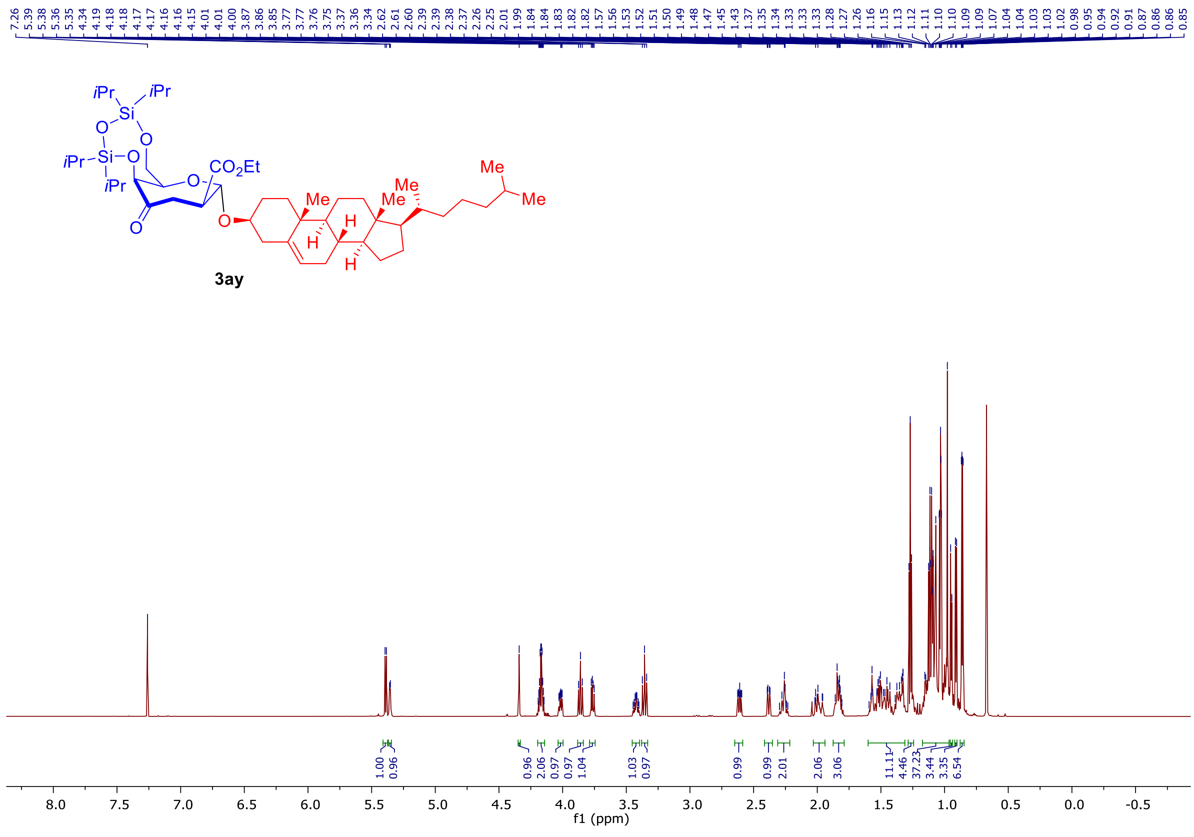

Supplementary Figure 430: ^{1}H spectra for **3ay**

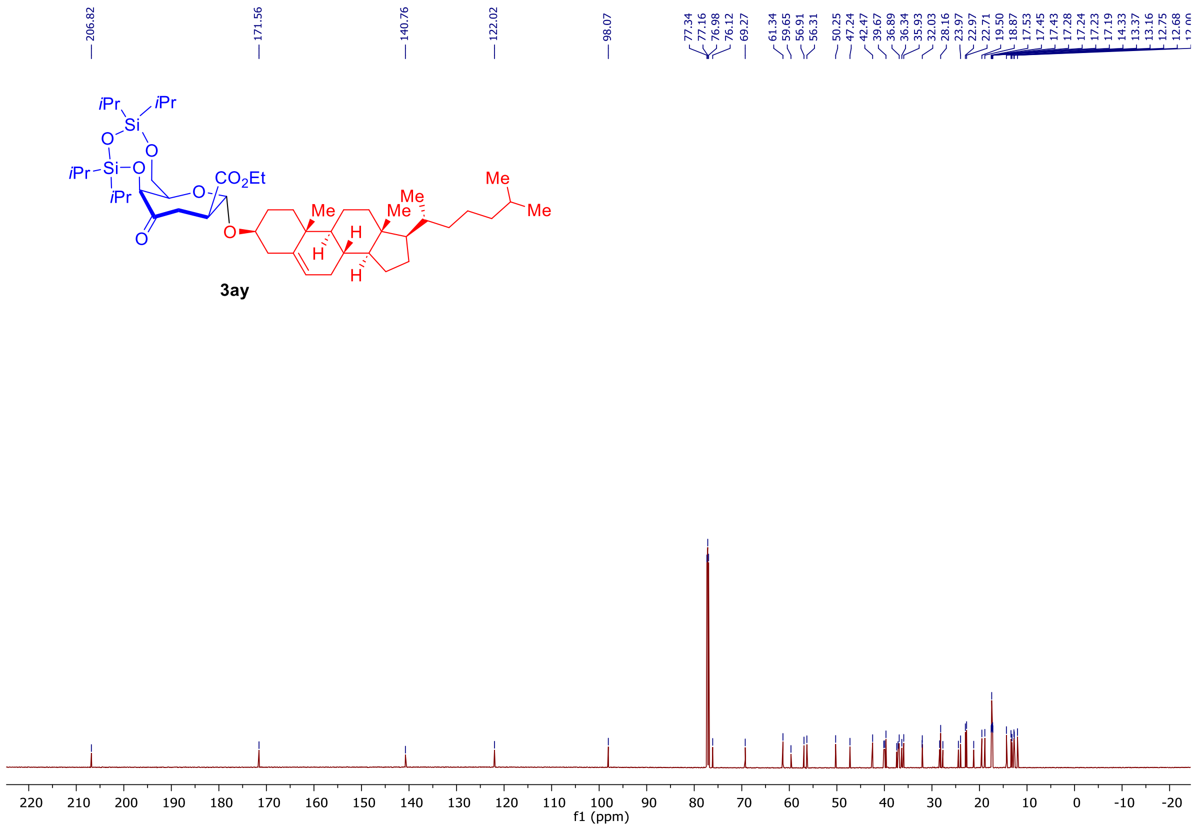

Supplementary Figure 431: ^{13}C spectra for **3ay**

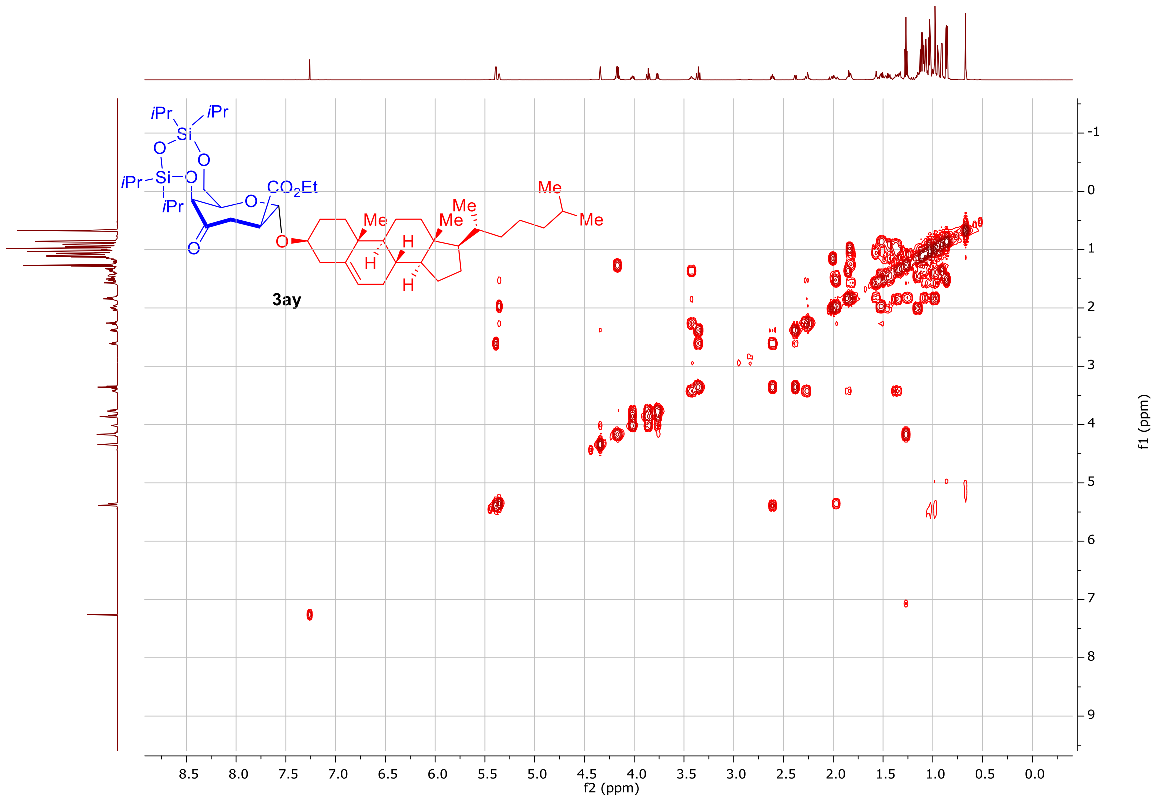

Supplementary Figure 432: COSY spectra for compound **3ay**

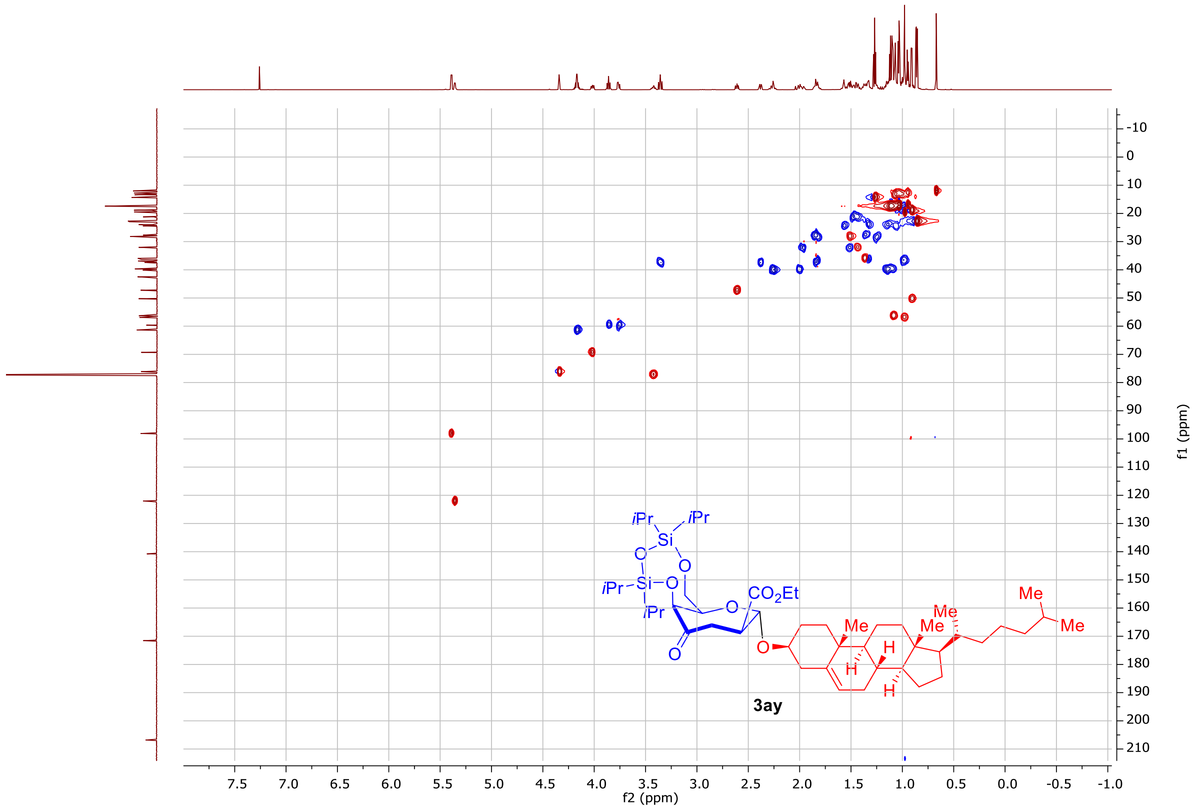

Supplementary Figure 433: HSQC spectra for compound **3ay**

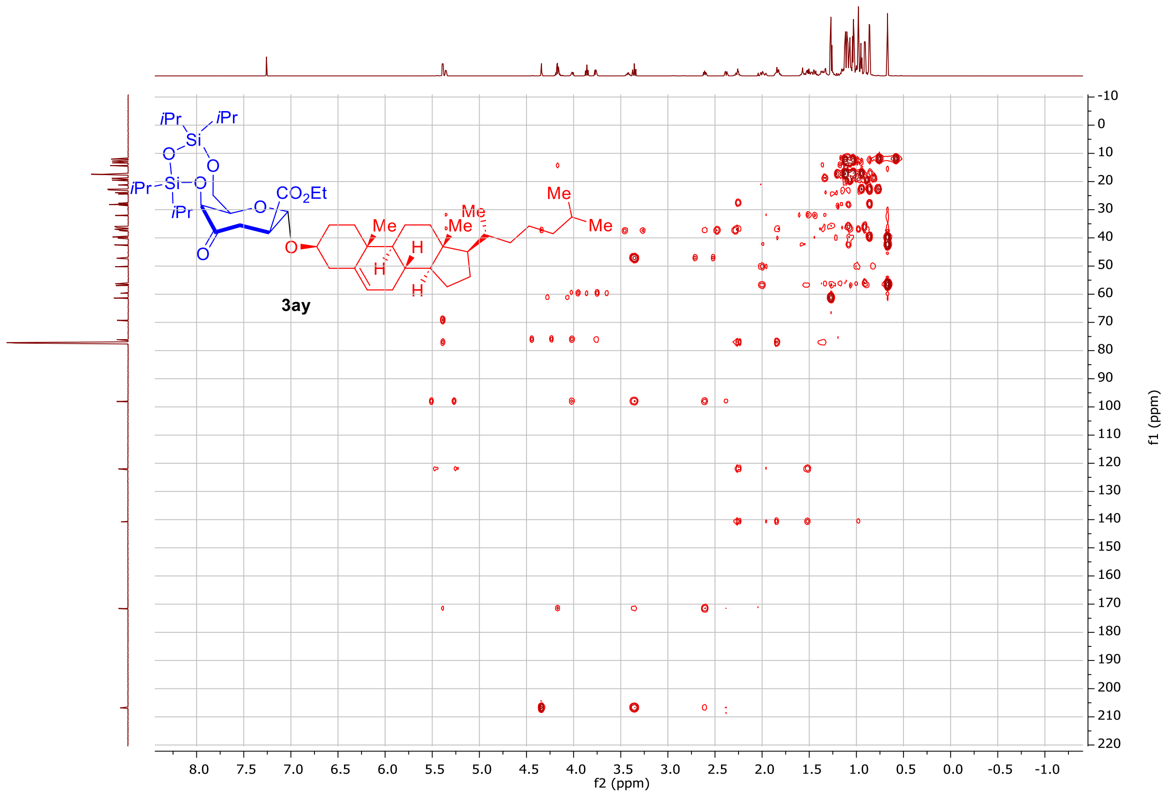

Supplementary Figure 434: HMBC spectra for compound **3ay**

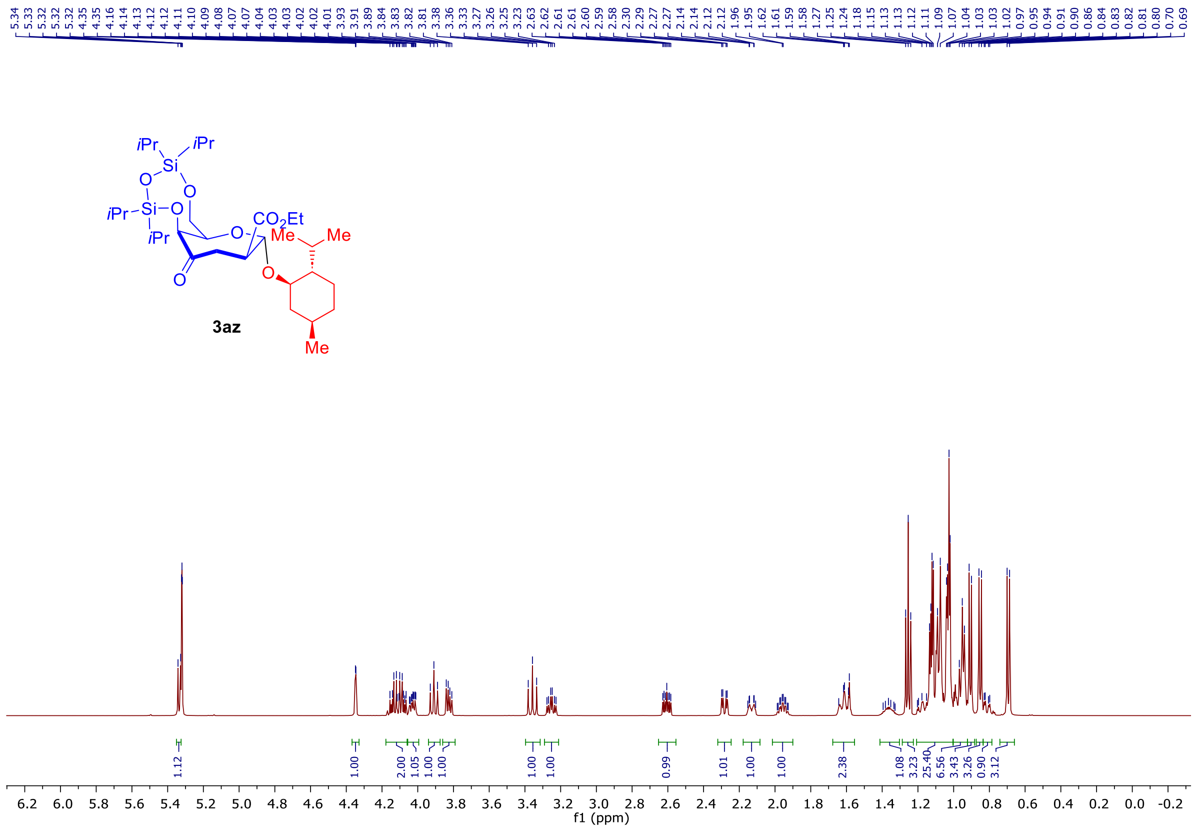

Supplementary Figure 435: ^{1}H spectra for **3az**

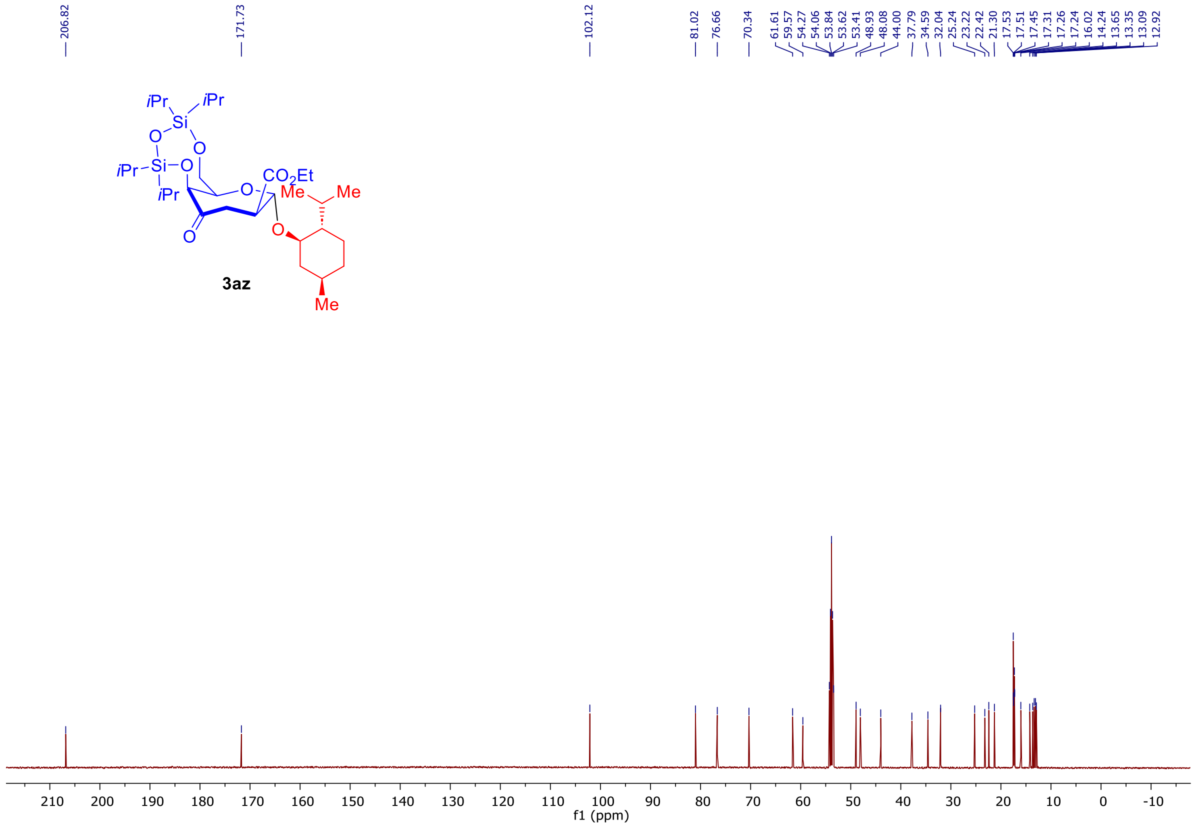

Supplementary Figure 436: ^{13}C spectra for **3az**

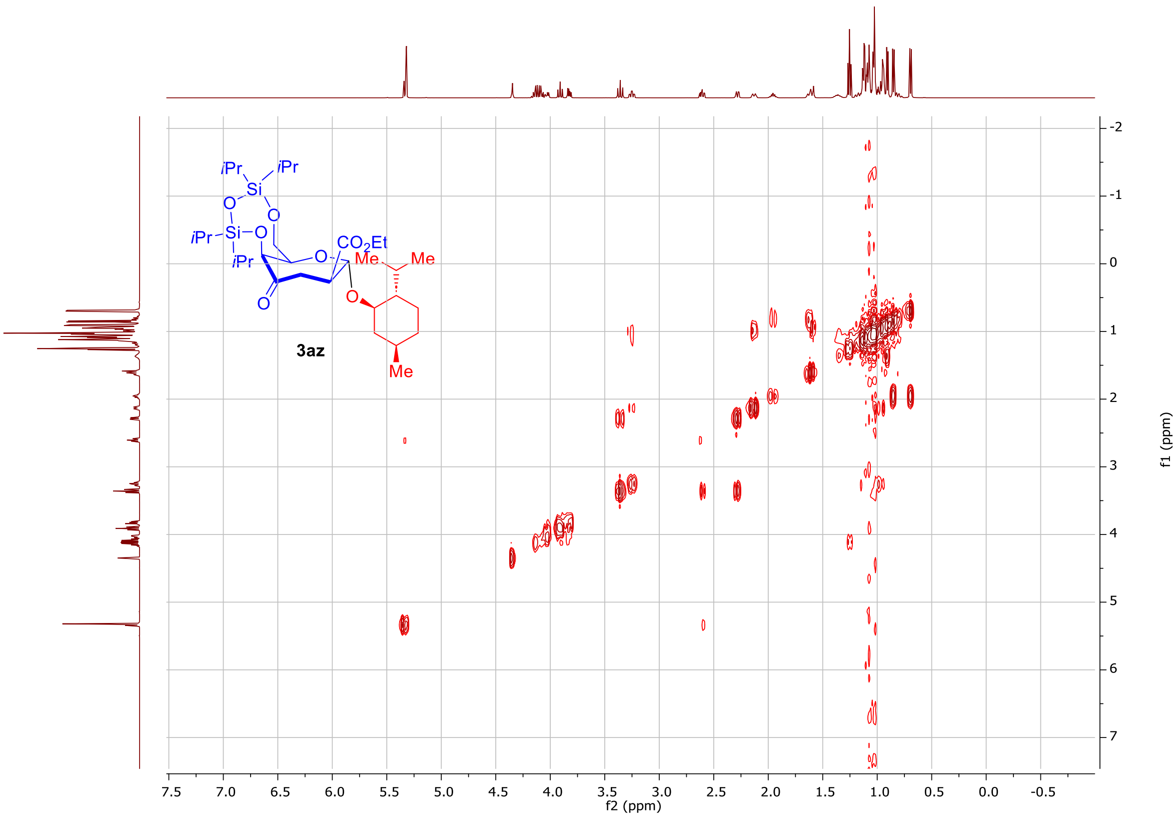

Supplementary Figure 437: COSY spectra for compound **3az**

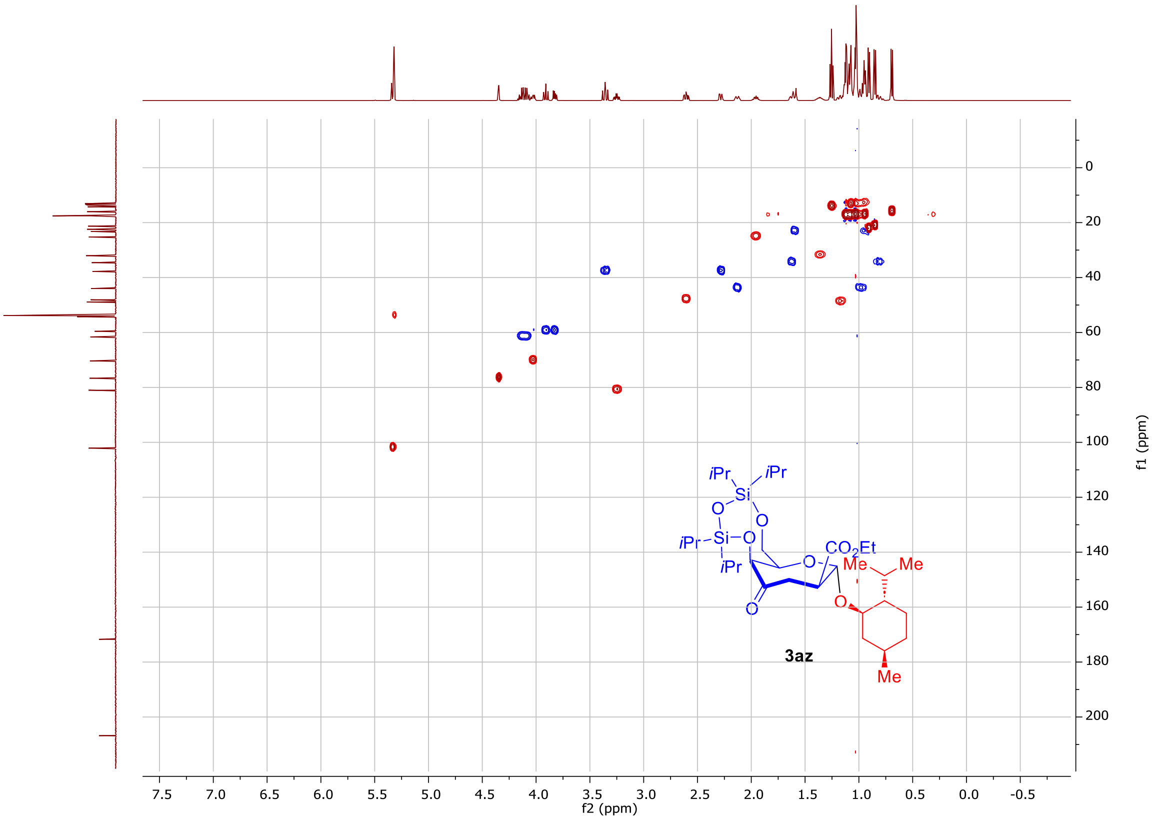

Supplementary Figure 438: HSQC spectra for compound **3az**

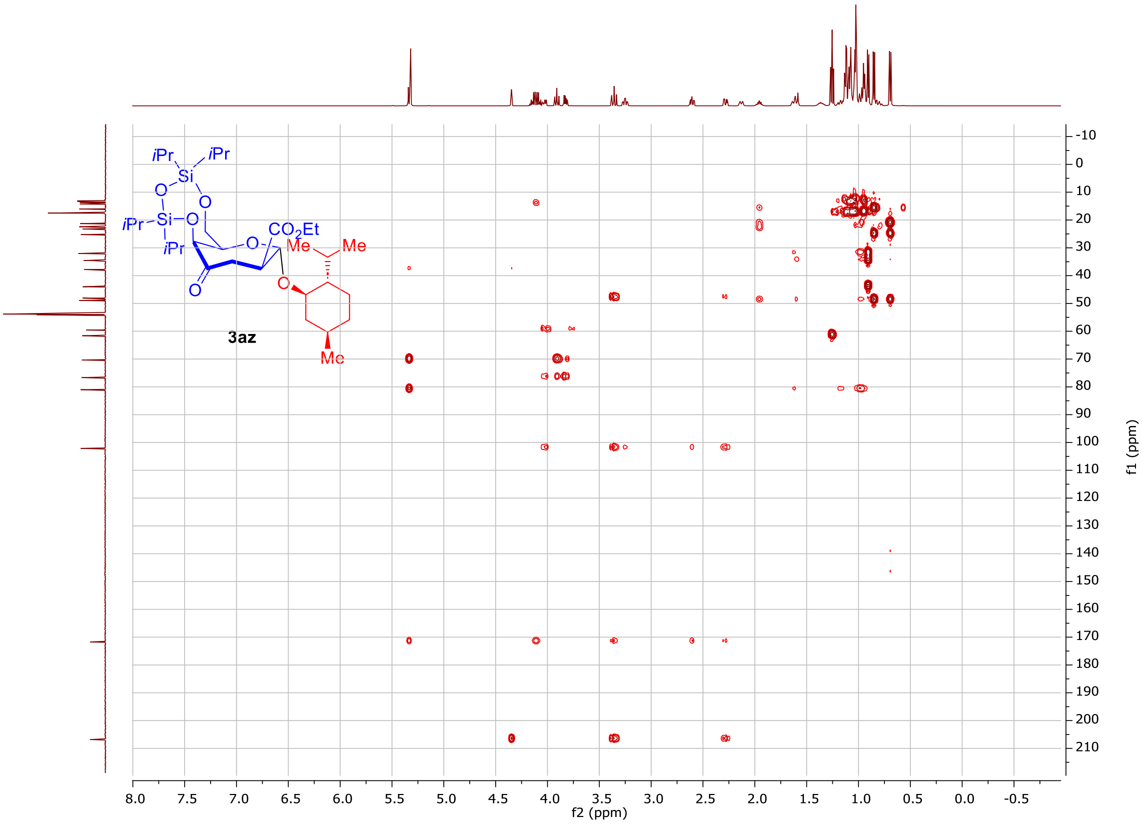

Supplementary Figure 439: HMBC spectra for compound **3az**

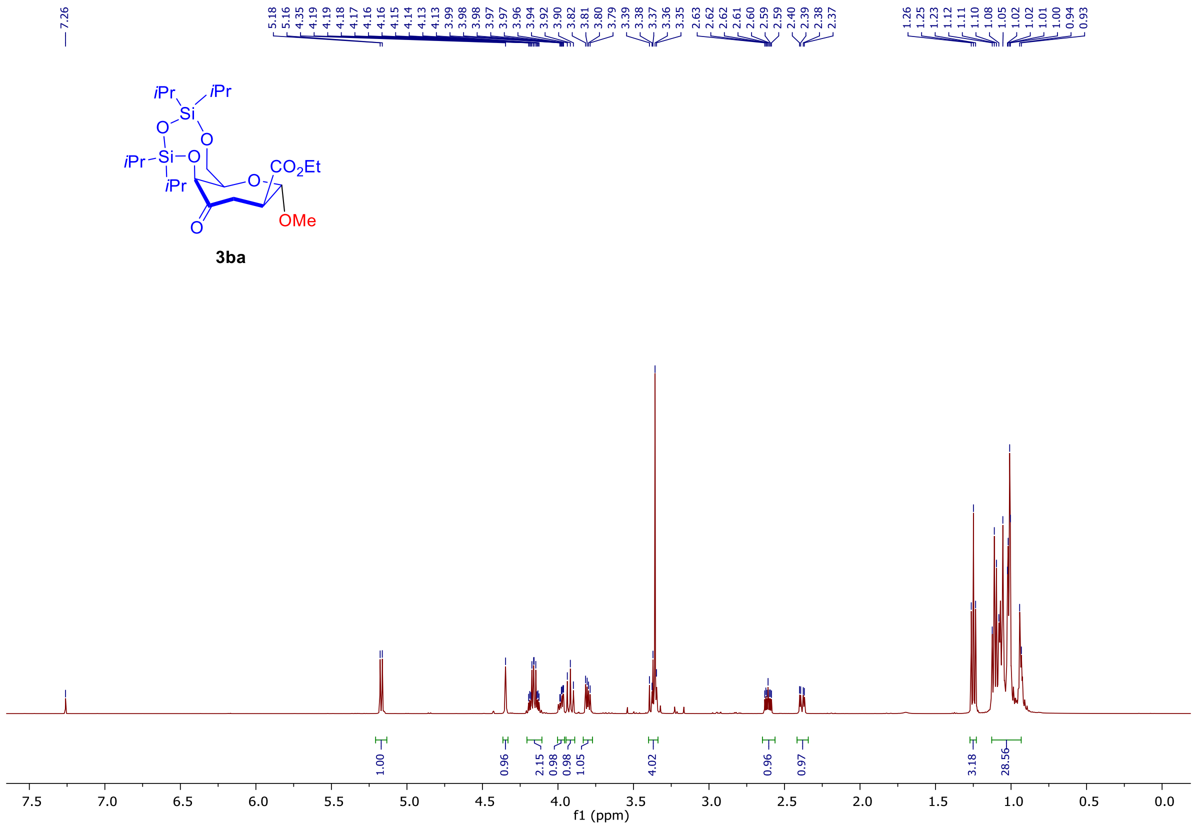

Supplementary Figure 440: ^{1}H spectra for **3ba**

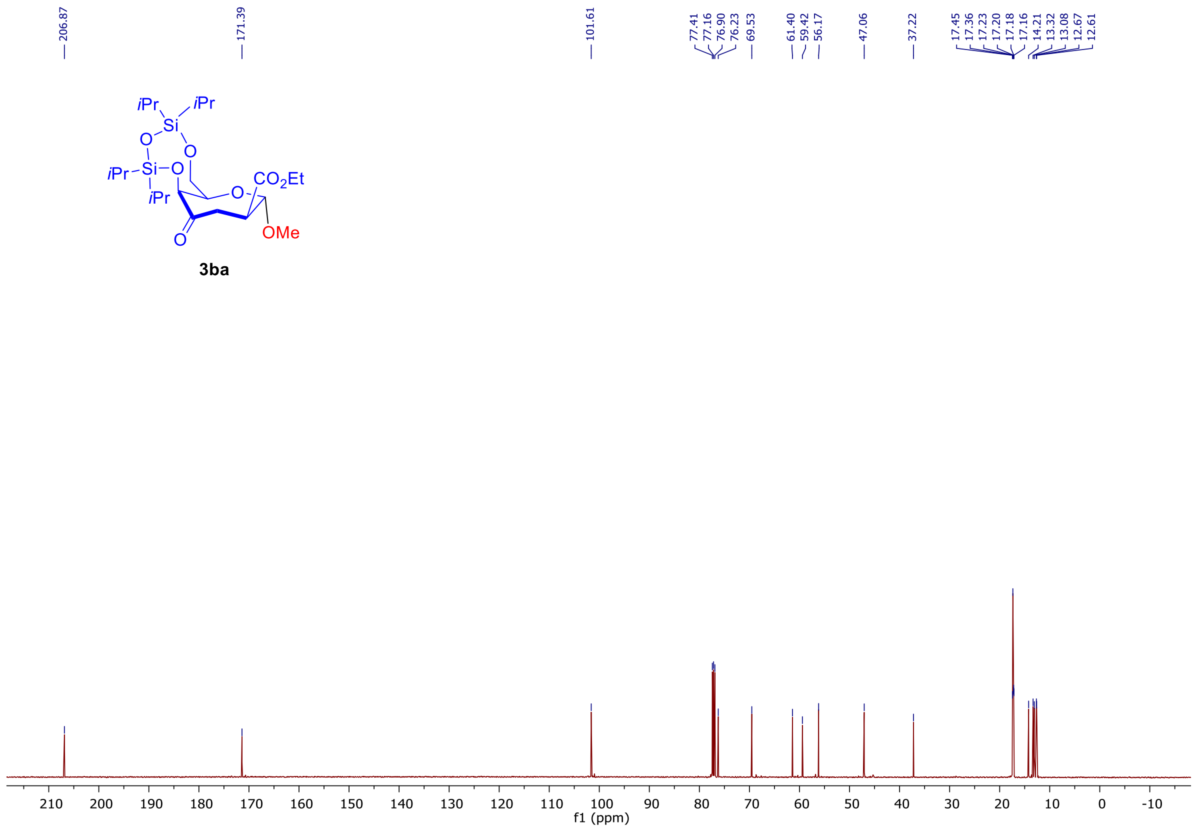

Supplementary Figure 441: ^{13}C spectra for **3ba**

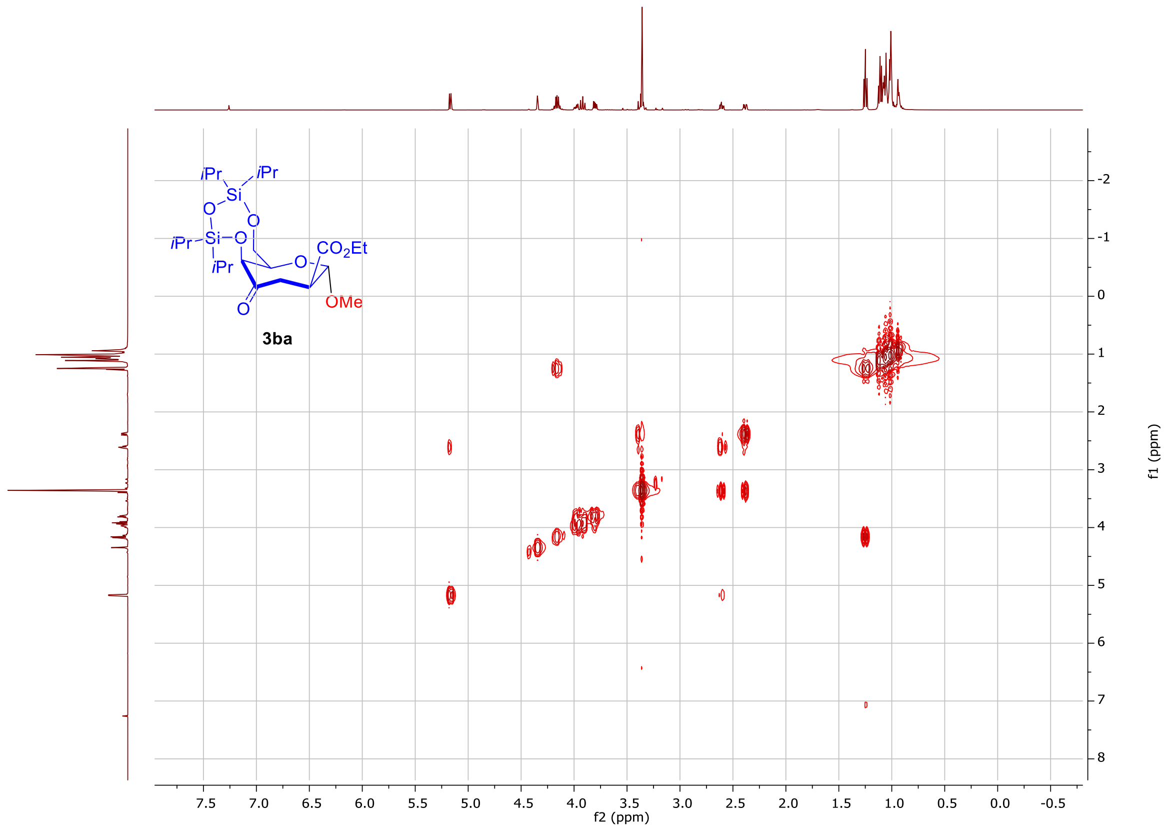

Supplementary Figure 442: COSY spectra for compound **3ba**

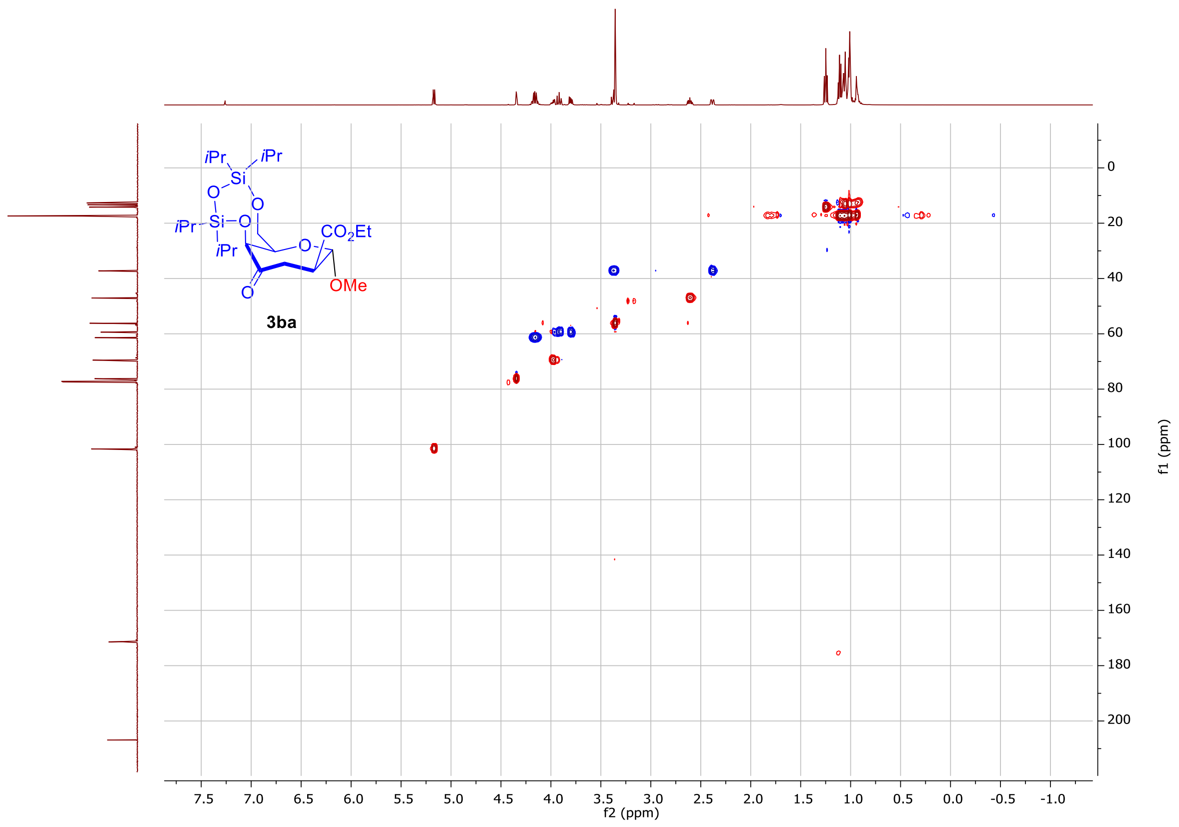

Supplementary Figure 443: HSQC spectra for compound **3ba**

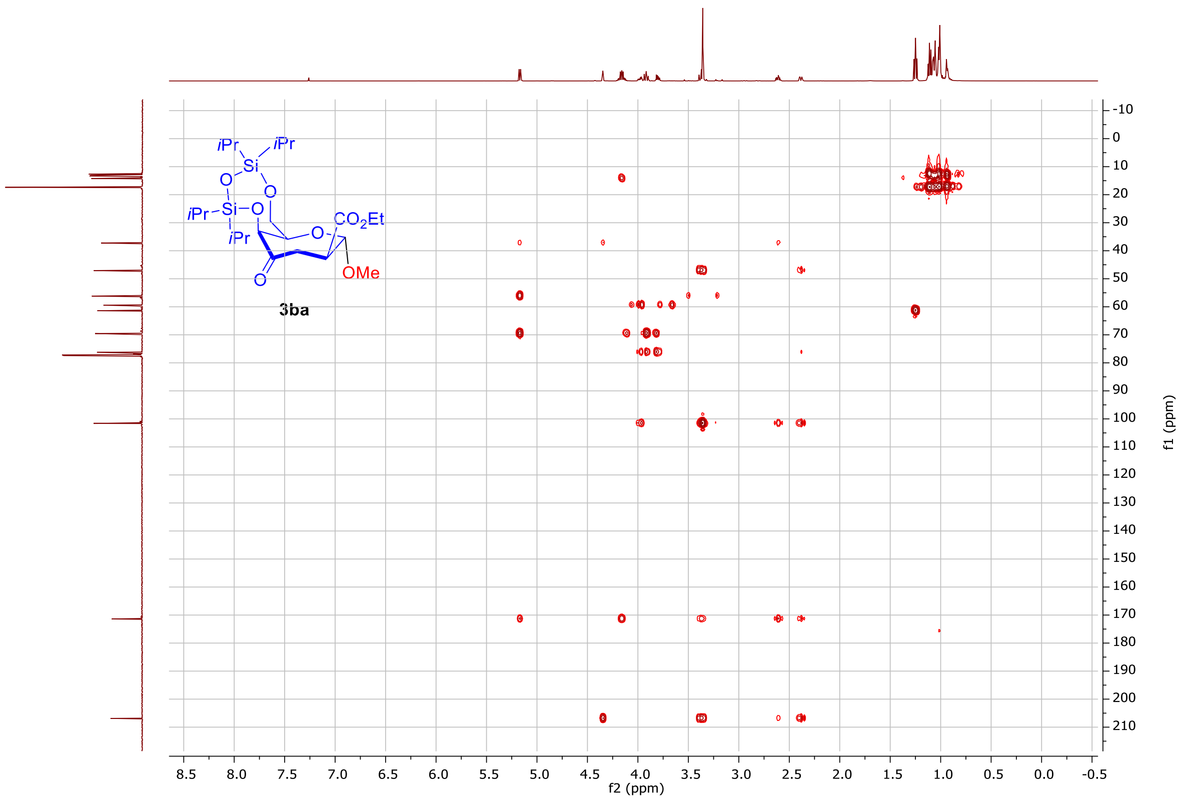

Supplementary Figure 444: HMBC spectra for compound **3ba**

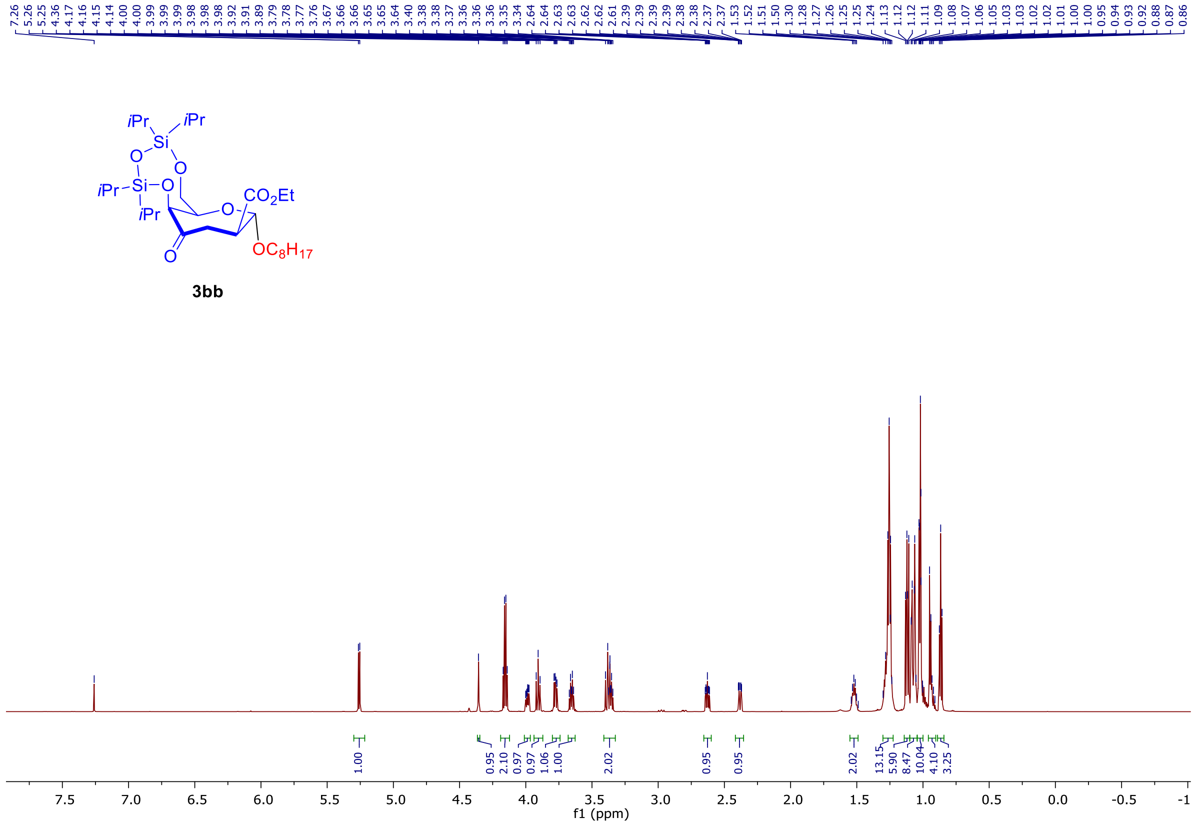

Supplementary Figure 445: ^{1}H spectra for **3bb**

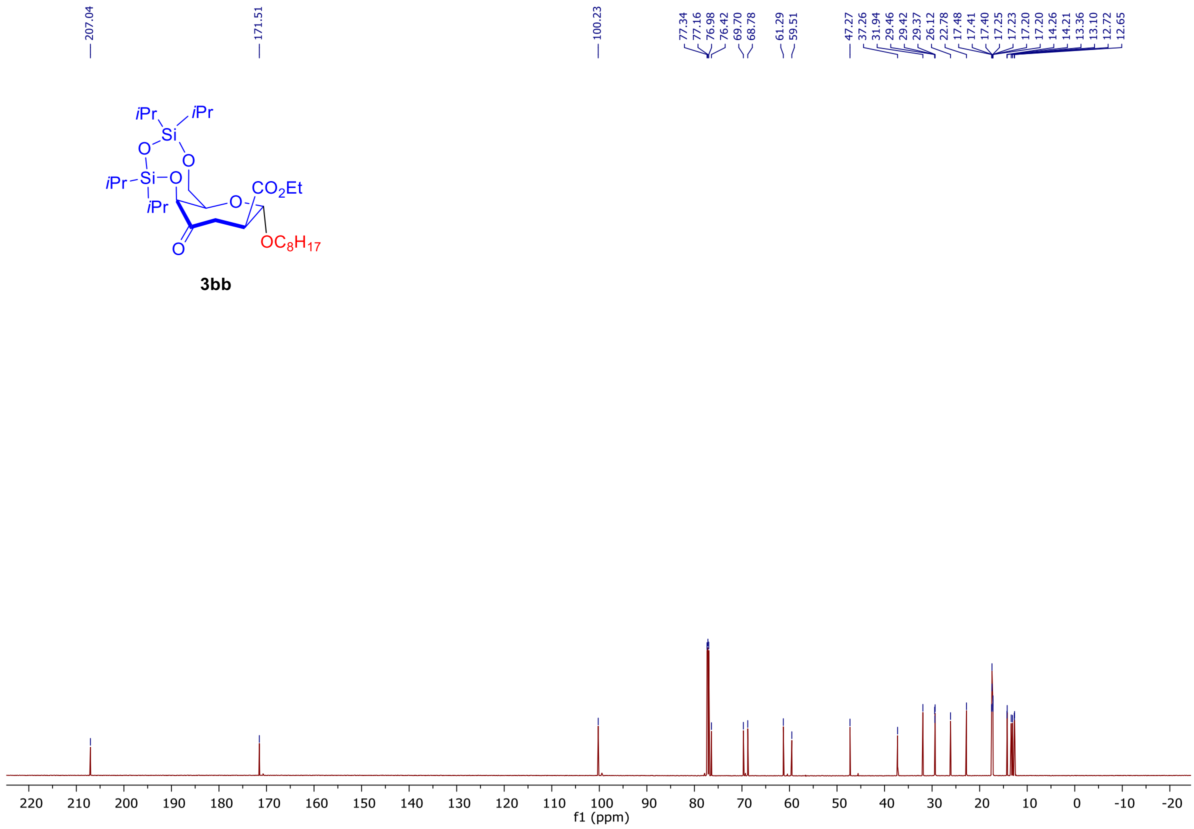

Supplementary Figure 446: ^{13}C spectra for **3bb**

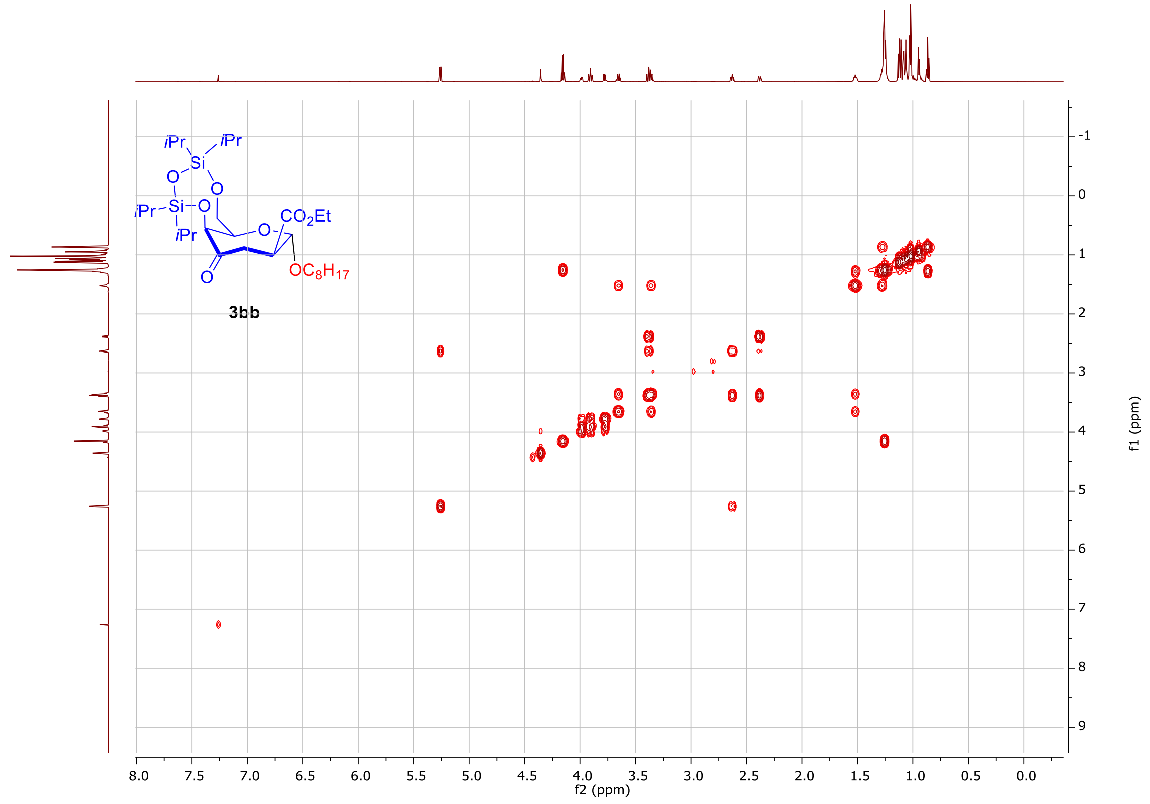

Supplementary Figure 447: COSY spectra for compound **3bb**

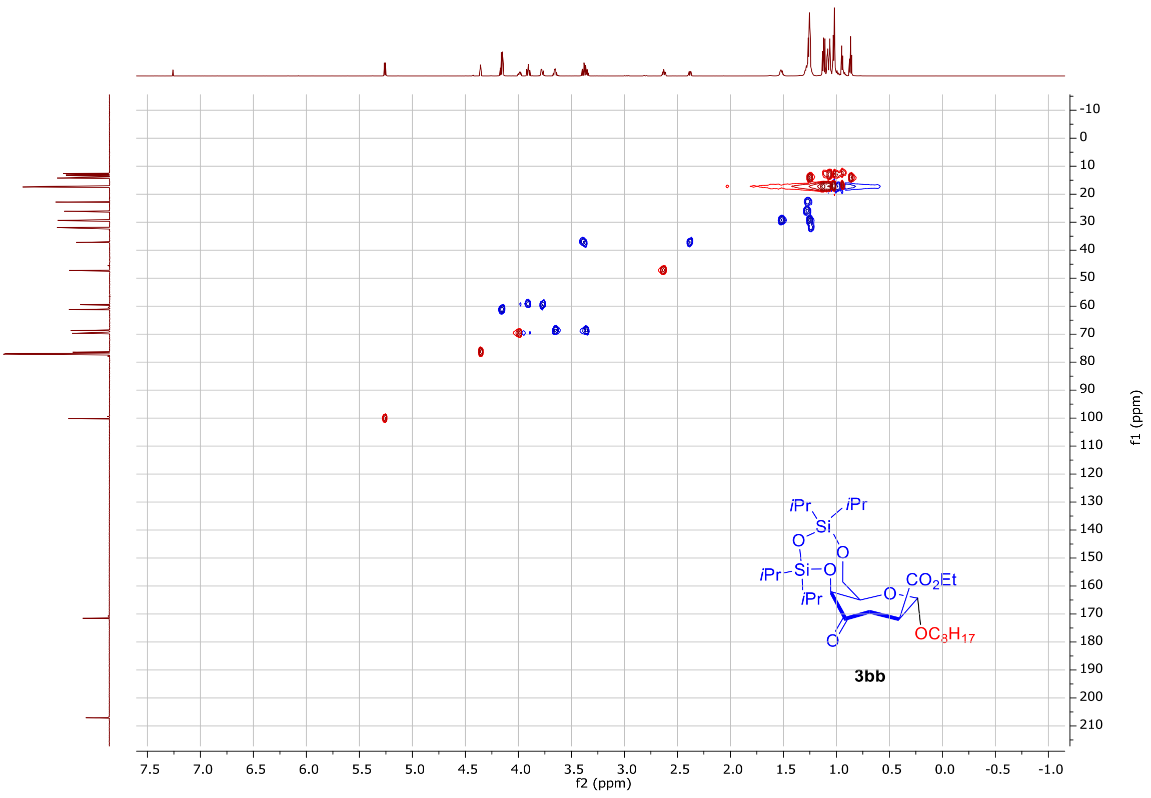

Supplementary Figure 448: HSQC spectra for compound **3bb**

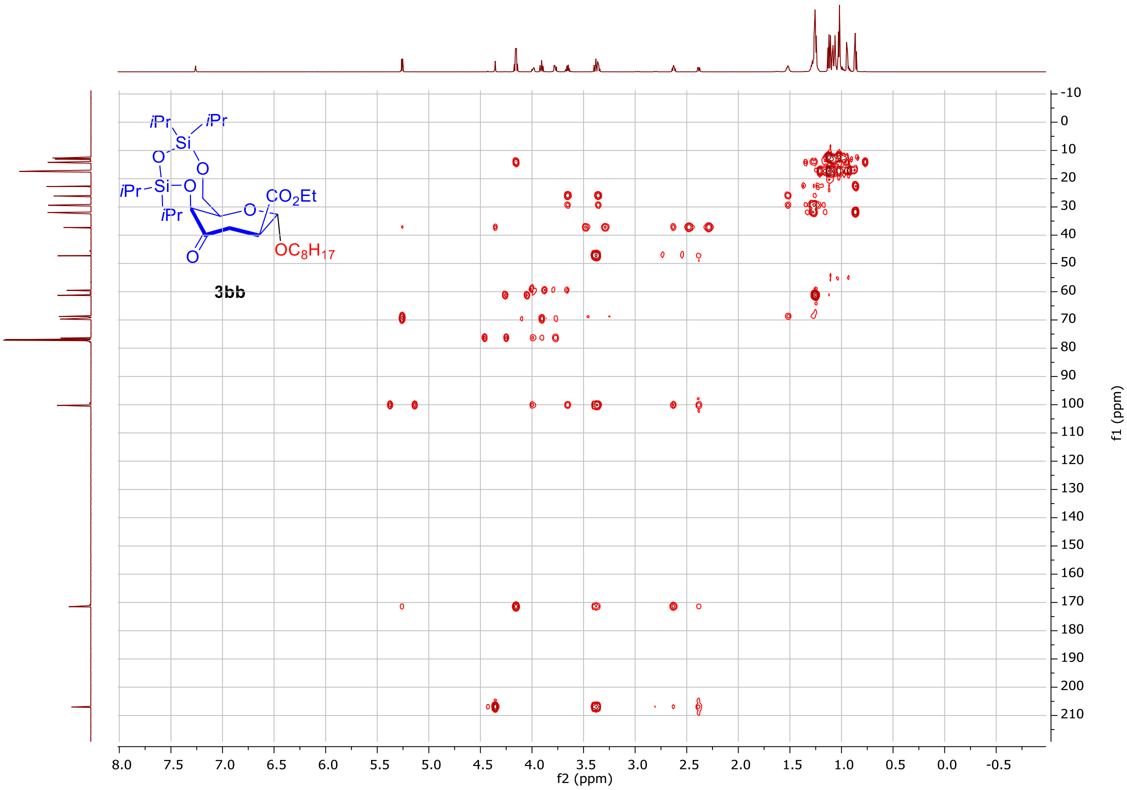

Supplementary Figure 449: HMBC spectra for compound **3bb**

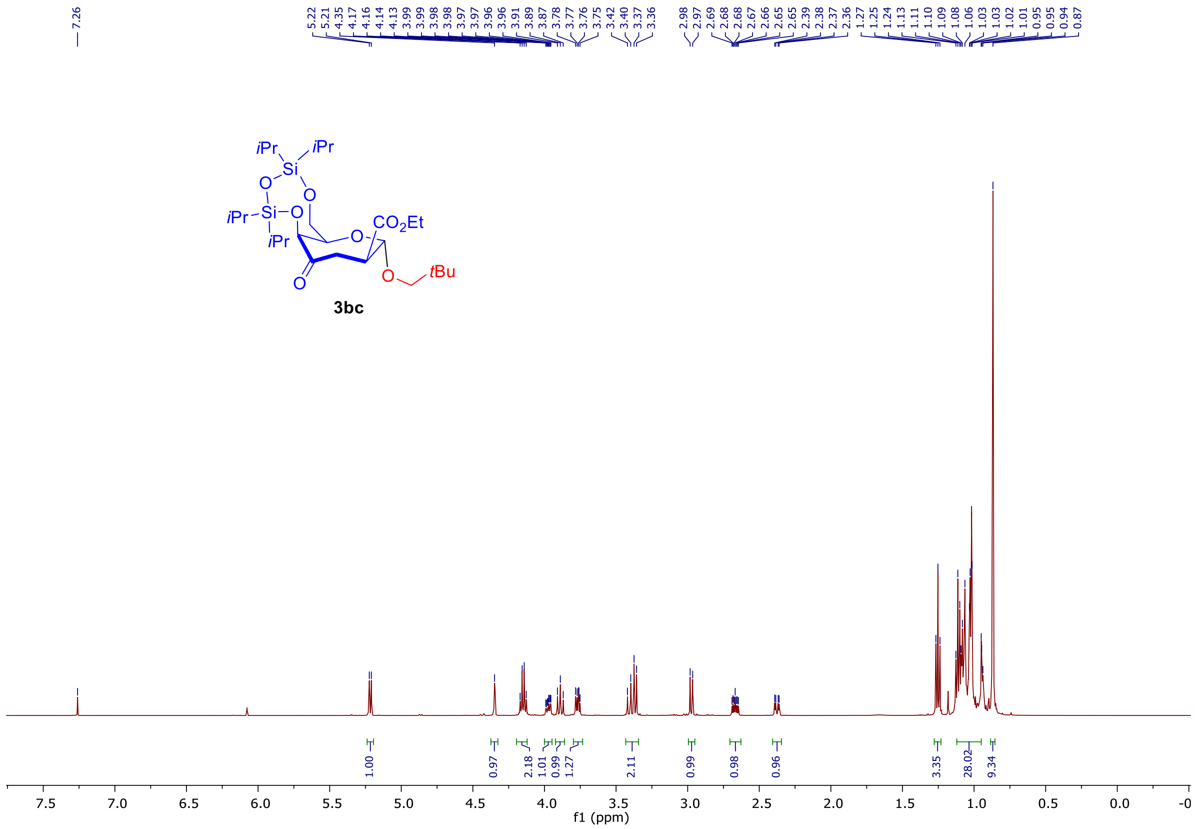

Supplementary Figure 450: ^{1}H spectra for **3bc**

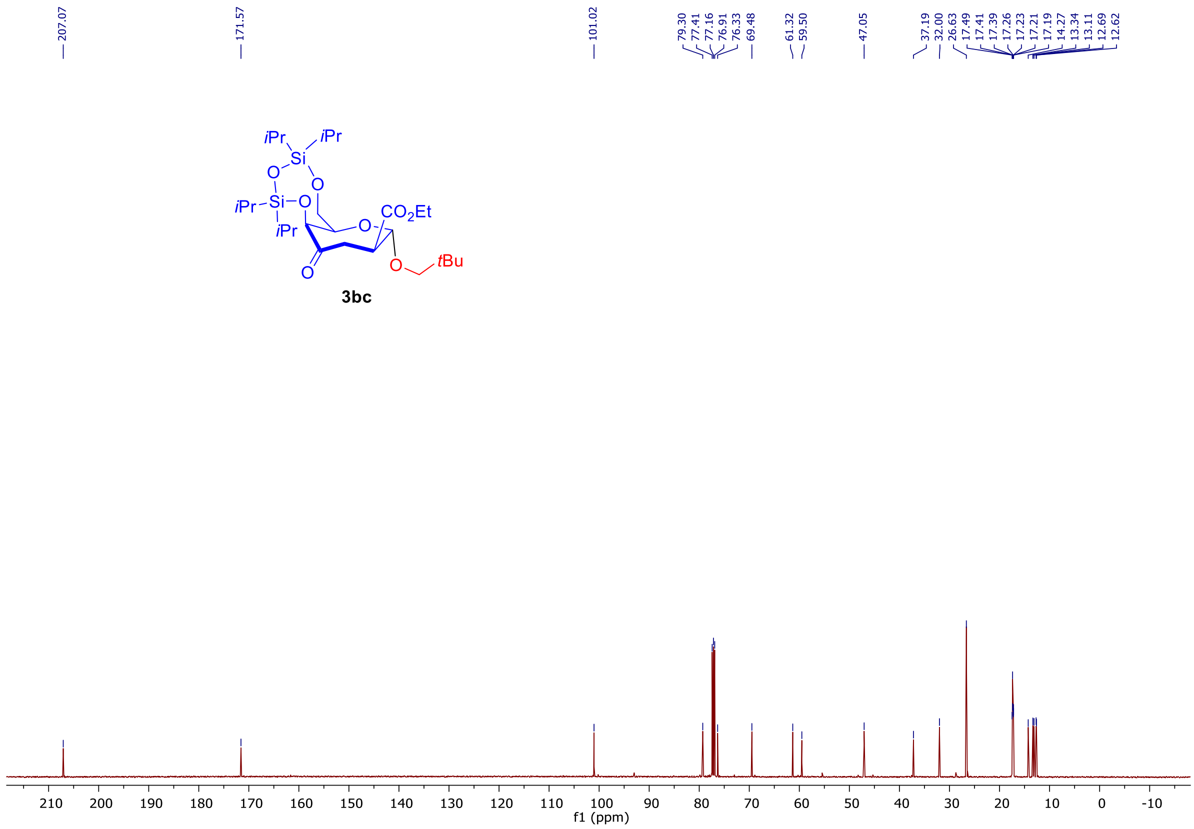

Supplementary Figure 451: ^{13}C spectra for **3bc**

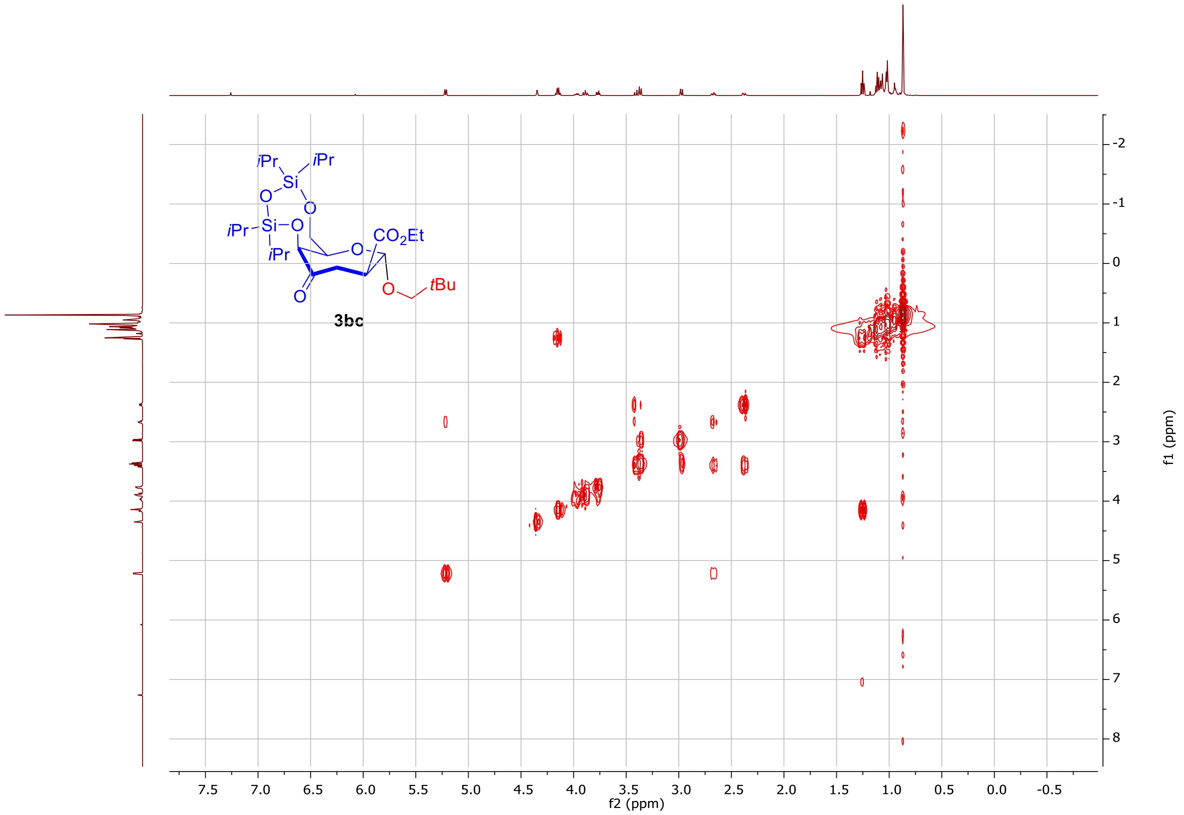

Supplementary Figure 452: COSY spectra for compound **3bc**

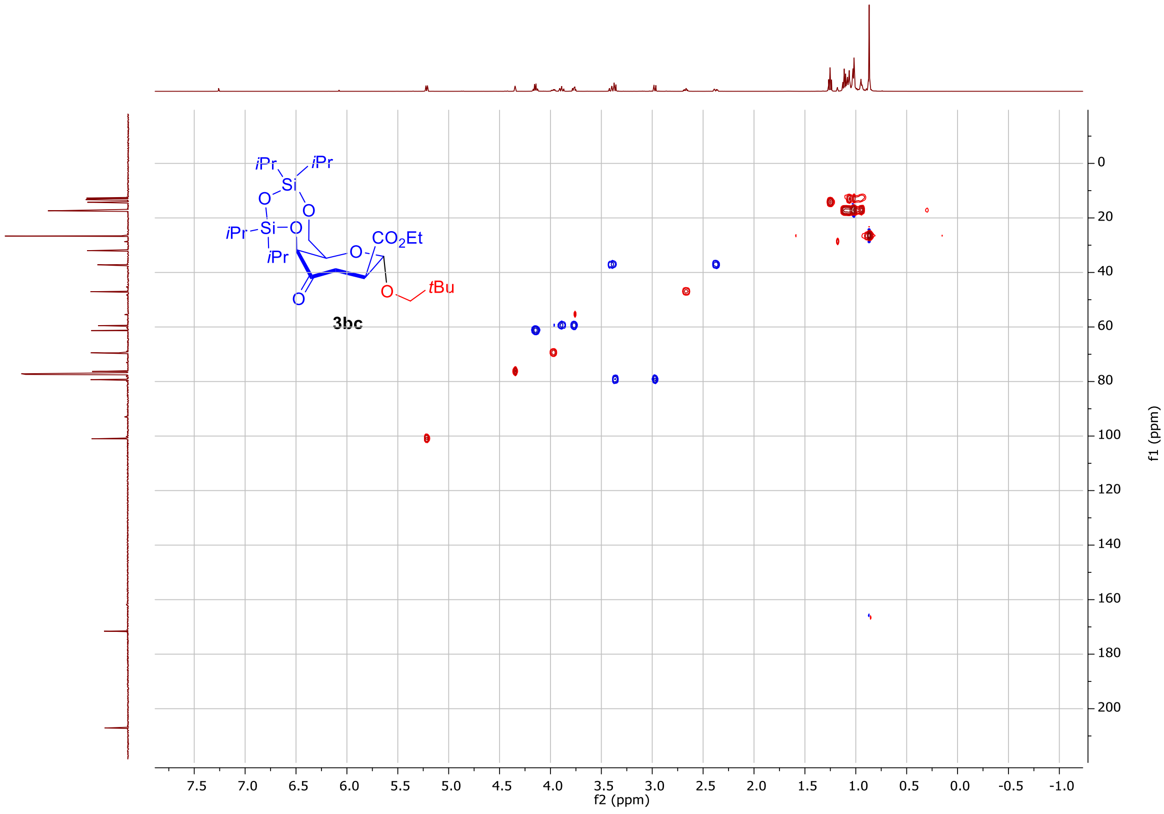

Supplementary Figure 453: HSQC spectra for compound **3bc**

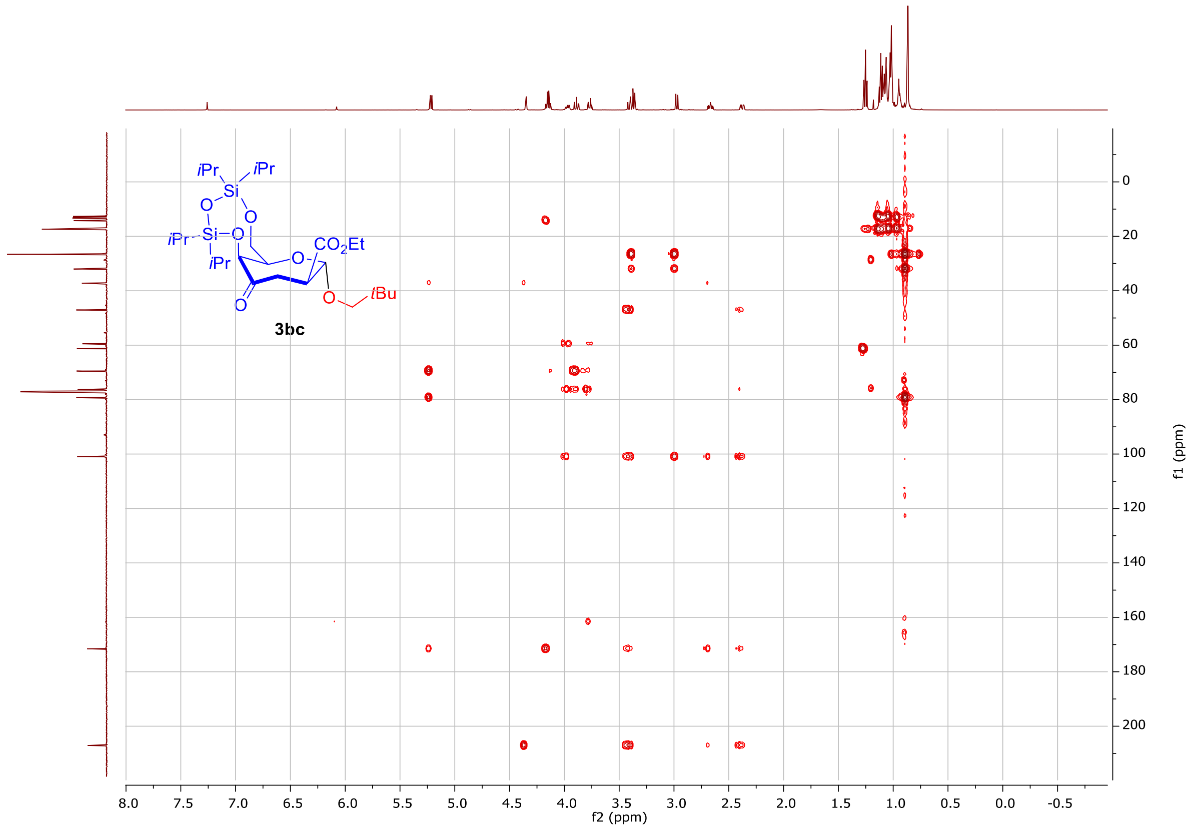

Supplementary Figure 454: HMBC spectra for compound **3bc**

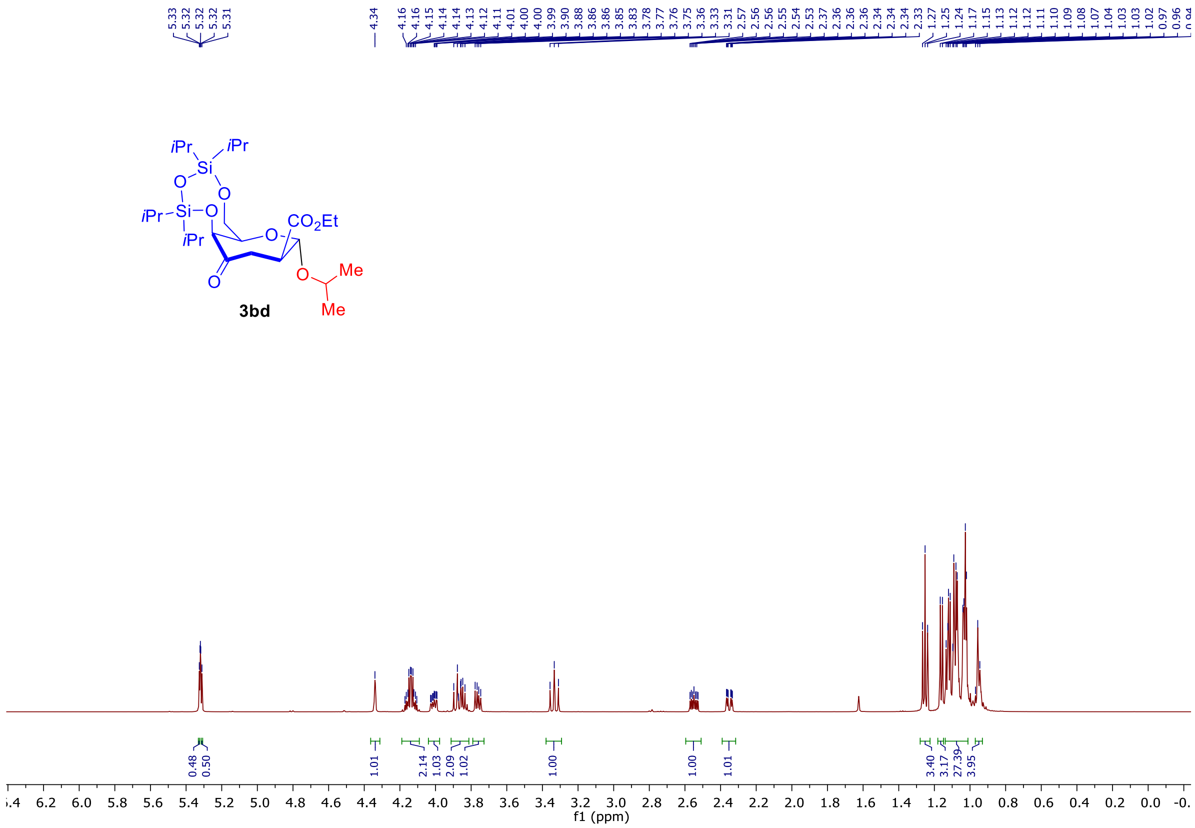

Supplementary Figure 455: ^{1}H spectra for **3bd**

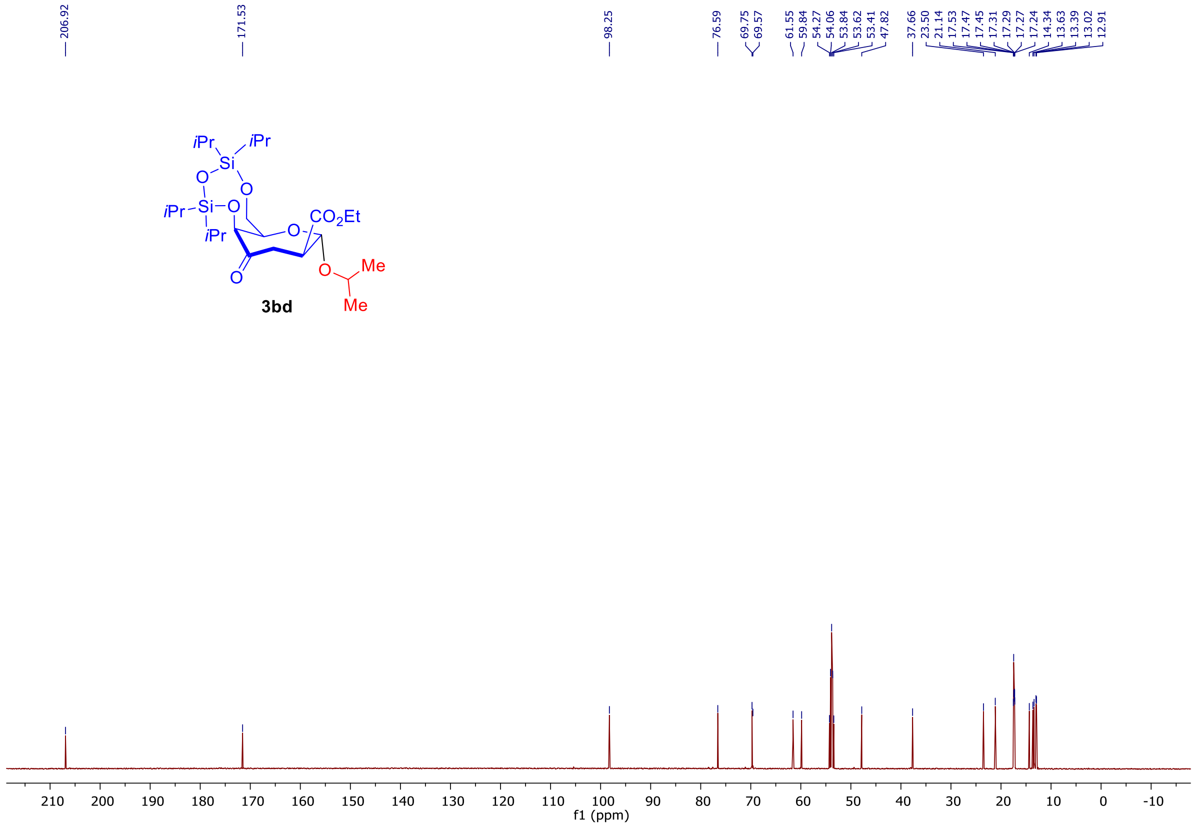

Supplementary Figure 456: ^{13}C spectra for **3bd**

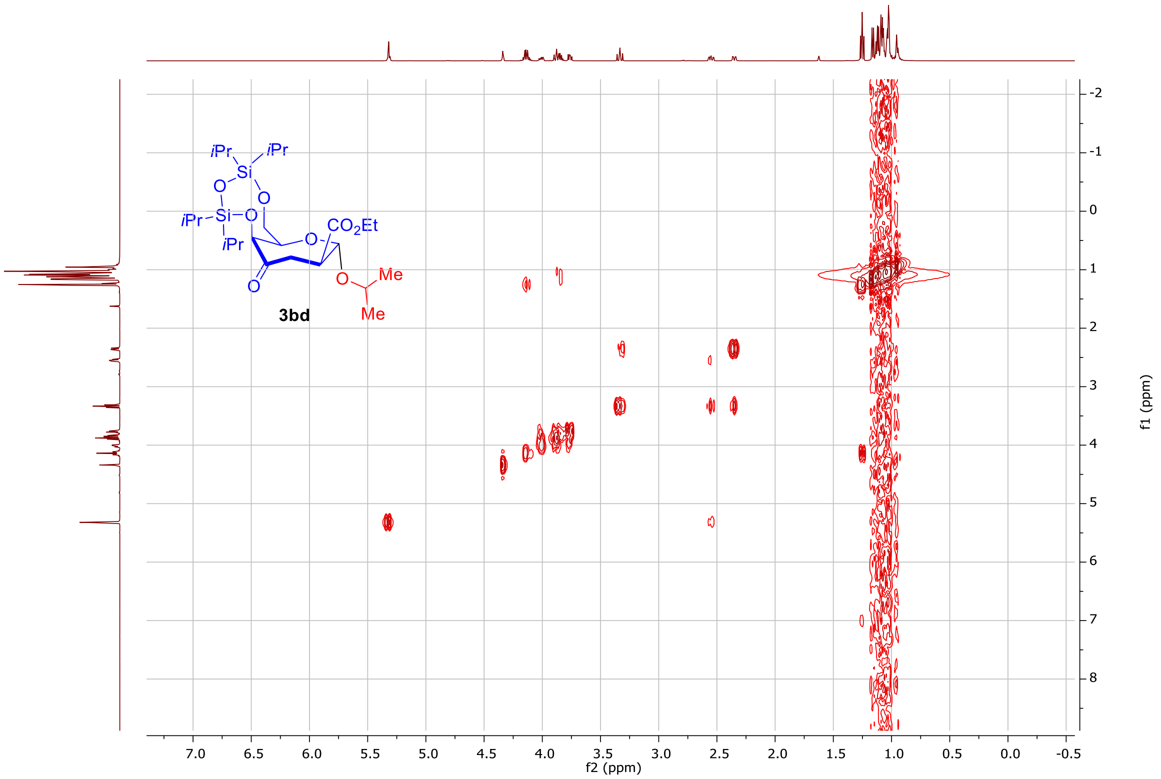

Supplementary Figure 457: COSY spectra for compound **3bd**

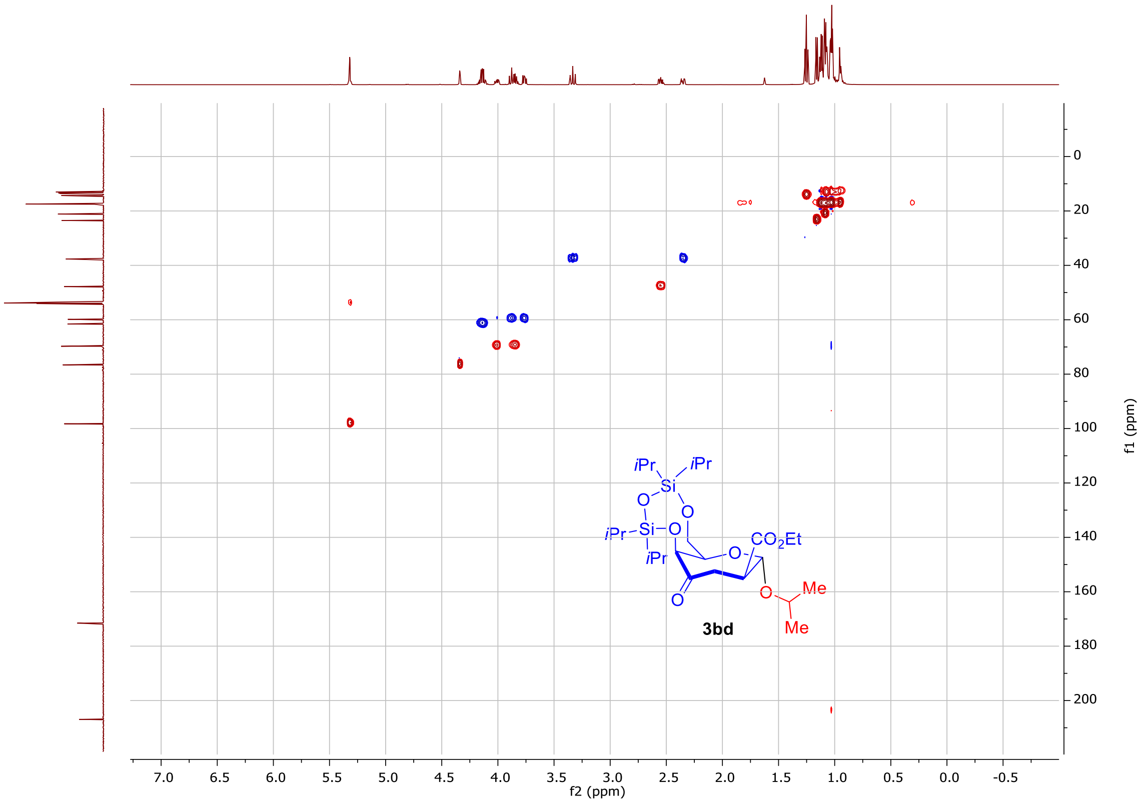

Supplementary Figure 458: HSQC spectra for compound **3bd**

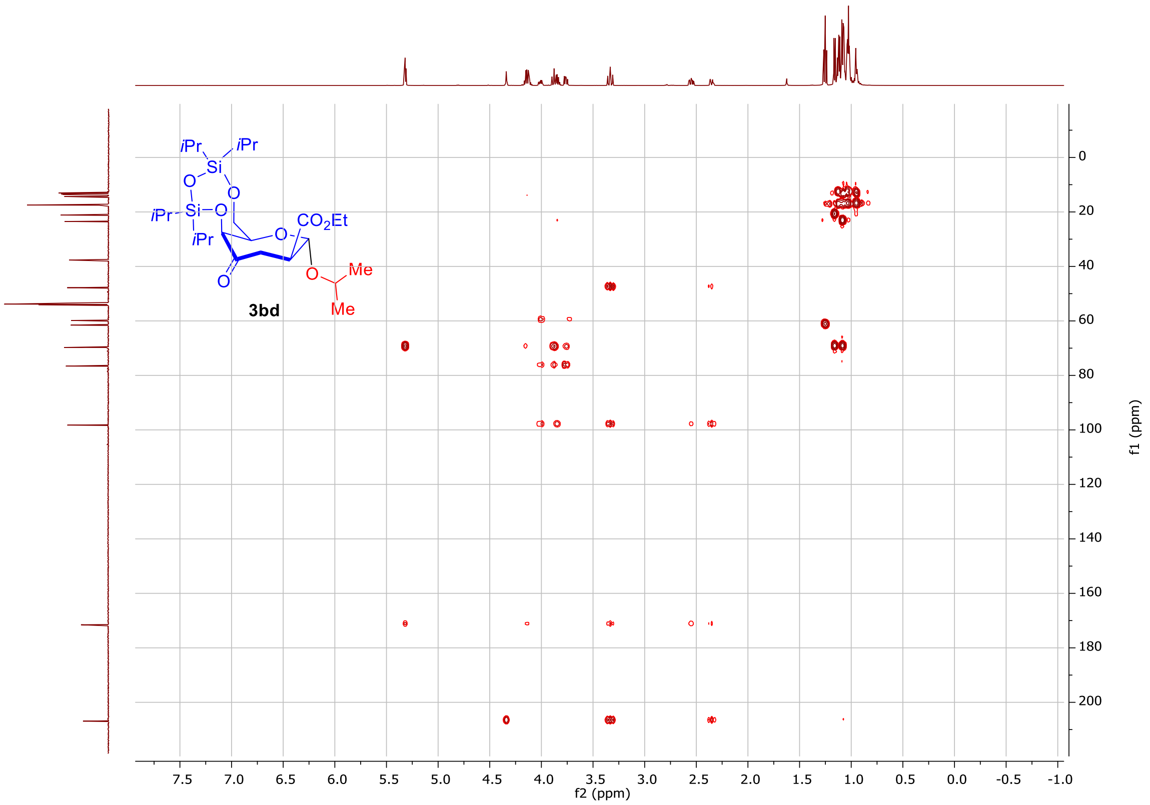

Supplementary Figure 459: HMBC spectra for compound **3bd**

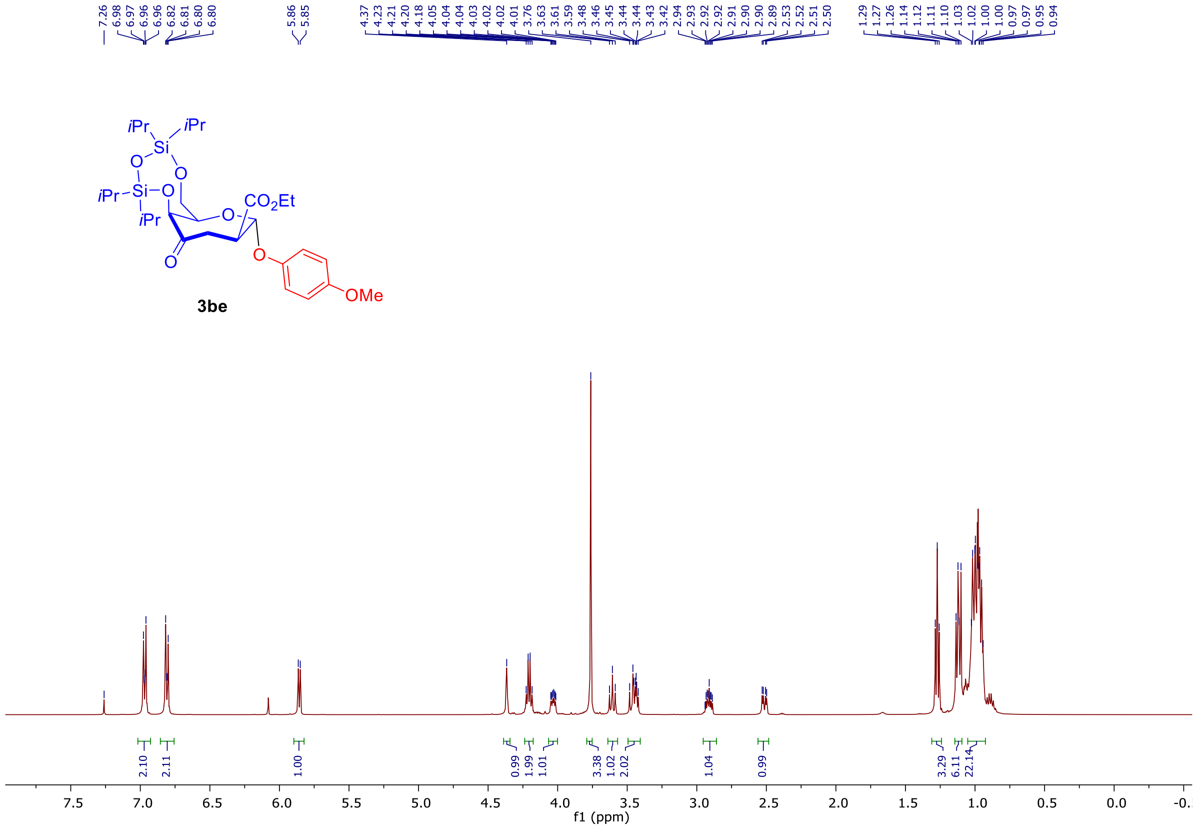

Supplementary Figure 460: ^{1}H spectra for **3be**

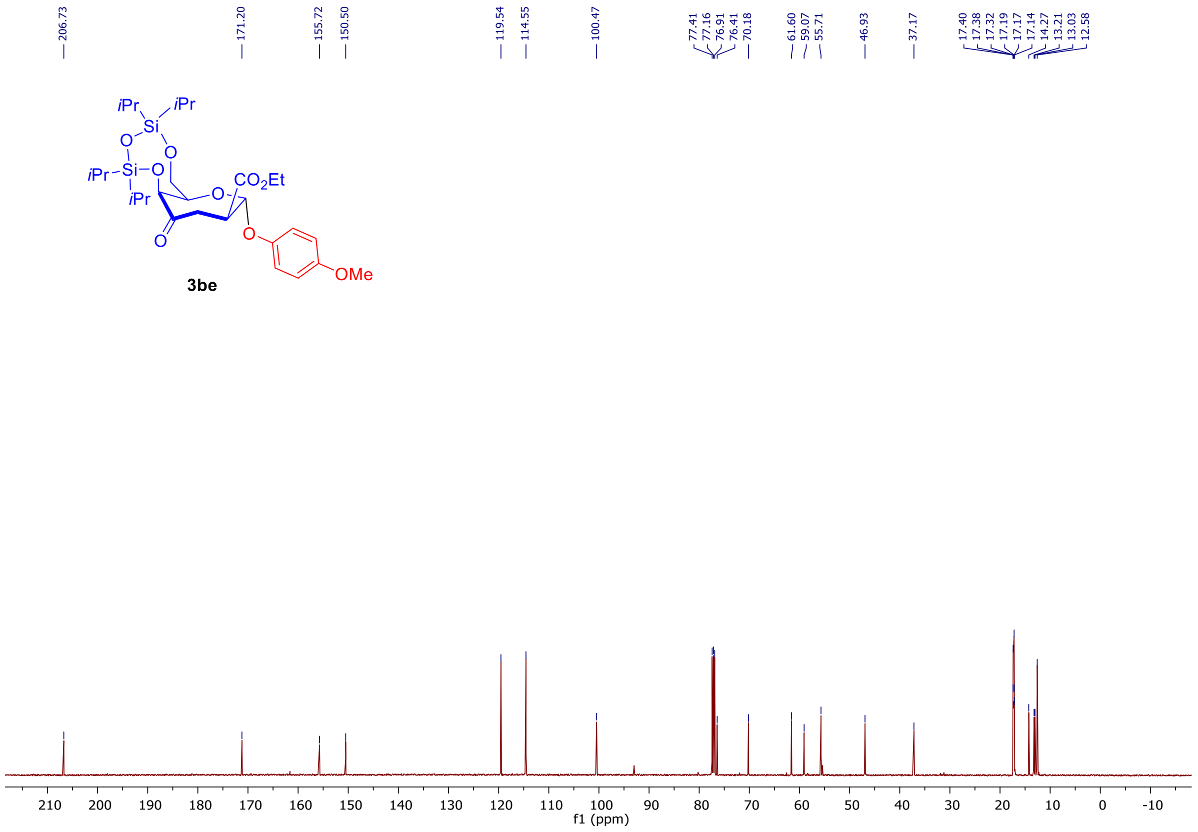

Supplementary Figure 461: ^{13}C spectra for **3be**

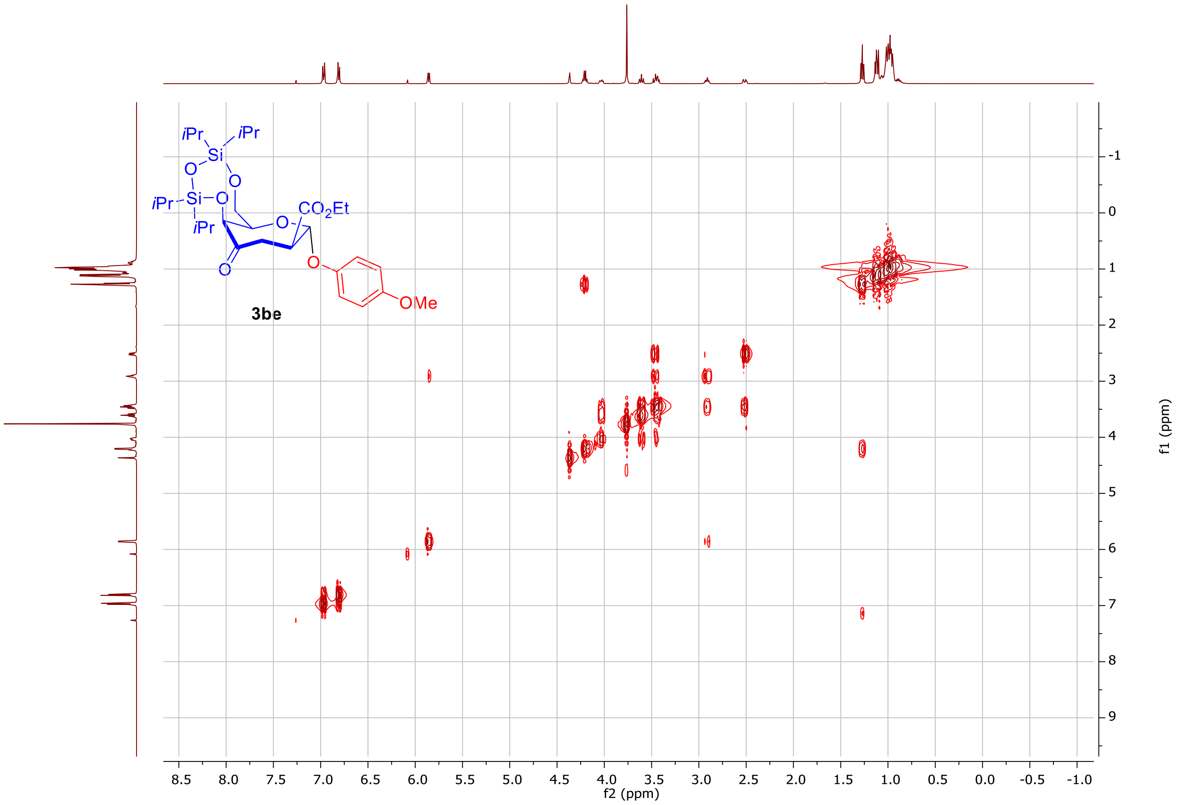

Supplementary Figure 462: COSY spectra for compound **3be**

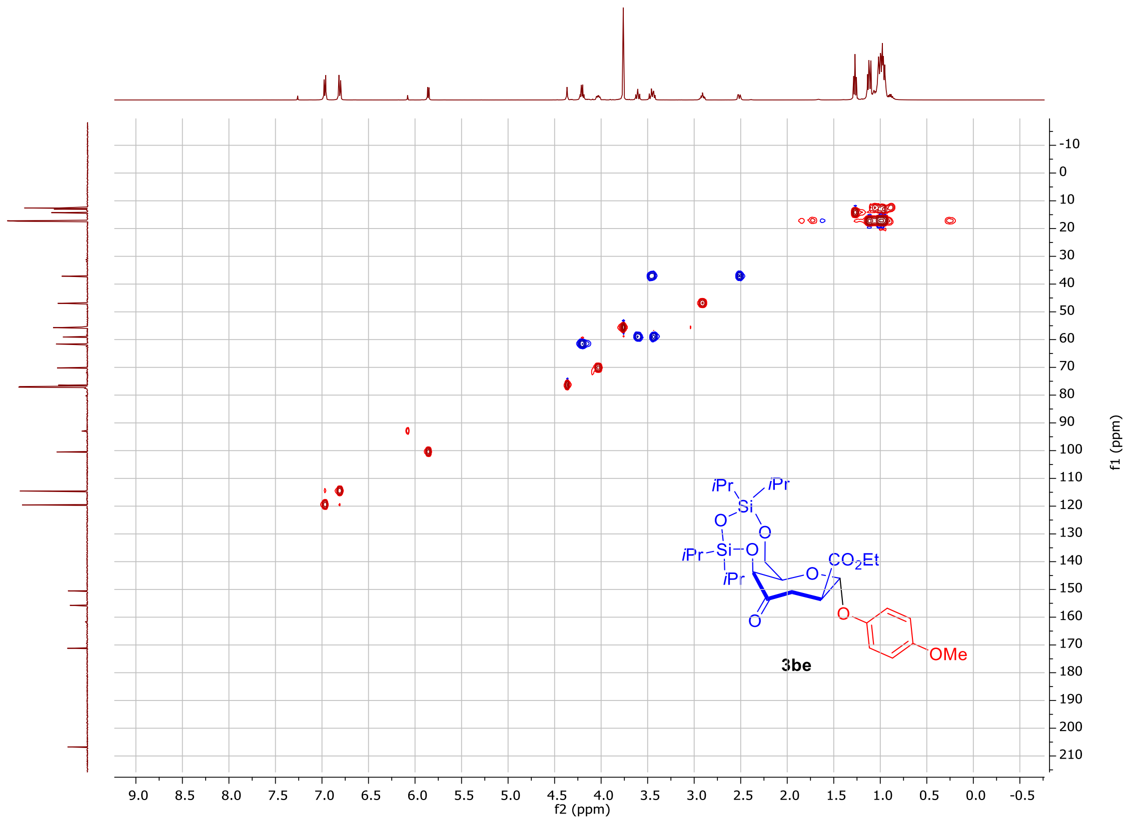

Supplementary Figure 463: HSQC spectra for compound **3be**

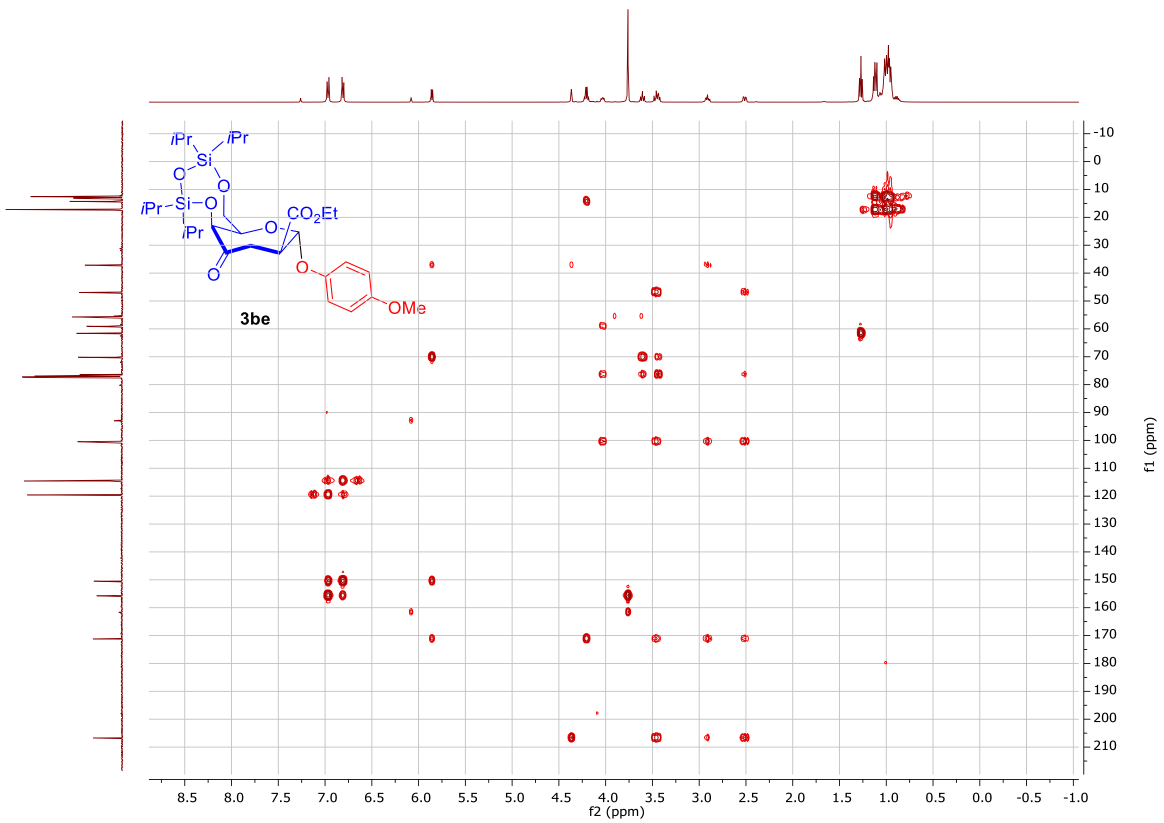

Supplementary Figure 464: HMBC spectra for compound **3be**

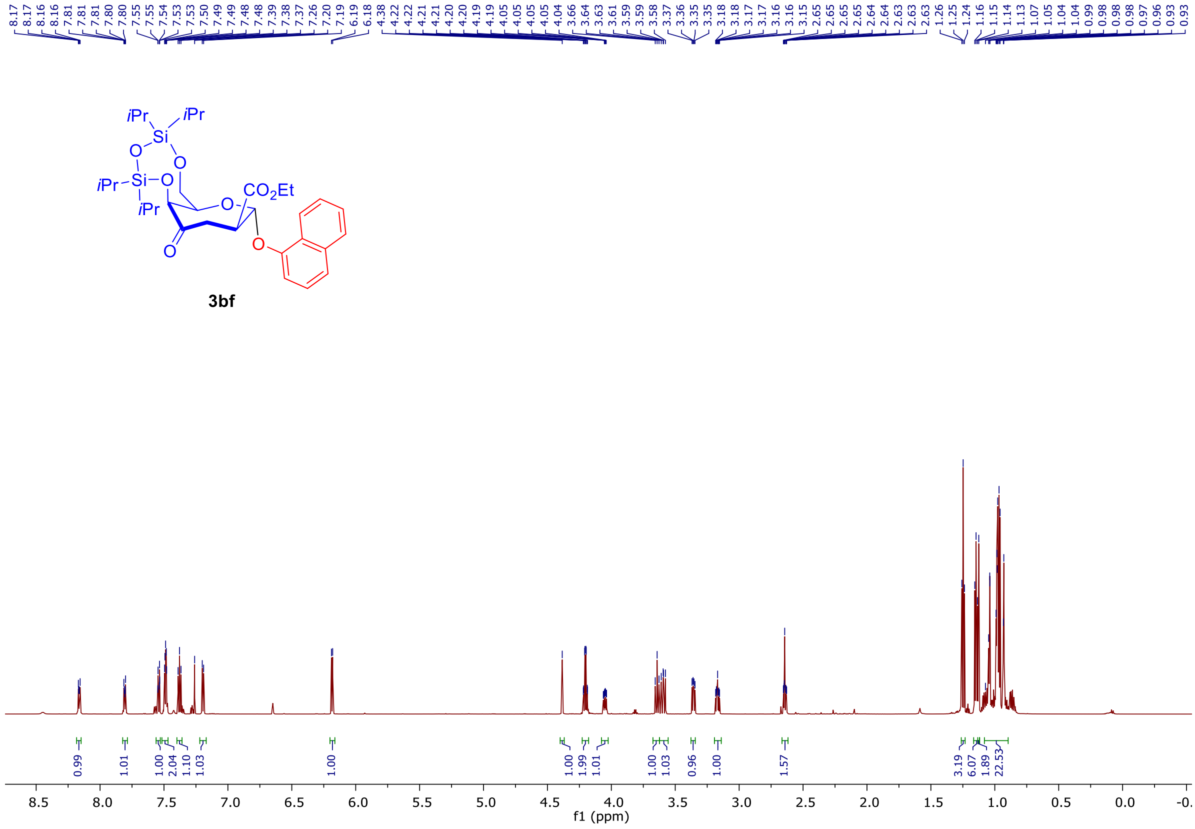

Supplementary Figure 465: ^{1}H spectra for **3bf**

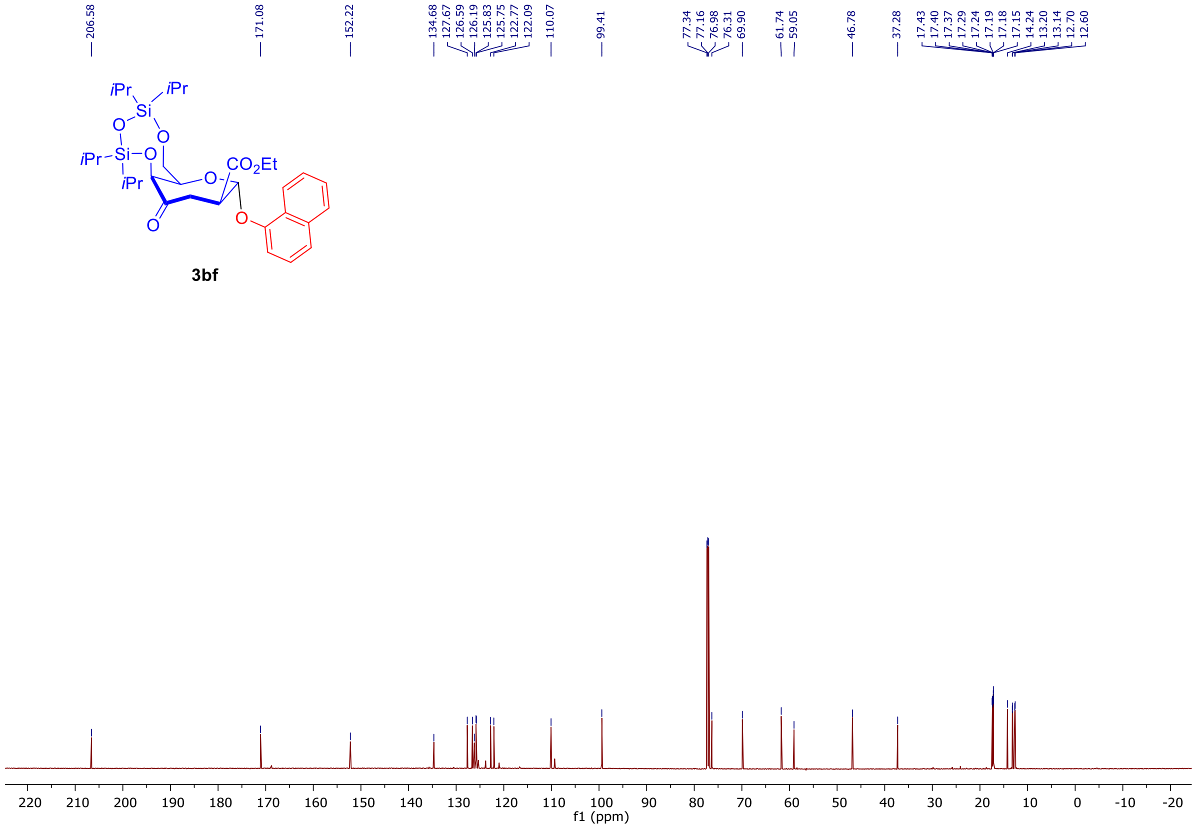

Supplementary Figure 466: ^{13}C spectra for **3bf**

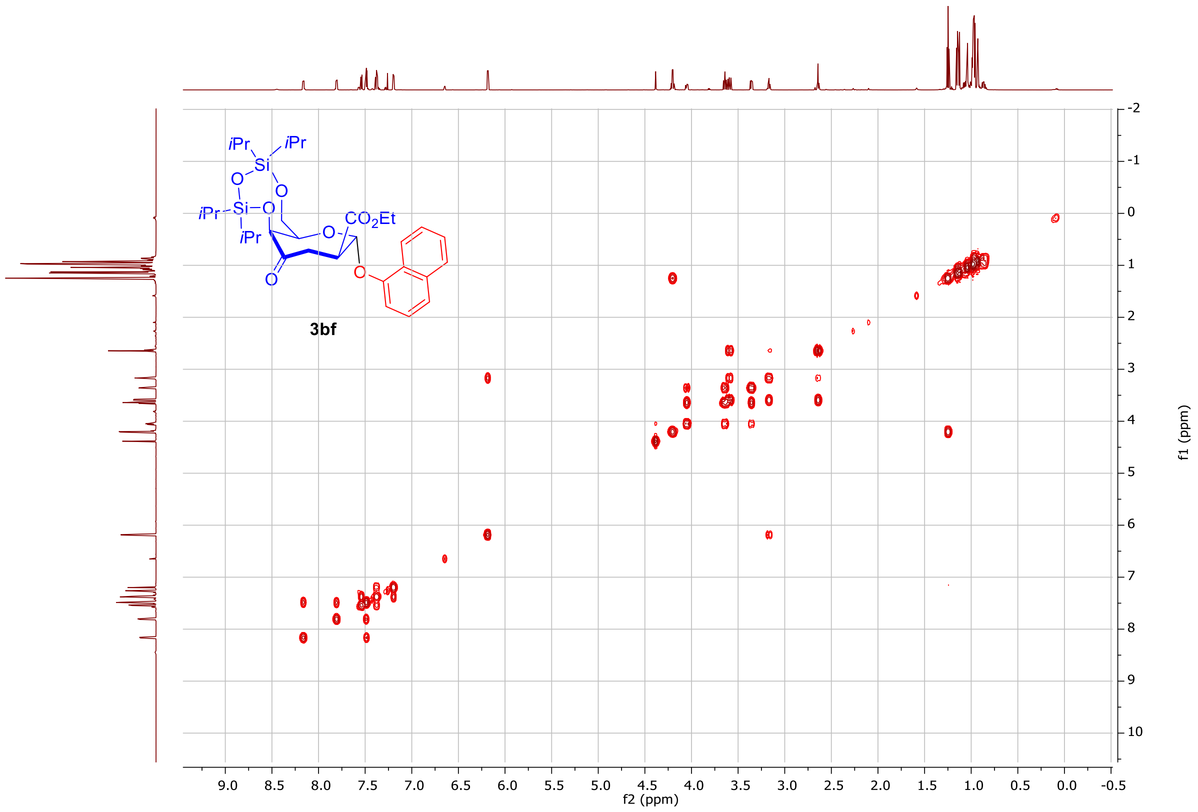

Supplementary Figure 467: COSY spectra for compound **3bf**

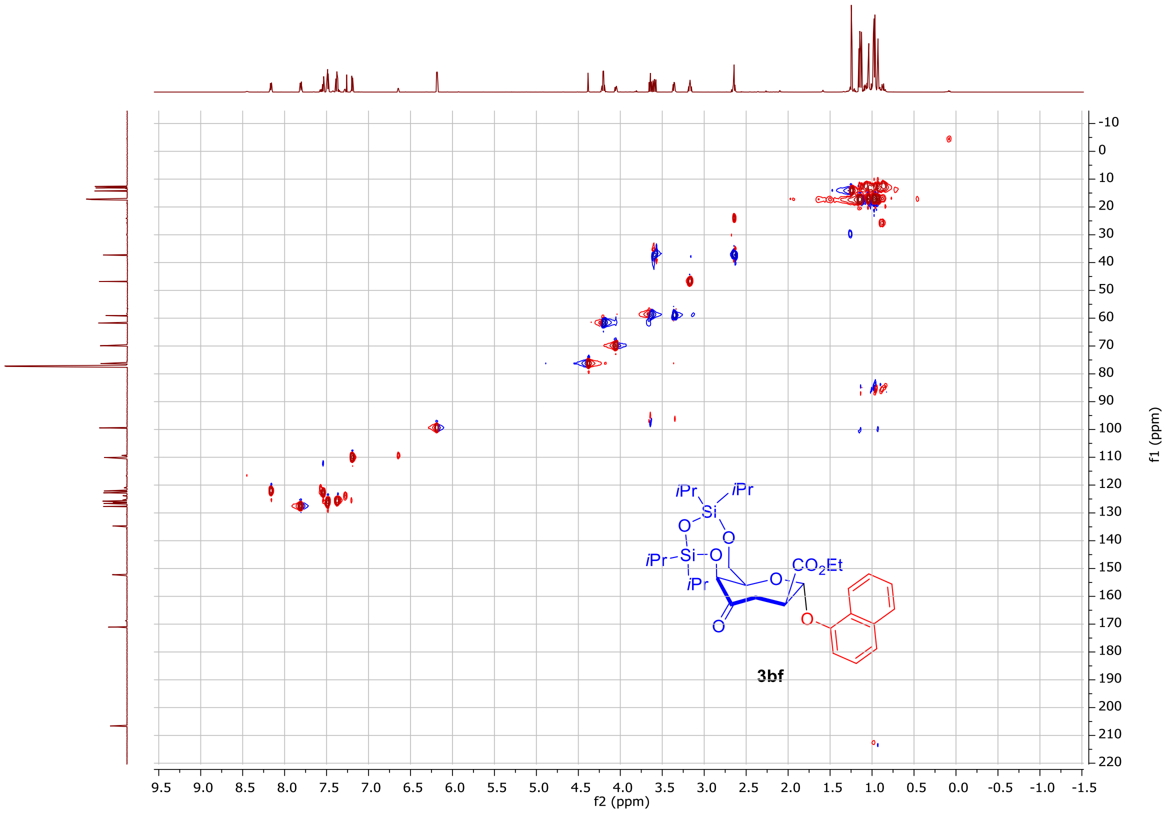

Supplementary Figure 468: HSQC spectra for compound **3bf**

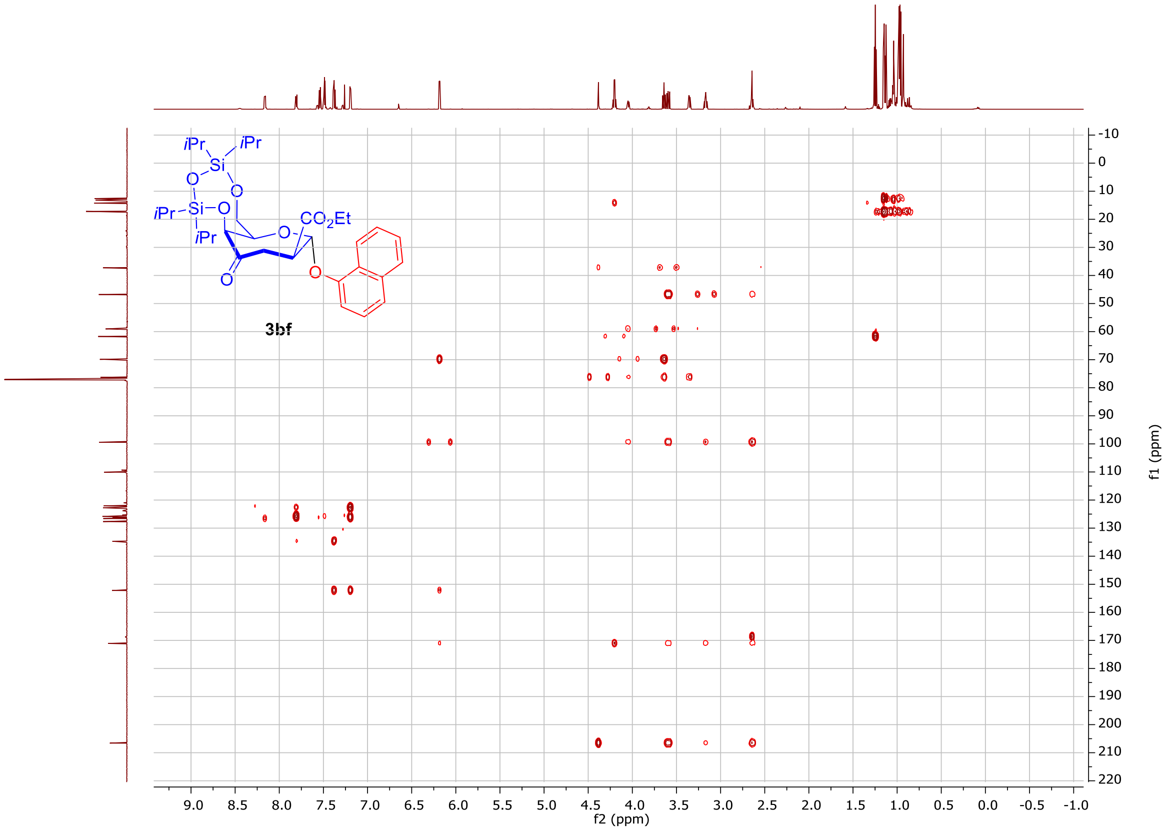

Supplementary Figure 469: HMBC spectra for compound **3bf**

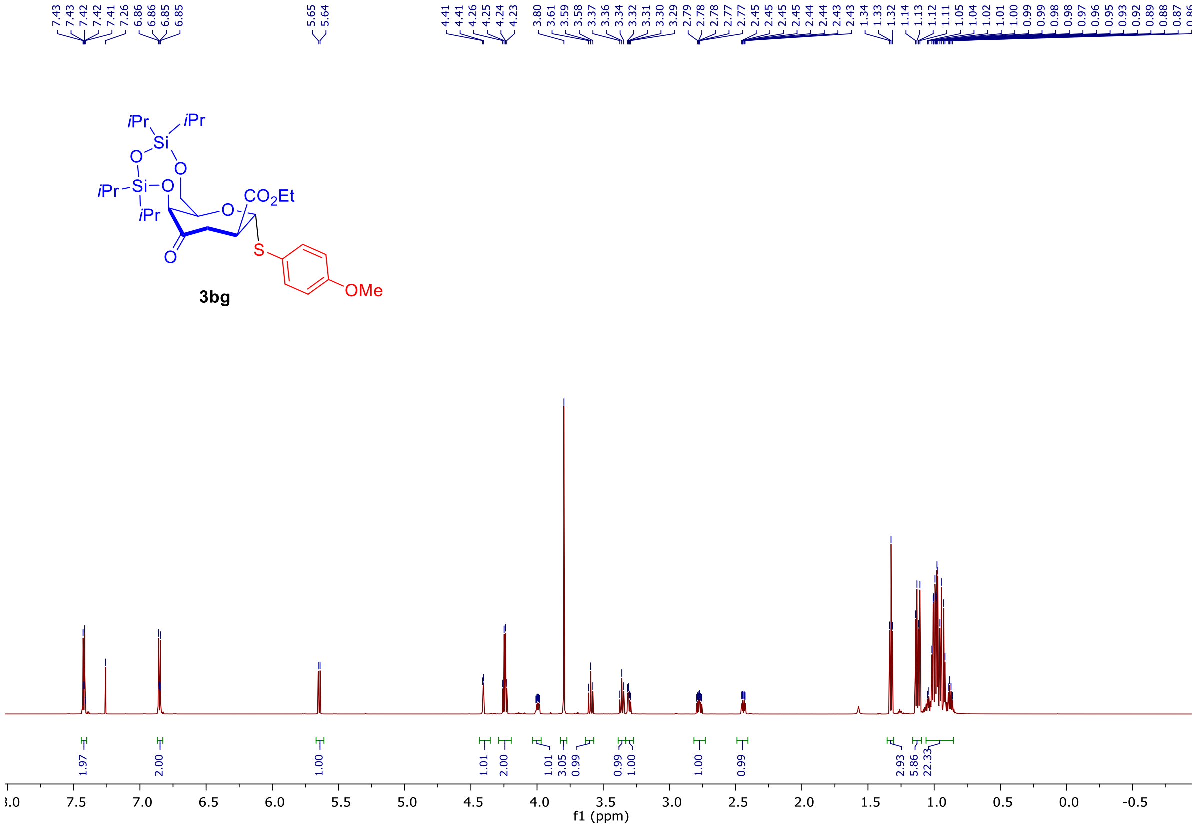

Supplementary Figure 470: ^{1}H spectra for **3bg**

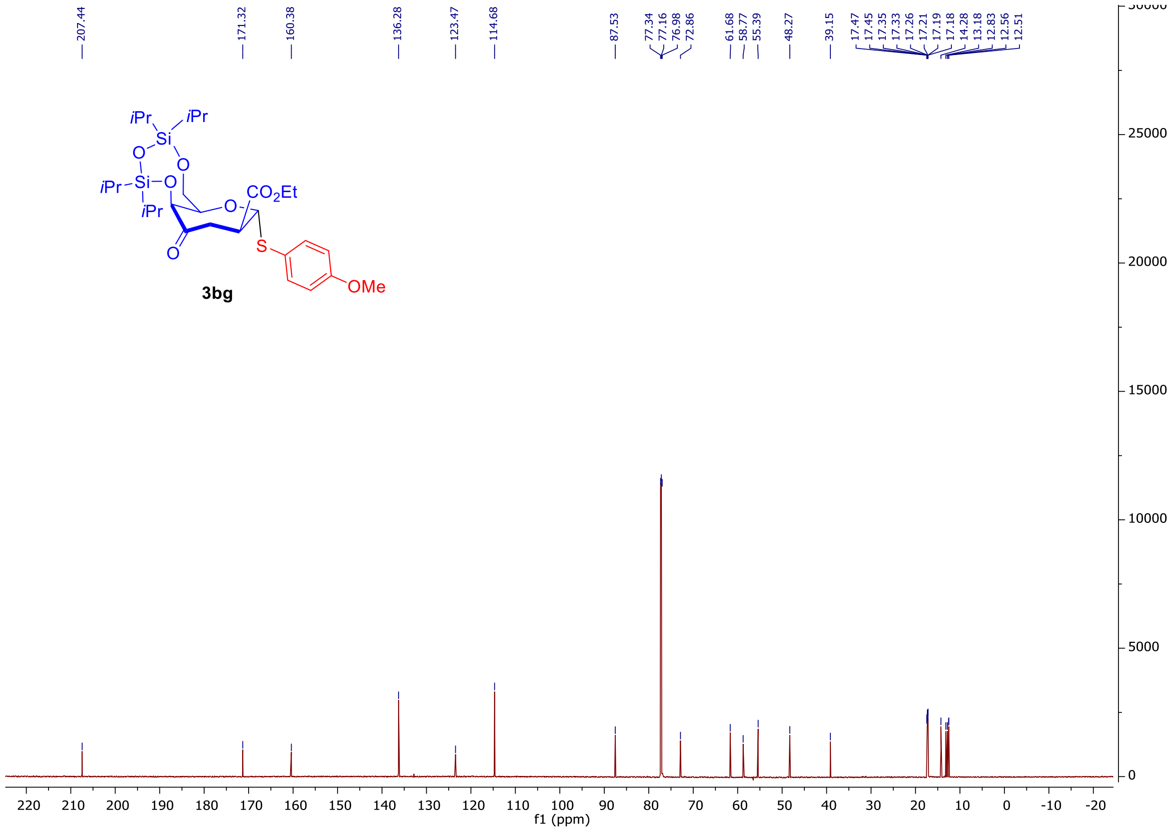

Supplementary Figure 471: ^{13}C spectra for **3bg**

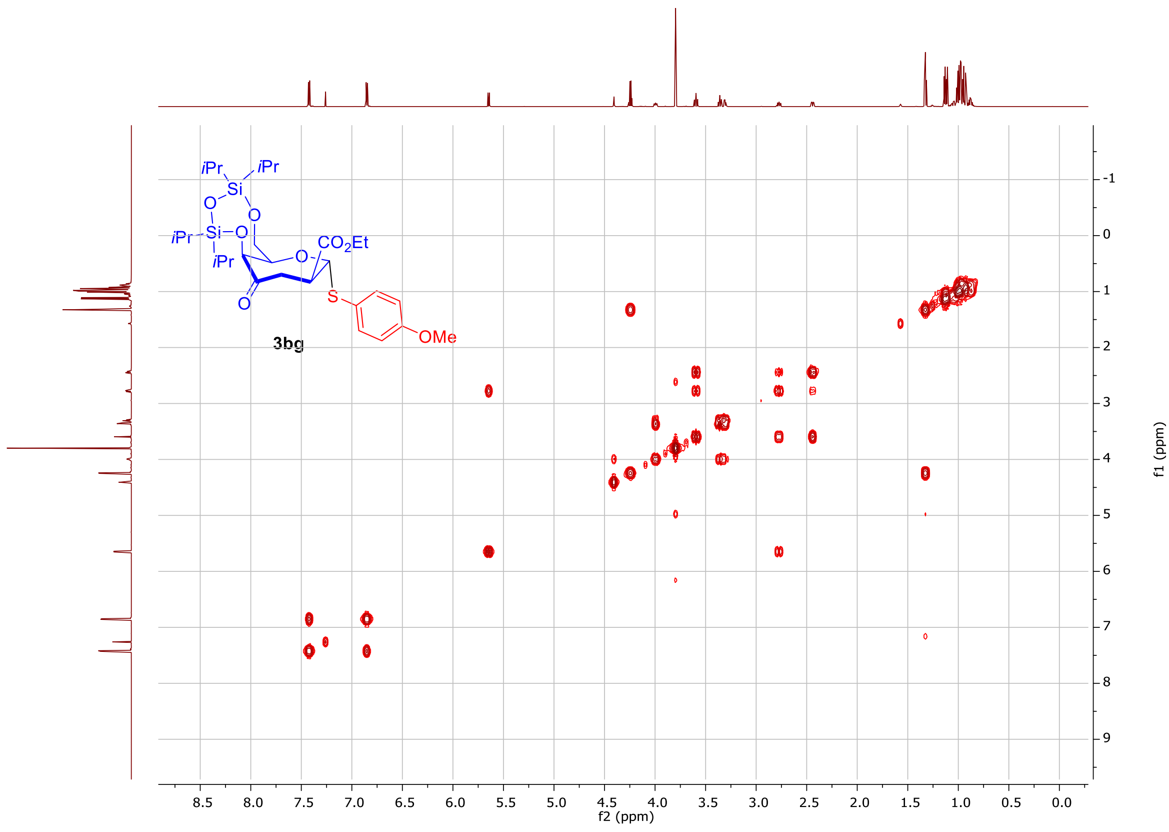

Supplementary Figure 472: COSY spectra for compound **3bg**

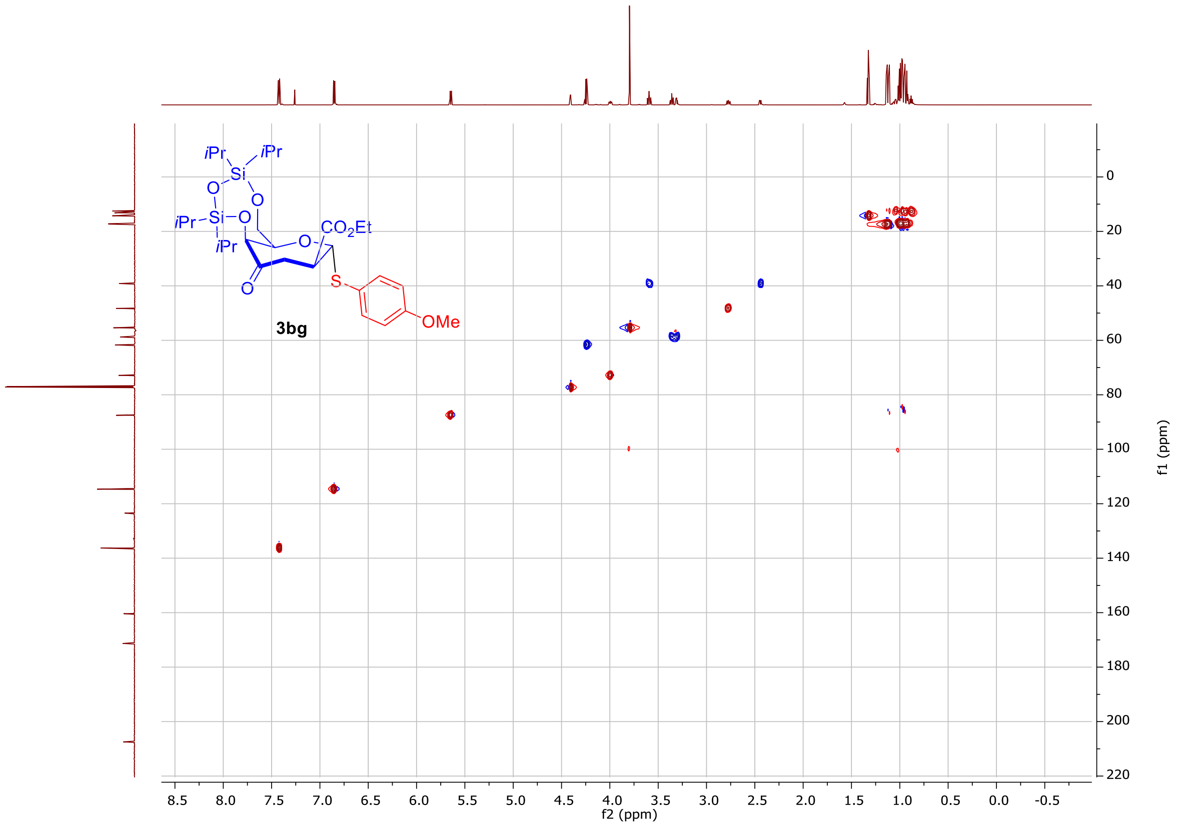

Supplementary Figure 473: HSQC spectra for compound **3bg**

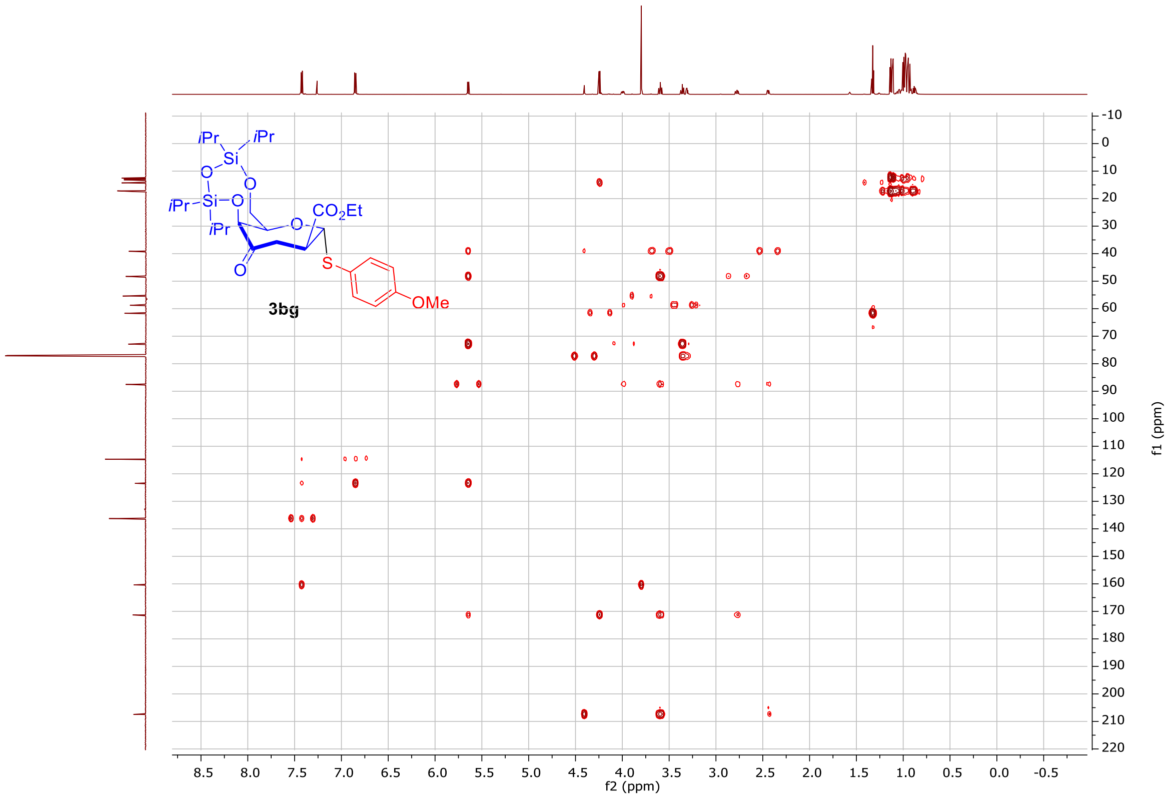

Supplementary Figure 474: HMBC spectra for compound **3bg**

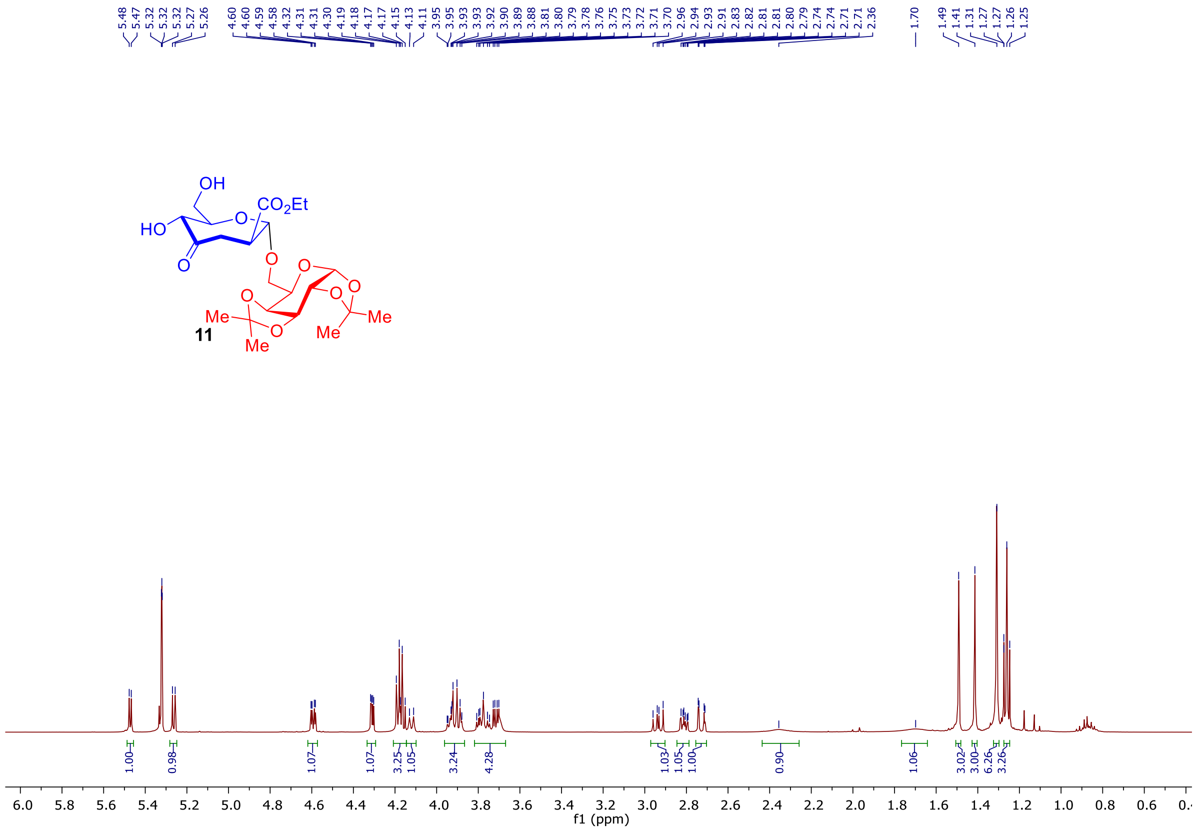

Supplementary Figure 475: ^{1}H spectra for **11**

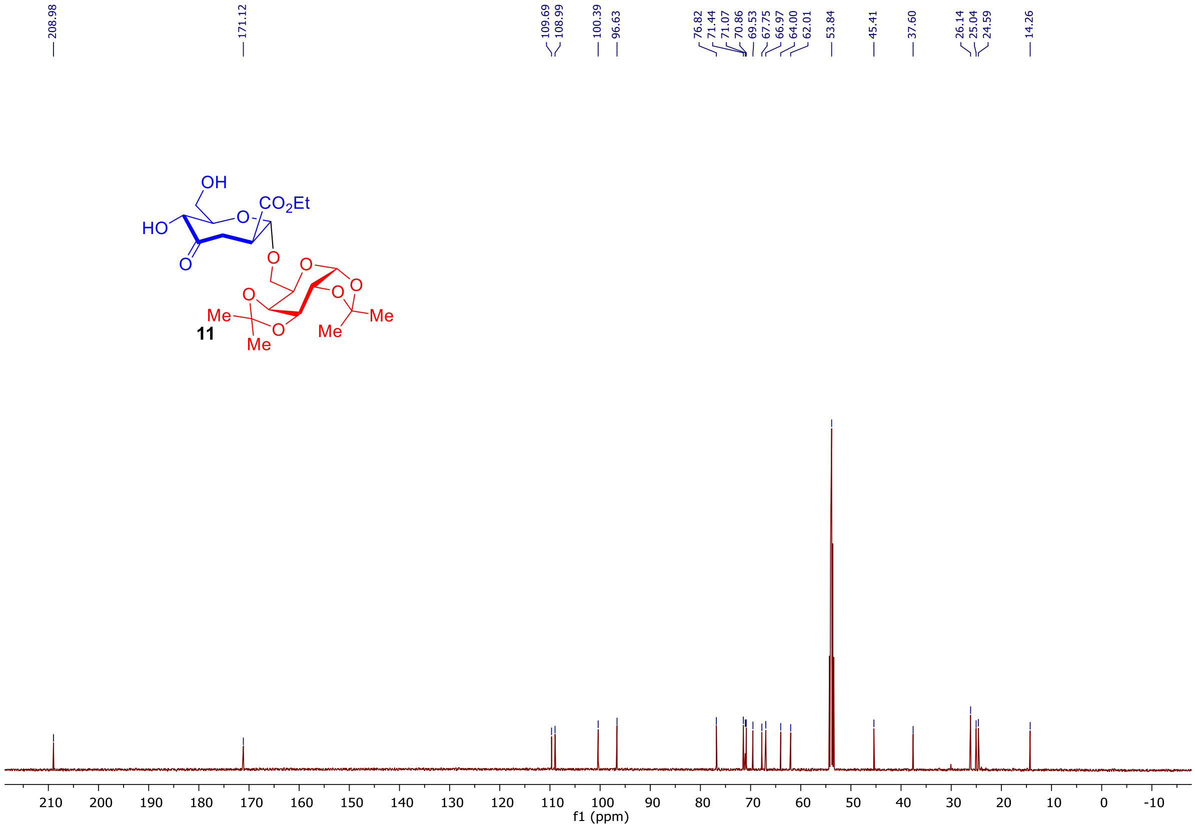

Supplementary Figure 476: ^{13}C spectra for **11**

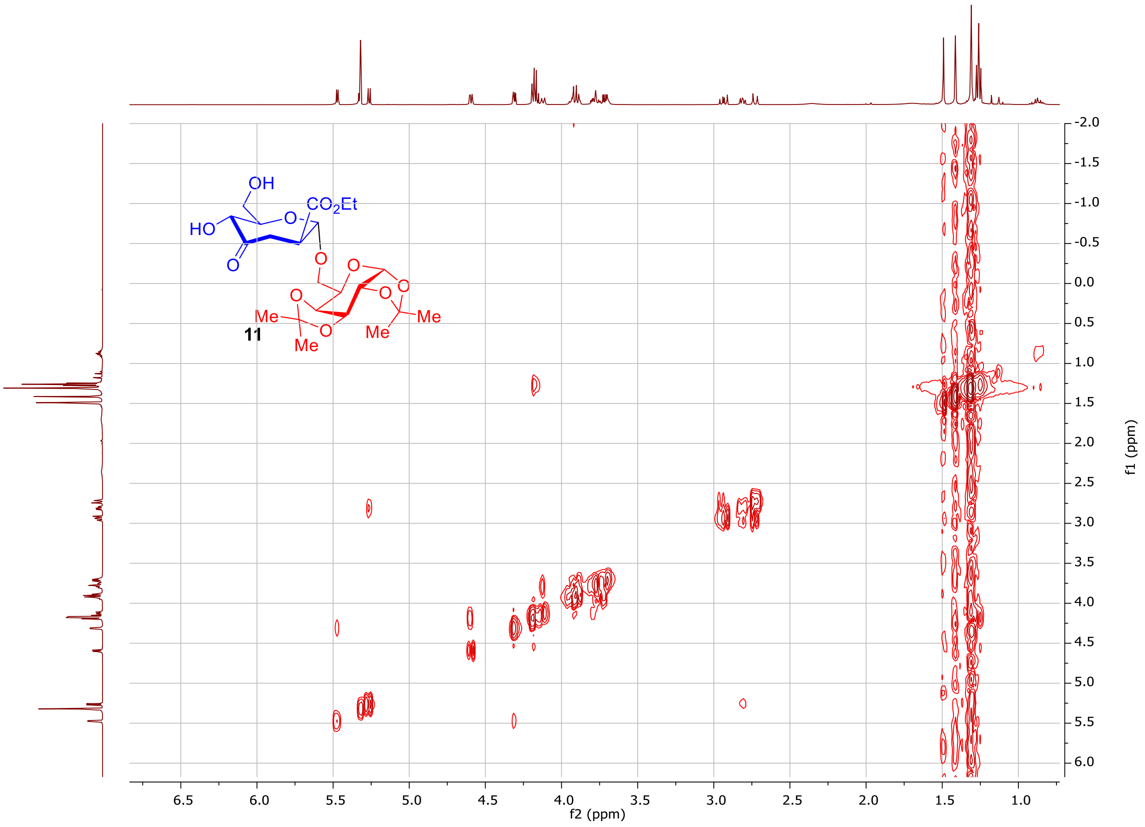

Supplementary Figure 477: COSY spectra for compound **11**

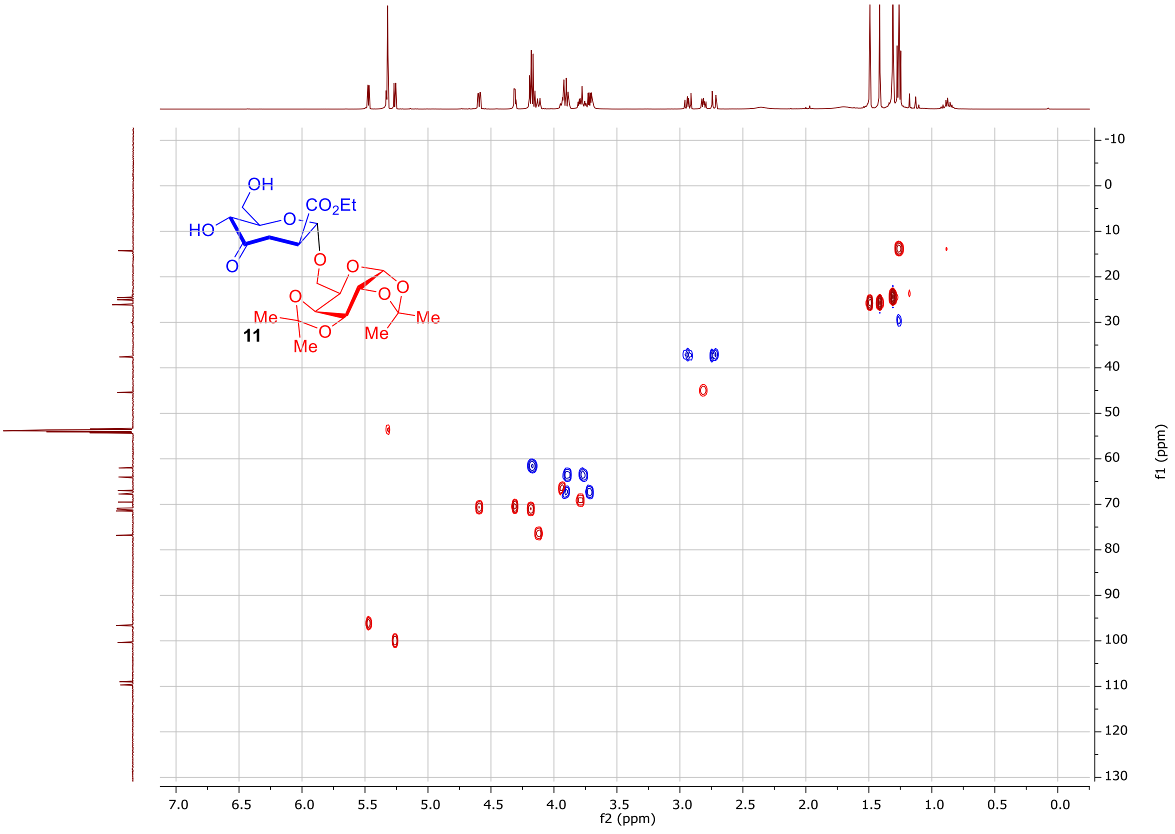

Supplementary Figure 478: HSQC spectra for compound **11**

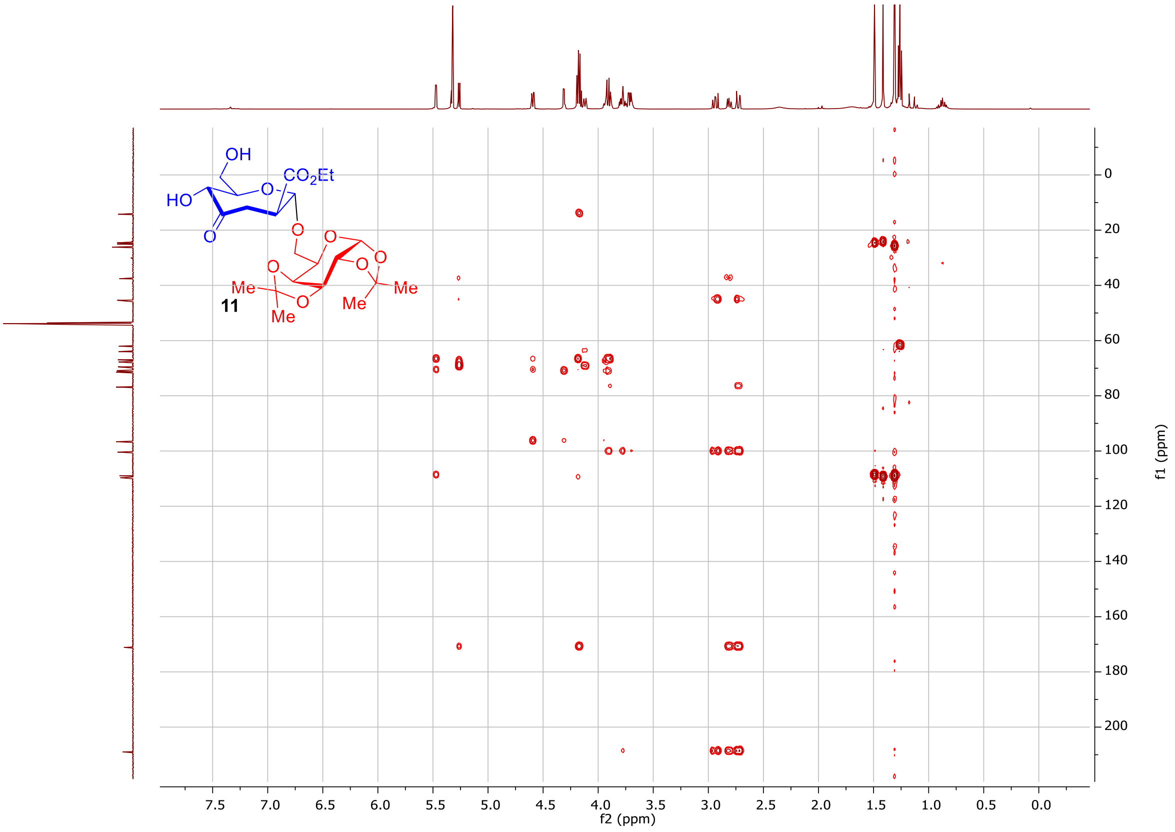

Supplementary Figure 479: HMBC spectra for compound **11**

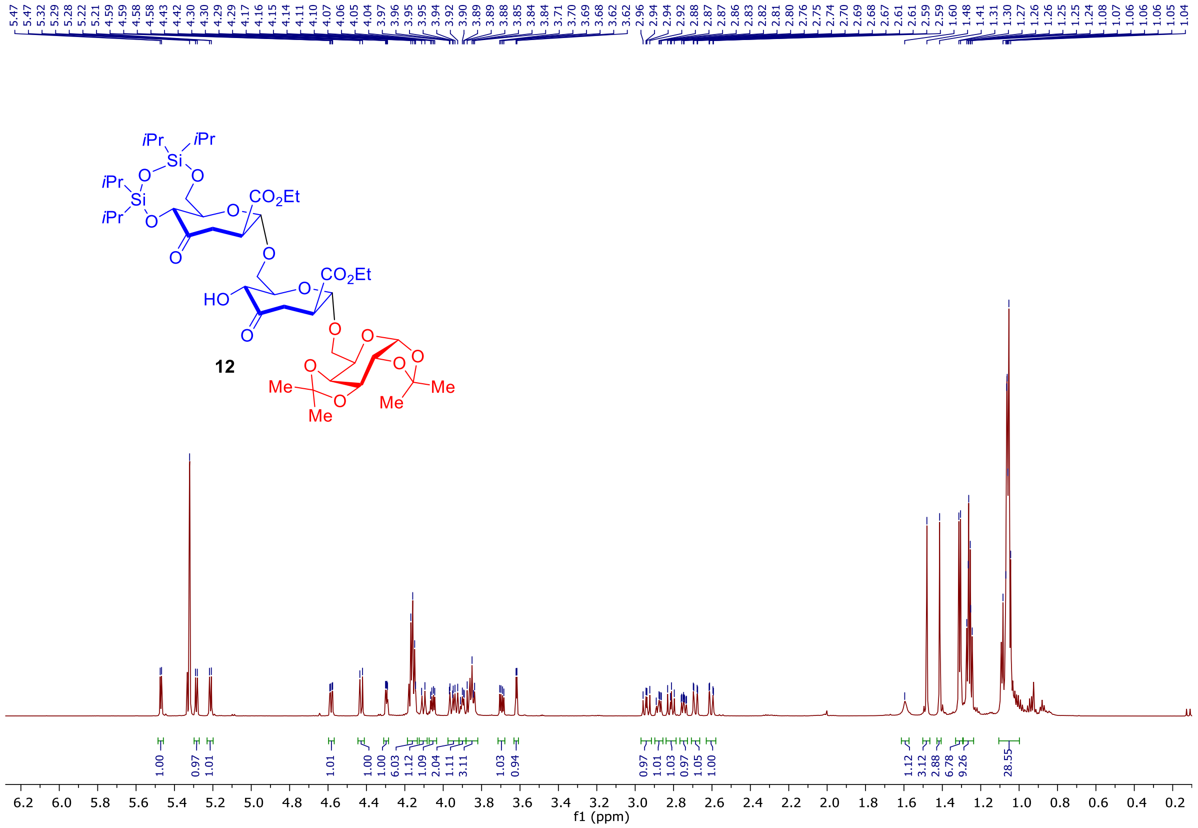

Supplementary Figure 480: ^{1}H spectra for **12**

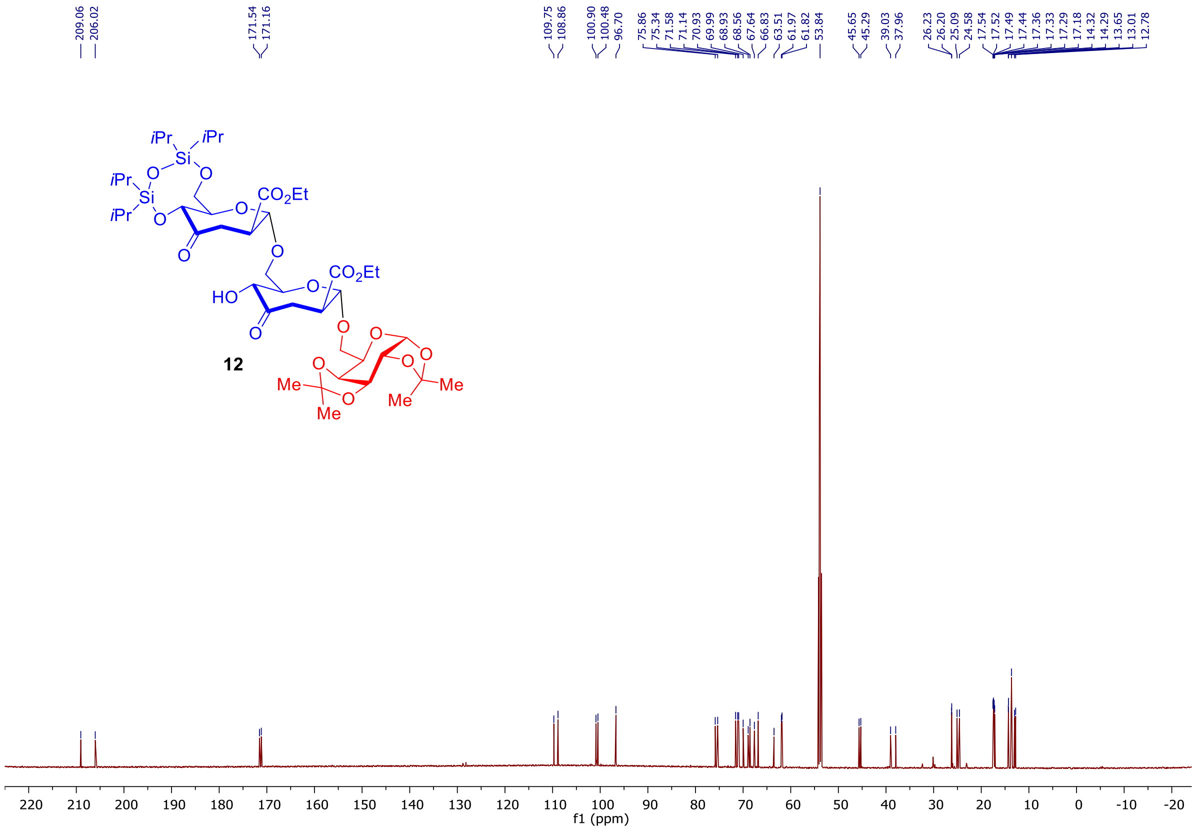

Supplementary Figure 481: ^{13}C spectra for **12**

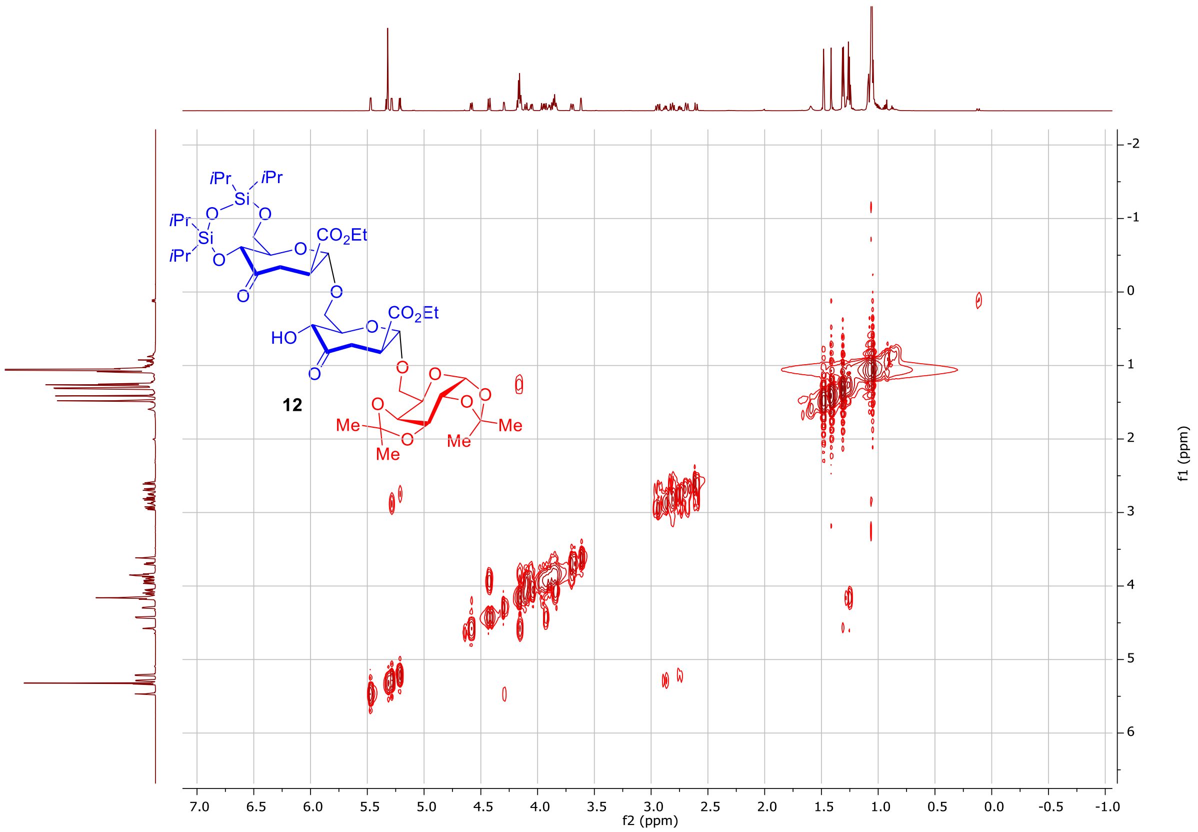

Supplementary Figure 482: COSY spectra for compound **12**

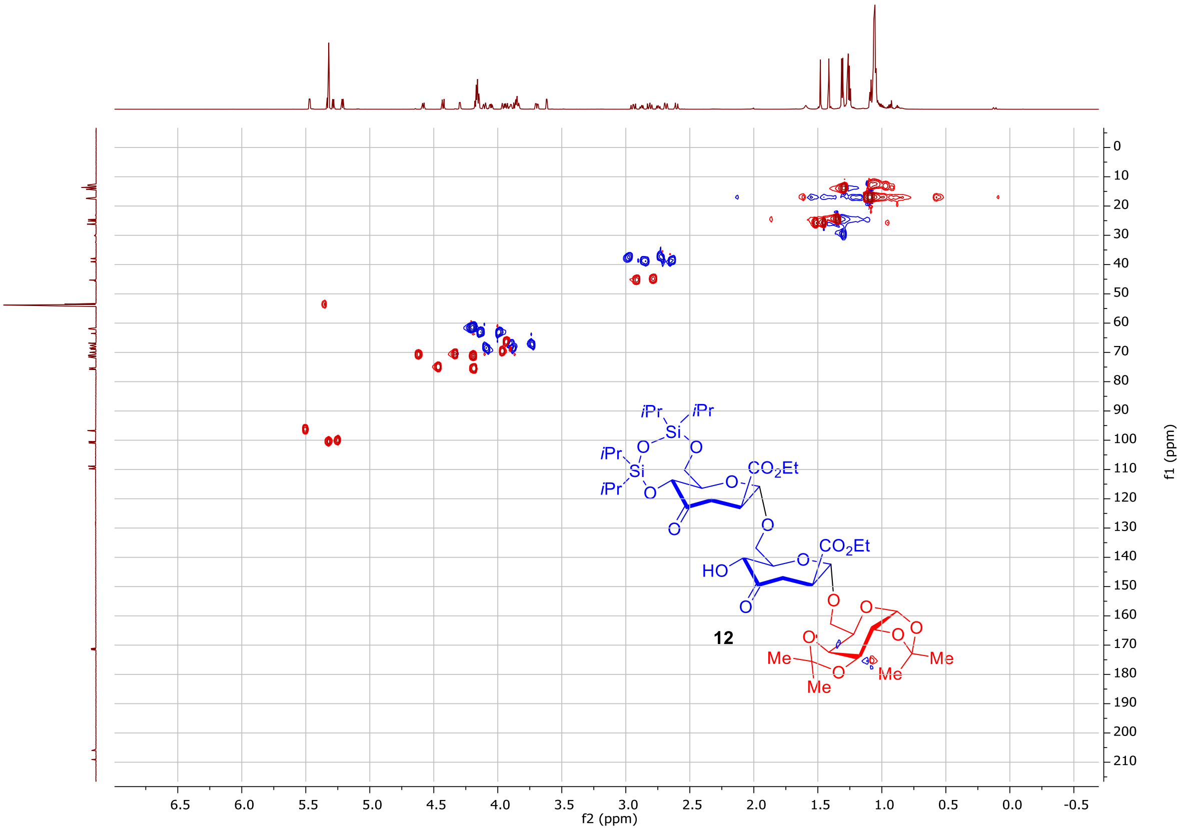

Supplementary Figure 483: HSQC spectra for compound **12**

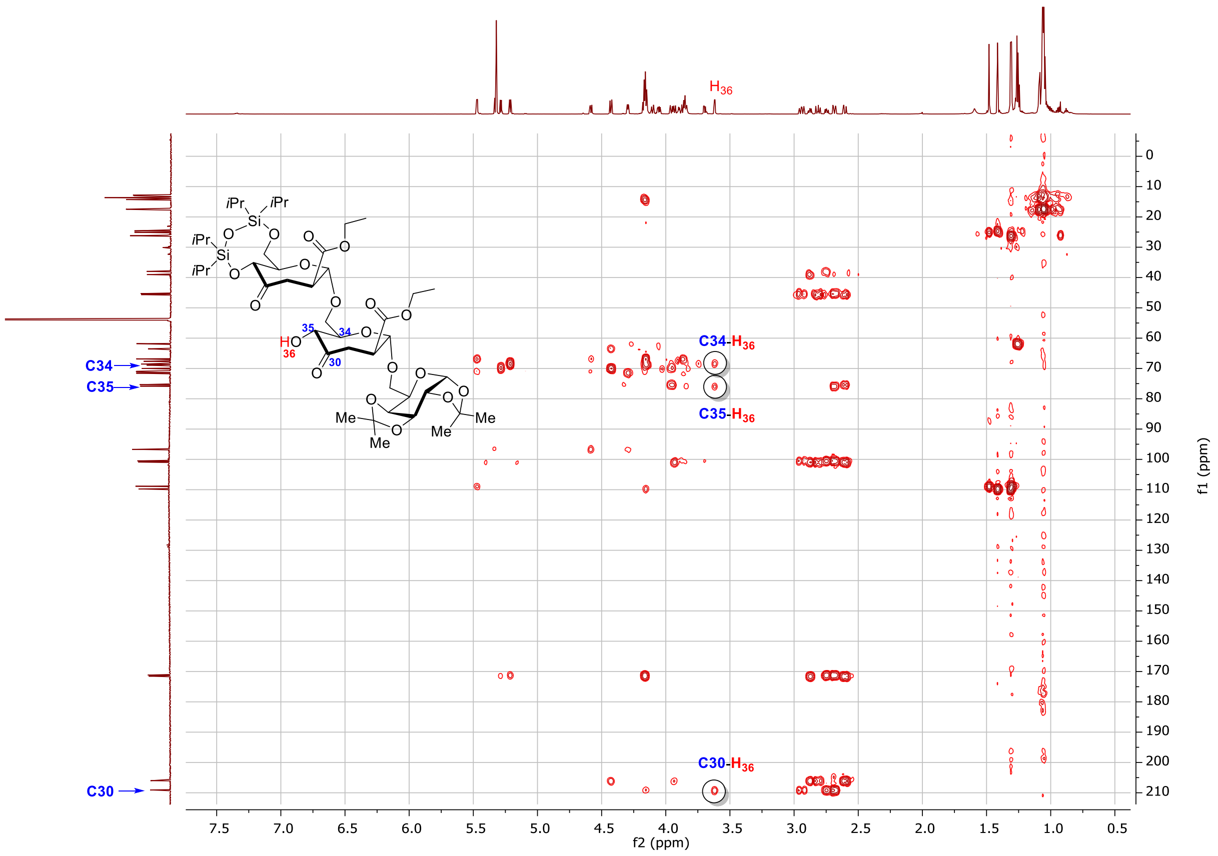

Supplementary Figure 484: HMBC spectra for compound **12**

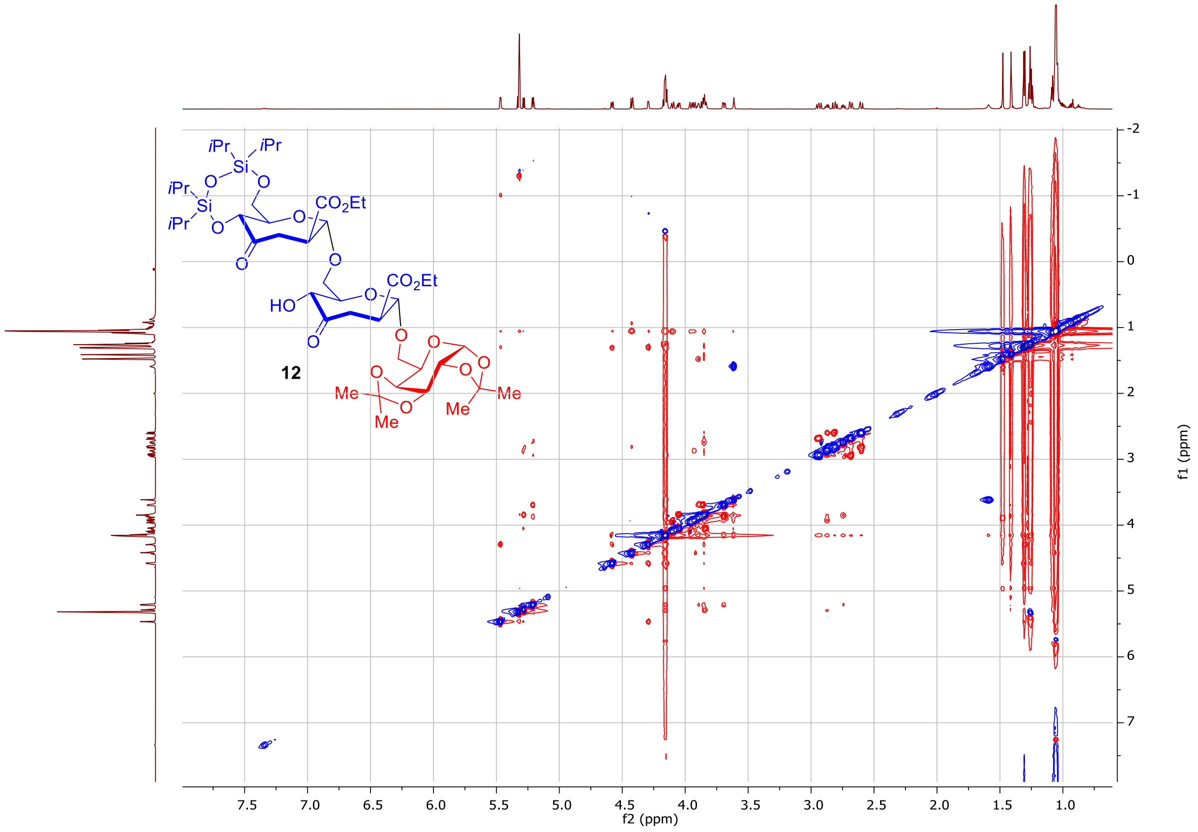

Supplementary Figure 485: NOESY spectra for compound **12**

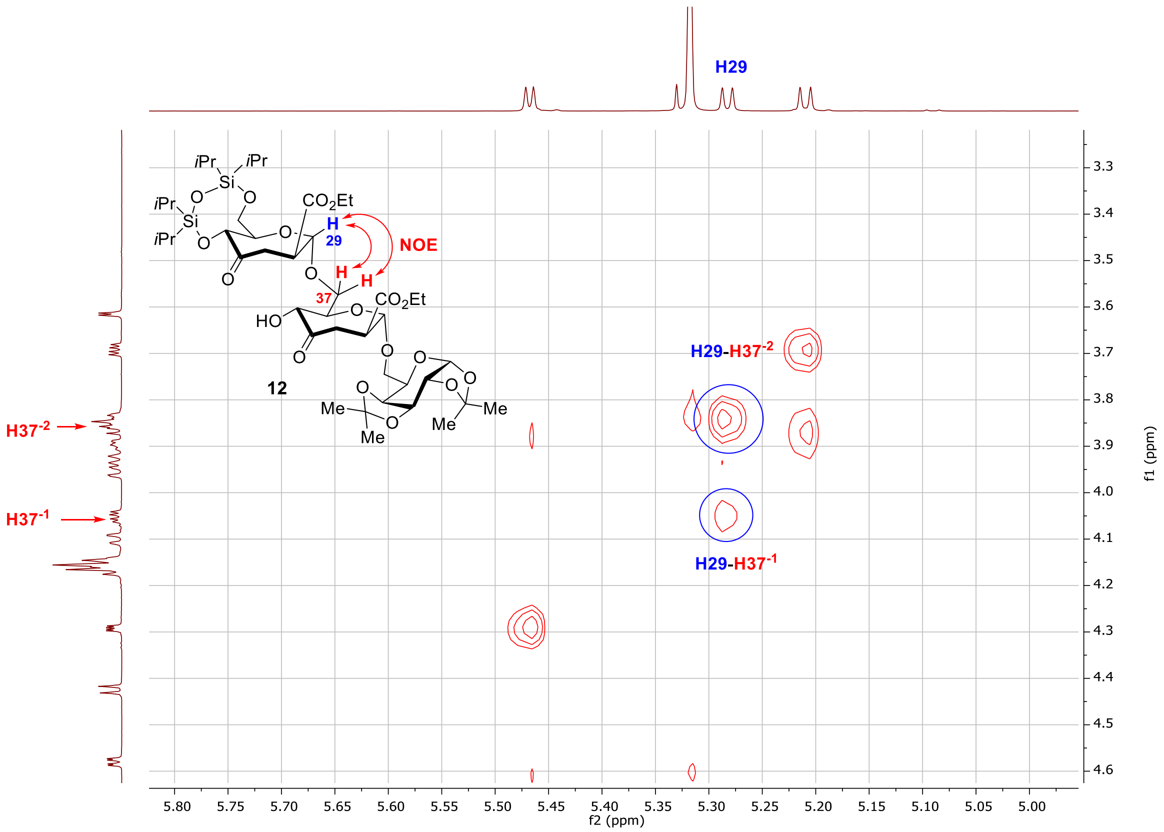

Supplementary Figure 486: NOESY spectra for compound **12**

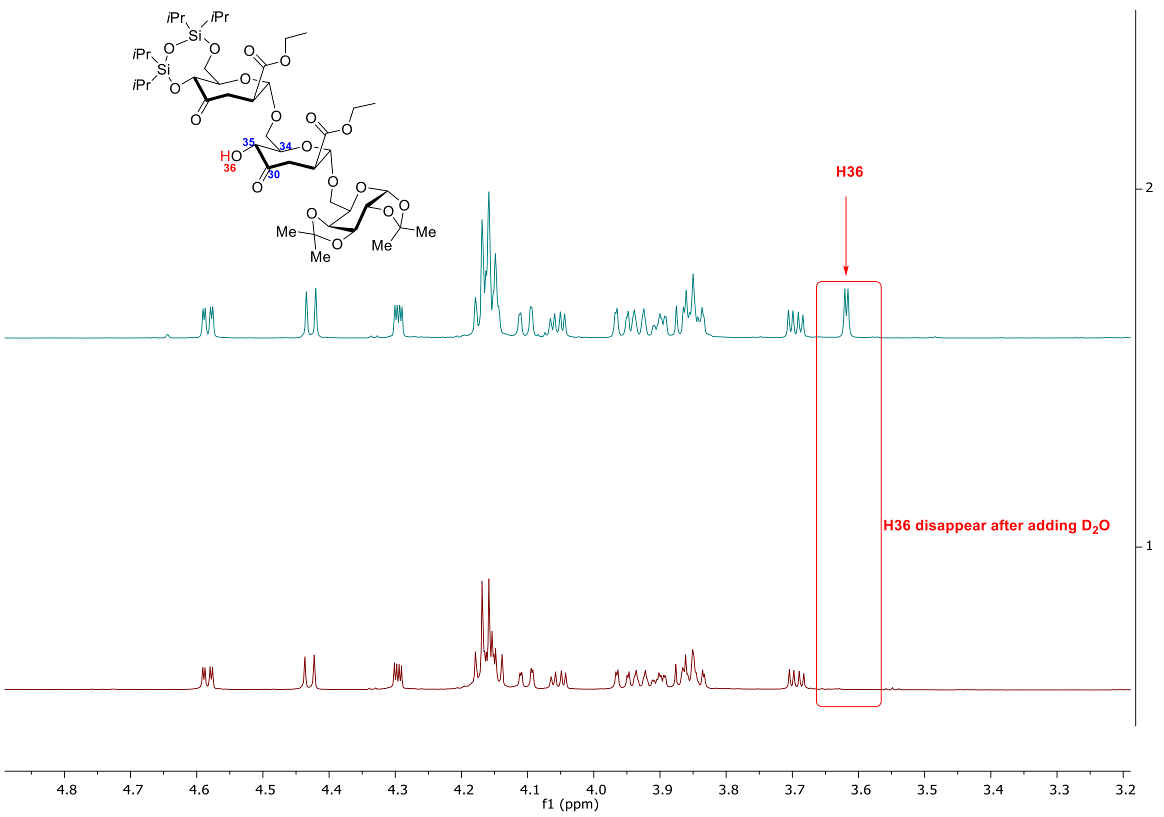

Supplementary Figure 487: ^{1}H NMR upon addition of D_2O to compound **12** to exchange the labile proton on the hydroxyl group

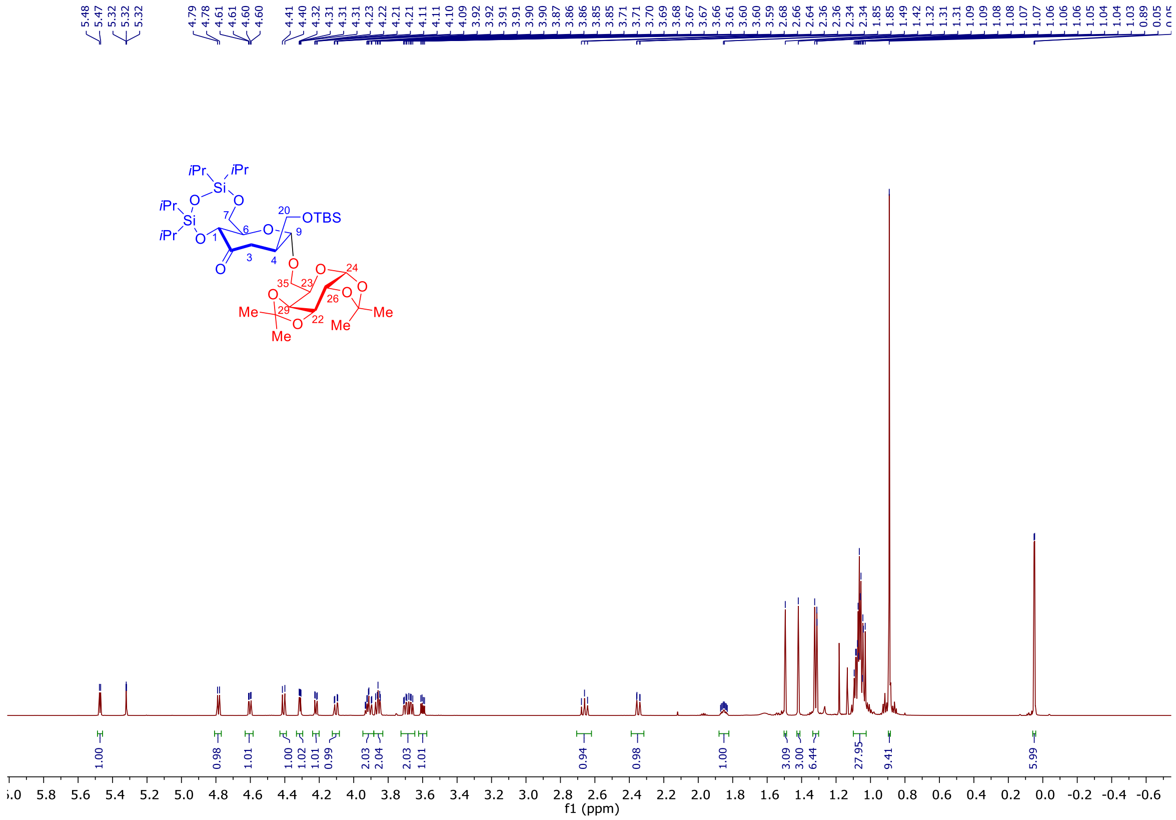

Supplementary Figure 488: ^{1}H spectra for **alpha-3bh**

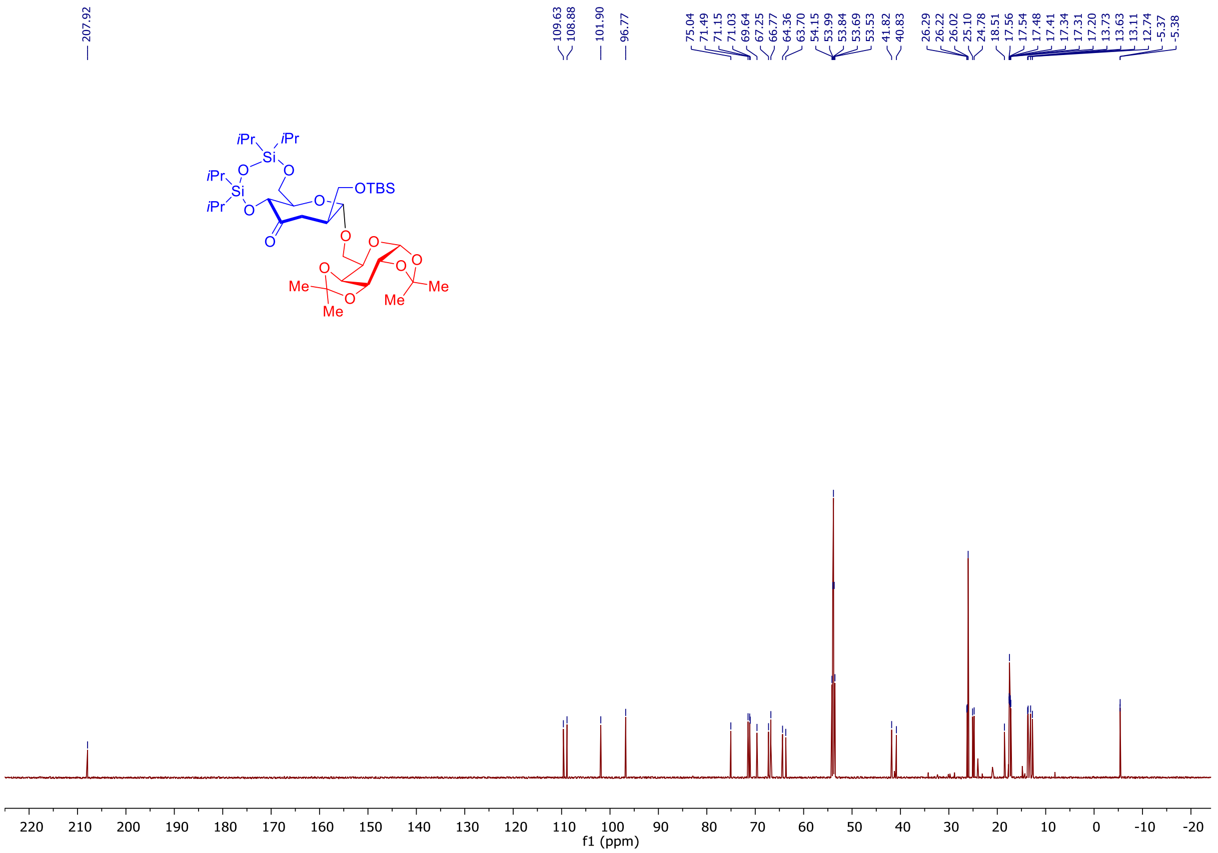

Supplementary Figure 489: ^{13}C spectra for **alpha-3bh**

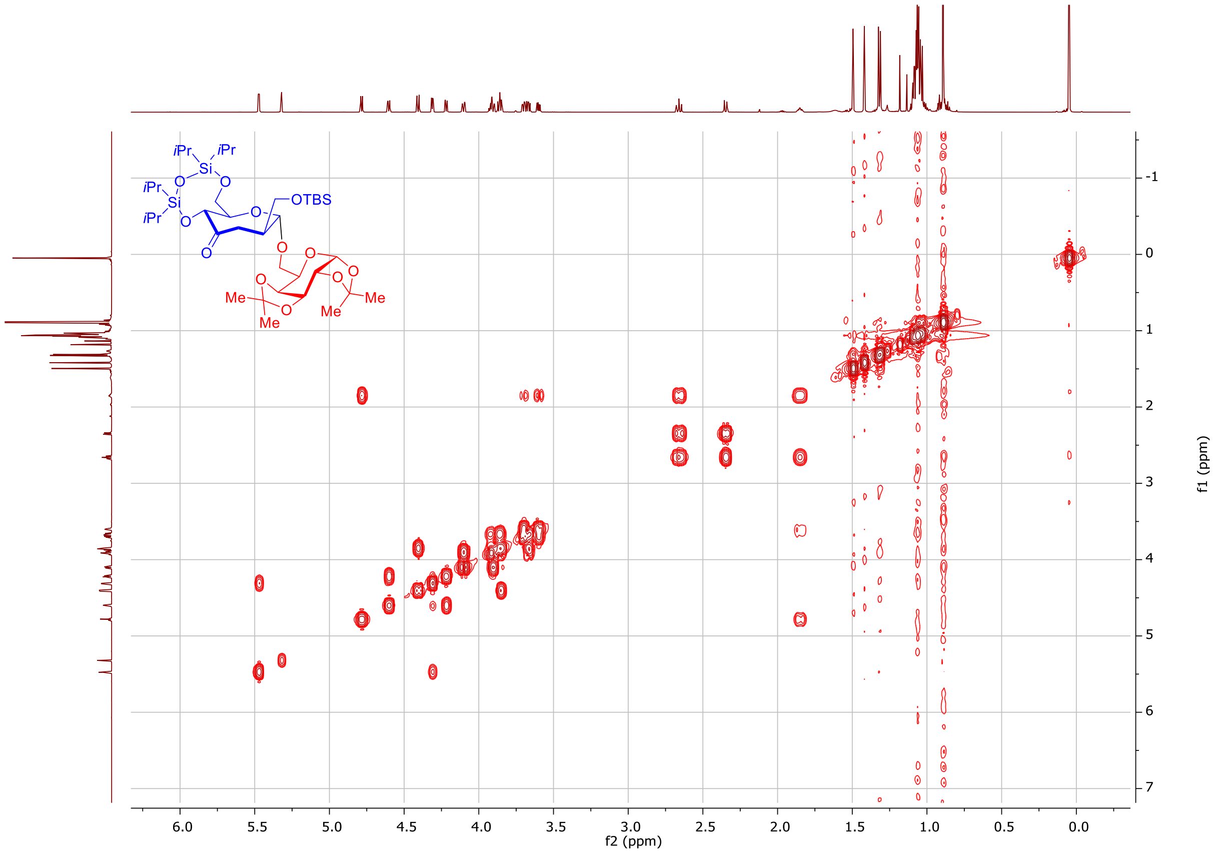

Supplementary Figure 490: COSY spectra for compound **alpha-3bh**

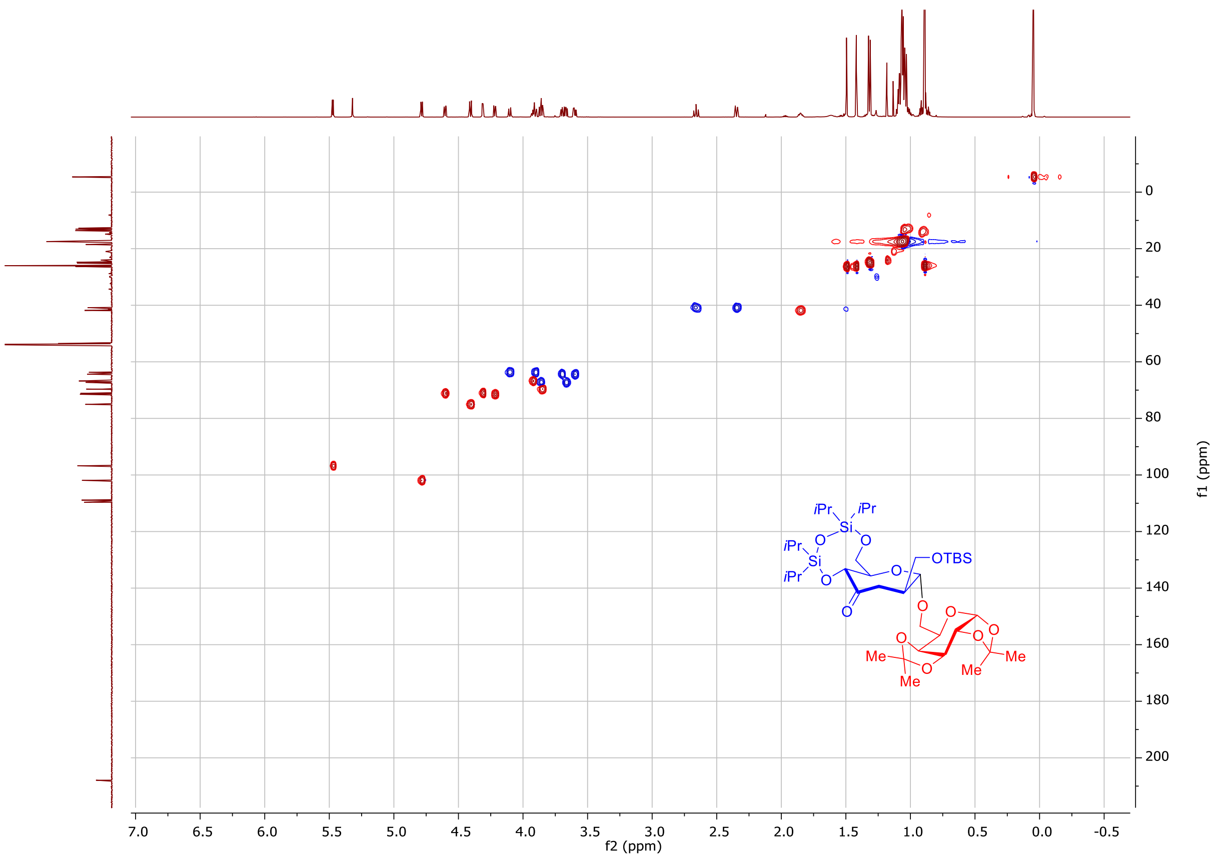

Supplementary Figure 491: HSQC spectra for compound **alpha-3bh**

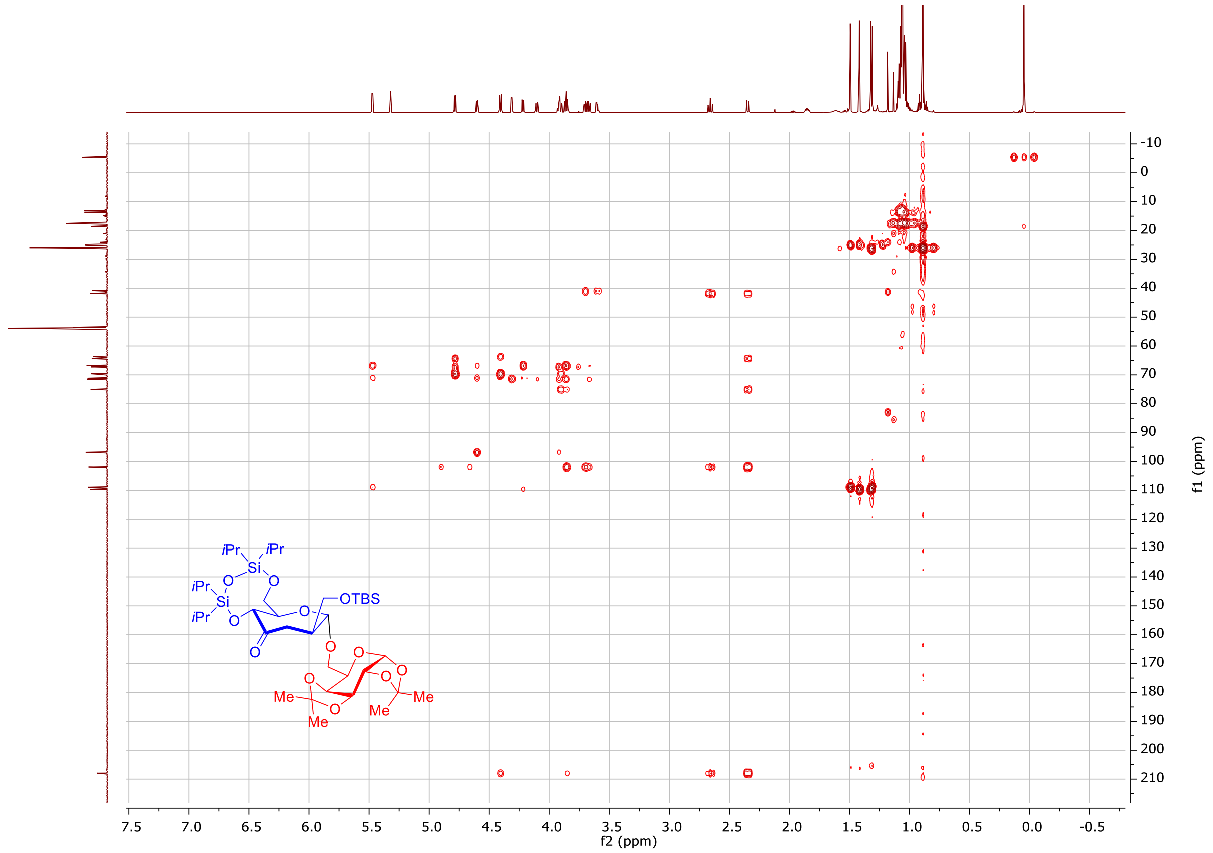

Supplementary Figure 492: HMBC spectra for compound **alpha-3bh**

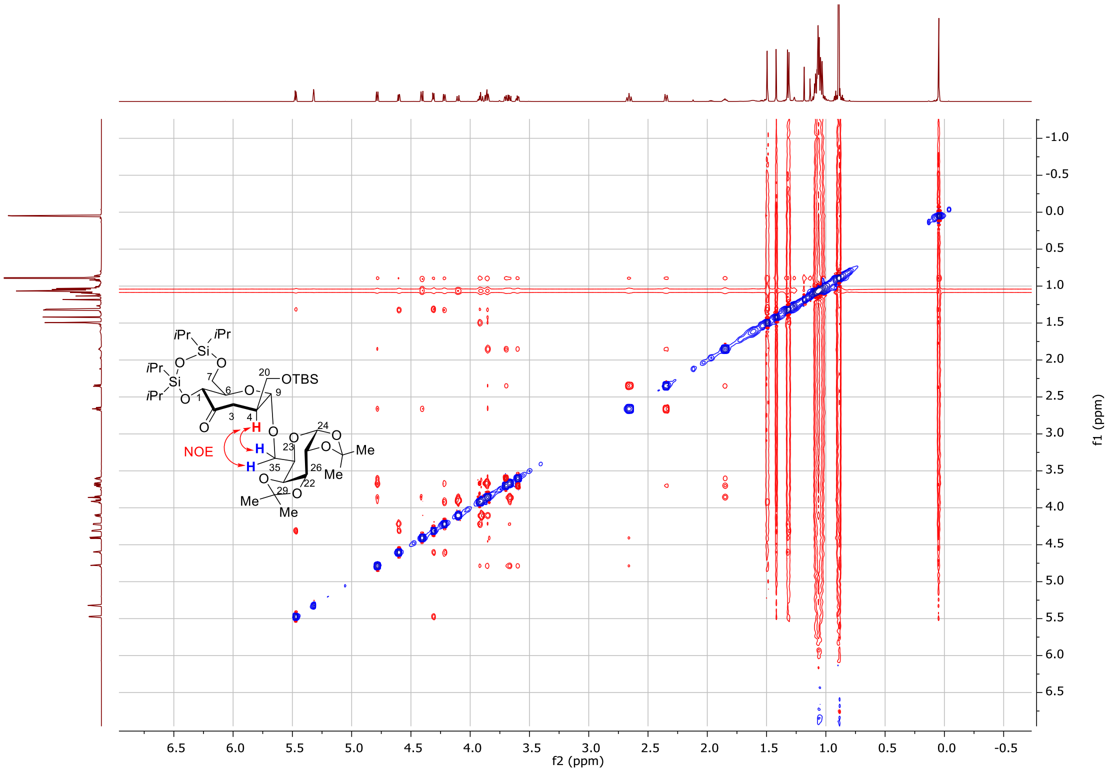

Supplementary Figure 493: NOESY spectra for compound **alpha-3bh**

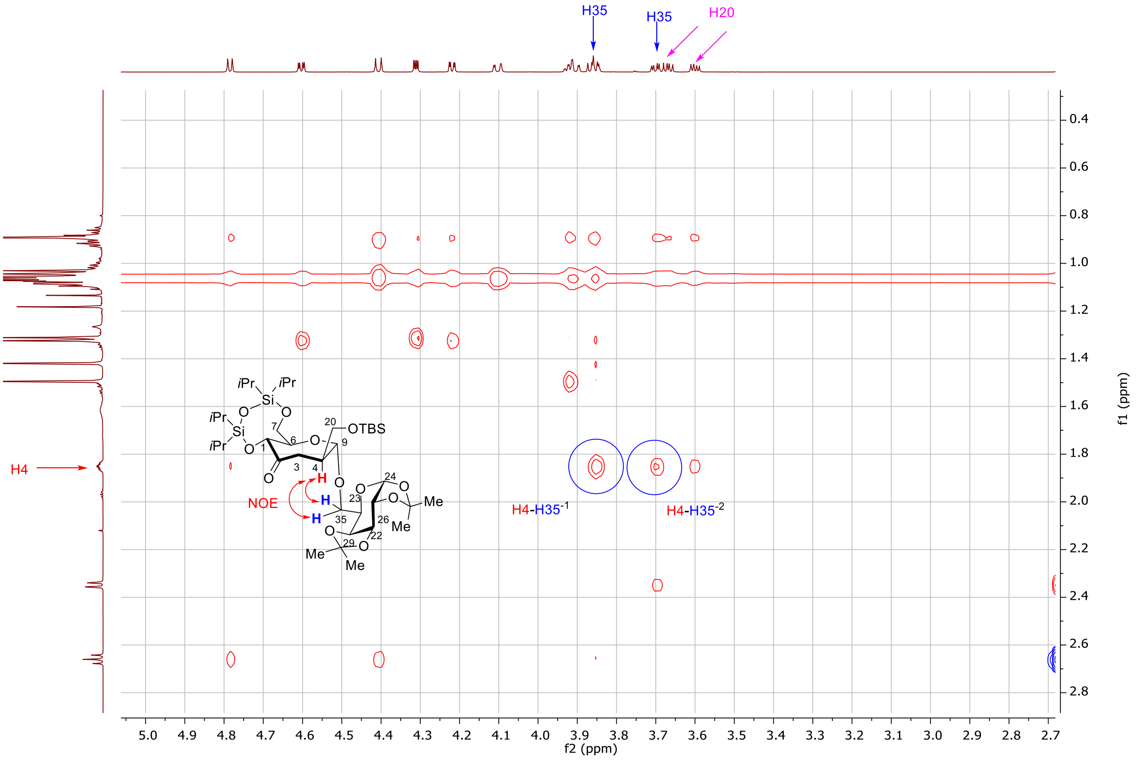

Supplementary Figure 494: NOESY spectra for compound **alpha-3bh**
